# iTRAQ-Based Proteomics Analysis and Network Integration for Kernel Tissue Development in Maize

**DOI:** 10.3390/ijms18091840

**Published:** 2017-08-24

**Authors:** Long Zhang, Yongbin Dong, Qilei Wang, Chunguang Du, Wenwei Xiong, Xinyu Li, Sailan Zhu, Yuling Li

**Affiliations:** 1 College of Agronomy, Henan Agricultural University, Collaborative Innovation Center of Henan Grain Crops, National Key Laboratory of Wheat and Maize Crop Science, 63 Nongye Rd., Zhengzhou 450002, China; swgczl@163.com (L.Z.); dyb0816@163.com (Y.D.); wangqilei2008@yeah.net (Q.W.); wumingzhilixinyu@sina.com (X.L.); sailanzhu@163.com (S.Z.); 2 Deptment of Biology and Molecular Biology, Montclair State University, Montclair, NJ 07043, USA; duc@mail.montclair.edu (C.D.); xiongwenwei@gmail.com (W.X.)

**Keywords:** *Zea mays*, kernel development, quantitative proteomics, iTRAQ, protein network integration

## Abstract

Grain weight is one of the most important yield components and a developmentally complex structure comprised of two major compartments (endosperm and pericarp) in maize (*Zea mays* L.), however, very little is known concerning the coordinated accumulation of the numerous proteins involved. Herein, we used isobaric tags for relative and absolute quantitation (iTRAQ)-based comparative proteomic method to analyze the characteristics of dynamic proteomics for endosperm and pericarp during grain development. Totally, 9539 proteins were identified for both components at four development stages, among which 1401 proteins were non-redundant, 232 proteins were specific in pericarp and 153 proteins were specific in endosperm. A functional annotation of the identified proteins revealed the importance of metabolic and cellular processes, and binding and catalytic activities for the tissue development. Three and 76 proteins involved in 49 Kyoto Encyclopedia of Genes and Genomes (KEGG) pathways were integrated for the specific endosperm and pericarp proteins, respectively, reflecting their complex metabolic interactions. In addition, four proteins with important functions and different expression levels were chosen for gene cloning and expression analysis. Different concordance between mRNA level and the protein abundance was observed across different proteins, stages, and tissues as in previous research. These results could provide useful message for understanding the developmental mechanisms in grain development in maize.

## 1. Introduction

Seed development in flowering plants is a complicated dynamic process and very important for agricultural production. As a model organism, maize (*Zea mays* L.) is the largest crop in the world, and is a critical food source as human consumption and animal feed source [1]. Its mature seed is composed of three components, the diploid embryo and the triploid endosperm developed from double fertilization, and the maternally derived pericarp enclosed [2].

Endosperm not only plays an important role in determining the economic and nutritional value, but also supports the embryo at germination [3]. It makes up the majority of the kernel dry matter (roughly 85%), containing over 80% of total seed starch and about 58% of the total proteins [4]. The development of endosperm undergoes a rapid growth phase through four main stages: syncitial, cellularization, cell fate specification and differentiation [5]. Pericarp primarily protects both embryo and endosperm from physical and biological damage. Besides, it plays important roles in determining grain size [6,7] and grain quality [8]. Hood et al. [9] pointed out that the amounts of hydroxyproline and thus extensin related to pericarp thickness and toughness through the comparison of pericarp cell wall dry weight, whole grain dry weight, total hydroxypoline and PC-1 accumulation in three varieties with different kernel size and pericarp thickness.

Understanding the molecular mechanisms underlying seed development is one of the major goals to improve grain weight and grain constituents. Transcriptome and proteome maps provide a powerful tool to investigate the maize seed development [10]. In addition, the rapid development of proteomic technologies provides an unprecedented opportunity for plant proteomic profiling [11]. Proteomics research in maize seed development involves the identification and characterization of proteins in order to elucidate their function and interactions [12]. A complex quantitative proteome and phosphorylation profile during wheat grain development was revealed using isobaric tags for relative and absolute quantitation (iTRAQ)-based quantitative proteome approach [13]. Wang et al. [14] detected the proteome changes in rice hull at the booting, flowering, and milk-ripe growth stages through iTRAQ method.

In maize, several mutants with effect on seed development have been identified, such as *shrunken-2* (*sh2*), *opaque-2* (*o2*), *Miniture1* (*Mn1*), *empty pericarp5* (*emp5*) [15,16,17,18]. Additionally, seed development has been studied in many plants, including rice [14], maize [19], and wheat [20] in recent years. However, few systematic and comprehensive protein expression profiles for endosperm and pericarp development in maize have been reported. In our previous study, some specific and highly expressed proteins in the pericarp have been identified, which might simultaneously determine its special structure [21].

Herein, the differently expressed proteins for both endosperm and pericarp at four key developmental stages were studied through the iTRAQ-based protein method by using an inbred line Dan232 with Chinese pedigree. In addition, the protein networks reflecting the relationship among detected proteins and their functions were constructed. Our main objective was to reveal much more key proteins in controlling grain development by uncovering the dynamic protein expression profiles and the comprehensive differences between the two tissues. The result could help in understanding the dynamic mechanisms during seed development in maize.

## 2. Results

### 2.1. The Developmental Process of Grain and Its Two Components

**Figure 1:**
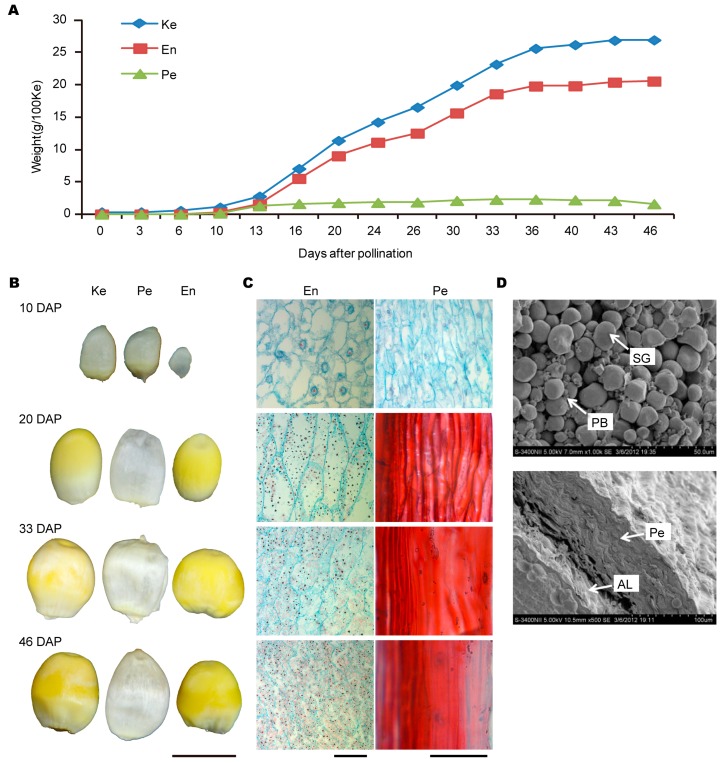
The developmental pattern and the morphological and cytological characteristics of kernels for inbred Dan232. (**A**) Dry matter weight of grain and its two component parts, the endosperm and pericarp. (**B**) The exact appearance for complete kernel (Ke), endosperm (En), and pericarp (Pe) at 10, 20, 33 and 46 DAP. (**C**). Microscopic section of pericarp (**right**) and endosperm (**left**) during kernel development. (**D**) The characteristics of pericarp (the **right**) and starch granules in the endosperm (the **left**) at 46 DAP mature kernels by scanning electron microscopy illustrating starch granules (SG), protein bodies (PB), aleurone layer (AL). (**A**) Bar = 8 cm; and (**B**,**C**) bar = 50 μm for endosperm, and bar = 100 μm for pericarp.

The weights for the whole grain from 3 to 46 days after pollination (DAP), and for the two component parts (endosperm and pericarp) from 10 to 46 DAP for inbred Dan232, were quantified, respectively. The whole grain weight increased slowly before 10 DAP, followed a rapid increase during 10–36 DAP, and then increased slowly again (Figure 1A). The same pattern was observed for endosperm, but different for pericarp. At 46 DAP, both the whole grain weight and endosperm weight reached the highest point, which were 26.93 g/100 grains and 20.56 g/100 grains, respectively.

Microstructures of the endosperm and pericarp at 10, 20 33 and 46 DAP showed that the cell size changed in a similar manner at all developmental stages (Figure 1B,C). Endosperm at 10 DAP was in the cell division stage, and then, accumulated substances rapidly. At 20 DAP, endosperm cells were filled with starch grains. Highly different characteristics were observed for starch granules in endosperm and pericarp at maturity using light microscopy and SEM (Figure 1D). The pericarp texture was rough. The starch granules in the endosperm were small polygonal, densely packed with protein bodies. Simultaneously considering the development patterns and the structures of the whole grain, both the endosperm and pericarp at 10, 20, 33 and 46 DAP were chosen for further analyzing the differentially expressed proteins (DEPs) during grain development.

### 2.2. Quantitative Protein Identifications Using iTRAQ

**Figure 2:**
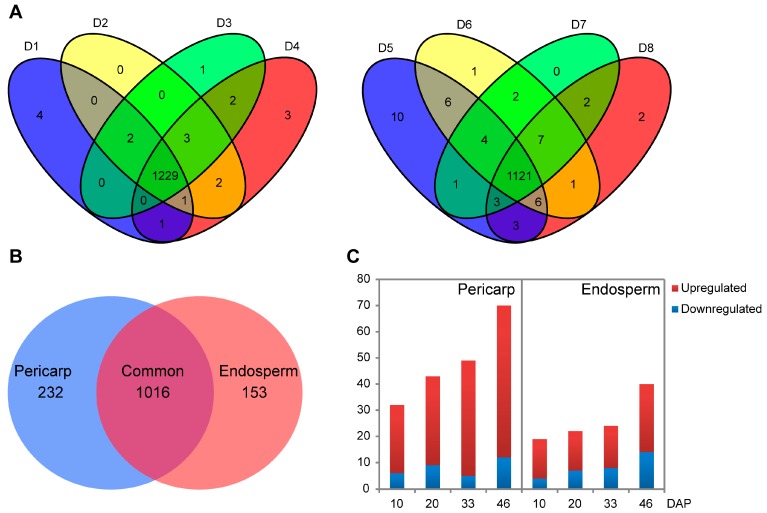
Quantities of differentially expressed proteins according to the developmental stages of the two tissues for inbred Dan232. (**A**) Venn diagrams for identified proteins in the pericarp (**left**) and endosperm (**right**). (**B**) Venn diagram showing the number of proteins in the pericarp and endosperm. (**C**) The numbers of up-regulated and down-regulated proteins in endosperm and pericarp at each developmental stage.

A total of 9539 proteins were identified in all samples, 1248 proteins from pericarp and 1169 proteins from endosperm, among which 1401 were non-redundant proteins in both replicates (Table 1 and Table S1). The peptides and the quantity information for identified proteins were listed in Table S2. For the pericarp, a total of 4952 proteins were obtained, 1237, 1237, 1237 and 1241 proteins were recognized at the 10, 20, 33 and 46 DAP, respectively, of which 1086 proteins were commonly found at all developmental stages, and 4, 0, 1 and 3 proteins were stage specific, respectively. For the endosperm, a total of 4587 proteins were obtained, 1154, 1148, 1140, 1145 proteins were recognized at 10, 20, 33 and 46 DAP, respectively, of which 985 proteins were commonly found at all developmental stages, and 10, 1, 0 and 2 proteins were stage specific, respectively (Figure 2A).

There were 232 and 153 uniquely identified proteins from pericarp and from endosperm, respectively (Figure 2B, Table S3). As shown in Figure 2C, 162 up-regulated proteins were identified in pericarp, among which 26, 34, 44 and 58 proteins were particularly up-regulated at 10, 20, 33, and 46 DAP, respectively. Bioinformation analysis showed that the upregulated proteins were mainly involved in cell wall, membrane, envelope biogenesis, carbohydrate transport and metabolism, lipid transport and metabolism and signal transduction mechanisms. For endosperm, 72 up-regulated proteins were shared by all developmental stages, among which 15, 15, 16 and 26 proteins were up-regulated at 10, 20, 33, and 46 DAP, respectively. These proteins were involved in amino acid transport and metabolism, nucleotide transport and metabolism, posttranslational modification, protein turnover, chaperones, and translation, ribosomal structure and biogenesis.

### 2.3. Functional Annotation of Identified Proteins

**Figure 3:**
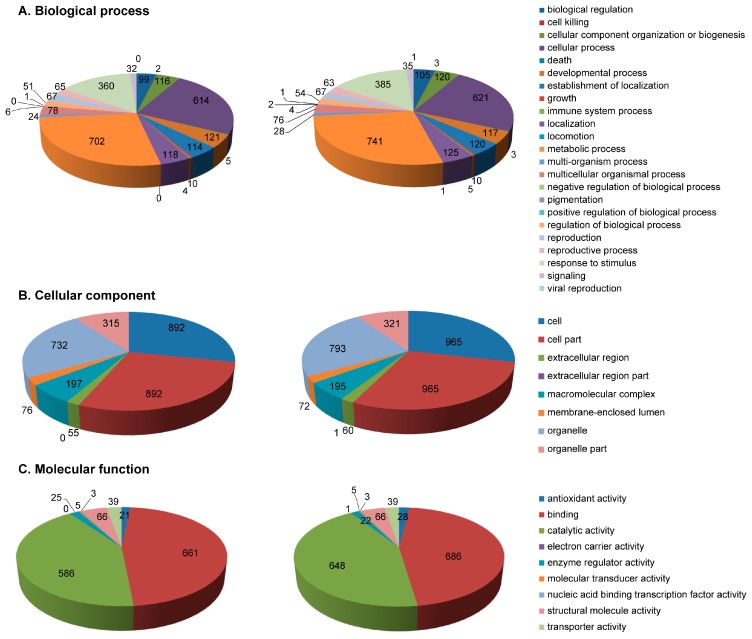
Functional classification of the identified proteins at all developmental stages for both endosperm and pericarp according to: GO annotations in biological process (**A**); molecular function (**B**); and cellular component (**C**). **Left**, pericarp; **right**, endosperm.

A total of 1285 proteins were functionally annotated using Blast2GO according to the biological process, cellular component, and molecular function in pericarp and endosperm, respectively (Figure 3).

For biological process, the proteins could be classified into 23 categories according to gene ontology (GO). The three functional categories with high number of DEPs were metabolic process (702 in pericarp and 741 in endosperm), cellular process (614 in pericarp and 621 in endosperm), and response to stimulus (360 in pericarp and 385 in endosperm). Therefore, these functional categories were the very important in the development of pericarp and endosperm. Some proteins were only identified in pericarp, including locomotion (GRMZM2G050193_P01, calcium lipid binding-like protein), pigmentation (GRMZM2G034083_P01, ATP-citrate synthase and GRMZM2G107082_P01, hypothetical protein), and viral reproduction (GRMZM2G050193_P01, calcium lipid binding-like protein).

For cell component, the top three categories with high number of DEPs were cell part (892 in pericarp and 965 in endosperm), cell (892 in pericarp and 965 in endosperm), and organelle (702 in pericarp and 741 in endosperm) into eight categories. GRMZM2G136895_P01 (putative O-Glycosyl hydrolase superfamily protein) involved in extracellular region part was only found in pericarp as shown in Figure 2B.

For molecular function, the top three categories with high number of DEPs were binding (686 in pericarp and 661 in endosperm), catalytic activity (648 in pericarp and 586 in endosperm) and structural molecule activity (66 both in pericarp and endosperm). GRMZM2G032367_P01 (hypothetical protein) involved in electron carrier activity was specific in pericarp.

By Clusters of Orthologous Groups (COG) database, the 1030 proteins in pericarp and the 1002 proteins in endosperm were classified into 22 metabolic processes (Figure S1). These processes mainly included energy production and conversion, amino acid transport and metabolism, carbohydrate transport and metabolism, translation, ribosomal structure and biogenesis and posttranslational modification, protein turnover, and chaperones.

### 2.4. Hierarchical Clustering Analysis of Protein Expression during Pericarp and Endosperm Development

**Figure 4:**
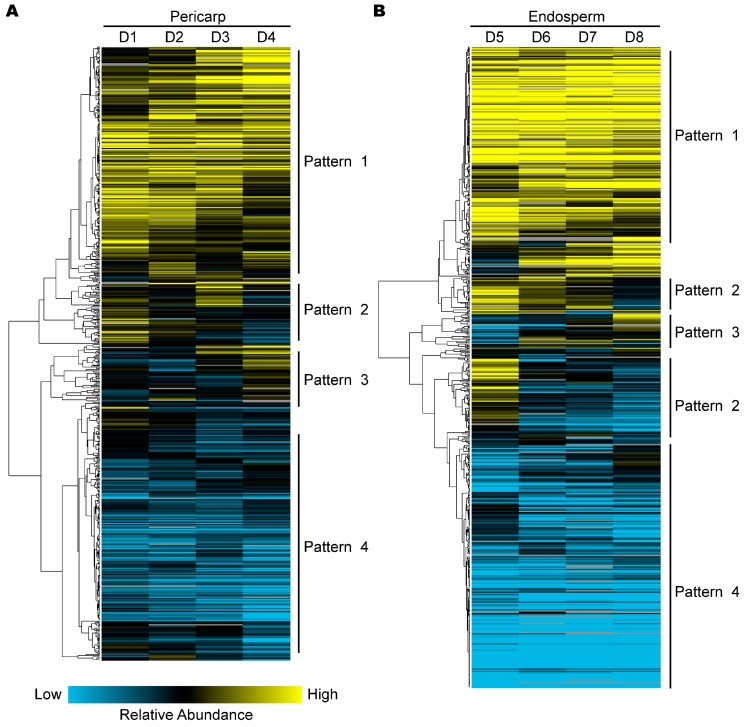
Hierarchical clustering analysis for expression patterns of differentially expressed proteins identified in: pericarp (**A**); and endosperm (**B**) by using Cluster 3.0 software. Pattern 1: up-regulated expression; Pattern 2: up–down-regulated expression; Pattern 3: down–up-regulated expression; Pattern 4: down-regulated expression.

Four expression patterns (patterns 1–4) were present for proteins in both pericarp and endosperm (Figure 4), including up-regulated expression, up–down-regulated expression, down–up-regulated expression and down-regulated expression. In the pericarp, Pattern 1 showed a up-regulated expression trend, and represented by 457 proteins mainly involved in carbohydrate transport and metabolism and cell wall, membrane, envelope biogenesis. Pattern 2, an up–down-regulated expression trend, represented by 77 proteins related to translation, ribosomal structure and biogenesis and cytoskeleton. Pattern 3, a down–up-regulated expression trend, represented by 97 proteins mainly involved carbohydrate metabolism, lipid transport and metabolism. Pattern 4 displayed a generally down-regulated expression trend, and included 412 proteins primarily participated in carbohydrate metabolism, amino acid metabolism and posttranslational modification, protein turnover, chaperones.

In the endosperm, Pattern 1 included 338 proteins mainly involved in energy production and conversion, carbohydrate metabolism and translation, ribosomal structure and biogenesis. Pattern 2 represented by 98 proteins related to carbohydrate metabolism and posttranslational modification, protein turnover, chaperones. Pattern 3 included 66 proteins with functions similar to those in Pattern 1. Pattern 4 included 389 proteins mainly related to amino acid metabolism, carbohydrate metabolism, posttranslational modification, protein turnover, chaperones, and intracellular trafficking, secretion, and vesicular transport.

### 2.5. Interaction Network Construction for Identified Proteins

**Figure 5:**
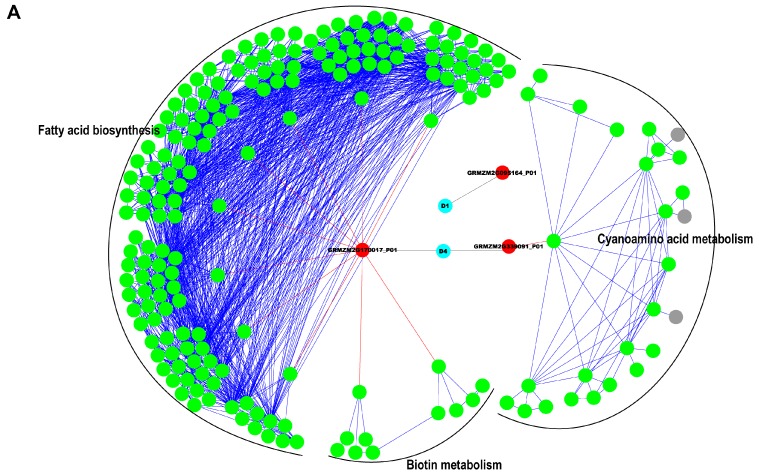

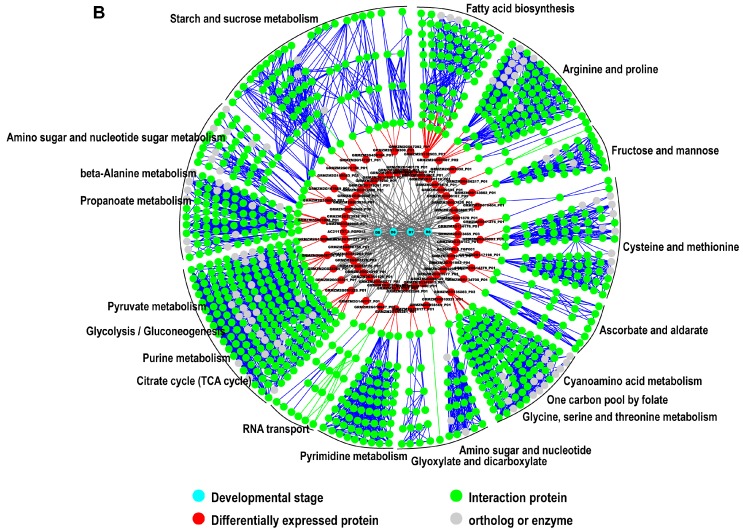
Interaction network of proteins identified by iTRAQ. The protein–protein interaction network of the differentially expressed proteins identified in this study was constructed using Kyoto Encyclopedia of Genes and Genomes (KEGG) database from the: pericarp (**A**); and endosperm (**B**), at 10, 20, 33 and 46 DAP. The interaction network was illustrated by Cytoscape software. In the network, the blue nodes represent the developmental stage, red nodes represent differentially expressed proteins identified in this study, green nodes display interaction proteins, and gray nodes represent annotated orthologs or enzymes from the KEGG database.

Hierarchical clustering-based network was constructed for proteins with at least five-fold change of abundance according to the Kyoto Encyclopedia of Genes and Genomes (KEGG) database of pathways. In total, 79 DEPs involved in 49 KEGG pathways were included, 201 and 980 potential interaction proteins were annotated in pericarp and endosperm, respectively (Figure 5, Table S4). The top10 pathways involving the most quantity of proteins in both networks were listed in Table S5.

For the pericarp protein network (Figure 5A), three DEPs with 11 first-degree interactions and 189 second-degree interactions were illustrated at three stages. The function of these interactions include 3-oxoacyl-[acyl-carrier-protein] reductase FabG and hydroxymandelonitrile lyase, which participated in the pathway of fatty acid biosynthesis, biotin metabolism and cyanoamino acid metabolism. GRMZM2G170017_P01, one of the DEPs in pericarp, was a protein related to short-chain alcohol dehydrogenases with 10 interactions.

For the endosperm protein network (Figure 5B), DEPs were red nodes in the inner cycle, and the interactions were green nodes in the outer cycle. According to the network, 76 DEPs with 97 first-degree interactions and 883 second-degree interactions were constructed at four stages. Several functional interactions were found by KEGG pathway database, such as enoyl-CoA hydratase, 3-hydroxyacyl-CoA dehydrogenase, glycine hydroxymethyltransferase, malonate-semialdehyde dehydrogenase, pyruvate kinase and UDPglucose 6-dehydrogenase. These interactions were enriched for fatty acid metabolism, starch and sucrose metabolism, glycolysis, gluconeogenesis, and pyruvate metabolism. As the DEPs in endosperm, GRMZM2G097226_P01 involved glycolysis and applied to pyruvate dehydrogenase E1 component subunit beta with seven interactions. GRMZM2G158043_P02 involved pullulanase-type starch debranching enzyme with only interaction. Taken together, it was reasonable to believe that these functional proteins participated in the interaction network during endosperm development.

### 2.6. Expression Validation by Quantitative RT-qPCR and Western Blot

**Figure 6:**
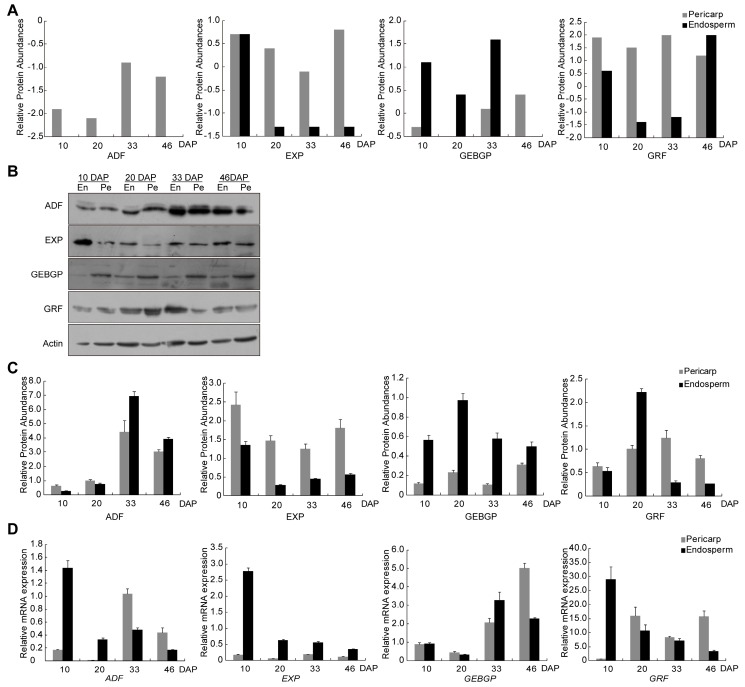
Real-time qPCR and Western blot analysis for the four proteins from iTRAQ. (**A**) Relative abundance of the protein from iTRAQ; (**B**) Accumulation of different proteins: ADF (actin-depolymerizing factor), EXP (exoglucanase1 precursor), GEBGP (glucan endo-1,3-β-glucosidase precursor), GRF (general regulatory factor) and Actin was analyzed by western-blot using specific antibodies; (**C**) Western blot analysis for the proteins after separation on SDS-PAGE using actin as the control; and (**D**) Real-time qPCR for the four proteins at mRNA expression at 10, 20, 33 and 46 DAP. The results are presented as means ± SEM pooled from three independent experiments in (**C**,**D**).

To confirm the veracity and reliability of the proteomic assays, the expression levels of four candidate proteins were measured by western blot, including ADF (actin-depolymerizing factor), EXP (exoglucanase1 precursor), GEBGP (glucan endo-1,3-β-glucosidase precursor) and GRF (general regulatory factor) (Figure 6). These four proteins followed different expression patterns at various developmental stages, which was mainly consistent with the results in proteomic analysis. In addition, the expression levels of ADF, EXP, GEBGP and GRF were more than five folds at different stages between pericarp and endosperm.

Quantitative RT-PCR analysis was also used to study the transcript levels for the same four candidate proteins. The transcript levels of these genes were inconsistent with their protein abundant. For example, the EXP was up–down-regulated from 10 to 46 DAP in endosperm between mRNA and protein. ADF shown high expressed protein at 33 DAP in endosperm, but the high transcript was found at 10 DAP endosperm by RT-qPCR. This result was consistent with several previous studies for the high non-concordance between mRNA and protein expression levels [22,23].

## 3. Discussion

In this study, the protein expression profiles of pericarp and endosperm at four grain development stages were investigated by iTRAQ using the Chinese pedigree inbred line Dan232. Using iTRAQ-based method, we compared the relative quantitative changes in protein abundance between pericarp and endosperm during maize grain development. Important DEPs involved during grain development as well as their dynamic expression pattern were revealed. Two interaction networks were constructed for identified proteins in pericarp and endosperm. Four candidate proteins were chosen to explore the relationship between transcript level and protein abundance.

### 3.1. The Role of Pericarp and Endosperm Specific Proteins during Seed Development

Large interest has been paid in seed development with particular emphasis on understanding the developmental mechanisms that underlie pattern formation in diverse tissue in the past decade [24,25,26,27,28]. Identification and functional characterization of tissue-specific proteins can help us to understand the underlying control of tissue or organ identity. To elucidate key mechanisms and regulatory networks that underlie seed development, 91 transcription factors and 1167 other seed-specific genes were identified according to the published non-seed high-throughput RNA sequencing data [26]. The endosperm acts as a critical integrator of seed growth, Nie et al. [29] performed a genomic survey to identify and functionally characterize the endosperm-specific genes in rice using Affymetrix microarray data and GO analysis. Cao et al. [30] identified 116 and 113 unique differentially expressed proteins respectively in embryo and endosperm, during grain development using two-dimensional difference gel electrophoresis (2D-DIGE)-based proteomics approach in two Chinese bread wheat cultivars. We also found many specific proteins and highly expressed proteins in the pericarp, which determine its special structure for the popcorn [21]. Despite embryo and endosperm samples were well distinguished, a powerful system for studying the pericarp development still has not been reported. In this study, the dynamic of tissue-specific protein in pericarp and endosperm combined with the prediction of their functions based on the GO database could be extremely useful for understanding their tissue-specific roles.

The functions for the 232 pericarp-specific proteins were involved in cell wall, membrane, envelope biogenesis, carbohydrate transport and metabolism, lipid transport and metabolism and signal transduction mechanisms. Among them, cell growth and cell wall expansion was indispensable, and nine correlating proteins in this functional category were all specifically expressed in the pericarp, such as GRMZM2G007404_P01, involved in the biosynthesis of UDP-xylose, a nucleotide sugar required for the synthesis of diverse plant cell wall polysaccharides including xyloglucan, expressed in pericarp [31,32]. GRMZM2G326116_P01, an isoflavone reductase-like protein, is negatively correlated with active secondary cell wall synthesis and lignification [33]. Confirming biological process at different stages has an important significance in seed development. Such as starch biosynthesis, there was no significant difference in starch content of pericarp, and its fast increase in endosperm after 12 DAP was the result of rapid increase in starch biosynthetic enzyme activities during seed development [34]. In the pericarp, pigmentation patterns within seed tissues were significantly enriched in the early phase [35], suggesting a specific process during maturation. In addition, calcium lipid binding-like protein was identified in pericarp, which was a protein involved in the fusion of synaptic vesicles to the plasma membrane [36]. These data indicated that the protein content involved in cell growth and cell wall expansion were up-regulated during early pericarp development.

For the 153 endosperm-specific proteins, most DEPs were related to amino acid transport and metabolism, nucleotide transport and metabolism, posttranslational modification, protein turnover, chaperones, translation, ribosomal structure and biogenesis. Several proteins representing complex physiological and molecular responses were activated in the stress physiology of whole multicellular eukaryotes, such as heat shock proteins (HSPs), malate/lactate dehydrogenases (MDH) and Golgi nucleoside diphosphatase (NDPase) [37,38,39].

### 3.2. Reconstruction of Protein Network with Pathway Information for Pericarp and Endosperm

A vast interaction of various proteins and the cooperation of different processes existed in seed development. Although several protein expression networks have been reported in previous research, most only represented the associations between proteins, which could not tell how proteins interact with each other in the pathway. For instance, Walley et al. [40] constructed a protein co-expression network to present the kinase–substrate relationships with protein abundance and phosphorylation data in maize. Fichlin and Feltus [41] built the gene co-expression networks between maize and rice according to the publicly available expression arrays, which incorporated both gene homology and network topology for the alignment. With the protein interactions predicted in the website of STRING, interaction network was illustrated by important myogenic proteins, which were identified by iTRAQ in Landrace (LR) and Wuzhishan (WZS) pig [42]. In our previous study, some proteins with significant role in biological and chemical processes during popcorn kernel development have been identified.

In this study, two complete protein interaction networks for pericarp and endosperm were constructed simultaneously considering the DEPs with at least 5-fold differences from a Chinese pedigree inbred Dan232 and their functions, which provided both protein abundance and predicted functional interactions collected from KEGG databases. In total, 79 DEPs were included in the networks, 3 and 76 DEPs were derived from pericarp and endosperm, respectively. The function of these proteins mainly involved fatty acid metabolism, starch and sucrose metabolism and amino sugar and nucleotide sugar metabolism. Many DEPs were known to interact with potential proteins (Table S4). Indeed, our analyses identified interaction proteins that are involved in fatty acid degradation, starch and sucrose metabolism, glycolysis and gluconeogenesis, fatty acid biosynthesis, pyrimidine metabolism, cyanoamino acid metabolism, pyruvate metabolism, citrate cycle (TCA cycle) and galactose metabolism pathways. Several key proteins and functional proteins involving the two networks were found. For example, serine carboxypeptidase (SCPs) identified in pericarp had broad functions including response to wound and environmental stress [43]. β-fructosidase wtih high expression in endosperm at all four stages might play a crucial role in the catalytic mechanism of the glycosidic bond hydrolysis [44]. The network including DEPs and their potential interaction proteins could help to understand the developmental mechanisms underlying seed development and to enhance increase grain yield in maize.

### 3.3. Differential Regulatory Mechanisms in Pericarp and Endosperm

**Figure 7:**
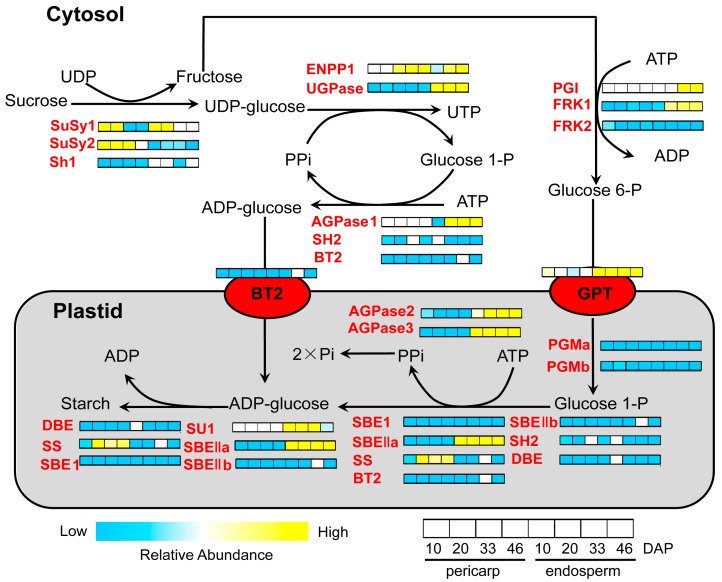
Dynmics of starch biosynthetic pathway during development stages of pericarp and endosperm. The starch biosynthesis pathway was adapted from Nougue and Corbi [52]. Heat maps depict the relative abundance of individual proteins throughout development in pericarp and endosperm. Metabolic pathway showing the major points of regulation for starch biosynthetic. The color scale shows the relative abundance of individual proteins throughout development (blue, ≤0.67-fold; yellow, ≥1.5-fold). Abbreviations for enzymes (red) are: SH2, shrunken2 (GRMZM2G429899); SuSy2, sucrose synthase 2 (GRMZM2G318780); AGPase 1, ADP glucose pyrophosphorylase (GRMZM2G163437); DBE, debranching enzyme (GRMZM2G158043); SuSy1, sucrose synthase1 (GRMZM2G152908); ENPP1, ectonucleotide pyrophosphatase (GRMZM2G143165; SU1, sugary 1 (GRMZM2G138060); AGPase 2, ADP glucose pyrophosphorylase 2 (GRMZM2G106213); Sh1, shrunken1 (GRMZM2G089713); SBE1, starch branching enzyme 1 (GRMZM2G088753); FRK1, fructokinase 1 (GRMZM2G086845); SBEa, starch branching enzyme IIa (GRMZM2G073054); BT2, brittle endosperm 2 (GRMZM2G068506); PGI, phosphoglucose isomerase (GRMZM2G065083); FRK2, fructokinase 2 (GRMZM2G051677); SBEb, Starch branching enzyme IIb (GRMZM2G032628); UGPase, UDP glucose pyrophosphorylase (GRMZM2G032003); AGPase3, ADP glucose pyrophosphorylase 3 (GRMZM2G027955); SS, starch synthase (GRMZM2G024993); PGMa, phosphoglucomutase (GRMZM2G023289); GPT, glucose-6-phosphate transmembrane transporter (GRMZM2G140614); PGMb, phosphoglucomutase (GRMZM2G109383).

In our dataset, 1401 proteins were detected in pericarp and endosperm with significant threshold values of ≥1.5 or ≤0.67 at each stage of development (Table 1 and Table S1), and demonstrating a more complex biological process in endosperm than in pericarp (Figure 3). It was previously reported that the economic and nutritional value of maize kernels is mainly approximately 75% of mature seed weight [45]. The fully development cereal endosperm consists of our main cell types: the starchy endosperm, the aleurone layer, transfer cells, and cells of the embryo-surrounding region [46]. The starchy endosperm cells represent the largest body of cells in endosperm [46], the relative abundance of proteins involved in starch and sucrose metabolism were found in pericarp and endosperm in this study (Figure 7). This enable identification and quantification of starch synthesis enzymes at each stage of development. Sucrose synthase (SuSy) and starch synthase (SS) exhibited maximal abundance in the pericarp, which corresponds with the peak time of starch synthesis [35]. Several proteins, such as UDP glucose pyrophosphorylase (UGPase), phosphoglucose isomerase (FGI), fructokinase (FRK), glucose-6-phosphate transmembrane transporter (GPT), sugary (SU), starch branching enzyme (SBE), were up-regulated in endosperm (Figure 7). Indeed, the metabolic steps from sucrose import to the synthesis of amylose and amylopectin, the starch granule components are established ranging from 12 to 40 DAP in endosperm [47,48]. Our results thereby suggest that the identification of these proteins enables targeted mutations, which are most likely regulating starch synthesis complex assembly.

Additionally, as an alternative to looking at individual transcription factor, a bZIP protein (GRMZM2G149150) might regulate vascular development [49]. In our study, the up-regulated expression of the bZIP in pericarp probably also indicates an important function of the protein in pericarp development (Table S3). Phosphorylation can also serve as important candidate regulators of tissue identity [40]. Three 14-3-3 isoforms have been identified both in pericarp and endosperm in this study. 14-3-3 protein have been implicated in diverse biological processes such as signal transduction, cell cycle control and primary metabolism and stress responses [50,51]. Taken together, these regulators involved in starch synthesis, transcription factors signal transduction were closely related to the development of pericarp and endosperm. This comprehensive comparison will be valuable for us to understanding the different molecular mechanisms between pericarp and endosperm at variable expression levels.

## 4. Materials and Methods

### 4.1. Plant Materials

The normal maize inbred line Dan232 developed from the cross between two Chinese inbred lines Lu 9 kuan and Dan340 was planted in the experimental field at the Scientific Research and Education Center of Henan Agricultural University in Zhengzhou, Henan, China (113°42′ E, 34°48′ N) during summer 2011 [21]. The plots consisted of each row of 4 m length with 0.67 m spacing between rows. Standard cultivation management practices were carried out. Each plant was self- or sib-pollinated when more than 80% of silks appeared. Fresh ears were harvested once every 2 or 3 DAP. To enable a precise comparison of the isolated grains, the upper half and about one-sixth from the bottom of the ears were cut out and discarded. Grains were isolated from the remaining parts of the ears. All samples were collected from at least six ears and pooled at each time point, for three replications. Three replicates were used for iTRAQ analysis. Some of the collected samples were frozen in liquid nitrogen immediately and stored at −80 °C, while the remaining parts were used to measure fresh and dry weight.

### 4.2. Light Microscopy and Scanning Electron Microscopy (SEM) of Cytological Sections

Light of microscopy analysis of the kernels were observed as described by Takacs et al. [53] with some modifications. We used formaldehyde-acetic acid solution that contained 10% formalin, 5% acetic acid and 50% ethanol to fix the kernels as in our previous study [21]. The samples were embedded and sectioned at 8–10 μm thickness under a Leica RM2235 Biocut (Leica, Wetzlar, Germany). The sections were stained with Safranin O and Fast Green, and photographed with a Leica DM4000B microscope (Leica, Wetzlar, Germany). Scanning electron microscopy (SEM) analysis of the kernels were prepared as described by Lending and Larkins [54]. Dry mature kernels were rifted with a blade along the longitudinal axis and spray coated with gold in an E-100 ion sputter. Goldcoated samples were examined using a S3400N microscope (Hitachi, Tokyo, Japan) at 5 kV.

### 4.3. Protein Isolation, Digestion, and iTRAQ Labeling

Both endosperm and pericarp tissues were processed as described in our previous study [21]. Proteins were labeled with the 8-plex iTRAQ reagents (Applied Biosystems, Waltham, MA, USA) according to the manufacturer’s recommendations. The samples from pericarp at 10, 20, 33 and 46 DAP were labeled with reagents 114, 115, 116 and 117, respectively. The samples from endosperm at 10, 20, 33 and 46 DAP were labeled with reagents 118, 119, 120 and 121, respectively. The reactions were performed at room temperature for 2 h. All samples with equal fractions were collected and lyophilized using a SpeedVac, and then stored at −80 °C. Two biological replicates were labeled with iTRAQ reagents.

### 4.4. LC-MS/MS Analysis, Database Search and Protein Identify

The labeled samples were dissolved in 100 μL of strong cation exchange (SCX) chromatography buffer A, which contained 10 mmol/L KH_2_PO_4_ and 25% acetonitrile, pH 2.6 on an HPLC (Shimadzu, Kyoto, Japan). Each sample was separated into 20 gradients by the SCX column (250 mm × 4.6 mm, Phenomenex, Torrance, CA, USA) according to the spike and time, and separated using a reversed-phase column (C18 coulmn, 100 mm × 75 µm, 3 µm particle sizes, 200 Å aperture size). The LC-MS/MS analysis was performed on a LTQ-Orbitrap-Velos system (Thermo Fisher Scientific, Waltham, MA, USA) after desalinization. The MS spectra were acquired across the mass range of 350–1800 *m*/*z* in the positive ion mode at a resolution of 70,000 (at 200 *m*/*z*). The isolation window was 2 *m*/*z*, and maximum ion injection times were set at 10 ms for the survey scan and 60 ms for the MS/MS scans, and the automatic gain control target values for scan mode was set to 3.6 × 10⁻^6^. The normalized collision energy for MS was set to 30 eV. The underfill ratio was defined as 0.1%.

Tandem MS spectra raw data were used to carry out ion peak detection, and peak listings were determined using the software Proteomics Tools [55]. The raw data were transformed into the mgf format as the initial files. The software Mascot 2.3.02 (Matrix Science, Available online: http://www.matrixscience.com) was used to identify and quantify proteins. Searches were performed against the maize protein database (released in January 2012) (Available online: http://www.plantgdb.org/ZmGDB/). The following search parameters were used: peptide mass tolerance ± 10 ppm, fragment mass tolerance ± 0.05 Da and maximum missed cleavages = 1. Trypsin was used as the enzyme with two missed cleavages. Fixed modifications were carbamidomethyl (C), iTRAQ8plex (N-term) and iTRAQ8plex (K). Variable modifications were Gln- > pyro-Glu (N-term Q), oxidation (M) and iTRAQ8plex (Y). Mass values were set as monoisotopic, instrument type as default and protein mass as unrestricted. The filter parameters for proteins were set as false discovery rate (FDR) ≤ 0.05, and for peptides, FDR ≤ 0.05. For relative protein quantitative analyses, the mass-to-charge ratio (*m*/*z*) 113 was performed as the control sample according to the peak area integral of mass-to-charge ratio (*m*/*z*) 114, 115, 116, 117, 118, 119, 120 and 121 reporter ions. N1 was also used as the control in our previous study [21]. The significant threshold values of ≥0.5 or ≤0.67 were regarded as differential protein expression.

### 4.5. Bioinformatic Analysis and Hierarchical Cluster Analysis of Identified Proteins

Functional annotations of differentially accumulated proteins were analyzed by matching to NCBInr (Available online: http://www.ncbi.nlm.nih.gov/) and Swiss-Prot/UniProt (Available online: http://www.uniprot.org/) databases, and further analyzed using the Gene Ontology (GO, Available online: http://www.geneontology.org) and the Cluster of Orthologous Groups of proteins (COGs, Available online: http://www.ncbi.nlm.nih.gov/COG/) databases. The metabolic pathways and signal transduction pathways of the identified proteins were analyzed according to the KEGG public database [56]. Hierarchical clusters of protein expression between the samples were performed by Cluster 3.0 software (Michael Eisen, Stanford, CA, USA) [57]. To view the clustering results, we recommend using TreeView software (Michael Eisen, Stanford, CA, USA).

### 4.6. Integrated Network Analysis on Proteome Data from Both Tissues

Proteins interactions were predicted in the website of KEGG and the interaction networks were illustrated by Cytoscape software (Paul Shannon, Seattle, WA, USA) [58]. Because the expression intensities of proteins varied over time, protein candidates were filtered using expression intensity with the criterion of a fold change (absolute value) of no less than 5 [21]. Each protein was assigned a gene model from the MaizeGDB website. To annotate these proteins, a BLAST analysis was performed using the gene model sequences as query against the well-annotated maize orthology data of the KEGG database. Based on KEGG orthology, we linked proteome data to KEGG pathways describing molecular interactions and reaction networks. Given a group of KEGG orthology entries, we used KEGG API (Available online: http://www.kegg.jp/kegg/rest/keggapi.html) to retrieve their related pathways with a URL format of http://rest.kegg.jp/link/pathway/K01778+K02725.

### 4.7. RNA Extraction and Real-Time qPCR Analysis

Four iTRAQ proteins were chosen for gene cloning based on their functions and differential expression levels in different tissue and stage samples, which were GRMZM2G060702 (ADF, actin-depolymerizing factor), GRMZM2G147687 (EXP, exoglucanase1 precursor), GRMZM2G111143 (GEBGP, glucan endo-1,3-beta-glucosidase precursor) and GRMZM2G102499 (GRF, general regulatory factor). RNA was prepared with the RNAiso reagent (Takara, Kyoto, Japan) in accordance with the manufacturer’s instructions. Total RNA was treated with DNaseI to remove genomic DNA contamination. Template cDNAs were obtained from the reverse transcription of the RNA extracted from collected samples. Real-time qPCR (RT-qPCR) was performed using a Bio-Rad real-time detection system. The reaction liquid consisted of 12.5 μL 2× SYBR Premix Ex Taq II, 1 μL PCR forward primer (10 μM), 1 μL PCR reverse primer (10 μM), 2 μL of a 1/5 dilution of the cDNA as the template, and 8.5 μL sterilized distilled water. The total volume was 25 μL. The amplification procedure was as follows: 95 °C for 3 min, then 40 cycles each of denaturation at 95 °C for 10 s, annealing at 58 °C for 20 s, and extension at 72 °C for 30 s. The β-actin gene was used as the control, and each sample was repeated three times. The area under the curve for the PCR product of each nucleotide was compared to that of its respective internal standard (*C*_t_) to determine gene expression values [59]. The primers used for RT-qPCR analysis are described in Table S6.

### 4.8. Western Blot Analysis

The same four proteins analyzed by RT-qPCR were also chosen for western blot analysis. Total proteins for all samples as in iTRAQ were extracted by a method combining the usage of Borax/PVPP/Phe (BPP) as described by [22]. Five micrograms of the actin and the total sample proteins were separated by 12% (*w*/*v*) sodium dodecyl sulfate polyacrylamide gel electrophoresis (SDS-PAGE), and transferred onto PVDF microporous membranes. Blocking for 2.5 h in TBST buffer (20 mM TrisHCl, pH 7.6, 150 mM NaCl, and 0.05% Tween 20) with 5% nonfat dry milk at room temperature, membranes were incubated with the special antibodies of the four proteins at 1:1,000 dilution for overnight at 4 °C. Custom rabbit polyclonal antisera for 4 select proteins were produced by Abmart Inc. (Shanghai, China). Following three times washing with TBST, membranes were incubated with secondary antibodies (Goat anti-Rabbit IgG-HRP, Abmart, Shanghai, China) at 1:5000 dilution for 1.5 h at room temperature away from light. After three washings with TBST, signals were detected using an ECL Western Blotting Kit (Amersham, Waltham, MA, USA) following the manufacturer’s instructions. The ratio of the validation proteins were compared to actin (#M20009, Abmart, Shanghai, China) and were densitometric measured by Image J software (NIH, Bethsda, MD, USA).

### 4.9. Statistical Analysis

All data were presented as mean values and were analyzed by ANOVA (SPSS 16.0). *p* Values < 0.05 were considered significantly different. Lists of identified proteins, the correlating RT-qPCR and Western blot experiments data are provided in the Figure 6 and Tables S1–S3.

## Figures and Tables

**Table 1:** The information of proteins identified using isobaric tags for relative and absolute quantitation (iTRAQ).

Name	10 DAP	20 DAP	33 DAP	46 DAP	Total	Nonredundance
pericarp	1237	1237	1237	1241	4952	1248
endosperm	1154	1148	1140	1145	4587	1169
Total	2391	2385	2377	2386	9539	1401
Nonredundance	1396	1398	1400	1399	1401	1401

## Supplementary Materials

Supplementary materials can be found at www.mdpi.com/1422-0067/18/9/1840/s1.
