# Supplementary material for: iTRAQ-Based Proteomics Analysis and Network Integration for Kernel Tissue Development in Maize
Source: Int J Mol Sci. 2017 Aug 24;18(9):1840. doi: 10.3390/ijms18091840 (PMC5618489; doi:10.3390/ijms18091840)
Supplement: Supplementary file 1 [file ijms-18-01840-s001.pdf]

Supplemental Figures and Tables

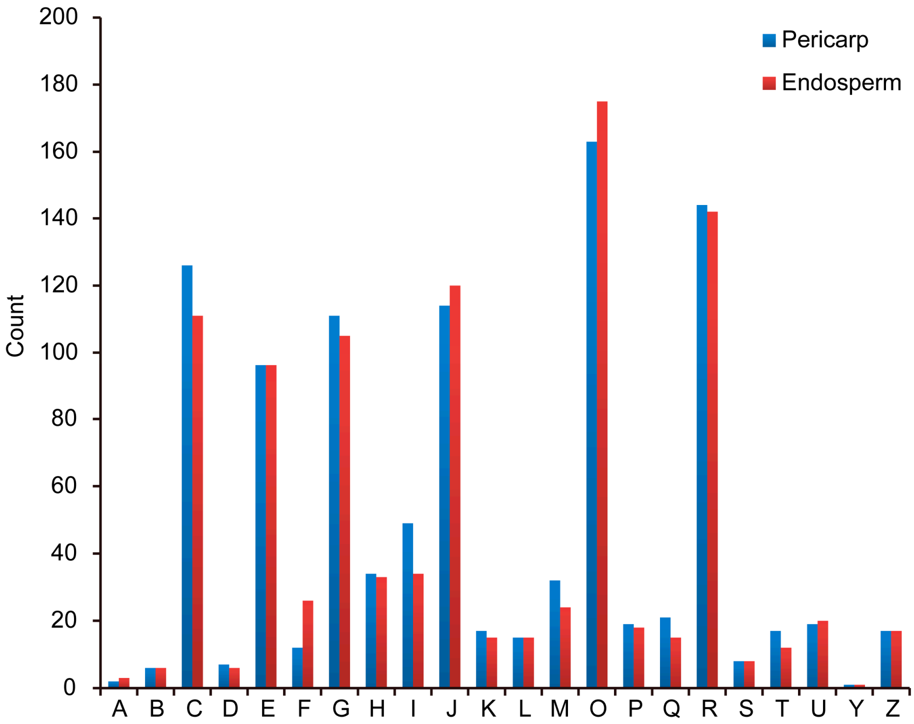

Figure S1. Cluster of orthologous groups (COG) analysis of pericarp and endosperm in Dan232.
A, RNA processing and modification; B, Chromatin structure and dynamics; C, Energy production and conversion; D, Cell cycle control, cell division, chromosome partitioning; E, Amino acid transport and metabolism; F, Nucleotide transport and metabolism; G, Carbohydrate transport and metabolism; H, Coenzyme transport and metabolism; I, Lipid transport and metabolism; J, Translation, ribosomal structure and biogenesis; K, Transcription; L, Replication, recombination and repair; M, Cell wall/membrane/envelope biogenesis; O, Posttranslational modification, protein turnover, chaperones; P, Inorganic ion transport and metabolism; Q, Secondary metabolites biosynthesis, transport and catabolism; R, General function prediction only; S, Function unknown; T, Signal transduction mechanisms; U, Intracellular trafficking, secretion, and vesicular transport; Y, Nuclear structure; Z, Cytoskeleton.

Table S1. The quantitation of identified proteins in endosperm and pericarp using iTRAQ.

Id	Identified Proteins	Accession Number	Molecular Weight	N1	D1	D2	D3	D4	D5	D6	D7	D8
1	seq=translation; coord=8:107003965..107014830:1; parent_transcript=GRMZM2G097457_T01; parent_gene=GRMZM2G097457	GRMZM2G097457_P01 (+1)	103 kDa	Ref	-0.5	0.3	-1.9	-1.9	Reference Missing	2.7	1.8	1.1
2	seq=translation; coord=9:11496011..11502772:1; parent_transcript=GRMZM2G089713_T01; parent_gene=GRMZM2G089713	GRMZM2G089713_P01 (+1)	92 kDa	Ref	-1.2	-1.1	-2	-0.4	Reference Missing	Reference Missing	-0.4	Reference Missing
3	seq=translation; coord=9:22100864..22105552:1; parent_transcript=GRMZM2G064302_T01; parent_gene=GRMZM2G064302	GRMZM2G064302_P01	48 kDa	Ref	-0.7	-0.7	-0.6	-0.3	0.5	0.9	1.2	1.3
4	seq=translation; coord=1:176837841..176844200:1; parent_transcript=GRMZM2G149751_T01; parent_gene=GRMZM2G149751	GRMZM2G149751_P01 (+2)	84 kDa	Ref	-0.7	Value Missing	-0.8	-0.6	-1.4	-1.9	-2.1	-2
5	seq=translation; coord=6:6898695..6903246:-1; parent_transcript=GRMZM2G180625_T01; parent_gene=GRMZM2G180625	GRMZM2G180625_P01 (+2)	32 kDa	Ref	-0.3	0.4	0	0.5	0.4	0.3	0.5	0.8
6	seq=translation; coord=1:296477860..296482170:1; parent_transcript=GRMZM2G040369_T01; parent_gene=GRMZM2G040369	GRMZM2G040369_P01 (+3)	94 kDa	Ref	-0.5	-1	-1	-0.5	-0.6	0.5	0.3	0.2
7	seq=translation; coord=4:14854376..14859167:-1; parent_transcript=GRMZM2G091481_T01; parent_gene=GRMZM2G091481	GRMZM2G091481_P01	57 kDa	Ref	0.1	0.9	0.7	0	0.6	1.3	Reference Missing	Reference Missing
8	seq=translation; coord=1:197267922..197271011:1; parent_transcript=GRMZM2G175423_T01; parent_gene=GRMZM2G175423	GRMZM2G175423_P01	39 kDa	Ref	-0.9	0.6	-0.6	-0.3	Reference Missing	Reference Missing	1.8	Reference Missing
9	seq=translation; coord=3:126460606..126465045:-1; parent_transcript=GRMZM2G340251_T01; parent_gene=GRMZM2G340251	GRMZM2G340251_P01 (+2)	71 kDa	Ref	Reference Missing	-0.1	-0.3	0	1.9	1.6	1.4	1.5
10	seq=translation; coord=3:207616747..207621242:-1; parent_transcript=GRMZM2G041275_T01; parent_gene=GRMZM2G041275	GRMZM2G041275_P01	59 kDa	Ref	Reference Missing	No Values	No Values	Reference Missing	-4.1	Value Missing	-5.3	Value Missing
11	seq=translation; coord=5:167751373..167752582:1; parent_transcript=GRMZM2G326111_T01; parent_gene=GRMZM2G326111	GRMZM2G326111_P01	18 kDa	Ref	-0.4	-0.8	-1.5	-2.2	-1.8	Value Missing	-3.2	-3.1
12	seq=translation; coord=4:240050091..240053865:1; parent_transcript=GRMZM2G415007_T01; parent_gene=GRMZM2G415007	GRMZM2G415007_P01	73 kDa	Ref	-0.2	-0.3	0.1	-0.1	0.1	0.2	0.7	0.9

13	seq=translation; coord=3:165722970..165725581:1; parent_transcript=GRMZM2G057823_T01; parent_gene=GRMZM2G057823	GRMZM2G057823_P01	39 kDa	Ref	-0.5	-0.5	0.2	0.4	0.4	1.6	2.1	2.6
14	seq=translation; coord=4:198369976..198374059:-1; parent_transcript=GRMZM2G012631_T01; parent_gene=GRMZM2G012631	GRMZM2G012631_P01	80 kDa	Ref	-0.1	-0.4	-0.2	-0.5	-0.5	-0.1	-0.6	-0.6
15	seq=translation; coord=4:21135870..21139019:1; parent_transcript=GRMZM2G015295_T01; parent_gene=GRMZM2G015295	GRMZM2G015295_P01 (+1)	53 kDa	Ref	-0.8	0	-0.3	0.3	0.1	-0.7	-1	-0.3
16	seq=translation; coord=6:39891824..39893787:1; parent_transcript=GRMZM2G174883_T01; parent_gene=GRMZM2G174883	GRMZM2G174883_P01	53 kDa	Ref	-1.1	-0.4	-1	-1.3	Reference Missing	Reference Missing	2.4	2
17	seq=translation; coord=4:28118892..28124783:1; parent_transcript=GRMZM2G020801_T01; parent_gene=GRMZM2G020801	GRMZM2G020801_P01	87 kDa	Ref	-0.7	-0.5	-0.5	-0.4	-0.7	-0.3	-0.2	-0.2
18	seq=translation; coord=1:53621183..53623074:-1; parent_transcript=GRMZM2G020940_T01; parent_gene=GRMZM2G020940	GRMZM2G020940_P01	38 kDa	Ref	Reference Missing	1	Reference Missing	0.7	-0.5	-1.6	-0.7	0.2
19	seq=translation; coord=9:138424253..138428598:1; parent_transcript=GRMZM2G054300_T01; parent_gene=GRMZM2G054300	GRMZM2G054300_P01 (+1)	27 kDa	Ref	-0.2	-0.2	0	-0.8	-1.2	-0.9	-1.4	-0.8
20	seq=translation; coord=5:144808460..144816059:-1; parent_transcript=GRMZM2G069542_T01; parent_gene=GRMZM2G069542	GRMZM2G069542_P01	109 kDa	Ref	-0.1	-0.8	-0.4	0.2	-0.9	-0.1	-0.3	0.2
21	seq=translation; coord=4:133103720..133107109:1; parent_transcript=GRMZM2G071630_T01; parent_gene=GRMZM2G071630	GRMZM2G071630_P01 (+2)	36 kDa	Ref	0.1	0.4	0.4	0.8	2.9	Reference Missing	Reference Missing	1.8
22	seq=translation; coord=7:146491263..146498155:-1; parent_transcript=GRMZM2G032003_T02; parent_gene=GRMZM2G032003	GRMZM2G032003_P02 (+1)	52 kDa	Ref	-0.6	-0.3	-0.6	-0.5	-0.4	0.5	0.4	0.9
23	seq=translation; coord=2:162466718..162472431:1; parent_transcript=GRMZM2G102829_T01; parent_gene=GRMZM2G102829	GRMZM2G102829_P01 (+1)	71 kDa	Ref	-1	-0.7	-0.7	-1.3	0.1	Reference Missing	0.6	0.5
24	seq=translation; coord=3:187441065..187444892:-1; parent_transcript=GRMZM2G071790_T01; parent_gene=GRMZM2G071790	GRMZM2G071790_P01 (+4)	55 kDa	Ref	1.1	0.3	-0.9	-1.2	-0.4	-2.4	-2.7	-3.2
25	seq=translation; coord=1:267887520..267894179:1; parent_transcript=GRMZM2G023289_T03; parent_gene=GRMZM2G023289	GRMZM2G023289_P03	61 kDa	Ref	-0.8	-0.6	-1	-0.5	-1.5	-1.2	-1.2	-0.9

26	seq=translation; coord=6:34438543..34440508:1; parent_transcript=GRMZM2G034724_T01; parent_gene=GRMZM2G034724	GRMZM2G034724_P01	38 kDa	Ref	-0.2	0	-0.9	-0.7	Reference Missing	Reference Missing	Reference Missing	1.7
27	seq=translation; coord=3:17824258..17828243:1; parent_transcript=GRMZM2G030784_T01; parent_gene=GRMZM2G030784	GRMZM2G030784_P01 (+1)	27 kDa	Ref	-0.8	Value Missing	-0.5	-0.9	-1.6	-4	-3	-2.9
28	seq=translation; coord=6:145987055..145992446:-1; parent_transcript=GRMZM2G306345_T03; parent_gene=GRMZM2G306345	GRMZM2G306345_P03 (+2)	67 kDa	Ref	-0.2	1.2	-0.6	-0.8	1.5	2.6	2.1	1.1
29	seq=translation; coord=5:84822215..84825942:1; parent_transcript=GRMZM2G382914_T01; parent_gene=GRMZM2G382914	GRMZM2G382914_P01 (+3)	42 kDa	Ref	-1.1	-0.9	-0.8	-1.3	0.6	1	0.6	0.8
30	seq=translation; coord=10:127370249..127371829:-1; parent_transcript=GRMZM2G005633_T02; parent_gene=GRMZM2G005633	GRMZM2G005633_P02	29 kDa	Ref	0.2	0.2	0.4	Reference Missing	1.5	1.8	3.3	4.4
31	seq=translation; coord=10:21722658..21727770:1; parent_transcript=GRMZM2G001500_T02; parent_gene=GRMZM2G001500	GRMZM2G001500_P02	75 kDa	Ref	-1.5	-1.4	-1.7	-1.3	-1.5	Reference Missing	Reference Missing	-0.5
32	seq=translation; coord=2:209289645..209292956:-1; parent_transcript=GRMZM2G113696_T01; parent_gene=GRMZM2G113696	GRMZM2G113696_P01 (+1)	17 kDa	Ref	-1.1	-1.5	-1.7	Value Missing	0.6	0.2	0	-0.1
33	seq=translation; coord=1:47172055..47173158:1; parent_transcript=GRMZM2G112524_T02; parent_gene=GRMZM2G112524	GRMZM2G112524_P02 (+1)	17 kDa	Ref	Value Missing	1.1	2	2.2	-4.2	-4	Value Missing	-2.1
34	seq=translation; coord=5:9471280..9475018:-1; parent_transcript=GRMZM2G152466_T01; parent_gene=GRMZM2G152466	GRMZM2G152466_P01 (+6)	50 kDa	Ref	0.6	-0.2	-1.7	-1.8	0.3	-1.1	-1.6	-2.7
35	seq=translation; coord=1:278130299..278132841:1; parent_transcript=GRMZM2G328500_T01; parent_gene=GRMZM2G328500	GRMZM2G328500_P01 (+2)	56 kDa	Ref	-0.9	-0.3	Value Missing	-0.7	-3.9	Value Missing	Value Missing	-5.4
36	seq=translation; coord=6:91630823..91634799:1; parent_transcript=GRMZM2G428518_T01; parent_gene=GRMZM2G428518	GRMZM2G428518_P01	97 kDa	Ref	-1.4	-0.8	0.6	-0.9	-3.9	-0.8	-0.6	0.4
37	seq=translation; coord=1:125074232..125080812:-1; parent_transcript=GRMZM2G048371_T01; parent_gene=GRMZM2G048371	GRMZM2G048371_P01 (+1)	48 kDa	Ref	0.2	0.1	0	-0.1	-0.3	0.2	0.7	0.9
38	seq=translation; coord=5:18767810..18770458:1; parent_transcript=GRMZM2G087326_T01; parent_gene=GRMZM2G087326	GRMZM2G087326_P01	41 kDa	Ref	Value Missing	Value Missing	Value Missing	0	No Values	No Values	No Values	No Values

39	seq=translation; coord=8:134521573..134531903:1; parent_transcript=GRMZM2G067985_T04; parent_gene=GRMZM2G067985	GRMZM2G067985_P04 (+13)	47 kDa	Ref	0	0.1	-0.4	-0.2	0.5	-0.2	-0.7	-1.5
40	seq=translation; coord=1:205422404..205424254:-1; parent_transcript=GRMZM2G456217_T01; parent_gene=GRMZM2G456217	GRMZM2G456217_P01	53 kDa	Ref	0.8	0.4	1.5	1.1	-3.1	-5.1	-4.4	-4.4
41	seq=translation; coord=10:1135114..1145295:1; parent_transcript=GRMZM2G057576_T01; parent_gene=GRMZM2G057576	GRMZM2G057576_P01	193 kDa	Ref	0.3	0	-0.6	-0.1	0.5	-0.4	-0.8	-1.8
42	seq=translation; coord=3:195313538..195319148:1; parent_transcript=GRMZM2G094712_T01; parent_gene=GRMZM2G094712	GRMZM2G094712_P01	50 kDa	Ref	-0.2	-0.1	-0.5	-0.2	0.1	0.6	0.6	1.1
43	seq=translation; coord=1:231336278..231341950:-1; parent_transcript=GRMZM2G415359_T02; parent_gene=GRMZM2G415359	GRMZM2G415359_P02	36 kDa	Ref	-0.4	-0.3	-0.8	-0.6	-1.5	-2.4	-1.8	-1.4
44	seq=translation; coord=6:87119198..87122939:1; parent_transcript=GRMZM2G027995_T01; parent_gene=GRMZM2G027995	GRMZM2G027995_P01 (+1)	47 kDa	Ref	-1.4	-1.8	-1	-0.1	-0.4	0	-1.9	-1.2
45	seq=translation; coord=9:87062457..87064566:1; parent_transcript=GRMZM2G153541_T06; parent_gene=GRMZM2G153541	GRMZM2G153541_P06	49 kDa	Ref	1	1.3	0.6	0.8	3	3.1	3.1	2.3
46	seq=translation; coord=9:122220190..122226863:-1; parent_transcript=GRMZM2G152908_T01; parent_gene=GRMZM2G152908	GRMZM2G152908_P01	93 kDa	Ref	1.3	1.6	-0.7	-1	1.7	2.2	Reference Missing	0
47	seq=translation; coord=7:10651271..10653006:1; parent_transcript=GRMZM2G162359_T01; parent_gene=GRMZM2G162359	GRMZM2G162359_P01	34 kDa	Ref	0.7	1.9	1.6	2	1.1	2.2	3.2	4.2
48	seq=translation; coord=4:238121524..238123238:-1; parent_transcript=GRMZM2G389768_T01; parent_gene=GRMZM2G389768	GRMZM2G389768_P01	23 kDa	Ref	-0.6	-0.9	-1.4	-2	-1.2	-1.5	Value Missing	-2.1
49	seq=translation; coord=8:170762955..170767680:-1; parent_transcript=GRMZM5G833389_T02; parent_gene=GRMZM5G833389	GRMZM5G833389_P02	61 kDa	Ref	-0.2	-0.6	0.1	-0.1	0.2	-0.3	-0.5	-1.2
50	seq=translation; coord=1:287062939..287065014:-1; parent_transcript=GRMZM2G026703_T01; parent_gene=GRMZM2G026703	GRMZM2G026703_P01	50 kDa	Ref	0.5	0.8	0.9	2.4	Reference Missing	-0.6	0.9	2.7
51	seq=translation; coord=5:59268337..59270968:1; parent_transcript=GRMZM2G084521_T01; parent_gene=GRMZM2G084521	GRMZM2G084521_P01 (+1)	23 kDa	Ref	-0.9	-1.2	-1.4	-1.6	-1.1	-0.6	-0.2	0

52	seq=translation; coord=4:235443029..235449150:-1; parent_transcript=GRMZM2G421857_T01; parent_gene=GRMZM2G421857	GRMZM2G421857_P01 (+1)	69 kDa	Ref	0	-0.1	-0.7	-0.2	0.9	0.1	-0.1	-0.4
53	seq=translation; coord=1:70424654..70429257:1; parent_transcript=GRMZM2G048324_T01; parent_gene=GRMZM2G048324	GRMZM2G048324_P01	63 kDa	Ref	-0.8	0	0	0	-1.1	0.1	0.4	0.7
54	seq=translation; coord=7:24410021..24413947:-1; parent_transcript=GRMZM2G358059_T01; parent_gene=GRMZM2G358059	GRMZM2G358059_P01	48 kDa	Ref	-1.1	-1	-1.5	-1.6	-2.5	-3.2	-3.3	-3.7
55	seq=translation; coord=9:36545543..36547162:1; parent_transcript=GRMZM2G144610_T01; parent_gene=GRMZM2G144610	GRMZM2G144610_P01	48 kDa	Ref	0.7	-0.1	-0.2	-0.9	-0.4	-0.6	Reference Missing	Reference Missing
56	seq=translation; coord=6:130343970..130350095:1; parent_transcript=GRMZM2G023232_T01; parent_gene=GRMZM2G023232	GRMZM2G023232_P01	93 kDa	Ref	0.1	0	-0.4	-0.3	1.2	0.6	0.3	0.5
57	seq=translation; coord=5:32089861..32094927:-1; parent_transcript=AC233949.1_FGT004; parent_gene=AC233949.1_FG004	AC233949.1_FGP004	94 kDa	Ref	0.2	-0.4	0.2	-0.1	-0.2	0.2	0.1	-0.3
58	seq=translation; coord=3:216414684..216424048:-1; parent_transcript=GRMZM2G429899_T01; parent_gene=GRMZM2G429899	GRMZM2G429899_P01	74 kDa	Ref	-0.3	-1	0	-0.4	Reference Missing	-1.4	-1.8	-2
59	seq=translation; coord=5:21697782..21703415:1; parent_transcript=GRMZM2G153815_T01; parent_gene=GRMZM2G153815	GRMZM2G153815_P01	73 kDa	Ref	-0.4	-0.5	-0.4	-0.5	0	-0.9	-0.8	-0.6
60	seq=translation; coord=2:71110426..71138423:1; parent_transcript=GRMZM2G024933_T02; parent_gene=GRMZM2G024933	GRMZM2G024933_P02	17 kDa	Ref	-1.2	-0.6	-0.6	-0.5	-1	0.3	0.7	Reference Missing
61	seq=translation; coord=1:45512802..45515978:-1; parent_transcript=GRMZM2G087186_T01; parent_gene=GRMZM2G087186	GRMZM2G087186_P01 (+1)	65 kDa	Ref	-0.7	-0.4	-0.7	-0.3	-1.4	-1.7	-1	-0.9
62	seq=translation; coord=6:162882094..162884481:1; parent_transcript=GRMZM2G038032_T01; parent_gene=GRMZM2G038032	GRMZM2G038032_P01 (+1)	36 kDa	Ref	0	-0.5	-1.1	-0.4	1.6	-0.1	-0.1	-0.9
63	seq=translation; coord=6:104066600..104071298:1; parent_transcript=GRMZM2G122871_T01; parent_gene=GRMZM2G122871	GRMZM2G122871_P01	47 kDa	Ref	0.5	1.1	-0.2	0.2	1	1.8	0.9	1.5
64	seq=translation; coord=5:189820639..189827796:1; parent_transcript=GRMZM2G162688_T01; parent_gene=GRMZM2G162688	GRMZM2G162688_P01	65 kDa	Ref	1.3	1.3	0.9	-0.2	2.3	2.1	2.2	3.3

65	seq=translation; coord=8:147686846..147691734:-1; parent_transcript=GRMZM2G432128_T01; parent_gene=GRMZM2G432128	GRMZM2G432128_P01	46 kDa	Ref	-1.8	-1.3	-0.9	-0.5	-1.6	-1.9	-1.9	-1.4
66	seq=translation; coord=6:74407967..74410838:1; parent_transcript=GRMZM2G108780_T01; parent_gene=GRMZM2G108780	GRMZM2G108780_P01	14 kDa	Ref	Value Missing	-0.3	-0.6	-1.4	-3	-3	Value Missing	Value Missing
67	seq=translation; coord=1:273983281..273986931:-1; parent_transcript=GRMZM2G442658_T02; parent_gene=GRMZM2G442658	GRMZM2G442658_P02 (+1)	41 kDa	Ref	0	0.7	0.2	0.9	1.3	1.3	1.5	1.2
68	seq=translation; coord=1:178476591..178478157:-1; parent_transcript=GRMZM2G080603_T01; parent_gene=GRMZM2G080603	GRMZM2G080603_P01 (+2)	15 kDa	Ref	0.6	0.5	0.3	-0.6	0.4	-0.3	-0.3	0.5
69	seq=translation; coord=8:109163217..109164387:-1; parent_transcript=GRMZM2G063536_T01; parent_gene=GRMZM2G063536	GRMZM2G063536_P01	33 kDa	Ref	-0.4	0.7	0	0.3	-5.4	Reference Missing	Reference Missing	-1.6
70	seq=translation; coord=6:165631098..165635858:-1; parent_transcript=GRMZM2G154595_T01; parent_gene=GRMZM2G154595	GRMZM2G154595_P01	35 kDa	Ref	-0.1	Value Missing	-0.2	Value Missing	-3.5	-3.9	-3.5	-3.3
71	seq=translation; coord=4:94432879..94438375:1; parent_transcript=AC207890.3_FGT002; parent_gene=AC207890.3_FG002	AC207890.3_FGP002	108 kDa	Ref	No Values	No Values	No Values	No Values	0.5	0.6	0.7	0.9
72	seq=translation; coord=2:5064059..5067153:-1; parent_transcript=GRMZM2G081886_T01; parent_gene=GRMZM2G081886	GRMZM2G081886_P01	65 kDa	Ref	-0.5	-0.5	-0.4	0	1.2	0.2	-0.1	-0.1
73	seq=translation; coord=1:264200949..264205758:1; parent_transcript=GRMZM2G109130_T01; parent_gene=GRMZM2G109130	GRMZM2G109130_P01	96 kDa	Ref	0	0.9	1.5	2.2	No Values	No Values	No Values	No Values
74	seq=translation; coord=6:57744396..57747092:-1; parent_transcript=GRMZM2G127798_T01; parent_gene=GRMZM2G127798	GRMZM2G127798_P01 (+2)	53 kDa	Ref	0	-0.3	0.3	0.3	3.8	3.8	3.8	2.6
75	seq=translation; coord=7:120173851..120175165:1; parent_transcript=GRMZM2G138689_T01; parent_gene=GRMZM2G138689	GRMZM2G138689_P01	36 kDa	Ref	-1.2	0.6	-0.3	-1.1	-3.9	-3.7	Reference Missing	-4
76	seq=translation; coord=2:40588194..40589174:-1; parent_transcript=GRMZM2G102356_T01; parent_gene=GRMZM2G102356	GRMZM2G102356_P01	17 kDa	Ref	-1.1	-2.6	-2.8	-3	-1	0.1	0.1	-0.2
77	seq=translation; coord=8:133171791..133174888:1; parent_transcript=GRMZM5G855672_T02; parent_gene=GRMZM5G855672	GRMZM5G855672_P02	23 kDa	Ref	0.2	0.2	0.3	-1.2	-3.6	-1.8	-1.8	-0.2

78	seq=translation; coord=6:3570233..3573134:1; parent_transcript=GRMZM2G051677_T01; parent_gene=GRMZM2G051677	GRMZM2G051677_P01 (+1)	35 kDa	Ref	-0.2	-0.5	-0.5	-0.4	-1	-2.8	-3.5	-3.8
79	seq=translation; coord=2:184127551..184138715:-1; parent_transcript=GRMZM2G130440_T02; parent_gene=GRMZM2G130440	GRMZM2G130440_P02	54 kDa	Ref	-0.9	-0.7	-1	-0.2	0.5	0.9	0.5	1.2
80	seq=translation; coord=4:241239335..241241698:1; parent_transcript=GRMZM2G134806_T01; parent_gene=GRMZM2G134806	GRMZM2G134806_P01 (+1)	40 kDa	Ref	1.8	1.6	1.1	0.6	-0.2	-0.3	-0.4	1.9
81	seq=translation; coord=4:13395375..13398777:-1; parent_transcript=GRMZM2G098346_T01; parent_gene=GRMZM2G098346	GRMZM2G098346_P01	41 kDa	Ref	-0.3	0.3	-0.4	0.3	-1.6	-0.4	-1.4	-1.1
82	seq=translation; coord=2:41302953..41306124:-1; parent_transcript=GRMZM2G102499_T02; parent_gene=GRMZM2G102499	GRMZM2G102499_P02 (+2)	32 kDa	Ref	1.9	1.5	2	1.2	0.6	-1.4	-1.2	2
83	seq=translation; coord=7:168785597..168786938:1; parent_transcript=GRMZM2G129761_T01; parent_gene=GRMZM2G129761	GRMZM2G129761_P01	28 kDa	Ref	0.2	2.1	0.5	1.1	-1.9	Reference Missing	-1.1	Reference Missing
84	seq=translation; coord=3:186793464..186794596:-1; parent_transcript=GRMZM2G092474_T01; parent_gene=GRMZM2G092474	GRMZM2G092474_P01	23 kDa	Ref	0.3	0.3	Reference Missing	4.4	1.8	2.7	3.5	Reference Missing
85	seq=translation; coord=4:105179813..105180991:-1; parent_transcript=GRMZM2G162388_T01; parent_gene=GRMZM2G162388	GRMZM2G162388_P01	18 kDa	Ref	Value Missing	-2.8	-3.1	Value Missing	Value Missing	-4.4	-4.5	-4.8
86	seq=translation; coord=10:60080812..60087313:-1; parent_transcript=GRMZM2G181192_T01; parent_gene=GRMZM2G181192	GRMZM2G181192_P01 (+2)	35 kDa	Ref	-0.8	-0.5	-0.4	-0.5	-0.2	1.2	1.9	2.7
87	seq=translation; coord=9:129274385..129278107:-1; parent_transcript=GRMZM2G058522_T01; parent_gene=GRMZM2G058522	GRMZM2G058522_P01 (+5)	15 kDa	Ref	-1	-1	-1	-1.3	Value Missing	Value Missing	Value Missing	Value Missing
88	seq=translation; coord=8:168955391..168958754:-1; parent_transcript=GRMZM2G310431_T01; parent_gene=GRMZM2G310431	GRMZM2G310431_P01	71 kDa	Ref	-0.3	0.1	0.1	-0.7	1	2.2	2.5	2.4
89	seq=translation; coord=6:115191842..115198242:-1; parent_transcript=GRMZM2G059151_T01; parent_gene=GRMZM2G059151	GRMZM2G059151_P01	61 kDa	Ref	0.1	-0.1	-0.3	-0.3	-1.3	-1.8	-1.8	-2.2
90	seq=translation; coord=5:179010381..179015571:1; parent_transcript=GRMZM2G013324_T01; parent_gene=GRMZM2G013324	GRMZM2G013324_P01	81 kDa	Ref	-1.1	-0.4	-1.9	-2.6	-0.3	-1.9	-1.4	-1.4

91	seq=translation; coord=4:240742463..240747734:-1; parent_transcript=GRMZM2G015989_T01; parent_gene=GRMZM2G015989	GRMZM2G015989_P01 (+1)	64 kDa	Ref	Value Missing	Value Missing	-1.9	-2.2	-2.1	-1.9	Reference Missing	-2.5
92	seq=translation; coord=7:173553684..173555507:-1; parent_transcript=GRMZM2G469380_T02; parent_gene=GRMZM2G469380	GRMZM2G469380_P02	38 kDa	Ref	1	1.2	0.8	0	4.1	1.3	1.4	1.7
93	seq=translation; coord=4:58954361..58960521:-1; parent_transcript=GRMZM2G068506_T01; parent_gene=GRMZM2G068506	GRMZM2G068506_P01	52 kDa	Ref	-2.1	-1.7	-0.8	-1.3	-4.6	-2.6	Reference Missing	-2.5
94	seq=translation; coord=6:83777893..83783487:1; parent_transcript=GRMZM2G141931_T01; parent_gene=GRMZM2G141931	GRMZM2G141931_P01	93 kDa	Ref	0.5	0.2	0.5	0.1	No Values	No Values	No Values	No Values
95	seq=translation; coord=5:155951379..155960766:1; parent_transcript=GRMZM2G027875_T01; parent_gene=GRMZM2G027875	GRMZM2G027875_P01	96 kDa	Ref	-0.2	-0.5	-0.6	-0.3	-0.1	-0.2	-0.1	0.2
96	seq=translation; coord=4:160954463..160955623:1; parent_transcript=GRMZM2G342515_T01; parent_gene=GRMZM2G342515	GRMZM2G342515_P01	17 kDa	Ref	0.5	-0.1	0.2	0	-0.9	-5.1	Value Missing	-4.5
97	seq=translation; coord=3:202432697..202435947:1; parent_transcript=GRMZM2G055276_T01; parent_gene=GRMZM2G055276	GRMZM2G055276_P01	40 kDa	Ref	2	0.5	0.7	0.4	Reference Missing	Reference Missing	Reference Missing	Reference Missing
98	seq=translation; coord=3:213623450..213627174:-1; parent_transcript=GRMZM2G004382_T01; parent_gene=GRMZM2G004382	GRMZM2G004382_P01	63 kDa	Ref	-0.8	-0.6	-1.4	-1.2	0.4	0.1	0.1	0.6
99	seq=translation; coord=4:220508652..220512101:1; parent_transcript=GRMZM2G027378_T01; parent_gene=GRMZM2G027378	GRMZM2G027378_P01 (+1)	17 kDa	Ref	-0.7	-0.7	-0.6	-1.9	-0.9	-0.3	-0.7	-0.5
100	seq=translation; coord=5:29990567..29996686:1; parent_transcript=GRMZM2G167505_T01; parent_gene=GRMZM2G167505	GRMZM2G167505_P01 (+2)	45 kDa	Ref	0.2	-0.4	-0.3	-1.2	-0.2	-2.4	-1.6	-0.8
101	seq=translation; coord=5:186517098..186522957:-1; parent_transcript=GRMZM2G540538_T01; parent_gene=GRMZM2G540538	GRMZM2G540538_P01	37 kDa	Ref	-1.4	-0.6	-0.5	-0.1	0.2	-0.4	-0.8	-0.3
102	seq=translation; coord=9:141825338..141830182:1; parent_transcript=GRMZM5G870932_T01; parent_gene=GRMZM5G870932	GRMZM5G870932_P01	73 kDa	Ref	0.1	0	1.1	Reference Missing	1.6	Reference Missing	2.7	3.1
103	seq=translation; coord=1:177027402..177032403:1; parent_transcript=GRMZM2G005887_T01; parent_gene=GRMZM2G005887	GRMZM2G005887_P01 (+2)	34 kDa	Ref	0.5	0.7	0.8	0.6	-1	-0.1	-0.2	-0.3

104	seq=translation; coord=2:33534101..33535449:1; parent_transcript=GRMZM2G051943_T01; parent_gene=GRMZM2G051943	GRMZM2G051943_P01	29 kDa	Ref	0.3	0.4	1.8	Reference Missing	Reference Missing	-1.7	0.1	Reference Missing
105	seq=translation; coord=1:292089538..292095812:-1; parent_transcript=GRMZM2G347056_T01; parent_gene=GRMZM2G347056	GRMZM2G347056_P01	66 kDa	Ref	-0.4	-0.3	-0.6	-0.1	0	-1.2	-1.2	-1
106	seq=translation; coord=7:155357370..155360570:1; parent_transcript=GRMZM2G058310_T01; parent_gene=GRMZM2G058310	GRMZM2G058310_P01	55 kDa	Ref	-0.5	-0.8	-0.7	0	2.9	2.9	2.9	2.8
107	seq=translation; coord=4:32249665..32251536:-1; parent_transcript=AC196475.3_FGT004; parent_gene=AC196475.3_FG004	AC196475.3_FGP004	40 kDa	Ref	-0.3	1.1	0.7	1.6	-1.8	-2.1	-2.2	-2.5
108	seq=translation; coord=6:122797861..122838407:1; parent_transcript=GRMZM2G032505_T02; parent_gene=GRMZM2G032505	GRMZM2G032505_P02 (+1)	47 kDa	Ref	0.1	-0.3	0.1	-0.2	-0.5	0.8	0.2	-0.5
109	seq=translation; coord=3:151819389..151821475:1; parent_transcript=GRMZM2G125032_T01; parent_gene=GRMZM2G125032	GRMZM2G125032_P01	36 kDa	Ref	0.1	Value Missing	0.4	1.7	0	0	1.6	2.5
110	seq=translation; coord=3:56281784..56287937:1; parent_transcript=GRMZM2G136106_T01; parent_gene=GRMZM2G136106	GRMZM2G136106_P01	60 kDa	Ref	-0.1	0.8	0.2	0.7	1.5	1.3	1.1	1.2
111	seq=translation; coord=5:213541443..213545321:-1; parent_transcript=GRMZM2G178958_T01; parent_gene=GRMZM2G178958	GRMZM2G178958_P01	62 kDa	Ref	-0.3	-0.5	-0.5	-0.5	-0.9	-1.4	-1	-1.2
112	seq=translation; coord=10:142329418..142336291:1; parent_transcript=GRMZM2G114182_T01; parent_gene=GRMZM2G114182	GRMZM2G114182_P01	150 kDa	Ref	-0.3	-0.6	-0.5	-1.1	0.2	0.3	0.2	-0.2
113	seq=translation; coord=1:12140829..12142573:1; parent_transcript=GRMZM2G300801_T01; parent_gene=GRMZM2G300801	GRMZM2G300801_P01	45 kDa	Ref	0	0.3	0.1	0.5	0.3	-0.1	-0.3	-0.5
114	seq=translation; coord=5:168451664..168468750:-1; parent_transcript=GRMZM2G032628_T01; parent_gene=GRMZM2G032628	GRMZM2G032628_P01	91 kDa	Ref	-0.9	-0.6	-1.9	-1.5	-1.9	-1.1	Reference Missing	-2.7
115	seq=translation; coord=8:21816689..21823022:1; parent_transcript=GRMZM2G032049_T01; parent_gene=GRMZM2G032049	GRMZM2G032049_P01	90 kDa	Ref	0.3	-0.4	-0.3	-0.9	-4.9	-3.3	-3.2	-2.7
116	seq=translation; coord=1:299988513..299993142:-1; parent_transcript=GRMZM2G434173_T01; parent_gene=GRMZM2G434173	GRMZM2G434173_P01	61 kDa	Ref	-1.2	-0.4	-0.7	-0.6	-2.2	-2.5	-2.5	-2.7

117	seq=translation; coord=5:65023010..65024952:-1; parent_transcript=GRMZM2G005771_T01; parent_gene=GRMZM2G005771	GRMZM2G005771_P01 (+1)	18 kDa	Ref	-1.5	-0.9	-0.7	-0.9	-2.1	-1.7	-1.4	-1.2
118	seq=translation; coord=10:1725712..1731899:-1; parent_transcript=GRMZM2G130062_T01; parent_gene=GRMZM2G130062	GRMZM2G130062_P01	67 kDa	Ref	-0.7	0.5	-0.1	0.8	-6.1	-5.1	-4.9	-4.5
119	seq=translation; coord=3:31898226..31901626:-1; parent_transcript=GRMZM2G120304_T01; parent_gene=GRMZM2G120304	GRMZM2G120304_P01 (+1)	33 kDa	Ref	0.5	-0.2	-0.2	-0.5	-1.5	-2.1	-1.7	-1.7
120	seq=translation; coord=4:20543360..20550099:-1; parent_transcript=GRMZM2G101446_T01; parent_gene=GRMZM2G101446	GRMZM2G101446_P01 (+1)	19 kDa	Ref	0	-0.6	0.8	-0.7	-0.9	0.2	-0.1	1.1
121	seq=translation; coord=1:297016125..297019051:-1; parent_transcript=GRMZM2G352415_T01; parent_gene=GRMZM2G352415	GRMZM2G352415_P01 (+2)	35 kDa	Ref	1.2	1.4	0.6	0.7	0.2	-0.2	-0.6	-0.9
122	seq=translation; coord=3:146522696..146524904:-1; parent_transcript=GRMZM2G103342_T01; parent_gene=GRMZM2G103342	GRMZM2G103342_P01	38 kDa	Ref	0.2	0	1.7	3.6	No Values	No Values	No Values	No Values
123	seq=translation; coord=2:16219232..16221297:1; parent_transcript=GRMZM2G003752_T01; parent_gene=GRMZM2G003752	GRMZM2G003752_P01	44 kDa	Ref	-0.5	0	-1.1	-1.2	0	-0.9	0	0.4
124	seq=translation; coord=6:160621048..160624717:1; parent_transcript=GRMZM2G360681_T01; parent_gene=GRMZM2G360681	GRMZM2G360681_P01	101 kDa	Ref	-0.9	-1.3	-1	-1.3	-4.7	-2.6	-2.5	-2.3
125	seq=translation; coord=9:11079860..11085004:-1; parent_transcript=GRMZM2G127609_T01; parent_gene=GRMZM2G127609	GRMZM2G127609_P01 (+6)	26 kDa	Ref	-0.7	-1.1	-1.4	-2	0.8	0.8	Reference Missing	1.3
126	seq=translation; coord=2:165615333..165620418:-1; parent_transcript=GRMZM2G084881_T01; parent_gene=GRMZM2G084881	GRMZM2G084881_P01	47 kDa	Ref	-0.3	-0.2	-0.4	0	1.5	0.6	0.3	0.8
127	seq=translation; coord=8:158089079..158099475:-1; parent_transcript=GRMZM2G180988_T02; parent_gene=GRMZM2G180988	GRMZM2G180988_P02 (+1)	108 kDa	Ref	-0.4	-0.6	-0.6	-0.6	-0.1	0	-0.1	-0.6
128	seq=translation; coord=2:167237699..167240039:1; parent_transcript=GRMZM2G352855_T01; parent_gene=GRMZM2G352855	GRMZM2G352855_P01 (+3)	49 kDa	Ref	0.4	0.8	1.7	2.6	No Values	No Values	No Values	No Values
129	seq=translation; coord=7:10287294..10291100:1; parent_transcript=GRMZM2G100403_T02; parent_gene=GRMZM2G100403	GRMZM2G100403_P02	44 kDa	Ref	0.7	0.5	0.6	0.4	1.1	0.4	0.4	-0.6

130	seq=translation; coord=1:12514731..12519380:1; parent_transcript=GRMZM2G165998_T01; parent_gene=GRMZM2G165998	GRMZM2G165998_P01 (+1)	23 kDa	Ref	-0.8	-1	-0.1	-0.5	-1.5	-0.8	-0.9	-0.1
131	seq=translation; coord=9:19393494..19397648:-1; parent_transcript=GRMZM2G140051_T01; parent_gene=GRMZM2G140051	GRMZM2G140051_P01 (+1)	43 kDa	Ref	0.7	-0.1	-0.2	-1.3	0	-1.4	-1.7	-1.5
132	seq=translation; coord=5:146841857..146847013:-1; parent_transcript=GRMZM2G146677_T01; parent_gene=GRMZM2G146677	GRMZM2G146677_P01	48 kDa	Ref	0.2	0.1	0.2	0.8	-0.4	0.1	0.1	0
133	seq=translation; coord=1:258878414..258878962:1; parent_transcript=GRMZM2G704005_T01; parent_gene=GRMZM2G704005	GRMZM2G704005_P01	20 kDa	Ref	-0.9	-0.3	0	-0.7	-4.1	-2.6	-1.9	-1.1
134	seq=translation; coord=10:96330046..96338713:-1; parent_transcript=GRMZM5G858454_T02; parent_gene=GRMZM5G858454	GRMZM5G858454_P02	99 kDa	Ref	0	-0.3	-0.7	-0.2	1.7	1.9	2	2.2
135	seq=translation; coord=2:218589206..218592127:-1; parent_transcript=GRMZM2G140667_T01; parent_gene=GRMZM2G140667	GRMZM2G140667_P01 (+1)	31 kDa	Ref	0.2	0.1	0.1	-0.4	-2	-1	-1	-0.8
136	seq=translation; coord=6:145087390..145092201:1; parent_transcript=GRMZM2G179301_T02; parent_gene=GRMZM2G179301	GRMZM2G179301_P02 (+1)	26 kDa	Ref	-0.2	-0.1	0.1	-0.4	-0.7	0.4	1.1	1.8
137	seq=translation; coord=1:79067709..79069201:-1; parent_transcript=GRMZM2G148925_T01; parent_gene=GRMZM2G148925	GRMZM2G148925_P01	14 kDa	Ref	0.4	-0.5	1.5	-0.1	-2.8	-2.3	-1.8	0.5
138	seq=translation; coord=6:113557759..113560731:-1; parent_transcript=GRMZM2G701082_T04; parent_gene=GRMZM2G701082	GRMZM2G701082_P04 (+2)	36 kDa	Ref	-0.6	-0.9	-0.8	-1.2	-4.6	-5.1	-5.8	-6.2
139	seq=translation; coord=3:224673584..224677732:1; parent_transcript=GRMZM2G034985_T01; parent_gene=GRMZM2G034985	GRMZM2G034985_P01 (+1)	41 kDa	Ref	0.2	0.4	0.4	0.7	-4	-4.3	-4.3	-4.6
140	seq=translation; coord=5:15546617..15552849:-1; parent_transcript=GRMZM2G112149_T01; parent_gene=GRMZM2G112149	GRMZM2G112149_P01 (+2)	85 kDa	Ref	-0.2	-0.1	0.3	0.4	No Values	No Values	No Values	No Values
141	seq=translation; coord=2:37325095..37329643:1; parent_transcript=GRMZM2G163471_T01; parent_gene=GRMZM2G163471	GRMZM2G163471_P01	57 kDa	Ref	2.5	1.8	1.8	1.3	Reference Missing	Reference Missing	Reference Missing	0.7
142	seq=translation; coord=7:18473070..18475522:1; parent_transcript=GRMZM2G168149_T01; parent_gene=GRMZM2G168149	GRMZM2G168149_P01	30 kDa	Ref	0.7	0.5	-0.2	0.6	3.6	2.9	2.7	1.9

143	seq=translation; coord=6:44382950..44385913:-1; parent_transcript=GRMZM2G087570_T01; parent_gene=GRMZM2G087570	GRMZM2G087570_P01	26 kDa	Ref	-0.3	0	-0.1	-0.7	-1.2	-1.2	-1	-0.7
144	seq=translation; coord=5:186677004..186680745:-1; parent_transcript=GRMZM2G074604_T01; parent_gene=GRMZM2G074604	GRMZM2G074604_P01	75 kDa	Ref	-0.1	0.3	0.6	1.4	No Values	No Values	No Values	No Values
145	seq=translation; coord=9:78409591..78421558:1; parent_transcript=GRMZM2G094497_T01; parent_gene=GRMZM2G094497	GRMZM2G094497_P01 (+2)	54 kDa	Ref	-0.6	-0.4	-0.9	-0.6	1	-0.1	0	-0.2
146	seq=translation; coord=3:161729177..161732426:-1; parent_transcript=GRMZM2G103812_T01; parent_gene=GRMZM2G103812	GRMZM2G103812_P01 (+1)	54 kDa	Ref	-0.4	-0.3	-0.5	0.1	-0.9	-0.7	-1.5	-1.9
147	seq=translation; coord=1:204259324..204264422:-1; parent_transcript=AC234528.1_FGT005; parent_gene=AC234528.1_FG005	AC234528.1_FGP005 (+1)	63 kDa	Ref	-0.1	-0.2	-0.3	-0.2	0	-0.1	0	-0.3
148	seq=translation; coord=7:149915726..149918129:-1; parent_transcript=GRMZM2G134797_T02; parent_gene=GRMZM2G134797	GRMZM2G134797_P02	17 kDa	Ref	-1.6	-1.1	-0.6	-1.8	-0.9	1.5	2	3.1
149	seq=translation; coord=5:212375127..212379000:-1; parent_transcript=GRMZM2G110185_T02; parent_gene=GRMZM2G110185	GRMZM2G110185_P02 (+1)	48 kDa	Ref	0.1	-0.1	-0.2	-0.1	-0.2	0.4	0.2	-0.5
150	seq=translation; coord=2:106286427..106287698:1; parent_transcript=GRMZM2G045809_T01; parent_gene=GRMZM2G045809	GRMZM2G045809_P01	25 kDa	Ref	0.8	2.3	Reference Missing	Reference Missing	No Values	No Values	No Values	No Values
151	seq=translation; coord=7:165383929..165386707:-1; parent_transcript=GRMZM2G031545_T01; parent_gene=GRMZM2G031545	GRMZM2G031545_P01 (+2)	25 kDa	Ref	-1	-1.5	-0.3	-1.3	-0.5	0.1	0.1	0.7
152	seq=translation; coord=6:125241154..125242106:-1; parent_transcript=GRMZM2G357296_T01; parent_gene=GRMZM2G357296	GRMZM2G357296_P01	18 kDa	Ref	0.4	0.2	-0.1	-0.3	0.4	-1.7	-1	-2.4
153	seq=translation; coord=9:98777728..98779013:-1; parent_transcript=GRMZM2G036921_T01; parent_gene=GRMZM2G036921	GRMZM2G036921_P01	24 kDa	Ref	-1.3	-1	-1.5	-2.3	-1.4	-1.6	-1.5	-1.6
154	seq=translation; coord=3:146602562..146604776:1; parent_transcript=GRMZM2G171430_T01; parent_gene=GRMZM2G171430	GRMZM2G171430_P01	10 kDa	Ref	-0.7	-0.9	-1.3	-2.4	-0.4	0.9	0.6	0.6
155	seq=translation; coord=4:149167730..149173878:1; parent_transcript=GRMZM2G063850_T01; parent_gene=GRMZM2G063850	GRMZM2G063850_P01	65 kDa	Ref	1	1	0.7	0	-0.7	-1.6	-2	-0.3

156	seq=translation; coord=5:1488844..1491571:1; parent_transcript=GRMZM2G125271_T01; parent_gene=GRMZM2G125271	GRMZM2G125271_P01	30 kDa	Ref	No Values	No Values	No Values	No Values	1	1.4	0.9	0.2
157	seq=translation; coord=9:142495842..142498679:-1; parent_transcript=GRMZM2G067303_T01; parent_gene=GRMZM2G067303	GRMZM2G067303_P01 (+3)	14 kDa	Ref	0.9	0.5	0.1	-0.7	1.8	1	1	0.2
158	seq=translation; coord=8:162236975..162239554:-1; parent_transcript=GRMZM2G042118_T01; parent_gene=GRMZM2G042118	GRMZM2G042118_P01	16 kDa	Ref	0.4	-0.7	-0.4	-1	3	0	0.7	0.3
159	seq=translation; coord=1:258363419..258365809:1; parent_transcript=GRMZM2G067919_T02; parent_gene=GRMZM2G067919	GRMZM2G067919_P02	65 kDa	Ref	0.2	0.6	1.3	3.5	-1	-1.2	Reference Missing	Reference Missing
160	seq=translation; coord=8:18231012..18236007:-1; parent_transcript=GRMZM2G107639_T01; parent_gene=GRMZM2G107639	GRMZM2G107639_P01 (+1)	44 kDa	Ref	-0.4	-0.1	-0.2	-0.3	-0.9	-0.1	0.1	0.2
161	seq=translation; coord=5:37229486..37234332:-1; parent_transcript=GRMZM2G088064_T03; parent_gene=GRMZM2G088064	GRMZM2G088064_P03	53 kDa	Ref	0.1	0.2	-0.3	0.1	1.6	1.5	1.3	1.1
162	seq=translation; coord=6:34430048..34433142:1; parent_transcript=GRMZM2G035502_T01; parent_gene=GRMZM2G035502	GRMZM2G035502_P01	23 kDa	Ref	-0.2	-0.7	-0.3	-1.3	Value Missing	-1	-1	0.4
163	seq=translation; coord=4:70066543..70068959:-1; parent_transcript=GRMZM2G073079_T01; parent_gene=GRMZM2G073079	GRMZM2G073079_P01 (+1)	29 kDa	Ref	0.4	1.7	1.9	1.9	0	0	0.5	0.8
164	seq=translation; coord=7:8218240..8220274:1; parent_transcript=GRMZM2G170397_T01; parent_gene=GRMZM2G170397	GRMZM2G170397_P01 (+2)	19 kDa	Ref	-0.9	-1	-1.9	-2.2	0.2	-1.1	-1.2	-1.2
165	seq=translation; coord=9:148445803..148448625:-1; parent_transcript=GRMZM2G078441_T01; parent_gene=GRMZM2G078441	GRMZM2G078441_P01	71 kDa	Ref	0.2	1.5	0.7	1	-0.2	Reference Missing	1.3	1.5
166	seq=translation; coord=3:218323126..218325508:1; parent_transcript=GRMZM2G176903_T01; parent_gene=GRMZM2G176903	GRMZM2G176903_P01 (+1)	38 kDa	Ref	0.4	0.1	0.1	0	1	0.9	1.1	1.5
167	seq=translation; coord=5:205777032..205781614:-1; parent_transcript=GRMZM2G140150_T01; parent_gene=GRMZM2G140150	GRMZM2G140150_P01	43 kDa	Ref	1.3	0.3	1.2	0.4	No Values	No Values	No Values	No Values
168	seq=translation; coord=5:63564993..63569468:1; parent_transcript=GRMZM2G088212_T01; parent_gene=GRMZM2G088212	GRMZM2G088212_P01	57 kDa	Ref	0	0	0	-0.1	1.6	2.1	1.3	1.2

169	seq=translation; coord=6:161659850..161663090:-1; parent_transcript=GRMZM2G121186_T01; parent_gene=GRMZM2G121186	GRMZM2G121186_P01	43 kDa	Ref	0.4	0.3	0.2	-0.4	0	-0.9	-0.9	-0.2
170	seq=translation; coord=4:27749082..27753340:1; parent_transcript=GRMZM2G131943_T01; parent_gene=GRMZM2G131943	GRMZM2G131943_P01 (+3)	74 kDa	Ref	-1.5	-1.8	-1.8	-2.1	1.4	-0.1	-0.5	-0.8
171	seq=translation; coord=10:61794133..61802175:-1; parent_transcript=GRMZM2G146115_T01; parent_gene=GRMZM2G146115	GRMZM2G146115_P01 (+1)	44 kDa	Ref	0.2	-0.2	0.2	-0.1	-0.7	0.1	-0.3	0
172	seq=translation; coord=9:123775912..123778018:1; parent_transcript=GRMZM5G881775_T02; parent_gene=GRMZM5G881775	GRMZM5G881775_P02	16 kDa	Ref	2	Reference Missing	1.3	1.4	2.6	1.6	1.4	1.1
173	seq=translation; coord=3:230606444..230608235:-1; parent_transcript=GRMZM2G010868_T01; parent_gene=GRMZM2G010868	GRMZM2G010868_P01 (+1)	12 kDa	Ref	1.4	2.1	2.2	2.1	Value Missing	Value Missing	Value Missing	-3.5
174	seq=translation; coord=1:192400083..192403759:1; parent_transcript=GRMZM2G096585_T03; parent_gene=GRMZM2G096585	GRMZM2G096585_P03 (+2)	62 kDa	Ref	-1.3	-1.5	-1.4	-2.2	-1.4	-1.8	-1.6	-0.9
175	seq=translation; coord=4:183449044..183455471:-1; parent_transcript=GRMZM2G111818_T01; parent_gene=GRMZM2G111818	GRMZM2G111818_P01 (+1)	117 kDa	Ref	-0.3	-0.6	-1	-0.5	-0.6	-0.8	-1.1	-0.7
176	seq=translation; coord=4:163768734..163771529:1; parent_transcript=GRMZM2G135186_T01; parent_gene=GRMZM2G135186	GRMZM2G135186_P01 (+2)	42 kDa	Ref	1.6	1.8	0.9	1.6	2.1	0.1	-0.1	-0.8
177	seq=translation; coord=1:234775005..234780894:-1; parent_transcript=GRMZM2G416120_T01; parent_gene=GRMZM2G416120	GRMZM2G416120_P01	61 kDa	Ref	-0.8	-0.8	-0.2	-0.4	Reference Missing	-2.8	Reference Missing	-3
178	seq=translation; coord=1:84919829..84924586:-1; parent_transcript=GRMZM2G136769_T01; parent_gene=GRMZM2G136769	GRMZM2G136769_P01	60 kDa	Ref	-0.7	-0.8	-0.6	-1.3	-2	-1.2	-1.1	-1.1
179	seq=translation; coord=1:293163457..293166106:1; parent_transcript=GRMZM2G060702_T02; parent_gene=GRMZM2G060702	GRMZM2G060702_P02	16 kDa	Ref	-1.9	-2.1	-0.9	-1.2	No Values	No Values	No Values	No Values
180	seq=translation; coord=4:198559982..198560791:-1; parent_transcript=AC234156.1_FGT005; parent_gene=AC234156.1_FG005	AC234156.1_FGP005	28 kDa	Ref	-0.4	-0.8	-0.9	-1.7	-1.2	-1.1	-0.2	-0.1
181	seq=translation; coord=7:174283500..174287760:-1; parent_transcript=GRMZM2G430600_T01; parent_gene=GRMZM2G430600	GRMZM2G430600_P01 (+1)	36 kDa	Ref	0.4	-0.3	0.6	-0.1	-2.3	-1.5	-1.4	0.8

182	seq=translation; coord=8:163307256..163309969:-1; parent_transcript=GRMZM2G066024_T01; parent_gene=GRMZM2G066024	GRMZM2G066024_P01	38 kDa	Ref	-0.6	-0.5	0.2	0.1	0.8	1.8	2.3	2.8
183	seq=translation; coord=9:18329697..18331617:-1; parent_transcript=AC231745.1_FGT003; parent_gene=AC231745.1_FG003	AC231745.1_FGP003	41 kDa	Ref	0	2	1.5	2.3	No Values	No Values	No Values	No Values
184	seq=translation; coord=9:106398227..106409066:1; parent_transcript=GRMZM2G035417_T01; parent_gene=GRMZM2G035417	GRMZM2G035417_P01	43 kDa	Ref	0.8	0.8	0.9	0.7	-0.1	-0.5	-0.2	0.1
185	seq=translation; coord=9:148111509..148115129:1; parent_transcript=GRMZM2G145258_T01; parent_gene=GRMZM2G145258	GRMZM2G145258_P01	30 kDa	Ref	0.7	0.9	0.3	0.6	2.1	1.2	1	1
186	seq=translation; coord=6:124803945..124805261:1; parent_transcript=GRMZM2G084812_T01; parent_gene=GRMZM2G084812	GRMZM2G084812_P01	32 kDa	Ref	0.1	0.5	-1	-1.9	-1.7	-1.5	-1.1	-0.8
187	seq=translation; coord=6:164551086..164552313:1; parent_transcript=GRMZM2G021794_T01; parent_gene=GRMZM2G021794	GRMZM2G021794_P01	28 kDa	Ref	-1.1	-0.7	-1.2	-1.7	-2.2	-2.9	-3.1	-2.9
188	seq=translation; coord=1:278183921..278192926:-1; parent_transcript=GRMZM2G074158_T01; parent_gene=GRMZM2G074158	GRMZM2G074158_P01 (+1)	110 kDa	Ref	-0.2	-0.7	-1	0.5	-6.8	-5.5	-5.5	-5.8
189	seq=translation; coord=4:215212969..215240759:1; parent_transcript=GRMZM2G094123_T01; parent_gene=GRMZM2G094123	GRMZM2G094123_P01	58 kDa	Ref	0.3	0.3	-0.2	-0.3	-2.3	-2	-2.2	-2.2
190	seq=translation; coord=4:237610321..237618962:-1; parent_transcript=GRMZM2G155384_T02; parent_gene=GRMZM2G155384	GRMZM2G155384_P02	97 kDa	Ref	0.6	0.2	-0.2	0.4	2.3	1.2	1.1	0.7
191	seq=translation; coord=5:215817786..215821620:-1; parent_transcript=GRMZM5G848768_T02; parent_gene=GRMZM5G848768	GRMZM5G848768_P02	48 kDa	Ref	-0.4	0	0.3	1	0.6	1	1.4	1.5
192	seq=translation; coord=9:150090384..150093179:-1; parent_transcript=GRMZM2G099657_T01; parent_gene=GRMZM2G099657	GRMZM2G099657_P01 (+2)	33 kDa	Ref	-0.2	-0.6	-1.4	-0.7	1.9	-2.4	0.2	-0.3
193	seq=translation; coord=3:113472963..113480259:-1; parent_transcript=GRMZM2G093347_T01; parent_gene=GRMZM2G093347	GRMZM2G093347_P01 (+1)	24 kDa	Ref	-1.3	-1.4	-1.3	-1.1	0.8	0.5	0.1	0
194	seq=translation; coord=2:231986102..231987473:-1; parent_transcript=GRMZM2G053206_T01; parent_gene=GRMZM2G053206	GRMZM2G053206_P01	39 kDa	Ref	-0.3	0.2	1.2	Reference Missing	0.7	2.6	4.6	4.8

195	seq=translation; coord=3:201684716..201689628:-1; parent_transcript=GRMZM2G159724_T01; parent_gene=GRMZM2G159724	GRMZM2G159724_P01 (+1)	66 kDa	Ref	-0.4	-0.3	0.8	1.8	Value Missing	Value Missing	Value Missing	Value Missing
196	seq=translation; coord=5:153543150..153551579:1; parent_transcript=GRMZM2G177781_T01; parent_gene=GRMZM2G177781	GRMZM2G177781_P01 (+1)	70 kDa	Ref	-0.6	-1.1	-1.2	-1.8	-2	-2.4	-2.2	-2.5
197	seq=translation; coord=5:215289055..215297830:1; parent_transcript=GRMZM5G806449_T01; parent_gene=GRMZM5G806449	GRMZM5G806449_P01 (+1)	53 kDa	Ref	-0.7	-0.4	-0.2	0.3	0.5	-0.3	0.1	0.5
198	seq=translation; coord=4:2762573..2764339:1; parent_transcript=GRMZM2G126541_T01; parent_gene=GRMZM2G126541	GRMZM2G126541_P01	54 kDa	Ref	-0.3	0	1.1	2.1	No Values	No Values	No Values	No Values
199	seq=translation; coord=9:33125819..33139794:1; parent_transcript=GRMZM2G058675_T02; parent_gene=GRMZM2G058675	GRMZM2G058675_P02	59 kDa	Ref	-0.7	-0.4	-0.6	-0.1	-3.8	-3.4	-3.2	-3.2
200	seq=translation; coord=1:228251511..228256942:-1; parent_transcript=GRMZM2G346455_T05; parent_gene=GRMZM2G346455	GRMZM2G346455_P05 (+1)	46 kDa	Ref	1.4	1.7	0.9	1.2	0.3	1.6	2.2	2.6
201	seq=translation; coord=10:144060305..144061413:1; parent_transcript=GRMZM5G895313_T01; parent_gene=GRMZM5G895313	GRMZM5G895313_P01	20 kDa	Ref	0.3	-0.1	-0.5	-1.1	0.6	-0.9	-0.8	-1.3
202	seq=translation; coord=2:2548940..2552572:1; parent_transcript=GRMZM2G010435_T01; parent_gene=GRMZM2G010435	GRMZM2G010435_P01	56 kDa	Ref	-2.1	Value Missing	-2.1	-1.8	-3.1	-3.5	-3.4	-3.2
203	seq=translation; coord=2:4299718..4306692:-1; parent_transcript=GRMZM2G019404_T01; parent_gene=GRMZM2G019404	GRMZM2G019404_P01 (+1)	105 kDa	Ref	0.7	0.8	0.8	1.6	6.3	5.4	6.3	Value Missing
204	seq=translation; coord=2:51927314..51930580:1; parent_transcript=GRMZM2G109677_T01; parent_gene=GRMZM2G109677	GRMZM2G109677_P01 (+11)	45 kDa	Ref	1	0.2	-0.6	-0.3	2	0.6	-0.1	-1.3
205	seq=translation; coord=3:64458994..64462315:1; parent_transcript=GRMZM2G128171_T02; parent_gene=GRMZM2G128171	GRMZM2G128171_P02	40 kDa	Ref	-0.8	-1.1	-0.4	-1.2	-1	-0.9	-0.6	1.2
206	seq=translation; coord=8:39208505..39211382:-1; parent_transcript=GRMZM2G335657_T01; parent_gene=GRMZM2G335657	GRMZM2G335657_P01	53 kDa	Ref	-0.4	0	0	0.4	2.3	1.2	1.2	0.9
207	seq=translation; coord=9:132550417..132551258:1; parent_transcript=GRMZM2G404249_T01; parent_gene=GRMZM2G404249	GRMZM2G404249_P01 (+1)	17 kDa	Ref	No Values	No Values	No Values	No Values	Reference Missing	1.4	2.5	3.4

208	seq=translation; coord=6:125154676..125157008:1; parent_transcript=GRMZM2G450233_T01; parent_gene=GRMZM2G450233	GRMZM2G450233_P01	38 kDa	Ref	0	0.6	1	2.4	No Values	No Values	No Values	No Values
209	seq=translation; coord=8:160390396..160393378:1; parent_transcript=GRMZM2G134256_T01; parent_gene=GRMZM2G134256	GRMZM2G134256_P01	46 kDa	Ref	0.4	0.4	0.3	0.5	1.3	0.3	0.3	1.3
210	seq=translation; coord=2:210034645..210036617:1; parent_transcript=GRMZM2G339994_T01; parent_gene=GRMZM2G339994	GRMZM2G339994_P01 (+1)	39 kDa	Ref	0.4	1.4	0.2	1	2	2.5	3.6	4.3
211	seq=translation; coord=3:11894936..11898354:1; parent_transcript=GRMZM2G096240_T01; parent_gene=GRMZM2G096240	GRMZM2G096240_P01 (+1)	16 kDa	Ref	0	-0.6	0.1	-1.3	-1	-2.1	-1.9	-1.9
212	seq=translation; coord=4:44859117..44861815:-1; parent_transcript=GRMZM2G006953_T02; parent_gene=GRMZM2G006953	GRMZM2G006953_P02 (+1)	14 kDa	Ref	-0.4	-0.2	-0.4	-0.6	-0.8	0.2	0.4	0.7
213	seq=translation; coord=5:63800401..63802605:-1; parent_transcript=GRMZM5G834758_T02; parent_gene=GRMZM5G834758	GRMZM5G834758_P02 (+1)	17 kDa	Ref	1.9	1	1.2	1.2	2.2	0.9	0.8	1.3
214	seq=translation; coord=5:209262549..209264524:-1; parent_transcript=GRMZM2G015784_T01; parent_gene=GRMZM2G015784	GRMZM2G015784_P01 (+1)	12 kDa	Ref	-0.9	-1.3	-1.4	-2.3	-3.1	-2.4	-2	-1.8
215	seq=translation; coord=3:210506972..210508715:1; parent_transcript=GRMZM2G022931_T01; parent_gene=GRMZM2G022931	GRMZM2G022931_P01	25 kDa	Ref	-1	-1	-1.1	-1.4	-5.1	-5.9	Value Missing	-4.8
216	seq=translation; coord=7:4234486..4239316:1; parent_transcript=GRMZM2G064799_T01; parent_gene=GRMZM2G064799	GRMZM2G064799_P01 (+2)	68 kDa	Ref	0	0	-0.3	0.1	1.2	1.1	1.3	0.9
217	seq=translation; coord=4:153394405..153398468:1; parent_transcript=GRMZM2G135893_T01; parent_gene=GRMZM2G135893	GRMZM2G135893_P01	27 kDa	Ref	0	-0.1	-0.3	-0.6	-0.6	-1	-1	-0.3
218	seq=translation; coord=3:112198031..112206945:-1; parent_transcript=GRMZM2G086882_T01; parent_gene=GRMZM2G086882	GRMZM2G086882_P01	26 kDa	Ref	1.9	1.6	2	0.6	-0.3	0	-0.1	1.1
219	seq=translation; coord=7:54363440..54365032:-1; parent_transcript=GRMZM2G032766_T01; parent_gene=GRMZM2G032766	GRMZM2G032766_P01	42 kDa	Ref	1.4	0.8	2.2	2	1.9	1.1	1.4	0.7
220	seq=translation; coord=5:4664985..4668821:1; parent_transcript=GRMZM2G027825_T01; parent_gene=GRMZM2G027825	GRMZM2G027825_P01	50 kDa	Ref	-1.7	-2	-2.5	-2.5	-4.6	-5.2	-4.3	-4.6

221	seq=translation; coord=6:165013896..165020496:1; parent_transcript=GRMZM2G065757_T01; parent_gene=GRMZM2G065757	GRMZM2G065757_P01 (+1)	55 kDa	Ref	0.6	1	0.5	2.2	-4	Reference Missing	-4.1	-3.5
222	seq=translation; coord=10:18124892..18142878:-1; parent_transcript=GRMZM2G082271_T01; parent_gene=GRMZM2G082271	GRMZM2G082271_P01	110 kDa	Ref	0	-0.2	-0.4	-0.2	-0.1	0	-0.2	-0.5
223	seq=translation; coord=4:3210119..3212030:-1; parent_transcript=GRMZM2G085054_T02; parent_gene=GRMZM2G085054	GRMZM2G085054_P02	49 kDa	Ref	0	-0.1	0	-0.6	-1.2	-1.1	-1	-0.8
224	seq=translation; coord=3:8299415..8303114:-1; parent_transcript=GRMZM2G314898_T01; parent_gene=GRMZM2G314898	GRMZM2G314898_P01	46 kDa	Ref	-1.1	-0.8	-1.1	-0.2	-2.6	-2.3	-2	-2.1
225	seq=translation; coord=6:140347883..140348297:-1; parent_transcript=AC233879.1_FGT002; parent_gene=AC233879.1_FG002	AC233879.1_FGP002	10 kDa	Ref	Reference Missing	1.5	Reference Missing	Reference Missing	-4.4	-4.3	-3.9	-3.6
226	seq=translation; coord=10:4673451..4676086:-1; parent_transcript=GRMZM2G003762_T03; parent_gene=GRMZM2G003762	GRMZM2G003762_P03	25 kDa	Ref	-0.8	-0.6	-0.7	-1.1	-0.9	-0.6	-1.3	-1.6
227	seq=translation; coord=6:145987055..145988456:-1; parent_transcript=GRMZM2G306345_T01; parent_gene=GRMZM2G306345	GRMZM2G306345_P01	21 kDa	Ref	-0.3	1.1	-0.9	-1	Reference Missing	0.2	1.5	-0.6
228	seq=translation; coord=7:160215143..160216461:-1; parent_transcript=GRMZM2G374971_T01; parent_gene=GRMZM2G374971	GRMZM2G374971_P01	24 kDa	Ref	0.3	0.8	3	4.7	0.5	Reference Missing	2.7	4.3
229	seq=translation; coord=2:217342775..217345487:1; parent_transcript=GRMZM2G003409_T01; parent_gene=GRMZM2G003409	GRMZM2G003409_P01 (+1)	49 kDa	Ref	0.8	0.5	0.9	1.2	2.6	-0.4	0.2	0.5
230	seq=translation; coord=9:139405932..139410290:-1; parent_transcript=GRMZM2G165357_T01; parent_gene=GRMZM2G165357	GRMZM2G165357_P01 (+1)	39 kDa	Ref	0.4	0.8	0.2	-0.2	1.5	0.9	1.3	1.6
231	seq=translation; coord=10:148968847..148972562:1; parent_transcript=GRMZM2G104613_T01; parent_gene=GRMZM2G104613	GRMZM2G104613_P01	43 kDa	Ref	No Values	No Values	No Values	No Values	Reference Missing	Reference Missing	Reference Missing	Reference Missing
232	seq=translation; coord=4:128262885..128265210:1; parent_transcript=GRMZM2G008748_T02; parent_gene=GRMZM2G008748	GRMZM2G008748_P02	24 kDa	Ref	0.9	1.3	1.2	0.1	1	0.5	0	0.7
233	seq=translation; coord=1:298408224..298413579:-1; parent_transcript=GRMZM2G022269_T01; parent_gene=GRMZM2G022269	GRMZM2G022269_P01 (+1)	48 kDa	Ref	-0.5	-0.2	0.1	Value Missing	-5.4	-5.9	-5.7	-6.6

234	seq=translation; coord=6:140407545..140410127:1; parent_transcript=AC233895.1_FGT001; parent_gene=AC233895.1_FG001	AC233895.1_FGP001	43 kDa	Ref	0.5	0.5	0.3	0	1.1	0.6	0.5	0.9
235	seq=translation; coord=10:82668664..82669799:-1; parent_transcript=GRMZM2G121137_T01; parent_gene=GRMZM2G121137	GRMZM2G121137_P01	12 kDa	Ref	-2.6	-3	0	-3.1	0.4	0.5	0.5	0.2
236	seq=translation; coord=1:275835279..275836493:-1; parent_transcript=GRMZM2G025857_T01; parent_gene=GRMZM2G025857	GRMZM2G025857_P01	27 kDa	Ref	0	1.2	0.3	0.8	-3.2	Reference Missing	-3	-3.5
237	seq=translation; coord=8:129620163..129623583:1; parent_transcript=GRMZM2G117198_T01; parent_gene=GRMZM2G117198	GRMZM2G117198_P01 (+1)	43 kDa	Ref	0.6	0.7	0.4	0.5	6.1	3.8	4.1	3.8
238	seq=translation; coord=2:215654249..215658893:-1; parent_transcript=GRMZM2G005493_T01; parent_gene=GRMZM2G005493	GRMZM2G005493_P01	54 kDa	Ref	0.6	1.2	0.7	1.1	2.6	2.9	2.8	4.3
239	seq=translation; coord=5:149458858..149463589:-1; parent_transcript=GRMZM2G135588_T01; parent_gene=GRMZM2G135588	GRMZM2G135588_P01	55 kDa	Ref	-0.8	-1.5	-1.1	-0.5	-2.9	-1.6	-1.5	-1.3
240	seq=translation; coord=10:109798193..109804126:1; parent_transcript=GRMZM2G151041_T01; parent_gene=GRMZM2G151041	GRMZM2G151041_P01 (+1)	116 kDa	Ref	-0.1	0.2	-0.3	0.4	-2	-1.8	-1.5	-1.5
241	seq=translation; coord=1:263143605..263149463:1; parent_transcript=GRMZM2G079668_T01; parent_gene=GRMZM2G079668	GRMZM2G079668_P01 (+1)	73 kDa	Ref	No Values	No Values	No Values	No Values	0	0.8	0.2	0
242	seq=translation; coord=3:41741731..41743930:-1; parent_transcript=GRMZM2G144648_T01; parent_gene=GRMZM2G144648	GRMZM2G144648_P01	36 kDa	Ref	-0.1	0.8	0.2	-0.2	0.9	-1.2	-0.9	-0.7
243	seq=translation; coord=4:232510364..232513106:1; parent_transcript=GRMZM2G030228_T01; parent_gene=GRMZM2G030228	GRMZM2G030228_P01 (+9)	25 kDa	Ref	0.3	-0.2	-0.6	-1	0.5	-0.1	-0.2	-1.2
244	seq=translation; coord=5:199786088..199787380:1; parent_transcript=GRMZM2G054201_T01; parent_gene=GRMZM2G054201	GRMZM2G054201_P01 (+4)	18 kDa	Ref	0.7	0.5	0.7	0.4	-1.2	-0.9	-1.3	-1.3
245	seq=translation; coord=4:176858064..176868636:-1; parent_transcript=GRMZM2G149281_T01; parent_gene=GRMZM2G149281	GRMZM2G149281_P01 (+3)	23 kDa	Ref	-1.8	-1.6	-1.8	-2.9	-4.3	-4.5	Value Missing	-5.2
246	seq=translation; coord=4:221070736..221076802:-1; parent_transcript=GRMZM5G854613_T01; parent_gene=GRMZM5G854613	GRMZM5G854613_P01	45 kDa	Ref	-0.4	-0.4	-0.4	-0.2	-0.6	-1.1	-1.4	-1.7

247	seq=translation; coord=1:12248908..12249912:-1; parent_transcript=GRMZM2G472236_T01; parent_gene=GRMZM2G472236	GRMZM2G472236_P01	28 kDa	Ref	-1.5	-0.7	-1	Value Missing	-2.1	-2.4	-2.1	-0.3
248	seq=translation; coord=2:205943881..205945286:-1; parent_transcript=GRMZM2G099295_T01; parent_gene=GRMZM2G099295	GRMZM2G099295_P01	35 kDa	Ref	0.1	-0.8	-0.3	0.2	-1.7	-3	-2.9	-3.1
249	seq=translation; coord=1:77258356..77350693:1; parent_transcript=GRMZM2G020523_T01; parent_gene=GRMZM2G020523	GRMZM2G020523_P01	35 kDa	Ref	-0.3	1.2	1.1	0.8	1	-0.9	0	0.1
250	seq=translation; coord=6:127495800..127501815:-1; parent_transcript=GRMZM2G009845_T01; parent_gene=GRMZM2G009845	GRMZM2G009845_P01 (+3)	58 kDa	Ref	0.3	0.4	0.5	0.1	-0.9	-0.6	-0.5	-0.6
251	seq=translation; coord=4:36880092..36884475:-1; parent_transcript=GRMZM2G046804_T01; parent_gene=GRMZM2G046804	GRMZM2G046804_P01 (+6)	36 kDa	Ref	-0.7	-0.5	-1	-0.7	-2.1	-1.5	-1.4	-1.5
252	seq=translation; coord=6:135883373..135887597:1; parent_transcript=GRMZM2G059991_T01; parent_gene=GRMZM2G059991	GRMZM2G059991_P01	26 kDa	Ref	0.3	0.2	0.5	0.4	1.3	0.7	0.9	1.6
253	seq=translation; coord=4:20772978..20777200:-1; parent_transcript=GRMZM2G078143_T01; parent_gene=GRMZM2G078143	GRMZM2G078143_P01	57 kDa	Ref	0.3	0.7	0.2	0.7	1.7	1.8	1.5	1.3
254	seq=translation; coord=5:216695478..216702082:-1; parent_transcript=GRMZM2G133919_T01; parent_gene=GRMZM2G133919	GRMZM2G133919_P01	87 kDa	Ref	-0.5	-0.8	-0.7	-0.6	-0.9	-0.3	-0.7	-0.8
255	seq=translation; coord=8:103922075..103930908:-1; parent_transcript=GRMZM2G435373_T01; parent_gene=GRMZM2G435373	GRMZM2G435373_P01 (+1)	50 kDa	Ref	-0.1	-0.1	-0.5	-0.2	-0.4	-0.3	-0.3	-0.1
256	seq=translation; coord=3:168231994..168235001:-1; parent_transcript=GRMZM5G815894_T03; parent_gene=GRMZM5G815894	GRMZM5G815894_P03	34 kDa	Ref	1.5	1.6	1.4	0.9	2.6	1.2	0.6	0.9
257	seq=translation; coord=1:276305013..276310701:1; parent_transcript=GRMZM2G147687_T01; parent_gene=GRMZM2G147687	GRMZM2G147687_P01 (+3)	67 kDa	Ref	0.7	0.4	-0.1	0.8	0.7	-1.3	-1.3	-1.3
258	seq=translation; coord=6:156819019..156827574:1; parent_transcript=GRMZM2G701221_T01; parent_gene=GRMZM2G701221	GRMZM2G701221_P01	28 kDa	Ref	-0.3	-0.6	-0.4	-0.8	-8.5	-8.4	-8.4	-7.8
259	seq=translation; coord=8:90052293..90060254:-1; parent_transcript=GRMZM2G063676_T01; parent_gene=GRMZM2G063676	GRMZM2G063676_P01	94 kDa	Ref	0.3	0	-0.6	-1.3	-0.6	-0.8	-0.3	-0.5

260	seq=translation; coord=7:137455248..137460051:1; parent_transcript=GRMZM2G115757_T01; parent_gene=GRMZM2G115757	GRMZM2G115757_P01 (+1)	16 kDa	Ref	-1	-1.4	-1.2	-1.8	-4.9	-5.7	-5.2	-5.3
261	seq=translation; coord=1:66023613..66026784:-1; parent_transcript=GRMZM2G078876_T01; parent_gene=GRMZM2G078876	GRMZM2G078876_P01	17 kDa	Ref	-0.1	-0.2	-0.3	-0.3	-3.4	Value Missing	Value Missing	-5.1
262	seq=translation; coord=10:145479598..145483674:-1; parent_transcript=GRMZM2G073465_T03; parent_gene=GRMZM2G073465	GRMZM2G073465_P03	51 kDa	Ref	-0.9	-1.5	-0.1	1.3	-4.9	-6.1	-5.8	-5
263	seq=translation; coord=6:9203729..9206286:-1; parent_transcript=GRMZM2G066460_T01; parent_gene=GRMZM2G066460	GRMZM2G066460_P01	34 kDa	Ref	No Values	No Values	No Values	No Values	1	0.1	-0.6	-0.6
264	seq=translation; coord=1:180306606..180308510:1; parent_transcript=GRMZM2G161335_T01; parent_gene=GRMZM2G161335	GRMZM2G161335_P01	50 kDa	Ref	-1.6	-1.6	-1.4	-0.9	-2.2	-2.3	-1.9	-1.6
265	seq=translation; coord=1:203587258..203590599:-1; parent_transcript=GRMZM2G106928_T01; parent_gene=GRMZM2G106928	GRMZM2G106928_P01	21 kDa	Ref	-0.5	Value Missing	-0.6	-1.1	-0.7	-0.2	0.3	0.6
266	seq=translation; coord=7:160032302..160036034:1; parent_transcript=GRMZM2G083243_T01; parent_gene=GRMZM2G083243	GRMZM2G083243_P01 (+1)	50 kDa	Ref	0.2	0.2	-0.9	-0.4	0.5	-0.5	-0.3	-1.4
267	seq=translation; coord=3:24095213..24095931:-1; parent_transcript=GRMZM2G011523_T01; parent_gene=GRMZM2G011523	GRMZM2G011523_P01 (+2)	10 kDa	Ref	0	2.1	2.7	1.1	Value Missing	Value Missing	0.2	0.1
268	seq=translation; coord=6:161966268..161967646:1; parent_transcript=GRMZM2G096475_T01; parent_gene=GRMZM2G096475	GRMZM2G096475_P01	19 kDa	Ref	0.2	0.4	1.9	2.5	-1.4	Value Missing	Value Missing	Reference Missing
269	seq=translation; coord=2:86767115..86790532:1; parent_transcript=GRMZM2G028313_T01; parent_gene=GRMZM2G028313	GRMZM2G028313_P01 (+1)	58 kDa	Ref	-0.2	-0.2	-0.5	-0.2	-0.2	-0.2	-0.3	-0.6
270	seq=translation; coord=1:94680007..94684730:-1; parent_transcript=GRMZM2G178576_T02; parent_gene=GRMZM2G178576	GRMZM2G178576_P02 (+1)	18 kDa	Ref	-1.4	-1.5	-1.5	-2.1	1.5	2.3	2.9	3.5
271	seq=translation; coord=10:75695843..75698244:1; parent_transcript=GRMZM2G099352_T03; parent_gene=GRMZM2G099352	GRMZM2G099352_P03	26 kDa	Ref	-0.6	-0.8	-0.4	-0.2	0.9	1	0.6	0
272	seq=translation; coord=3:19924648..19927549:1; parent_transcript=GRMZM2G169182_T01; parent_gene=GRMZM2G169182	GRMZM2G169182_P01 (+1)	70 kDa	Ref	No Values	No Values	No Values	No Values	-0.7	0.2	0.1	-0.2

273	seq=translation; coord=8:168745391..168749427:1; parent_transcript=GRMZM2G146206_T04; parent_gene=GRMZM2G146206	GRMZM2G146206_P04	27 kDa	Ref	2.5	2.8	2.5	2.4	-1.5	Reference Missing	-1.8	-1.3
274	seq=translation; coord=2:48112888..48133114:1; parent_transcript=GRMZM2G053019_T01; parent_gene=GRMZM2G053019	GRMZM2G053019_P01	47 kDa	Ref	No Values	No Values	No Values	No Values	1.3	1.2	1.3	1.5
275	seq=translation; coord=10:4694280..4699187:-1; parent_transcript=GRMZM2G004534_T01; parent_gene=GRMZM2G004534	GRMZM2G004534_P01	57 kDa	Ref	0	-0.1	-0.1	0.5	1.7	0.5	0.4	1.1
276	seq=translation; coord=4:37224780..37227724:1; parent_transcript=GRMZM2G064133_T01; parent_gene=GRMZM2G064133	GRMZM2G064133_P01	32 kDa	Ref	0.2	-0.2	0.5	-0.6	0.5	1.2	1	0.8
277	seq=translation; coord=6:90306950..90312408:-1; parent_transcript=GRMZM2G094074_T01; parent_gene=GRMZM2G094074	GRMZM2G094074_P01 (+6)	16 kDa	Ref	0.7	1.1	0.7	-0.1	1.5	1.1	0.6	0.1
278	seq=translation; coord=7:171631430..171634129:1; parent_transcript=GRMZM2G439201_T02; parent_gene=GRMZM2G439201	GRMZM2G439201_P02	31 kDa	Ref	-1	-1.2	-0.7	-2	-0.1	-0.3	-0.3	-0.3
279	seq=translation; coord=8:100394854..100398658:-1; parent_transcript=GRMZM2G126010_T01; parent_gene=GRMZM2G126010	GRMZM2G126010_P01 (+2)	42 kDa	Ref	-0.1	0.7	0.6	1.9	-0.7	-0.2	-0.5	-1.3
280	seq=translation; coord=1:198230317..198233618:-1; parent_transcript=GRMZM2G017110_T02; parent_gene=GRMZM2G017110	GRMZM2G017110_P02 (+1)	56 kDa	Ref	Value Missing	-2.5	-2.4	-2.3	-1.2	-0.7	-1	-0.9
281	seq=translation; coord=1:56160677..56162858:-1; parent_transcript=GRMZM2G054916_T01; parent_gene=GRMZM2G054916	GRMZM2G054916_P01	54 kDa	Ref	Reference Missing	0.5	0.4	1.4	Reference Missing	1.2	Reference Missing	Reference Missing
282	seq=translation; coord=4:26893142..26901172:1; parent_transcript=GRMZM2G093900_T01; parent_gene=GRMZM2G093900	GRMZM2G093900_P01	56 kDa	Ref	0.8	0.2	-0.1	0.2	-0.4	1	0	0.2
283	seq=translation; coord=8:37409326..37412866:1; parent_transcript=GRMZM2G002416_T01; parent_gene=GRMZM2G002416	GRMZM2G002416_P01	50 kDa	Ref	-0.2	-0.5	-0.5	-0.3	0.1	-0.6	-0.9	-1.3
284	seq=translation; coord=2:58586007..58596234:1; parent_transcript=GRMZM2G073054_T01; parent_gene=GRMZM2G073054	GRMZM2G073054_P01 (+1)	93 kDa	Ref	-0.7	-1	-1.4	-1.7	3.3	3.3	3.5	3.2
285	seq=translation; coord=1:2801080..2803977:-1; parent_transcript=GRMZM2G041881_T01; parent_gene=GRMZM2G041881	GRMZM2G041881_P01	18 kDa	Ref	1.4	1.7	1	0.4	1.3	Reference Missing	0.2	0.8

286	seq=translation;<br>coord=10:110621503..110626726:1;<br>parent_transcript=GRMZM2G079538_T01;<br>parent_gene=GRMZM2G079538	GRMZM2G079538_P01 (+3)	49 kDa	Ref	0	0.4	0.5	0.6	0.7	0.8	0.3	0.1
287	seq=translation; coord=8:9901033..9904862:-<br>1;<br>parent_transcript=GRMZM2G069195_T01;<br>parent_gene=GRMZM2G069195	GRMZM2G069195_P01	42 kDa	Ref	-0.3	-0.5	-0.4	-0.3	-0.9	-0.9	-1.2	-1
288	seq=translation;<br>coord=1:56785163..56792250:-1;<br>parent_transcript=GRMZM2G318780_T02;<br>parent_gene=GRMZM2G318780	GRMZM2G318780_P02	92 kDa	Ref	1.4	2.3	1.3	Reference Missing	-0.9	-0.2	-0.2	-0.5
289	seq=translation;<br>coord=1:222203896..222206239:-1;<br>parent_transcript=GRMZM2G320269_T01;<br>parent_gene=GRMZM2G320269	GRMZM2G320269_P01	39 kDa	Ref	0.5	-0.4	-0.8	-1.5	-0.3	-1.6	-1.6	-1.9
290	seq=translation;<br>coord=10:132619525..132621315:1;<br>parent_transcript=GRMZM2G468855_T02;<br>parent_gene=GRMZM2G468855	GRMZM2G468855_P02	33 kDa	Ref	-0.9	-0.9	1.4	Value Missing	Reference Missing	-2.5	-3	-3.7
291	seq=translation;<br>coord=8:162019785..162023951:1;<br>parent_transcript=GRMZM2G053898_T01;<br>parent_gene=GRMZM2G053898	GRMZM2G053898_P01 (+3)	10 kDa	Ref	2.2	1.5	1.6	-0.1	-0.3	0.4	0.3	Reference Missing
292	seq=translation;<br>coord=8:173117675..173120538:1;<br>parent_transcript=GRMZM2G116273_T01;<br>parent_gene=GRMZM2G116273	GRMZM2G116273_P01	24 kDa	Ref	-1.1	-0.8	0	0.1	-2.1	-0.7	-0.5	0.4
293	seq=translation;<br>coord=1:250061761..250067450:1;<br>parent_transcript=GRMZM2G157470_T01;<br>parent_gene=GRMZM2G157470	GRMZM2G157470_P01	38 kDa	Ref	1.2	1	0.9	0.1	0.2	-0.7	Value Missing	-1.8
294	seq=translation;<br>coord=9:130484606..130489460:-1;<br>parent_transcript=GRMZM2G436092_T01;<br>parent_gene=GRMZM2G436092	GRMZM2G436092_P01 (+3)	29 kDa	Ref	-0.9	-1	-1.4	-1.2	0.1	-1.4	-1.6	-2.1
295	seq=translation;<br>coord=4:236362229..236373144:1;<br>parent_transcript=GRMZM2G168629_T01;<br>parent_gene=GRMZM2G168629	GRMZM2G168629_P01 (+1)	134 kDa	Ref	0.3	0.4	0.3	0.3	0.3	0.5	0.3	-0.3
296	seq=translation;<br>coord=7:131783728..131787563:1;<br>parent_transcript=GRMZM2G069651_T01;<br>parent_gene=GRMZM2G069651	GRMZM2G069651_P01	80 kDa	Ref	No Values	No Values	No Values	No Values	-1.3	-0.5	-1	-1.1
297	seq=translation;<br>coord=8:171565757..171566487:-1;<br>parent_transcript=GRMZM2G091054_T01;<br>parent_gene=GRMZM2G091054	GRMZM2G091054_P01	12 kDa	Ref	1.5	1.2	1	0	Reference Missing	0.9	1.2	1.2
298	seq=translation;<br>coord=5:63317874..63324803:-1;<br>parent_transcript=GRMZM2G088753_T01;<br>parent_gene=GRMZM2G088753	GRMZM2G088753_P01 (+1)	93 kDa	Ref	-0.8	-0.8	-0.7	-0.6	-3.1	-2.7	-2.3	-1.9

299	seq=translation; coord=1:233228509..233231671:-1; parent_transcript=GRMZM2G134747_T01; parent_gene=GRMZM2G134747	GRMZM2G134747_P01	19 kDa	Ref	-0.7	-0.9	-0.3	-1.2	-1.3	-0.3	-0.3	-0.2
300	seq=translation; coord=6:92813319..92815990:-1; parent_transcript=GRMZM2G043822_T02; parent_gene=GRMZM2G043822	GRMZM2G043822_P02 (+1)	50 kDa	Ref	0.7	0.2	-0.5	-0.7	1.2	0.6	0.7	-0.2
301	seq=translation; coord=9:59153476..59160133:1; parent_transcript=GRMZM2G104481_T01; parent_gene=GRMZM2G104481	GRMZM2G104481_P01 (+1)	42 kDa	Ref	-0.6	-1.3	-1.6	-1.9	-0.5	-1.7	-1.7	-2.3
302	seq=translation; coord=1:264798453..264802516:-1; parent_transcript=GRMZM2G118003_T03; parent_gene=GRMZM2G118003	GRMZM2G118003_P03	56 kDa	Ref	0.3	-0.3	-0.3	-0.8	0.3	0.6	1	0.7
303	seq=translation; coord=1:35455268..35465683:-1; parent_transcript=GRMZM2G164562_T01; parent_gene=GRMZM2G164562	GRMZM2G164562_P01	47 kDa	Ref	0.6	0.6	0.6	0.6	1.6	1.3	1.5	1.3
304	seq=translation; coord=5:77434003..77436424:1; parent_transcript=GRMZM5G805485_T01; parent_gene=GRMZM5G805485	GRMZM5G805485_P01	68 kDa	Ref	0.6	0	0.7	-0.1	0.9	1.1	0.6	0.7
305	seq=translation; coord=3:220506759..220509488:1; parent_transcript=GRMZM2G116135_T01; parent_gene=GRMZM2G116135	GRMZM2G116135_P01	24 kDa	Ref	0.8	0.3	-0.1	-0.7	1.5	0.7	1.5	-0.8
306	seq=translation; coord=6:39092081..39092901:1; parent_transcript=GRMZM2G012806_T01; parent_gene=GRMZM2G012806	GRMZM2G012806_P01	10 kDa	Ref	-0.6	-0.8	-0.7	-0.6	Reference Missing	-2.7	-2	-2.4
307	seq=translation; coord=10:55753301..55756434:-1; parent_transcript=GRMZM2G127087_T01; parent_gene=GRMZM2G127087	GRMZM2G127087_P01 (+1)	66 kDa	Ref	0.4	0.1	2.1	3.7	No Values	No Values	No Values	No Values
308	seq=translation; coord=8:142871869..142875661:-1; parent_transcript=GRMZM2G134738_T01; parent_gene=GRMZM2G134738	GRMZM2G134738_P01 (+1)	18 kDa	Ref	1.8	0.8	1.4	0.7	-0.6	-0.9	-2.3	-1.6
309	seq=translation; coord=2:3098932..3104990:1; parent_transcript=GRMZM2G071333_T01; parent_gene=GRMZM2G071333	GRMZM2G071333_P01	123 kDa	Ref	-0.2	-0.5	-0.7	-0.5	2.2	2.3	2.4	2
310	seq=translation; coord=1:177045380..177050536:-1; parent_transcript=GRMZM2G094742_T03; parent_gene=GRMZM2G094742	GRMZM2G094742_P03	9 kDa	Ref	-0.1	0.6	-0.5	-0.7	1.3	1.4	1.6	1.9
311	seq=translation; coord=2:29943554..29946951:-1; parent_transcript=GRMZM2G150295_T01; parent_gene=GRMZM2G150295	GRMZM2G150295_P01	14 kDa	Ref	0	-0.8	-1.4	-2.1	0.1	-0.2	-1	0.5

312	seq=translation; coord=4:191578887..191581169:1; parent_transcript=GRMZM2G004699_T01; parent_gene=GRMZM2G004699	GRMZM2G004699_P01	34 kDa	Ref	-0.7	0.3	0.6	0.2	No Values	No Values	No Values	No Values
313	seq=translation; coord=3:1711864..1714237:1; parent_transcript=GRMZM2G123558_T01; parent_gene=GRMZM2G123558	GRMZM2G123558_P01 (+1)	23 kDa	Ref	Reference Missing	2.3	Reference Missing	Reference Missing	No Values	No Values	No Values	No Values
314	seq=translation; coord=4:230101062..230104782:-1; parent_transcript=GRMZM2G134582_T01; parent_gene=GRMZM2G134582	GRMZM2G134582_P01 (+1)	47 kDa	Ref	0.9	1.2	0.8	0.6	0.6	0.8	0.7	0.7
315	seq=translation; coord=7:124381997..124386923:1; parent_transcript=GRMZM2G389173_T01; parent_gene=GRMZM2G389173	GRMZM2G389173_P01	47 kDa	Ref	0.5	0.8	0.8	0.5	1.4	1.3	Reference Missing	Reference Missing
316	seq=translation; coord=2:7091727..7098725:-1; parent_transcript=GRMZM2G379758_T01; parent_gene=GRMZM2G379758	GRMZM2G379758_P01	106 kDa	Ref	0.6	0.3	0	0.5	-0.4	-1.1	-1.2	-2.3
317	seq=translation; coord=8:150358554..150362799:-1; parent_transcript=GRMZM2G055936_T01; parent_gene=GRMZM2G055936	GRMZM2G055936_P01	17 kDa	Ref	-1.7	-1.8	-1	-2.3	-1.5	-0.6	-0.7	-0.3
318	seq=translation; coord=7:172711231..172713259:1; parent_transcript=GRMZM2G063617_T01; parent_gene=GRMZM2G063617	GRMZM2G063617_P01	15 kDa	Ref	0.8	1	1	0.6	-0.7	-1	-1.2	-2
319	seq=translation; coord=5:84934242..84936493:1; parent_transcript=GRMZM2G010321_T01; parent_gene=GRMZM2G010321	GRMZM2G010321_P01	17 kDa	Ref	-2	-1.8	-2.1	-3	-3.7	-6.4	-5.9	-6.6
320	seq=translation; coord=10:132006574..132009975:-1; parent_transcript=GRMZM2G164714_T02; parent_gene=GRMZM2G164714	GRMZM2G164714_P02	52 kDa	Ref	-0.2	-0.2	0.1	-0.2	-3.6	-2.9	-2.8	-3
321	seq=translation; coord=2:71086139..71088448:-1; parent_transcript=GRMZM2G112176_T01; parent_gene=GRMZM2G112176	GRMZM2G112176_P01 (+1)	16 kDa	Ref	0.2	0	-0.3	-0.3	-3	-1.4	-0.9	-0.3
322	seq=translation; coord=1:76732179..76733770:-1; parent_transcript=GRMZM2G077316_T01; parent_gene=GRMZM2G077316	GRMZM2G077316_P01	26 kDa	Ref	-0.5	0	-0.4	-0.3	-2	-1.6	-1.7	-2
323	seq=translation; coord=8:157452514..157453996:-1; parent_transcript=GRMZM2G059299_T01; parent_gene=GRMZM2G059299	GRMZM2G059299_P01	49 kDa	Ref	Reference Missing	1.5	2	Reference Missing	-6.1	Reference Missing	Reference Missing	-2.3
324	seq=translation; coord=2:197582100..197595530:1; parent_transcript=GRMZM2G117870_T01; parent_gene=GRMZM2G117870	GRMZM2G117870_P01	120 kDa	Ref	0.5	0.1	0	0.3	-0.2	-0.3	-0.6	0

325	seq=translation; coord=4:233024312..233031203:1; parent_transcript=GRMZM2G165817_T01; parent_gene=GRMZM2G165817	GRMZM2G165817_P01 (+1)	45 kDa	Ref	-0.4	-0.7	-0.2	-0.3	-0.5	0.6	0.3	0.2
326	seq=translation; coord=1:216050547..216055495:-1; parent_transcript=GRMZM2G016189_T01; parent_gene=GRMZM2G016189	GRMZM2G016189_P01	49 kDa	Ref	0.2	0	0.3	0.6	-2.4	-2.1	-1.4	-0.9
327	seq=translation; coord=8:38410669..38412926:1; parent_transcript=GRMZM2G054123_T01; parent_gene=GRMZM2G054123	GRMZM2G054123_P01 (+4)	43 kDa	Ref	-0.3	0.2	-0.1	0.3	-1.1	-4.3	-3.9	-4
328	seq=translation; coord=9:139187765..139188857:1; parent_transcript=GRMZM2G305046_T01; parent_gene=GRMZM2G305046	GRMZM2G305046_P01 (+1)	17 kDa	Ref	1.8	0.9	1.3	1	No Values	No Values	No Values	No Values
329	seq=translation; coord=8:160396937..160401812:1; parent_transcript=GRMZM2G134176_T01; parent_gene=GRMZM2G134176	GRMZM2G134176_P01	21 kDa	Ref	-0.1	-0.2	0.1	0.2	-5.2	-4.3	-4.5	-4.5
330	seq=translation; coord=8:135138380..135140767:-1; parent_transcript=GRMZM2G076544_T01; parent_gene=GRMZM2G076544	GRMZM2G076544_P01	26 kDa	Ref	-1.2	-1.2	-1.4	-1.8	-1.6	-0.8	-1.3	-1.6
331	seq=translation; coord=5:169454598..169459090:1; parent_transcript=GRMZM2G139300_T01; parent_gene=GRMZM2G139300	GRMZM2G139300_P01	67 kDa	Ref	0.4	0.5	0.7	1.5	4.7	5.7	5.9	4.8
332	seq=translation; coord=3:173015163..173022969:-1; parent_transcript=GRMZM2G038281_T01; parent_gene=GRMZM2G038281	GRMZM2G038281_P01 (+1)	94 kDa	Ref	-1.1	-1.5	-1.9	-1.8	-1.8	-1.7	-2.2	-2.3
333	seq=translation; coord=2:82394515..82407013:1; parent_transcript=GRMZM5G858094_T01; parent_gene=GRMZM5G858094	GRMZM5G858094_P01 (+1)	257 kDa	Ref	0	-0.2	-0.7	-0.5	0.9	0.5	0.5	-0.2
334	seq=translation; coord=8:74938636..74939549:-1; parent_transcript=GRMZM2G045664_T01; parent_gene=GRMZM2G045664	GRMZM2G045664_P01	18 kDa	Ref	-1.7	-0.6	-1	-1.5	-1	-0.7	-2	-0.8
335	seq=translation; coord=3:26352719..26355885:1; parent_transcript=GRMZM2G410916_T02; parent_gene=GRMZM2G410916	GRMZM2G410916_P02	67 kDa	Ref	-1.1	-0.4	-1.7	-2.1	-2.2	-2	-1.6	-2
336	seq=translation; coord=6:165704660..165707098:-1; parent_transcript=GRMZM2G354604_T01; parent_gene=GRMZM2G354604	GRMZM2G354604_P01 (+2)	26 kDa	Ref	-0.1	-0.1	-0.4	-0.2	0.8	0.1	0	-0.4
337	seq=translation; coord=7:169267359..169269362:1; parent_transcript=GRMZM2G013652_T01; parent_gene=GRMZM2G013652	GRMZM2G013652_P01 (+1)	11 kDa	Ref	-0.5	-1.3	-0.7	-1.1	Value Missing	-0.1	Value Missing	0.5

338	seq=translation; coord=6:31411848..31415600:-1; parent_transcript=GRMZM2G099186_T02; parent_gene=GRMZM2G099186	GRMZM2G099186_P02 (+1)	35 kDa	Ref	0.8	0.9	0.2	0.3	0.9	0	0.1	-0.7
339	seq=translation; coord=10:148535505..148536885:1; parent_transcript=GRMZM2G011513_T01; parent_gene=GRMZM2G011513	GRMZM2G011513_P01	16 kDa	Ref	-1.1	-1.5	-1.2	-2.2	-4.4	-4.4	-4.2	-4.5
340	seq=translation; coord=2:61490885..61494711:-1; parent_transcript=GRMZM2G134668_T02; parent_gene=GRMZM2G134668	GRMZM2G134668_P02	60 kDa	Ref	1.1	1	1	1.3	3.8	2.2	2.2	1.8
341	seq=translation; coord=1:58418770..58420264:-1; parent_transcript=GRMZM2G075290_T01; parent_gene=GRMZM2G075290	GRMZM2G075290_P01	38 kDa	Ref	-0.1	0.6	0.6	0.1	4.7	5.4	5.5	4.9
342	seq=translation; coord=7:171733893..171737098:-1; parent_transcript=GRMZM2G025992_T01; parent_gene=GRMZM2G025992	GRMZM2G025992_P01 (+3)	22 kDa	Ref	-0.8	-1.1	-0.9	-1.4	-2.1	-2	-1.3	-0.9
343	seq=translation; coord=4:217021920..217028774:-1; parent_transcript=GRMZM2G180578_T01; parent_gene=GRMZM2G180578	GRMZM2G180578_P01	56 kDa	Ref	0.4	0	-0.6	-0.4	3.3	1.2	0.6	1.3
344	seq=translation; coord=3:226624022..226632817:-1; parent_transcript=GRMZM2G017086_T01; parent_gene=GRMZM2G017086	GRMZM2G017086_P01 (+1)	54 kDa	Ref	0.1	-0.3	-0.2	-0.2	0.5	0	0	-0.4
345	seq=translation; coord=2:183929119..183934914:-1; parent_transcript=GRMZM2G399284_T01; parent_gene=GRMZM2G399284	GRMZM2G399284_P01 (+1)	26 kDa	Ref	-0.4	-0.6	-0.2	-0.6	0.8	1.5	2	2
346	seq=translation; coord=4:170332797..170339707:-1; parent_transcript=GRMZM2G008410_T01; parent_gene=GRMZM2G008410	GRMZM2G008410_P01	37 kDa	Ref	-0.5	-0.8	-0.7	-0.9	-0.8	-1.6	-1.6	-1.3
347	seq=translation; coord=3:218785554..218795824:-1; parent_transcript=GRMZM2G090779_T01; parent_gene=GRMZM2G090779	GRMZM2G090779_P01	48 kDa	Ref	0.5	0.6	0.7	0.5	-0.8	-0.3	0.3	-0.4
348	seq=translation; coord=8:171852196..171855863:-1; parent_transcript=GRMZM2G159643_T01; parent_gene=GRMZM2G159643	GRMZM2G159643_P01	22 kDa	Ref	1.1	1.5	1.4	2.2	No Values	No Values	No Values	No Values
349	seq=translation; coord=2:10210584..10215840:-1; parent_transcript=GRMZM5G817886_T02; parent_gene=GRMZM5G817886	GRMZM5G817886_P02	56 kDa	Ref	0	0.2	-0.5	0.4	0.9	2	1.4	1.1
350	seq=translation; coord=1:246702682..246710172:-1; parent_transcript=GRMZM2G146589_T01; parent_gene=GRMZM2G146589	GRMZM2G146589_P01 (+3)	69 kDa	Ref	0.7	1.1	0.5	0.4	2.5	1.5	1.3	2.5

351	seq=translation; coord=7:123475099..123479587:1; parent_transcript=GRMZM5G824944_T01; parent_gene=GRMZM5G824944	GRMZM5G824944_P01 (+1)	68 kDa	Ref	0.1	-0.2	-0.4	0.2	-0.1	0.2	0.2	0.5
352	seq=translation; coord=1:220892398..220897302:-1; parent_transcript=GRMZM2G078022_T03; parent_gene=GRMZM2G078022	GRMZM2G078022_P03 (+2)	23 kDa	Ref	-0.5	-0.5	-1.1	-1.2	-1.9	-3.7	-3.9	-3.7
353	seq=translation; coord=7:136570996..136574421:1; parent_transcript=GRMZM2G074386_T01; parent_gene=GRMZM2G074386	GRMZM2G074386_P01	24 kDa	Ref	-0.2	-0.2	0	0.3	0.2	-0.3	0	-0.2
354	seq=translation; coord=1:20418930..20420739:-1; parent_transcript=GRMZM2G134367_T01; parent_gene=GRMZM2G134367	GRMZM2G134367_P01	47 kDa	Ref	-0.1	-1.1	-0.7	-1.1	-2.5	-2.4	-2.7	-3.1
355	seq=translation; coord=8:79300397..79307702:1; parent_transcript=GRMZM2G073774_T01; parent_gene=GRMZM2G073774	GRMZM2G073774_P01 (+2)	59 kDa	Ref	0	0.2	-0.3	-0.3	-2.2	-2.4	-2.4	-2.7
356	seq=translation; coord=6:75746632..75749530:-1; parent_transcript=GRMZM2G130528_T01; parent_gene=GRMZM2G130528	GRMZM2G130528_P01	40 kDa	Ref	0.8	2.6	1.9	3	2.3	1.8	2.9	3.4
357	seq=translation; coord=1:200480447..200483616:-1; parent_transcript=GRMZM2G157018_T01; parent_gene=GRMZM2G157018	GRMZM2G157018_P01	20 kDa	Ref	1.7	0.9	1.9	0.6	No Values	No Values	No Values	No Values
358	seq=translation; coord=9:48437559..48441823:-1; parent_transcript=GRMZM2G049811_T01; parent_gene=GRMZM2G049811	GRMZM2G049811_P01	41 kDa	Ref	-0.5	0	0	0.3	-1.6	-0.8	-1.5	-1.1
359	seq=translation; coord=1:180400813..180406565:-1; parent_transcript=GRMZM2G085474_T01; parent_gene=GRMZM2G085474	GRMZM2G085474_P01 (+1)	41 kDa	Ref	0.2	-0.4	-0.9	-0.4	0.3	-1.2	-1.5	-2.3
360	seq=translation; coord=9:139511132..139514364:1; parent_transcript=GRMZM2G366532_T01; parent_gene=GRMZM2G366532	GRMZM2G366532_P01 (+1)	72 kDa	Ref	-0.4	0.5	0.5	Reference Missing	-0.4	0.7	1.2	1.5
361	seq=translation; coord=4:38181649..38185320:-1; parent_transcript=GRMZM2G103430_T01; parent_gene=GRMZM2G103430	GRMZM2G103430_P01 (+4)	51 kDa	Ref	-0.3	-0.6	-0.9	-0.5	-1.3	-1.6	-2.5	-2.9
362	seq=translation; coord=4:53954699..53962275:1; parent_transcript=GRMZM2G423027_T01; parent_gene=GRMZM2G423027	GRMZM2G423027_P01 (+1)	25 kDa	Ref	-0.3	-0.2	-0.1	-1.1	-2.5	-1.6	-0.5	0
363	seq=translation; coord=4:237508371..237511930:-1; parent_transcript=GRMZM2G166035_T01; parent_gene=GRMZM2G166035	GRMZM2G166035_P01 (+1)	31 kDa	Ref	-0.8	-1	-1.1	-0.5	-4.5	-4.1	-3.9	-3.9

364	seq=translation; coord=5:199376854..199379060:1; parent_transcript=GRMZM2G027741_T01; parent_gene=GRMZM2G027741	GRMZM2G027741_P01 (+3)	29 kDa	Ref	0	-0.2	0	-0.5	-2.5	-2.4	-2	-0.5
365	seq=translation; coord=3:161953049..161955801:1; parent_transcript=GRMZM2G153991_T01; parent_gene=GRMZM2G153991	GRMZM2G153991_P01	16 kDa	Ref	-0.8	-0.8	-1	-1.6	-2.1	-1.6	-2.3	-2.1
366	seq=translation; coord=6:161454531..161462066:-1; parent_transcript=GRMZM2G090087_T02; parent_gene=GRMZM2G090087	GRMZM2G090087_P02 (+1)	61 kDa	Ref	-0.8	-0.8	-0.8	-0.5	4.9	4.7	5	5
367	seq=translation; coord=7:23715448..23717527:-1; parent_transcript=GRMZM2G097030_T01; parent_gene=GRMZM2G097030	GRMZM2G097030_P01	51 kDa	Ref	-0.3	Value Missing	Value Missing	Value Missing	-5.5	-5.1	-5.7	-6.2
368	seq=translation; coord=6:31868128..31869802:-1; parent_transcript=GRMZM2G163406_T01; parent_gene=GRMZM2G163406	GRMZM2G163406_P01	33 kDa	Ref	2.2	1.2	0.9	1.2	2.7	3.7	4.5	4.6
369	seq=translation; coord=8:95715355..95726032:1; parent_transcript=GRMZM2G375504_T01; parent_gene=GRMZM2G375504	GRMZM2G375504_P01 (+2)	78 kDa	Ref	-0.7	-0.7	-1.4	-1	1.3	0.7	0.5	-0.3
370	seq=translation; coord=7:147132517..147134161:-1; parent_transcript=GRMZM2G168330_T02; parent_gene=GRMZM2G168330	GRMZM2G168330_P02	15 kDa	Ref	0.1	-0.1	0.3	-0.1	0.4	-0.4	-1.3	-1.9
371	seq=translation; coord=1:60163114..60165406:1; parent_transcript=GRMZM2G000923_T01; parent_gene=GRMZM2G000923	GRMZM2G000923_P01 (+2)	23 kDa	Ref	0.8	0.6	0.6	0	1.8	0.8	1.2	1.6
372	seq=translation; coord=9:107209042..107212141:1; parent_transcript=GRMZM2G172357_T01; parent_gene=GRMZM2G172357	GRMZM2G172357_P01 (+3)	15 kDa	Ref	0	-0.4	0.9	-1.1	-4.2	-3.9	-4.2	-3.5
373	seq=translation; coord=1:293135180..293138761:1; parent_transcript=GRMZM2G061900_T01; parent_gene=GRMZM2G061900	GRMZM2G061900_P01 (+3)	24 kDa	Ref	0.4	0.5	-0.4	-0.3	0.9	-1.5	-1.5	-2.2
374	seq=translation; coord=1:234307913..234320907:1; parent_transcript=GRMZM2G008607_T01; parent_gene=GRMZM2G008607	GRMZM2G008607_P01	40 kDa	Ref	-0.1	0.7	0.4	1.3	-1.4	0	0.3	0.8
375	seq=translation; coord=6:79193305..79195490:1; parent_transcript=GRMZM2G127948_T01; parent_gene=GRMZM2G127948	GRMZM2G127948_P01	29 kDa	Ref	0.7	1.2	0.9	0.2	0.1	0.8	0.6	0.8
376	seq=translation; coord=3:231823510..231824603:1; parent_transcript=GRMZM2G107839_T01; parent_gene=GRMZM2G107839	GRMZM2G107839_P01 (+1)	16 kDa	Ref	2	2.3	1.5	1.6	0	-0.7	-0.3	-0.1

377	seq=translation; coord=4:239151749..239155703:-1; parent_transcript=GRMZM2G024310_T01; parent_gene=GRMZM2G024310	GRMZM2G024310_P01	28 kDa	Ref	1.4	0.8	1.5	0.6	No Values	No Values	No Values	No Values
378	seq=translation; coord=9:145346839..145349218:-1; parent_transcript=GRMZM2G067225_T01; parent_gene=GRMZM2G067225	GRMZM2G067225_P01	53 kDa	Ref	1	0.6	1	1.7	No Values	No Values	No Values	No Values
379	seq=translation; coord=5:163984090..163986394:1; parent_transcript=GRMZM2G143008_T01; parent_gene=GRMZM2G143008	GRMZM2G143008_P01	69 kDa	Ref	No Values	No Values	No Values	No Values	1.2	1.5	1.2	0.9
380	seq=translation; coord=2:232101270..232109417:-1; parent_transcript=GRMZM2G036034_T01; parent_gene=GRMZM2G036034	GRMZM2G036034_P01 (+1)	99 kDa	Ref	-0.9	-1.1	-1.2	-1.1	2.6	1.4	2.5	2.8
381	seq=translation; coord=1:26684241..26696594:-1; parent_transcript=GRMZM2G099628_T02; parent_gene=GRMZM2G099628	GRMZM2G099628_P02	89 kDa	Ref	-1	-1	-0.7	-1.2	-1.3	-0.9	-0.8	-0.8
382	seq=translation; coord=3:12189233..12194178:1; parent_transcript=GRMZM2G054559_T01; parent_gene=GRMZM2G054559	GRMZM2G054559_P01 (+1)	92 kDa	Ref	-0.5	-0.5	-0.7	-0.1	3.2	2.6	2.8	3.2
383	seq=translation; coord=2:31644513..31646214:-1; parent_transcript=GRMZM2G083253_T02; parent_gene=GRMZM2G083253	GRMZM2G083253_P02 (+6)	17 kDa	Ref	-0.5	-0.4	-0.9	-1.5	0.1	0.9	0.6	-0.6
384	seq=translation; coord=7:150542721..150550925:1; parent_transcript=GRMZM2G138220_T01; parent_gene=GRMZM2G138220	GRMZM2G138220_P01 (+1)	21 kDa	Ref	-0.7	-0.7	-0.4	-0.8	-1.7	-1.6	-1.3	-1.3
385	seq=translation; coord=9:37790950..37793111:1; parent_transcript=GRMZM2G118873_T01; parent_gene=GRMZM2G118873	GRMZM2G118873_P01 (+2)	25 kDa	Ref	3.3	-0.8	-0.8	-1	4	Value Missing	-0.4	-0.4
386	seq=translation; coord=2:220832499..220836411:-1; parent_transcript=GRMZM2G148769_T01; parent_gene=GRMZM2G148769	GRMZM2G148769_P01 (+1)	35 kDa	Ref	-1.1	-1.4	-0.9	Value Missing	-4.4	-2.9	-2.9	-1.3
387	seq=translation; coord=7:153098952..153101687:1; parent_transcript=GRMZM2G117642_T02; parent_gene=GRMZM2G117642	GRMZM2G117642_P02	19 kDa	Ref	0.7	0.5	-0.5	-0.7	-4.8	-4.8	-4.9	-2.7
388	seq=translation; coord=4:197363864..197368446:-1; parent_transcript=GRMZM2G002220_T01; parent_gene=GRMZM2G002220	GRMZM2G002220_P01	89 kDa	Ref	No Values	No Values	No Values	No Values	1	0.6	0.1	0.1
389	seq=translation; coord=10:34232717..34238135:1; parent_transcript=GRMZM2G016890_T01; parent_gene=GRMZM2G016890	GRMZM2G016890_P01	64 kDa	Ref	-0.8	-1.5	-0.6	-1	No Values	No Values	No Values	No Values

390	seq=translation; coord=8:162756043..162756672:-1; parent_transcript=GRMZM2G170969_T01; parent_gene=GRMZM2G170969	GRMZM2G170969_P01	10 kDa	Ref	2.7	2.4	3.7	2.6	No Values	No Values	No Values	No Values
391	seq=translation; coord=9:24144173..24150987:-1; parent_transcript=GRMZM2G122135_T03; parent_gene=GRMZM2G122135	GRMZM2G122135_P03	66 kDa	Ref	-0.8	-1	0	-0.1	-1.3	0	0	-0.2
392	seq=translation; coord=8:163654067..163656422:-1; parent_transcript=GRMZM2G333861_T01; parent_gene=GRMZM2G333861	GRMZM2G333861_P01	53 kDa	Ref	-0.4	-0.5	0	-0.3	0.6	0.7	0.9	0.9
393	seq=translation; coord=3:127004080..127006073:-1; parent_transcript=GRMZM2G067456_T01; parent_gene=GRMZM2G067456	GRMZM2G067456_P01 (+3)	28 kDa	Ref	0.3	-0.4	-0.7	-0.5	2	0.9	0.3	-0.8
394	seq=translation; coord=3:208332821..208344531:1; parent_transcript=GRMZM2G085078_T01; parent_gene=GRMZM2G085078	GRMZM2G085078_P01 (+1)	239 kDa	Ref	0.1	0.1	0.2	0.7	1.7	1.2	1.3	1.2
395	seq=translation; coord=5:154983595..154988021:1; parent_transcript=GRMZM2G167356_T01; parent_gene=GRMZM2G167356	GRMZM2G167356_P01 (+1)	39 kDa	Ref	-1.4	-1.8	-1.2	-2	-2	-3.4	-2.8	-4
396	seq=translation; coord=4:28340661..28347586:1; parent_transcript=GRMZM2G426591_T01; parent_gene=GRMZM2G426591	GRMZM2G426591_P01 (+2)	49 kDa	Ref	Value Missing	-0.4	-0.3	-0.7	-0.5	-0.4	Value Missing	-1.4
397	seq=translation; coord=3:135594596..135597030:-1; parent_transcript=GRMZM2G108348_T01; parent_gene=GRMZM2G108348	GRMZM2G108348_P01 (+1)	22 kDa	Ref	0.6	0.1	0.1	-0.1	0.9	Reference Missing	1.4	0.7
398	seq=translation; coord=3:197717130..197718439:-1; parent_transcript=GRMZM2G043521_T01; parent_gene=GRMZM2G043521	GRMZM2G043521_P01	28 kDa	Ref	0.8	2.6	1.3	1.5	-1.2	Reference Missing	-0.6	1.5
399	seq=translation; coord=5:72919285..72923425:-1; parent_transcript=GRMZM2G370852_T01; parent_gene=GRMZM2G370852	GRMZM2G370852_P01 (+2)	55 kDa	Ref	-0.2	0.1	-0.1	0.1	-0.6	-0.7	-0.3	-0.5
400	seq=translation; coord=4:175134158..175138628:1; parent_transcript=GRMZM2G070239_T01; parent_gene=GRMZM2G070239	GRMZM2G070239_P01 (+1)	19 kDa	Ref	-0.3	-0.3	-1	-0.8	0.6	-0.4	-0.4	-0.8
401	seq=translation; coord=4:179822113..179823382:-1; parent_transcript=GRMZM2G007729_T02; parent_gene=GRMZM2G007729	GRMZM2G007729_P02	24 kDa	Ref	Reference Missing	-1.2	Reference Missing	-1.2	-0.4	-1	-1.9	-0.8
402	seq=translation; coord=5:68020016..68022909:-1; parent_transcript=GRMZM2G108474_T01; parent_gene=GRMZM2G108474	GRMZM2G108474_P01	19 kDa	Ref	-2.3	Value Missing	-1.7	-2.4	-2.8	-1.5	-2.9	-2.1

403	seq=translation; coord=5:213336713..213337573:1; parent_transcript=GRMZM2G119782_T01; parent_gene=GRMZM2G119782	GRMZM2G119782_P01	11 kDa	Ref	-0.8	-1.1	-0.7	-2	-4	-1.4	-1.5	-0.2
404	seq=translation; coord=3:215654139..215663757:-1; parent_transcript=GRMZM2G125193_T01; parent_gene=GRMZM2G125193	GRMZM2G125193_P01	26 kDa	Ref	0.8	0	1.1	-0.3	0.3	0.9	0.2	1.8
405	seq=translation; coord=7:134970635..134972839:-1; parent_transcript=GRMZM2G052266_T01; parent_gene=GRMZM2G052266	GRMZM2G052266_P01 (+1)	44 kDa	Ref	-1.7	-1.5	-1.8	-1.1	-4.9	-4.5	-5	-5.3
406	seq=translation; coord=3:202362837..202365659:1; parent_transcript=GRMZM2G161868_T01; parent_gene=GRMZM2G161868	GRMZM2G161868_P01	62 kDa	Ref	-1	-0.9	-1.6	-1.8	-0.4	Reference Missing	Reference Missing	0.8
407	seq=translation; coord=8:60632430..60633244:-1; parent_transcript=GRMZM2G050607_T01; parent_gene=GRMZM2G050607	GRMZM2G050607_P01	16 kDa	Ref	No Values	No Values	No Values	No Values	Reference Missing	Reference Missing	0.4	2.1
408	seq=translation; coord=1:15773796..15778609:-1; parent_transcript=GRMZM2G119852_T01; parent_gene=GRMZM2G119852	GRMZM2G119852_P01	52 kDa	Ref	0.1	-0.4	-0.6	-0.5	0.7	0.1	0.1	-0.1
409	seq=translation; coord=8:170316262..170329055:1; parent_transcript=GRMZM2G166345_T02; parent_gene=GRMZM2G166345	GRMZM2G166345_P02	38 kDa	Ref	0	0	-0.3	-0.9	0.6	-0.9	-1.4	-0.9
410	seq=translation; coord=5:75935116..75939320:-1; parent_transcript=GRMZM2G019121_T01; parent_gene=GRMZM2G019121	GRMZM2G019121_P01	61 kDa	Ref	0.4	0.3	0.4	0.2	0.3	0	-0.1	-0.2
411	seq=translation; coord=6:158451653..158457625:-1; parent_transcript=GRMZM2G176396_T01; parent_gene=GRMZM2G176396	GRMZM2G176396_P01 (+2)	42 kDa	Ref	-0.1	-0.3	-0.3	0.1	-0.8	0	-0.2	0.6
412	seq=translation; coord=5:163209220..163228678:1; parent_transcript=GRMZM2G012690_T01; parent_gene=GRMZM2G012690	GRMZM2G012690_P01	47 kDa	Ref	-1.1	-1.3	-1.2	-2.2	-1.2	-2	-2.3	-1.9
413	seq=translation; coord=4:161070624..161077204:-1; parent_transcript=GRMZM2G015875_T01; parent_gene=GRMZM2G015875	GRMZM2G015875_P01	133 kDa	Ref	0.9	0.2	0.5	-0.1	1.1	-0.3	-0.5	-0.6
414	seq=translation; coord=6:71732900..71733812:-1; parent_transcript=GRMZM2G101859_T01; parent_gene=GRMZM2G101859	GRMZM2G101859_P01	16 kDa	Ref	1	0.9	0.6	0	-2.1	-2.7	-3.2	-2.4
415	seq=translation; coord=7:165243551..165244574:-1; parent_transcript=GRMZM2G030731_T01; parent_gene=GRMZM2G030731	GRMZM2G030731_P01 (+1)	16 kDa	Ref	1	0.6	0.2	0.3	1.4	0.3	-0.6	-1.2

416	seq=translation; coord=2:192576240..192582957:1; parent_transcript=GRMZM2G097226_T01; parent_gene=GRMZM2G097226	GRMZM2G097226_P01 (+1)	40 kDa	Ref	-0.1	0	0	0.2	5.7	5.5	5.9	5.6
417	seq=translation; coord=4:230189571..230193924:-1; parent_transcript=GRMZM2G161969_T01; parent_gene=GRMZM2G161969	GRMZM2G161969_P01	40 kDa	Ref	0	-0.1	0	-0.1	2.2	1.5	1.6	2
418	seq=translation; coord=1:46852658..46861936:1; parent_transcript=GRMZM2G010328_T01; parent_gene=GRMZM2G010328	GRMZM2G010328_P01	50 kDa	Ref	0	-0.3	-0.6	-0.3	0.8	0.8	0.7	0.5
419	seq=translation; coord=5:741455..752529:1; parent_transcript=GRMZM2G022258_T01; parent_gene=GRMZM2G022258	GRMZM2G022258_P01 (+1)	122 kDa	Ref	0.1	-0.3	-0.8	-0.4	0.8	-0.4	-0.7	-1.1
420	seq=translation; coord=2:133839420..133842192:1; parent_transcript=GRMZM2G111143_T01; parent_gene=GRMZM2G111143	GRMZM2G111143_P01 (+2)	49 kDa	Ref	-0.3	Value Missing	0.1	0.4	1.1	0.4	1.6	Reference Missing
421	seq=translation; coord=2:4174237..4178923:-1; parent_transcript=GRMZM2G039588_T03; parent_gene=GRMZM2G039588	GRMZM2G039588_P03	38 kDa	Ref	-0.5	-0.3	-0.2	0	-3.3	-2.7	-2.1	-1.6
422	seq=translation; coord=4:155907661..155908892:-1; parent_transcript=GRMZM2G044627_T01; parent_gene=GRMZM2G044627	GRMZM2G044627_P01	26 kDa	Ref	0.6	1.9	0.8	1.2	-2.3	Reference Missing	-0.6	-0.2
423	seq=translation; coord=3:132396184..132400373:-1; parent_transcript=GRMZM2G063949_T01; parent_gene=GRMZM2G063949	GRMZM2G063949_P01 (+2)	44 kDa	Ref	0.1	-0.1	-0.4	-0.4	-2.4	-1.7	-1.6	-1.4
424	seq=translation; coord=6:162010397..162015714:1; parent_transcript=GRMZM2G079263_T01; parent_gene=GRMZM2G079263	GRMZM2G079263_P01 (+2)	52 kDa	Ref	-3.1	-1.2	-1.5	-1.8	-4.2	Value Missing	Value Missing	Value Missing
425	seq=translation; coord=5:142403718..142405631:1; parent_transcript=GRMZM2G412436_T01; parent_gene=GRMZM2G412436	GRMZM2G412436_P01	62 kDa	Ref	No Values	No Values	No Values	No Values	Reference Missing	Reference Missing	Reference Missing	2.1
426	seq=translation; coord=3:47448537..47463984:-1; parent_transcript=GRMZM2G011101_T01; parent_gene=GRMZM2G011101	GRMZM2G011101_P01 (+1)	105 kDa	Ref	0	Value Missing	-0.2	Value Missing	0.4	-0.1	-0.8	-1.2
427	seq=translation; coord=9:143165107..143170341:1; parent_transcript=GRMZM2G147671_T01; parent_gene=GRMZM2G147671	GRMZM2G147671_P01 (+3)	42 kDa	Ref	0	0	-0.2	-0.1	1.1	0.6	0.9	0.9
428	seq=translation; coord=1:202752380..202764238:1; parent_transcript=GRMZM2G389233_T03; parent_gene=GRMZM2G389233	GRMZM2G389233_P03	100 kDa	Ref	0.3	-0.4	-0.3	-0.3	-1.1	-1.7	-1.4	Reference Missing

429	seq=translation; coord=1:290265297..290286811:1; parent_transcript=GRMZM2G082664_T01; parent_gene=GRMZM2G082664	GRMZM2G082664_P01	52 kDa	Ref	0.6	0.8	0.9	0.9	2.6	2.5	2.6	2.2
430	seq=translation; coord=5:10855539..10861668:-1; parent_transcript=GRMZM2G109383_T01; parent_gene=GRMZM2G109383	GRMZM2G109383_P01	71 kDa	Ref	-0.7	-0.3	-0.9	-1	-2.1	-1.8	-1.3	-1.4
431	seq=translation; coord=2:160150779..160152270:1; parent_transcript=GRMZM2G304548_T01; parent_gene=GRMZM2G304548	GRMZM2G304548_P01	20 kDa	Ref	No Values	No Values	No Values	No Values	1.2	Reference Missing	0.1	-0.1
432	seq=translation; coord=1:192404194..192408778:-1; parent_transcript=GRMZM2G096806_T01; parent_gene=GRMZM2G096806	GRMZM2G096806_P01	115 kDa	Ref	No Values	No Values	No Values	No Values	0.1	0.3	0.7	0.2
433	seq=translation; coord=2:139185152..139187472:-1; parent_transcript=GRMZM2G047292_T01; parent_gene=GRMZM2G047292	GRMZM2G047292_P01	54 kDa	Ref	No Values	Reference Missing	Reference Missing	Reference Missing	-8.1	-7.6	-7.3	-7.2
434	seq=translation; coord=5:47578824..47582423:1; parent_transcript=GRMZM2G085967_T01; parent_gene=GRMZM2G085967	GRMZM2G085967_P01 (+1)	35 kDa	Ref	0.3	0.7	-1.3	-1.8	-1.6	-2.3	-1.7	-2.7
435	seq=translation; coord=6:1338452..1339388:-1; parent_transcript=GRMZM2G122357_T01; parent_gene=GRMZM2G122357	GRMZM2G122357_P01	14 kDa	Ref	No Values	No Values	No Values	No Values	-0.4	0	Reference Missing	0.6
436	seq=translation; coord=1:25381004..25387870:-1; parent_transcript=GRMZM2G069765_T01; parent_gene=GRMZM2G069765	GRMZM2G069765_P01	61 kDa	Ref	-0.4	-0.5	-0.4	-0.2	-0.4	0.1	-0.5	-0.7
437	seq=translation; coord=4:125089612..125098586:-1; parent_transcript=GRMZM2G157019_T01; parent_gene=GRMZM2G157019	GRMZM2G157019_P01	28 kDa	Ref	-0.6	-1.2	-1.3	-1.3	0.7	0.7	0.1	0.3
438	seq=translation; coord=5:186082222..186092865:1; parent_transcript=GRMZM2G064695_T04; parent_gene=GRMZM2G064695	GRMZM2G064695_P04	45 kDa	Ref	-0.8	-0.9	0	0.1	-0.2	-0.1	0	0.5
439	seq=translation; coord=3:3887574..3888713:1; parent_transcript=GRMZM2G093405_T01; parent_gene=GRMZM2G093405	GRMZM2G093405_P01 (+1)	24 kDa	Ref	1	1.3	0.4	-0.6	0.7	0.9	0.6	0.7
440	seq=translation; coord=5:157277567..157288178:1; parent_transcript=GRMZM2G124886_T01; parent_gene=GRMZM2G124886	GRMZM2G124886_P01 (+8)	104 kDa	Ref	0.2	0	-0.4	-0.3	2.2	1.7	0.9	0.4
441	seq=translation; coord=7:174582270..174585306:-1; parent_transcript=GRMZM2G056569_T01; parent_gene=GRMZM2G056569	GRMZM2G056569_P01	49 kDa	Ref	0.1	0.3	0.1	-0.4	4.1	Reference Missing	5.1	3.9

442	seq=translation; coord=8:116533013..116535098:1; parent_transcript=GRMZM2G009936_T01; parent_gene=GRMZM2G009936	GRMZM2G009936_P01 (+2)	13 kDa	Ref	1.3	1.3	0.5	-0.3	1.3	-0.3	-0.3	-0.7
443	seq=translation; coord=1:62530194..62532719:1; parent_transcript=GRMZM2G577677_T01; parent_gene=GRMZM2G577677	GRMZM2G577677_P01	15 kDa	Ref	0.1	-0.3	-0.6	-1.1	-0.2	-0.2	0.2	-0.3
444	seq=translation; coord=9:106627644..106628551:-1; parent_transcript=GRMZM2G361699_T01; parent_gene=GRMZM2G361699	GRMZM2G361699_P01	12 kDa	Ref	0.3	0.1	-0.1	-0.3	-4	-2.8	Reference Missing	-2.6
445	seq=translation; coord=7:116376688..116380988:1; parent_transcript=GRMZM2G116689_T01; parent_gene=GRMZM2G116689	GRMZM2G116689_P01 (+1)	17 kDa	Ref	0.7	-0.2	1	0.4	-1.2	0.1	-0.8	0.6
446	seq=translation; coord=8:4232302..4235855:1; parent_transcript=GRMZM2G015361_T01; parent_gene=GRMZM2G015361	GRMZM2G015361_P01 (+17)	24 kDa	Ref	0.2	0.1	-0.2	-0.3	1.8	1.4	0.6	0.3
447	seq=translation; coord=3:228250718..228270546:-1; parent_transcript=GRMZM2G035620_T01; parent_gene=GRMZM2G035620	GRMZM2G035620_P01	31 kDa	Ref	Value Missing	-0.5	Value Missing	Value Missing	-3.4	-2.2	-2	-1.3
448	seq=translation; coord=1:38637065..38637541:1; parent_transcript=AC208204.3_FGT006; parent_gene=AC208204.3_FG006	AC208204.3_FGP006 (+1)	18 kDa	Ref	-0.5	0.8	1.1	-0.2	-0.3	Reference Missing	2	2.9
449	seq=translation; coord=3:168695539..168699190:1; parent_transcript=GRMZM2G156861_T02; parent_gene=GRMZM2G156861	GRMZM2G156861_P02	98 kDa	Ref	1.6	2.2	0.5	0.9	No Values	No Values	No Values	No Values
450	seq=translation; coord=4:193146957..193151153:1; parent_transcript=GRMZM5G821551_T02; parent_gene=GRMZM5G821551	GRMZM5G821551_P02 (+1)	70 kDa	Ref	-0.7	-0.9	-1.1	-1.2	-3	-3.2	-3.4	-3.7
451	seq=translation; coord=1:221739264..221743951:-1; parent_transcript=GRMZM2G450163_T01; parent_gene=GRMZM2G450163	GRMZM2G450163_P01	67 kDa	Ref	-0.4	-0.2	-0.3	-0.5	0.2	0.8	0.7	0.5
452	seq=translation; coord=6:4896839..4897613:-1; parent_transcript=GRMZM2G391364_T01; parent_gene=GRMZM2G391364	GRMZM2G391364_P01	17 kDa	Ref	0.6	0.1	1	-0.6	0	1.1	1	2.3
453	seq=translation; coord=5:185405145..185408787:1; parent_transcript=GRMZM2G148387_T01; parent_gene=GRMZM2G148387	GRMZM2G148387_P01	14 kDa	Ref	0.2	-1.5	0.3	0	-5	-4.5	-4.1	-3.2
454	seq=translation; coord=3:141261688..141325830:1; parent_transcript=GRMZM2G073584_T02; parent_gene=GRMZM2G073584	GRMZM2G073584_P02	102 kDa	Ref	-0.2	0	-0.5	0.3	-1.6	-0.7	-1.5	-1.2

455	seq=translation; coord=10:148228962..148233420:1; parent_transcript=GRMZM2G008714_T01; parent_gene=GRMZM2G008714	GRMZM2G008714_P01 (+1)	66 kDa	Ref	-0.9	-0.5	-0.1	0.5	5.1	4.8	4.9	4.7
456	seq=translation; coord=5:213904655..213909590:1; parent_transcript=GRMZM5G836910_T01; parent_gene=GRMZM5G836910	GRMZM5G836910_P01	50 kDa	Ref	No Values	No Values	No Values	No Values	0.4	1.4	Reference Missing	Reference Missing
457	seq=translation; coord=1:91875822..91877199:-1; parent_transcript=GRMZM2G176595_T01; parent_gene=GRMZM2G176595	GRMZM2G176595_P01	30 kDa	Ref	0.8	1.1	1.9	1.7	No Values	No Values	No Values	No Values
458	seq=translation; coord=10:23213183..23215664:1; parent_transcript=GRMZM2G036427_T01; parent_gene=GRMZM2G036427	GRMZM2G036427_P01 (+1)	18 kDa	Ref	0.7	1.8	2	1.9	No Values	No Values	No Values	No Values
459	seq=translation; coord=9:136016928..136020802:-1; parent_transcript=GRMZM2G152417_T01; parent_gene=GRMZM2G152417	GRMZM2G152417_P01	63 kDa	Ref	-1	-0.2	-0.5	-0.5	-0.4	0.2	-0.3	-0.7
460	seq=translation; coord=8:77429944..77433319:1; parent_transcript=GRMZM2G464401_T01; parent_gene=GRMZM2G464401	GRMZM2G464401_P01	39 kDa	Ref	2	1.7	1.5	1.1	Reference Missing	Reference Missing	1.3	1.4
461	seq=translation; coord=3:43991720..43996960:1; parent_transcript=GRMZM2G018566_T01; parent_gene=GRMZM2G018566	GRMZM2G018566_P01 (+1)	40 kDa	Ref	-1.2	-1.1	-1	-0.7	0.8	1.7	0.5	0.5
462	seq=translation; coord=2:176644918..176648601:1; parent_transcript=GRMZM2G150616_T01; parent_gene=GRMZM2G150616	GRMZM2G150616_P01	29 kDa	Ref	0.3	0.4	0.3	0.3	2.7	0.1	-0.2	-1
463	seq=translation; coord=4:154624996..154627067:1; parent_transcript=GRMZM2G156785_T01; parent_gene=GRMZM2G156785	GRMZM2G156785_P01 (+2)	15 kDa	Ref	2.4	1	0.7	0.2	3.8	2.5	2.7	2
464	seq=translation; coord=9:23256308..23260236:-1; parent_transcript=GRMZM2G024993_T01; parent_gene=GRMZM2G024993	GRMZM2G024993_P01 (+1)	67 kDa	Ref	-0.3	0.6	0.2	0.3	-1.5	-2.3	Reference Missing	-0.9
465	seq=translation; coord=4:37762302..37763543:-1; parent_transcript=GRMZM2G112050_T01; parent_gene=GRMZM2G112050	GRMZM2G112050_P01 (+2)	16 kDa	Ref	1.2	1.1	0.5	0.2	Reference Missing	Reference Missing	3.2	2.9
466	seq=translation; coord=9:28603999..28607933:1; parent_transcript=GRMZM2G153969_T01; parent_gene=GRMZM2G153969	GRMZM2G153969_P01	18 kDa	Ref	-1.1	-1.1	-1.1	-2.2	-0.7	-3.9	-3.2	-4
467	seq=translation; coord=3:197406823..197407784:-1; parent_transcript=GRMZM2G010762_T01; parent_gene=GRMZM2G010762	GRMZM2G010762_P01	23 kDa	Ref	1.2	0.9	2.4	1.9	0.8	-0.7	2.4	3.1

468	seq=translation; coord=1:7192641..7198439:-1; parent_transcript=GRMZM2G057158_T01; parent_gene=GRMZM2G057158	GRMZM2G057158_P01 (+1)	56 kDa	Ref	0.1	-0.3	0	-1	-0.4	-0.5	-0.8	-0.6
469	seq=translation; coord=2:110896551..110900469:-1; parent_transcript=GRMZM2G033894_T01; parent_gene=GRMZM2G033894	GRMZM2G033894_P01	46 kDa	Ref	0.5	0.1	0.3	0.1	0.1	-0.5	-0.8	-1
470	seq=translation; coord=8:116617793..116620719:-1; parent_transcript=GRMZM5G802801_T01; parent_gene=GRMZM5G802801	GRMZM5G802801_P01	71 kDa	Ref	No Values	No Values	No Values	No Values	-0.7	0.6	0.2	0.4
471	seq=translation; coord=2:125696253..125710943:1; parent_transcript=GRMZM5G877316_T02; parent_gene=GRMZM5G877316	GRMZM5G877316_P02 (+1)	47 kDa	Ref	0.7	0.3	0	0.3	-0.2	0.3	-0.5	-0.6
472	seq=translation; coord=6:120018887..120020772:1; parent_transcript=GRMZM2G383404_T01; parent_gene=GRMZM2G383404	GRMZM2G383404_P01	52 kDa	Ref	0.4	1.4	0.6	0.4	No Values	No Values	No Values	No Values
473	seq=translation; coord=2:60415730..60417474:1; parent_transcript=GRMZM2G085260_T01; parent_gene=GRMZM2G085260	GRMZM2G085260_P01	34 kDa	Ref	No Values	No Values	No Values	No Values	0	1	1.6	3.3
474	seq=translation; coord=10:22319764..22321055:-1; parent_transcript=GRMZM2G075283_T01; parent_gene=GRMZM2G075283	GRMZM2G075283_P01	17 kDa	Ref	0.3	0.2	2.2	2.9	No Values	No Values	No Values	No Values
475	seq=translation; coord=5:208194750..208197747:1; parent_transcript=GRMZM2G061950_T01; parent_gene=GRMZM2G061950	GRMZM2G061950_P01	35 kDa	Ref	0.1	-0.4	0.1	-0.4	No Values	No Values	No Values	No Values
476	seq=translation; coord=2:1436938..1441569:-1; parent_transcript=GRMZM2G019500_T01; parent_gene=GRMZM2G019500	GRMZM2G019500_P01	32 kDa	Ref	0.8	0.4	0.5	-0.4	-6.3	-7.1	-7.5	-7.4
477	seq=translation; coord=3:126628409..126636305:-1; parent_transcript=GRMZM2G066650_T01; parent_gene=GRMZM2G066650	GRMZM2G066650_P01	96 kDa	Ref	-0.6	-0.5	0.3	0	1.1	0.8	-0.2	-0.2
478	seq=translation; coord=1:236088923..236128384:-1; parent_transcript=GRMZM2G053764_T01; parent_gene=GRMZM2G053764	GRMZM2G053764_P01 (+3)	42 kDa	Ref	-0.8	0	-0.6	-0.8	-0.5	-0.4	0.1	-0.5
479	seq=translation; coord=4:233821047..233828196:1; parent_transcript=GRMZM2G064023_T01; parent_gene=GRMZM2G064023	GRMZM2G064023_P01 (+3)	52 kDa	Ref	No Values	No Values	No Values	No Values	-0.3	0.3	-0.4	-0.3
480	seq=translation; coord=8:6456722..6458297:1; parent_transcript=GRMZM2G328094_T01; parent_gene=GRMZM2G328094	GRMZM2G328094_P01	39 kDa	Ref	0.1	-0.2	-0.6	-0.2	0	-0.3	-0.3	-0.3

481	seq=translation; coord=5:215477924..215482853:1; parent_transcript=GRMZM2G078396_T01; parent_gene=GRMZM2G078396	GRMZM2G078396_P01 (+4)	15 kDa	Ref	1.1	1.2	0.7	0.9	1.6	0.9	0.5	-0.6
482	seq=translation; coord=3:6926705..6931400:-1; parent_transcript=GRMZM2G166646_T01; parent_gene=GRMZM2G166646	GRMZM2G166646_P01	54 kDa	Ref	1.7	0.7	1.3	0.8	3.7	2.3	2.4	1.5
483	seq=translation; coord=7:19072056..19090250:-1; parent_transcript=GRMZM2G462325_T01; parent_gene=GRMZM2G462325	GRMZM2G462325_P01	49 kDa	Ref	No Values	No Values	No Values	No Values	1.3	1.2	1	0.3
484	seq=translation; coord=1:273865741..273870770:-1; parent_transcript=GRMZM2G416388_T01; parent_gene=GRMZM2G416388	GRMZM2G416388_P01 (+3)	22 kDa	Ref	-1.2	-1.3	-0.6	-1.1	-2.7	-2	-2.2	-2.1
485	seq=translation; coord=1:52807627..52810251:-1; parent_transcript=GRMZM2G018728_T01; parent_gene=GRMZM2G018728	GRMZM2G018728_P01	14 kDa	Ref	-0.6	-1.3	-1.6	-2.1	-4.4	-3.4	-3.7	-2.6
486	seq=translation; coord=1:79131877..79132681:-1; parent_transcript=GRMZM2G401328_T01; parent_gene=GRMZM2G401328	GRMZM2G401328_P01	19 kDa	Ref	-0.4	-0.7	0.3	0.9	-3	-2.3	-1	-0.4
487	seq=translation; coord=8:162579348..162580103:-1; parent_transcript=GRMZM2G030717_T01; parent_gene=GRMZM2G030717	GRMZM2G030717_P01	16 kDa	Ref	-0.1	0.6	1	0.8	Reference Missing	Reference Missing	Reference Missing	4
488	seq=translation; coord=8:133178452..133180059:1; parent_transcript=GRMZM2G005552_T01; parent_gene=GRMZM2G005552	GRMZM2G005552_P01	38 kDa	Ref	-0.1	-0.5	-0.2	-0.3	Value Missing	Value Missing	Value Missing	Value Missing
489	seq=translation; coord=7:168744978..168747272:1; parent_transcript=GRMZM2G039757_T01; parent_gene=GRMZM2G039757	GRMZM2G039757_P01	72 kDa	Ref	-0.1	0.4	0.9	1.3	5.8	5.8	6.8	7.1
490	seq=translation; coord=4:131010547..131027664:-1; parent_transcript=GRMZM2G006672_T02; parent_gene=GRMZM2G006672	GRMZM2G006672_P02	40 kDa	Ref	0.4	0.3	0.3	1.1	Reference Missing	Reference Missing	0.9	Reference Missing
491	seq=translation; coord=7:159130478..159136959:-1; parent_transcript=GRMZM2G107562_T01; parent_gene=GRMZM2G107562	GRMZM2G107562_P01	18 kDa	Ref	-1	-1	-1.2	-1.4	-2.6	-2.4	-3	-2.9
492	seq=translation; coord=2:22990515..22995670:1; parent_transcript=GRMZM2G122937_T01; parent_gene=GRMZM2G122937	GRMZM2G122937_P01 (+1)	22 kDa	Ref	2.3	1.6	2	0.3	No Values	No Values	No Values	No Values
493	seq=translation; coord=5:210169906..210174618:-1; parent_transcript=GRMZM2G015401_T01; parent_gene=GRMZM2G015401	GRMZM2G015401_P01 (+1)	39 kDa	Ref	0.9	0.7	0.2	1.2	2.4	0.1	0	-1.1

494	seq=translation; coord=7:127859191..127864034:-1; parent_transcript=GRMZM2G140614_T01; parent_gene=GRMZM2G140614	GRMZM2G140614_P01 (+1)	68 kDa	Ref	0.1	0	-0.1	0	3.4	2.8	3.1	2.6
495	seq=translation; coord=1:217021624..217059348:1; parent_transcript=GRMZM2G090542_T01; parent_gene=GRMZM2G090542	GRMZM2G090542_P01	98 kDa	Ref	-0.1	-0.1	-0.1	0	0.4	-0.3	0.1	0
496	seq=translation; coord=3:171894936..171898201:1; parent_transcript=GRMZM2G167637_T01; parent_gene=GRMZM2G167637	GRMZM2G167637_P01	59 kDa	Ref	-0.2	-0.1	-0.3	0.3	-0.6	-0.4	0.3	0.7
497	seq=translation; coord=1:267609498..267614894:-1; parent_transcript=GRMZM2G010054_T01; parent_gene=GRMZM2G010054	GRMZM2G010054_P01 (+1)	136 kDa	Ref	0.2	0.1	-0.1	-0.2	1.2	0.6	0.4	-0.2
498	seq=translation; coord=5:202811453..202814808:1; parent_transcript=GRMZM2G180930_T02; parent_gene=GRMZM2G180930	GRMZM2G180930_P02 (+1)	36 kDa	Ref	-0.1	0.1	-0.4	-0.1	-0.9	-1	-0.9	-0.5
499	seq=translation; coord=2:233428481..233430995:-1; parent_transcript=GRMZM2G018607_T01; parent_gene=GRMZM2G018607	GRMZM2G018607_P01	78 kDa	Ref	1.8	2.1	2.1	1.1	Reference Missing	1.1	3.4	Reference Missing
500	seq=translation; coord=6:56216948..56219146:-1; parent_transcript=GRMZM2G385287_T01; parent_gene=GRMZM2G385287	GRMZM2G385287_P01	21 kDa	Ref	0	-0.1	-0.1	-0.3	0.8	0.6	0.1	-0.3
501	seq=translation; coord=7:150418464..150423441:1; parent_transcript=GRMZM2G075719_T01; parent_gene=GRMZM2G075719	GRMZM2G075719_P01 (+1)	23 kDa	Ref	-0.3	-0.1	-0.5	-0.2	1.5	1.6	1.6	1.7
502	seq=translation; coord=8:173702854..173715423:1; parent_transcript=GRMZM2G110714_T01; parent_gene=GRMZM2G110714	GRMZM2G110714_P01 (+1)	110 kDa	Ref	-0.7	-1.2	-0.9	-0.7	0.4	0.9	0.7	1.3
503	seq=translation; coord=6:138057377..138059762:-1; parent_transcript=GRMZM2G078985_T01; parent_gene=GRMZM2G078985	GRMZM2G078985_P01 (+2)	22 kDa	Ref	0.6	0	1	0.1	-0.4	0.7	0.1	0.2
504	seq=translation; coord=6:108069110..108070157:1; parent_transcript=GRMZM2G073150_T01; parent_gene=GRMZM2G073150	GRMZM2G073150_P01	16 kDa	Ref	1.2	0.9	1.5	0.3	2	2.1	1.2	2.3
505	seq=translation; coord=8:63271714..63275991:-1; parent_transcript=GRMZM2G168681_T01; parent_gene=GRMZM2G168681	GRMZM2G168681_P01	40 kDa	Ref	No Values	No Values	No Values	No Values	1.6	1.1	1.2	3
506	seq=translation; coord=3:186781963..186783499:-1; parent_transcript=GRMZM2G460860_T01; parent_gene=GRMZM2G460860	GRMZM2G460860_P01	35 kDa	Ref	Reference Missing	Reference Missing	Reference Missing	Reference Missing	-3.1	-4.5	Reference Missing	-6

507	seq=translation; coord=8:69592559..69594672:1; parent_transcript=GRMZM2G001514_T01; parent_gene=GRMZM2G001514	GRMZM2G001514_P01	29 kDa	Ref	-1.5	-0.8	-1.6	-2.1	-1.8	-2.6	-2.6	-3.3
508	seq=translation; coord=6:142310594..142315324:-1; parent_transcript=GRMZM2G137535_T01; parent_gene=GRMZM2G137535	GRMZM2G137535_P01 (+1)	35 kDa	Ref	0.2	-0.1	1.5	3	No Values	No Values	No Values	No Values
509	seq=translation; coord=5:169817842..169818381:1; parent_transcript=AC233850.1_FGT002; parent_gene=AC233850.1_FG002	AC233850.1_FGP002	19 kDa	Ref	No Values	No Values	No Values	No Values	Reference Missing	Reference Missing	Reference Missing	Reference Missing
510	seq=translation; coord=6:69568276..69571793:-1; parent_transcript=GRMZM2G152775_T04; parent_gene=GRMZM2G152775	GRMZM2G152775_P04	26 kDa	Ref	-1.2	-0.8	-0.8	-0.7	-1.1	-1.7	-1.6	-1.9
511	seq=translation; coord=4:193735481..193760758:1; parent_transcript=AC215244.3_FGT002; parent_gene=AC215244.3_FG002	AC215244.3_FGP002	36 kDa	Ref	2.7	2.9	2.3	2	2	2	1.6	3.6
512	seq=translation; coord=2:2326285..2329820:1; parent_transcript=GRMZM2G077541_T01; parent_gene=GRMZM2G077541	GRMZM2G077541_P01 (+1)	41 kDa	Ref	-0.2	0	0	0.1	-1.2	-0.8	Reference Missing	-0.1
513	seq=translation; coord=7:41731651..41795285:-1; parent_transcript=GRMZM2G069676_T02; parent_gene=GRMZM2G069676	GRMZM2G069676_P02 (+1)	59 kDa	Ref	-0.3	0	0.1	-0.1	-0.6	-0.3	0.9	0.7
514	seq=translation; coord=6:165533613..165537241:1; parent_transcript=GRMZM5G829778_T01; parent_gene=GRMZM5G829778	GRMZM5G829778_P01	46 kDa	Ref	-0.2	0.1	0.2	0.2	0.8	0.9	1.3	1.6
515	seq=translation; coord=10:106428641..106433063:1; parent_transcript=GRMZM2G139407_T01; parent_gene=GRMZM2G139407	GRMZM2G139407_P01 (+1)	52 kDa	Ref	-0.4	-0.3	-0.5	-0.3	-0.4	-1.3	-0.8	-0.7
516	seq=translation; coord=4:83505768..83508091:1; parent_transcript=GRMZM2G068455_T01; parent_gene=GRMZM2G068455	GRMZM2G068455_P01 (+2)	42 kDa	Ref	-0.1	0.1	0.2	0.2	-1.6	-0.7	-0.6	-0.7
517	seq=translation; coord=3:7404885..7413183:1; parent_transcript=GRMZM2G093050_T01; parent_gene=GRMZM2G093050	GRMZM2G093050_P01 (+1)	112 kDa	Ref	1.7	1	1	0.6	1.7	0.6	0.2	-0.3
518	seq=translation; coord=1:215329050..215330533:1; parent_transcript=GRMZM2G106133_T02; parent_gene=GRMZM2G106133	GRMZM2G106133_P02	20 kDa	Ref	Reference Missing	1.8	1.2	1.3	1.3	-0.3	0	-0.3
519	seq=translation; coord=5:215493131..215497186:1; parent_transcript=GRMZM5G824600_T03; parent_gene=GRMZM5G824600	GRMZM5G824600_P03	41 kDa	Ref	-0.4	-0.4	-0.6	0.3	-2.7	-3.2	-3.2	-3.3

520	seq=translation; coord=7:46478537..46482054:1; parent_transcript=GRMZM2G011129_T01; parent_gene=GRMZM2G011129	GRMZM2G011129_P01	33 kDa	Ref	-0.3	-0.7	-0.4	-1.4	-0.6	-1.3	-0.8	-0.7
521	seq=translation; coord=1:10991438..10998924:-1; parent_transcript=GRMZM2G056870_T01; parent_gene=GRMZM2G056870	GRMZM2G056870_P01	26 kDa	Ref	-0.6	-1.1	1	-0.3	No Values	No Values	No Values	No Values
522	seq=translation; coord=3:223089120..223091094:-1; parent_transcript=GRMZM2G042008_T01; parent_gene=GRMZM2G042008	GRMZM2G042008_P01	50 kDa	Ref	1.3	0.7	0.3	1.1	5.3	2.7	2.7	1.5
523	seq=translation; coord=3:190774183..190777585:1; parent_transcript=GRMZM2G062373_T01; parent_gene=GRMZM2G062373	GRMZM2G062373_P01 (+1)	27 kDa	Ref	1.1	1	0.6	-0.2	-0.1	0.4	0.7	1
524	seq=translation; coord=7:148082947..148083258:1; parent_transcript=AC196961.2_FGT003; parent_gene=AC196961.2_FG003	AC196961.2_FGP003 (+20)	11 kDa	Ref	1.8	0.6	0.3	-0.8	2.7	-1.6	-1	-0.2
525	seq=translation; coord=10:85640717..85645627:1; parent_transcript=GRMZM2G394500_T01; parent_gene=GRMZM2G394500	GRMZM2G394500_P01 (+1)	38 kDa	Ref	0	1.7	1.7	1.7	No Values	No Values	No Values	No Values
526	seq=translation; coord=1:41376931..41378234:-1; parent_transcript=GRMZM2G172574_T01; parent_gene=GRMZM2G172574	GRMZM2G172574_P01	15 kDa	Ref	No Values	No Values	No Values	No Values	-0.3	Reference Missing	2.1	3.5
527	seq=translation; coord=1:96834561..96839284:-1; parent_transcript=GRMZM2G174757_T01; parent_gene=GRMZM2G174757	GRMZM2G174757_P01	83 kDa	Ref	0.1	-0.4	Value Missing	-0.1	0.8	Reference Missing	-0.1	-0.3
528	seq=translation; coord=6:131159543..131163430:-1; parent_transcript=GRMZM2G051630_T01; parent_gene=GRMZM2G051630	GRMZM2G051630_P01	33 kDa	Ref	0.8	0.9	0.7	1.2	2.2	0.9	0.6	-0.6
529	seq=translation; coord=4:19423579..19439397:-1; parent_transcript=GRMZM2G019236_T01; parent_gene=GRMZM2G019236	GRMZM2G019236_P01 (+2)	118 kDa	Ref	-0.8	-1.3	-1.7	-0.7	-0.3	-0.4	-0.1	-0.6
530	seq=translation; coord=7:41420177..41423983:-1; parent_transcript=GRMZM2G014914_T01; parent_gene=GRMZM2G014914	GRMZM2G014914_P01 (+1)	30 kDa	Ref	1.1	0.8	0.1	0.5	3.2	3	2.4	1.4
531	seq=translation; coord=5:205338436..205344337:-1; parent_transcript=GRMZM2G181505_T01; parent_gene=GRMZM2G181505	GRMZM2G181505_P01	46 kDa	Ref	1.1	0.8	0.9	1.5	1.6	2.5	3	3.5
532	seq=translation; coord=8:12989704..12991151:-1; parent_transcript=GRMZM2G097900_T01; parent_gene=GRMZM2G097900	GRMZM2G097900_P01	21 kDa	Ref	0.6	0	-0.3	-0.5	-0.3	0.4	0.3	0.9

533	seq=translation; coord=1:224236124..224244611:-1; parent_transcript=GRMZM2G031572_T01; parent_gene=GRMZM2G031572	GRMZM2G031572_P01	56 kDa	Ref	-2.1	-2.6	-2.5	-2.2	Value Missing	0.3	-0.5	-0.4
534	seq=translation; coord=8:101408887..101413622:1; parent_transcript=GRMZM2G033799_T01; parent_gene=GRMZM2G033799	GRMZM2G033799_P01 (+2)	49 kDa	Ref	-0.1	-0.1	0	0.3	-1.1	-0.4	-0.4	-0.3
535	seq=translation; coord=1:8135964..8147853:-1; parent_transcript=GRMZM2G176397_T01; parent_gene=GRMZM2G176397	GRMZM2G176397_P01 (+1)	74 kDa	Ref	-0.6	-0.7	-0.7	-0.7	1.9	1.5	1.7	1.9
536	seq=translation; coord=3:136407818..136421472:1; parent_transcript=GRMZM2G168510_T02; parent_gene=GRMZM2G168510	GRMZM2G168510_P02	32 kDa	Ref	-0.6	-0.3	-0.5	-0.8	0.4	-0.4	-1	-0.9
537	seq=translation; coord=8:21615163..21616013:1; parent_transcript=GRMZM2G034157_T01; parent_gene=GRMZM2G034157	GRMZM2G034157_P01	18 kDa	Ref	0.5	0.6	Reference Missing	Reference Missing	-1.2	Reference Missing	Reference Missing	2.9
538	seq=translation; coord=5:125802058..125817596:1; parent_transcript=GRMZM2G038126_T01; parent_gene=GRMZM2G038126	GRMZM2G038126_P01 (+3)	53 kDa	Ref	0	Value Missing	Value Missing	-0.6	-6.4	-5.5	-5.7	-6.7
539	seq=translation; coord=1:14449794..14451164:-1; parent_transcript=GRMZM2G063287_T01; parent_gene=GRMZM2G063287	GRMZM2G063287_P01	34 kDa	Ref	0.6	1.1	1.3	3.4	-4.5	Reference Missing	Reference Missing	Reference Missing
540	seq=translation; coord=1:19327055..19329850:-1; parent_transcript=GRMZM2G154007_T01; parent_gene=GRMZM2G154007	GRMZM2G154007_P01	41 kDa	Ref	-0.7	-0.9	-1.2	-1.4	-1	-1.3	-1.3	-1.7
541	seq=translation; coord=5:46946530..46947446:-1; parent_transcript=AC209987.4_FGT010; parent_gene=AC209987.4_FG010	AC209987.4_FGP010	26 kDa	Ref	-0.2	-0.2	1	2.1	-2.4	-1.3	-0.4	0.3
542	seq=translation; coord=10:137458157..137460537:-1; parent_transcript=AC209206.3_FGT014; parent_gene=AC209206.3_FG014	AC209206.3_FGP014	60 kDa	Ref	0.3	0.3	0.8	1.5	No Values	No Values	No Values	No Values
543	seq=translation; coord=1:228643379..228644379:-1; parent_transcript=GRMZM2G153208_T01; parent_gene=GRMZM2G153208	GRMZM2G153208_P01	24 kDa	Ref	-0.5	1.5	1.3	1.8	No Values	No Values	No Values	No Values
544	seq=translation; coord=4:231475847..231481286:1; parent_transcript=GRMZM2G107362_T02; parent_gene=GRMZM2G107362	GRMZM2G107362_P02 (+3)	47 kDa	Ref	-0.3	-0.4	-0.4	-0.4	-0.8	-0.1	-0.1	-0.8
545	seq=translation; coord=5:18452211..18455290:-1; parent_transcript=GRMZM2G066996_T01; parent_gene=GRMZM2G066996	GRMZM2G066996_P01	42 kDa	Ref	-1.3	-1.4	-0.9	-0.6	0.8	1	1.2	1.6

546	seq=translation; coord=9:8196377..8197429:-1; parent_transcript=GRMZM2G150656_T03; parent_gene=GRMZM2G150656	GRMZM2G150656_P03	13 kDa	Ref	-1.6	-1.9	-1.7	-2.6	-2.8	-2.6	-1.9	-1.4
547	seq=translation; coord=7:165014609..165016387:1; parent_transcript=GRMZM2G393671_T01; parent_gene=GRMZM2G393671	GRMZM2G393671_P01	26 kDa	Ref	-0.7	-0.8	-0.4	-0.8	-1.7	-1.2	-1.2	-0.2
548	seq=translation; coord=8:158113092..158118053:-1; parent_transcript=GRMZM2G109472_T01; parent_gene=GRMZM2G109472	GRMZM2G109472_P01 (+1)	51 kDa	Ref	-0.8	-0.9	-1.3	-1.1	0.8	1.1	1.1	1.5
549	seq=translation; coord=7:170244063..170247650:1; parent_transcript=GRMZM2G024484_T01; parent_gene=GRMZM2G024484	GRMZM2G024484_P01 (+2)	55 kDa	Ref	0.7	0.7	-0.1	0.3	1.2	0.1	0.5	0.4
550	seq=translation; coord=9:7615635..7616809:-1; parent_transcript=GRMZM5G898880_T01; parent_gene=GRMZM5G898880	GRMZM5G898880_P01	35 kDa	Ref	-0.9	-0.4	-0.7	-0.2	-0.3	-0.4	-0.1	-0.1
551	seq=translation; coord=2:1928487..1932811:-1; parent_transcript=GRMZM2G407347_T01; parent_gene=GRMZM2G407347	GRMZM2G407347_P01 (+1)	27 kDa	Ref	-0.8	-0.4	-0.1	-1	-4	-4.1	-4.2	-4.4
552	seq=translation; coord=1:68430654..68436197:1; parent_transcript=GRMZM5G866758_T02; parent_gene=GRMZM5G866758	GRMZM5G866758_P02	41 kDa	Ref	-0.1	-0.1	-0.5	0	-0.7	-0.8	-0.7	-1
553	seq=translation; coord=4:14930698..14950684:-1; parent_transcript=GRMZM2G151050_T01; parent_gene=GRMZM2G151050	GRMZM2G151050_P01 (+1)	63 kDa	Ref	Reference Missing	Reference Missing	Reference Missing	0.9	Reference Missing	Reference Missing	Reference Missing	Reference Missing
554	seq=translation; coord=1:39213217..39219522:-1; parent_transcript=GRMZM2G141799_T01; parent_gene=GRMZM2G141799	GRMZM2G141799_P01	29 kDa	Ref	-0.9	-0.9	-1	-0.8	2.4	2.4	2.8	2.9
555	seq=translation; coord=4:53078446..53083109:-1; parent_transcript=GRMZM2G134708_T01; parent_gene=GRMZM2G134708	GRMZM2G134708_P01	46 kDa	Ref	-0.3	-0.4	-0.1	0.3	6.8	5	4.7	Value Missing
556	seq=translation; coord=8:83335717..83339016:1; parent_transcript=GRMZM2G104025_T01; parent_gene=GRMZM2G104025	GRMZM2G104025_P01 (+1)	21 kDa	Ref	0.5	0.5	0.3	0.2	-0.1	-0.6	-1.3	-1.8
557	seq=translation; coord=5:67403566..67405443:1; parent_transcript=GRMZM2G128929_T01; parent_gene=GRMZM2G128929	GRMZM2G128929_P01	38 kDa	Ref	0.3	0.4	0.3	0.3	0.9	0.8	0.8	0.3
558	seq=translation; coord=9:133421526..133422671:1; parent_transcript=GRMZM2G704475_T02; parent_gene=GRMZM2G704475	GRMZM2G704475_P02	38 kDa	Ref	No Values	No Values	No Values	No Values	-0.2	-1.1	-0.4	0.8

559	seq=translation; coord=1:293793122..293796213:1; parent_transcript=GRMZM2G459811_T02; parent_gene=GRMZM2G459811	GRMZM2G459811_P02 (+1)	41 kDa	Ref	1	0.8	0.9	0.6	-1.3	-1.5	-1.8	-0.2
560	seq=translation; coord=5:85252669..85257667:1; parent_transcript=GRMZM2G104632_T01; parent_gene=GRMZM2G104632	GRMZM2G104632_P01	43 kDa	Ref	0.4	0.2	-0.3	-0.1	1.5	1.2	1.4	0.7
561	seq=translation; coord=10:123440513..123444639:1; parent_transcript=GRMZM2G134539_T01; parent_gene=GRMZM2G134539	GRMZM2G134539_P01	33 kDa	Ref	1.7	1.2	1.1	0.8	4.4	4.3	4.6	3.5
562	seq=translation; coord=6:75510288..75518697:1; parent_transcript=GRMZM2G060870_T01; parent_gene=GRMZM2G060870	GRMZM2G060870_P01 (+1)	23 kDa	Ref	-1.2	-1.8	-1.2	-1.4	-2.3	-2	-2.2	-0.8
563	seq=translation; coord=8:14389329..14393515:-1; parent_transcript=GRMZM2G018177_T01; parent_gene=GRMZM2G018177	GRMZM2G018177_P01	27 kDa	Ref	0.1	0.7	0.4	0.3	-0.4	-0.5	0.3	0.6
564	seq=translation; coord=1:3000161..3000884:-1; parent_transcript=GRMZM2G137329_T01; parent_gene=GRMZM2G137329	GRMZM2G137329_P01	10 kDa	Ref	2.3	3.4	2.6	2.3	0.9	0.7	0.3	Reference Missing
565	seq=translation; coord=4:217521316..217523853:1; parent_transcript=GRMZM2G066222_T01; parent_gene=GRMZM2G066222	GRMZM2G066222_P01 (+2)	10 kDa	Ref	0.8	0.4	0.1	0.7	-1.2	-3.5	-3.4	-3.7
566	seq=translation; coord=8:148264071..148273363:1; parent_transcript=GRMZM2G030167_T01; parent_gene=GRMZM2G030167	GRMZM2G030167_P01	27 kDa	Ref	0.2	1	1	0	-1.4	-0.8	-0.9	-0.3
567	seq=translation; coord=3:11529201..11534274:1; parent_transcript=GRMZM2G061830_T02; parent_gene=GRMZM2G061830	GRMZM2G061830_P02	34 kDa	Ref	-0.4	-0.5	-0.2	-0.8	1.6	1.7	1.4	1.5
568	seq=translation; coord=9:124034244..124040497:-1; parent_transcript=GRMZM2G162968_T01; parent_gene=GRMZM2G162968	GRMZM2G162968_P01	109 kDa	Ref	-0.4	-0.6	-0.1	-0.9	0.2	1	1	1.2
569	seq=translation; coord=7:1284259..1285978:1; parent_transcript=GRMZM2G120652_T01; parent_gene=GRMZM2G120652	GRMZM2G120652_P01	44 kDa	Ref	2	1.9	2.4	1.4	-2.3	-3.4	-3.4	-3.8
570	seq=translation; coord=4:69841345..69843586:1; parent_transcript=GRMZM2G100225_T01; parent_gene=GRMZM2G100225	GRMZM2G100225_P01 (+1)	28 kDa	Ref	0.8	0.1	1.1	0	1.6	1.7	1.5	1.7
571	seq=translation; coord=6:157770836..157775279:1; parent_transcript=GRMZM2G424053_T02; parent_gene=GRMZM2G424053	GRMZM2G424053_P02	14 kDa	Ref	-1.7	-1.7	-1.1	-1.3	-1.4	-1.6	-1.5	-0.2

572	seq=translation; coord=2:190158113..190161171:-1; parent_transcript=GRMZM2G365374_T01; parent_gene=GRMZM2G365374	GRMZM2G365374_P01	73 kDa	Ref	-0.4	-0.1	0.8	-0.3	-0.1	-2.3	-2.6	-2.9
573	seq=translation; coord=1:167851430..167863929:-1; parent_transcript=GRMZM2G130034_T01; parent_gene=GRMZM2G130034	GRMZM2G130034_P01 (+1)	50 kDa	Ref	2.9	3	2	2.4	-2.9	-2.7	Reference Missing	-2.9
574	seq=translation; coord=1:233560736..233562244:-1; parent_transcript=GRMZM2G001850_T01; parent_gene=GRMZM2G001850	GRMZM2G001850_P01	21 kDa	Ref	-1.3	-1.9	-1.3	-1.6	-0.4	-2.2	-2.2	-2.8
575	seq=translation; coord=6:43621161..43627397:1; parent_transcript=GRMZM2G025214_T02; parent_gene=GRMZM2G025214	GRMZM2G025214_P02	40 kDa	Ref	No Values	No Values	No Values	No Values	0.4	0.4	0.1	0.1
576	seq=translation; coord=5:10781545..10785536:1; parent_transcript=GRMZM2G145854_T01; parent_gene=GRMZM2G145854	GRMZM2G145854_P01	81 kDa	Ref	0.4	0.2	0.1	0.2	4.6	3.5	2.9	2.8
577	seq=translation; coord=9:94249084..94255465:1; parent_transcript=AC211394.4_FGT004; parent_gene=AC211394.4_FG004	AC211394.4_FGP004	55 kDa	Ref	0	0.8	0.9	0.4	No Values	No Values	No Values	No Values
578	seq=translation; coord=6:115546691..115548383:1; parent_transcript=GRMZM2G156310_T01; parent_gene=GRMZM2G156310	GRMZM2G156310_P01	35 kDa	Ref	0.4	0.3	0.1	0.1	No Values	No Values	No Values	No Values
579	seq=translation; coord=10:96759871..96761276:-1; parent_transcript=GRMZM2G330635_T01; parent_gene=GRMZM2G330635	GRMZM2G330635_P01	25 kDa	Ref	No Values	No Values	No Values	No Values	-0.1	1.5	1.8	1.9
580	seq=translation; coord=6:107821435..107825807:-1; parent_transcript=GRMZM2G135498_T01; parent_gene=GRMZM2G135498	GRMZM2G135498_P01	49 kDa	Ref	1.2	1.2	0.3	1.1	1.2	1.1	Reference Missing	-0.6
581	seq=translation; coord=4:201542946..201547734:-1; parent_transcript=GRMZM2G381744_T01; parent_gene=GRMZM2G381744	GRMZM2G381744_P01	59 kDa	Ref	-1.1	-1.1	-0.9	-1	0.5	0.1	-0.3	-0.9
582	seq=translation; coord=1:247547660..247549896:1; parent_transcript=GRMZM2G027451_T02; parent_gene=GRMZM2G027451	GRMZM2G027451_P02 (+3)	24 kDa	Ref	1.1	0.8	-0.1	0.4	1.1	1.4	0.9	-0.4
583	seq=translation; coord=4:39092198..39097412:-1; parent_transcript=GRMZM2G158153_T01; parent_gene=GRMZM2G158153	GRMZM2G158153_P01 (+1)	73 kDa	Ref	0.9	0.4	0.3	-0.1	0.8	0	-0.1	-0.1
584	seq=translation; coord=1:99538428..99539066:-1; parent_transcript=AC194914.3_FGT002; parent_gene=AC194914.3_FG002	AC194914.3_FGP002	23 kDa	Ref	-0.3	-0.7	-0.2	-0.1	-2	-0.7	-0.6	-1.1

585	seq=translation; coord=6:137412311..137414138:-1; parent_transcript=AC233866.1_FGT006; parent_gene=AC233866.1_FG006	AC233866.1_FGP006 (+3)	49 kDa	Ref	No Values	No Values	No Values	No Values	No Values	No Values	No Values	No Values
586	seq=translation; coord=4:236107257..236109295:-1; parent_transcript=GRMZM2G051208_T01; parent_gene=GRMZM2G051208	GRMZM2G051208_P01	17 kDa	Ref	-0.7	-0.8	-0.8	-1.4	-1.5	-3.4	-2	-3.4
587	seq=translation; coord=5:168671806..168673715:1; parent_transcript=GRMZM2G113332_T01; parent_gene=GRMZM2G113332	GRMZM2G113332_P01	11 kDa	Ref	2.4	1.5	0.8	-0.5	2.5	2.7	3.1	2.9
588	seq=translation; coord=4:194761789..194765861:1; parent_transcript=GRMZM2G149150_T01; parent_gene=GRMZM2G149150	GRMZM2G149150_P01	50 kDa	Ref	Reference Missing	1.4	Reference Missing	0.3	-2.6	-3.1	-3	-3.2
589	seq=translation; coord=9:25922353..25927460:1; parent_transcript=GRMZM2G083716_T01; parent_gene=GRMZM2G083716	GRMZM2G083716_P01 (+3)	64 kDa	Ref	-1.9	-1.3	-0.3	-1.3	-0.6	0	-0.6	-0.8
590	seq=translation; coord=7:86433388..86439128:-1; parent_transcript=GRMZM2G473001_T01; parent_gene=GRMZM2G473001	GRMZM2G473001_P01	110 kDa	Ref	0.1	-0.9	-0.9	-0.9	Value Missing	2.9	Value Missing	2.3
591	seq=translation; coord=3:179988293..179993427:-1; parent_transcript=GRMZM2G014136_T01; parent_gene=GRMZM2G014136	GRMZM2G014136_P01	76 kDa	Ref	-0.4	-0.7	-0.8	-0.1	3.2	2.9	3	3
592	seq=translation; coord=5:14853348..14857185:-1; parent_transcript=GRMZM2G066191_T01; parent_gene=GRMZM2G066191	GRMZM2G066191_P01 (+1)	50 kDa	Ref	0.1	0.6	-0.5	-0.5	1.3	0.2	0.1	-1
593	seq=translation; coord=7:25903581..25911265:1; parent_transcript=GRMZM2G016958_T01; parent_gene=GRMZM2G016958	GRMZM2G016958_P01	48 kDa	Ref	-0.2	0.3	0.6	0.7	-4.9	-3.8	Reference Missing	-3.3
594	seq=translation; coord=10:93684085..93687059:-1; parent_transcript=GRMZM5G833699_T01; parent_gene=GRMZM5G833699	GRMZM5G833699_P01	82 kDa	Ref	No Values	No Values	No Values	No Values	-0.3	2	2.4	2.5
595	seq=translation; coord=7:39873483..39874398:1; parent_transcript=GRMZM5G896560_T01; parent_gene=GRMZM5G896560	GRMZM5G896560_P01	24 kDa	Ref	2.9	3.1	Reference Missing	Reference Missing	No Values	No Values	No Values	No Values
596	seq=translation; coord=2:220539596..220543690:1; parent_transcript=GRMZM2G163421_T01; parent_gene=GRMZM2G163421	GRMZM2G163421_P01	57 kDa	Ref	0.4	1	0.3	0	-2	-2.5	-2.7	-2.2
597	seq=translation; coord=10:119822322..119823346:-1; parent_transcript=GRMZM2G096695_T01; parent_gene=GRMZM2G096695	GRMZM2G096695_P01	16 kDa	Ref	1.2	1.1	0.5	0.3	-3.2	-2.5	-2.6	-1.9

598	seq=translation; coord=4:65004377..65017210:-1; parent_transcript=GRMZM2G002440_T01; parent_gene=GRMZM2G002440	GRMZM2G002440_P01 (+1)	36 kDa	Ref	1.4	1.2	1.2	1	2.8	2.2	1.8	2.2
599	seq=translation; coord=2:207032350..207038547:1; parent_transcript=GRMZM2G039251_T01; parent_gene=GRMZM2G039251	GRMZM2G039251_P01 (+1)	34 kDa	Ref	0	-0.4	-0.6	0.2	-0.3	-1.3	-1	-0.9
600	seq=translation; coord=7:116285614..116288886:-1; parent_transcript=GRMZM2G160770_T01; parent_gene=GRMZM2G160770	GRMZM2G160770_P01	23 kDa	Ref	0.3	-0.4	0	-1.3	-1.8	-0.4	-0.9	-0.5
601	seq=translation; coord=1:296201923..296204120:1; parent_transcript=GRMZM5G874478_T01; parent_gene=GRMZM5G874478	GRMZM5G874478_P01 (+2)	22 kDa	Ref	1.3	1.1	0.6	0.6	2.8	1.4	Reference Missing	1.7
602	seq=translation; coord=2:189276282..189278019:-1; parent_transcript=GRMZM2G004138_T01; parent_gene=GRMZM2G004138	GRMZM2G004138_P01	31 kDa	Ref	0	0.2	1.8	-0.2	-2.9	-1.9	-1.3	-1
603	seq=translation; coord=1:204681269..204683434:-1; parent_transcript=GRMZM2G057608_T01; parent_gene=GRMZM2G057608	GRMZM2G057608_P01 (+5)	12 kDa	Ref	1.9	2	1.2	1.2	1.8	-0.8	-1.2	-0.9
604	seq=translation; coord=4:241233055..241239106:-1; parent_transcript=GRMZM2G134889_T01; parent_gene=GRMZM2G134889	GRMZM2G134889_P01	62 kDa	Ref	0	-0.1	-0.5	-0.3	-4	-4.3	-4.5	-4.2
605	seq=translation; coord=4:63419630..63429299:-1; parent_transcript=GRMZM2G075775_T01; parent_gene=GRMZM2G075775	GRMZM2G075775_P01	23 kDa	Ref	-0.8	-1	-1.7	-1.6	-1.8	-2.5	-3.3	-3.3
606	seq=translation; coord=1:193791442..193794419:-1; parent_transcript=GRMZM2G148744_T01; parent_gene=GRMZM2G148744	GRMZM2G148744_P01 (+2)	19 kDa	Ref	0.7	1.1	0.8	0.5	No Values	Reference Missing	No Values	Reference Missing
607	seq=translation; coord=7:21276565..21279263:-1; parent_transcript=GRMZM2G063340_T01; parent_gene=GRMZM2G063340	GRMZM2G063340_P01 (+4)	15 kDa	Ref	-2.8	-2.5	-1.7	-2.1	-0.3	-2.8	-2.7	-2.9
608	seq=translation; coord=3:33565292..33570884:-1; parent_transcript=GRMZM2G328893_T01; parent_gene=GRMZM2G328893	GRMZM2G328893_P01	32 kDa	Ref	0	0	0	0	4.9	6.4	4.6	4.9
609	seq=translation; coord=9:92916278..92921897:-1; parent_transcript=GRMZM2G326472_T01; parent_gene=GRMZM2G326472	GRMZM2G326472_P01 (+2)	110 kDa	Ref	-0.5	-1.2	-1.7	-1.9	-1.5	-0.8	-0.6	-0.7
610	seq=translation; coord=5:146886556..146888542:-1; parent_transcript=GRMZM2G014444_T01; parent_gene=GRMZM2G014444	GRMZM2G014444_P01 (+4)	20 kDa	Ref	-0.1	-0.4	-0.2	0.1	0.1	-0.7	-1.1	-1.5

611	seq=translation; coord=1:35919768..35937067:-1; parent_transcript=GRMZM2G174589_T01; parent_gene=GRMZM2G174589	GRMZM2G174589_P01	63 kDa	Ref	0	0.1	-0.5	-0.1	-3.1	-3.3	-3.2	-3.9
612	seq=translation; coord=4:183698889..183701902:1; parent_transcript=GRMZM2G088847_T01; parent_gene=GRMZM2G088847	GRMZM2G088847_P01 (+5)	12 kDa	Ref	-1.6	Value Missing	-0.9	Value Missing	-3	-1.6	-1.7	-1.6
613	seq=translation; coord=5:191366452..191369776:1; parent_transcript=GRMZM5G840002_T01; parent_gene=GRMZM5G840002	GRMZM5G840002_P01 (+2)	26 kDa	Ref	-1.4	-0.6	-0.5	-1	-2.9	-2.5	-2.9	-3.3
614	seq=translation; coord=3:213298401..213303662:1; parent_transcript=GRMZM2G033515_T01; parent_gene=GRMZM2G033515	GRMZM2G033515_P01	46 kDa	Ref	Value Missing	Value Missing	Value Missing	-3.7	Value Missing	Value Missing	Value Missing	Value Missing
615	seq=translation; coord=3:158348059..158355046:1; parent_transcript=GRMZM2G139617_T01; parent_gene=GRMZM2G139617	GRMZM2G139617_P01	55 kDa	Ref	1.3	Reference Missing	Reference Missing	-0.3	4.3	3.8	6.5	4.3
616	seq=translation; coord=1:268251834..268259468:1; parent_transcript=GRMZM5G881950_T01; parent_gene=GRMZM5G881950	GRMZM5G881950_P01 (+1)	108 kDa	Ref	Reference Missing	No Values	No Values	No Values	-7.7	-9.1	-8.9	-9.9
617	seq=translation; coord=2:2487249..2492988:-1; parent_transcript=GRMZM2G104430_T01; parent_gene=GRMZM2G104430	GRMZM2G104430_P01 (+1)	51 kDa	Ref	1	1.4	1.1	0.9	-2.3	-1.6	-1.5	-1.5
618	seq=translation; coord=4:110069837..110073965:1; parent_transcript=GRMZM2G139680_T01; parent_gene=GRMZM2G139680	GRMZM2G139680_P01	28 kDa	Ref	No Values	No Values	No Values	No Values	-0.6	-0.4	-0.4	-0.1
619	seq=translation; coord=7:13844415..13850805:1; parent_transcript=GRMZM2G001898_T01; parent_gene=GRMZM2G001898	GRMZM2G001898_P01	43 kDa	Ref	0	0.1	-0.1	0.1	7.4	6.5	6.2	6
620	seq=translation; coord=3:218835389..218837093:-1; parent_transcript=GRMZM2G074097_T01; parent_gene=GRMZM2G074097	GRMZM2G074097_P01	37 kDa	Ref	-0.7	-1.3	-1.6	-2.2	-0.4	-2.2	-1.5	-1.9
621	seq=translation; coord=1:7566145..7569296:1; parent_transcript=GRMZM2G410991_T01; parent_gene=GRMZM2G410991	GRMZM2G410991_P01	37 kDa	Ref	-1.1	0	0.2	0.5	No Values	No Values	No Values	No Values
622	seq=translation; coord=1:8972308..8976833:-1; parent_transcript=GRMZM2G046932_T01; parent_gene=GRMZM2G046932	GRMZM2G046932_P01	81 kDa	Ref	0.5	-0.2	-0.4	0	0.3	0.7	0.3	-0.2
623	seq=translation; coord=4:237041435..237046635:-1; parent_transcript=GRMZM2G009871_T01; parent_gene=GRMZM2G009871	GRMZM2G009871_P01 (+4)	60 kDa	Ref	0.1	-0.3	-0.4	-0.3	-0.1	-0.7	-1	-1.2

624	seq=translation; coord=9:22779373..22783918:1; parent_transcript=GRMZM2G033208_T01; parent_gene=GRMZM2G033208	GRMZM2G033208_P01 (+2)	69 kDa	Ref	0.7	0.6	0.3	1.3	2.7	1.1	0.9	1.2
625	seq=translation; coord=9:138752306..138754760:1; parent_transcript=GRMZM2G162486_T01; parent_gene=GRMZM2G162486	GRMZM2G162486_P01	27 kDa	Ref	-1	-0.8	0.1	0.7	0.3	0.5	0.7	1.5
626	seq=translation; coord=3:230900751..230906058:-1; parent_transcript=GRMZM2G152688_T01; parent_gene=GRMZM2G152688	GRMZM2G152688_P01 (+2)	40 kDa	Ref	0.8	0.3	0.4	-0.2	2.6	2.3	1.6	1.4
627	seq=translation; coord=1:214719298..214723588:1; parent_transcript=GRMZM2G058702_T01; parent_gene=GRMZM2G058702	GRMZM2G058702_P01 (+1)	49 kDa	Ref	-0.4	-0.8	-0.7	-0.2	4.8	3.7	3.6	3.3
628	seq=translation; coord=2:190424050..190431552:1; parent_transcript=GRMZM2G446050_T01; parent_gene=GRMZM2G446050	GRMZM2G446050_P01	170 kDa	Ref	-0.7	-0.8	-1	-1.1	-0.5	-1.7	-2.2	-2.8
629	seq=translation; coord=9:150809669..150813518:1; parent_transcript=GRMZM2G169384_T01; parent_gene=GRMZM2G169384	GRMZM2G169384_P01	44 kDa	Ref	1.3	1.1	0.9	0.1	1.3	-1	-1	-0.6
630	seq=translation; coord=4:54122481..54127439:-1; parent_transcript=GRMZM2G070422_T01; parent_gene=GRMZM2G070422	GRMZM2G070422_P01 (+1)	39 kDa	Ref	-0.1	-0.3	-0.1	-0.4	0.1	-1	-1.2	-1.9
631	seq=translation; coord=6:161725359..161731090:-1; parent_transcript=GRMZM2G074790_T01; parent_gene=GRMZM2G074790	GRMZM2G074790_P01 (+2)	61 kDa	Ref	-0.9	-0.2	-0.1	-0.1	-3	-2.8	-2.5	-2.7
632	seq=translation; coord=6:113343612..113346307:1; parent_transcript=GRMZM2G168119_T01; parent_gene=GRMZM2G168119	GRMZM2G168119_P01	35 kDa	Ref	1.1	0.6	1	0	2.9	2.7	2.6	5.2
633	seq=translation; coord=10:29873986..29885675:1; parent_transcript=GRMZM2G042089_T01; parent_gene=GRMZM2G042089	GRMZM2G042089_P01 (+2)	57 kDa	Ref	0.1	-0.1	-0.5	-0.1	0.5	-0.3	-0.6	-1.1
634	seq=translation; coord=8:40257571..40264535:-1; parent_transcript=GRMZM2G132903_T01; parent_gene=GRMZM2G132903	GRMZM2G132903_P01	79 kDa	Ref	-0.4	-0.7	-1.3	-0.3	4.9	4.1	5.8	4.8
635	seq=translation; coord=9:129405892..129410419:1; parent_transcript=GRMZM2G125148_T01; parent_gene=GRMZM2G125148	GRMZM2G125148_P01 (+2)	62 kDa	Ref	0.5	-0.6	-0.4	-0.9	-1.1	-4.1	-3.3	-3.7
636	seq=translation; coord=7:13173972..13176516:-1; parent_transcript=GRMZM2G144653_T02; parent_gene=GRMZM2G144653	GRMZM2G144653_P02	13 kDa	Ref	-1.8	-1.6	-1.2	-1.5	0.5	0.3	0.3	3.2

637	seq=translation; coord=1:154077677..154081800:1; parent_transcript=GRMZM2G056039_T01; parent_gene=GRMZM2G056039	GRMZM2G056039_P01	71 kDa	Ref	0.9	0.6	0.6	0.2	-0.7	-0.7	-0.8	-1.2
638	seq=translation; coord=4:9677072..9677727:-1; parent_transcript=GRMZM2G419675_T01; parent_gene=GRMZM2G419675	GRMZM2G419675_P01 (+1)	16 kDa	Ref	-0.1	1.7	2.1	2.3	0.2	-0.1	-0.3	0
639	seq=translation; coord=1:25625764..25630712:-1; parent_transcript=GRMZM2G091563_T01; parent_gene=GRMZM2G091563	GRMZM2G091563_P01	58 kDa	Ref	0.2	Value Missing	0.7	-0.3	No Values	No Values	No Values	No Values
640	seq=translation; coord=5:23016144..23019861:1; parent_transcript=GRMZM2G030169_T01; parent_gene=GRMZM2G030169	GRMZM2G030169_P01 (+3)	42 kDa	Ref	0.1	0.2	-0.6	0.2	-0.5	-1.4	-1.6	-2.5
641	seq=translation; coord=8:8351675..8357336:1; parent_transcript=GRMZM2G132796_T01; parent_gene=GRMZM2G132796	GRMZM2G132796_P01	68 kDa	Ref	No Values	No Values	No Values	No Values	Reference Missing	Reference Missing	Reference Missing	Reference Missing
642	seq=translation; coord=4:12082697..12084089:1; parent_transcript=GRMZM2G358153_T01; parent_gene=GRMZM2G358153	GRMZM2G358153_P01	31 kDa	Ref	0.2	0	1.2	2.9	4.2	5	5.2	6
643	seq=translation; coord=3:128521980..128525993:1; parent_transcript=GRMZM2G034083_T01; parent_gene=GRMZM2G034083	GRMZM2G034083_P01	47 kDa	Ref	-1.1	-0.8	-0.7	-0.6	No Values	No Values	No Values	No Values
644	seq=translation; coord=1:298687651..298689984:1; parent_transcript=GRMZM5G821637_T05; parent_gene=GRMZM5G821637	GRMZM5G821637_P05	38 kDa	Ref	1.8	1.6	0.7	-0.2	No Values	No Values	No Values	No Values
645	seq=translation; coord=7:1266505..1269350:-1; parent_transcript=GRMZM2G420743_T01; parent_gene=GRMZM2G420743	GRMZM2G420743_P01	26 kDa	Ref	-0.3	0.8	0	0.6	No Values	No Values	No Values	No Values
646	seq=translation; coord=1:172122450..172128491:-1; parent_transcript=GRMZM2G029262_T01; parent_gene=GRMZM2G029262	GRMZM2G029262_P01 (+2)	39 kDa	Ref	-0.2	-0.2	-0.4	-0.4	0.8	0.4	0.3	0.3
647	seq=translation; coord=1:167864280..167870604:1; parent_transcript=GRMZM2G130095_T01; parent_gene=GRMZM2G130095	GRMZM2G130095_P01 (+1)	54 kDa	Ref	1.4	0.7	1.1	0.5	0.4	-1.2	Reference Missing	-1.3
648	seq=translation; coord=1:230179502..230183279:1; parent_transcript=GRMZM2G131577_T01; parent_gene=GRMZM2G131577	GRMZM2G131577_P01 (+1)	18 kDa	Ref	No Values	No Values	No Values	No Values	No Values	No Values	No Values	No Values
649	seq=translation; coord=2:212180089..212183936:-1; parent_transcript=GRMZM2G365160_T01; parent_gene=GRMZM2G365160	GRMZM2G365160_P01 (+1)	59 kDa	Ref	1	1	0.6	1.1	2.1	1.6	1.2	0.8

650	seq=translation; coord=4:33112293..33117947:1; parent_transcript=GRMZM2G104907_T01; parent_gene=GRMZM2G104907	GRMZM2G104907_P01	27 kDa	Ref	-0.5	-1.1	-0.9	-1.4	-1.2	-2.7	-2.6	-3.2
651	seq=translation; coord=3:168523033..168526394:1; parent_transcript=GRMZM2G086845_T01; parent_gene=GRMZM2G086845	GRMZM2G086845_P01	35 kDa	Ref	-0.5	-0.8	-0.3	-0.6	-0.3	0.2	0.3	0.3
652	seq=translation; coord=4:63703567..63707582:1; parent_transcript=GRMZM2G024959_T01; parent_gene=GRMZM2G024959	GRMZM2G024959_P01 (+1)	45 kDa	Ref	-0.5	-0.8	-0.1	-0.3	0.8	1.7	1.5	0.8
653	seq=translation; coord=4:165966482..165969746:-1; parent_transcript=GRMZM2G125268_T01; parent_gene=GRMZM2G125268	GRMZM2G125268_P01	59 kDa	Ref	-0.5	-1.1	-1.7	-1.5	-2.5	-1.1	-0.6	-0.6
654	seq=translation; coord=10:142067983..142070677:1; parent_transcript=GRMZM2G019325_T01; parent_gene=GRMZM2G019325	GRMZM2G019325_P01 (+1)	18 kDa	Ref	0.3	-0.4	-1.3	-0.6	0.1	-0.7	-1	-1.9
655	seq=translation; coord=2:196050685..196097541:1; parent_transcript=GRMZM2G058105_T01; parent_gene=GRMZM2G058105	GRMZM2G058105_P01	35 kDa	Ref	0.4	0	-0.6	-0.7	1.6	0.9	0.8	0.3
656	seq=translation; coord=10:4243574..4244555:-1; parent_transcript=GRMZM5G898755_T01; parent_gene=GRMZM5G898755	GRMZM5G898755_P01 (+1)	12 kDa	Ref	2	0.8	-0.4	-1	-5.1	-5.4	-5.4	-5.3
657	seq=translation; coord=10:76402669..76405224:1; parent_transcript=GRMZM2G079908_T01; parent_gene=GRMZM2G079908	GRMZM2G079908_P01 (+2)	13 kDa	Ref	2.9	1.4	2.2	0.3	-1.2	-0.8	-1.1	0.5
658	seq=translation; coord=10:14302293..14305526:1; parent_transcript=GRMZM2G106622_T01; parent_gene=GRMZM2G106622	GRMZM2G106622_P01	29 kDa	Ref	0.3	1	1.4	1.8	-1.1	-1	-0.2	0.1
659	seq=translation; coord=4:229408072..229414150:-1; parent_transcript=GRMZM2G162992_T02; parent_gene=GRMZM2G162992	GRMZM2G162992_P02	71 kDa	Ref	0.2	-0.3	-0.4	-1	-0.9	-1.7	-1.1	-2
660	seq=translation; coord=3:230006629..230017669:-1; parent_transcript=GRMZM2G006130_T01; parent_gene=GRMZM2G006130	GRMZM2G006130_P01 (+6)	72 kDa	Ref	0.6	0.2	1.1	1.2	1.2	1.1	2.8	2.9
661	seq=translation; coord=1:214899491..214901246:-1; parent_transcript=AC217050.4_FGT006; parent_gene=AC217050.4_FG006	AC217050.4_FGP006	29 kDa	Ref	2.1	1.5	2	1.5	0.9	-1.2	-1.5	1.4
662	seq=translation; coord=UNKNOWN:5070241..5074601:-1; parent_transcript=GRMZM2G351125_T01; parent_gene=GRMZM2G351125	GRMZM2G351125_P01	146 kDa	Ref	1.1	1	0.8	0.8	-4.9	-5.4	-6.2	-5.8

663	seq=translation; coord=4:197713557..197717259:-1; parent_transcript=GRMZM2G050218_T01; parent_gene=GRMZM2G050218	GRMZM2G050218_P01	40 kDa	Ref	-0.2	-0.8	-0.8	-1.2	3.4	1.6	1.6	1.7
664	seq=translation; coord=9:14322326..14327546:-1; parent_transcript=GRMZM2G014240_T01; parent_gene=GRMZM2G014240	GRMZM2G014240_P01	80 kDa	Ref	1.3	1.5	0.3	1.2	3.5	1.3	0.9	0.2
665	seq=translation; coord=10:1152679..1158648:-1; parent_transcript=GRMZM2G057441_T01; parent_gene=GRMZM2G057441	GRMZM2G057441_P01	117 kDa	Ref	-0.2	-0.4	-0.7	0	-1.6	-2.2	-2	-1.7
666	seq=translation; coord=5:193849196..193850728:-1; parent_transcript=GRMZM2G147014_T01; parent_gene=GRMZM2G147014	GRMZM2G147014_P01	31 kDa	Ref	0.4	1.2	1.8	1.3	No Values	No Values	No Values	No Values
667	seq=translation; coord=8:25334083..25343016:1; parent_transcript=GRMZM2G373928_T01; parent_gene=GRMZM2G373928	GRMZM2G373928_P01 (+1)	19 kDa	Ref	-0.7	-0.9	-0.9	-1	5.9	6.5	6.2	Value Missing
668	seq=translation; coord=4:147218569..147222484:-1; parent_transcript=GRMZM2G070343_T01; parent_gene=GRMZM2G070343	GRMZM2G070343_P01 (+2)	19 kDa	Ref	-0.2	-0.6	0.8	-0.6	-2.9	-1.4	-1.6	0.3
669	seq=translation; coord=8:136129273..136131879:-1; parent_transcript=GRMZM2G100146_T01; parent_gene=GRMZM2G100146	GRMZM2G100146_P01	33 kDa	Ref	No Values	No Values	No Values	No Values	0.5	-0.9	-1.5	-1.5
670	seq=translation; coord=6:6317365..6323357:-1; parent_transcript=GRMZM2G412470_T02; parent_gene=GRMZM2G412470	GRMZM2G412470_P02	79 kDa	Ref	No Values	No Values	No Values	No Values	0.9	0.9	0.4	0.8
671	seq=translation; coord=10:82038497..82043288:1; parent_transcript=GRMZM5G828229_T02; parent_gene=GRMZM5G828229	GRMZM5G828229_P02	54 kDa	Ref	-1.1	-1.5	-1.7	-1.5	No Values	No Values	No Values	No Values
672	seq=translation; coord=8:118103124..118103722:-1; parent_transcript=AC197705.4_FGT003; parent_gene=AC197705.4_FG003	AC197705.4_FGP003	19 kDa	Ref	No Values	No Values	No Values	No Values	0.2	0.1	1.1	3.9
673	seq=translation; coord=3:59325157..59327502:1; parent_transcript=GRMZM2G107228_T01; parent_gene=GRMZM2G107228	GRMZM2G107228_P01	38 kDa	Ref	0.3	0.1	Reference Missing	1.7	No Values	No Values	No Values	No Values
674	seq=translation; coord=3:5595065..5596815:-1; parent_transcript=GRMZM2G143165_T01; parent_gene=GRMZM2G143165	GRMZM2G143165_P01	51 kDa	Ref	0	0	0.3	1.2	0.7	-0.1	0.3	0.6
675	seq=translation; coord=6:49040773..49051761:1; parent_transcript=GRMZM2G700926_T01; parent_gene=GRMZM2G700926	GRMZM2G700926_P01	67 kDa	Ref	-0.4	-0.5	-0.8	-0.5	0.5	1	0.9	0.4

676	seq=translation; coord=3:106336887..106353717:1; parent_transcript=GRMZM2G014805_T01; parent_gene=GRMZM2G014805	GRMZM2G014805_P01	132 kDa	Ref	-0.7	-0.9	-0.9	-0.7	-0.4	-0.9	-1	-1.1
677	seq=translation; coord=1:175294828..175302644:-1; parent_transcript=GRMZM2G022365_T01; parent_gene=GRMZM2G022365	GRMZM2G022365_P01	43 kDa	Ref	1.1	0.1	0.4	0.6	1.5	1.8	1.5	1.2
678	seq=translation; coord=7:155957641..155960914:1; parent_transcript=GRMZM2G173863_T01; parent_gene=GRMZM2G173863	GRMZM2G173863_P01 (+1)	22 kDa	Ref	0.5	0	0	-1	-0.8	-2.1	-2.6	-1.6
679	seq=translation; coord=5:9900674..9908640:1; parent_transcript=GRMZM2G096596_T01; parent_gene=GRMZM2G096596	GRMZM2G096596_P01	52 kDa	Ref	-1.2	-1.2	-0.8	-1.1	0.2	-0.8	-0.4	-1.2
680	seq=translation; coord=2:222875577..222883048:1; parent_transcript=GRMZM2G172101_T01; parent_gene=GRMZM2G172101	GRMZM2G172101_P01 (+2)	52 kDa	Ref	0.2	0.6	0.4	0.5	-0.9	-0.3	-0.1	0
681	seq=translation; coord=8:138510107..138515042:1; parent_transcript=GRMZM2G700683_T01; parent_gene=GRMZM2G700683	GRMZM2G700683_P01	127 kDa	Ref	No Values	No Values	No Values	No Values	0.8	0.7	0.4	0.2
682	seq=translation; coord=7:6064236..6068299:1; parent_transcript=GRMZM2G025977_T01; parent_gene=GRMZM2G025977	GRMZM2G025977_P01 (+2)	40 kDa	Ref	0.6	0.5	1	0.5	-5.7	-5	-4.9	-5.5
683	seq=translation; coord=1:12146189..12150797:1; parent_transcript=GRMZM2G000823_T01; parent_gene=GRMZM2G000823	GRMZM2G000823_P01	52 kDa	Ref	-0.4	-0.5	0	-0.1	1.9	3.1	3.1	3.3
684	seq=translation; coord=8:116620898..116625716:1; parent_transcript=GRMZM5G874500_T02; parent_gene=GRMZM5G874500	GRMZM5G874500_P02 (+1)	65 kDa	Ref	0.5	0.2	-0.1	-0.3	-1.3	-0.9	-0.9	-1.2
685	seq=translation; coord=2:233433323..233435947:-1; parent_transcript=GRMZM2G018197_T01; parent_gene=GRMZM2G018197	GRMZM2G018197_P01	44 kDa	Ref	1	0.6	0.4	0.5	1.9	0.8	0.4	-0.5
686	seq=translation; coord=3:8746797..8754263:1; parent_transcript=GRMZM2G120271_T01; parent_gene=GRMZM2G120271	GRMZM2G120271_P01	28 kDa	Ref	0.1	-0.2	0.1	-0.8	-0.4	-0.8	-0.7	-1.5
687	seq=translation; coord=1:288732967..288734700:1; parent_transcript=GRMZM2G164020_T01; parent_gene=GRMZM2G164020	GRMZM2G164020_P01 (+1)	38 kDa	Ref	-0.2	-1.4	-1.4	-3	-3.1	-4.2	-4.1	-5
688	seq=translation; coord=7:39219675..39225654:-1; parent_transcript=GRMZM2G471269_T04; parent_gene=GRMZM2G471269	GRMZM2G471269_P04	56 kDa	Ref	0.5	1.1	1.1	2	0.8	2.1	2.2	2.8

689	seq=translation; coord=2:176000645..176001838:1; parent_transcript=GRMZM2G046961_T01; parent_gene=GRMZM2G046961	GRMZM2G046961_P01	18 kDa	Ref	0.3	-0.3	-0.6	-0.9	-1.4	-1.8	-1.6	-2
690	seq=translation; coord=3:135219277..135223126:-1; parent_transcript=GRMZM2G150521_T01; parent_gene=GRMZM2G150521	GRMZM2G150521_P01	25 kDa	Ref	-0.5	-1	-0.8	-1.3	-1.2	-3.1	-2.7	-3.5
691	seq=translation; coord=8:78643850..78648764:1; parent_transcript=GRMZM5G892645_T01; parent_gene=GRMZM5G892645	GRMZM5G892645_P01 (+3)	49 kDa	Ref	-0.4	-1.2	-1.4	-0.9	0.4	-0.4	-0.7	-1
692	seq=translation; coord=2:108630218..108681539:-1; parent_transcript=GRMZM2G158043_T02; parent_gene=GRMZM2G158043	GRMZM2G158043_P02	106 kDa	Ref	-1.8	-1.3	-1.2	-1.9	Value Missing	-6.9	-6.6	-6.7
693	seq=translation; coord=10:124299211..124301978:1; parent_transcript=GRMZM2G173878_T01; parent_gene=GRMZM2G173878	GRMZM2G173878_P01 (+1)	23 kDa	Ref	0.5	0.4	0	0.6	4.8	3.9	3.8	4.1
694	seq=translation; coord=3:150265213..150269134:1; parent_transcript=GRMZM2G068665_T01; parent_gene=GRMZM2G068665	GRMZM2G068665_P01	30 kDa	Ref	No Values	No Values	No Values	No Values	0.6	0.8	0.7	0
695	seq=translation; coord=5:203926164..203934465:-1; parent_transcript=GRMZM2G150172_T01; parent_gene=GRMZM2G150172	GRMZM2G150172_P01	18 kDa	Ref	1.3	1.8	1.6	1.4	No Values	No Values	No Values	No Values
696	seq=translation; coord=5:112928080..112930204:-1; parent_transcript=GRMZM2G144081_T01; parent_gene=GRMZM2G144081	GRMZM2G144081_P01 (+1)	47 kDa	Ref	No Values	No Values	No Values	No Values	Reference Missing	2.9	0.8	0
697	seq=translation; coord=3:8884623..8885815:-1; parent_transcript=GRMZM2G083810_T01; parent_gene=GRMZM2G083810	GRMZM2G083810_P01	18 kDa	Ref	No Values	No Values	No Values	No Values	-0.1	0.2	0.7	2.6
698	seq=translation; coord=10:102318701..102320375:-1; parent_transcript=AC204711.3_FGT003; parent_gene=AC204711.3_FG003	AC204711.3_FGP003	16 kDa	Ref	No Values	No Values	No Values	No Values	0.6	0.5	Reference Missing	0.7
699	seq=translation; coord=6:31707585..31709218:-1; parent_transcript=GRMZM2G050412_T01; parent_gene=GRMZM2G050412	GRMZM2G050412_P01	34 kDa	Ref	2.4	1	0.6	0.7	No Values	No Values	No Values	No Values
700	seq=translation; coord=9:128714706..128721565:1; parent_transcript=GRMZM2G095124_T01; parent_gene=GRMZM2G095124	GRMZM2G095124_P01	99 kDa	Ref	-0.1	0	-0.6	-0.5	-0.2	-0.7	-0.7	-0.8
701	seq=translation; coord=2:214832665..214852459:1; parent_transcript=GRMZM2G035985_T01; parent_gene=GRMZM2G035985	GRMZM2G035985_P01 (+1)	122 kDa	Ref	-0.1	-0.4	-0.8	-0.5	1.8	0.7	0.3	-0.2

702	seq=translation; coord=4:234901650..234908817:1; parent_transcript=GRMZM2G143462_T01; parent_gene=GRMZM2G143462	GRMZM2G143462_P01 (+5)	40 kDa	Ref	0	-1	-0.9	-0.8	-1.7	-1.8	-2	-1.6
703	seq=translation; coord=2:212405484..212408361:-1; parent_transcript=GRMZM5G879278_T02; parent_gene=GRMZM5G879278	GRMZM5G879278_P02 (+2)	35 kDa	Ref	Reference Missing	Reference Missing	Reference Missing	Reference Missing	Reference Missing	Reference Missing	Reference Missing	0.2
704	seq=translation; coord=6:157814627..157816731:-1; parent_transcript=GRMZM2G097040_T01; parent_gene=GRMZM2G097040	GRMZM2G097040_P01	18 kDa	Ref	0.7	0.8	0.8	0.2	0.6	0.1	0.4	0.4
705	seq=translation; coord=5:120784559..120792367:1; parent_transcript=GRMZM2G093359_T01; parent_gene=GRMZM2G093359	GRMZM2G093359_P01 (+1)	49 kDa	Ref	No Values	No Values	No Values	No Values	Reference Missing	Reference Missing	Reference Missing	Reference Missing
706	seq=translation; coord=3:185834707..185839417:-1; parent_transcript=GRMZM2G003385_T01; parent_gene=GRMZM2G003385	GRMZM2G003385_P01 (+1)	60 kDa	Ref	0	-0.2	-0.5	0.2	2.3	2.7	2.2	1.5
707	seq=translation; coord=5:188243871..188290615:-1; parent_transcript=GRMZM5G824831_T01; parent_gene=GRMZM5G824831	GRMZM5G824831_P01 (+7)	26 kDa	Ref	0.1	-0.4	-0.3	-0.1	-1.8	-1.9	-1.5	-1.4
708	seq=translation; coord=2:32257322..32262715:1; parent_transcript=GRMZM2G157061_T01; parent_gene=GRMZM2G157061	GRMZM2G157061_P01	86 kDa	Ref	2.7	2.3	2.6	1.8	-0.4	-1	-0.4	-1.4
709	seq=translation; coord=6:112268596..112280791:-1; parent_transcript=GRMZM2G553687_T01; parent_gene=GRMZM2G553687	GRMZM2G553687_P01	204 kDa	Ref	0.2	0.9	-0.4	0.1	2.3	2.3	1.9	1.1
710	seq=translation; coord=3:34547144..34558771:1; parent_transcript=EF517601.1_FGT012; parent_gene=EF517601.1_FG012	EF517601.1_FGP012	87 kDa	Ref	-0.8	-0.8	-0.3	-0.4	-2	-1.5	-1.9	-1.8
711	seq=translation; coord=3:162825591..162831818:-1; parent_transcript=GRMZM2G133173_T01; parent_gene=GRMZM2G133173	GRMZM2G133173_P01	77 kDa	Ref	0.6	0.4	0.5	0.4	-0.2	-1.4	-1.4	-2.1
712	seq=translation; coord=1:143622295..143626633:1; parent_transcript=GRMZM2G152526_T01; parent_gene=GRMZM2G152526	GRMZM2G152526_P01	37 kDa	Ref	0.8	0.3	0.3	-0.4	3.3	2.1	2.2	2.4
713	seq=translation; coord=10:90200523..90201913:-1; parent_transcript=GRMZM2G132093_T01; parent_gene=GRMZM2G132093	GRMZM2G132093_P01	25 kDa	Ref	-0.6	-0.1	1.3	1.3	0.3	0.5	0.6	-0.1
714	seq=translation; coord=5:12565068..12568390:1; parent_transcript=GRMZM2G111566_T01; parent_gene=GRMZM2G111566	GRMZM2G111566_P01	23 kDa	Ref	-1.2	-1.5	-0.4	-0.2	-4.2	-4.7	-4.8	-3.7

715	seq=translation; coord=9:22655251..22657461:1; parent_transcript=GRMZM2G092296_T01; parent_gene=GRMZM2G092296	GRMZM2G092296_P01	14 kDa	Ref	0.2	0.2	0	-0.6	1	0.6	0.8	0.4
716	seq=translation; coord=9:6307326..6312829:-1; parent_transcript=GRMZM2G082198_T01; parent_gene=GRMZM2G082198	GRMZM2G082198_P01	23 kDa	Ref	No Values	No Values	No Values	No Values	-0.3	-0.3	-1.1	-0.3
717	seq=translation; coord=7:120200894..120203011:1; parent_transcript=GRMZM2G138727_T01; parent_gene=GRMZM2G138727	GRMZM2G138727_P01	24 kDa	Ref	1.1	2.2	1	0.6	2	Reference Missing	Value Missing	Value Missing
718	seq=translation; coord=2:34985296..34990180:-1; parent_transcript=GRMZM2G021219_T01; parent_gene=GRMZM2G021219	GRMZM2G021219_P01	41 kDa	Ref	-0.1	-0.1	0.3	0.5	6.9	Value Missing	Value Missing	7.3
719	seq=translation; coord=2:17796828..17799226:-1; parent_transcript=GRMZM2G101408_T01; parent_gene=GRMZM2G101408	GRMZM2G101408_P01 (+2)	16 kDa	Ref	2.7	1.7	2.3	1.1	1.2	1.5	1.4	0.4
720	seq=translation; coord=6:86343600..86346720:-1; parent_transcript=GRMZM2G082823_T01; parent_gene=GRMZM2G082823	GRMZM2G082823_P01	80 kDa	Ref	0.9	Value Missing	-0.1	-0.1	1.6	2.1	2	1.1
721	seq=translation; coord=8:160297692..160299701:-1; parent_transcript=GRMZM2G105005_T01; parent_gene=GRMZM2G105005	GRMZM2G105005_P01 (+2)	37 kDa	Ref	0.7	0.9	1.2	2	-0.6	0.4	0.6	1.8
722	seq=translation; coord=5:14072358..14073760:-1; parent_transcript=GRMZM2G165901_T01; parent_gene=GRMZM2G165901	GRMZM2G165901_P01	16 kDa	Ref	0.5	-1	0.2	0.5	2.1	-1.8	-0.2	0.1
723	seq=translation; coord=4:136936001..136939330:1; parent_transcript=GRMZM2G044684_T01; parent_gene=GRMZM2G044684	GRMZM2G044684_P01	36 kDa	Ref	No Values	No Values	No Values	No Values	-0.2	0	-0.4	2
724	seq=translation; coord=9:64028902..64031969:1; parent_transcript=GRMZM2G469898_T01; parent_gene=GRMZM2G469898	GRMZM2G469898_P01 (+1)	43 kDa	Ref	1.2	0.3	-0.6	-0.6	No Values	No Values	No Values	No Values
725	seq=translation; coord=3:202766518..202768685:-1; parent_transcript=GRMZM2G181135_T01; parent_gene=GRMZM2G181135	GRMZM2G181135_P01	54 kDa	Ref	-0.1	0.5	1.5	2.3	No Values	No Values	No Values	No Values
726	seq=translation; coord=2:189488589..189490512:-1; parent_transcript=GRMZM2G081017_T01; parent_gene=GRMZM2G081017	GRMZM2G081017_P01 (+3)	33 kDa	Ref	0.1	0.6	0.4	0.7	No Values	No Values	No Values	No Values
727	seq=translation; coord=8:118165588..118167724:1; parent_transcript=AC197705.4_FGT001; parent_gene=AC197705.4_FG001	AC197705.4_FGP001	65 kDa	Ref	No Values	No Values	No Values	No Values	2.1	1.2	1.6	1.3

728	seq=translation; coord=8:87034106..87038095:-1; parent_transcript=GRMZM2G007404_T01; parent_gene=GRMZM2G007404	GRMZM2G007404_P01 (+1)	48 kDa	Ref	0.1	0.3	0.3	0.3	No Values	No Values	No Values	No Values
729	seq=translation; coord=1:262889860..262892469:1; parent_transcript=GRMZM2G024131_T01; parent_gene=GRMZM2G024131	GRMZM2G024131_P01 (+1)	50 kDa	Ref	No Values	No Values	No Values	No Values	2.3	3.2	3	2.1
730	seq=translation; coord=3:204689100..204690354:1; parent_transcript=GRMZM2G425629_T02; parent_gene=GRMZM2G425629	GRMZM2G425629_P02	24 kDa	Ref	No Values	No Values	No Values	No Values	Reference Missing	Reference Missing	Reference Missing	3.5
731	seq=translation; coord=2:223290295..223311152:1; parent_transcript=GRMZM2G172369_T03; parent_gene=GRMZM2G172369	GRMZM2G172369_P03	114 kDa	Ref	0.3	0.1	-0.2	0.7	No Values	No Values	No Values	No Values
732	seq=translation; coord=9:151682948..151683563:1; parent_transcript=GRMZM2G428040_T01; parent_gene=GRMZM2G428040	GRMZM2G428040_P01	13 kDa	Ref	No Values	No Values	No Values	No Values	0.4	-0.9	1.3	Reference Missing
733	seq=translation; coord=10:87090618..87092722:1; parent_transcript=GRMZM2G123029_T01; parent_gene=GRMZM2G123029	GRMZM2G123029_P01 (+4)	32 kDa	Ref	0.3	0.6	1.3	2	No Values	No Values	No Values	No Values
734	seq=translation; coord=4:233120026..233126298:-1; parent_transcript=GRMZM2G049839_T01; parent_gene=GRMZM2G049839	GRMZM2G049839_P01 (+1)	45 kDa	Ref	0.1	0.6	0.4	0.3	1.8	1.7	2	2.3
735	seq=translation; coord=6:141280696..141285603:-1; parent_transcript=GRMZM2G084767_T01; parent_gene=GRMZM2G084767	GRMZM2G084767_P01	96 kDa	Ref	0	-0.8	-0.9	-1.2	-1.2	-1	-1.7	-1.8
736	seq=translation; coord=3:217464401..217471380:-1; parent_transcript=GRMZM2G105019_T01; parent_gene=GRMZM2G105019	GRMZM2G105019_P01	40 kDa	Ref	-0.4	-0.2	-0.5	-0.7	-0.9	-0.2	-0.3	-0.5
737	seq=translation; coord=3:111553578..111570930:1; parent_transcript=GRMZM2G411536_T01; parent_gene=GRMZM2G411536	GRMZM2G411536_P01 (+1)	401 kDa	Ref	0.4	0.5	0	0.4	0.7	0.3	0.4	-0.5
738	seq=translation; coord=5:167061160..167068865:-1; parent_transcript=GRMZM2G136296_T01; parent_gene=GRMZM2G136296	GRMZM2G136296_P01 (+1)	99 kDa	Ref	-0.2	0	-0.4	-0.3	-2.2	-2.6	-2.8	-2.9
739	seq=translation; coord=8:115478841..115482432:1; parent_transcript=GRMZM2G111411_T01; parent_gene=GRMZM2G111411	GRMZM2G111411_P01	24 kDa	Ref	0	-0.4	0	Value Missing	-1.2	-2.7	-3.5	-2.8
740	seq=translation; coord=3:1479593..1481033:-1; parent_transcript=GRMZM5G825437_T04; parent_gene=GRMZM5G825437	GRMZM5G825437_P04	15 kDa	Ref	-0.1	-0.5	-0.2	-0.5	-1.4	-2.5	-1.5	-1

741	seq=translation; coord=8:16994084..16999444:-1; parent_transcript=GRMZM2G025215_T01; parent_gene=GRMZM2G025215	GRMZM2G025215_P01	59 kDa	Ref	1.5	0.9	1.4	1.1	3.4	3.2	2.5	3.2
742	seq=translation; coord=4:127851768..127857102:1; parent_transcript=GRMZM2G018416_T01; parent_gene=GRMZM2G018416	GRMZM2G018416_P01	82 kDa	Ref	Value Missing	0.6	-0.2	0.1	-0.8	-2.5	-2.2	-1.7
743	seq=translation; coord=9:18896527..18899443:1; parent_transcript=GRMZM2G102596_T01; parent_gene=GRMZM2G102596	GRMZM2G102596_P01 (+2)	30 kDa	Ref	0.8	0.6	0.4	0.7	1.3	0.8	-0.1	-0.4
744	seq=translation; coord=1:5952987..5954672:1; parent_transcript=GRMZM2G065718_T01; parent_gene=GRMZM2G065718	GRMZM2G065718_P01	41 kDa	Ref	-0.7	0.4	-1	-1.3	-1	-2.2	-2.2	-2
745	seq=translation; coord=3:133888888..133893638:1; parent_transcript=GRMZM2G116204_T01; parent_gene=GRMZM2G116204	GRMZM2G116204_P01	22 kDa	Ref	0	-0.7	-1.3	-0.6	-0.1	0.7	0.7	1.2
746	seq=translation; coord=3:9805845..9808735:-1; parent_transcript=GRMZM2G083418_T01; parent_gene=GRMZM2G083418	GRMZM2G083418_P01	35 kDa	Ref	2.7	2.3	1.8	1.9	Reference Missing	3.3	3.4	Reference Missing
747	seq=translation; coord=10:148849233..148855901:1; parent_transcript=GRMZM2G406074_T01; parent_gene=GRMZM2G406074	GRMZM2G406074_P01	25 kDa	Ref	0.2	-0.1	0.1	-0.1	1.8	2.6	1.6	2.4
748	seq=translation; coord=2:146194741..146200435:-1; parent_transcript=GRMZM2G537291_T01; parent_gene=GRMZM2G537291	GRMZM2G537291_P01	68 kDa	Ref	-1	-1	-0.7	-1.2	-0.4	-0.8	-1	-1.5
749	seq=translation; coord=9:93447494..93469121:-1; parent_transcript=GRMZM2G348666_T01; parent_gene=GRMZM2G348666	GRMZM2G348666_P01 (+1)	133 kDa	Ref	-0.2	-0.2	-0.6	0.2	-3.3	-3.2	-3.2	-3.6
750	seq=translation; coord=2:8906086..8909067:-1; parent_transcript=GRMZM5G876898_T01; parent_gene=GRMZM5G876898	GRMZM5G876898_P01 (+1)	44 kDa	Ref	-0.6	0.1	-0.1	-0.1	-4.1	Reference Missing	Reference Missing	-3.8
751	seq=translation; coord=1:67644786..67647420:1; parent_transcript=GRMZM2G051879_T01; parent_gene=GRMZM2G051879	GRMZM2G051879_P01 (+13)	15 kDa	Ref	1.7	1.7	1	0.3	-3.5	-6.5	-6.2	-4.8
752	seq=translation; coord=1:68543805..68546269:-1; parent_transcript=GRMZM2G077991_T01; parent_gene=GRMZM2G077991	GRMZM2G077991_P01 (+3)	16 kDa	Ref	0.6	0.3	-1.2	-1.6	1.6	Value Missing	-3.3	Value Missing
753	seq=translation; coord=10:8848146..8849526:-1; parent_transcript=GRMZM2G136910_T01; parent_gene=GRMZM2G136910	GRMZM2G136910_P01	16 kDa	Ref	-2.4	-2.7	-2	-2.2	1.1	0.1	0.6	-0.2

754	seq=translation; coord=9:28003772..28008138:1; parent_transcript=GRMZM2G049693_T01; parent_gene=GRMZM2G049693	GRMZM2G049693_P01 (+1)	66 kDa	Ref	Reference Missing	Reference Missing	0.2	1.3	-2.4	-3.7	-3.3	-2.7
755	seq=translation; coord=6:132760746..132763409:1; parent_transcript=GRMZM2G160925_T01; parent_gene=GRMZM2G160925	GRMZM2G160925_P01	41 kDa	Ref	0	-0.1	-0.1	-0.1	-0.3	-0.8	-0.8	-1
756	seq=translation; coord=1:279565504..279568174:1; parent_transcript=GRMZM2G047456_T02; parent_gene=GRMZM2G047456	GRMZM2G047456_P02 (+2)	34 kDa	Ref	-1.1	-0.5	-0.5	-0.7	-0.8	-2.7	-2.1	-1.3
757	seq=translation; coord=8:143279539..143283510:-1; parent_transcript=GRMZM2G050270_T01; parent_gene=GRMZM2G050270	GRMZM2G050270_P01	46 kDa	Ref	-0.6	-0.7	-1	-0.6	-0.5	-1.2	-1	-1
758	seq=translation; coord=5:193157657..193165368:1; parent_transcript=AC211737.3_FGT012; parent_gene=AC211737.3_FG012	AC211737.3_FGP012	75 kDa	Ref	-0.4	-0.7	-0.2	0	6.4	6.5	7.6	Value Missing
759	seq=translation; coord=4:237521017..237524915:-1; parent_transcript=GRMZM2G165917_T01; parent_gene=GRMZM2G165917	GRMZM2G165917_P01 (+3)	49 kDa	Ref	0.6	0.6	1	0.1	3.9	1.9	1.9	2.6
760	seq=translation; coord=5:69096740..69101545:-1; parent_transcript=GRMZM2G020146_T01; parent_gene=GRMZM2G020146	GRMZM2G020146_P01	57 kDa	Ref	-1.4	-1.7	-1.4	-1	0.2	1.1	3.1	2.8
761	seq=translation; coord=10:96835856..96839456:-1; parent_transcript=GRMZM2G021170_T01; parent_gene=GRMZM2G021170	GRMZM2G021170_P01	29 kDa	Ref	-1	-1.9	-1.5	Value Missing	-2.9	-3	-4	-4.3
762	seq=translation; coord=6:164377053..164379535:-1; parent_transcript=GRMZM2G039886_T01; parent_gene=GRMZM2G039886	GRMZM2G039886_P01	39 kDa	Ref	No Values	No Values	No Values	No Values	-0.1	0.8	0.9	1.2
763	seq=translation; coord=6:153256043..153259238:1; parent_transcript=GRMZM2G169943_T01; parent_gene=GRMZM2G169943	GRMZM2G169943_P01	36 kDa	Ref	No Values	No Values	No Values	No Values	-0.2	1.2	1.8	3.2
764	seq=translation; coord=6:156534704..156535855:-1; parent_transcript=GRMZM2G410134_T01; parent_gene=GRMZM2G410134	GRMZM2G410134_P01	22 kDa	Ref	No Values	No Values	No Values	No Values	-0.2	0.3	Reference Missing	-0.7
765	seq=translation; coord=6:157582700..157589894:1; parent_transcript=GRMZM2G006377_T01; parent_gene=GRMZM2G006377	GRMZM2G006377_P01	49 kDa	Ref	0.3	0.6	1.3	1.7	No Values	No Values	No Values	No Values
766	seq=translation; coord=6:63919594..63950390:1; parent_transcript=GRMZM2G052812_T02; parent_gene=GRMZM2G052812	GRMZM2G052812_P02 (+1)	37 kDa	Ref	-0.4	0.2	0.2	0.5	No Values	No Values	No Values	No Values

767	seq=translation; coord=1:41468889..41473581:1; parent_transcript=GRMZM2G044027_T01; parent_gene=GRMZM2G044027	GRMZM2G044027_P01 (+1)	39 kDa	Ref	0.6	1.3	0.7	-0.2	No Values	No Values	No Values	No Values
768	seq=translation; coord=8:17872894..17877539:1; parent_transcript=GRMZM2G080722_T01; parent_gene=GRMZM2G080722	GRMZM2G080722_P01	20 kDa	Ref	0	0	0	-0.3	-1.4	-1.6	-2.5	-2.2
769	seq=translation; coord=1:290521787..290523917:1; parent_transcript=GRMZM2G012224_T01; parent_gene=GRMZM2G012224	GRMZM2G012224_P01	41 kDa	Ref	0.8	-0.4	-1	-0.6	-2	-3	-2.5	-2.8
770	seq=translation; coord=6:148590842..148593721:1; parent_transcript=GRMZM2G033641_T01; parent_gene=GRMZM2G033641	GRMZM2G033641_P01	68 kDa	Ref	0.9	0.8	1.3	1.4	2.7	1.3	1.2	1
771	seq=translation; coord=6:168917945..168919116:1; parent_transcript=GRMZM5G833747_T01; parent_gene=GRMZM5G833747	GRMZM5G833747_P01 (+2)	17 kDa	Ref	-1.5	-1.8	-1.8	-2	1.9	2.6	3.2	3.6
772	seq=translation; coord=1:41535916..41540223:-1; parent_transcript=GRMZM2G157329_T01; parent_gene=GRMZM2G157329	GRMZM2G157329_P01 (+1)	27 kDa	Ref	0.8	0.2	0.3	0.3	3.6	4.2	3.8	2.3
773	seq=translation; coord=5:19123690..19126862:1; parent_transcript=GRMZM2G074898_T01; parent_gene=GRMZM2G074898	GRMZM2G074898_P01 (+8)	18 kDa	Ref	1.1	0.4	-0.3	0	1.7	0.7	0.4	-0.6
774	seq=translation; coord=2:38192410..38194128:1; parent_transcript=GRMZM2G121700_T01; parent_gene=GRMZM2G121700	GRMZM2G121700_P01	32 kDa	Ref	-1.1	-0.8	-1.2	-1.6	-1.8	-1.9	-1.9	-1.8
775	seq=translation; coord=2:17348097..17355999:-1; parent_transcript=GRMZM2G145968_T01; parent_gene=GRMZM2G145968	GRMZM2G145968_P01	14 kDa	Ref	2.2	2	1.8	1.3	2.7	2	2.5	2.3
776	seq=translation; coord=5:180669231..180673418:1; parent_transcript=GRMZM2G139614_T01; parent_gene=GRMZM2G139614	GRMZM2G139614_P01	56 kDa	Ref	3.3	2.8	2.6	2.2	Reference Missing	Reference Missing	Reference Missing	Reference Missing
777	seq=translation; coord=7:10575103..10576227:-1; parent_transcript=GRMZM2G328171_T01; parent_gene=GRMZM2G328171	GRMZM2G328171_P01	33 kDa	Ref	-0.2	-0.1	-0.5	0	3.7	4.6	5.5	6.4
778	seq=translation; coord=4:199076122..199077395:-1; parent_transcript=GRMZM2G177720_T01; parent_gene=GRMZM2G177720	GRMZM2G177720_P01	17 kDa	Ref	-0.1	-0.1	-0.5	-0.3	0.2	0.3	-0.2	Reference Missing
779	seq=translation; coord=2:24608141..24615659:1; parent_transcript=GRMZM2G061928_T01; parent_gene=GRMZM2G061928	GRMZM2G061928_P01 (+2)	24 kDa	Ref	No Values	No Values	No Values	No Values	0.3	-0.2	-0.9	0.1

780	seq=translation; coord=4:195054129..195060931:1; parent_transcript=GRMZM2G084406_T02; parent_gene=GRMZM2G084406	GRMZM2G084406_P02	66 kDa	Ref	-0.1	0.4	0.5	0.4	-5.5	-5.9	-5.8	-5.8
781	seq=translation; coord=6:98313412..98316578:1; parent_transcript=GRMZM2G110402_T01; parent_gene=GRMZM2G110402	GRMZM2G110402_P01 (+2)	24 kDa	Ref	1.1	0.4	0.5	-0.1	-2.3	-2.7	-2.4	-3.1
782	seq=translation; coord=3:151293748..151299116:1; parent_transcript=GRMZM2G030902_T01; parent_gene=GRMZM2G030902	GRMZM2G030902_P01 (+7)	40 kDa	Ref	-1.4	-1.5	-2.1	-2.7	-2.7	-4.5	-4.6	-5.5
783	seq=translation; coord=1:293644603..293647117:-1; parent_transcript=GRMZM2G068244_T01; parent_gene=GRMZM2G068244	GRMZM2G068244_P01	28 kDa	Ref	-0.8	-1.1	-0.2	-1.2	-4.4	-4.2	Reference Missing	-2.8
784	seq=translation; coord=10:103630422..103635991:-1; parent_transcript=GRMZM2G359298_T01; parent_gene=GRMZM2G359298	GRMZM2G359298_P01	83 kDa	Ref	0.8	1.8	1.9	0.6	-0.2	0.2	1.6	0.7
785	seq=translation; coord=8:171328319..171331663:-1; parent_transcript=GRMZM2G172932_T01; parent_gene=GRMZM2G172932	GRMZM2G172932_P01 (+2)	50 kDa	Ref	0.7	-0.1	-0.9	-1.1	-0.3	-1.7	-1.8	-2.9
786	seq=translation; coord=1:16603599..16606666:-1; parent_transcript=GRMZM2G016511_T01; parent_gene=GRMZM2G016511	GRMZM2G016511_P01	27 kDa	Ref	No Values	No Values	No Values	No Values	-0.3	0	-0.1	-0.6
787	seq=translation; coord=5:212248891..212249987:1; parent_transcript=GRMZM2G098167_T01; parent_gene=GRMZM2G098167	GRMZM2G098167_P01	18 kDa	Ref	0.9	1.4	1.3	0.4	5.2	5.1	5.4	6
788	seq=translation; coord=7:7118087..7119389:-1; parent_transcript=GRMZM2G320298_T01; parent_gene=GRMZM2G320298	GRMZM2G320298_P01	35 kDa	Ref	-1.5	-0.7	-1.2	0.2	0.5	1.6	1.7	0.8
789	seq=translation; coord=6:147724800..147731064:1; parent_transcript=GRMZM2G070199_T01; parent_gene=GRMZM2G070199	GRMZM2G070199_P01	12 kDa	Ref	1.9	2.3	2.2	0.9	0.4	Value Missing	-0.8	-1
790	seq=translation; coord=4:7172367..7173014:-1; parent_transcript=GRMZM2G132162_T01; parent_gene=GRMZM2G132162	GRMZM2G132162_P01	10 kDa	Ref	1.7	1.6	4.3	-0.1	2.3	1.7	1.4	0.6
791	seq=translation; coord=7:152391333..152401606:1; parent_transcript=GRMZM2G325008_T01; parent_gene=GRMZM2G325008	GRMZM2G325008_P01 (+4)	70 kDa	Ref	-0.1	0.6	0	0.2	2.2	1.2	1.5	1.6
792	seq=translation; coord=5:31178435..31182856:-1; parent_transcript=GRMZM2G102639_T01; parent_gene=GRMZM2G102639	GRMZM2G102639_P01 (+1)	39 kDa	Ref	-0.6	-0.5	-0.5	-0.5	0.2	-0.5	-0.4	-0.9

793	seq=translation; coord=3:221273481..221276486:-1; parent_transcript=GRMZM2G051764_T01; parent_gene=GRMZM2G051764	GRMZM2G051764_P01 (+2)	37 kDa	Ref	0.2	0.5	0.5	0.7	-4.6	-4	-3.7	-4.5
794	seq=translation; coord=1:14103040..14104304:1; parent_transcript=GRMZM5G850455_T01; parent_gene=GRMZM5G850455	GRMZM5G850455_P01 (+2)	21 kDa	Ref	1.2	0.1	-0.1	-0.2	-0.9	Value Missing	-4.5	-4.2
795	seq=translation; coord=7:10513154..10517482:1; parent_transcript=GRMZM2G066290_T01; parent_gene=GRMZM2G066290	GRMZM2G066290_P01 (+1)	63 kDa	Ref	No Values	No Values	No Values	No Values	0.5	-0.3	-0.8	-0.9
796	seq=translation; coord=2:36456199..36464613:-1; parent_transcript=GRMZM2G130230_T01; parent_gene=GRMZM2G130230	GRMZM2G130230_P01 (+2)	58 kDa	Ref	-0.7	-1.3	-0.7	-0.5	No Values	No Values	No Values	No Values
797	seq=translation; coord=9:149239142..149244150:1; parent_transcript=GRMZM2G004528_T03; parent_gene=GRMZM2G004528	GRMZM2G004528_P03 (+1)	56 kDa	Ref	No Values	No Values	No Values	No Values	2	0.4	0.2	0.2
798	seq=translation; coord=4:237868446..237932504:1; parent_transcript=GRMZM2G092627_T01; parent_gene=GRMZM2G092627	GRMZM2G092627_P01	155 kDa	Ref	No Values	No Values	No Values	No Values	1.2	1.5	0.6	-0.2
799	seq=translation; coord=5:128105440..128107392:-1; parent_transcript=GRMZM2G071089_T01; parent_gene=GRMZM2G071089	GRMZM2G071089_P01	25 kDa	Ref	3.6	2.6	1.8	0.5	No Values	No Values	No Values	No Values
800	seq=translation; coord=3:52892215..52895069:-1; parent_transcript=GRMZM2G033226_T01; parent_gene=GRMZM2G033226	GRMZM2G033226_P01	20 kDa	Ref	-0.2	-0.3	-0.8	-0.4	No Values	No Values	No Values	No Values
801	seq=translation; coord=2:224904656..224907978:1; parent_transcript=GRMZM2G408768_T01; parent_gene=GRMZM2G408768	GRMZM2G408768_P01	29 kDa	Ref	1.4	0.9	1.1	0.9	No Values	No Values	No Values	No Values
802	seq=translation; coord=1:298431972..298436349:1; parent_transcript=GRMZM2G021742_T01; parent_gene=GRMZM2G021742	GRMZM2G021742_P01	32 kDa	Ref	0.1	0.2	0.1	0.2	-0.7	-1.9	-1.8	-0.9
803	seq=translation; coord=4:100154058..100157650:1; parent_transcript=GRMZM2G091995_T01; parent_gene=GRMZM2G091995	GRMZM2G091995_P01 (+1)	44 kDa	Ref	-0.3	-0.6	-0.5	-1.2	-0.4	-1.3	-0.6	-0.9
804	seq=translation; coord=9:86866692..86874101:-1; parent_transcript=GRMZM2G022275_T01; parent_gene=GRMZM2G022275	GRMZM2G022275_P01 (+1)	53 kDa	Ref	-0.2	-0.4	-0.8	-0.5	No Values	No Values	No Values	No Values
805	seq=translation; coord=8:155644343..155646847:-1; parent_transcript=GRMZM2G151252_T01; parent_gene=GRMZM2G151252	GRMZM2G151252_P01 (+2)	16 kDa	Ref	0.9	0.9	0.1	0.2	-0.2	-1.5	-1.4	-1.6

806	seq=translation; coord=7:108281609..108388418:-1; parent_transcript=GRMZM2G700188_T04; parent_gene=GRMZM2G700188	GRMZM2G700188_P04 (+1)	43 kDa	Ref	0.8	1	0.2	0.5	0.9	Reference Missing	1.7	1.3
807	seq=translation; coord=4:2112375..2118463:-1; parent_transcript=GRMZM2G057450_T01; parent_gene=GRMZM2G057450	GRMZM2G057450_P01 (+6)	60 kDa	Ref	0.5	0.3	0.4	0.3	-2.5	-3	-3.2	-3.3
808	seq=translation; coord=7:166387724..166390583:1; parent_transcript=GRMZM2G054136_T01; parent_gene=GRMZM2G054136	GRMZM2G054136_P01 (+6)	29 kDa	Ref	1	0.8	0.3	0.4	1.7	0.5	0.1	-0.2
809	seq=translation; coord=6:167112832..167116884:-1; parent_transcript=GRMZM2G144372_T01; parent_gene=GRMZM2G144372	GRMZM2G144372_P01	43 kDa	Ref	-0.5	-0.2	-0.7	-0.8	-2.9	-2.7	-2.8	-2.8
810	seq=translation; coord=3:230441023..230443000:1; parent_transcript=GRMZM2G353103_T01; parent_gene=GRMZM2G353103	GRMZM2G353103_P01 (+1)	17 kDa	Ref	0.9	0.4	0.3	0.5	1.6	1.7	0.8	1.5
811	seq=translation; coord=8:5159968..5162055:1; parent_transcript=GRMZM2G044128_T01; parent_gene=GRMZM2G044128	GRMZM2G044128_P01	64 kDa	Ref	No Values	No Values	No Values	No Values	Reference Missing	Value Missing	Value Missing	Value Missing
812	seq=translation; coord=2:235441754..235443878:-1; parent_transcript=GRMZM2G082974_T01; parent_gene=GRMZM2G082974	GRMZM2G082974_P01	17 kDa	Ref	-0.4	-1.7	-2.6	-3	0	-0.5	-0.8	-0.7
813	seq=translation; coord=1:275223498..275227310:-1; parent_transcript=GRMZM2G169671_T01; parent_gene=GRMZM2G169671	GRMZM2G169671_P01	27 kDa	Ref	0.1	-0.2	-0.5	-0.4	-0.6	-1	-0.7	-1
814	seq=translation; coord=5:115978543..115983314:-1; parent_transcript=GRMZM2G452633_T01; parent_gene=GRMZM2G452633	GRMZM2G452633_P01 (+2)	40 kDa	Ref	2.6	2	1.1	0.7	2.4	-0.8	-1.1	-1.3
815	seq=translation; coord=7:16096163..16099019:1; parent_transcript=GRMZM2G170044_T01; parent_gene=GRMZM2G170044	GRMZM2G170044_P01	21 kDa	Ref	-0.3	0.4	0.6	0.3	-1.6	-3	-3.1	-2
816	seq=translation; coord=3:188328660..188331609:-1; parent_transcript=GRMZM2G047274_T01; parent_gene=GRMZM2G047274	GRMZM2G047274_P01	46 kDa	Ref	0.7	0.9	0.9	0.9	4.1	2.3	1.7	0.9
817	seq=translation; coord=10:38522742..38534648:1; parent_transcript=GRMZM2G010406_T01; parent_gene=GRMZM2G010406	GRMZM2G010406_P01 (+2)	53 kDa	Ref	0.1	0.1	-0.4	-0.3	-1.2	-1.5	-1.4	-1.5
818	seq=translation; coord=10:3062645..3066771:-1; parent_transcript=GRMZM2G068465_T01; parent_gene=GRMZM2G068465	GRMZM2G068465_P01 (+3)	56 kDa	Ref	1.6	0.8	1.2	0.6	Reference Missing	Reference Missing	Reference Missing	Reference Missing

819	seq=translation; coord=2:21258086..21262803:1; parent_transcript=GRMZM2G019919_T01; parent_gene=GRMZM2G019919	GRMZM2G019919_P01 (+2)	89 kDa	Ref	0.6	0.1	-0.3	-0.5	0.5	-0.9	-0.8	-1.1
820	seq=translation; coord=6:150509782..150519705:1; parent_transcript=GRMZM2G118037_T01; parent_gene=GRMZM2G118037	GRMZM2G118037_P01	14 kDa	Ref	2	1.4	1.5	0.3	-1.9	-1.1	-1	0.2
821	seq=translation; coord=8:134463276..134464095:1; parent_transcript=GRMZM2G028393_T01; parent_gene=GRMZM2G028393	GRMZM2G028393_P01 (+1)	8 kDa	Ref	0.5	1.1	1.7	1.3	-2.7	-1.4	-0.8	0.1
822	seq=translation; coord=1:292879723..292882219:-1; parent_transcript=AC225147.4_FGT002; parent_gene=AC225147.4_FG002	AC225147.4_FGP002 (+5)	16 kDa	Ref	0.6	0.2	-1.1	-0.8	-1.7	-2.5	-2.8	-4
823	seq=translation; coord=8:15778520..15799328:1; parent_transcript=GRMZM2G097775_T01; parent_gene=GRMZM2G097775	GRMZM2G097775_P01 (+1)	47 kDa	Ref	0	0.1	0.2	0.1	-0.5	Value Missing	-3.9	-3.8
824	seq=translation; coord=8:113008265..113010860:-1; parent_transcript=GRMZM2G044947_T01; parent_gene=GRMZM2G044947	GRMZM2G044947_P01 (+2)	40 kDa	Ref	0.3	0.7	0.3	0.5	-1	-2.1	-2.9	-2.7
825	seq=translation; coord=6:129086004..129087221:1; parent_transcript=GRMZM2G447795_T01; parent_gene=GRMZM2G447795	GRMZM2G447795_P01	35 kDa	Ref	0.4	0.2	0.4	1.2	2.3	2.5	3.7	4.4
826	seq=translation; coord=1:84413344..84417967:1; parent_transcript=GRMZM2G083173_T01; parent_gene=GRMZM2G083173	GRMZM2G083173_P01 (+1)	80 kDa	Ref	1.7	1.4	1	1.8	4.4	2.1	2.3	1.3
827	seq=translation; coord=4:14570220..14574637:-1; parent_transcript=GRMZM5G840928_T01; parent_gene=GRMZM5G840928	GRMZM5G840928_P01 (+2)	44 kDa	Ref	-0.2	-0.1	0.4	0.6	-1.1	-0.2	-1.1	-1
828	seq=translation; coord=6:88903392..88908316:-1; parent_transcript=GRMZM2G150256_T01; parent_gene=GRMZM2G150256	GRMZM2G150256_P01	52 kDa	Ref	0.1	-0.2	-0.1	0.1	No Values	No Values	No Values	No Values
829	seq=translation; coord=2:13403335..13406342:-1; parent_transcript=GRMZM2G354053_T01; parent_gene=GRMZM2G354053	GRMZM2G354053_P01	84 kDa	Ref	1	0.1	-0.5	-1.6	No Values	No Values	No Values	No Values
830	seq=translation; coord=8:171412337..171415029:-1; parent_transcript=GRMZM2G012160_T01; parent_gene=GRMZM2G012160	GRMZM2G012160_P01	15 kDa	Ref	0.8	0.5	3.5	2.7	No Values	No Values	No Values	No Values
831	seq=translation; coord=3:213775068..213778961:1; parent_transcript=GRMZM2G047298_T01; parent_gene=GRMZM2G047298	GRMZM2G047298_P01	42 kDa	Ref	0	-0.2	-1.8	-1.5	No Values	No Values	No Values	No Values

832	seq=translation; coord=2:33498879..33500909:-1; parent_transcript=GRMZM2G051898_T01; parent_gene=GRMZM2G051898	GRMZM2G051898_P01	40 kDa	Ref	0.8	0.8	Reference Missing	2.8	No Values	No Values	No Values	No Values
833	seq=translation; coord=4:182566741..182604691:1; parent_transcript=GRMZM2G439339_T04; parent_gene=GRMZM2G439339	GRMZM2G439339_P04 (+1)	226 kDa	Ref	No Values	No Values	No Values	No Values	0.7	0.6	0.1	-0.2
834	seq=translation; coord=7:173600066..173601522:1; parent_transcript=GRMZM2G348125_T01; parent_gene=GRMZM2G348125	GRMZM2G348125_P01 (+1)	23 kDa	Ref	-0.1	0.3	2	0.3	No Values	No Values	No Values	No Values
835	seq=translation; coord=1:240863375..240866448:1; parent_transcript=GRMZM2G124434_T01; parent_gene=GRMZM2G124434	GRMZM2G124434_P01	43 kDa	Ref	-0.9	-0.6	-0.7	-0.3	0.2	-0.7	-1	-1.2
836	seq=translation; coord=3:38743793..38746868:-1; parent_transcript=GRMZM2G033626_T01; parent_gene=GRMZM2G033626	GRMZM2G033626_P01 (+2)	34 kDa	Ref	0.3	0	-0.1	0	0.8	0.8	1	0.3
837	seq=translation; coord=3:71260323..71320829:1; parent_transcript=GRMZM2G314652_T02; parent_gene=GRMZM2G314652	GRMZM2G314652_P02	55 kDa	Ref	-0.2	0.4	0.3	0.8	-0.5	-0.1	0.4	-0.1
838	seq=translation; coord=1:226551729..226556786:-1; parent_transcript=GRMZM2G131769_T01; parent_gene=GRMZM2G131769	GRMZM2G131769_P01	54 kDa	Ref	-1.3	-1.4	-1.1	-1.6	2	1.7	1.9	1.5
839	seq=translation; coord=2:224332394..224344331:1; parent_transcript=GRMZM2G169095_T01; parent_gene=GRMZM2G169095	GRMZM2G169095_P01	71 kDa	Ref	-0.1	-0.4	-0.9	-0.3	1.8	1.3	1.2	1
840	seq=translation; coord=6:134096678..134101001:-1; parent_transcript=GRMZM2G339091_T01; parent_gene=GRMZM2G339091	GRMZM2G339091_P01	55 kDa	Ref	0.8	0.8	2.7	5.5	-0.6	0.3	1.5	1.5
841	seq=translation; coord=1:22629310..22633408:1; parent_transcript=GRMZM2G055025_T01; parent_gene=GRMZM2G055025	GRMZM2G055025_P01	29 kDa	Ref	0.6	0.4	0.4	0.9	2.6	0.7	1.1	0.4
842	seq=translation; coord=1:5345118..5348832:1; parent_transcript=GRMZM2G119494_T01; parent_gene=GRMZM2G119494	GRMZM2G119494_P01	50 kDa	Ref	-0.7	-0.7	-0.6	-0.4	-3.5	-2.8	-2.3	-2.4
843	seq=translation; coord=5:175584484..175585836:1; parent_transcript=GRMZM2G049675_T01; parent_gene=GRMZM2G049675	GRMZM2G049675_P01 (+1)	35 kDa	Ref	-0.6	-0.2	-0.2	0.8	1.7	2.7	1.3	1.4
844	seq=translation; coord=1:250092343..250095992:-1; parent_transcript=GRMZM2G364069_T01; parent_gene=GRMZM2G364069	GRMZM2G364069_P01	47 kDa	Ref	1	0.5	1	0.7	-1.9	-3.2	-3.3	-3.1

845	seq=translation; coord=1:65994545..65996015:-1; parent_transcript=GRMZM2G004298_T01; parent_gene=GRMZM2G004298	GRMZM2G004298_P01 (+5)	23 kDa	Ref	0.6	-0.3	0.6	1.4	0.3	-1	0.2	0
846	seq=translation; coord=1:274494460..274510748:-1; parent_transcript=GRMZM2G396451_T02; parent_gene=GRMZM2G396451	GRMZM2G396451_P02 (+1)	161 kDa	Ref	0.1	-0.4	-0.5	-0.3	-0.6	-1.9	-2	-2.1
847	seq=translation; coord=7:159001810..159027357:-1; parent_transcript=GRMZM2G081155_T01; parent_gene=GRMZM2G081155	GRMZM2G081155_P01 (+2)	70 kDa	Ref	-0.2	-0.1	-0.4	0	-1.6	-1.8	-2.3	-2.4
848	seq=translation; coord=2:62991591..62998602:-1; parent_transcript=GRMZM2G171688_T01; parent_gene=GRMZM2G171688	GRMZM2G171688_P01	76 kDa	Ref	-0.4	-1.3	-0.6	-0.6	-1.5	0	0.2	0
849	seq=translation; coord=5:13253262..13257697:-1; parent_transcript=GRMZM2G076524_T01; parent_gene=GRMZM2G076524	GRMZM2G076524_P01 (+1)	34 kDa	Ref	0.3	0.5	0.7	-0.7	-0.6	-0.2	-0.2	-0.1
850	seq=translation; coord=8:2052922..2061036:1; parent_transcript=GRMZM2G052875_T01; parent_gene=GRMZM2G052875	GRMZM2G052875_P01 (+1)	69 kDa	Ref	0.2	0	0.4	0.4	0	-0.5	-0.2	0.1
851	seq=translation; coord=1:194657095..194659202:-1; parent_transcript=GRMZM2G083725_T01; parent_gene=GRMZM2G083725	GRMZM2G083725_P01 (+1)	19 kDa	Ref	Reference Missing	0.4	0.1	-0.3	1.6	1.1	1.2	0.6
852	seq=translation; coord=3:154973863..154975444:-1; parent_transcript=GRMZM2G146246_T02; parent_gene=GRMZM2G146246	GRMZM2G146246_P02	24 kDa	Ref	-1.1	-0.8	0	-1.5	-1.7	-1	-1.3	0.4
853	seq=translation; coord=1:174313373..174317518:1; parent_transcript=GRMZM2G088565_T01; parent_gene=GRMZM2G088565	GRMZM2G088565_P01	42 kDa	Ref	0.9	0.5	0.2	0.5	-0.7	-0.9	-1.3	-1.7
854	seq=translation; coord=7:137275571..137279379:1; parent_transcript=GRMZM2G458164_T01; parent_gene=GRMZM2G458164	GRMZM2G458164_P01	52 kDa	Ref	0.6	0	0.1	0.5	4.5	3.4	4.1	5.3
855	seq=translation; coord=1:296874949..296877709:-1; parent_transcript=GRMZM2G035017_T01; parent_gene=GRMZM2G035017	GRMZM2G035017_P01	30 kDa	Ref	1.4	1.9	1.4	1.6	1	1.8	-0.3	-0.2
856	seq=translation; coord=2:160538409..160543692:1; parent_transcript=GRMZM2G151549_T01; parent_gene=GRMZM2G151549	GRMZM2G151549_P01 (+1)	51 kDa	Ref	0.3	-0.2	0.4	-0.4	0	-1	-0.4	-1
857	seq=translation; coord=3:205620317..205627720:1; parent_transcript=GRMZM2G157317_T01; parent_gene=GRMZM2G157317	GRMZM2G157317_P01 (+1)	47 kDa	Ref	0.1	-0.5	-0.3	-0.5	-2.1	-2.5	-2.8	-2.7

858	seq=translation; coord=10:121538895..121542946:-1; parent_transcript=GRMZM2G381267_T01; parent_gene=GRMZM2G381267	GRMZM2G381267_P01 (+1)	33 kDa	Ref	3.1	2.2	1.6	0.8	-0.8	-1.2	-1.8	-2.4
859	seq=translation; coord=7:155559747..155562921:1; parent_transcript=GRMZM2G108285_T01; parent_gene=GRMZM2G108285	GRMZM2G108285_P01	25 kDa	Ref	-0.8	-1	0.3	0.7	-2	-1.1	-1.9	0.3
860	seq=translation; coord=8:13369483..13372340:-1; parent_transcript=GRMZM2G119361_T01; parent_gene=GRMZM2G119361	GRMZM2G119361_P01 (+1)	70 kDa	Ref	-0.3	1	-0.3	0.2	-9	-8.3	-8.7	-8.7
861	seq=translation; coord=1:296017136..296022027:1; parent_transcript=GRMZM2G053458_T01; parent_gene=GRMZM2G053458	GRMZM2G053458_P01 (+1)	16 kDa	Ref	-0.6	-1.6	-1	-1.5	0.5	Value Missing	-0.2	Value Missing
862	seq=translation; coord=4:1508758..1509325:-1; parent_transcript=GRMZM2G107302_T01; parent_gene=GRMZM2G107302	GRMZM2G107302_P01	10 kDa	Ref	1.2	0.5	0.7	-1.1	Reference Missing	-2.7	-1.5	-2.8
863	seq=translation; coord=10:4071331..4073937:-1; parent_transcript=GRMZM2G129804_T01; parent_gene=GRMZM2G129804	GRMZM2G129804_P01	26 kDa	Ref	-1.4	-0.8	-0.7	-1.7	-1.2	-0.6	-0.3	-0.3
864	seq=translation; coord=1:216692560..216696036:1; parent_transcript=GRMZM2G167669_T01; parent_gene=GRMZM2G167669	GRMZM2G167669_P01	55 kDa	Ref	0.6	0.2	-1.3	-0.5	Value Missing	7.4	7.8	Value Missing
865	seq=translation; coord=1:262391932..262394279:1; parent_transcript=GRMZM2G063503_T02; parent_gene=GRMZM2G063503	GRMZM2G063503_P02	10 kDa	Ref	-1.4	-2.3	-0.8	-2.2	0.4	0.8	-0.2	2.3
866	seq=translation; coord=5:62197397..62202236:1; parent_transcript=GRMZM2G152111_T01; parent_gene=GRMZM2G152111	GRMZM2G152111_P01 (+4)	25 kDa	Ref	-0.4	-0.5	-1	-1	1.1	0	-0.8	-1.2
867	seq=translation; coord=8:39126474..39127702:1; parent_transcript=GRMZM2G047732_T01; parent_gene=GRMZM2G047732	GRMZM2G047732_P01 (+1)	18 kDa	Ref	0.5	-0.1	-0.2	-0.4	-2	-2.9	-4.8	-4.7
868	seq=translation; coord=5:1122734..1125202:1; parent_transcript=GRMZM2G034326_T01; parent_gene=GRMZM2G034326	GRMZM2G034326_P01 (+3)	17 kDa	Ref	No Values	No Values	No Values	No Values	-0.1	0.5	0.4	0
869	seq=translation; coord=5:202050924..202059781:1; parent_transcript=GRMZM2G020040_T01; parent_gene=GRMZM2G020040	GRMZM2G020040_P01 (+1)	104 kDa	Ref	-0.2	0	0	-0.5	No Values	No Values	No Values	No Values
870	seq=translation; coord=1:221648603..221653196:1; parent_transcript=GRMZM2G163437_T01; parent_gene=GRMZM2G163437	GRMZM2G163437_P01 (+1)	56 kDa	Ref	No Values	No Values	No Values	No Values	-0.9	2.5	2.9	2.9

871	seq=translation; coord=10:125428223..125432495:1; parent_transcript=GRMZM2G066815_T01; parent_gene=GRMZM2G066815	GRMZM2G066815_P01	57 kDa	Ref	No Values	No Values	No Values	No Values	0.5	0.7	0.5	0.5
872	seq=translation; coord=5:23763272..23765390:-1; parent_transcript=GRMZM2G376743_T01; parent_gene=GRMZM2G376743	GRMZM2G376743_P01	59 kDa	Ref	No Values	No Values	No Values	No Values	-0.4	-0.1	0.3	0.9
873	seq=translation; coord=9:77151999..77153846:1; parent_transcript=GRMZM2G080839_T01; parent_gene=GRMZM2G080839	GRMZM2G080839_P01	58 kDa	Ref	0.6	0.6	1.7	3.3	No Values	No Values	No Values	No Values
874	seq=translation; coord=4:9657544..9658965:-1; parent_transcript=GRMZM2G117989_T01; parent_gene=GRMZM2G117989	GRMZM2G117989_P01 (+5)	18 kDa	Ref	0.1	0	4.1	4.2	No Values	No Values	No Values	No Values
875	seq=translation; coord=5:6444041..6445037:1; parent_transcript=GRMZM2G077034_T01; parent_gene=GRMZM2G077034	GRMZM2G077034_P01	10 kDa	Ref	0.4	0.7	0.8	0.9	No Values	No Values	No Values	No Values
876	seq=translation; coord=5:193174093..193205154:1; parent_transcript=GRMZM5G883741_T02; parent_gene=GRMZM5G883741	GRMZM5G883741_P02	66 kDa	Ref	-0.3	-0.7	-0.6	-0.3	No Values	No Values	No Values	No Values
877	seq=translation; coord=1:27178033..27184068:-1; parent_transcript=GRMZM5G891739_T01; parent_gene=GRMZM5G891739	GRMZM5G891739_P01	17 kDa	Ref	-0.5	-0.8	-0.5	-0.7	-2	-1.4	-0.7	-1
878	seq=translation; coord=9:11741082..11743899:1; parent_transcript=GRMZM2G177098_T03; parent_gene=GRMZM2G177098	GRMZM2G177098_P03	13 kDa	Ref	0.5	0.1	-0.3	0	2	1.6	1.2	0.8
879	seq=translation; coord=8:126595171..126599674:1; parent_transcript=GRMZM2G389118_T01; parent_gene=GRMZM2G389118	GRMZM2G389118_P01	143 kDa	Ref	0.1	-0.4	0.2	-0.5	-3.6	-4	-4.1	-4.3
880	seq=translation; coord=2:164667085..164668830:-1; parent_transcript=AC210204.3_FGT002; parent_gene=AC210204.3_FG002	AC210204.3_FGP002	34 kDa	Ref	0.5	0.2	1.4	1	1.3	2	2.3	2.1
881	seq=translation; coord=5:171688070..171693270:-1; parent_transcript=GRMZM2G000278_T01; parent_gene=GRMZM2G000278	GRMZM2G000278_P01 (+4)	42 kDa	Ref	0.7	0.5	-0.1	0.2	2.4	1.7	1.6	1.5
882	seq=translation; coord=10:5718777..5728532:-1; parent_transcript=GRMZM2G020446_T01; parent_gene=GRMZM2G020446	GRMZM2G020446_P01	53 kDa	Ref	-0.5	-0.8	-0.9	-0.2	1.4	0.3	1.6	-0.5
883	seq=translation; coord=1:67436204..67440532:-1; parent_transcript=GRMZM2G107473_T01; parent_gene=GRMZM2G107473	GRMZM2G107473_P01	30 kDa	Ref	1.1	1.6	0.9	Reference Missing	0.5	0	-0.2	-0.8

884	seq=translation; coord=7:157907062..157937456:-1; parent_transcript=GRMZM2G335287_T01; parent_gene=GRMZM2G335287	GRMZM2G335287_P01	191 kDa	Ref	0.5	0.4	-0.3	0.2	2.2	-0.1	-0.3	-0.8
885	seq=translation; coord=1:300990204..300994789:1; parent_transcript=GRMZM2G122267_T01; parent_gene=GRMZM2G122267	GRMZM2G122267_P01 (+1)	63 kDa	Ref	-0.5	-0.6	-0.4	-0.2	-1.6	-1.8	-1.6	-1.3
886	seq=translation; coord=4:162075480..162081835:-1; parent_transcript=GRMZM2G042502_T01; parent_gene=GRMZM2G042502	GRMZM2G042502_P01 (+2)	67 kDa	Ref	0.6	-0.3	-0.9	-0.3	4.1	3.4	3.4	3.7
887	seq=translation; coord=3:158520150..158525636:-1; parent_transcript=GRMZM2G081102_T01; parent_gene=GRMZM2G081102	GRMZM2G081102_P01 (+6)	24 kDa	Ref	1.1	1.2	0.1	-0.1	0.4	-0.4	-0.6	-1.4
888	seq=translation; coord=5:4422815..4424855:1; parent_transcript=GRMZM2G130544_T02; parent_gene=GRMZM2G130544	GRMZM2G130544_P02	18 kDa	Ref	0.4	0.6	0.4	0.2	No Values	No Values	No Values	No Values
889	seq=translation; coord=10:17606896..17610628:1; parent_transcript=GRMZM2G009448_T01; parent_gene=GRMZM2G009448	GRMZM2G009448_P01	26 kDa	Ref	-0.6	-1.3	-1.5	-1.8	5.8	3.6	4.2	3.3
890	seq=translation; coord=1:213095235..213096735:1; parent_transcript=GRMZM2G161274_T02; parent_gene=GRMZM2G161274	GRMZM2G161274_P02	25 kDa	Ref	1.1	1.2	2.6	4.5	-0.6	-0.8	-0.1	3.1
891	seq=translation; coord=10:128106169..128112118:-1; parent_transcript=GRMZM2G098577_T01; parent_gene=GRMZM2G098577	GRMZM2G098577_P01	87 kDa	Ref	1.4	1.4	1.3	0.7	0.3	-0.1	-0.5	0
892	seq=translation; coord=1:289108489..289111192:-1; parent_transcript=GRMZM2G053023_T01; parent_gene=GRMZM2G053023	GRMZM2G053023_P01 (+1)	15 kDa	Ref	-1.6	-1.8	-1.4	-1.6	-1.8	-1.8	-1.9	-0.1
893	seq=translation; coord=9:1634017..1638812:-1; parent_transcript=GRMZM2G143862_T01; parent_gene=GRMZM2G143862	GRMZM2G143862_P01	32 kDa	Ref	-0.1	-0.2	-0.8	0.2	-4.4	-4.8	-5.1	-5
894	seq=translation; coord=9:126016122..126022567:1; parent_transcript=GRMZM2G170843_T01; parent_gene=GRMZM2G170843	GRMZM2G170843_P01 (+1)	51 kDa	Ref	-0.1	-0.3	-0.2	-0.9	-1.8	-1.2	-0.6	-0.2
895	seq=translation; coord=5:70685080..70691262:-1; parent_transcript=GRMZM2G120578_T01; parent_gene=GRMZM2G120578	GRMZM2G120578_P01 (+1)	42 kDa	Ref	-0.1	-0.2	-0.4	-0.4	1.4	0	0.5	0
896	seq=translation; coord=1:190997916..191004927:1; parent_transcript=GRMZM2G084149_T01; parent_gene=GRMZM2G084149	GRMZM2G084149_P01	51 kDa	Ref	1.5	1.6	1.9	1.3	6.3	5.5	5.7	5.3

897	seq=translation; coord=5:83557023..83560095:1; parent_transcript=AC234515.1_FGT003; parent_gene=AC234515.1_FG003	AC234515.1_FGP003	50 kDa	Ref	0.1	-0.6	-1.6	-1.6	-2	Value Missing	Value Missing	Value Missing
898	seq=translation; coord=8:79074521..79075661:-1; parent_transcript=GRMZM2G125893_T01; parent_gene=GRMZM2G125893	GRMZM2G125893_P01	15 kDa	Ref	-0.2	0.2	0.1	-0.8	0.6	1.1	1.5	1.8
899	seq=translation; coord=2:19443026..19444255:1; parent_transcript=GRMZM2G132777_T01; parent_gene=GRMZM2G132777	GRMZM2G132777_P01	35 kDa	Ref	-1	-1.7	-1.3	-1.6	-3.3	-3.2	-3.1	-3.3
900	seq=translation; coord=3:224922109..224926422:1; parent_transcript=GRMZM2G064753_T01; parent_gene=GRMZM2G064753	GRMZM2G064753_P01	18 kDa	Ref	1.2	0.4	0.5	0.1	No Values	No Values	No Values	No Values
901	seq=translation; coord=2:184451593..184456789:1; parent_transcript=GRMZM5G820822_T01; parent_gene=GRMZM5G820822	GRMZM5G820822_P01	27 kDa	Ref	No Values	No Values	No Values	No Values	No Values	No Values	No Values	No Values
902	seq=translation; coord=7:104173693..104176328:-1; parent_transcript=GRMZM2G032160_T01; parent_gene=GRMZM2G032160	GRMZM2G032160_P01	82 kDa	Ref	0.1	0	0.6	1.3	No Values	No Values	No Values	No Values
903	seq=translation; coord=2:6820716..6853179:1; parent_transcript=GRMZM2G138782_T01; parent_gene=GRMZM2G138782	GRMZM2G138782_P01	67 kDa	Ref	1.6	1.3	1.2	1.3	No Values	No Values	No Values	No Values
904	seq=translation; coord=2:24834408..24836108:-1; parent_transcript=GRMZM2G108153_T01; parent_gene=GRMZM2G108153	GRMZM2G108153_P01	35 kDa	Ref	1.6	0.7	Reference Missing	2.4	No Values	No Values	No Values	No Values
905	seq=translation; coord=8:125902255..125906710:1; parent_transcript=GRMZM2G059693_T01; parent_gene=GRMZM2G059693	GRMZM2G059693_P01 (+2)	58 kDa	Ref	1.6	1.1	-0.4	-0.3	No Values	No Values	No Values	No Values
906	seq=translation; coord=7:151190045..151192101:1; parent_transcript=GRMZM2G155911_T01; parent_gene=GRMZM2G155911	GRMZM2G155911_P01	52 kDa	Ref	-0.8	0	0.1	0.3	No Values	No Values	No Values	No Values
907	seq=translation; coord=7:129358205..129362749:-1; parent_transcript=GRMZM5G843748_T02; parent_gene=GRMZM5G843748	GRMZM5G843748_P02	38 kDa	Ref	-0.1	-0.2	0.4	0.8	No Values	No Values	No Values	No Values
908	seq=translation; coord=5:77916981..77922073:1; parent_transcript=GRMZM2G097207_T01; parent_gene=GRMZM2G097207	GRMZM2G097207_P01	53 kDa	Ref	No Values	No Values	No Values	No Values	0.4	-0.5	0.3	0.7
909	seq=translation; coord=10:86984445..86986672:1; parent_transcript=GRMZM2G028104_T01; parent_gene=GRMZM2G028104	GRMZM2G028104_P01	47 kDa	Ref	0.5	1.5	1.4	2.7	No Values	No Values	No Values	No Values

910	seq=translation; coord=7:137518004..137519265:-1; parent_transcript=GRMZM2G066202_T01; parent_gene=GRMZM2G066202	GRMZM2G066202_P01	27 kDa	Ref	1.3	0.6	1.1	0.2	No Values	No Values	No Values	No Values
911	seq=translation; coord=9:41048627..41058528:-1; parent_transcript=GRMZM2G538535_T01; parent_gene=GRMZM2G538535	GRMZM2G538535_P01	49 kDa	Ref	0.5	0.2	1.2	1.8	No Values	No Values	No Values	No Values
912	seq=translation; coord=1:232041908..232053896:-1; parent_transcript=GRMZM2G148323_T01; parent_gene=GRMZM2G148323	GRMZM2G148323_P01	62 kDa	Ref	No Values	No Values	No Values	No Values	0.1	0.4	0.1	-0.1
913	seq=translation; coord=2:2338643..2346346:-1; parent_transcript=GRMZM2G077744_T01; parent_gene=GRMZM2G077744	GRMZM2G077744_P01 (+3)	16 kDa	Ref	1.1	1.4	1.4	0.8	0.7	-0.9	-0.8	-1.3
914	seq=translation; coord=2:196218998..196222283:1; parent_transcript=GRMZM2G042604_T01; parent_gene=GRMZM2G042604	GRMZM2G042604_P01 (+3)	27 kDa	Ref	0.1	0	0.4	0	-1.3	-0.4	-0.4	0.1
915	seq=translation; coord=8:18214060..18227316:1; parent_transcript=GRMZM2G107696_T01; parent_gene=GRMZM2G107696	GRMZM2G107696_P01 (+1)	93 kDa	Ref	-0.3	0.1	-0.1	-0.4	-1.7	-1.7	-1.3	-1.4
916	seq=translation; coord=9:115668526..115674221:1; parent_transcript=GRMZM5G818887_T01; parent_gene=GRMZM5G818887	GRMZM5G818887_P01	35 kDa	Ref	0.1	0.2	0	0.3	-0.1	0.7	0.7	0.4
917	seq=translation; coord=1:248590498..248595499:-1; parent_transcript=GRMZM5G830403_T02; parent_gene=GRMZM5G830403	GRMZM5G830403_P02 (+2)	36 kDa	Ref	0.5	-0.9	-0.6	-1.3	0	-1.1	Value Missing	Value Missing
918	seq=translation; coord=7:155961862..155967544:-1; parent_transcript=GRMZM2G173910_T01; parent_gene=GRMZM2G173910	GRMZM2G173910_P01	32 kDa	Ref	0.1	-0.1	-0.4	-0.6	-0.3	-1	-1.1	-1.3
919	seq=translation; coord=5:15703665..15718967:1; parent_transcript=AC197122.3_FGT003; parent_gene=AC197122.3_FG003	AC197122.3_FGP003	65 kDa	Ref	0	-0.3	-0.4	-0.5	-1.4	-2.1	-2.9	-2.4
920	seq=translation; coord=6:117850410..117855773:-1; parent_transcript=GRMZM2G074454_T01; parent_gene=GRMZM2G074454	GRMZM2G074454_P01	57 kDa	Ref	-0.3	-0.6	-1.2	-0.6	0.9	1.2	1.1	0.3
921	seq=translation; coord=8:83396432..83400437:1; parent_transcript=GRMZM2G131249_T02; parent_gene=GRMZM2G131249	GRMZM2G131249_P02	29 kDa	Ref	-0.5	-0.7	-0.6	-0.6	0.5	-0.6	-0.2	0.1
922	seq=translation; coord=6:167006647..167019258:1; parent_transcript=AC233870.1_FGT006; parent_gene=AC233870.1_FG006	AC233870.1_FGP006	23 kDa	Ref	0	-0.3	0.2	-0.6	2.2	1.1	1.3	0.9

923	seq=translation; coord=8:3347680..3350552:1; parent_transcript=GRMZM2G013461_T01; parent_gene=GRMZM2G013461	GRMZM2G013461_P01 (+1)	27 kDa	Ref	0.6	0.2	0.9	-0.2	2	3.1	3.7	3.4
924	seq=translation; coord=9:134504096..134514418:-1; parent_transcript=GRMZM2G051613_T01; parent_gene=GRMZM2G051613	GRMZM2G051613_P01 (+1)	19 kDa	Ref	0	-0.3	-0.1	-0.9	-1.1	-2.2	-2.5	-2.4
925	seq=translation; coord=2:232093952..232099171:1; parent_transcript=GRMZM2G035807_T01; parent_gene=GRMZM2G035807	GRMZM2G035807_P01 (+3)	68 kDa	Ref	1.1	0.7	0.3	1	2.4	0.6	0.4	-0.2
926	seq=translation; coord=1:66948065..66952666:-1; parent_transcript=GRMZM2G152925_T01; parent_gene=GRMZM2G152925	GRMZM2G152925_P01	19 kDa	Ref	2.5	2.2	2.6	2	No Values	No Values	No Values	No Values
927	seq=translation; coord=8:143277512..143279395:-1; parent_transcript=GRMZM2G050072_T01; parent_gene=GRMZM2G050072	GRMZM2G050072_P01	45 kDa	Ref	-0.1	0.2	-0.1	0.1	0.8	0.9	1	1.2
928	seq=translation; coord=10:25975417..25980229:-1; parent_transcript=GRMZM2G014376_T01; parent_gene=GRMZM2G014376	GRMZM2G014376_P01 (+2)	58 kDa	Ref	0	0	-0.5	-0.2	3.9	4.8	5	4.3
929	seq=translation; coord=1:264743594..264751569:1; parent_transcript=GRMZM2G002687_T01; parent_gene=GRMZM2G002687	GRMZM2G002687_P01	46 kDa	Ref	-1.5	-1.7	-1.3	-1.7	-0.6	-0.3	0.3	0.4
930	seq=translation; coord=4:170517685..170521446:-1; parent_transcript=GRMZM2G150648_T03; parent_gene=GRMZM2G150648	GRMZM2G150648_P03 (+2)	32 kDa	Ref	1.2	0.3	0.1	0.4	Reference Missing	0.2	-0.1	Value Missing
931	seq=translation; coord=4:174901704..174907176:-1; parent_transcript=GRMZM2G039263_T01; parent_gene=GRMZM2G039263	GRMZM2G039263_P01 (+2)	59 kDa	Ref	-1.1	Value Missing	Value Missing	Value Missing	-5	-5.5	Value Missing	-6.2
932	seq=translation; coord=1:226171571..226174821:1; parent_transcript=GRMZM5G882228_T02; parent_gene=GRMZM5G882228	GRMZM5G882228_P02	23 kDa	Ref	0.2	0.2	0.5	0.4	-4.1	-5.7	-5.7	-5.8
933	seq=translation; coord=3:19453334..19462209:1; parent_transcript=GRMZM2G088834_T03; parent_gene=GRMZM2G088834	GRMZM2G088834_P03 (+1)	48 kDa	Ref	1.6	1.9	1.7	1.5	1.3	1.3	1.1	0.4
934	seq=translation; coord=2:170263508..170267458:-1; parent_transcript=GRMZM2G074687_T01; parent_gene=GRMZM2G074687	GRMZM2G074687_P01	48 kDa	Ref	1.2	1.2	0.9	0.5	0.4	-1	-0.6	-0.8
935	seq=translation; coord=1:94486908..94489534:1; parent_transcript=GRMZM2G052666_T01; parent_gene=GRMZM2G052666	GRMZM2G052666_P01	28 kDa	Ref	0.1	0	0.7	0.1	1	1.8	2.2	2.1

936	seq=translation; coord=3:47497754..47499644:-1; parent_transcript=GRMZM2G123407_T01; parent_gene=GRMZM2G123407	GRMZM2G123407_P01	24 kDa	Ref	-1.2	-1.2	-1.6	-2.6	-1.4	-1.2	-1	-1.7
937	seq=translation; coord=1:277739114..277741150:1; parent_transcript=GRMZM2G090422_T01; parent_gene=GRMZM2G090422	GRMZM2G090422_P01	24 kDa	Ref	0.6	0.2	0.1	0.1	3	2.1	2.3	1.7
938	seq=translation; coord=4:229500502..229510053:1; parent_transcript=GRMZM2G069631_T01; parent_gene=GRMZM2G069631	GRMZM2G069631_P01 (+5)	58 kDa	Ref	0.1	-0.1	-0.1	-0.1	0.1	-0.4	-0.5	-0.5
939	seq=translation; coord=2:30905783..30911404:-1; parent_transcript=GRMZM2G352129_T01; parent_gene=GRMZM2G352129	GRMZM2G352129_P01	72 kDa	Ref	No Values	No Values	No Values	No Values	0.6	0.5	0.7	0.4
940	seq=translation; coord=1:84814341..84815071:1; parent_transcript=GRMZM2G402564_T01; parent_gene=GRMZM2G402564	GRMZM2G402564_P01	8 kDa	Ref	No Values	No Values	No Values	No Values	No Values	Reference Missing	Reference Missing	Reference Missing
941	seq=translation; coord=2:53844493..53846005:1; parent_transcript=GRMZM2G044132_T01; parent_gene=GRMZM2G044132	GRMZM2G044132_P01	28 kDa	Ref	0.9	1.8	2.7	3.7	No Values	No Values	No Values	No Values
942	seq=translation; coord=5:183144788..183149469:1; parent_transcript=GRMZM2G007647_T01; parent_gene=GRMZM2G007647	GRMZM2G007647_P01	52 kDa	Ref	No Values	No Values	No Values	No Values	1.9	1.5	0.7	0.3
943	seq=translation; coord=5:6445850..6453287:-1; parent_transcript=GRMZM2G076885_T01; parent_gene=GRMZM2G076885	GRMZM2G076885_P01	41 kDa	Ref	No Values	No Values	No Values	No Values	0.6	0.5	0.1	-0.7
944	seq=translation; coord=8:144270470..144273473:-1; parent_transcript=GRMZM2G024315_T01; parent_gene=GRMZM2G024315	GRMZM2G024315_P01	38 kDa	Ref	0.1	0.1	0.9	1.2	No Values	No Values	No Values	No Values
945	seq=translation; coord=3:30996380..30997528:1; parent_transcript=AC226235.2_FGT001; parent_gene=AC226235.2_FG001	AC226235.2_FGP001	33 kDa	Ref	-1.8	-1.9	-1.1	-1.9	No Values	No Values	No Values	No Values
946	seq=translation; coord=5:65417067..65419686:1; parent_transcript=GRMZM2G112805_T01; parent_gene=GRMZM2G112805	GRMZM2G112805_P01	30 kDa	Ref	Reference Missing	2	Reference Missing	Reference Missing	No Values	No Values	No Values	No Values
947	seq=translation; coord=2:34740703..34741540:1; parent_transcript=AC155352.2_FGT010; parent_gene=AC155352.2_FG010	AC155352.2_FGP010 (+2)	14 kDa	Ref	1.1	1.3	1	0.5	No Values	No Values	No Values	No Values
948	seq=translation; coord=10:4776409..4777145:-1; parent_transcript=GRMZM2G031033_T01; parent_gene=GRMZM2G031033	GRMZM2G031033_P01	16 kDa	Ref	No Values	No Values	No Values	No Values	0.3	0.2	0.6	-0.1

949	seq=translation; coord=6:14560251..14563314:-1; parent_transcript=GRMZM2G076943_T01; parent_gene=GRMZM2G076943	GRMZM2G076943_P01	76 kDa	Ref	1.4	2.2	1	1.4	No Values	No Values	No Values	No Values
950	seq=translation; coord=4:1482217..1485577:-1; parent_transcript=GRMZM2G107082_T01; parent_gene=GRMZM2G107082	GRMZM2G107082_P01	47 kDa	Ref	0.1	0.1	-0.1	0.1	No Values	No Values	No Values	No Values
951	seq=translation; coord=5:181383017..181386545:1; parent_transcript=GRMZM2G029583_T02; parent_gene=GRMZM2G029583	GRMZM2G029583_P02 (+1)	44 kDa	Ref	0.8	0.1	0.2	0.7	0.3	-2.5	-2.3	-1.3
952	seq=translation; coord=1:297509820..297513009:-1; parent_transcript=GRMZM5G898915_T01; parent_gene=GRMZM5G898915	GRMZM5G898915_P01	37 kDa	Ref	-0.6	-0.6	-0.9	-0.8	0.7	0.8	0.7	0.8
953	seq=translation; coord=4:26128851..26131595:1; parent_transcript=GRMZM2G024482_T02; parent_gene=GRMZM2G024482	GRMZM2G024482_P02	24 kDa	Ref	-0.5	-0.3	-0.7	-0.6	2.1	2.3	2.6	2.8
954	seq=translation; coord=2:82246568..82258715:1; parent_transcript=GRMZM2G125310_T01; parent_gene=GRMZM2G125310	GRMZM2G125310_P01 (+1)	25 kDa	Ref	-0.2	0.1	-0.3	-0.5	-1.7	-1.1	-0.7	-0.4
955	seq=translation; coord=10:70222469..70228198:-1; parent_transcript=GRMZM2G103287_T01; parent_gene=GRMZM2G103287	GRMZM2G103287_P01 (+1)	37 kDa	Ref	0.1	0	-0.3	-0.1	-0.9	-0.8	-0.6	-0.2
956	seq=translation; coord=7:8520216..8522520:-1; parent_transcript=GRMZM2G024354_T01; parent_gene=GRMZM2G024354	GRMZM2G024354_P01	24 kDa	Ref	1	-0.1	-1.7	-1.2	0	-1.2	-2.2	-3.2
957	seq=translation; coord=1:225492875..225495179:1; parent_transcript=GRMZM2G056431_T01; parent_gene=GRMZM2G056431	GRMZM2G056431_P01 (+1)	13 kDa	Ref	1.8	1.7	1.9	1.4	4.2	4.3	4.6	4.7
958	seq=translation; coord=10:69423694..69433754:-1; parent_transcript=GRMZM2G145870_T01; parent_gene=GRMZM2G145870	GRMZM2G145870_P01 (+1)	42 kDa	Ref	-0.8	-0.8	-0.5	-0.2	-1.6	-1.7	-1.6	-1.1
959	seq=translation; coord=4:5375612..5380694:1; parent_transcript=GRMZM2G154169_T01; parent_gene=GRMZM2G154169	GRMZM2G154169_P01	23 kDa	Ref	0.9	0.9	1	0.8	0.7	Reference Missing	0.2	0.2
960	seq=translation; coord=9:110247484..110252966:-1; parent_transcript=GRMZM2G174444_T01; parent_gene=GRMZM2G174444	GRMZM2G174444_P01 (+1)	12 kDa	Ref	-0.8	-0.5	0.1	-0.3	0.7	0.1	0.5	1
961	seq=translation; coord=5:29321627..29325204:1; parent_transcript=GRMZM2G158277_T01; parent_gene=GRMZM2G158277	GRMZM2G158277_P01 (+2)	30 kDa	Ref	0.6	0.4	0.7	0	0.7	-0.1	-0.3	-0.5

962	seq=translation; coord=8:21874081..21876830:1; parent_transcript=GRMZM2G033283_T01; parent_gene=GRMZM2G033283	GRMZM2G033283_P01	21 kDa	Ref	0	-0.1	-0.4	-0.1	-1.1	-1.2	-1.2	-0.7
963	seq=translation; coord=6:158946942..158950117:1; parent_transcript=GRMZM2G044368_T01; parent_gene=GRMZM2G044368	GRMZM2G044368_P01 (+3)	23 kDa	Ref	-0.3	-0.4	-0.2	-0.2	0.7	0	-0.2	-0.4
964	seq=translation; coord=2:46344346..46345626:-1; parent_transcript=AC206968.3_FGT003; parent_gene=AC206968.3_FG003	AC206968.3_FGP003	48 kDa	Ref	-1.1	-1	-0.8	-0.9	-2.8	-3.4	-3.4	-3.2
965	seq=translation; coord=6:130963745..130976059:-1; parent_transcript=GRMZM2G361605_T01; parent_gene=GRMZM2G361605	GRMZM2G361605_P01 (+1)	94 kDa	Ref	-0.4	-0.6	-0.8	-0.8	No Values	No Values	No Values	No Values
966	seq=translation; coord=5:160443383..160458679:1; parent_transcript=GRMZM2G052435_T03; parent_gene=GRMZM2G052435	GRMZM2G052435_P03	119 kDa	Ref	No Values	No Values	No Values	No Values	0.8	-0.3	-0.5	-0.1
967	seq=translation; coord=7:170026036..170029198:1; parent_transcript=GRMZM2G055527_T01; parent_gene=GRMZM2G055527	GRMZM2G055527_P01	11 kDa	Ref	-0.6	-1	-0.9	-2.1	2.6	3.1	3	3
968	seq=translation; coord=4:45262758..45263990:-1; parent_transcript=GRMZM2G071575_T01; parent_gene=GRMZM2G071575	GRMZM2G071575_P01	19 kDa	Ref	1.2	0.9	0.6	0.4	-0.6	-0.9	-0.8	-1.4
969	seq=translation; coord=3:57145425..57150481:1; parent_transcript=GRMZM2G033644_T01; parent_gene=GRMZM2G033644	GRMZM2G033644_P01	57 kDa	Ref	-1.2	-0.8	-0.9	-0.5	Value Missing	0.7	1	-0.1
970	seq=translation; coord=10:79271116..79273839:-1; parent_transcript=GRMZM2G032564_T01; parent_gene=GRMZM2G032564	GRMZM2G032564_P01 (+5)	14 kDa	Ref	0.8	0.1	-0.9	-0.5	0	-1.2	-1.6	-2.5
971	seq=translation; coord=2:217631963..217637147:1; parent_transcript=GRMZM2G109425_T01; parent_gene=GRMZM2G109425	GRMZM2G109425_P01 (+1)	59 kDa	Ref	-1	-0.6	-0.6	0.3	-3.2	-3.2	-3.4	-3.8
972	seq=translation; coord=8:122751582..122753805:1; parent_transcript=GRMZM2G154936_T01; parent_gene=GRMZM2G154936	GRMZM2G154936_P01 (+2)	18 kDa	Ref	2.1	1.6	1.3	1.1	1	-3.8	-3.4	-3.8
973	seq=translation; coord=10:35583589..35590383:1; parent_transcript=GRMZM2G009443_T01; parent_gene=GRMZM2G009443	GRMZM2G009443_P01 (+1)	102 kDa	Ref	0.8	Value Missing	0.2	0.6	Reference Missing	Reference Missing	Reference Missing	Reference Missing
974	seq=translation; coord=8:1370962..1373972:-1; parent_transcript=GRMZM2G042636_T01; parent_gene=GRMZM2G042636	GRMZM2G042636_P01 (+6)	50 kDa	Ref	1.4	0.2	-1.1	-1.4	1.6	-0.7	-0.9	-1.9

975	seq=translation; coord=4:93237748..93253880:1; parent_transcript=GRMZM2G451443_T01; parent_gene=GRMZM2G451443	GRMZM2G451443_P01	63 kDa	Ref	0.3	0.1	-0.1	-0.3	Reference Missing	-0.5	-0.8	-1
976	seq=translation; coord=5:24616889..24618887:-1; parent_transcript=GRMZM2G032910_T01; parent_gene=GRMZM2G032910	GRMZM2G032910_P01 (+1)	35 kDa	Ref	-0.5	-0.3	-0.2	0.8	2.6	2.6	3	3.1
977	seq=translation; coord=1:12987102..12993471:1; parent_transcript=GRMZM2G172322_T01; parent_gene=GRMZM2G172322	GRMZM2G172322_P01 (+1)	59 kDa	Ref	-0.1	-0.2	0.2	0.3	1.5	1.4	1.9	2.2
978	seq=translation; coord=4:54600217..54609072:-1; parent_transcript=GRMZM2G098496_T01; parent_gene=GRMZM2G098496	GRMZM2G098496_P01 (+1)	32 kDa	Ref	0.8	0.8	1.5	0.4	-1	-0.7	-1.1	1.7
979	seq=translation; coord=3:148834092..148846161:-1; parent_transcript=GRMZM2G047310_T02; parent_gene=GRMZM2G047310	GRMZM2G047310_P02	108 kDa	Ref	-0.6	-0.7	-0.8	-0.6	2.3	2	2.5	1.7
980	seq=translation; coord=5:155530969..155538363:-1; parent_transcript=GRMZM2G015132_T01; parent_gene=GRMZM2G015132	GRMZM2G015132_P01	58 kDa	Ref	-0.2	-0.4	-0.4	0.3	-0.1	-1	-1.2	-0.8
981	seq=translation; coord=8:98594898..98599276:-1; parent_transcript=GRMZM2G114954_T01; parent_gene=GRMZM2G114954	GRMZM2G114954_P01 (+3)	12 kDa	Ref	1	-0.9	1.2	0.2	-2.9	-2.9	-3.7	-3.1
982	seq=translation; coord=8:9919345..9920090:-1; parent_transcript=AC204893.3_FGT001; parent_gene=AC204893.3_FG001	AC204893.3_FGP001 (+1)	13 kDa	Ref	-1.6	-1.7	-2.6	-3.5	-4	-5.6	-5.8	-5.8
983	seq=translation; coord=8:159703050..159707801:1; parent_transcript=GRMZM2G122607_T01; parent_gene=GRMZM2G122607	GRMZM2G122607_P01	48 kDa	Ref	Reference Missing	0.6	Reference Missing	0.4	2.7	3.1	2.9	2.2
984	seq=translation; coord=4:161027635..161031592:-1; parent_transcript=GRMZM2G156068_T01; parent_gene=GRMZM2G156068	GRMZM2G156068_P01 (+1)	26 kDa	Ref	-0.8	-0.3	0.1	0	0.8	1.3	1.2	0.8
985	seq=translation; coord=3:88528132..88533870:-1; parent_transcript=GRMZM2G476933_T01; parent_gene=GRMZM2G476933	GRMZM2G476933_P01	29 kDa	Ref	0.5	-0.3	-0.2	-0.6	0.8	0	-0.2	-0.7
986	seq=translation; coord=1:56223520..56226018:1; parent_transcript=GRMZM2G062425_T01; parent_gene=GRMZM2G062425	GRMZM2G062425_P01 (+1)	28 kDa	Ref	No Values	No Values	No Values	No Values	-1.3	-0.4	-1.1	-1.2
987	seq=translation; coord=2:207265904..207268967:1; parent_transcript=AC194970.5_FGT009; parent_gene=AC194970.5_FG009	AC194970.5_FGP009	52 kDa	Ref	1.7	0.9	1.4	0.1	-1.6	-1.8	-2.1	-2.5

988	seq=translation; coord=3:224163656..224168718:-1; parent_transcript=GRMZM2G339540_T04; parent_gene=GRMZM2G339540	GRMZM2G339540_P04 (+1)	70 kDa	Ref	0.3	0.8	0.4	0.9	No Values	No Values	No Values	No Values
989	seq=translation; coord=7:154714410..154723867:1; parent_transcript=GRMZM2G375593_T01; parent_gene=GRMZM2G375593	GRMZM2G375593_P01	104 kDa	Ref	No Values	No Values	No Values	No Values	1	1.1	0.5	0.5
990	seq=translation; coord=7:7507005..7510800:-1; parent_transcript=GRMZM2G143330_T01; parent_gene=GRMZM2G143330	GRMZM2G143330_P01	36 kDa	Ref	No Values	No Values	No Values	No Values	0.9	1	0.8	0.3
991	seq=translation; coord=7:124863398..124868735:1; parent_transcript=GRMZM2G067265_T01; parent_gene=GRMZM2G067265	GRMZM2G067265_P01 (+1)	54 kDa	Ref	No Values	No Values	No Values	No Values	0.2	Reference Missing	0.6	1
992	seq=translation; coord=1:289786005..289788499:1; parent_transcript=GRMZM2G168833_T01; parent_gene=GRMZM2G168833	GRMZM2G168833_P01	21 kDa	Ref	-0.1	0.7	1.3	2.5	No Values	No Values	No Values	No Values
993	seq=translation; coord=5:159290030..159322686:-1; parent_transcript=GRMZM2G386155_T01; parent_gene=GRMZM2G386155	GRMZM2G386155_P01 (+1)	54 kDa	Ref	No Values	No Values	No Values	No Values	1.6	1.6	1.8	1.8
994	seq=translation; coord=6:103857605..103858686:-1; parent_transcript=GRMZM2G335618_T01; parent_gene=GRMZM2G335618	GRMZM2G335618_P01	26 kDa	Ref	-0.7	-0.7	-0.3	0.7	No Values	No Values	No Values	No Values
995	seq=translation; coord=2:42784980..42792494:1; parent_transcript=GRMZM2G065073_T02; parent_gene=GRMZM2G065073	GRMZM2G065073_P02	61 kDa	Ref	0.4	0.7	0.9	1.3	No Values	No Values	No Values	No Values
996	seq=translation; coord=8:607069..619737:1; parent_transcript=GRMZM2G077233_T01; parent_gene=GRMZM2G077233	GRMZM2G077233_P01 (+4)	55 kDa	Ref	0.5	0	0.9	3	No Values	No Values	No Values	No Values
997	seq=translation; coord=3:184434264..184435351:1; parent_transcript=GRMZM2G126397_T01; parent_gene=GRMZM2G126397	GRMZM2G126397_P01	13 kDa	Ref	3.1	0.4	0.3	0.1	No Values	No Values	No Values	No Values
998	seq=translation; coord=5:168890072..168891220:-1; parent_transcript=GRMZM2G115491_T01; parent_gene=GRMZM2G115491	GRMZM2G115491_P01	25 kDa	Ref	0.2	0.8	0.7	1.1	No Values	No Values	No Values	No Values
999	seq=translation; coord=8:5023676..5024789:1; parent_transcript=GRMZM2G095164_T01; parent_gene=GRMZM2G095164	GRMZM2G095164_P01 (+1)	13 kDa	Ref	5	4.1	4.5	3.4	No Values	No Values	No Values	No Values
1000	seq=translation; coord=4:35209593..35215535:1; parent_transcript=GRMZM2G145972_T02; parent_gene=GRMZM2G145972	GRMZM2G145972_P02	65 kDa	Ref	0.9	0.8	1.4	2.5	No Values	No Values	No Values	No Values

1001	seq=translation; coord=9:143036859..143040005:-1; parent_transcript=GRMZM2G149809_T01; parent_gene=GRMZM2G149809	GRMZM2G149809_P01 (+1)	32 kDa	Ref	0.4	0.8	2.6	3.3	No Values	No Values	No Values	No Values
1002	seq=translation; coord=3:147969444..147972018:1; parent_transcript=GRMZM2G701801_T02; parent_gene=GRMZM2G701801	GRMZM2G701801_P02	22 kDa	Ref	0.4	0.4	0.8	1.1	No Values	No Values	No Values	No Values
1003	seq=translation; coord=8:159254849..159263818:-1; parent_transcript=GRMZM2G046558_T02; parent_gene=GRMZM2G046558	GRMZM2G046558_P02	32 kDa	Ref	-0.2	-0.4	0	-0.6	-3.1	-3	-3.4	-2.6
1004	seq=translation; coord=9:64132094..64145762:1; parent_transcript=GRMZM2G072909_T01; parent_gene=GRMZM2G072909	GRMZM2G072909_P01	47 kDa	Ref	-1.1	-1.2	-1.4	-1.1	0.2	0.6	-0.4	-0.1
1005	seq=translation; coord=4:194605171..194610906:-1; parent_transcript=GRMZM2G097043_T01; parent_gene=GRMZM2G097043	GRMZM2G097043_P01	88 kDa	Ref	1.1	0.4	0.8	0.3	-1.4	-0.7	-0.9	-0.8
1006	seq=translation; coord=1:3830541..3834833:1; parent_transcript=GRMZM2G085909_T01; parent_gene=GRMZM2G085909	GRMZM2G085909_P01	58 kDa	Ref	-1.1	-1.1	-0.6	-0.9	-2.1	-2	-2.3	-3.1
1007	seq=translation; coord=2:212802942..212807188:1; parent_transcript=GRMZM2G141510_T01; parent_gene=GRMZM2G141510	GRMZM2G141510_P01	47 kDa	Ref	0.3	0.2	-0.4	-0.3	1.5	1.2	1.5	0.9
1008	seq=translation; coord=8:10870907..10877056:1; parent_transcript=GRMZM2G030384_T01; parent_gene=GRMZM2G030384	GRMZM2G030384_P01	66 kDa	Ref	0.1	-0.2	-0.4	0.1	-2.9	-2.8	-2.4	-2.1
1009	seq=translation; coord=10:141573795..141580886:-1; parent_transcript=GRMZM2G156145_T01; parent_gene=GRMZM2G156145	GRMZM2G156145_P01	110 kDa	Ref	0.3	0.4	0.3	0.1	-0.3	-0.8	-0.7	-1.3
1010	seq=translation; coord=6:161231386..161234113:-1; parent_transcript=GRMZM2G159032_T01; parent_gene=GRMZM2G159032	GRMZM2G159032_P01 (+2)	33 kDa	Ref	-0.4	-1	-1.2	-1	-0.7	-3	-3.1	-3
1011	seq=translation; coord=5:183882422..183889051:-1; parent_transcript=GRMZM2G142088_T01; parent_gene=GRMZM2G142088	GRMZM2G142088_P01 (+1)	87 kDa	Ref	0.4	-0.5	-1.2	-0.7	-0.7	-2.7	-3.9	-3.4
1012	seq=translation; coord=5:204089494..204100246:-1; parent_transcript=GRMZM2G029186_T01; parent_gene=GRMZM2G029186	GRMZM2G029186_P01 (+3)	146 kDa	Ref	-0.6	-0.6	-1	-1	-4.1	-4.1	-4.5	-4.6
1013	seq=translation; coord=5:74450298..74452700:-1; parent_transcript=GRMZM5G831200_T01; parent_gene=GRMZM5G831200	GRMZM5G831200_P01	55 kDa	Ref	-0.3	-0.5	-0.6	-1	-3.7	-1.9	-0.6	-1

1014	seq=translation; coord=3:133205499..133209390:-1; parent_transcript=GRMZM2G136262_T01; parent_gene=GRMZM2G136262	GRMZM2G136262_P01	15 kDa	Ref	-0.5	-0.6	-0.3	-0.6	-1.3	-1.1	-1	-0.9
1015	seq=translation; coord=6:131028969..131032495:1; parent_transcript=GRMZM2G133631_T01; parent_gene=GRMZM2G133631	GRMZM2G133631_P01 (+5)	22 kDa	Ref	0.1	0.2	0.2	-0.1	3.2	3.3	3.4	3
1016	seq=translation; coord=6:140465764..140469654:1; parent_transcript=AC213884.3_FGT001; parent_gene=AC213884.3_FG001	AC213884.3_FGP001	24 kDa	Ref	0.2	0.2	0.5	-0.2	No Values	No Values	No Values	No Values
1017	seq=translation; coord=10:23245983..23251624:-1; parent_transcript=GRMZM2G013478_T01; parent_gene=GRMZM2G013478	GRMZM2G013478_P01	24 kDa	Ref	No Values	No Values	No Values	No Values	-0.8	-0.3	0.3	0.9
1018	seq=translation; coord=9:153591547..153607432:1; parent_transcript=GRMZM2G051219_T01; parent_gene=GRMZM2G051219	GRMZM2G051219_P01 (+1)	41 kDa	Ref	-2.3	-1.7	-1.3	-2.1	No Values	No Values	No Values	No Values
1019	seq=translation; coord=4:235606747..235623515:-1; parent_transcript=GRMZM2G119482_T01; parent_gene=GRMZM2G119482	GRMZM2G119482_P01	56 kDa	Ref	Reference Missing	0.1	Reference Missing	0.8	-3	-2.4	-2.5	-2.9
1020	seq=translation; coord=6:102399027..102412128:1; parent_transcript=GRMZM2G106427_T01; parent_gene=GRMZM2G106427	GRMZM2G106427_P01 (+3)	35 kDa	Ref	0.2	0.2	0.2	0.1	1.6	1.4	1.4	1.1
1021	seq=translation; coord=1:46061768..46066293:1; parent_transcript=GRMZM2G058138_T01; parent_gene=GRMZM2G058138	GRMZM2G058138_P01 (+2)	38 kDa	Ref	1	1	0.1	1	Reference Missing	Reference Missing	Reference Missing	Reference Missing
1022	seq=translation; coord=4:102065832..102092090:1; parent_transcript=GRMZM2G028766_T01; parent_gene=GRMZM2G028766	GRMZM2G028766_P01 (+1)	47 kDa	Ref	-0.5	-0.5	-1.4	-2.1	-3.4	-4	-4.1	-3.8
1023	seq=translation; coord=5:176347442..176349538:1; parent_transcript=GRMZM2G056629_T01; parent_gene=GRMZM2G056629	GRMZM2G056629_P01	41 kDa	Ref	2.3	2.3	1.8	1.4	Reference Missing	Reference Missing	Reference Missing	Reference Missing
1024	seq=translation; coord=6:160040980..160045154:-1; parent_transcript=GRMZM2G005036_T01; parent_gene=GRMZM2G005036	GRMZM2G005036_P01	54 kDa	Ref	0.7	0.6	0.2	1	5.3	3.6	3.6	3.1
1025	seq=translation; coord=1:274275100..274280175:1; parent_transcript=GRMZM2G135283_T03; parent_gene=GRMZM2G135283	GRMZM2G135283_P03 (+1)	57 kDa	Ref	0.1	-0.4	-0.3	0.2	5	5.5	5.4	4.6
1026	seq=translation; coord=1:191893507..191894902:1; parent_transcript=GRMZM2G427451_T01; parent_gene=GRMZM2G427451	GRMZM2G427451_P01	25 kDa	Ref	-0.4	-0.9	-2.3	-3.3	-0.5	-1	-0.9	-0.6

1027	seq=translation; coord=1:56746484..56752553:1; parent_transcript=GRMZM2G010823_T01; parent_gene=GRMZM2G010823	GRMZM2G010823_P01	54 kDa	Ref	0.4	0.3	0	0.5	1	0.6	0.4	0.1
1028	seq=translation; coord=8:116304892..116311147:-1; parent_transcript=GRMZM2G055489_T01; parent_gene=GRMZM2G055489	GRMZM2G055489_P01 (+2)	47 kDa	Ref	0.1	-0.1	-0.3	-0.1	1.6	1.8	2.2	2.2
1029	seq=translation; coord=5:56740173..56751799:1; parent_transcript=GRMZM2G115939_T01; parent_gene=GRMZM2G115939	GRMZM2G115939_P01 (+2)	21 kDa	Ref	-0.2	-0.3	-0.4	-0.6	-0.8	-0.6	-0.8	-0.6
1030	seq=translation; coord=4:163464001..163467089:-1; parent_transcript=GRMZM2G072612_T01; parent_gene=GRMZM2G072612	GRMZM2G072612_P01 (+1)	39 kDa	Ref	0.1	-0.2	0.1	0.9	1.2	1.9	2.3	2.9
1031	seq=translation; coord=10:106434010..106436707:-1; parent_transcript=GRMZM2G139512_T01; parent_gene=GRMZM2G139512	GRMZM2G139512_P01 (+3)	29 kDa	Ref	0.2	-0.4	-0.1	0.8	-0.8	-0.2	-0.6	-0.5
1032	seq=translation; coord=7:164069905..164076083:1; parent_transcript=GRMZM2G136918_T01; parent_gene=GRMZM2G136918	GRMZM2G136918_P01 (+4)	31 kDa	Ref	-1	-1.4	-0.7	-1.4	-1.9	-0.3	0	-0.3
1033	seq=translation; coord=10:148101348..148102945:1; parent_transcript=GRMZM5G892522_T01; parent_gene=GRMZM5G892522	GRMZM5G892522_P01	19 kDa	Ref	-1.6	-1.6	-1.2	-1.6	-3.8	-2.8	-2.5	0
1034	seq=translation; coord=4:154310162..154311883:-1; parent_transcript=GRMZM2G373522_T01; parent_gene=GRMZM2G373522	GRMZM2G373522_P01	31 kDa	Ref	2.3	2	2.2	2.5	3.1	2.4	2.1	2.3
1035	seq=translation; coord=5:113730181..113780006:1; parent_transcript=GRMZM2G056661_T01; parent_gene=GRMZM2G056661	GRMZM2G056661_P01 (+1)	222 kDa	Ref	No Values	No Values	No Values	No Values	1.5	0.3	0.8	-1.9
1036	seq=translation; coord=8:173103213..173107748:1; parent_transcript=GRMZM2G124365_T01; parent_gene=GRMZM2G124365	GRMZM2G124365_P01	35 kDa	Ref	No Values	No Values	No Values	No Values	-0.8	-0.4	-0.7	1.7
1037	seq=translation; coord=1:60216405..60219476:-1; parent_transcript=GRMZM2G144995_T01; parent_gene=GRMZM2G144995	GRMZM2G144995_P01 (+1)	61 kDa	Ref	No Values	No Values	No Values	No Values	1.1	0.5	0.7	-1.1
1038	seq=translation; coord=5:64107900..64117086:1; parent_transcript=GRMZM2G145088_T01; parent_gene=GRMZM2G145088	GRMZM2G145088_P01	131 kDa	Ref	-0.2	-0.4	-0.6	-0.6	No Values	No Values	No Values	No Values
1039	seq=translation; coord=4:238325251..238328383:-1; parent_transcript=GRMZM2G060567_T01; parent_gene=GRMZM2G060567	GRMZM2G060567_P01	26 kDa	Ref	2.6	2.5	2.5	1.4	No Values	No Values	No Values	No Values

1040	seq=translation; coord=7:106230652..106231840:1; parent_transcript=GRMZM2G025646_T01; parent_gene=GRMZM2G025646	GRMZM2G025646_P01 (+1)	15 kDa	Ref	No Values	No Values	No Values	No Values	3.2	2.9	2.3	1.6
1041	seq=translation; coord=3:22814532..22820273:-1; parent_transcript=GRMZM2G009282_T01; parent_gene=GRMZM2G009282	GRMZM2G009282_P01	102 kDa	Ref	1.1	0.1	-0.7	0.7	No Values	No Values	No Values	No Values
1042	seq=translation; coord=2:130291275..130295558:-1; parent_transcript=GRMZM2G118800_T01; parent_gene=GRMZM2G118800	GRMZM2G118800_P01	52 kDa	Ref	1.4	0.5	0.9	1.9	No Values	No Values	No Values	No Values
1043	seq=translation; coord=4:172748163..172754261:1; parent_transcript=GRMZM2G178398_T01; parent_gene=GRMZM2G178398	GRMZM2G178398_P01	99 kDa	Ref	0.8	0.5	0.2	0.1	No Values	No Values	No Values	No Values
1044	seq=translation; coord=5:182124005..182130631:-1; parent_transcript=GRMZM2G137409_T01; parent_gene=GRMZM2G137409	GRMZM2G137409_P01	82 kDa	Ref	0.1	0.2	1.4	2.2	No Values	No Values	No Values	No Values
1045	seq=translation; coord=1:293041177..293042705:-1; parent_transcript=GRMZM2G155329_T01; parent_gene=GRMZM2G155329	GRMZM2G155329_P01	24 kDa	Ref	-0.1	-0.3	1.2	2.1	No Values	No Values	No Values	No Values
1046	seq=translation; coord=7:156569238..156573041:1; parent_transcript=GRMZM2G153162_T02; parent_gene=GRMZM2G153162	GRMZM2G153162_P02	101 kDa	Ref	No Values	No Values	No Values	No Values	0.1	-0.9	-0.5	-1
1047	seq=translation; coord=3:123772202..123775285:1; parent_transcript=GRMZM2G164868_T01; parent_gene=GRMZM2G164868	GRMZM2G164868_P01 (+1)	58 kDa	Ref	No Values	No Values	No Values	No Values	0.8	-1.5	-0.4	-0.7
1048	seq=translation; coord=4:163261154..163272206:1; parent_transcript=GRMZM2G014994_T01; parent_gene=GRMZM2G014994	GRMZM2G014994_P01	18 kDa	Ref	0.5	-0.4	-1.2	-2	No Values	No Values	No Values	No Values
1049	seq=translation; coord=10:12167272..12169103:-1; parent_transcript=GRMZM2G094375_T01; parent_gene=GRMZM2G094375	GRMZM2G094375_P01 (+1)	47 kDa	Ref	0.5	0.3	1	2	No Values	No Values	No Values	No Values
1050	seq=translation; coord=3:231890529..231892088:-1; parent_transcript=GRMZM2G175076_T01; parent_gene=GRMZM2G175076	GRMZM2G175076_P01	24 kDa	Ref	0.1	-0.2	1.6	2.4	No Values	No Values	No Values	No Values
1051	seq=translation; coord=6:166587013..166592611:1; parent_transcript=GRMZM2G027955_T01; parent_gene=GRMZM2G027955	GRMZM2G027955_P01 (+2)	59 kDa	Ref	-1.2	-1.4	-2	-2.2	1.6	1.7	1.2	1.2
1052	seq=translation; coord=2:36317292..36319768:1; parent_transcript=GRMZM2G102183_T01; parent_gene=GRMZM2G102183	GRMZM2G102183_P01 (+2)	47 kDa	Ref	0.2	0.4	-0.1	1	0.4	0.6	Reference Missing	Reference Missing

1053	seq=translation; coord=4:173177749..173179720:-1; parent_transcript=GRMZM2G054012_T01; parent_gene=GRMZM2G054012	GRMZM2G054012_P01 (+3)	7 kDa	Ref	1	0.3	-0.3	-0.8	1.6	0.4	0.5	0
1054	seq=translation; coord=10:63619774..63622627:-1; parent_transcript=GRMZM2G368861_T01; parent_gene=GRMZM2G368861	GRMZM2G368861_P01	9 kDa	Ref	0.3	3.9	3	1.8	0.7	0.5	0.6	-0.2
1055	seq=translation; coord=1:191987932..191990561:1; parent_transcript=AC197246.3_FGT003; parent_gene=AC197246.3_FG003	AC197246.3_FGP003 (+4)	16 kDa	Ref	0.2	0	0	0.4	-3.1	-2.8	-3	-3.9
1056	seq=translation; coord=10:127861555..127867760:-1; parent_transcript=GRMZM2G096705_T01; parent_gene=GRMZM2G096705	GRMZM2G096705_P01	49 kDa	Ref	0.3	-0.3	-0.4	-0.6	0.9	-1.2	-0.8	-0.6
1057	seq=translation; coord=7:82007091..82010522:1; parent_transcript=GRMZM2G056501_T01; parent_gene=GRMZM2G056501	GRMZM2G056501_P01 (+2)	18 kDa	Ref	-1	-1	-1	-1.6	-0.8	-0.3	-1.1	-0.8
1058	seq=translation; coord=2:189443283..189447454:1; parent_transcript=GRMZM2G112165_T01; parent_gene=GRMZM2G112165	GRMZM2G112165_P01	80 kDa	Ref	-2.1	-2.5	-2.6	-2.3	1.3	1	1.2	1.3
1059	seq=translation; coord=8:127487910..127490998:1; parent_transcript=GRMZM2G150485_T01; parent_gene=GRMZM2G150485	GRMZM2G150485_P01 (+2)	33 kDa	Ref	-1	-0.6	-1	-1.2	3	2.8	2.9	1.6
1060	seq=translation; coord=2:217093149..217097528:-1; parent_transcript=GRMZM2G030646_T01; parent_gene=GRMZM2G030646	GRMZM2G030646_P01	30 kDa	Ref	1.4	1.1	0.4	0.9	-1.2	-1.7	-1.7	-1.6
1061	seq=translation; coord=1:27094498..27098727:1; parent_transcript=GRMZM2G163129_T01; parent_gene=GRMZM2G163129	GRMZM2G163129_P01	103 kDa	Ref	0	-0.5	-0.4	-0.3	-0.8	-1.2	-0.8	-0.7
1062	seq=translation; coord=6:109292609..109296377:1; parent_transcript=GRMZM2G048194_T01; parent_gene=GRMZM2G048194	GRMZM2G048194_P01 (+1)	41 kDa	Ref	No Values	No Values	No Values	No Values	-0.4	-1.4	-1.6	-2.2
1063	seq=translation; coord=8:78073258..78079706:1; parent_transcript=GRMZM2G023418_T01; parent_gene=GRMZM2G023418	GRMZM2G023418_P01	144 kDa	Ref	1	-0.2	0.4	-0.1	-1	-2	-1.9	-2.5
1064	seq=translation; coord=9:150816519..150821326:-1; parent_transcript=GRMZM2G169365_T01; parent_gene=GRMZM2G169365	GRMZM2G169365_P01	39 kDa	Ref	0.3	-0.6	-0.5	-0.7	-3.6	-3.5	-2.7	-2.6
1065	seq=translation; coord=2:193416339..193419729:-1; parent_transcript=GRMZM2G023080_T01; parent_gene=GRMZM2G023080	GRMZM2G023080_P01 (+3)	29 kDa	Ref	-0.1	-0.6	-0.2	-0.4	0.2	0.7	-0.5	0.1

1066	seq=translation; coord=5:30771033..30772796:1; parent_transcript=GRMZM2G026216_T01; parent_gene=GRMZM2G026216	GRMZM2G026216_P01 (+3)	19 kDa	Ref	1.1	1	0	0.3	1.1	0.8	0.4	-0.7
1067	seq=translation; coord=5:215656610..215661825:1; parent_transcript=GRMZM2G143651_T01; parent_gene=GRMZM2G143651	GRMZM2G143651_P01	50 kDa	Ref	2.1	1.6	1.1	2.1	Reference Missing	Reference Missing	Reference Missing	Value Missing
1068	seq=translation; coord=6:28127232..28129613:1; parent_transcript=GRMZM2G165815_T02; parent_gene=GRMZM2G165815	GRMZM2G165815_P02	23 kDa	Ref	0.6	0.3	0.5	-0.7	-0.9	0.2	-0.5	1.7
1069	seq=translation; coord=8:154456893..154470550:1; parent_transcript=GRMZM2G316113_T01; parent_gene=GRMZM2G316113	GRMZM2G316113_P01 (+2)	155 kDa	Ref	0.2	-0.4	-0.9	0.6	-0.5	-1.4	-1.1	-1.1
1070	seq=translation; coord=6:24549898..24554957:-1; parent_transcript=GRMZM2G111579_T01; parent_gene=GRMZM2G111579	GRMZM2G111579_P01	57 kDa	Ref	-0.7	-0.9	-0.4	-0.1	-0.8	-0.1	0.1	-0.1
1071	seq=translation; coord=5:163610246..163620614:1; parent_transcript=GRMZM2G360589_T01; parent_gene=GRMZM2G360589	GRMZM2G360589_P01	46 kDa	Ref	-0.8	-0.7	-0.6	0.3	-1.4	-0.5	-1.3	-1.5
1072	seq=translation; coord=2:76993674..76995064:1; parent_transcript=GRMZM2G069523_T01; parent_gene=GRMZM2G069523	GRMZM2G069523_P01	28 kDa	Ref	0	-0.2	0.2	0.2	0.9	0.6	1.1	0.7
1073	seq=translation; coord=4:240105137..240108983:1; parent_transcript=GRMZM2G079348_T01; parent_gene=GRMZM2G079348	GRMZM2G079348_P01 (+3)	79 kDa	Ref	-1.5	-2.6	-2.2	-1.3	-1.8	-2.1	-2.3	-2.7
1074	seq=translation; coord=5:3195909..3207226:1; parent_transcript=GRMZM2G071071_T01; parent_gene=GRMZM2G071071	GRMZM2G071071_P01	72 kDa	Ref	1	1.2	0.4	0.9	Reference Missing	Reference Missing	Reference Missing	Reference Missing
1075	seq=translation; coord=8:94750778..94753384:1; parent_transcript=GRMZM2G147221_T01; parent_gene=GRMZM2G147221	GRMZM2G147221_P01	59 kDa	Ref	-1	Value Missing	-1.4	Value Missing	5	4.5	4.2	4.6
1076	seq=translation; coord=6:6269340..6271805:-1; parent_transcript=GRMZM2G134134_T02; parent_gene=GRMZM2G134134	GRMZM2G134134_P02	40 kDa	Ref	-0.2	-0.1	-0.2	0.2	-2.1	-2.4	-2.2	-2.1
1077	seq=translation; coord=9:19034931..19038665:-1; parent_transcript=GRMZM5G877388_T02; parent_gene=GRMZM5G877388	GRMZM5G877388_P02	14 kDa	Ref	-1.3	-1.4	-1.3	-1.7	-2.2	-3.1	-2.8	-3.5
1078	seq=translation; coord=1:34664608..34673432:1; parent_transcript=GRMZM2G058870_T01; parent_gene=GRMZM2G058870	GRMZM2G058870_P01	198 kDa	Ref	1.1	0.9	0.1	0.4	0.7	-0.7	-1.3	-1.5

1079	seq=translation; coord=5:6688332..6691221:1; parent_transcript=GRMZM5G862540_T01; parent_gene=GRMZM5G862540	GRMZM5G862540_P01 (+1)	53 kDa	Ref	-0.6	-0.7	-1	-0.6	0.2	Value Missing	-1.5	-0.5
1080	seq=translation; coord=9:57980157..57982564:1; parent_transcript=GRMZM2G066111_T01; parent_gene=GRMZM2G066111	GRMZM2G066111_P01 (+3)	24 kDa	Ref	1.2	1.2	0.4	1.2	2.1	0.4	0.1	-0.4
1081	seq=translation; coord=3:87798819..87808953:-1; parent_transcript=GRMZM2G009593_T01; parent_gene=GRMZM2G009593	GRMZM2G009593_P01 (+2)	106 kDa	Ref	0	0.7	-0.1	-0.2	-4.7	-5	-5.5	-5.5
1082	seq=translation; coord=2:196359627..196363692:-1; parent_transcript=GRMZM2G573867_T01; parent_gene=GRMZM2G573867	GRMZM2G573867_P01 (+1)	48 kDa	Ref	No Values	No Values	No Values	No Values	-0.9	-0.3	-0.3	-0.4
1083	seq=translation; coord=1:49207907..49216831:1; parent_transcript=GRMZM2G140432_T01; parent_gene=GRMZM2G140432	GRMZM2G140432_P01	95 kDa	Ref	0.6	-0.1	0.9	-0.3	No Values	No Values	No Values	No Values
1084	seq=translation; coord=5:214501921..214505061:1; parent_transcript=GRMZM2G008728_T02; parent_gene=GRMZM2G008728	GRMZM2G008728_P02	34 kDa	Ref	0	0.1	0.5	0.2	No Values	No Values	No Values	No Values
1085	seq=translation; coord=1:298585659..298587727:-1; parent_transcript=GRMZM2G058568_T03; parent_gene=GRMZM2G058568	GRMZM2G058568_P03 (+1)	57 kDa	Ref	0.4	0.1	0.4	0.4	No Values	No Values	No Values	No Values
1086	seq=translation; coord=9:116241684..116244580:-1; parent_transcript=GRMZM2G013002_T01; parent_gene=GRMZM2G013002	GRMZM2G013002_P01	32 kDa	Ref	1.1	0.8	0.6	1.1	No Values	No Values	No Values	No Values
1087	seq=translation; coord=9:107790531..107797125:1; parent_transcript=GRMZM2G055320_T01; parent_gene=GRMZM2G055320	GRMZM2G055320_P01	68 kDa	Ref	-0.9	-0.8	-1.2	-1.6	No Values	No Values	No Values	No Values
1088	seq=translation; coord=8:143676986..143682414:1; parent_transcript=GRMZM2G107757_T01; parent_gene=GRMZM2G107757	GRMZM2G107757_P01 (+2)	25 kDa	Ref	Reference Missing	2.3	1.9	1.3	No Values	No Values	No Values	No Values
1089	seq=translation; coord=3:147676601..147679180:-1; parent_transcript=GRMZM2G479112_T01; parent_gene=GRMZM2G479112	GRMZM2G479112_P01	57 kDa	Ref	-0.1	0.1	0.1	0.8	No Values	No Values	No Values	No Values
1090	seq=translation; coord=8:8472359..8474789:1; parent_transcript=GRMZM2G021614_T01; parent_gene=GRMZM2G021614	GRMZM2G021614_P01 (+1)	19 kDa	Ref	No Values	No Values	No Values	No Values	-0.4	1.1	1.6	2.1
1091	seq=translation; coord=6:123432811..123435820:-1; parent_transcript=GRMZM2G127160_T01; parent_gene=GRMZM2G127160	GRMZM2G127160_P01 (+1)	47 kDa	Ref	No Values	No Values	No Values	No Values	2.1	0.6	0.6	0.2

1092	seq=translation; coord=9:7614057..7616811:1; parent_transcript=GRMZM2G079949_T01; parent_gene=GRMZM2G079949	GRMZM2G079949_P01	35 kDa	Ref	-0.3	-0.2	-0.1	0.3	No Values	No Values	No Values	No Values
1093	seq=translation; coord=5:188857165..188858944:1; parent_transcript=GRMZM2G170017_T01; parent_gene=GRMZM2G170017	GRMZM2G170017_P01	34 kDa	Ref	Reference Missing	0.1	3.7	5.5	No Values	No Values	No Values	No Values
1094	seq=translation; coord=6:160034201..160039816:-1; parent_transcript=GRMZM2G004932_T01; parent_gene=GRMZM2G004932	GRMZM2G004932_P01	63 kDa	Ref	0.6	0.1	0.1	1.1	No Values	No Values	No Values	No Values
1095	seq=translation; coord=1:38548724..38550734:-1; parent_transcript=GRMZM2G012566_T02; parent_gene=GRMZM2G012566	GRMZM2G012566_P02	18 kDa	Ref	1.4	1.8	1	0	No Values	No Values	No Values	No Values
1096	seq=translation; coord=4:53673036..53676735:-1; parent_transcript=GRMZM2G032367_T01; parent_gene=GRMZM2G032367	GRMZM2G032367_P01 (+5)	34 kDa	Ref	1.3	1.2	1.8	1.8	No Values	No Values	No Values	No Values
1097	seq=translation; coord=1:258447609..258448792:1; parent_transcript=GRMZM2G039639_T01; parent_gene=GRMZM2G039639	GRMZM2G039639_P01	24 kDa	Ref	-0.1	-0.5	Reference Missing	Reference Missing	No Values	No Values	No Values	No Values
1098	seq=translation; coord=1:7406938..7408184:1; parent_transcript=GRMZM2G099454_T02; parent_gene=GRMZM2G099454	GRMZM2G099454_P02 (+3)	28 kDa	Ref	1.3	2.1	1.2	2.5	No Values	No Values	No Values	No Values
1099	seq=translation; coord=5:49897243..49899430:-1; parent_transcript=GRMZM2G177828_T01; parent_gene=GRMZM2G177828	GRMZM2G177828_P01	14 kDa	Ref	4.9	4.5	3.2	1.8	No Values	No Values	No Values	No Values
1100	seq=translation; coord=8:2199781..2200562:-1; parent_transcript=GRMZM2G353266_T01; parent_gene=GRMZM2G353266	GRMZM2G353266_P01	14 kDa	Ref	No Values	No Values	No Values	No Values	-0.1	-0.2	-0.1	0
1101	seq=translation; coord=7:3483732..3484794:1; parent_transcript=GRMZM2G465226_T01; parent_gene=GRMZM2G465226	GRMZM2G465226_P01	17 kDa	Ref	0.3	0	-0.2	-0.1	No Values	No Values	No Values	No Values
1102	seq=translation; coord=7:29542055..29544356:-1; parent_transcript=GRMZM5G872934_T01; parent_gene=GRMZM5G872934	GRMZM5G872934_P01	31 kDa	Ref	0.3	1.3	1.5	1.6	No Values	No Values	No Values	No Values
1103	seq=translation; coord=2:12351628..12357486:-1; parent_transcript=GRMZM2G125728_T01; parent_gene=GRMZM2G125728	GRMZM2G125728_P01 (+1)	63 kDa	Ref	0.5	0.3	-0.1	-0.2	Reference Missing	2.8	2.8	2.6
1104	seq=translation; coord=2:236283794..236288865:1; parent_transcript=GRMZM2G103955_T02; parent_gene=GRMZM2G103955	GRMZM2G103955_P02	49 kDa	Ref	1.5	0.9	0.7	0.2	3.2	1.1	0.8	-0.6

1105	seq=translation; coord=1:194210583..194219751:1; parent_transcript=GRMZM2G171111_T02; parent_gene=GRMZM2G171111	GRMZM2G171111_P02 (+1)	27 kDa	Ref	-0.8	-0.4	-0.6	-0.4	0.7	0.3	0.7	0.7
1106	seq=translation; coord=2:115064269..115065362:-1; parent_transcript=GRMZM2G008649_T01; parent_gene=GRMZM2G008649	GRMZM2G008649_P01 (+1)	25 kDa	Ref	No Values	No Values	No Values	No Values	-1.7	-2	-2.2	-2.3
1107	seq=translation; coord=3:193091877..193119714:1; parent_transcript=GRMZM2G071441_T01; parent_gene=GRMZM2G071441	GRMZM2G071441_P01 (+1)	113 kDa	Ref	0.3	0.6	-0.1	0.3	1.8	1.9	1.3	1.1
1108	seq=translation; coord=5:1526460..1528840:-1; parent_transcript=GRMZM2G448001_T01; parent_gene=GRMZM2G448001	GRMZM2G448001_P01	74 kDa	Ref	-0.1	0.5	1	1.7	-2.6	-0.8	0	0.6
1109	seq=translation; coord=5:14752854..14761623:1; parent_transcript=GRMZM2G553314_T01; parent_gene=GRMZM2G553314	GRMZM2G553314_P01	84 kDa	Ref	-0.2	-0.5	-0.3	-0.1	-2	-2	-2.3	-2.4
1110	seq=translation; coord=1:67523715..67533727:-1; parent_transcript=GRMZM2G410479_T01; parent_gene=GRMZM2G410479	GRMZM2G410479_P01	169 kDa	Ref	-0.1	-0.3	-0.8	-0.1	1.8	1.7	0.4	0.5
1111	seq=translation; coord=7:156205011..156210949:-1; parent_transcript=GRMZM5G890190_T01; parent_gene=GRMZM5G890190	GRMZM5G890190_P01 (+1)	28 kDa	Ref	0	-0.2	-0.4	-0.7	-3.3	-3.9	-4.3	-4.2
1112	seq=translation; coord=4:201936215..201938145:1; parent_transcript=GRMZM2G325118_T01; parent_gene=GRMZM2G325118	GRMZM2G325118_P01	46 kDa	Ref	0.8	3	4.3	3.9	0	Value Missing	0.2	Reference Missing
1113	seq=translation; coord=9:130498884..130500159:1; parent_transcript=GRMZM2G134107_T01; parent_gene=GRMZM2G134107	GRMZM2G134107_P01	14 kDa	Ref	0.7	1.1	1	0.5	3.4	3	3	2.4
1114	seq=translation; coord=2:8578099..8582568:-1; parent_transcript=GRMZM2G012628_T01; parent_gene=GRMZM2G012628	GRMZM2G012628_P01	47 kDa	Ref	-0.5	-0.4	-0.7	-1	1	0.1	-0.6	-0.3
1115	seq=translation; coord=7:11441042..11445373:-1; parent_transcript=GRMZM2G401308_T02; parent_gene=GRMZM2G401308	GRMZM2G401308_P02	22 kDa	Ref	0.6	-0.2	-1.2	-1.9	-1	-2.1	-2.1	-2.1
1116	seq=translation; coord=3:44223365..44228905:-1; parent_transcript=GRMZM2G003883_T01; parent_gene=GRMZM2G003883	GRMZM2G003883_P01	55 kDa	Ref	-0.9	-1	-0.5	-0.4	2.4	1.3	1.1	0.5
1117	seq=translation; coord=8:29610407..29612711:1; parent_transcript=GRMZM2G067522_T01; parent_gene=GRMZM2G067522	GRMZM2G067522_P01	16 kDa	Ref	0.2	0.3	0.7	-0.1	-2.7	-1.7	-1.7	-3.6

1118	seq=translation; coord=4:17087184..17096848:1; parent_transcript=GRMZM2G045987_T01; parent_gene=GRMZM2G045987	GRMZM2G045987_P01	86 kDa	Ref	0.1	-0.2	-0.5	-0.1	-2	-2.5	-2.9	-3.2
1119	seq=translation; coord=10:11749519..11756094:-1; parent_transcript=GRMZM2G036534_T01; parent_gene=GRMZM2G036534	GRMZM2G036534_P01	48 kDa	Ref	Value Missing	1	0.9	0.9	-2.1	Reference Missing	-3.7	-3.7
1120	seq=translation; coord=4:187884467..187890415:-1; parent_transcript=GRMZM2G119175_T01; parent_gene=GRMZM2G119175	GRMZM2G119175_P01 (+1)	57 kDa	Ref	-0.2	-0.2	-0.8	-0.2	2.4	1.9	0.7	0.7
1121	seq=translation; coord=7:154188635..154191068:-1; parent_transcript=GRMZM2G056369_T01; parent_gene=GRMZM2G056369	GRMZM2G056369_P01	63 kDa	Ref	0.3	1.1	0.1	0.4	2.6	Reference Missing	Reference Missing	Reference Missing
1122	seq=translation; coord=5:30994200..30995891:-1; parent_transcript=GRMZM2G111477_T01; parent_gene=GRMZM2G111477	GRMZM2G111477_P01	21 kDa	Ref	-1	-0.6	-0.1	-0.8	-2.6	-2.2	-1.8	-2
1123	seq=translation; coord=4:140347561..140350401:1; parent_transcript=GRMZM2G079817_T01; parent_gene=GRMZM2G079817	GRMZM2G079817_P01	59 kDa	Ref	-0.4	-0.6	-0.1	0.4	2	2.3	2.6	2.8
1124	seq=translation; coord=1:189145769..189150346:-1; parent_transcript=GRMZM2G088627_T01; parent_gene=GRMZM2G088627	GRMZM2G088627_P01	49 kDa	Ref	1.4	1.6	1.2	1.4	2.7	1.4	1.4	2.8
1125	seq=translation; coord=6:151454748..151457344:-1; parent_transcript=GRMZM2G059314_T01; parent_gene=GRMZM2G059314	GRMZM2G059314_P01	41 kDa	Ref	-0.2	-0.2	-0.4	-0.8	2.2	2	2.3	3.4
1126	seq=translation; coord=5:13726531..13727525:-1; parent_transcript=GRMZM2G129083_T01; parent_gene=GRMZM2G129083	GRMZM2G129083_P01	16 kDa	Ref	1.5	1.8	2.7	2.2	1.4	-0.5	-0.4	-0.9
1127	seq=translation; coord=9:22677939..22681262:-1; parent_transcript=GRMZM5G877500_T01; parent_gene=GRMZM5G877500	GRMZM5G877500_P01	42 kDa	Ref	-0.7	-0.5	-0.5	-0.1	1.4	1.2	0.9	0.9
1128	seq=translation; coord=3:160397311..160408336:1; parent_transcript=GRMZM2G129155_T01; parent_gene=GRMZM2G129155	GRMZM2G129155_P01 (+1)	90 kDa	Ref	1.1	0.1	-0.3	1.1	2.3	1.1	1.9	1.1
1129	seq=translation; coord=1:132810230..132856187:1; parent_transcript=GRMZM2G019673_T01; parent_gene=GRMZM2G019673	GRMZM2G019673_P01 (+2)	80 kDa	Ref	-0.4	-0.4	-0.1	0.6	-0.4	-0.6	-0.7	-1
1130	seq=translation; coord=4:237676754..237682663:-1; parent_transcript=GRMZM2G117346_T01; parent_gene=GRMZM2G117346	GRMZM2G117346_P01 (+5)	142 kDa	Ref	3.8	3.3	2.9	2.6	Reference Missing	Reference Missing	Reference Missing	Reference Missing

1131	seq=translation; coord=2:45890416..45891705:-1; parent_transcript=GRMZM2G152141_T01; parent_gene=GRMZM2G152141	GRMZM2G152141_P01	19 kDa	Ref	-1.6	-1.3	-1.6	-1.3	Value Missing	3.3	Value Missing	Value Missing
1132	seq=translation; coord=8:135417381..135418239:1; parent_transcript=GRMZM2G175273_T01; parent_gene=GRMZM2G175273	GRMZM2G175273_P01	8 kDa	Ref	2.3	2.1	1.9	0.9	No Values	No Values	No Values	No Values
1133	seq=translation; coord=4:183588190..183591209:-1; parent_transcript=GRMZM2G325575_T01; parent_gene=GRMZM2G325575	GRMZM2G325575_P01	37 kDa	Ref	-0.2	0.7	0.4	1	1	1.1	0.9	2.2
1134	seq=translation; coord=6:115639609..115647217:-1; parent_transcript=GRMZM2G008556_T01; parent_gene=GRMZM2G008556	GRMZM2G008556_P01 (+3)	68 kDa	Ref	0.8	-0.1	0	-0.5	3.1	2.6	2.8	3.2
1135	seq=translation; coord=3:196019180..196024451:1; parent_transcript=GRMZM2G324886_T01; parent_gene=GRMZM2G324886	GRMZM2G324886_P01	25 kDa	Ref	0.4	-0.3	-0.5	-1.1	-4.3	-3.2	-4.5	-3.9
1136	seq=translation; coord=4:168987228..168990822:1; parent_transcript=GRMZM2G018947_T01; parent_gene=GRMZM2G018947	GRMZM2G018947_P01 (+2)	46 kDa	Ref	-1.1	-1.4	-1.2	-0.7	-4.3	-4.9	-5.6	-5.8
1137	seq=translation; coord=8:157966228..157972830:-1; parent_transcript=GRMZM2G016827_T02; parent_gene=GRMZM2G016827	GRMZM2G016827_P02	33 kDa	Ref	No Values	No Values	No Values	No Values	-0.6	Reference Missing	0.1	0.4
1138	seq=translation; coord=8:2349948..2353138:1; parent_transcript=GRMZM2G027728_T01; parent_gene=GRMZM2G027728	GRMZM2G027728_P01 (+1)	12 kDa	Ref	0.8	1	0.4	0.3	0.1	-0.7	-1	-1
1139	seq=translation; coord=7:1306578..1314769:-1; parent_transcript=GRMZM2G120563_T06; parent_gene=GRMZM2G120563	GRMZM2G120563_P06 (+1)	44 kDa	Ref	-0.7	-1.9	-1.8	-1.2	-0.9	-0.6	-0.5	0.1
1140	seq=translation; coord=1:197854710..197858129:-1; parent_transcript=GRMZM2G021816_T01; parent_gene=GRMZM2G021816	GRMZM2G021816_P01	39 kDa	Ref	-0.6	-0.7	-0.3	-0.9	No Values	No Values	No Values	No Values
1141	seq=translation; coord=3:134285297..134289508:1; parent_transcript=GRMZM2G047434_T01; parent_gene=GRMZM2G047434	GRMZM2G047434_P01	38 kDa	Ref	No Values	No Values	No Values	No Values	1	-1.9	-1.3	-1.3
1142	seq=translation; coord=6:76236848..76238660:1; parent_transcript=GRMZM2G051338_T01; parent_gene=GRMZM2G051338	GRMZM2G051338_P01	20 kDa	Ref	-0.3	0	1.9	0.7	No Values	No Values	No Values	No Values
1143	seq=translation; coord=2:20765568..20767537:1; parent_transcript=GRMZM2G337229_T01; parent_gene=GRMZM2G337229	GRMZM2G337229_P01	16 kDa	Ref	-0.5	2.1	0.7	0.4	No Values	No Values	No Values	No Values

1144	seq=translation; coord=3:221235623..221241542:1; parent_transcript=GRMZM2G149321_T01; parent_gene=GRMZM2G149321	GRMZM2G149321_P01	53 kDa	Ref	No Values	No Values	No Values	No Values	0.7	-0.6	0.6	-1.6
1145	seq=translation; coord=1:264855610..264864472:-1; parent_transcript=AC217401.3_FGT002; parent_gene=AC217401.3_FG002	AC217401.3_FGP002	63 kDa	Ref	-0.1	-0.4	-0.6	0.5	No Values	No Values	No Values	No Values
1146	seq=translation; coord=1:269493908..269506409:1; parent_transcript=GRMZM5G826838_T01; parent_gene=GRMZM5G826838	GRMZM5G826838_P01	55 kDa	Ref	0.1	0.2	0.3	0.3	No Values	No Values	No Values	No Values
1147	seq=translation; coord=9:12103632..12110868:-1; parent_transcript=GRMZM2G467169_T01; parent_gene=GRMZM2G467169	GRMZM2G467169_P01 (+1)	107 kDa	Ref	No Values	No Values	No Values	No Values	Reference Missing	Reference Missing	1.4	0.8
1148	seq=translation; coord=1:278837830..278839720:1; parent_transcript=GRMZM2G118809_T01; parent_gene=GRMZM2G118809	GRMZM2G118809_P01 (+1)	57 kDa	Ref	0.3	0.1	Reference Missing	Reference Missing	No Values	No Values	No Values	No Values
1149	seq=translation; coord=9:138771755..138777249:1; parent_transcript=GRMZM2G162426_T01; parent_gene=GRMZM2G162426	GRMZM2G162426_P01	116 kDa	Ref	0.5	0	-0.4	0.2	No Values	No Values	No Values	No Values
1150	seq=translation; coord=1:276433043..276437555:-1; parent_transcript=GRMZM2G100084_T01; parent_gene=GRMZM2G100084	GRMZM2G100084_P01	53 kDa	Ref	No Values	No Values	No Values	No Values	0.3	0.8	0.9	1
1151	seq=translation; coord=4:177628395..177630803:-1; parent_transcript=GRMZM2G126732_T01; parent_gene=GRMZM2G126732	GRMZM2G126732_P01 (+1)	41 kDa	Ref	0.6	1.5	2	1.8	No Values	No Values	No Values	No Values
1152	seq=translation; coord=9:26817670..26821498:1; parent_transcript=GRMZM2G475293_T01; parent_gene=GRMZM2G475293	GRMZM2G475293_P01 (+1)	27 kDa	Ref	No Values	No Values	No Values	No Values	-1.2	-0.5	-0.3	1.4
1153	seq=translation; coord=3:166381506..166384595:-1; parent_transcript=GRMZM2G359397_T01; parent_gene=GRMZM2G359397	GRMZM2G359397_P01 (+2)	51 kDa	Ref	No Values	No Values	No Values	No Values	0.8	1.7	2	2
1154	seq=translation; coord=2:199547128..199550293:1; parent_transcript=GRMZM5G868679_T01; parent_gene=GRMZM5G868679	GRMZM5G868679_P01	69 kDa	Ref	0.2	0.2	0.4	1	No Values	No Values	No Values	No Values
1155	seq=translation; coord=4:11466211..11470058:-1; parent_transcript=GRMZM2G085711_T02; parent_gene=GRMZM2G085711	GRMZM2G085711_P02	50 kDa	Ref	No Values	No Values	No Values	No Values	0.6	1.5	1.9	2.5
1156	seq=translation; coord=7:7954180..7955128:1; parent_transcript=GRMZM2G021149_T01; parent_gene=GRMZM2G021149	GRMZM2G021149_P01 (+1)	28 kDa	Ref	0.3	0.1	0.2	0.1	No Values	No Values	No Values	No Values

1157	seq=translation; coord=7:152772009..152776227:1; parent_transcript=GRMZM2G108849_T02; parent_gene=GRMZM2G108849	GRMZM2G108849_P02 (+2)	23 kDa	Ref	0.4	0.1	0.3	1.3	No Values	No Values	No Values	No Values
1158	seq=translation; coord=10:144683393..144689170:-1; parent_transcript=GRMZM2G136895_T01; parent_gene=GRMZM2G136895	GRMZM2G136895_P01	81 kDa	Ref	0	-0.1	0.3	0.4	No Values	No Values	No Values	No Values
1159	seq=translation; coord=3:91357080..91360381:-1; parent_transcript=GRMZM2G077415_T01; parent_gene=GRMZM2G077415	GRMZM2G077415_P01	24 kDa	Ref	-0.4	0.1	-0.2	0	No Values	No Values	No Values	No Values
1160	seq=translation; coord=7:149132369..149134157:-1; parent_transcript=GRMZM2G077673_T01; parent_gene=GRMZM2G077673	GRMZM2G077673_P01	38 kDa	Ref	-0.5	0	-0.2	0.2	No Values	No Values	No Values	No Values
1161	seq=translation; coord=4:184547632..184552118:-1; parent_transcript=GRMZM2G347541_T01; parent_gene=GRMZM2G347541	GRMZM2G347541_P01 (+1)	69 kDa	Ref	No Values	No Values	No Values	No Values	0.3	-0.2	-0.1	0.1
1162	seq=translation; coord=7:1269463..1270529:-1; parent_transcript=GRMZM2G420733_T01; parent_gene=GRMZM2G420733	GRMZM2G420733_P01	25 kDa	Ref	-0.2	0.3	0.2	-0.4	No Values	No Values	No Values	No Values
1163	seq=translation; coord=3:207473256..207484650:-1; parent_transcript=GRMZM2G073045_T01; parent_gene=GRMZM2G073045	GRMZM2G073045_P01	38 kDa	Ref	-0.3	-0.3	-0.3	-0.5	No Values	No Values	No Values	No Values
1164	seq=translation; coord=6:124736591..124745000:-1; parent_transcript=GRMZM2G148709_T01; parent_gene=GRMZM2G148709	GRMZM2G148709_P01 (+3)	73 kDa	Ref	-0.1	-0.4	-0.5	-0.5	-0.1	-0.6	-0.4	-0.8
1165	seq=translation; coord=7:160059330..160068443:-1; parent_transcript=GRMZM5G887631_T01; parent_gene=GRMZM5G887631	GRMZM5G887631_P01	79 kDa	Ref	0	-0.6	0	0.5	-1.1	0.3	-0.2	-0.8
1166	seq=translation; coord=9:87461648..87475465:-1; parent_transcript=GRMZM2G047564_T03; parent_gene=GRMZM2G047564	GRMZM2G047564_P03 (+1)	46 kDa	Ref	-0.4	-0.6	-0.4	-0.3	0.1	-0.2	-0.3	0.3
1167	seq=translation; coord=5:211206297..211209456:-1; parent_transcript=GRMZM2G151440_T01; parent_gene=GRMZM2G151440	GRMZM2G151440_P01 (+1)	42 kDa	Ref	-0.3	-0.1	-0.2	-0.2	-1.7	-1.5	-1.5	-1.6
1168	seq=translation; coord=4:230556470..230569433:1; parent_transcript=GRMZM2G038195_T01; parent_gene=GRMZM2G038195	GRMZM2G038195_P01 (+3)	35 kDa	Ref	-0.3	-0.2	-0.3	-0.2	-0.1	-0.3	-0.6	-1
1169	seq=translation; coord=6:133205650..133225934:-1; parent_transcript=GRMZM2G076006_T03; parent_gene=GRMZM2G076006	GRMZM2G076006_P03	38 kDa	Ref	-0.3	-0.1	0	0.4	-0.7	-0.5	0	0.5

1170	seq=translation; coord=6:81686909..81693610:1; parent_transcript=GRMZM2G103266_T01; parent_gene=GRMZM2G103266	GRMZM2G103266_P01 (+2)	78 kDa	Ref	0.6	0.8	1	0.8	-1.5	-1.2	-0.7	-0.5
1171	seq=translation; coord=9:59939835..59941693:-1; parent_transcript=GRMZM2G471357_T01; parent_gene=GRMZM2G471357	GRMZM2G471357_P01 (+1)	33 kDa	Ref	Value Missing	0.6	Value Missing	0.2	-3.6	-3.7	-2.5	-1.4
1172	seq=translation; coord=7:168913721..168922316:-1; parent_transcript=GRMZM2G026991_T01; parent_gene=GRMZM2G026991	GRMZM2G026991_P01	249 kDa	Ref	0.5	0	-0.4	-0.3	0.8	-0.5	-0.9	-1.4
1173	seq=translation; coord=2:179742429..179748543:1; parent_transcript=GRMZM2G105772_T01; parent_gene=GRMZM2G105772	GRMZM2G105772_P01 (+1)	42 kDa	Ref	1.3	1.2	0.9	1.2	-0.5	Reference Missing	-0.3	Reference Missing
1174	seq=translation; coord=3:225222066..225226693:-1; parent_transcript=GRMZM2G049866_T01; parent_gene=GRMZM2G049866	GRMZM2G049866_P01 (+1)	45 kDa	Ref	No Values	No Values	No Values	No Values	-0.3	0.1	-1.2	-1.2
1175	seq=translation; coord=5:194043178..194045076:1; parent_transcript=GRMZM2G013821_T01; parent_gene=GRMZM2G013821	GRMZM2G013821_P01 (+1)	15 kDa	Ref	No Values	No Values	No Values	No Values	-0.3	-1.9	-1.8	-2
1176	seq=translation; coord=1:292988864..292997657:1; parent_transcript=GRMZM2G155580_T01; parent_gene=GRMZM2G155580	GRMZM2G155580_P01 (+2)	68 kDa	Ref	0.6	0	-0.5	-0.3	-2.2	-2.2	-2.3	-2.5
1177	seq=translation; coord=6:140312649..140313648:-1; parent_transcript=GRMZM2G162659_T01; parent_gene=GRMZM2G162659	GRMZM2G162659_P01	12 kDa	Ref	0	-0.1	-0.3	0.9	-1.3	-1.1	Reference Missing	-0.6
1178	seq=translation; coord=5:157517807..157521679:-1; parent_transcript=GRMZM2G057642_T02; parent_gene=GRMZM2G057642	GRMZM2G057642_P02 (+1)	38 kDa	Ref	1.1	0.4	0.1	0.1	1.4	-0.1	-0.2	-0.7
1179	seq=translation; coord=3:1481217..1482997:-1; parent_transcript=GRMZM2G087103_T02; parent_gene=GRMZM2G087103	GRMZM2G087103_P02 (+2)	36 kDa	Ref	-2	-0.3	-1.6	-1.6	-1.6	0.9	1.2	0.4
1180	seq=translation; coord=1:74628405..74631787:1; parent_transcript=GRMZM2G130121_T01; parent_gene=GRMZM2G130121	GRMZM2G130121_P01	74 kDa	Ref	Reference Missing	No Values	Reference Missing	Reference Missing	Reference Missing	Reference Missing	Reference Missing	-2.3
1181	seq=translation; coord=9:150085751..150089143:1; parent_transcript=GRMZM2G100120_T01; parent_gene=GRMZM2G100120	GRMZM2G100120_P01 (+5)	26 kDa	Ref	0.3	0.2	0	-0.1	0.1	-0.9	-0.9	-1.1
1182	seq=translation; coord=8:157386210..157395665:-1; parent_transcript=GRMZM2G133926_T01; parent_gene=GRMZM2G133926	GRMZM2G133926_P01	47 kDa	Ref	-1.2	-0.4	-0.6	-0.6	-0.2	-1.3	-1.7	-1.8

1183	seq=translation; coord=3:39476674..39479631:1; parent_transcript=GRMZM2G132465_T01; parent_gene=GRMZM2G132465	GRMZM2G132465_P01 (+4)	47 kDa	Ref	-0.2	-0.6	-0.7	-0.8	-3.6	-4.3	-4	-4.4
1184	seq=translation; coord=3:182713971..182717776:-1; parent_transcript=GRMZM2G504401_T01; parent_gene=GRMZM2G504401	GRMZM2G504401_P01	27 kDa	Ref	-0.7	-0.4	-0.4	-0.8	-2.2	-1.2	-0.8	-0.8
1185	seq=translation; coord=7:165748301..165751201:-1; parent_transcript=GRMZM2G053999_T01; parent_gene=GRMZM2G053999	GRMZM2G053999_P01	36 kDa	Ref	-0.1	-0.1	-0.2	0.3	2.4	0.9	0.9	0.8
1186	seq=translation; coord=1:273298297..273299437:-1; parent_transcript=GRMZM2G481194_T01; parent_gene=GRMZM2G481194	GRMZM2G481194_P01	21 kDa	Ref	-0.1	-0.4	-0.8	-1.1	0.6	0.6	0.6	0.5
1187	seq=translation; coord=4:162677644..162687691:1; parent_transcript=GRMZM2G020775_T01; parent_gene=GRMZM2G020775	GRMZM2G020775_P01	47 kDa	Ref	-1.2	-0.9	-1.1	-1	1.4	0.8	1	0.3
1188	seq=translation; coord=1:269035042..269037767:1; parent_transcript=GRMZM2G047055_T01; parent_gene=GRMZM2G047055	GRMZM2G047055_P01 (+4)	42 kDa	Ref	0.4	-0.4	-1.3	-0.8	2.1	0.7	-0.1	-0.6
1189	seq=translation; coord=3:186233588..186237469:1; parent_transcript=GRMZM2G133213_T01; parent_gene=GRMZM2G133213	GRMZM2G133213_P01	31 kDa	Ref	1.1	1.1	1.1	2.3	3.9	1.8	2.3	1.6
1190	seq=translation; coord=4:197674970..197676500:1; parent_transcript=GRMZM2G301908_T01; parent_gene=GRMZM2G301908	GRMZM2G301908_P01	27 kDa	Ref	0	-0.9	-1.4	-1.6	2.6	2.5	2.6	2.4
1191	seq=translation; coord=9:4832748..4841265:1; parent_transcript=GRMZM2G124288_T01; parent_gene=GRMZM2G124288	GRMZM2G124288_P01 (+1)	68 kDa	Ref	1.3	0.9	1	0.7	3.7	2.4	2.7	2.4
1192	seq=translation; coord=7:52251311..52252353:1; parent_transcript=GRMZM2G027198_T01; parent_gene=GRMZM2G027198	GRMZM2G027198_P01	18 kDa	Ref	-0.1	0.4	1.9	3	0.5	0	0.8	0.4
1193	seq=translation; coord=6:128560617..128567071:1; parent_transcript=GRMZM2G027723_T01; parent_gene=GRMZM2G027723	GRMZM2G027723_P01 (+2)	121 kDa	Ref	-1.7	-1.6	-2.7	-3.1	0.9	Value Missing	Value Missing	Value Missing
1194	seq=translation; coord=7:14459963..14466085:-1; parent_transcript=GRMZM2G104325_T01; parent_gene=GRMZM2G104325	GRMZM2G104325_P01	105 kDa	Ref	1.6	1.2	Reference Missing	0.6	No Values	No Values	No Values	No Values
1195	seq=translation; coord=7:158136732..158153170:1; parent_transcript=GRMZM2G107654_T01; parent_gene=GRMZM2G107654	GRMZM2G107654_P01	53 kDa	Ref	0.1	-0.4	-0.2	-0.3	-0.3	-0.6	-0.5	-0.5

1196	seq=translation; coord=8:157237785..157240528:-1; parent_transcript=GRMZM2G446960_T01; parent_gene=GRMZM2G446960	GRMZM2G446960_P01	20 kDa	Ref	1.2	0.9	0.4	0.4	No Values	No Values	No Values	No Values
1197	seq=translation; coord=5:31131578..31135872:-1; parent_transcript=GRMZM2G110201_T01; parent_gene=GRMZM2G110201	GRMZM2G110201_P01	48 kDa	Ref	-0.3	-0.5	-0.5	-0.8	No Values	No Values	No Values	No Values
1198	seq=translation; coord=6:2438893..2441433:1; parent_transcript=GRMZM2G024303_T01; parent_gene=GRMZM2G024303	GRMZM2G024303_P01	44 kDa	Ref	0.4	0.5	0.3	1.2	No Values	No Values	No Values	No Values
1199	seq=translation; coord=5:98993016..98997371:-1; parent_transcript=GRMZM5G844562_T01; parent_gene=GRMZM5G844562	GRMZM5G844562_P01 (+1)	39 kDa	Ref	-0.2	0.6	0.7	1.5	No Values	No Values	No Values	No Values
1200	seq=translation; coord=2:23469563..23473424:1; parent_transcript=GRMZM2G177923_T01; parent_gene=GRMZM2G177923	GRMZM2G177923_P01	83 kDa	Ref	-0.7	-0.5	-0.4	0.3	No Values	No Values	No Values	No Values
1201	seq=translation; coord=10:123811073..123815007:1; parent_transcript=GRMZM2G064159_T01; parent_gene=GRMZM2G064159	GRMZM2G064159_P01	42 kDa	Ref	0.1	-0.2	0.2	0.1	No Values	No Values	No Values	No Values
1202	seq=translation; coord=3:168695539..168839001:1; parent_transcript=GRMZM2G156861_T03; parent_gene=GRMZM2G156861	GRMZM2G156861_P03	98 kDa	Ref	0.7	Reference Missing	Reference Missing	-0.1	No Values	No Values	No Values	No Values
1203	seq=translation; coord=6:143138266..143147623:1; parent_transcript=GRMZM2G305851_T01; parent_gene=GRMZM2G305851	GRMZM2G305851_P01	41 kDa	Ref	1.7	1.6	1	1.7	No Values	No Values	No Values	No Values
1204	seq=translation; coord=4:18573473..18575395:-1; parent_transcript=GRMZM2G440208_T01; parent_gene=GRMZM2G440208	GRMZM2G440208_P01	54 kDa	Ref	No Values	No Values	No Values	No Values	1	0.7	0.4	0.4
1205	seq=translation; coord=7:143292623..143296263:1; parent_transcript=GRMZM2G109405_T01; parent_gene=GRMZM2G109405	GRMZM2G109405_P01 (+2)	43 kDa	Ref	0.2	0.3	0.3	0.6	No Values	No Values	No Values	No Values
1206	seq=translation; coord=9:89703050..89707450:1; parent_transcript=GRMZM2G442551_T01; parent_gene=GRMZM2G442551	GRMZM2G442551_P01 (+1)	91 kDa	Ref	0.4	0.3	-0.1	0.3	No Values	No Values	No Values	No Values
1207	seq=translation; coord=8:173066444..173072416:1; parent_transcript=GRMZM2G424857_T01; parent_gene=GRMZM2G424857	GRMZM2G424857_P01	50 kDa	Ref	No Values	No Values	No Values	No Values	-0.4	0	Reference Missing	Reference Missing
1208	seq=translation; coord=7:126805106..126806657:1; parent_transcript=GRMZM5G826321_T01; parent_gene=GRMZM5G826321	GRMZM5G826321_P01	34 kDa	Ref	-0.8	-1	-0.9	-1.2	No Values	No Values	No Values	No Values

1209	seq=translation; coord=7:131436732..131438757:1; parent_transcript=GRMZM2G018108_T01; parent_gene=GRMZM2G018108	GRMZM2G018108_P01	50 kDa	Ref	0.5	1.5	2.2	2.9	No Values	No Values	No Values	No Values
1210	seq=translation; coord=5:201664982..201667877:1; parent_transcript=GRMZM2G170602_T01; parent_gene=GRMZM2G170602	GRMZM2G170602_P01	42 kDa	Ref	0.2	0	0.7	2.4	No Values	No Values	No Values	No Values
1211	seq=translation; coord=5:3285681..3289943:1; parent_transcript=GRMZM2G017532_T01; parent_gene=GRMZM2G017532	GRMZM2G017532_P01	21 kDa	Ref	No Values	No Values	No Values	No Values	-0.2	1.1	0.9	2.7
1212	seq=translation; coord=4:180245812..180247211:1; parent_transcript=GRMZM2G448883_T01; parent_gene=GRMZM2G448883	GRMZM2G448883_P01	33 kDa	Ref	2	2.2	2.3	3	No Values	No Values	No Values	No Values
1213	seq=translation; coord=4:238055009..238057832:-1; parent_transcript=AC233922.1_FGT005; parent_gene=AC233922.1_FG005	AC233922.1_FGP005	40 kDa	Ref	No Values	No Values	No Values	No Values	0.6	0.6	1.6	0.7
1214	seq=translation; coord=8:17966967..17971201:-1; parent_transcript=GRMZM2G175171_T01; parent_gene=GRMZM2G175171	GRMZM2G175171_P01	60 kDa	Ref	0.3	-0.1	0.4	1	No Values	No Values	No Values	No Values
1215	seq=translation; coord=1:228560968..228564785:1; parent_transcript=GRMZM5G888696_T02; parent_gene=GRMZM5G888696	GRMZM5G888696_P02	68 kDa	Ref	No Values	No Values	No Values	No Values	0.5	0.7	0.3	-0.2
1216	seq=translation; coord=6:161451718..161452634:-1; parent_transcript=GRMZM2G090245_T01; parent_gene=GRMZM2G090245	GRMZM2G090245_P01	23 kDa	Ref	Reference Missing	0.4	0.5	Reference Missing	No Values	No Values	No Values	No Values
1217	seq=translation; coord=2:43428306..43433991:1; parent_transcript=GRMZM2G031028_T02; parent_gene=GRMZM2G031028	GRMZM2G031028_P02	34 kDa	Ref	-0.8	-0.7	-0.4	-0.5	No Values	No Values	No Values	No Values
1218	seq=translation; coord=3:206531676..206533642:1; parent_transcript=GRMZM2G050570_T01; parent_gene=GRMZM2G050570	GRMZM2G050570_P01	58 kDa	Ref	-0.1	-0.3	-0.7	-0.7	0.5	0.5	0.4	0.4
1219	seq=translation; coord=6:115289930..115298874:1; parent_transcript=GRMZM2G157462_T01; parent_gene=GRMZM2G157462	GRMZM2G157462_P01	100 kDa	Ref	0.6	0.5	0.5	0.7	0.9	0.4	0.2	-0.2
1220	seq=translation; coord=6:127249169..127252532:1; parent_transcript=GRMZM2G018020_T01; parent_gene=GRMZM2G018020	GRMZM2G018020_P01	28 kDa	Ref	0.5	0.3	-0.4	-0.5	1.5	-0.3	-1.6	-1.2
1221	seq=translation; coord=6:126779719..126784379:1; parent_transcript=GRMZM2G159369_T01; parent_gene=GRMZM2G159369	GRMZM2G159369_P01	40 kDa	Ref	0	0.2	0	0.1	-1.3	-2.1	-1.9	-0.3

1222	seq=translation; coord=1:218981641..218987465:-1; parent_transcript=GRMZM5G895175_T01; parent_gene=GRMZM5G895175	GRMZM5G895175_P01	85 kDa	Ref	1.2	0.3	0.4	-0.3	Reference Missing	Reference Missing	Reference Missing	Reference Missing
1223	seq=translation; coord=7:10365682..10368158:1; parent_transcript=GRMZM2G018074_T01; parent_gene=GRMZM2G018074	GRMZM2G018074_P01	78 kDa	Ref	0.7	0.8	0.9	0.3	3.6	3.6	4.3	3.4
1224	seq=translation; coord=9:12323292..12327304:-1; parent_transcript=GRMZM2G151923_T01; parent_gene=GRMZM2G151923	GRMZM2G151923_P01 (+4)	21 kDa	Ref	0.7	0.6	0.3	0.2	0.5	-0.6	-0.9	-1.4
1225	seq=translation; coord=8:171187220..171190525:-1; parent_transcript=GRMZM2G028346_T01; parent_gene=GRMZM2G028346	GRMZM2G028346_P01 (+3)	25 kDa	Ref	No Values	No Values	No Values	No Values	0.1	0	-0.2	-0.5
1226	seq=translation; coord=9:7578400..7585814:1; parent_transcript=GRMZM2G120302_T01; parent_gene=GRMZM2G120302	GRMZM2G120302_P01	79 kDa	Ref	0.4	0	0.4	0.5	-2.5	-2.5	-2.9	-2.6
1227	seq=translation; coord=UNKNOWN:5634502..5650723:1; parent_transcript=GRMZM2G011070_T01; parent_gene=GRMZM2G011070	GRMZM2G011070_P01	135 kDa	Ref	0.4	0.4	0	0.1	1.1	1.4	1	0.9
1228	seq=translation; coord=8:156372419..156374495:-1; parent_transcript=GRMZM2G073622_T01; parent_gene=GRMZM2G073622	GRMZM2G073622_P01 (+2)	24 kDa	Ref	-1.9	-1.5	-1.5	-2	0.5	0.6	0.1	-0.1
1229	seq=translation; coord=4:175739965..175751055:-1; parent_transcript=GRMZM2G181362_T01; parent_gene=GRMZM2G181362	GRMZM2G181362_P01 (+1)	116 kDa	Ref	-1.3	-0.4	-1.2	-0.4	-1.5	-0.6	-0.9	-1.1
1230	seq=translation; coord=2:173380725..173391388:-1; parent_transcript=GRMZM2G106213_T01; parent_gene=GRMZM2G106213	GRMZM2G106213_P01 (+1)	56 kDa	Ref	-0.2	-0.8	-1.2	-1.1	0.1	1.2	1.2	0.9
1231	seq=translation; coord=6:148703816..148706201:1; parent_transcript=AC235543.1_FGT003; parent_gene=AC235543.1_FG003	AC235543.1_FGP003	19 kDa	Ref	1.3	1	0.8	0.5	2.5	2.7	2.6	1.9
1232	seq=translation; coord=4:223661025..223669104:-1; parent_transcript=GRMZM2G068489_T01; parent_gene=GRMZM2G068489	GRMZM2G068489_P01 (+2)	21 kDa	Ref	1	-0.5	0.1	0	No Values	Reference Missing	Reference Missing	Reference Missing
1233	seq=translation; coord=4:214239084..214241779:1; parent_transcript=GRMZM2G126190_T01; parent_gene=GRMZM2G126190	GRMZM2G126190_P01 (+2)	42 kDa	Ref	0.4	-0.3	-0.3	-0.2	No Values	No Values	No Values	No Values
1234	seq=translation; coord=2:25300018..25310642:1; parent_transcript=GRMZM5G827171_T01; parent_gene=GRMZM5G827171	GRMZM5G827171_P01 (+2)	48 kDa	Ref	0.8	0.5	0.3	-0.1	2	0.9	0.9	0.7

1235	seq=translation; coord=7:33992878..33998025:-1; parent_transcript=GRMZM2G051771_T01; parent_gene=GRMZM2G051771	GRMZM2G051771_P01 (+1)	31 kDa	Ref	-0.5	-0.5	-0.5	0	1.7	2	2.1	3.1
1236	seq=translation; coord=2:2545896..2547904:1; parent_transcript=GRMZM2G010257_T01; parent_gene=GRMZM2G010257	GRMZM2G010257_P01 (+14)	11 kDa	Ref	0.3	-0.9	-0.1	0.6	-1	-2	-3.5	-3
1237	seq=translation; coord=8:70525339..70528822:1; parent_transcript=GRMZM2G413652_T01; parent_gene=GRMZM2G413652	GRMZM2G413652_P01 (+1)	30 kDa	Ref	No Values	No Values	No Values	No Values	-1.4	-1.3	-1.6	-1.5
1238	seq=translation; coord=1:289192462..289217741:1; parent_transcript=GRMZM5G825524_T01; parent_gene=GRMZM5G825524	GRMZM5G825524_P01 (+2)	91 kDa	Ref	-0.5	-0.7	-0.5	-0.4	2.8	1.2	Value Missing	1.4
1239	seq=translation; coord=1:2038186..2040079:1; parent_transcript=GRMZM2G164696_T01; parent_gene=GRMZM2G164696	GRMZM2G164696_P01 (+1)	50 kDa	Ref	1.3	-0.3	-0.2	-0.9	-0.2	-2.7	-3.1	-3.9
1240	seq=translation; coord=6:160675824..160684398:1; parent_transcript=GRMZM2G587327_T01; parent_gene=GRMZM2G587327	GRMZM2G587327_P01	81 kDa	Ref	No Values	No Values	No Values	No Values	0.9	-0.8	-1.2	-1.6
1241	seq=translation; coord=9:139381433..139385689:-1; parent_transcript=GRMZM2G166767_T01; parent_gene=GRMZM2G166767	GRMZM2G166767_P01 (+1)	76 kDa	Ref	0	-0.6	-0.8	-0.5	No Values	No Values	No Values	No Values
1242	seq=translation; coord=5:181396172..181399390:-1; parent_transcript=GRMZM2G031496_T01; parent_gene=GRMZM2G031496	GRMZM2G031496_P01 (+1)	37 kDa	Ref	0.3	-0.4	0.9	0	No Values	No Values	No Values	No Values
1243	seq=translation; coord=1:268558195..268564501:-1; parent_transcript=GRMZM2G000622_T01; parent_gene=GRMZM2G000622	GRMZM2G000622_P01	155 kDa	Ref	No Values	No Values	No Values	No Values	0.8	-0.1	-0.2	-0.4
1244	seq=translation; coord=4:41369510..41378299:1; parent_transcript=GRMZM2G138060_T01; parent_gene=GRMZM2G138060	GRMZM2G138060_P01	88 kDa	Ref	No Values	No Values	No Values	No Values	0.5	0.7	0.5	-0.1
1245	seq=translation; coord=9:119710257..119715669:-1; parent_transcript=GRMZM2G151387_T01; parent_gene=GRMZM2G151387	GRMZM2G151387_P01 (+1)	80 kDa	Ref	No Values	No Values	No Values	No Values	0.6	0	-0.2	-0.2
1246	seq=translation; coord=5:74640062..74641850:-1; parent_transcript=GRMZM2G017249_T01; parent_gene=GRMZM2G017249	GRMZM2G017249_P01	64 kDa	Ref	0.9	0.5	0.4	0.9	No Values	No Values	No Values	No Values
1247	seq=translation; coord=4:5455601..5457606:1; parent_transcript=GRMZM2G154523_T01; parent_gene=GRMZM2G154523	GRMZM2G154523_P01	44 kDa	Ref	1.2	2.8	2.1	2.4	No Values	No Values	No Values	No Values

1248	seq=translation; coord=2:3805095..3807781:-1; parent_transcript=GRMZM2G173341_T01; parent_gene=GRMZM2G173341	GRMZM2G173341_P01 (+2)	39 kDa	Ref	-1.3	-1.1	-1.4	-0.9	No Values	No Values	No Values	No Values
1249	seq=translation; coord=3:213896741..213905427:1; parent_transcript=GRMZM2G466833_T01; parent_gene=GRMZM2G466833	GRMZM2G466833_P01	44 kDa	Ref	No Values	No Values	No Values	No Values	0.1	-0.1	-0.2	0.3
1250	seq=translation; coord=10:19475272..19481312:1; parent_transcript=GRMZM2G181227_T01; parent_gene=GRMZM2G181227	GRMZM2G181227_P01	43 kDa	Ref	-0.9	-0.5	1.1	1.7	No Values	No Values	No Values	No Values
1251	seq=translation; coord=4:42919325..42920272:1; parent_transcript=GRMZM2G154578_T01; parent_gene=GRMZM2G154578	GRMZM2G154578_P01	25 kDa	Ref	-0.2	0	-0.9	-1	No Values	No Values	No Values	No Values
1252	seq=translation; coord=4:81250432..81251801:-1; parent_transcript=GRMZM2G004878_T01; parent_gene=GRMZM2G004878	GRMZM2G004878_P01	19 kDa	Ref	2.7	0.9	0.1	-0.1	No Values	No Values	No Values	No Values
1253	seq=translation; coord=6:142082329..142086400:1; parent_transcript=AC215201.3_FGT005; parent_gene=AC215201.3_FG005	AC215201.3_FGP005	63 kDa	Ref	No Values	No Values	No Values	No Values	-0.5	0.8	1.2	1.3
1254	seq=translation; coord=1:34386070..34400519:-1; parent_transcript=GRMZM2G102483_T02; parent_gene=GRMZM2G102483	GRMZM2G102483_P02 (+1)	42 kDa	Ref	No Values	No Values	No Values	No Values	Reference Missing	-0.1	Reference Missing	Reference Missing
1255	seq=translation; coord=4:237941870..237948667:1; parent_transcript=GRMZM2G139341_T01; parent_gene=GRMZM2G139341	GRMZM2G139341_P01 (+1)	73 kDa	Ref	No Values	No Values	No Values	No Values	2.2	1.4	1.3	0.2
1256	seq=translation; coord=1:285124791..285129572:-1; parent_transcript=GRMZM2G058584_T01; parent_gene=GRMZM2G058584	GRMZM2G058584_P01 (+1)	51 kDa	Ref	No Values	No Values	No Values	No Values	1.4	1.2	2.2	2.1
1257	seq=translation; coord=6:154130608..154132782:-1; parent_transcript=GRMZM2G361064_T01; parent_gene=GRMZM2G361064	GRMZM2G361064_P01	26 kDa	Ref	0	0.4	0.3	0.6	No Values	No Values	No Values	No Values
1258	seq=translation; coord=6:39088029..39088716:1; parent_transcript=GRMZM2G012928_T01; parent_gene=GRMZM2G012928	GRMZM2G012928_P01	8 kDa	Ref	No Values	No Values	No Values	No Values	0	Reference Missing	Reference Missing	Reference Missing
1259	seq=translation; coord=4:12818434..12823201:-1; parent_transcript=GRMZM2G139441_T01; parent_gene=GRMZM2G139441	GRMZM2G139441_P01 (+1)	45 kDa	Ref	1	0.3	0.1	0.1	-1.4	-1.3	-2.8	-2.2
1260	seq=translation; coord=6:114177100..114187293:-1; parent_transcript=GRMZM2G010960_T01; parent_gene=GRMZM2G010960	GRMZM2G010960_P01	157 kDa	Ref	1.4	2	1.8	1.4	1.6	2.2	2.5	1.7

1261	seq=translation; coord=8:48994683..48999789:-1; parent_transcript=GRMZM2G083836_T01; parent_gene=GRMZM2G083836	GRMZM2G083836_P01 (+1)	63 kDa	Ref	No Values	No Values	No Values	No Values	0.4	-0.1	0.1	-0.6
1262	seq=translation; coord=2:176238563..176244250:1; parent_transcript=GRMZM2G098076_T01; parent_gene=GRMZM2G098076	GRMZM2G098076_P01 (+1)	69 kDa	Ref	No Values	No Values	No Values	No Values	1.1	0.1	-0.3	0.3
1263	seq=translation; coord=1:234254283..234259941:1; parent_transcript=GRMZM2G445905_T01; parent_gene=GRMZM2G445905	GRMZM2G445905_P01 (+1)	24 kDa	Ref	Value Missing	0.6	Value Missing	0.5	Reference Missing	Reference Missing	Reference Missing	Reference Missing
1264	seq=translation; coord=3:217231435..217244458:-1; parent_transcript=AC198725.4_FGT007; parent_gene=AC198725.4_FG007	AC198725.4_FGP007	114 kDa	Ref	No Values	No Values	No Values	Reference Missing	-3.3	-3.5	-4.8	-4.3
1265	seq=translation; coord=8:138088772..138094325:-1; parent_transcript=GRMZM2G018901_T01; parent_gene=GRMZM2G018901	GRMZM2G018901_P01 (+1)	20 kDa	Ref	-1.1	-1.1	-1.4	-0.6	-0.8	-1	-0.9	-0.8
1266	seq=translation; coord=8:15548387..15551947:-1; parent_transcript=GRMZM2G038821_T01; parent_gene=GRMZM2G038821	GRMZM2G038821_P01	69 kDa	Ref	No Values	No Values	No Values	Reference Missing	-7.1	-6.6	-5.9	-5.9
1267	seq=translation; coord=8:14541175..14542451:-1; parent_transcript=GRMZM2G052869_T02; parent_gene=GRMZM2G052869	GRMZM2G052869_P02 (+1)	6 kDa	Ref	0.1	1.7	0.8	-0.7	-0.4	-0.6	0.5	-0.7
1268	seq=translation; coord=2:208530771..208535295:-1; parent_transcript=GRMZM2G090274_T01; parent_gene=GRMZM2G090274	GRMZM2G090274_P01 (+1)	49 kDa	Ref	0.4	-0.2	-0.1	-0.1	0.8	1	0	-0.4
1269	seq=translation; coord=4:187295953..187301881:-1; parent_transcript=GRMZM2G055538_T01; parent_gene=GRMZM2G055538	GRMZM2G055538_P01 (+2)	21 kDa	Ref	0.8	0.3	0.3	0.1	-0.3	-1.7	-1.8	-1.6
1270	seq=translation; coord=1:250554600..250557666:-1; parent_transcript=GRMZM2G020142_T01; parent_gene=GRMZM2G020142	GRMZM2G020142_P01 (+1)	41 kDa	Ref	0.1	0.2	-0.9	-0.1	0.4	0.3	-1.2	-2
1271	seq=translation; coord=5:213944858..213947602:-1; parent_transcript=GRMZM2G469111_T01; parent_gene=GRMZM2G469111	GRMZM2G469111_P01	74 kDa	Ref	1.3	1.3	0.4	0.9	1.7	-0.5	-1	-2.3
1272	seq=translation; coord=5:76210306..76210828:1; parent_transcript=AC192244.3_FGT001; parent_gene=AC192244.3_FG001	AC192244.3_FGP001	13 kDa	Ref	-0.1	-0.2	0.5	0.1	-0.3	0.1	0.4	0.1
1273	seq=translation; coord=6:87790147..87797085:1; parent_transcript=GRMZM2G156099_T01; parent_gene=GRMZM2G156099	GRMZM2G156099_P01 (+2)	79 kDa	Ref	0.3	0.3	-0.4	-0.1	1.7	0.6	1.2	1

1274	seq=translation; coord=6:163428972..163435944:-1; parent_transcript=GRMZM2G149717_T01; parent_gene=GRMZM2G149717	GRMZM2G149717_P01 (+1)	73 kDa	Ref	0.8	0.4	0.4	0.4	5.2	6.2	5.9	5.3
1275	seq=translation; coord=4:53963570..53966108:-1; parent_transcript=GRMZM2G123234_T01; parent_gene=GRMZM2G123234	GRMZM2G123234_P01	31 kDa	Ref	No Values	No Values	No Values	No Values	-1	-1.7	-1	-2
1276	seq=translation; coord=2:209110460..209113041:-1; parent_transcript=GRMZM2G378106_T01; parent_gene=GRMZM2G378106	GRMZM2G378106_P01 (+1)	67 kDa	Ref	-0.9	-0.6	-0.2	0.1	No Values	No Values	No Values	No Values
1277	seq=translation; coord=10:125125070..125130397:1; parent_transcript=GRMZM2G065292_T01; parent_gene=GRMZM2G065292	GRMZM2G065292_P01	82 kDa	Ref	No Values	No Values	No Values	No Values	0.3	0.6	0.6	0.5
1278	seq=translation; coord=6:112003443..112007827:-1; parent_transcript=GRMZM2G073700_T01; parent_gene=GRMZM2G073700	GRMZM2G073700_P01	35 kDa	Ref	No Values	No Values	No Values	No Values	1.2	0.8	0.8	0.9
1279	seq=translation; coord=7:162255747..162259720:1; parent_transcript=GRMZM2G167932_T01; parent_gene=GRMZM2G167932	GRMZM2G167932_P01 (+1)	49 kDa	Ref	No Values	No Values	No Values	No Values	-0.9	-1.7	-1.6	-1.6
1280	seq=translation; coord=4:162903711..162910128:1; parent_transcript=GRMZM2G453424_T03; parent_gene=GRMZM2G453424	GRMZM2G453424_P03 (+4)	23 kDa	Ref	No Values	No Values	No Values	No Values	0.2	-0.2	-0.8	-0.7
1281	seq=translation; coord=5:183319186..183326822:-1; parent_transcript=GRMZM2G014750_T01; parent_gene=GRMZM2G014750	GRMZM2G014750_P01 (+1)	52 kDa	Ref	No Values	No Values	No Values	No Values	0.4	-0.5	-0.7	-0.9
1282	seq=translation; coord=9:131582315..131589080:-1; parent_transcript=GRMZM2G006178_T01; parent_gene=GRMZM2G006178	GRMZM2G006178_P01 (+3)	68 kDa	Ref	No Values	No Values	No Values	No Values	0.1	0.4	0.4	-0.2
1283	seq=translation; coord=5:18992634..18995244:-1; parent_transcript=GRMZM2G045192_T01; parent_gene=GRMZM2G045192	GRMZM2G045192_P01 (+2)	27 kDa	Ref	-0.7	-0.7	-0.1	-0.6	No Values	No Values	No Values	No Values
1284	seq=translation; coord=9:30842366..30844467:1; parent_transcript=GRMZM2G060163_T01; parent_gene=GRMZM2G060163	GRMZM2G060163_P01	39 kDa	Ref	No Values	No Values	No Values	No Values	3.2	-0.6	0.1	-1
1285	seq=translation; coord=6:149942002..149944565:-1; parent_transcript=GRMZM2G161534_T01; parent_gene=GRMZM2G161534	GRMZM2G161534_P01	39 kDa	Ref	No Values	No Values	No Values	No Values	Reference Missing	Reference Missing	0.9	1.1
1286	seq=translation; coord=1:295450010..295457140:-1; parent_transcript=GRMZM2G458549_T01; parent_gene=GRMZM2G458549	GRMZM2G458549_P01 (+1)	64 kDa	Ref	-0.3	-0.4	-0.4	0.1	No Values	No Values	No Values	No Values

1287	seq=translation; coord=2:28117445..28120590:1; parent_transcript=GRMZM2G441347_T01; parent_gene=GRMZM2G441347	GRMZM2G441347_P01	76 kDa	Ref	0.2	0.4	0.1	0	No Values	No Values	No Values	No Values
1288	seq=translation; coord=6:164983203..164994562:1; parent_transcript=GRMZM2G101515_T01; parent_gene=GRMZM2G101515	GRMZM2G101515_P01	91 kDa	Ref	No Values	No Values	No Values	No Values	1	-0.1	-0.8	-1.3
1289	seq=translation; coord=8:173177746..173180822:1; parent_transcript=GRMZM2G074028_T01; parent_gene=GRMZM2G074028	GRMZM2G074028_P01 (+4)	16 kDa	Ref	1.4	1.2	1.2	1.3	No Values	No Values	No Values	No Values
1290	seq=translation; coord=2:22504092..22513426:-1; parent_transcript=GRMZM2G070863_T01; parent_gene=GRMZM2G070863	GRMZM2G070863_P01	65 kDa	Ref	No Values	No Values	No Values	No Values	1	0.4	0.4	2.3
1291	seq=translation; coord=7:13851997..13853595:1; parent_transcript=GRMZM2G108076_T01; parent_gene=GRMZM2G108076	GRMZM2G108076_P01	12 kDa	Ref	0.2	0.4	0.4	0.9	No Values	No Values	No Values	No Values
1292	seq=translation; coord=3:36465309..36469955:-1; parent_transcript=GRMZM2G038606_T01; parent_gene=GRMZM2G038606	GRMZM2G038606_P01 (+3)	19 kDa	Ref	1.6	1.1	0.9	0	No Values	No Values	No Values	No Values
1293	seq=translation; coord=3:198588979..198593726:-1; parent_transcript=GRMZM2G042818_T01; parent_gene=GRMZM2G042818	GRMZM2G042818_P01	43 kDa	Ref	-0.3	0.2	-0.1	0.2	No Values	No Values	No Values	No Values
1294	seq=translation; coord=2:196329725..196336472:-1; parent_transcript=GRMZM2G050193_T01; parent_gene=GRMZM2G050193	GRMZM2G050193_P01 (+1)	61 kDa	Ref	1	1	0.7	0.9	No Values	No Values	No Values	No Values
1295	seq=translation; coord=4:176500156..176502218:1; parent_transcript=GRMZM2G091189_T01; parent_gene=GRMZM2G091189	GRMZM2G091189_P01	26 kDa	Ref	No Values	No Values	No Values	No Values	1	0	-0.8	-0.7
1296	seq=translation; coord=1:260939345..260957939:1; parent_transcript=GRMZM2G104542_T01; parent_gene=GRMZM2G104542	GRMZM2G104542_P01	64 kDa	Ref	Reference Missing	Reference Missing	0.2	Reference Missing	No Values	No Values	No Values	No Values
1297	seq=translation; coord=5:216598113..216599835:1; parent_transcript=GRMZM2G145133_T01; parent_gene=GRMZM2G145133	GRMZM2G145133_P01 (+1)	48 kDa	Ref	-1	-0.6	-0.9	-0.8	No Values	No Values	No Values	No Values
1298	seq=translation; coord=3:39292213..39295100:1; parent_transcript=GRMZM2G145449_T01; parent_gene=GRMZM2G145449	GRMZM2G145449_P01	21 kDa	Ref	-2.5	-3.1	-2.7	-2.2	No Values	No Values	No Values	No Values
1299	seq=translation; coord=9:150649384..150656114:-1; parent_transcript=GRMZM2G172523_T01; parent_gene=GRMZM2G172523	GRMZM2G172523_P01 (+2)	60 kDa	Ref	No Values	No Values	No Values	No Values	0.1	0.5	0.7	0.9

1300	seq=translation; coord=4:185694322..185697081:1; parent_transcript=GRMZM5G840955_T01; parent_gene=GRMZM5G840955	GRMZM5G840955_P01	33 kDa	Ref	1.4	1	1.2	1.6	No Values	No Values	No Values	No Values
1301	seq=translation; coord=8:157100232..157101209:-1; parent_transcript=GRMZM2G168552_T01; parent_gene=GRMZM2G168552	GRMZM2G168552_P01	20 kDa	Ref	0.4	0	2.3	-0.2	No Values	No Values	No Values	No Values
1302	seq=translation; coord=1:3714426..3715519:1; parent_transcript=GRMZM2G030173_T01; parent_gene=GRMZM2G030173	GRMZM2G030173_P01	27 kDa	Ref	-0.2	-0.2	-0.4	-1	No Values	No Values	No Values	No Values
1303	seq=translation; coord=7:18222228..18224357:1; parent_transcript=GRMZM2G094928_T01; parent_gene=GRMZM2G094928	GRMZM2G094928_P01 (+1)	12 kDa	Ref	1.3	0.8	1.4	0.3	No Values	No Values	No Values	No Values
1304	seq=translation; coord=3:4757467..4758786:-1; parent_transcript=GRMZM2G055434_T01; parent_gene=GRMZM2G055434	GRMZM2G055434_P01	23 kDa	Ref	No Values	No Values	No Values	No Values	2.6	0.1	1.3	0.7
1305	seq=translation; coord=1:299124462..299129776:-1; parent_transcript=GRMZM2G176612_T02; parent_gene=GRMZM2G176612	GRMZM2G176612_P02	45 kDa	Ref	3.3	3	2.3	1.2	-1.5	-2.3	-2.1	-2.4
1306	seq=translation; coord=10:16487570..16504336:1; parent_transcript=GRMZM2G028307_T02; parent_gene=GRMZM2G028307	GRMZM2G028307_P02	73 kDa	Ref	-0.1	-0.3	0	0.4	1	0.6	0.7	1
1307	seq=translation; coord=1:165814484..165965196:1; parent_transcript=GRMZM2G003595_T01; parent_gene=GRMZM2G003595	GRMZM2G003595_P01 (+2)	7 kDa	Ref	3.3	2.9	2.4	1.4	4.9	4.5	4.3	3.9
1308	seq=translation; coord=2:9503022..9507292:-1; parent_transcript=GRMZM2G040397_T01; parent_gene=GRMZM2G040397	GRMZM2G040397_P01 (+1)	45 kDa	Ref	0.6	0.3	0.3	0.5	0.1	-0.9	-1.3	-1.3
1309	seq=translation; coord=2:56806976..56810257:1; parent_transcript=GRMZM2G119689_T01; parent_gene=GRMZM2G119689	GRMZM2G119689_P01 (+2)	66 kDa	Ref	-0.1	-0.6	0.5	-0.4	-0.8	-2.3	-1.6	-2.7
1310	seq=translation; coord=9:99409919..99416720:1; parent_transcript=GRMZM2G033555_T01; parent_gene=GRMZM2G033555	GRMZM2G033555_P01 (+2)	38 kDa	Ref	-0.4	-0.8	-0.7	-0.3	1.5	1.6	1.3	1.3
1311	seq=translation; coord=8:4108305..4111250:-1; parent_transcript=GRMZM2G028156_T01; parent_gene=GRMZM2G028156	GRMZM2G028156_P01 (+3)	88 kDa	Ref	0	0.3	0	-0.1	0.3	-0.2	-0.1	-0.6
1312	seq=translation; coord=2:198568661..198574971:1; parent_transcript=GRMZM2G081571_T04; parent_gene=GRMZM2G081571	GRMZM2G081571_P04 (+1)	36 kDa	Ref	-1	-0.7	-0.8	-0.4	-2.1	-0.9	-0.7	-0.6

1313	seq=translation; coord=3:159873679..159879241:-1; parent_transcript=GRMZM2G105531_T01; parent_gene=GRMZM2G105531	GRMZM2G105531_P01	22 kDa	Ref	0.8	0.3	1	0.4	1.7	0.7	0.9	0.5
1314	seq=translation; coord=6:161446540..161449925:1; parent_transcript=GRMZM2G115049_T01; parent_gene=GRMZM2G115049	GRMZM2G115049_P01	29 kDa	Ref	1.3	0.5	0.4	0.8	1.1	-1.9	-1.2	-1.5
1315	seq=translation; coord=9:83796519..83798959:1; parent_transcript=GRMZM2G140179_T01; parent_gene=GRMZM2G140179	GRMZM2G140179_P01	74 kDa	Ref	Reference Missing	Reference Missing	Reference Missing	Reference Missing	0.4	-2.3	-4.8	-5
1316	seq=translation; coord=7:169836954..169839970:-1; parent_transcript=GRMZM2G037177_T01; parent_gene=GRMZM2G037177	GRMZM2G037177_P01	26 kDa	Ref	0	0.1	0	0.4	2.9	2.2	1.6	1.4
1317	seq=translation; coord=8:174419193..174424343:1; parent_transcript=GRMZM2G118462_T01; parent_gene=GRMZM2G118462	GRMZM2G118462_P01 (+2)	102 kDa	Ref	No Values	No Values	No Values	No Values	1	0.6	0.6	0.2
1318	seq=translation; coord=4:186948838..186952130:-1; parent_transcript=GRMZM2G357595_T01; parent_gene=GRMZM2G357595	GRMZM2G357595_P01	31 kDa	Ref	0.5	0.4	0	0.7	No Values	No Values	No Values	No Values
1319	seq=translation; coord=3:37865603..37869803:1; parent_transcript=GRMZM2G069208_T01; parent_gene=GRMZM2G069208	GRMZM2G069208_P01 (+2)	33 kDa	Ref	No Values	No Values	No Values	No Values	1.3	1.6	1	0.8
1320	seq=translation; coord=3:7078308..7082094:1; parent_transcript=GRMZM2G104081_T01; parent_gene=GRMZM2G104081	GRMZM2G104081_P01 (+2)	54 kDa	Ref	No Values	No Values	No Values	No Values	-0.6	0.9	0.5	-0.5
1321	seq=translation; coord=10:61378667..61384801:1; parent_transcript=GRMZM2G143568_T01; parent_gene=GRMZM2G143568	GRMZM2G143568_P01	69 kDa	Ref	0	-0.4	-0.6	-0.2	No Values	No Values	No Values	No Values
1322	seq=translation; coord=2:12023721..12027357:-1; parent_transcript=GRMZM2G156365_T02; parent_gene=GRMZM2G156365	GRMZM2G156365_P02 (+4)	33 kDa	Ref	0.7	0.1	-1.1	-1.5	No Values	No Values	No Values	No Values
1323	seq=translation; coord=5:18170761..18172340:1; parent_transcript=GRMZM2G060940_T01; parent_gene=GRMZM2G060940	GRMZM2G060940_P01 (+2)	40 kDa	Ref	No Values	No Values	No Values	No Values	1	2.1	Reference Missing	1.1
1324	seq=translation; coord=3:127303985..127310112:-1; parent_transcript=AC182617.3_FGT001; parent_gene=AC182617.3_FG001	AC182617.3_FGP001 (+4)	38 kDa	Ref	No Values	No Values	No Values	No Values	0.4	0.3	0.1	-0.1
1325	seq=translation; coord=7:132157014..132162257:1; parent_transcript=GRMZM2G068862_T01; parent_gene=GRMZM2G068862	GRMZM2G068862_P01	44 kDa	Ref	No Values	No Values	No Values	No Values	0.2	0.4	0.1	0.5

1326	seq=translation; coord=4:120394113..120399917:1; parent_transcript=GRMZM2G153984_T01; parent_gene=GRMZM2G153984	GRMZM2G153984_P01	31 kDa	Ref	-0.3	-0.3	-0.3	-0.3	No Values	No Values	No Values	No Values
1327	seq=translation; coord=2:130522633..130525928:-1; parent_transcript=GRMZM2G128268_T01; parent_gene=GRMZM2G128268	GRMZM2G128268_P01	45 kDa	Ref	1.3	-0.7	-0.2	-0.1	No Values	No Values	No Values	No Values
1328	seq=translation; coord=3:173472565..173475730:-1; parent_transcript=GRMZM2G081652_T01; parent_gene=GRMZM2G081652	GRMZM2G081652_P01	62 kDa	Ref	0.8	0.2	0.5	0.6	No Values	No Values	No Values	No Values
1329	seq=translation; coord=1:205090665..205094372:1; parent_transcript=GRMZM2G144387_T01; parent_gene=GRMZM2G144387	GRMZM2G144387_P01 (+1)	25 kDa	Ref	0.9	1.1	1.3	0.3	No Values	No Values	No Values	No Values
1330	seq=translation; coord=10:98135181..98154139:1; parent_transcript=AC197672.3_FGT002; parent_gene=AC197672.3_FG002	AC197672.3_FGP002	257 kDa	Ref	No Values	No Values	No Values	No Values	-0.2	-0.5	Reference Missing	-0.3
1331	seq=translation; coord=9:9638146..9648092:-1; parent_transcript=GRMZM2G033130_T01; parent_gene=GRMZM2G033130	GRMZM2G033130_P01 (+4)	104 kDa	Ref	No Values	No Values	No Values	No Values	Reference Missing	Reference Missing	Reference Missing	Reference Missing
1332	seq=translation; coord=9:99437276..99441674:1; parent_transcript=GRMZM2G034069_T01; parent_gene=GRMZM2G034069	GRMZM2G034069_P01	35 kDa	Ref	No Values	No Values	No Values	No Values	0.2	0.4	0	0
1333	seq=translation; coord=9:146299403..146305484:1; parent_transcript=GRMZM2G051782_T01; parent_gene=GRMZM2G051782	GRMZM2G051782_P01	50 kDa	Ref	No Values	No Values	No Values	No Values	0.3	1.6	1.3	0.8
1334	seq=translation; coord=1:283086410..283088116:1; parent_transcript=GRMZM2G065083_T01; parent_gene=GRMZM2G065083	GRMZM2G065083_P01	15 kDa	Ref	No Values	No Values	No Values	No Values	0	0	0.5	0.7
1335	seq=translation; coord=5:184168891..184173292:1; parent_transcript=GRMZM2G071846_T01; parent_gene=GRMZM2G071846	GRMZM2G071846_P01	24 kDa	Ref	No Values	No Values	No Values	No Values	-0.2	0.6	0.5	-0.3
1336	seq=translation; coord=2:189499850..189501644:1; parent_transcript=GRMZM2G080466_T01; parent_gene=GRMZM2G080466	GRMZM2G080466_P01 (+3)	14 kDa	Ref	2.9	2	2.7	3.3	No Values	No Values	No Values	No Values
1337	seq=translation; coord=1:183983464..183984705:-1; parent_transcript=GRMZM2G080724_T01; parent_gene=GRMZM2G080724	GRMZM2G080724_P01	27 kDa	Ref	No Values	No Values	No Values	No Values	-1.3	-0.2	-0.8	-0.3
1338	seq=translation; coord=2:29975938..29981822:1; parent_transcript=GRMZM2G082581_T01; parent_gene=GRMZM2G082581	GRMZM2G082581_P01	36 kDa	Ref	No Values	No Values	No Values	No Values	0	-0.2	-0.1	0.3

1339	seq=translation; coord=8:137403695..137407159:-1; parent_transcript=GRMZM2G106960_T01; parent_gene=GRMZM2G106960	GRMZM2G106960_P01	23 kDa	Ref	-1.6	-1.7	-1.4	-1.4	No Values	No Values	No Values	No Values
1340	seq=translation; coord=5:13615570..13621897:1; parent_transcript=GRMZM2G161222_T03; parent_gene=GRMZM2G161222	GRMZM2G161222_P03	58 kDa	Ref	No Values	No Values	No Values	No Values	Reference Missing	Reference Missing	Reference Missing	Reference Missing
1341	seq=translation; coord=9:92025063..92027986:-1; parent_transcript=GRMZM2G316232_T01; parent_gene=GRMZM2G316232	GRMZM2G316232_P01	57 kDa	Ref	No Values	No Values	No Values	No Values	1.6	0.9	0.2	0.3
1342	seq=translation; coord=10:117993529..118000841:-1; parent_transcript=GRMZM2G353213_T01; parent_gene=GRMZM2G353213	GRMZM2G353213_P01 (+1)	35 kDa	Ref	No Values	No Values	No Values	No Values	-0.5	-0.9	-0.5	0
1343	seq=translation; coord=9:137069559..137072594:-1; parent_transcript=GRMZM2G053652_T01; parent_gene=GRMZM2G053652	GRMZM2G053652_P01 (+1)	22 kDa	Ref	No Values	No Values	No Values	No Values	Reference Missing	Reference Missing	Reference Missing	Reference Missing
1344	seq=translation; coord=1:297376727..297379573:-1; parent_transcript=GRMZM2G369815_T01; parent_gene=GRMZM2G369815	GRMZM2G369815_P01 (+1)	42 kDa	Ref	2	-1	-2.5	-2.3	No Values	No Values	No Values	No Values
1345	seq=translation; coord=4:159862612..159866537:1; parent_transcript=GRMZM2G050371_T01; parent_gene=GRMZM2G050371	GRMZM2G050371_P01	40 kDa	Ref	No Values	No Values	No Values	No Values	-1	-1.3	-1.3	-1
1346	seq=translation; coord=8:17388792..17393555:-1; parent_transcript=GRMZM2G061969_T01; parent_gene=GRMZM2G061969	GRMZM2G061969_P01	92 kDa	Ref	-0.1	-0.3	-0.7	-0.4	No Values	No Values	No Values	No Values
1347	seq=translation; coord=5:215652783..215654639:1; parent_transcript=GRMZM2G143627_T01; parent_gene=GRMZM2G143627	GRMZM2G143627_P01 (+1)	13 kDa	Ref	1.4	0.5	1.2	-0.4	No Values	No Values	No Values	No Values
1348	seq=translation; coord=1:286506118..286513080:-1; parent_transcript=GRMZM2G141473_T01; parent_gene=GRMZM2G141473	GRMZM2G141473_P01	146 kDa	Ref	0	0	0.9	2.3	No Values	No Values	No Values	No Values
1349	seq=translation; coord=5:18753627..18756558:1; parent_transcript=GRMZM2G138572_T01; parent_gene=GRMZM2G138572	GRMZM2G138572_P01	19 kDa	Ref	0.8	-0.2	1	-0.8	No Values	No Values	No Values	No Values
1350	seq=translation; coord=2:26769801..26771736:1; parent_transcript=GRMZM2G474575_T01; parent_gene=GRMZM2G474575	GRMZM2G474575_P01	23 kDa	Ref	No Values	No Values	No Values	No Values	-1.1	-0.2	1.1	2.4
1351	seq=translation; coord=7:110983097..110986540:-1; parent_transcript=GRMZM2G048277_T01; parent_gene=GRMZM2G048277	GRMZM2G048277_P01 (+2)	16 kDa	Ref	-0.4	0.1	-0.3	Value Missing	-2.8	-5	-6.2	-5.9

1352	seq=translation; coord=8:42281387..42289748:-1; parent_transcript=GRMZM2G145595_T01; parent_gene=GRMZM2G145595	GRMZM2G145595_P01	60 kDa	Ref	0.1	0.6	0.3	0.5	-2.4	-2.7	-2.8	-3.1
1353	seq=translation; coord=1:31242217..31244585:-1; parent_transcript=GRMZM2G116846_T01; parent_gene=GRMZM2G116846	GRMZM2G116846_P01	36 kDa	Ref	-0.7	-0.1	-1.2	-0.7	2.4	2.2	1.9	1.9
1354	seq=translation; coord=1:292354165..292360031:-1; parent_transcript=GRMZM2G151967_T01; parent_gene=GRMZM2G151967	GRMZM2G151967_P01	59 kDa	Ref	0	-0.2	-0.1	0.4	4.4	2.5	2.8	3.7
1355	seq=translation; coord=7:4538433..4546019:1; parent_transcript=GRMZM2G480002_T01; parent_gene=GRMZM2G480002	GRMZM2G480002_P01	187 kDa	Ref	No Values	No Values	No Values	No Values	1.6	1.2	0.7	0.7
1356	seq=translation; coord=5:21571290..21578409:-1; parent_transcript=GRMZM2G073934_T01; parent_gene=GRMZM2G073934	GRMZM2G073934_P01	53 kDa	Ref	0.8	0.5	0.1	0.4	No Values	No Values	No Values	No Values
1357	seq=translation; coord=1:298331067..298333316:1; parent_transcript=GRMZM2G023242_T01; parent_gene=GRMZM2G023242	GRMZM2G023242_P01 (+2)	17 kDa	Ref	No Values	No Values	No Values	No Values	0.5	0.2	-0.2	0.3
1358	seq=translation; coord=2:25913684..25932129:1; parent_transcript=GRMZM2G376731_T01; parent_gene=GRMZM2G376731	GRMZM2G376731_P01	99 kDa	Ref	No Values	No Values	No Values	No Values	0.9	-0.7	-0.9	-0.9
1359	seq=translation; coord=5:14176514..14178456:-1; parent_transcript=GRMZM5G858249_T01; parent_gene=GRMZM5G858249	GRMZM5G858249_P01	33 kDa	Ref	0.4	0.7	1.3	2	No Values	No Values	No Values	No Values
1360	seq=translation; coord=3:135084015..135105483:1; parent_transcript=GRMZM2G326116_T01; parent_gene=GRMZM2G326116	GRMZM2G326116_P01	35 kDa	Ref	-1.7	-1.6	-0.9	-1.1	No Values	No Values	No Values	No Values
1361	seq=translation; coord=6:110695290..110707260:1; parent_transcript=GRMZM2G412611_T01; parent_gene=GRMZM2G412611	GRMZM2G412611_P01	164 kDa	Ref	-0.8	-0.8	-0.4	-0.9	No Values	No Values	No Values	No Values
1362	seq=translation; coord=3:1767353..1776209:-1; parent_transcript=GRMZM2G123499_T01; parent_gene=GRMZM2G123499	GRMZM2G123499_P01 (+1)	74 kDa	Ref	0.1	0.1	-0.2	0.2	No Values	No Values	No Values	No Values
1363	seq=translation; coord=1:122162499..122163689:1; parent_transcript=AC202185.4_FGT004; parent_gene=AC202185.4_FG004	AC202185.4_FGP004	23 kDa	Ref	-0.2	0	1.6	0.3	No Values	No Values	No Values	No Values
1364	seq=translation; coord=2:71785920..71790206:-1; parent_transcript=GRMZM2G000980_T01; parent_gene=GRMZM2G000980	GRMZM2G000980_P01 (+1)	80 kDa	Ref	No Values	No Values	No Values	No Values	Reference Missing	1	Reference Missing	1.1

1365	seq=translation; coord=5:17058108..17064678:1; parent_transcript=GRMZM2G018943_T01; parent_gene=GRMZM2G018943	GRMZM2G018943_P01 (+1)	80 kDa	Ref	No Values	No Values	No Values	No Values	0.1	0.1	0.2	-0.3
1366	seq=translation; coord=6:112137413..112140985:1; parent_transcript=GRMZM2G064993_T01; parent_gene=GRMZM2G064993	GRMZM2G064993_P01	38 kDa	Ref	0.5	-0.3	0.1	-0.2	No Values	No Values	No Values	No Values
1367	seq=translation; coord=5:59290440..59291930:-1; parent_transcript=GRMZM2G084942_T01; parent_gene=GRMZM2G084942	GRMZM2G084942_P01 (+1)	40 kDa	Ref	0.8	0.8	0.7	0.9	No Values	No Values	No Values	No Values
1368	seq=translation; coord=5:209693887..209699045:1; parent_transcript=GRMZM2G101463_T01; parent_gene=GRMZM2G101463	GRMZM2G101463_P01	56 kDa	Ref	No Values	No Values	No Values	No Values	0.2	0.9	0.6	0.3
1369	seq=translation; coord=3:184059147..184065670:1; parent_transcript=GRMZM2G118363_T01; parent_gene=GRMZM2G118363	GRMZM2G118363_P01	67 kDa	Ref	No Values	No Values	No Values	No Values	Reference Missing	Reference Missing	Reference Missing	Reference Missing
1370	seq=translation; coord=9:140970479..140979075:-1; parent_transcript=GRMZM2G119627_T01; parent_gene=GRMZM2G119627	GRMZM2G119627_P01	54 kDa	Ref	No Values	No Values	No Values	No Values	-1.6	-1.3	-1.7	-1.7
1371	seq=translation; coord=6:111182254..111183500:-1; parent_transcript=GRMZM2G122302_T01; parent_gene=GRMZM2G122302	GRMZM2G122302_P01	20 kDa	Ref	0.1	1.6	0.6	-0.5	No Values	No Values	No Values	No Values
1372	seq=translation; coord=8:85844666..85847518:-1; parent_transcript=GRMZM2G132130_T01; parent_gene=GRMZM2G132130	GRMZM2G132130_P01	29 kDa	Ref	No Values	No Values	No Values	No Values	-0.8	-0.4	-0.2	0.4
1373	seq=translation; coord=1:256071969..256085112:1; parent_transcript=GRMZM2G150772_T02; parent_gene=GRMZM2G150772	GRMZM2G150772_P02	179 kDa	Ref	-0.4	-0.3	-1.1	-1.1	No Values	No Values	No Values	No Values
1374	seq=translation; coord=6:111253708..111260162:-1; parent_transcript=GRMZM2G329306_T01; parent_gene=GRMZM2G329306	GRMZM2G329306_P01	43 kDa	Ref	No Values	No Values	No Values	No Values	-0.1	-0.6	0.5	-0.9
1375	seq=translation; coord=10:136292132..136296887:1; parent_transcript=GRMZM2G414915_T01; parent_gene=GRMZM2G414915	GRMZM2G414915_P01	82 kDa	Ref	-0.7	-0.7	-1.2	-1.2	No Values	No Values	No Values	No Values
1376	seq=translation; coord=6:140865431..140872714:-1; parent_transcript=GRMZM2G442057_T01; parent_gene=GRMZM2G442057	GRMZM2G442057_P01	47 kDa	Ref	No Values	No Values	No Values	No Values	1.3	0.6	Reference Missing	0.8
1377	seq=translation; coord=10:10725550..10730964:1; parent_transcript=GRMZM2G014397_T02; parent_gene=GRMZM2G014397	GRMZM2G014397_P02 (+1)	34 kDa	Ref	1.2	1.5	1.7	1.7	No Values	No Values	No Values	No Values

1378	seq=translation; coord=1:257994567..257995838:-1; parent_transcript=GRMZM2G044762_T01; parent_gene=GRMZM2G044762	GRMZM2G044762_P01 (+1)	23 kDa	Ref	No Values	No Values	No Values	No Values	0.3	-0.2	0	-0.4
1379	seq=translation; coord=1:152164284..152165382:1; parent_transcript=GRMZM2G132077_T01; parent_gene=GRMZM2G132077	GRMZM2G132077_P01	18 kDa	Ref	0.6	-0.3	-0.3	0.1	No Values	No Values	No Values	No Values
1380	seq=translation; coord=3:124642615..124647335:-1; parent_transcript=GRMZM2G098397_T01; parent_gene=GRMZM2G098397	GRMZM2G098397_P01 (+3)	63 kDa	Ref	0.2	0.2	0.1	0.7	No Values	No Values	No Values	No Values
1381	seq=translation; coord=8:23799793..23801891:-1; parent_transcript=GRMZM5G861077_T01; parent_gene=GRMZM5G861077	GRMZM5G861077_P01	66 kDa	Ref	-0.3	-0.1	-0.4	0.2	No Values	No Values	No Values	No Values
1382	seq=translation; coord=7:174339610..174341979:-1; parent_transcript=GRMZM2G424205_T01; parent_gene=GRMZM2G424205	GRMZM2G424205_P01	26 kDa	Ref	-0.7	-1.2	-0.4	0.2	No Values	No Values	No Values	No Values
1383	seq=translation; coord=8:121435529..121437647:-1; parent_transcript=GRMZM2G042865_T01; parent_gene=GRMZM2G042865	GRMZM2G042865_P01 (+1)	51 kDa	Ref	-0.2	-0.4	-0.4	-0.4	No Values	No Values	No Values	No Values
1384	seq=translation; coord=8:76128223..76129850:1; parent_transcript=AC231180.2_FGT006; parent_gene=AC231180.2_FG006	AC231180.2_FGP006	48 kDa	Ref	0.6	-0.2	-1.4	-1.6	No Values	No Values	No Values	No Values
1385	seq=translation; coord=7:25812344..25817942:1; parent_transcript=GRMZM2G003789_T01; parent_gene=GRMZM2G003789	GRMZM2G003789_P01	47 kDa	Ref	No Values	No Values	No Values	No Values	-0.4	0.1	0.7	0.5
1386	seq=translation; coord=1:171489898..171496325:1; parent_transcript=GRMZM2G019926_T02; parent_gene=GRMZM2G019926	GRMZM2G019926_P02 (+2)	35 kDa	Ref	0	0.2	-0.1	0.7	No Values	No Values	No Values	No Values
1387	seq=translation; coord=2:8914517..8917818:-1; parent_transcript=GRMZM2G076239_T02; parent_gene=GRMZM2G076239	GRMZM2G076239_P02 (+3)	40 kDa	Ref	No Values	No Values	No Values	No Values	1.2	1.6	1.4	0.9
1388	seq=translation; coord=3:205146239..205150759:-1; parent_transcript=GRMZM2G098569_T01; parent_gene=GRMZM2G098569	GRMZM2G098569_P01	41 kDa	Ref	0.7	0.5	0.6	1.1	No Values	No Values	No Values	No Values
1389	seq=translation; coord=6:105434141..105436791:1; parent_transcript=GRMZM2G403076_T01; parent_gene=GRMZM2G403076	GRMZM2G403076_P01	62 kDa	Ref	0.8	-0.1	0.3	2.2	No Values	No Values	No Values	No Values
1390	seq=translation; coord=4:18676199..18682894:1; parent_transcript=GRMZM2G574782_T01; parent_gene=GRMZM2G574782	GRMZM2G574782_P01	57 kDa	Ref	0.3	0.2	0.4	0.3	No Values	No Values	No Values	No Values

1391	seq=translation; coord=8:152750732..152754708:1; parent_transcript=GRMZM5G823017_T01; parent_gene=GRMZM5G823017	GRMZM5G823017_P01 (+2)	46 kDa	Ref	No Values	No Values	No Values	No Values	-0.6	-1.4	-0.3	-0.1
1392	seq=translation; coord=8:118191461..118195143:-1; parent_transcript=AC197705.4_FGT007; parent_gene=AC197705.4_FG007	AC197705.4_FGP007	57 kDa	Ref	No Values	No Values	No Values	No Values	1.2	-1	-0.8	-2
1393	seq=translation; coord=8:29145636..29147578:-1; parent_transcript=GRMZM2G007151_T01; parent_gene=GRMZM2G007151	GRMZM2G007151_P01 (+1)	21 kDa	Ref	1.8	1.7	2.7	1	No Values	No Values	No Values	No Values
1394	seq=translation; coord=3:2080450..2084636:1; parent_transcript=GRMZM2G153181_T01; parent_gene=GRMZM2G153181	GRMZM2G153181_P01	61 kDa	Ref	1.5	0.5	0.1	0.6	No Values	No Values	No Values	No Values
1395	seq=translation; coord=5:58525626..58534933:-1; parent_transcript=GRMZM2G397247_T04; parent_gene=GRMZM2G397247	GRMZM2G397247_P04	46 kDa	Ref	0.3	0	0.4	0.2	No Values	No Values	No Values	No Values
1396	seq=translation; coord=1:210463083..210467521:1; parent_transcript=GRMZM2G073814_T02; parent_gene=GRMZM2G073814	GRMZM2G073814_P02	65 kDa	Ref	-0.4	-1.7	-1	-0.4	No Values	No Values	No Values	No Values
1397	seq=translation; coord=10:16517475..16520678:1; parent_transcript=GRMZM2G091819_T01; parent_gene=GRMZM2G091819	GRMZM2G091819_P01	44 kDa	Ref	No Values	No Values	No Values	No Values	2.7	0.3	0	-1.5
1398	seq=translation; coord=4:151648685..151655399:1; parent_transcript=GRMZM2G145226_T01; parent_gene=GRMZM2G145226	GRMZM2G145226_P01	25 kDa	Ref	No Values	No Values	No Values	No Values	-1.6	-0.8	-0.5	-0.6
1399	seq=translation; coord=1:287289676..287296815:-1; parent_transcript=GRMZM2G178415_T01; parent_gene=GRMZM2G178415	GRMZM2G178415_P01 (+3)	44 kDa	Ref	-0.6	-0.1	-0.3	-0.4	No Values	No Values	No Values	No Values
1400	seq=translation; coord=8:64567512..64568715:-1; parent_transcript=GRMZM2G075796_T01; parent_gene=GRMZM2G075796	GRMZM2G075796_P01	21 kDa	Ref	0.4	0.1	0.5	-0.1	No Values	No Values	No Values	No Values
1401	seq=translation; coord=5:6157585..6167807:-1; parent_transcript=GRMZM2G171588_T01; parent_gene=GRMZM2G171588	GRMZM2G171588_P01 (+2)	36 kDa	Ref	1.1	0.4	0.2	0	No Values	No Values	No Values	No Values

Table S2. The peptides of identified proteins using iTRAQ.

Id	Protein Name	Accession Numbers	Assigned	Unique	Sequence	Prob	Modifications	Ion Score	Identity Score
1	seq=translation; coord=8:107003965..107014830:1; parent_transcript=GRMZM2G097457_T01; parent_gene=GRMZM2G097457	GRMZM2G097457_P01,GRMZM2G097457_P02	TRUE	TRUE	aETSPEDVGGMHAAAGILTER	95%	n+304 (+304)	84.15	25.00
2	seq=translation; coord=8:107003965..107014830:1; parent_transcript=GRMZM2G097457_T01; parent_gene=GRMZM2G097457	GRMZM2G097457_P01,GRMZM2G097457_P02	TRUE	TRUE	aETSPEDVGGmHAAAGILTER	95%	n+304 (+304), Oxidation (+16)	48.54	25.00
3	seq=translation; coord=8:107003965..107014830:1; parent_transcript=GRMZM2G097457_T01; parent_gene=GRMZM2G097457	GRMZM2G097457_P01,GRMZM2G097457_P02	TRUE	TRUE	aLPPGLWAEVLDGLR	95%	n+304 (+304)	59.90	26.05
4	seq=translation; coord=8:107003965..107014830:1; parent_transcript=GRMZM2G097457_T01; parent_gene=GRMZM2G097457	GRMZM2G097457_P01,GRMZM2G097457_P02	TRUE	TRUE	aMDGLSVTIR	95%	n+304 (+304)	38.70	25.00
5	seq=translation; coord=8:107003965..107014830:1; parent_transcript=GRMZM2G097457_T01; parent_gene=GRMZM2G097457	GRMZM2G097457_P01,GRMZM2G097457_P02	TRUE	TRUE	eLcSETGANQEDALAR	95%	n+304 (+304), Carbamidomethyl (+57)	93.42	25.00
6	seq=translation; coord=8:107003965..107014830:1; parent_transcript=GRMZM2G097457_T01; parent_gene=GRMZM2G097457	GRMZM2G097457_P01,GRMZM2G097457_P02	TRUE	TRUE	eLVSQYk	88%	n+304 (+304), K+304 (+304)	27.63	26.32
7	seq=translation; coord=8:107003965..107014830:1; parent_transcript=GRMZM2G097457_T01; parent_gene=GRMZM2G097457	GRMZM2G097457_P01,GRMZM2G097457_P02	TRUE	TRUE	eMQDIEFTVQESR	95%	n+304 (+304)	51.51	25.00
8	seq=translation; coord=8:107003965..107014830:1; parent_transcript=GRMZM2G097457_T01; parent_gene=GRMZM2G097457	GRMZM2G097457_P01,GRMZM2G097457_P02	TRUE	TRUE	emQDIEFTVQESR	95%	n+304 (+304), Oxidation (+16)	44.89	25.00
9	seq=translation; coord=8:107003965..107014830:1; parent_transcript=GRMZM2G097457_T01; parent_gene=GRMZM2G097457	GRMZM2G097457_P01,GRMZM2G097457_P02	TRUE	TRUE	fIPIYLAQGILQHDPFEVLDQR	95%	n+304 (+304)	55.80	25.15
10	seq=translation; coord=8:107003965..107014830:1; parent_transcript=GRMZM2G097457_T01; parent_gene=GRMZM2G097457	GRMZM2G097457_P01,GRMZM2G097457_P02	TRUE	TRUE	fLDMFGNVVMDIPHALFEEk	95%	n+304 (+304), K+304 (+304)	34.23	25.47
11	seq=translation; coord=8:107003965..107014830:1; parent_transcript=GRMZM2G097457_T01; parent_gene=GRMZM2G097457	GRMZM2G097457_P01,GRMZM2G097457_P02	TRUE	TRUE	fLDmFGNVVMDIPHALFEEk	95%	n+304 (+304), Oxidation (+16), K+304 (+304)	33.23	25.00
12	seq=translation; coord=8:107003965..107014830:1; parent_transcript=GRMZM2G097457_T01; parent_gene=GRMZM2G097457	GRMZM2G097457_P01,GRMZM2G097457_P02	TRUE	TRUE	fLDMFGNVVmDIPHALFEEk	95%	n+304 (+304), Oxidation (+16), K+304 (+304)	46.80	25.04
13	seq=translation; coord=8:107003965..107014830:1; parent_transcript=GRMZM2G097457_T01; parent_gene=GRMZM2G097457	GRMZM2G097457_P01,GRMZM2G097457_P02	TRUE	TRUE	fLDmFGNVVmDIPHALFEEk	95%	n+304 (+304), Oxidation (+16), Oxidation (+16), K+304 (+304)	38.67	25.00
14	seq=translation; coord=8:107003965..107014830:1; parent_transcript=GRMZM2G097457_T01; parent_gene=GRMZM2G097457	GRMZM2G097457_P01,GRMZM2G097457_P02	TRUE	TRUE	iAVDMVNEGLVER	95%	n+304 (+304)	56.80	26.73
15	seq=translation; coord=8:107003965..107014830:1; parent_transcript=GRMZM2G097457_T01; parent_gene=GRMZM2G097457	GRMZM2G097457_P01,GRMZM2G097457_P02	TRUE	TRUE	iAVDmVNEGLVER	95%	n+304 (+304), Oxidation (+16)	49.13	25.37
16	seq=translation; coord=8:107003965..107014830:1; parent_transcript=GRMZM2G097457_T01; parent_gene=GRMZM2G097457	GRMZM2G097457_P01,GRMZM2G097457_P02	TRUE	TRUE	iGTMIEIPR	86%	n+304 (+304)	26.90	26.59

17	seq=translation; coord=8:107003965..107014830:1; parent_transcript=GRMZM2G097457_T01; parent_gene=GRMZM2G097457	GRMZM2G097457_P01,GRMZM2G097457_P02	TRUE	TRUE	lGISYPELTEMQAR	95%	n+304 (+304)	42.94	25.00
18	seq=translation; coord=8:107003965..107014830:1; parent_transcript=GRMZM2G097457_T01; parent_gene=GRMZM2G097457	GRMZM2G097457_P01,GRMZM2G097457_P02	TRUE	TRUE	lGISYPELTEmQAR	95%	n+304 (+304), Oxidation (+16)	54.35	25.00
19	seq=translation; coord=8:107003965..107014830:1; parent_transcript=GRMZM2G097457_T01; parent_gene=GRMZM2G097457	GRMZM2G097457_P01,GRMZM2G097457_P02	TRUE	TRUE	lLDPPLHEFLPEGNVEEIVR	95%	n+304 (+304)	48.29	25.55
20	seq=translation; coord=8:107003965..107014830:1; parent_transcript=GRMZM2G097457_T01; parent_gene=GRMZM2G097457	GRMZM2G097457_P01,GRMZM2G097457_P02	TRUE	TRUE	lSEVNPMLGFR	89%	n+304 (+304)	27.22	25.73
21	seq=translation; coord=8:107003965..107014830:1; parent_transcript=GRMZM2G097457_T01; parent_gene=GRMZM2G097457	GRMZM2G097457_P01,GRMZM2G097457_P02	TRUE	TRUE	nDTDLTATDLk	95%	n+304 (+304), K+304 (+304)	31.03	25.16
22	seq=translation; coord=8:107003965..107014830:1; parent_transcript=GRMZM2G097457_T01; parent_gene=GRMZM2G097457	GRMZM2G097457_P01,GRMZM2G097457_P02	TRUE	TRUE	nNGAEGIGLcR	95%	n+304 (+304), Carbamidomethyl (+57)	31.09	25.00
23	seq=translation; coord=8:107003965..107014830:1; parent_transcript=GRMZM2G097457_T01; parent_gene=GRMZM2G097457	GRMZM2G097457_P01,GRMZM2G097457_P02	TRUE	TRUE	nVYVEAk	91%	n+304 (+304), K+304 (+304)	28.08	26.24
24	seq=translation; coord=8:107003965..107014830:1; parent_transcript=GRMZM2G097457_T01; parent_gene=GRMZM2G097457	GRMZM2G097457_P01,GRMZM2G097457_P02	TRUE	TRUE	qLELAVLAVFDSWESPR	95%	n+304 (+304)	39.73	25.66
25	seq=translation; coord=8:107003965..107014830:1; parent_transcript=GRMZM2G097457_T01; parent_gene=GRMZM2G097457	GRMZM2G097457_P01,GRMZM2G097457_P02	TRUE	TRUE	qMIMAPTVELR	95%	n+304 (+304)	33.07	26.15
26	seq=translation; coord=8:107003965..107014830:1; parent_transcript=GRMZM2G097457_T01; parent_gene=GRMZM2G097457	GRMZM2G097457_P01,GRMZM2G097457_P02	TRUE	TRUE	qmIMAPTVELR	95%	n+304 (+304), Oxidation (+16)	34.08	25.09
27	seq=translation; coord=8:107003965..107014830:1; parent_transcript=GRMZM2G097457_T01; parent_gene=GRMZM2G097457	GRMZM2G097457_P01,GRMZM2G097457_P02	TRUE	TRUE	qPLSPPALSGDLGTFMSWVDDVRk	95%	n+304 (+304), K+304 (+304)	34.50	25.00
28	seq=translation; coord=8:107003965..107014830:1; parent_transcript=GRMZM2G097457_T01; parent_gene=GRMZM2G097457	GRMZM2G097457_P01,GRMZM2G097457_P02	TRUE	TRUE	rFLDMFGNVVMDIPHALFEEk	95%	n+304 (+304), K+304 (+304)	52.78	25.00
29	seq=translation; coord=8:107003965..107014830:1; parent_transcript=GRMZM2G097457_T01; parent_gene=GRMZM2G097457	GRMZM2G097457_P01,GRMZM2G097457_P02	TRUE	TRUE	rPLLLSVR	95%	n+304 (+304)	29.87	25.00
30	seq=translation; coord=8:107003965..107014830:1; parent_transcript=GRMZM2G097457_T01; parent_gene=GRMZM2G097457	GRMZM2G097457_P01,GRMZM2G097457_P02	TRUE	TRUE	sGAAVSMPGMMDTVLNLGLNDQVAAGLAAk	95%	n+304 (+304), K+304 (+304)	57.89	25.00
31	seq=translation; coord=8:107003965..107014830:1; parent_transcript=GRMZM2G097457_T01; parent_gene=GRMZM2G097457	GRMZM2G097457_P01,GRMZM2G097457_P02	TRUE	TRUE	sVILVR	94%	n+304 (+304)	31.34	25.00
32	seq=translation; coord=8:107003965..107014830:1; parent_transcript=GRMZM2G097457_T01; parent_gene=GRMZM2G097457	GRMZM2G097457_P01,GRMZM2G097457_P02	TRUE	TRUE	tGLDYVScSPFR	95%	n+304 (+304), Carbamidomethyl (+57)	41.27	25.00
33	seq=translation; coord=8:107003965..107014830:1; parent_transcript=GRMZM2G097457_T01; parent_gene=GRMZM2G097457	GRMZM2G097457_P01,GRMZM2G097457_P02	TRUE	TRUE	vLANADTPEDALAAR	95%	n+304 (+304)	84.55	25.54
34	seq=translation; coord=8:107003965..107014830:1; parent_transcript=GRMZM2G097457_T01; parent_gene=GRMZM2G097457	GRMZM2G097457_P01,GRMZM2G097457_P02	TRUE	TRUE	wVEEYMGAALGDPR	94%	n+304 (+304)	31.32	25.00

35	seq=translation; coord=9:11496011..11502772:1; parent_transcript=GRMZM2G089713_T01; parent_gene=GRMZM2G089713	GRMZM2G089713_P01,GRMZM2G089713_P02	TRUE	TRUE	aADILVNFFDk	95%	n+304 (+304), K+304 (+304)	54.96	26.91
36	seq=translation; coord=9:11496011..11502772:1; parent_transcript=GRMZM2G089713_T01; parent_gene=GRMZM2G089713	GRMZM2G089713_P01,GRMZM2G089713_P02	TRUE	TRUE	aDPSYWDk	94%	n+304 (+304), K+304 (+304)	27.05	25.00
37	seq=translation; coord=9:11496011..11502772:1; parent_transcript=GRMZM2G089713_T01; parent_gene=GRMZM2G089713	GRMZM2G089713_P01,GRMZM2G089713_P02	TRUE	TRUE	aLENEMLLR	92%	n+304 (+304)	29.01	26.18
38	seq=translation; coord=9:11496011..11502772:1; parent_transcript=GRMZM2G089713_T01; parent_gene=GRMZM2G089713	GRMZM2G089713_P01,GRMZM2G089713_P02	TRUE	TRUE	aLENEmLLR	93%	n+304 (+304), Oxidation (+16)	28.72	25.51
39	seq=translation; coord=9:11496011..11502772:1; parent_transcript=GRMZM2G089713_T01; parent_gene=GRMZM2G089713	GRMZM2G089713_P01,GRMZM2G089713_P02	TRUE	TRUE	dTVGQYESHIAFTLPGLYR	95%	n+304 (+304)	78.26	25.30
40	seq=translation; coord=9:11496011..11502772:1; parent_transcript=GRMZM2G089713_T01; parent_gene=GRMZM2G089713	GRMZM2G089713_P01,GRMZM2G089713_P02	TRUE	TRUE	eLANLVIVAGDHGk	95%	n+304 (+304), K+304 (+304)	66.03	25.00
41	seq=translation; coord=9:11496011..11502772:1; parent_transcript=GRMZM2G089713_T01; parent_gene=GRMZM2G089713	GRMZM2G089713_P01,GRMZM2G089713_P02	TRUE	TRUE	eMQAkPDLIIGNYSDGNLVATLLAHk	95%	n+304 (+304), K+304 (+304), K+304 (+304)	34.51	25.00
42	seq=translation; coord=9:11496011..11502772:1; parent_transcript=GRMZM2G089713_T01; parent_gene=GRMZM2G089713	GRMZM2G089713_P01,GRMZM2G089713_P02	TRUE	TRUE	eSLYPLLNFLk	95%	n+304 (+304), K+304 (+304)	36.54	25.00
43	seq=translation; coord=9:11496011..11502772:1; parent_transcript=GRMZM2G089713_T01; parent_gene=GRMZM2G089713	GRMZM2G089713_P01,GRMZM2G089713_P02	TRUE	TRUE	fDVWPYLETYTEDVSSEIMk	95%	n+304 (+304), K+304 (+304)	33.47	25.00
44	seq=translation; coord=9:11496011..11502772:1; parent_transcript=GRMZM2G089713_T01; parent_gene=GRMZM2G089713	GRMZM2G089713_P01,GRMZM2G089713_P02	TRUE	TRUE	fDVWPyLETYTEDVSSEIMk	95%	n+304 (+304), iTRAQ8plex (+304), K+304 (+304)	28.53	25.00
45	seq=translation; coord=9:11496011..11502772:1; parent_transcript=GRMZM2G089713_T01; parent_gene=GRMZM2G089713	GRMZM2G089713_P01,GRMZM2G089713_P02	TRUE	TRUE	fDVWPYLETYTEDVSSEImk	95%	n+304 (+304), Oxidation (+16), K+304 (+304)	57.47	25.00
46	seq=translation; coord=9:11496011..11502772:1; parent_transcript=GRMZM2G089713_T01; parent_gene=GRMZM2G089713	GRMZM2G089713_P01,GRMZM2G089713_P02	TRUE	TRUE	fNIVSPGADMSVYYPYTETDk	95%	n+304 (+304), K+304 (+304)	38.41	25.00
47	seq=translation; coord=9:11496011..11502772:1; parent_transcript=GRMZM2G089713_T01; parent_gene=GRMZM2G089713	GRMZM2G089713_P01,GRMZM2G089713_P02	TRUE	TRUE	fNIVSPGADMSVYYPYTETDkR	95%	n+304 (+304), K+304 (+304)	45.01	25.00
48	seq=translation; coord=9:11496011..11502772:1; parent_transcript=GRMZM2G089713_T01; parent_gene=GRMZM2G089713	GRMZM2G089713_P01,GRMZM2G089713_P02	TRUE	TRUE	fNIVSPGADmSVYYPYTETDkR	95%	n+304 (+304), Oxidation (+16), K+304 (+304)	32.83	25.00
49	seq=translation; coord=9:11496011..11502772:1; parent_transcript=GRMZM2G089713_T01; parent_gene=GRMZM2G089713	GRMZM2G089713_P01,GRMZM2G089713_P02	TRUE	TRUE	gTTMMLNDR	95%	n+304 (+304)	29.71	25.00
50	seq=translation; coord=9:11496011..11502772:1; parent_transcript=GRMZM2G089713_T01; parent_gene=GRMZM2G089713	GRMZM2G089713_P01,GRMZM2G089713_P02	TRUE	TRUE	gTTmMLNDR	94%	n+304 (+304), Oxidation (+16)	28.99	25.00
51	seq=translation; coord=9:11496011..11502772:1; parent_transcript=GRMZM2G089713_T01; parent_gene=GRMZM2G089713	GRMZM2G089713_P01,GRMZM2G089713_P02	TRUE	TRUE	gTTMmLNDR	95%	n+304 (+304), Oxidation (+16)	31.56	25.00
52	seq=translation; coord=9:11496011..11502772:1; parent_transcript=GRMZM2G089713_T01; parent_gene=GRMZM2G089713	GRMZM2G089713_P01,GRMZM2G089713_P02	TRUE	TRUE	gWGDTAk	94%	n+304 (+304), K+304 (+304)	29.40	25.00

53	seq=translation; coord=9:11496011..11502772:1; parent_transcript=GRMZM2G089713_T01; parent_gene=GRMZM2G089713	GRMZM2G089713_P01,GRMZM2G089713_P02	TRUE	TRUE	hQLLAEFDALFDSDk	95%	n+304 (+304), K+304 (+304)	43.06	25.00
54	seq=translation; coord=9:11496011..11502772:1; parent_transcript=GRMZM2G089713_T01; parent_gene=GRMZM2G089713	GRMZM2G089713_P01,GRMZM2G089713_P02	TRUE	TRUE	hQLLAEFDALFDSDkEk	95%	n+304 (+304), K+304 (+304), K+304 (+304)	37.10	25.75
55	seq=translation; coord=9:11496011..11502772:1; parent_transcript=GRMZM2G089713_T01; parent_gene=GRMZM2G089713	GRMZM2G089713_P01,GRMZM2G089713_P02	TRUE	TRUE	kAEEYLLSVPQDTPYSEFNHR	95%	K+304 (+304), n+304 (+304)	43.05	25.00
56	seq=translation; coord=9:11496011..11502772:1; parent_transcript=GRMZM2G089713_T01; parent_gene=GRMZM2G089713	GRMZM2G089713_P01,GRMZM2G089713_P02	TRUE	TRUE	lFQDkESLYPLLNFLk	95%	n+304 (+304), K+304 (+304), K+304 (+304)	27.47	25.00
57	seq=translation; coord=9:11496011..11502772:1; parent_transcript=GRMZM2G089713_T01; parent_gene=GRMZM2G089713	GRMZM2G089713_P01,GRMZM2G089713_P02	TRUE	TRUE	lGATFSSHPNELIALFSR	95%	n+304 (+304)	68.82	25.89
58	seq=translation; coord=9:11496011..11502772:1; parent_transcript=GRMZM2G089713_T01; parent_gene=GRMZM2G089713	GRMZM2G089713_P01,GRMZM2G089713_P02	TRUE	TRUE	lGVTQcTIAHALEk	93%	n+304 (+304), Carbamidomethyl (+57), K+304 (+304)	26.46	25.01
59	seq=translation; coord=9:11496011..11502772:1; parent_transcript=GRMZM2G089713_T01; parent_gene=GRMZM2G089713	GRMZM2G089713_P01,GRMZM2G089713_P02	TRUE	TRUE	lLPDAAGTTcGQR	85%	n+304 (+304), Carbamidomethyl (+57)	25.05	25.00
60	seq=translation; coord=9:11496011..11502772:1; parent_transcript=GRMZM2G089713_T01; parent_gene=GRMZM2G089713	GRMZM2G089713_P01,GRMZM2G089713_P02	TRUE	TRUE	lTAFHPEIEELIYSDVENSEHk	95%	n+304 (+304), K+304 (+304)	37.51	25.00
61	seq=translation; coord=9:11496011..11502772:1; parent_transcript=GRMZM2G089713_T01; parent_gene=GRMZM2G089713	GRMZM2G089713_P01,GRMZM2G089713_P02	TRUE	TRUE	mYSLIDEYk	95%	n+304 (+304), K+304 (+304)	43.83	25.00
62	seq=translation; coord=9:11496011..11502772:1; parent_transcript=GRMZM2G089713_T01; parent_gene=GRMZM2G089713	GRMZM2G089713_P01,GRMZM2G089713_P02	TRUE	TRUE	nMTGLVEMYGk	95%	n+304 (+304), K+304 (+304)	42.22	25.05
63	seq=translation; coord=9:11496011..11502772:1; parent_transcript=GRMZM2G089713_T01; parent_gene=GRMZM2G089713	GRMZM2G089713_P01,GRMZM2G089713_P02	TRUE	TRUE	nmTGLVEMYGk	93%	n+304 (+304), Oxidation (+16), K+304 (+304)	28.72	25.00
64	seq=translation; coord=9:11496011..11502772:1; parent_transcript=GRMZM2G089713_T01; parent_gene=GRMZM2G089713	GRMZM2G089713_P01,GRMZM2G089713_P02	TRUE	TRUE	qQGLDITPk	95%	n+304 (+304), K+304 (+304)	36.03	25.69
65	seq=translation; coord=9:11496011..11502772:1; parent_transcript=GRMZM2G089713_T01; parent_gene=GRMZM2G089713	GRMZM2G089713_P01,GRMZM2G089713_P02	TRUE	TRUE	sLASQVPLSFD	95%	n+304 (+304)	30.22	25.19
66	seq=translation; coord=9:11496011..11502772:1; parent_transcript=GRMZM2G089713_T01; parent_gene=GRMZM2G089713	GRMZM2G089713_P01,GRMZM2G089713_P02	TRUE	TRUE	tkYPNSDIYLDk	94%	n+304 (+304), K+304 (+304), K+304 (+304)	27.79	25.45
67	seq=translation; coord=9:11496011..11502772:1; parent_transcript=GRMZM2G089713_T01; parent_gene=GRMZM2G089713	GRMZM2G089713_P01,GRMZM2G089713_P02	TRUE	TRUE	vIGTEHTDIIR	95%	n+304 (+304)	32.67	26.43
68	seq=translation; coord=9:11496011..11502772:1; parent_transcript=GRMZM2G089713_T01; parent_gene=GRMZM2G089713	GRMZM2G089713_P01,GRMZM2G089713_P02	TRUE	TRUE	vNVSELAVEELSVSEYLAFk	95%	n+304 (+304), K+304 (+304)	44.70	25.25
69	seq=translation; coord=9:11496011..11502772:1; parent_transcript=GRMZM2G089713_T01; parent_gene=GRMZM2G089713	GRMZM2G089713_P01,GRMZM2G089713_P02	TRUE	TRUE	yAPFEDILR	94%	n+304 (+304)	30.83	25.00
70	seq=translation; coord=9:11496011..11502772:1; parent_transcript=GRMZM2G089713_T01; parent_gene=GRMZM2G089713	GRMZM2G089713_P01,GRMZM2G089713_P02	TRUE	TRUE	yIEMFYALk	95%	n+304 (+304), K+304 (+304)	34.41	25.67

71	seq=translation; coord=9:22100864..22105552:1; parent_transcript=GRMZM2G064302_T01; parent_gene=GRMZM2G064302	GRMZM2G064302_P01	TRUE	TRUE	aGWGVMASHR	95%	n+304 (+304)	49.48	25.00
72	seq=translation; coord=9:22100864..22105552:1; parent_transcript=GRMZM2G064302_T01; parent_gene=GRMZM2G064302	GRMZM2G064302_P01	TRUE	TRUE	aVSNVNNIIGPAIVGk	93%	n+304 (+304), K+304 (+304)	27.13	25.00
73	seq=translation; coord=9:22100864..22105552:1; parent_transcript=GRMZM2G064302_T01; parent_gene=GRMZM2G064302	GRMZM2G064302_P01	TRUE	TRUE	aVTITWVk	95%	n+304 (+304), K+304 (+304)	32.04	25.76
74	seq=translation; coord=9:22100864..22105552:1; parent_transcript=GRMZM2G064302_T01; parent_gene=GRMZM2G064302	GRMZM2G064302_P01	TRUE	TRUE	gAVPSGASTGIYEALELR	95%	n+304 (+304)	72.41	25.47
75	seq=translation; coord=9:22100864..22105552:1; parent_transcript=GRMZM2G064302_T01; parent_gene=GRMZM2G064302	GRMZM2G064302_P01	TRUE	TRUE	gNPTVEVDVGLSDGSYAR	95%	n+304 (+304)	93.58	25.00
76	seq=translation; coord=9:22100864..22105552:1; parent_transcript=GRMZM2G064302_T01; parent_gene=GRMZM2G064302	GRMZM2G064302_P01	TRUE	TRUE	gNPTVEVDVGLSDGSyAR	95%	n+304 (+304), iTRAQ8plex (+304)	36.35	25.09
77	seq=translation; coord=9:22100864..22105552:1; parent_transcript=GRMZM2G064302_T01; parent_gene=GRMZM2G064302	GRMZM2G064302_P01	TRUE	TRUE	iEEELGDAAVYAGAk	95%	n+304 (+304), K+304 (+304)	31.25	25.85
78	seq=translation; coord=9:22100864..22105552:1; parent_transcript=GRMZM2G064302_T01; parent_gene=GRMZM2G064302	GRMZM2G064302_P01	TRUE	TRUE	iEEELGDAAVyAGAk	94%	n+304 (+304), iTRAQ8plex (+304), K+304 (+304)	28.00	25.72
79	seq=translation; coord=9:22100864..22105552:1; parent_transcript=GRMZM2G064302_T01; parent_gene=GRMZM2G064302	GRMZM2G064302_P01	TRUE	TRUE	lAMQEFMILPTGASSFk	95%	n+304 (+304), K+304 (+304)	39.90	25.81
80	seq=translation; coord=9:22100864..22105552:1; parent_transcript=GRMZM2G064302_T01; parent_gene=GRMZM2G064302	GRMZM2G064302_P01	TRUE	TRUE	lAmQEFMILPTGASSFk	95%	n+304 (+304), Oxidation (+16), K+304 (+304)	42.45	25.75
81	seq=translation; coord=9:22100864..22105552:1; parent_transcript=GRMZM2G064302_T01; parent_gene=GRMZM2G064302	GRMZM2G064302_P01	TRUE	TRUE	lAMQEFmILPTGASSFk	95%	n+304 (+304), Oxidation (+16), K+304 (+304)	38.48	25.88
82	seq=translation; coord=9:22100864..22105552:1; parent_transcript=GRMZM2G064302_T01; parent_gene=GRMZM2G064302	GRMZM2G064302_P01	TRUE	TRUE	lTDEIGQk	95%	n+304 (+304), K+304 (+304)	37.01	25.69
83	seq=translation; coord=9:22100864..22105552:1; parent_transcript=GRMZM2G064302_T01; parent_gene=GRMZM2G064302	GRMZM2G064302_P01	TRUE	TRUE	mGVEVYHNLk	95%	n+304 (+304), K+304 (+304)	43.63	26.16
84	seq=translation; coord=9:22100864..22105552:1; parent_transcript=GRMZM2G064302_T01; parent_gene=GRMZM2G064302	GRMZM2G064302_P01	TRUE	TRUE	sGETEDTFIADLSVGLSTGQIk	95%	n+304 (+304), K+304 (+304)	56.38	25.16
85	seq=translation; coord=9:22100864..22105552:1; parent_transcript=GRMZM2G064302_T01; parent_gene=GRMZM2G064302	GRMZM2G064302_P01	TRUE	TRUE	tcNALLLk	93%	n+304 (+304), Carbamidomethyl (+57), K+304 (+304)	29.81	25.00
86	seq=translation; coord=9:22100864..22105552:1; parent_transcript=GRMZM2G064302_T01; parent_gene=GRMZM2G064302	GRMZM2G064302_P01	TRUE	TRUE	vNQIGSVTESIEAVR	95%	n+304 (+304)	70.85	25.30
87	seq=translation; coord=9:22100864..22105552:1; parent_transcript=GRMZM2G064302_T01; parent_gene=GRMZM2G064302	GRMZM2G064302_P01	TRUE	TRUE	vQIVGDDLLVTNPTR	95%	n+304 (+304)	31.29	26.71
88	seq=translation; coord=9:22100864..22105552:1; parent_transcript=GRMZM2G064302_T01; parent_gene=GRMZM2G064302	GRMZM2G064302_P01	TRUE	TRUE	vVIGMDVAASEFFGEk	95%	n+304 (+304), K+304 (+304)	61.37	25.38

89	seq=translation; coord=9:22100864..22105552:1; parent_transcript=GRMZM2G064302_T01; parent_gene=GRMZM2G064302	GRMZM2G064302_P01	TRUE	TRUE	vVIGmDVAASEFFGEk	95%	n+304 (+304), Oxidation (+16), K+304 (+304)	34.42	25.40
90	seq=translation; coord=9:22100864..22105552:1; parent_transcript=GRMZM2G064302_T01; parent_gene=GRMZM2G064302	GRMZM2G064302_P01	TRUE	TRUE	vVIGMDVAASEFFGEkDk	95%	n+304 (+304), K+304 (+304), K+304 (+304)	45.72	26.12
91	seq=translation; coord=1:176837841..176844200:1; parent_transcript=GRMZM2G149751_T01; parent_gene=GRMZM2G149751	GRMZM2G149751_P01,GRMZM2G149751_P03, GRMZM2G149751_P05	TRUE	TRUE	aAGASWIQFDEPTLVLDLDSDk	95%	n+304 (+304), K+304 (+304)	63.94	25.55
92	seq=translation; coord=1:176837841..176844200:1; parent_transcript=GRMZM2G149751_T01; parent_gene=GRMZM2G149751	GRMZM2G149751_P01,GRMZM2G149751_P03, GRMZM2G149751_P05	TRUE	TRUE	aEHAFYLDWAVHSFR	95%	n+304 (+304)	58.42	25.00
93	seq=translation; coord=1:176837841..176844200:1; parent_transcript=GRMZM2G149751_T01; parent_gene=GRMZM2G149751	GRMZM2G149751_P01,GRMZM2G149751_P03, GRMZM2G149751_P05	TRUE	TRUE	eVIAELk	94%	n+304 (+304), K+304 (+304)	28.71	25.00
94	seq=translation; coord=1:176837841..176844200:1; parent_transcript=GRMZM2G149751_T01; parent_gene=GRMZM2G149751	GRMZM2G149751_P01,GRMZM2G149751_P03, GRMZM2G149751_P05	TRUE	TRUE	gFPLLSLLSSILPVYk	95%	n+304 (+304), K+304 (+304)	32.68	25.00
95	seq=translation; coord=1:176837841..176844200:1; parent_transcript=GRMZM2G149751_T01; parent_gene=GRMZM2G149751	GRMZM2G149751_P01,GRMZM2G149751_P03, GRMZM2G149751_P05	TRUE	TRUE	gTQTLGLVTSAGFPAGk	95%	n+304 (+304), K+304 (+304)	50.73	25.00
96	seq=translation; coord=1:176837841..176844200:1; parent_transcript=GRMZM2G149751_T01; parent_gene=GRMZM2G149751	GRMZM2G149751_P01,GRMZM2G149751_P03, GRMZM2G149751_P05	TRUE	TRUE	iPSAEEIADR	95%	n+304 (+304)	32.83	25.13
97	seq=translation; coord=1:176837841..176844200:1; parent_transcript=GRMZM2G149751_T01; parent_gene=GRMZM2G149751	GRMZM2G149751_P01,GRMZM2G149751_P03, GRMZM2G149751_P05	TRUE	TRUE	iSEEEYVTAIk	95%	n+304 (+304), K+304 (+304)	37.64	25.77
98	seq=translation; coord=1:176837841..176844200:1; parent_transcript=GRMZM2G149751_T01; parent_gene=GRMZM2G149751	GRMZM2G149751_P01,GRMZM2G149751_P03, GRMZM2G149751_P05	TRUE	TRUE	lNLPILPTTTIGSFPQTVELR	95%	n+304 (+304)	36.48	25.00
99	seq=translation; coord=1:176837841..176844200:1; parent_transcript=GRMZM2G149751_T01; parent_gene=GRMZM2G149751	GRMZM2G149751_P01,GRMZM2G149751_P03, GRMZM2G149751_P05	TRUE	TRUE	nIWADDLATSLSTLQSLEAVVGk	95%	n+304 (+304), K+304 (+304)	59.25	25.00
100	seq=translation; coord=1:176837841..176844200:1; parent_transcript=GRMZM2G149751_T01; parent_gene=GRMZM2G149751	GRMZM2G149751_P01,GRMZM2G149751_P03, GRMZM2G149751_P05	TRUE	TRUE	qMADAGIk	95%	n+304 (+304), K+304 (+304)	35.37	26.50
101	seq=translation; coord=1:176837841..176844200:1; parent_transcript=GRMZM2G149751_T01; parent_gene=GRMZM2G149751	GRMZM2G149751_P01,GRMZM2G149751_P03, GRMZM2G149751_P05	TRUE	TRUE	sTAEDLEk	95%	n+304 (+304), K+304 (+304)	32.28	25.51
102	seq=translation; coord=1:176837841..176844200:1; parent_transcript=GRMZM2G149751_T01; parent_gene=GRMZM2G149751	GRMZM2G149751_P01,GRMZM2G149751_P03, GRMZM2G149751_P05	TRUE	TRUE	tLTSLSSVTAYGFDLVR	95%	n+304 (+304)	63.90	25.37
103	seq=translation; coord=1:176837841..176844200:1; parent_transcript=GRMZM2G149751_T01; parent_gene=GRMZM2G149751	GRMZM2G149751_P01,GRMZM2G149751_P03, GRMZM2G149751_P05	TRUE	TRUE	tLTSLSSVTAyGFDLVR	95%	n+304 (+304), iTRAQ8plex (+304)	30.46	25.73
104	seq=translation; coord=1:176837841..176844200:1; parent_transcript=GRMZM2G149751_T01; parent_gene=GRMZM2G149751	GRMZM2G149751_P01,GRMZM2G149751_P03, GRMZM2G149751_P05	TRUE	TRUE	vVEVDALAk	95%	n+304 (+304), K+304 (+304)	44.89	26.14
105	seq=translation; coord=1:176837841..176844200:1; parent_transcript=GRMZM2G149751_T01; parent_gene=GRMZM2G149751	GRMZM2G149751_P01,GRMZM2G149751_P03, GRMZM2G149751_P05	TRUE	TRUE	wFDTNYHFIVPELGPNTk	95%	n+304 (+304), K+304 (+304)	33.31	25.00
106	seq=translation; coord=1:176837841..176844200:1; parent_transcript=GRMZM2G149751_T01; parent_gene=GRMZM2G149751	GRMZM2G149751_P01,GRMZM2G149751_P03, GRMZM2G149751_P05	TRUE	TRUE	ySWTGGEIGFDTYFSMAR	95%	n+304 (+304), iTRAQ8plex (+304)	32.96	25.00

107	seq=translation; coord=6:6898695..6903246:-1; parent_transcript=GRMZM2G180625_T01; parent_gene=GRMZM2G180625	GRMZM2G180625_P01,GRMZM2G180625_P02, GRMZM2G180625_P03	TRUE	TRUE	aGIALNDHFIk	95%	n+304 (+304), K+304 (+304)	34.23	25.00
108	seq=translation; coord=6:6898695..6903246:-1; parent_transcript=GRMZM2G180625_T01; parent_gene=GRMZM2G180625	GRMZM2G180625_P01,GRMZM2G180625_P02, GRMZM2G180625_P03	TRUE	TRUE	gASYEEIk	95%	n+304 (+304), K+304 (+304)	35.27	26.06
109	seq=translation; coord=6:6898695..6903246:-1; parent_transcript=GRMZM2G180625_T01; parent_gene=GRMZM2G180625	GRMZM2G180625_P01,GRMZM2G180625_P02, GRMZM2G180625_P03	TRUE	TRUE	gIMGYVEEDLVSTDFTGDSR	95%	n+304 (+304)	42.80	25.00
110	seq=translation; coord=6:6898695..6903246:-1; parent_transcript=GRMZM2G180625_T01; parent_gene=GRMZM2G180625	GRMZM2G180625_P01,GRMZM2G180625_P02, GRMZM2G180625_P03	TRUE	TRUE	gImGYVEEDLVSTDFTGDSR	95%	n+304 (+304), Oxidation (+16)	32.48	25.00
111	seq=translation; coord=6:6898695..6903246:-1; parent_transcript=GRMZM2G180625_T01; parent_gene=GRMZM2G180625	GRMZM2G180625_P01,GRMZM2G180625_P02, GRMZM2G180625_P03	TRUE	TRUE	gIMGyVEEDLVSTDFTGDSR	95%	n+304 (+304), iTRAQ8plex (+304)	32.72	25.00
112	seq=translation; coord=6:6898695..6903246:-1; parent_transcript=GRMZM2G180625_T01; parent_gene=GRMZM2G180625	GRMZM2G180625_P01,GRMZM2G180625_P02, GRMZM2G180625_P03	TRUE	TRUE	hSDIALk	95%	n+304 (+304), K+304 (+304)	28.78	25.00
113	seq=translation; coord=6:6898695..6903246:-1; parent_transcript=GRMZM2G180625_T01; parent_gene=GRMZM2G180625	GRMZM2G180625_P01,GRMZM2G180625_P02, GRMZM2G180625_P03	TRUE	TRUE	tLLFGEkPVTVFGIR	94%	n+304 (+304), K+304 (+304)	30.25	25.00
114	seq=translation; coord=6:6898695..6903246:-1; parent_transcript=GRMZM2G180625_T01; parent_gene=GRMZM2G180625	GRMZM2G180625_P01,GRMZM2G180625_P02, GRMZM2G180625_P03	TRUE	TRUE	vIHDNFGIIEGLMTTVHAITATQk	95%	n+304 (+304), K+304 (+304)	32.71	25.00
115	seq=translation; coord=1:296477860..296482170:1; parent_transcript=GRMZM2G040369_T01; parent_gene=GRMZM2G040369	GRMZM2G040369_P01,GRMZM2G040369_P02, GRMZM2G095851_P01,GRMZM2G095851_P02	TRUE	TRUE	aFLPVIESFGFSSQLR	95%	n+304 (+304)	61.54	25.91
116	seq=translation; coord=1:296477860..296482170:1; parent_transcript=GRMZM2G040369_T01; parent_gene=GRMZM2G040369	GRMZM2G040369_P01,GRMZM2G040369_P02, GRMZM2G095851_P01,GRMZM2G095851_P02	TRUE	TRUE	eQMTPLSEFEDk	95%	n+304 (+304), K+304 (+304)	35.90	25.00
117	seq=translation; coord=1:296477860..296482170:1; parent_transcript=GRMZM2G040369_T01; parent_gene=GRMZM2G040369	GRMZM2G040369_P01,GRMZM2G040369_P02, GRMZM2G095851_P01,GRMZM2G095851_P02	TRUE	TRUE	eQMTPLSEFEDkL	95%	n+304 (+304), K+304 (+304)	41.41	25.02
118	seq=translation; coord=1:296477860..296482170:1; parent_transcript=GRMZM2G040369_T01; parent_gene=GRMZM2G040369	GRMZM2G040369_P01,GRMZM2G040369_P02, GRMZM2G095851_P01,GRMZM2G095851_P02	TRUE	TRUE	fGVDETk	86%	n+304 (+304), K+304 (+304)	26.19	26.10
119	seq=translation; coord=4:14854376..14859167:-1; parent_transcript=GRMZM2G091481_T01; parent_gene=GRMZM2G091481	GRMZM2G091481_P01	TRUE	TRUE	aAAAEPVkDEL	94%	n+304 (+304), K+304 (+304)	30.52	25.16
120	seq=translation; coord=4:14854376..14859167:-1; parent_transcript=GRMZM2G091481_T01; parent_gene=GRMZM2G091481	GRMZM2G091481_P01	TRUE	TRUE	dFDVAALMk	95%	n+304 (+304), K+304 (+304)	36.54	26.68
121	seq=translation; coord=4:14854376..14859167:-1; parent_transcript=GRMZM2G091481_T01; parent_gene=GRMZM2G091481	GRMZM2G091481_P01	TRUE	TRUE	fIDASTIPR	88%	n+304 (+304)	26.32	25.00
122	seq=translation; coord=4:14854376..14859167:-1; parent_transcript=GRMZM2G091481_T01; parent_gene=GRMZM2G091481	GRMZM2G091481_P01	TRUE	TRUE	kLAPEYENAAk	95%	K+304 (+304), n+304 (+304), K+304 (+304)	31.89	25.00
123	seq=translation; coord=4:14854376..14859167:-1; parent_transcript=GRMZM2G091481_T01; parent_gene=GRMZM2G091481	GRMZM2G091481_P01	TRUE	TRUE	lAPEYENAAk	95%	n+304 (+304), K+304 (+304)	30.42	25.54
124	seq=translation; coord=4:14854376..14859167:-1; parent_transcript=GRMZM2G091481_T01; parent_gene=GRMZM2G091481	GRMZM2G091481_P01	TRUE	TRUE	lAPILDEAATTLQSDEEVVIAk	95%	n+304 (+304), K+304 (+304)	38.57	25.00

125	seq=translation; coord=4:14854376..14859167:-1; parent_transcript=GRMZM2G091481_T01; parent_gene=GRMZM2G091481	GRMZM2G091481_P01	TRUE	TRUE	mDATANDVPSEFDVQGYPTLYFVTPSGk	95%	n+304 (+304), K+304 (+304)	31.68	25.00
126	seq=translation; coord=4:14854376..14859167:-1; parent_transcript=GRMZM2G091481_T01; parent_gene=GRMZM2G091481	GRMZM2G091481_P01	TRUE	TRUE	mDATANDVPSEFDVQGYPTLYFVTPSGk	95%	Oxidation (+16), n+304 (+304), K+304 (+304)	36.09	25.00
127	seq=translation; coord=4:14854376..14859167:-1; parent_transcript=GRMZM2G091481_T01; parent_gene=GRMZM2G091481	GRMZM2G091481_P01	TRUE	TRUE	nPDNHPYLMk	95%	n+304 (+304), K+304 (+304)	31.09	25.00
128	seq=translation; coord=4:14854376..14859167:-1; parent_transcript=GRMZM2G091481_T01; parent_gene=GRMZM2G091481	GRMZM2G091481_P01	TRUE	TRUE	sAYSAAAEEFk	95%	n+304 (+304), K+304 (+304)	53.40	25.00
129	seq=translation; coord=4:14854376..14859167:-1; parent_transcript=GRMZM2G091481_T01; parent_gene=GRMZM2G091481	GRMZM2G091481_P01	TRUE	TRUE	tADDIVDFIk	95%	n+304 (+304), K+304 (+304)	33.75	26.53
130	seq=translation; coord=4:14854376..14859167:-1; parent_transcript=GRMZM2G091481_T01; parent_gene=GRMZM2G091481	GRMZM2G091481_P01	TRUE	TRUE	vVVADNVHDFVFk	95%	n+304 (+304), K+304 (+304)	29.77	25.76
131	seq=translation; coord=1:197267922..197271011:1; parent_transcript=GRMZM2G175423_T01; parent_gene=GRMZM2G175423	GRMZM2G175423_P01	TRUE	TRUE	aAMGSDIDVSLDcAGFSk	95%	n+304 (+304), Carbamidomethyl (+57), K+304 (+304)	52.54	25.00
132	seq=translation; coord=1:197267922..197271011:1; parent_transcript=GRMZM2G175423_T01; parent_gene=GRMZM2G175423	GRMZM2G175423_P01	TRUE	TRUE	aAmGSDIDVSLDcAGFSk	95%	n+304 (+304), Oxidation (+16), Carbamidomethyl (+57), K+304 (+304)	73.41	25.00
133	seq=translation; coord=1:197267922..197271011:1; parent_transcript=GRMZM2G175423_T01; parent_gene=GRMZM2G175423	GRMZM2G175423_P01	TRUE	TRUE	aGVGPETGVLVVGAGPIGLVSLLAAR	95%	n+304 (+304)	42.20	25.00
134	seq=translation; coord=1:197267922..197271011:1; parent_transcript=GRMZM2G175423_T01; parent_gene=GRMZM2G175423	GRMZM2G175423_P01	TRUE	TRUE	aVGIcGSDVHYLR	95%	n+304 (+304), Carbamidomethyl (+57)	56.07	25.16
135	seq=translation; coord=1:197267922..197271011:1; parent_transcript=GRMZM2G175423_T01; parent_gene=GRMZM2G175423	GRMZM2G175423_P01	TRUE	TRUE	dTWPLcIDFLR	95%	n+304 (+304), Carbamidomethyl (+57)	32.72	25.00
136	seq=translation; coord=1:197267922..197271011:1; parent_transcript=GRMZM2G175423_T01; parent_gene=GRMZM2G175423	GRMZM2G175423_P01	TRUE	TRUE	dVEEAFEVSAR	95%	n+304 (+304)	36.62	25.00
137	seq=translation; coord=1:197267922..197271011:1; parent_transcript=GRMZM2G175423_T01; parent_gene=GRMZM2G175423	GRMZM2G175423_P01	TRUE	TRUE	eVDVVGVFR	95%	n+304 (+304)	36.37	25.00
138	seq=translation; coord=1:197267922..197271011:1; parent_transcript=GRMZM2G175423_T01; parent_gene=GRMZM2G175423	GRMZM2G175423_P01	TRUE	TRUE	gAQGSDAAAAGGEVEENMAAWLVAk	95%	n+304 (+304), K+304 (+304)	77.37	25.00
139	seq=translation; coord=1:197267922..197271011:1; parent_transcript=GRMZM2G175423_T01; parent_gene=GRMZM2G175423	GRMZM2G175423_P01	TRUE	TRUE	iAHFVVk	91%	n+304 (+304), K+304 (+304)	26.11	25.00
140	seq=translation; coord=1:197267922..197271011:1; parent_transcript=GRMZM2G175423_T01; parent_gene=GRMZM2G175423	GRMZM2G175423_P01	TRUE	TRUE	lPPVGPYDVR	95%	n+304 (+304)	33.40	25.25
141	seq=translation; coord=1:197267922..197271011:1; parent_transcript=GRMZM2G175423_T01; parent_gene=GRMZM2G175423	GRMZM2G175423_P01	TRUE	TRUE	rAGVGPETGVLVVGAGPIGLVSLLAAR	95%	n+304 (+304)	54.61	25.00
142	seq=translation; coord=1:197267922..197271011:1; parent_transcript=GRMZM2G175423_T01; parent_gene=GRMZM2G175423	GRMZM2G175423_P01	TRUE	TRUE	sLGADAAVR	95%	n+304 (+304)	36.93	26.63

143	seq=translation; coord=1:197267922..197271011:1; parent_transcript=GRMZM2G175423_T01; parent_gene=GRMZM2G175423	GRMZM2G175423_P01	TRUE	TRUE	vALEPGVScWR	95%	n+304 (+304), Carbamidomethyl (+57)	35.09	25.00
144	seq=translation; coord=1:197267922..197271011:1; parent_transcript=GRMZM2G175423_T01; parent_gene=GRMZM2G175423	GRMZM2G175423_P01	TRUE	TRUE	vcLVGMGHNEMTLPLTAAAAR	95%	n+304 (+304), Carbamidomethyl (+57)	47.28	25.00
145	seq=translation; coord=1:197267922..197271011:1; parent_transcript=GRMZM2G175423_T01; parent_gene=GRMZM2G175423	GRMZM2G175423_P01	TRUE	TRUE	vcLVGmGHNEMTLPLTAAAAR	95%	n+304 (+304), Carbamidomethyl (+57), Oxidation (+16)	35.06	25.00
146	seq=translation; coord=1:197267922..197271011:1; parent_transcript=GRMZM2G175423_T01; parent_gene=GRMZM2G175423	GRMZM2G175423_P01	TRUE	TRUE	vcLVGMGHNEmTLPLTAAAAR	95%	n+304 (+304), Carbamidomethyl (+57), Oxidation (+16)	54.34	25.00
147	seq=translation; coord=1:197267922..197271011:1; parent_transcript=GRMZM2G175423_T01; parent_gene=GRMZM2G175423	GRMZM2G175423_P01	TRUE	TRUE	vVVVDVDDHR	95%	n+304 (+304)	50.75	25.47
148	seq=translation; coord=1:197267922..197271011:1; parent_transcript=GRMZM2G175423_T01; parent_gene=GRMZM2G175423	GRMZM2G175423_P01	TRUE	TRUE	ykDTWPLcIDFLR	94%	n+304 (+304), K+304 (+304), Carbamidomethyl (+57)	29.13	25.99
149	seq=translation; coord=1:197267922..197271011:1; parent_transcript=GRMZM2G175423_T01; parent_gene=GRMZM2G175423	GRMZM2G175423_P01	TRUE	TRUE	yNLcEDMk	88%	n+304 (+304), Carbamidomethyl (+57), K+304 (+304)	26.10	25.00
150	seq=translation; coord=3:126460606..126465045:-1; parent_transcript=GRMZM2G340251_T01; parent_gene=GRMZM2G340251	GRMZM2G340251_P01,GRMZM2G340251_P02, GRMZM2G340251_P03	TRUE	TRUE	kIEDAIDAAISWLDANQLAEADEFEDk	95%	K+304 (+304), n+304 (+304), K+304 (+304)	31.25	25.00
151	seq=translation; coord=3:126460606..126465045:-1; parent_transcript=GRMZM2G340251_T01; parent_gene=GRMZM2G340251	GRMZM2G340251_P01,GRMZM2G340251_P02, GRMZM2G340251_P03	TRUE	TRUE	mYQGAGEDMGGAGGMDEDAPAGSGGPGPk	95%	n+304 (+304), K+304 (+304)	85.45	25.00
152	seq=translation; coord=3:126460606..126465045:-1; parent_transcript=GRMZM2G340251_T01; parent_gene=GRMZM2G340251	GRMZM2G340251_P01,GRMZM2G340251_P02, GRMZM2G340251_P03	TRUE	TRUE	nSLENYSYNMR	95%	n+304 (+304)	37.71	25.00
153	seq=translation; coord=3:207616747..207621242:-1; parent_transcript=GRMZM2G041275_T01; parent_gene=GRMZM2G041275	GRMZM2G041275_P01	TRUE	TRUE	fDEGLPPILTALEVLDNNIR	95%	n+304 (+304)	59.91	25.25
154	seq=translation; coord=5:167751373..167752582:1; parent_transcript=GRMZM2G326111_T01; parent_gene=GRMZM2G326111	GRMZM2G326111_P01	TRUE	TRUE	hVVFGQVVEGMDVVk	95%	n+304 (+304), K+304 (+304)	60.28	25.01
155	seq=translation; coord=5:167751373..167752582:1; parent_transcript=GRMZM2G326111_T01; parent_gene=GRMZM2G326111	GRMZM2G326111_P01	TRUE	TRUE	hVVFGQVVEGmDVVk	95%	n+304 (+304), Oxidation (+16), K+304 (+304)	41.29	25.84
156	seq=translation; coord=5:167751373..167752582:1; parent_transcript=GRMZM2G326111_T01; parent_gene=GRMZM2G326111	GRMZM2G326111_P01	TRUE	TRUE	vADcGQLS	95%	n+304 (+304), Carbamidomethyl (+57)	51.57	25.00
157	seq=translation; coord=5:167751373..167752582:1; parent_transcript=GRMZM2G326111_T01; parent_gene=GRMZM2G326111	GRMZM2G326111_P01	TRUE	TRUE	vFFDMTVGGAPAGR	95%	n+304 (+304)	49.09	25.00
158	seq=translation; coord=5:167751373..167752582:1; parent_transcript=GRMZM2G326111_T01; parent_gene=GRMZM2G326111	GRMZM2G326111_P01	TRUE	TRUE	vFFDmTVGGAPAGR	95%	n+304 (+304), Oxidation (+16)	49.58	25.00
159	seq=translation; coord=5:167751373..167752582:1; parent_transcript=GRMZM2G326111_T01; parent_gene=GRMZM2G326111	GRMZM2G326111_P01	TRUE	TRUE	vIPEFMcQGGDFTR	95%	n+304 (+304), Carbamidomethyl (+57)	74.65	25.00
160	seq=translation; coord=5:167751373..167752582:1; parent_transcript=GRMZM2G326111_T01; parent_gene=GRMZM2G326111	GRMZM2G326111_P01	TRUE	TRUE	vIPEFmcQGGDFTR	95%	n+304 (+304), Oxidation (+16), Carbamidomethyl (+57)	64.65	25.00

161	seq=translation; coord=4:240050091..240053865:1; parent_transcript=GRMZM2G415007_T01; parent_gene=GRMZM2G415007	GRMZM2G415007_P01	TRUE	TRUE	aMEDAGLEk	95%	n+304 (+304), K+304 (+304)	48.21	25.00
162	seq=translation; coord=4:240050091..240053865:1; parent_transcript=GRMZM2G415007_T01; parent_gene=GRMZM2G415007	GRMZM2G415007_P01	TRUE	TRUE	dAGVIAGLNVAR	95%	n+304 (+304)	42.06	26.62
163	seq=translation; coord=4:240050091..240053865:1; parent_transcript=GRMZM2G415007_T01; parent_gene=GRMZM2G415007	GRMZM2G415007_P01	TRUE	TRUE	dILLLDVAPLTLGIETVGGVMTk	95%	n+304 (+304), K+304 (+304)	41.60	25.00
164	seq=translation; coord=4:240050091..240053865:1; parent_transcript=GRMZM2G415007_T01; parent_gene=GRMZM2G415007	GRMZM2G415007_P01	TRUE	TRUE	dILLLDVAPLTLGIETVGGVmTk	95%	n+304 (+304), Oxidation (+16), K+304 (+304)	47.82	25.00
165	seq=translation; coord=4:240050091..240053865:1; parent_transcript=GRMZM2G415007_T01; parent_gene=GRMZM2G415007	GRMZM2G415007_P01	TRUE	TRUE	eAEEFAEEDk	95%	n+304 (+304), K+304 (+304)	51.02	25.00
166	seq=translation; coord=4:240050091..240053865:1; parent_transcript=GRMZM2G415007_T01; parent_gene=GRMZM2G415007	GRMZM2G415007_P01	TRUE	TRUE	eALEWLDDNQSAEk	95%	n+304 (+304), K+304 (+304)	57.75	25.00
167	seq=translation; coord=4:240050091..240053865:1; parent_transcript=GRMZM2G415007_T01; parent_gene=GRMZM2G415007	GRMZM2G415007_P01	TRUE	TRUE	eALEWLDDNQSAEkEDYEEk	95%	n+304 (+304), K+304 (+304), K+304 (+304)	30.81	25.00
168	seq=translation; coord=4:240050091..240053865:1; parent_transcript=GRMZM2G415007_T01; parent_gene=GRMZM2G415007	GRMZM2G415007_P01	TRUE	TRUE	eVEAVcNPIVSAVYQR	95%	n+304 (+304), Carbamidomethyl (+57)	66.19	25.00
169	seq=translation; coord=4:240050091..240053865:1; parent_transcript=GRMZM2G415007_T01; parent_gene=GRMZM2G415007	GRMZM2G415007_P01	TRUE	TRUE	fEELNNDLFR	95%	n+304 (+304)	33.48	25.00
170	seq=translation; coord=4:240050091..240053865:1; parent_transcript=GRMZM2G415007_T01; parent_gene=GRMZM2G415007	GRMZM2G415007_P01	TRUE	TRUE	iMEYFIk	95%	n+304 (+304), K+304 (+304)	37.24	25.29
171	seq=translation; coord=4:240050091..240053865:1; parent_transcript=GRMZM2G415007_T01; parent_gene=GRMZM2G415007	GRMZM2G415007_P01	TRUE	TRUE	iNDAVVTVPAYFNDAQR	95%	n+304 (+304)	52.12	25.00
172	seq=translation; coord=4:240050091..240053865:1; parent_transcript=GRMZM2G415007_T01; parent_gene=GRMZM2G415007	GRMZM2G415007_P01	TRUE	TRUE	iTPSWVAFTDSER	95%	n+304 (+304)	47.75	25.00
173	seq=translation; coord=4:240050091..240053865:1; parent_transcript=GRMZM2G415007_T01; parent_gene=GRMZM2G415007	GRMZM2G415007_P01	TRUE	TRUE	lkEVEAVcNPIVSAVYQR	95%	n+304 (+304), K+304 (+304), Carbamidomethyl (+57)	44.46	25.00
174	seq=translation; coord=4:240050091..240053865:1; parent_transcript=GRMZM2G415007_T01; parent_gene=GRMZM2G415007	GRMZM2G415007_P01	TRUE	TRUE	nQLETYVYNMk	93%	n+304 (+304), K+304 (+304)	27.40	25.07
175	seq=translation; coord=4:240050091..240053865:1; parent_transcript=GRMZM2G415007_T01; parent_gene=GRMZM2G415007	GRMZM2G415007_P01	TRUE	TRUE	sGGAPGGDADGGVDDDHDEL	95%	n+304 (+304)	38.42	25.00
176	seq=translation; coord=4:240050091..240053865:1; parent_transcript=GRMZM2G415007_T01; parent_gene=GRMZM2G415007	GRMZM2G415007_P01	TRUE	TRUE	sQIHEIVLVGGSTR	95%	n+304 (+304)	41.57	26.16
177	seq=translation; coord=4:240050091..240053865:1; parent_transcript=GRMZM2G415007_T01; parent_gene=GRMZM2G415007	GRMZM2G415007_P01	TRUE	TRUE	sQVFTTYQDQQTTVSIQVFEGER	90%	n+304 (+304)	26.49	25.00
178	seq=translation; coord=4:240050091..240053865:1; parent_transcript=GRMZM2G415007_T01; parent_gene=GRMZM2G415007	GRMZM2G415007_P01	TRUE	TRUE	tIFDVk	90%	n+304 (+304), K+304 (+304)	27.25	25.53

179	seq=translation; coord=4:240050091..240053865:1; parent_transcript=GRMZM2G415007_T01; parent_gene=GRMZM2G415007	GRMZM2G415007_P01	TRUE	TRUE	vEIESLFDGTDFSEPLTR	95%	n+304 (+304)	64.01	25.00
180	seq=translation; coord=4:240050091..240053865:1; parent_transcript=GRMZM2G415007_T01; parent_gene=GRMZM2G415007	GRMZM2G415007_P01	TRUE	TRUE	vFSPEEISAMILGk	95%	n+304 (+304), K+304 (+304)	49.46	25.66
181	seq=translation; coord=4:240050091..240053865:1; parent_transcript=GRMZM2G415007_T01; parent_gene=GRMZM2G415007	GRMZM2G415007_P01	TRUE	TRUE	vFSPEEISAmILGk	95%	n+304 (+304), Oxidation (+16), K+304 (+304)	34.64	25.47
182	seq=translation; coord=3:165722970..165725581:1; parent_transcript=GRMZM2G057823_T01; parent_gene=GRMZM2G057823	GRMZM2G057823_P01	TRUE	TRUE	kPWSLSFSFGR	94%	K+304 (+304), n+304 (+304)	26.16	25.87
183	seq=translation; coord=3:165722970..165725581:1; parent_transcript=GRMZM2G057823_T01; parent_gene=GRMZM2G057823	GRMZM2G057823_P01	TRUE	TRUE	kVTPEVIAEYTVR	91%	K+304 (+304), n+304 (+304)	27.74	25.00
184	seq=translation; coord=3:165722970..165725581:1; parent_transcript=GRMZM2G057823_T01; parent_gene=GRMZM2G057823	GRMZM2G057823_P01	TRUE	TRUE	nLNAMNk	89%	n+304 (+304), K+304 (+304)	28.67	26.70
185	seq=translation; coord=3:165722970..165725581:1; parent_transcript=GRMZM2G057823_T01; parent_gene=GRMZM2G057823	GRMZM2G057823_P01	TRUE	TRUE	tVPAAVPAVVFLSGGQSEEEATR	95%	n+304 (+304)	32.80	25.38
186	seq=translation; coord=3:165722970..165725581:1; parent_transcript=GRMZM2G057823_T01; parent_gene=GRMZM2G057823	GRMZM2G057823_P01	TRUE	TRUE	vENLEk	91%	n+304 (+304), K+304 (+304)	30.55	27.77
187	seq=translation; coord=3:165722970..165725581:1; parent_transcript=GRMZM2G057823_T01; parent_gene=GRMZM2G057823	GRMZM2G057823_P01	TRUE	TRUE	vTPEVIAEYTVR	95%	n+304 (+304)	44.81	25.00
188	seq=translation; coord=3:165722970..165725581:1; parent_transcript=GRMZM2G057823_T01; parent_gene=GRMZM2G057823	GRMZM2G057823_P01	TRUE	TRUE	vTPEVIAEyTVR	94%	n+304 (+304), iTRAQ8plex (+304)	29.97	25.31
189	seq=translation; coord=4:198369976..198374059:-1; parent_transcript=GRMZM2G012631_T01; parent_gene=GRMZM2G012631	GRMZM2G012631_P01	TRUE	TRUE	aNNTLTIIDSGIGMTk	91%	n+304 (+304), K+304 (+304)	27.39	26.25
190	seq=translation; coord=4:198369976..198374059:-1; parent_transcript=GRMZM2G012631_T01; parent_gene=GRMZM2G012631	GRMZM2G012631_P01	TRUE	TRUE	eVSHEWQLVNk	95%	n+304 (+304), K+304 (+304)	32.29	25.74
191	seq=translation; coord=4:198369976..198374059:-1; parent_transcript=GRMZM2G012631_T01; parent_gene=GRMZM2G012631	GRMZM2G012631_P01	TRUE	TRUE	gYEVLFMVDAIDEySIGQLk	95%	n+304 (+304), iTRAQ8plex (+304), K+304 (+304)	37.92	25.00
192	seq=translation; coord=4:198369976..198374059:-1; parent_transcript=GRMZM2G012631_T01; parent_gene=GRMZM2G012631	GRMZM2G012631_P01	TRUE	TRUE	hNDDEQYVWESQAGGSFTVAR	95%	n+304 (+304)	43.38	25.00
193	seq=translation; coord=4:198369976..198374059:-1; parent_transcript=GRMZM2G012631_T01; parent_gene=GRMZM2G012631	GRMZM2G012631_P01	TRUE	TRUE	hSEFISYPISLWIEk	95%	n+304 (+304), K+304 (+304)	29.92	25.51
194	seq=translation; coord=4:198369976..198374059:-1; parent_transcript=GRMZM2G012631_T01; parent_gene=GRMZM2G012631	GRMZM2G012631_P01	TRUE	TRUE	kTMEINPENAIMEELR	95%	K+304 (+304), n+304 (+304)	72.86	25.73
195	seq=translation; coord=4:198369976..198374059:-1; parent_transcript=GRMZM2G012631_T01; parent_gene=GRMZM2G012631	GRMZM2G012631_P01	TRUE	TRUE	lDESEDEk	95%	n+304 (+304), K+304 (+304)	41.39	25.00
196	seq=translation; coord=4:198369976..198374059:-1; parent_transcript=GRMZM2G012631_T01; parent_gene=GRMZM2G012631	GRMZM2G012631_P01	TRUE	TRUE	mTLYLkDDQLEYLEER	93%	n+304 (+304), K+304 (+304)	27.05	25.37

197	seq=translation; coord=4:198369976..198374059:-1; parent_transcript=GRMZM2G012631_T01; parent_gene=GRMZM2G012631	GRMZM2G012631_P01	TRUE	TRUE	tMEINPENAIMEELR	95%	n+304 (+304)	44.66	25.00
198	seq=translation; coord=4:198369976..198374059:-1; parent_transcript=GRMZM2G012631_T01; parent_gene=GRMZM2G012631	GRMZM2G012631_P01	TRUE	TRUE	tmEINPENAIMEELR	95%	n+304 (+304), Oxidation (+16)	57.21	25.00
199	seq=translation; coord=4:21135870..21139019:1; parent_transcript=GRMZM2G015295_T01; parent_gene=GRMZM2G015295	GRMZM2G015295_P01,GRMZM2G015295_P02	TRUE	TRUE	aEEDYEk	95%	n+304 (+304), K+304 (+304)	38.20	25.00
200	seq=translation; coord=4:21135870..21139019:1; parent_transcript=GRMZM2G015295_T01; parent_gene=GRMZM2G015295	GRMZM2G015295_P01,GRMZM2G015295_P02	TRUE	TRUE	aTDVMIAGk	95%	n+304 (+304), K+304 (+304)	41.31	26.61
201	seq=translation; coord=4:21135870..21139019:1; parent_transcript=GRMZM2G015295_T01; parent_gene=GRMZM2G015295	GRMZM2G015295_P01,GRMZM2G015295_P02	TRUE	TRUE	dIIMVDHMR	95%	n+304 (+304)	65.07	25.00
202	seq=translation; coord=4:21135870..21139019:1; parent_transcript=GRMZM2G015295_T01; parent_gene=GRMZM2G015295	GRMZM2G015295_P01,GRMZM2G015295_P02	TRUE	TRUE	dLSQADFGR	93%	n+304 (+304)	29.43	25.00
203	seq=translation; coord=4:21135870..21139019:1; parent_transcript=GRMZM2G015295_T01; parent_gene=GRMZM2G015295	GRMZM2G015295_P01,GRMZM2G015295_P02	TRUE	TRUE	dSAAVFAWk	95%	n+304 (+304), K+304 (+304)	35.13	26.69
204	seq=translation; coord=4:21135870..21139019:1; parent_transcript=GRMZM2G015295_T01; parent_gene=GRMZM2G015295	GRMZM2G015295_P01,GRMZM2G015295_P02	TRUE	TRUE	gcAAALk	89%	n+304 (+304), Carbamidomethyl (+57), K+304 (+304)	28.11	26.54
205	seq=translation; coord=4:21135870..21139019:1; parent_transcript=GRMZM2G015295_T01; parent_gene=GRMZM2G015295	GRMZM2G015295_P01,GRMZM2G015295_P02	TRUE	TRUE	gETLEEYWWcTER	95%	n+304 (+304), Carbamidomethyl (+57)	46.24	25.00
206	seq=translation; coord=4:21135870..21139019:1; parent_transcript=GRMZM2G015295_T01; parent_gene=GRMZM2G015295	GRMZM2G015295_P01,GRMZM2G015295_P02	TRUE	TRUE	hSLPDGLMR	95%	n+304 (+304)	36.87	25.04
207	seq=translation; coord=4:21135870..21139019:1; parent_transcript=GRMZM2G015295_T01; parent_gene=GRMZM2G015295	GRMZM2G015295_P01,GRMZM2G015295_P02	TRUE	TRUE	iPDPESTDNAEFk	95%	n+304 (+304), K+304 (+304)	30.77	25.00
208	seq=translation; coord=4:21135870..21139019:1; parent_transcript=GRMZM2G015295_T01; parent_gene=GRMZM2G015295	GRMZM2G015295_P01,GRMZM2G015295_P02	TRUE	TRUE	iVLTIIR	95%	n+304 (+304)	30.38	25.00
209	seq=translation; coord=4:21135870..21139019:1; parent_transcript=GRMZM2G015295_T01; parent_gene=GRMZM2G015295	GRMZM2G015295_P01,GRMZM2G015295_P02	TRUE	TRUE	lEIELAEVEMPGLMAcR	95%	n+304 (+304), Carbamidomethyl (+57)	63.81	25.00
210	seq=translation; coord=4:21135870..21139019:1; parent_transcript=GRMZM2G015295_T01; parent_gene=GRMZM2G015295	GRMZM2G015295_P01,GRMZM2G015295_P02	TRUE	TRUE	lEIELAEVEmPGLMAcR	91%	n+304 (+304), Oxidation (+16), Carbamidomethyl (+57)	26.22	25.00
211	seq=translation; coord=4:21135870..21139019:1; parent_transcript=GRMZM2G015295_T01; parent_gene=GRMZM2G015295	GRMZM2G015295_P01,GRMZM2G015295_P02	TRUE	TRUE	lEIELAEVEMPGLmAcR	94%	n+304 (+304), Oxidation (+16), Carbamidomethyl (+57)	30.24	25.00
212	seq=translation; coord=4:21135870..21139019:1; parent_transcript=GRMZM2G015295_T01; parent_gene=GRMZM2G015295	GRMZM2G015295_P01,GRMZM2G015295_P02	TRUE	TRUE	lEIELAEVEmPGLmAcR	95%	n+304 (+304), Oxidation (+16), Oxidation (+16), Carbamidomethyl (+57)	40.36	25.00
213	seq=translation; coord=4:21135870..21139019:1; parent_transcript=GRMZM2G015295_T01; parent_gene=GRMZM2G015295	GRMZM2G015295_P01,GRMZM2G015295_P02	TRUE	TRUE	lVGVSEETTTGVk	95%	n+304 (+304), K+304 (+304)	66.77	26.56
214	seq=translation; coord=4:21135870..21139019:1; parent_transcript=GRMZM2G015295_T01; parent_gene=GRMZM2G015295	GRMZM2G015295_P01,GRMZM2G015295_P02	TRUE	TRUE	lYQMQETGALLFPAINVNDSVTk	95%	n+304 (+304), K+304 (+304)	77.73	25.22

215	seq=translation; coord=4:21135870..21139019:1; parent_transcript=GRMZM2G015295_T01; parent_gene=GRMZM2G015295	GRMZM2G015295_P01,GRMZM2G015295_P02	TRUE	TRUE	nNAIVcNIGHFDNEIDMLGLETYPGVk	95%	n+304 (+304), Carbamidomethyl (+57), K+304 (+304)	50.74	25.00
216	seq=translation; coord=4:21135870..21139019:1; parent_transcript=GRMZM2G015295_T01; parent_gene=GRMZM2G015295	GRMZM2G015295_P01,GRMZM2G015295_P02	TRUE	TRUE	vAALHLGk	95%	n+304 (+304), K+304 (+304)	33.11	25.00
217	seq=translation; coord=4:21135870..21139019:1; parent_transcript=GRMZM2G015295_T01; parent_gene=GRMZM2G015295	GRMZM2G015295_P01,GRMZM2G015295_P02	TRUE	TRUE	vAVVcGYGDVGk	95%	n+304 (+304), Carbamidomethyl (+57), K+304 (+304)	58.88	25.88
218	seq=translation; coord=4:21135870..21139019:1; parent_transcript=GRMZM2G015295_T01; parent_gene=GRMZM2G015295	GRMZM2G015295_P01,GRMZM2G015295_P02	TRUE	TRUE	wcScNIFSTQDHAAAAIAR	95%	n+304 (+304), Carbamidomethyl (+57), Carbamidomethyl (+57)	51.69	25.00
219	seq=translation; coord=4:21135870..21139019:1; parent_transcript=GRMZM2G015295_T01; parent_gene=GRMZM2G015295	GRMZM2G015295_P01,GRMZM2G015295_P02	TRUE	TRUE	wVFPETNTGIIVLAEGR	95%	n+304 (+304)	53.80	25.20
220	seq=translation; coord=6:39891824..39893787:1; parent_transcript=GRMZM2G174883_T01; parent_gene=GRMZM2G174883	GRMZM2G174883_P01	TRUE	TRUE	aGQLLIVPQGYLVATk	95%	n+304 (+304), K+304 (+304)	48.21	25.00
221	seq=translation; coord=6:39891824..39893787:1; parent_transcript=GRMZM2G174883_T01; parent_gene=GRMZM2G174883	GRMZM2G174883_P01	TRUE	TRUE	aQGEGFQYIAFETNPDTMVSHVAGk	95%	n+304 (+304), K+304 (+304)	98.46	25.00
222	seq=translation; coord=6:39891824..39893787:1; parent_transcript=GRMZM2G174883_T01; parent_gene=GRMZM2G174883	GRMZM2G174883_P01	TRUE	TRUE	eLTcAGIFAVR	95%	n+304 (+304), Carbamidomethyl (+57)	65.08	25.00
223	seq=translation; coord=6:39891824..39893787:1; parent_transcript=GRMZM2G174883_T01; parent_gene=GRMZM2G174883	GRMZM2G174883_P01	TRUE	TRUE	fLLAGGFSk	95%	n+304 (+304), K+304 (+304)	36.79	25.00
224	seq=translation; coord=6:39891824..39893787:1; parent_transcript=GRMZM2G174883_T01; parent_gene=GRMZM2G174883	GRMZM2G174883_P01	TRUE	TRUE	fLSEALGVSMHVAEk	95%	n+304 (+304), K+304 (+304)	31.18	25.67
225	seq=translation; coord=6:39891824..39893787:1; parent_transcript=GRMZM2G174883_T01; parent_gene=GRMZM2G174883	GRMZM2G174883_P01	TRUE	TRUE	fPVLNLVQMSAVR	95%	n+304 (+304)	37.06	25.43
226	seq=translation; coord=6:39891824..39893787:1; parent_transcript=GRMZM2G174883_T01; parent_gene=GRMZM2G174883	GRMZM2G174883_P01	TRUE	TRUE	gQPHFAENIFk	95%	n+304 (+304), K+304 (+304)	36.09	26.56
227	seq=translation; coord=6:39891824..39893787:1; parent_transcript=GRMZM2G174883_T01; parent_gene=GRMZM2G174883	GRMZM2G174883_P01	TRUE	TRUE	hNVcAMEVR	95%	n+304 (+304), Carbamidomethyl (+57)	31.54	25.00
228	seq=translation; coord=6:39891824..39893787:1; parent_transcript=GRMZM2G174883_T01; parent_gene=GRMZM2G174883	GRMZM2G174883_P01	TRUE	TRUE	kHELAVLTPAGSGSYQQGQAGSAQQ	94%	K+304 (+304), n+304 (+304)	25.82	25.72
229	seq=translation; coord=6:39891824..39893787:1; parent_transcript=GRMZM2G174883_T01; parent_gene=GRMZM2G174883	GRMZM2G174883_P01	TRUE	TRUE	lDQADVYSPGAGR	95%	n+304 (+304)	40.33	25.00
230	seq=translation; coord=6:39891824..39893787:1; parent_transcript=GRMZM2G174883_T01; parent_gene=GRMZM2G174883	GRMZM2G174883_P01	TRUE	TRUE	lDQADVySPGAGR	95%	n+304 (+304), iTRAQ8plex (+304)	38.05	25.00
231	seq=translation; coord=6:39891824..39893787:1; parent_transcript=GRMZM2G174883_T01; parent_gene=GRMZM2G174883	GRMZM2G174883_P01	TRUE	TRUE	lEALEPR	88%	n+304 (+304)	27.04	26.39
232	seq=translation; coord=6:39891824..39893787:1; parent_transcript=GRMZM2G174883_T01; parent_gene=GRMZM2G174883	GRMZM2G174883_P01	TRUE	TRUE	nSVLSDLPAAVIASSYAISMEEAAELk	95%	n+304 (+304), K+304 (+304)	37.01	25.00

233	seq=translation; coord=6:39891824..39893787:1; parent_transcript=GRMZM2G174883_T01; parent_gene=GRMZM2G174883	GRMZM2G174883_P01	TRUE	TRUE	nSVLSDLPAAVIASSYAISmEEAAELk	95%	n+304 (+304), Oxidation (+16), K+304 (+304)	49.15	25.12
234	seq=translation; coord=6:39891824..39893787:1; parent_transcript=GRMZM2G174883_T01; parent_gene=GRMZM2G174883	GRMZM2G174883_P01	TRUE	TRUE	vDLYQDAIMSPFWNFNAHSAMYGIR	95%	n+304 (+304)	79.93	25.00
235	seq=translation; coord=6:39891824..39893787:1; parent_transcript=GRMZM2G174883_T01; parent_gene=GRMZM2G174883	GRMZM2G174883_P01	TRUE	TRUE	vQSEAGSVQYFSR	95%	n+304 (+304)	49.26	25.00
236	seq=translation; coord=6:39891824..39893787:1; parent_transcript=GRMZM2G174883_T01; parent_gene=GRMZM2G174883	GRMZM2G174883_P01	TRUE	TRUE	vQSEAGSVQyFSR	95%	n+304 (+304), iTRAQ8plex (+304)	41.27	25.62
237	seq=translation; coord=6:39891824..39893787:1; parent_transcript=GRMZM2G174883_T01; parent_gene=GRMZM2G174883	GRMZM2G174883_P01	TRUE	TRUE	vVVDAMGLLLPR	95%	n+304 (+304)	60.02	25.16
238	seq=translation; coord=4:28118892..28124783:1; parent_transcript=GRMZM2G020801_T01; parent_gene=GRMZM2G020801	GRMZM2G020801_P01	TRUE	TRUE	aGEDADSLGLTGHER	95%	n+304 (+304)	72.80	25.00
239	seq=translation; coord=4:28118892..28124783:1; parent_transcript=GRMZM2G020801_T01; parent_gene=GRMZM2G020801	GRMZM2G020801_P01	TRUE	TRUE	aTIANMSPEYGATMGFFPVDHVTLDyLk	95%	n+304 (+304), iTRAQ8plex (+304), K+304 (+304)	32.16	25.00
240	seq=translation; coord=4:28118892..28124783:1; parent_transcript=GRMZM2G020801_T01; parent_gene=GRMZM2G020801	GRMZM2G020801_P01	TRUE	TRUE	dIWPSTEEIAQVVQSSVLPDMFk	95%	n+304 (+304), K+304 (+304)	29.46	25.71
241	seq=translation; coord=4:28118892..28124783:1; parent_transcript=GRMZM2G020801_T01; parent_gene=GRMZM2G020801	GRMZM2G020801_P01	TRUE	TRUE	dMTMSPPGPSTVk	94%	n+304 (+304), K+304 (+304)	28.99	25.35
242	seq=translation; coord=4:28118892..28124783:1; parent_transcript=GRMZM2G020801_T01; parent_gene=GRMZM2G020801	GRMZM2G020801_P01	TRUE	TRUE	fDFHGQPAEMk	95%	n+304 (+304), K+304 (+304)	29.46	25.00
243	seq=translation; coord=4:28118892..28124783:1; parent_transcript=GRMZM2G020801_T01; parent_gene=GRMZM2G020801	GRMZM2G020801_P01	TRUE	TRUE	fVEFYGEGMGk	95%	n+304 (+304), K+304 (+304)	46.64	25.00
244	seq=translation; coord=4:28118892..28124783:1; parent_transcript=GRMZM2G020801_T01; parent_gene=GRMZM2G020801	GRMZM2G020801_P01	TRUE	TRUE	gNPMWNQLTVPEASLYSWDSk	95%	n+304 (+304), K+304 (+304)	59.68	25.00
245	seq=translation; coord=4:28118892..28124783:1; parent_transcript=GRMZM2G020801_T01; parent_gene=GRMZM2G020801	GRMZM2G020801_P01	TRUE	TRUE	iNPLVPVDLVIDHSVQVDVAR	95%	n+304 (+304)	88.50	25.08
246	seq=translation; coord=4:28118892..28124783:1; parent_transcript=GRMZM2G020801_T01; parent_gene=GRMZM2G020801	GRMZM2G020801_P01	TRUE	TRUE	iYSSYLELNLDEVEPSMSGPk	95%	n+304 (+304), K+304 (+304)	40.66	25.00
247	seq=translation; coord=4:28118892..28124783:1; parent_transcript=GRMZM2G020801_T01; parent_gene=GRMZM2G020801	GRMZM2G020801_P01	TRUE	TRUE	iySSYLELNLDEVEPSMSGPk	95%	n+304 (+304), iTRAQ8plex (+304), K+304 (+304)	29.44	25.00
248	seq=translation; coord=4:28118892..28124783:1; parent_transcript=GRMZM2G020801_T01; parent_gene=GRMZM2G020801	GRMZM2G020801_P01	TRUE	TRUE	lSVFDAAMR	89%	n+304 (+304)	26.89	25.00
249	seq=translation; coord=4:28118892..28124783:1; parent_transcript=GRMZM2G020801_T01; parent_gene=GRMZM2G020801	GRMZM2G020801_P01	TRUE	TRUE	mFVDYNEPPTER	95%	n+304 (+304)	44.68	25.00
250	seq=translation; coord=4:28118892..28124783:1; parent_transcript=GRMZM2G020801_T01; parent_gene=GRMZM2G020801	GRMZM2G020801_P01	TRUE	TRUE	sFTcIVR	87%	n+304 (+304), Carbamidomethyl (+57)	25.69	25.00

251	seq=translation; coord=4:28118892..28124783:1; parent_transcript=GRMZM2G020801_T01; parent_gene=GRMZM2G020801	GRMZM2G020801_P01	TRUE	TRUE	sGVTATDLVLTVTQMLR	95%	n+304 (+304)	32.62	26.02
252	seq=translation; coord=4:28118892..28124783:1; parent_transcript=GRMZM2G020801_T01; parent_gene=GRMZM2G020801	GRMZM2G020801_P01	TRUE	TRUE	sGVTATDLVLTVTQmLR	95%	n+304 (+304), Oxidation (+16)	59.22	25.95
253	seq=translation; coord=4:28118892..28124783:1; parent_transcript=GRMZM2G020801_T01; parent_gene=GRMZM2G020801	GRMZM2G020801_P01	TRUE	TRUE	sQNAVQANMELEFSR	95%	n+304 (+304)	83.53	25.00
254	seq=translation; coord=4:28118892..28124783:1; parent_transcript=GRMZM2G020801_T01; parent_gene=GRMZM2G020801	GRMZM2G020801_P01	TRUE	TRUE	tSLAPGSGVVTk	95%	n+304 (+304), K+304 (+304)	37.98	25.45
255	seq=translation; coord=1:53621183..53623074:-1; parent_transcript=GRMZM2G020940_T01; parent_gene=GRMZM2G020940	GRMZM2G020940_P01	TRUE	TRUE	aDEGFSATVR	90%	n+304 (+304)	27.24	25.00
256	seq=translation; coord=1:53621183..53623074:-1; parent_transcript=GRMZM2G020940_T01; parent_gene=GRMZM2G020940	GRMZM2G020940_P01	TRUE	TRUE	dEEGNPAFALVNk	95%	n+304 (+304), K+304 (+304)	46.60	25.44
257	seq=translation; coord=1:53621183..53623074:-1; parent_transcript=GRMZM2G020940_T01; parent_gene=GRMZM2G020940	GRMZM2G020940_P01	TRUE	TRUE	dEEGYPAFALVNR	95%	n+304 (+304)	58.94	25.00
258	seq=translation; coord=1:53621183..53623074:-1; parent_transcript=GRMZM2G020940_T01; parent_gene=GRMZM2G020940	GRMZM2G020940_P01	TRUE	TRUE	dGNVVLAPANPR	95%	n+304 (+304)	61.90	25.21
259	seq=translation; coord=1:53621183..53623074:-1; parent_transcript=GRMZM2G020940_T01; parent_gene=GRMZM2G020940	GRMZM2G020940_P01	TRUE	TRUE	dGTNIVLWk	95%	n+304 (+304), K+304 (+304)	33.76	26.71
260	seq=translation; coord=1:53621183..53623074:-1; parent_transcript=GRMZM2G020940_T01; parent_gene=GRMZM2G020940	GRMZM2G020940_P01	TRUE	TRUE	fGGGGEPTVR	91%	n+304 (+304)	27.70	25.00
261	seq=translation; coord=1:53621183..53623074:-1; parent_transcript=GRMZM2G020940_T01; parent_gene=GRMZM2G020940	GRMZM2G020940_P01	TRUE	TRUE	gDNQSWk	91%	n+304 (+304), K+304 (+304)	28.32	25.00
262	seq=translation; coord=1:53621183..53623074:-1; parent_transcript=GRMZM2G020940_T01; parent_gene=GRMZM2G020940	GRMZM2G020940_P01	TRUE	TRUE	gHGGVHDGTTVVLWEWAk	95%	n+304 (+304), K+304 (+304)	36.42	25.49
263	seq=translation; coord=1:53621183..53623074:-1; parent_transcript=GRMZM2G020940_T01; parent_gene=GRMZM2G020940	GRMZM2G020940_P01	TRUE	TRUE	iLPWGDEAYAAGGSSAANAPR	95%	n+304 (+304)	94.97	25.00
264	seq=translation; coord=1:53621183..53623074:-1; parent_transcript=GRMZM2G020940_T01; parent_gene=GRMZM2G020940	GRMZM2G020940_P01	TRUE	TRUE	iLPWGDEAyAAGGSSAANAPR	95%	n+304 (+304), iTRAQ8plex (+304)	29.08	25.09
265	seq=translation; coord=1:53621183..53623074:-1; parent_transcript=GRMZM2G020940_T01; parent_gene=GRMZM2G020940	GRMZM2G020940_P01	TRUE	TRUE	lVPFNPEYQDESVLWTESGDVGk	95%	n+304 (+304), K+304 (+304)	50.61	25.00
266	seq=translation; coord=1:53621183..53623074:-1; parent_transcript=GRMZM2G020940_T01; parent_gene=GRMZM2G020940	GRMZM2G020940_P01	TRUE	TRUE	lVPYNPGYQDESVLWTESR	95%	n+304 (+304)	41.74	25.00
267	seq=translation; coord=1:53621183..53623074:-1; parent_transcript=GRMZM2G020940_T01; parent_gene=GRMZM2G020940	GRMZM2G020940_P01	TRUE	TRUE	lVPyNPGYQDESVLWTESR	93%	n+304 (+304), iTRAQ8plex (+304)	27.69	25.40
268	seq=translation; coord=9:138424253..138428598:1; parent_transcript=GRMZM2G054300_T01; parent_gene=GRMZM2G054300	GRMZM2G054300_P01,GRMZM2G054300_P04	TRUE	TRUE	cPAELAHGANAGLDIAVR	95%	Carbamidomethyl (+57), n+304 (+304)	62.68	25.00

269	seq=translation; coord=9:138424253..138428598:1; parent_transcript=GRMZM2G054300_T01; parent_gene=GRMZM2G054300	GRMZM2G054300_P01,GRMZM2G054300_P04	TRUE	TRUE	nYPTVSAEYSEAVEk	95%	n+304 (+304), K+304 (+304)	44.91	25.00
270	seq=translation; coord=5:144808460..144816059:-1; parent_transcript=GRMZM2G069542_T01; parent_gene=GRMZM2G069542	GRMZM2G069542_P01	TRUE	TRUE	aLMDEMAVVATk	94%	n+304 (+304), K+304 (+304)	31.29	25.81
271	seq=translation; coord=5:144808460..144816059:-1; parent_transcript=GRMZM2G069542_T01; parent_gene=GRMZM2G069542	GRMZM2G069542_P01	TRUE	TRUE	eEVFDALk	95%	n+304 (+304), K+304 (+304)	32.16	26.25
272	seq=translation; coord=5:144808460..144816059:-1; parent_transcript=GRMZM2G069542_T01; parent_gene=GRMZM2G069542	GRMZM2G069542_P01	TRUE	TRUE	eFVQEcYELSAEYENDRDEAR	95%	n+304 (+304), Carbamidomethyl (+57)	37.05	25.00
273	seq=translation; coord=5:144808460..144816059:-1; parent_transcript=GRMZM2G069542_T01; parent_gene=GRMZM2G069542	GRMZM2G069542_P01	TRUE	TRUE	eLLLQVAGHk	95%	n+304 (+304), K+304 (+304)	39.52	25.00
274	seq=translation; coord=5:144808460..144816059:-1; parent_transcript=GRMZM2G069542_T01; parent_gene=GRMZM2G069542	GRMZM2G069542_P01	TRUE	TRUE	eMYNEWPFFR	92%	n+304 (+304)	28.89	25.00
275	seq=translation; coord=5:144808460..144816059:-1; parent_transcript=GRMZM2G069542_T01; parent_gene=GRMZM2G069542	GRMZM2G069542_P01	TRUE	TRUE	eSYITTLNVcQAYTLk	90%	n+304 (+304), Carbamidomethyl (+57), K+304 (+304)	25.96	25.58
276	seq=translation; coord=5:144808460..144816059:-1; parent_transcript=GRMZM2G069542_T01; parent_gene=GRMZM2G069542	GRMZM2G069542_P01	TRUE	TRUE	fHLPVWLGFGAAIk	95%	n+304 (+304), K+304 (+304)	30.83	25.00
277	seq=translation; coord=5:144808460..144816059:-1; parent_transcript=GRMZM2G069542_T01; parent_gene=GRMZM2G069542	GRMZM2G069542_P01	TRUE	TRUE	fLDILQDLHGPHLR	95%	n+304 (+304)	32.19	25.29
278	seq=translation; coord=5:144808460..144816059:-1; parent_transcript=GRMZM2G069542_T01; parent_gene=GRMZM2G069542	GRMZM2G069542_P01	TRUE	TRUE	gDPGIAAVYDk	95%	n+304 (+304), K+304 (+304)	50.60	25.90
279	seq=translation; coord=5:144808460..144816059:-1; parent_transcript=GRMZM2G069542_T01; parent_gene=GRMZM2G069542	GRMZM2G069542_P01	TRUE	TRUE	iRDPSFQVSPQPPLSk	95%	n+304 (+304), K+304 (+304)	32.50	25.00
280	seq=translation; coord=5:144808460..144816059:-1; parent_transcript=GRMZM2G069542_T01; parent_gene=GRMZM2G069542	GRMZM2G069542_P01	TRUE	TRUE	lADLEAAPAAVAR	95%	n+304 (+304)	38.40	26.47
281	seq=translation; coord=5:144808460..144816059:-1; parent_transcript=GRMZM2G069542_T01; parent_gene=GRMZM2G069542	GRMZM2G069542_P01	TRUE	TRUE	lLVADDLQSFGEQLR	95%	n+304 (+304)	58.88	25.00
282	seq=translation; coord=5:144808460..144816059:-1; parent_transcript=GRMZM2G069542_T01; parent_gene=GRMZM2G069542	GRMZM2G069542_P01	TRUE	TRUE	lVEYDALLVDR	95%	n+304 (+304)	36.60	25.00
283	seq=translation; coord=5:144808460..144816059:-1; parent_transcript=GRMZM2G069542_T01; parent_gene=GRMZM2G069542	GRMZM2G069542_P01	TRUE	TRUE	qVSTFGLALVk	95%	n+304 (+304), K+304 (+304)	48.47	25.00
284	seq=translation; coord=5:144808460..144816059:-1; parent_transcript=GRMZM2G069542_T01; parent_gene=GRMZM2G069542	GRMZM2G069542_P01	TRUE	TRUE	rQDWLLSELR	95%	n+304 (+304)	35.01	26.34
285	seq=translation; coord=5:144808460..144816059:-1; parent_transcript=GRMZM2G069542_T01; parent_gene=GRMZM2G069542	GRMZM2G069542_P01	TRUE	TRUE	rTPPTPQDEMR	95%	n+304 (+304)	29.44	25.00
286	seq=translation; coord=5:144808460..144816059:-1; parent_transcript=GRMZM2G069542_T01; parent_gene=GRMZM2G069542	GRMZM2G069542_P01	TRUE	TRUE	sATPETEYGR	95%	n+304 (+304)	42.81	25.00

287	seq=translation; coord=5:144808460..144816059:-1; parent_transcript=GRMZM2G069542_T01; parent_gene=GRMZM2G069542	GRMZM2G069542_P01	TRUE	TRUE	sIVFQEPR	91%	n+304 (+304)	28.29	25.38
288	seq=translation; coord=5:144808460..144816059:-1; parent_transcript=GRMZM2G069542_T01; parent_gene=GRMZM2G069542	GRMZM2G069542_P01	TRUE	TRUE	sLcAcGDkPIADGSLLDFLR	93%	n+304 (+304), Carbamidomethyl (+57), Carbamidomethyl (+57), K+304 (+304)	25.86	25.67
289	seq=translation; coord=5:144808460..144816059:-1; parent_transcript=GRMZM2G069542_T01; parent_gene=GRMZM2G069542	GRMZM2G069542_P01	TRUE	TRUE	tPPTPQDEMR	95%	n+304 (+304)	33.52	25.00
290	seq=translation; coord=5:144808460..144816059:-1; parent_transcript=GRMZM2G069542_T01; parent_gene=GRMZM2G069542	GRMZM2G069542_P01	TRUE	TRUE	vILGDVR	92%	n+304 (+304)	28.96	25.00
291	seq=translation; coord=5:144808460..144816059:-1; parent_transcript=GRMZM2G069542_T01; parent_gene=GRMZM2G069542	GRMZM2G069542_P01	TRUE	TRUE	vTLDLLEMVFAk	95%	n+304 (+304), K+304 (+304)	37.03	25.00
292	seq=translation; coord=5:144808460..144816059:-1; parent_transcript=GRMZM2G069542_T01; parent_gene=GRMZM2G069542	GRMZM2G069542_P01	TRUE	TRUE	vTVQGEVIEHSFGEELLcFR	95%	n+304 (+304), Carbamidomethyl (+57)	42.29	25.00
293	seq=translation; coord=4:133103720..133107109:1; parent_transcript=GRMZM2G071630_T01; parent_gene=GRMZM2G071630	GRMZM2G071630_P01,GRMZM2G176307_P01, GRMZM2G176307_P02	TRUE	TRUE	aEAEGSLk	87%	n+304 (+304), K+304 (+304)	27.80	26.93
294	seq=translation; coord=4:133103720..133107109:1; parent_transcript=GRMZM2G071630_T01; parent_gene=GRMZM2G071630	GRMZM2G071630_P01,GRMZM2G176307_P01, GRMZM2G176307_P02	TRUE	TRUE	dAPMFVVGVNEk	95%	n+304 (+304), K+304 (+304)	41.29	25.76
295	seq=translation; coord=4:133103720..133107109:1; parent_transcript=GRMZM2G071630_T01; parent_gene=GRMZM2G071630	GRMZM2G071630_P01,GRMZM2G176307_P01, GRMZM2G176307_P02	TRUE	TRUE	eVAVFGcR	95%	n+304 (+304), Carbamidomethyl (+57)	35.11	25.00
296	seq=translation; coord=4:133103720..133107109:1; parent_transcript=GRMZM2G071630_T01; parent_gene=GRMZM2G071630	GRMZM2G071630_P01,GRMZM2G176307_P01, GRMZM2G176307_P02	TRUE	TRUE	fGIVEGLMTTVHAITATQk	95%	n+304 (+304), K+304 (+304)	40.03	25.00
297	seq=translation; coord=4:133103720..133107109:1; parent_transcript=GRMZM2G071630_T01; parent_gene=GRMZM2G071630	GRMZM2G071630_P01,GRMZM2G176307_P01, GRMZM2G176307_P02	TRUE	TRUE	fGIVEGLmTTVHAITATQk	94%	n+304 (+304), Oxidation (+16), K+304 (+304)	26.22	25.00
298	seq=translation; coord=4:133103720..133107109:1; parent_transcript=GRMZM2G071630_T01; parent_gene=GRMZM2G071630	GRMZM2G071630_P01,GRMZM2G176307_P01, GRMZM2G176307_P02	TRUE	TRUE	gILGYVEEDLVSTDFQGDSR	92%	n+304 (+304)	28.55	25.00
299	seq=translation; coord=4:133103720..133107109:1; parent_transcript=GRMZM2G071630_T01; parent_gene=GRMZM2G071630	GRMZM2G071630_P01,GRMZM2G176307_P01, GRMZM2G176307_P02	TRUE	TRUE	gILGyVEEDLVSTDFQGDSR	95%	n+304 (+304), iTRAQ8plex (+304)	43.27	25.00
300	seq=translation; coord=4:133103720..133107109:1; parent_transcript=GRMZM2G071630_T01; parent_gene=GRMZM2G071630	GRMZM2G071630_P01,GRMZM2G176307_P01, GRMZM2G176307_P02	TRUE	TRUE	lVSWYDNEWGYSTR	95%	n+304 (+304)	54.65	25.00
301	seq=translation; coord=4:133103720..133107109:1; parent_transcript=GRMZM2G071630_T01; parent_gene=GRMZM2G071630	GRMZM2G071630_P01,GRMZM2G176307_P01, GRMZM2G176307_P02	TRUE	TRUE	lVSWyDNEWGYSTR	95%	n+304 (+304), iTRAQ8plex (+304)	52.49	25.00
302	seq=translation; coord=4:133103720..133107109:1; parent_transcript=GRMZM2G071630_T01; parent_gene=GRMZM2G071630	GRMZM2G071630_P01,GRMZM2G176307_P01, GRMZM2G176307_P02	TRUE	TRUE	nPEEIPWGSVGAEYVVESTGVFTDQEk	95%	n+304 (+304), K+304 (+304)	47.06	25.00
303	seq=translation; coord=4:133103720..133107109:1; parent_transcript=GRMZM2G071630_T01; parent_gene=GRMZM2G071630	GRMZM2G071630_P01,GRMZM2G176307_P01, GRMZM2G176307_P02	TRUE	TRUE	sATYDEIk	95%	n+304 (+304), K+304 (+304)	40.37	26.77

304	seq=translation; coord=4:133103720..133107109:1; parent_transcript=GRMZM2G071630_T01; parent_gene=GRMZM2G071630	GRMZM2G071630_P01,GRMZM2G176307_P01, GRMZM2G176307_P02	TRUE	TRUE	sDINIVSNAScTTNcLAPLAk	95%	n+304 (+304), Carbamidomethyl (+57), Carbamidomethyl (+57), K+304 (+304)	37.15	25.00
305	seq=translation; coord=4:133103720..133107109:1; parent_transcript=GRMZM2G071630_T01; parent_gene=GRMZM2G071630	GRMZM2G071630_P01,GRMZM2G176307_P01, GRMZM2G176307_P02	TRUE	TRUE	tLLFGEk	89%	n+304 (+304), K+304 (+304)	26.99	25.19
306	seq=translation; coord=4:133103720..133107109:1; parent_transcript=GRMZM2G071630_T01; parent_gene=GRMZM2G071630	GRMZM2G071630_P01,GRMZM2G176307_P01, GRMZM2G176307_P02	TRUE	TRUE	tVDGPSSk	91%	n+304 (+304), K+304 (+304)	28.20	25.16
307	seq=translation; coord=7:146491263..146498155:-1; parent_transcript=GRMZM2G032003_T02; parent_gene=GRMZM2G032003	GRMZM2G032003_P02,GRMZM2G032003_P05	TRUE	TRUE	eYVFVANSDNLGAIVDIk	95%	n+304 (+304), K+304 (+304)	67.57	25.38
308	seq=translation; coord=7:146491263..146498155:-1; parent_transcript=GRMZM2G032003_T02; parent_gene=GRMZM2G032003	GRMZM2G032003_P02,GRMZM2G032003_P05	TRUE	TRUE	eyVFVANSDNLGAIVDIk	94%	n+304 (+304), iTRAQ8plex (+304), K+304 (+304)	25.35	25.00
309	seq=translation; coord=7:146491263..146498155:-1; parent_transcript=GRMZM2G032003_T02; parent_gene=GRMZM2G032003	GRMZM2G032003_P02,GRMZM2G032003_P05	TRUE	TRUE	iFNTNNLWVNLk	95%	n+304 (+304), K+304 (+304)	37.38	25.15
310	seq=translation; coord=7:146491263..146498155:-1; parent_transcript=GRMZM2G032003_T02; parent_gene=GRMZM2G032003	GRMZM2G032003_P02,GRMZM2G032003_P05	TRUE	TRUE	lEIPDGDVLENk	95%	n+304 (+304), K+304 (+304)	32.31	25.68
311	seq=translation; coord=7:146491263..146498155:-1; parent_transcript=GRMZM2G032003_T02; parent_gene=GRMZM2G032003	GRMZM2G032003_P02,GRMZM2G032003_P05	TRUE	TRUE	lEIPDGDVLENkDVNGPEDL	91%	n+304 (+304), K+304 (+304)	25.77	25.00
312	seq=translation; coord=7:146491263..146498155:-1; parent_transcript=GRMZM2G032003_T02; parent_gene=GRMZM2G032003	GRMZM2G032003_P02,GRMZM2G032003_P05	TRUE	TRUE	nGFTFLDLIVIQIESLNkk	95%	n+304 (+304), K+304 (+304), K+304 (+304)	31.13	25.00
313	seq=translation; coord=7:146491263..146498155:-1; parent_transcript=GRMZM2G032003_T02; parent_gene=GRMZM2G032003	GRMZM2G032003_P02,GRMZM2G032003_P05	TRUE	TRUE	sGFISLVSR	95%	n+304 (+304)	33.35	25.00
314	seq=translation; coord=7:146491263..146498155:-1; parent_transcript=GRMZM2G032003_T02; parent_gene=GRMZM2G032003	GRMZM2G032003_P02,GRMZM2G032003_P05	TRUE	TRUE	sVIEVR	87%	n+304 (+304)	26.65	25.76
315	seq=translation; coord=7:146491263..146498155:-1; parent_transcript=GRMZM2G032003_T02; parent_gene=GRMZM2G032003	GRMZM2G032003_P02,GRMZM2G032003_P05	TRUE	TRUE	vQLLEIAQVPDEHVNEFk	95%	n+304 (+304), K+304 (+304)	35.38	25.63
316	seq=translation; coord=7:146491263..146498155:-1; parent_transcript=GRMZM2G032003_T02; parent_gene=GRMZM2G032003	GRMZM2G032003_P02,GRMZM2G032003_P05	TRUE	TRUE	yGcNVPLLLMNSFNTHDDTQk	95%	n+304 (+304), Carbamidomethyl (+57), K+304 (+304)	33.05	25.00
317	seq=translation; coord=7:146491263..146498155:-1; parent_transcript=GRMZM2G032003_T02; parent_gene=GRMZM2G032003	GRMZM2G032003_P02,GRMZM2G032003_P05	TRUE	TRUE	yLSGEAEQIEWSk	95%	n+304 (+304), K+304 (+304)	47.81	25.54
318	seq=translation; coord=7:146491263..146498155:-1; parent_transcript=GRMZM2G032003_T02; parent_gene=GRMZM2G032003	GRMZM2G032003_P02,GRMZM2G032003_P05	TRUE	TRUE	yLSGEAEQIEWSk	95%	n+304 (+304), iTRAQ8plex (+304), K+304 (+304)	45.29	25.89
319	seq=translation; coord=2:162466718..162472431:1; parent_transcript=GRMZM2G102829_T01; parent_gene=GRMZM2G102829	GRMZM2G102829_P01,GRMZM2G102829_P02	TRUE	TRUE	aGGMQQPMPMGGQQQVFPR	95%	n+304 (+304)	49.31	25.00
320	seq=translation; coord=2:162466718..162472431:1; parent_transcript=GRMZM2G102829_T01; parent_gene=GRMZM2G102829	GRMZM2G102829_P01,GRMZM2G102829_P02	TRUE	TRUE	aLYDTFcAFGNILSck	95%	n+304 (+304), Carbamidomethyl (+57), Carbamidomethyl (+57), K+304 (+304)	66.31	25.00

321	seq=translation; coord=2:162466718..162472431:1; parent_transcript=GRMZM2G102829_T01; parent_gene=GRMZM2G102829	GRMZM2G102829_P01,GRMZM2G102829_P02	TRUE	TRUE	aLyDTFcAFGNILSck	95%	n+304 (+304), iTRAQ8plex (+304), Carbamidomethyl (+57), Carbamidomethyl (+57), K+304 (+304)	30.26	25.00
322	seq=translation; coord=2:162466718..162472431:1; parent_transcript=GRMZM2G102829_T01; parent_gene=GRMZM2G102829	GRMZM2G102829_P01,GRMZM2G102829_P02	TRUE	TRUE	eLFAEYGNITSck	92%	n+304 (+304), Carbamidomethyl (+57), K+304 (+304)	26.63	25.00
323	seq=translation; coord=2:162466718..162472431:1; parent_transcript=GRMZM2G102829_T01; parent_gene=GRMZM2G102829	GRMZM2G102829_P01,GRMZM2G102829_P02	TRUE	TRUE	eNVSSNIk	95%	n+304 (+304), K+304 (+304)	32.28	26.47
324	seq=translation; coord=2:162466718..162472431:1; parent_transcript=GRMZM2G102829_T01; parent_gene=GRMZM2G102829	GRMZM2G102829_P01,GRMZM2G102829_P02	TRUE	TRUE	fQNTNLYLk	95%	n+304 (+304), K+304 (+304)	37.14	25.47
325	seq=translation; coord=2:162466718..162472431:1; parent_transcript=GRMZM2G102829_T01; parent_gene=GRMZM2G102829	GRMZM2G102829_P01,GRMZM2G102829_P02	TRUE	TRUE	fSNVYVk	86%	n+304 (+304), K+304 (+304)	25.26	25.00
326	seq=translation; coord=2:162466718..162472431:1; parent_transcript=GRMZM2G102829_T01; parent_gene=GRMZM2G102829	GRMZM2G102829_P01,GRMZM2G102829_P02	TRUE	TRUE	gYGFVQFEk	95%	n+304 (+304), K+304 (+304)	47.49	25.00
327	seq=translation; coord=2:162466718..162472431:1; parent_transcript=GRMZM2G102829_T01; parent_gene=GRMZM2G102829	GRMZM2G102829_P01,GRMZM2G102829_P02	TRUE	TRUE	iATDPSGESR	94%	n+304 (+304)	30.68	25.00
328	seq=translation; coord=2:162466718..162472431:1; parent_transcript=GRMZM2G102829_T01; parent_gene=GRMZM2G102829	GRMZM2G102829_P01,GRMZM2G102829_P02	TRUE	TRUE	lMLGENLYPLVEQLER	95%	n+304 (+304)	40.70	25.40
329	seq=translation; coord=2:162466718..162472431:1; parent_transcript=GRMZM2G102829_T01; parent_gene=GRMZM2G102829	GRMZM2G102829_P01,GRMZM2G102829_P02	TRUE	TRUE	lmLGENLYPLVEQLER	95%	n+304 (+304), Oxidation (+16)	33.42	25.55
330	seq=translation; coord=2:162466718..162472431:1; parent_transcript=GRMZM2G102829_T01; parent_gene=GRMZM2G102829	GRMZM2G102829_P01,GRMZM2G102829_P02	TRUE	TRUE	lMLGENLyPLVEQLER	94%	n+304 (+304), iTRAQ8plex (+304)	27.42	25.07
331	seq=translation; coord=2:162466718..162472431:1; parent_transcript=GRMZM2G102829_T01; parent_gene=GRMZM2G102829	GRMZM2G102829_P01,GRMZM2G102829_P02	TRUE	TRUE	nLEENIDDEk	95%	n+304 (+304), K+304 (+304)	51.00	25.00
332	seq=translation; coord=2:162466718..162472431:1; parent_transcript=GRMZM2G102829_T01; parent_gene=GRMZM2G102829	GRMZM2G102829_P01,GRMZM2G102829_P02	TRUE	TRUE	nLEENIDDEkLR	95%	n+304 (+304), K+304 (+304)	32.79	25.53
333	seq=translation; coord=2:162466718..162472431:1; parent_transcript=GRMZM2G102829_T01; parent_gene=GRMZM2G102829	GRMZM2G102829_P01,GRMZM2G102829_P02	TRUE	TRUE	nLSDTVTDDELk	95%	n+304 (+304), K+304 (+304)	72.93	25.00
334	seq=translation; coord=2:162466718..162472431:1; parent_transcript=GRMZM2G102829_T01; parent_gene=GRMZM2G102829	GRMZM2G102829_P01,GRMZM2G102829_P02	TRUE	TRUE	nVQEVAEk	95%	n+304 (+304), K+304 (+304)	32.83	25.34
335	seq=translation; coord=2:162466718..162472431:1; parent_transcript=GRMZM2G102829_T01; parent_gene=GRMZM2G102829	GRMZM2G102829_P01,GRMZM2G102829_P02	TRUE	TRUE	sAQHLQQSNVSTEQQLANLSLNDGVVSS	95%	n+304 (+304)	42.68	25.00
336	seq=translation; coord=2:162466718..162472431:1; parent_transcript=GRMZM2G102829_T01; parent_gene=GRMZM2G102829	GRMZM2G102829_P01,GRMZM2G102829_P02	TRUE	TRUE	sGTGNIFIk	95%	n+304 (+304), K+304 (+304)	44.07	26.22
337	seq=translation; coord=2:162466718..162472431:1; parent_transcript=GRMZM2G102829_T01; parent_gene=GRMZM2G102829	GRMZM2G102829_P01,GRMZM2G102829_P02	TRUE	TRUE	sLGYAYVNYNNQGDAAR	95%	n+304 (+304)	89.41	25.00

338	seq=translation; coord=2:162466718..162472431:1; parent_transcript=GRMZM2G102829_T01; parent_gene=GRMZM2G102829	GRMZM2G102829_P01,GRMZM2G102829_P02	TRUE	TRUE	sLGyAYVNYNNQGDAAR	90%	n+304 (+304), iTRAQ8plex (+304)	25.03	25.00
339	seq=translation; coord=2:162466718..162472431:1; parent_transcript=GRMZM2G102829_T01; parent_gene=GRMZM2G102829	GRMZM2G102829_P01,GRMZM2G102829_P02	TRUE	TRUE	vAEAMEVLR	94%	n+304 (+304)	30.69	25.22
340	seq=translation; coord=2:162466718..162472431:1; parent_transcript=GRMZM2G102829_T01; parent_gene=GRMZM2G102829	GRMZM2G102829_P01,GRMZM2G102829_P02	TRUE	TRUE	vFVGPFVR	95%	n+304 (+304)	34.90	25.00
341	seq=translation; coord=2:162466718..162472431:1; parent_transcript=GRMZM2G102829_T01; parent_gene=GRMZM2G102829	GRMZM2G102829_P01,GRMZM2G102829_P02	TRUE	TRUE	yGTITSAVVMR	95%	n+304 (+304)	34.37	25.37
342	seq=translation; coord=3:187441065..187444892:-1; parent_transcript=GRMZM2G071790_T01; parent_gene=GRMZM2G071790	GRMZM2G071790_P01,GRMZM2G071790_P02, GRMZM2G071790_P03,GRMZM2G071790_P04, GRMZM2G071790_P05	TRUE	TRUE	yTGNSDLQLER	95%	n+304 (+304)	39.48	25.00
343	seq=translation; coord=1:267887520..267894179:1; parent_transcript=GRMZM2G023289_T03; parent_gene=GRMZM2G023289	GRMZM2G023289_P03	TRUE	TRUE	aTGAFILTASHNPGGPTEDFGIk	93%	n+304 (+304), K+304 (+304)	25.18	25.02
344	seq=translation; coord=1:267887520..267894179:1; parent_transcript=GRMZM2G023289_T03; parent_gene=GRMZM2G023289	GRMZM2G023289_P03	TRUE	TRUE	dSQEALAPLVDVALk	95%	n+304 (+304), K+304 (+304)	42.63	25.00
345	seq=translation; coord=1:267887520..267894179:1; parent_transcript=GRMZM2G023289_T03; parent_gene=GRMZM2G023289	GRMZM2G023289_P03	TRUE	TRUE	gLFTVTk	95%	n+304 (+304), K+304 (+304)	36.92	25.00
346	seq=translation; coord=1:267887520..267894179:1; parent_transcript=GRMZM2G023289_T03; parent_gene=GRMZM2G023289	GRMZM2G023289_P03	TRUE	TRUE	vTVFQQPHYLQNFVQSTFNALPADQVk	95%	n+304 (+304), K+304 (+304)	33.70	25.00
347	seq=translation; coord=1:267887520..267894179:1; parent_transcript=GRMZM2G023289_T03; parent_gene=GRMZM2G023289	GRMZM2G023289_P03	TRUE	TRUE	vYIEQYEk	95%	n+304 (+304), K+304 (+304)	34.25	25.16
348	seq=translation; coord=6:34438543..34440508:1; parent_transcript=GRMZM2G034724_T01; parent_gene=GRMZM2G034724	GRMZM2G034724_P01	TRUE	TRUE	aGVALNcLEAPLDVDIPGGGR	95%	n+304 (+304), Carbamidomethyl (+57)	48.74	25.28
349	seq=translation; coord=6:34438543..34440508:1; parent_transcript=GRMZM2G034724_T01; parent_gene=GRMZM2G034724	GRMZM2G034724_P01	TRUE	TRUE	aISPEVLQASFNTTPEMEk	95%	n+304 (+304), K+304 (+304)	50.01	25.71
350	seq=translation; coord=6:34438543..34440508:1; parent_transcript=GRMZM2G034724_T01; parent_gene=GRMZM2G034724	GRMZM2G034724_P01	TRUE	TRUE	aWDLPEPDAAR	95%	n+304 (+304)	38.07	25.00
351	seq=translation; coord=6:34438543..34440508:1; parent_transcript=GRMZM2G034724_T01; parent_gene=GRMZM2G034724	GRMZM2G034724_P01	TRUE	TRUE	aYGGDGGAYYEWSPADLPMLAVASIGAAk	95%	n+304 (+304), K+304 (+304)	44.05	25.00
352	seq=translation; coord=6:34438543..34440508:1; parent_transcript=GRMZM2G034724_T01; parent_gene=GRMZM2G034724	GRMZM2G034724_P01	TRUE	TRUE	ayGGDGGAYYEWSPADLPMLAVASIGAAk	95%	n+304 (+304), iTRAQ8plex (+304), K+304 (+304)	38.45	25.00
353	seq=translation; coord=6:34438543..34440508:1; parent_transcript=GRMZM2G034724_T01; parent_gene=GRMZM2G034724	GRMZM2G034724_P01	TRUE	TRUE	aYGGDGGAYyEWSPADLPMLAVASIGAAk	95%	n+304 (+304), iTRAQ8plex (+304), K+304 (+304)	26.00	25.00
354	seq=translation; coord=6:34438543..34440508:1; parent_transcript=GRMZM2G034724_T01; parent_gene=GRMZM2G034724	GRMZM2G034724_P01	TRUE	TRUE	iADASGMEWFSIITTPNPVFSHLAGk	95%	n+304 (+304), K+304 (+304)	61.17	25.00
355	seq=translation; coord=6:34438543..34440508:1; parent_transcript=GRMZM2G034724_T01; parent_gene=GRMZM2G034724	GRMZM2G034724_P01	TRUE	TRUE	iADASGmEWFSIITTPNPVFSHLAGk	95%	n+304 (+304), Oxidation (+16), K+304 (+304)	30.06	25.35

356	seq=translation; coord=6:34438543..34440508:1; parent_transcript=GRMZM2G034724_T01; parent_gene=GRMZM2G034724	GRMZM2G034724_P01	TRUE	TRUE	iEGGSLFIVPR	95%	n+304 (+304)	37.00	25.02
357	seq=translation; coord=6:34438543..34440508:1; parent_transcript=GRMZM2G034724_T01; parent_gene=GRMZM2G034724	GRMZM2G034724_P01	TRUE	TRUE	lPASAAALPAPSPQDR	95%	n+304 (+304)	68.69	25.54
358	seq=translation; coord=6:34438543..34440508:1; parent_transcript=GRMZM2G034724_T01; parent_gene=GRMZM2G034724	GRMZM2G034724_P01	TRUE	TRUE	lSLAAGGLSLPSYSDSAk	95%	n+304 (+304), K+304 (+304)	63.14	25.26
359	seq=translation; coord=6:34438543..34440508:1; parent_transcript=GRMZM2G034724_T01; parent_gene=GRMZM2G034724	GRMZM2G034724_P01	TRUE	TRUE	lVSSQPASGIVk	95%	n+304 (+304), K+304 (+304)	49.28	25.13
360	seq=translation; coord=6:34438543..34440508:1; parent_transcript=GRMZM2G034724_T01; parent_gene=GRMZM2G034724	GRMZM2G034724_P01	TRUE	TRUE	rLDSEIFFAPPSSN	95%	n+304 (+304)	33.78	25.00
361	seq=translation; coord=6:34438543..34440508:1; parent_transcript=GRMZM2G034724_T01; parent_gene=GRMZM2G034724	GRMZM2G034724_P01	TRUE	TRUE	vAYVLQGVGTcGLVLPEATk	95%	n+304 (+304), Carbamidomethyl (+57), K+304 (+304)	75.32	25.00
362	seq=translation; coord=6:34438543..34440508:1; parent_transcript=GRMZM2G034724_T01; parent_gene=GRMZM2G034724	GRMZM2G034724_P01	TRUE	TRUE	vQVVGPDGR	94%	n+304 (+304)	30.74	25.00
363	seq=translation; coord=6:34438543..34440508:1; parent_transcript=GRMZM2G034724_T01; parent_gene=GRMZM2G034724	GRMZM2G034724_P01	TRUE	TRUE	vVVLNTANLPLVR	95%	n+304 (+304)	39.99	25.00
364	seq=translation; coord=3:17824258..17828243:1; parent_transcript=GRMZM2G030784_T01; parent_gene=GRMZM2G030784	GRMZM2G030784_P01,GRMZM2G030784_P02	TRUE	TRUE	aLLGESNEFVGDkVAYALSQGLk	95%	n+304 (+304), K+304 (+304), K+304 (+304)	31.60	25.00
365	seq=translation; coord=3:17824258..17828243:1; parent_transcript=GRMZM2G030784_T01; parent_gene=GRMZM2G030784	GRMZM2G030784_P01,GRMZM2G030784_P02	TRUE	TRUE	eLAAQPDVDGFLVGGASLkPEFIDIINAATVk	95%	n+304 (+304), K+304 (+304), K+304 (+304)	27.72	25.00
366	seq=translation; coord=3:17824258..17828243:1; parent_transcript=GRMZM2G030784_T01; parent_gene=GRMZM2G030784	GRMZM2G030784_P01,GRMZM2G030784_P02	TRUE	TRUE	kGGAFTGEVSAEMLVNLGVPWVILGHSER	95%	K+304 (+304), n+304 (+304)	28.95	25.00
367	seq=translation; coord=3:17824258..17828243:1; parent_transcript=GRMZM2G030784_T01; parent_gene=GRMZM2G030784	GRMZM2G030784_P01,GRMZM2G030784_P02	TRUE	TRUE	qEFHVAAQNcWVk	95%	n+304 (+304), Carbamidomethyl (+57), K+304 (+304)	39.34	25.76
368	seq=translation; coord=3:17824258..17828243:1; parent_transcript=GRMZM2G030784_T01; parent_gene=GRMZM2G030784	GRMZM2G030784_P01,GRMZM2G030784_P02	TRUE	TRUE	qEFHVAAQNcWVk	90%	Pyro-cmC (-17), n+304 (+304), Carbamidomethyl (+57), K+304 (+304)	27.24	25.00
369	seq=translation; coord=3:17824258..17828243:1; parent_transcript=GRMZM2G030784_T01; parent_gene=GRMZM2G030784	GRMZM2G030784_P01,GRMZM2G030784_P02	TRUE	TRUE	tLNEGQVPPSDVVEVVVSPPYVFLPVVk	95%	n+304 (+304), K+304 (+304)	30.04	25.00
370	seq=translation; coord=3:17824258..17828243:1; parent_transcript=GRMZM2G030784_T01; parent_gene=GRMZM2G030784	GRMZM2G030784_P01,GRMZM2G030784_P02	TRUE	TRUE	tNASPEVAESTR	95%	n+304 (+304)	50.41	25.00
371	seq=translation; coord=3:17824258..17828243:1; parent_transcript=GRMZM2G030784_T01; parent_gene=GRMZM2G030784	GRMZM2G030784_P01,GRMZM2G030784_P02	TRUE	TRUE	vAYALSQGLk	95%	n+304 (+304), K+304 (+304)	47.66	25.00
372	seq=translation; coord=3:17824258..17828243:1; parent_transcript=GRMZM2G030784_T01; parent_gene=GRMZM2G030784	GRMZM2G030784_P01,GRMZM2G030784_P02	TRUE	TRUE	vIAcVGETLEQR	95%	n+304 (+304), Carbamidomethyl (+57)	64.10	25.00
373	seq=translation; coord=6:145987055..145992446:-1; parent_transcript=GRMZM2G306345_T03; parent_gene=GRMZM2G306345	GRMZM2G306345_P03,GRMZM2G306345_P04, GRMZM2G306345_P05	TRUE	TRUE	aAILVR	95%	n+304 (+304)	30.65	25.00

374	seq=translation; coord=6:145987055..145992446:-1; parent_transcript=GRMZM2G306345_T03; parent_gene=GRMZM2G306345	GRMZM2G306345_P03,GRMZM2G306345_P04, GRMZM2G306345_P05	TRUE	TRUE	aETSPEDVGGMHAAVGILTER	95%	n+304 (+304)	69.85	25.00
375	seq=translation; coord=6:145987055..145992446:-1; parent_transcript=GRMZM2G306345_T03; parent_gene=GRMZM2G306345	GRMZM2G306345_P03,GRMZM2G306345_P04, GRMZM2G306345_P05	TRUE	TRUE	aETSPEDVGGmHAAVGILTER	95%	n+304 (+304), Oxidation (+16)	60.40	25.00
376	seq=translation; coord=6:145987055..145992446:-1; parent_transcript=GRMZM2G306345_T03; parent_gene=GRMZM2G306345	GRMZM2G306345_P03,GRMZM2G306345_P04, GRMZM2G306345_P05	TRUE	TRUE	eLVGQYk	91%	n+304 (+304), K+304 (+304)	28.16	25.92
377	seq=translation; coord=6:145987055..145992446:-1; parent_transcript=GRMZM2G306345_T03; parent_gene=GRMZM2G306345	GRMZM2G306345_P03,GRMZM2G306345_P04, GRMZM2G306345_P05	TRUE	TRUE	eMQDIEFTVQENR	95%	n+304 (+304)	56.01	25.00
378	seq=translation; coord=6:145987055..145992446:-1; parent_transcript=GRMZM2G306345_T03; parent_gene=GRMZM2G306345	GRMZM2G306345_P03,GRMZM2G306345_P04, GRMZM2G306345_P05	TRUE	TRUE	emQDIEFTVQENR	95%	n+304 (+304), Oxidation (+16)	62.98	25.00
379	seq=translation; coord=6:145987055..145992446:-1; parent_transcript=GRMZM2G306345_T03; parent_gene=GRMZM2G306345	GRMZM2G306345_P03,GRMZM2G306345_P04, GRMZM2G306345_P05	TRUE	TRUE	fLDMFGNVVMDIPR	95%	n+304 (+304)	30.55	25.00
380	seq=translation; coord=6:145987055..145992446:-1; parent_transcript=GRMZM2G306345_T03; parent_gene=GRMZM2G306345	GRMZM2G306345_P03,GRMZM2G306345_P04, GRMZM2G306345_P05	TRUE	TRUE	fLDmFGNVVMDIPR	95%	n+304 (+304), Oxidation (+16)	50.21	25.00
381	seq=translation; coord=6:145987055..145992446:-1; parent_transcript=GRMZM2G306345_T03; parent_gene=GRMZM2G306345	GRMZM2G306345_P03,GRMZM2G306345_P04, GRMZM2G306345_P05	TRUE	TRUE	fLDMFGNVVmDIPR	95%	n+304 (+304), Oxidation (+16)	45.79	25.00
382	seq=translation; coord=6:145987055..145992446:-1; parent_transcript=GRMZM2G306345_T03; parent_gene=GRMZM2G306345	GRMZM2G306345_P03,GRMZM2G306345_P04, GRMZM2G306345_P05	TRUE	TRUE	iAVDMVNEGLVEPR	95%	n+304 (+304)	71.96	26.15
383	seq=translation; coord=6:145987055..145992446:-1; parent_transcript=GRMZM2G306345_T03; parent_gene=GRMZM2G306345	GRMZM2G306345_P03,GRMZM2G306345_P04, GRMZM2G306345_P05	TRUE	TRUE	kQLELAVLAVFNSWESPR	95%	K+304 (+304), n+304 (+304)	33.08	25.00
384	seq=translation; coord=6:145987055..145992446:-1; parent_transcript=GRMZM2G306345_T03; parent_gene=GRMZM2G306345	GRMZM2G306345_P03,GRMZM2G306345_P04, GRMZM2G306345_P05	TRUE	TRUE	lVTIGGHVLR	95%	n+304 (+304)	52.11	25.00
385	seq=translation; coord=6:145987055..145992446:-1; parent_transcript=GRMZM2G306345_T03; parent_gene=GRMZM2G306345	GRMZM2G306345_P03,GRMZM2G306345_P04, GRMZM2G306345_P05	TRUE	TRUE	nDTDLTASDLk	95%	n+304 (+304), K+304 (+304)	41.72	25.44
386	seq=translation; coord=6:145987055..145992446:-1; parent_transcript=GRMZM2G306345_T03; parent_gene=GRMZM2G306345	GRMZM2G306345_P03,GRMZM2G306345_P04, GRMZM2G306345_P05	TRUE	TRUE	nLMPQAYDELVENcNILESHYk	95%	n+304 (+304), Carbamidomethyl (+57), K+304 (+304)	77.36	25.00
387	seq=translation; coord=6:145987055..145992446:-1; parent_transcript=GRMZM2G306345_T03; parent_gene=GRMZM2G306345	GRMZM2G306345_P03,GRMZM2G306345_P04, GRMZM2G306345_P05	TRUE	TRUE	qLELAVLAVFNSWESPR	95%	n+304 (+304)	50.76	26.24
388	seq=translation; coord=6:145987055..145992446:-1; parent_transcript=GRMZM2G306345_T03; parent_gene=GRMZM2G306345	GRMZM2G306345_P03,GRMZM2G306345_P04, GRMZM2G306345_P05	TRUE	TRUE	qMIMAPTLELR	95%	n+304 (+304)	33.90	25.54
389	seq=translation; coord=6:145987055..145992446:-1; parent_transcript=GRMZM2G306345_T03; parent_gene=GRMZM2G306345	GRMZM2G306345_P03,GRMZM2G306345_P04, GRMZM2G306345_P05	TRUE	TRUE	rFLDMFGNVVMDIPR	95%	n+304 (+304)	57.75	25.24
390	seq=translation; coord=6:145987055..145992446:-1; parent_transcript=GRMZM2G306345_T03; parent_gene=GRMZM2G306345	GRMZM2G306345_P03,GRMZM2G306345_P04, GRMZM2G306345_P05	TRUE	TRUE	sGAAVSMPGMMDTVLNLGLNDEVAAGLAAk	95%	n+304 (+304), K+304 (+304)	56.23	25.00
391	seq=translation; coord=6:145987055..145992446:-1; parent_transcript=GRMZM2G306345_T03; parent_gene=GRMZM2G306345	GRMZM2G306345_P03,GRMZM2G306345_P04, GRMZM2G306345_P05	TRUE	TRUE	sLFEEk	88%	n+304 (+304), K+304 (+304)	26.76	25.44

392	seq=translation; coord=6:145987055..145992446:-1; parent_transcript=GRMZM2G306345_T03; parent_gene=GRMZM2G306345	GRMZM2G306345_P03,GRMZM2G306345_P04, GRMZM2G306345_P05	TRUE	TRUE	vLANADTPDDALTAR	95%	n+304 (+304)	84.22	25.00
393	seq=translation; coord=5:84822215..84825942:1; parent_transcript=GRMZM2G382914_T01; parent_gene=GRMZM2G382914	GRMZM2G382914_P01,GRMZM2G382914_P02, GRMZM2G382914_P03,GRMZM2G382914_P04	TRUE	TRUE	aDLNVPLDDAQk	95%	n+304 (+304), K+304 (+304)	32.64	25.26
394	seq=translation; coord=5:84822215..84825942:1; parent_transcript=GRMZM2G382914_T01; parent_gene=GRMZM2G382914	GRMZM2G382914_P01,GRMZM2G382914_P02, GRMZM2G382914_P03,GRMZM2G382914_P04	TRUE	TRUE	eLDYLVGAVANPk	95%	n+304 (+304), K+304 (+304)	30.50	26.64
395	seq=translation; coord=5:84822215..84825942:1; parent_transcript=GRMZM2G382914_T01; parent_gene=GRMZM2G382914	GRMZM2G382914_P01,GRMZM2G382914_P02, GRMZM2G382914_P03,GRMZM2G382914_P04	TRUE	TRUE	eLDyLVGAVANPk	95%	n+304 (+304), iTRAQ8plex (+304), K+304 (+304)	30.61	25.00
396	seq=translation; coord=5:84822215..84825942:1; parent_transcript=GRMZM2G382914_T01; parent_gene=GRMZM2G382914	GRMZM2G382914_P01,GRMZM2G382914_P02, GRMZM2G382914_P03,GRMZM2G382914_P04	TRUE	TRUE	fAADAESk	91%	n+304 (+304), K+304 (+304)	28.16	25.15
397	seq=translation; coord=5:84822215..84825942:1; parent_transcript=GRMZM2G382914_T01; parent_gene=GRMZM2G382914	GRMZM2G382914_P01,GRMZM2G382914_P02, GRMZM2G382914_P03,GRMZM2G382914_P04	TRUE	TRUE	fAAGTEAIAk	95%	n+304 (+304), K+304 (+304)	38.84	25.61
398	seq=translation; coord=5:84822215..84825942:1; parent_transcript=GRMZM2G382914_T01; parent_gene=GRMZM2G382914	GRMZM2G382914_P01,GRMZM2G382914_P02, GRMZM2G382914_P03,GRMZM2G382914_P04	TRUE	TRUE	gVSLLLPTDIVVADk	95%	n+304 (+304), K+304 (+304)	48.42	25.00
399	seq=translation; coord=5:84822215..84825942:1; parent_transcript=GRMZM2G382914_T01; parent_gene=GRMZM2G382914	GRMZM2G382914_P01,GRMZM2G382914_P02, GRMZM2G382914_P03,GRMZM2G382914_P04	TRUE	TRUE	iGVIESLLAk	95%	n+304 (+304), K+304 (+304)	47.54	25.00
400	seq=translation; coord=5:84822215..84825942:1; parent_transcript=GRMZM2G382914_T01; parent_gene=GRMZM2G382914	GRMZM2G382914_P01,GRMZM2G382914_P02, GRMZM2G382914_P03,GRMZM2G382914_P04	TRUE	TRUE	iVPATAIPDDWMGLDVGPDATk	90%	n+304 (+304), K+304 (+304)	25.78	25.44
401	seq=translation; coord=5:84822215..84825942:1; parent_transcript=GRMZM2G382914_T01; parent_gene=GRMZM2G382914	GRMZM2G382914_P01,GRMZM2G382914_P02, GRMZM2G382914_P03,GRMZM2G382914_P04	TRUE	TRUE	lAAALPEGGVLLLENVR	95%	n+304 (+304)	74.29	25.00
402	seq=translation; coord=5:84822215..84825942:1; parent_transcript=GRMZM2G382914_T01; parent_gene=GRMZM2G382914	GRMZM2G382914_P01,GRMZM2G382914_P02, GRMZM2G382914_P03,GRMZM2G382914_P04	TRUE	TRUE	lAELTTTk	95%	n+304 (+304), K+304 (+304)	31.46	26.41
403	seq=translation; coord=5:84822215..84825942:1; parent_transcript=GRMZM2G382914_T01; parent_gene=GRMZM2G382914	GRMZM2G382914_P01,GRMZM2G382914_P02, GRMZM2G382914_P03,GRMZM2G382914_P04	TRUE	TRUE	lELATSLIEk	92%	n+304 (+304), K+304 (+304)	26.06	25.00
404	seq=translation; coord=5:84822215..84825942:1; parent_transcript=GRMZM2G382914_T01; parent_gene=GRMZM2G382914	GRMZM2G382914_P01,GRMZM2G382914_P02, GRMZM2G382914_P03,GRMZM2G382914_P04	TRUE	TRUE	sVGTLGEADLk	92%	n+304 (+304), K+304 (+304)	28.48	25.84
405	seq=translation; coord=5:84822215..84825942:1; parent_transcript=GRMZM2G382914_T01; parent_gene=GRMZM2G382914	GRMZM2G382914_P01,GRMZM2G382914_P02, GRMZM2G382914_P03,GRMZM2G382914_P04	TRUE	TRUE	tFDEALDTTk	95%	n+304 (+304), K+304 (+304)	56.77	26.36
406	seq=translation; coord=5:84822215..84825942:1; parent_transcript=GRMZM2G382914_T01; parent_gene=GRMZM2G382914	GRMZM2G382914_P01,GRMZM2G382914_P02, GRMZM2G382914_P03,GRMZM2G382914_P04	TRUE	TRUE	vDILILGGGMIYTFYk	95%	n+304 (+304), K+304 (+304)	48.28	25.00
407	seq=translation; coord=5:84822215..84825942:1; parent_transcript=GRMZM2G382914_T01; parent_gene=GRMZM2G382914	GRMZM2G382914_P01,GRMZM2G382914_P02, GRMZM2G382914_P03,GRMZM2G382914_P04	TRUE	TRUE	vDILILGGGMIYTFyk	95%	n+304 (+304), iTRAQ8plex (+304), K+304 (+304)	32.75	25.00
408	seq=translation; coord=10:127370249..127371829:-1; parent_transcript=GRMZM2G005633_T02; parent_gene=GRMZM2G005633	GRMZM2G005633_P02	TRUE	TRUE	aALWFWMNSVHGVVPQGFGATTR	95%	n+304 (+304)	44.52	25.00
409	seq=translation; coord=10:127370249..127371829:-1; parent_transcript=GRMZM2G005633_T02; parent_gene=GRMZM2G005633	GRMZM2G005633_P02	TRUE	TRUE	aIGFDGLGDPGR	92%	n+304 (+304)	29.16	25.00

410	seq=translation; coord=10:127370249..127371829:-1; parent_transcript=GRMZM2G005633_T02; parent_gene=GRMZM2G005633	GRMZM2G005633_P02	TRUE	TRUE	aYPGFAHGGSQVQGk	95%	n+304 (+304), K+304 (+304)	36.48	25.26
411	seq=translation; coord=10:127370249..127371829:-1; parent_transcript=GRMZM2G005633_T02; parent_gene=GRMZM2G005633	GRMZM2G005633_P02	TRUE	TRUE	dAVVAFk	89%	n+304 (+304), K+304 (+304)	27.99	26.40
412	seq=translation; coord=10:127370249..127371829:-1; parent_transcript=GRMZM2G005633_T02; parent_gene=GRMZM2G005633	GRMZM2G005633_P02	TRUE	TRUE	fGYcGTTDEYcGDGcQSGPcR	95%	n+304 (+304), Carbamidomethyl (+57), Carbamidomethyl (+57), Carbamidomethyl (+57), Carbamidomethyl (+57)	32.75	25.00
413	seq=translation; coord=10:127370249..127371829:-1; parent_transcript=GRMZM2G005633_T02; parent_gene=GRMZM2G005633	GRMZM2G005633_P02	TRUE	TRUE	fGycGTTDEYcGDGcQSGPcR	95%	n+304 (+304), iTRAQ8plex (+304), Carbamidomethyl (+57), Carbamidomethyl (+57), Carbamidomethyl (+57), Carbamidomethyl (+57)	29.86	25.00
414	seq=translation; coord=10:127370249..127371829:-1; parent_transcript=GRMZM2G005633_T02; parent_gene=GRMZM2G005633	GRMZM2G005633_P02	TRUE	TRUE	fGYcGTTDEycGDGcQSGPcR	95%	n+304 (+304), Carbamidomethyl (+57), iTRAQ8plex (+304), Carbamidomethyl (+57), Carbamidomethyl (+57), Carbamidomethyl (+57)	35.81	25.00
415	seq=translation; coord=10:127370249..127371829:-1; parent_transcript=GRMZM2G005633_T02; parent_gene=GRMZM2G005633	GRMZM2G005633_P02	TRUE	TRUE	qLGVDPGPNLTc	95%	n+304 (+304), Carbamidomethyl (+57)	36.58	25.00
416	seq=translation; coord=10:127370249..127371829:-1; parent_transcript=GRMZM2G005633_T02; parent_gene=GRMZM2G005633	GRMZM2G005633_P02	TRUE	TRUE	sAFLSAVk	94%	n+304 (+304), K+304 (+304)	28.65	25.00
417	seq=translation; coord=10:127370249..127371829:-1; parent_transcript=GRMZM2G005633_T02; parent_gene=GRMZM2G005633	GRMZM2G005633_P02	TRUE	TRUE	sNAYcDPTk	91%	n+304 (+304), Carbamidomethyl (+57), K+304 (+304)	28.36	25.00
418	seq=translation; coord=10:21722658..21727770:1; parent_transcript=GRMZM2G001500_T02; parent_gene=GRMZM2G001500	GRMZM2G001500_P02	TRUE	TRUE	aVVTVPAYFNDSQR	95%	n+304 (+304)	53.26	25.00
419	seq=translation; coord=10:21722658..21727770:1; parent_transcript=GRMZM2G001500_T02; parent_gene=GRMZM2G001500	GRMZM2G001500_P02	TRUE	TRUE	dAISGGSTQSmk	95%	n+304 (+304), Oxidation (+16), K+304 (+304)	39.25	25.00
420	seq=translation; coord=10:21722658..21727770:1; parent_transcript=GRMZM2G001500_T02; parent_gene=GRMZM2G001500	GRMZM2G001500_P02	TRUE	TRUE	dVVLLDVTPLSLGLETLGGVMTk	95%	n+304 (+304), K+304 (+304)	60.98	25.00
421	seq=translation; coord=10:21722658..21727770:1; parent_transcript=GRMZM2G001500_T02; parent_gene=GRMZM2G001500	GRMZM2G001500_P02	TRUE	TRUE	dVVLLDVTPLSLGLETLGGVmTk	95%	n+304 (+304), Oxidation (+16), K+304 (+304)	65.27	25.00
422	seq=translation; coord=10:21722658..21727770:1; parent_transcript=GRMZM2G001500_T02; parent_gene=GRMZM2G001500	GRMZM2G001500_P02	TRUE	TRUE	hIEATLSR	94%	n+304 (+304)	28.41	26.07
423	seq=translation; coord=10:21722658..21727770:1; parent_transcript=GRMZM2G001500_T02; parent_gene=GRMZM2G001500	GRMZM2G001500_P02	TRUE	TRUE	iPAVQELVR	87%	n+304 (+304)	25.59	25.00
424	seq=translation; coord=10:21722658..21727770:1; parent_transcript=GRMZM2G001500_T02; parent_gene=GRMZM2G001500	GRMZM2G001500_P02	TRUE	TRUE	iVDWLASNFk	95%	n+304 (+304), K+304 (+304)	47.90	26.53

425	seq=translation; coord=10:21722658..21727770:1; parent_transcript=GRMZM2G001500_T02; parent_gene=GRMZM2G001500	GRMZM2G001500_P02	TRUE	TRUE	lSLSDLDEVILVGGSTR	95%	n+304 (+304)	51.28	25.39
426	seq=translation; coord=10:21722658..21727770:1; parent_transcript=GRMZM2G001500_T02; parent_gene=GRMZM2G001500	GRMZM2G001500_P02	TRUE	TRUE	mAEVDDEAk	93%	Oxidation (+16), n+304 (+304), K+304 (+304)	28.33	25.00
427	seq=translation; coord=10:21722658..21727770:1; parent_transcript=GRMZM2G001500_T02; parent_gene=GRMZM2G001500	GRMZM2G001500_P02	TRUE	TRUE	mELSTLTQANISLPFITATADGPk	95%	n+304 (+304), K+304 (+304)	36.73	25.01
428	seq=translation; coord=10:21722658..21727770:1; parent_transcript=GRMZM2G001500_T02; parent_gene=GRMZM2G001500	GRMZM2G001500_P02	TRUE	TRUE	mVEEADk	94%	Oxidation (+16), n+304 (+304), K+304 (+304)	27.70	25.00
429	seq=translation; coord=10:21722658..21727770:1; parent_transcript=GRMZM2G001500_T02; parent_gene=GRMZM2G001500	GRMZM2G001500_P02	TRUE	TRUE	nQADSVVYQTEk	95%	n+304 (+304), K+304 (+304)	64.79	25.00
430	seq=translation; coord=10:21722658..21727770:1; parent_transcript=GRMZM2G001500_T02; parent_gene=GRMZM2G001500	GRMZM2G001500_P02	TRUE	TRUE	vVGIDLGTTNSAVAAMEGGkPTVVTNAEGAR	95%	n+304 (+304), K+304 (+304)	28.88	25.00
431	seq=translation; coord=2:209289645..209292956:-1; parent_transcript=GRMZM2G113696_T01; parent_gene=GRMZM2G113696	GRMZM2G113696_P01,GRMZM2G144030_P01	TRUE	TRUE	dDLRLPTDETLVAQIk	93%	n+304 (+304), K+304 (+304)	26.34	25.00
432	seq=translation; coord=2:209289645..209292956:-1; parent_transcript=GRMZM2G113696_T01; parent_gene=GRMZM2G113696	GRMZM2G113696_P01,GRMZM2G144030_P01	TRUE	TRUE	dLVVTVQSAMGEEQIcALk	95%	n+304 (+304), Carbamidomethyl (+57), K+304 (+304)	40.68	25.01
433	seq=translation; coord=2:209289645..209292956:-1; parent_transcript=GRMZM2G113696_T01; parent_gene=GRMZM2G113696	GRMZM2G113696_P01,GRMZM2G144030_P01	TRUE	TRUE	dLVVTVQSAmGEEQIcALk	95%	n+304 (+304), Oxidation (+16), Carbamidomethyl (+57), K+304 (+304)	48.72	25.49
434	seq=translation; coord=2:209289645..209292956:-1; parent_transcript=GRMZM2G113696_T01; parent_gene=GRMZM2G113696	GRMZM2G113696_P01,GRMZM2G144030_P01	TRUE	TRUE	eGFESGk	86%	n+304 (+304), K+304 (+304)	26.19	25.89
435	seq=translation; coord=2:209289645..209292956:-1; parent_transcript=GRMZM2G113696_T01; parent_gene=GRMZM2G113696	GRMZM2G113696_P01,GRMZM2G144030_P01	TRUE	TRUE	lPTDETLVAQIk	95%	n+304 (+304), K+304 (+304)	32.90	25.37
436	seq=translation; coord=2:209289645..209292956:-1; parent_transcript=GRMZM2G113696_T01; parent_gene=GRMZM2G113696	GRMZM2G113696_P01,GRMZM2G144030_P01	TRUE	TRUE	tEYQLIDISEDGFVSLLTSDGNTk	95%	n+304 (+304), K+304 (+304)	55.83	25.00
437	seq=translation; coord=2:209289645..209292956:-1; parent_transcript=GRMZM2G113696_T01; parent_gene=GRMZM2G113696	GRMZM2G113696_P01,GRMZM2G144030_P01	TRUE	TRUE	tEyQLIDISEDGFVSLLTSDGNTk	95%	n+304 (+304), iTRAQ8plex (+304), K+304 (+304)	35.50	25.00
438	seq=translation; coord=2:209289645..209292956:-1; parent_transcript=GRMZM2G113696_T01; parent_gene=GRMZM2G113696	GRMZM2G113696_P01,GRMZM2G144030_P01	TRUE	TRUE	tEYQLIDISEDGFVSLLTSDGNTkDDLR	95%	n+304 (+304), K+304 (+304)	38.48	25.00
439	seq=translation; coord=2:209289645..209292956:-1; parent_transcript=GRMZM2G113696_T01; parent_gene=GRMZM2G113696	GRMZM2G113696_P01,GRMZM2G144030_P01	TRUE	TRUE	tYPQQAGTVR	89%	n+304 (+304)	26.78	25.00
440	seq=translation; coord=2:209289645..209292956:-1; parent_transcript=GRMZM2G113696_T01; parent_gene=GRMZM2G113696	GRMZM2G113696_P01,GRMZM2G144030_P01	TRUE	TRUE	tyPQQAGTVR	95%	n+304 (+304), iTRAQ8plex (+304)	30.56	26.21
441	seq=translation; coord=1:47172055..47173158:1; parent_transcript=GRMZM2G112524_T02; parent_gene=GRMZM2G112524	GRMZM2G112524_P02,GRMZM2G112538_P01	TRUE	TRUE	eSVTAIFk	95%	n+304 (+304), K+304 (+304)	34.17	26.23
442	seq=translation; coord=1:47172055..47173158:1; parent_transcript=GRMZM2G112524_T02; parent_gene=GRMZM2G112524	GRMZM2G112524_P02,GRMZM2G112538_P01	TRUE	TRUE	gAEAYLVANPDAYN	95%	n+304 (+304)	68.92	25.00

443	seq=translation; coord=1:47172055..47173158:1; parent_transcript=GRMZM2G112524_T02; parent_gene=GRMZM2G112524	GRMZM2G112524_P02,GRMZM2G112538_P01	TRUE	TRUE	gAEAyLVANPDAYN	94%	n+304 (+304), iTRAQ8plex (+304)	31.33	25.00
444	seq=translation; coord=1:47172055..47173158:1; parent_transcript=GRMZM2G112524_T02; parent_gene=GRMZM2G112524	GRMZM2G112524_P02,GRMZM2G112538_P01	TRUE	TRUE	gAEAYLVANPDAyN	95%	n+304 (+304), iTRAQ8plex (+304)	42.98	25.00
445	seq=translation; coord=1:47172055..47173158:1; parent_transcript=GRMZM2G112524_T02; parent_gene=GRMZM2G112524	GRMZM2G112524_P02,GRMZM2G112538_P01	TRUE	TRUE	lEFLDADk	90%	n+304 (+304), K+304 (+304)	26.38	26.13
446	seq=translation; coord=1:47172055..47173158:1; parent_transcript=GRMZM2G112524_T02; parent_gene=GRMZM2G112524	GRMZM2G112524_P02,GRMZM2G112538_P01	TRUE	TRUE	lLPGVEVk	95%	n+304 (+304), K+304 (+304)	37.96	25.00
447	seq=translation; coord=1:47172055..47173158:1; parent_transcript=GRMZM2G112524_T02; parent_gene=GRMZM2G112524	GRMZM2G112524_P02,GRMZM2G112538_P01	TRUE	TRUE	nTLIEGGGIGVAIETATSHIk	95%	n+304 (+304), K+304 (+304)	96.76	25.00
448	seq=translation; coord=1:47172055..47173158:1; parent_transcript=GRMZM2G112524_T02; parent_gene=GRMZM2G112524	GRMZM2G112524_P02,GRMZM2G112538_P01	TRUE	TRUE	qFNFTSVMPFSFMk	95%	n+304 (+304), K+304 (+304)	30.06	25.25
449	seq=translation; coord=1:47172055..47173158:1; parent_transcript=GRMZM2G112524_T02; parent_gene=GRMZM2G112524	GRMZM2G112524_P02,GRMZM2G112538_P01	TRUE	TRUE	qFNFTSVmPFSFMk	93%	n+304 (+304), Oxidation (+16), K+304 (+304)	27.31	25.00
450	seq=translation; coord=1:47172055..47173158:1; parent_transcript=GRMZM2G112524_T02; parent_gene=GRMZM2G112524	GRMZM2G112524_P02,GRMZM2G112538_P01	TRUE	TRUE	qFNFTSVMPFSFmk	92%	n+304 (+304), Oxidation (+16), K+304 (+304)	26.69	25.00
451	seq=translation; coord=1:47172055..47173158:1; parent_transcript=GRMZM2G112524_T02; parent_gene=GRMZM2G112524	GRMZM2G112524_P02,GRMZM2G112538_P01	TRUE	TRUE	qFNFTSVmPFSFmk	94%	n+304 (+304), Oxidation (+16), Oxidation (+16), K+304 (+304)	28.55	25.00
452	seq=translation; coord=1:47172055..47173158:1; parent_transcript=GRMZM2G112524_T02; parent_gene=GRMZM2G112524	GRMZM2G112524_P02,GRMZM2G112538_P01	TRUE	TRUE	vASHVVASAQPVEGDGGVGSVR	95%	n+304 (+304)	87.41	25.00
453	seq=translation; coord=1:47172055..47173158:1; parent_transcript=GRMZM2G112524_T02; parent_gene=GRMZM2G112524	GRMZM2G112524_P02,GRMZM2G112538_P01	TRUE	TRUE	vEPAAGGGSVVk	91%	n+304 (+304), K+304 (+304)	28.28	25.33
454	seq=translation; coord=5:9471280..9475018:-1; parent_transcript=GRMZM2G152466_T01; parent_gene=GRMZM2G152466	GRMZM2G152466_P01,GRMZM2G152466_P03, GRMZM2G153292_P01,GRMZM2G153292_P02, GRMZM2G153292_P03,GRMZM2G153292_P04, GRMZM2G153292_P08	TRUE	TRUE	aVFVDLEPTVIDEVR	95%	n+304 (+304)	64.68	25.99
455	seq=translation; coord=5:9471280..9475018:-1; parent_transcript=GRMZM2G152466_T01; parent_gene=GRMZM2G152466	GRMZM2G152466_P01,GRMZM2G152466_P03, GRMZM2G153292_P01,GRMZM2G153292_P02, GRMZM2G153292_P03,GRMZM2G153292_P04, GRMZM2G153292_P08	TRUE	TRUE	aYHEQLSVAEITNSAFEPSSMMAk	95%	n+304 (+304), K+304 (+304)	36.64	25.00
456	seq=translation; coord=5:9471280..9475018:-1; parent_transcript=GRMZM2G152466_T01; parent_gene=GRMZM2G152466	GRMZM2G152466_P01,GRMZM2G152466_P03, GRMZM2G153292_P01,GRMZM2G153292_P02, GRMZM2G153292_P03,GRMZM2G153292_P04, GRMZM2G153292_P08	TRUE	TRUE	tIGGGDDAFNTFFSETGAGk	95%	n+304 (+304), K+304 (+304)	44.29	25.00
457	seq=translation; coord=1:278130299..278132841:1; parent_transcript=GRMZM2G328500_T01; parent_gene=GRMZM2G328500	GRMZM2G328500_P01,GRMZM2G328500_P02, GRMZM2G328500_P03	TRUE	TRUE	gAHGLcILTEWDEFk	95%	n+304 (+304), Carbamidomethyl (+57), K+304 (+304)	29.15	25.05
458	seq=translation; coord=1:278130299..278132841:1; parent_transcript=GRMZM2G328500_T01; parent_gene=GRMZM2G328500	GRMZM2G328500_P01,GRMZM2G328500_P02, GRMZM2G328500_P03	TRUE	TRUE	iSSVNAISALcEATGANVTEVAYAVGk	95%	n+304 (+304), Carbamidomethyl (+57), K+304 (+304)	31.45	25.31
459	seq=translation; coord=6:91630823..91634799:1; parent_transcript=GRMZM2G428518_T01; parent_gene=GRMZM2G428518	GRMZM2G428518_P01	TRUE	TRUE	dEATYGVDR	93%	n+304 (+304)	28.09	25.00

460	seq=translation; coord=6:91630823..91634799:1; parent_transcript=GRMZM2G428518_T01; parent_gene=GRMZM2G428518	GRMZM2G428518_P01	TRUE	TRUE	gVLVSPVLEPGATTVEAYFPAGR	95%	n+304 (+304)	52.48	25.33
461	seq=translation; coord=6:91630823..91634799:1; parent_transcript=GRMZM2G428518_T01; parent_gene=GRMZM2G428518	GRMZM2G428518_P01	TRUE	TRUE	gVLVSPVLEPGATTVEAyFPAGR	95%	n+304 (+304), iTRAQ8plex (+304)	53.77	25.00
462	seq=translation; coord=6:91630823..91634799:1; parent_transcript=GRMZM2G428518_T01; parent_gene=GRMZM2G428518	GRMZM2G428518_P01	TRUE	TRUE	wIQLGAFYPFAR	94%	n+304 (+304)	31.70	26.13
463	seq=translation; coord=6:91630823..91634799:1; parent_transcript=GRMZM2G428518_T01; parent_gene=GRMZM2G428518	GRMZM2G428518_P01	TRUE	TRUE	wIQLGAFyPFAR	93%	n+304 (+304), iTRAQ8plex (+304)	26.81	25.30
464	seq=translation; coord=1:125074232..125080812:-1; parent_transcript=GRMZM2G048371_T01; parent_gene=GRMZM2G048371	GRMZM2G048371_P01,GRMZM2G048371_P03	TRUE	TRUE	aAVPSGASTGVYEALELR	95%	n+304 (+304)	61.54	26.04
465	seq=translation; coord=1:125074232..125080812:-1; parent_transcript=GRMZM2G048371_T01; parent_gene=GRMZM2G048371	GRMZM2G048371_P01,GRMZM2G048371_P03	TRUE	TRUE	dPTAQTEIDNFMVQQLDGTk	95%	n+304 (+304), K+304 (+304)	32.54	25.00
466	seq=translation; coord=1:125074232..125080812:-1; parent_transcript=GRMZM2G048371_T01; parent_gene=GRMZM2G048371	GRMZM2G048371_P01,GRMZM2G048371_P03	TRUE	TRUE	gNPTVEVDVFcSDGTFAR	92%	n+304 (+304), Carbamidomethyl (+57)	26.35	25.00
467	seq=translation; coord=1:125074232..125080812:-1; parent_transcript=GRMZM2G048371_T01; parent_gene=GRMZM2G048371	GRMZM2G048371_P01,GRMZM2G048371_P03	TRUE	TRUE	iEEELGAIAVYAGAk	95%	n+304 (+304), K+304 (+304)	37.70	25.17
468	seq=translation; coord=1:125074232..125080812:-1; parent_transcript=GRMZM2G048371_T01; parent_gene=GRMZM2G048371	GRMZM2G048371_P01,GRMZM2G048371_P03	TRUE	TRUE	lAMQEFMILPTGAASFk	95%	n+304 (+304), K+304 (+304)	50.33	25.45
469	seq=translation; coord=1:125074232..125080812:-1; parent_transcript=GRMZM2G048371_T01; parent_gene=GRMZM2G048371	GRMZM2G048371_P01,GRMZM2G048371_P03	TRUE	TRUE	lAMQEFmILPTGAASFk	95%	n+304 (+304), Oxidation (+16), K+304 (+304)	30.84	25.44
470	seq=translation; coord=1:125074232..125080812:-1; parent_transcript=GRMZM2G048371_T01; parent_gene=GRMZM2G048371	GRMZM2G048371_P01,GRMZM2G048371_P03	TRUE	TRUE	mGVEVYHHLk	95%	n+304 (+304), K+304 (+304)	28.82	25.19
471	seq=translation; coord=1:125074232..125080812:-1; parent_transcript=GRMZM2G048371_T01; parent_gene=GRMZM2G048371	GRMZM2G048371_P01,GRMZM2G048371_P03	TRUE	TRUE	mTEEIGEQVQIVGDDLLVTNPTR	95%	n+304 (+304)	31.43	25.11
472	seq=translation; coord=1:125074232..125080812:-1; parent_transcript=GRMZM2G048371_T01; parent_gene=GRMZM2G048371	GRMZM2G048371_P01,GRMZM2G048371_P03	TRUE	TRUE	scNALLLk	95%	n+304 (+304), Carbamidomethyl (+57), K+304 (+304)	40.91	25.15
473	seq=translation; coord=1:125074232..125080812:-1; parent_transcript=GRMZM2G048371_T01; parent_gene=GRMZM2G048371	GRMZM2G048371_P01,GRMZM2G048371_P03	TRUE	TRUE	sFVSEYPIVSIEDPFDQDDWVHYAk	95%	n+304 (+304), K+304 (+304)	41.14	25.00
474	seq=translation; coord=1:125074232..125080812:-1; parent_transcript=GRMZM2G048371_T01; parent_gene=GRMZM2G048371	GRMZM2G048371_P01,GRMZM2G048371_P03	TRUE	TRUE	sGETEDTFIADLAVGLSTGQIk	95%	n+304 (+304), K+304 (+304)	54.23	25.69
475	seq=translation; coord=1:125074232..125080812:-1; parent_transcript=GRMZM2G048371_T01; parent_gene=GRMZM2G048371	GRMZM2G048371_P01,GRMZM2G048371_P03	TRUE	TRUE	vNQIGSVTESIEAVk	95%	n+304 (+304), K+304 (+304)	44.52	25.76
476	seq=translation; coord=5:18767810..18770458:1; parent_transcript=GRMZM2G087326_T01; parent_gene=GRMZM2G087326	GRMZM2G087326_P01	TRUE	TRUE	lGDAMVTWIEAWDELNPAGPAAGk	95%	n+304 (+304), K+304 (+304)	51.17	25.42

477	seq=translation; coord=8:134521573..134531903:1; parent_transcript=GRMZM2G067985_T04; parent_gene=GRMZM2G067985	GRMZM2G067985_P04,GRMZM2G067985_P05, GRMZM2G067985_P06,GRMZM2G067985_P09, GRMZM2G067985_P10,GRMZM2G067985_P12, GRMZM2G067985_P15,GRMZM2G104017_P02, GRMZM2G104017_P03,GRMZM2G104017_P04, GRMZM2G110378_P01,GRMZM2G110378_P02, GRMZM2G110378_P04,GRMZM2G110378_P05	TRUE	TRUE	eEYDESGPAIVHR	95%	n+304 (+304)	72.35	25.00
478	seq=translation; coord=8:134521573..134531903:1; parent_transcript=GRMZM2G067985_T04; parent_gene=GRMZM2G067985	GRMZM2G067985_P04,GRMZM2G067985_P05, GRMZM2G067985_P06,GRMZM2G067985_P09, GRMZM2G067985_P10,GRMZM2G067985_P12, GRMZM2G067985_P15,GRMZM2G104017_P02, GRMZM2G104017_P03,GRMZM2G104017_P04, GRMZM2G110378_P01,GRMZM2G110378_P02, GRMZM2G110378_P04,GRMZM2G110378_P05	TRUE	TRUE	lAYIALDYEQELETAk	95%	n+304 (+304), K+304 (+304)	61.48	25.75
479	seq=translation; coord=8:134521573..134531903:1; parent_transcript=GRMZM2G067985_T04; parent_gene=GRMZM2G067985	GRMZM2G067985_P04,GRMZM2G067985_P05, GRMZM2G067985_P06,GRMZM2G067985_P09, GRMZM2G067985_P10,GRMZM2G067985_P12, GRMZM2G067985_P15,GRMZM2G104017_P02, GRMZM2G104017_P03,GRMZM2G104017_P04, GRMZM2G110378_P01,GRMZM2G110378_P02, GRMZM2G110378_P04,GRMZM2G110378_P05	TRUE	TRUE	lAyIALDYEQELETAk	95%	n+304 (+304), iTRAQ8plex (+304), K+304 (+304)	39.89	25.00
480	seq=translation; coord=1:205422404..205424254:-1; parent_transcript=GRMZM2G456217_T01; parent_gene=GRMZM2G456217	GRMZM2G456217_P01	TRUE	TRUE	aNAGcNGGLMDYAFQYIAk	90%	n+304 (+304), Carbamidomethyl (+57), K+304 (+304)	25.59	25.00
481	seq=translation; coord=1:205422404..205424254:-1; parent_transcript=GRMZM2G456217_T01; parent_gene=GRMZM2G456217	GRMZM2G456217_P01	TRUE	TRUE	aVAHQPVSVAIEASGSHFQFYSEGVFSGR	95%	n+304 (+304)	41.62	25.00
482	seq=translation; coord=1:205422404..205424254:-1; parent_transcript=GRMZM2G456217_T01; parent_gene=GRMZM2G456217	GRMZM2G456217_P01	TRUE	TRUE	cGTELDHGVAAVGYGVTADGTk	89%	Carbamidomethyl (+57), n+304 (+304), K+304 (+304)	25.15	25.00
483	seq=translation; coord=1:205422404..205424254:-1; parent_transcript=GRMZM2G456217_T01; parent_gene=GRMZM2G456217	GRMZM2G456217_P01	TRUE	TRUE	dQGQcGScWAFSTIAAVEGINAIk	95%	n+304 (+304), Carbamidomethyl (+57), Carbamidomethyl (+57), K+304 (+304)	50.59	25.00
484	seq=translation; coord=1:205422404..205424254:-1; parent_transcript=GRMZM2G456217_T01; parent_gene=GRMZM2G456217	GRMZM2G456217_P01	TRUE	TRUE	dVPASVDWR	93%	n+304 (+304)	29.71	25.00
485	seq=translation; coord=1:205422404..205424254:-1; parent_transcript=GRMZM2G456217_T01; parent_gene=GRMZM2G456217	GRMZM2G456217_P01	TRUE	TRUE	eGHcGIAMEASYPVk	95%	n+304 (+304), Carbamidomethyl (+57), K+304 (+304)	65.01	25.00
486	seq=translation; coord=1:205422404..205424254:-1; parent_transcript=GRMZM2G456217_T01; parent_gene=GRMZM2G456217	GRMZM2G456217_P01	TRUE	TRUE	eGHcGIAmEASYPVk	95%	n+304 (+304), Carbamidomethyl (+57), Oxidation (+16), K+304 (+304)	34.38	25.00
487	seq=translation; coord=1:205422404..205424254:-1; parent_transcript=GRMZM2G456217_T01; parent_gene=GRMZM2G456217	GRMZM2G456217_P01	TRUE	TRUE	fGDMTADEFR	95%	n+304 (+304)	35.11	25.00
488	seq=translation; coord=1:205422404..205424254:-1; parent_transcript=GRMZM2G456217_T01; parent_gene=GRMZM2G456217	GRMZM2G456217_P01	TRUE	TRUE	fGDmTADEFR	92%	n+304 (+304), Oxidation (+16)	28.90	25.00

489	seq=translation; coord=1:205422404..205424254:-1; parent_transcript=GRMZM2G456217_T01; parent_gene=GRMZM2G456217	GRMZM2G456217_P01	TRUE	TRUE	gAVTDVk	87%	n+304 (+304), K+304 (+304)	28.48	28.19
490	seq=translation; coord=1:205422404..205424254:-1; parent_transcript=GRMZM2G456217_T01; parent_gene=GRMZM2G456217	GRMZM2G456217_P01	TRUE	TRUE	hGGVAAEDAYPYR	95%	n+304 (+304)	49.45	25.00
491	seq=translation; coord=1:205422404..205424254:-1; parent_transcript=GRMZM2G456217_T01; parent_gene=GRMZM2G456217	GRMZM2G456217_P01	TRUE	TRUE	hGGVAAEDAYPyR	91%	n+304 (+304), iTRAQ8plex (+304)	28.38	25.00
492	seq=translation; coord=1:205422404..205424254:-1; parent_transcript=GRMZM2G456217_T01; parent_gene=GRMZM2G456217	GRMZM2G456217_P01	TRUE	TRUE	kSPAPVVTIDGYEDVPANDESALk	95%	K+304 (+304), n+304 (+304), K+304 (+304)	40.73	25.00
493	seq=translation; coord=1:205422404..205424254:-1; parent_transcript=GRMZM2G456217_T01; parent_gene=GRMZM2G456217	GRMZM2G456217_P01	TRUE	TRUE	lIHEFNR	89%	n+304 (+304)	25.01	25.00
494	seq=translation; coord=1:205422404..205424254:-1; parent_transcript=GRMZM2G456217_T01; parent_gene=GRMZM2G456217	GRMZM2G456217_P01	TRUE	TRUE	nSWGPEWGEk	95%	n+304 (+304), K+304 (+304)	43.94	25.00
495	seq=translation; coord=1:205422404..205424254:-1; parent_transcript=GRMZM2G456217_T01; parent_gene=GRMZM2G456217	GRMZM2G456217_P01	TRUE	TRUE	qGSSASASSFMYADAR	93%	n+304 (+304)	29.43	25.00
496	seq=translation; coord=1:205422404..205424254:-1; parent_transcript=GRMZM2G456217_T01; parent_gene=GRMZM2G456217	GRMZM2G456217_P01	TRUE	TRUE	qGSSASASSFMyADAR	95%	Pyro-cmC (-17), n+304 (+304), iTRAQ8plex (+304)	37.89	25.00
497	seq=translation; coord=1:205422404..205424254:-1; parent_transcript=GRMZM2G456217_T01; parent_gene=GRMZM2G456217	GRMZM2G456217_P01	TRUE	TRUE	sPAPVVTIDGYEDVPANDESALk	95%	n+304 (+304), K+304 (+304)	63.46	25.31
498	seq=translation; coord=1:205422404..205424254:-1; parent_transcript=GRMZM2G456217_T01; parent_gene=GRMZM2G456217	GRMZM2G456217_P01	TRUE	TRUE	vHAVVDEDGSSHDEL	95%	n+304 (+304)	46.86	25.00
499	seq=translation; coord=10:1135114..1145295:1; parent_transcript=GRMZM2G057576_T01; parent_gene=GRMZM2G057576	GRMZM2G057576_P01	TRUE	TRUE	aAEEANVYNDLVk	95%	n+304 (+304), K+304 (+304)	43.67	25.21
500	seq=translation; coord=10:1135114..1145295:1; parent_transcript=GRMZM2G057576_T01; parent_gene=GRMZM2G057576	GRMZM2G057576_P01	TRUE	TRUE	aDDAAHFLDVIR	95%	n+304 (+304)	50.80	25.02
501	seq=translation; coord=10:1135114..1145295:1; parent_transcript=GRMZM2G057576_T01; parent_gene=GRMZM2G057576	GRMZM2G057576_P01	TRUE	TRUE	aFMTADLPHELIELLEk	94%	n+304 (+304), K+304 (+304)	25.67	25.15
502	seq=translation; coord=10:1135114..1145295:1; parent_transcript=GRMZM2G057576_T01; parent_gene=GRMZM2G057576	GRMZM2G057576_P01	TRUE	TRUE	aHMGIFTELGVLYAR	95%	n+304 (+304)	66.61	25.28
503	seq=translation; coord=10:1135114..1145295:1; parent_transcript=GRMZM2G057576_T01; parent_gene=GRMZM2G057576	GRMZM2G057576_P01	TRUE	TRUE	aHMGIFTELGVLyAR	95%	n+304 (+304), iTRAQ8plex (+304)	29.40	25.80
504	seq=translation; coord=10:1135114..1145295:1; parent_transcript=GRMZM2G057576_T01; parent_gene=GRMZM2G057576	GRMZM2G057576_P01	TRUE	TRUE	aNLPGAENLVVQR	95%	n+304 (+304)	53.25	26.33
505	seq=translation; coord=10:1135114..1145295:1; parent_transcript=GRMZM2G057576_T01; parent_gene=GRMZM2G057576	GRMZM2G057576_P01	TRUE	TRUE	aVHFYLQEHPDLINDMLNVLALR	95%	n+304 (+304)	37.60	25.56
506	seq=translation; coord=10:1135114..1145295:1; parent_transcript=GRMZM2G057576_T01; parent_gene=GRMZM2G057576	GRMZM2G057576_P01	TRUE	TRUE	eAAELAAESPQGLLR	95%	n+304 (+304)	56.86	26.27

507	seq=translation; coord=10:1135114..1145295:1; parent_transcript=GRMZM2G057576_T01; parent_gene=GRMZM2G057576	GRMZM2G057576_P01	TRUE	TRUE	eALTLTSLGIAPQFVTFTHVTMESEk	95%	n+304 (+304), K+304 (+304)	29.03	25.00
508	seq=translation; coord=10:1135114..1145295:1; parent_transcript=GRMZM2G057576_T01; parent_gene=GRMZM2G057576	GRMZM2G057576_P01	TRUE	TRUE	eGLVSEAIESFIR	95%	n+304 (+304)	31.75	25.00
509	seq=translation; coord=10:1135114..1145295:1; parent_transcript=GRMZM2G057576_T01; parent_gene=GRMZM2G057576	GRMZM2G057576_P01	TRUE	TRUE	eVcFAcVDAEEFR	95%	n+304 (+304), Carbamidomethyl (+57), Carbamidomethyl (+57)	64.97	25.00
510	seq=translation; coord=10:1135114..1145295:1; parent_transcript=GRMZM2G057576_T01; parent_gene=GRMZM2G057576	GRMZM2G057576_P01	TRUE	TRUE	fGFVPDLTHYLYTNNMLR	95%	n+304 (+304)	34.23	25.00
511	seq=translation; coord=10:1135114..1145295:1; parent_transcript=GRMZM2G057576_T01; parent_gene=GRMZM2G057576	GRMZM2G057576_P01	TRUE	TRUE	fNLNVQAVDVLLDNIR	95%	n+304 (+304)	62.18	25.21
512	seq=translation; coord=10:1135114..1145295:1; parent_transcript=GRMZM2G057576_T01; parent_gene=GRMZM2G057576	GRMZM2G057576_P01	TRUE	TRUE	fQELFAQTk	95%	n+304 (+304), K+304 (+304)	38.14	25.00
513	seq=translation; coord=10:1135114..1145295:1; parent_transcript=GRMZM2G057576_T01; parent_gene=GRMZM2G057576	GRMZM2G057576_P01	TRUE	TRUE	gcFSELIALMESGLGLER	95%	n+304 (+304), Carbamidomethyl (+57)	44.20	25.00
514	seq=translation; coord=10:1135114..1145295:1; parent_transcript=GRMZM2G057576_T01; parent_gene=GRMZM2G057576	GRMZM2G057576_P01	TRUE	TRUE	gNMQLFSVDQQR	95%	n+304 (+304)	35.52	25.00
515	seq=translation; coord=10:1135114..1145295:1; parent_transcript=GRMZM2G057576_T01; parent_gene=GRMZM2G057576	GRMZM2G057576_P01	TRUE	TRUE	gQcDDELINVTNk	95%	n+304 (+304), Carbamidomethyl (+57), K+304 (+304)	41.37	25.00
516	seq=translation; coord=10:1135114..1145295:1; parent_transcript=GRMZM2G057576_T01; parent_gene=GRMZM2G057576	GRMZM2G057576_P01	TRUE	TRUE	iIYAFISNWAk	95%	n+304 (+304), K+304 (+304)	30.48	25.40
517	seq=translation; coord=10:1135114..1145295:1; parent_transcript=GRMZM2G057576_T01; parent_gene=GRMZM2G057576	GRMZM2G057576_P01	TRUE	TRUE	lNAFESLELSR	95%	n+304 (+304)	41.82	25.00
518	seq=translation; coord=10:1135114..1145295:1; parent_transcript=GRMZM2G057576_T01; parent_gene=GRMZM2G057576	GRMZM2G057576_P01	TRUE	TRUE	qVGYTPDYLFLLQTILR	95%	n+304 (+304)	39.16	25.44
519	seq=translation; coord=10:1135114..1145295:1; parent_transcript=GRMZM2G057576_T01; parent_gene=GRMZM2G057576	GRMZM2G057576_P01	TRUE	TRUE	qVGyTPDYLFLLQTILR	95%	n+304 (+304), iTRAQ8plex (+304)	34.79	25.00
520	seq=translation; coord=10:1135114..1145295:1; parent_transcript=GRMZM2G057576_T01; parent_gene=GRMZM2G057576	GRMZM2G057576_P01	TRUE	TRUE	rDPTLAVVAYR	89%	n+304 (+304)	25.20	25.13
521	seq=translation; coord=10:1135114..1145295:1; parent_transcript=GRMZM2G057576_T01; parent_gene=GRMZM2G057576	GRMZM2G057576_P01	TRUE	TRUE	sHQMPEQVVFWk	95%	n+304 (+304), K+304 (+304)	33.03	26.21
522	seq=translation; coord=10:1135114..1145295:1; parent_transcript=GRMZM2G057576_T01; parent_gene=GRMZM2G057576	GRMZM2G057576_P01	TRUE	TRUE	tANLANNQIINYR	95%	n+304 (+304)	55.34	25.90
523	seq=translation; coord=10:1135114..1145295:1; parent_transcript=GRMZM2G057576_T01; parent_gene=GRMZM2G057576	GRMZM2G057576_P01	TRUE	TRUE	tVDNDLALk	95%	n+304 (+304), K+304 (+304)	37.70	26.41
524	seq=translation; coord=10:1135114..1145295:1; parent_transcript=GRMZM2G057576_T01; parent_gene=GRMZM2G057576	GRMZM2G057576_P01	TRUE	TRUE	vDGELIFAYAk	95%	n+304 (+304), K+304 (+304)	37.24	25.54

525	seq=translation; coord=10:1135114..1145295:1; parent_transcript=GRMZM2G057576_T01; parent_gene=GRMZM2G057576	GRMZM2G057576_P01	TRUE	TRUE	vEEDAVWSQVAk	95%	n+304 (+304), K+304 (+304)	36.47	25.93
526	seq=translation; coord=10:1135114..1145295:1; parent_transcript=GRMZM2G057576_T01; parent_gene=GRMZM2G057576	GRMZM2G057576_P01	TRUE	TRUE	vLQPENEYR	88%	n+304 (+304)	26.35	25.00
527	seq=translation; coord=10:1135114..1145295:1; parent_transcript=GRMZM2G057576_T01; parent_gene=GRMZM2G057576	GRMZM2G057576_P01	TRUE	TRUE	vNPGNAPLVVGQLLDDEcPEDFIk	95%	n+304 (+304), Carbamidomethyl (+57), K+304 (+304)	64.03	25.42
528	seq=translation; coord=10:1135114..1145295:1; parent_transcript=GRMZM2G057576_T01; parent_gene=GRMZM2G057576	GRMZM2G057576_P01	TRUE	TRUE	vVGNENPSTLIcFASk	95%	n+304 (+304), Carbamidomethyl (+57), K+304 (+304)	30.11	25.80
529	seq=translation; coord=10:1135114..1145295:1; parent_transcript=GRMZM2G057576_T01; parent_gene=GRMZM2G057576	GRMZM2G057576_P01	TRUE	TRUE	wLVLIGIAPGAPERPQLVk	95%	n+304 (+304), K+304 (+304)	31.20	25.00
530	seq=translation; coord=10:1135114..1145295:1; parent_transcript=GRMZM2G057576_T01; parent_gene=GRMZM2G057576	GRMZM2G057576_P01	TRUE	TRUE	ykEAAELAAESPQGLLR	95%	n+304 (+304), K+304 (+304)	58.37	26.01
531	seq=translation; coord=3:195313538..195319148:1; parent_transcript=GRMZM2G094712_T01; parent_gene=GRMZM2G094712	GRMZM2G094712_P01	TRUE	TRUE	aEQMLINNPSR	95%	n+304 (+304)	42.17	25.00
532	seq=translation; coord=3:195313538..195319148:1; parent_transcript=GRMZM2G094712_T01; parent_gene=GRMZM2G094712	GRMZM2G094712_P01	TRUE	TRUE	dSEMFNEWTLELk	95%	n+304 (+304), K+304 (+304)	30.60	25.00
533	seq=translation; coord=3:195313538..195319148:1; parent_transcript=GRMZM2G094712_T01; parent_gene=GRMZM2G094712	GRMZM2G094712_P01	TRUE	TRUE	eYLPITGLAEFNk	95%	n+304 (+304), K+304 (+304)	31.95	25.56
534	seq=translation; coord=3:195313538..195319148:1; parent_transcript=GRMZM2G094712_T01; parent_gene=GRMZM2G094712	GRMZM2G094712_P01	TRUE	TRUE	eyLPITGLAEFNk	95%	n+304 (+304), iTRAQ8plex (+304), K+304 (+304)	33.96	25.00
535	seq=translation; coord=3:195313538..195319148:1; parent_transcript=GRMZM2G094712_T01; parent_gene=GRMZM2G094712	GRMZM2G094712_P01	TRUE	TRUE	gTPGDWSHIIk	95%	n+304 (+304), K+304 (+304)	36.52	26.52
536	seq=translation; coord=3:195313538..195319148:1; parent_transcript=GRMZM2G094712_T01; parent_gene=GRMZM2G094712	GRMZM2G094712_P01	TRUE	TRUE	iSMAGLSMR	95%	n+304 (+304)	40.37	25.00
537	seq=translation; coord=3:195313538..195319148:1; parent_transcript=GRMZM2G094712_T01; parent_gene=GRMZM2G094712	GRMZM2G094712_P01	TRUE	TRUE	lIFGADSPAIQENR	95%	n+304 (+304)	63.48	25.74
538	seq=translation; coord=3:195313538..195319148:1; parent_transcript=GRMZM2G094712_T01; parent_gene=GRMZM2G094712	GRMZM2G094712_P01	TRUE	TRUE	mFVADGGELLMAQSYAk	95%	n+304 (+304), K+304 (+304)	55.63	25.00
539	seq=translation; coord=3:195313538..195319148:1; parent_transcript=GRMZM2G094712_T01; parent_gene=GRMZM2G094712	GRMZM2G094712_P01	TRUE	TRUE	mFVADGGELLmAQSYAk	95%	n+304 (+304), Oxidation (+16), K+304 (+304)	47.72	25.22
540	seq=translation; coord=3:195313538..195319148:1; parent_transcript=GRMZM2G094712_T01; parent_gene=GRMZM2G094712	GRMZM2G094712_P01	TRUE	TRUE	qEYHIYMTSDGR	91%	Pyro-cmC (-17), n+304 (+304)	27.15	25.00
541	seq=translation; coord=3:195313538..195319148:1; parent_transcript=GRMZM2G094712_T01; parent_gene=GRMZM2G094712	GRMZM2G094712_P01	TRUE	TRUE	qIGMFTFTGLNSEQVAFMR	95%	n+304 (+304)	85.83	25.00
542	seq=translation; coord=3:195313538..195319148:1; parent_transcript=GRMZM2G094712_T01; parent_gene=GRMZM2G094712	GRMZM2G094712_P01	TRUE	TRUE	qQLFNALk	95%	n+304 (+304), K+304 (+304)	37.73	26.35

543	seq=translation; coord=3:195313538..195319148:1; parent_transcript=GRMZM2G094712_T01; parent_gene=GRMZM2G094712	GRMZM2G094712_P01	TRUE	TRUE	rAEQMLINNPSR	91%	n+304 (+304)	27.19	26.23
544	seq=translation; coord=3:195313538..195319148:1; parent_transcript=GRMZM2G094712_T01; parent_gene=GRMZM2G094712	GRMZM2G094712_P01	TRUE	TRUE	sLLPFFDSAYQGFASGSLDk	95%	n+304 (+304), K+304 (+304)	48.61	25.56
545	seq=translation; coord=3:195313538..195319148:1; parent_transcript=GRMZM2G094712_T01; parent_gene=GRMZM2G094712	GRMZM2G094712_P01	TRUE	TRUE	sLLPFFDSAYQGFASGSLDkDAQSVR	95%	n+304 (+304), K+304 (+304)	52.96	25.00
546	seq=translation; coord=3:195313538..195319148:1; parent_transcript=GRMZM2G094712_T01; parent_gene=GRMZM2G094712	GRMZM2G094712_P01	TRUE	TRUE	tEEGkPLVLNVVR	95%	n+304 (+304), K+304 (+304)	29.98	25.00
547	seq=translation; coord=3:195313538..195319148:1; parent_transcript=GRMZM2G094712_T01; parent_gene=GRMZM2G094712	GRMZM2G094712_P01	TRUE	TRUE	tIYIPQPTWGNHPk	92%	n+304 (+304), K+304 (+304)	28.23	25.49
548	seq=translation; coord=3:195313538..195319148:1; parent_transcript=GRMZM2G094712_T01; parent_gene=GRMZM2G094712	GRMZM2G094712_P01	TRUE	TRUE	tVPHLADAIHAAVTQLk	95%	n+304 (+304), K+304 (+304)	31.69	25.00
549	seq=translation; coord=3:195313538..195319148:1; parent_transcript=GRMZM2G094712_T01; parent_gene=GRMZM2G094712	GRMZM2G094712_P01	TRUE	TRUE	vATVQcLSGTGSLR	95%	n+304 (+304), Carbamidomethyl (+57)	47.32	25.00
550	seq=translation; coord=3:195313538..195319148:1; parent_transcript=GRMZM2G094712_T01; parent_gene=GRMZM2G094712	GRMZM2G094712_P01	TRUE	TRUE	vFTLSGLNVR	95%	n+304 (+304)	32.61	25.00
551	seq=translation; coord=3:195313538..195319148:1; parent_transcript=GRMZM2G094712_T01; parent_gene=GRMZM2G094712	GRMZM2G094712_P01	TRUE	TRUE	vGGEFLAR	88%	n+304 (+304)	26.95	26.29
552	seq=translation; coord=3:195313538..195319148:1; parent_transcript=GRMZM2G094712_T01; parent_gene=GRMZM2G094712	GRMZM2G094712_P01	TRUE	TRUE	vkEYLPITGLAEFNk	95%	n+304 (+304), K+304 (+304), K+304 (+304)	28.18	25.00
553	seq=translation; coord=3:195313538..195319148:1; parent_transcript=GRMZM2G094712_T01; parent_gene=GRMZM2G094712	GRMZM2G094712_P01	TRUE	TRUE	vNLGVGAYR	88%	n+304 (+304)	26.20	25.00
554	seq=translation; coord=1:231336278..231341950:-1; parent_transcript=GRMZM2G415359_T02; parent_gene=GRMZM2G415359	GRMZM2G415359_P02	TRUE	TRUE	eFAPSIPEk	94%	n+304 (+304), K+304 (+304)	30.85	26.71
555	seq=translation; coord=1:231336278..231341950:-1; parent_transcript=GRMZM2G415359_T02; parent_gene=GRMZM2G415359	GRMZM2G415359_P02	TRUE	TRUE	eLVSDDEWLNGEFITTVQQR	91%	n+304 (+304)	25.06	25.00
556	seq=translation; coord=1:231336278..231341950:-1; parent_transcript=GRMZM2G415359_T02; parent_gene=GRMZM2G415359	GRMZM2G415359_P02	TRUE	TRUE	gAAIIk	92%	n+304 (+304), K+304 (+304)	28.69	27.28
557	seq=translation; coord=1:231336278..231341950:-1; parent_transcript=GRMZM2G415359_T02; parent_gene=GRMZM2G415359	GRMZM2G415359_P02	TRUE	TRUE	iVQGLPIDEFSR	95%	n+304 (+304)	45.26	25.44
558	seq=translation; coord=1:231336278..231341950:-1; parent_transcript=GRMZM2G415359_T02; parent_gene=GRMZM2G415359	GRMZM2G415359_P02	TRUE	TRUE	kMDATAQELTEEk	95%	K+304 (+304), n+304 (+304), K+304 (+304)	39.66	25.53
559	seq=translation; coord=1:231336278..231341950:-1; parent_transcript=GRMZM2G415359_T02; parent_gene=GRMZM2G415359	GRMZM2G415359_P02	TRUE	TRUE	lNVQVSDVk	95%	n+304 (+304), K+304 (+304)	30.61	26.60
560	seq=translation; coord=1:231336278..231341950:-1; parent_transcript=GRMZM2G415359_T02; parent_gene=GRMZM2G415359	GRMZM2G415359_P02	TRUE	TRUE	mDATAQELTEEk	95%	n+304 (+304), K+304 (+304)	40.59	25.00

561	seq=translation; coord=1:231336278..231341950:-1; parent_transcript=GRMZM2G415359_T02; parent_gene=GRMZM2G415359	GRMZM2G415359_P02	TRUE	TRUE	mELVDAAFPLLk	95%	n+304 (+304), K+304 (+304)	34.48	25.75
562	seq=translation; coord=1:231336278..231341950:-1; parent_transcript=GRMZM2G415359_T02; parent_gene=GRMZM2G415359	GRMZM2G415359_P02	TRUE	TRUE	mELVDAAFPLLk	90%	Oxidation (+16), n+304 (+304), K+304 (+304)	26.20	25.63
563	seq=translation; coord=1:231336278..231341950:-1; parent_transcript=GRMZM2G415359_T02; parent_gene=GRMZM2G415359	GRMZM2G415359_P02	TRUE	TRUE	sQASALEAHAAPNck	95%	n+304 (+304), Carbamidomethyl (+57), K+304 (+304)	29.27	25.02
564	seq=translation; coord=1:231336278..231341950:-1; parent_transcript=GRMZM2G415359_T02; parent_gene=GRMZM2G415359	GRMZM2G415359_P02	TRUE	TRUE	vLVTGAAGQIGYALVPMIAR	95%	n+304 (+304)	50.37	25.00
565	seq=translation; coord=1:231336278..231341950:-1; parent_transcript=GRMZM2G415359_T02; parent_gene=GRMZM2G415359	GRMZM2G415359_P02	TRUE	TRUE	vLVTGAAGQIGyALVPMIAR	95%	n+304 (+304), iTRAQ8plex (+304)	35.40	25.00
566	seq=translation; coord=1:231336278..231341950:-1; parent_transcript=GRMZM2G415359_T02; parent_gene=GRMZM2G415359	GRMZM2G415359_P02	TRUE	TRUE	vLVVANPANTNALILk	95%	n+304 (+304), K+304 (+304)	61.82	25.00
567	seq=translation; coord=6:87119198..87122939:1; parent_transcript=GRMZM2G027995_T01; parent_gene=GRMZM2G027995	GRMZM2G027995_P01,GRMZM2G027995_P02	TRUE	TRUE	dQIYDIFQLLPSk	95%	n+304 (+304), K+304 (+304)	40.66	25.15
568	seq=translation; coord=6:87119198..87122939:1; parent_transcript=GRMZM2G027995_T01; parent_gene=GRMZM2G027995	GRMZM2G027995_P01,GRMZM2G027995_P02	TRUE	TRUE	gFkDQIYDIFQLLPSk	94%	n+304 (+304), K+304 (+304), K+304 (+304)	25.32	25.00
569	seq=translation; coord=6:87119198..87122939:1; parent_transcript=GRMZM2G027995_T01; parent_gene=GRMZM2G027995	GRMZM2G027995_P01,GRMZM2G027995_P02	TRUE	TRUE	iLASGVHVVVGTPGR	95%	n+304 (+304)	41.55	25.31
570	seq=translation; coord=9:87062457..87064566:1; parent_transcript=GRMZM2G153541_T06; parent_gene=GRMZM2G153541	GRMZM2G153541_P06	TRUE	TRUE	mVPTkPMVVETFSQYPPLGR	95%	n+304 (+304), K+304 (+304)	37.98	25.00
571	seq=translation; coord=9:87062457..87064566:1; parent_transcript=GRMZM2G153541_T06; parent_gene=GRMZM2G153541	GRMZM2G153541_P06	TRUE	TRUE	tHINIVVIGHVDSGk	95%	n+304 (+304), K+304 (+304)	27.76	25.56
572	seq=translation; coord=9:122220190..122226863:-1; parent_transcript=GRMZM2G152908_T01; parent_gene=GRMZM2G152908	GRMZM2G152908_P01	TRUE	TRUE	aMENEMLLR	95%	n+304 (+304)	32.36	25.00
573	seq=translation; coord=9:122220190..122226863:-1; parent_transcript=GRMZM2G152908_T01; parent_gene=GRMZM2G152908	GRMZM2G152908_P01	TRUE	TRUE	aSALLVDFFDk	95%	n+304 (+304), K+304 (+304)	44.72	25.88
574	seq=translation; coord=9:122220190..122226863:-1; parent_transcript=GRMZM2G152908_T01; parent_gene=GRMZM2G152908	GRMZM2G152908_P01	TRUE	TRUE	dGAFEDVLR	95%	n+304 (+304)	40.63	25.00
575	seq=translation; coord=9:122220190..122226863:-1; parent_transcript=GRMZM2G152908_T01; parent_gene=GRMZM2G152908	GRMZM2G152908_P01	TRUE	TRUE	dTVGQYESHMAFTMPGLYR	95%	n+304 (+304)	44.52	25.00
576	seq=translation; coord=9:122220190..122226863:-1; parent_transcript=GRMZM2G152908_T01; parent_gene=GRMZM2G152908	GRMZM2G152908_P01	TRUE	TRUE	eSMYPLLNFLR	93%	n+304 (+304)	29.70	25.05
577	seq=translation; coord=9:122220190..122226863:-1; parent_transcript=GRMZM2G152908_T01; parent_gene=GRMZM2G152908	GRMZM2G152908_P01	TRUE	TRUE	fNIVSPGADLSIYFPYTESHk	95%	n+304 (+304), K+304 (+304)	61.23	25.48
578	seq=translation; coord=9:122220190..122226863:-1; parent_transcript=GRMZM2G152908_T01; parent_gene=GRMZM2G152908	GRMZM2G152908_P01	TRUE	TRUE	gMLQPHQIIAEYNNAIPEAER	95%	n+304 (+304)	47.03	25.00

579	seq=translation; coord=9:122220190..122226863:-1; parent_transcript=GRMZM2G152908_T01; parent_gene=GRMZM2G152908	GRMZM2G152908_P01	TRUE	TRUE	gMTMMLNDR	91%	n+304 (+304)	28.10	25.00
580	seq=translation; coord=9:122220190..122226863:-1; parent_transcript=GRMZM2G152908_T01; parent_gene=GRMZM2G152908	GRMZM2G152908_P01	TRUE	TRUE	iGDSLSAHPNELVAVFTR	92%	n+304 (+304)	27.90	25.91
581	seq=translation; coord=9:122220190..122226863:-1; parent_transcript=GRMZM2G152908_T01; parent_gene=GRMZM2G152908	GRMZM2G152908_P01	TRUE	TRUE	lFHDkESMYPLLNFLR	94%	n+304 (+304), K+304 (+304)	26.77	25.75
582	seq=translation; coord=9:122220190..122226863:-1; parent_transcript=GRMZM2G152908_T01; parent_gene=GRMZM2G152908	GRMZM2G152908_P01	TRUE	TRUE	lkDGAFEDVLR	95%	n+304 (+304), K+304 (+304)	37.59	26.00
583	seq=translation; coord=9:122220190..122226863:-1; parent_transcript=GRMZM2G152908_T01; parent_gene=GRMZM2G152908	GRMZM2G152908_P01	TRUE	TRUE	lLPDATGTTcGQR	95%	n+304 (+304), Carbamidomethyl (+57)	38.44	25.00
584	seq=translation; coord=9:122220190..122226863:-1; parent_transcript=GRMZM2G152908_T01; parent_gene=GRMZM2G152908	GRMZM2G152908_P01	TRUE	TRUE	nLTGLVELYGR	95%	n+304 (+304)	40.54	25.00
585	seq=translation; coord=9:122220190..122226863:-1; parent_transcript=GRMZM2G152908_T01; parent_gene=GRMZM2G152908	GRMZM2G152908_P01	TRUE	TRUE	qcGLDITPk	91%	n+304 (+304), Carbamidomethyl (+57), K+304 (+304)	28.24	25.53
586	seq=translation; coord=9:122220190..122226863:-1; parent_transcript=GRMZM2G152908_T01; parent_gene=GRMZM2G152908	GRMZM2G152908_P01	TRUE	TRUE	tMASTVPLAVEGEPSSk	95%	n+304 (+304), K+304 (+304)	30.39	25.28
587	seq=translation; coord=9:122220190..122226863:-1; parent_transcript=GRMZM2G152908_T01; parent_gene=GRMZM2G152908	GRMZM2G152908_P01	TRUE	TRUE	vLGTEHcHILR	95%	n+304 (+304), Carbamidomethyl (+57)	38.99	25.11
588	seq=translation; coord=9:122220190..122226863:-1; parent_transcript=GRMZM2G152908_T01; parent_gene=GRMZM2G152908	GRMZM2G152908_P01	TRUE	TRUE	vNVSELAVEELR	95%	n+304 (+304)	65.16	25.98
589	seq=translation; coord=9:122220190..122226863:-1; parent_transcript=GRMZM2G152908_T01; parent_gene=GRMZM2G152908	GRMZM2G152908_P01	TRUE	TRUE	vPEYLQFk	95%	n+304 (+304), K+304 (+304)	30.98	25.48
590	seq=translation; coord=9:122220190..122226863:-1; parent_transcript=GRMZM2G152908_T01; parent_gene=GRMZM2G152908	GRMZM2G152908_P01	TRUE	TRUE	yLEMLYALk	95%	n+304 (+304), K+304 (+304)	37.58	25.88
591	seq=translation; coord=7:10651271..10653006:1; parent_transcript=GRMZM2G162359_T01; parent_gene=GRMZM2G162359	GRMZM2G162359_P01	TRUE	TRUE	aDNYGGFMIWDR	95%	n+304 (+304)	42.11	25.00
592	seq=translation; coord=7:10651271..10653006:1; parent_transcript=GRMZM2G162359_T01; parent_gene=GRMZM2G162359	GRMZM2G162359_P01	TRUE	TRUE	aLDTGIFER	95%	n+304 (+304)	43.91	25.00
593	seq=translation; coord=7:10651271..10653006:1; parent_transcript=GRMZM2G162359_T01; parent_gene=GRMZM2G162359	GRMZM2G162359_P01	TRUE	TRUE	eAcDSGLYTMVIMSFLDVyGPQR	95%	n+304 (+304), Carbamidomethyl (+57), iTRAQ8plex (+304)	27.93	25.00
594	seq=translation; coord=7:10651271..10653006:1; parent_transcript=GRMZM2G162359_T01; parent_gene=GRMZM2G162359	GRMZM2G162359_P01	TRUE	TRUE	fYVGLTASEmTHGWVHPk	95%	n+304 (+304), Oxidation (+16), K+304 (+304)	29.46	25.00
595	seq=translation; coord=7:10651271..10653006:1; parent_transcript=GRMZM2G162359_T01; parent_gene=GRMZM2G162359	GRMZM2G162359_P01	TRUE	TRUE	gSPADRYDVLALELAk	95%	n+304 (+304), K+304 (+304)	34.20	25.66
596	seq=translation; coord=7:10651271..10653006:1; parent_transcript=GRMZM2G162359_T01; parent_gene=GRMZM2G162359	GRMZM2G162359_P01	TRUE	TRUE	iYDDADcEAR	95%	n+304 (+304), Carbamidomethyl (+57)	48.61	25.00

597	seq=translation; coord=7:10651271..10653006:1; parent_transcript=GRMZM2G162359_T01; parent_gene=GRMZM2G162359	GRMZM2G162359_P01	TRUE	TRUE	nVYYDVAPSAQk	95%	n+304 (+304), K+304 (+304)	45.95	25.51
598	seq=translation; coord=7:10651271..10653006:1; parent_transcript=GRMZM2G162359_T01; parent_gene=GRMZM2G162359	GRMZM2G162359_P01	TRUE	TRUE	rPFGDAWLDGVDLFLER	95%	n+304 (+304)	58.09	25.00
599	seq=translation; coord=7:10651271..10653006:1; parent_transcript=GRMZM2G162359_T01; parent_gene=GRMZM2G162359	GRMZM2G162359_P01	TRUE	TRUE	wHLAWDEWTAAYPATR	95%	n+304 (+304)	62.16	25.00
600	seq=translation; coord=7:10651271..10653006:1; parent_transcript=GRMZM2G162359_T01; parent_gene=GRMZM2G162359	GRMZM2G162359_P01	TRUE	TRUE	wHLAWDEWTAAyPATR	95%	n+304 (+304), iTRAQ8plex (+304)	29.97	25.00
601	seq=translation; coord=7:10651271..10653006:1; parent_transcript=GRMZM2G162359_T01; parent_gene=GRMZM2G162359	GRMZM2G162359_P01	TRUE	TRUE	yDVLALELAk	95%	n+304 (+304), K+304 (+304)	33.41	25.31
602	seq=translation; coord=7:10651271..10653006:1; parent_transcript=GRMZM2G162359_T01; parent_gene=GRMZM2G162359	GRMZM2G162359_P01	TRUE	TRUE	yDVLALELAk	94%	n+304 (+304), iTRAQ8plex (+304), K+304 (+304)	25.37	25.00
603	seq=translation; coord=4:238121524..238123238:-1; parent_transcript=GRMZM2G389768_T01; parent_gene=GRMZM2G389768	GRMZM2G389768_P01	TRUE	TRUE	aVDVTGPDGSFVR	95%	n+304 (+304)	53.84	25.01
604	seq=translation; coord=4:238121524..238123238:-1; parent_transcript=GRMZM2G389768_T01; parent_gene=GRMZM2G389768	GRMZM2G389768_P01	TRUE	TRUE	cGEPGHMAR	95%	Carbamidomethyl (+57), n+304 (+304)	30.80	25.00
605	seq=translation; coord=4:238121524..238123238:-1; parent_transcript=GRMZM2G389768_T01; parent_gene=GRMZM2G389768	GRMZM2G389768_P01	TRUE	TRUE	dcPSADGGGGYGGGGYGGGGYGGGGGGGGGcFk	95%	n+304 (+304), Carbamidomethyl (+57), Carbamidomethyl (+57), K+304 (+304)	34.57	25.00
606	seq=translation; coord=4:238121524..238123238:-1; parent_transcript=GRMZM2G389768_T01; parent_gene=GRMZM2G389768	GRMZM2G389768_P01	TRUE	TRUE	dcSSGGGGYGGGGGGGGGcYNcGQAGHMAR	95%	n+304 (+304), Carbamidomethyl (+57), Carbamidomethyl (+57), Carbamidomethyl (+57)	174.05	25.00
607	seq=translation; coord=4:238121524..238123238:-1; parent_transcript=GRMZM2G389768_T01; parent_gene=GRMZM2G389768	GRMZM2G389768_P01	TRUE	TRUE	dcSSGGGGYGGGGGGGGGcyNcGQAGHMAR	95%	n+304 (+304), Carbamidomethyl (+57), Carbamidomethyl (+57), iTRAQ8plex (+304), Carbamidomethyl (+57)	34.83	25.00
608	seq=translation; coord=4:238121524..238123238:-1; parent_transcript=GRMZM2G389768_T01; parent_gene=GRMZM2G389768	GRMZM2G389768_P01	TRUE	TRUE	dcSSGGGGYGGGGGGGGGcYNcGQAGHmAR	95%	n+304 (+304), Carbamidomethyl (+57), Carbamidomethyl (+57), Carbamidomethyl (+57), Oxidation (+16)	39.47	25.00
609	seq=translation; coord=4:238121524..238123238:-1; parent_transcript=GRMZM2G389768_T01; parent_gene=GRMZM2G389768	GRMZM2G389768_P01	TRUE	TRUE	dcSSGGGGYGGGGGGGGGcyNcGQAGHmAR	95%	n+304 (+304), Carbamidomethyl (+57), Carbamidomethyl (+57), iTRAQ8plex (+304), Carbamidomethyl (+57), Oxidation (+16)	27.90	25.00
610	seq=translation; coord=4:238121524..238123238:-1; parent_transcript=GRMZM2G389768_T01; parent_gene=GRMZM2G389768	GRMZM2G389768_P01	TRUE	TRUE	fGGGGGGGGDR	95%	n+304 (+304)	46.31	25.00
611	seq=translation; coord=4:238121524..238123238:-1; parent_transcript=GRMZM2G389768_T01; parent_gene=GRMZM2G389768	GRMZM2G389768_P01	TRUE	TRUE	scYNcGEAGHIAR	95%	n+304 (+304), Carbamidomethyl (+57), Carbamidomethyl (+57)	74.37	25.00

612	seq=translation; coord=4:238121524..238123238:-1; parent_transcript=GRMZM2G389768_T01; parent_gene=GRMZM2G389768	GRMZM2G389768_P01	TRUE	TRUE	scyNcGEAGHIAR	95%	n+304 (+304), Carbamidomethyl (+57), iTRAQ8plex (+304), Carbamidomethyl (+57)	37.07	25.00
613	seq=translation; coord=4:238121524..238123238:-1; parent_transcript=GRMZM2G389768_T01; parent_gene=GRMZM2G389768	GRMZM2G389768_P01	TRUE	TRUE	sGGGGGPGAcYk	95%	n+304 (+304), Carbamidomethyl (+57), K+304 (+304)	38.82	25.00
614	seq=translation; coord=4:238121524..238123238:-1; parent_transcript=GRMZM2G389768_T01; parent_gene=GRMZM2G389768	GRMZM2G389768_P01	TRUE	TRUE	sLAEGEEVEFSVSEGDDGR	95%	n+304 (+304)	102.09	25.00
615	seq=translation; coord=4:238121524..238123238:-1; parent_transcript=GRMZM2G389768_T01; parent_gene=GRMZM2G389768	GRMZM2G389768_P01	TRUE	TRUE	sYGGSWGGGR	90%	n+304 (+304)	27.23	25.00
616	seq=translation; coord=8:170762955..170767680:-1; parent_transcript=GRMZM5G833389_T02; parent_gene=GRMZM5G833389	GRMZM5G833389_P02	TRUE	TRUE	aLEIAEk	91%	n+304 (+304), K+304 (+304)	28.16	25.92
617	seq=translation; coord=8:170762955..170767680:-1; parent_transcript=GRMZM5G833389_T02; parent_gene=GRMZM5G833389	GRMZM5G833389_P02	TRUE	TRUE	aLEYADFDNFDR	95%	n+304 (+304)	53.24	25.00
618	seq=translation; coord=8:170762955..170767680:-1; parent_transcript=GRMZM5G833389_T02; parent_gene=GRMZM5G833389	GRMZM5G833389_P02	TRUE	TRUE	eSFESGTLHLIGLLSDGGVHSR	95%	n+304 (+304)	61.07	25.69
619	seq=translation; coord=8:170762955..170767680:-1; parent_transcript=GRMZM5G833389_T02; parent_gene=GRMZM5G833389	GRMZM5G833389_P02	TRUE	TRUE	iILDAVEQVGGIYLVTADHGNAEDMVk	94%	n+304 (+304), K+304 (+304)	25.53	25.00
620	seq=translation; coord=1:287062939..287065014:-1; parent_transcript=GRMZM2G026703_T01; parent_gene=GRMZM2G026703	GRMZM2G026703_P01	TRUE	TRUE	aFLQPSHYDADEVMFVk	94%	n+304 (+304), K+304 (+304)	25.06	25.00
621	seq=translation; coord=1:287062939..287065014:-1; parent_transcript=GRMZM2G026703_T01; parent_gene=GRMZM2G026703	GRMZM2G026703_P01	TRUE	TRUE	eGEGVIVLLR	95%	n+304 (+304)	43.22	26.09
622	seq=translation; coord=1:287062939..287065014:-1; parent_transcript=GRMZM2G026703_T01; parent_gene=GRMZM2G026703	GRMZM2G026703_P01	TRUE	TRUE	fTHELLEDAVGNYR	95%	n+304 (+304)	32.46	25.00
623	seq=translation; coord=1:287062939..287065014:-1; parent_transcript=GRMZM2G026703_T01; parent_gene=GRMZM2G026703	GRMZM2G026703_P01	TRUE	TRUE	gEITTASEEQIR	95%	n+304 (+304)	59.98	25.00
624	seq=translation; coord=1:287062939..287065014:-1; parent_transcript=GRMZM2G026703_T01; parent_gene=GRMZM2G026703	GRMZM2G026703_P01	TRUE	TRUE	gSMMAPSYNTR	95%	n+304 (+304)	35.72	25.00
625	seq=translation; coord=1:287062939..287065014:-1; parent_transcript=GRMZM2G026703_T01; parent_gene=GRMZM2G026703	GRMZM2G026703_P01	TRUE	TRUE	iAIVLk	94%	n+304 (+304), K+304 (+304)	27.89	25.00
626	seq=translation; coord=1:287062939..287065014:-1; parent_transcript=GRMZM2G026703_T01; parent_gene=GRMZM2G026703	GRMZM2G026703_P01	TRUE	TRUE	lLAFGADEEQQVDR	95%	n+304 (+304)	64.97	25.00
627	seq=translation; coord=1:287062939..287065014:-1; parent_transcript=GRMZM2G026703_T01; parent_gene=GRMZM2G026703	GRMZM2G026703_P01	TRUE	TRUE	lLDMDVGLANIAR	88%	n+304 (+304)	27.77	26.50
628	seq=translation; coord=1:287062939..287065014:-1; parent_transcript=GRMZM2G026703_T01; parent_gene=GRMZM2G026703	GRMZM2G026703_P01	TRUE	TRUE	vAELEAAPR	95%	n+304 (+304)	34.89	27.43

629	seq=translation; coord=1:287062939..287065014:-1; parent_transcript=GRMZM2G026703_T01; parent_gene=GRMZM2G026703	GRMZM2G026703_P01	TRUE	TRUE	vFLAGTNSALQk	95%	n+304 (+304), K+304 (+304)	40.15	25.53
630	seq=translation; coord=1:287062939..287065014:-1; parent_transcript=GRMZM2G026703_T01; parent_gene=GRMZM2G026703	GRMZM2G026703_P01	TRUE	TRUE	vVMLLSPVVSTSGR	95%	n+304 (+304)	47.97	25.54
631	seq=translation; coord=5:59268337..59270968:1; parent_transcript=GRMZM2G084521_T01; parent_gene=GRMZM2G084521	GRMZM2G084521_P01,GRMZM2G084521_P03	TRUE	TRUE	dLTEVTHk	95%	n+304 (+304), K+304 (+304)	36.22	25.00
632	seq=translation; coord=5:59268337..59270968:1; parent_transcript=GRMZM2G084521_T01; parent_gene=GRMZM2G084521	GRMZM2G084521_P01,GRMZM2G084521_P03	TRUE	TRUE	gGESIYGLk	92%	n+304 (+304), K+304 (+304)	28.53	26.64
633	seq=translation; coord=5:59268337..59270968:1; parent_transcript=GRMZM2G084521_T01; parent_gene=GRMZM2G084521	GRMZM2G084521_P01,GRMZM2G084521_P03	TRUE	TRUE	hTGPGLLSMANAGR	95%	n+304 (+304)	39.18	25.00
634	seq=translation; coord=5:59268337..59270968:1; parent_transcript=GRMZM2G084521_T01; parent_gene=GRMZM2G084521	GRMZM2G084521_P01,GRMZM2G084521_P03	TRUE	TRUE	iIPSFMLQGGDFTLGDGR	95%	n+304 (+304)	41.94	25.00
635	seq=translation; coord=5:59268337..59270968:1; parent_transcript=GRMZM2G084521_T01; parent_gene=GRMZM2G084521	GRMZM2G084521_P01,GRMZM2G084521_P03	TRUE	TRUE	iVMGLFGk	92%	n+304 (+304), K+304 (+304)	28.60	25.00
636	seq=translation; coord=5:59268337..59270968:1; parent_transcript=GRMZM2G084521_T01; parent_gene=GRMZM2G084521	GRMZM2G084521_P01,GRMZM2G084521_P03	TRUE	TRUE	vLSGMDVVYk	95%	n+304 (+304), K+304 (+304)	40.14	25.47
637	seq=translation; coord=5:59268337..59270968:1; parent_transcript=GRMZM2G084521_T01; parent_gene=GRMZM2G084521	GRMZM2G084521_P01,GRMZM2G084521_P03	TRUE	TRUE	vYFDIEIDGkPAGR	92%	n+304 (+304), K+304 (+304)	27.63	26.12
638	seq=translation; coord=4:235443029..235449150:-1; parent_transcript=GRMZM2G421857_T01; parent_gene=GRMZM2G421857	GRMZM2G421857_P01,GRMZM2G421857_P02	TRUE	TRUE	dALAESDk	90%	n+304 (+304), K+304 (+304)	27.90	25.25
639	seq=translation; coord=4:235443029..235449150:-1; parent_transcript=GRMZM2G421857_T01; parent_gene=GRMZM2G421857	GRMZM2G421857_P01,GRMZM2G421857_P02	TRUE	TRUE	dMGYNVSMMADSTSR	95%	n+304 (+304)	72.53	25.00
640	seq=translation; coord=4:235443029..235449150:-1; parent_transcript=GRMZM2G421857_T01; parent_gene=GRMZM2G421857	GRMZM2G421857_P01,GRMZM2G421857_P02	TRUE	TRUE	fDPDFIDIR	95%	n+304 (+304)	34.18	25.00
641	seq=translation; coord=4:235443029..235449150:-1; parent_transcript=GRMZM2G421857_T01; parent_gene=GRMZM2G421857	GRMZM2G421857_P01,GRMZM2G421857_P02	TRUE	TRUE	fEDPAEGEEALVGk	95%	n+304 (+304), K+304 (+304)	43.05	25.00
642	seq=translation; coord=4:235443029..235449150:-1; parent_transcript=GRMZM2G421857_T01; parent_gene=GRMZM2G421857	GRMZM2G421857_P01,GRMZM2G421857_P02	TRUE	TRUE	fTMLQTWPVR	95%	n+304 (+304)	32.82	25.00
643	seq=translation; coord=4:235443029..235449150:-1; parent_transcript=GRMZM2G421857_T01; parent_gene=GRMZM2G421857	GRMZM2G421857_P01,GRMZM2G421857_P02	TRUE	TRUE	kVSGPVVVADGMGGAAMYELVR	95%	K+304 (+304), n+304 (+304)	56.35	25.00
644	seq=translation; coord=4:235443029..235449150:-1; parent_transcript=GRMZM2G421857_T01; parent_gene=GRMZM2G421857	GRMZM2G421857_P01,GRMZM2G421857_P02	TRUE	TRUE	lAADTPLLTGQR	95%	n+304 (+304)	34.65	26.52
645	seq=translation; coord=4:235443029..235449150:-1; parent_transcript=GRMZM2G421857_T01; parent_gene=GRMZM2G421857	GRMZM2G421857_P01,GRMZM2G421857_P02	TRUE	TRUE	lAEMPADSGYPAYLAAR	95%	n+304 (+304)	74.50	25.00
646	seq=translation; coord=4:235443029..235449150:-1; parent_transcript=GRMZM2G421857_T01; parent_gene=GRMZM2G421857	GRMZM2G421857_P01,GRMZM2G421857_P02	TRUE	TRUE	lGDLFYR	92%	n+304 (+304)	28.59	25.00

647	seq=translation; coord=4:235443029..235449150:-1; parent_transcript=GRMZM2G421857_T01; parent_gene=GRMZM2G421857	GRMZM2G421857_P01,GRMZM2G421857_P02	TRUE	TRUE	lYDDLTTGFR	94%	n+304 (+304)	30.30	25.00
648	seq=translation; coord=4:235443029..235449150:-1; parent_transcript=GRMZM2G421857_T01; parent_gene=GRMZM2G421857	GRMZM2G421857_P01,GRMZM2G421857_P02	TRUE	TRUE	sGDVYIPR	95%	n+304 (+304)	37.38	25.00
649	seq=translation; coord=4:235443029..235449150:-1; parent_transcript=GRMZM2G421857_T01; parent_gene=GRMZM2G421857	GRMZM2G421857_P01,GRMZM2G421857_P02	TRUE	TRUE	tTLVANTSNMPVAAR	95%	n+304 (+304)	59.66	25.91
650	seq=translation; coord=4:235443029..235449150:-1; parent_transcript=GRMZM2G421857_T01; parent_gene=GRMZM2G421857	GRMZM2G421857_P01,GRMZM2G421857_P02	TRUE	TRUE	vGHDNLIGEIIR	95%	n+304 (+304)	39.63	26.53
651	seq=translation; coord=4:235443029..235449150:-1; parent_transcript=GRMZM2G421857_T01; parent_gene=GRMZM2G421857	GRMZM2G421857_P01,GRMZM2G421857_P02	TRUE	TRUE	vLDALFPSVLGGTcAIPGAFGcGk	95%	n+304 (+304), Carbamidomethyl (+57), Carbamidomethyl (+57), K+304 (+304)	44.96	25.91
652	seq=translation; coord=4:235443029..235449150:-1; parent_transcript=GRMZM2G421857_T01; parent_gene=GRMZM2G421857	GRMZM2G421857_P01,GRMZM2G421857_P02	TRUE	TRUE	vSGPVVVADGMGGAAMYELVR	95%	n+304 (+304)	69.24	25.00
653	seq=translation; coord=4:235443029..235449150:-1; parent_transcript=GRMZM2G421857_T01; parent_gene=GRMZM2G421857	GRMZM2G421857_P01,GRMZM2G421857_P02	TRUE	TRUE	vTTFEDSEk	95%	n+304 (+304), K+304 (+304)	43.80	25.60
654	seq=translation; coord=4:235443029..235449150:-1; parent_transcript=GRMZM2G421857_T01; parent_gene=GRMZM2G421857	GRMZM2G421857_P01,GRMZM2G421857_P02	TRUE	TRUE	ySNSEAVVYVGcGER	93%	n+304 (+304), Carbamidomethyl (+57)	29.56	25.00
655	seq=translation; coord=1:70424654..70429257:1; parent_transcript=GRMZM2G048324_T01; parent_gene=GRMZM2G048324	GRMZM2G048324_P01	TRUE	TRUE	eNQTIQSVLGTSTR	95%	n+304 (+304)	58.71	25.40
656	seq=translation; coord=1:70424654..70429257:1; parent_transcript=GRMZM2G048324_T01; parent_gene=GRMZM2G048324	GRMZM2G048324_P01	TRUE	TRUE	gIPSLVAIGPTGQTVSR	95%	n+304 (+304)	49.21	25.26
657	seq=translation; coord=1:70424654..70429257:1; parent_transcript=GRMZM2G048324_T01; parent_gene=GRMZM2G048324	GRMZM2G048324_P01	TRUE	TRUE	lEILAEk	90%	n+304 (+304), K+304 (+304)	25.56	25.00
658	seq=translation; coord=1:70424654..70429257:1; parent_transcript=GRMZM2G048324_T01; parent_gene=GRMZM2G048324	GRMZM2G048324_P01	TRUE	TRUE	mPWLAIPQGDIk	95%	n+304 (+304), K+304 (+304)	37.33	25.00
659	seq=translation; coord=1:70424654..70429257:1; parent_transcript=GRMZM2G048324_T01; parent_gene=GRMZM2G048324	GRMZM2G048324_P01	TRUE	TRUE	mPWLAVPFSDSEGR	95%	n+304 (+304)	59.36	25.00
660	seq=translation; coord=1:70424654..70429257:1; parent_transcript=GRMZM2G048324_T01; parent_gene=GRMZM2G048324	GRMZM2G048324_P01	TRUE	TRUE	nEEAFNEYFAk	95%	n+304 (+304), K+304 (+304)	34.82	25.00
661	seq=translation; coord=1:70424654..70429257:1; parent_transcript=GRMZM2G048324_T01; parent_gene=GRMZM2G048324	GRMZM2G048324_P01	TRUE	TRUE	sFEVVFASADR	95%	n+304 (+304)	34.37	25.00
662	seq=translation; coord=1:70424654..70429257:1; parent_transcript=GRMZM2G048324_T01; parent_gene=GRMZM2G048324	GRMZM2G048324_P01	TRUE	TRUE	sFEVVFASADRNEEAFNEYFAk	95%	n+304 (+304), K+304 (+304)	32.00	25.00
663	seq=translation; coord=1:70424654..70429257:1; parent_transcript=GRMZM2G048324_T01; parent_gene=GRMZM2G048324	GRMZM2G048324_P01	TRUE	TRUE	sQLMIHGADAFPFTEER	95%	n+304 (+304)	36.22	25.00

664	seq=translation; coord=1:70424654..70429257:1; parent_transcript=GRMZM2G048324_T01; parent_gene=GRMZM2G048324	GRMZM2G048324_P01	TRUE	TRUE	tVLVYFSAk	95%	n+304 (+304), K+304 (+304)	45.12	25.69
665	seq=translation; coord=1:70424654..70429257:1; parent_transcript=GRMZM2G048324_T01; parent_gene=GRMZM2G048324	GRMZM2G048324_P01	TRUE	TRUE	vPISELEGk	95%	n+304 (+304), K+304 (+304)	31.02	26.13
666	seq=translation; coord=1:70424654..70429257:1; parent_transcript=GRMZM2G048324_T01; parent_gene=GRMZM2G048324	GRMZM2G048324_P01	TRUE	TRUE	vPVSELVGk	95%	n+304 (+304), K+304 (+304)	46.28	25.00
667	seq=translation; coord=1:70424654..70429257:1; parent_transcript=GRMZM2G048324_T01; parent_gene=GRMZM2G048324	GRMZM2G048324_P01	TRUE	TRUE	vSGIPHLVILDAk	95%	n+304 (+304), K+304 (+304)	34.29	25.00
668	seq=translation; coord=1:70424654..70429257:1; parent_transcript=GRMZM2G048324_T01; parent_gene=GRMZM2G048324	GRMZM2G048324_P01	TRUE	TRUE	yFELSSLPTLVLIGPDGk	95%	n+304 (+304), K+304 (+304)	40.62	25.00
669	seq=translation; coord=7:24410021..24413947:-1; parent_transcript=GRMZM2G358059_T01; parent_gene=GRMZM2G358059	GRMZM2G358059_P01	TRUE	TRUE	aAFDEAEk	93%	n+304 (+304), K+304 (+304)	28.26	26.00
670	seq=translation; coord=7:24410021..24413947:-1; parent_transcript=GRMZM2G358059_T01; parent_gene=GRMZM2G358059	GRMZM2G358059_P01	TRUE	TRUE	fGGDTSYSIMFGPDIcGYSTk	95%	n+304 (+304), Carbamidomethyl (+57), K+304 (+304)	59.50	25.00
671	seq=translation; coord=7:24410021..24413947:-1; parent_transcript=GRMZM2G358059_T01; parent_gene=GRMZM2G358059	GRMZM2G358059_P01	TRUE	TRUE	kFGGDTSYSIMFGPDIcGYSTk	95%	K+304 (+304), n+304 (+304), Carbamidomethyl (+57), K+304 (+304)	26.83	25.00
672	seq=translation; coord=7:24410021..24413947:-1; parent_transcript=GRMZM2G358059_T01; parent_gene=GRMZM2G358059	GRMZM2G358059_P01	TRUE	TRUE	lLGGGDVDQk	87%	n+304 (+304), K+304 (+304)	26.82	26.29
673	seq=translation; coord=9:36545543..36547162:1; parent_transcript=GRMZM2G144610_T01; parent_gene=GRMZM2G144610	GRMZM2G144610_P01	TRUE	TRUE	aDGDTVk	93%	n+304 (+304), K+304 (+304)	30.85	27.15
674	seq=translation; coord=9:36545543..36547162:1; parent_transcript=GRMZM2G144610_T01; parent_gene=GRMZM2G144610	GRMZM2G144610_P01	TRUE	TRUE	aINGDVSTLASDGSk	95%	n+304 (+304), K+304 (+304)	33.45	25.87
675	seq=translation; coord=9:36545543..36547162:1; parent_transcript=GRMZM2G144610_T01; parent_gene=GRMZM2G144610	GRMZM2G144610_P01	TRUE	TRUE	aPLSVYLIDAVLLPR	95%	n+304 (+304)	55.34	25.00
676	seq=translation; coord=9:36545543..36547162:1; parent_transcript=GRMZM2G144610_T01; parent_gene=GRMZM2G144610	GRMZM2G144610_P01	TRUE	TRUE	aPLSVyLIDAVLLPR	95%	n+304 (+304), iTRAQ8plex (+304)	34.94	25.00
677	seq=translation; coord=9:36545543..36547162:1; parent_transcript=GRMZM2G144610_T01; parent_gene=GRMZM2G144610	GRMZM2G144610_P01	TRUE	TRUE	aQGSDGIVNITVFPDGR	95%	n+304 (+304)	87.74	25.26
678	seq=translation; coord=9:36545543..36547162:1; parent_transcript=GRMZM2G144610_T01; parent_gene=GRMZM2G144610	GRMZM2G144610_P01	TRUE	TRUE	eQPYYIAVLQVR	95%	n+304 (+304)	32.30	25.59
679	seq=translation; coord=9:36545543..36547162:1; parent_transcript=GRMZM2G144610_T01; parent_gene=GRMZM2G144610	GRMZM2G144610_P01	TRUE	TRUE	eQPyYIAVLQVR	90%	n+304 (+304), iTRAQ8plex (+304)	26.48	25.09
680	seq=translation; coord=9:36545543..36547162:1; parent_transcript=GRMZM2G144610_T01; parent_gene=GRMZM2G144610	GRMZM2G144610_P01	TRUE	TRUE	eQPYyIAVLQVR	90%	n+304 (+304), iTRAQ8plex (+304)	26.74	25.28
681	seq=translation; coord=9:36545543..36547162:1; parent_transcript=GRMZM2G144610_T01; parent_gene=GRMZM2G144610	GRMZM2G144610_P01	TRUE	TRUE	iSSASASAATVTk	95%	n+304 (+304), K+304 (+304)	35.23	25.74

682	seq=translation; coord=9:36545543..36547162:1; parent_transcript=GRMZM2G144610_T01; parent_gene=GRMZM2G144610	GRMZM2G144610_P01	TRUE	TRUE	lGSIQGGFAQAASLYQASGk	95%	n+304 (+304), K+304 (+304)	65.90	25.08
683	seq=translation; coord=9:36545543..36547162:1; parent_transcript=GRMZM2G144610_T01; parent_gene=GRMZM2G144610	GRMZM2G144610_P01	TRUE	TRUE	lQLDDLQR	92%	n+304 (+304)	30.59	26.77
684	seq=translation; coord=9:36545543..36547162:1; parent_transcript=GRMZM2G144610_T01; parent_gene=GRMZM2G144610	GRMZM2G144610_P01	TRUE	TRUE	sTVTVLAVDNAVMAR	95%	n+304 (+304)	69.28	25.47
685	seq=translation; coord=9:36545543..36547162:1; parent_transcript=GRMZM2G144610_T01; parent_gene=GRMZM2G144610	GRMZM2G144610_P01	TRUE	TRUE	sTVTVLAVDNAVmAR	95%	n+304 (+304), Oxidation (+16)	29.67	25.40
686	seq=translation; coord=9:36545543..36547162:1; parent_transcript=GRMZM2G144610_T01; parent_gene=GRMZM2G144610	GRMZM2G144610_P01	TRUE	TRUE	tLLDkAPLSVYLIDAVLLPR	95%	n+304 (+304), K+304 (+304)	38.70	25.00
687	seq=translation; coord=9:36545543..36547162:1; parent_transcript=GRMZM2G144610_T01; parent_gene=GRMZM2G144610	GRMZM2G144610_P01	TRUE	TRUE	vAAFTPSGPSNR	89%	n+304 (+304)	26.95	25.47
688	seq=translation; coord=9:36545543..36547162:1; parent_transcript=GRMZM2G144610_T01; parent_gene=GRMZM2G144610	GRMZM2G144610_P01	TRUE	TRUE	vIYLHVLLDYFDAAk	95%	n+304 (+304), K+304 (+304)	29.45	25.00
689	seq=translation; coord=6:130343970..130350095:1; parent_transcript=GRMZM2G023232_T01; parent_gene=GRMZM2G023232	GRMZM2G023232_P01	TRUE	TRUE	aVLDAATIAGLQPLR	95%	n+304 (+304)	63.07	25.48
690	seq=translation; coord=6:130343970..130350095:1; parent_transcript=GRMZM2G023232_T01; parent_gene=GRMZM2G023232	GRMZM2G023232_P01	TRUE	TRUE	fEHIDMSEk	93%	n+304 (+304), K+304 (+304)	27.22	25.00
691	seq=translation; coord=6:130343970..130350095:1; parent_transcript=GRMZM2G023232_T01; parent_gene=GRMZM2G023232	GRMZM2G023232_P01	TRUE	TRUE	mDTDDAPSDPAVASDVNMQEPk	95%	n+304 (+304), K+304 (+304)	61.61	25.00
692	seq=translation; coord=6:130343970..130350095:1; parent_transcript=GRMZM2G023232_T01; parent_gene=GRMZM2G023232	GRMZM2G023232_P01	TRUE	TRUE	rEEFEQISASVLER	95%	n+304 (+304)	35.21	25.20
693	seq=translation; coord=6:130343970..130350095:1; parent_transcript=GRMZM2G023232_T01; parent_gene=GRMZM2G023232	GRMZM2G023232_P01	TRUE	TRUE	tFTSTQLLAMVLSNLk	95%	n+304 (+304), K+304 (+304)	35.68	25.00
694	seq=translation; coord=6:130343970..130350095:1; parent_transcript=GRMZM2G023232_T01; parent_gene=GRMZM2G023232	GRMZM2G023232_P01	TRUE	TRUE	vGDPIELR	95%	n+304 (+304)	33.67	26.69
695	seq=translation; coord=6:130343970..130350095:1; parent_transcript=GRMZM2G023232_T01; parent_gene=GRMZM2G023232	GRMZM2G023232_P01	TRUE	TRUE	vLTFFk	92%	n+304 (+304), K+304 (+304)	25.71	25.00
696	seq=translation; coord=5:32089861..32094927:-1; parent_transcript=AC233949.1_FGT004; parent_gene=AC233949.1_FG004	AC233949.1_FGP004	TRUE	TRUE	aPHFEESMk	95%	n+304 (+304), K+304 (+304)	29.69	25.00
697	seq=translation; coord=5:32089861..32094927:-1; parent_transcript=AC233949.1_FGT004; parent_gene=AC233949.1_FG004	AC233949.1_FGP004	TRUE	TRUE	dFSTAILER	94%	n+304 (+304)	30.96	25.00
698	seq=translation; coord=5:32089861..32094927:-1; parent_transcript=AC233949.1_FGT004; parent_gene=AC233949.1_FG004	AC233949.1_FGP004	TRUE	TRUE	dTIcIVLADETcEEPk	93%	n+304 (+304), Carbamidomethyl (+57), Carbamidomethyl (+57), K+304 (+304)	27.64	25.00

699	seq=translation; coord=3:216414684..216424048:-1; parent_transcript=GRMZM2G429899_T01; parent_gene=GRMZM2G429899	GRMZM2G429899_P01	TRUE	TRUE	aTPAVPVGGcYR	95%	n+304 (+304), Carbamidomethyl (+57)	31.29	25.00
700	seq=translation; coord=3:216414684..216424048:-1; parent_transcript=GRMZM2G429899_T01; parent_gene=GRMZM2G429899	GRMZM2G429899_P01	TRUE	TRUE	aVLDHSVQAcIFTGYWEDVGTIk	95%	n+304 (+304), Carbamidomethyl (+57), K+304 (+304)	40.37	25.24
701	seq=translation; coord=3:216414684..216424048:-1; parent_transcript=GRMZM2G429899_T01; parent_gene=GRMZM2G429899	GRMZM2G429899_P01	TRUE	TRUE	dALLDLLk	92%	n+304 (+304), K+304 (+304)	26.64	25.00
702	seq=translation; coord=3:216414684..216424048:-1; parent_transcript=GRMZM2G429899_T01; parent_gene=GRMZM2G429899	GRMZM2G429899_P01	TRUE	TRUE	dSVMMGADTYETEEEASk	95%	n+304 (+304), K+304 (+304)	55.81	25.00
703	seq=translation; coord=3:216414684..216424048:-1; parent_transcript=GRMZM2G429899_T01; parent_gene=GRMZM2G429899	GRMZM2G429899_P01	TRUE	TRUE	fDFYDPk	91%	n+304 (+304), K+304 (+304)	25.32	25.00
704	seq=translation; coord=3:216414684..216424048:-1; parent_transcript=GRMZM2G429899_T01; parent_gene=GRMZM2G429899	GRMZM2G429899_P01	TRUE	TRUE	fIWVLEDYYSHk	95%	n+304 (+304), K+304 (+304)	72.39	25.74
705	seq=translation; coord=3:216414684..216424048:-1; parent_transcript=GRMZM2G429899_T01; parent_gene=GRMZM2G429899	GRMZM2G429899_P01	TRUE	TRUE	gIQEADHPEEGYYIR	95%	n+304 (+304)	47.27	25.00
706	seq=translation; coord=3:216414684..216424048:-1; parent_transcript=GRMZM2G429899_T01; parent_gene=GRMZM2G429899	GRMZM2G429899_P01	TRUE	TRUE	hVEDDADITISCAPVDESR	95%	n+304 (+304), Carbamidomethyl (+57)	33.70	25.00
707	seq=translation; coord=3:216414684..216424048:-1; parent_transcript=GRMZM2G429899_T01; parent_gene=GRMZM2G429899	GRMZM2G429899_P01	TRUE	TRUE	iFVMSQFNSTSLNR	95%	n+304 (+304)	58.13	25.00
708	seq=translation; coord=3:216414684..216424048:-1; parent_transcript=GRMZM2G429899_T01; parent_gene=GRMZM2G429899	GRMZM2G429899_P01	TRUE	TRUE	mNYMELVQk	95%	n+304 (+304), K+304 (+304)	46.75	25.25
709	seq=translation; coord=3:216414684..216424048:-1; parent_transcript=GRMZM2G429899_T01; parent_gene=GRMZM2G429899	GRMZM2G429899_P01	TRUE	TRUE	nATINDGSVI	95%	n+304 (+304)	35.43	25.00
710	seq=translation; coord=3:216414684..216424048:-1; parent_transcript=GRMZM2G429899_T01; parent_gene=GRMZM2G429899	GRMZM2G429899_P01	TRUE	TRUE	nVVITNSk	93%	n+304 (+304), K+304 (+304)	29.92	26.62
711	seq=translation; coord=3:216414684..216424048:-1; parent_transcript=GRMZM2G429899_T01; parent_gene=GRMZM2G429899	GRMZM2G429899_P01	TRUE	TRUE	sFFDANLALTEQPSk	95%	n+304 (+304), K+304 (+304)	33.05	25.77
712	seq=translation; coord=3:216414684..216424048:-1; parent_transcript=GRMZM2G429899_T01; parent_gene=GRMZM2G429899	GRMZM2G429899_P01	TRUE	TRUE	sGIVVILk	95%	n+304 (+304), K+304 (+304)	39.65	25.00
713	seq=translation; coord=3:216414684..216424048:-1; parent_transcript=GRMZM2G429899_T01; parent_gene=GRMZM2G429899	GRMZM2G429899_P01	TRUE	TRUE	sIDNIVILSGDQLYR	95%	n+304 (+304)	65.65	25.76
714	seq=translation; coord=3:216414684..216424048:-1; parent_transcript=GRMZM2G429899_T01; parent_gene=GRMZM2G429899	GRMZM2G429899_P01	TRUE	TRUE	tPFFTAPR	91%	n+304 (+304)	25.21	25.00
715	seq=translation; coord=3:216414684..216424048:-1; parent_transcript=GRMZM2G429899_T01; parent_gene=GRMZM2G429899	GRMZM2G429899_P01	TRUE	TRUE	vAATTQcILTSDAcPETLHSQTQSSR	95%	n+304 (+304), Carbamidomethyl (+57), Carbamidomethyl (+57)	69.35	25.00
716	seq=translation; coord=3:216414684..216424048:-1; parent_transcript=GRMZM2G429899_T01; parent_gene=GRMZM2G429899	GRMZM2G429899_P01	TRUE	TRUE	vETNFLSYAIDDAQk	95%	n+304 (+304), K+304 (+304)	66.06	25.83

717	seq=translation; coord=3:216414684..216424048:-1; parent_transcript=GRMZM2G429899_T01; parent_gene=GRMZM2G429899	GRMZM2G429899_P01	TRUE	TRUE	vSAIILGGGTGSQLFPLTSTR	95%	n+304 (+304)	32.25	25.89
718	seq=translation; coord=3:216414684..216424048:-1; parent_transcript=GRMZM2G429899_T01; parent_gene=GRMZM2G429899	GRMZM2G429899_P01	TRUE	TRUE	yPYLASMGIYVFk	95%	n+304 (+304), K+304 (+304)	39.71	25.50
719	seq=translation; coord=5:21697782..21703415:1; parent_transcript=GRMZM2G153815_T01; parent_gene=GRMZM2G153815	GRMZM2G153815_P01	TRUE	TRUE	aAGNEVIGIDLGTTNScVSVMEGk	95%	n+304 (+304), Carbamidomethyl (+57), K+304 (+304)	33.60	25.00
720	seq=translation; coord=5:21697782..21703415:1; parent_transcript=GRMZM2G153815_T01; parent_gene=GRMZM2G153815	GRMZM2G153815_P01	TRUE	TRUE	aLIDIR	95%	n+304 (+304)	30.25	25.00
721	seq=translation; coord=5:21697782..21703415:1; parent_transcript=GRMZM2G153815_T01; parent_gene=GRMZM2G153815	GRMZM2G153815_P01	TRUE	TRUE	dkIPAEVASEIEAAIADLR	95%	n+304 (+304), K+304 (+304)	60.29	25.00
722	seq=translation; coord=5:21697782..21703415:1; parent_transcript=GRMZM2G153815_T01; parent_gene=GRMZM2G153815	GRMZM2G153815_P01	TRUE	TRUE	eTAESYLGk	95%	n+304 (+304), K+304 (+304)	34.13	25.97
723	seq=translation; coord=5:21697782..21703415:1; parent_transcript=GRMZM2G153815_T01; parent_gene=GRMZM2G153815	GRMZM2G153815_P01	TRUE	TRUE	eVDEVLLVGGMTR	95%	n+304 (+304)	45.08	25.00
724	seq=translation; coord=5:21697782..21703415:1; parent_transcript=GRMZM2G153815_T01; parent_gene=GRMZM2G153815	GRMZM2G153815_P01	TRUE	TRUE	fESLVHNLIER	95%	n+304 (+304)	54.54	26.40
725	seq=translation; coord=5:21697782..21703415:1; parent_transcript=GRMZM2G153815_T01; parent_gene=GRMZM2G153815	GRMZM2G153815_P01	TRUE	TRUE	hLNITLTR	95%	n+304 (+304)	33.56	25.39
726	seq=translation; coord=5:21697782..21703415:1; parent_transcript=GRMZM2G153815_T01; parent_gene=GRMZM2G153815	GRMZM2G153815_P01	TRUE	TRUE	iPAEVASEIEAAIADLR	95%	n+304 (+304)	47.45	25.92
727	seq=translation; coord=5:21697782..21703415:1; parent_transcript=GRMZM2G153815_T01; parent_gene=GRMZM2G153815	GRMZM2G153815_P01	TRUE	TRUE	mVQEAELHAQk	95%	n+304 (+304), K+304 (+304)	39.30	25.74
728	seq=translation; coord=5:21697782..21703415:1; parent_transcript=GRMZM2G153815_T01; parent_gene=GRMZM2G153815	GRMZM2G153815_P01	TRUE	TRUE	qAVTNPQNTFFGTk	95%	n+304 (+304), K+304 (+304)	37.07	25.68
729	seq=translation; coord=5:21697782..21703415:1; parent_transcript=GRMZM2G153815_T01; parent_gene=GRMZM2G153815	GRMZM2G153815_P01	TRUE	TRUE	qEMASDDIEk	95%	n+304 (+304), K+304 (+304)	55.41	25.00
730	seq=translation; coord=5:21697782..21703415:1; parent_transcript=GRMZM2G153815_T01; parent_gene=GRMZM2G153815	GRMZM2G153815_P01	TRUE	TRUE	qYSPSQVGAFVLTk	93%	n+304 (+304), K+304 (+304)	27.63	25.00
731	seq=translation; coord=5:21697782..21703415:1; parent_transcript=GRMZM2G153815_T01; parent_gene=GRMZM2G153815	GRMZM2G153815_P01	TRUE	TRUE	sQVFSTAADNQTQVGIR	95%	n+304 (+304)	74.47	25.04
732	seq=translation; coord=5:21697782..21703415:1; parent_transcript=GRMZM2G153815_T01; parent_gene=GRMZM2G153815	GRMZM2G153815_P01	TRUE	TRUE	sSGGLSEADIQk	95%	n+304 (+304), K+304 (+304)	69.89	25.30
733	seq=translation; coord=5:21697782..21703415:1; parent_transcript=GRMZM2G153815_T01; parent_gene=GRMZM2G153815	GRMZM2G153815_P01	TRUE	TRUE	tTPSVVAFTQk	95%	n+304 (+304), K+304 (+304)	32.59	25.00
734	seq=translation; coord=5:21697782..21703415:1; parent_transcript=GRMZM2G153815_T01; parent_gene=GRMZM2G153815	GRMZM2G153815_P01	TRUE	TRUE	vQEVVSEIFGk	95%	n+304 (+304), K+304 (+304)	41.19	26.04

735	seq=translation; coord=2:71110426..71138423:1; parent_transcript=GRMZM2G024933_T02; parent_gene=GRMZM2G024933	GRMZM2G024933_P02	TRUE	TRUE	aFADAGADVLFIDALASVEEMk	95%	n+304 (+304), K+304 (+304)	44.89	25.99
736	seq=translation; coord=2:71110426..71138423:1; parent_transcript=GRMZM2G024933_T02; parent_gene=GRMZM2G024933	GRMZM2G024933_P02	TRUE	TRUE	aFADAGADVLFIDALASVEEmk	95%	n+304 (+304), Oxidation (+16), K+304 (+304)	36.93	25.16
737	seq=translation; coord=2:71110426..71138423:1; parent_transcript=GRMZM2G024933_T02; parent_gene=GRMZM2G024933	GRMZM2G024933_P02	TRUE	TRUE	aMQDALVAIk	95%	n+304 (+304), K+304 (+304)	46.42	26.57
738	seq=translation; coord=2:71110426..71138423:1; parent_transcript=GRMZM2G024933_T02; parent_gene=GRMZM2G024933	GRMZM2G024933_P02	TRUE	TRUE	dGGVPPPSVLPSFQEIk	95%	n+304 (+304), K+304 (+304)	30.16	25.44
739	seq=translation; coord=2:71110426..71138423:1; parent_transcript=GRMZM2G024933_T02; parent_gene=GRMZM2G024933	GRMZM2G024933_P02	TRUE	TRUE	eSGSDIVIVAR	93%	n+304 (+304)	30.06	25.61
740	seq=translation; coord=2:71110426..71138423:1; parent_transcript=GRMZM2G024933_T02; parent_gene=GRMZM2G024933	GRMZM2G024933_P02	TRUE	TRUE	kESGSDIVIVAR	93%	K+304 (+304), n+304 (+304)	29.72	26.25
741	seq=translation; coord=2:71110426..71138423:1; parent_transcript=GRMZM2G024933_T02; parent_gene=GRMZM2G024933	GRMZM2G024933_P02	TRUE	TRUE	lVVYPLSLVGVSMR	95%	n+304 (+304)	49.75	25.35
742	seq=translation; coord=2:71110426..71138423:1; parent_transcript=GRMZM2G024933_T02; parent_gene=GRMZM2G024933	GRMZM2G024933_P02	TRUE	TRUE	lVVyPLSLVGVSMR	94%	n+304 (+304), iTRAQ8plex (+304)	29.01	25.00
743	seq=translation; coord=2:71110426..71138423:1; parent_transcript=GRMZM2G024933_T02; parent_gene=GRMZM2G024933	GRMZM2G024933_P02	TRUE	TRUE	qAISHDEALWR	95%	n+304 (+304)	44.91	25.00
744	seq=translation; coord=2:71110426..71138423:1; parent_transcript=GRMZM2G024933_T02; parent_gene=GRMZM2G024933	GRMZM2G024933_P02	TRUE	TRUE	qAISHDEALWR	86%	Pyro-cmC (-17), n+304 (+304)	25.45	25.00
745	seq=translation; coord=2:71110426..71138423:1; parent_transcript=GRMZM2G024933_T02; parent_gene=GRMZM2G024933	GRMZM2G024933_P02	TRUE	TRUE	tPILSPAELEEIGFR	95%	n+304 (+304)	71.47	25.66
746	seq=translation; coord=1:45512802..45515978:-1; parent_transcript=GRMZM2G087186_T01; parent_gene=GRMZM2G087186	GRMZM2G087186_P01,GRMZM2G087186_P02	TRUE	TRUE	aFVDMVDASGYAYAVMPSAk	95%	n+304 (+304), K+304 (+304)	64.67	25.00
747	seq=translation; coord=1:45512802..45515978:-1; parent_transcript=GRMZM2G087186_T01; parent_gene=GRMZM2G087186	GRMZM2G087186_P01,GRMZM2G087186_P02	TRUE	TRUE	dcLcFIEVIAHk	95%	n+304 (+304), Carbamidomethyl (+57), Carbamidomethyl (+57), K+304 (+304)	47.88	26.32
748	seq=translation; coord=1:45512802..45515978:-1; parent_transcript=GRMZM2G087186_T01; parent_gene=GRMZM2G087186	GRMZM2G087186_P01,GRMZM2G087186_P02	TRUE	TRUE	dPVPFFLTPR	91%	n+304 (+304)	26.58	25.65
749	seq=translation; coord=1:45512802..45515978:-1; parent_transcript=GRMZM2G087186_T01; parent_gene=GRMZM2G087186	GRMZM2G087186_P01,GRMZM2G087186_P02	TRUE	TRUE	eFLSELAk	93%	n+304 (+304), K+304 (+304)	28.32	26.22
750	seq=translation; coord=1:45512802..45515978:-1; parent_transcript=GRMZM2G087186_T01; parent_gene=GRMZM2G087186	GRMZM2G087186_P01,GRMZM2G087186_P02	TRUE	TRUE	iFVPEGQPLESEPNEPLR	95%	n+304 (+304)	47.87	25.58
751	seq=translation; coord=1:45512802..45515978:-1; parent_transcript=GRMZM2G087186_T01; parent_gene=GRMZM2G087186	GRMZM2G087186_P01,GRMZM2G087186_P02	TRUE	TRUE	lVGccNELNAGYAADGYAR	95%	n+304 (+304), Carbamidomethyl (+57), Carbamidomethyl (+57)	37.93	25.00

752	seq=translation; coord=1:45512802..45515978:-1; parent_transcript=GRMZM2G087186_T01; parent_gene=GRMZM2G087186	GRMZM2G087186_P01,GRMZM2G087186_P02	TRUE	TRUE	lVGccNELNAGyAADGYAR	95%	n+304 (+304), Carbamidomethyl (+57), Carbamidomethyl (+57), iTRAQ8plex (+304)	33.69	25.00
753	seq=translation; coord=1:45512802..45515978:-1; parent_transcript=GRMZM2G087186_T01; parent_gene=GRMZM2G087186	GRMZM2G087186_P01,GRMZM2G087186_P02	TRUE	TRUE	mGLEAAVEATVEFLNk	95%	n+304 (+304), K+304 (+304)	36.57	25.37
754	seq=translation; coord=1:45512802..45515978:-1; parent_transcript=GRMZM2G087186_T01; parent_gene=GRMZM2G087186	GRMZM2G087186_P01,GRMZM2G087186_P02	TRUE	TRUE	mGLEAAVEATVEFLNk	95%	Oxidation (+16), n+304 (+304), K+304 (+304)	33.51	25.77
755	seq=translation; coord=1:45512802..45515978:-1; parent_transcript=GRMZM2G087186_T01; parent_gene=GRMZM2G087186	GRMZM2G087186_P01,GRMZM2G087186_P02	TRUE	TRUE	mLTGDSAVIAETGDSWFNcQk	91%	n+304 (+304), Carbamidomethyl (+57), K+304 (+304)	26.09	25.00
756	seq=translation; coord=6:162882094..162884481:1; parent_transcript=GRMZM2G038032_T01; parent_gene=GRMZM2G038032	GRMZM2G038032_P01,GRMZM2G040477_P01	TRUE	TRUE	dGVTLLWDLAEGk	95%	n+304 (+304), K+304 (+304)	35.54	25.61
757	seq=translation; coord=6:162882094..162884481:1; parent_transcript=GRMZM2G038032_T01; parent_gene=GRMZM2G038032	GRMZM2G038032_P01,GRMZM2G040477_P01	TRUE	TRUE	dVLSVAFSVDNR	95%	n+304 (+304)	33.46	25.31
758	seq=translation; coord=6:162882094..162884481:1; parent_transcript=GRMZM2G038032_T01; parent_gene=GRMZM2G038032	GRMZM2G038032_P01,GRMZM2G040477_P01	TRUE	TRUE	fSPNTFQPTIVSGSWDR	95%	n+304 (+304)	73.17	25.00
759	seq=translation; coord=6:162882094..162884481:1; parent_transcript=GRMZM2G038032_T01; parent_gene=GRMZM2G038032	GRMZM2G038032_P01,GRMZM2G040477_P01	TRUE	TRUE	fVGHEk	90%	n+304 (+304), K+304 (+304)	25.58	25.00
760	seq=translation; coord=6:162882094..162884481:1; parent_transcript=GRMZM2G038032_T01; parent_gene=GRMZM2G038032	GRMZM2G038032_P01,GRMZM2G040477_P01	TRUE	TRUE	lWDLSTGLTTR	95%	n+304 (+304)	37.83	25.21
761	seq=translation; coord=6:162882094..162884481:1; parent_transcript=GRMZM2G038032_T01; parent_gene=GRMZM2G038032	GRMZM2G038032_P01,GRMZM2G040477_P01	TRUE	TRUE	lWNTLGEck	95%	n+304 (+304), Carbamidomethyl (+57), K+304 (+304)	33.25	25.16
762	seq=translation; coord=6:162882094..162884481:1; parent_transcript=GRMZM2G038032_T01; parent_gene=GRMZM2G038032	GRMZM2G038032_P01,GRMZM2G040477_P01	TRUE	TRUE	lYSLDAGSIIHSLcFSPNR	95%	n+304 (+304), Carbamidomethyl (+57)	71.33	25.00
763	seq=translation; coord=6:162882094..162884481:1; parent_transcript=GRMZM2G038032_T01; parent_gene=GRMZM2G038032	GRMZM2G038032_P01,GRMZM2G040477_P01	TRUE	TRUE	vWNLTNck	93%	n+304 (+304), Carbamidomethyl (+57), K+304 (+304)	28.23	26.18
764	seq=translation; coord=6:162882094..162884481:1; parent_transcript=GRMZM2G038032_T01; parent_gene=GRMZM2G038032	GRMZM2G038032_P01,GRMZM2G040477_P01	TRUE	TRUE	yWLcAATQDSVk	95%	n+304 (+304), Carbamidomethyl (+57), K+304 (+304)	32.76	25.31
765	seq=translation; coord=6:104066600..104071298:1; parent_transcript=GRMZM2G122871_T01; parent_gene=GRMZM2G122871	GRMZM2G122871_P01	TRUE	TRUE	aLIAAEYSGIk	95%	n+304 (+304), K+304 (+304)	54.83	25.61
766	seq=translation; coord=6:104066600..104071298:1; parent_transcript=GRMZM2G122871_T01; parent_gene=GRMZM2G122871	GRMZM2G122871_P01	TRUE	TRUE	lGYLPYVSTTEETAISSLk	95%	n+304 (+304), K+304 (+304)	71.41	25.72
767	seq=translation; coord=6:104066600..104071298:1; parent_transcript=GRMZM2G122871_T01; parent_gene=GRMZM2G122871	GRMZM2G122871_P01	TRUE	TRUE	lGYLPYVSTTEETAISSLkR	95%	n+304 (+304), K+304 (+304)	32.48	25.00
768	seq=translation; coord=6:104066600..104071298:1; parent_transcript=GRMZM2G122871_T01; parent_gene=GRMZM2G122871	GRMZM2G122871_P01	TRUE	TRUE	mLVIGSEPPFk	95%	n+304 (+304), K+304 (+304)	35.54	25.85

769	seq=translation; coord=5:189820639..189827796:1; parent_transcript=GRMZM2G162688_T01; parent_gene=GRMZM2G162688	GRMZM2G162688_P01	TRUE	TRUE	aGLEDAk	87%	n+304 (+304), K+304 (+304)	28.07	27.57
770	seq=translation; coord=5:189820639..189827796:1; parent_transcript=GRMZM2G162688_T01; parent_gene=GRMZM2G162688	GRMZM2G162688_P01	TRUE	TRUE	aLEHDDEDISYLTNR	95%	n+304 (+304)	41.58	25.00
771	seq=translation; coord=5:189820639..189827796:1; parent_transcript=GRMZM2G162688_T01; parent_gene=GRMZM2G162688	GRMZM2G162688_P01	TRUE	TRUE	aMETYQAGLk	95%	n+304 (+304), K+304 (+304)	51.87	25.74
772	seq=translation; coord=5:189820639..189827796:1; parent_transcript=GRMZM2G162688_T01; parent_gene=GRMZM2G162688	GRMZM2G162688_P01	TRUE	TRUE	aYLNEPDFMQMLR	95%	n+304 (+304)	47.91	25.00
773	seq=translation; coord=5:189820639..189827796:1; parent_transcript=GRMZM2G162688_T01; parent_gene=GRMZM2G162688	GRMZM2G162688_P01	TRUE	TRUE	ayLNEPDFMQMLR	92%	n+304 (+304), iTRAQ8plex (+304)	26.55	25.00
774	seq=translation; coord=5:189820639..189827796:1; parent_transcript=GRMZM2G162688_T01; parent_gene=GRMZM2G162688	GRMZM2G162688_P01	TRUE	TRUE	dFDIAIETYQk	91%	n+304 (+304), K+304 (+304)	26.87	25.73
775	seq=translation; coord=5:189820639..189827796:1; parent_transcript=GRMZM2G162688_T01; parent_gene=GRMZM2G162688	GRMZM2G162688_P01	TRUE	TRUE	dFEAAIQHYTk	94%	n+304 (+304), K+304 (+304)	28.51	25.33
776	seq=translation; coord=5:189820639..189827796:1; parent_transcript=GRMZM2G162688_T01; parent_gene=GRMZM2G162688	GRMZM2G162688_P01	TRUE	TRUE	eVEPEPEPEPMDFTDEEk	95%	n+304 (+304), K+304 (+304)	30.06	25.00
777	seq=translation; coord=5:189820639..189827796:1; parent_transcript=GRMZM2G162688_T01; parent_gene=GRMZM2G162688	GRMZM2G162688_P01	TRUE	TRUE	gLALDPSNEGLk	95%	n+304 (+304), K+304 (+304)	32.38	25.00
778	seq=translation; coord=5:189820639..189827796:1; parent_transcript=GRMZM2G162688_T01; parent_gene=GRMZM2G162688	GRMZM2G162688_P01	TRUE	TRUE	qVLNDFQENPR	95%	n+304 (+304)	35.56	25.00
779	seq=translation; coord=5:189820639..189827796:1; parent_transcript=GRMZM2G162688_T01; parent_gene=GRMZM2G162688	GRMZM2G162688_P01	TRUE	TRUE	rGPSGPDAIGQMFQGPELWSk	95%	n+304 (+304), K+304 (+304)	30.57	25.16
780	seq=translation; coord=8:147686846..147691734:-1; parent_transcript=GRMZM2G432128_T01; parent_gene=GRMZM2G432128	GRMZM2G432128_P01	TRUE	TRUE	aFAEASMTTAYEk	95%	n+304 (+304), K+304 (+304)	38.76	25.00
781	seq=translation; coord=8:147686846..147691734:-1; parent_transcript=GRMZM2G432128_T01; parent_gene=GRMZM2G432128	GRMZM2G432128_P01	TRUE	TRUE	lEAAcVETVESGk	95%	n+304 (+304), Carbamidomethyl (+57), K+304 (+304)	40.31	25.45
782	seq=translation; coord=8:147686846..147691734:-1; parent_transcript=GRMZM2G432128_T01; parent_gene=GRMZM2G432128	GRMZM2G432128_P01	TRUE	TRUE	lILPFLDLDIk	95%	n+304 (+304), K+304 (+304)	45.61	25.00
783	seq=translation; coord=6:74407967..74410838:1; parent_transcript=GRMZM2G108780_T01; parent_gene=GRMZM2G108780	GRMZM2G108780_P01	TRUE	TRUE	kTGMSLIIGVYDEPMTPGQcNMVVER	95%	K+304 (+304), n+304 (+304), Carbamidomethyl (+57)	63.08	25.00
784	seq=translation; coord=6:74407967..74410838:1; parent_transcript=GRMZM2G108780_T01; parent_gene=GRMZM2G108780	GRMZM2G108780_P01	TRUE	TRUE	tGMSLIIGVyDEPMTPGQcNMVVER	95%	n+304 (+304), iTRAQ8plex (+304), Carbamidomethyl (+57)	30.65	25.00
785	seq=translation; coord=1:273983281..273986931:-1; parent_transcript=GRMZM2G442658_T02; parent_gene=GRMZM2G442658	GRMZM2G442658_P02,GRMZM2G442658_P04	TRUE	TRUE	gSTVAVFGLGAVGLAAAEGAR	95%	n+304 (+304)	104.91	25.29
786	seq=translation; coord=1:273983281..273986931:-1; parent_transcript=GRMZM2G442658_T02; parent_gene=GRMZM2G442658	GRMZM2G442658_P02,GRMZM2G442658_P04	TRUE	TRUE	gVMIADGk	95%	n+304 (+304), K+304 (+304)	34.51	26.61

787	seq=translation; coord=1:273983281..273986931:-1;<br>parent_transcript=GRMZM2G442658_T02;<br>parent_gene=GRMZM2G442658	GRMZM2G442658_P02,GRMZM2G442658_P04	TRUE	TRUE	iFGHEAGGIIESVGEGVTDVAPGDHVLPVFTGEck	95%	n+304 (+304), Carbamidomethyl (+57), K+304 (+304)	53.33	25.00
788	seq=translation; coord=1:273983281..273986931:-1;<br>parent_transcript=GRMZM2G442658_T02;<br>parent_gene=GRMZM2G442658	GRMZM2G442658_P02,GRMZM2G442658_P04	TRUE	TRUE	iIGVDLNPSR	95%	n+304 (+304)	33.76	26.95
789	seq=translation; coord=1:273983281..273986931:-1;<br>parent_transcript=GRMZM2G442658_T02;<br>parent_gene=GRMZM2G442658	GRMZM2G442658_P02,GRMZM2G442658_P04	TRUE	TRUE	sAESNMcDLLR	95%	n+304 (+304), Carbamidomethyl (+57)	47.66	25.00
790	seq=translation; coord=1:273983281..273986931:-1;<br>parent_transcript=GRMZM2G442658_T02;<br>parent_gene=GRMZM2G442658	GRMZM2G442658_P02,GRMZM2G442658_P04	TRUE	TRUE	sAESNmcDLLR	88%	n+304 (+304), Oxidation (+16), Carbamidomethyl (+57)	26.04	25.00
791	seq=translation; coord=1:273983281..273986931:-1;<br>parent_transcript=GRMZM2G442658_T02;<br>parent_gene=GRMZM2G442658	GRMZM2G442658_P02,GRMZM2G442658_P04	TRUE	TRUE	sVEcTGNINAMIQAFEcVHDGWGVAVLVGVPHk	95%	n+304 (+304), Carbamidomethyl (+57), Carbamidomethyl (+57), K+304 (+304)	44.66	25.00
792	seq=translation; coord=1:273983281..273986931:-1;<br>parent_transcript=GRMZM2G442658_T02;<br>parent_gene=GRMZM2G442658	GRMZM2G442658_P02,GRMZM2G442658_P04	TRUE	TRUE	tDLPNVVELYMk	94%	n+304 (+304), K+304 (+304)	31.78	26.21
793	seq=translation; coord=1:273983281..273986931:-1;<br>parent_transcript=GRMZM2G442658_T02;<br>parent_gene=GRMZM2G442658	GRMZM2G442658_P02,GRMZM2G442658_P04	TRUE	TRUE	tHPMNFLNER	95%	n+304 (+304)	31.05	25.00
794	seq=translation; coord=1:178476591..178478157:-1;<br>parent_transcript=GRMZM2G080603_T01;<br>parent_gene=GRMZM2G080603	GRMZM2G080603_P01,GRMZM2G080603_P03, GRMZM2G080603_P04	TRUE	TRUE	dGGGGYGGGGGYGGGGGYGGGGGGYGGGNR	95%	n+304 (+304)	114.03	25.00
795	seq=translation; coord=1:178476591..178478157:-1;<br>parent_transcript=GRMZM2G080603_T01;<br>parent_gene=GRMZM2G080603	GRMZM2G080603_P01,GRMZM2G080603_P03, GRMZM2G080603_P04	TRUE	TRUE	dGGGGYGGGGGYGGGGGyGGGGGGYGGGNR	95%	n+304 (+304), iTRAQ8plex (+304)	40.59	25.00
796	seq=translation; coord=1:178476591..178478157:-1;<br>parent_transcript=GRMZM2G080603_T01;<br>parent_gene=GRMZM2G080603	GRMZM2G080603_P01,GRMZM2G080603_P03, GRMZM2G080603_P04	TRUE	TRUE	gFGFVTFSTEEAMR	95%	n+304 (+304)	59.81	25.00
797	seq=translation; coord=1:178476591..178478157:-1;<br>parent_transcript=GRMZM2G080603_T01;<br>parent_gene=GRMZM2G080603	GRMZM2G080603_P01,GRMZM2G080603_P03, GRMZM2G080603_P04	TRUE	TRUE	gFGFVTFSTEEAmR	95%	n+304 (+304), Oxidation (+16)	51.00	25.00
798	seq=translation; coord=1:178476591..178478157:-1;<br>parent_transcript=GRMZM2G080603_T01;<br>parent_gene=GRMZM2G080603	GRMZM2G080603_P01,GRMZM2G080603_P03, GRMZM2G080603_P04	TRUE	TRUE	gGGYGNSDGNWR	95%	n+304 (+304)	50.61	25.00
799	seq=translation; coord=1:178476591..178478157:-1;<br>parent_transcript=GRMZM2G080603_T01;<br>parent_gene=GRMZM2G080603	GRMZM2G080603_P01,GRMZM2G080603_P03, GRMZM2G080603_P04	TRUE	TRUE	nITVNEAQSR	95%	n+304 (+304)	37.12	25.47
800	seq=translation; coord=8:109163217..109164387:-1;<br>parent_transcript=GRMZM2G063536_T01;<br>parent_gene=GRMZM2G063536	GRMZM2G063536_P01	TRUE	TRUE	aAFEWADHPTAVIPDMQk	95%	n+304 (+304), K+304 (+304)	42.05	25.00
801	seq=translation; coord=8:109163217..109164387:-1;<br>parent_transcript=GRMZM2G063536_T01;<br>parent_gene=GRMZM2G063536	GRMZM2G063536_P01	TRUE	TRUE	aVNDLAk	93%	n+304 (+304), K+304 (+304)	28.82	25.65
802	seq=translation; coord=8:109163217..109164387:-1;<br>parent_transcript=GRMZM2G063536_T01;<br>parent_gene=GRMZM2G063536	GRMZM2G063536_P01	TRUE	TRUE	dGDTHLLGDNPR	95%	n+304 (+304)	57.46	25.00
803	seq=translation; coord=8:109163217..109164387:-1;<br>parent_transcript=GRMZM2G063536_T01;<br>parent_gene=GRMZM2G063536	GRMZM2G063536_P01	TRUE	TRUE	gIFQPVLPPEk	95%	n+304 (+304), K+304 (+304)	36.76	25.00

804	seq=translation; coord=8:109163217..109164387:-1; parent_transcript=GRMZM2G063536_T01; parent_gene=GRMZM2G063536	GRMZM2G063536_P01	TRUE	TRUE	gLETVTMGR	93%	n+304 (+304)	28.17	25.00
805	seq=translation; coord=8:109163217..109164387:-1; parent_transcript=GRMZM2G063536_T01; parent_gene=GRMZM2G063536	GRMZM2G063536_P01	TRUE	TRUE	kVPELWFYTELk	95%	K+304 (+304), n+304 (+304), K+304 (+304)	27.10	25.00
806	seq=translation; coord=8:109163217..109164387:-1; parent_transcript=GRMZM2G063536_T01; parent_gene=GRMZM2G063536	GRMZM2G063536_P01	TRUE	TRUE	lVVMVcEGLR	90%	n+304 (+304), Carbamidomethyl (+57)	27.06	25.00
807	seq=translation; coord=8:109163217..109164387:-1; parent_transcript=GRMZM2G063536_T01; parent_gene=GRMZM2G063536	GRMZM2G063536_P01	TRUE	TRUE	mDNLYLVGFR	92%	n+304 (+304)	28.44	25.00
808	seq=translation; coord=8:109163217..109164387:-1; parent_transcript=GRMZM2G063536_T01; parent_gene=GRMZM2G063536	GRMZM2G063536_P01	TRUE	TRUE	mDNLYLVGFR	95%	Oxidation (+16), n+304 (+304)	32.67	25.00
809	seq=translation; coord=8:109163217..109164387:-1; parent_transcript=GRMZM2G063536_T01; parent_gene=GRMZM2G063536	GRMZM2G063536_P01	TRUE	TRUE	tPGGVWWEFGk	95%	n+304 (+304), K+304 (+304)	32.52	25.44
810	seq=translation; coord=8:109163217..109164387:-1; parent_transcript=GRMZM2G063536_T01; parent_gene=GRMZM2G063536	GRMZM2G063536_P01	TRUE	TRUE	tSSITLAIR	95%	n+304 (+304)	33.07	25.00
811	seq=translation; coord=8:109163217..109164387:-1; parent_transcript=GRMZM2G063536_T01; parent_gene=GRMZM2G063536	GRMZM2G063536_P01	TRUE	TRUE	vPELWFYTELk	95%	n+304 (+304), K+304 (+304)	40.91	25.58
812	seq=translation; coord=8:109163217..109164387:-1; parent_transcript=GRMZM2G063536_T01; parent_gene=GRMZM2G063536	GRMZM2G063536_P01	TRUE	TRUE	yQDLIGNk	95%	n+304 (+304), K+304 (+304)	34.28	26.34
813	seq=translation; coord=6:165631098..165635858:-1; parent_transcript=GRMZM2G154595_T01; parent_gene=GRMZM2G154595	GRMZM2G154595_P01	TRUE	TRUE	gFMGDDQLGEALEGSDVVIIPAGVPR	95%	n+304 (+304)	62.81	25.00
814	seq=translation; coord=6:165631098..165635858:-1; parent_transcript=GRMZM2G154595_T01; parent_gene=GRMZM2G154595	GRMZM2G154595_P01	TRUE	TRUE	hcPNALVNMISNPVNSTVPIAAEVFk	95%	n+304 (+304), Carbamidomethyl (+57), K+304 (+304)	36.21	25.00
815	seq=translation; coord=6:165631098..165635858:-1; parent_transcript=GRMZM2G154595_T01; parent_gene=GRMZM2G154595	GRMZM2G154595_P01	TRUE	TRUE	nGVEEVLGLGELNEFEk	95%	n+304 (+304), K+304 (+304)	41.93	25.43
816	seq=translation; coord=4:94432879..94438375:1; parent_transcript=AC207890.3_FGT002; parent_gene=AC207890.3_FG002	AC207890.3_FGP002	TRUE	TRUE	dLPPSAIGEGSGFDAk	95%	n+304 (+304), K+304 (+304)	38.74	25.75
817	seq=translation; coord=4:94432879..94438375:1; parent_transcript=AC207890.3_FGT002; parent_gene=AC207890.3_FG002	AC207890.3_FGP002	TRUE	TRUE	eFGTVYLGDk	95%	n+304 (+304), K+304 (+304)	44.89	25.37
818	seq=translation; coord=4:94432879..94438375:1; parent_transcript=AC207890.3_FGT002; parent_gene=AC207890.3_FG002	AC207890.3_FGP002	TRUE	TRUE	gEIVLAQFSVDNSWNR	95%	n+304 (+304)	57.72	25.00
819	seq=translation; coord=4:94432879..94438375:1; parent_transcript=AC207890.3_FGT002; parent_gene=AC207890.3_FG002	AC207890.3_FGP002	TRUE	TRUE	gFAVSNk	92%	n+304 (+304), K+304 (+304)	29.87	26.95
820	seq=translation; coord=4:94432879..94438375:1; parent_transcript=AC207890.3_FGT002; parent_gene=AC207890.3_FG002	AC207890.3_FGP002	TRUE	TRUE	gGEQNSYLAELLR	95%	n+304 (+304)	56.62	25.00
821	seq=translation; coord=4:94432879..94438375:1; parent_transcript=AC207890.3_FGT002; parent_gene=AC207890.3_FG002	AC207890.3_FGP002	TRUE	TRUE	nVAYSVISAGWAR	95%	n+304 (+304)	41.18	25.00

822	seq=translation; coord=4:94432879..94438375:1; parent_transcript=AC207890.3_FGT002; parent_gene=AC207890.3_FG002	AC207890.3_FGP002	TRUE	TRUE	tALQNLEQFQDk	94%	n+304 (+304), K+304 (+304)	28.60	26.46
823	seq=translation; coord=2:5064059..5067153:-1; parent_transcript=GRMZM2G081886_T01; parent_gene=GRMZM2G081886	GRMZM2G081886_P01	TRUE	TRUE	aLDSGVVAQAALDVFTk	95%	n+304 (+304), K+304 (+304)	45.67	25.00
824	seq=translation; coord=2:5064059..5067153:-1; parent_transcript=GRMZM2G081886_T01; parent_gene=GRMZM2G081886	GRMZM2G081886_P01	TRUE	TRUE	eFANVDcSYGLSPEDLR	95%	n+304 (+304), Carbamidomethyl (+57)	60.42	25.00
825	seq=translation; coord=2:5064059..5067153:-1; parent_transcript=GRMZM2G081886_T01; parent_gene=GRMZM2G081886	GRMZM2G081886_P01	TRUE	TRUE	ePPAADNk	95%	n+304 (+304), K+304 (+304)	29.23	25.47
826	seq=translation; coord=2:5064059..5067153:-1; parent_transcript=GRMZM2G081886_T01; parent_gene=GRMZM2G081886	GRMZM2G081886_P01	TRUE	TRUE	fPSAISETGEITVEGR	95%	n+304 (+304)	90.55	25.00
827	seq=translation; coord=2:5064059..5067153:-1; parent_transcript=GRMZM2G081886_T01; parent_gene=GRMZM2G081886	GRMZM2G081886_P01	TRUE	TRUE	gELAASAVNAPMVPAEVLSELAPFVVLAEk	95%	n+304 (+304), K+304 (+304)	39.53	25.00
828	seq=translation; coord=2:5064059..5067153:-1; parent_transcript=GRMZM2G081886_T01; parent_gene=GRMZM2G081886	GRMZM2G081886_P01	TRUE	TRUE	gGVIDEEALVR	95%	n+304 (+304)	45.98	25.90
829	seq=translation; coord=2:5064059..5067153:-1; parent_transcript=GRMZM2G081886_T01; parent_gene=GRMZM2G081886	GRMZM2G081886_P01	TRUE	TRUE	gLIEPISSVFVNLVNADFTAk	95%	n+304 (+304), K+304 (+304)	43.32	25.00
830	seq=translation; coord=2:5064059..5067153:-1; parent_transcript=GRMZM2G081886_T01; parent_gene=GRMZM2G081886	GRMZM2G081886_P01	TRUE	TRUE	hAVMAIGVDEEPSk	95%	n+304 (+304), K+304 (+304)	44.33	25.42
831	seq=translation; coord=2:5064059..5067153:-1; parent_transcript=GRMZM2G081886_T01; parent_gene=GRMZM2G081886	GRMZM2G081886_P01	TRUE	TRUE	iGEIPAIEEFVFLk	95%	n+304 (+304), K+304 (+304)	47.03	25.00
832	seq=translation; coord=2:5064059..5067153:-1; parent_transcript=GRMZM2G081886_T01; parent_gene=GRMZM2G081886	GRMZM2G081886_P01	TRUE	TRUE	iSLcDALIVR	93%	n+304 (+304), Carbamidomethyl (+57)	29.71	25.13
833	seq=translation; coord=2:5064059..5067153:-1; parent_transcript=GRMZM2G081886_T01; parent_gene=GRMZM2G081886	GRMZM2G081886_P01	TRUE	TRUE	lAVQLVAGGGGIk	95%	n+304 (+304), K+304 (+304)	42.34	25.00
834	seq=translation; coord=2:5064059..5067153:-1; parent_transcript=GRMZM2G081886_T01; parent_gene=GRMZM2G081886	GRMZM2G081886_P01	TRUE	TRUE	lGAAGLALLR	95%	n+304 (+304)	29.09	25.00
835	seq=translation; coord=2:5064059..5067153:-1; parent_transcript=GRMZM2G081886_T01; parent_gene=GRMZM2G081886	GRMZM2G081886_P01	TRUE	TRUE	mLNDEAFAk	95%	n+304 (+304), K+304 (+304)	37.15	25.85
836	seq=translation; coord=2:5064059..5067153:-1; parent_transcript=GRMZM2G081886_T01; parent_gene=GRMZM2G081886	GRMZM2G081886_P01	TRUE	TRUE	nIAQADASLk	95%	n+304 (+304), K+304 (+304)	48.50	26.38
837	seq=translation; coord=2:5064059..5067153:-1; parent_transcript=GRMZM2G081886_T01; parent_gene=GRMZM2G081886	GRMZM2G081886_P01	TRUE	TRUE	tLAILGFGk	95%	n+304 (+304), K+304 (+304)	34.70	25.00
838	seq=translation; coord=1:264200949..264205758:1; parent_transcript=GRMZM2G109130_T01; parent_gene=GRMZM2G109130	GRMZM2G109130_P01	TRUE	TRUE	aITQGIIPAVR	95%	n+304 (+304)	61.50	25.73
839	seq=translation; coord=1:264200949..264205758:1; parent_transcript=GRMZM2G109130_T01; parent_gene=GRMZM2G109130	GRMZM2G109130_P01	TRUE	TRUE	dLLPAGGDYLLk	95%	n+304 (+304), K+304 (+304)	32.38	25.61

840	seq=translation; coord=1:264200949..264205758:1; parent_transcript=GRMZM2G109130_T01; parent_gene=GRMZM2G109130	GRMZM2G109130_P01	TRUE	TRUE	dTPEWTSDAR	95%	n+304 (+304)	34.73	25.00
841	seq=translation; coord=1:264200949..264205758:1; parent_transcript=GRMZM2G109130_T01; parent_gene=GRMZM2G109130	GRMZM2G109130_P01	TRUE	TRUE	eVLAGVNPMVITR	95%	n+304 (+304)	38.22	26.44
842	seq=translation; coord=1:264200949..264205758:1; parent_transcript=GRMZM2G109130_T01; parent_gene=GRMZM2G109130	GRMZM2G109130_P01	TRUE	TRUE	fGVTFDWEVEk	95%	n+304 (+304), K+304 (+304)	40.23	25.94
843	seq=translation; coord=1:264200949..264205758:1; parent_transcript=GRMZM2G109130_T01; parent_gene=GRMZM2G109130	GRMZM2G109130_P01	TRUE	TRUE	fMPFLIDVNNLEGNFIyATR	95%	n+304 (+304), iTRAQ8plex (+304)	39.90	25.66
844	seq=translation; coord=1:264200949..264205758:1; parent_transcript=GRMZM2G109130_T01; parent_gene=GRMZM2G109130	GRMZM2G109130_P01	TRUE	TRUE	gAVTFVANSWVYPAGk	95%	n+304 (+304), K+304 (+304)	33.35	25.25
845	seq=translation; coord=1:264200949..264205758:1; parent_transcript=GRMZM2G109130_T01; parent_gene=GRMZM2G109130	GRMZM2G109130_P01	TRUE	TRUE	gDEELQAWWk	95%	n+304 (+304), K+304 (+304)	31.11	25.00
846	seq=translation; coord=1:264200949..264205758:1; parent_transcript=GRMZM2G109130_T01; parent_gene=GRMZM2G109130	GRMZM2G109130_P01	TRUE	TRUE	gVAVADPSSPYk	95%	n+304 (+304), K+304 (+304)	38.93	25.37
847	seq=translation; coord=1:264200949..264205758:1; parent_transcript=GRMZM2G109130_T01; parent_gene=GRMZM2G109130	GRMZM2G109130_P01	TRUE	TRUE	iQALEDMR	94%	n+304 (+304)	31.84	26.03
848	seq=translation; coord=1:264200949..264205758:1; parent_transcript=GRMZM2G109130_T01; parent_gene=GRMZM2G109130	GRMZM2G109130_P01	TRUE	TRUE	lFPLQLVk	89%	n+304 (+304), K+304 (+304)	25.01	25.00
849	seq=translation; coord=1:264200949..264205758:1; parent_transcript=GRMZM2G109130_T01; parent_gene=GRMZM2G109130	GRMZM2G109130_P01	TRUE	TRUE	lPIPQIIQEDk	95%	n+304 (+304), K+304 (+304)	41.85	25.00
850	seq=translation; coord=1:264200949..264205758:1; parent_transcript=GRMZM2G109130_T01; parent_gene=GRMZM2G109130	GRMZM2G109130_P01	TRUE	TRUE	lSLVEQIYVPR	95%	n+304 (+304)	49.25	25.00
851	seq=translation; coord=1:264200949..264205758:1; parent_transcript=GRMZM2G109130_T01; parent_gene=GRMZM2G109130	GRMZM2G109130_P01	TRUE	TRUE	nLEGLTVQQALDGNR	95%	n+304 (+304)	52.74	25.51
852	seq=translation; coord=1:264200949..264205758:1; parent_transcript=GRMZM2G109130_T01; parent_gene=GRMZM2G109130	GRMZM2G109130_P01	TRUE	TRUE	sWNFTEQGLPADLVk	95%	n+304 (+304), K+304 (+304)	45.13	25.58
853	seq=translation; coord=1:264200949..264205758:1; parent_transcript=GRMZM2G109130_T01; parent_gene=GRMZM2G109130	GRMZM2G109130_P01	TRUE	TRUE	tITLDDVPGR	95%	n+304 (+304)	55.27	25.77
854	seq=translation; coord=1:264200949..264205758:1; parent_transcript=GRMZM2G109130_T01; parent_gene=GRMZM2G109130	GRMZM2G109130_P01	TRUE	TRUE	vGAEANLEQWLTSLPSLTTGESk	94%	n+304 (+304), K+304 (+304)	28.70	25.00
855	seq=translation; coord=1:264200949..264205758:1; parent_transcript=GRMZM2G109130_T01; parent_gene=GRMZM2G109130	GRMZM2G109130_P01	TRUE	TRUE	vYTPASSGVEAWVWQLAk	95%	n+304 (+304), K+304 (+304)	59.51	26.20
856	seq=translation; coord=1:264200949..264205758:1; parent_transcript=GRMZM2G109130_T01; parent_gene=GRMZM2G109130	GRMZM2G109130_P01	TRUE	TRUE	yALGMSSVVYk	94%	n+304 (+304), K+304 (+304)	28.57	25.33
857	seq=translation; coord=6:57744396..57747092:-1; parent_transcript=GRMZM2G127798_T01; parent_gene=GRMZM2G127798	GRMZM2G127798_P01,GRMZM2G127798_P02, GRMZM2G127798_P03	TRUE	TRUE	aEGNLPVYGFHDPASFVk	95%	n+304 (+304), K+304 (+304)	40.57	25.21

858	seq=translation; coord=6:57744396..57747092:-1; parent_transcript=GRMZM2G127798_T01; parent_gene=GRMZM2G127798	GRMZM2G127798_P01,GRMZM2G127798_P02, GRMZM2G127798_P03	TRUE	TRUE	aYDRNPGLASLLVDPEFAQEIMDR	91%	n+304 (+304)	25.78	25.00
859	seq=translation; coord=6:57744396..57747092:-1; parent_transcript=GRMZM2G127798_T01; parent_gene=GRMZM2G127798	GRMZM2G127798_P01,GRMZM2G127798_P02, GRMZM2G127798_P03	TRUE	TRUE	iFQGDYYSTGSPVDk	95%	n+304 (+304), K+304 (+304)	54.01	25.00
860	seq=translation; coord=6:57744396..57747092:-1; parent_transcript=GRMZM2G127798_T01; parent_gene=GRMZM2G127798	GRMZM2G127798_P01,GRMZM2G127798_P02, GRMZM2G127798_P03	TRUE	TRUE	nPGLASLLVDPEFAQEIMDR	95%	n+304 (+304)	65.80	25.00
861	seq=translation; coord=6:57744396..57747092:-1; parent_transcript=GRMZM2G127798_T01; parent_gene=GRMZM2G127798	GRMZM2G127798_P01,GRMZM2G127798_P02, GRMZM2G127798_P03	TRUE	TRUE	yVEDIVLk	90%	n+304 (+304), K+304 (+304)	25.34	25.11
862	seq=translation; coord=7:120173851..120175165:1; parent_transcript=GRMZM2G138689_T01; parent_gene=GRMZM2G138689	GRMZM2G138689_P01	TRUE	TRUE	cSYNYYSSSSNLk	95%	Carbamidomethyl (+57), n+304 (+304), K+304 (+304)	46.89	25.00
863	seq=translation; coord=7:120173851..120175165:1; parent_transcript=GRMZM2G138689_T01; parent_gene=GRMZM2G138689	GRMZM2G138689_P01	TRUE	TRUE	lIQPSScQVLQQQccHDLR	95%	n+304 (+304), Carbamidomethyl (+57), Carbamidomethyl (+57), Carbamidomethyl (+57)	70.90	25.00
864	seq=translation; coord=7:120173851..120175165:1; parent_transcript=GRMZM2G138689_T01; parent_gene=GRMZM2G138689	GRMZM2G138689_P01	TRUE	TRUE	ncHEFLR	95%	n+304 (+304), Carbamidomethyl (+57)	32.25	25.00
865	seq=translation; coord=7:120173851..120175165:1; parent_transcript=GRMZM2G138689_T01; parent_gene=GRMZM2G138689	GRMZM2G138689_P01	TRUE	TRUE	qQcSPLVMPFLQSR	95%	n+304 (+304), Carbamidomethyl (+57)	34.64	25.00
866	seq=translation; coord=7:120173851..120175165:1; parent_transcript=GRMZM2G138689_T01; parent_gene=GRMZM2G138689	GRMZM2G138689_P01	TRUE	TRUE	qQcSPLVmPFLQSR	95%	n+304 (+304), Carbamidomethyl (+57), Oxidation (+16)	50.10	25.00
867	seq=translation; coord=7:120173851..120175165:1; parent_transcript=GRMZM2G138689_T01; parent_gene=GRMZM2G138689	GRMZM2G138689_P01	TRUE	TRUE	qQQPQQPQQYQQGQEk	95%	n+304 (+304), K+304 (+304)	40.36	25.17
868	seq=translation; coord=7:120173851..120175165:1; parent_transcript=GRMZM2G138689_T01; parent_gene=GRMZM2G138689	GRMZM2G138689_P01	TRUE	TRUE	qQQPQQPQQYQQGQEk	95%	Pyro-cmC (-17), n+304 (+304), K+304 (+304)	48.57	25.00
869	seq=translation; coord=7:120173851..120175165:1; parent_transcript=GRMZM2G138689_T01; parent_gene=GRMZM2G138689	GRMZM2G138689_P01	TRUE	TRUE	sQQQQcHcQEQQQTTR	95%	n+304 (+304), Carbamidomethyl (+57), Carbamidomethyl (+57)	30.81	25.00
870	seq=translation; coord=2:40588194..40589174:-1; parent_transcript=GRMZM2G102356_T01; parent_gene=GRMZM2G102356	GRMZM2G102356_P01	TRUE	TRUE	aEGEGGAVVSWAMEFDk	95%	n+304 (+304), K+304 (+304)	54.64	25.00
871	seq=translation; coord=2:40588194..40589174:-1; parent_transcript=GRMZM2G102356_T01; parent_gene=GRMZM2G102356	GRMZM2G102356_P01	TRUE	TRUE	aEGEGGAVVSWAmEFDk	95%	n+304 (+304), Oxidation (+16), K+304 (+304)	32.63	25.00
872	seq=translation; coord=2:40588194..40589174:-1; parent_transcript=GRMZM2G102356_T01; parent_gene=GRMZM2G102356	GRMZM2G102356_P01	TRUE	TRUE	aNDQVPDPDVIk	95%	n+304 (+304), K+304 (+304)	40.08	26.02
873	seq=translation; coord=2:40588194..40589174:-1; parent_transcript=GRMZM2G102356_T01; parent_gene=GRMZM2G102356	GRMZM2G102356_P01	TRUE	TRUE	dSTELFPk	93%	n+304 (+304), K+304 (+304)	30.70	25.53
874	seq=translation; coord=2:40588194..40589174:-1; parent_transcript=GRMZM2G102356_T01; parent_gene=GRMZM2G102356	GRMZM2G102356_P01	TRUE	TRUE	iFPEQYk	87%	n+304 (+304), K+304 (+304)	25.92	25.19

875	seq=translation; coord=2:40588194..40589174:-1; parent_transcript=GRMZM2G102356_T01; parent_gene=GRMZM2G102356	GRMZM2G102356_P01	TRUE	TRUE	lETADDENk	95%	n+304 (+304), K+304 (+304)	39.11	25.00
876	seq=translation; coord=2:40588194..40589174:-1; parent_transcript=GRMZM2G102356_T01; parent_gene=GRMZM2G102356	GRMZM2G102356_P01	TRUE	TRUE	sIETVEGDGk	94%	n+304 (+304), K+304 (+304)	31.46	26.04
877	seq=translation; coord=2:40588194..40589174:-1; parent_transcript=GRMZM2G102356_T01; parent_gene=GRMZM2G102356	GRMZM2G102356_P01	TRUE	TRUE	tFHDLDDYLLk	95%	n+304 (+304), K+304 (+304)	30.67	26.07
878	seq=translation; coord=2:40588194..40589174:-1; parent_transcript=GRMZM2G102356_T01; parent_gene=GRMZM2G102356	GRMZM2G102356_P01	TRUE	TRUE	tFHDLDDYLLkN	94%	n+304 (+304), K+304 (+304)	25.81	25.68
879	seq=translation; coord=2:40588194..40589174:-1; parent_transcript=GRMZM2G102356_T01; parent_gene=GRMZM2G102356	GRMZM2G102356_P01	TRUE	TRUE	vELVVEVk	95%	n+304 (+304), K+304 (+304)	31.53	25.00
880	seq=translation; coord=2:40588194..40589174:-1; parent_transcript=GRMZM2G102356_T01; parent_gene=GRMZM2G102356	GRMZM2G102356_P01	TRUE	TRUE	vVSYSVVDGELADFYk	95%	n+304 (+304), K+304 (+304)	40.78	25.47
881	seq=translation; coord=2:40588194..40589174:-1; parent_transcript=GRMZM2G102356_T01; parent_gene=GRMZM2G102356	GRMZM2G102356_P01	TRUE	TRUE	yTEAVPMLTFAk	95%	n+304 (+304), K+304 (+304)	35.24	25.97
882	seq=translation; coord=8:133171791..133174888:1; parent_transcript=GRMZM5G855672_T02; parent_gene=GRMZM5G855672	GRMZM5G855672_P02	TRUE	TRUE	aAAGNPDTLGDcPFSQR	95%	n+304 (+304), Carbamidomethyl (+57)	56.54	25.00
883	seq=translation; coord=8:133171791..133174888:1; parent_transcript=GRMZM5G855672_T02; parent_gene=GRMZM5G855672	GRMZM5G855672_P02	TRUE	TRUE	aLLDELQALDDHLk	95%	n+304 (+304), K+304 (+304)	41.09	25.31
884	seq=translation; coord=8:133171791..133174888:1; parent_transcript=GRMZM5G855672_T02; parent_gene=GRMZM5G855672	GRMZM5G855672_P02	TRUE	TRUE	cIADSDVITQVIEEk	95%	Carbamidomethyl (+57), n+304 (+304), K+304 (+304)	35.44	25.31
885	seq=translation; coord=8:133171791..133174888:1; parent_transcript=GRMZM5G855672_T02; parent_gene=GRMZM5G855672	GRMZM5G855672_P02	TRUE	TRUE	fPTPSLVTPPEYASVGSk	95%	n+304 (+304), K+304 (+304)	41.00	25.62
886	seq=translation; coord=8:133171791..133174888:1; parent_transcript=GRMZM5G855672_T02; parent_gene=GRMZM5G855672	GRMZM5G855672_P02	TRUE	TRUE	iFPAFVk	86%	n+304 (+304), K+304 (+304)	25.16	25.00
887	seq=translation; coord=8:133171791..133174888:1; parent_transcript=GRMZM5G855672_T02; parent_gene=GRMZM5G855672	GRMZM5G855672_P02	TRUE	TRUE	lFHLQIALEHFk	95%	n+304 (+304), K+304 (+304)	49.59	25.00
888	seq=translation; coord=6:3570233..3573134:1; parent_transcript=GRMZM2G051677_T01; parent_gene=GRMZM2G051677	GRMZM2G051677_P01,GRMZM2G051677_P02	TRUE	TRUE	aAGVLcSYDPNVR	95%	n+304 (+304), Carbamidomethyl (+57)	39.15	25.00
889	seq=translation; coord=6:3570233..3573134:1; parent_transcript=GRMZM2G051677_T01; parent_gene=GRMZM2G051677	GRMZM2G051677_P01,GRMZM2G051677_P02	TRUE	TRUE	aPGGAPANVAcAIAk	95%	n+304 (+304), Carbamidomethyl (+57), K+304 (+304)	39.94	25.44
890	seq=translation; coord=6:3570233..3573134:1; parent_transcript=GRMZM2G051677_T01; parent_gene=GRMZM2G051677	GRMZM2G051677_P01,GRMZM2G051677_P02	TRUE	TRUE	eGILSIWk	93%	n+304 (+304), K+304 (+304)	31.69	26.66
891	seq=translation; coord=6:3570233..3573134:1; parent_transcript=GRMZM2G051677_T01; parent_gene=GRMZM2G051677	GRMZM2G051677_P01,GRMZM2G051677_P02	TRUE	TRUE	fGDDEFGHMLVNILk	95%	n+304 (+304), K+304 (+304)	31.65	25.43
892	seq=translation; coord=6:3570233..3573134:1; parent_transcript=GRMZM2G051677_T01; parent_gene=GRMZM2G051677	GRMZM2G051677_P01,GRMZM2G051677_P02	TRUE	TRUE	gAIPALPTVATAQDLIAk	95%	n+304 (+304), K+304 (+304)	52.60	25.00

893	seq=translation; coord=6:3570233..3573134:1; parent_transcript=GRMZM2G051677_T01; parent_gene=GRMZM2G051677	GRMZM2G051677_P01,GRMZM2G051677_P02	TRUE	TRUE	lGGSSAFVGk	95%	n+304 (+304), K+304 (+304)	28.35	25.54
894	seq=translation; coord=6:3570233..3573134:1; parent_transcript=GRMZM2G051677_T01; parent_gene=GRMZM2G051677	GRMZM2G051677_P01,GRMZM2G051677_P02	TRUE	TRUE	lLVVTDGDk	95%	n+304 (+304), K+304 (+304)	35.69	25.28
895	seq=translation; coord=6:3570233..3573134:1; parent_transcript=GRMZM2G051677_T01; parent_gene=GRMZM2G051677	GRMZM2G051677_P01,GRMZM2G051677_P02	TRUE	TRUE	lPLWPSPDAAR	90%	n+304 (+304)	27.64	25.28
896	seq=translation; coord=6:3570233..3573134:1; parent_transcript=GRMZM2G051677_T01; parent_gene=GRMZM2G051677	GRMZM2G051677_P01,GRMZM2G051677_P02	TRUE	TRUE	nPSADMLLTEAELDLGLVR	95%	n+304 (+304)	77.12	26.25
897	seq=translation; coord=6:3570233..3573134:1; parent_transcript=GRMZM2G051677_T01; parent_gene=GRMZM2G051677	GRMZM2G051677_P01,GRMZM2G051677_P02	TRUE	TRUE	nVLSLWFDGLk	95%	n+304 (+304), K+304 (+304)	57.87	25.69
898	seq=translation; coord=6:3570233..3573134:1; parent_transcript=GRMZM2G051677_T01; parent_gene=GRMZM2G051677	GRMZM2G051677_P01,GRMZM2G051677_P02	TRUE	TRUE	tALAFVTLk	95%	n+304 (+304), K+304 (+304)	31.53	25.00
899	seq=translation; coord=6:3570233..3573134:1; parent_transcript=GRMZM2G051677_T01; parent_gene=GRMZM2G051677	GRMZM2G051677_P01,GRMZM2G051677_P02	TRUE	TRUE	vFHYGSISLISEPcR	95%	n+304 (+304), Carbamidomethyl (+57)	59.59	25.00
900	seq=translation; coord=6:3570233..3573134:1; parent_transcript=GRMZM2G051677_T01; parent_gene=GRMZM2G051677	GRMZM2G051677_P01,GRMZM2G051677_P02	TRUE	TRUE	vSDDEVAFLTR	95%	n+304 (+304)	38.02	25.00
901	seq=translation; coord=2:184127551..184138715:-1; parent_transcript=GRMZM2G130440_T02; parent_gene=GRMZM2G130440	GRMZM2G130440_P02	TRUE	TRUE	aGLMVQQQVSAR	95%	n+304 (+304)	40.83	25.83
902	seq=translation; coord=2:184127551..184138715:-1; parent_transcript=GRMZM2G130440_T02; parent_gene=GRMZM2G130440	GRMZM2G130440_P02	TRUE	TRUE	eAGSDSWk	91%	n+304 (+304), K+304 (+304)	26.84	25.00
903	seq=translation; coord=2:184127551..184138715:-1; parent_transcript=GRMZM2G130440_T02; parent_gene=GRMZM2G130440	GRMZM2G130440_P02	TRUE	TRUE	eAIEINNSVPQGLSSSIFTk	95%	n+304 (+304), K+304 (+304)	30.42	25.68
904	seq=translation; coord=2:184127551..184138715:-1; parent_transcript=GRMZM2G130440_T02; parent_gene=GRMZM2G130440	GRMZM2G130440_P02	TRUE	TRUE	eEHQFLAELGLAQR	95%	n+304 (+304)	51.98	25.51
905	seq=translation; coord=2:184127551..184138715:-1; parent_transcript=GRMZM2G130440_T02; parent_gene=GRMZM2G130440	GRMZM2G130440_P02	TRUE	TRUE	eELFGPVLYVMk	95%	n+304 (+304), K+304 (+304)	42.34	25.05
906	seq=translation; coord=2:184127551..184138715:-1; parent_transcript=GRMZM2G130440_T02; parent_gene=GRMZM2G130440	GRMZM2G130440_P02	TRUE	TRUE	gAPTTPLITIAMTk	95%	n+304 (+304), K+304 (+304)	29.25	25.00
907	seq=translation; coord=2:184127551..184138715:-1; parent_transcript=GRMZM2G130440_T02; parent_gene=GRMZM2G130440	GRMZM2G130440_P02	TRUE	TRUE	gTLLGPLHTPASk	94%	n+304 (+304), K+304 (+304)	27.94	25.00
908	seq=translation; coord=2:184127551..184138715:-1; parent_transcript=GRMZM2G130440_T02; parent_gene=GRMZM2G130440	GRMZM2G130440_P02	TRUE	TRUE	iLFGGSAIESEGNFVQPTIVEITPSAPVVk	95%	n+304 (+304), K+304 (+304)	65.01	25.00
909	seq=translation; coord=2:184127551..184138715:-1; parent_transcript=GRMZM2G130440_T02; parent_gene=GRMZM2G130440	GRMZM2G130440_P02	TRUE	TRUE	iPLVSFTGSTR	95%	n+304 (+304)	40.58	25.20
910	seq=translation; coord=2:184127551..184138715:-1; parent_transcript=GRMZM2G130440_T02; parent_gene=GRMZM2G130440	GRMZM2G130440_P02	TRUE	TRUE	iVASVLEk	95%	n+304 (+304), K+304 (+304)	31.22	25.00

911	seq=translation; coord=2:184127551..184138715:-1; parent_transcript=GRMZM2G130440_T02; parent_gene=GRMZM2G130440	GRMZM2G130440_P02	TRUE	TRUE	lILHENIYQTFLDQLVEVYk	95%	n+304 (+304), K+304 (+304)	39.87	25.00
912	seq=translation; coord=2:184127551..184138715:-1; parent_transcript=GRMZM2G130440_T02; parent_gene=GRMZM2G130440	GRMZM2G130440_P02	TRUE	TRUE	lVSLEMGk	93%	n+304 (+304), K+304 (+304)	29.16	27.02
913	seq=translation; coord=2:184127551..184138715:-1; parent_transcript=GRMZM2G130440_T02; parent_gene=GRMZM2G130440	GRMZM2G130440_P02	TRUE	TRUE	qIGDALR	91%	n+304 (+304)	27.81	25.00
914	seq=translation; coord=2:184127551..184138715:-1; parent_transcript=GRMZM2G130440_T02; parent_gene=GRMZM2G130440	GRMZM2G130440_P02	TRUE	TRUE	sVLFAAVGTAGQR	95%	n+304 (+304)	44.77	25.51
915	seq=translation; coord=2:184127551..184138715:-1; parent_transcript=GRMZM2G130440_T02; parent_gene=GRMZM2G130440	GRMZM2G130440_P02	TRUE	TRUE	tWMAIPAPk	91%	n+304 (+304), K+304 (+304)	28.56	25.42
916	seq=translation; coord=4:241239335..241241698:1; parent_transcript=GRMZM2G134806_T01; parent_gene=GRMZM2G134806	GRMZM2G134806_P01,GRMZM2G134806_P02	TRUE	TRUE	aEAQAAYDk	95%	n+304 (+304), K+304 (+304)	48.94	25.43
917	seq=translation; coord=4:241239335..241241698:1; parent_transcript=GRMZM2G134806_T01; parent_gene=GRMZM2G134806	GRMZM2G134806_P01,GRMZM2G134806_P02	TRUE	TRUE	aMEAISEGk	95%	n+304 (+304), K+304 (+304)	50.35	26.54
918	seq=translation; coord=4:241239335..241241698:1; parent_transcript=GRMZM2G134806_T01; parent_gene=GRMZM2G134806	GRMZM2G134806_P01,GRMZM2G134806_P02	TRUE	TRUE	dANAALEINPDSAk	95%	n+304 (+304), K+304 (+304)	54.43	25.00
919	seq=translation; coord=4:241239335..241241698:1; parent_transcript=GRMZM2G134806_T01; parent_gene=GRMZM2G134806	GRMZM2G134806_P01,GRMZM2G134806_P02	TRUE	TRUE	dFVEAck	89%	n+304 (+304), Carbamidomethyl (+57), K+304 (+304)	26.81	25.31
920	seq=translation; coord=4:241239335..241241698:1; parent_transcript=GRMZM2G134806_T01; parent_gene=GRMZM2G134806	GRMZM2G134806_P01,GRMZM2G134806_P02	TRUE	TRUE	dYLQSLGAk	94%	n+304 (+304), K+304 (+304)	29.46	26.51
921	seq=translation; coord=4:241239335..241241698:1; parent_transcript=GRMZM2G134806_T01; parent_gene=GRMZM2G134806	GRMZM2G134806_P01,GRMZM2G134806_P02	TRUE	TRUE	gFPGGMPGGGFPR	95%	n+304 (+304)	31.69	25.00
922	seq=translation; coord=4:241239335..241241698:1; parent_transcript=GRMZM2G134806_T01; parent_gene=GRMZM2G134806	GRMZM2G134806_P01,GRMZM2G134806_P02	TRUE	TRUE	gMAYAMLGk	95%	n+304 (+304), K+304 (+304)	34.01	25.00
923	seq=translation; coord=4:241239335..241241698:1; parent_transcript=GRMZM2G134806_T01; parent_gene=GRMZM2G134806	GRMZM2G134806_P01,GRMZM2G134806_P02	TRUE	TRUE	iPAAAPSFESPk	95%	n+304 (+304), K+304 (+304)	33.23	25.54
924	seq=translation; coord=4:241239335..241241698:1; parent_transcript=GRMZM2G134806_T01; parent_gene=GRMZM2G134806	GRMZM2G134806_P01,GRMZM2G134806_P02	TRUE	TRUE	kNPSLLADPNLSFFR	95%	K+304 (+304), n+304 (+304)	28.54	25.19
925	seq=translation; coord=4:241239335..241241698:1; parent_transcript=GRMZM2G134806_T01; parent_gene=GRMZM2G134806	GRMZM2G134806_P01,GRMZM2G134806_P02	TRUE	TRUE	mGNPSVEVTEENR	95%	n+304 (+304)	75.29	25.00
926	seq=translation; coord=4:241239335..241241698:1; parent_transcript=GRMZM2G134806_T01; parent_gene=GRMZM2G134806	GRMZM2G134806_P01,GRMZM2G134806_P02	TRUE	TRUE	mGNPSVEVTEENR	95%	Oxidation (+16), n+304 (+304)	38.08	25.00
927	seq=translation; coord=4:241239335..241241698:1; parent_transcript=GRMZM2G134806_T01; parent_gene=GRMZM2G134806	GRMZM2G134806_P01,GRMZM2G134806_P02	TRUE	TRUE	nPSLLADPNLSFFR	95%	n+304 (+304)	38.86	25.04
928	seq=translation; coord=4:241239335..241241698:1; parent_transcript=GRMZM2G134806_T01; parent_gene=GRMZM2G134806	GRMZM2G134806_P01,GRMZM2G134806_P02	TRUE	TRUE	wEEAAHDLHTASNMDYDEEINAVLk	92%	n+304 (+304), K+304 (+304)	26.47	25.00

929	seq=translation; coord=4:13395375..13398777:-1; parent_transcript=GRMZM2G098346_T01; parent_gene=GRMZM2G098346	GRMZM2G098346_P01	TRUE	TRUE	eLELEk	85%	n+304 (+304), K+304 (+304)	26.10	26.09
930	seq=translation; coord=4:13395375..13398777:-1; parent_transcript=GRMZM2G098346_T01; parent_gene=GRMZM2G098346	GRMZM2G098346_P01	TRUE	TRUE	fITHSVPFSEINTAFDLMLk	95%	n+304 (+304), K+304 (+304)	29.76	25.68
931	seq=translation; coord=4:13395375..13398777:-1; parent_transcript=GRMZM2G098346_T01; parent_gene=GRMZM2G098346	GRMZM2G098346_P01	TRUE	TRUE	gSTVAIFGLGAVGLAAMEGAR	95%	n+304 (+304)	79.12	25.00
932	seq=translation; coord=4:13395375..13398777:-1; parent_transcript=GRMZM2G098346_T01; parent_gene=GRMZM2G098346	GRMZM2G098346_P01	TRUE	TRUE	gVMIGDGk	93%	n+304 (+304), K+304 (+304)	31.03	26.48
933	seq=translation; coord=4:13395375..13398777:-1; parent_transcript=GRMZM2G098346_T01; parent_gene=GRMZM2G098346	GRMZM2G098346_P01	TRUE	TRUE	iIGVDINPAk	90%	n+304 (+304), K+304 (+304)	25.43	25.00
934	seq=translation; coord=4:13395375..13398777:-1; parent_transcript=GRMZM2G098346_T01; parent_gene=GRMZM2G098346	GRMZM2G098346_P01	TRUE	TRUE	iLGHEAGGIVESVGEGVTELAPGDHVLPVFTGEck	95%	n+304 (+304), Carbamidomethyl (+57), K+304 (+304)	33.13	25.00
935	seq=translation; coord=4:13395375..13398777:-1; parent_transcript=GRMZM2G098346_T01; parent_gene=GRMZM2G098346	GRMZM2G098346_P01	TRUE	TRUE	iLYTALcHTDVYFWEAk	95%	n+304 (+304), Carbamidomethyl (+57), K+304 (+304)	50.37	26.04
936	seq=translation; coord=4:13395375..13398777:-1; parent_transcript=GRMZM2G098346_T01; parent_gene=GRMZM2G098346	GRMZM2G098346_P01	TRUE	TRUE	iNPEAPLDk	95%	n+304 (+304), K+304 (+304)	36.08	25.00
937	seq=translation; coord=4:13395375..13398777:-1; parent_transcript=GRMZM2G098346_T01; parent_gene=GRMZM2G098346	GRMZM2G098346_P01	TRUE	TRUE	sEESNMcDLLR	95%	n+304 (+304), Carbamidomethyl (+57)	54.92	25.00
938	seq=translation; coord=4:13395375..13398777:-1; parent_transcript=GRMZM2G098346_T01; parent_gene=GRMZM2G098346	GRMZM2G098346_P01	TRUE	TRUE	sEESNmcDLLR	95%	n+304 (+304), Oxidation (+16), Carbamidomethyl (+57)	34.67	25.00
939	seq=translation; coord=4:13395375..13398777:-1; parent_transcript=GRMZM2G098346_T01; parent_gene=GRMZM2G098346	GRMZM2G098346_P01	TRUE	TRUE	tDLPNVVEMYMk	95%	n+304 (+304), K+304 (+304)	40.33	25.43
940	seq=translation; coord=4:13395375..13398777:-1; parent_transcript=GRMZM2G098346_T01; parent_gene=GRMZM2G098346	GRMZM2G098346_P01	TRUE	TRUE	tDLPNVVEMYmk	95%	n+304 (+304), Oxidation (+16), K+304 (+304)	38.67	25.00
941	seq=translation; coord=2:41302953..41306124:-1; parent_transcript=GRMZM2G102499_T02; parent_gene=GRMZM2G102499	GRMZM2G102499_P02,GRMZM2G102499_P03, GRMZM2G102499_P04	TRUE	TRUE	dAAENTMVAYk	95%	n+304 (+304), K+304 (+304)	68.97	25.00
942	seq=translation; coord=2:41302953..41306124:-1; parent_transcript=GRMZM2G102499_T02; parent_gene=GRMZM2G102499	GRMZM2G102499_P02,GRMZM2G102499_P03, GRMZM2G102499_P04	TRUE	TRUE	dNLTLWTSDISEDPAEEIR	95%	n+304 (+304)	45.42	25.00
943	seq=translation; coord=2:41302953..41306124:-1; parent_transcript=GRMZM2G102499_T02; parent_gene=GRMZM2G102499	GRMZM2G102499_P02,GRMZM2G102499_P03, GRMZM2G102499_P04	TRUE	TRUE	icDGILk	86%	n+304 (+304), Carbamidomethyl (+57), K+304 (+304)	27.40	27.00
944	seq=translation; coord=2:41302953..41306124:-1; parent_transcript=GRMZM2G102499_T02; parent_gene=GRMZM2G102499	GRMZM2G102499_P02,GRMZM2G102499_P03, GRMZM2G102499_P04	TRUE	TRUE	iETELTk	95%	n+304 (+304), K+304 (+304)	36.02	25.00
945	seq=translation; coord=2:41302953..41306124:-1; parent_transcript=GRMZM2G102499_T02; parent_gene=GRMZM2G102499	GRMZM2G102499_P02,GRMZM2G102499_P03, GRMZM2G102499_P04	TRUE	TRUE	tVDSEELTVEER	95%	n+304 (+304)	42.87	25.00
946	seq=translation; coord=2:41302953..41306124:-1; parent_transcript=GRMZM2G102499_T02; parent_gene=GRMZM2G102499	GRMZM2G102499_P02,GRMZM2G102499_P03, GRMZM2G102499_P04	TRUE	TRUE	yEEMVEFMEk	95%	n+304 (+304), K+304 (+304)	30.93	25.00

947	seq=translation; coord=7:168785597..168786938:1; parent_transcript=GRMZM2G129761_T01; parent_gene=GRMZM2G129761	GRMZM2G129761_P01	TRUE	TRUE	aLHVVGPDk	95%	n+304 (+304), K+304 (+304)	31.13	25.90
948	seq=translation; coord=7:168785597..168786938:1; parent_transcript=GRMZM2G129761_T01; parent_gene=GRMZM2G129761	GRMZM2G129761_P01	TRUE	TRUE	aVDSLLTAAk	95%	n+304 (+304), K+304 (+304)	34.86	25.00
949	seq=translation; coord=7:168785597..168786938:1; parent_transcript=GRMZM2G129761_T01; parent_gene=GRMZM2G129761	GRMZM2G129761_P01	TRUE	TRUE	dVEAYGGk	95%	n+304 (+304), K+304 (+304)	29.21	25.42
950	seq=translation; coord=7:168785597..168786938:1; parent_transcript=GRMZM2G129761_T01; parent_gene=GRMZM2G129761	GRMZM2G129761_P01	TRUE	TRUE	iHDYVGDGYAIIFSHPADFTPVcTTEMAAMAGYAk	94%	n+304 (+304), Carbamidomethyl (+57), K+304 (+304)	25.81	25.00
951	seq=translation; coord=7:168785597..168786938:1; parent_transcript=GRMZM2G129761_T01; parent_gene=GRMZM2G129761	GRMZM2G129761_P01	TRUE	TRUE	lLGIScDDVESHR	95%	n+304 (+304), Carbamidomethyl (+57)	64.68	25.00
952	seq=translation; coord=7:168785597..168786938:1; parent_transcript=GRMZM2G129761_T01; parent_gene=GRMZM2G129761	GRMZM2G129761_P01	TRUE	TRUE	lSFLYPATTGR	87%	n+304 (+304)	26.92	26.31
953	seq=translation; coord=7:168785597..168786938:1; parent_transcript=GRMZM2G129761_T01; parent_gene=GRMZM2G129761	GRMZM2G129761_P01	TRUE	TRUE	mFPQGFETADLPSk	95%	n+304 (+304), K+304 (+304)	32.61	25.51
954	seq=translation; coord=7:168785597..168786938:1; parent_transcript=GRMZM2G129761_T01; parent_gene=GRMZM2G129761	GRMZM2G129761_P01	TRUE	TRUE	mFPQGFETADLPSk	95%	Oxidation (+16), n+304 (+304), K+304 (+304)	39.97	25.02
955	seq=translation; coord=7:168785597..168786938:1; parent_transcript=GRMZM2G129761_T01; parent_gene=GRMZM2G129761	GRMZM2G129761_P01	TRUE	TRUE	mFPQGFETADLPSkk	95%	n+304 (+304), K+304 (+304), K+304 (+304)	30.17	26.50
956	seq=translation; coord=7:168785597..168786938:1; parent_transcript=GRMZM2G129761_T01; parent_gene=GRMZM2G129761	GRMZM2G129761_P01	TRUE	TRUE	nMDEVLR	92%	n+304 (+304)	28.44	25.00
957	seq=translation; coord=7:168785597..168786938:1; parent_transcript=GRMZM2G129761_T01; parent_gene=GRMZM2G129761	GRMZM2G129761_P01	TRUE	TRUE	qLNMVDPDEk	95%	n+304 (+304), K+304 (+304)	42.37	25.09
958	seq=translation; coord=7:168785597..168786938:1; parent_transcript=GRMZM2G129761_T01; parent_gene=GRMZM2G129761	GRMZM2G129761_P01	TRUE	TRUE	qLNMVDPDEkDAAGR	95%	n+304 (+304), K+304 (+304)	45.87	25.00
959	seq=translation; coord=7:168785597..168786938:1; parent_transcript=GRMZM2G129761_T01; parent_gene=GRMZM2G129761	GRMZM2G129761_P01	TRUE	TRUE	qLNMVDPDEkDAAGR	91%	Pyro-cmC (-17), n+304 (+304), K+304 (+304)	28.33	25.00
960	seq=translation; coord=7:168785597..168786938:1; parent_transcript=GRMZM2G129761_T01; parent_gene=GRMZM2G129761	GRMZM2G129761_P01	TRUE	TRUE	vATPANWkPGEcAVIAPGVSDEEAR	94%	n+304 (+304), K+304 (+304), Carbamidomethyl (+57)	28.17	25.22
961	seq=translation; coord=7:168785597..168786938:1; parent_transcript=GRMZM2G129761_T01; parent_gene=GRMZM2G129761	GRMZM2G129761_P01	TRUE	TRUE	vTFPILADPAR	94%	n+304 (+304)	30.38	26.41
962	seq=translation; coord=3:186793464..186794596:-1; parent_transcript=GRMZM2G092474_T01; parent_gene=GRMZM2G092474	GRMZM2G092474_P01	TRUE	TRUE	aDINAQcPAALk	95%	n+304 (+304), Carbamidomethyl (+57), K+304 (+304)	41.35	25.35
963	seq=translation; coord=3:186793464..186794596:-1; parent_transcript=GRMZM2G092474_T01; parent_gene=GRMZM2G092474	GRMZM2G092474_P01	TRUE	TRUE	aDINAQcPAALkVPGGcASAcEk	94%	n+304 (+304), Carbamidomethyl (+57), K+304 (+304), Carbamidomethyl (+57), Carbamidomethyl (+57), K+304 (+304)	26.17	25.00

964	seq=translation; coord=3:186793464..186794596:-1; parent_transcript=GRMZM2G092474_T01; parent_gene=GRMZM2G092474	GRMZM2G092474_P01	TRUE	TRUE	gTcQTGDcGGALAcTVSGR	95%	n+304 (+304), Carbamidomethyl (+57), Carbamidomethyl (+57), Carbamidomethyl (+57)	78.55	25.00
965	seq=translation; coord=3:186793464..186794596:-1; parent_transcript=GRMZM2G092474_T01; parent_gene=GRMZM2G092474	GRMZM2G092474_P01	TRUE	TRUE	lcPDAYSYAk	95%	n+304 (+304), Carbamidomethyl (+57), K+304 (+304)	38.60	25.00
966	seq=translation; coord=3:186793464..186794596:-1; parent_transcript=GRMZM2G092474_T01; parent_gene=GRMZM2G092474	GRMZM2G092474_P01	TRUE	TRUE	lGGDTYccR	95%	n+304 (+304), Carbamidomethyl (+57), Carbamidomethyl (+57)	35.59	25.00
967	seq=translation; coord=3:186793464..186794596:-1; parent_transcript=GRMZM2G092474_T01; parent_gene=GRMZM2G092474	GRMZM2G092474_P01	TRUE	TRUE	nTLWPAALPGGGAR	95%	n+304 (+304)	37.75	25.00
968	seq=translation; coord=3:186793464..186794596:-1; parent_transcript=GRMZM2G092474_T01; parent_gene=GRMZM2G092474	GRMZM2G092474_P01	TRUE	TRUE	tGcSFDGSGR	95%	n+304 (+304), Carbamidomethyl (+57)	40.39	25.00
969	seq=translation; coord=3:186793464..186794596:-1; parent_transcript=GRMZM2G092474_T01; parent_gene=GRMZM2G092474	GRMZM2G092474_P01	TRUE	TRUE	tPATLAEYTLNR	95%	n+304 (+304)	40.02	25.00
970	seq=translation; coord=3:186793464..186794596:-1; parent_transcript=GRMZM2G092474_T01; parent_gene=GRMZM2G092474	GRMZM2G092474_P01	TRUE	TRUE	tPATLAEyTLNR	95%	n+304 (+304), iTRAQ8plex (+304)	31.98	26.75
971	seq=translation; coord=3:186793464..186794596:-1; parent_transcript=GRMZM2G092474_T01; parent_gene=GRMZM2G092474	GRMZM2G092474_P01	TRUE	TRUE	tWTVQVPAGTAHAR	95%	n+304 (+304)	33.74	25.43
972	seq=translation; coord=3:186793464..186794596:-1; parent_transcript=GRMZM2G092474_T01; parent_gene=GRMZM2G092474	GRMZM2G092474_P01	TRUE	TRUE	vPGGcASAcEk	95%	n+304 (+304), Carbamidomethyl (+57), Carbamidomethyl (+57), K+304 (+304)	43.46	25.00
973	seq=translation; coord=4:105179813..105180991:-1; parent_transcript=GRMZM2G162388_T01; parent_gene=GRMZM2G162388	GRMZM2G162388_P01	TRUE	TRUE	hVVFGQVVEGMEVVk	95%	n+304 (+304), K+304 (+304)	104.78	25.02
974	seq=translation; coord=4:105179813..105180991:-1; parent_transcript=GRMZM2G162388_T01; parent_gene=GRMZM2G162388	GRMZM2G162388_P01	TRUE	TRUE	hVVFGQVVEGmEVVk	95%	n+304 (+304), Oxidation (+16), K+304 (+304)	50.72	25.79
975	seq=translation; coord=4:105179813..105180991:-1; parent_transcript=GRMZM2G162388_T01; parent_gene=GRMZM2G162388	GRMZM2G162388_P01	TRUE	TRUE	vADcGQL	95%	n+304 (+304), Carbamidomethyl (+57)	35.97	25.00
976	seq=translation; coord=4:105179813..105180991:-1; parent_transcript=GRMZM2G162388_T01; parent_gene=GRMZM2G162388	GRMZM2G162388_P01	TRUE	TRUE	vIPQFMcQGGDFTR	95%	n+304 (+304), Carbamidomethyl (+57)	52.32	25.00
977	seq=translation; coord=4:105179813..105180991:-1; parent_transcript=GRMZM2G162388_T01; parent_gene=GRMZM2G162388	GRMZM2G162388_P01	TRUE	TRUE	vIPQFmcQGGDFTR	95%	n+304 (+304), Oxidation (+16), Carbamidomethyl (+57)	45.71	25.00
978	seq=translation; coord=10:60080812..60087313:-1; parent_transcript=GRMZM2G181192_T01; parent_gene=GRMZM2G181192	GRMZM2G181192_P01,GRMZM2G181192_P02, GRMZM2G181192_P03	TRUE	TRUE	aAEAVVDWHk	95%	n+304 (+304), K+304 (+304)	41.15	25.77
979	seq=translation; coord=10:60080812..60087313:-1; parent_transcript=GRMZM2G181192_T01; parent_gene=GRMZM2G181192	GRMZM2G181192_P01,GRMZM2G181192_P02, GRMZM2G181192_P03	TRUE	TRUE	aDTPEPLcQVMLR	95%	n+304 (+304), Carbamidomethyl (+57)	50.39	25.00
980	seq=translation; coord=10:60080812..60087313:-1; parent_transcript=GRMZM2G181192_T01; parent_gene=GRMZM2G181192	GRMZM2G181192_P01,GRMZM2G181192_P02, GRMZM2G181192_P03	TRUE	TRUE	gGSTVIAFAQDPDGyMFELIQR	95%	n+304 (+304), iTRAQ8plex (+304)	30.61	25.04

981	seq=translation; coord=10:60080812..60087313:-1; parent_transcript=GRMZM2G181192_T01; parent_gene=GRMZM2G181192	GRMZM2G181192_P01,GRMZM2G181192_P02, GRMZM2G181192_P03	TRUE	TRUE	gNAYAQVAIGTNDVYk	95%	n+304 (+304), K+304 (+304)	49.32	25.37
982	seq=translation; coord=10:60080812..60087313:-1; parent_transcript=GRMZM2G181192_T01; parent_gene=GRMZM2G181192	GRMZM2G181192_P01,GRMZM2G181192_P02, GRMZM2G181192_P03	TRUE	TRUE	iASFVDPDGWk	95%	n+304 (+304), K+304 (+304)	29.50	25.00
983	seq=translation; coord=10:60080812..60087313:-1; parent_transcript=GRMZM2G181192_T01; parent_gene=GRMZM2G181192	GRMZM2G181192_P01,GRMZM2G181192_P02, GRMZM2G181192_P03	TRUE	TRUE	mLHAVYR	95%	n+304 (+304)	30.34	26.71
984	seq=translation; coord=10:60080812..60087313:-1; parent_transcript=GRMZM2G181192_T01; parent_gene=GRMZM2G181192	GRMZM2G181192_P01,GRMZM2G181192_P02, GRMZM2G181192_P03	TRUE	TRUE	qPGPLPGINTk	95%	n+304 (+304), K+304 (+304)	46.79	25.00
985	seq=translation; coord=10:60080812..60087313:-1; parent_transcript=GRMZM2G181192_T01; parent_gene=GRMZM2G181192	GRMZM2G181192_P01,GRMZM2G181192_P02, GRMZM2G181192_P03	TRUE	TRUE	qPGPLPGINTk	95%	Pyro-cmC (-17), n+304 (+304), K+304 (+304)	32.43	25.00
986	seq=translation; coord=10:60080812..60087313:-1; parent_transcript=GRMZM2G181192_T01; parent_gene=GRMZM2G181192	GRMZM2G181192_P01,GRMZM2G181192_P02, GRMZM2G181192_P03	TRUE	TRUE	sAEAVDLATk	95%	n+304 (+304), K+304 (+304)	54.68	25.55
987	seq=translation; coord=10:60080812..60087313:-1; parent_transcript=GRMZM2G181192_T01; parent_gene=GRMZM2G181192	GRMZM2G181192_P01,GRMZM2G181192_P02, GRMZM2G181192_P03	TRUE	TRUE	vVLVDNTDFLk	95%	n+304 (+304), K+304 (+304)	51.74	25.73
988	seq=translation; coord=10:60080812..60087313:-1; parent_transcript=GRMZM2G181192_T01; parent_gene=GRMZM2G181192	GRMZM2G181192_P01,GRMZM2G181192_P02, GRMZM2G181192_P03	TRUE	TRUE	yDIGTGFGHFAIANDDVYk	95%	n+304 (+304), K+304 (+304)	40.51	25.00
989	seq=translation; coord=10:60080812..60087313:-1; parent_transcript=GRMZM2G181192_T01; parent_gene=GRMZM2G181192	GRMZM2G181192_P01,GRMZM2G181192_P02, GRMZM2G181192_P03	TRUE	TRUE	yTIAMLGYADEDk	95%	n+304 (+304), K+304 (+304)	31.69	25.00
990	seq=translation; coord=9:129274385..129278107:-1; parent_transcript=GRMZM2G058522_T01; parent_gene=GRMZM2G058522	GRMZM2G058522_P01,GRMZM2G058522_P04, GRMZM2G058522_P05,GRMZM2G058522_P06, GRMZM2G058522_P08,GRMZM2G058522_P09	TRUE	TRUE	aVAVLGSSDGVk	95%	n+304 (+304), K+304 (+304)	61.57	26.31
991	seq=translation; coord=9:129274385..129278107:-1; parent_transcript=GRMZM2G058522_T01; parent_gene=GRMZM2G058522	GRMZM2G058522_P01,GRMZM2G058522_P04, GRMZM2G058522_P05,GRMZM2G058522_P06, GRMZM2G058522_P08,GRMZM2G058522_P09	TRUE	TRUE	eHGAPEDENR	95%	n+304 (+304)	43.28	25.00
992	seq=translation; coord=9:129274385..129278107:-1; parent_transcript=GRMZM2G058522_T01; parent_gene=GRMZM2G058522	GRMZM2G058522_P01,GRMZM2G058522_P04, GRMZM2G058522_P05,GRMZM2G058522_P06, GRMZM2G058522_P08,GRMZM2G058522_P09	TRUE	TRUE	hAGDLGNVTAGADGVANINVTDSQIPLTGPNSIIGR	95%	n+304 (+304)	54.53	25.00
993	seq=translation; coord=8:168955391..168958754:-1; parent_transcript=GRMZM2G310431_T01; parent_gene=GRMZM2G310431	GRMZM2G310431_P01	TRUE	TRUE	kIEDAVDGAISWLDSNQLAEVEEFEDk	94%	K+304 (+304), n+304 (+304), K+304 (+304)	25.51	25.00
994	seq=translation; coord=8:168955391..168958754:-1; parent_transcript=GRMZM2G310431_T01; parent_gene=GRMZM2G310431	GRMZM2G310431_P01	TRUE	TRUE	mYQGEGAGMGAAAGMDEDAPSGGSGAGPk	95%	n+304 (+304), K+304 (+304)	92.74	25.00
995	seq=translation; coord=8:168955391..168958754:-1; parent_transcript=GRMZM2G310431_T01; parent_gene=GRMZM2G310431	GRMZM2G310431_P01	TRUE	TRUE	myQGEGAGMGAAAGMDEDAPSGGSGAGPk	95%	n+304 (+304), iTRAQ8plex (+304), K+304 (+304)	51.62	25.00
996	seq=translation; coord=8:168955391..168958754:-1; parent_transcript=GRMZM2G310431_T01; parent_gene=GRMZM2G310431	GRMZM2G310431_P01	TRUE	TRUE	sEGPAIGIDLGTTYScVGVWQHDR	95%	n+304 (+304), Carbamidomethyl (+57)	102.07	25.00
997	seq=translation; coord=8:168955391..168958754:-1; parent_transcript=GRMZM2G310431_T01; parent_gene=GRMZM2G310431	GRMZM2G310431_P01	TRUE	TRUE	tTPSYVGFTDTER	95%	n+304 (+304)	58.06	25.00

998	seq=translation; coord=6:115191842..115198242:-1; parent_transcript=GRMZM2G059151_T01; parent_gene=GRMZM2G059151	GRMZM2G059151_P01	TRUE	TRUE	eLFEFLPNTIQEQLMLER	95%	n+304 (+304)	41.58	25.40
999	seq=translation; coord=6:115191842..115198242:-1; parent_transcript=GRMZM2G059151_T01; parent_gene=GRMZM2G059151	GRMZM2G059151_P01	TRUE	TRUE	eVPTSFGFDTAck	95%	n+304 (+304), Carbamidomethyl (+57), K+304 (+304)	38.91	25.00
1000	seq=translation; coord=6:115191842..115198242:-1; parent_transcript=GRMZM2G059151_T01; parent_gene=GRMZM2G059151	GRMZM2G059151_P01	TRUE	TRUE	gGPAGIMk	86%	n+304 (+304), K+304 (+304)	26.43	25.94
1001	seq=translation; coord=6:115191842..115198242:-1; parent_transcript=GRMZM2G059151_T01; parent_gene=GRMZM2G059151	GRMZM2G059151_P01	TRUE	TRUE	iYSEMIGNVMTDAR	95%	n+304 (+304)	64.40	25.00
1002	seq=translation; coord=6:115191842..115198242:-1; parent_transcript=GRMZM2G059151_T01; parent_gene=GRMZM2G059151	GRMZM2G059151_P01	TRUE	TRUE	lLHALPLPSVLR	95%	n+304 (+304)	32.71	25.00
1003	seq=translation; coord=6:115191842..115198242:-1; parent_transcript=GRMZM2G059151_T01; parent_gene=GRMZM2G059151	GRMZM2G059151_P01	TRUE	TRUE	nQGGFDMIcSGR	95%	n+304 (+304), Carbamidomethyl (+57)	31.61	25.00
1004	seq=translation; coord=6:115191842..115198242:-1; parent_transcript=GRMZM2G059151_T01; parent_gene=GRMZM2G059151	GRMZM2G059151_P01	TRUE	TRUE	yVELTSDFVYPYR	95%	n+304 (+304)	48.99	25.00
1005	seq=translation; coord=6:115191842..115198242:-1; parent_transcript=GRMZM2G059151_T01; parent_gene=GRMZM2G059151	GRMZM2G059151_P01	TRUE	TRUE	yVELTSDFVyPYR	92%	n+304 (+304), iTRAQ8plex (+304)	27.19	26.03
1006	seq=translation; coord=5:179010381..179015571:1; parent_transcript=GRMZM2G013324_T01; parent_gene=GRMZM2G013324	GRMZM2G013324_P01	TRUE	TRUE	aPVIVAk	94%	n+304 (+304), K+304 (+304)	30.40	25.00
1007	seq=translation; coord=5:179010381..179015571:1; parent_transcript=GRMZM2G013324_T01; parent_gene=GRMZM2G013324	GRMZM2G013324_P01	TRUE	TRUE	dLSGIMITVEHAAFMAEk	95%	n+304 (+304), K+304 (+304)	37.28	25.68
1008	seq=translation; coord=5:179010381..179015571:1; parent_transcript=GRMZM2G013324_T01; parent_gene=GRMZM2G013324	GRMZM2G013324_P01	TRUE	TRUE	gGFSGLFPDSSDFAYR	95%	n+304 (+304)	47.16	25.00
1009	seq=translation; coord=5:179010381..179015571:1; parent_transcript=GRMZM2G013324_T01; parent_gene=GRMZM2G013324	GRMZM2G013324_P01	TRUE	TRUE	iLGTISSPDTVMWcDVR	95%	n+304 (+304), Carbamidomethyl (+57)	34.97	25.00
1010	seq=translation; coord=5:179010381..179015571:1; parent_transcript=GRMZM2G013324_T01; parent_gene=GRMZM2G013324	GRMZM2G013324_P01	TRUE	TRUE	lGFGVVDAVVk	95%	n+304 (+304), K+304 (+304)	40.34	25.00
1011	seq=translation; coord=5:179010381..179015571:1; parent_transcript=GRMZM2G013324_T01; parent_gene=GRMZM2G013324	GRMZM2G013324_P01	TRUE	TRUE	qFITDYISSPEVNFLTSISGR	95%	n+304 (+304)	33.62	25.08
1012	seq=translation; coord=5:179010381..179015571:1; parent_transcript=GRMZM2G013324_T01; parent_gene=GRMZM2G013324	GRMZM2G013324_P01	TRUE	TRUE	sSFTSQVTTINDLk	93%	n+304 (+304), K+304 (+304)	28.43	25.73
1013	seq=translation; coord=5:179010381..179015571:1; parent_transcript=GRMZM2G013324_T01; parent_gene=GRMZM2G013324	GRMZM2G013324_P01	TRUE	TRUE	ySLVYMIEEGVR	95%	n+304 (+304)	39.27	25.00
1014	seq=translation; coord=4:240742463..240747734:-1; parent_transcript=GRMZM2G015989_T01; parent_gene=GRMZM2G015989	GRMZM2G015989_P01,GRMZM2G015989_P02	TRUE	TRUE	dLITILEDAIR	95%	n+304 (+304)	37.47	25.80
1015	seq=translation; coord=4:240742463..240747734:-1; parent_transcript=GRMZM2G015989_T01; parent_gene=GRMZM2G015989	GRMZM2G015989_P01,GRMZM2G015989_P02	TRUE	TRUE	dSTTIVGDGTTQEEVNk	95%	n+304 (+304), K+304 (+304)	57.00	25.00

1016	seq=translation; coord=4:240742463..240747734:-1; parent_transcript=GRMZM2G015989_T01; parent_gene=GRMZM2G015989	GRMZM2G015989_P01,GRMZM2G015989_P02	TRUE	TRUE	eEVGLSLDk	95%	n+304 (+304), K+304 (+304)	37.64	26.89
1017	seq=translation; coord=4:240742463..240747734:-1; parent_transcript=GRMZM2G015989_T01; parent_gene=GRMZM2G015989	GRMZM2G015989_P01,GRMZM2G015989_P02	TRUE	TRUE	eLHFNk	95%	n+304 (+304), K+304 (+304)	28.48	25.00
1018	seq=translation; coord=4:240742463..240747734:-1; parent_transcript=GRMZM2G015989_T01; parent_gene=GRMZM2G015989	GRMZM2G015989_P01,GRMZM2G015989_P02	TRUE	TRUE	kSQYLDDIATLTGGTVIR	95%	K+304 (+304), n+304 (+304)	27.33	25.43
1019	seq=translation; coord=4:240742463..240747734:-1; parent_transcript=GRMZM2G015989_T01; parent_gene=GRMZM2G015989	GRMZM2G015989_P01,GRMZM2G015989_P02	TRUE	TRUE	nQIEATEQEYER	93%	n+304 (+304)	29.58	25.00
1020	seq=translation; coord=4:240742463..240747734:-1; parent_transcript=GRMZM2G015989_T01; parent_gene=GRMZM2G015989	GRMZM2G015989_P01,GRMZM2G015989_P02	TRUE	TRUE	sAENNLYSVEGMQFDR	95%	n+304 (+304)	55.10	25.00
1021	seq=translation; coord=4:240742463..240747734:-1; parent_transcript=GRMZM2G015989_T01; parent_gene=GRMZM2G015989	GRMZM2G015989_P01,GRMZM2G015989_P02	TRUE	TRUE	sGyPILIVAEDIEQEALATLVVNR	95%	n+304 (+304), iTRAQ8plex (+304)	46.42	25.00
1022	seq=translation; coord=4:240742463..240747734:-1; parent_transcript=GRMZM2G015989_T01; parent_gene=GRMZM2G015989	GRMZM2G015989_P01,GRMZM2G015989_P02	TRUE	TRUE	tFITSDAVVVDAk	95%	n+304 (+304), K+304 (+304)	40.13	26.37
1023	seq=translation; coord=4:240742463..240747734:-1; parent_transcript=GRMZM2G015989_T01; parent_gene=GRMZM2G015989	GRMZM2G015989_P01,GRMZM2G015989_P02	TRUE	TRUE	tNDLAGDGTTTSVVLAQGMITEGVk	95%	n+304 (+304), K+304 (+304)	47.85	25.69
1024	seq=translation; coord=4:240742463..240747734:-1; parent_transcript=GRMZM2G015989_T01; parent_gene=GRMZM2G015989	GRMZM2G015989_P01,GRMZM2G015989_P02	TRUE	TRUE	vDAIIETLENDEQk	95%	n+304 (+304), K+304 (+304)	44.89	26.11
1025	seq=translation; coord=4:240742463..240747734:-1; parent_transcript=GRMZM2G015989_T01; parent_gene=GRMZM2G015989	GRMZM2G015989_P01,GRMZM2G015989_P02	TRUE	TRUE	yGYNAATGEYEDLMAAGIIDPTk	95%	n+304 (+304), K+304 (+304)	51.13	25.00
1026	seq=translation; coord=7:173553684..173555507:-1; parent_transcript=GRMZM2G469380_T02; parent_gene=GRMZM2G469380	GRMZM2G469380_P02	TRUE	TRUE	aGGEDYSLTVR	95%	n+304 (+304)	37.34	25.00
1027	seq=translation; coord=7:173553684..173555507:-1; parent_transcript=GRMZM2G469380_T02; parent_gene=GRMZM2G469380	GRMZM2G469380_P02	TRUE	TRUE	aHGGVHDGTEIVLWk	95%	n+304 (+304), K+304 (+304)	57.86	25.19
1028	seq=translation; coord=7:173553684..173555507:-1; parent_transcript=GRMZM2G469380_T02; parent_gene=GRMZM2G469380	GRMZM2G469380_P02	TRUE	TRUE	aNENYcLAVR	95%	n+304 (+304), Carbamidomethyl (+57)	37.89	25.00
1029	seq=translation; coord=7:173553684..173555507:-1; parent_transcript=GRMZM2G469380_T02; parent_gene=GRMZM2G469380	GRMZM2G469380_P02	TRUE	TRUE	dEYQHWVk	95%	n+304 (+304), K+304 (+304)	34.35	25.00
1030	seq=translation; coord=7:173553684..173555507:-1; parent_transcript=GRMZM2G469380_T02; parent_gene=GRMZM2G469380	GRMZM2G469380_P02	TRUE	TRUE	dGAVVLAPVNPk	95%	n+304 (+304), K+304 (+304)	30.71	25.00
1031	seq=translation; coord=7:173553684..173555507:-1; parent_transcript=GRMZM2G469380_T02; parent_gene=GRMZM2G469380	GRMZM2G469380_P02	TRUE	TRUE	gENQSWk	89%	n+304 (+304), K+304 (+304)	26.52	25.00
1032	seq=translation; coord=7:173553684..173555507:-1; parent_transcript=GRMZM2G469380_T02; parent_gene=GRMZM2G469380	GRMZM2G469380_P02	TRUE	TRUE	lAPFSPDQEDASVLWTESk	95%	n+304 (+304), K+304 (+304)	35.56	25.00
1033	seq=translation; coord=7:173553684..173555507:-1; parent_transcript=GRMZM2G469380_T02; parent_gene=GRMZM2G469380	GRMZM2G469380_P02	TRUE	TRUE	lVPYNPDYQDESVLWTESR	95%	n+304 (+304)	37.31	25.00

1034	seq=translation; coord=7:173553684..173555507:-1; parent_transcript=GRMZM2G469380_T02; parent_gene=GRMZM2G469380	GRMZM2G469380_P02	TRUE	TRUE	vkDEEGMPAFALVNk	95%	n+304 (+304), K+304 (+304), K+304 (+304)	31.02	25.28
1035	seq=translation; coord=7:173553684..173555507:-1; parent_transcript=GRMZM2G469380_T02; parent_gene=GRMZM2G469380	GRMZM2G469380_P02	TRUE	TRUE	vRDEEGYPAFALVNk	95%	n+304 (+304), K+304 (+304)	43.35	26.19
1036	seq=translation; coord=4:58954361..58960521:-1; parent_transcript=GRMZM2G068506_T01; parent_gene=GRMZM2G068506	GRMZM2G068506_P01	TRUE	TRUE	aMMVDTTILGLDDVR	87%	n+304 (+304)	26.19	25.65
1037	seq=translation; coord=4:58954361..58960521:-1; parent_transcript=GRMZM2G068506_T01; parent_gene=GRMZM2G068506	GRMZM2G068506_P01	TRUE	TRUE	aSPPPWNATAAEQPIPk	95%	n+304 (+304), K+304 (+304)	36.75	26.01
1038	seq=translation; coord=4:58954361..58960521:-1; parent_transcript=GRMZM2G068506_T01; parent_gene=GRMZM2G068506	GRMZM2G068506_P01	TRUE	TRUE	aYGSNIGGYk	95%	n+304 (+304), K+304 (+304)	36.80	25.47
1039	seq=translation; coord=4:58954361..58960521:-1; parent_transcript=GRMZM2G068506_T01; parent_gene=GRMZM2G068506	GRMZM2G068506_P01	TRUE	TRUE	eQFPEANDFGSEVIPGATSIGk	95%	n+304 (+304), K+304 (+304)	46.49	25.00
1040	seq=translation; coord=4:58954361..58960521:-1; parent_transcript=GRMZM2G068506_T01; parent_gene=GRMZM2G068506	GRMZM2G068506_P01	TRUE	TRUE	eTNADITVAALPMDEk	95%	n+304 (+304), K+304 (+304)	57.93	25.38
1041	seq=translation; coord=4:58954361..58960521:-1; parent_transcript=GRMZM2G068506_T01; parent_gene=GRMZM2G068506	GRMZM2G068506_P01	TRUE	TRUE	fAPIYTQPR	89%	n+304 (+304)	26.15	25.28
1042	seq=translation; coord=4:58954361..58960521:-1; parent_transcript=GRMZM2G068506_T01; parent_gene=GRMZM2G068506	GRMZM2G068506_P01	TRUE	TRUE	gGIPIGIGk	91%	n+304 (+304), K+304 (+304)	25.86	25.00
1043	seq=translation; coord=4:58954361..58960521:-1; parent_transcript=GRMZM2G068506_T01; parent_gene=GRMZM2G068506	GRMZM2G068506_P01	TRUE	TRUE	iNHSVVGLR	93%	n+304 (+304)	27.40	26.14
1044	seq=translation; coord=4:58954361..58960521:-1; parent_transcript=GRMZM2G068506_T01; parent_gene=GRMZM2G068506	GRMZM2G068506_P01	TRUE	TRUE	nEGFVEVLAAQQSPDNPNWFQGTADAVR	95%	n+304 (+304)	52.91	25.00
1045	seq=translation; coord=6:83777893..83783487:1; parent_transcript=GRMZM2G141931_T01; parent_gene=GRMZM2G141931	GRMZM2G141931_P01	TRUE	TRUE	aLESENVDSVk	95%	n+304 (+304), K+304 (+304)	50.54	25.48
1046	seq=translation; coord=5:155951379..155960766:1; parent_transcript=GRMZM2G027875_T01; parent_gene=GRMZM2G027875	GRMZM2G027875_P01	TRUE	TRUE	aAYVALMQTVSk	95%	n+304 (+304), K+304 (+304)	31.00	25.43
1047	seq=translation; coord=5:155951379..155960766:1; parent_transcript=GRMZM2G027875_T01; parent_gene=GRMZM2G027875	GRMZM2G027875_P01	TRUE	TRUE	aEADLGNVLk	95%	n+304 (+304), K+304 (+304)	51.36	25.68
1048	seq=translation; coord=5:155951379..155960766:1; parent_transcript=GRMZM2G027875_T01; parent_gene=GRMZM2G027875	GRMZM2G027875_P01	TRUE	TRUE	eTALLFDEMHSAAANk	95%	n+304 (+304), K+304 (+304)	28.01	25.21
1049	seq=translation; coord=5:155951379..155960766:1; parent_transcript=GRMZM2G027875_T01; parent_gene=GRMZM2G027875	GRMZM2G027875_P01	TRUE	TRUE	eVLNFILSPEVR	95%	n+304 (+304)	49.16	25.00
1050	seq=translation; coord=5:155951379..155960766:1; parent_transcript=GRMZM2G027875_T01; parent_gene=GRMZM2G027875	GRMZM2G027875_P01	TRUE	TRUE	fLIDFLEPFALk	95%	n+304 (+304), K+304 (+304)	30.99	25.00
1051	seq=translation; coord=5:155951379..155960766:1; parent_transcript=GRMZM2G027875_T01; parent_gene=GRMZM2G027875	GRMZM2G027875_P01	TRUE	TRUE	gTLLTALAELGHEATINEAVR	95%	n+304 (+304)	69.11	25.00

1052	seq=translation; coord=5:155951379..155960766:1; parent_transcript=GRMZM2G027875_T01; parent_gene=GRMZM2G027875	GRMZM2G027875_P01	TRUE	TRUE	iNAQWVk	94%	n+304 (+304), K+304 (+304)	29.13	25.00
1053	seq=translation; coord=5:155951379..155960766:1; parent_transcript=GRMZM2G027875_T01; parent_gene=GRMZM2G027875	GRMZM2G027875_P01	TRUE	TRUE	iTLEVPSETIALSNMPVIEEk	95%	n+304 (+304), K+304 (+304)	31.35	25.00
1054	seq=translation; coord=5:155951379..155960766:1; parent_transcript=GRMZM2G027875_T01; parent_gene=GRMZM2G027875	GRMZM2G027875_P01	TRUE	TRUE	kFLIDFLEPFALk	95%	K+304 (+304), n+304 (+304), K+304 (+304)	29.15	25.00
1055	seq=translation; coord=5:155951379..155960766:1; parent_transcript=GRMZM2G027875_T01; parent_gene=GRMZM2G027875	GRMZM2G027875_P01	TRUE	TRUE	lDALAGSHPIEVDINHVDEIDEIFDAISYR	95%	n+304 (+304)	26.51	25.00
1056	seq=translation; coord=5:155951379..155960766:1; parent_transcript=GRMZM2G027875_T01; parent_gene=GRMZM2G027875	GRMZM2G027875_P01	TRUE	TRUE	lNVNQTSFYR	93%	n+304 (+304)	30.05	25.00
1057	seq=translation; coord=5:155951379..155960766:1; parent_transcript=GRMZM2G027875_T01; parent_gene=GRMZM2G027875	GRMZM2G027875_P01	TRUE	TRUE	mDMVAIPDFAAGAMENyGLVTYR	95%	n+304 (+304), iTRAQ8plex (+304)	31.89	25.00
1058	seq=translation; coord=5:155951379..155960766:1; parent_transcript=GRMZM2G027875_T01; parent_gene=GRMZM2G027875	GRMZM2G027875_P01	TRUE	TRUE	mLQNYLGAEVFQNSLAAYIk	95%	n+304 (+304), K+304 (+304)	46.42	25.07
1059	seq=translation; coord=5:155951379..155960766:1; parent_transcript=GRMZM2G027875_T01; parent_gene=GRMZM2G027875	GRMZM2G027875_P01	TRUE	TRUE	nMAVTQFEPADAR	95%	n+304 (+304)	42.04	25.00
1060	seq=translation; coord=5:155951379..155960766:1; parent_transcript=GRMZM2G027875_T01; parent_gene=GRMZM2G027875	GRMZM2G027875_P01	TRUE	TRUE	nQDAIFLLR	93%	n+304 (+304)	29.79	25.00
1061	seq=translation; coord=5:155951379..155960766:1; parent_transcript=GRMZM2G027875_T01; parent_gene=GRMZM2G027875	GRMZM2G027875_P01	TRUE	TRUE	rFNVFLEDR	95%	n+304 (+304)	42.22	25.45
1062	seq=translation; coord=5:155951379..155960766:1; parent_transcript=GRMZM2G027875_T01; parent_gene=GRMZM2G027875	GRMZM2G027875_P01	TRUE	TRUE	tEDLWAALEEGSGEPVR	95%	n+304 (+304)	32.86	25.00
1063	seq=translation; coord=5:155951379..155960766:1; parent_transcript=GRMZM2G027875_T01; parent_gene=GRMZM2G027875	GRMZM2G027875_P01	TRUE	TRUE	tLMHSWTk	95%	n+304 (+304), K+304 (+304)	30.97	26.12
1064	seq=translation; coord=5:155951379..155960766:1; parent_transcript=GRMZM2G027875_T01; parent_gene=GRMZM2G027875	GRMZM2G027875_P01	TRUE	TRUE	vSYDEELAAR	92%	n+304 (+304)	28.57	25.00
1065	seq=translation; coord=4:160954463..160955623:1; parent_transcript=GRMZM2G342515_T01; parent_gene=GRMZM2G342515	GRMZM2G342515_P01	TRUE	TRUE	aMSIMNSFINDIFEk	95%	n+304 (+304), K+304 (+304)	43.14	25.61
1066	seq=translation; coord=4:160954463..160955623:1; parent_transcript=GRMZM2G342515_T01; parent_gene=GRMZM2G342515	GRMZM2G342515_P01	TRUE	TRUE	amSIMNSFINDIFEk	95%	n+304 (+304), Oxidation (+16), K+304 (+304)	46.44	25.01
1067	seq=translation; coord=4:160954463..160955623:1; parent_transcript=GRMZM2G342515_T01; parent_gene=GRMZM2G342515	GRMZM2G342515_P01	TRUE	TRUE	hAVSEGTk	95%	n+304 (+304), K+304 (+304)	48.59	25.00
1068	seq=translation; coord=4:160954463..160955623:1; parent_transcript=GRMZM2G342515_T01; parent_gene=GRMZM2G342515	GRMZM2G342515_P01	TRUE	TRUE	lVLPGELAk	95%	n+304 (+304), K+304 (+304)	35.80	25.00
1069	seq=translation; coord=4:160954463..160955623:1; parent_transcript=GRMZM2G342515_T01; parent_gene=GRMZM2G342515	GRMZM2G342515_P01	TRUE	TRUE	qVHPDIGISSk	95%	Pyro-cmC (-17), n+304 (+304), K+304 (+304)	34.51	25.00

1070	seq=translation; coord=4:160954463..160955623:1; parent_transcript=GRMZM2G342515_T01; parent_gene=GRMZM2G342515	GRMZM2G342515_P01	TRUE	TRUE	vAEEEPSEk	93%	n+304 (+304), K+304 (+304)	30.31	25.50
1071	seq=translation; coord=3:202432697..202435947:1; parent_transcript=GRMZM2G055276_T01; parent_gene=GRMZM2G055276	GRMZM2G055276_P01	TRUE	TRUE	nVEFADDSERPPR	95%	n+304 (+304)	30.31	25.00
1072	seq=translation; coord=3:202432697..202435947:1; parent_transcript=GRMZM2G055276_T01; parent_gene=GRMZM2G055276	GRMZM2G055276_P01	TRUE	TRUE	qGEQNDAPAADENk	95%	n+304 (+304), K+304 (+304)	39.03	25.00
1073	seq=translation; coord=3:202432697..202435947:1; parent_transcript=GRMZM2G055276_T01; parent_gene=GRMZM2G055276	GRMZM2G055276_P01	TRUE	TRUE	qGEQNDAPAADENkDNk	95%	Pyro-cmC (-17), n+304 (+304), K+304 (+304), K+304 (+304)	33.44	25.00
1074	seq=translation; coord=3:213623450..213627174:-1; parent_transcript=GRMZM2G004382_T01; parent_gene=GRMZM2G004382	GRMZM2G004382_P01	TRUE	TRUE	dLFPLLPEAFk	95%	n+304 (+304), K+304 (+304)	32.11	25.00
1075	seq=translation; coord=3:213623450..213627174:-1; parent_transcript=GRMZM2G004382_T01; parent_gene=GRMZM2G004382	GRMZM2G004382_P01	TRUE	TRUE	eGLPAFPMGk	95%	n+304 (+304), K+304 (+304)	34.79	25.61
1076	seq=translation; coord=3:213623450..213627174:-1; parent_transcript=GRMZM2G004382_T01; parent_gene=GRMZM2G004382	GRMZM2G004382_P01	TRUE	TRUE	fDYILTQQAFVTVDk	95%	n+304 (+304), K+304 (+304)	33.45	25.40
1077	seq=translation; coord=3:213623450..213627174:-1; parent_transcript=GRMZM2G004382_T01; parent_gene=GRMZM2G004382	GRMZM2G004382_P01	TRUE	TRUE	gHSYSEIINESLIESVDSLNPFMHAR	95%	n+304 (+304)	56.17	25.00
1078	seq=translation; coord=3:213623450..213627174:-1; parent_transcript=GRMZM2G004382_T01; parent_gene=GRMZM2G004382	GRMZM2G004382_P01	TRUE	TRUE	gILLGAVHGMVEALFR	95%	n+304 (+304)	69.03	25.00
1079	seq=translation; coord=3:213623450..213627174:-1; parent_transcript=GRMZM2G004382_T01; parent_gene=GRMZM2G004382	GRMZM2G004382_P01	TRUE	TRUE	gILLGAVHGmVEALFR	95%	n+304 (+304), Oxidation (+16)	33.23	25.40
1080	seq=translation; coord=3:213623450..213627174:-1; parent_transcript=GRMZM2G004382_T01; parent_gene=GRMZM2G004382	GRMZM2G004382_P01	TRUE	TRUE	gMLEVYNSLTEEGk	95%	n+304 (+304), K+304 (+304)	50.90	26.23
1081	seq=translation; coord=3:213623450..213627174:-1; parent_transcript=GRMZM2G004382_T01; parent_gene=GRMZM2G004382	GRMZM2G004382_P01	TRUE	TRUE	gVAFMVDNcSTTAR	95%	n+304 (+304), Carbamidomethyl (+57)	59.41	25.00
1082	seq=translation; coord=3:213623450..213627174:-1; parent_transcript=GRMZM2G004382_T01; parent_gene=GRMZM2G004382	GRMZM2G004382_P01	TRUE	TRUE	nTVESITGIISk	95%	n+304 (+304), K+304 (+304)	34.25	25.00
1083	seq=translation; coord=3:213623450..213627174:-1; parent_transcript=GRMZM2G004382_T01; parent_gene=GRMZM2G004382	GRMZM2G004382_P01	TRUE	TRUE	qIGVIGWGSQGPAQAQNLR	95%	n+304 (+304)	67.06	25.49
1084	seq=translation; coord=4:220508652..220512101:1; parent_transcript=GRMZM2G027378_T01; parent_gene=GRMZM2G027378	GRMZM2G027378_P01,GRMZM2G071010_P01	TRUE	TRUE	dYTMENILTQLk	95%	n+304 (+304), K+304 (+304)	32.05	26.54
1085	seq=translation; coord=4:220508652..220512101:1; parent_transcript=GRMZM2G027378_T01; parent_gene=GRMZM2G027378	GRMZM2G027378_P01,GRMZM2G071010_P01	TRUE	TRUE	fGLLANWQR	85%	n+304 (+304)	25.05	25.00
1086	seq=translation; coord=4:220508652..220512101:1; parent_transcript=GRMZM2G027378_T01; parent_gene=GRMZM2G027378	GRMZM2G027378_P01,GRMZM2G071010_P01	TRUE	TRUE	tLGSSGAGSSVVVPR	95%	n+304 (+304)	90.40	25.13
1087	seq=translation; coord=5:29990567..29996686:1; parent_transcript=GRMZM2G167505_T01; parent_gene=GRMZM2G167505	GRMZM2G167505_P01,GRMZM2G167505_P02, GRMZM2G167505_P03	TRUE	TRUE	gFGFITFDSEDAVDR	95%	n+304 (+304)	35.57	25.00

1088	seq=translation; coord=5:29990567..29996686:1; parent_transcript=GRMZM2G167505_T01; parent_gene=GRMZM2G167505	GRMZM2G167505_P01,GRMZM2G167505_P02, GRMZM2G167505_P03	TRUE	TRUE	gFGFVVFADPAAVDR	95%	n+304 (+304)	59.53	25.00
1089	seq=translation; coord=5:29990567..29996686:1; parent_transcript=GRMZM2G167505_T01; parent_gene=GRMZM2G167505	GRMZM2G167505_P01,GRMZM2G167505_P02, GRMZM2G167505_P03	TRUE	TRUE	qYFETFGIVTDVVVMYDQNTQRPR	95%	n+304 (+304)	29.50	25.00
1090	seq=translation; coord=5:186517098..186522957:-1; parent_transcript=GRMZM2G540538_T01; parent_gene=GRMZM2G540538	GRMZM2G540538_P01	TRUE	TRUE	gWETENIEEIALk	95%	n+304 (+304), K+304 (+304)	32.76	25.43
1091	seq=translation; coord=5:186517098..186522957:-1; parent_transcript=GRMZM2G540538_T01; parent_gene=GRMZM2G540538	GRMZM2G540538_P01	TRUE	TRUE	hLPMYDELASk	95%	n+304 (+304), K+304 (+304)	41.67	25.53
1092	seq=translation; coord=5:186517098..186522957:-1; parent_transcript=GRMZM2G540538_T01; parent_gene=GRMZM2G540538	GRMZM2G540538_P01	TRUE	TRUE	sNVEYIAGGATQNSIR	95%	n+304 (+304)	34.09	25.00
1093	seq=translation; coord=5:186517098..186522957:-1; parent_transcript=GRMZM2G540538_T01; parent_gene=GRMZM2G540538	GRMZM2G540538_P01	TRUE	TRUE	vLPYADYIFGNETEAk	95%	n+304 (+304), K+304 (+304)	61.22	25.56
1094	seq=translation; coord=9:141825338..141830182:1; parent_transcript=GRMZM5G870932_T01; parent_gene=GRMZM5G870932	GRMZM5G870932_P01	TRUE	TRUE	aAYPIEYIPNAk	95%	n+304 (+304), K+304 (+304)	30.63	26.65
1095	seq=translation; coord=9:141825338..141830182:1; parent_transcript=GRMZM5G870932_T01; parent_gene=GRMZM5G870932	GRMZM5G870932_P01	TRUE	TRUE	dGAASTFAAALSEEER	95%	n+304 (+304)	70.96	25.00
1096	seq=translation; coord=9:141825338..141830182:1; parent_transcript=GRMZM5G870932_T01; parent_gene=GRMZM5G870932	GRMZM5G870932_P01	TRUE	TRUE	dGDVALFFGLSGTGk	95%	n+304 (+304), K+304 (+304)	55.31	25.38
1097	seq=translation; coord=9:141825338..141830182:1; parent_transcript=GRMZM5G870932_T01; parent_gene=GRMZM5G870932	GRMZM5G870932_P01	TRUE	TRUE	eMVILGTQYAGEMk	95%	n+304 (+304), K+304 (+304)	40.43	26.20
1098	seq=translation; coord=9:141825338..141830182:1; parent_transcript=GRMZM5G870932_T01; parent_gene=GRMZM5G870932	GRMZM5G870932_P01	TRUE	TRUE	fGAVLENVVFDEHTR	95%	n+304 (+304)	46.54	25.00
1099	seq=translation; coord=9:141825338..141830182:1; parent_transcript=GRMZM5G870932_T01; parent_gene=GRMZM5G870932	GRMZM5G870932_P01	TRUE	TRUE	fTHVLYNLSPAELYEQAIk	95%	n+304 (+304), K+304 (+304)	36.48	25.02
1100	seq=translation; coord=9:141825338..141830182:1; parent_transcript=GRMZM5G870932_T01; parent_gene=GRMZM5G870932	GRMZM5G870932_P01	TRUE	TRUE	gLFGVMHYLMPk	95%	n+304 (+304), K+304 (+304)	43.28	26.02
1101	seq=translation; coord=9:141825338..141830182:1; parent_transcript=GRMZM5G870932_T01; parent_gene=GRMZM5G870932	GRMZM5G870932_P01	TRUE	TRUE	gSFITSTGALATLSGAk	95%	n+304 (+304), K+304 (+304)	35.31	25.00
1102	seq=translation; coord=9:141825338..141830182:1; parent_transcript=GRMZM5G870932_T01; parent_gene=GRMZM5G870932	GRMZM5G870932_P01	TRUE	TRUE	gSPNIEMDEHTFLTNR	95%	n+304 (+304)	60.16	25.00
1103	seq=translation; coord=9:141825338..141830182:1; parent_transcript=GRMZM5G870932_T01; parent_gene=GRMZM5G870932	GRMZM5G870932_P01	TRUE	TRUE	gVPSEILDPINTWTDk	95%	n+304 (+304), K+304 (+304)	48.77	25.51
1104	seq=translation; coord=9:141825338..141830182:1; parent_transcript=GRMZM5G870932_T01; parent_gene=GRMZM5G870932	GRMZM5G870932_P01	TRUE	TRUE	nFEVFASYk	95%	n+304 (+304), K+304 (+304)	29.62	25.72
1105	seq=translation; coord=9:141825338..141830182:1; parent_transcript=GRMZM5G870932_T01; parent_gene=GRMZM5G870932	GRMZM5G870932_P01	TRUE	TRUE	nVILLAcDAFGVLPPVSk	95%	n+304 (+304), Carbamidomethyl (+57), K+304 (+304)	27.79	25.00

1106	seq=translation; coord=9:141825338..141830182:1; parent_transcript=GRMZM5G870932_T01; parent_gene=GRMZM5G870932	GRMZM5G870932_P01	TRUE	TRUE	qQLQSISASLASLTR	95%	n+304 (+304)	55.89	25.08
1107	seq=translation; coord=9:141825338..141830182:1; parent_transcript=GRMZM5G870932_T01; parent_gene=GRMZM5G870932	GRMZM5G870932_P01	TRUE	TRUE	tEVFGLEIPTEIk	95%	n+304 (+304), K+304 (+304)	37.39	25.98
1108	seq=translation; coord=9:141825338..141830182:1; parent_transcript=GRMZM5G870932_T01; parent_gene=GRMZM5G870932	GRMZM5G870932_P01	TRUE	TRUE	vFVNDQFLNWDPENR	95%	n+304 (+304)	38.00	25.00
1109	seq=translation; coord=9:141825338..141830182:1; parent_transcript=GRMZM5G870932_T01; parent_gene=GRMZM5G870932	GRMZM5G870932_P01	TRUE	TRUE	yGATGWLVNTGWSGGR	95%	n+304 (+304)	75.98	25.00
1110	seq=translation; coord=1:177027402..177032403:1; parent_transcript=GRMZM2G005887_T01; parent_gene=GRMZM2G005887	GRMZM2G005887_P01,GRMZM2G005887_P02, GRMZM2G005887_P05	TRUE	TRUE	dVTELIGNTPLVYLNk	95%	n+304 (+304), K+304 (+304)	38.81	25.00
1111	seq=translation; coord=1:177027402..177032403:1; parent_transcript=GRMZM2G005887_T01; parent_gene=GRMZM2G005887	GRMZM2G005887_P01,GRMZM2G005887_P02, GRMZM2G005887_P05	TRUE	TRUE	eGLLVGISSGAAAAAAVR	95%	n+304 (+304)	86.87	25.45
1112	seq=translation; coord=1:177027402..177032403:1; parent_transcript=GRMZM2G005887_T01; parent_gene=GRMZM2G005887	GRMZM2G005887_P01,GRMZM2G005887_P02, GRMZM2G005887_P05	TRUE	TRUE	eQNPNVk	94%	n+304 (+304), K+304 (+304)	27.66	25.00
1113	seq=translation; coord=1:177027402..177032403:1; parent_transcript=GRMZM2G005887_T01; parent_gene=GRMZM2G005887	GRMZM2G005887_P01,GRMZM2G005887_P02, GRMZM2G005887_P05	TRUE	TRUE	iDGLVSGIGTGGTITGTGR	95%	n+304 (+304)	83.75	25.34
1114	seq=translation; coord=1:177027402..177032403:1; parent_transcript=GRMZM2G005887_T01; parent_gene=GRMZM2G005887	GRMZM2G005887_P01,GRMZM2G005887_P02, GRMZM2G005887_P05	TRUE	TRUE	lFVVVFPSFGER	95%	n+304 (+304)	33.75	26.36
1115	seq=translation; coord=1:177027402..177032403:1; parent_transcript=GRMZM2G005887_T01; parent_gene=GRMZM2G005887	GRMZM2G005887_P01,GRMZM2G005887_P02, GRMZM2G005887_P05	TRUE	TRUE	lTLTMPASMSMER	90%	n+304 (+304)	27.36	25.00
1116	seq=translation; coord=1:177027402..177032403:1; parent_transcript=GRMZM2G005887_T01; parent_gene=GRMZM2G005887	GRMZM2G005887_P01,GRMZM2G005887_P02, GRMZM2G005887_P05	TRUE	TRUE	tPNSYILQQFENPANPk	95%	n+304 (+304), K+304 (+304)	47.61	26.11
1117	seq=translation; coord=1:177027402..177032403:1; parent_transcript=GRMZM2G005887_T01; parent_gene=GRMZM2G005887	GRMZM2G005887_P01,GRMZM2G005887_P02, GRMZM2G005887_P05	TRUE	TRUE	yLSSVLFQSIk	95%	n+304 (+304), K+304 (+304)	29.33	25.00
1118	seq=translation; coord=2:33534101..33535449:1; parent_transcript=GRMZM2G051943_T01; parent_gene=GRMZM2G051943	GRMZM2G051943_P01	TRUE	TRUE	nQAGSGcEGk	95%	n+304 (+304), Carbamidomethyl (+57), K+304 (+304)	34.11	25.00
1119	seq=translation; coord=2:33534101..33535449:1; parent_transcript=GRMZM2G051943_T01; parent_gene=GRMZM2G051943	GRMZM2G051943_P01	TRUE	TRUE	sAFLSAVNAYPGFAHGGTEVEGk	95%	n+304 (+304), K+304 (+304)	66.35	25.00
1120	seq=translation; coord=2:33534101..33535449:1; parent_transcript=GRMZM2G051943_T01; parent_gene=GRMZM2G051943	GRMZM2G051943_P01	TRUE	TRUE	sNAYcDASNR	95%	n+304 (+304), Carbamidomethyl (+57)	36.89	25.00
1121	seq=translation; coord=2:33534101..33535449:1; parent_transcript=GRMZM2G051943_T01; parent_gene=GRMZM2G051943	GRMZM2G051943_P01	TRUE	TRUE	tALWFWMNNVHR	95%	n+304 (+304)	39.67	25.00
1122	seq=translation; coord=2:33534101..33535449:1; parent_transcript=GRMZM2G051943_T01; parent_gene=GRMZM2G051943	GRMZM2G051943_P01	TRUE	TRUE	vAQDAVIAFk	95%	n+304 (+304), K+304 (+304)	44.95	25.51
1123	seq=translation; coord=2:33534101..33535449:1; parent_transcript=GRMZM2G051943_T01; parent_gene=GRMZM2G051943	GRMZM2G051943_P01	TRUE	TRUE	vAQDAVIAFkTALWFWMNNVHR	95%	n+304 (+304), K+304 (+304)	34.53	25.00

1124	seq=translation; coord=2:33534101..33535449:1; parent_transcript=GRMZM2G051943_T01; parent_gene=GRMZM2G051943	GRMZM2G051943_P01	TRUE	TRUE	vDPGPNLTc	90%	n+304 (+304), Carbamidomethyl (+57)	26.36	25.00
1125	seq=translation; coord=2:33534101..33535449:1; parent_transcript=GRMZM2G051943_T01; parent_gene=GRMZM2G051943	GRMZM2G051943_P01	TRUE	TRUE	vMPQGFGATIR	95%	n+304 (+304)	31.95	25.00
1126	seq=translation; coord=1:292089538..292095812:-1; parent_transcript=GRMZM2G347056_T01; parent_gene=GRMZM2G347056	GRMZM2G347056_P01	TRUE	TRUE	aFPSLTYIAVNk	95%	n+304 (+304), K+304 (+304)	33.85	25.00
1127	seq=translation; coord=1:292089538..292095812:-1; parent_transcript=GRMZM2G347056_T01; parent_gene=GRMZM2G347056	GRMZM2G347056_P01	TRUE	TRUE	aYLEFFcAk	95%	n+304 (+304), Carbamidomethyl (+57), K+304 (+304)	29.85	25.00
1128	seq=translation; coord=1:292089538..292095812:-1; parent_transcript=GRMZM2G347056_T01; parent_gene=GRMZM2G347056	GRMZM2G347056_P01	TRUE	TRUE	fTNFcQGk	92%	n+304 (+304), Carbamidomethyl (+57), K+304 (+304)	28.60	25.00
1129	seq=translation; coord=1:292089538..292095812:-1; parent_transcript=GRMZM2G347056_T01; parent_gene=GRMZM2G347056	GRMZM2G347056_P01	TRUE	TRUE	fVQVEGGFAcALDLVQHIR	95%	n+304 (+304), Carbamidomethyl (+57)	95.77	25.28
1130	seq=translation; coord=1:292089538..292095812:-1; parent_transcript=GRMZM2G347056_T01; parent_gene=GRMZM2G347056	GRMZM2G347056_P01	TRUE	TRUE	gWGcMFPEGDSSR	95%	n+304 (+304), Carbamidomethyl (+57)	46.78	25.00
1131	seq=translation; coord=1:292089538..292095812:-1; parent_transcript=GRMZM2G347056_T01; parent_gene=GRMZM2G347056	GRMZM2G347056_P01	TRUE	TRUE	iDHALETIk	92%	n+304 (+304), K+304 (+304)	29.25	25.00
1132	seq=translation; coord=1:292089538..292095812:-1; parent_transcript=GRMZM2G347056_T01; parent_gene=GRMZM2G347056	GRMZM2G347056_P01	TRUE	TRUE	iIDDQLVNINQk	95%	n+304 (+304), K+304 (+304)	57.98	25.12
1133	seq=translation; coord=1:292089538..292095812:-1; parent_transcript=GRMZM2G347056_T01; parent_gene=GRMZM2G347056	GRMZM2G347056_P01	TRUE	TRUE	iLEAAGDGR	95%	n+304 (+304)	36.09	25.50
1134	seq=translation; coord=1:292089538..292095812:-1; parent_transcript=GRMZM2G347056_T01; parent_gene=GRMZM2G347056	GRMZM2G347056_P01	TRUE	TRUE	iPSEITAALDPIkDNEEAVR	93%	n+304 (+304), K+304 (+304)	27.64	25.00
1135	seq=translation; coord=1:292089538..292095812:-1; parent_transcript=GRMZM2G347056_T01; parent_gene=GRMZM2G347056	GRMZM2G347056_P01	TRUE	TRUE	lQEEWAVPLk	95%	n+304 (+304), K+304 (+304)	39.62	25.00
1136	seq=translation; coord=1:292089538..292095812:-1; parent_transcript=GRMZM2G347056_T01; parent_gene=GRMZM2G347056	GRMZM2G347056_P01	TRUE	TRUE	lTSSPWSELDGLQPETk	95%	n+304 (+304), K+304 (+304)	55.97	25.29
1137	seq=translation; coord=1:292089538..292095812:-1; parent_transcript=GRMZM2G347056_T01; parent_gene=GRMZM2G347056	GRMZM2G347056_P01	TRUE	TRUE	sAIGILMNLGLIEESk	95%	n+304 (+304), K+304 (+304)	57.10	25.00
1138	seq=translation; coord=1:292089538..292095812:-1; parent_transcript=GRMZM2G347056_T01; parent_gene=GRMZM2G347056	GRMZM2G347056_P01	TRUE	TRUE	sDSPTVGWGGPGGYVYQk	95%	n+304 (+304), K+304 (+304)	57.13	25.00
1139	seq=translation; coord=1:292089538..292095812:-1; parent_transcript=GRMZM2G347056_T01; parent_gene=GRMZM2G347056	GRMZM2G347056_P01	TRUE	TRUE	tEEGVENLFER	95%	n+304 (+304)	53.47	25.00
1140	seq=translation; coord=1:292089538..292095812:-1; parent_transcript=GRMZM2G347056_T01; parent_gene=GRMZM2G347056	GRMZM2G347056_P01	TRUE	TRUE	tLGWDQYPHGR	95%	n+304 (+304)	38.59	25.00
1141	seq=translation; coord=7:155357370..155360570:1; parent_transcript=GRMZM2G058310_T01; parent_gene=GRMZM2G058310	GRMZM2G058310_P01	TRUE	TRUE	aAAEEAGHPEWDLPDDAGTYNDTPEk	95%	n+304 (+304), K+304 (+304)	31.80	25.00

1142	seq=translation; coord=7:155357370..155360570:1; parent_transcript=GRMZM2G058310_T01; parent_gene=GRMZM2G058310	GRMZM2G058310_P01	TRUE	TRUE	aEIPIEEILEVAQPk	95%	n+304 (+304), K+304 (+304)	67.32	25.00
1143	seq=translation; coord=7:155357370..155360570:1; parent_transcript=GRMZM2G058310_T01; parent_gene=GRMZM2G058310	GRMZM2G058310_P01	TRUE	TRUE	aSMNFTcAEMR	91%	n+304 (+304), Carbamidomethyl (+57)	28.10	25.00
1144	seq=translation; coord=7:155357370..155360570:1; parent_transcript=GRMZM2G058310_T01; parent_gene=GRMZM2G058310	GRMZM2G058310_P01	TRUE	TRUE	eGLNLAcENALNR	95%	n+304 (+304), Carbamidomethyl (+57)	57.24	25.00
1145	seq=translation; coord=7:155357370..155360570:1; parent_transcript=GRMZM2G058310_T01; parent_gene=GRMZM2G058310	GRMZM2G058310_P01	TRUE	TRUE	ePGVYDWSAYR	94%	n+304 (+304)	30.94	25.00
1146	seq=translation; coord=7:155357370..155360570:1; parent_transcript=GRMZM2G058310_T01; parent_gene=GRMZM2G058310	GRMZM2G058310_P01	TRUE	TRUE	fFLTWYSNk	93%	n+304 (+304), K+304 (+304)	28.72	26.25
1147	seq=translation; coord=7:155357370..155360570:1; parent_transcript=GRMZM2G058310_T01; parent_gene=GRMZM2G058310	GRMZM2G058310_P01	TRUE	TRUE	lVQEAGLk	93%	n+304 (+304), K+304 (+304)	32.75	27.85
1148	seq=translation; coord=7:155357370..155360570:1; parent_transcript=GRMZM2G058310_T01; parent_gene=GRMZM2G058310	GRMZM2G058310_P01	TRUE	TRUE	mHANLDYNPNVDPVAPLER	95%	n+304 (+304)	41.01	25.00
1149	seq=translation; coord=7:155357370..155360570:1; parent_transcript=GRMZM2G058310_T01; parent_gene=GRMZM2G058310	GRMZM2G058310_P01	TRUE	TRUE	sAPEELVQQVLSAGWR	95%	n+304 (+304)	51.50	25.31
1150	seq=translation; coord=7:155357370..155360570:1; parent_transcript=GRMZM2G058310_T01; parent_gene=GRMZM2G058310	GRMZM2G058310_P01	TRUE	TRUE	sGLTNIEYLTLGVDDQPLFHGR	95%	n+304 (+304)	77.78	25.45
1151	seq=translation; coord=7:155357370..155360570:1; parent_transcript=GRMZM2G058310_T01; parent_gene=GRMZM2G058310	GRMZM2G058310_P01	TRUE	TRUE	sGLTNIEyLTLGVDDQPLFHGR	95%	n+304 (+304), iTRAQ8plex (+304)	42.96	25.69
1152	seq=translation; coord=7:155357370..155360570:1; parent_transcript=GRMZM2G058310_T01; parent_gene=GRMZM2G058310	GRMZM2G058310_P01	TRUE	TRUE	vSDELFQEQNYTTFk	89%	n+304 (+304), K+304 (+304)	25.11	25.00
1153	seq=translation; coord=7:155357370..155360570:1; parent_transcript=GRMZM2G058310_T01; parent_gene=GRMZM2G058310	GRMZM2G058310_P01	TRUE	TRUE	yDATAYNTILR	95%	n+304 (+304)	34.13	25.00
1154	seq=translation; coord=7:155357370..155360570:1; parent_transcript=GRMZM2G058310_T01; parent_gene=GRMZM2G058310	GRMZM2G058310_P01	TRUE	TRUE	yPSYPQSQGWVFPGVGEFIcYDk	95%	n+304 (+304), Carbamidomethyl (+57), K+304 (+304)	29.35	25.00
1155	seq=translation; coord=4:32249665..32251536:-1; parent_transcript=AC196475.3_FGT004; parent_gene=AC196475.3_FG004	AC196475.3_FGP004	TRUE	TRUE	aALAPEEVVAR	95%	n+304 (+304)	46.45	25.90
1156	seq=translation; coord=4:32249665..32251536:-1; parent_transcript=AC196475.3_FGT004; parent_gene=AC196475.3_FG004	AC196475.3_FGP004	TRUE	TRUE	aQGVFHVDMIMLAHNPGGk	95%	n+304 (+304), K+304 (+304)	34.29	26.74
1157	seq=translation; coord=4:32249665..32251536:-1; parent_transcript=AC196475.3_FGT004; parent_gene=AC196475.3_FG004	AC196475.3_FGP004	TRUE	TRUE	dAVLDGGIPFNk	91%	n+304 (+304), K+304 (+304)	26.84	25.95
1158	seq=translation; coord=4:32249665..32251536:-1; parent_transcript=AC196475.3_FGT004; parent_gene=AC196475.3_FG004	AC196475.3_FGP004	TRUE	TRUE	gAGFSGFk	92%	n+304 (+304), K+304 (+304)	27.61	26.00
1159	seq=translation; coord=4:32249665..32251536:-1; parent_transcript=AC196475.3_FGT004; parent_gene=AC196475.3_FG004	AC196475.3_FGP004	TRUE	TRUE	hVGGDMFASVPAGDAILMk	95%	n+304 (+304), K+304 (+304)	39.01	25.17

1160	seq=translation; coord=4:32249665..32251536:-1; parent_transcript=AC196475.3_FGT004; parent_gene=AC196475.3_FG004	AC196475.3_FGP004	TRUE	TRUE	lLASYDVVR	95%	n+304 (+304)	37.03	25.00
1161	seq=translation; coord=4:32249665..32251536:-1; parent_transcript=AC196475.3_FGT004; parent_gene=AC196475.3_FG004	AC196475.3_FGP004	TRUE	TRUE	mPAAPGDPAAAAAMVDR	94%	n+304 (+304)	31.00	25.00
1162	seq=translation; coord=4:32249665..32251536:-1; parent_transcript=AC196475.3_FGT004; parent_gene=AC196475.3_FG004	AC196475.3_FGP004	TRUE	TRUE	nAIELGLLEVLQk	95%	n+304 (+304), K+304 (+304)	63.35	25.00
1163	seq=translation; coord=4:32249665..32251536:-1; parent_transcript=AC196475.3_FGT004; parent_gene=AC196475.3_FG004	AC196475.3_FGP004	TRUE	TRUE	vIVVEcVLPVNTEATPk	95%	n+304 (+304), Carbamidomethyl (+57), K+304 (+304)	32.83	25.54
1164	seq=translation; coord=4:32249665..32251536:-1; parent_transcript=AC196475.3_FGT004; parent_gene=AC196475.3_FG004	AC196475.3_FGP004	TRUE	TRUE	vLMESWYYLk	94%	n+304 (+304), K+304 (+304)	29.04	25.60
1165	seq=translation; coord=4:32249665..32251536:-1; parent_transcript=AC196475.3_FGT004; parent_gene=AC196475.3_FG004	AC196475.3_FGP004	TRUE	TRUE	wILHDWSDAHcATLLk	95%	n+304 (+304), Carbamidomethyl (+57), K+304 (+304)	34.05	25.69
1166	seq=translation; coord=4:32249665..32251536:-1; parent_transcript=AC196475.3_FGT004; parent_gene=AC196475.3_FG004	AC196475.3_FGP004	TRUE	TRUE	wLTPNEDGVSMAALALMNQDk	95%	n+304 (+304), K+304 (+304)	35.73	25.19
1167	seq=translation; coord=4:32249665..32251536:-1; parent_transcript=AC196475.3_FGT004; parent_gene=AC196475.3_FG004	AC196475.3_FGP004	TRUE	TRUE	wLTPNEDGVSmAALALMNQDk	95%	n+304 (+304), Oxidation (+16), K+304 (+304)	33.43	25.51
1168	seq=translation; coord=6:122797861..122838407:1; parent_transcript=GRMZM2G032505_T02; parent_gene=GRMZM2G032505	GRMZM2G032505_P02,GRMZM2G032505_P03	TRUE	TRUE	aDILDPALMR	95%	n+304 (+304)	42.25	25.93
1169	seq=translation; coord=6:122797861..122838407:1; parent_transcript=GRMZM2G032505_T02; parent_gene=GRMZM2G032505	GRMZM2G032505_P02,GRMZM2G032505_P03	TRUE	TRUE	aPcIIFIDEIDAIGTk	95%	n+304 (+304), Carbamidomethyl (+57), K+304 (+304)	52.44	25.28
1170	seq=translation; coord=6:122797861..122838407:1; parent_transcript=GRMZM2G032505_T02; parent_gene=GRMZM2G032505	GRMZM2G032505_P02,GRMZM2G032505_P03	TRUE	TRUE	dATEVTHEDFNEGIVQVQAk	95%	n+304 (+304), K+304 (+304)	37.96	25.22
1171	seq=translation; coord=6:122797861..122838407:1; parent_transcript=GRMZM2G032505_T02; parent_gene=GRMZM2G032505	GRMZM2G032505_P02,GRMZM2G032505_P03	TRUE	TRUE	dSYLILDTLPSEYDSR	95%	n+304 (+304)	31.51	25.00
1172	seq=translation; coord=6:122797861..122838407:1; parent_transcript=GRMZM2G032505_T02; parent_gene=GRMZM2G032505	GRMZM2G032505_P02,GRMZM2G032505_P03	TRUE	TRUE	kMNVNPDVNFEELAR	95%	K+304 (+304), n+304 (+304)	41.23	25.91
1173	seq=translation; coord=6:122797861..122838407:1; parent_transcript=GRMZM2G032505_T02; parent_gene=GRMZM2G032505	GRMZM2G032505_P02,GRMZM2G032505_P03	TRUE	TRUE	lAGPQLVQMFIGDGAk	95%	n+304 (+304), K+304 (+304)	39.98	26.24
1174	seq=translation; coord=6:122797861..122838407:1; parent_transcript=GRMZM2G032505_T02; parent_gene=GRMZM2G032505	GRMZM2G032505_P02,GRMZM2G032505_P03	TRUE	TRUE	qIQELVEAIVLPMTHk	95%	n+304 (+304), K+304 (+304)	45.95	25.00
1175	seq=translation; coord=6:122797861..122838407:1; parent_transcript=GRMZM2G032505_T02; parent_gene=GRMZM2G032505	GRMZM2G032505_P02,GRMZM2G032505_P03	TRUE	TRUE	qIQELVEAIVLPMTHk	92%	Pyro-cmC (-17), n+304 (+304), K+304 (+304)	27.52	25.00
1176	seq=translation; coord=6:122797861..122838407:1; parent_transcript=GRMZM2G032505_T02; parent_gene=GRMZM2G032505	GRMZM2G032505_P02,GRMZM2G032505_P03	TRUE	TRUE	tMLELLNQLDGFSSDER	95%	n+304 (+304)	35.75	25.00
1177	seq=translation; coord=3:151819389..151821475:1; parent_transcript=GRMZM2G125032_T01; parent_gene=GRMZM2G125032	GRMZM2G125032_P01	TRUE	TRUE	dISLGYATFQPGTTVR	95%	n+304 (+304)	63.17	25.07

1178	seq=translation; coord=3:151819389..151821475:1; parent_transcript=GRMZM2G125032_T01; parent_gene=GRMZM2G125032	GRMZM2G125032_P01	TRUE	TRUE	fDVVANSFPPSSGSFAQGyMADVAR	95%	n+304 (+304), iTRAQ8plex (+304)	41.42	25.00
1179	seq=translation; coord=3:151819389..151821475:1; parent_transcript=GRMZM2G125032_T01; parent_gene=GRMZM2G125032	GRMZM2G125032_P01	TRUE	TRUE	nLDAALAR	94%	n+304 (+304)	31.07	25.68
1180	seq=translation; coord=3:151819389..151821475:1; parent_transcript=GRMZM2G125032_T01; parent_gene=GRMZM2G125032	GRMZM2G125032_P01	TRUE	TRUE	rTGPLETFVFAMFNENQk	95%	n+304 (+304), K+304 (+304)	29.42	25.00
1181	seq=translation; coord=3:151819389..151821475:1; parent_transcript=GRMZM2G125032_T01; parent_gene=GRMZM2G125032	GRMZM2G125032_P01	TRUE	TRUE	sASFADSWVQSNVRPYYPAVGIk	95%	n+304 (+304), K+304 (+304)	30.20	25.00
1182	seq=translation; coord=3:151819389..151821475:1; parent_transcript=GRMZM2G125032_T01; parent_gene=GRMZM2G125032	GRMZM2G125032_P01	TRUE	TRUE	tGPLETFVFAMFNENQk	95%	n+304 (+304), K+304 (+304)	41.18	25.00
1183	seq=translation; coord=3:151819389..151821475:1; parent_transcript=GRMZM2G125032_T01; parent_gene=GRMZM2G125032	GRMZM2G125032_P01	TRUE	TRUE	vVVSESGWPSAGGFGASVDNAR	95%	n+304 (+304)	62.64	25.00
1184	seq=translation; coord=3:151819389..151821475:1; parent_transcript=GRMZM2G125032_T01; parent_gene=GRMZM2G125032	GRMZM2G125032_P01	TRUE	TRUE	yLAGTGAPLLANVYPYFAYR	95%	n+304 (+304)	29.78	25.00
1185	seq=translation; coord=3:151819389..151821475:1; parent_transcript=GRMZM2G125032_T01; parent_gene=GRMZM2G125032	GRMZM2G125032_P01	TRUE	TRUE	yVAVGNEVQGDDTR	95%	n+304 (+304)	62.03	25.00
1186	seq=translation; coord=3:56281784..56287937:1; parent_transcript=GRMZM2G136106_T01; parent_gene=GRMZM2G136106	GRMZM2G136106_P01	TRUE	TRUE	aGAYmENVEVGk	95%	n+304 (+304), Oxidation (+16), K+304 (+304)	52.10	25.00
1187	seq=translation; coord=3:56281784..56287937:1; parent_transcript=GRMZM2G136106_T01; parent_gene=GRMZM2G136106	GRMZM2G136106_P01	TRUE	TRUE	aSTADLASPAGR	95%	n+304 (+304)	44.17	25.00
1188	seq=translation; coord=3:56281784..56287937:1; parent_transcript=GRMZM2G136106_T01; parent_gene=GRMZM2G136106	GRMZM2G136106_P01	TRUE	TRUE	aTMEHVMTVLSAAITNQYTcLDGFAYQSGGR	95%	n+304 (+304), Carbamidomethyl (+57)	29.17	25.00
1189	seq=translation; coord=3:56281784..56287937:1; parent_transcript=GRMZM2G136106_T01; parent_gene=GRMZM2G136106	GRMZM2G136106_P01	TRUE	TRUE	aTmEHVMTVLSAAITNQYTcLDGFAYQSGGR	95%	n+304 (+304), Oxidation (+16), Carbamidomethyl (+57)	49.16	25.00
1190	seq=translation; coord=3:56281784..56287937:1; parent_transcript=GRMZM2G136106_T01; parent_gene=GRMZM2G136106	GRMZM2G136106_P01	TRUE	TRUE	dGSGDYTTVAAAVAAAPTNSk	95%	n+304 (+304), K+304 (+304)	29.32	25.00
1191	seq=translation; coord=3:56281784..56287937:1; parent_transcript=GRMZM2G136106_T01; parent_gene=GRMZM2G136106	GRMZM2G136106_P01	TRUE	TRUE	dLTIENSAGPSk	95%	n+304 (+304), K+304 (+304)	44.68	26.05
1192	seq=translation; coord=3:56281784..56287937:1; parent_transcript=GRMZM2G136106_T01; parent_gene=GRMZM2G136106	GRMZM2G136106_P01	TRUE	TRUE	eDPNQNTGISIQR	95%	n+304 (+304)	40.56	25.00
1193	seq=translation; coord=3:56281784..56287937:1; parent_transcript=GRMZM2G136106_T01; parent_gene=GRMZM2G136106	GRMZM2G136106_P01	TRUE	TRUE	hVIYIk	95%	n+304 (+304), K+304 (+304)	35.03	27.90
1194	seq=translation; coord=3:56281784..56287937:1; parent_transcript=GRMZM2G136106_T01; parent_gene=GRMZM2G136106	GRMZM2G136106_P01	TRUE	TRUE	hVNLMFVGDGIGk	95%	n+304 (+304), K+304 (+304)	45.99	26.41
1195	seq=translation; coord=3:56281784..56287937:1; parent_transcript=GRMZM2G136106_T01; parent_gene=GRMZM2G136106	GRMZM2G136106_P01	TRUE	TRUE	lAINDcLELLSTTMDELR	90%	n+304 (+304), Carbamidomethyl (+57)	27.51	25.00

1196	seq=translation; coord=3:56281784..56287937:1; parent_transcript=GRMZM2G136106_T01; parent_gene=GRMZM2G136106	GRMZM2G136106_P01	TRUE	TRUE	lAINDcLELLSTTmDELR	95%	n+304 (+304), Carbamidomethyl (+57), Oxidation (+16)	33.19	25.00
1197	seq=translation; coord=3:56281784..56287937:1; parent_transcript=GRMZM2G136106_T01; parent_gene=GRMZM2G136106	GRMZM2G136106_P01	TRUE	TRUE	mVSNSLAMAk	95%	n+304 (+304), K+304 (+304)	40.99	26.07
1198	seq=translation; coord=3:56281784..56287937:1; parent_transcript=GRMZM2G136106_T01; parent_gene=GRMZM2G136106	GRMZM2G136106_P01	TRUE	TRUE	nVVDGYTTFR	95%	n+304 (+304)	37.73	25.00
1199	seq=translation; coord=3:56281784..56287937:1; parent_transcript=GRMZM2G136106_T01; parent_gene=GRMZM2G136106	GRMZM2G136106_P01	TRUE	TRUE	sATVAVVGNNFLAR	95%	n+304 (+304)	70.75	25.02
1200	seq=translation; coord=3:56281784..56287937:1; parent_transcript=GRMZM2G136106_T01; parent_gene=GRMZM2G136106	GRMZM2G136106_P01	TRUE	TRUE	vAAAADLAAAQSSTk	95%	n+304 (+304), K+304 (+304)	94.00	25.99
1201	seq=translation; coord=3:56281784..56287937:1; parent_transcript=GRMZM2G136106_T01; parent_gene=GRMZM2G136106	GRMZM2G136106_P01	TRUE	TRUE	vGADLSAFYR	95%	n+304 (+304)	40.26	25.00
1202	seq=translation; coord=5:213541443..213545321:-1; parent_transcript=GRMZM2G178958_T01; parent_gene=GRMZM2G178958	GRMZM2G178958_P01	TRUE	TRUE	dIEFSEWk	95%	n+304 (+304), K+304 (+304)	29.53	25.00
1203	seq=translation; coord=5:213541443..213545321:-1; parent_transcript=GRMZM2G178958_T01; parent_gene=GRMZM2G178958	GRMZM2G178958_P01	TRUE	TRUE	eVAAASEVSGEk	95%	n+304 (+304), K+304 (+304)	52.01	25.98
1204	seq=translation; coord=5:213541443..213545321:-1; parent_transcript=GRMZM2G178958_T01; parent_gene=GRMZM2G178958	GRMZM2G178958_P01	TRUE	TRUE	gDILAVAVTEk	95%	n+304 (+304), K+304 (+304)	45.29	25.66
1205	seq=translation; coord=5:213541443..213545321:-1; parent_transcript=GRMZM2G178958_T01; parent_gene=GRMZM2G178958	GRMZM2G178958_P01	TRUE	TRUE	gLTFDSGGYNIk	95%	n+304 (+304), K+304 (+304)	33.00	25.33
1206	seq=translation; coord=5:213541443..213545321:-1; parent_transcript=GRMZM2G178958_T01; parent_gene=GRMZM2G178958	GRMZM2G178958_P01	TRUE	TRUE	lAGQGFk	91%	n+304 (+304), K+304 (+304)	26.43	25.97
1207	seq=translation; coord=5:213541443..213545321:-1; parent_transcript=GRMZM2G178958_T01; parent_gene=GRMZM2G178958	GRMZM2G178958_P01	TRUE	TRUE	lPLEESYWESMk	90%	n+304 (+304), K+304 (+304)	27.40	25.74
1208	seq=translation; coord=5:213541443..213545321:-1; parent_transcript=GRMZM2G178958_T01; parent_gene=GRMZM2G178958	GRMZM2G178958_P01	TRUE	TRUE	lTAAAAVASGTVLGLYEDSR	95%	n+304 (+304)	31.14	25.94
1209	seq=translation; coord=5:213541443..213545321:-1; parent_transcript=GRMZM2G178958_T01; parent_gene=GRMZM2G178958	GRMZM2G178958_P01	TRUE	TRUE	lTLADALVYAcNQGVEk	95%	n+304 (+304), Carbamidomethyl (+57), K+304 (+304)	40.84	25.08
1210	seq=translation; coord=5:213541443..213545321:-1; parent_transcript=GRMZM2G178958_T01; parent_gene=GRMZM2G178958	GRMZM2G178958_P01	TRUE	TRUE	qGGSITAALFLk	95%	n+304 (+304), K+304 (+304)	32.50	25.00
1211	seq=translation; coord=5:213541443..213545321:-1; parent_transcript=GRMZM2G178958_T01; parent_gene=GRMZM2G178958	GRMZM2G178958_P01	TRUE	TRUE	qVDIIGLGSGAEVDQk	95%	n+304 (+304), K+304 (+304)	65.01	25.60
1212	seq=translation; coord=5:213541443..213545321:-1; parent_transcript=GRMZM2G178958_T01; parent_gene=GRMZM2G178958	GRMZM2G178958_P01	TRUE	TRUE	tIEVNNTDAEGR	95%	n+304 (+304)	38.65	25.00
1213	seq=translation; coord=5:213541443..213545321:-1; parent_transcript=GRMZM2G178958_T01; parent_gene=GRMZM2G178958	GRMZM2G178958_P01	TRUE	TRUE	vGLIGLGQSAPSTAAASR	94%	n+304 (+304)	29.02	25.58

1214	seq=translation; coord=5:213541443..213545321:-1; parent_transcript=GRMZM2G178958_T01; parent_gene=GRMZM2G178958	GRMZM2G178958_P01	TRUE	TRUE	vQWMHIDMAGPVWSDk	94%	n+304 (+304), K+304 (+304)	26.07	25.00
1215	seq=translation; coord=5:213541443..213545321:-1; parent_transcript=GRMZM2G178958_T01; parent_gene=GRMZM2G178958	GRMZM2G178958_P01	TRUE	TRUE	yANDLSSGVIFGR	95%	n+304 (+304)	33.14	25.00
1216	seq=translation; coord=10:142329418..142336291:1; parent_transcript=GRMZM2G114182_T01; parent_gene=GRMZM2G114182	GRMZM2G114182_P01	TRUE	TRUE	aASLEAVVEAASEk	95%	n+304 (+304), K+304 (+304)	49.91	25.83
1217	seq=translation; coord=10:142329418..142336291:1; parent_transcript=GRMZM2G114182_T01; parent_gene=GRMZM2G114182	GRMZM2G114182_P01	TRUE	TRUE	aEELELQVAALk	95%	n+304 (+304), K+304 (+304)	34.29	25.29
1218	seq=translation; coord=10:142329418..142336291:1; parent_transcript=GRMZM2G114182_T01; parent_gene=GRMZM2G114182	GRMZM2G114182_P01	TRUE	TRUE	aQLVLLEALSQk	95%	n+304 (+304), K+304 (+304)	47.61	25.00
1219	seq=translation; coord=10:142329418..142336291:1; parent_transcript=GRMZM2G114182_T01; parent_gene=GRMZM2G114182	GRMZM2G114182_P01	TRUE	TRUE	aSQLSDDLEAYQTk	95%	n+304 (+304), K+304 (+304)	54.63	25.00
1220	seq=translation; coord=10:142329418..142336291:1; parent_transcript=GRMZM2G114182_T01; parent_gene=GRMZM2G114182	GRMZM2G114182_P01	TRUE	TRUE	dLTEAENk	95%	n+304 (+304), K+304 (+304)	49.33	25.00
1221	seq=translation; coord=10:142329418..142336291:1; parent_transcript=GRMZM2G114182_T01; parent_gene=GRMZM2G114182	GRMZM2G114182_P01	TRUE	TRUE	gVEEELEQYR	88%	n+304 (+304)	26.15	25.00
1222	seq=translation; coord=10:142329418..142336291:1; parent_transcript=GRMZM2G114182_T01; parent_gene=GRMZM2G114182	GRMZM2G114182_P01	TRUE	TRUE	iLDLEAQIQAMHAAEQALk	95%	n+304 (+304), K+304 (+304)	45.43	25.01
1223	seq=translation; coord=10:142329418..142336291:1; parent_transcript=GRMZM2G114182_T01; parent_gene=GRMZM2G114182	GRMZM2G114182_P01	TRUE	TRUE	iQEIEAELDSSADk	95%	n+304 (+304), K+304 (+304)	28.23	25.44
1224	seq=translation; coord=10:142329418..142336291:1; parent_transcript=GRMZM2G114182_T01; parent_gene=GRMZM2G114182	GRMZM2G114182_P01	TRUE	TRUE	iQQEAAVSAER	95%	n+304 (+304)	35.37	25.43
1225	seq=translation; coord=10:142329418..142336291:1; parent_transcript=GRMZM2G114182_T01; parent_gene=GRMZM2G114182	GRMZM2G114182_P01	TRUE	TRUE	lEEEMNVDk	95%	n+304 (+304), K+304 (+304)	50.97	25.00
1226	seq=translation; coord=10:142329418..142336291:1; parent_transcript=GRMZM2G114182_T01; parent_gene=GRMZM2G114182	GRMZM2G114182_P01	TRUE	TRUE	lETELTTVLEELQAk	95%	n+304 (+304), K+304 (+304)	44.56	25.00
1227	seq=translation; coord=10:142329418..142336291:1; parent_transcript=GRMZM2G114182_T01; parent_gene=GRMZM2G114182	GRMZM2G114182_P01	TRUE	TRUE	lQLAYSk	95%	n+304 (+304), K+304 (+304)	31.95	25.00
1228	seq=translation; coord=10:142329418..142336291:1; parent_transcript=GRMZM2G114182_T01; parent_gene=GRMZM2G114182	GRMZM2G114182_P01	TRUE	TRUE	lVMVDELQEk	95%	n+304 (+304), K+304 (+304)	38.17	25.79
1229	seq=translation; coord=10:142329418..142336291:1; parent_transcript=GRMZM2G114182_T01; parent_gene=GRMZM2G114182	GRMZM2G114182_P01	TRUE	TRUE	mLELAQSNMk	95%	n+304 (+304), K+304 (+304)	30.34	26.41
1230	seq=translation; coord=10:142329418..142336291:1; parent_transcript=GRMZM2G114182_T01; parent_gene=GRMZM2G114182	GRMZM2G114182_P01	TRUE	TRUE	qITLSEIk	95%	n+304 (+304), K+304 (+304)	36.83	25.00
1231	seq=translation; coord=10:142329418..142336291:1; parent_transcript=GRMZM2G114182_T01; parent_gene=GRMZM2G114182	GRMZM2G114182_P01	TRUE	TRUE	tLDGMIEHk	95%	n+304 (+304), K+304 (+304)	28.83	25.43

1232	seq=translation; coord=10:142329418..142336291:1; parent_transcript=GRMZM2G114182_T01; parent_gene=GRMZM2G114182	GRMZM2G114182_P01	TRUE	TRUE	vVQEALELSk	95%	n+304 (+304), K+304 (+304)	35.28	25.21
1233	seq=translation; coord=1:12140829..12142573:1; parent_transcript=GRMZM2G300801_T01; parent_gene=GRMZM2G300801	GRMZM2G300801_P01	TRUE	TRUE	aGALYDTIDASGGYYVcPVDk	95%	n+304 (+304), Carbamidomethyl (+57), K+304 (+304)	54.54	25.00
1234	seq=translation; coord=1:12140829..12142573:1; parent_transcript=GRMZM2G300801_T01; parent_gene=GRMZM2G300801	GRMZM2G300801_P01	TRUE	TRUE	aSIYNAMPLAGVEk	95%	n+304 (+304), K+304 (+304)	43.13	26.18
1235	seq=translation; coord=1:12140829..12142573:1; parent_transcript=GRMZM2G300801_T01; parent_gene=GRMZM2G300801	GRMZM2G300801_P01	TRUE	TRUE	fGVIYAGAQk	95%	n+304 (+304), K+304 (+304)	45.02	25.00
1236	seq=translation; coord=1:12140829..12142573:1; parent_transcript=GRMZM2G300801_T01; parent_gene=GRMZM2G300801	GRMZM2G300801_P01	TRUE	TRUE	fSAASVAWSGk	92%	n+304 (+304), K+304 (+304)	28.90	26.22
1237	seq=translation; coord=1:12140829..12142573:1; parent_transcript=GRMZM2G300801_T01; parent_gene=GRMZM2G300801	GRMZM2G300801_P01	TRUE	TRUE	gVYNFAAGPATLPLSVLk	95%	n+304 (+304), K+304 (+304)	43.56	25.00
1238	seq=translation; coord=1:12140829..12142573:1; parent_transcript=GRMZM2G300801_T01; parent_gene=GRMZM2G300801	GRMZM2G300801_P01	TRUE	TRUE	kAGALYDTIDASGGYYVcPVDk	95%	K+304 (+304), n+304 (+304), Carbamidomethyl (+57), K+304 (+304)	27.90	25.00
1239	seq=translation; coord=1:12140829..12142573:1; parent_transcript=GRMZM2G300801_T01; parent_gene=GRMZM2G300801	GRMZM2G300801_P01	TRUE	TRUE	kDLIGAAQPITPVMLDFk	95%	K+304 (+304), n+304 (+304), K+304 (+304)	31.38	25.00
1240	seq=translation; coord=1:12140829..12142573:1; parent_transcript=GRMZM2G300801_T01; parent_gene=GRMZM2G300801	GRMZM2G300801_P01	TRUE	TRUE	lVAFMk	90%	n+304 (+304), K+304 (+304)	25.35	25.00
1241	seq=translation; coord=1:12140829..12142573:1; parent_transcript=GRMZM2G300801_T01; parent_gene=GRMZM2G300801	GRMZM2G300801_P01	TRUE	TRUE	nVGPSGVTIAIVR	95%	n+304 (+304)	53.46	25.68
1242	seq=translation; coord=1:12140829..12142573:1; parent_transcript=GRMZM2G300801_T01; parent_gene=GRMZM2G300801	GRMZM2G300801_P01	TRUE	TRUE	qFIAEAAk	95%	n+304 (+304), K+304 (+304)	35.05	25.93
1243	seq=translation; coord=1:12140829..12142573:1; parent_transcript=GRMZM2G300801_T01; parent_gene=GRMZM2G300801	GRMZM2G300801_P01	TRUE	TRUE	sGILVADMSSNFcSkPVDVSR	95%	n+304 (+304), Carbamidomethyl (+57), K+304 (+304)	34.34	25.00
1244	seq=translation; coord=1:12140829..12142573:1; parent_transcript=GRMZM2G300801_T01; parent_gene=GRMZM2G300801	GRMZM2G300801_P01	TRUE	TRUE	sHMNVPFTLAk	95%	n+304 (+304), K+304 (+304)	28.96	26.05
1245	seq=translation; coord=1:12140829..12142573:1; parent_transcript=GRMZM2G300801_T01; parent_gene=GRMZM2G300801	GRMZM2G300801_P01	TRUE	TRUE	yTALPPFDAIAQNPEAR	95%	n+304 (+304)	49.47	25.00
1246	seq=translation; coord=5:168451664..168468750:-1; parent_transcript=GRMZM2G032628_T01; parent_gene=GRMZM2G032628	GRMZM2G032628_P01	TRUE	TRUE	aVMVPEGENDGLASR	95%	n+304 (+304)	57.87	25.00
1247	seq=translation; coord=5:168451664..168468750:-1; parent_transcript=GRMZM2G032628_T01; parent_gene=GRMZM2G032628	GRMZM2G032628_P01	TRUE	TRUE	iFQIDPMLQGYk	95%	n+304 (+304), K+304 (+304)	31.08	25.58
1248	seq=translation; coord=5:168451664..168468750:-1; parent_transcript=GRMZM2G032628_T01; parent_gene=GRMZM2G032628	GRMZM2G032628_P01	TRUE	TRUE	lFNYGNWEVLR	95%	n+304 (+304)	30.05	25.00
1249	seq=translation; coord=5:168451664..168468750:-1; parent_transcript=GRMZM2G032628_T01; parent_gene=GRMZM2G032628	GRMZM2G032628_P01	TRUE	TRUE	mGDIVHTLTNR	95%	n+304 (+304)	41.63	25.00

1250	seq=translation; coord=5:168451664..168468750:-1; parent_transcript=GRMZM2G032628_T01; parent_gene=GRMZM2G032628	GRMZM2G032628_P01	TRUE	TRUE	vVLDSDAGLFGGFSR	95%	n+304 (+304)	57.07	25.00
1251	seq=translation; coord=5:168451664..168468750:-1; parent_transcript=GRMZM2G032628_T01; parent_gene=GRMZM2G032628	GRMZM2G032628_P01	TRUE	TRUE	vVPPPSDGQk	95%	n+304 (+304), K+304 (+304)	42.24	25.77
1252	seq=translation; coord=5:168451664..168468750:-1; parent_transcript=GRMZM2G032628_T01; parent_gene=GRMZM2G032628	GRMZM2G032628_P01	TRUE	TRUE	ySVQAPGEIPYDGIYYDPPEEVk	92%	n+304 (+304), K+304 (+304)	25.76	25.00
1253	seq=translation; coord=8:21816689..21823022:1; parent_transcript=GRMZM2G032049_T01; parent_gene=GRMZM2G032049	GRMZM2G032049_P01	TRUE	TRUE	aEYDPLk	93%	n+304 (+304), K+304 (+304)	29.72	26.27
1254	seq=translation; coord=8:21816689..21823022:1; parent_transcript=GRMZM2G032049_T01; parent_gene=GRMZM2G032049	GRMZM2G032049_P01	TRUE	TRUE	aGPALPLEALLALGLDQR	95%	n+304 (+304)	48.30	25.00
1255	seq=translation; coord=8:21816689..21823022:1; parent_transcript=GRMZM2G032049_T01; parent_gene=GRMZM2G032049	GRMZM2G032049_P01	TRUE	TRUE	aMFIDFGLAk	95%	n+304 (+304), K+304 (+304)	40.46	25.12
1256	seq=translation; coord=8:21816689..21823022:1; parent_transcript=GRMZM2G032049_T01; parent_gene=GRMZM2G032049	GRMZM2G032049_P01	TRUE	TRUE	dAYAVPSLATAVLGDk	95%	n+304 (+304), K+304 (+304)	40.82	25.79
1257	seq=translation; coord=8:21816689..21823022:1; parent_transcript=GRMZM2G032049_T01; parent_gene=GRMZM2G032049	GRMZM2G032049_P01	TRUE	TRUE	fDDTNPEAEk	95%	n+304 (+304), K+304 (+304)	55.13	25.00
1258	seq=translation; coord=8:21816689..21823022:1; parent_transcript=GRMZM2G032049_T01; parent_gene=GRMZM2G032049	GRMZM2G032049_P01	TRUE	TRUE	gLIAEGAATLR	95%	n+304 (+304)	33.56	26.40
1259	seq=translation; coord=8:21816689..21823022:1; parent_transcript=GRMZM2G032049_T01; parent_gene=GRMZM2G032049	GRMZM2G032049_P01	TRUE	TRUE	gVLHWIAEPAPGVEPLk	95%	n+304 (+304), K+304 (+304)	41.56	25.00
1260	seq=translation; coord=8:21816689..21823022:1; parent_transcript=GRMZM2G032049_T01; parent_gene=GRMZM2G032049	GRMZM2G032049_P01	TRUE	TRUE	gVSSTAINSFIR	95%	n+304 (+304)	42.07	25.71
1261	seq=translation; coord=8:21816689..21823022:1; parent_transcript=GRMZM2G032049_T01; parent_gene=GRMZM2G032049	GRMZM2G032049_P01	TRUE	TRUE	lAEILGPk	95%	n+304 (+304), K+304 (+304)	29.74	25.00
1262	seq=translation; coord=8:21816689..21823022:1; parent_transcript=GRMZM2G032049_T01; parent_gene=GRMZM2G032049	GRMZM2G032049_P01	TRUE	TRUE	lFMSENPAELEDWLGDLNPHSk	95%	n+304 (+304), K+304 (+304)	30.66	25.00
1263	seq=translation; coord=8:21816689..21823022:1; parent_transcript=GRMZM2G032049_T01; parent_gene=GRMZM2G032049	GRMZM2G032049_P01	TRUE	TRUE	lLTLAGLR	87%	n+304 (+304)	25.25	25.00
1264	seq=translation; coord=8:21816689..21823022:1; parent_transcript=GRMZM2G032049_T01; parent_gene=GRMZM2G032049	GRMZM2G032049_P01	TRUE	TRUE	lNISNTVMSk	93%	n+304 (+304), K+304 (+304)	28.15	26.59
1265	seq=translation; coord=8:21816689..21823022:1; parent_transcript=GRMZM2G032049_T01; parent_gene=GRMZM2G032049	GRMZM2G032049_P01	TRUE	TRUE	mWPDASDTDASSHYk	95%	n+304 (+304), K+304 (+304)	37.68	25.00
1266	seq=translation; coord=8:21816689..21823022:1; parent_transcript=GRMZM2G032049_T01; parent_gene=GRMZM2G032049	GRMZM2G032049_P01	TRUE	TRUE	nMSDLIAYR	95%	n+304 (+304)	35.68	25.00
1267	seq=translation; coord=8:21816689..21823022:1; parent_transcript=GRMZM2G032049_T01; parent_gene=GRMZM2G032049	GRMZM2G032049_P01	TRUE	TRUE	sTVTDVLEENMEAIk	91%	n+304 (+304), K+304 (+304)	26.63	25.80

1268	seq=translation; coord=8:21816689..21823022:1; parent_transcript=GRMZM2G032049_T01; parent_gene=GRMZM2G032049	GRMZM2G032049_P01	TRUE	TRUE	vVITNLEEGk	95%	n+304 (+304), K+304 (+304)	39.77	25.00
1269	seq=translation; coord=8:21816689..21823022:1; parent_transcript=GRMZM2G032049_T01; parent_gene=GRMZM2G032049	GRMZM2G032049_P01	TRUE	TRUE	wVDGWDDPR	87%	n+304 (+304)	25.18	25.00
1270	seq=translation; coord=1:299988513..299993142:-1; parent_transcript=GRMZM2G434173_T01; parent_gene=GRMZM2G434173	GRMZM2G434173_P01	TRUE	TRUE	aIELADPMENAGASLIR	95%	n+304 (+304)	75.37	25.00
1271	seq=translation; coord=1:299988513..299993142:-1; parent_transcript=GRMZM2G434173_T01; parent_gene=GRMZM2G434173	GRMZM2G434173_P01	TRUE	TRUE	aLLQDIAIVTGAEYQSk	95%	n+304 (+304), K+304 (+304)	61.85	25.16
1272	seq=translation; coord=1:299988513..299993142:-1; parent_transcript=GRMZM2G434173_T01; parent_gene=GRMZM2G434173	GRMZM2G434173_P01	TRUE	TRUE	aLVAPAALIAHNAGVEGEVIVDk	95%	n+304 (+304), K+304 (+304)	45.39	25.00
1273	seq=translation; coord=1:299988513..299993142:-1; parent_transcript=GRMZM2G434173_T01; parent_gene=GRMZM2G434173	GRMZM2G434173_P01	TRUE	TRUE	aPLLIIAEDVSGEALATLVINk	95%	n+304 (+304), K+304 (+304)	40.17	25.00
1274	seq=translation; coord=1:299988513..299993142:-1; parent_transcript=GRMZM2G434173_T01; parent_gene=GRMZM2G434173	GRMZM2G434173_P01	TRUE	TRUE	aVAAISAGNDEFVGTMIAEAIDk	95%	n+304 (+304), K+304 (+304)	74.68	25.08
1275	seq=translation; coord=1:299988513..299993142:-1; parent_transcript=GRMZM2G434173_T01; parent_gene=GRMZM2G434173	GRMZM2G434173_P01	TRUE	TRUE	dLGLLVEDTTVEQLGIAR	95%	n+304 (+304)	85.59	25.30
1276	seq=translation; coord=1:299988513..299993142:-1; parent_transcript=GRMZM2G434173_T01; parent_gene=GRMZM2G434173	GRMZM2G434173_P01	TRUE	TRUE	eIAFDQGSR	95%	n+304 (+304)	29.93	25.00
1277	seq=translation; coord=1:299988513..299993142:-1; parent_transcript=GRMZM2G434173_T01; parent_gene=GRMZM2G434173	GRMZM2G434173_P01	TRUE	TRUE	eIIPLLEQTTQLR	95%	n+304 (+304)	70.90	25.00
1278	seq=translation; coord=1:299988513..299993142:-1; parent_transcript=GRMZM2G434173_T01; parent_gene=GRMZM2G434173	GRMZM2G434173_P01	TRUE	TRUE	eTLDDPEER	95%	n+304 (+304)	35.83	25.00
1279	seq=translation; coord=1:299988513..299993142:-1; parent_transcript=GRMZM2G434173_T01; parent_gene=GRMZM2G434173	GRMZM2G434173_P01	TRUE	TRUE	lAAAVGVTLGPR	95%	n+304 (+304)	39.23	25.21
1280	seq=translation; coord=1:299988513..299993142:-1; parent_transcript=GRMZM2G434173_T01; parent_gene=GRMZM2G434173	GRMZM2G434173_P01	TRUE	TRUE	lGADIIQk	89%	n+304 (+304), K+304 (+304)	25.79	25.00
1281	seq=translation; coord=1:299988513..299993142:-1; parent_transcript=GRMZM2G434173_T01; parent_gene=GRMZM2G434173	GRMZM2G434173_P01	TRUE	TRUE	lGMLSITSGANPVSVk	95%	n+304 (+304), K+304 (+304)	41.62	25.29
1282	seq=translation; coord=1:299988513..299993142:-1; parent_transcript=GRMZM2G434173_T01; parent_gene=GRMZM2G434173	GRMZM2G434173_P01	TRUE	TRUE	vGASTEAELEDR	95%	n+304 (+304)	68.29	25.00
1283	seq=translation; coord=5:65023010..65024952:-1; parent_transcript=GRMZM2G005771_T01; parent_gene=GRMZM2G005771	GRMZM2G005771_P01,GRMZM2G005771_P02	TRUE	TRUE	aLQPIFQIYGR	95%	n+304 (+304)	34.57	25.40
1284	seq=translation; coord=5:65023010..65024952:-1; parent_transcript=GRMZM2G005771_T01; parent_gene=GRMZM2G005771	GRMZM2G005771_P01,GRMZM2G005771_P02	TRUE	TRUE	hVPVNIAGTR	95%	n+304 (+304)	43.60	25.00
1285	seq=translation; coord=5:65023010..65024952:-1; parent_transcript=GRMZM2G005771_T01; parent_gene=GRMZM2G005771	GRMZM2G005771_P01,GRMZM2G005771_P02	TRUE	TRUE	icDGEWLYADPDGILVSR	95%	n+304 (+304), Carbamidomethyl (+57)	41.65	25.00

1286	seq=translation; coord=5:65023010..65024952:-1; parent_transcript=GRMZM2G005771_T01; parent_gene=GRMZM2G005771	GRMZM2G005771_P01,GRMZM2G005771_P02	TRUE	TRUE	qVFAGPIVTLk	95%	n+304 (+304), K+304 (+304)	44.43	25.00
1287	seq=translation; coord=5:65023010..65024952:-1; parent_transcript=GRMZM2G005771_T01; parent_gene=GRMZM2G005771	GRMZM2G005771_P01,GRMZM2G005771_P02	TRUE	TRUE	vLVVDGGGSMR	95%	n+304 (+304)	39.42	25.00
1288	seq=translation; coord=5:65023010..65024952:-1; parent_transcript=GRMZM2G005771_T01; parent_gene=GRMZM2G005771	GRMZM2G005771_P01,GRMZM2G005771_P02	TRUE	TRUE	vYEDNVLVR	95%	n+304 (+304)	32.44	25.00
1289	seq=translation; coord=10:1725712..1731899:-1; parent_transcript=GRMZM2G130062_T01; parent_gene=GRMZM2G130062	GRMZM2G130062_P01	TRUE	TRUE	aGATTLNIPDTVGYNLPYEFGk	95%	n+304 (+304), K+304 (+304)	41.27	25.61
1290	seq=translation; coord=10:1725712..1731899:-1; parent_transcript=GRMZM2G130062_T01; parent_gene=GRMZM2G130062	GRMZM2G130062_P01	TRUE	TRUE	aNTPGIEk	92%	n+304 (+304), K+304 (+304)	27.73	25.00
1291	seq=translation; coord=10:1725712..1731899:-1; parent_transcript=GRMZM2G130062_T01; parent_gene=GRMZM2G130062	GRMZM2G130062_P01	TRUE	TRUE	eFLYHILGEVIk	95%	n+304 (+304), K+304 (+304)	32.46	25.00
1292	seq=translation; coord=10:1725712..1731899:-1; parent_transcript=GRMZM2G130062_T01; parent_gene=GRMZM2G130062	GRMZM2G130062_P01	TRUE	TRUE	eLLDGLYTGIDSR	95%	n+304 (+304)	39.03	25.00
1293	seq=translation; coord=10:1725712..1731899:-1; parent_transcript=GRMZM2G130062_T01; parent_gene=GRMZM2G130062	GRMZM2G130062_P01	TRUE	TRUE	eYSMTSVTEGIDAIATTR	95%	n+304 (+304)	90.58	25.00
1294	seq=translation; coord=10:1725712..1731899:-1; parent_transcript=GRMZM2G130062_T01; parent_gene=GRMZM2G130062	GRMZM2G130062_P01	TRUE	TRUE	eySMTSVTEGIDAIATTR	95%	n+304 (+304), iTRAQ8plex (+304)	65.44	25.20
1295	seq=translation; coord=10:1725712..1731899:-1; parent_transcript=GRMZM2G130062_T01; parent_gene=GRMZM2G130062	GRMZM2G130062_P01	TRUE	TRUE	sFSGSGASMDIVVSSVR	92%	n+304 (+304)	26.12	25.00
1296	seq=translation; coord=10:1725712..1731899:-1; parent_transcript=GRMZM2G130062_T01; parent_gene=GRMZM2G130062	GRMZM2G130062_P01	TRUE	TRUE	sLGcTDVEFSPEDAGR	95%	n+304 (+304), Carbamidomethyl (+57)	59.75	25.00
1297	seq=translation; coord=10:1725712..1731899:-1; parent_transcript=GRMZM2G130062_T01; parent_gene=GRMZM2G130062	GRMZM2G130062_P01	TRUE	TRUE	vVVTGDVSNNAk	95%	n+304 (+304), K+304 (+304)	47.39	26.04
1298	seq=translation; coord=3:31898226..31901626:-1; parent_transcript=GRMZM2G120304_T01; parent_gene=GRMZM2G120304	GRMZM2G120304_P01,GRMZM2G120304_P02	TRUE	TRUE	aGEDQYSLASR	95%	n+304 (+304)	41.13	25.00
1299	seq=translation; coord=3:31898226..31901626:-1; parent_transcript=GRMZM2G120304_T01; parent_gene=GRMZM2G120304	GRMZM2G120304_P01,GRMZM2G120304_P02	TRUE	TRUE	dGTALVLWEWcEGDNQR	95%	n+304 (+304), Carbamidomethyl (+57)	75.54	25.00
1300	seq=translation; coord=3:31898226..31901626:-1; parent_transcript=GRMZM2G120304_T01; parent_gene=GRMZM2G120304	GRMZM2G120304_P01,GRMZM2G120304_P02	TRUE	TRUE	dVGDGFR	86%	n+304 (+304)	25.48	25.00
1301	seq=translation; coord=3:31898226..31901626:-1; parent_transcript=GRMZM2G120304_T01; parent_gene=GRMZM2G120304	GRMZM2G120304_P01,GRMZM2G120304_P02	TRUE	TRUE	gPPPPVYGGYGQPPPPDPYGR	93%	n+304 (+304)	27.39	25.00
1302	seq=translation; coord=3:31898226..31901626:-1; parent_transcript=GRMZM2G120304_T01; parent_gene=GRMZM2G120304	GRMZM2G120304_P01,GRMZM2G120304_P02	TRUE	TRUE	hNPDSLDESVLWTESR	95%	n+304 (+304)	95.75	25.00
1303	seq=translation; coord=3:31898226..31901626:-1; parent_transcript=GRMZM2G120304_T01; parent_gene=GRMZM2G120304	GRMZM2G120304_P01,GRMZM2G120304_P02	TRUE	TRUE	rGPPPPVYGGYGQPPPPDPYGR	95%	n+304 (+304)	44.22	25.00

1304	seq=translation; coord=4:20543360..20550099:-1; parent_transcript=GRMZM2G101446_T01; parent_gene=GRMZM2G101446	GRMZM2G101446_P01,GRMZM2G101446_P02	TRUE	TRUE	aGSDDVGAASANSTAASGEDLk	95%	n+304 (+304), K+304 (+304)	80.87	25.00
1305	seq=translation; coord=4:20543360..20550099:-1; parent_transcript=GRMZM2G101446_T01; parent_gene=GRMZM2G101446	GRMZM2G101446_P01,GRMZM2G101446_P02	TRUE	TRUE	gLLDLTcQTVADMIk	95%	n+304 (+304), Carbamidomethyl (+57), K+304 (+304)	45.03	26.27
1306	seq=translation; coord=4:20543360..20550099:-1; parent_transcript=GRMZM2G101446_T01; parent_gene=GRMZM2G101446	GRMZM2G101446_P01,GRMZM2G101446_P02	TRUE	TRUE	nDFTPEEEEEIR	95%	n+304 (+304)	52.45	25.00
1307	seq=translation; coord=4:20543360..20550099:-1; parent_transcript=GRMZM2G101446_T01; parent_gene=GRMZM2G101446	GRMZM2G101446_P01,GRMZM2G101446_P02	TRUE	TRUE	nDFTPEEEEEIRR	94%	n+304 (+304)	28.44	25.00
1308	seq=translation; coord=4:20543360..20550099:-1; parent_transcript=GRMZM2G101446_T01; parent_gene=GRMZM2G101446	GRMZM2G101446_P01,GRMZM2G101446_P02	TRUE	TRUE	nWDADFVk	86%	n+304 (+304), K+304 (+304)	25.08	25.00
1309	seq=translation; coord=4:20543360..20550099:-1; parent_transcript=GRMZM2G101446_T01; parent_gene=GRMZM2G101446	GRMZM2G101446_P01,GRMZM2G101446_P02	TRUE	TRUE	sSDGEEFEVEEAVAMESQTIR	95%	n+304 (+304)	57.99	25.00
1310	seq=translation; coord=4:20543360..20550099:-1; parent_transcript=GRMZM2G101446_T01; parent_gene=GRMZM2G101446	GRMZM2G101446_P01,GRMZM2G101446_P02	TRUE	TRUE	vDQATLFDLILAANYLNIk	95%	n+304 (+304), K+304 (+304)	40.80	25.00
1311	seq=translation; coord=4:20543360..20550099:-1; parent_transcript=GRMZM2G101446_T01; parent_gene=GRMZM2G101446	GRMZM2G101446_P01,GRMZM2G101446_P02	TRUE	TRUE	vIEYcNk	94%	n+304 (+304), Carbamidomethyl (+57), K+304 (+304)	30.97	25.66
1312	seq=translation; coord=1:297016125..297019051:-1; parent_transcript=GRMZM2G352415_T01; parent_gene=GRMZM2G352415	GRMZM2G352415_P01,GRMZM2G352415_P02, GRMZM2G352415_P03	TRUE	TRUE	aDLVVDVLIk	95%	n+304 (+304), K+304 (+304)	43.62	25.00
1313	seq=translation; coord=1:297016125..297019051:-1; parent_transcript=GRMZM2G352415_T01; parent_gene=GRMZM2G352415	GRMZM2G352415_P01,GRMZM2G352415_P02, GRMZM2G352415_P03	TRUE	TRUE	dFGSAVWDMIR	95%	n+304 (+304)	49.44	25.00
1314	seq=translation; coord=1:297016125..297019051:-1; parent_transcript=GRMZM2G352415_T01; parent_gene=GRMZM2G352415	GRMZM2G352415_P01,GRMZM2G352415_P02, GRMZM2G352415_P03	TRUE	TRUE	dIQPGSIIPYLVR	93%	n+304 (+304)	29.27	25.00
1315	seq=translation; coord=1:297016125..297019051:-1; parent_transcript=GRMZM2G352415_T01; parent_gene=GRMZM2G352415	GRMZM2G352415_P01,GRMZM2G352415_P02, GRMZM2G352415_P03	TRUE	TRUE	gTGYTIk	86%	n+304 (+304), K+304 (+304)	25.95	25.48
1316	seq=translation; coord=1:297016125..297019051:-1; parent_transcript=GRMZM2G352415_T01; parent_gene=GRMZM2G352415	GRMZM2G352415_P01,GRMZM2G352415_P02, GRMZM2G352415_P03	TRUE	TRUE	iDVDTPFGNMk	95%	n+304 (+304), K+304 (+304)	43.07	25.20
1317	seq=translation; coord=1:297016125..297019051:-1; parent_transcript=GRMZM2G352415_T01; parent_gene=GRMZM2G352415	GRMZM2G352415_P01,GRMZM2G352415_P02, GRMZM2G352415_P03	TRUE	TRUE	iPVSLVFDDIk	94%	n+304 (+304), K+304 (+304)	28.34	25.00
1318	seq=translation; coord=1:297016125..297019051:-1; parent_transcript=GRMZM2G352415_T01; parent_gene=GRMZM2G352415	GRMZM2G352415_P01,GRMZM2G352415_P02, GRMZM2G352415_P03	TRUE	TRUE	nPNPVPIPLVDIDYLIDSDGR	95%	n+304 (+304)	61.58	25.42
1319	seq=translation; coord=1:297016125..297019051:-1; parent_transcript=GRMZM2G352415_T01; parent_gene=GRMZM2G352415	GRMZM2G352415_P01,GRMZM2G352415_P02, GRMZM2G352415_P03	TRUE	TRUE	vVLLVDVPIIGR	95%	n+304 (+304)	38.25	25.00
1320	seq=translation; coord=3:146522696..146524904:-1; parent_transcript=GRMZM2G103342_T01; parent_gene=GRMZM2G103342	GRMZM2G103342_P01	TRUE	TRUE	eFLASAVR	91%	n+304 (+304)	28.35	25.08
1321	seq=translation; coord=3:146522696..146524904:-1; parent_transcript=GRMZM2G103342_T01; parent_gene=GRMZM2G103342	GRMZM2G103342_P01	TRUE	TRUE	eSVALGGGPAYk	95%	n+304 (+304), K+304 (+304)	54.86	26.28

1322	seq=translation; coord=3:146522696..146524904:-1; parent_transcript=GRMZM2G103342_T01; parent_gene=GRMZM2G103342	GRMZM2G103342_P01	TRUE	TRUE	iNLDVTDLVALSGGHTVGIAHcGSFDNR	95%	n+304 (+304), Carbamidomethyl (+57)	37.84	25.00
1323	seq=translation; coord=3:146522696..146524904:-1; parent_transcript=GRMZM2G103342_T01; parent_gene=GRMZM2G103342	GRMZM2G103342_P01	TRUE	TRUE	lFPTQDPTLNk	95%	n+304 (+304), K+304 (+304)	45.40	25.97
1324	seq=translation; coord=3:146522696..146524904:-1; parent_transcript=GRMZM2G103342_T01; parent_gene=GRMZM2G103342	GRMZM2G103342_P01	TRUE	TRUE	mGQVNVLTGSQGQVR	95%	n+304 (+304)	64.91	25.77
1325	seq=translation; coord=3:146522696..146524904:-1; parent_transcript=GRMZM2G103342_T01; parent_gene=GRMZM2G103342	GRMZM2G103342_P01	TRUE	TRUE	qNVGLAAALIR	95%	n+304 (+304)	68.15	25.00
1326	seq=translation; coord=3:146522696..146524904:-1; parent_transcript=GRMZM2G103342_T01; parent_gene=GRMZM2G103342	GRMZM2G103342_P01	TRUE	TRUE	vPTLLSFLAk	95%	n+304 (+304), K+304 (+304)	56.05	25.00
1327	seq=translation; coord=3:146522696..146524904:-1; parent_transcript=GRMZM2G103342_T01; parent_gene=GRMZM2G103342	GRMZM2G103342_P01	TRUE	TRUE	vVScADIVALAAR	95%	n+304 (+304), Carbamidomethyl (+57)	54.62	25.68
1328	seq=translation; coord=3:146522696..146524904:-1; parent_transcript=GRMZM2G103342_T01; parent_gene=GRMZM2G103342	GRMZM2G103342_P01	TRUE	TRUE	yPPLAPGLSFDFYk	95%	n+304 (+304), K+304 (+304)	52.28	25.87
1329	seq=translation; coord=3:146522696..146524904:-1; parent_transcript=GRMZM2G103342_T01; parent_gene=GRMZM2G103342	GRMZM2G103342_P01	TRUE	TRUE	yYVDLLNR	91%	n+304 (+304)	28.95	25.69
1330	seq=translation; coord=2:16219232..16221297:1; parent_transcript=GRMZM2G003752_T01; parent_gene=GRMZM2G003752	GRMZM2G003752_P01	TRUE	TRUE	aLTLFAPNDDAFk	95%	n+304 (+304), K+304 (+304)	45.09	26.10
1331	seq=translation; coord=2:16219232..16221297:1; parent_transcript=GRMZM2G003752_T01; parent_gene=GRMZM2G003752	GRMZM2G003752_P01	TRUE	TRUE	dLPDLSk	91%	n+304 (+304), K+304 (+304)	28.44	25.11
1332	seq=translation; coord=2:16219232..16221297:1; parent_transcript=GRMZM2G003752_T01; parent_gene=GRMZM2G003752	GRMZM2G003752_P01	TRUE	TRUE	iPTLASTAAGk	95%	n+304 (+304), K+304 (+304)	68.54	26.30
1333	seq=translation; coord=2:16219232..16221297:1; parent_transcript=GRMZM2G003752_T01; parent_gene=GRMZM2G003752	GRMZM2G003752_P01	TRUE	TRUE	lIVSSGVVk	95%	n+304 (+304), K+304 (+304)	32.20	25.00
1334	seq=translation; coord=2:16219232..16221297:1; parent_transcript=GRMZM2G003752_T01; parent_gene=GRMZM2G003752	GRMZM2G003752_P01	TRUE	TRUE	lLTLLDYFDEk	95%	n+304 (+304), K+304 (+304)	46.41	25.44
1335	seq=translation; coord=2:16219232..16221297:1; parent_transcript=GRMZM2G003752_T01; parent_gene=GRMZM2G003752	GRMZM2G003752_P01	TRUE	TRUE	lLTLLDYFDEkk	95%	n+304 (+304), K+304 (+304), K+304 (+304)	33.18	25.00
1336	seq=translation; coord=2:16219232..16221297:1; parent_transcript=GRMZM2G003752_T01; parent_gene=GRMZM2G003752	GRMZM2G003752_P01	TRUE	TRUE	lTSADLVALLQYHALPQYAPk	95%	n+304 (+304), K+304 (+304)	50.20	25.00
1337	seq=translation; coord=2:16219232..16221297:1; parent_transcript=GRMZM2G003752_T01; parent_gene=GRMZM2G003752	GRMZM2G003752_P01	TRUE	TRUE	vAFASAAPGAk	95%	n+304 (+304), K+304 (+304)	38.13	25.38
1338	seq=translation; coord=2:16219232..16221297:1; parent_transcript=GRMZM2G003752_T01; parent_gene=GRMZM2G003752	GRMZM2G003752_P01	TRUE	TRUE	yDLAVASSGDEVTLDTGVDk	95%	n+304 (+304), K+304 (+304)	78.65	25.00
1339	seq=translation; coord=6:160621048..160624717:1; parent_transcript=GRMZM2G360681_T01; parent_gene=GRMZM2G360681	GRMZM2G360681_P01	TRUE	TRUE	aHVAVFNTLLQVLDDGR	93%	n+304 (+304)	29.59	26.53

1340	seq=translation; coord=6:160621048..160624717:1; parent_transcript=GRMZM2G360681_T01; parent_gene=GRMZM2G360681	GRMZM2G360681_P01	TRUE	TRUE	aLAEQLFDDENLLVR	95%	n+304 (+304)	44.94	25.16
1341	seq=translation; coord=6:160621048..160624717:1; parent_transcript=GRMZM2G360681_T01; parent_gene=GRMZM2G360681	GRMZM2G360681_P01	TRUE	TRUE	dLVMQEVR	95%	n+304 (+304)	32.27	25.48
1342	seq=translation; coord=6:160621048..160624717:1; parent_transcript=GRMZM2G360681_T01; parent_gene=GRMZM2G360681	GRMZM2G360681_P01	TRUE	TRUE	eELQFTLQEAER	95%	n+304 (+304)	47.89	25.00
1343	seq=translation; coord=6:160621048..160624717:1; parent_transcript=GRMZM2G360681_T01; parent_gene=GRMZM2G360681	GRMZM2G360681_P01	TRUE	TRUE	gDSHLAVDQLLLGLLEDSQISDcLk	95%	n+304 (+304), Carbamidomethyl (+57), K+304 (+304)	30.09	25.00
1344	seq=translation; coord=6:160621048..160624717:1; parent_transcript=GRMZM2G360681_T01; parent_gene=GRMZM2G360681	GRMZM2G360681_P01	TRUE	TRUE	gDVPSNLLDVR	93%	n+304 (+304)	28.72	25.42
1345	seq=translation; coord=6:160621048..160624717:1; parent_transcript=GRMZM2G360681_T01; parent_gene=GRMZM2G360681	GRMZM2G360681_P01	TRUE	TRUE	iDMSEYMEQHSVAR	95%	n+304 (+304)	65.11	25.00
1346	seq=translation; coord=6:160621048..160624717:1; parent_transcript=GRMZM2G360681_T01; parent_gene=GRMZM2G360681	GRMZM2G360681_P01	TRUE	TRUE	lDEIVIFDPLSHEQLR	95%	n+304 (+304)	52.58	25.66
1347	seq=translation; coord=6:160621048..160624717:1; parent_transcript=GRMZM2G360681_T01; parent_gene=GRMZM2G360681	GRMZM2G360681_P01	TRUE	TRUE	lIALDMGALVAGAk	95%	n+304 (+304), K+304 (+304)	32.71	25.00
1348	seq=translation; coord=6:160621048..160624717:1; parent_transcript=GRMZM2G360681_T01; parent_gene=GRMZM2G360681	GRMZM2G360681_P01	TRUE	TRUE	qAITGASGGDGAAGDSFER	95%	n+304 (+304)	96.05	25.00
1349	seq=translation; coord=6:160621048..160624717:1; parent_transcript=GRMZM2G360681_T01; parent_gene=GRMZM2G360681	GRMZM2G360681_P01	TRUE	TRUE	rEELQFTLQEAER	95%	n+304 (+304)	31.15	25.00
1350	seq=translation; coord=6:160621048..160624717:1; parent_transcript=GRMZM2G360681_T01; parent_gene=GRMZM2G360681	GRMZM2G360681_P01	TRUE	TRUE	tEGSMDAANLFkPMLAR	95%	n+304 (+304), K+304 (+304)	33.55	25.76
1351	seq=translation; coord=6:160621048..160624717:1; parent_transcript=GRMZM2G360681_T01; parent_gene=GRMZM2G360681	GRMZM2G360681_P01	TRUE	TRUE	vQLDSQPEEIDNLER	95%	n+304 (+304)	64.81	25.00
1352	seq=translation; coord=6:160621048..160624717:1; parent_transcript=GRMZM2G360681_T01; parent_gene=GRMZM2G360681	GRMZM2G360681_P01	TRUE	TRUE	vVGQTEAVSAVAEAVLR	95%	n+304 (+304)	56.32	26.07
1353	seq=translation; coord=6:160621048..160624717:1; parent_transcript=GRMZM2G360681_T01; parent_gene=GRMZM2G360681	GRMZM2G360681_P01	TRUE	TRUE	yGALQEIDAAISk	95%	n+304 (+304), K+304 (+304)	61.86	25.48
1354	seq=translation; coord=9:11079860..11085004:-1; parent_transcript=GRMZM2G127609_T01; parent_gene=GRMZM2G127609	GRMZM2G127609_P01,GRMZM2G127609_P02, GRMZM2G127609_P03,GRMZM2G127609_P04, GRMZM2G127609_P06,GRMZM2G127609_P07, GRMZM2G127609_P08	TRUE	TRUE	aATVVTPk	94%	n+304 (+304), K+304 (+304)	29.35	25.00
1355	seq=translation; coord=9:11079860..11085004:-1; parent_transcript=GRMZM2G127609_T01; parent_gene=GRMZM2G127609	GRMZM2G127609_P01,GRMZM2G127609_P02, GRMZM2G127609_P03,GRMZM2G127609_P04, GRMZM2G127609_P06,GRMZM2G127609_P07, GRMZM2G127609_P08	TRUE	TRUE	eDDIIGILETDDVk	95%	n+304 (+304), K+304 (+304)	47.04	26.21
1356	seq=translation; coord=9:11079860..11085004:-1; parent_transcript=GRMZM2G127609_T01; parent_gene=GRMZM2G127609	GRMZM2G127609_P01,GRMZM2G127609_P02, GRMZM2G127609_P03,GRMZM2G127609_P04, GRMZM2G127609_P06,GRMZM2G127609_P07, GRMZM2G127609_P08	TRUE	TRUE	gADGTAYIVLR	95%	n+304 (+304)	47.24	25.00

1357	seq=translation; coord=9:11079860..11085004:-1; parent_transcript=GRMZM2G127609_T01; parent_gene=GRMZM2G127609	GRMZM2G127609_P01,GRMZM2G127609_P02, GRMZM2G127609_P03,GRMZM2G127609_P04, GRMZM2G127609_P06,GRMZM2G127609_P07, GRMZM2G127609_P08	TRUE	TRUE	iAVDIETGAQVVYSk	95%	n+304 (+304), K+304 (+304)	51.63	25.00
1358	seq=translation; coord=9:11079860..11085004:-1; parent_transcript=GRMZM2G127609_T01; parent_gene=GRMZM2G127609	GRMZM2G127609_P01,GRMZM2G127609_P02, GRMZM2G127609_P03,GRMZM2G127609_P04, GRMZM2G127609_P06,GRMZM2G127609_P07, GRMZM2G127609_P08	TRUE	TRUE	qPLSVSAGSTVLYSk	91%	n+304 (+304), K+304 (+304)	25.93	25.00
1359	seq=translation; coord=9:11079860..11085004:-1; parent_transcript=GRMZM2G127609_T01; parent_gene=GRMZM2G127609	GRMZM2G127609_P01,GRMZM2G127609_P02, GRMZM2G127609_P03,GRMZM2G127609_P04, GRMZM2G127609_P06,GRMZM2G127609_P07, GRMZM2G127609_P08	TRUE	TRUE	qPLSVSAGSTVLYSk	95%	Pyro-cmC (-17), n+304 (+304), K+304 (+304)	31.29	25.00
1360	seq=translation; coord=9:11079860..11085004:-1; parent_transcript=GRMZM2G127609_T01; parent_gene=GRMZM2G127609	GRMZM2G127609_P01,GRMZM2G127609_P02, GRMZM2G127609_P03,GRMZM2G127609_P04, GRMZM2G127609_P06,GRMZM2G127609_P07, GRMZM2G127609_P08	TRUE	TRUE	rQPLSVSAGSTVLYSk	94%	n+304 (+304), K+304 (+304)	25.69	25.00
1361	seq=translation; coord=9:11079860..11085004:-1; parent_transcript=GRMZM2G127609_T01; parent_gene=GRMZM2G127609	GRMZM2G127609_P01,GRMZM2G127609_P02, GRMZM2G127609_P03,GRMZM2G127609_P04, GRMZM2G127609_P06,GRMZM2G127609_P07, GRMZM2G127609_P08	TRUE	TRUE	tPGGLILTETTk	95%	n+304 (+304), K+304 (+304)	37.77	25.00
1362	seq=translation; coord=9:11079860..11085004:-1; parent_transcript=GRMZM2G127609_T01; parent_gene=GRMZM2G127609	GRMZM2G127609_P01,GRMZM2G127609_P02, GRMZM2G127609_P03,GRMZM2G127609_P04, GRMZM2G127609_P06,GRMZM2G127609_P07, GRMZM2G127609_P08	TRUE	TRUE	tVGGILLPSTAQTkPQGGEVVAVGAGR	95%	n+304 (+304), K+304 (+304)	38.05	25.00
1363	seq=translation; coord=9:11079860..11085004:-1; parent_transcript=GRMZM2G127609_T01; parent_gene=GRMZM2G127609	GRMZM2G127609_P01,GRMZM2G127609_P02, GRMZM2G127609_P03,GRMZM2G127609_P04, GRMZM2G127609_P06,GRMZM2G127609_P07, GRMZM2G127609_P08	TRUE	TRUE	yAGTEVEFNDSk	95%	n+304 (+304), K+304 (+304)	28.78	25.00
1364	seq=translation; coord=9:11079860..11085004:-1; parent_transcript=GRMZM2G127609_T01; parent_gene=GRMZM2G127609	GRMZM2G127609_P01,GRMZM2G127609_P02, GRMZM2G127609_P03,GRMZM2G127609_P04, GRMZM2G127609_P06,GRMZM2G127609_P07, GRMZM2G127609_P08	TRUE	TRUE	yAGTEVEFNDSk	94%	n+304 (+304), iTRAQ8plex (+304), K+304 (+304)	26.53	25.91
1365	seq=translation; coord=2:165615333..165620418:-1; parent_transcript=GRMZM2G084881_T01; parent_gene=GRMZM2G084881	GRMZM2G084881_P01	TRUE	TRUE	dGLQFASk	95%	n+304 (+304), K+304 (+304)	34.93	26.16
1366	seq=translation; coord=2:165615333..165620418:-1; parent_transcript=GRMZM2G084881_T01; parent_gene=GRMZM2G084881	GRMZM2G084881_P01	TRUE	TRUE	eSGEPVPEYDYLPyFYSR	95%	n+304 (+304), iTRAQ8plex (+304)	31.72	25.00
1367	seq=translation; coord=2:165615333..165620418:-1; parent_transcript=GRMZM2G084881_T01; parent_gene=GRMZM2G084881	GRMZM2G084881_P01	TRUE	TRUE	lPGFYVcVGSGGEk	95%	n+304 (+304), Carbamidomethyl (+57), K+304 (+304)	49.17	25.00
1368	seq=translation; coord=2:165615333..165620418:-1; parent_transcript=GRMZM2G084881_T01; parent_gene=GRMZM2G084881	GRMZM2G084881_P01	TRUE	TRUE	lTDFGTQGADSNNILYLR	95%	n+304 (+304)	32.60	25.00
1369	seq=translation; coord=2:165615333..165620418:-1; parent_transcript=GRMZM2G084881_T01; parent_gene=GRMZM2G084881	GRMZM2G084881_P01	TRUE	TRUE	lVAAIQAk	95%	n+304 (+304), K+304 (+304)	40.47	25.45
1370	seq=translation; coord=2:165615333..165620418:-1; parent_transcript=GRMZM2G084881_T01; parent_gene=GRMZM2G084881	GRMZM2G084881_P01	TRUE	TRUE	qGVkPGELAIISk	86%	Pyro-cmC (-17), n+304 (+304), K+304 (+304), K+304 (+304)	25.37	25.00
1371	seq=translation; coord=2:165615333..165620418:-1; parent_transcript=GRMZM2G084881_T01; parent_gene=GRMZM2G084881	GRMZM2G084881_P01	TRUE	TRUE	sFDLAWQFYGDNVGETILFGDSDPTSSkPk	94%	n+304 (+304), K+304 (+304), K+304 (+304)	25.85	25.00

1372	seq=translation; coord=2:165615333..165620418:-1; parent_transcript=GRMZM2G084881_T01; parent_gene=GRMZM2G084881	GRMZM2G084881_P01	TRUE	TRUE	tLTSAAGANFTYEILLIATGSSVIk	95%	n+304 (+304), K+304 (+304)	34.60	25.00
1373	seq=translation; coord=2:165615333..165620418:-1; parent_transcript=GRMZM2G084881_T01; parent_gene=GRMZM2G084881	GRMZM2G084881_P01	TRUE	TRUE	tQPPVANLEELk	95%	n+304 (+304), K+304 (+304)	47.43	25.00
1374	seq=translation; coord=2:165615333..165620418:-1; parent_transcript=GRMZM2G084881_T01; parent_gene=GRMZM2G084881	GRMZM2G084881_P01	TRUE	TRUE	vLGAFLEGGSPDENk	95%	n+304 (+304), K+304 (+304)	57.77	25.26
1375	seq=translation; coord=8:158089079..158099475:-1; parent_transcript=GRMZM2G180988_T02; parent_gene=GRMZM2G180988	GRMZM2G180988_P02,GRMZM2G180988_P03	TRUE	TRUE	aAAVAALSSVLTAEQSGSSENLR	95%	n+304 (+304)	71.36	25.05
1376	seq=translation; coord=8:158089079..158099475:-1; parent_transcript=GRMZM2G180988_T02; parent_gene=GRMZM2G180988	GRMZM2G180988_P02,GRMZM2G180988_P03	TRUE	TRUE	dAAVDGAPSDTDGAVAETR	95%	n+304 (+304)	83.74	25.00
1377	seq=translation; coord=8:158089079..158099475:-1; parent_transcript=GRMZM2G180988_T02; parent_gene=GRMZM2G180988	GRMZM2G180988_P02,GRMZM2G180988_P03	TRUE	TRUE	eEETTENVGEATFSYDR	95%	n+304 (+304)	102.32	25.00
1378	seq=translation; coord=8:158089079..158099475:-1; parent_transcript=GRMZM2G180988_T02; parent_gene=GRMZM2G180988	GRMZM2G180988_P02,GRMZM2G180988_P03	TRUE	TRUE	ePPQFVALFQPMIILk	95%	n+304 (+304), K+304 (+304)	36.70	25.00
1379	seq=translation; coord=8:158089079..158099475:-1; parent_transcript=GRMZM2G180988_T02; parent_gene=GRMZM2G180988	GRMZM2G180988_P02,GRMZM2G180988_P03	TRUE	TRUE	gATGETYTTEGIALIR	95%	n+304 (+304)	72.42	25.00
1380	seq=translation; coord=8:158089079..158099475:-1; parent_transcript=GRMZM2G180988_T02; parent_gene=GRMZM2G180988	GRMZM2G180988_P02,GRMZM2G180988_P03	TRUE	TRUE	sTTPVNEEVPPLLEGGGk	95%	n+304 (+304), K+304 (+304)	29.31	25.08
1381	seq=translation; coord=2:167237699..167240039:1; parent_transcript=GRMZM2G352855_T01; parent_gene=GRMZM2G352855	GRMZM2G352855_P01,GRMZM2G352855_P03, GRMZM2G352855_P04,GRMZM2G352855_P05	TRUE	TRUE	aVILGHSFGGMVALEFVR	95%	n+304 (+304)	50.64	26.05
1382	seq=translation; coord=2:167237699..167240039:1; parent_transcript=GRMZM2G352855_T01; parent_gene=GRMZM2G352855	GRMZM2G352855_P01,GRMZM2G352855_P03, GRMZM2G352855_P04,GRMZM2G352855_P05	TRUE	TRUE	eLMELVEAASER	95%	n+304 (+304)	57.39	25.00
1383	seq=translation; coord=2:167237699..167240039:1; parent_transcript=GRMZM2G352855_T01; parent_gene=GRMZM2G352855	GRMZM2G352855_P01,GRMZM2G352855_P03, GRMZM2G352855_P04,GRMZM2G352855_P05	TRUE	TRUE	iNLISVLAFEk	95%	n+304 (+304), K+304 (+304)	34.11	25.00
1384	seq=translation; coord=2:167237699..167240039:1; parent_transcript=GRMZM2G352855_T01; parent_gene=GRMZM2G352855	GRMZM2G352855_P01,GRMZM2G352855_P03, GRMZM2G352855_P04,GRMZM2G352855_P05	TRUE	TRUE	lQAPLVVTR	95%	n+304 (+304)	36.58	25.98
1385	seq=translation; coord=2:167237699..167240039:1; parent_transcript=GRMZM2G352855_T01; parent_gene=GRMZM2G352855	GRMZM2G352855_P01,GRMZM2G352855_P03, GRMZM2G352855_P04,GRMZM2G352855_P05	TRUE	TRUE	lVLVAPTLPGGFLEPVR	95%	n+304 (+304)	36.30	25.00
1386	seq=translation; coord=2:167237699..167240039:1; parent_transcript=GRMZM2G352855_T01; parent_gene=GRMZM2G352855	GRMZM2G352855_P01,GRMZM2G352855_P03, GRMZM2G352855_P04,GRMZM2G352855_P05	TRUE	TRUE	mGSFAAPMVPMTYISGVGNR	91%	n+304 (+304)	25.77	25.00
1387	seq=translation; coord=2:167237699..167240039:1; parent_transcript=GRMZM2G352855_T01; parent_gene=GRMZM2G352855	GRMZM2G352855_P01,GRMZM2G352855_P03, GRMZM2G352855_P04,GRMZM2G352855_P05	TRUE	TRUE	sFESAIVNFPSPAVFGR	95%	n+304 (+304)	35.41	25.63
1388	seq=translation; coord=2:167237699..167240039:1; parent_transcript=GRMZM2G352855_T01; parent_gene=GRMZM2G352855	GRMZM2G352855_P01,GRMZM2G352855_P03, GRMZM2G352855_P04,GRMZM2G352855_P05	TRUE	TRUE	yAPPSPGQTSEVYSR	95%	n+304 (+304)	58.86	25.00
1389	seq=translation; coord=7:10287294..10291100:1; parent_transcript=GRMZM2G100403_T02; parent_gene=GRMZM2G100403	GRMZM2G100403_P02	TRUE	TRUE	fVIWTQSAFNk	95%	n+304 (+304), K+304 (+304)	51.29	25.87

1390	seq=translation; coord=7:10287294..10291100:1; parent_transcript=GRMZM2G100403_T02; parent_gene=GRMZM2G100403	GRMZM2G100403_P02	TRUE	TRUE	iESVPEMPLVISDSAESIEk	95%	n+304 (+304), K+304 (+304)	48.26	25.95
1391	seq=translation; coord=7:10287294..10291100:1; parent_transcript=GRMZM2G100403_T02; parent_gene=GRMZM2G100403	GRMZM2G100403_P02	TRUE	TRUE	tMVSDSDYTEFENFSk	94%	n+304 (+304), K+304 (+304)	28.72	25.00
1392	seq=translation; coord=1:12514731..12519380:1; parent_transcript=GRMZM2G165998_T01; parent_gene=GRMZM2G165998	GRMZM2G165998_P01,GRMZM2G165998_P03	TRUE	TRUE	dQDDQWIR	87%	n+304 (+304)	25.63	25.00
1393	seq=translation; coord=1:12514731..12519380:1; parent_transcript=GRMZM2G165998_T01; parent_gene=GRMZM2G165998	GRMZM2G165998_P01,GRMZM2G165998_P03	TRUE	TRUE	eEVIQAWYMDDSEEDQR	93%	n+304 (+304)	29.81	25.00
1394	seq=translation; coord=1:12514731..12519380:1; parent_transcript=GRMZM2G165998_T01; parent_gene=GRMZM2G165998	GRMZM2G165998_P01,GRMZM2G165998_P03	TRUE	TRUE	eFIPLDk	90%	n+304 (+304), K+304 (+304)	26.63	25.26
1395	seq=translation; coord=1:12514731..12519380:1; parent_transcript=GRMZM2G165998_T01; parent_gene=GRMZM2G165998	GRMZM2G165998_P01,GRMZM2G165998_P03	TRUE	TRUE	fTLDSDNYIk	95%	n+304 (+304), K+304 (+304)	51.52	25.00
1396	seq=translation; coord=1:12514731..12519380:1; parent_transcript=GRMZM2G165998_T01; parent_gene=GRMZM2G165998	GRMZM2G165998_P01,GRMZM2G165998_P03	TRUE	TRUE	gYSYMDIcDVcPEk	95%	n+304 (+304), Carbamidomethyl (+57), Carbamidomethyl (+57), K+304 (+304)	56.77	25.00
1397	seq=translation; coord=1:12514731..12519380:1; parent_transcript=GRMZM2G165998_T01; parent_gene=GRMZM2G165998	GRMZM2G165998_P01,GRMZM2G165998_P03	TRUE	TRUE	lNADDWENDENLk	95%	n+304 (+304), K+304 (+304)	47.85	25.00
1398	seq=translation; coord=1:12514731..12519380:1; parent_transcript=GRMZM2G165998_T01; parent_gene=GRMZM2G165998	GRMZM2G165998_P01,GRMZM2G165998_P03	TRUE	TRUE	lNADDWENDENLkk	95%	n+304 (+304), K+304 (+304), K+304 (+304)	32.41	25.48
1399	seq=translation; coord=1:12514731..12519380:1; parent_transcript=GRMZM2G165998_T01; parent_gene=GRMZM2G165998	GRMZM2G165998_P01,GRMZM2G165998_P03	TRUE	TRUE	lSELGILSWR	95%	n+304 (+304)	35.66	25.33
1400	seq=translation; coord=1:12514731..12519380:1; parent_transcript=GRMZM2G165998_T01; parent_gene=GRMZM2G165998	GRMZM2G165998_P01,GRMZM2G165998_P03	TRUE	TRUE	nFFEEHLHTDEEIR	95%	n+304 (+304)	38.78	25.00
1401	seq=translation; coord=1:12514731..12519380:1; parent_transcript=GRMZM2G165998_T01; parent_gene=GRMZM2G165998	GRMZM2G165998_P01,GRMZM2G165998_P03	TRUE	TRUE	ycLEGSGYFDVR	95%	n+304 (+304), Carbamidomethyl (+57)	61.86	25.00
1402	seq=translation; coord=9:19393494..19397648:-1; parent_transcript=GRMZM2G140051_T01; parent_gene=GRMZM2G140051	GRMZM2G140051_P01,GRMZM2G140051_P02	TRUE	TRUE	aGLVNALk	95%	n+304 (+304), K+304 (+304)	34.16	25.00
1403	seq=translation; coord=9:19393494..19397648:-1; parent_transcript=GRMZM2G140051_T01; parent_gene=GRMZM2G140051	GRMZM2G140051_P01,GRMZM2G140051_P02	TRUE	TRUE	eTFDTSDLNASLPAAAAALSAEDR	93%	n+304 (+304)	28.07	25.00
1404	seq=translation; coord=9:19393494..19397648:-1; parent_transcript=GRMZM2G140051_T01; parent_gene=GRMZM2G140051	GRMZM2G140051_P01,GRMZM2G140051_P02	TRUE	TRUE	gVPDFWLTAMk	89%	n+304 (+304), K+304 (+304)	28.03	26.11
1405	seq=translation; coord=9:19393494..19397648:-1; parent_transcript=GRMZM2G140051_T01; parent_gene=GRMZM2G140051	GRMZM2G140051_P01,GRMZM2G140051_P02	TRUE	TRUE	lDFFFDTNPFFk	95%	n+304 (+304), K+304 (+304)	40.24	26.04
1406	seq=translation; coord=9:19393494..19397648:-1; parent_transcript=GRMZM2G140051_T01; parent_gene=GRMZM2G140051	GRMZM2G140051_P01,GRMZM2G140051_P02	TRUE	TRUE	lYEPLYTk	95%	n+304 (+304), K+304 (+304)	30.54	25.00

1407	seq=translation; coord=9:19393494..19397648:-1; parent_transcript=GRMZM2G140051_T01; parent_gene=GRMZM2G140051	GRMZM2G140051_P01,GRMZM2G140051_P02	TRUE	TRUE	tNEVLSEEIQER	95%	n+304 (+304)	46.01	25.00
1408	seq=translation; coord=9:19393494..19397648:-1; parent_transcript=GRMZM2G140051_T01; parent_gene=GRMZM2G140051	GRMZM2G140051_P01,GRMZM2G140051_P02	TRUE	TRUE	tYHMVDEDDPILEk	95%	n+304 (+304), K+304 (+304)	40.29	25.00
1409	seq=translation; coord=5:146841857..146847013:-1; parent_transcript=GRMZM2G146677_T01; parent_gene=GRMZM2G146677	GRMZM2G146677_P01	TRUE	TRUE	aGcLSILcEDEMQAVAVk	95%	n+304 (+304), Carbamidomethyl (+57), Carbamidomethyl (+57), K+304 (+304)	59.21	25.00
1410	seq=translation; coord=5:146841857..146847013:-1; parent_transcript=GRMZM2G146677_T01; parent_gene=GRMZM2G146677	GRMZM2G146677_P01	TRUE	TRUE	dPILGVTEAFLADPSSDk	95%	n+304 (+304), K+304 (+304)	39.10	25.95
1411	seq=translation; coord=5:146841857..146847013:-1; parent_transcript=GRMZM2G146677_T01; parent_gene=GRMZM2G146677	GRMZM2G146677_P01	TRUE	TRUE	eISHQFk	91%	n+304 (+304), K+304 (+304)	25.24	25.00
1412	seq=translation; coord=5:146841857..146847013:-1; parent_transcript=GRMZM2G146677_T01; parent_gene=GRMZM2G146677	GRMZM2G146677_P01	TRUE	TRUE	gLDFSGLMNEIk	95%	n+304 (+304), K+304 (+304)	39.41	25.69
1413	seq=translation; coord=5:146841857..146847013:-1; parent_transcript=GRMZM2G146677_T01; parent_gene=GRMZM2G146677	GRMZM2G146677_P01	TRUE	TRUE	hFPFFDMAYQGFASGDPER	95%	n+304 (+304)	58.49	25.00
1414	seq=translation; coord=5:146841857..146847013:-1; parent_transcript=GRMZM2G146677_T01; parent_gene=GRMZM2G146677	GRMZM2G146677_P01	TRUE	TRUE	hFPFFDMAyQGFASGDPER	95%	n+304 (+304), iTRAQ8plex (+304)	47.29	25.00
1415	seq=translation; coord=5:146841857..146847013:-1; parent_transcript=GRMZM2G146677_T01; parent_gene=GRMZM2G146677	GRMZM2G146677_P01	TRUE	TRUE	iAGNLNMEYLPMGGSVk	95%	n+304 (+304), K+304 (+304)	51.78	25.85
1416	seq=translation; coord=5:146841857..146847013:-1; parent_transcript=GRMZM2G146677_T01; parent_gene=GRMZM2G146677	GRMZM2G146677_P01	TRUE	TRUE	iSMAGVTTGNVGYLANAIHEVTkPN	95%	n+304 (+304), K+304 (+304)	59.90	25.00
1417	seq=translation; coord=5:146841857..146847013:-1; parent_transcript=GRMZM2G146677_T01; parent_gene=GRMZM2G146677	GRMZM2G146677_P01	TRUE	TRUE	kHFPFFDMAYQGFASGDPER	95%	K+304 (+304), n+304 (+304)	30.08	25.00
1418	seq=translation; coord=5:146841857..146847013:-1; parent_transcript=GRMZM2G146677_T01; parent_gene=GRMZM2G146677	GRMZM2G146677_P01	TRUE	TRUE	lAYGEDSDFIk	95%	n+304 (+304), K+304 (+304)	55.50	25.00
1419	seq=translation; coord=5:146841857..146847013:-1; parent_transcript=GRMZM2G146677_T01; parent_gene=GRMZM2G146677	GRMZM2G146677_P01	TRUE	TRUE	lFADFQk	92%	n+304 (+304), K+304 (+304)	28.68	26.35
1420	seq=translation; coord=5:146841857..146847013:-1; parent_transcript=GRMZM2G146677_T01; parent_gene=GRMZM2G146677	GRMZM2G146677_P01	TRUE	TRUE	nAPDGSFFLLHAcAHNPTGVDPTEEQWR	93%	n+304 (+304), Carbamidomethyl (+57)	25.10	25.00
1421	seq=translation; coord=1:258878414..258878962:1; parent_transcript=GRMZM2G704005_T01; parent_gene=GRMZM2G704005	GRMZM2G704005_P01	TRUE	TRUE	dPAAAPVAVGPGAEGAPPSQLPR	95%	n+304 (+304)	71.98	25.31
1422	seq=translation; coord=1:258878414..258878962:1; parent_transcript=GRMZM2G704005_T01; parent_gene=GRMZM2G704005	GRMZM2G704005_P01	TRUE	TRUE	hHLAFSVADYDGFVTGLk	95%	n+304 (+304), K+304 (+304)	28.83	25.88
1423	seq=translation; coord=1:258878414..258878962:1; parent_transcript=GRMZM2G704005_T01; parent_gene=GRMZM2G704005	GRMZM2G704005_P01	TRUE	TRUE	iPSPTYSGFQVAWLR	95%	n+304 (+304)	44.06	25.38

1424	seq=translation; coord=1:258878414..258878962:1; parent_transcript=GRMZM2G704005_T01; parent_gene=GRMZM2G704005	GRMZM2G704005_P01	TRUE	TRUE	lAAFYEAVLGFER	95%	n+304 (+304)	31.85	25.34
1425	seq=translation; coord=1:258878414..258878962:1; parent_transcript=GRMZM2G704005_T01; parent_gene=GRMZM2G704005	GRMZM2G704005_P01	TRUE	TRUE	lPSSPDVALHLIER	95%	n+304 (+304)	91.78	25.91
1426	seq=translation; coord=10:96330046..96338713:-1; parent_transcript=GRMZM5G858454_T02; parent_gene=GRMZM5G858454	GRMZM5G858454_P02	TRUE	TRUE	aGEDADSLGLTGR	95%	n+304 (+304)	45.37	25.00
1427	seq=translation; coord=10:96330046..96338713:-1; parent_transcript=GRMZM5G858454_T02; parent_gene=GRMZM5G858454	GRMZM5G858454_P02	TRUE	TRUE	cILMDNTGVPAVVDLAAMR	95%	Carbamidomethyl (+57), n+304 (+304)	33.27	25.00
1428	seq=translation; coord=10:96330046..96338713:-1; parent_transcript=GRMZM5G858454_T02; parent_gene=GRMZM5G858454	GRMZM5G858454_P02	TRUE	TRUE	dGVTTTDIVLTMTQMLR	90%	n+304 (+304)	28.47	25.88
1429	seq=translation; coord=10:96330046..96338713:-1; parent_transcript=GRMZM5G858454_T02; parent_gene=GRMZM5G858454	GRMZM5G858454_P02	TRUE	TRUE	dIWPSNQEIDEVVESSVQTHLFk	95%	n+304 (+304), K+304 (+304)	33.21	25.13
1430	seq=translation; coord=10:96330046..96338713:-1; parent_transcript=GRMZM5G858454_T02; parent_gene=GRMZM5G858454	GRMZM5G858454_P02	TRUE	TRUE	eALYPWEDR	95%	n+304 (+304)	43.70	25.00
1431	seq=translation; coord=10:96330046..96338713:-1; parent_transcript=GRMZM5G858454_T02; parent_gene=GRMZM5G858454	GRMZM5G858454_P02	TRUE	TRUE	iIDWENTSPk	95%	n+304 (+304), K+304 (+304)	43.81	25.39
1432	seq=translation; coord=10:96330046..96338713:-1; parent_transcript=GRMZM5G858454_T02; parent_gene=GRMZM5G858454	GRMZM5G858454_P02	TRUE	TRUE	iNPLIPVDAVIDHAVR	95%	n+304 (+304)	50.50	25.08
1433	seq=translation; coord=10:96330046..96338713:-1; parent_transcript=GRMZM5G858454_T02; parent_gene=GRMZM5G858454	GRMZM5G858454_P02	TRUE	TRUE	lAEIPFkPAR	94%	n+304 (+304), K+304 (+304)	28.25	25.00
1434	seq=translation; coord=10:96330046..96338713:-1; parent_transcript=GRMZM5G858454_T02; parent_gene=GRMZM5G858454	GRMZM5G858454_P02	TRUE	TRUE	lYVYDAAMk	95%	n+304 (+304), K+304 (+304)	36.55	25.75
1435	seq=translation; coord=10:96330046..96338713:-1; parent_transcript=GRMZM5G858454_T02; parent_gene=GRMZM5G858454	GRMZM5G858454_P02	TRUE	TRUE	tSLTPGSVVATEYLk	95%	n+304 (+304), K+304 (+304)	40.31	25.00
1436	seq=translation; coord=10:96330046..96338713:-1; parent_transcript=GRMZM5G858454_T02; parent_gene=GRMZM5G858454	GRMZM5G858454_P02	TRUE	TRUE	vDVAGTYDALDR	95%	n+304 (+304)	44.23	25.00
1437	seq=translation; coord=10:96330046..96338713:-1; parent_transcript=GRMZM5G858454_T02; parent_gene=GRMZM5G858454	GRMZM5G858454_P02	TRUE	TRUE	vLLESAIR	89%	n+304 (+304)	26.96	25.00
1438	seq=translation; coord=10:96330046..96338713:-1; parent_transcript=GRMZM5G858454_T02; parent_gene=GRMZM5G858454	GRMZM5G858454_P02	TRUE	TRUE	vYDSIMER	95%	n+304 (+304)	34.08	25.00
1439	seq=translation; coord=10:96330046..96338713:-1; parent_transcript=GRMZM5G858454_T02; parent_gene=GRMZM5G858454	GRMZM5G858454_P02	TRUE	TRUE	wNQLPVPk	95%	n+304 (+304), K+304 (+304)	34.74	25.37
1440	seq=translation; coord=2:218589206..218592127:-1; parent_transcript=GRMZM2G140667_T01; parent_gene=GRMZM2G140667	GRMZM2G140667_P01,GRMZM2G140667_P02	TRUE	TRUE	aYPTVNEDYLk	95%	n+304 (+304), K+304 (+304)	36.60	26.15
1441	seq=translation; coord=2:218589206..218592127:-1; parent_transcript=GRMZM2G140667_T01; parent_gene=GRMZM2G140667	GRMZM2G140667_P01,GRMZM2G140667_P02	TRUE	TRUE	eLLSGEk	92%	n+304 (+304), K+304 (+304)	29.41	26.76

1442	seq=translation; coord=2:218589206..218592127:-1; parent_transcript=GRMZM2G140667_T01; parent_gene=GRMZM2G140667	GRMZM2G140667_P01,GRMZM2G140667_P02	TRUE	TRUE	lAWHSAGTFDVATk	92%	n+304 (+304), K+304 (+304)	27.62	25.73
1443	seq=translation; coord=2:218589206..218592127:-1; parent_transcript=GRMZM2G140667_T01; parent_gene=GRMZM2G140667	GRMZM2G140667_P01,GRMZM2G140667_P02	TRUE	TRUE	nPAEQAHGANAGLEIAIR	95%	n+304 (+304)	42.17	25.92
1444	seq=translation; coord=2:218589206..218592127:-1; parent_transcript=GRMZM2G140667_T01; parent_gene=GRMZM2G140667	GRMZM2G140667_P01,GRMZM2G140667_P02	TRUE	TRUE	qDkPEPPPEGR	91%	Pyro-cmC (-17), n+304 (+304), K+304 (+304)	25.38	25.00
1445	seq=translation; coord=2:218589206..218592127:-1; parent_transcript=GRMZM2G140667_T01; parent_gene=GRMZM2G140667	GRMZM2G140667_P01,GRMZM2G140667_P02	TRUE	TRUE	qVFSTQMGLSDQDIVALSGGHTLGR	95%	n+304 (+304)	93.33	25.00
1446	seq=translation; coord=2:218589206..218592127:-1; parent_transcript=GRMZM2G140667_T01; parent_gene=GRMZM2G140667	GRMZM2G140667_P01,GRMZM2G140667_P02	TRUE	TRUE	sGFEGAWTSNPLIFDNSYFk	90%	n+304 (+304), K+304 (+304)	25.69	25.00
1447	seq=translation; coord=2:218589206..218592127:-1; parent_transcript=GRMZM2G140667_T01; parent_gene=GRMZM2G140667	GRMZM2G140667_P01,GRMZM2G140667_P02	TRUE	TRUE	yAADEDAFFADYAEAHLk	95%	n+304 (+304), K+304 (+304)	45.28	25.00
1448	seq=translation; coord=6:145087390..145092201:1; parent_transcript=GRMZM2G179301_T02; parent_gene=GRMZM2G179301	GRMZM2G179301_P02,GRMZM2G179301_P03	TRUE	TRUE	aAVVLVADVFGFEAPIMR	95%	n+304 (+304)	42.76	26.55
1449	seq=translation; coord=6:145087390..145092201:1; parent_transcript=GRMZM2G179301_T02; parent_gene=GRMZM2G179301	GRMZM2G179301_P02,GRMZM2G179301_P03	TRUE	TRUE	aAVVLVADVFGFEAPImR	92%	n+304 (+304), Oxidation (+16)	26.70	26.04
1450	seq=translation; coord=6:145087390..145092201:1; parent_transcript=GRMZM2G179301_T02; parent_gene=GRMZM2G179301	GRMZM2G179301_P02,GRMZM2G179301_P03	TRUE	TRUE	aYVAGPEDSk	95%	n+304 (+304), K+304 (+304)	38.19	25.81
1451	seq=translation; coord=6:145087390..145092201:1; parent_transcript=GRMZM2G179301_T02; parent_gene=GRMZM2G179301	GRMZM2G179301_P02,GRMZM2G179301_P03	TRUE	TRUE	gFEETk	87%	n+304 (+304), K+304 (+304)	27.48	26.67
1452	seq=translation; coord=6:145087390..145092201:1; parent_transcript=GRMZM2G179301_T02; parent_gene=GRMZM2G179301	GRMZM2G179301_P02,GRMZM2G179301_P03	TRUE	TRUE	iFPGVAHGWSVR	95%	n+304 (+304)	50.39	25.95
1453	seq=translation; coord=6:145087390..145092201:1; parent_transcript=GRMZM2G179301_T02; parent_gene=GRMZM2G179301	GRMZM2G179301_P02,GRMZM2G179301_P03	TRUE	TRUE	qVIGALk	95%	n+304 (+304), K+304 (+304)	29.04	25.00
1454	seq=translation; coord=6:145087390..145092201:1; parent_transcript=GRMZM2G179301_T02; parent_gene=GRMZM2G179301	GRMZM2G179301_P02,GRMZM2G179301_P03	TRUE	TRUE	sAEEAFADMLDWFNk	95%	n+304 (+304), K+304 (+304)	49.03	25.00
1455	seq=translation; coord=6:145087390..145092201:1; parent_transcript=GRMZM2G179301_T02; parent_gene=GRMZM2G179301	GRMZM2G179301_P02,GRMZM2G179301_P03	TRUE	TRUE	vVDSFGGLk	95%	n+304 (+304), K+304 (+304)	35.95	25.62
1456	seq=translation; coord=1:79067709..79069201:-1; parent_transcript=GRMZM2G148925_T01; parent_gene=GRMZM2G148925	GRMZM2G148925_P01	TRUE	TRUE	gEQQVVSGMNYR	95%	n+304 (+304)	49.70	25.00
1457	seq=translation; coord=1:79067709..79069201:-1; parent_transcript=GRMZM2G148925_T01; parent_gene=GRMZM2G148925	GRMZM2G148925_P01	TRUE	TRUE	gEQQVVSGmNYR	95%	n+304 (+304), Oxidation (+16)	39.32	25.00
1458	seq=translation; coord=1:79067709..79069201:-1; parent_transcript=GRMZM2G148925_T01; parent_gene=GRMZM2G148925	GRMZM2G148925_P01	TRUE	TRUE	lAADGLR	93%	n+304 (+304)	30.15	25.87
1459	seq=translation; coord=1:79067709..79069201:-1; parent_transcript=GRMZM2G148925_T01; parent_gene=GRMZM2G148925	GRMZM2G148925_P01	TRUE	TRUE	lYVDAADPAGR	93%	n+304 (+304)	29.66	25.00

1460	seq=translation; coord=1:79067709..79069201:-1; parent_transcript=GRMZM2G148925_T01; parent_gene=GRMZM2G148925	GRMZM2G148925_P01	TRUE	TRUE	qLASFNPVPR	95%	n+304 (+304)	35.09	26.01
1461	seq=translation; coord=1:79067709..79069201:-1; parent_transcript=GRMZM2G148925_T01; parent_gene=GRMZM2G148925	GRMZM2G148925_P01	TRUE	TRUE	tVPYVAVVYEQVWTR	95%	n+304 (+304)	43.88	26.01
1462	seq=translation; coord=1:79067709..79069201:-1; parent_transcript=GRMZM2G148925_T01; parent_gene=GRMZM2G148925	GRMZM2G148925_P01	TRUE	TRUE	tVPyVAVVYEQVWTR	95%	n+304 (+304), iTRAQ8plex (+304)	32.45	25.45
1463	seq=translation; coord=1:79067709..79069201:-1; parent_transcript=GRMZM2G148925_T01; parent_gene=GRMZM2G148925	GRMZM2G148925_P01	TRUE	TRUE	tVPYVAVVyEQVWTR	95%	n+304 (+304), iTRAQ8plex (+304)	56.35	25.49
1464	seq=translation; coord=6:113557759..113560731:-1; parent_transcript=GRMZM2G701082_T04; parent_gene=GRMZM2G701082	GRMZM2G701082_P04,GRMZM2G701082_P05, GRMZM2G701082_P06	TRUE	TRUE	aQEETYGSSGYGYGR	95%	n+304 (+304)	33.95	25.00
1465	seq=translation; coord=6:113557759..113560731:-1; parent_transcript=GRMZM2G701082_T04; parent_gene=GRMZM2G701082	GRMZM2G701082_P04,GRMZM2G701082_P05, GRMZM2G701082_P06	TRUE	TRUE	kASEEDEGAFGSGGYR	95%	K+304 (+304), n+304 (+304)	34.44	25.00
1466	seq=translation; coord=6:113557759..113560731:-1; parent_transcript=GRMZM2G701082_T04; parent_gene=GRMZM2G701082	GRMZM2G701082_P04,GRMZM2G701082_P05, GRMZM2G701082_P06	TRUE	TRUE	kNEEQSYGGSGYGYEk	95%	K+304 (+304), n+304 (+304), K+304 (+304)	34.10	25.00
1467	seq=translation; coord=6:113557759..113560731:-1; parent_transcript=GRMZM2G701082_T04; parent_gene=GRMZM2G701082	GRMZM2G701082_P04,GRMZM2G701082_P05, GRMZM2G701082_P06	TRUE	TRUE	kPQVEESYGSEYGSGFGR	95%	K+304 (+304), n+304 (+304)	38.82	25.00
1468	seq=translation; coord=6:113557759..113560731:-1; parent_transcript=GRMZM2G701082_T04; parent_gene=GRMZM2G701082	GRMZM2G701082_P04,GRMZM2G701082_P05, GRMZM2G701082_P06	TRUE	TRUE	qEEESYGSGYGR	95%	n+304 (+304)	36.52	25.00
1469	seq=translation; coord=6:113557759..113560731:-1; parent_transcript=GRMZM2G701082_T04; parent_gene=GRMZM2G701082	GRMZM2G701082_P04,GRMZM2G701082_P05, GRMZM2G701082_P06	TRUE	TRUE	tPQVEESYGSGYGR	95%	n+304 (+304)	52.52	25.00
1470	seq=translation; coord=3:224673584..224677732:1; parent_transcript=GRMZM2G034985_T01; parent_gene=GRMZM2G034985	GRMZM2G034985_P01,GRMZM2G034985_P03	TRUE	TRUE	aVLDFTAIANR	90%	n+304 (+304)	28.17	25.81
1471	seq=translation; coord=3:224673584..224677732:1; parent_transcript=GRMZM2G034985_T01; parent_gene=GRMZM2G034985	GRMZM2G034985_P01,GRMZM2G034985_P03	TRUE	TRUE	eSALVQEVR	93%	n+304 (+304)	29.32	25.00
1472	seq=translation; coord=3:224673584..224677732:1; parent_transcript=GRMZM2G034985_T01; parent_gene=GRMZM2G034985	GRMZM2G034985_P01,GRMZM2G034985_P03	TRUE	TRUE	lAFAVGDSGDGcAGSMAAEAYAAR	95%	n+304 (+304), Carbamidomethyl (+57)	40.63	25.00
1473	seq=translation; coord=3:224673584..224677732:1; parent_transcript=GRMZM2G034985_T01; parent_gene=GRMZM2G034985	GRMZM2G034985_P01,GRMZM2G034985_P03	TRUE	TRUE	lAFAVGDSGDGcAGSMAAEAyAAR	95%	n+304 (+304), Carbamidomethyl (+57), iTRAQ8plex (+304)	41.64	25.00
1474	seq=translation; coord=3:224673584..224677732:1; parent_transcript=GRMZM2G034985_T01; parent_gene=GRMZM2G034985	GRMZM2G034985_P01,GRMZM2G034985_P03	TRUE	TRUE	lGNEASIR	93%	n+304 (+304)	31.36	26.61
1475	seq=translation; coord=3:224673584..224677732:1; parent_transcript=GRMZM2G034985_T01; parent_gene=GRMZM2G034985	GRMZM2G034985_P01,GRMZM2G034985_P03	TRUE	TRUE	mDAVTLVIHNPGVEEDPAcGPLIDGVAIR	95%	n+304 (+304), Carbamidomethyl (+57)	64.32	25.00
1476	seq=translation; coord=3:224673584..224677732:1; parent_transcript=GRMZM2G034985_T01; parent_gene=GRMZM2G034985	GRMZM2G034985_P01,GRMZM2G034985_P03	TRUE	TRUE	mDAVTLVIHNPGVEEDPAcGPLIDGVAIR	95%	Oxidation (+16), n+304 (+304), Carbamidomethyl (+57)	72.23	25.00
1477	seq=translation; coord=3:224673584..224677732:1; parent_transcript=GRMZM2G034985_T01; parent_gene=GRMZM2G034985	GRMZM2G034985_P01,GRMZM2G034985_P03	TRUE	TRUE	qGDMLLVVPQGAHAVR	95%	n+304 (+304)	41.61	26.16

1478	seq=translation; coord=3:224673584..224677732:1; parent_transcript=GRMZM2G034985_T01; parent_gene=GRMZM2G034985	GRMZM2G034985_P01,GRMZM2G034985_P03	TRUE	TRUE	qGDMLLVVPQGAHAVR	95%	Pyro-cmC (-17), n+304 (+304)	47.91	25.00
1479	seq=translation; coord=3:224673584..224677732:1; parent_transcript=GRMZM2G034985_T01; parent_gene=GRMZM2G034985	GRMZM2G034985_P01,GRMZM2G034985_P03	TRUE	TRUE	qGDmLLVVPQGAHAVR	95%	n+304 (+304), Oxidation (+16)	38.80	25.87
1480	seq=translation; coord=3:224673584..224677732:1; parent_transcript=GRMZM2G034985_T01; parent_gene=GRMZM2G034985	GRMZM2G034985_P01,GRMZM2G034985_P03	TRUE	TRUE	qGDmLLVVPQGAHAVR	95%	Pyro-cmC (-17), n+304 (+304), Oxidation (+16)	57.40	25.00
1481	seq=translation; coord=3:224673584..224677732:1; parent_transcript=GRMZM2G034985_T01; parent_gene=GRMZM2G034985	GRMZM2G034985_P01,GRMZM2G034985_P03	TRUE	TRUE	wETSGFVEYIESGHk	95%	n+304 (+304), K+304 (+304)	57.23	25.00
1482	seq=translation; coord=3:224673584..224677732:1; parent_transcript=GRMZM2G034985_T01; parent_gene=GRMZM2G034985	GRMZM2G034985_P01,GRMZM2G034985_P03	TRUE	TRUE	yVDAAHFAVPQGAR	95%	n+304 (+304)	35.93	25.48
1483	seq=translation; coord=5:15546617..15552849:-1; parent_transcript=GRMZM2G112149_T01; parent_gene=GRMZM2G112149	GRMZM2G112149_P01,GRMZM2G112149_P02, GRMZM2G112149_P03	TRUE	TRUE	aAGASWIQLDEPTLVk	95%	n+304 (+304), K+304 (+304)	47.84	25.40
1484	seq=translation; coord=5:15546617..15552849:-1; parent_transcript=GRMZM2G112149_T01; parent_gene=GRMZM2G112149	GRMZM2G112149_P01,GRMZM2G112149_P02, GRMZM2G112149_P03	TRUE	TRUE	dEVYFAANAAAQASR	95%	n+304 (+304)	80.40	25.00
1485	seq=translation; coord=5:15546617..15552849:-1; parent_transcript=GRMZM2G112149_T01; parent_gene=GRMZM2G112149	GRMZM2G112149_P01,GRMZM2G112149_P02, GRMZM2G112149_P03	TRUE	TRUE	nIWADDLAASLSTLHSLEAVAGk	95%	n+304 (+304), K+304 (+304)	30.05	25.00
1486	seq=translation; coord=5:15546617..15552849:-1; parent_transcript=GRMZM2G112149_T01; parent_gene=GRMZM2G112149	GRMZM2G112149_P01,GRMZM2G112149_P02, GRMZM2G112149_P03	TRUE	TRUE	sSAEDLEk	94%	n+304 (+304), K+304 (+304)	30.54	25.00
1487	seq=translation; coord=2:37325095..37329643:1; parent_transcript=GRMZM2G163471_T01; parent_gene=GRMZM2G163471	GRMZM2G163471_P01	TRUE	TRUE	fTPAPADSGR	89%	n+304 (+304)	26.14	25.00
1488	seq=translation; coord=2:37325095..37329643:1; parent_transcript=GRMZM2G163471_T01; parent_gene=GRMZM2G163471	GRMZM2G163471_P01	TRUE	TRUE	gLTHEEMMLLPTGPR	95%	n+304 (+304)	48.41	25.00
1489	seq=translation; coord=2:37325095..37329643:1; parent_transcript=GRMZM2G163471_T01; parent_gene=GRMZM2G163471	GRMZM2G163471_P01	TRUE	TRUE	gTTLSLSEFTTFGAAAAQR	95%	n+304 (+304)	63.98	25.16
1490	seq=translation; coord=2:37325095..37329643:1; parent_transcript=GRMZM2G163471_T01; parent_gene=GRMZM2G163471	GRMZM2G163471_P01	TRUE	TRUE	iDVELEHR	95%	n+304 (+304)	44.36	25.40
1491	seq=translation; coord=2:37325095..37329643:1; parent_transcript=GRMZM2G163471_T01; parent_gene=GRMZM2G163471	GRMZM2G163471_P01	TRUE	TRUE	iSMLESQLEQLSR	95%	n+304 (+304)	47.06	25.75
1492	seq=translation; coord=2:37325095..37329643:1; parent_transcript=GRMZM2G163471_T01; parent_gene=GRMZM2G163471	GRMZM2G163471_P01	TRUE	TRUE	sSDLDMTSR	91%	n+304 (+304)	26.76	25.00
1493	seq=translation; coord=2:37325095..37329643:1; parent_transcript=GRMZM2G163471_T01; parent_gene=GRMZM2G163471	GRMZM2G163471_P01	TRUE	TRUE	vNPFGDAkPR	95%	n+304 (+304), K+304 (+304)	41.71	25.37
1494	seq=translation; coord=2:37325095..37329643:1; parent_transcript=GRMZM2G163471_T01; parent_gene=GRMZM2G163471	GRMZM2G163471_P01	TRUE	TRUE	vTTLAPTSLGEEPQATVVDRPR	95%	n+304 (+304)	33.35	25.67
1495	seq=translation; coord=7:18473070..18475522:1; parent_transcript=GRMZM2G168149_T01; parent_gene=GRMZM2G168149	GRMZM2G168149_P01	TRUE	TRUE	tYGFLTPEFWAETk	95%	n+304 (+304), K+304 (+304)	48.94	25.29

1496	seq=translation; coord=6:44382950..44385913:-1; parent_transcript=GRMZM2G087570_T01; parent_gene=GRMZM2G087570	GRMZM2G087570_P01	TRUE	TRUE	hVVFGQVVEGMDIVR	95%	n+304 (+304)	62.61	25.11
1497	seq=translation; coord=6:44382950..44385913:-1; parent_transcript=GRMZM2G087570_T01; parent_gene=GRMZM2G087570	GRMZM2G087570_P01	TRUE	TRUE	iVIGLYGDDVPQTAENFR	95%	n+304 (+304)	77.56	25.17
1498	seq=translation; coord=6:44382950..44385913:-1; parent_transcript=GRMZM2G087570_T01; parent_gene=GRMZM2G087570	GRMZM2G087570_P01	TRUE	TRUE	iVIGLyGDDVPQTAENFR	91%	n+304 (+304), iTRAQ8plex (+304)	26.20	25.79
1499	seq=translation; coord=6:44382950..44385913:-1; parent_transcript=GRMZM2G087570_T01; parent_gene=GRMZM2G087570	GRMZM2G087570_P01	TRUE	TRUE	lIESQETDR	93%	n+304 (+304)	30.03	25.00
1500	seq=translation; coord=5:186677004..186680745:-1; parent_transcript=GRMZM2G074604_T01; parent_gene=GRMZM2G074604	GRMZM2G074604_P01	TRUE	TRUE	aVLPQEVEAAR	95%	n+304 (+304)	33.63	26.63
1501	seq=translation; coord=5:186677004..186680745:-1; parent_transcript=GRMZM2G074604_T01; parent_gene=GRMZM2G074604	GRMZM2G074604_P01	TRUE	TRUE	dGPALQVELLR	95%	n+304 (+304)	40.74	26.20
1502	seq=translation; coord=5:186677004..186680745:-1; parent_transcript=GRMZM2G074604_T01; parent_gene=GRMZM2G074604	GRMZM2G074604_P01	TRUE	TRUE	eAVFTYAEDAASGSLPLMQk	95%	n+304 (+304), K+304 (+304)	54.97	25.26
1503	seq=translation; coord=5:186677004..186680745:-1; parent_transcript=GRMZM2G074604_T01; parent_gene=GRMZM2G074604	GRMZM2G074604_P01	TRUE	TRUE	eELGcVFLTGER	95%	n+304 (+304), Carbamidomethyl (+57)	49.17	25.00
1504	seq=translation; coord=5:186677004..186680745:-1; parent_transcript=GRMZM2G074604_T01; parent_gene=GRMZM2G074604	GRMZM2G074604_P01	TRUE	TRUE	eLISAIDR	95%	n+304 (+304)	32.84	25.49
1505	seq=translation; coord=5:186677004..186680745:-1; parent_transcript=GRMZM2G074604_T01; parent_gene=GRMZM2G074604	GRMZM2G074604_P01	TRUE	TRUE	iAGIEGGFFk	95%	n+304 (+304), K+304 (+304)	45.34	25.00
1506	seq=translation; coord=5:186677004..186680745:-1; parent_transcript=GRMZM2G074604_T01; parent_gene=GRMZM2G074604	GRMZM2G074604_P01	TRUE	TRUE	lAIANIGk	92%	n+304 (+304), K+304 (+304)	28.58	25.00
1507	seq=translation; coord=5:186677004..186680745:-1; parent_transcript=GRMZM2G074604_T01; parent_gene=GRMZM2G074604	GRMZM2G074604_P01	TRUE	TRUE	lVDPMLEcLk	94%	n+304 (+304), Carbamidomethyl (+57), K+304 (+304)	31.70	25.90
1508	seq=translation; coord=5:186677004..186680745:-1; parent_transcript=GRMZM2G074604_T01; parent_gene=GRMZM2G074604	GRMZM2G074604_P01	TRUE	TRUE	vAVAEGTAPVANR	95%	n+304 (+304)	34.28	26.07
1509	seq=translation; coord=5:186677004..186680745:-1; parent_transcript=GRMZM2G074604_T01; parent_gene=GRMZM2G074604	GRMZM2G074604_P01	TRUE	TRUE	vFVGISQGk	95%	n+304 (+304), K+304 (+304)	29.96	25.00
1510	seq=translation; coord=5:186677004..186680745:-1; parent_transcript=GRMZM2G074604_T01; parent_gene=GRMZM2G074604	GRMZM2G074604_P01	TRUE	TRUE	vLTMNPSGELSSAR	95%	n+304 (+304)	41.77	25.00
1511	seq=translation; coord=9:78409591..78421558:1; parent_transcript=GRMZM2G094497_T01; parent_gene=GRMZM2G094497	GRMZM2G094497_P01,GRMZM2G181151_P01, GRMZM2G181151_P02	TRUE	TRUE	aVVGEEALSSEDLLYLEFLDkFER	95%	n+304 (+304), K+304 (+304)	70.27	25.47
1512	seq=translation; coord=9:78409591..78421558:1; parent_transcript=GRMZM2G094497_T01; parent_gene=GRMZM2G094497	GRMZM2G094497_P01,GRMZM2G181151_P01, GRMZM2G181151_P02	TRUE	TRUE	gYPGYMYTDLATIYER	90%	n+304 (+304)	27.19	25.00
1513	seq=translation; coord=9:78409591..78421558:1; parent_transcript=GRMZM2G094497_T01; parent_gene=GRMZM2G094497	GRMZM2G094497_P01,GRMZM2G181151_P01, GRMZM2G181151_P02	TRUE	TRUE	gyPGYMYTDLATIYER	95%	n+304 (+304), iTRAQ8plex (+304)	41.22	25.00

1514	seq=translation; coord=9:78409591..78421558:1; parent_transcript=GRMZM2G094497_T01; parent_gene=GRMZM2G094497	GRMZM2G094497_P01,GRMZM2G181151_P01, GRMZM2G181151_P02	TRUE	TRUE	hVLVILTDMSSYADALR	95%	n+304 (+304)	50.23	25.24
1515	seq=translation; coord=9:78409591..78421558:1; parent_transcript=GRMZM2G094497_T01; parent_gene=GRMZM2G094497	GRMZM2G094497_P01,GRMZM2G181151_P01, GRMZM2G181151_P02	TRUE	TRUE	iALTTAEYLAYEcGk	95%	n+304 (+304), Carbamidomethyl (+57), K+304 (+304)	53.53	25.09
1516	seq=translation; coord=9:78409591..78421558:1; parent_transcript=GRMZM2G094497_T01; parent_gene=GRMZM2G094497	GRMZM2G094497_P01,GRMZM2G181151_P01, GRMZM2G181151_P02	TRUE	TRUE	iPLFSAAGLPHNEIAAQIcR	95%	n+304 (+304), Carbamidomethyl (+57)	50.48	26.07
1517	seq=translation; coord=9:78409591..78421558:1; parent_transcript=GRMZM2G094497_T01; parent_gene=GRMZM2G094497	GRMZM2G094497_P01,GRMZM2G181151_P01, GRMZM2G181151_P02	TRUE	TRUE	nIFQSLDLAWTLLR	95%	n+304 (+304)	56.73	25.07
1518	seq=translation; coord=9:78409591..78421558:1; parent_transcript=GRMZM2G094497_T01; parent_gene=GRMZM2G094497	GRMZM2G094497_P01,GRMZM2G181151_P01, GRMZM2G181151_P02	TRUE	TRUE	qIYPPINVLPSLSR	95%	n+304 (+304)	33.21	25.76
1519	seq=translation; coord=9:78409591..78421558:1; parent_transcript=GRMZM2G094497_T01; parent_gene=GRMZM2G094497	GRMZM2G094497_P01,GRMZM2G181151_P01, GRMZM2G181151_P02	TRUE	TRUE	sAIGEGMTR	87%	n+304 (+304)	26.11	25.19
1520	seq=translation; coord=9:78409591..78421558:1; parent_transcript=GRMZM2G094497_T01; parent_gene=GRMZM2G094497	GRMZM2G094497_P01,GRMZM2G181151_P01, GRMZM2G181151_P02	TRUE	TRUE	tPVSLDMLGR	94%	n+304 (+304)	32.01	25.72
1521	seq=translation; coord=9:78409591..78421558:1; parent_transcript=GRMZM2G094497_T01; parent_gene=GRMZM2G094497	GRMZM2G094497_P01,GRMZM2G181151_P01, GRMZM2G181151_P02	TRUE	TRUE	tVSGVAGPLVILDk	95%	n+304 (+304), K+304 (+304)	46.79	25.00
1522	seq=translation; coord=9:78409591..78421558:1; parent_transcript=GRMZM2G094497_T01; parent_gene=GRMZM2G094497	GRMZM2G094497_P01,GRMZM2G181151_P01, GRMZM2G181151_P02	TRUE	TRUE	vTLFLNLANDPTIER	95%	n+304 (+304)	72.30	25.47
1523	seq=translation; coord=9:78409591..78421558:1; parent_transcript=GRMZM2G094497_T01; parent_gene=GRMZM2G094497	GRMZM2G094497_P01,GRMZM2G181151_P01, GRMZM2G181151_P02	TRUE	TRUE	yTTVQFTGEVLk	95%	n+304 (+304), K+304 (+304)	52.94	25.00
1524	seq=translation; coord=3:161729177..161732426:-1; parent_transcript=GRMZM2G103812_T01; parent_gene=GRMZM2G103812	GRMZM2G103812_P01,GRMZM2G103812_P02	TRUE	TRUE	fLILPSLLSGR	95%	n+304 (+304)	34.45	25.00
1525	seq=translation; coord=3:161729177..161732426:-1; parent_transcript=GRMZM2G103812_T01; parent_gene=GRMZM2G103812	GRMZM2G103812_P01,GRMZM2G103812_P02	TRUE	TRUE	gGPQMIQLSLDGk	95%	n+304 (+304), K+304 (+304)	35.00	26.32
1526	seq=translation; coord=3:161729177..161732426:-1; parent_transcript=GRMZM2G103812_T01; parent_gene=GRMZM2G103812	GRMZM2G103812_P01,GRMZM2G103812_P02	TRUE	TRUE	gPGYATPLEAMEk	89%	n+304 (+304), K+304 (+304)	26.18	25.00
1527	seq=translation; coord=3:161729177..161732426:-1; parent_transcript=GRMZM2G103812_T01; parent_gene=GRMZM2G103812	GRMZM2G103812_P01,GRMZM2G103812_P02	TRUE	TRUE	gSDVVYVTDDGQEQQYNVPQVk	95%	n+304 (+304), K+304 (+304)	39.51	25.00
1528	seq=translation; coord=3:161729177..161732426:-1; parent_transcript=GRMZM2G103812_T01; parent_gene=GRMZM2G103812	GRMZM2G103812_P01,GRMZM2G103812_P02	TRUE	TRUE	qTLDLGDTGLLPLEVR	95%	n+304 (+304)	46.37	25.40
1529	seq=translation; coord=3:161729177..161732426:-1; parent_transcript=GRMZM2G103812_T01; parent_gene=GRMZM2G103812	GRMZM2G103812_P01,GRMZM2G103812_P02	TRUE	TRUE	tMISSSWGAPAAFR	95%	n+304 (+304)	44.03	25.00
1530	seq=translation; coord=3:161729177..161732426:-1; parent_transcript=GRMZM2G103812_T01; parent_gene=GRMZM2G103812	GRMZM2G103812_P01,GRMZM2G103812_P02	TRUE	TRUE	wDEQFFGDDLVk	95%	n+304 (+304), K+304 (+304)	30.97	25.00
1531	seq=translation; coord=3:161729177..161732426:-1; parent_transcript=GRMZM2G103812_T01; parent_gene=GRMZM2G103812	GRMZM2G103812_P01,GRMZM2G103812_P02	TRUE	TRUE	wEkPGHSPLFGYDFWYQPR	95%	n+304 (+304), K+304 (+304)	37.81	25.00

1532	seq=translation; coord=3:161729177..161732426:-1; parent_transcript=GRMZM2G103812_T01; parent_gene=GRMZM2G103812	GRMZM2G103812_P01,GRMZM2G103812_P02	TRUE	TRUE	yLYFVNWLHGDIR	95%	n+304 (+304)	42.06	25.17
1533	seq=translation; coord=1:204259324..204264422:-1; parent_transcript=AC234528.1_FGT005; parent_gene=AC234528.1_FG005	AC234528.1_FGP005,GRMZM2G014069_P01	TRUE	TRUE	eAGMVGFR	88%	n+304 (+304)	26.41	25.00
1534	seq=translation; coord=1:204259324..204264422:-1; parent_transcript=AC234528.1_FGT005; parent_gene=AC234528.1_FG005	AC234528.1_FGP005,GRMZM2G014069_P01	TRUE	TRUE	ecALLTDGR	92%	n+304 (+304), Carbamidomethyl (+57)	28.92	25.00
1535	seq=translation; coord=1:204259324..204264422:-1; parent_transcript=AC234528.1_FGT005; parent_gene=AC234528.1_FG005	AC234528.1_FGP005,GRMZM2G014069_P01	TRUE	TRUE	fNTVGVSDAISMGTR	95%	n+304 (+304)	70.56	25.00
1536	seq=translation; coord=1:204259324..204264422:-1; parent_transcript=AC234528.1_FGT005; parent_gene=AC234528.1_FG005	AC234528.1_FGP005,GRMZM2G014069_P01	TRUE	TRUE	gGPGMPEMLTPTSAIMGAGLGk	95%	n+304 (+304), K+304 (+304)	47.74	25.51
1537	seq=translation; coord=1:204259324..204264422:-1; parent_transcript=AC234528.1_FGT005; parent_gene=AC234528.1_FG005	AC234528.1_FGP005,GRMZM2G014069_P01	TRUE	TRUE	sQGASQAVLYGVGLTDADLR	95%	n+304 (+304)	81.93	25.15
1538	seq=translation; coord=1:204259324..204264422:-1; parent_transcript=AC234528.1_FGT005; parent_gene=AC234528.1_FG005	AC234528.1_FGP005,GRMZM2G014069_P01	TRUE	TRUE	vIDVDLTEQQLEER	95%	n+304 (+304)	62.90	25.67
1539	seq=translation; coord=1:204259324..204264422:-1; parent_transcript=AC234528.1_FGT005; parent_gene=AC234528.1_FG005	AC234528.1_FGP005,GRMZM2G014069_P01	TRUE	TRUE	wTPPPYk	94%	n+304 (+304), K+304 (+304)	30.15	26.09
1540	seq=translation; coord=1:204259324..204264422:-1; parent_transcript=AC234528.1_FGT005; parent_gene=AC234528.1_FG005	AC234528.1_FGP005,GRMZM2G014069_P01	TRUE	TRUE	yLLELLk	90%	n+304 (+304), K+304 (+304)	25.27	25.00
1541	seq=translation; coord=7:149915726..149918129:-1; parent_transcript=GRMZM2G134797_T02; parent_gene=GRMZM2G134797	GRMZM2G134797_P02	TRUE	TRUE	dVVLTGR	92%	n+304 (+304)	29.79	25.55
1542	seq=translation; coord=7:149915726..149918129:-1; parent_transcript=GRMZM2G134797_T02; parent_gene=GRMZM2G134797	GRMZM2G134797_P02	TRUE	TRUE	eIALWFPEGVAQWk	95%	n+304 (+304), K+304 (+304)	49.47	25.69
1543	seq=translation; coord=7:149915726..149918129:-1; parent_transcript=GRMZM2G134797_T02; parent_gene=GRMZM2G134797	GRMZM2G134797_P02	TRUE	TRUE	gDYAVEVGR	94%	n+304 (+304)	30.45	25.00
1544	seq=translation; coord=7:149915726..149918129:-1; parent_transcript=GRMZM2G134797_T02; parent_gene=GRMZM2G134797	GRMZM2G134797_P02	TRUE	TRUE	gLIGDIISR	95%	n+304 (+304)	41.20	25.00
1545	seq=translation; coord=7:149915726..149918129:-1; parent_transcript=GRMZM2G134797_T02; parent_gene=GRMZM2G134797	GRMZM2G134797_P02	TRUE	TRUE	iIGATRPWEAAPGTIR	91%	n+304 (+304)	28.99	25.65
1546	seq=translation; coord=7:149915726..149918129:-1; parent_transcript=GRMZM2G134797_T02; parent_gene=GRMZM2G134797	GRMZM2G134797_P02	TRUE	TRUE	kEIALWFPEGVAQWk	95%	K+304 (+304), n+304 (+304), K+304 (+304)	27.03	25.00
1547	seq=translation; coord=5:212375127..212379000:-1; parent_transcript=GRMZM2G110185_T02; parent_gene=GRMZM2G110185	GRMZM2G110185_P02,GRMZM2G181359_P01	TRUE	TRUE	acIVFFDEVDAIGGAR	95%	n+304 (+304), Carbamidomethyl (+57)	66.66	25.00
1548	seq=translation; coord=5:212375127..212379000:-1; parent_transcript=GRMZM2G110185_T02; parent_gene=GRMZM2G110185	GRMZM2G110185_P02,GRMZM2G181359_P01	TRUE	TRUE	eSDTGLAPPSQWDLVSDk	92%	n+304 (+304), K+304 (+304)	27.92	25.00
1549	seq=translation; coord=5:212375127..212379000:-1; parent_transcript=GRMZM2G110185_T02; parent_gene=GRMZM2G110185	GRMZM2G110185_P02,GRMZM2G181359_P01	TRUE	TRUE	fDDGVGGDNEVQR	91%	n+304 (+304)	27.71	25.00

1550	seq=translation; coord=5:212375127..212379000:-1; parent_transcript=GRMZM2G110185_T02; parent_gene=GRMZM2G110185	GRMZM2G110185_P02,GRMZM2G181359_P01	TRUE	TRUE	gVLcYGPPGTGk	90%	n+304 (+304), Carbamidomethyl (+57), K+304 (+304)	28.93	26.36
1551	seq=translation; coord=5:212375127..212379000:-1; parent_transcript=GRMZM2G110185_T02; parent_gene=GRMZM2G110185	GRMZM2G110185_P02,GRMZM2G181359_P01	TRUE	TRUE	iISPNTDDAk	95%	n+304 (+304), K+304 (+304)	36.54	26.01
1552	seq=translation; coord=5:212375127..212379000:-1; parent_transcript=GRMZM2G110185_T02; parent_gene=GRMZM2G110185	GRMZM2G110185_P02,GRMZM2G181359_P01	TRUE	TRUE	qMMQEEQPLQVAR	95%	n+304 (+304)	42.47	25.00
1553	seq=translation; coord=5:212375127..212379000:-1; parent_transcript=GRMZM2G110185_T02; parent_gene=GRMZM2G110185	GRMZM2G110185_P02,GRMZM2G181359_P01	TRUE	TRUE	sVcTEAGMYAIR	95%	n+304 (+304), Carbamidomethyl (+57)	36.39	25.00
1554	seq=translation; coord=5:212375127..212379000:-1; parent_transcript=GRMZM2G110185_T02; parent_gene=GRMZM2G110185	GRMZM2G110185_P02,GRMZM2G181359_P01	TRUE	TRUE	tMLEIVNQLDGFDAR	95%	n+304 (+304)	53.61	25.00
1555	seq=translation; coord=5:212375127..212379000:-1; parent_transcript=GRMZM2G110185_T02; parent_gene=GRMZM2G110185	GRMZM2G110185_P02,GRMZM2G181359_P01	TRUE	TRUE	vSPTDIEEGMR	95%	n+304 (+304)	40.28	25.00
1556	seq=translation; coord=5:212375127..212379000:-1; parent_transcript=GRMZM2G110185_T02; parent_gene=GRMZM2G110185	GRMZM2G110185_P02,GRMZM2G181359_P01	TRUE	TRUE	yQIQIPLPPk	89%	n+304 (+304), K+304 (+304)	25.05	25.00
1557	seq=translation; coord=2:106286427..106287698:1; parent_transcript=GRMZM2G045809_T01; parent_gene=GRMZM2G045809	GRMZM2G045809_P01	TRUE	TRUE	dPMAVTPDDFFNAAMIIDk	95%	n+304 (+304), K+304 (+304)	28.06	25.00
1558	seq=translation; coord=2:106286427..106287698:1; parent_transcript=GRMZM2G045809_T01; parent_gene=GRMZM2G045809	GRMZM2G045809_P01	TRUE	TRUE	kLIDWLQSQFWDTNY	95%	K+304 (+304), n+304 (+304)	39.84	25.53
1559	seq=translation; coord=7:165383929..165386707:-1; parent_transcript=GRMZM2G031545_T01; parent_gene=GRMZM2G031545	GRMZM2G031545_P01,GRMZM2G031545_P02, GRMZM2G031545_P04	TRUE	TRUE	lSGITAEGQGVk	93%	n+304 (+304), K+304 (+304)	28.80	26.40
1560	seq=translation; coord=7:165383929..165386707:-1; parent_transcript=GRMZM2G031545_T01; parent_gene=GRMZM2G031545	GRMZM2G031545_P01,GRMZM2G031545_P02, GRMZM2G031545_P04	TRUE	TRUE	wFNHIDALVR	95%	n+304 (+304)	51.71	25.77
1561	seq=translation; coord=6:125241154..125242106:-1; parent_transcript=GRMZM2G357296_T01; parent_gene=GRMZM2G357296	GRMZM2G357296_P01	TRUE	TRUE	ecPNTEcGAGVFMANHFDR	95%	n+304 (+304), Carbamidomethyl (+57), Carbamidomethyl (+57)	65.46	25.00
1562	seq=translation; coord=6:125241154..125242106:-1; parent_transcript=GRMZM2G357296_T01; parent_gene=GRMZM2G357296	GRMZM2G357296_P01	TRUE	TRUE	lSVLQFYk	95%	n+304 (+304), K+304 (+304)	32.18	25.00
1563	seq=translation; coord=9:98777728..98779013:-1; parent_transcript=GRMZM2G036921_T01; parent_gene=GRMZM2G036921	GRMZM2G036921_P01	TRUE	TRUE	aMGVELDLSDkPVGLGVR	95%	n+304 (+304), K+304 (+304)	36.81	25.00
1564	seq=translation; coord=9:98777728..98779013:-1; parent_transcript=GRMZM2G036921_T01; parent_gene=GRMZM2G036921	GRMZM2G036921_P01	TRUE	TRUE	amGVELDLSDkPVGLGVR	95%	n+304 (+304), Oxidation (+16), K+304 (+304)	32.31	25.11
1565	seq=translation; coord=9:98777728..98779013:-1; parent_transcript=GRMZM2G036921_T01; parent_gene=GRMZM2G036921	GRMZM2G036921_P01	TRUE	TRUE	aVASSASAAPAAAAk	95%	n+304 (+304), K+304 (+304)	46.91	26.83
1566	seq=translation; coord=9:98777728..98779013:-1; parent_transcript=GRMZM2G036921_T01; parent_gene=GRMZM2G036921	GRMZM2G036921_P01	TRUE	TRUE	gVDTVAcVSVNDAFVMR	95%	n+304 (+304), Carbamidomethyl (+57)	69.77	25.00
1567	seq=translation; coord=9:98777728..98779013:-1; parent_transcript=GRMZM2G036921_T01; parent_gene=GRMZM2G036921	GRMZM2G036921_P01	TRUE	TRUE	hLPGFVAk	95%	n+304 (+304), K+304 (+304)	42.07	25.00

1568	seq=translation; coord=9:98777728..98779013:-1; parent_transcript=GRMZM2G036921_T01; parent_gene=GRMZM2G036921	GRMZM2G036921_P01	TRUE	TRUE	vVLFAVPGAFTPTcTQk	95%	n+304 (+304), Carbamidomethyl (+57), K+304 (+304)	32.89	25.16
1569	seq=translation; coord=9:98777728..98779013:-1; parent_transcript=GRMZM2G036921_T01; parent_gene=GRMZM2G036921	GRMZM2G036921_P01	TRUE	TRUE	yALLAEDGVVk	95%	n+304 (+304), K+304 (+304)	64.92	26.01
1570	seq=translation; coord=9:98777728..98779013:-1; parent_transcript=GRMZM2G036921_T01; parent_gene=GRMZM2G036921	GRMZM2G036921_P01	TRUE	TRUE	yALLAEDGVVk	95%	n+304 (+304), iTRAQ8plex (+304), K+304 (+304)	38.44	25.00
1571	seq=translation; coord=3:146602562..146604776:1; parent_transcript=GRMZM2G171430_T01; parent_gene=GRMZM2G171430	GRMZM2G171430_P01	TRUE	TRUE	iILTSDPk	95%	n+304 (+304), K+304 (+304)	29.70	25.75
1572	seq=translation; coord=3:146602562..146604776:1; parent_transcript=GRMZM2G171430_T01; parent_gene=GRMZM2G171430	GRMZM2G171430_P01	TRUE	TRUE	vFSVPEAAPFTAVLk	95%	n+304 (+304), K+304 (+304)	31.57	25.00
1573	seq=translation; coord=3:146602562..146604776:1; parent_transcript=GRMZM2G171430_T01; parent_gene=GRMZM2G171430	GRMZM2G171430_P01	TRUE	TRUE	vPPQTSAIITNDGIGINPQQSAGNVFLk	95%	n+304 (+304), K+304 (+304)	33.89	25.00
1574	seq=translation; coord=3:146602562..146604776:1; parent_transcript=GRMZM2G171430_T01; parent_gene=GRMZM2G171430	GRMZM2G171430_P01	TRUE	TRUE	yAAEEFk	92%	n+304 (+304), K+304 (+304)	26.43	25.00
1575	seq=translation; coord=3:146602562..146604776:1; parent_transcript=GRMZM2G171430_T01; parent_gene=GRMZM2G171430	GRMZM2G171430_P01	TRUE	TRUE	yAAEEFkVPPQTSAIITNDGIGINPQQSAGNVFLk	95%	n+304 (+304), K+304 (+304), K+304 (+304)	28.17	25.00
1576	seq=translation; coord=4:149167730..149173878:1; parent_transcript=GRMZM2G063850_T01; parent_gene=GRMZM2G063850	GRMZM2G063850_P01	TRUE	TRUE	aLELDDEDISYLTNR	95%	n+304 (+304)	64.72	25.00
1577	seq=translation; coord=4:149167730..149173878:1; parent_transcript=GRMZM2G063850_T01; parent_gene=GRMZM2G063850	GRMZM2G063850_P01	TRUE	TRUE	aVETYQAGLk	90%	n+304 (+304), K+304 (+304)	26.31	25.69
1578	seq=translation; coord=4:149167730..149173878:1; parent_transcript=GRMZM2G063850_T01; parent_gene=GRMZM2G063850	GRMZM2G063850_P01	TRUE	TRUE	aYLNEPDFMHMMR	95%	n+304 (+304)	58.31	25.00
1579	seq=translation; coord=4:149167730..149173878:1; parent_transcript=GRMZM2G063850_T01; parent_gene=GRMZM2G063850	GRMZM2G063850_P01	TRUE	TRUE	ayLNEPDFMHMMR	95%	n+304 (+304), iTRAQ8plex (+304)	28.05	25.00
1580	seq=translation; coord=4:149167730..149173878:1; parent_transcript=GRMZM2G063850_T01; parent_gene=GRMZM2G063850	GRMZM2G063850_P01	TRUE	TRUE	dFDIAIETFQk	95%	n+304 (+304), K+304 (+304)	37.33	26.24
1581	seq=translation; coord=4:149167730..149173878:1; parent_transcript=GRMZM2G063850_T01; parent_gene=GRMZM2G063850	GRMZM2G063850_P01	TRUE	TRUE	gLELDPSNEGLk	95%	n+304 (+304), K+304 (+304)	48.68	25.07
1582	seq=translation; coord=4:149167730..149173878:1; parent_transcript=GRMZM2G063850_T01; parent_gene=GRMZM2G063850	GRMZM2G063850_P01	TRUE	TRUE	gSSGPDAIGQMFQGPELWSk	95%	n+304 (+304), K+304 (+304)	39.01	25.00
1583	seq=translation; coord=4:149167730..149173878:1; parent_transcript=GRMZM2G063850_T01; parent_gene=GRMZM2G063850	GRMZM2G063850_P01	TRUE	TRUE	qQQQQQTPPSETk	95%	n+304 (+304), K+304 (+304)	33.84	26.17
1584	seq=translation; coord=4:149167730..149173878:1; parent_transcript=GRMZM2G063850_T01; parent_gene=GRMZM2G063850	GRMZM2G063850_P01	TRUE	TRUE	qVLIDFQENPSAAQEHLk	95%	n+304 (+304), K+304 (+304)	64.57	25.65
1585	seq=translation; coord=4:149167730..149173878:1; parent_transcript=GRMZM2G063850_T01; parent_gene=GRMZM2G063850	GRMZM2G063850_P01	TRUE	TRUE	qVLIDFQENPSAAQEHLk	95%	Pyro-cmC (-17), n+304 (+304), K+304 (+304)	38.36	25.00

1586	seq=translation; coord=4:149167730..149173878:1; parent_transcript=GRMZM2G063850_T01; parent_gene=GRMZM2G063850	GRMZM2G063850_P01	TRUE	TRUE	rGSSGPDAIGQMFQGPELWSk	95%	n+304 (+304), K+304 (+304)	29.67	25.00
1587	seq=translation; coord=5:1488844..1491571:1; parent_transcript=GRMZM2G125271_T01; parent_gene=GRMZM2G125271	GRMZM2G125271_P01	TRUE	TRUE	dAAAQAAANA	95%	n+304 (+304)	53.03	25.00
1588	seq=translation; coord=5:1488844..1491571:1; parent_transcript=GRMZM2G125271_T01; parent_gene=GRMZM2G125271	GRMZM2G125271_P01	TRUE	TRUE	gSFETIHVEDSLGHQFATR	95%	n+304 (+304)	50.93	25.00
1589	seq=translation; coord=9:142495842..142498679:-1; parent_transcript=GRMZM2G067303_T01; parent_gene=GRMZM2G067303	GRMZM2G067303_P01,GRMZM2G067303_P02, GRMZM2G170336_P01,GRMZM2G170336_P02	TRUE	TRUE	lGVEDAPELQLNR	95%	n+304 (+304)	40.01	25.00
1590	seq=translation; coord=9:142495842..142498679:-1; parent_transcript=GRMZM2G067303_T01; parent_gene=GRMZM2G067303	GRMZM2G067303_P01,GRMZM2G067303_P02, GRMZM2G170336_P01,GRMZM2G170336_P02	TRUE	TRUE	vIDLISSPDVVk	95%	n+304 (+304), K+304 (+304)	42.50	25.00
1591	seq=translation; coord=8:162236975..162239554:-1; parent_transcript=GRMZM2G042118_T01; parent_gene=GRMZM2G042118	GRMZM2G042118_P01	TRUE	TRUE	eAFSSFGEVTEAR	95%	n+304 (+304)	44.14	25.00
1592	seq=translation; coord=8:162236975..162239554:-1; parent_transcript=GRMZM2G042118_T01; parent_gene=GRMZM2G042118	GRMZM2G042118_P01	TRUE	TRUE	eAISAMDGk	95%	n+304 (+304), K+304 (+304)	36.54	25.90
1593	seq=translation; coord=8:162236975..162239554:-1; parent_transcript=GRMZM2G042118_T01; parent_gene=GRMZM2G042118	GRMZM2G042118_P01	TRUE	TRUE	gFGFVNYSDSDAAk	95%	n+304 (+304), K+304 (+304)	47.76	25.00
1594	seq=translation; coord=8:162236975..162239554:-1; parent_transcript=GRMZM2G042118_T01; parent_gene=GRMZM2G042118	GRMZM2G042118_P01	TRUE	TRUE	gGGGYGGGGYGGGGyGGGGGYGGGGYGGGSQSYDA	95%	n+304 (+304), iTRAQ8plex (+304)	44.08	25.00
1595	seq=translation; coord=8:162236975..162239554:-1; parent_transcript=GRMZM2G042118_T01; parent_gene=GRMZM2G042118	GRMZM2G042118_P01	TRUE	TRUE	lFIGGLDWGVDDVk	95%	n+304 (+304), K+304 (+304)	50.38	26.35
1596	seq=translation; coord=1:258363419..258365809:1; parent_transcript=GRMZM2G067919_T02; parent_gene=GRMZM2G067919	GRMZM2G067919_P02	TRUE	TRUE	aEEVDEVLGSR	95%	n+304 (+304)	44.26	25.00
1597	seq=translation; coord=1:258363419..258365809:1; parent_transcript=GRMZM2G067919_T02; parent_gene=GRMZM2G067919	GRMZM2G067919_P02	TRUE	TRUE	aLSFASk	88%	n+304 (+304), K+304 (+304)	26.22	25.73
1598	seq=translation; coord=1:258363419..258365809:1; parent_transcript=GRMZM2G067919_T02; parent_gene=GRMZM2G067919	GRMZM2G067919_P02	TRUE	TRUE	dSNLQIVcFEVHADR	95%	n+304 (+304), Carbamidomethyl (+57)	70.26	25.00
1599	seq=translation; coord=1:258363419..258365809:1; parent_transcript=GRMZM2G067919_T02; parent_gene=GRMZM2G067919	GRMZM2G067919_P02	TRUE	TRUE	iLHTISVPGEFQFFFGPGGR	95%	n+304 (+304)	52.36	26.20
1600	seq=translation; coord=1:258363419..258365809:1; parent_transcript=GRMZM2G067919_T02; parent_gene=GRMZM2G067919	GRMZM2G067919_P02	TRUE	TRUE	lSPGTAFVVPAGHPFVAVASR	95%	n+304 (+304)	45.87	25.56
1601	seq=translation; coord=1:258363419..258365809:1; parent_transcript=GRMZM2G067919_T02; parent_gene=GRMZM2G067919	GRMZM2G067919_P02	TRUE	TRUE	nPESFLSSFSk	92%	n+304 (+304), K+304 (+304)	27.47	25.53
1602	seq=translation; coord=1:258363419..258365809:1; parent_transcript=GRMZM2G067919_T02; parent_gene=GRMZM2G067919	GRMZM2G067919_P02	TRUE	TRUE	qGHVFVAPAGAVTYLANTDGR	95%	n+304 (+304)	86.91	25.00
1603	seq=translation; coord=1:258363419..258365809:1; parent_transcript=GRMZM2G067919_T02; parent_gene=GRMZM2G067919	GRMZM2G067919_P02	TRUE	TRUE	qGHVFVAPAGAVTYLANTDGR	95%	Pyro-cmC (-17), n+304 (+304)	92.28	25.00

1604	seq=translation; coord=1:258363419..258365809:1; parent_transcript=GRMZM2G067919_T02; parent_gene=GRMZM2G067919	GRMZM2G067919_P02	TRUE	TRUE	qGHVFVAPAGAVTyLANTDGR	95%	Pyro-cmC (-17), n+304 (+304), iTRAQ8plex (+304)	30.21	25.00
1605	seq=translation; coord=1:258363419..258365809:1; parent_transcript=GRMZM2G067919_T02; parent_gene=GRMZM2G067919	GRMZM2G067919_P02	TRUE	TRUE	sEEEEEESSEEQEEAGQGYHTIR	95%	n+304 (+304)	80.50	25.00
1606	seq=translation; coord=1:258363419..258365809:1; parent_transcript=GRMZM2G067919_T02; parent_gene=GRMZM2G067919	GRMZM2G067919_P02	TRUE	TRUE	vFLAGADNVLQk	95%	n+304 (+304), K+304 (+304)	54.06	25.00
1607	seq=translation; coord=8:18231012..18236007:-1; parent_transcript=GRMZM2G107639_T01; parent_gene=GRMZM2G107639	GRMZM2G107639_P01,GRMZM2G107639_P02	TRUE	TRUE	aLLVEALSPLGEDNVk	95%	n+304 (+304), K+304 (+304)	50.16	25.00
1608	seq=translation; coord=8:18231012..18236007:-1; parent_transcript=GRMZM2G107639_T01; parent_gene=GRMZM2G107639	GRMZM2G107639_P01,GRMZM2G107639_P02	TRUE	TRUE	aVETDAPVMVk	95%	n+304 (+304), K+304 (+304)	42.16	26.71
1609	seq=translation; coord=8:18231012..18236007:-1; parent_transcript=GRMZM2G107639_T01; parent_gene=GRMZM2G107639	GRMZM2G107639_P01,GRMZM2G107639_P02	TRUE	TRUE	dVMSLAQGVVYWQPPESAMDk	95%	n+304 (+304), K+304 (+304)	30.50	25.00
1610	seq=translation; coord=8:18231012..18236007:-1; parent_transcript=GRMZM2G107639_T01; parent_gene=GRMZM2G107639	GRMZM2G107639_P01,GRMZM2G107639_P02	TRUE	TRUE	gGEGAIYLWAk	95%	n+304 (+304), K+304 (+304)	49.47	25.29
1611	seq=translation; coord=8:18231012..18236007:-1; parent_transcript=GRMZM2G107639_T01; parent_gene=GRMZM2G107639	GRMZM2G107639_P01,GRMZM2G107639_P02	TRUE	TRUE	iQELLR	90%	n+304 (+304)	27.42	25.00
1612	seq=translation; coord=8:18231012..18236007:-1; parent_transcript=GRMZM2G107639_T01; parent_gene=GRMZM2G107639	GRMZM2G107639_P01,GRMZM2G107639_P02	TRUE	TRUE	lALYSLEAGPEWIk	95%	n+304 (+304), K+304 (+304)	44.24	25.00
1613	seq=translation; coord=8:18231012..18236007:-1; parent_transcript=GRMZM2G107639_T01; parent_gene=GRMZM2G107639	GRMZM2G107639_P01,GRMZM2G107639_P02	TRUE	TRUE	lPDScSDDFEAVR	95%	n+304 (+304), Carbamidomethyl (+57)	68.19	25.00
1614	seq=translation; coord=8:18231012..18236007:-1; parent_transcript=GRMZM2G107639_T01; parent_gene=GRMZM2G107639	GRMZM2G107639_P01,GRMZM2G107639_P02	TRUE	TRUE	rGLQELVTDGMVQ	95%	n+304 (+304)	39.54	25.71
1615	seq=translation; coord=8:18231012..18236007:-1; parent_transcript=GRMZM2G107639_T01; parent_gene=GRMZM2G107639	GRMZM2G107639_P01,GRMZM2G107639_P02	TRUE	TRUE	tLHPDVDWLEk	95%	n+304 (+304), K+304 (+304)	32.77	26.16
1616	seq=translation; coord=8:18231012..18236007:-1; parent_transcript=GRMZM2G107639_T01; parent_gene=GRMZM2G107639	GRMZM2G107639_P01,GRMZM2G107639_P02	TRUE	TRUE	vGYIAFPNEADGFHDQLLk	94%	n+304 (+304), K+304 (+304)	28.76	25.62
1617	seq=translation; coord=8:18231012..18236007:-1; parent_transcript=GRMZM2G107639_T01; parent_gene=GRMZM2G107639	GRMZM2G107639_P01,GRMZM2G107639_P02	TRUE	TRUE	vQDNIPIcASIIGQR	95%	n+304 (+304), Carbamidomethyl (+57)	48.93	26.04
1618	seq=translation; coord=8:18231012..18236007:-1; parent_transcript=GRMZM2G107639_T01; parent_gene=GRMZM2G107639	GRMZM2G107639_P01,GRMZM2G107639_P02	TRUE	TRUE	vSFGGLk	95%	n+304 (+304), K+304 (+304)	29.83	26.19
1619	seq=translation; coord=8:18231012..18236007:-1; parent_transcript=GRMZM2G107639_T01; parent_gene=GRMZM2G107639	GRMZM2G107639_P01,GRMZM2G107639_P02	TRUE	TRUE	yGSDDGLPELR	92%	n+304 (+304)	28.48	25.00
1620	seq=translation; coord=5:37229486..37234332:-1; parent_transcript=GRMZM2G088064_T03; parent_gene=GRMZM2G088064	GRMZM2G088064_P03	TRUE	TRUE	aFHEAFLAEYRD	95%	n+304 (+304)	40.39	25.00
1621	seq=translation; coord=5:37229486..37234332:-1; parent_transcript=GRMZM2G088064_T03; parent_gene=GRMZM2G088064	GRMZM2G088064_P03	TRUE	TRUE	aLEDAFNk	95%	n+304 (+304), K+304 (+304)	33.72	26.18

1622	seq=translation; coord=5:37229486..37234332:-1; parent_transcript=GRMZM2G088064_T03; parent_gene=GRMZM2G088064	GRMZM2G088064_P03	TRUE	TRUE	aLVVINPGNPTGQVLAEDNQYDIVk	95%	n+304 (+304), K+304 (+304)	34.83	25.00
1623	seq=translation; coord=5:37229486..37234332:-1; parent_transcript=GRMZM2G088064_T03; parent_gene=GRMZM2G088064	GRMZM2G088064_P03	TRUE	TRUE	dAIAAGIMSR	95%	n+304 (+304)	32.38	25.00
1624	seq=translation; coord=5:37229486..37234332:-1; parent_transcript=GRMZM2G088064_T03; parent_gene=GRMZM2G088064	GRMZM2G088064_P03	TRUE	TRUE	eVLALcDHPcLLEk	95%	n+304 (+304), Carbamidomethyl (+57), Carbamidomethyl (+57), K+304 (+304)	38.37	26.28
1625	seq=translation; coord=5:37229486..37234332:-1; parent_transcript=GRMZM2G088064_T03; parent_gene=GRMZM2G088064	GRMZM2G088064_P03	TRUE	TRUE	gEIVIHAQR	91%	n+304 (+304)	26.55	26.14
1626	seq=translation; coord=5:37229486..37234332:-1; parent_transcript=GRMZM2G088064_T03; parent_gene=GRMZM2G088064	GRMZM2G088064_P03	TRUE	TRUE	gGYMEITGFSAPVR	95%	n+304 (+304)	65.54	25.00
1627	seq=translation; coord=5:37229486..37234332:-1; parent_transcript=GRMZM2G088064_T03; parent_gene=GRMZM2G088064	GRMZM2G088064_P03	TRUE	TRUE	lLESTGIVVVPGSGFGQVPGTWHIR	95%	n+304 (+304)	65.44	25.00
1628	seq=translation; coord=5:37229486..37234332:-1; parent_transcript=GRMZM2G088064_T03; parent_gene=GRMZM2G088064	GRMZM2G088064_P03	TRUE	TRUE	nEGLVLLADEVYQENIYVDNk	95%	n+304 (+304), K+304 (+304)	64.68	25.20
1629	seq=translation; coord=5:37229486..37234332:-1; parent_transcript=GRMZM2G088064_T03; parent_gene=GRMZM2G088064	GRMZM2G088064_P03	TRUE	TRUE	rGGYMEITGFSAPVR	95%	n+304 (+304)	51.41	25.44
1630	seq=translation; coord=5:37229486..37234332:-1; parent_transcript=GRMZM2G088064_T03; parent_gene=GRMZM2G088064	GRMZM2G088064_P03	TRUE	TRUE	sLFSADAISR	95%	n+304 (+304)	37.22	25.00
1631	seq=translation; coord=5:37229486..37234332:-1; parent_transcript=GRMZM2G088064_T03; parent_gene=GRMZM2G088064	GRMZM2G088064_P03	TRUE	TRUE	tGWGLEISDLk	94%	n+304 (+304), K+304 (+304)	31.21	25.98
1632	seq=translation; coord=5:37229486..37234332:-1; parent_transcript=GRMZM2G088064_T03; parent_gene=GRMZM2G088064	GRMZM2G088064_P03	TRUE	TRUE	vGDESYASYk	95%	n+304 (+304), K+304 (+304)	42.79	25.00
1633	seq=translation; coord=6:34430048..34433142:1; parent_transcript=GRMZM2G035502_T01; parent_gene=GRMZM2G035502	GRMZM2G035502_P01	TRUE	TRUE	aATGkPDTLGDcPFSQR	93%	n+304 (+304), K+304 (+304), Carbamidomethyl (+57)	27.78	25.00
1634	seq=translation; coord=6:34430048..34433142:1; parent_transcript=GRMZM2G035502_T01; parent_gene=GRMZM2G035502	GRMZM2G035502_P01	TRUE	TRUE	aLLDELQALDEHLk	95%	n+304 (+304), K+304 (+304)	72.83	25.00
1635	seq=translation; coord=6:34430048..34433142:1; parent_transcript=GRMZM2G035502_T01; parent_gene=GRMZM2G035502	GRMZM2G035502_P01	TRUE	TRUE	fPTPSLVTPVEYASVGSk	95%	n+304 (+304), K+304 (+304)	59.25	25.98
1636	seq=translation; coord=6:34430048..34433142:1; parent_transcript=GRMZM2G035502_T01; parent_gene=GRMZM2G035502	GRMZM2G035502_P01	TRUE	TRUE	iFPAFITFLk	95%	n+304 (+304), K+304 (+304)	36.98	25.00
1637	seq=translation; coord=6:34430048..34433142:1; parent_transcript=GRMZM2G035502_T01; parent_gene=GRMZM2G035502	GRMZM2G035502_P01	TRUE	TRUE	lFHLQVALEHFk	95%	n+304 (+304), K+304 (+304)	32.34	25.00
1638	seq=translation; coord=4:70066543..70068959:-1; parent_transcript=GRMZM2G073079_T01; parent_gene=GRMZM2G073079	GRMZM2G073079_P01,GRMZM2G076348_P01	TRUE	TRUE	aGYFVVVPDFLk	95%	n+304 (+304), K+304 (+304)	37.03	25.44

1639	seq=translation; coord=4:70066543..70068959:-1; parent_transcript=GRMZM2G073079_T01; parent_gene=GRMZM2G073079	GRMZM2G073079_P01,GRMZM2G076348_P01	TRUE	TRUE	aVcLSHPYSVTADDMk	95%	n+304 (+304), Carbamidomethyl (+57), K+304 (+304)	47.83	25.00
1640	seq=translation; coord=4:70066543..70068959:-1; parent_transcript=GRMZM2G073079_T01; parent_gene=GRMZM2G073079	GRMZM2G073079_P01,GRMZM2G076348_P01	TRUE	TRUE	aVVLASDVFGYEAPLLR	95%	n+304 (+304)	67.00	25.67
1641	seq=translation; coord=4:70066543..70068959:-1; parent_transcript=GRMZM2G073079_T01; parent_gene=GRMZM2G073079	GRMZM2G073079_P01,GRMZM2G076348_P01	TRUE	TRUE	aVVLASDVFGyEAPLLR	95%	n+304 (+304), iTRAQ8plex (+304)	31.84	25.19
1642	seq=translation; coord=4:70066543..70068959:-1; parent_transcript=GRMZM2G073079_T01; parent_gene=GRMZM2G073079	GRMZM2G073079_P01,GRMZM2G076348_P01	TRUE	TRUE	aYVSGAASSSR	95%	n+304 (+304)	39.60	25.00
1643	seq=translation; coord=4:70066543..70068959:-1; parent_transcript=GRMZM2G073079_T01; parent_gene=GRMZM2G073079	GRMZM2G073079_P01,GRMZM2G076348_P01	TRUE	TRUE	fVHVLR	92%	n+304 (+304)	26.88	25.24
1644	seq=translation; coord=4:70066543..70068959:-1; parent_transcript=GRMZM2G073079_T01; parent_gene=GRMZM2G073079	GRMZM2G073079_P01,GRMZM2G076348_P01	TRUE	TRUE	iFQGVEHGFAcR	95%	n+304 (+304), Carbamidomethyl (+57)	46.10	25.00
1645	seq=translation; coord=4:70066543..70068959:-1; parent_transcript=GRMZM2G073079_T01; parent_gene=GRMZM2G073079	GRMZM2G073079_P01,GRMZM2G076348_P01	TRUE	TRUE	tAETALAYMVSWFNk	95%	n+304 (+304), K+304 (+304)	42.77	25.00
1646	seq=translation; coord=7:8218240..8220274:1; parent_transcript=GRMZM2G170397_T01; parent_gene=GRMZM2G170397	GRMZM2G170397_P01,GRMZM2G170397_P02, GRMZM2G170397_P03	TRUE	TRUE	mEAVGSQSGATAESVR	95%	n+304 (+304)	85.57	25.00
1647	seq=translation; coord=7:8218240..8220274:1; parent_transcript=GRMZM2G170397_T01; parent_gene=GRMZM2G170397	GRMZM2G170397_P01,GRMZM2G170397_P02, GRMZM2G170397_P03	TRUE	TRUE	mEAVGSQSGATAESVR	95%	Oxidation (+16), n+304 (+304)	36.93	25.00
1648	seq=translation; coord=7:8218240..8220274:1; parent_transcript=GRMZM2G170397_T01; parent_gene=GRMZM2G170397	GRMZM2G170397_P01,GRMZM2G170397_P02, GRMZM2G170397_P03	TRUE	TRUE	vFFDILIGk	95%	n+304 (+304), K+304 (+304)	32.20	25.00
1649	seq=translation; coord=7:8218240..8220274:1; parent_transcript=GRMZM2G170397_T01; parent_gene=GRMZM2G170397	GRMZM2G170397_P01,GRMZM2G170397_P02, GRMZM2G170397_P03	TRUE	TRUE	vIPGFMcQGGDFTR	95%	n+304 (+304), Carbamidomethyl (+57)	37.70	25.00
1650	seq=translation; coord=7:8218240..8220274:1; parent_transcript=GRMZM2G170397_T01; parent_gene=GRMZM2G170397	GRMZM2G170397_P01,GRMZM2G170397_P02, GRMZM2G170397_P03	TRUE	TRUE	vVDGYAVVDk	95%	n+304 (+304), K+304 (+304)	46.88	25.76
1651	seq=translation; coord=7:8218240..8220274:1; parent_transcript=GRMZM2G170397_T01; parent_gene=GRMZM2G170397	GRMZM2G170397_P01,GRMZM2G170397_P02, GRMZM2G170397_P03	TRUE	TRUE	vVMELFADk	95%	n+304 (+304), K+304 (+304)	31.26	26.51
1652	seq=translation; coord=9:148445803..148448625:-1; parent_transcript=GRMZM2G078441_T01; parent_gene=GRMZM2G078441	GRMZM2G078441_P01	TRUE	TRUE	eGDVFVVPR	95%	n+304 (+304)	33.22	25.00
1653	seq=translation; coord=9:148445803..148448625:-1; parent_transcript=GRMZM2G078441_T01; parent_gene=GRMZM2G078441	GRMZM2G078441_P01	TRUE	TRUE	eSTILPcVScAEELAEk	95%	n+304 (+304), Carbamidomethyl (+57), Carbamidomethyl (+57), K+304 (+304)	54.16	25.00
1654	seq=translation; coord=9:148445803..148448625:-1; parent_transcript=GRMZM2G078441_T01; parent_gene=GRMZM2G078441	GRMZM2G078441_P01	TRUE	TRUE	eSTILPcVScAEELAEkAEEER	95%	n+304 (+304), Carbamidomethyl (+57), Carbamidomethyl (+57), K+304 (+304)	55.00	25.00
1655	seq=translation; coord=9:148445803..148448625:-1; parent_transcript=GRMZM2G078441_T01; parent_gene=GRMZM2G078441	GRMZM2G078441_P01	TRUE	TRUE	gDVYNFEQGSILYIQSyPNASR	95%	n+304 (+304), iTRAQ8plex (+304)	42.49	25.00

1656	seq=translation; coord=9:148445803..148448625:-1; parent_transcript=GRMZM2G078441_T01; parent_gene=GRMZM2G078441	GRMZM2G078441_P01	TRUE	TRUE	gFETDVLR	95%	n+304 (+304)	32.06	25.00
1657	seq=translation; coord=9:148445803..148448625:-1; parent_transcript=GRMZM2G078441_T01; parent_gene=GRMZM2G078441	GRMZM2G078441_P01	TRUE	TRUE	gkVTSIEEESSEQSSLEVER	95%	n+304 (+304), K+304 (+304)	85.94	25.00
1658	seq=translation; coord=9:148445803..148448625:-1; parent_transcript=GRMZM2G078441_T01; parent_gene=GRMZM2G078441	GRMZM2G078441_P01	TRUE	TRUE	gSVLQAIGk	95%	n+304 (+304), K+304 (+304)	41.32	25.00
1659	seq=translation; coord=9:148445803..148448625:-1; parent_transcript=GRMZM2G078441_T01; parent_gene=GRMZM2G078441	GRMZM2G078441_P01	TRUE	TRUE	vkEGDVFVVPR	95%	n+304 (+304), K+304 (+304)	33.04	25.00
1660	seq=translation; coord=9:148445803..148448625:-1; parent_transcript=GRMZM2G078441_T01; parent_gene=GRMZM2G078441	GRMZM2G078441_P01	TRUE	TRUE	vLALALGQR	87%	n+304 (+304)	25.67	25.00
1661	seq=translation; coord=9:148445803..148448625:-1; parent_transcript=GRMZM2G078441_T01; parent_gene=GRMZM2G078441	GRMZM2G078441_P01	TRUE	TRUE	vTSIEEESSEQSSLEVER	95%	n+304 (+304)	85.84	25.00
1662	seq=translation; coord=9:148445803..148448625:-1; parent_transcript=GRMZM2G078441_T01; parent_gene=GRMZM2G078441	GRMZM2G078441_P01	TRUE	TRUE	vVAESEAGSVSAVDVADAAGTAYR	95%	n+304 (+304)	99.04	25.00
1663	seq=translation; coord=3:218323126..218325508:1; parent_transcript=GRMZM2G176903_T01; parent_gene=GRMZM2G176903	GRMZM2G176903_P01,GRMZM2G176903_P02	TRUE	TRUE	fGIGQGAAGMTVcGTPEYVR	95%	n+304 (+304), Carbamidomethyl (+57)	54.49	25.00
1664	seq=translation; coord=3:218323126..218325508:1; parent_transcript=GRMZM2G176903_T01; parent_gene=GRMZM2G176903	GRMZM2G176903_P01,GRMZM2G176903_P02	TRUE	TRUE	fTTESLEk	91%	n+304 (+304), K+304 (+304)	28.26	25.37
1665	seq=translation; coord=3:218323126..218325508:1; parent_transcript=GRMZM2G176903_T01; parent_gene=GRMZM2G176903	GRMZM2G176903_P01,GRMZM2G176903_P02	TRUE	TRUE	gITFFDTSDAYGPR	95%	n+304 (+304)	62.68	25.00
1666	seq=translation; coord=3:218323126..218325508:1; parent_transcript=GRMZM2G176903_T01; parent_gene=GRMZM2G176903	GRMZM2G176903_P01,GRMZM2G176903_P02	TRUE	TRUE	iEDLAk	91%	n+304 (+304), K+304 (+304)	27.36	26.90
1667	seq=translation; coord=3:218323126..218325508:1; parent_transcript=GRMZM2G176903_T01; parent_gene=GRMZM2G176903	GRMZM2G176903_P01,GRMZM2G176903_P02	TRUE	TRUE	kYQcSPAQLALAWVLR	95%	K+304 (+304), n+304 (+304), Carbamidomethyl (+57)	61.29	25.00
1668	seq=translation; coord=3:218323126..218325508:1; parent_transcript=GRMZM2G176903_T01; parent_gene=GRMZM2G176903	GRMZM2G176903_P01,GRMZM2G176903_P02	TRUE	TRUE	lDAGYIDLYyQHR	95%	n+304 (+304), iTRAQ8plex (+304)	27.78	25.25
1669	seq=translation; coord=3:218323126..218325508:1; parent_transcript=GRMZM2G176903_T01; parent_gene=GRMZM2G176903	GRMZM2G176903_P01,GRMZM2G176903_P02	TRUE	TRUE	lGFGcMGLTGTYNAPLGDEAVAAVVAHAFR	95%	n+304 (+304), Carbamidomethyl (+57)	74.65	25.00
1670	seq=translation; coord=3:218323126..218325508:1; parent_transcript=GRMZM2G176903_T01; parent_gene=GRMZM2G176903	GRMZM2G176903_P01,GRMZM2G176903_P02	TRUE	TRUE	yQcSPAQLALAWVLR	95%	n+304 (+304), Carbamidomethyl (+57)	60.09	25.74
1671	seq=translation; coord=5:205777032..205781614:-1; parent_transcript=GRMZM2G140150_T01; parent_gene=GRMZM2G140150	GRMZM2G140150_P01	TRUE	TRUE	gFNVESFGPDR	95%	n+304 (+304)	46.25	25.00
1672	seq=translation; coord=5:63564993..63569468:1; parent_transcript=GRMZM2G088212_T01; parent_gene=GRMZM2G088212	GRMZM2G088212_P01	TRUE	TRUE	aPGVQTPVIVR	87%	n+304 (+304)	25.63	25.00
1673	seq=translation; coord=5:63564993..63569468:1; parent_transcript=GRMZM2G088212_T01; parent_gene=GRMZM2G088212	GRMZM2G088212_P01	TRUE	TRUE	dEEVNYFPSR	95%	n+304 (+304)	38.09	25.00

1674	seq=translation; coord=5:63564993..63569468:1; parent_transcript=GRMZM2G088212_T01; parent_gene=GRMZM2G088212	GRMZM2G088212_P01	TRUE	TRUE	eGNFDLVGNNMPVFFIR	95%	n+304 (+304)	48.78	25.00
1675	seq=translation; coord=5:63564993..63569468:1; parent_transcript=GRMZM2G088212_T01; parent_gene=GRMZM2G088212	GRMZM2G088212_P01	TRUE	TRUE	gFFEVTHDVSHLTcADFLR	95%	n+304 (+304), Carbamidomethyl (+57)	57.04	25.00
1676	seq=translation; coord=5:63564993..63569468:1; parent_transcript=GRMZM2G088212_T01; parent_gene=GRMZM2G088212	GRMZM2G088212_P01	TRUE	TRUE	hMEGFGVNTYTLINR	95%	n+304 (+304)	63.66	25.00
1677	seq=translation; coord=5:63564993..63569468:1; parent_transcript=GRMZM2G088212_T01; parent_gene=GRMZM2G088212	GRMZM2G088212_P01	TRUE	TRUE	hMEGFGVNTyTLINR	94%	n+304 (+304), iTRAQ8plex (+304)	26.28	26.01
1678	seq=translation; coord=5:63564993..63569468:1; parent_transcript=GRMZM2G088212_T01; parent_gene=GRMZM2G088212	GRMZM2G088212_P01	TRUE	TRUE	lGPNYLMLPVNAPk	91%	n+304 (+304), K+304 (+304)	25.39	25.09
1679	seq=translation; coord=5:63564993..63569468:1; parent_transcript=GRMZM2G088212_T01; parent_gene=GRMZM2G088212	GRMZM2G088212_P01	TRUE	TRUE	tNLQENWR	92%	n+304 (+304)	28.71	25.07
1680	seq=translation; coord=5:63564993..63569468:1; parent_transcript=GRMZM2G088212_T01; parent_gene=GRMZM2G088212	GRMZM2G088212_P01	TRUE	TRUE	tWPEDIIPLQPVGR	93%	n+304 (+304)	28.08	25.60
1681	seq=translation; coord=5:63564993..63569468:1; parent_transcript=GRMZM2G088212_T01; parent_gene=GRMZM2G088212	GRMZM2G088212_P01	TRUE	TRUE	wVDALTDPR	95%	n+304 (+304)	32.87	25.00
1682	seq=translation; coord=6:161659850..161663090:-1; parent_transcript=GRMZM2G121186_T01; parent_gene=GRMZM2G121186	GRMZM2G121186_P01	TRUE	TRUE	eSAAETPEEQk	95%	n+304 (+304), K+304 (+304)	30.78	25.00
1683	seq=translation; coord=6:161659850..161663090:-1; parent_transcript=GRMZM2G121186_T01; parent_gene=GRMZM2G121186	GRMZM2G121186_P01	TRUE	TRUE	hTDVLENLEPk	95%	n+304 (+304), K+304 (+304)	57.35	26.00
1684	seq=translation; coord=4:27749082..27753340:1; parent_transcript=GRMZM2G131943_T01; parent_gene=GRMZM2G131943	GRMZM2G131943_P01,GRMZM2G131943_P02, GRMZM2G131943_P03,GRMZM2G131943_P04	TRUE	TRUE	aAAVAAAPAAVPk	93%	n+304 (+304), K+304 (+304)	26.24	25.00
1685	seq=translation; coord=4:27749082..27753340:1; parent_transcript=GRMZM2G131943_T01; parent_gene=GRMZM2G131943	GRMZM2G131943_P01,GRMZM2G131943_P02, GRMZM2G131943_P03,GRMZM2G131943_P04	TRUE	TRUE	aEFDDVk	95%	n+304 (+304), K+304 (+304)	33.06	25.47
1686	seq=translation; coord=4:27749082..27753340:1; parent_transcript=GRMZM2G131943_T01; parent_gene=GRMZM2G131943	GRMZM2G131943_P01,GRMZM2G131943_P02, GRMZM2G131943_P03,GRMZM2G131943_P04	TRUE	TRUE	aLELSGSDIGGGYELYVDEAkPR	95%	n+304 (+304), K+304 (+304)	76.67	25.00
1687	seq=translation; coord=4:27749082..27753340:1; parent_transcript=GRMZM2G131943_T01; parent_gene=GRMZM2G131943	GRMZM2G131943_P01,GRMZM2G131943_P02, GRMZM2G131943_P03,GRMZM2G131943_P04	TRUE	TRUE	aPAATIk	91%	n+304 (+304), K+304 (+304)	29.82	27.95
1688	seq=translation; coord=4:27749082..27753340:1; parent_transcript=GRMZM2G131943_T01; parent_gene=GRMZM2G131943	GRMZM2G131943_P01,GRMZM2G131943_P02, GRMZM2G131943_P03,GRMZM2G131943_P04	TRUE	TRUE	eFFEDVGEVVDVR	95%	n+304 (+304)	56.04	25.00
1689	seq=translation; coord=4:27749082..27753340:1; parent_transcript=GRMZM2G131943_T01; parent_gene=GRMZM2G131943	GRMZM2G131943_P01,GRMZM2G131943_P02, GRMZM2G131943_P03,GRMZM2G131943_P04	TRUE	TRUE	fPTHDDGNR	95%	n+304 (+304)	32.51	25.00
1690	seq=translation; coord=4:27749082..27753340:1; parent_transcript=GRMZM2G131943_T01; parent_gene=GRMZM2G131943	GRMZM2G131943_P01,GRMZM2G131943_P02, GRMZM2G131943_P03,GRMZM2G131943_P04	TRUE	TRUE	gFcYVEFVSAEAAAk	95%	n+304 (+304), Carbamidomethyl (+57), K+304 (+304)	43.23	25.00
1691	seq=translation; coord=4:27749082..27753340:1; parent_transcript=GRMZM2G131943_T01; parent_gene=GRMZM2G131943	GRMZM2G131943_P01,GRMZM2G131943_P02, GRMZM2G131943_P03,GRMZM2G131943_P04	TRUE	TRUE	gMAYIDFk	95%	n+304 (+304), K+304 (+304)	39.88	25.00

1692	seq=translation; coord=4:27749082..27753340:1; parent_transcript=GRMZM2G131943_T01; parent_gene=GRMZM2G131943	GRMZM2G131943_P01,GRMZM2G131943_P02, GRMZM2G131943_P03,GRMZM2G131943_P04	TRUE	TRUE	gNSSSIFIR	94%	n+304 (+304)	29.06	25.00
1693	seq=translation; coord=4:27749082..27753340:1; parent_transcript=GRMZM2G131943_T01; parent_gene=GRMZM2G131943	GRMZM2G131943_P01,GRMZM2G131943_P02, GRMZM2G131943_P03,GRMZM2G131943_P04	TRUE	TRUE	nLSEDEIR	95%	n+304 (+304)	33.21	25.00
1694	seq=translation; coord=4:27749082..27753340:1; parent_transcript=GRMZM2G131943_T01; parent_gene=GRMZM2G131943	GRMZM2G131943_P01,GRMZM2G131943_P02, GRMZM2G131943_P03,GRMZM2G131943_P04	TRUE	TRUE	sGNDGSFQk	94%	n+304 (+304), K+304 (+304)	27.90	25.39
1695	seq=translation; coord=10:61794133..61802175:-1; parent_transcript=GRMZM2G146115_T01; parent_gene=GRMZM2G146115	GRMZM2G146115_P01,GRMZM2G146115_P02	TRUE	TRUE	aFEDAEITHVDDTVDPVR	95%	n+304 (+304)	81.20	25.00
1696	seq=translation; coord=10:61794133..61802175:-1; parent_transcript=GRMZM2G146115_T01; parent_gene=GRMZM2G146115	GRMZM2G146115_P01,GRMZM2G146115_P02	TRUE	TRUE	aPQAAGAIHTDFER	95%	n+304 (+304)	63.19	25.00
1697	seq=translation; coord=10:61794133..61802175:-1; parent_transcript=GRMZM2G146115_T01; parent_gene=GRMZM2G146115	GRMZM2G146115_P01,GRMZM2G146115_P02	TRUE	TRUE	aVDGIFHVLR	95%	n+304 (+304)	57.58	25.76
1698	seq=translation; coord=10:61794133..61802175:-1; parent_transcript=GRMZM2G146115_T01; parent_gene=GRMZM2G146115	GRMZM2G146115_P01,GRMZM2G146115_P02	TRUE	TRUE	dMETISEELR	95%	n+304 (+304)	33.44	25.00
1699	seq=translation; coord=10:61794133..61802175:-1; parent_transcript=GRMZM2G146115_T01; parent_gene=GRMZM2G146115	GRMZM2G146115_P01,GRMZM2G146115_P02	TRUE	TRUE	gAHAGDGLGNAFLSHIR	95%	n+304 (+304)	48.25	25.20
1700	seq=translation; coord=10:61794133..61802175:-1; parent_transcript=GRMZM2G146115_T01; parent_gene=GRMZM2G146115	GRMZM2G146115_P01,GRMZM2G146115_P02	TRUE	TRUE	gFIcAEVMk	95%	n+304 (+304), Carbamidomethyl (+57), K+304 (+304)	35.18	26.53
1701	seq=translation; coord=10:61794133..61802175:-1; parent_transcript=GRMZM2G146115_T01; parent_gene=GRMZM2G146115	GRMZM2G146115_P01,GRMZM2G146115_P02	TRUE	TRUE	iGIVGLPNVGk	95%	n+304 (+304), K+304 (+304)	39.73	25.00
1702	seq=translation; coord=10:61794133..61802175:-1; parent_transcript=GRMZM2G146115_T01; parent_gene=GRMZM2G146115	GRMZM2G146115_P01,GRMZM2G146115_P02	TRUE	TRUE	lVDMPEDEAAk	94%	n+304 (+304), K+304 (+304)	28.42	25.00
1703	seq=translation; coord=10:61794133..61802175:-1; parent_transcript=GRMZM2G146115_T01; parent_gene=GRMZM2G146115	GRMZM2G146115_P01,GRMZM2G146115_P02	TRUE	TRUE	sEVPAYLEVTDIAGLIR	95%	n+304 (+304)	39.21	26.19
1704	seq=translation; coord=10:61794133..61802175:-1; parent_transcript=GRMZM2G146115_T01; parent_gene=GRMZM2G146115	GRMZM2G146115_P01,GRMZM2G146115_P02	TRUE	TRUE	tGFAAIHLIYFFTAGPDEVk	95%	n+304 (+304), K+304 (+304)	30.63	25.40
1705	seq=translation; coord=9:123775912..123778018:1; parent_transcript=GRMZM5G881775_T02; parent_gene=GRMZM5G881775	GRMZM5G881775_P02	TRUE	TRUE	dLDQVAGR	86%	n+304 (+304)	25.34	25.00
1706	seq=translation; coord=9:123775912..123778018:1; parent_transcript=GRMZM5G881775_T02; parent_gene=GRMZM5G881775	GRMZM5G881775_P02	TRUE	TRUE	dVNPHEFVk	95%	n+304 (+304), K+304 (+304)	41.19	25.53
1707	seq=translation; coord=9:123775912..123778018:1; parent_transcript=GRMZM5G881775_T02; parent_gene=GRMZM5G881775	GRMZM5G881775_P02	TRUE	TRUE	eLPPYDPDWYYIR	95%	n+304 (+304)	38.75	25.00
1708	seq=translation; coord=9:123775912..123778018:1; parent_transcript=GRMZM5G881775_T02; parent_gene=GRMZM5G881775	GRMZM5G881775_P02	TRUE	TRUE	eLPPYDPDWYyIR	91%	n+304 (+304), iTRAQ8plex (+304)	25.16	25.00
1709	seq=translation; coord=9:123775912..123778018:1; parent_transcript=GRMZM5G881775_T02; parent_gene=GRMZM5G881775	GRMZM5G881775_P02	TRUE	TRUE	mELPEWVDIVk	95%	n+304 (+304), K+304 (+304)	36.21	25.71

1710	seq=translation; coord=9:123775912..123778018:1; parent_transcript=GRMZM5G881775_T02; parent_gene=GRMZM5G881775	GRMZM5G881775_P02	TRUE	TRUE	nILQQLQEMGIIDVDPk	95%	n+304 (+304), K+304 (+304)	52.66	25.00
1711	seq=translation; coord=9:123775912..123778018:1; parent_transcript=GRMZM5G881775_T02; parent_gene=GRMZM5G881775	GRMZM5G881775_P02	TRUE	TRUE	qGIGVGGFQk	95%	n+304 (+304), K+304 (+304)	51.35	25.12
1712	seq=translation; coord=9:123775912..123778018:1; parent_transcript=GRMZM5G881775_T02; parent_gene=GRMZM5G881775	GRMZM5G881775_P02	TRUE	TRUE	qGIGVGGFQk	95%	Pyro-cmC (-17), n+304 (+304), K+304 (+304)	33.88	25.60
1713	seq=translation; coord=3:230606444..230608235:-1; parent_transcript=GRMZM2G010868_T01; parent_gene=GRMZM2G010868	GRMZM2G010868_P01,GRMZM2G010868_P02	TRUE	TRUE	gTGSGPSAScc SGVR	95%	n+304 (+304), Carbamidomethyl (+57), Carbamidomethyl (+57)	31.32	25.00
1714	seq=translation; coord=3:230606444..230608235:-1; parent_transcript=GRMZM2G010868_T01; parent_gene=GRMZM2G010868	GRMZM2G010868_P01,GRMZM2G010868_P02	TRUE	TRUE	gVSGLNAGNAASIPSk	95%	n+304 (+304), K+304 (+304)	78.07	25.81
1715	seq=translation; coord=1:192400083..192403759:1; parent_transcript=GRMZM2G096585_T03; parent_gene=GRMZM2G096585	GRMZM2G096585_P03,GRMZM2G096585_P04, GRMZM2G096585_P06	TRUE	TRUE	aLEIDPDNR	94%	n+304 (+304)	30.93	25.00
1716	seq=translation; coord=1:192400083..192403759:1; parent_transcript=GRMZM2G096585_T03; parent_gene=GRMZM2G096585	GRMZM2G096585_P03,GRMZM2G096585_P04, GRMZM2G096585_P06	TRUE	TRUE	dGYFcPALAk	95%	n+304 (+304), Carbamidomethyl (+57), K+304 (+304)	32.12	25.47
1717	seq=translation; coord=1:192400083..192403759:1; parent_transcript=GRMZM2G096585_T03; parent_gene=GRMZM2G096585	GRMZM2G096585_P03,GRMZM2G096585_P04, GRMZM2G096585_P06	TRUE	TRUE	gWDQGIk	91%	n+304 (+304), K+304 (+304)	28.61	26.39
1718	seq=translation; coord=1:192400083..192403759:1; parent_transcript=GRMZM2G096585_T03; parent_gene=GRMZM2G096585	GRMZM2G096585_P03,GRMZM2G096585_P04, GRMZM2G096585_P06	TRUE	TRUE	lEDGTVVSk	90%	n+304 (+304), K+304 (+304)	26.40	26.18
1719	seq=translation; coord=1:192400083..192403759:1; parent_transcript=GRMZM2G096585_T03; parent_gene=GRMZM2G096585	GRMZM2G096585_P03,GRMZM2G096585_P04, GRMZM2G096585_P06	TRUE	TRUE	lGQGQVIk	91%	n+304 (+304), K+304 (+304)	27.71	25.00
1720	seq=translation; coord=1:192400083..192403759:1; parent_transcript=GRMZM2G096585_T03; parent_gene=GRMZM2G096585	GRMZM2G096585_P03,GRMZM2G096585_P04, GRMZM2G096585_P06	TRUE	TRUE	lQDGAVFTk	95%	n+304 (+304), K+304 (+304)	44.10	26.15
1721	seq=translation; coord=1:192400083..192403759:1; parent_transcript=GRMZM2G096585_T03; parent_gene=GRMZM2G096585	GRMZM2G096585_P03,GRMZM2G096585_P04, GRMZM2G096585_P06	TRUE	TRUE	tDEEEVIAGLDR	95%	n+304 (+304)	54.56	25.00
1722	seq=translation; coord=1:192400083..192403759:1; parent_transcript=GRMZM2G096585_T03; parent_gene=GRMZM2G096585	GRMZM2G096585_P03,GRMZM2G096585_P04, GRMZM2G096585_P06	TRUE	TRUE	vLELDSQNVk	95%	n+304 (+304), K+304 (+304)	40.38	26.15
1723	seq=translation; coord=1:192400083..192403759:1; parent_transcript=GRMZM2G096585_T03; parent_gene=GRMZM2G096585	GRMZM2G096585_P03,GRMZM2G096585_P04, GRMZM2G096585_P06	TRUE	TRUE	vQAYIQLADLELAEADIk	95%	n+304 (+304), K+304 (+304)	77.72	25.35
1724	seq=translation; coord=1:192400083..192403759:1; parent_transcript=GRMZM2G096585_T03; parent_gene=GRMZM2G096585	GRMZM2G096585_P03,GRMZM2G096585_P04, GRMZM2G096585_P06	TRUE	TRUE	yIEYDTSFSEDEk	95%	n+304 (+304), K+304 (+304)	33.05	25.00
1725	seq=translation; coord=4:183449044..183455471:-1; parent_transcript=GRMZM2G111818_T01; parent_gene=GRMZM2G111818	GRMZM2G111818_P01,GRMZM2G111818_P04	TRUE	TRUE	aETFGIPIPDWAk	95%	n+304 (+304), K+304 (+304)	42.39	26.00
1726	seq=translation; coord=4:183449044..183455471:-1; parent_transcript=GRMZM2G111818_T01; parent_gene=GRMZM2G111818	GRMZM2G111818_P01,GRMZM2G111818_P04	TRUE	TRUE	fPIAGSADDAQk	95%	n+304 (+304), K+304 (+304)	38.35	25.95
1727	seq=translation; coord=4:183449044..183455471:-1; parent_transcript=GRMZM2G111818_T01; parent_gene=GRMZM2G111818	GRMZM2G111818_P01,GRMZM2G111818_P04	TRUE	TRUE	lIDLAIGINETLGESk	95%	n+304 (+304), K+304 (+304)	72.24	25.00

1728	seq=translation; coord=4:183449044..183455471:-1; parent_transcript=GRMZM2G111818_T01; parent_gene=GRMZM2G111818	GRMZM2G111818_P01,GRMZM2G111818_P04	TRUE	TRUE	lQELNNAVIISTITGDLTk	95%	n+304 (+304), K+304 (+304)	50.95	25.00
1729	seq=translation; coord=4:183449044..183455471:-1; parent_transcript=GRMZM2G111818_T01; parent_gene=GRMZM2G111818	GRMZM2G111818_P01,GRMZM2G111818_P04	TRUE	TRUE	sTVAATAAMTINPELHVEALQNR	95%	n+304 (+304)	64.33	25.61
1730	seq=translation; coord=4:183449044..183455471:-1; parent_transcript=GRMZM2G111818_T01; parent_gene=GRMZM2G111818	GRMZM2G111818_P01,GRMZM2G111818_P04	TRUE	TRUE	tLPPGFHMNPIQFEk	94%	n+304 (+304), K+304 (+304)	26.21	25.56
1731	seq=translation; coord=4:183449044..183455471:-1; parent_transcript=GRMZM2G111818_T01; parent_gene=GRMZM2G111818	GRMZM2G111818_P01,GRMZM2G111818_P04	TRUE	TRUE	tPTEVNAFLSNPGGYATAAR	95%	n+304 (+304)	41.10	25.00
1732	seq=translation; coord=4:163768734..163771529:1; parent_transcript=GRMZM2G135186_T01; parent_gene=GRMZM2G135186	GRMZM2G135186_P01,GRMZM2G135186_P02, GRMZM2G135186_P03	TRUE	TRUE	dEGFSSLWR	95%	n+304 (+304)	36.94	25.00
1733	seq=translation; coord=4:163768734..163771529:1; parent_transcript=GRMZM2G135186_T01; parent_gene=GRMZM2G135186	GRMZM2G135186_P01,GRMZM2G135186_P02, GRMZM2G135186_P03	TRUE	TRUE	gIADcFk	89%	n+304 (+304), Carbamidomethyl (+57), K+304 (+304)	26.34	25.54
1734	seq=translation; coord=4:163768734..163771529:1; parent_transcript=GRMZM2G135186_T01; parent_gene=GRMZM2G135186	GRMZM2G135186_P01,GRMZM2G135186_P02, GRMZM2G135186_P03	TRUE	TRUE	nFMIDFMMGGVSAAVSk	95%	n+304 (+304), K+304 (+304)	57.14	25.00
1735	seq=translation; coord=4:163768734..163771529:1; parent_transcript=GRMZM2G135186_T01; parent_gene=GRMZM2G135186	GRMZM2G135186_P01,GRMZM2G135186_P02, GRMZM2G135186_P03	TRUE	TRUE	sDGIAGLYR	94%	n+304 (+304)	30.85	25.00
1736	seq=translation; coord=4:163768734..163771529:1; parent_transcript=GRMZM2G135186_T01; parent_gene=GRMZM2G135186	GRMZM2G135186_P01,GRMZM2G135186_P02, GRMZM2G135186_P03	TRUE	TRUE	sSLDAFQQILk	95%	n+304 (+304), K+304 (+304)	36.74	25.00
1737	seq=translation; coord=4:163768734..163771529:1; parent_transcript=GRMZM2G135186_T01; parent_gene=GRMZM2G135186	GRMZM2G135186_P01,GRMZM2G135186_P02, GRMZM2G135186_P03	TRUE	TRUE	tIkDEGFSSLWR	95%	n+304 (+304), K+304 (+304)	32.29	25.82
1738	seq=translation; coord=1:234775005..234780894:-1; parent_transcript=GRMZM2G416120_T01; parent_gene=GRMZM2G416120	GRMZM2G416120_P01	TRUE	TRUE	aIFTEGck	93%	n+304 (+304), Carbamidomethyl (+57), K+304 (+304)	27.17	25.87
1739	seq=translation; coord=1:234775005..234780894:-1; parent_transcript=GRMZM2G416120_T01; parent_gene=GRMZM2G416120	GRMZM2G416120_P01	TRUE	TRUE	eGVITIADGNTLYNELEVVEGMk	95%	n+304 (+304), K+304 (+304)	75.67	25.54
1740	seq=translation; coord=1:234775005..234780894:-1; parent_transcript=GRMZM2G416120_T01; parent_gene=GRMZM2G416120	GRMZM2G416120_P01	TRUE	TRUE	gISMAVDAVVTNLk	95%	n+304 (+304), K+304 (+304)	48.04	25.00
1741	seq=translation; coord=1:234775005..234780894:-1; parent_transcript=GRMZM2G416120_T01; parent_gene=GRMZM2G416120	GRMZM2G416120_P01	TRUE	TRUE	gVEELADAVk	95%	n+304 (+304), K+304 (+304)	46.85	25.66
1742	seq=translation; coord=1:234775005..234780894:-1; parent_transcript=GRMZM2G416120_T01; parent_gene=GRMZM2G416120	GRMZM2G416120_P01	TRUE	TRUE	nVVIEQSFGAPk	95%	n+304 (+304), K+304 (+304)	40.29	25.67
1743	seq=translation; coord=1:234775005..234780894:-1; parent_transcript=GRMZM2G416120_T01; parent_gene=GRMZM2G416120	GRMZM2G416120_P01	TRUE	TRUE	qRPLLIVAEDVESEALGTLIINk	94%	n+304 (+304), K+304 (+304)	25.31	25.00
1744	seq=translation; coord=1:234775005..234780894:-1; parent_transcript=GRMZM2G416120_T01; parent_gene=GRMZM2G416120	GRMZM2G416120_P01	TRUE	TRUE	sVAAGMNAMDLR	95%	n+304 (+304)	40.85	25.00
1745	seq=translation; coord=1:234775005..234780894:-1; parent_transcript=GRMZM2G416120_T01; parent_gene=GRMZM2G416120	GRMZM2G416120_P01	TRUE	TRUE	vLEMALk	95%	n+304 (+304), K+304 (+304)	28.34	25.00

1746	seq=translation; coord=1:84919829..84924586:-1; parent_transcript=GRMZM2G136769_T01; parent_gene=GRMZM2G136769	GRMZM2G136769_P01	TRUE	TRUE	eIFQNPEFLR	89%	n+304 (+304)	25.92	25.00
1747	seq=translation; coord=1:84919829..84924586:-1; parent_transcript=GRMZM2G136769_T01; parent_gene=GRMZM2G136769	GRMZM2G136769_P01	TRUE	TRUE	eVVAGScDVPAPQQR	95%	n+304 (+304), Carbamidomethyl (+57)	51.56	25.00
1748	seq=translation; coord=1:84919829..84924586:-1; parent_transcript=GRMZM2G136769_T01; parent_gene=GRMZM2G136769	GRMZM2G136769_P01	TRUE	TRUE	fTVQTDLGATVGGFk	95%	n+304 (+304), K+304 (+304)	49.48	25.58
1749	seq=translation; coord=1:84919829..84924586:-1; parent_transcript=GRMZM2G136769_T01; parent_gene=GRMZM2G136769	GRMZM2G136769_P01	TRUE	TRUE	nLMESNPQMR	95%	n+304 (+304)	32.42	25.00
1750	seq=translation; coord=1:84919829..84924586:-1; parent_transcript=GRMZM2G136769_T01; parent_gene=GRMZM2G136769	GRMZM2G136769_P01	TRUE	TRUE	nMIMSNPQMR	95%	n+304 (+304)	32.54	25.00
1751	seq=translation; coord=1:84919829..84924586:-1; parent_transcript=GRMZM2G136769_T01; parent_gene=GRMZM2G136769	GRMZM2G136769_P01	TRUE	TRUE	qLTSPETLQQLLLFQQSLLGQLGQHQPNQGR	95%	n+304 (+304)	54.29	25.00
1752	seq=translation; coord=1:293163457..293166106:1; parent_transcript=GRMZM2G060702_T02; parent_gene=GRMZM2G060702	GRMZM2G060702_P02	TRUE	TRUE	aTSYDDFTNSLPENDcR	95%	n+304 (+304), Carbamidomethyl (+57)	55.51	25.00
1753	seq=translation; coord=4:198559982..198560791:-1; parent_transcript=AC234156.1_FGT005; parent_gene=AC234156.1_FG005	AC234156.1_FGP005	TRUE	TRUE	dSAFAALk	91%	n+304 (+304), K+304 (+304)	27.48	26.41
1754	seq=translation; coord=4:198559982..198560791:-1; parent_transcript=AC234156.1_FGT005; parent_gene=AC234156.1_FG005	AC234156.1_FGP005	TRUE	TRUE	gGITVFVPk	95%	n+304 (+304), K+304 (+304)	36.50	25.20
1755	seq=translation; coord=4:198559982..198560791:-1; parent_transcript=AC234156.1_FGT005; parent_gene=AC234156.1_FG005	AC234156.1_FGP005	TRUE	TRUE	rGGITVFVPk	95%	n+304 (+304), K+304 (+304)	28.30	25.00
1756	seq=translation; coord=4:198559982..198560791:-1; parent_transcript=AC234156.1_FGT005; parent_gene=AC234156.1_FG005	AC234156.1_FGP005	TRUE	TRUE	sLLLcHALPk	95%	n+304 (+304), Carbamidomethyl (+57), K+304 (+304)	32.97	25.00
1757	seq=translation; coord=4:198559982..198560791:-1; parent_transcript=AC234156.1_FGT005; parent_gene=AC234156.1_FG005	AC234156.1_FGP005	TRUE	TRUE	vLLPMQIFk	95%	n+304 (+304), K+304 (+304)	32.85	25.00
1758	seq=translation; coord=4:198559982..198560791:-1; parent_transcript=AC234156.1_FGT005; parent_gene=AC234156.1_FG005	AC234156.1_FGP005	TRUE	TRUE	vLLPmQIFk	95%	n+304 (+304), Oxidation (+16), K+304 (+304)	46.82	25.00
1759	seq=translation; coord=4:198559982..198560791:-1; parent_transcript=AC234156.1_FGT005; parent_gene=AC234156.1_FG005	AC234156.1_FGP005	TRUE	TRUE	yYSLAEFDR	95%	n+304 (+304)	37.48	25.00
1760	seq=translation; coord=7:174283500..174287760:-1; parent_transcript=GRMZM2G430600_T01; parent_gene=GRMZM2G430600	GRMZM2G430600_P01,GRMZM2G430600_P02	TRUE	TRUE	eADVDGDGQINYEEFVk	95%	n+304 (+304), K+304 (+304)	58.01	25.00
1761	seq=translation; coord=7:174283500..174287760:-1; parent_transcript=GRMZM2G430600_T01; parent_gene=GRMZM2G430600	GRMZM2G430600_P01,GRMZM2G430600_P02	TRUE	TRUE	eADVDGDGQINyEEFVk	95%	n+304 (+304), iTRAQ8plex (+304), K+304 (+304)	28.36	25.00
1762	seq=translation; coord=7:174283500..174287760:-1; parent_transcript=GRMZM2G430600_T01; parent_gene=GRMZM2G430600	GRMZM2G430600_P01,GRMZM2G430600_P02	TRUE	TRUE	vLcLHGFR	94%	n+304 (+304), Carbamidomethyl (+57)	28.93	25.76
1763	seq=translation; coord=8:163307256..163309969:-1; parent_transcript=GRMZM2G066024_T01; parent_gene=GRMZM2G066024	GRMZM2G066024_P01	TRUE	TRUE	kPWALSFSFGR	94%	K+304 (+304), n+304 (+304)	26.20	25.38

1764	seq=translation; coord=8:163307256..163309969:-1; parent_transcript=GRMZM2G066024_T01; parent_gene=GRMZM2G066024	GRMZM2G066024_P01	TRUE	TRUE	kVTPEVIAQYTVR	95%	K+304 (+304), n+304 (+304)	40.71	25.00
1765	seq=translation; coord=8:163307256..163309969:-1; parent_transcript=GRMZM2G066024_T01; parent_gene=GRMZM2G066024	GRMZM2G066024_P01	TRUE	TRUE	tVPAAVPAVVFLSGGQSEEEATLNLNAMNk	95%	n+304 (+304), K+304 (+304)	69.63	25.00
1766	seq=translation; coord=8:163307256..163309969:-1; parent_transcript=GRMZM2G066024_T01; parent_gene=GRMZM2G066024	GRMZM2G066024_P01	TRUE	TRUE	vTPEVIAQYTVR	95%	n+304 (+304)	42.42	25.01
1767	seq=translation; coord=8:163307256..163309969:-1; parent_transcript=GRMZM2G066024_T01; parent_gene=GRMZM2G066024	GRMZM2G066024_P01	TRUE	TRUE	vTPEVIAQyTVR	94%	n+304 (+304), iTRAQ8plex (+304)	29.01	25.00
1768	seq=translation; coord=8:163307256..163309969:-1; parent_transcript=GRMZM2G066024_T01; parent_gene=GRMZM2G066024	GRMZM2G066024_P01	TRUE	TRUE	yADELIk	95%	n+304 (+304), K+304 (+304)	34.79	26.69
1769	seq=translation; coord=9:18329697..18331617:-1; parent_transcript=AC231745.1_FGT003; parent_gene=AC231745.1_FG003	AC231745.1_FGP003	TRUE	TRUE	gANMAITGATAMDAPFFR	95%	n+304 (+304)	69.88	25.00
1770	seq=translation; coord=9:18329697..18331617:-1; parent_transcript=AC231745.1_FGT003; parent_gene=AC231745.1_FG003	AC231745.1_FGP003	TRUE	TRUE	gANmAITGATAMDAPFFR	86%	n+304 (+304), Oxidation (+16)	25.36	25.00
1771	seq=translation; coord=9:18329697..18331617:-1; parent_transcript=AC231745.1_FGT003; parent_gene=AC231745.1_FG003	AC231745.1_FGP003	TRUE	TRUE	kGANMAITGATAMDAPFFR	95%	K+304 (+304), n+304 (+304)	60.34	25.20
1772	seq=translation; coord=9:18329697..18331617:-1; parent_transcript=AC231745.1_FGT003; parent_gene=AC231745.1_FG003	AC231745.1_FGP003	TRUE	TRUE	nPGSYGFSSVFQAccGSGGGk	95%	n+304 (+304), Carbamidomethyl (+57), Carbamidomethyl (+57), K+304 (+304)	47.66	25.00
1773	seq=translation; coord=9:18329697..18331617:-1; parent_transcript=AC231745.1_FGT003; parent_gene=AC231745.1_FG003	AC231745.1_FGP003	TRUE	TRUE	vIPDFLcSR	88%	n+304 (+304), Carbamidomethyl (+57)	26.01	25.00
1774	seq=translation; coord=9:106398227..106409066:1; parent_transcript=GRMZM2G035417_T01; parent_gene=GRMZM2G035417	GRMZM2G035417_P01	TRUE	TRUE	aDQQMLIYQGk	95%	n+304 (+304), K+304 (+304)	43.69	26.26
1775	seq=translation; coord=9:106398227..106409066:1; parent_transcript=GRMZM2G035417_T01; parent_gene=GRMZM2G035417	GRMZM2G035417_P01	TRUE	TRUE	aIDYLYSGIPENVEAQPVAR	95%	n+304 (+304)	38.70	25.43
1776	seq=translation; coord=9:106398227..106409066:1; parent_transcript=GRMZM2G035417_T01; parent_gene=GRMZM2G035417	GRMZM2G035417_P01	TRUE	TRUE	aPATLAQPAAPVAPAASVAR	95%	n+304 (+304)	93.15	25.00
1777	seq=translation; coord=9:106398227..106409066:1; parent_transcript=GRMZM2G035417_T01; parent_gene=GRMZM2G035417	GRMZM2G035417_P01	TRUE	TRUE	aSSSGASTATTAk	94%	n+304 (+304), K+304 (+304)	27.67	25.65
1778	seq=translation; coord=9:106398227..106409066:1; parent_transcript=GRMZM2G035417_T01; parent_gene=GRMZM2G035417	GRMZM2G035417_P01	TRUE	TRUE	eLVLEVFFAcNk	95%	n+304 (+304), Carbamidomethyl (+57), K+304 (+304)	30.38	25.74
1779	seq=translation; coord=9:106398227..106409066:1; parent_transcript=GRMZM2G035417_T01; parent_gene=GRMZM2G035417	GRMZM2G035417_P01	TRUE	TRUE	gTNFEIEASPDASVADVk	95%	n+304 (+304), K+304 (+304)	46.09	25.00
1780	seq=translation; coord=9:106398227..106409066:1; parent_transcript=GRMZM2G035417_T01; parent_gene=GRMZM2G035417	GRMZM2G035417_P01	TRUE	TRUE	iIETTQGQSTYR	95%	n+304 (+304)	34.17	25.00

1781	seq=translation; coord=9:106398227..106409066:1; parent_transcript=GRMZM2G035417_T01; parent_gene=GRMZM2G035417	GRMZM2G035417_P01	TRUE	TRUE	lIQENQAEFLR	95%	n+304 (+304)	41.84	25.16
1782	seq=translation; coord=9:148111509..148115129:1; parent_transcript=GRMZM2G145258_T01; parent_gene=GRMZM2G145258	GRMZM2G145258_P01	TRUE	TRUE	lERPVEGDEAGQEVAAAE	95%	n+304 (+304)	36.26	25.00
1783	seq=translation; coord=6:124803945..124805261:1; parent_transcript=GRMZM2G084812_T01; parent_gene=GRMZM2G084812	GRMZM2G084812_P01	TRUE	TRUE	dDDPLAVYAcSk	95%	n+304 (+304), Carbamidomethyl (+57), K+304 (+304)	41.39	25.00
1784	seq=translation; coord=6:124803945..124805261:1; parent_transcript=GRMZM2G084812_T01; parent_gene=GRMZM2G084812	GRMZM2G084812_P01	TRUE	TRUE	dDTVTLDTTVVTSTIQATVR	95%	n+304 (+304)	48.54	25.22
1785	seq=translation; coord=6:124803945..124805261:1; parent_transcript=GRMZM2G084812_T01; parent_gene=GRMZM2G084812	GRMZM2G084812_P01	TRUE	TRUE	dTALTVFcPVDAAVAAFmPk	95%	n+304 (+304), Carbamidomethyl (+57), Oxidation (+16), K+304 (+304)	32.65	25.00
1786	seq=translation; coord=6:124803945..124805261:1; parent_transcript=GRMZM2G084812_T01; parent_gene=GRMZM2G084812	GRMZM2G084812_P01	TRUE	TRUE	sFAGLLAADPk	95%	n+304 (+304), K+304 (+304)	50.91	26.14
1787	seq=translation; coord=6:124803945..124805261:1; parent_transcript=GRMZM2G084812_T01; parent_gene=GRMZM2G084812	GRMZM2G084812_P01	TRUE	TRUE	tAILLYHAVPDYYSMQFLk	95%	n+304 (+304), K+304 (+304)	49.31	25.77
1788	seq=translation; coord=6:124803945..124805261:1; parent_transcript=GRMZM2G084812_T01; parent_gene=GRMZM2G084812	GRMZM2G084812_P01	TRUE	TRUE	vTTLATTSVAk	93%	n+304 (+304), K+304 (+304)	27.08	25.00
1789	seq=translation; coord=6:164551086..164552313:1; parent_transcript=GRMZM2G021794_T01; parent_gene=GRMZM2G021794	GRMZM2G021794_P01	TRUE	TRUE	aLkDEQFSEFR	94%	n+304 (+304), K+304 (+304)	31.12	25.37
1790	seq=translation; coord=6:164551086..164552313:1; parent_transcript=GRMZM2G021794_T01; parent_gene=GRMZM2G021794	GRMZM2G021794_P01	TRUE	TRUE	dEQFSEFR	95%	n+304 (+304)	32.83	25.00
1791	seq=translation; coord=6:164551086..164552313:1; parent_transcript=GRMZM2G021794_T01; parent_gene=GRMZM2G021794	GRMZM2G021794_P01	TRUE	TRUE	fYSLSMLGTLSGk	95%	n+304 (+304), K+304 (+304)	28.41	25.93
1792	seq=translation; coord=6:164551086..164552313:1; parent_transcript=GRMZM2G021794_T01; parent_gene=GRMZM2G021794	GRMZM2G021794_P01	TRUE	TRUE	fYSLSmLGTLSGk	95%	n+304 (+304), Oxidation (+16), K+304 (+304)	35.98	25.81
1793	seq=translation; coord=1:278183921..278192926:-1; parent_transcript=GRMZM2G074158_T01; parent_gene=GRMZM2G074158	GRMZM2G074158_P01,GRMZM2G074158_P02	TRUE	TRUE	aDYFLVGk	95%	n+304 (+304), K+304 (+304)	42.58	25.35
1794	seq=translation; coord=1:278183921..278192926:-1; parent_transcript=GRMZM2G074158_T01; parent_gene=GRMZM2G074158	GRMZM2G074158_P01,GRMZM2G074158_P02	TRUE	TRUE	aGESLNWEDFPSk	95%	n+304 (+304), K+304 (+304)	37.80	25.00
1795	seq=translation; coord=1:278183921..278192926:-1; parent_transcript=GRMZM2G074158_T01; parent_gene=GRMZM2G074158	GRMZM2G074158_P01,GRMZM2G074158_P02	TRUE	TRUE	aLTNAIGNLEITGEYAEALk	94%	n+304 (+304), K+304 (+304)	26.06	25.34
1796	seq=translation; coord=1:278183921..278192926:-1; parent_transcript=GRMZM2G074158_T01; parent_gene=GRMZM2G074158	GRMZM2G074158_P01,GRMZM2G074158_P02	TRUE	TRUE	aVAHDVPIPGYk	95%	n+304 (+304), K+304 (+304)	29.54	25.77
1797	seq=translation; coord=1:278183921..278192926:-1; parent_transcript=GRMZM2G074158_T01; parent_gene=GRMZM2G074158	GRMZM2G074158_P01,GRMZM2G074158_P02	TRUE	TRUE	dFPSYIEcQEk	93%	n+304 (+304), Carbamidomethyl (+57), K+304 (+304)	26.36	25.00

1798	seq=translation; coord=1:278183921..278192926:-1; parent_transcript=GRMZM2G074158_T01; parent_gene=GRMZM2G074158	GRMZM2G074158_P01,GRMZM2G074158_P02	TRUE	TRUE	dGQEEIAENWLEMGyPWEVVR	95%	n+304 (+304), iTRAQ8plex (+304)	40.03	25.00
1799	seq=translation; coord=1:278183921..278192926:-1; parent_transcript=GRMZM2G074158_T01; parent_gene=GRMZM2G074158	GRMZM2G074158_P01,GRMZM2G074158_P02	TRUE	TRUE	dIWDISPAILP	95%	n+304 (+304)	60.53	26.34
1800	seq=translation; coord=1:278183921..278192926:-1; parent_transcript=GRMZM2G074158_T01; parent_gene=GRMZM2G074158	GRMZM2G074158_P01,GRMZM2G074158_P02	TRUE	TRUE	dVQGPVSPAEGLPSVLNSIGSSAIASNIk	95%	n+304 (+304), K+304 (+304)	44.59	25.00
1801	seq=translation; coord=1:278183921..278192926:-1; parent_transcript=GRMZM2G074158_T01; parent_gene=GRMZM2G074158	GRMZM2G074158_P01,GRMZM2G074158_P02	TRUE	TRUE	eEVGEENFFLFGAEAHEIAGLR	95%	n+304 (+304)	83.67	25.00
1802	seq=translation; coord=1:278183921..278192926:-1; parent_transcript=GRMZM2G074158_T01; parent_gene=GRMZM2G074158	GRMZM2G074158_P01,GRMZM2G074158_P02	TRUE	TRUE	fADNEDLHSEWR	95%	n+304 (+304)	41.43	25.00
1803	seq=translation; coord=1:278183921..278192926:-1; parent_transcript=GRMZM2G074158_T01; parent_gene=GRMZM2G074158	GRMZM2G074158_P01,GRMZM2G074158_P02	TRUE	TRUE	fcNPALSALISk	95%	n+304 (+304), Carbamidomethyl (+57), K+304 (+304)	35.21	25.63
1804	seq=translation; coord=1:278183921..278192926:-1; parent_transcript=GRMZM2G074158_T01; parent_gene=GRMZM2G074158	GRMZM2G074158_P01,GRMZM2G074158_P02	TRUE	TRUE	gLSWSEAWSITER	95%	n+304 (+304)	64.17	25.00
1805	seq=translation; coord=1:278183921..278192926:-1; parent_transcript=GRMZM2G074158_T01; parent_gene=GRMZM2G074158	GRMZM2G074158_P01,GRMZM2G074158_P02	TRUE	TRUE	iLMDVk	90%	n+304 (+304), K+304 (+304)	27.47	25.85
1806	seq=translation; coord=1:278183921..278192926:-1; parent_transcript=GRMZM2G074158_T01; parent_gene=GRMZM2G074158	GRMZM2G074158_P01,GRMZM2G074158_P02	TRUE	TRUE	mSILNTAGSSk	95%	n+304 (+304), K+304 (+304)	44.73	26.44
1807	seq=translation; coord=1:278183921..278192926:-1; parent_transcript=GRMZM2G074158_T01; parent_gene=GRMZM2G074158	GRMZM2G074158_P01,GRMZM2G074158_P02	TRUE	TRUE	qLLNILGIVYR	95%	n+304 (+304)	33.11	25.00
1808	seq=translation; coord=1:278183921..278192926:-1; parent_transcript=GRMZM2G074158_T01; parent_gene=GRMZM2G074158	GRMZM2G074158_P01,GRMZM2G074158_P02	TRUE	TRUE	sGVFGTySYDELMGSLEGNEGYGR	95%	n+304 (+304), iTRAQ8plex (+304)	29.82	25.00
1809	seq=translation; coord=1:278183921..278192926:-1; parent_transcript=GRMZM2G074158_T01; parent_gene=GRMZM2G074158	GRMZM2G074158_P01,GRMZM2G074158_P02	TRUE	TRUE	sGVFGTYSYDELMGSLEGNEGyGR	94%	n+304 (+304), iTRAQ8plex (+304)	25.31	25.00
1810	seq=translation; coord=1:278183921..278192926:-1; parent_transcript=GRMZM2G074158_T01; parent_gene=GRMZM2G074158	GRMZM2G074158_P01,GRMZM2G074158_P02	TRUE	TRUE	tGYIVSPDAMFDVQVk	95%	n+304 (+304), K+304 (+304)	43.88	25.60
1811	seq=translation; coord=1:278183921..278192926:-1; parent_transcript=GRMZM2G074158_T01; parent_gene=GRMZM2G074158	GRMZM2G074158_P01,GRMZM2G074158_P02	TRUE	TRUE	wIGSDDWVLNTDk	95%	n+304 (+304), K+304 (+304)	33.69	25.00
1812	seq=translation; coord=1:278183921..278192926:-1; parent_transcript=GRMZM2G074158_T01; parent_gene=GRMZM2G074158	GRMZM2G074158_P01,GRMZM2G074158_P02	TRUE	TRUE	wSLDIMQk	90%	n+304 (+304), K+304 (+304)	27.63	26.04
1813	seq=translation; coord=4:215212969..215240759:1; parent_transcript=GRMZM2G094123_T01; parent_gene=GRMZM2G094123	GRMZM2G094123_P01	TRUE	TRUE	eEADEEVLQILELYR	95%	n+304 (+304)	78.34	25.00
1814	seq=translation; coord=4:215212969..215240759:1; parent_transcript=GRMZM2G094123_T01; parent_gene=GRMZM2G094123	GRMZM2G094123_P01	TRUE	TRUE	eFLWQEGHTAFATk	95%	n+304 (+304), K+304 (+304)	42.29	25.00
1815	seq=translation; coord=4:215212969..215240759:1; parent_transcript=GRMZM2G094123_T01; parent_gene=GRMZM2G094123	GRMZM2G094123_P01	TRUE	TRUE	eMVWQNSWAYTTR	95%	n+304 (+304)	47.84	25.00

1816	seq=translation; coord=4:215212969..215240759:1; parent_transcript=GRMZM2G094123_T01; parent_gene=GRMZM2G094123	GRMZM2G094123_P01	TRUE	TRUE	fAGGLYTTSVEAFIPNTGR	95%	n+304 (+304)	90.62	25.00
1817	seq=translation; coord=4:215212969..215240759:1; parent_transcript=GRMZM2G094123_T01; parent_gene=GRMZM2G094123	GRMZM2G094123_P01	TRUE	TRUE	gAcESTVYTLDQSGIR	92%	n+304 (+304), Carbamidomethyl (+57)	26.94	25.00
1818	seq=translation; coord=4:215212969..215240759:1; parent_transcript=GRMZM2G094123_T01; parent_gene=GRMZM2G094123	GRMZM2G094123_P01	TRUE	TRUE	iYEEFLAVPVSk	95%	n+304 (+304), K+304 (+304)	32.85	25.00
1819	seq=translation; coord=4:215212969..215240759:1; parent_transcript=GRMZM2G094123_T01; parent_gene=GRMZM2G094123	GRMZM2G094123_P01	TRUE	TRUE	lILAPWcDEEEIEk	95%	n+304 (+304), Carbamidomethyl (+57), K+304 (+304)	40.67	26.25
1820	seq=translation; coord=4:215212969..215240759:1; parent_transcript=GRMZM2G094123_T01; parent_gene=GRMZM2G094123	GRMZM2G094123_P01	TRUE	TRUE	sIGVMVMTHGDDk	95%	n+304 (+304), K+304 (+304)	45.17	25.00
1821	seq=translation; coord=4:215212969..215240759:1; parent_transcript=GRMZM2G094123_T01; parent_gene=GRMZM2G094123	GRMZM2G094123_P01	TRUE	TRUE	vDIPVTNLVEEVk	95%	n+304 (+304), K+304 (+304)	45.49	25.00
1822	seq=translation; coord=4:215212969..215240759:1; parent_transcript=GRMZM2G094123_T01; parent_gene=GRMZM2G094123	GRMZM2G094123_P01	TRUE	TRUE	vLLDEIQk	95%	n+304 (+304), K+304 (+304)	34.70	25.00
1823	seq=translation; coord=4:237610321..237618962:-1; parent_transcript=GRMZM2G155384_T02; parent_gene=GRMZM2G155384	GRMZM2G155384_P02	TRUE	TRUE	aVPLALGILcISNPk	95%	n+304 (+304), Carbamidomethyl (+57), K+304 (+304)	62.16	25.00
1824	seq=translation; coord=4:237610321..237618962:-1; parent_transcript=GRMZM2G155384_T02; parent_gene=GRMZM2G155384	GRMZM2G155384_P02	TRUE	TRUE	lLPVALGLLYLGk	95%	n+304 (+304), K+304 (+304)	33.87	25.00
1825	seq=translation; coord=4:237610321..237618962:-1; parent_transcript=GRMZM2G155384_T02; parent_gene=GRMZM2G155384	GRMZM2G155384_P02	TRUE	TRUE	mLLTVDEDLkPLSVPVR	94%	n+304 (+304), K+304 (+304)	25.46	25.00
1826	seq=translation; coord=4:237610321..237618962:-1; parent_transcript=GRMZM2G155384_T02; parent_gene=GRMZM2G155384	GRMZM2G155384_P02	TRUE	TRUE	nLAGEIAQEFQk	95%	n+304 (+304), K+304 (+304)	35.94	25.63
1827	seq=translation; coord=4:237610321..237618962:-1; parent_transcript=GRMZM2G155384_T02; parent_gene=GRMZM2G155384	GRMZM2G155384_P02	TRUE	TRUE	sEAELAEPIIR	95%	n+304 (+304)	40.92	26.50
1828	seq=translation; coord=4:237610321..237618962:-1; parent_transcript=GRMZM2G155384_T02; parent_gene=GRMZM2G155384	GRMZM2G155384_P02	TRUE	TRUE	vGQAVDVVGQAGRPk	95%	n+304 (+304), K+304 (+304)	28.19	25.00
1829	seq=translation; coord=4:237610321..237618962:-1; parent_transcript=GRMZM2G155384_T02; parent_gene=GRMZM2G155384	GRMZM2G155384_P02	TRUE	TRUE	vNVMDTLSR	88%	n+304 (+304)	26.24	25.00
1830	seq=translation; coord=4:237610321..237618962:-1; parent_transcript=GRMZM2G155384_T02; parent_gene=GRMZM2G155384	GRMZM2G155384_P02	TRUE	TRUE	ycDVTLMSLAYAGTGNVLk	95%	n+304 (+304), Carbamidomethyl (+57), K+304 (+304)	36.78	25.00
1831	seq=translation; coord=4:237610321..237618962:-1; parent_transcript=GRMZM2G155384_T02; parent_gene=GRMZM2G155384	GRMZM2G155384_P02	TRUE	TRUE	yIPLTPVLEGFVILk	95%	n+304 (+304), K+304 (+304)	50.30	25.00
1832	seq=translation; coord=5:215817786..215821620:-1; parent_transcript=GRMZM5G848768_T02; parent_gene=GRMZM5G848768	GRMZM5G848768_P02	TRUE	TRUE	dGTTTAGNSSQVSDGAGAVLLMk	95%	n+304 (+304), K+304 (+304)	32.16	25.20
1833	seq=translation; coord=5:215817786..215821620:-1; parent_transcript=GRMZM5G848768_T02; parent_gene=GRMZM5G848768	GRMZM5G848768_P02	TRUE	TRUE	dTYPEDLLTVVLk	95%	n+304 (+304), K+304 (+304)	31.07	25.69

1834	seq=translation; coord=5:215817786..215821620:-1; parent_transcript=GRMZM5G848768_T02; parent_gene=GRMZM5G848768	GRMZM5G848768_P02	TRUE	TRUE	gDAVDGLSNVR	88%	n+304 (+304)	26.09	25.00
1835	seq=translation; coord=5:215817786..215821620:-1; parent_transcript=GRMZM5G848768_T02; parent_gene=GRMZM5G848768	GRMZM5G848768_P02	TRUE	TRUE	iNPADIGDIVVGTVLGPGSQR	95%	n+304 (+304)	60.94	25.65
1836	seq=translation; coord=5:215817786..215821620:-1; parent_transcript=GRMZM5G848768_T02; parent_gene=GRMZM5G848768	GRMZM5G848768_P02	TRUE	TRUE	mAALFAGFPETVPVR	95%	n+304 (+304)	76.69	25.00
1837	seq=translation; coord=5:215817786..215821620:-1; parent_transcript=GRMZM5G848768_T02; parent_gene=GRMZM5G848768	GRMZM5G848768_P02	TRUE	TRUE	sFAAVGVDPAVMGVGPAVAIPAAVk	95%	n+304 (+304), K+304 (+304)	38.81	25.00
1838	seq=translation; coord=5:215817786..215821620:-1; parent_transcript=GRMZM5G848768_T02; parent_gene=GRMZM5G848768	GRMZM5G848768_P02	TRUE	TRUE	sSSFGDDVVVVAAYR	95%	n+304 (+304)	56.86	25.00
1839	seq=translation; coord=5:215817786..215821620:-1; parent_transcript=GRMZM5G848768_T02; parent_gene=GRMZM5G848768	GRMZM5G848768_P02	TRUE	TRUE	sSSFGDDVVVVAAyR	95%	n+304 (+304), iTRAQ8plex (+304)	38.16	25.39
1840	seq=translation; coord=9:150090384..150093179:-1; parent_transcript=GRMZM2G099657_T01; parent_gene=GRMZM2G099657	GRMZM2G099657_P01,GRMZM2G126821_P01, GRMZM2G145308_P01	TRUE	TRUE	nSIGcLFWLLAR	95%	n+304 (+304), Carbamidomethyl (+57)	47.43	25.00
1841	seq=translation; coord=3:113472963..113480259:-1; parent_transcript=GRMZM2G093347_T01; parent_gene=GRMZM2G093347	GRMZM2G093347_P01,GRMZM2G131907_P01	TRUE	TRUE	aLVVDDLIATGGTLcAAVk	95%	n+304 (+304), Carbamidomethyl (+57), K+304 (+304)	34.80	25.00
1842	seq=translation; coord=3:113472963..113480259:-1; parent_transcript=GRMZM2G093347_T01; parent_gene=GRMZM2G093347	GRMZM2G093347_P01,GRMZM2G131907_P01	TRUE	TRUE	dTIDLFVER	95%	n+304 (+304)	32.63	25.00
1843	seq=translation; coord=3:113472963..113480259:-1; parent_transcript=GRMZM2G093347_T01; parent_gene=GRMZM2G093347	GRMZM2G093347_P01,GRMZM2G131907_P01	TRUE	TRUE	gFIFGPPIALAIGAk	95%	n+304 (+304), K+304 (+304)	32.96	25.00
1844	seq=translation; coord=3:113472963..113480259:-1; parent_transcript=GRMZM2G093347_T01; parent_gene=GRMZM2G093347	GRMZM2G093347_P01,GRMZM2G131907_P01	TRUE	TRUE	kLPGEVISEEYSLEYGTDk	95%	K+304 (+304), n+304 (+304), K+304 (+304)	45.54	25.12
1845	seq=translation; coord=3:113472963..113480259:-1; parent_transcript=GRMZM2G093347_T01; parent_gene=GRMZM2G093347	GRMZM2G093347_P01,GRMZM2G131907_P01	TRUE	TRUE	lAGIASSIR	95%	n+304 (+304)	40.53	25.00
1846	seq=translation; coord=3:113472963..113480259:-1; parent_transcript=GRMZM2G093347_T01; parent_gene=GRMZM2G093347	GRMZM2G093347_P01,GRMZM2G131907_P01	TRUE	TRUE	lGDRPVFVLVEADA	95%	n+304 (+304)	34.08	25.80
1847	seq=translation; coord=2:231986102..231987473:-1; parent_transcript=GRMZM2G053206_T01; parent_gene=GRMZM2G053206	GRMZM2G053206_P01	TRUE	TRUE	aFDAAAAGPNFPWMSR	95%	n+304 (+304)	75.60	25.00
1848	seq=translation; coord=2:231986102..231987473:-1; parent_transcript=GRMZM2G053206_T01; parent_gene=GRMZM2G053206	GRMZM2G053206_P01	TRUE	TRUE	aFDAAAAGPNFPWmSR	95%	n+304 (+304), Oxidation (+16)	55.92	25.00
1849	seq=translation; coord=2:231986102..231987473:-1; parent_transcript=GRMZM2G053206_T01; parent_gene=GRMZM2G053206	GRMZM2G053206_P01	TRUE	TRUE	dASTSLYTAPLk	95%	n+304 (+304), K+304 (+304)	38.51	25.81
1850	seq=translation; coord=2:231986102..231987473:-1; parent_transcript=GRMZM2G053206_T01; parent_gene=GRMZM2G053206	GRMZM2G053206_P01	TRUE	TRUE	dGHPLVLDLTSPVISLATcASSSk	95%	n+304 (+304), Carbamidomethyl (+57), K+304 (+304)	33.43	25.05
1851	seq=translation; coord=2:231986102..231987473:-1; parent_transcript=GRMZM2G053206_T01; parent_gene=GRMZM2G053206	GRMZM2G053206_P01	TRUE	TRUE	gDFTTmLAGTAPLHAGAGAGAPGYYVSSTGIAVEQAR	95%	n+304 (+304), Oxidation (+16)	59.35	25.00

1852	seq=translation; coord=2:231986102..231987473:-1; parent_transcript=GRMZM2G053206_T01; parent_gene=GRMZM2G053206	GRMZM2G053206_P01	TRUE	TRUE	sTSGDSVGVAIFGGGPLFFVPPDR	95%	n+304 (+304)	38.57	25.00
1853	seq=translation; coord=2:231986102..231987473:-1; parent_transcript=GRMZM2G053206_T01; parent_gene=GRMZM2G053206	GRMZM2G053206_P01	TRUE	TRUE	vAAVAPFDR	93%	n+304 (+304)	30.39	25.87
1854	seq=translation; coord=2:231986102..231987473:-1; parent_transcript=GRMZM2G053206_T01; parent_gene=GRMZM2G053206	GRMZM2G053206_P01	TRUE	TRUE	vALcLPSDGR	95%	n+304 (+304), Carbamidomethyl (+57)	34.32	25.13
1855	seq=translation; coord=3:201684716..201689628:-1; parent_transcript=GRMZM2G159724_T01; parent_gene=GRMZM2G159724	GRMZM2G159724_P01,GRMZM2G159724_P03	TRUE	TRUE	aTGQEYTDFLQEFMTAVk	95%	n+304 (+304), K+304 (+304)	70.41	25.00
1856	seq=translation; coord=3:201684716..201689628:-1; parent_transcript=GRMZM2G159724_T01; parent_gene=GRMZM2G159724	GRMZM2G159724_P01,GRMZM2G159724_P03	TRUE	TRUE	gLLPPAIVSQELQER	95%	n+304 (+304)	47.24	25.91
1857	seq=translation; coord=3:201684716..201689628:-1; parent_transcript=GRMZM2G159724_T01; parent_gene=GRMZM2G159724	GRMZM2G159724_P01,GRMZM2G159724_P03	TRUE	TRUE	iWLVDSk	93%	n+304 (+304), K+304 (+304)	28.73	26.53
1858	seq=translation; coord=3:201684716..201689628:-1; parent_transcript=GRMZM2G159724_T01; parent_gene=GRMZM2G159724	GRMZM2G159724_P01,GRMZM2G159724_P03	TRUE	TRUE	lLIDNVEELLPIVYTPTVGEAcQk	95%	n+304 (+304), Carbamidomethyl (+57), K+304 (+304)	58.78	25.00
1859	seq=translation; coord=3:201684716..201689628:-1; parent_transcript=GRMZM2G159724_T01; parent_gene=GRMZM2G159724	GRMZM2G159724_P01,GRMZM2G159724_P03	TRUE	TRUE	vHDDMLLAASEALAQQVTEENFEk	95%	n+304 (+304), K+304 (+304)	75.54	25.00
1860	seq=translation; coord=3:201684716..201689628:-1; parent_transcript=GRMZM2G159724_T01; parent_gene=GRMZM2G159724	GRMZM2G159724_P01,GRMZM2G159724_P03	TRUE	TRUE	vLIQFEDFANHNAFDLLAR	95%	n+304 (+304)	43.01	25.26
1861	seq=translation; coord=3:201684716..201689628:-1; parent_transcript=GRMZM2G159724_T01; parent_gene=GRMZM2G159724	GRMZM2G159724_P01,GRMZM2G159724_P03	TRUE	TRUE	yAEScMYSPIYR	95%	n+304 (+304), Carbamidomethyl (+57)	52.58	25.00
1862	seq=translation; coord=3:201684716..201689628:-1; parent_transcript=GRMZM2G159724_T01; parent_gene=GRMZM2G159724	GRMZM2G159724_P01,GRMZM2G159724_P03	TRUE	TRUE	yMALMDLQEGNER	95%	n+304 (+304)	60.31	25.00
1863	seq=translation; coord=5:153543150..153551579:1; parent_transcript=GRMZM2G177781_T01; parent_gene=GRMZM2G177781	GRMZM2G177781_P01,GRMZM2G177781_P02	TRUE	TRUE	eVIAEADAGSSGAVSGGR	95%	n+304 (+304)	86.51	25.00
1864	seq=translation; coord=5:153543150..153551579:1; parent_transcript=GRMZM2G177781_T01; parent_gene=GRMZM2G177781	GRMZM2G177781_P01,GRMZM2G177781_P02	TRUE	TRUE	qLVAEVTSENR	94%	n+304 (+304)	30.05	25.59
1865	seq=translation; coord=5:153543150..153551579:1; parent_transcript=GRMZM2G177781_T01; parent_gene=GRMZM2G177781	GRMZM2G177781_P01,GRMZM2G177781_P02	TRUE	TRUE	qVELSGNPEQISk	95%	n+304 (+304), K+304 (+304)	36.24	25.83
1866	seq=translation; coord=5:153543150..153551579:1; parent_transcript=GRMZM2G177781_T01; parent_gene=GRMZM2G177781	GRMZM2G177781_P01,GRMZM2G177781_P02	TRUE	TRUE	qVELSGNPEQISk	95%	Pyro-cmC (-17), n+304 (+304), K+304 (+304)	31.86	25.00
1867	seq=translation; coord=5:215289055..215297830:1; parent_transcript=GRMZM5G806449_T01; parent_gene=GRMZM5G806449	GRMZM5G806449_P01,GRMZM5G806449_P03	TRUE	TRUE	aIVASNLEGR	95%	n+304 (+304)	36.04	25.44
1868	seq=translation; coord=5:215289055..215297830:1; parent_transcript=GRMZM5G806449_T01; parent_gene=GRMZM5G806449	GRMZM5G806449_P01,GRMZM5G806449_P03	TRUE	TRUE	gEELIADVVLFATGR	95%	n+304 (+304)	36.72	25.55
1869	seq=translation; coord=5:215289055..215297830:1; parent_transcript=GRMZM5G806449_T01; parent_gene=GRMZM5G806449	GRMZM5G806449_P01,GRMZM5G806449_P03	TRUE	TRUE	gMGAEVDLFYR	95%	n+304 (+304)	31.72	25.00

1870	seq=translation; coord=5:215289055..215297830:1; parent_transcript=GRMZM5G806449_T01; parent_gene=GRMZM5G806449	GRMZM5G806449_P01,GRMZM5G806449_P03	TRUE	TRUE	iNLTPVALMEATcFAk	95%	n+304 (+304), Carbamidomethyl (+57), K+304 (+304)	47.37	25.43
1871	seq=translation; coord=5:215289055..215297830:1; parent_transcript=GRMZM5G806449_T01; parent_gene=GRMZM5G806449	GRMZM5G806449_P01,GRMZM5G806449_P03	TRUE	TRUE	lNLQAAGVEIDR	95%	n+304 (+304)	47.30	26.72
1872	seq=translation; coord=5:215289055..215297830:1; parent_transcript=GRMZM5G806449_T01; parent_gene=GRMZM5G806449	GRMZM5G806449_P01,GRMZM5G806449_P03	TRUE	TRUE	tSAPSVWAVGDVTNR	95%	n+304 (+304)	67.71	25.00
1873	seq=translation; coord=5:215289055..215297830:1; parent_transcript=GRMZM5G806449_T01; parent_gene=GRMZM5G806449	GRMZM5G806449_P01,GRMZM5G806449_P03	TRUE	TRUE	vVTDkGEELIADVVLFATGR	95%	n+304 (+304), K+304 (+304)	32.80	25.00
1874	seq=translation; coord=4:2762573..2764339:1; parent_transcript=GRMZM2G126541_T01; parent_gene=GRMZM2G126541	GRMZM2G126541_P01	TRUE	TRUE	aAGFMNDAGVR	91%	n+304 (+304)	28.01	25.00
1875	seq=translation; coord=4:2762573..2764339:1; parent_transcript=GRMZM2G126541_T01; parent_gene=GRMZM2G126541	GRMZM2G126541_P01	TRUE	TRUE	aRPLFLTGESYAGk	95%	n+304 (+304), K+304 (+304)	41.29	25.33
1876	seq=translation; coord=4:2762573..2764339:1; parent_transcript=GRMZM2G126541_T01; parent_gene=GRMZM2G126541	GRMZM2G126541_P01	TRUE	TRUE	dAPAPPWELVSAAVVAALHDDVMk	95%	n+304 (+304), K+304 (+304)	31.35	25.00
1877	seq=translation; coord=4:2762573..2764339:1; parent_transcript=GRMZM2G126541_T01; parent_gene=GRMZM2G126541	GRMZM2G126541_P01	TRUE	TRUE	dAQVGVVSVEALLR	95%	n+304 (+304)	62.59	25.90
1878	seq=translation; coord=4:2762573..2764339:1; parent_transcript=GRMZM2G126541_T01; parent_gene=GRMZM2G126541	GRMZM2G126541_P01	TRUE	TRUE	eAAAMQAEAAALAAAGR	95%	n+304 (+304)	88.81	25.00
1879	seq=translation; coord=4:2762573..2764339:1; parent_transcript=GRMZM2G126541_T01; parent_gene=GRMZM2G126541	GRMZM2G126541_P01	TRUE	TRUE	eAVDAWFR	91%	n+304 (+304)	27.95	25.00
1880	seq=translation; coord=4:2762573..2764339:1; parent_transcript=GRMZM2G126541_T01; parent_gene=GRMZM2G126541	GRMZM2G126541_P01	TRUE	TRUE	eLDWDGLAAFR	95%	n+304 (+304)	48.31	25.00
1881	seq=translation; coord=4:2762573..2764339:1; parent_transcript=GRMZM2G126541_T01; parent_gene=GRMZM2G126541	GRMZM2G126541_P01	TRUE	TRUE	rDAPAPPWELVSAAVVAALHDDVMk	95%	n+304 (+304), K+304 (+304)	48.17	25.00
1882	seq=translation; coord=4:2762573..2764339:1; parent_transcript=GRMZM2G126541_T01; parent_gene=GRMZM2G126541	GRMZM2G126541_P01	TRUE	TRUE	rEAAAMQAEAAALAAAGR	93%	n+304 (+304)	28.35	25.69
1883	seq=translation; coord=9:33125819..33139794:1; parent_transcript=GRMZM2G058675_T02; parent_gene=GRMZM2G058675	GRMZM2G058675_P02	TRUE	TRUE	fADLIEk	92%	n+304 (+304), K+304 (+304)	26.59	25.00
1884	seq=translation; coord=9:33125819..33139794:1; parent_transcript=GRMZM2G058675_T02; parent_gene=GRMZM2G058675	GRMZM2G058675_P02	TRUE	TRUE	gFYIQPTIFSDVQDGMk	95%	n+304 (+304), K+304 (+304)	64.38	25.15
1885	seq=translation; coord=9:33125819..33139794:1; parent_transcript=GRMZM2G058675_T02; parent_gene=GRMZM2G058675	GRMZM2G058675_P02	TRUE	TRUE	gVEQGPQIDDEQFNk	95%	n+304 (+304), K+304 (+304)	45.47	25.00
1886	seq=translation; coord=9:33125819..33139794:1; parent_transcript=GRMZM2G058675_T02; parent_gene=GRMZM2G058675	GRMZM2G058675_P02	TRUE	TRUE	iAQEEIFGPVQSILk	95%	n+304 (+304), K+304 (+304)	54.95	25.00
1887	seq=translation; coord=9:33125819..33139794:1; parent_transcript=GRMZM2G058675_T02; parent_gene=GRMZM2G058675	GRMZM2G058675_P02	TRUE	TRUE	iILELAAk	95%	n+304 (+304), K+304 (+304)	37.97	25.00

1888	seq=translation; coord=9:33125819..33139794:1; parent_transcript=GRMZM2G058675_T02; parent_gene=GRMZM2G058675	GRMZM2G058675_P02	TRUE	TRUE	lLHEAGLPEGVVNVVSGFGPTAGAALASHMDVDk	95%	n+304 (+304), K+304 (+304)	34.28	25.00
1889	seq=translation; coord=9:33125819..33139794:1; parent_transcript=GRMZM2G058675_T02; parent_gene=GRMZM2G058675	GRMZM2G058675_P02	TRUE	TRUE	tAEQTPLSALYISk	95%	n+304 (+304), K+304 (+304)	29.55	25.00
1890	seq=translation; coord=9:33125819..33139794:1; parent_transcript=GRMZM2G058675_T02; parent_gene=GRMZM2G058675	GRMZM2G058675_P02	TRUE	TRUE	tGEVIAHVAEGDAEDINR	95%	n+304 (+304)	61.83	25.00
1891	seq=translation; coord=9:33125819..33139794:1; parent_transcript=GRMZM2G058675_T02; parent_gene=GRMZM2G058675	GRMZM2G058675_P02	TRUE	TRUE	tVTLELGGk	95%	n+304 (+304), K+304 (+304)	35.29	26.39
1892	seq=translation; coord=9:33125819..33139794:1; parent_transcript=GRMZM2G058675_T02; parent_gene=GRMZM2G058675	GRMZM2G058675_P02	TRUE	TRUE	yGVDGGATLVTGGDR	95%	n+304 (+304)	35.85	25.00
1893	seq=translation; coord=9:33125819..33139794:1; parent_transcript=GRMZM2G058675_T02; parent_gene=GRMZM2G058675	GRMZM2G058675_P02	TRUE	TRUE	yGVDGGATLVTGGDR	95%	n+304 (+304), iTRAQ8plex (+304)	40.07	26.10
1894	seq=translation; coord=1:228251511..228256942:-1; parent_transcript=GRMZM2G346455_T05; parent_gene=GRMZM2G346455	GRMZM2G346455_P05,GRMZM2G346455_P06	TRUE	TRUE	aLADYVHAk	95%	n+304 (+304), K+304 (+304)	36.08	26.44
1895	seq=translation; coord=1:228251511..228256942:-1; parent_transcript=GRMZM2G346455_T05; parent_gene=GRMZM2G346455	GRMZM2G346455_P05,GRMZM2G346455_P06	TRUE	TRUE	aMSQQTMGILSNSEVIAVNQDSQGAQGk	95%	n+304 (+304), K+304 (+304)	56.46	25.00
1896	seq=translation; coord=1:228251511..228256942:-1; parent_transcript=GRMZM2G346455_T05; parent_gene=GRMZM2G346455	GRMZM2G346455_P05,GRMZM2G346455_P06	TRUE	TRUE	aPLLIGcDVR	89%	n+304 (+304), Carbamidomethyl (+57)	27.38	25.56
1897	seq=translation; coord=1:228251511..228256942:-1; parent_transcript=GRMZM2G346455_T05; parent_gene=GRMZM2G346455	GRMZM2G346455_P05,GRMZM2G346455_P06	TRUE	TRUE	dYQGSFVANR	93%	n+304 (+304)	29.66	25.00
1898	seq=translation; coord=1:228251511..228256942:-1; parent_transcript=GRMZM2G346455_T05; parent_gene=GRMZM2G346455	GRMZM2G346455_P05,GRMZM2G346455_P06	TRUE	TRUE	gIFFSLcEWGR	95%	n+304 (+304), Carbamidomethyl (+57)	36.56	25.00
1899	seq=translation; coord=1:228251511..228256942:-1; parent_transcript=GRMZM2G346455_T05; parent_gene=GRMZM2G346455	GRMZM2G346455_P05,GRMZM2G346455_P06	TRUE	TRUE	qNPATWAGGMGNSWR	95%	n+304 (+304)	50.67	25.00
1900	seq=translation; coord=1:228251511..228256942:-1; parent_transcript=GRMZM2G346455_T05; parent_gene=GRMZM2G346455	GRMZM2G346455_P05,GRMZM2G346455_P06	TRUE	TRUE	qTADALVNTGLAk	95%	n+304 (+304), K+304 (+304)	42.57	26.19
1901	seq=translation; coord=1:228251511..228256942:-1; parent_transcript=GRMZM2G346455_T05; parent_gene=GRMZM2G346455	GRMZM2G346455_P05,GRMZM2G346455_P06	TRUE	TRUE	tFSSWGIDYLk	95%	n+304 (+304), K+304 (+304)	36.34	26.02
1902	seq=translation; coord=1:228251511..228256942:-1; parent_transcript=GRMZM2G346455_T05; parent_gene=GRMZM2G346455	GRMZM2G346455_P05,GRMZM2G346455_P06	TRUE	TRUE	tTDDIADNWGSMTSR	95%	n+304 (+304)	81.64	25.00
1903	seq=translation; coord=1:228251511..228256942:-1; parent_transcript=GRMZM2G346455_T05; parent_gene=GRMZM2G346455	GRMZM2G346455_P05,GRMZM2G346455_P06	TRUE	TRUE	tTDDIADNWGSmTSR	95%	n+304 (+304), Oxidation (+16)	37.87	25.00
1904	seq=translation; coord=10:144060305..144061413:1; parent_transcript=GRMZM5G895313_T01; parent_gene=GRMZM5G895313	GRMZM5G895313_P01	TRUE	TRUE	dcSQGGGYGGGGGGGR	95%	n+304 (+304), Carbamidomethyl (+57)	43.20	25.00
1905	seq=translation; coord=10:144060305..144061413:1; parent_transcript=GRMZM5G895313_T01; parent_gene=GRMZM5G895313	GRMZM5G895313_P01	TRUE	TRUE	dcSQGGGyGGGGGGGR	95%	n+304 (+304), Carbamidomethyl (+57), iTRAQ8plex (+304)	27.37	25.00

1906	seq=translation; coord=10:144060305..144061413:1; parent_transcript=GRMZM5G895313_T01; parent_gene=GRMZM5G895313	GRMZM5G895313_P01	TRUE	TRUE	gYGGGGGYGGGYGGGGGGGGR	95%	n+304 (+304)	93.86	25.00
1907	seq=translation; coord=10:144060305..144061413:1; parent_transcript=GRMZM5G895313_T01; parent_gene=GRMZM5G895313	GRMZM5G895313_P01	TRUE	TRUE	gYGGGGGYGGGyGGGGGGGGR	94%	n+304 (+304), iTRAQ8plex (+304)	27.96	25.00
1908	seq=translation; coord=10:144060305..144061413:1; parent_transcript=GRMZM5G895313_T01; parent_gene=GRMZM5G895313	GRMZM5G895313_P01	TRUE	TRUE	sLNDGDAVEYTVGSGNDGR	95%	n+304 (+304)	79.57	25.00
1909	seq=translation; coord=2:2548940..2552572:1; parent_transcript=GRMZM2G010435_T01; parent_gene=GRMZM2G010435	GRMZM2G010435_P01	TRUE	TRUE	aVAHQPVSVAIEAGGR	95%	n+304 (+304)	61.78	25.61
1910	seq=translation; coord=2:2548940..2552572:1; parent_transcript=GRMZM2G010435_T01; parent_gene=GRMZM2G010435	GRMZM2G010435_P01	TRUE	TRUE	eFQLYk	92%	n+304 (+304), K+304 (+304)	28.71	25.00
1911	seq=translation; coord=2:2548940..2552572:1; parent_transcript=GRMZM2G010435_T01; parent_gene=GRMZM2G010435	GRMZM2G010435_P01	TRUE	TRUE	gAVAPVk	90%	n+304 (+304), K+304 (+304)	25.41	25.00
1912	seq=translation; coord=2:2548940..2552572:1; parent_transcript=GRMZM2G010435_T01; parent_gene=GRMZM2G010435	GRMZM2G010435_P01	TRUE	TRUE	vVSIDGFEDVPENDEk	95%	n+304 (+304), K+304 (+304)	47.03	25.00
1913	seq=translation; coord=2:2548940..2552572:1; parent_transcript=GRMZM2G010435_T01; parent_gene=GRMZM2G010435	GRMZM2G010435_P01	TRUE	TRUE	wGEDGYIR	91%	n+304 (+304)	27.89	25.00
1914	seq=translation; coord=2:4299718..4306692:-1; parent_transcript=GRMZM2G019404_T01; parent_gene=GRMZM2G019404	GRMZM2G019404_P01,GRMZM2G019404_P02	TRUE	TRUE	aDIGIAVADATDAAR	95%	n+304 (+304)	55.22	25.28
1915	seq=translation; coord=2:4299718..4306692:-1; parent_transcript=GRMZM2G019404_T01; parent_gene=GRMZM2G019404	GRMZM2G019404_P01,GRMZM2G019404_P02	TRUE	TRUE	eIHFLPFNPVDk	95%	n+304 (+304), K+304 (+304)	36.57	26.70
1916	seq=translation; coord=2:4299718..4306692:-1; parent_transcript=GRMZM2G019404_T01; parent_gene=GRMZM2G019404	GRMZM2G019404_P01,GRMZM2G019404_P02	TRUE	TRUE	eSPGGPWQFVGLLPLFDPPR	95%	n+304 (+304)	49.59	25.42
1917	seq=translation; coord=2:4299718..4306692:-1; parent_transcript=GRMZM2G019404_T01; parent_gene=GRMZM2G019404	GRMZM2G019404_P01,GRMZM2G019404_P02	TRUE	TRUE	gAPEQILDLcHck	95%	n+304 (+304), Carbamidomethyl (+57), Carbamidomethyl (+57), K+304 (+304)	43.60	25.35
1918	seq=translation; coord=2:4299718..4306692:-1; parent_transcript=GRMZM2G019404_T01; parent_gene=GRMZM2G019404	GRMZM2G019404_P01,GRMZM2G019404_P02	TRUE	TRUE	hIVGMTGDGVNDAPALk	95%	n+304 (+304), K+304 (+304)	52.75	26.68
1919	seq=translation; coord=2:4299718..4306692:-1; parent_transcript=GRMZM2G019404_T01; parent_gene=GRMZM2G019404	GRMZM2G019404_P01,GRMZM2G019404_P02	TRUE	TRUE	lGDIVPADAR	91%	n+304 (+304)	29.72	26.36
1920	seq=translation; coord=2:4299718..4306692:-1; parent_transcript=GRMZM2G019404_T01; parent_gene=GRMZM2G019404	GRMZM2G019404_P01,GRMZM2G019404_P02	TRUE	TRUE	nEAVDLENIPIEEVFEQLk	94%	n+304 (+304), K+304 (+304)	26.03	25.07
1921	seq=translation; coord=2:4299718..4306692:-1; parent_transcript=GRMZM2G019404_T01; parent_gene=GRMZM2G019404	GRMZM2G019404_P01,GRMZM2G019404_P02	TRUE	TRUE	nkESPGGPWQFVGLLPLFDPPR	89%	n+304 (+304), K+304 (+304)	25.32	25.31
1922	seq=translation; coord=2:4299718..4306692:-1; parent_transcript=GRMZM2G019404_T01; parent_gene=GRMZM2G019404	GRMZM2G019404_P01,GRMZM2G019404_P02	TRUE	TRUE	nLVEVFck	95%	n+304 (+304), Carbamidomethyl (+57), K+304 (+304)	39.36	25.43

1923	seq=translation; coord=2:4299718..4306692:-1; parent_transcript=GRMZM2G019404_T01; parent_gene=GRMZM2G019404	GRMZM2G019404_P01,GRMZM2G019404_P02	TRUE	TRUE	wGEQEAAILVPGDIISIk	95%	n+304 (+304), K+304 (+304)	37.92	25.56
1924	seq=translation; coord=2:51927314..51930580:1; parent_transcript=GRMZM2G109677_T01; parent_gene=GRMZM2G109677	GRMZM2G109677_P01,GRMZM2G109677_P02, GRMZM2G109677_P04,GRMZM2G132968_P02, GRMZM2G135727_P01,GRMZM2G135727_P02, GRMZM2G324314_P01,GRMZM2G324314_P03, GRMZM2G324314_P04,GRMZM5G801409_P02, GRMZM5G801409_P03,GRMZM5G801409_P04	TRUE	TRUE	aGQETHDASTEFDR	95%	n+304 (+304)	34.61	25.00
1925	seq=translation; coord=2:51927314..51930580:1; parent_transcript=GRMZM2G109677_T01; parent_gene=GRMZM2G109677	GRMZM2G109677_P01,GRMZM2G109677_P02, GRMZM2G109677_P04,GRMZM2G132968_P02, GRMZM2G135727_P01,GRMZM2G135727_P02, GRMZM2G324314_P01,GRMZM2G324314_P03, GRMZM2G324314_P04,GRMZM5G801409_P02, GRMZM5G801409_P03,GRMZM5G801409_P04	TRUE	TRUE	dEMIDIIGVTk	95%	n+304 (+304), K+304 (+304)	47.85	26.00
1926	seq=translation; coord=2:51927314..51930580:1; parent_transcript=GRMZM2G109677_T01; parent_gene=GRMZM2G109677	GRMZM2G109677_P01,GRMZM2G109677_P02, GRMZM2G109677_P04,GRMZM2G132968_P02, GRMZM2G135727_P01,GRMZM2G135727_P02, GRMZM2G324314_P01,GRMZM2G324314_P03, GRMZM2G324314_P04,GRMZM5G801409_P02, GRMZM5G801409_P03,GRMZM5G801409_P04	TRUE	TRUE	dITPMGGFPHYGIVk	95%	n+304 (+304), K+304 (+304)	31.02	26.78
1927	seq=translation; coord=2:51927314..51930580:1; parent_transcript=GRMZM2G109677_T01; parent_gene=GRMZM2G109677	GRMZM2G109677_P01,GRMZM2G109677_P02, GRMZM2G109677_P04,GRMZM2G132968_P02, GRMZM2G135727_P01,GRMZM2G135727_P02, GRMZM2G324314_P01,GRMZM2G324314_P03, GRMZM2G324314_P04,GRMZM5G801409_P02, GRMZM5G801409_P03,GRMZM5G801409_P04	TRUE	TRUE	eIQLQLEk	95%	n+304 (+304), K+304 (+304)	48.97	26.28
1928	seq=translation; coord=2:51927314..51930580:1; parent_transcript=GRMZM2G109677_T01; parent_gene=GRMZM2G109677	GRMZM2G109677_P01,GRMZM2G109677_P02, GRMZM2G109677_P04,GRMZM2G132968_P02, GRMZM2G135727_P01,GRMZM2G135727_P02, GRMZM2G324314_P01,GRMZM2G324314_P03, GRMZM2G324314_P04,GRMZM5G801409_P02, GRMZM5G801409_P03,GRMZM5G801409_P04	TRUE	TRUE	eVPVDAVFQk	95%	n+304 (+304), K+304 (+304)	52.36	26.45
1929	seq=translation; coord=2:51927314..51930580:1; parent_transcript=GRMZM2G109677_T01; parent_gene=GRMZM2G109677	GRMZM2G109677_P01,GRMZM2G109677_P02, GRMZM2G109677_P04,GRMZM2G132968_P02, GRMZM2G135727_P01,GRMZM2G135727_P02, GRMZM2G324314_P01,GRMZM2G324314_P03, GRMZM2G324314_P04,GRMZM5G801409_P02, GRMZM5G801409_P03,GRMZM5G801409_P04	TRUE	TRUE	gDYLMIk	93%	n+304 (+304), K+304 (+304)	29.05	26.44
1930	seq=translation; coord=2:51927314..51930580:1; parent_transcript=GRMZM2G109677_T01; parent_gene=GRMZM2G109677	GRMZM2G109677_P01,GRMZM2G109677_P02, GRMZM2G109677_P04,GRMZM2G132968_P02, GRMZM2G135727_P01,GRMZM2G135727_P02, GRMZM2G324314_P01,GRMZM2G324314_P03, GRMZM2G324314_P04,GRMZM5G801409_P02, GRMZM5G801409_P03,GRMZM5G801409_P04	TRUE	TRUE	gYEGVVTR	86%	n+304 (+304)	25.25	25.00

1931	seq=translation; coord=2:51927314..51930580:1; parent_transcript=GRMZM2G109677_T01; parent_gene=GRMZM2G109677	GRMZM2G109677_P01,GRMZM2G109677_P02, GRMZM2G109677_P04,GRMZM2G132968_P02, GRMZM2G135727_P01,GRMZM2G135727_P02, GRMZM2G324314_P01,GRMZM2G324314_P03, GRMZM2G324314_P04,GRMZM5G801409_P02, GRMZM5G801409_P03,GRMZM5G801409_P04	TRUE	TRUE	lALEEIk	86%	n+304 (+304), K+304 (+304)	25.28	25.00
1932	seq=translation; coord=2:51927314..51930580:1; parent_transcript=GRMZM2G109677_T01; parent_gene=GRMZM2G109677	GRMZM2G109677_P01,GRMZM2G109677_P02, GRMZM2G109677_P04,GRMZM2G132968_P02, GRMZM2G135727_P01,GRMZM2G135727_P02, GRMZM2G324314_P01,GRMZM2G324314_P03, GRMZM2G324314_P04,GRMZM5G801409_P02, GRMZM5G801409_P03,GRMZM5G801409_P04	TRUE	TRUE	vAcIGAWHPAR	95%	n+304 (+304), Carbamidomethyl (+57)	52.17	25.00
1933	seq=translation; coord=3:64458994..64462315:1; parent_transcript=GRMZM2G128171_T02; parent_gene=GRMZM2G128171	GRMZM2G128171_P02	TRUE	TRUE	aDEFIIk	95%	n+304 (+304), K+304 (+304)	37.87	25.05
1934	seq=translation; coord=3:64458994..64462315:1; parent_transcript=GRMZM2G128171_T02; parent_gene=GRMZM2G128171	GRMZM2G128171_P02	TRUE	TRUE	eVLSSMEEEADk	95%	n+304 (+304), K+304 (+304)	35.26	25.00
1935	seq=translation; coord=3:64458994..64462315:1; parent_transcript=GRMZM2G128171_T02; parent_gene=GRMZM2G128171	GRMZM2G128171_P02	TRUE	TRUE	eVLSSMEEEADkLSGSAAR	94%	n+304 (+304), K+304 (+304)	28.50	25.00
1936	seq=translation; coord=3:64458994..64462315:1; parent_transcript=GRMZM2G128171_T02; parent_gene=GRMZM2G128171	GRMZM2G128171_P02	TRUE	TRUE	iASLDVLAk	95%	n+304 (+304), K+304 (+304)	37.61	25.00
1937	seq=translation; coord=3:64458994..64462315:1; parent_transcript=GRMZM2G128171_T02; parent_gene=GRMZM2G128171	GRMZM2G128171_P02	TRUE	TRUE	lAPEYER	86%	n+304 (+304)	25.40	25.00
1938	seq=translation; coord=3:64458994..64462315:1; parent_transcript=GRMZM2G128171_T02; parent_gene=GRMZM2G128171	GRMZM2G128171_P02	TRUE	TRUE	lATIPSSVVVLTPETFDSIVLDETk	95%	n+304 (+304), K+304 (+304)	35.26	25.00
1939	seq=translation; coord=3:64458994..64462315:1; parent_transcript=GRMZM2G128171_T02; parent_gene=GRMZM2G128171	GRMZM2G128171_P02	TRUE	TRUE	lDEGVVIANLDADk	93%	n+304 (+304), K+304 (+304)	28.80	26.56
1940	seq=translation; coord=3:64458994..64462315:1; parent_transcript=GRMZM2G128171_T02; parent_gene=GRMZM2G128171	GRMZM2G128171_P02	TRUE	TRUE	tAEALAEFLNTEGGTNVk	95%	n+304 (+304), K+304 (+304)	53.38	25.21
1941	seq=translation; coord=8:39208505..39211382:-1; parent_transcript=GRMZM2G335657_T01; parent_gene=GRMZM2G335657	GRMZM2G335657_P01	TRUE	TRUE	aAQLGLk	95%	n+304 (+304), K+304 (+304)	31.99	25.00
1942	seq=translation; coord=8:39208505..39211382:-1; parent_transcript=GRMZM2G335657_T01; parent_gene=GRMZM2G335657	GRMZM2G335657_P01	TRUE	TRUE	gTLGGTcLNVGcIPSk	95%	n+304 (+304), Carbamidomethyl (+57), Carbamidomethyl (+57), K+304 (+304)	57.54	25.73
1943	seq=translation; coord=8:39208505..39211382:-1; parent_transcript=GRMZM2G335657_T01; parent_gene=GRMZM2G335657	GRMZM2G335657_P01	TRUE	TRUE	tEEQVTALGIPYR	95%	n+304 (+304)	36.85	25.00
1944	seq=translation; coord=9:132550417..132551258:1; parent_transcript=GRMZM2G404249_T01; parent_gene=GRMZM2G404249	GRMZM2G404249_P01,GRMZM5G899188_P01	TRUE	TRUE	eLAGAYAFVVDMPGLSTGDIR	95%	n+304 (+304)	55.62	25.00
1945	seq=translation; coord=9:132550417..132551258:1; parent_transcript=GRMZM2G404249_T01; parent_gene=GRMZM2G404249	GRMZM2G404249_P01,GRMZM5G899188_P01	TRUE	TRUE	eLAGAyAFVVDMPGLSTGDIR	95%	n+304 (+304), iTRAQ8plex (+304)	33.45	25.00

1946	seq=translation; coord=9:132550417..132551258:1; parent_transcript=GRMZM2G404249_T01; parent_gene=GRMZM2G404249	GRMZM2G404249_P01,GRMZM5G899188_P01	TRUE	TRUE	mFGLETPR	92%	n+304 (+304)	27.39	25.00
1947	seq=translation; coord=6:125154676..125157008:1; parent_transcript=GRMZM2G450233_T01; parent_gene=GRMZM2G450233	GRMZM2G450233_P01	TRUE	TRUE	aLVDSFVR	92%	n+304 (+304)	28.70	25.00
1948	seq=translation; coord=6:125154676..125157008:1; parent_transcript=GRMZM2G450233_T01; parent_gene=GRMZM2G450233	GRMZM2G450233_P01	TRUE	TRUE	aYAFLLk	95%	n+304 (+304), K+304 (+304)	42.78	25.00
1949	seq=translation; coord=6:125154676..125157008:1; parent_transcript=GRMZM2G450233_T01; parent_gene=GRMZM2G450233	GRMZM2G450233_P01	TRUE	TRUE	dSIPNSPSLR	89%	n+304 (+304)	26.95	25.00
1950	seq=translation; coord=6:125154676..125157008:1; parent_transcript=GRMZM2G450233_T01; parent_gene=GRMZM2G450233	GRMZM2G450233_P01	TRUE	TRUE	dSVVLTGGLGYk	95%	n+304 (+304), K+304 (+304)	50.82	25.00
1951	seq=translation; coord=6:125154676..125157008:1; parent_transcript=GRMZM2G450233_T01; parent_gene=GRMZM2G450233	GRMZM2G450233_P01	TRUE	TRUE	fFDVVDR	93%	n+304 (+304)	29.34	25.00
1952	seq=translation; coord=6:125154676..125157008:1; parent_transcript=GRMZM2G450233_T01; parent_gene=GRMZM2G450233	GRMZM2G450233_P01	TRUE	TRUE	gcDGSVLIDSTANNk	95%	n+304 (+304), Carbamidomethyl (+57), K+304 (+304)	39.26	25.00
1953	seq=translation; coord=6:125154676..125157008:1; parent_transcript=GRMZM2G450233_T01; parent_gene=GRMZM2G450233	GRMZM2G450233_P01	TRUE	TRUE	mGQIEVLTGTQGEIR	95%	n+304 (+304)	55.07	25.33
1954	seq=translation; coord=6:125154676..125157008:1; parent_transcript=GRMZM2G450233_T01; parent_gene=GRMZM2G450233	GRMZM2G450233_P01	TRUE	TRUE	mHFHDcFVR	95%	n+304 (+304), Carbamidomethyl (+57)	37.68	25.00
1955	seq=translation; coord=8:160390396..160393378:1; parent_transcript=GRMZM2G134256_T01; parent_gene=GRMZM2G134256	GRMZM2G134256_P01	TRUE	TRUE	aISSSSAYDDQFk	95%	n+304 (+304), K+304 (+304)	54.18	25.00
1956	seq=translation; coord=8:160390396..160393378:1; parent_transcript=GRMZM2G134256_T01; parent_gene=GRMZM2G134256	GRMZM2G134256_P01	TRUE	TRUE	dAESAYWELVIk	95%	n+304 (+304), K+304 (+304)	47.72	26.11
1957	seq=translation; coord=8:160390396..160393378:1; parent_transcript=GRMZM2G134256_T01; parent_gene=GRMZM2G134256	GRMZM2G134256_P01	TRUE	TRUE	gVTSNPTIFQk	95%	n+304 (+304), K+304 (+304)	56.93	25.56
1958	seq=translation; coord=8:160390396..160393378:1; parent_transcript=GRMZM2G134256_T01; parent_gene=GRMZM2G134256	GRMZM2G134256_P01	TRUE	TRUE	iGTPEALALR	91%	n+304 (+304)	29.71	26.54
1959	seq=translation; coord=8:160390396..160393378:1; parent_transcript=GRMZM2G134256_T01; parent_gene=GRMZM2G134256	GRMZM2G134256_P01	TRUE	TRUE	lGIDWEEVGk	95%	n+304 (+304), K+304 (+304)	30.42	25.48
1960	seq=translation; coord=8:160390396..160393378:1; parent_transcript=GRMZM2G134256_T01; parent_gene=GRMZM2G134256	GRMZM2G134256_P01	TRUE	TRUE	lLWASTGVk	94%	n+304 (+304), K+304 (+304)	30.32	25.00
1961	seq=translation; coord=8:160390396..160393378:1; parent_transcript=GRMZM2G134256_T01; parent_gene=GRMZM2G134256	GRMZM2G134256_P01	TRUE	TRUE	qLELEGVDSFk	95%	n+304 (+304), K+304 (+304)	37.82	26.13
1962	seq=translation; coord=8:160390396..160393378:1; parent_transcript=GRMZM2G134256_T01; parent_gene=GRMZM2G134256	GRMZM2G134256_P01	TRUE	TRUE	sFDSLLVSLQEk	95%	n+304 (+304), K+304 (+304)	33.33	25.84
1963	seq=translation; coord=8:160390396..160393378:1; parent_transcript=GRMZM2G134256_T01; parent_gene=GRMZM2G134256	GRMZM2G134256_P01	TRUE	TRUE	tVDANVSEAEGVYSALEk	95%	n+304 (+304), K+304 (+304)	44.24	25.60

1964	seq=translation; coord=8:160390396..160393378:1; parent_transcript=GRMZM2G134256_T01; parent_gene=GRMZM2G134256	GRMZM2G134256_P01	TRUE	TRUE	vDTLIDk	87%	n+304 (+304), K+304 (+304)	25.87	25.00
1965	seq=translation; coord=8:160390396..160393378:1; parent_transcript=GRMZM2G134256_T01; parent_gene=GRMZM2G134256	GRMZM2G134256_P01	TRUE	TRUE	vTSVASFFVSR	88%	n+304 (+304)	27.06	25.68
1966	seq=translation; coord=2:210034645..210036617:1; parent_transcript=GRMZM2G339994_T01; parent_gene=GRMZM2G339994	GRMZM2G339994_P01,GRMZM2G339994_P02	TRUE	TRUE	aLSLQLADR	89%	n+304 (+304)	28.76	27.02
1967	seq=translation; coord=2:210034645..210036617:1; parent_transcript=GRMZM2G339994_T01; parent_gene=GRMZM2G339994	GRMZM2G339994_P01,GRMZM2G339994_P02	TRUE	TRUE	aVcLcFAk	95%	n+304 (+304), Carbamidomethyl (+57), Carbamidomethyl (+57), K+304 (+304)	30.54	26.50
1968	seq=translation; coord=2:210034645..210036617:1; parent_transcript=GRMZM2G339994_T01; parent_gene=GRMZM2G339994	GRMZM2G339994_P01,GRMZM2G339994_P02	TRUE	TRUE	eGATVAFTFVR	95%	n+304 (+304)	35.98	25.01
1969	seq=translation; coord=2:210034645..210036617:1; parent_transcript=GRMZM2G339994_T01; parent_gene=GRMZM2G339994	GRMZM2G339994_P01,GRMZM2G339994_P02	TRUE	TRUE	eSIGDVTEADLER	89%	n+304 (+304)	25.08	25.00
1970	seq=translation; coord=2:210034645..210036617:1; parent_transcript=GRMZM2G339994_T01; parent_gene=GRMZM2G339994	GRMZM2G339994_P01,GRMZM2G339994_P02	TRUE	TRUE	iDVVVNNAAEQYER	95%	n+304 (+304)	83.91	25.00
1971	seq=translation; coord=2:210034645..210036617:1; parent_transcript=GRMZM2G339994_T01; parent_gene=GRMZM2G339994	GRMZM2G339994_P01,GRMZM2G339994_P02	TRUE	TRUE	tNIFSYFLVSk	95%	n+304 (+304), K+304 (+304)	44.22	25.59
1972	seq=translation; coord=2:210034645..210036617:1; parent_transcript=GRMZM2G339994_T01; parent_gene=GRMZM2G339994	GRMZM2G339994_P01,GRMZM2G339994_P02	TRUE	TRUE	vALVTGGDSGIGR	95%	n+304 (+304)	51.25	26.45
1973	seq=translation; coord=3:11894936..11898354:1; parent_transcript=GRMZM2G096240_T01; parent_gene=GRMZM2G096240	GRMZM2G096240_P01,GRMZM2G098335_P01	TRUE	TRUE	fDELMAAAAEER	95%	n+304 (+304)	56.33	25.00
1974	seq=translation; coord=3:11894936..11898354:1; parent_transcript=GRMZM2G096240_T01; parent_gene=GRMZM2G096240	GRMZM2G096240_P01,GRMZM2G098335_P01	TRUE	TRUE	iEIILGk	95%	n+304 (+304), K+304 (+304)	30.47	25.00
1975	seq=translation; coord=3:11894936..11898354:1; parent_transcript=GRMZM2G096240_T01; parent_gene=GRMZM2G096240	GRMZM2G096240_P01,GRMZM2G098335_P01	TRUE	TRUE	nLTVTGDAAASGGEGQR	95%	n+304 (+304)	49.13	25.00
1976	seq=translation; coord=3:11894936..11898354:1; parent_transcript=GRMZM2G096240_T01; parent_gene=GRMZM2G096240	GRMZM2G096240_P01,GRMZM2G098335_P01	TRUE	TRUE	sDkFDELMAAAAEER	95%	n+304 (+304), K+304 (+304)	40.84	25.00
1977	seq=translation; coord=3:11894936..11898354:1; parent_transcript=GRMZM2G096240_T01; parent_gene=GRMZM2G096240	GRMZM2G096240_P01,GRMZM2G098335_P01	TRUE	TRUE	tSTVEINDESR	95%	n+304 (+304)	35.02	25.00
1978	seq=translation; coord=3:11894936..11898354:1; parent_transcript=GRMZM2G096240_T01; parent_gene=GRMZM2G096240	GRMZM2G096240_P01,GRMZM2G098335_P01	TRUE	TRUE	vVEEIAEGVk	95%	n+304 (+304), K+304 (+304)	42.61	26.39
1979	seq=translation; coord=4:44859117..44861815:-1; parent_transcript=GRMZM2G006953_T02; parent_gene=GRMZM2G006953	GRMZM2G006953_P02,GRMZM2G407249_P01	TRUE	TRUE	aALVGLYQETSMLTFEGQk	95%	n+304 (+304), K+304 (+304)	79.01	25.26
1980	seq=translation; coord=4:44859117..44861815:-1; parent_transcript=GRMZM2G006953_T02; parent_gene=GRMZM2G006953	GRMZM2G006953_P02,GRMZM2G407249_P01	TRUE	TRUE	aALVGLYQETSmLTFEGQk	89%	n+304 (+304), Oxidation (+16), K+304 (+304)	25.59	25.48

1981	seq=translation; coord=4:44859117..44861815:-1; parent_transcript=GRMZM2G006953_T02; parent_gene=GRMZM2G006953	GRMZM2G006953_P02,GRMZM2G407249_P01	TRUE	TRUE	aFVEHYYR	95%	n+304 (+304)	36.74	25.00
1982	seq=translation; coord=4:44859117..44861815:-1; parent_transcript=GRMZM2G006953_T02; parent_gene=GRMZM2G006953	GRMZM2G006953_P02,GRMZM2G407249_P01	TRUE	TRUE	fQGPSAIAGk	95%	n+304 (+304), K+304 (+304)	38.00	25.93
1983	seq=translation; coord=4:44859117..44861815:-1; parent_transcript=GRMZM2G006953_T02; parent_gene=GRMZM2G006953	GRMZM2G006953_P02,GRMZM2G407249_P01	TRUE	TRUE	fSQAFHLLPAAGSFFVQNDMFR	95%	n+304 (+304)	45.38	25.00
1984	seq=translation; coord=5:63800401..63802605:-1; parent_transcript=GRMZM5G834758_T02; parent_gene=GRMZM5G834758	GRMZM5G834758_P02,GRMZM5G834758_P03	TRUE	TRUE	aIAAYNk	92%	n+304 (+304), K+304 (+304)	27.12	25.40
1985	seq=translation; coord=5:63800401..63802605:-1; parent_transcript=GRMZM5G834758_T02; parent_gene=GRMZM5G834758	GRMZM5G834758_P02,GRMZM5G834758_P03	TRUE	TRUE	aPSAFFVFMEEFR	95%	n+304 (+304)	43.55	25.00
1986	seq=translation; coord=5:63800401..63802605:-1; parent_transcript=GRMZM5G834758_T02; parent_gene=GRMZM5G834758	GRMZM5G834758_P02,GRMZM5G834758_P03	TRUE	TRUE	rAPSAFFVFMEEFR	95%	n+304 (+304)	60.28	25.00
1987	seq=translation; coord=5:63800401..63802605:-1; parent_transcript=GRMZM5G834758_T02; parent_gene=GRMZM5G834758	GRMZM5G834758_P02,GRMZM5G834758_P03	TRUE	TRUE	rAPSAFFVFmEEFR	95%	n+304 (+304), Oxidation (+16)	41.61	25.00
1988	seq=translation; coord=5:209262549..209264524:-1; parent_transcript=GRMZM2G015784_T01; parent_gene=GRMZM2G015784	GRMZM2G015784_P01,GRMZM2G015784_P03	TRUE	TRUE	dPGQQPFSFSIGQGSVIk	95%	n+304 (+304), K+304 (+304)	45.08	25.50
1989	seq=translation; coord=5:209262549..209264524:-1; parent_transcript=GRMZM2G015784_T01; parent_gene=GRMZM2G015784	GRMZM2G015784_P01,GRMZM2G015784_P03	TRUE	TRUE	gWDEGVMTMQVGEVAR	95%	n+304 (+304)	62.09	25.00
1990	seq=translation; coord=5:209262549..209264524:-1; parent_transcript=GRMZM2G015784_T01; parent_gene=GRMZM2G015784	GRMZM2G015784_P01,GRMZM2G015784_P03	TRUE	TRUE	gWDEGVmTMQVGEVAR	95%	n+304 (+304), Oxidation (+16)	69.14	25.00
1991	seq=translation; coord=5:209262549..209264524:-1; parent_transcript=GRMZM2G015784_T01; parent_gene=GRMZM2G015784	GRMZM2G015784_P01,GRMZM2G015784_P03	TRUE	TRUE	gWDEGVMTmQVGEVAR	95%	n+304 (+304), Oxidation (+16)	64.93	25.00
1992	seq=translation; coord=5:209262549..209264524:-1; parent_transcript=GRMZM2G015784_T01; parent_gene=GRMZM2G015784	GRMZM2G015784_P01,GRMZM2G015784_P03	TRUE	TRUE	gWDEGVmTmQVGEVAR	93%	n+304 (+304), Oxidation (+16), Oxidation (+16)	29.92	25.00
1993	seq=translation; coord=3:210506972..210508715:1; parent_transcript=GRMZM2G022931_T01; parent_gene=GRMZM2G022931	GRMZM2G022931_P01	TRUE	TRUE	gGQFTMFMR	95%	n+304 (+304)	32.48	25.00
1994	seq=translation; coord=3:210506972..210508715:1; parent_transcript=GRMZM2G022931_T01; parent_gene=GRMZM2G022931	GRMZM2G022931_P01	TRUE	TRUE	gGQFTmFMR	90%	n+304 (+304), Oxidation (+16)	27.18	25.00
1995	seq=translation; coord=3:210506972..210508715:1; parent_transcript=GRMZM2G022931_T01; parent_gene=GRMZM2G022931	GRMZM2G022931_P01	TRUE	TRUE	vLLPLELFGAk	95%	n+304 (+304), K+304 (+304)	42.18	25.00
1996	seq=translation; coord=7:4234486..4239316:1; parent_transcript=GRMZM2G064799_T01; parent_gene=GRMZM2G064799	GRMZM2G064799_P01,GRMZM2G064799_P02, GRMZM2G079888_P01	TRUE	TRUE	aFGGQSLDFGk	95%	n+304 (+304), K+304 (+304)	50.96	26.55
1997	seq=translation; coord=7:4234486..4239316:1; parent_transcript=GRMZM2G064799_T01; parent_gene=GRMZM2G064799	GRMZM2G064799_P01,GRMZM2G064799_P02, GRMZM2G079888_P01	TRUE	TRUE	aSNTILATGGYGR	86%	n+304 (+304)	25.57	25.17
1998	seq=translation; coord=7:4234486..4239316:1; parent_transcript=GRMZM2G064799_T01; parent_gene=GRMZM2G064799	GRMZM2G064799_P01,GRMZM2G064799_P02, GRMZM2G079888_P01	TRUE	TRUE	aVIELENYGLPFSR	95%	n+304 (+304)	57.91	25.00

1999	seq=translation; coord=7:4234486..4239316:1; parent_transcript=GRMZM2G064799_T01; parent_gene=GRMZM2G064799	GRMZM2G064799_P01,GRMZM2G064799_P02, GRMZM2G079888_P01	TRUE	TRUE	lGANSLLDIVVFGR	95%	n+304 (+304)	66.38	25.00
2000	seq=translation; coord=7:4234486..4239316:1; parent_transcript=GRMZM2G064799_T01; parent_gene=GRMZM2G064799	GRMZM2G064799_P01,GRMZM2G064799_P02, GRMZM2G079888_P01	TRUE	TRUE	lPGISETAAIFAGVDVTk	94%	n+304 (+304), K+304 (+304)	27.99	25.00
2001	seq=translation; coord=7:4234486..4239316:1; parent_transcript=GRMZM2G064799_T01; parent_gene=GRMZM2G064799	GRMZM2G064799_P01,GRMZM2G064799_P02, GRMZM2G079888_P01	TRUE	TRUE	sMTMEIR	88%	n+304 (+304)	26.32	25.00
2002	seq=translation; coord=7:4234486..4239316:1; parent_transcript=GRMZM2G064799_T01; parent_gene=GRMZM2G064799	GRMZM2G064799_P01,GRMZM2G064799_P02, GRMZM2G079888_P01	TRUE	TRUE	vMQNNAAVFR	93%	n+304 (+304)	29.71	25.00
2003	seq=translation; coord=4:153394405..153398468:1; parent_transcript=GRMZM2G135893_T01; parent_gene=GRMZM2G135893	GRMZM2G135893_P01	TRUE	TRUE	gSLFGDNIk	92%	n+304 (+304), K+304 (+304)	29.66	26.18
2004	seq=translation; coord=3:112198031..112206945:-1; parent_transcript=GRMZM2G086882_T01; parent_gene=GRMZM2G086882	GRMZM2G086882_P01	TRUE	TRUE	gELVSDDLVVGIIDEAMk	95%	n+304 (+304), K+304 (+304)	52.85	25.29
2005	seq=translation; coord=3:112198031..112206945:-1; parent_transcript=GRMZM2G086882_T01; parent_gene=GRMZM2G086882	GRMZM2G086882_P01	TRUE	TRUE	lEAFHR	95%	n+304 (+304)	32.25	25.09
2006	seq=translation; coord=3:112198031..112206945:-1; parent_transcript=GRMZM2G086882_T01; parent_gene=GRMZM2G086882	GRMZM2G086882_P01	TRUE	TRUE	lILIGPPGSGk	95%	n+304 (+304), K+304 (+304)	42.73	25.00
2007	seq=translation; coord=3:112198031..112206945:-1; parent_transcript=GRMZM2G086882_T01; parent_gene=GRMZM2G086882	GRMZM2G086882_P01	TRUE	TRUE	vLNFAIDDAVLEER	95%	n+304 (+304)	91.69	25.89
2008	seq=translation; coord=7:54363440..54365032:-1; parent_transcript=GRMZM2G032766_T01; parent_gene=GRMZM2G032766	GRMZM2G032766_P01	TRUE	TRUE	dVLDDAGIGGk	95%	n+304 (+304), K+304 (+304)	56.55	26.19
2009	seq=translation; coord=7:54363440..54365032:-1; parent_transcript=GRMZM2G032766_T01; parent_gene=GRMZM2G032766	GRMZM2G032766_P01	TRUE	TRUE	vDLDNGENPTWDEk	90%	n+304 (+304), K+304 (+304)	25.54	25.00
2010	seq=translation; coord=7:54363440..54365032:-1; parent_transcript=GRMZM2G032766_T01; parent_gene=GRMZM2G032766	GRMZM2G032766_P01	TRUE	TRUE	vEENLER	91%	n+304 (+304)	27.96	25.00
2011	seq=translation; coord=7:54363440..54365032:-1; parent_transcript=GRMZM2G032766_T01; parent_gene=GRMZM2G032766	GRMZM2G032766_P01	TRUE	TRUE	yYDPNPYPAPAGYANAGTR	95%	n+304 (+304)	30.77	25.00
2012	seq=translation; coord=5:4664985..4668821:1; parent_transcript=GRMZM2G027825_T01; parent_gene=GRMZM2G027825	GRMZM2G027825_P01	TRUE	TRUE	eADGSVk	91%	n+304 (+304), K+304 (+304)	26.75	25.77
2013	seq=translation; coord=5:4664985..4668821:1; parent_transcript=GRMZM2G027825_T01; parent_gene=GRMZM2G027825	GRMZM2G027825_P01	TRUE	TRUE	fGQGEGSAYLFDPDIYTDGR	94%	n+304 (+304)	30.23	25.00
2014	seq=translation; coord=5:4664985..4668821:1; parent_transcript=GRMZM2G027825_T01; parent_gene=GRMZM2G027825	GRMZM2G027825_P01	TRUE	TRUE	iSVQGIDAVLFAPDDAk	95%	n+304 (+304), K+304 (+304)	64.45	25.28
2015	seq=translation; coord=5:4664985..4668821:1; parent_transcript=GRMZM2G027825_T01; parent_gene=GRMZM2G027825	GRMZM2G027825_P01	TRUE	TRUE	lVSEGYVLTVLAPNDEAMAR	93%	n+304 (+304)	27.31	25.00
2016	seq=translation; coord=5:4664985..4668821:1; parent_transcript=GRMZM2G027825_T01; parent_gene=GRMZM2G027825	GRMZM2G027825_P01	TRUE	TRUE	lVSEGyVLTVLAPNDEAMAR	95%	n+304 (+304), iTRAQ8plex (+304)	45.21	25.24

2017	seq=translation; coord=5:4664985..4668821:1; parent_transcript=GRMZM2G027825_T01; parent_gene=GRMZM2G027825	GRMZM2G027825_P01	TRUE	TRUE	sLQALLLYHVLPSR	95%	n+304 (+304)	37.28	25.00
2018	seq=translation; coord=6:165013896..165020496:1; parent_transcript=GRMZM2G065757_T01; parent_gene=GRMZM2G065757	GRMZM2G065757_P01,GRMZM2G065757_P02	TRUE	TRUE	dQEFIEATk	95%	n+304 (+304), K+304 (+304)	52.11	26.12
2019	seq=translation; coord=6:165013896..165020496:1; parent_transcript=GRMZM2G065757_T01; parent_gene=GRMZM2G065757	GRMZM2G065757_P01,GRMZM2G065757_P02	TRUE	TRUE	ePGLTFMVAk	94%	n+304 (+304), K+304 (+304)	29.79	26.11
2020	seq=translation; coord=6:165013896..165020496:1; parent_transcript=GRMZM2G065757_T01; parent_gene=GRMZM2G065757	GRMZM2G065757_P01,GRMZM2G065757_P02	TRUE	TRUE	fTVIFDTGSSNLWVPSSk	95%	n+304 (+304), K+304 (+304)	32.61	25.33
2021	seq=translation; coord=6:165013896..165020496:1; parent_transcript=GRMZM2G065757_T01; parent_gene=GRMZM2G065757	GRMZM2G065757_P01,GRMZM2G065757_P02	TRUE	TRUE	gyWQFNMGDVLVDGk	93%	n+304 (+304), iTRAQ8plex (+304), K+304 (+304)	25.64	25.49
2022	seq=translation; coord=6:165013896..165020496:1; parent_transcript=GRMZM2G065757_T01; parent_gene=GRMZM2G065757	GRMZM2G065757_P01,GRMZM2G065757_P02	TRUE	TRUE	hADEGEGGEIVFGGMDSSHYk	95%	n+304 (+304), K+304 (+304)	49.16	25.00
2023	seq=translation; coord=6:165013896..165020496:1; parent_transcript=GRMZM2G065757_T01; parent_gene=GRMZM2G065757	GRMZM2G065757_P01,GRMZM2G065757_P02	TRUE	TRUE	iGAAGVVSQEck	95%	n+304 (+304), Carbamidomethyl (+57), K+304 (+304)	51.12	25.56
2024	seq=translation; coord=6:165013896..165020496:1; parent_transcript=GRMZM2G065757_T01; parent_gene=GRMZM2G065757	GRMZM2G065757_P01,GRMZM2G065757_P02	TRUE	TRUE	lPSPMGESAVDcGSLASMPDIAFTIGGk	95%	n+304 (+304), Carbamidomethyl (+57), K+304 (+304)	38.45	25.00
2025	seq=translation; coord=6:165013896..165020496:1; parent_transcript=GRMZM2G065757_T01; parent_gene=GRMZM2G065757	GRMZM2G065757_P01,GRMZM2G065757_P02	TRUE	TRUE	nyMNAQYFGEIGVGSPQQk	95%	n+304 (+304), iTRAQ8plex (+304), K+304 (+304)	33.93	25.54
2026	seq=translation; coord=6:165013896..165020496:1; parent_transcript=GRMZM2G065757_T01; parent_gene=GRMZM2G065757	GRMZM2G065757_P01,GRMZM2G065757_P02	TRUE	TRUE	qGLISDPVFSFWFNR	93%	n+304 (+304)	29.29	25.00
2027	seq=translation; coord=6:165013896..165020496:1; parent_transcript=GRMZM2G065757_T01; parent_gene=GRMZM2G065757	GRMZM2G065757_P01,GRMZM2G065757_P02	TRUE	TRUE	tQELILNYINQLcER	95%	n+304 (+304), Carbamidomethyl (+57)	47.85	25.47
2028	seq=translation; coord=6:165013896..165020496:1; parent_transcript=GRMZM2G065757_T01; parent_gene=GRMZM2G065757	GRMZM2G065757_P01,GRMZM2G065757_P02	TRUE	TRUE	tVVSQYGQQILDLLLAETQPAk	95%	n+304 (+304), K+304 (+304)	43.06	25.00
2029	seq=translation; coord=10:18124892..18142878:-1; parent_transcript=GRMZM2G082271_T01; parent_gene=GRMZM2G082271	GRMZM2G082271_P01	TRUE	TRUE	aFALLSEEGIAk	95%	n+304 (+304), K+304 (+304)	55.03	25.72
2030	seq=translation; coord=10:18124892..18142878:-1; parent_transcript=GRMZM2G082271_T01; parent_gene=GRMZM2G082271	GRMZM2G082271_P01	TRUE	TRUE	aSQLEGALLEk	91%	n+304 (+304), K+304 (+304)	25.88	25.77
2031	seq=translation; coord=10:18124892..18142878:-1; parent_transcript=GRMZM2G082271_T01; parent_gene=GRMZM2G082271	GRMZM2G082271_P01	TRUE	TRUE	aVFGEIYPDPVR	95%	n+304 (+304)	30.18	25.00
2032	seq=translation; coord=10:18124892..18142878:-1; parent_transcript=GRMZM2G082271_T01; parent_gene=GRMZM2G082271	GRMZM2G082271_P01	TRUE	TRUE	eAMALANSIASMk	92%	n+304 (+304), K+304 (+304)	25.91	25.24
2033	seq=translation; coord=10:18124892..18142878:-1; parent_transcript=GRMZM2G082271_T01; parent_gene=GRMZM2G082271	GRMZM2G082271_P01	TRUE	TRUE	gFNASMEEAR	95%	n+304 (+304)	41.75	25.00
2034	seq=translation; coord=10:18124892..18142878:-1; parent_transcript=GRMZM2G082271_T01; parent_gene=GRMZM2G082271	GRMZM2G082271_P01	TRUE	TRUE	gLPIMLFSTDEASNk	95%	n+304 (+304), K+304 (+304)	48.02	26.07

2035	seq=translation; coord=10:18124892..18142878:-1; parent_transcript=GRMZM2G082271_T01; parent_gene=GRMZM2G082271	GRMZM2G082271_P01	TRUE	TRUE	gSIVLPEk	88%	n+304 (+304), K+304 (+304)	26.72	25.34
2036	seq=translation; coord=10:18124892..18142878:-1; parent_transcript=GRMZM2G082271_T01; parent_gene=GRMZM2G082271	GRMZM2G082271_P01	TRUE	TRUE	lEDELR	89%	n+304 (+304)	25.83	25.00
2037	seq=translation; coord=10:18124892..18142878:-1; parent_transcript=GRMZM2G082271_T01; parent_gene=GRMZM2G082271	GRMZM2G082271_P01	TRUE	TRUE	sGLDAAAIPAAR	95%	n+304 (+304)	33.02	25.00
2038	seq=translation; coord=10:18124892..18142878:-1; parent_transcript=GRMZM2G082271_T01; parent_gene=GRMZM2G082271	GRMZM2G082271_P01	TRUE	TRUE	tLIAPNHTcTHMLNFALR	95%	n+304 (+304), Carbamidomethyl (+57)	38.51	25.22
2039	seq=translation; coord=10:18124892..18142878:-1; parent_transcript=GRMZM2G082271_T01; parent_gene=GRMZM2G082271	GRMZM2G082271_P01	TRUE	TRUE	vLDWLTPSIAPLk	95%	n+304 (+304), K+304 (+304)	32.10	25.00
2040	seq=translation; coord=10:18124892..18142878:-1; parent_transcript=GRMZM2G082271_T01; parent_gene=GRMZM2G082271	GRMZM2G082271_P01	TRUE	TRUE	wAWELLTQVYk	95%	n+304 (+304), K+304 (+304)	30.38	26.06
2041	seq=translation; coord=10:18124892..18142878:-1; parent_transcript=GRMZM2G082271_T01; parent_gene=GRMZM2G082271	GRMZM2G082271_P01	TRUE	TRUE	wPSSPVVPVDDPTLLFANAGMNQFkPVFLGTAAPDSPLGR	95%	n+304 (+304), K+304 (+304)	26.97	25.00
2042	seq=translation; coord=4:3210119..3212030:-1; parent_transcript=GRMZM2G085054_T02; parent_gene=GRMZM2G085054	GRMZM2G085054_P02	TRUE	TRUE	aPDPADYPADYR	95%	n+304 (+304)	34.06	25.00
2043	seq=translation; coord=4:3210119..3212030:-1; parent_transcript=GRMZM2G085054_T02; parent_gene=GRMZM2G085054	GRMZM2G085054_P02	TRUE	TRUE	dDMSVPVYAVAPLNk	95%	n+304 (+304), K+304 (+304)	65.12	25.22
2044	seq=translation; coord=4:3210119..3212030:-1; parent_transcript=GRMZM2G085054_T02; parent_gene=GRMZM2G085054	GRMZM2G085054_P02	TRUE	TRUE	gFESGALPDGVEDR	95%	n+304 (+304)	52.36	25.00
2045	seq=translation; coord=4:3210119..3212030:-1; parent_transcript=GRMZM2G085054_T02; parent_gene=GRMZM2G085054	GRMZM2G085054_P02	TRUE	TRUE	gIDESAGSDLTNLVHLINSy	95%	n+304 (+304), iTRAQ8plex (+304)	65.57	25.00
2046	seq=translation; coord=4:3210119..3212030:-1; parent_transcript=GRMZM2G085054_T02; parent_gene=GRMZM2G085054	GRMZM2G085054_P02	TRUE	TRUE	gVGITVFHTAGAR	95%	n+304 (+304)	53.61	26.95
2047	seq=translation; coord=4:3210119..3212030:-1; parent_transcript=GRMZM2G085054_T02; parent_gene=GRMZM2G085054	GRMZM2G085054_P02	TRUE	TRUE	kDDAVAELPPYR	95%	K+304 (+304), n+304 (+304)	31.25	25.09
2048	seq=translation; coord=4:3210119..3212030:-1; parent_transcript=GRMZM2G085054_T02; parent_gene=GRMZM2G085054	GRMZM2G085054_P02	TRUE	TRUE	lMGGSEEGEGIR	95%	n+304 (+304)	40.02	25.00
2049	seq=translation; coord=4:3210119..3212030:-1; parent_transcript=GRMZM2G085054_T02; parent_gene=GRMZM2G085054	GRMZM2G085054_P02	TRUE	TRUE	lSALLSAADGEAGEAGGR	95%	n+304 (+304)	62.65	25.00
2050	seq=translation; coord=4:3210119..3212030:-1; parent_transcript=GRMZM2G085054_T02; parent_gene=GRMZM2G085054	GRMZM2G085054_P02	TRUE	TRUE	vGTEVAGDQLER	95%	n+304 (+304)	52.36	25.00
2051	seq=translation; coord=3:8299415..8303114:-1; parent_transcript=GRMZM2G314898_T01; parent_gene=GRMZM2G314898	GRMZM2G314898_P01	TRUE	TRUE	aLAFFYPLAGR	95%	n+304 (+304)	39.83	25.63
2052	seq=translation; coord=3:8299415..8303114:-1; parent_transcript=GRMZM2G314898_T01; parent_gene=GRMZM2G314898	GRMZM2G314898_P01	TRUE	TRUE	aLALDPAAEVk	95%	n+304 (+304), K+304 (+304)	43.56	25.00

2053	seq=translation; coord=3:8299415..8303114:-1; parent_transcript=GRMZM2G314898_T01; parent_gene=GRMZM2G314898	GRMZM2G314898_P01	TRUE	TRUE	gLVESMHIFR	95%	n+304 (+304)	51.39	26.24
2054	seq=translation; coord=3:8299415..8303114:-1; parent_transcript=GRMZM2G314898_T01; parent_gene=GRMZM2G314898	GRMZM2G314898_P01	TRUE	TRUE	iSEGPVkPASATPEETLPLAWVDR	95%	n+304 (+304), K+304 (+304)	38.82	25.35
2055	seq=translation; coord=3:8299415..8303114:-1; parent_transcript=GRMZM2G314898_T01; parent_gene=GRMZM2G314898	GRMZM2G314898_P01	TRUE	TRUE	lcFFASVR	85%	n+304 (+304), Carbamidomethyl (+57)	25.05	25.00
2056	seq=translation; coord=3:8299415..8303114:-1; parent_transcript=GRMZM2G314898_T01; parent_gene=GRMZM2G314898	GRMZM2G314898_P01	TRUE	TRUE	sGAGEAPAVIR	92%	n+304 (+304)	29.03	25.00
2057	seq=translation; coord=3:8299415..8303114:-1; parent_transcript=GRMZM2G314898_T01; parent_gene=GRMZM2G314898	GRMZM2G314898_P01	TRUE	TRUE	vLASSLVEVVDIIR	95%	n+304 (+304)	43.07	25.00
2058	seq=translation; coord=3:8299415..8303114:-1; parent_transcript=GRMZM2G314898_T01; parent_gene=GRMZM2G314898	GRMZM2G314898_P01	TRUE	TRUE	vLPVWHR	95%	n+304 (+304)	28.40	25.00
2059	seq=translation; coord=6:140347883..140348297:-1; parent_transcript=AC233879.1_FGT002; parent_gene=AC233879.1_FG002	AC233879.1_FGP002	TRUE	TRUE	eGETVVPGGTGGk	95%	n+304 (+304), K+304 (+304)	38.23	25.09
2060	seq=translation; coord=6:140347883..140348297:-1; parent_transcript=AC233879.1_FGT002; parent_gene=AC233879.1_FG002	AC233879.1_FGP002	TRUE	TRUE	eGVSIDESk	92%	n+304 (+304), K+304 (+304)	28.76	25.00
2061	seq=translation; coord=6:140347883..140348297:-1; parent_transcript=AC233879.1_FGT002; parent_gene=AC233879.1_FG002	AC233879.1_FGP002	TRUE	TRUE	eQLGQQGYSEMGk	95%	n+304 (+304), K+304 (+304)	63.67	25.00
2062	seq=translation; coord=6:140347883..140348297:-1; parent_transcript=AC233879.1_FGT002; parent_gene=AC233879.1_FG002	AC233879.1_FGP002	TRUE	TRUE	eQLGQQGYSEmGk	95%	n+304 (+304), Oxidation (+16), K+304 (+304)	31.49	25.00
2063	seq=translation; coord=6:140347883..140348297:-1; parent_transcript=AC233879.1_FGT002; parent_gene=AC233879.1_FG002	AC233879.1_FGP002	TRUE	TRUE	gGLSTTDESGGER	95%	n+304 (+304)	51.98	25.00
2064	seq=translation; coord=6:140347883..140348297:-1; parent_transcript=AC233879.1_FGT002; parent_gene=AC233879.1_FG002	AC233879.1_FGP002	TRUE	TRUE	rEQLGQQGYSEMGk	94%	n+304 (+304), K+304 (+304)	31.79	25.60
2065	seq=translation; coord=6:140347883..140348297:-1; parent_transcript=AC233879.1_FGT002; parent_gene=AC233879.1_FG002	AC233879.1_FGP002	TRUE	TRUE	sVEAQEHLAEGR	95%	n+304 (+304)	38.40	25.00
2066	seq=translation; coord=10:4673451..4676086:-1; parent_transcript=GRMZM2G003762_T03; parent_gene=GRMZM2G003762	GRMZM2G003762_P03	TRUE	TRUE	dEVFcLFEGVLDNLGR	95%	n+304 (+304), Carbamidomethyl (+57)	65.47	25.00
2067	seq=translation; coord=10:4673451..4676086:-1; parent_transcript=GRMZM2G003762_T03; parent_gene=GRMZM2G003762	GRMZM2G003762_P03	TRUE	TRUE	gANEVLLVIEAYk	95%	n+304 (+304), K+304 (+304)	42.96	25.00
2068	seq=translation; coord=10:4673451..4676086:-1; parent_transcript=GRMZM2G003762_T03; parent_gene=GRMZM2G003762	GRMZM2G003762_P03	TRUE	TRUE	sLAPFPQGcFYSNALGGLk	95%	n+304 (+304), Carbamidomethyl (+57), K+304 (+304)	34.27	25.88
2069	seq=translation; coord=10:4673451..4676086:-1; parent_transcript=GRMZM2G003762_T03; parent_gene=GRMZM2G003762	GRMZM2G003762_P03	TRUE	TRUE	sTNSLLVASDPEGR	95%	n+304 (+304)	45.20	25.00
2070	seq=translation; coord=10:4673451..4676086:-1; parent_transcript=GRMZM2G003762_T03; parent_gene=GRMZM2G003762	GRMZM2G003762_P03	TRUE	TRUE	vTAVPADEEEIcGATFk	95%	n+304 (+304), Carbamidomethyl (+57), K+304 (+304)	39.95	25.00

2071	seq=translation; coord=10:4673451..4676086:-1; parent_transcript=GRMZM2G003762_T03; parent_gene=GRMZM2G003762	GRMZM2G003762_P03	TRUE	TRUE	vVEGSTVLTALH	95%	n+304 (+304)	61.03	26.57
2072	seq=translation; coord=6:145987055..145988456:-1; parent_transcript=GRMZM2G306345_T01; parent_gene=GRMZM2G306345	GRMZM2G306345_P01	TRUE	TRUE	aGLDYVScSPFR	95%	n+304 (+304), Carbamidomethyl (+57)	56.26	25.00
2073	seq=translation; coord=6:145987055..145988456:-1; parent_transcript=GRMZM2G306345_T01; parent_gene=GRMZM2G306345	GRMZM2G306345_P01	TRUE	TRUE	fIPVYLAQGILQHDPFEVLDQR	95%	n+304 (+304)	54.50	25.19
2074	seq=translation; coord=6:145987055..145988456:-1; parent_transcript=GRMZM2G306345_T01; parent_gene=GRMZM2G306345	GRMZM2G306345_P01	TRUE	TRUE	vGTMIEIPR	94%	n+304 (+304)	31.11	25.69
2075	seq=translation; coord=7:160215143..160216461:-1; parent_transcript=GRMZM2G374971_T01; parent_gene=GRMZM2G374971	GRMZM2G374971_P01	TRUE	TRUE	aPNTLAEYALk	95%	n+304 (+304), K+304 (+304)	43.86	26.26
2076	seq=translation; coord=7:160215143..160216461:-1; parent_transcript=GRMZM2G374971_T01; parent_gene=GRMZM2G374971	GRMZM2G374971_P01	TRUE	TRUE	dDATSTFTcPAGTNYk	95%	n+304 (+304), Carbamidomethyl (+57), K+304 (+304)	49.86	25.00
2077	seq=translation; coord=7:160215143..160216461:-1; parent_transcript=GRMZM2G374971_T01; parent_gene=GRMZM2G374971	GRMZM2G374971_P01	TRUE	TRUE	gQcPDAYSYPk	95%	n+304 (+304), Carbamidomethyl (+57), K+304 (+304)	37.53	25.00
2078	seq=translation; coord=7:160215143..160216461:-1; parent_transcript=GRMZM2G374971_T01; parent_gene=GRMZM2G374971	GRMZM2G374971_P01	TRUE	TRUE	iTAPAGTTAAR	91%	n+304 (+304)	29.12	26.24
2079	seq=translation; coord=7:160215143..160216461:-1; parent_transcript=GRMZM2G374971_T01; parent_gene=GRMZM2G374971	GRMZM2G374971_P01	TRUE	TRUE	tGDcGGVLQcTGYGR	95%	n+304 (+304), Carbamidomethyl (+57), Carbamidomethyl (+57)	62.59	25.00
2080	seq=translation; coord=7:160215143..160216461:-1; parent_transcript=GRMZM2G374971_T01; parent_gene=GRMZM2G374971	GRMZM2G374971_P01	TRUE	TRUE	tGDcGGVLQcTGyGR	90%	n+304 (+304), Carbamidomethyl (+57), Carbamidomethyl (+57), iTRAQ8plex (+304)	25.50	25.00
2081	seq=translation; coord=7:160215143..160216461:-1; parent_transcript=GRMZM2G374971_T01; parent_gene=GRMZM2G374971	GRMZM2G374971_P01	TRUE	TRUE	vVFcP	95%	n+304 (+304), Carbamidomethyl (+57)	33.17	25.81
2082	seq=translation; coord=2:217342775..217345487:1; parent_transcript=GRMZM2G003409_T01; parent_gene=GRMZM2G003409	GRMZM2G003409_P01,GRMZM2G003409_P04	TRUE	TRUE	aDEAYcLTVR	94%	n+304 (+304), Carbamidomethyl (+57)	30.98	25.00
2083	seq=translation; coord=2:217342775..217345487:1; parent_transcript=GRMZM2G003409_T01; parent_gene=GRMZM2G003409	GRMZM2G003409_P01,GRMZM2G003409_P04	TRUE	TRUE	dAEGQPAFALINR	95%	n+304 (+304)	38.63	25.00
2084	seq=translation; coord=2:217342775..217345487:1; parent_transcript=GRMZM2G003409_T01; parent_gene=GRMZM2G003409	GRMZM2G003409_P01,GRMZM2G003409_P04	TRUE	TRUE	dEFQHWVk	94%	n+304 (+304), K+304 (+304)	28.04	25.42
2085	seq=translation; coord=2:217342775..217345487:1; parent_transcript=GRMZM2G003409_T01; parent_gene=GRMZM2G003409	GRMZM2G003409_P01,GRMZM2G003409_P04	TRUE	TRUE	lVPYNAEYLDESVLWTESHDVGk	95%	n+304 (+304), K+304 (+304)	36.39	25.00
2086	seq=translation; coord=2:217342775..217345487:1; parent_transcript=GRMZM2G003409_T01; parent_gene=GRMZM2G003409	GRMZM2G003409_P01,GRMZM2G003409_P04	TRUE	TRUE	lVPYNPEYVDESVLWTESR	95%	n+304 (+304)	55.25	25.00
2087	seq=translation; coord=2:217342775..217345487:1; parent_transcript=GRMZM2G003409_T01; parent_gene=GRMZM2G003409	GRMZM2G003409_P01,GRMZM2G003409_P04	TRUE	TRUE	lVPYNPEyVDESVLWTESR	89%	n+304 (+304), iTRAQ8plex (+304)	25.25	25.04

2088	seq=translation; coord=9:139405932..139410290:-1; parent_transcript=GRMZM2G165357_T01; parent_gene=GRMZM2G165357	GRMZM2G165357_P01,GRMZM2G165357_P02	TRUE	TRUE	iLVTGGAGFIGSHLVDk	95%	n+304 (+304), K+304 (+304)	40.27	25.00
2089	seq=translation; coord=9:139405932..139410290:-1; parent_transcript=GRMZM2G165357_T01; parent_gene=GRMZM2G165357	GRMZM2G165357_P01,GRMZM2G165357_P02	TRUE	TRUE	sFcYVADMVDGLIk	95%	n+304 (+304), Carbamidomethyl (+57), K+304 (+304)	42.19	25.00
2090	seq=translation; coord=9:139405932..139410290:-1; parent_transcript=GRMZM2G165357_T01; parent_gene=GRMZM2G165357	GRMZM2G165357_P01,GRMZM2G165357_P02	TRUE	TRUE	tGPINLGNPGEFTMLELAENVk	95%	n+304 (+304), K+304 (+304)	41.03	26.12
2091	seq=translation; coord=10:148968847..148972562:1; parent_transcript=GRMZM2G104613_T01; parent_gene=GRMZM2G104613	GRMZM2G104613_P01	TRUE	TRUE	dVLSFAGALEGVELR	95%	n+304 (+304)	69.58	25.40
2092	seq=translation; coord=10:148968847..148972562:1; parent_transcript=GRMZM2G104613_T01; parent_gene=GRMZM2G104613	GRMZM2G104613_P01	TRUE	TRUE	rIEAAVTETLNHGFR	95%	n+304 (+304)	37.07	26.47
2093	seq=translation; coord=4:128262885..128265210:1; parent_transcript=GRMZM2G008748_T02; parent_gene=GRMZM2G008748	GRMZM2G008748_P02	TRUE	TRUE	aAAAVEPk	94%	n+304 (+304), K+304 (+304)	31.08	25.00
2094	seq=translation; coord=4:128262885..128265210:1; parent_transcript=GRMZM2G008748_T02; parent_gene=GRMZM2G008748	GRMZM2G008748_P02	TRUE	TRUE	aIEAVPELk	94%	n+304 (+304), K+304 (+304)	30.88	25.00
2095	seq=translation; coord=4:128262885..128265210:1; parent_transcript=GRMZM2G008748_T02; parent_gene=GRMZM2G008748	GRMZM2G008748_P02	TRUE	TRUE	aVDSALIk	95%	n+304 (+304), K+304 (+304)	42.73	25.43
2096	seq=translation; coord=4:128262885..128265210:1; parent_transcript=GRMZM2G008748_T02; parent_gene=GRMZM2G008748	GRMZM2G008748_P02	TRUE	TRUE	sGLLLISGPFk	95%	n+304 (+304), K+304 (+304)	33.16	25.00
2097	seq=translation; coord=1:298408224..298413579:-1; parent_transcript=GRMZM2G022269_T01; parent_gene=GRMZM2G022269	GRMZM2G022269_P01,GRMZM2G022269_P03	TRUE	TRUE	qLDkPFLMPIEDVFSIQGR	95%	n+304 (+304), K+304 (+304)	42.60	25.16
2098	seq=translation; coord=1:298408224..298413579:-1; parent_transcript=GRMZM2G022269_T01; parent_gene=GRMZM2G022269	GRMZM2G022269_P01,GRMZM2G022269_P03	TRUE	TRUE	qLDkPFLMPIEDVFSIQGR	95%	Pyro-cmC (-17), n+304 (+304), K+304 (+304)	31.23	25.77
2099	seq=translation; coord=1:298408224..298413579:-1; parent_transcript=GRMZM2G022269_T01; parent_gene=GRMZM2G022269	GRMZM2G022269_P01,GRMZM2G022269_P03	TRUE	TRUE	tGEDVEILGLAQTGPLk	94%	n+304 (+304), K+304 (+304)	27.29	25.00
2100	seq=translation; coord=1:298408224..298413579:-1; parent_transcript=GRMZM2G022269_T01; parent_gene=GRMZM2G022269	GRMZM2G022269_P01,GRMZM2G022269_P03	TRUE	TRUE	vELLGEMk	95%	n+304 (+304), K+304 (+304)	34.60	25.31
2101	seq=translation; coord=6:140407545..140410127:1; parent_transcript=AC233895.1_FGT001; parent_gene=AC233895.1_FG001	AC233895.1_FGP001	TRUE	TRUE	aGDVLAAANTAAEVAMR	95%	n+304 (+304)	70.67	25.91
2102	seq=translation; coord=6:140407545..140410127:1; parent_transcript=AC233895.1_FGT001; parent_gene=AC233895.1_FG001	AC233895.1_FGP001	TRUE	TRUE	aWLALGTk	95%	n+304 (+304), K+304 (+304)	32.88	25.37
2103	seq=translation; coord=6:140407545..140410127:1; parent_transcript=AC233895.1_FGT001; parent_gene=AC233895.1_FG001	AC233895.1_FGP001	TRUE	TRUE	eLDLSSNDVVTk	95%	n+304 (+304), K+304 (+304)	54.50	25.63
2104	seq=translation; coord=6:140407545..140410127:1; parent_transcript=AC233895.1_FGT001; parent_gene=AC233895.1_FG001	AC233895.1_FGP001	TRUE	TRUE	eQTGNVYk	94%	n+304 (+304), K+304 (+304)	31.58	27.18
2105	seq=translation; coord=6:140407545..140410127:1; parent_transcript=AC233895.1_FGT001; parent_gene=AC233895.1_FG001	AC233895.1_FGP001	TRUE	TRUE	fIFSEISQk	95%	n+304 (+304), K+304 (+304)	29.44	25.17

2106	seq=translation; coord=6:140407545..140410127:1; parent_transcript=AC233895.1_FGT001; parent_gene=AC233895.1_FG001	AC233895.1_FGP001	TRUE	TRUE	iVDLcEk	86%	n+304 (+304), Carbamidomethyl (+57), K+304 (+304)	26.63	26.40
2107	seq=translation; coord=6:140407545..140410127:1; parent_transcript=AC233895.1_FGT001; parent_gene=AC233895.1_FG001	AC233895.1_FGP001	TRUE	TRUE	iVEGVLSHQLk	95%	n+304 (+304), K+304 (+304)	40.35	25.00
2108	seq=translation; coord=6:140407545..140410127:1; parent_transcript=AC233895.1_FGT001; parent_gene=AC233895.1_FG001	AC233895.1_FGP001	TRUE	TRUE	qFVIDGNk	88%	n+304 (+304), K+304 (+304)	26.95	26.53
2109	seq=translation; coord=6:140407545..140410127:1; parent_transcript=AC233895.1_FGT001; parent_gene=AC233895.1_FG001	AC233895.1_FGP001	TRUE	TRUE	sIEDNAEIk	95%	n+304 (+304), K+304 (+304)	58.00	26.25
2110	seq=translation; coord=6:140407545..140410127:1; parent_transcript=AC233895.1_FGT001; parent_gene=AC233895.1_FG001	AC233895.1_FGP001	TRUE	TRUE	vAAAYDck	95%	n+304 (+304), Carbamidomethyl (+57), K+304 (+304)	34.23	26.04
2111	seq=translation; coord=10:82668664..82669799:-1; parent_transcript=GRMZM2G121137_T01; parent_gene=GRMZM2G121137	GRMZM2G121137_P01	TRUE	TRUE	aVPNLDMEcIIR	95%	n+304 (+304), Carbamidomethyl (+57)	49.62	25.19
2112	seq=translation; coord=10:82668664..82669799:-1; parent_transcript=GRMZM2G121137_T01; parent_gene=GRMZM2G121137	GRMZM2G121137_P01	TRUE	TRUE	aVPNLDmEcIIR	95%	n+304 (+304), Oxidation (+16), Carbamidomethyl (+57)	50.30	25.00
2113	seq=translation; coord=10:82668664..82669799:-1; parent_transcript=GRMZM2G121137_T01; parent_gene=GRMZM2G121137	GRMZM2G121137_P01	TRUE	TRUE	gPVTAPSYTDDccVAIR	95%	n+304 (+304), Carbamidomethyl (+57), Carbamidomethyl (+57)	60.82	25.00
2114	seq=translation; coord=10:82668664..82669799:-1; parent_transcript=GRMZM2G121137_T01; parent_gene=GRMZM2G121137	GRMZM2G121137_P01	TRUE	TRUE	gPVTAPSyTDDccVAIR	95%	n+304 (+304), iTRAQ8plex (+304), Carbamidomethyl (+57), Carbamidomethyl (+57)	33.53	25.00
2115	seq=translation; coord=10:82668664..82669799:-1; parent_transcript=GRMZM2G121137_T01; parent_gene=GRMZM2G121137	GRMZM2G121137_P01	TRUE	TRUE	lkGPVTAPSYTDDccVAIR	95%	n+304 (+304), K+304 (+304), Carbamidomethyl (+57), Carbamidomethyl (+57)	50.67	25.51
2116	seq=translation; coord=1:275835279..275836493:-1; parent_transcript=GRMZM2G025857_T01; parent_gene=GRMZM2G025857	GRMZM2G025857_P01	TRUE	TRUE	aQTAcPVADAGATVTETELAGR	95%	n+304 (+304), Carbamidomethyl (+57)	105.41	25.00
2117	seq=translation; coord=1:275835279..275836493:-1; parent_transcript=GRMZM2G025857_T01; parent_gene=GRMZM2G025857	GRMZM2G025857_P01	TRUE	TRUE	fGDVLDVSGELADQPVAPR	95%	n+304 (+304)	49.39	25.00
2118	seq=translation; coord=1:275835279..275836493:-1; parent_transcript=GRMZM2G025857_T01; parent_gene=GRMZM2G025857	GRMZM2G025857_P01	TRUE	TRUE	gGPAAVLQSAATVNAR	95%	n+304 (+304)	66.67	25.44
2119	seq=translation; coord=1:275835279..275836493:-1; parent_transcript=GRMZM2G025857_T01; parent_gene=GRMZM2G025857	GRMZM2G025857_P01	TRUE	TRUE	lQAAEQSVLGGTQk	95%	n+304 (+304), K+304 (+304)	43.90	26.01
2120	seq=translation; coord=1:275835279..275836493:-1; parent_transcript=GRMZM2G025857_T01; parent_gene=GRMZM2G025857	GRMZM2G025857_P01	TRUE	TRUE	mVSPAPVAMTDPPGALEk	95%	n+304 (+304), K+304 (+304)	40.51	25.53
2121	seq=translation; coord=8:129620163..129623583:1; parent_transcript=GRMZM2G117198_T01; parent_gene=GRMZM2G117198	GRMZM2G117198_P01,GRMZM2G117198_P02	TRUE	TRUE	aNVDYEk	95%	n+304 (+304), K+304 (+304)	30.05	25.47
2122	seq=translation; coord=8:129620163..129623583:1; parent_transcript=GRMZM2G117198_T01; parent_gene=GRMZM2G117198	GRMZM2G117198_P01,GRMZM2G117198_P02	TRUE	TRUE	eHVIkPVIPEQYLDEk	93%	n+304 (+304), K+304 (+304), K+304 (+304)	25.01	25.00

2123	seq=translation; coord=8:129620163..129623583:1; parent_transcript=GRMZM2G117198_T01; parent_gene=GRMZM2G117198	GRMZM2G117198_P01,GRMZM2G117198_P02	TRUE	TRUE	lcDQVSDAVLDAcLAEDPDSk	95%	n+304 (+304), Carbamidomethyl (+57), Carbamidomethyl (+57), K+304 (+304)	34.10	25.00
2124	seq=translation; coord=8:129620163..129623583:1; parent_transcript=GRMZM2G117198_T01; parent_gene=GRMZM2G117198	GRMZM2G117198_P01,GRMZM2G117198_P02	TRUE	TRUE	tQVTVEYR	91%	n+304 (+304)	28.22	25.00
2125	seq=translation; coord=2:215654249..215658893:-1; parent_transcript=GRMZM2G005493_T01; parent_gene=GRMZM2G005493	GRMZM2G005493_P01	TRUE	TRUE	aHFEFMEk	95%	n+304 (+304), K+304 (+304)	30.81	25.59
2126	seq=translation; coord=2:215654249..215658893:-1; parent_transcript=GRMZM2G005493_T01; parent_gene=GRMZM2G005493	GRMZM2G005493_P01	TRUE	TRUE	aLEVTHYLGGENYVFWGGR	95%	n+304 (+304)	30.30	25.00
2127	seq=translation; coord=2:215654249..215658893:-1; parent_transcript=GRMZM2G005493_T01; parent_gene=GRMZM2G005493	GRMZM2G005493_P01	TRUE	TRUE	eLDHLANFLQAAVDYk	95%	n+304 (+304), K+304 (+304)	47.10	25.19
2128	seq=translation; coord=2:215654249..215658893:-1; parent_transcript=GRMZM2G005493_T01; parent_gene=GRMZM2G005493	GRMZM2G005493_P01	TRUE	TRUE	eSTDVEDLFLAHISGMDTLAR	95%	n+304 (+304)	48.03	25.00
2129	seq=translation; coord=2:215654249..215658893:-1; parent_transcript=GRMZM2G005493_T01; parent_gene=GRMZM2G005493	GRMZM2G005493_P01	TRUE	TRUE	fSVAFWHTFR	95%	n+304 (+304)	49.59	25.00
2130	seq=translation; coord=2:215654249..215658893:-1; parent_transcript=GRMZM2G005493_T01; parent_gene=GRMZM2G005493	GRMZM2G005493_P01	TRUE	TRUE	gTGADPFGAPTk	95%	n+304 (+304), K+304 (+304)	36.99	25.71
2131	seq=translation; coord=2:215654249..215658893:-1; parent_transcript=GRMZM2G005493_T01; parent_gene=GRMZM2G005493	GRMZM2G005493_P01	TRUE	TRUE	lIEDGSLDALVR	95%	n+304 (+304)	36.19	26.16
2132	seq=translation; coord=2:215654249..215658893:-1; parent_transcript=GRMZM2G005493_T01; parent_gene=GRMZM2G005493	GRMZM2G005493_P01	TRUE	TRUE	qELAEILFHSAL	95%	n+304 (+304)	39.44	25.63
2133	seq=translation; coord=2:215654249..215658893:-1; parent_transcript=GRMZM2G005493_T01; parent_gene=GRMZM2G005493	GRMZM2G005493_P01	TRUE	TRUE	qLQGETNIkPLWGTAQLFMHPR	95%	Pyro-cmC (-17), n+304 (+304), K+304 (+304)	36.60	25.21
2134	seq=translation; coord=2:215654249..215658893:-1; parent_transcript=GRMZM2G005493_T01; parent_gene=GRMZM2G005493	GRMZM2G005493_P01	TRUE	TRUE	yQSFDSEIGALIEAGk	95%	n+304 (+304), K+304 (+304)	44.90	25.11
2135	seq=translation; coord=5:149458858..149463589:-1; parent_transcript=GRMZM2G135588_T01; parent_gene=GRMZM2G135588	GRMZM2G135588_P01	TRUE	TRUE	aGALAVVDGR	95%	n+304 (+304)	31.39	25.21
2136	seq=translation; coord=5:149458858..149463589:-1; parent_transcript=GRMZM2G135588_T01; parent_gene=GRMZM2G135588	GRMZM2G135588_P01	TRUE	TRUE	aLDILFILHAEHEMNcSTAAVR	95%	n+304 (+304), Carbamidomethyl (+57)	55.40	25.00
2137	seq=translation; coord=5:149458858..149463589:-1; parent_transcript=GRMZM2G135588_T01; parent_gene=GRMZM2G135588	GRMZM2G135588_P01	TRUE	TRUE	aMGFPTEFFPVLFAIPR	95%	n+304 (+304)	50.82	25.39
2138	seq=translation; coord=5:149458858..149463589:-1; parent_transcript=GRMZM2G135588_T01; parent_gene=GRMZM2G135588	GRMZM2G135588_P01	TRUE	TRUE	dPLIEVAIALEk	95%	n+304 (+304), K+304 (+304)	39.69	25.00
2139	seq=translation; coord=5:149458858..149463589:-1; parent_transcript=GRMZM2G135588_T01; parent_gene=GRMZM2G135588	GRMZM2G135588_P01	TRUE	TRUE	kLADEVFSIVGR	89%	K+304 (+304), n+304 (+304)	25.16	25.00

2140	seq=translation; coord=5:149458858..149463589:-1; parent_transcript=GRMZM2G135588_T01; parent_gene=GRMZM2G135588	GRMZM2G135588_P01	TRUE	TRUE	lADEVFSIVGR	95%	n+304 (+304)	46.80	25.00
2141	seq=translation; coord=5:149458858..149463589:-1; parent_transcript=GRMZM2G135588_T01; parent_gene=GRMZM2G135588	GRMZM2G135588_P01	TRUE	TRUE	lAGRPPVLPLNTLSYSENFLYMLDSLGDR	95%	n+304 (+304)	42.49	25.00
2142	seq=translation; coord=5:149458858..149463589:-1; parent_transcript=GRMZM2G135588_T01; parent_gene=GRMZM2G135588	GRMZM2G135588_P01	TRUE	TRUE	mLNEIGSMENIPDFIVGVk	95%	n+304 (+304), K+304 (+304)	32.03	25.61
2143	seq=translation; coord=10:109798193..109804126:1; parent_transcript=GRMZM2G151041_T01; parent_gene=GRMZM2G151041	GRMZM2G151041_P01,GRMZM2G151041_P02	TRUE	TRUE	aAHLGVESIVIGMPHR	95%	n+304 (+304)	34.44	25.02
2144	seq=translation; coord=10:109798193..109804126:1; parent_transcript=GRMZM2G151041_T01; parent_gene=GRMZM2G151041	GRMZM2G151041_P01,GRMZM2G151041_P02	TRUE	TRUE	aWEADPSSVDESWDNFFR	95%	n+304 (+304)	67.65	25.00
2145	seq=translation; coord=10:109798193..109804126:1; parent_transcript=GRMZM2G151041_T01; parent_gene=GRMZM2G151041	GRMZM2G151041_P01,GRMZM2G151041_P02	TRUE	TRUE	dRIETVNPMDYTYDR	93%	n+304 (+304)	27.76	25.00
2146	seq=translation; coord=10:109798193..109804126:1; parent_transcript=GRMZM2G151041_T01; parent_gene=GRMZM2G151041	GRMZM2G151041_P01,GRMZM2G151041_P02	TRUE	TRUE	dWLSAYWTGFk	91%	n+304 (+304), K+304 (+304)	28.64	25.38
2147	seq=translation; coord=10:109798193..109804126:1; parent_transcript=GRMZM2G151041_T01; parent_gene=GRMZM2G151041	GRMZM2G151041_P01,GRMZM2G151041_P02	TRUE	TRUE	fGLEGAETLIPGMk	95%	n+304 (+304), K+304 (+304)	35.13	26.34
2148	seq=translation; coord=10:109798193..109804126:1; parent_transcript=GRMZM2G151041_T01; parent_gene=GRMZM2G151041	GRMZM2G151041_P01,GRMZM2G151041_P02	TRUE	TRUE	lIWSTQFESFLATk	95%	n+304 (+304), K+304 (+304)	34.20	25.60
2149	seq=translation; coord=10:109798193..109804126:1; parent_transcript=GRMZM2G151041_T01; parent_gene=GRMZM2G151041	GRMZM2G151041_P01,GRMZM2G151041_P02	TRUE	TRUE	lNVLGNVVR	95%	n+304 (+304)	29.74	25.00
2150	seq=translation; coord=10:109798193..109804126:1; parent_transcript=GRMZM2G151041_T01; parent_gene=GRMZM2G151041	GRMZM2G151041_P01,GRMZM2G151041_P02	TRUE	TRUE	lTDSFLDGTSSVYLEELQR	95%	n+304 (+304)	61.55	25.00
2151	seq=translation; coord=10:109798193..109804126:1; parent_transcript=GRMZM2G151041_T01; parent_gene=GRMZM2G151041	GRMZM2G151041_P01,GRMZM2G151041_P02	TRUE	TRUE	sNLSEFDDLAGHPGFDk	93%	n+304 (+304), K+304 (+304)	25.05	25.00
2152	seq=translation; coord=10:109798193..109804126:1; parent_transcript=GRMZM2G151041_T01; parent_gene=GRMZM2G151041	GRMZM2G151041_P01,GRMZM2G151041_P02	TRUE	TRUE	vYYELDEER	95%	n+304 (+304)	41.02	25.00
2153	seq=translation; coord=1:263143605..263149463:1; parent_transcript=GRMZM2G079668_T01; parent_gene=GRMZM2G079668	GRMZM2G079668_P01,GRMZM2G079668_P02	TRUE	TRUE	mELSSLTQTNMSLPFITATADGPk	95%	n+304 (+304), K+304 (+304)	76.47	25.63
2154	seq=translation; coord=1:263143605..263149463:1; parent_transcript=GRMZM2G079668_T01; parent_gene=GRMZM2G079668	GRMZM2G079668_P01,GRMZM2G079668_P02	TRUE	TRUE	qVSYLVIR	95%	n+304 (+304)	33.03	25.00
2155	seq=translation; coord=3:41741731..41743930:-1; parent_transcript=GRMZM2G144648_T01; parent_gene=GRMZM2G144648	GRMZM2G144648_P01	TRUE	TRUE	acPGVVScADIVALAAR	95%	n+304 (+304), Carbamidomethyl (+57), Carbamidomethyl (+57)	68.14	25.47
2156	seq=translation; coord=3:41741731..41743930:-1; parent_transcript=GRMZM2G144648_T01; parent_gene=GRMZM2G144648	GRMZM2G144648_P01	TRUE	TRUE	aSLWQVETGR	86%	n+304 (+304)	25.84	25.66
2157	seq=translation; coord=3:41741731..41743930:-1; parent_transcript=GRMZM2G144648_T01; parent_gene=GRMZM2G144648	GRMZM2G144648_P01	TRUE	TRUE	dAVSYQFk	92%	n+304 (+304), K+304 (+304)	29.13	25.00

2158	seq=translation; coord=3:41741731..41743930:-1; parent_transcript=GRMZM2G144648_T01; parent_gene=GRMZM2G144648	GRMZM2G144648_P01	TRUE	TRUE	gcDASILLDTAQSEk	95%	n+304 (+304), Carbamidomethyl (+57), K+304 (+304)	33.64	25.08
2159	seq=translation; coord=3:41741731..41743930:-1; parent_transcript=GRMZM2G144648_T01; parent_gene=GRMZM2G144648	GRMZM2G144648_P01	TRUE	TRUE	nScPSVDSIVR	95%	n+304 (+304), Carbamidomethyl (+57)	36.21	25.00
2160	seq=translation; coord=3:41741731..41743930:-1; parent_transcript=GRMZM2G144648_T01; parent_gene=GRMZM2G144648	GRMZM2G144648_P01	TRUE	TRUE	sVTWAQVAANPALPAR	95%	n+304 (+304)	49.27	25.61
2161	seq=translation; coord=3:41741731..41743930:-1; parent_transcript=GRMZM2G144648_T01; parent_gene=GRMZM2G144648	GRMZM2G144648_P01	TRUE	TRUE	tAAPNLSVGGYEVIDAIk	95%	n+304 (+304), K+304 (+304)	67.74	25.07
2162	seq=translation; coord=4:232510364..232513106:1; parent_transcript=GRMZM2G030228_T01; parent_gene=GRMZM2G030228	GRMZM2G030228_P01,GRMZM2G030228_P02, GRMZM2G030228_P03,GRMZM2G051848_P01, GRMZM2G063700_P01,GRMZM2G063700_P02, GRMZM2G336875_P01,GRMZM2G360677_P01, GRMZM2G360677_P02,GRMZM2G360677_P03	TRUE	TRUE	aDGYILEGk	95%	n+304 (+304), K+304 (+304)	37.89	26.52
2163	seq=translation; coord=4:232510364..232513106:1; parent_transcript=GRMZM2G030228_T01; parent_gene=GRMZM2G030228	GRMZM2G030228_P01,GRMZM2G030228_P02, GRMZM2G030228_P03,GRMZM2G051848_P01, GRMZM2G063700_P01,GRMZM2G063700_P02, GRMZM2G336875_P01,GRMZM2G360677_P01, GRMZM2G360677_P02,GRMZM2G360677_P03	TRUE	TRUE	eLEFYMk	95%	n+304 (+304), K+304 (+304)	34.77	25.00
2164	seq=translation; coord=4:232510364..232513106:1; parent_transcript=GRMZM2G030228_T01; parent_gene=GRMZM2G030228	GRMZM2G030228_P01,GRMZM2G030228_P02, GRMZM2G030228_P03,GRMZM2G051848_P01, GRMZM2G063700_P01,GRMZM2G063700_P02, GRMZM2G336875_P01,GRMZM2G360677_P01, GRMZM2G360677_P02,GRMZM2G360677_P03	TRUE	TRUE	iLDVVYNASNNELVR	95%	n+304 (+304)	48.17	26.19
2165	seq=translation; coord=4:232510364..232513106:1; parent_transcript=GRMZM2G030228_T01; parent_gene=GRMZM2G030228	GRMZM2G030228_P01,GRMZM2G030228_P02, GRMZM2G030228_P03,GRMZM2G051848_P01, GRMZM2G063700_P01,GRMZM2G063700_P02, GRMZM2G336875_P01,GRMZM2G360677_P01, GRMZM2G360677_P02,GRMZM2G360677_P03	TRUE	TRUE	kDNAEGQEVEAAAEETk	95%	K+304 (+304), n+304 (+304), K+304 (+304)	41.17	25.66
2166	seq=translation; coord=4:232510364..232513106:1; parent_transcript=GRMZM2G030228_T01; parent_gene=GRMZM2G030228	GRMZM2G030228_P01,GRMZM2G030228_P02, GRMZM2G030228_P03,GRMZM2G051848_P01, GRMZM2G063700_P01,GRMZM2G063700_P02, GRMZM2G336875_P01,GRMZM2G360677_P01, GRMZM2G360677_P02,GRMZM2G360677_P03	TRUE	TRUE	lDTGNYSWGSEAVTR	95%	n+304 (+304)	66.48	25.00
2167	seq=translation; coord=4:232510364..232513106:1; parent_transcript=GRMZM2G030228_T01; parent_gene=GRMZM2G030228	GRMZM2G030228_P01,GRMZM2G030228_P02, GRMZM2G030228_P03,GRMZM2G051848_P01, GRMZM2G063700_P01,GRMZM2G063700_P02, GRMZM2G336875_P01,GRMZM2G360677_P01, GRMZM2G360677_P02,GRMZM2G360677_P03	TRUE	TRUE	qWYLTHYGVDIGR	95%	n+304 (+304)	50.56	25.05
2168	seq=translation; coord=4:232510364..232513106:1; parent_transcript=GRMZM2G030228_T01; parent_gene=GRMZM2G030228	GRMZM2G030228_P01,GRMZM2G030228_P02, GRMZM2G030228_P03,GRMZM2G051848_P01, GRMZM2G063700_P01,GRMZM2G063700_P02, GRMZM2G336875_P01,GRMZM2G360677_P01, GRMZM2G360677_P02,GRMZM2G360677_P03	TRUE	TRUE	qWyLTHYGVDIGR	91%	Pyro-cmC (-17), n+304 (+304), iTRAQ8plex (+304)	25.53	25.00

2169	seq=translation; coord=4:232510364..232513106:1; parent_transcript=GRMZM2G030228_T01; parent_gene=GRMZM2G030228	GRMZM2G030228_P01,GRMZM2G030228_P02, GRMZM2G030228_P03,GRMZM2G051848_P01, GRMZM2G063700_P01,GRMZM2G063700_P02, GRMZM2G336875_P01,GRMZM2G360677_P01, GRMZM2G360677_P02,GRMZM2G360677_P03	TRUE	TRUE	sAIVQVDAAPFk	95%	n+304 (+304), K+304 (+304)	50.38	26.05
2170	seq=translation; coord=4:232510364..232513106:1; parent_transcript=GRMZM2G030228_T01; parent_gene=GRMZM2G030228	GRMZM2G030228_P01,GRMZM2G030228_P02, GRMZM2G030228_P03,GRMZM2G051848_P01, GRMZM2G063700_P01,GRMZM2G063700_P02, GRMZM2G336875_P01,GRMZM2G360677_P01, GRMZM2G360677_P02,GRMZM2G360677_P03	TRUE	TRUE	tLDPHIEEQFGSGR	95%	n+304 (+304)	48.72	25.00
2171	seq=translation; coord=5:199786088..199787380:1; parent_transcript=GRMZM2G054201_T01; parent_gene=GRMZM2G054201	GRMZM2G054201_P01,GRMZM2G054201_P02, GRMZM2G054201_P03,GRMZM2G121075_P01, GRMZM2G121075_P02	TRUE	TRUE	hSGNISLDDVIEIAR	95%	n+304 (+304)	47.70	25.22
2172	seq=translation; coord=4:176858064..176868636:-1; parent_transcript=GRMZM2G149281_T01; parent_gene=GRMZM2G149281	GRMZM2G149281_P01,GRMZM2G149281_P02, GRMZM2G149281_P03,GRMZM2G149281_P04	TRUE	TRUE	aIFAPYSLk	94%	n+304 (+304), K+304 (+304)	27.19	25.00
2173	seq=translation; coord=4:176858064..176868636:-1; parent_transcript=GRMZM2G149281_T01; parent_gene=GRMZM2G149281	GRMZM2G149281_P01,GRMZM2G149281_P02, GRMZM2G149281_P03,GRMZM2G149281_P04	TRUE	TRUE	iSPAFVLFFDcSEEDMEk	95%	n+304 (+304), Carbamidomethyl (+57), K+304 (+304)	62.62	25.00
2174	seq=translation; coord=4:176858064..176868636:-1; parent_transcript=GRMZM2G149281_T01; parent_gene=GRMZM2G149281	GRMZM2G149281_P01,GRMZM2G149281_P02, GRMZM2G149281_P03,GRMZM2G149281_P04	TRUE	TRUE	vTVVFVLGGPGSGk	95%	n+304 (+304), K+304 (+304)	50.29	25.00
2175	seq=translation; coord=4:221070736..221076802:-1; parent_transcript=GRMZM5G854613_T01; parent_gene=GRMZM5G854613	GRMZM5G854613_P01	TRUE	TRUE	aSDLDIASVLGMGFPk	95%	n+304 (+304), K+304 (+304)	48.44	25.93
2176	seq=translation; coord=4:221070736..221076802:-1; parent_transcript=GRMZM5G854613_T01; parent_gene=GRMZM5G854613	GRMZM5G854613_P01	TRUE	TRUE	dIYADAFGER	87%	n+304 (+304)	25.51	25.00
2177	seq=translation; coord=4:221070736..221076802:-1; parent_transcript=GRMZM5G854613_T01; parent_gene=GRMZM5G854613	GRMZM5G854613_P01	TRUE	TRUE	dVDMVIEAVIEk	95%	n+304 (+304), K+304 (+304)	33.75	26.82
2178	seq=translation; coord=4:221070736..221076802:-1; parent_transcript=GRMZM5G854613_T01; parent_gene=GRMZM5G854613	GRMZM5G854613_P01	TRUE	TRUE	eVNPQFLQR	89%	n+304 (+304)	26.78	25.25
2179	seq=translation; coord=4:221070736..221076802:-1; parent_transcript=GRMZM5G854613_T01; parent_gene=GRMZM5G854613	GRMZM5G854613_P01	TRUE	TRUE	nLDSDLVDLMVk	95%	n+304 (+304), K+304 (+304)	63.49	26.41
2180	seq=translation; coord=4:221070736..221076802:-1; parent_transcript=GRMZM5G854613_T01; parent_gene=GRMZM5G854613	GRMZM5G854613_P01	TRUE	TRUE	tIAGNLEGLVk	95%	n+304 (+304), K+304 (+304)	55.04	25.56
2181	seq=translation; coord=4:221070736..221076802:-1; parent_transcript=GRMZM5G854613_T01; parent_gene=GRMZM5G854613	GRMZM5G854613_P01	TRUE	TRUE	tSPQAILDLITIGk	95%	n+304 (+304), K+304 (+304)	36.62	25.00
2182	seq=translation; coord=4:221070736..221076802:-1; parent_transcript=GRMZM5G854613_T01; parent_gene=GRMZM5G854613	GRMZM5G854613_P01	TRUE	TRUE	vMDENVVIR	91%	n+304 (+304)	28.76	25.61
2183	seq=translation; coord=1:12248908..12249912:-1; parent_transcript=GRMZM2G472236_T01; parent_gene=GRMZM2G472236	GRMZM2G472236_P01	TRUE	TRUE	aTLGDVLANATAR	95%	n+304 (+304)	57.16	26.54
2184	seq=translation; coord=1:12248908..12249912:-1; parent_transcript=GRMZM2G472236_T01; parent_gene=GRMZM2G472236	GRMZM2G472236_P01	TRUE	TRUE	gGPAAAMQSAATANER	95%	n+304 (+304)	74.55	25.00

2185	seq=translation; coord=1:12248908..12249912:-1; parent_transcript=GRMZM2G472236_T01; parent_gene=GRMZM2G472236	GRMZM2G472236_P01	TRUE	TRUE	iVTEFVAGQAVGQyLAR	95%	n+304 (+304), iTRAQ8plex (+304)	32.86	25.00
2186	seq=translation; coord=1:12248908..12249912:-1; parent_transcript=GRMZM2G472236_T01; parent_gene=GRMZM2G472236	GRMZM2G472236_P01	TRUE	TRUE	mGAVGHDQATDATAVQGVTVSETR	95%	n+304 (+304)	81.65	25.00
2187	seq=translation; coord=1:12248908..12249912:-1; parent_transcript=GRMZM2G472236_T01; parent_gene=GRMZM2G472236	GRMZM2G472236_P01	TRUE	TRUE	sDAAAIQAAEAR	95%	n+304 (+304)	59.34	25.00
2188	seq=translation; coord=1:12248908..12249912:-1; parent_transcript=GRMZM2G472236_T01; parent_gene=GRMZM2G472236	GRMZM2G472236_P01	TRUE	TRUE	vTIGEALEATALAAGDAPVER	95%	n+304 (+304)	47.88	25.48
2189	seq=translation; coord=2:205943881..205945286:-1; parent_transcript=GRMZM2G099295_T01; parent_gene=GRMZM2G099295	GRMZM2G099295_P01	TRUE	TRUE	dDARDEINTVDLSHNR	95%	n+304 (+304)	27.29	25.00
2190	seq=translation; coord=2:205943881..205945286:-1; parent_transcript=GRMZM2G099295_T01; parent_gene=GRMZM2G099295	GRMZM2G099295_P01	TRUE	TRUE	dIHALLSVk	92%	n+304 (+304), K+304 (+304)	26.48	25.00
2191	seq=translation; coord=2:205943881..205945286:-1; parent_transcript=GRMZM2G099295_T01; parent_gene=GRMZM2G099295	GRMZM2G099295_P01	TRUE	TRUE	gLDSVDLSSNR	95%	n+304 (+304)	37.28	25.00
2192	seq=translation; coord=2:205943881..205945286:-1; parent_transcript=GRMZM2G099295_T01; parent_gene=GRMZM2G099295	GRMZM2G099295_P01	TRUE	TRUE	gQIPSAVGGLTELMSLTLFR	95%	n+304 (+304)	56.96	26.13
2193	seq=translation; coord=2:205943881..205945286:-1; parent_transcript=GRMZM2G099295_T01; parent_gene=GRMZM2G099295	GRMZM2G099295_P01	TRUE	TRUE	gQIPSAVGGLTELmSLTLFR	95%	n+304 (+304), Oxidation (+16)	65.75	25.22
2194	seq=translation; coord=2:205943881..205945286:-1; parent_transcript=GRMZM2G099295_T01; parent_gene=GRMZM2G099295	GRMZM2G099295_P01	TRUE	TRUE	hNQLTGPIPAGLVQGQFR	95%	n+304 (+304)	33.76	25.37
2195	seq=translation; coord=2:205943881..205945286:-1; parent_transcript=GRMZM2G099295_T01; parent_gene=GRMZM2G099295	GRMZM2G099295_P01	TRUE	TRUE	lTGGIPAAFADLPSLR	95%	n+304 (+304)	63.22	26.42
2196	seq=translation; coord=2:205943881..205945286:-1; parent_transcript=GRMZM2G099295_T01; parent_gene=GRMZM2G099295	GRMZM2G099295_P01	TRUE	TRUE	lVFPPELTYLDLSHNLIR	95%	n+304 (+304)	63.58	25.01
2197	seq=translation; coord=1:77258356..77350693:1; parent_transcript=GRMZM2G020523_T01; parent_gene=GRMZM2G020523	GRMZM2G020523_P01	TRUE	TRUE	dSVAQLGGPSWAVPLGR	95%	n+304 (+304)	80.08	26.28
2198	seq=translation; coord=1:77258356..77350693:1; parent_transcript=GRMZM2G020523_T01; parent_gene=GRMZM2G020523	GRMZM2G020523_P01	TRUE	TRUE	gAGPNAGSLR	93%	n+304 (+304)	30.10	25.00
2199	seq=translation; coord=1:77258356..77350693:1; parent_transcript=GRMZM2G020523_T01; parent_gene=GRMZM2G020523	GRMZM2G020523_P01	TRUE	TRUE	gFDVIDNIk	95%	n+304 (+304), K+304 (+304)	37.12	26.11
2200	seq=translation; coord=1:77258356..77350693:1; parent_transcript=GRMZM2G020523_T01; parent_gene=GRMZM2G020523	GRMZM2G020523_P01	TRUE	TRUE	gLSSTDMVALSGAHTVGR	95%	n+304 (+304)	57.69	25.00
2201	seq=translation; coord=1:77258356..77350693:1; parent_transcript=GRMZM2G020523_T01; parent_gene=GRMZM2G020523	GRMZM2G020523_P01	TRUE	TRUE	mGSISPLTGTDGEIR	95%	n+304 (+304)	54.24	25.00
2202	seq=translation; coord=1:77258356..77350693:1; parent_transcript=GRMZM2G020523_T01; parent_gene=GRMZM2G020523	GRMZM2G020523_P01	TRUE	TRUE	tAVSTAVLLEPR	95%	n+304 (+304)	50.03	25.59

2203	seq=translation; coord=6:127495800..127501815:-1; parent_transcript=GRMZM2G009845_T01; parent_gene=GRMZM2G009845	GRMZM2G009845_P01,GRMZM2G009845_P02, GRMZM2G088088_P01,GRMZM2G088088_P02	TRUE	TRUE	eGQMVDIR	86%	n+304 (+304)	25.24	25.00
2204	seq=translation; coord=6:127495800..127501815:-1; parent_transcript=GRMZM2G009845_T01; parent_gene=GRMZM2G009845	GRMZM2G009845_P01,GRMZM2G009845_P02, GRMZM2G088088_P01,GRMZM2G088088_P02	TRUE	TRUE	iQAVIESGVFPR	95%	n+304 (+304)	46.41	26.20
2205	seq=translation; coord=6:127495800..127501815:-1; parent_transcript=GRMZM2G009845_T01; parent_gene=GRMZM2G009845	GRMZM2G009845_P01,GRMZM2G009845_P02, GRMZM2G088088_P01,GRMZM2G088088_P02	TRUE	TRUE	lVELLMHPSASVLIPALR	95%	n+304 (+304)	56.07	25.00
2206	seq=translation; coord=6:127495800..127501815:-1; parent_transcript=GRMZM2G009845_T01; parent_gene=GRMZM2G009845	GRMZM2G009845_P01,GRMZM2G009845_P02, GRMZM2G088088_P01,GRMZM2G088088_P02	TRUE	TRUE	sPPIEEVISTGVVPR	95%	n+304 (+304)	46.54	26.04
2207	seq=translation; coord=6:127495800..127501815:-1; parent_transcript=GRMZM2G009845_T01; parent_gene=GRMZM2G009845	GRMZM2G009845_P01,GRMZM2G009845_P02, GRMZM2G088088_P01,GRMZM2G088088_P02	TRUE	TRUE	tVGNIVTGDDMQTQcVIDNQALPcLLNLLTTNHk	95%	n+304 (+304), Carbamidomethyl (+57), Carbamidomethyl (+57), K+304 (+304)	26.39	25.00
2208	seq=translation; coord=6:127495800..127501815:-1; parent_transcript=GRMZM2G009845_T01; parent_gene=GRMZM2G009845	GRMZM2G009845_P01,GRMZM2G009845_P02, GRMZM2G088088_P01,GRMZM2G088088_P02	TRUE	TRUE	yLVAQGcIkPLcDLLVcPDPR	95%	n+304 (+304), Carbamidomethyl (+57), K+304 (+304), Carbamidomethyl (+57), Carbamidomethyl (+57)	32.77	25.00
2209	seq=translation; coord=4:36880092..36884475:-1; parent_transcript=GRMZM2G046804_T01; parent_gene=GRMZM2G046804	GRMZM2G046804_P01,GRMZM2G046804_P02, GRMZM2G046804_P04,GRMZM2G046804_P05, GRMZM2G046804_P06,GRMZM2G046804_P07, GRMZM2G046804_P08	TRUE	TRUE	aGIALNDHFVk	95%	n+304 (+304), K+304 (+304)	34.42	25.02
2210	seq=translation; coord=4:36880092..36884475:-1; parent_transcript=GRMZM2G046804_T01; parent_gene=GRMZM2G046804	GRMZM2G046804_P01,GRMZM2G046804_P02, GRMZM2G046804_P04,GRMZM2G046804_P05, GRMZM2G046804_P06,GRMZM2G046804_P07, GRMZM2G046804_P08	TRUE	TRUE	gASYEDIk	95%	n+304 (+304), K+304 (+304)	35.67	26.13
2211	seq=translation; coord=4:36880092..36884475:-1; parent_transcript=GRMZM2G046804_T01; parent_gene=GRMZM2G046804	GRMZM2G046804_P01,GRMZM2G046804_P02, GRMZM2G046804_P04,GRMZM2G046804_P05, GRMZM2G046804_P06,GRMZM2G046804_P07, GRMZM2G046804_P08	TRUE	TRUE	gIMGYAEEDLVSTDFLGDSR	95%	n+304 (+304)	32.16	25.00
2212	seq=translation; coord=4:36880092..36884475:-1; parent_transcript=GRMZM2G046804_T01; parent_gene=GRMZM2G046804	GRMZM2G046804_P01,GRMZM2G046804_P02, GRMZM2G046804_P04,GRMZM2G046804_P05, GRMZM2G046804_P06,GRMZM2G046804_P07, GRMZM2G046804_P08	TRUE	TRUE	hSDITLk	91%	n+304 (+304), K+304 (+304)	25.97	25.00
2213	seq=translation; coord=4:36880092..36884475:-1; parent_transcript=GRMZM2G046804_T01; parent_gene=GRMZM2G046804	GRMZM2G046804_P01,GRMZM2G046804_P02, GRMZM2G046804_P04,GRMZM2G046804_P05, GRMZM2G046804_P06,GRMZM2G046804_P07, GRMZM2G046804_P08	TRUE	TRUE	tLLFGDkPVTVFGIR	95%	n+304 (+304), K+304 (+304)	36.53	25.00
2214	seq=translation; coord=4:36880092..36884475:-1; parent_transcript=GRMZM2G046804_T01; parent_gene=GRMZM2G046804	GRMZM2G046804_P01,GRMZM2G046804_P02, GRMZM2G046804_P04,GRMZM2G046804_P05, GRMZM2G046804_P06,GRMZM2G046804_P07, GRMZM2G046804_P08	TRUE	TRUE	vIHDNFGIVEGLMTTVHAITATQk	95%	n+304 (+304), K+304 (+304)	27.88	25.00
2215	seq=translation; coord=6:135883373..135887597:1; parent_transcript=GRMZM2G059991_T01; parent_gene=GRMZM2G059991	GRMZM2G059991_P01	TRUE	TRUE	aLEQLDTAVSk	95%	n+304 (+304), K+304 (+304)	36.28	26.89
2216	seq=translation; coord=4:20772978..20777200:-1; parent_transcript=GRMZM2G078143_T01; parent_gene=GRMZM2G078143	GRMZM2G078143_P01	TRUE	TRUE	aNAVAIGNYLMSk	95%	n+304 (+304), K+304 (+304)	58.30	25.45
2217	seq=translation; coord=4:20772978..20777200:-1; parent_transcript=GRMZM2G078143_T01; parent_gene=GRMZM2G078143	GRMZM2G078143_P01	TRUE	TRUE	fADSFDMPGFTLESMk	95%	n+304 (+304), K+304 (+304)	55.05	25.00

2218	seq=translation; coord=4:20772978..20777200:-1; parent_transcript=GRMZM2G078143_T01; parent_gene=GRMZM2G078143	GRMZM2G078143_P01	TRUE	TRUE	iGAPAMTSR	95%	n+304 (+304)	32.74	25.15
2219	seq=translation; coord=4:20772978..20777200:-1; parent_transcript=GRMZM2G078143_T01; parent_gene=GRMZM2G078143	GRMZM2G078143_P01	TRUE	TRUE	lIIcGGSAYPR	94%	n+304 (+304), Carbamidomethyl (+57)	28.32	25.00
2220	seq=translation; coord=4:20772978..20777200:-1; parent_transcript=GRMZM2G078143_T01; parent_gene=GRMZM2G078143	GRMZM2G078143_P01	TRUE	TRUE	nAVFGDSSALSPGGVR	95%	n+304 (+304)	34.04	25.00
2221	seq=translation; coord=4:20772978..20777200:-1; parent_transcript=GRMZM2G078143_T01; parent_gene=GRMZM2G078143	GRMZM2G078143_P01	TRUE	TRUE	vSAATGYIDYEk	91%	n+304 (+304), K+304 (+304)	26.76	25.50
2222	seq=translation; coord=4:20772978..20777200:-1; parent_transcript=GRMZM2G078143_T01; parent_gene=GRMZM2G078143	GRMZM2G078143_P01	TRUE	TRUE	yYGGNDVIDEIENLcR	95%	n+304 (+304), Carbamidomethyl (+57)	49.55	25.00
2223	seq=translation; coord=4:20772978..20777200:-1; parent_transcript=GRMZM2G078143_T01; parent_gene=GRMZM2G078143	GRMZM2G078143_P01	TRUE	TRUE	yYGGNDVIDEIENLcR	95%	n+304 (+304), iTRAQ8plex (+304), Carbamidomethyl (+57)	34.42	25.00
2224	seq=translation; coord=5:216695478..216702082:-1; parent_transcript=GRMZM2G133919_T01; parent_gene=GRMZM2G133919	GRMZM2G133919_P01	TRUE	TRUE	dSGSPEANDLTHWDLTFWSER	95%	n+304 (+304)	62.54	25.00
2225	seq=translation; coord=5:216695478..216702082:-1; parent_transcript=GRMZM2G133919_T01; parent_gene=GRMZM2G133919	GRMZM2G133919_P01	TRUE	TRUE	eAVLSGVALEDEQR	95%	n+304 (+304)	76.30	25.48
2226	seq=translation; coord=5:216695478..216702082:-1; parent_transcript=GRMZM2G133919_T01; parent_gene=GRMZM2G133919	GRMZM2G133919_P01	TRUE	TRUE	fSENVLDATk	95%	n+304 (+304), K+304 (+304)	35.35	26.20
2227	seq=translation; coord=5:216695478..216702082:-1; parent_transcript=GRMZM2G133919_T01; parent_gene=GRMZM2G133919	GRMZM2G133919_P01	TRUE	TRUE	gIEPTWAk	89%	n+304 (+304), K+304 (+304)	25.95	25.00
2228	seq=translation; coord=5:216695478..216702082:-1; parent_transcript=GRMZM2G133919_T01; parent_gene=GRMZM2G133919	GRMZM2G133919_P01	TRUE	TRUE	gREPSPEPLLR	95%	n+304 (+304)	42.82	26.87
2229	seq=translation; coord=5:216695478..216702082:-1; parent_transcript=GRMZM2G133919_T01; parent_gene=GRMZM2G133919	GRMZM2G133919_P01	TRUE	TRUE	gVEWDAVELPSQFMENWcYHk	94%	n+304 (+304), Carbamidomethyl (+57), K+304 (+304)	25.00	25.00
2230	seq=translation; coord=5:216695478..216702082:-1; parent_transcript=GRMZM2G133919_T01; parent_gene=GRMZM2G133919	GRMZM2G133919_P01	TRUE	TRUE	lEGELEELEk	95%	n+304 (+304), K+304 (+304)	42.56	25.00
2231	seq=translation; coord=5:216695478..216702082:-1; parent_transcript=GRMZM2G133919_T01; parent_gene=GRMZM2G133919	GRMZM2G133919_P01	TRUE	TRUE	lEVIWGMVDHLk	95%	n+304 (+304), K+304 (+304)	26.64	25.00
2232	seq=translation; coord=5:216695478..216702082:-1; parent_transcript=GRMZM2G133919_T01; parent_gene=GRMZM2G133919	GRMZM2G133919_P01	TRUE	TRUE	qDEGFVAGIR	89%	n+304 (+304)	27.05	25.00
2233	seq=translation; coord=5:216695478..216702082:-1; parent_transcript=GRMZM2G133919_T01; parent_gene=GRMZM2G133919	GRMZM2G133919_P01	TRUE	TRUE	tQVLAPLPEDR	89%	n+304 (+304)	28.33	26.69
2234	seq=translation; coord=5:216695478..216702082:-1; parent_transcript=GRMZM2G133919_T01; parent_gene=GRMZM2G133919	GRMZM2G133919_P01	TRUE	TRUE	vMDGLFTLAHk	95%	n+304 (+304), K+304 (+304)	34.70	26.10
2235	seq=translation; coord=8:103922075..103930908:-1; parent_transcript=GRMZM2G435373_T01; parent_gene=GRMZM2G435373	GRMZM2G435373_P01,GRMZM2G435373_P02	TRUE	TRUE	aVDGSYVFSk	95%	n+304 (+304), K+304 (+304)	37.13	25.00

2236	seq=translation; coord=8:103922075..103930908:-1; parent_transcript=GRMZM2G435373_T01; parent_gene=GRMZM2G435373	GRMZM2G435373_P01,GRMZM2G435373_P02	TRUE	TRUE	dYNVDMVPk	95%	n+304 (+304), K+304 (+304)	33.03	25.28
2237	seq=translation; coord=8:103922075..103930908:-1; parent_transcript=GRMZM2G435373_T01; parent_gene=GRMZM2G435373	GRMZM2G435373_P01,GRMZM2G435373_P02	TRUE	TRUE	ecILSGLLSVDGLk	95%	n+304 (+304), Carbamidomethyl (+57), K+304 (+304)	46.85	25.61
2238	seq=translation; coord=8:103922075..103930908:-1; parent_transcript=GRMZM2G435373_T01; parent_gene=GRMZM2G435373	GRMZM2G435373_P01,GRMZM2G435373_P02	TRUE	TRUE	lYSESLAR	90%	n+304 (+304)	26.47	25.00
2239	seq=translation; coord=8:103922075..103930908:-1; parent_transcript=GRMZM2G435373_T01; parent_gene=GRMZM2G435373	GRMZM2G435373_P01,GRMZM2G435373_P02	TRUE	TRUE	sPLMGLFEk	95%	n+304 (+304), K+304 (+304)	33.04	25.74
2240	seq=translation; coord=8:103922075..103930908:-1; parent_transcript=GRMZM2G435373_T01; parent_gene=GRMZM2G435373	GRMZM2G435373_P01,GRMZM2G435373_P02	TRUE	TRUE	vEFDMEGk	94%	n+304 (+304), K+304 (+304)	31.97	25.73
2241	seq=translation; coord=8:103922075..103930908:-1; parent_transcript=GRMZM2G435373_T01; parent_gene=GRMZM2G435373	GRMZM2G435373_P01,GRMZM2G435373_P02	TRUE	TRUE	yGLSDDTVDFIGHALALHR	95%	n+304 (+304)	63.77	25.07
2242	seq=translation; coord=8:103922075..103930908:-1; parent_transcript=GRMZM2G435373_T01; parent_gene=GRMZM2G435373	GRMZM2G435373_P01,GRMZM2G435373_P02	TRUE	TRUE	yGLSDDTVDFIGHALALHR	95%	n+304 (+304), iTRAQ8plex (+304)	34.28	25.35
2243	seq=translation; coord=8:103922075..103930908:-1; parent_transcript=GRMZM2G435373_T01; parent_gene=GRMZM2G435373	GRMZM2G435373_P01,GRMZM2G435373_P02	TRUE	TRUE	yLDEPALDTVk	95%	n+304 (+304), K+304 (+304)	39.55	26.51
2244	seq=translation; coord=3:168231994..168235001:-1; parent_transcript=GRMZM5G815894_T03; parent_gene=GRMZM5G815894	GRMZM5G815894_P03	TRUE	TRUE	aLLDVGLIR	94%	n+304 (+304)	30.29	25.00
2245	seq=translation; coord=3:168231994..168235001:-1; parent_transcript=GRMZM5G815894_T03; parent_gene=GRMZM5G815894	GRMZM5G815894_P03	TRUE	TRUE	dITAQIISASIAGDMVLASAYSHELPR	95%	n+304 (+304)	79.74	25.56
2246	seq=translation; coord=3:168231994..168235001:-1; parent_transcript=GRMZM5G815894_T03; parent_gene=GRMZM5G815894	GRMZM5G815894_P03	TRUE	TRUE	dITAQIISASIAGDMVLASAySHELPR	95%	n+304 (+304), iTRAQ8plex (+304)	42.75	25.09
2247	seq=translation; coord=3:168231994..168235001:-1; parent_transcript=GRMZM5G815894_T03; parent_gene=GRMZM5G815894	GRMZM5G815894_P03	TRUE	TRUE	gALDGGLDIPHSEk	94%	n+304 (+304), K+304 (+304)	29.52	26.05
2248	seq=translation; coord=3:168231994..168235001:-1; parent_transcript=GRMZM5G815894_T03; parent_gene=GRMZM5G815894	GRMZM5G815894_P03	TRUE	TRUE	gIEADDMEALYk	93%	n+304 (+304), K+304 (+304)	27.62	25.42
2249	seq=translation; coord=3:168231994..168235001:-1; parent_transcript=GRMZM5G815894_T03; parent_gene=GRMZM5G815894	GRMZM5G815894_P03	TRUE	TRUE	nLAEEEPEk	95%	n+304 (+304), K+304 (+304)	36.20	25.00
2250	seq=translation; coord=1:276305013..276310701:1; parent_transcript=GRMZM2G147687_T01; parent_gene=GRMZM2G147687	GRMZM2G147687_P01,GRMZM2G147687_P02, GRMZM2G147687_P03,GRMZM2G147687_P04	TRUE	TRUE	aTIFPHNVGLGATR	95%	n+304 (+304)	49.29	26.02
2251	seq=translation; coord=1:276305013..276310701:1; parent_transcript=GRMZM2G147687_T01; parent_gene=GRMZM2G147687	GRMZM2G147687_P01,GRMZM2G147687_P02, GRMZM2G147687_P03,GRMZM2G147687_P04	TRUE	TRUE	gFVISDWEGIDR	95%	n+304 (+304)	41.13	25.00
2252	seq=translation; coord=1:276305013..276310701:1; parent_transcript=GRMZM2G147687_T01; parent_gene=GRMZM2G147687	GRMZM2G147687_P01,GRMZM2G147687_P02, GRMZM2G147687_P03,GRMZM2G147687_P04	TRUE	TRUE	iDDAVYR	90%	n+304 (+304)	27.65	25.00
2253	seq=translation; coord=1:276305013..276310701:1; parent_transcript=GRMZM2G147687_T01; parent_gene=GRMZM2G147687	GRMZM2G147687_P01,GRMZM2G147687_P02, GRMZM2G147687_P03,GRMZM2G147687_P04	TRUE	TRUE	iGEATALEVR	94%	n+304 (+304)	32.49	26.19

2254	seq=translation; coord=1:276305013..276310701:1; parent_transcript=GRMZM2G147687_T01; parent_gene=GRMZM2G147687	GRMZM2G147687_P01,GRMZM2G147687_P02, GRMZM2G147687_P03,GRMZM2G147687_P04	TRUE	TRUE	iGQMTQIER	89%	n+304 (+304)	26.58	25.00
2255	seq=translation; coord=1:276305013..276310701:1; parent_transcript=GRMZM2G147687_T01; parent_gene=GRMZM2G147687	GRMZM2G147687_P01,GRMZM2G147687_P02, GRMZM2G147687_P03,GRMZM2G147687_P04	TRUE	TRUE	sLVLLk	95%	n+304 (+304), K+304 (+304)	32.74	25.00
2256	seq=translation; coord=1:276305013..276310701:1; parent_transcript=GRMZM2G147687_T01; parent_gene=GRMZM2G147687	GRMZM2G147687_P01,GRMZM2G147687_P02, GRMZM2G147687_P03,GRMZM2G147687_P04	TRUE	TRUE	sSYAPLLPLPk	95%	n+304 (+304), K+304 (+304)	50.88	25.02
2257	seq=translation; coord=1:276305013..276310701:1; parent_transcript=GRMZM2G147687_T01; parent_gene=GRMZM2G147687	GRMZM2G147687_P01,GRMZM2G147687_P02, GRMZM2G147687_P03,GRMZM2G147687_P04	TRUE	TRUE	sVDQLPMNVGDAHYDPLFPFGFGLTTk	95%	n+304 (+304), K+304 (+304)	47.31	25.00
2258	seq=translation; coord=6:156819019..156827574:1; parent_transcript=GRMZM2G701221_T01; parent_gene=GRMZM2G701221	GRMZM2G701221_P01	TRUE	TRUE	aAAAEEEPEEAk	95%	n+304 (+304), K+304 (+304)	51.45	25.00
2259	seq=translation; coord=6:156819019..156827574:1; parent_transcript=GRMZM2G701221_T01; parent_gene=GRMZM2G701221	GRMZM2G701221_P01	TRUE	TRUE	aQDLAQPLVGAQIPFk	95%	n+304 (+304), K+304 (+304)	64.16	25.00
2260	seq=translation; coord=6:156819019..156827574:1; parent_transcript=GRMZM2G701221_T01; parent_gene=GRMZM2G701221	GRMZM2G701221_P01	TRUE	TRUE	eEEFDSFTSTk	95%	n+304 (+304), K+304 (+304)	35.68	25.00
2261	seq=translation; coord=6:156819019..156827574:1; parent_transcript=GRMZM2G701221_T01; parent_gene=GRMZM2G701221	GRMZM2G701221_P01	TRUE	TRUE	iGIAVDLSDESAFAVk	95%	n+304 (+304), K+304 (+304)	55.14	25.94
2262	seq=translation; coord=6:156819019..156827574:1; parent_transcript=GRMZM2G701221_T01; parent_gene=GRMZM2G701221	GRMZM2G701221_P01	TRUE	TRUE	lSAAQAVAAIQPTSPR	95%	n+304 (+304)	53.97	26.28
2263	seq=translation; coord=6:156819019..156827574:1; parent_transcript=GRMZM2G701221_T01; parent_gene=GRMZM2G701221	GRMZM2G701221_P01	TRUE	TRUE	yPDDAFGDELR	95%	n+304 (+304)	64.26	25.00
2264	seq=translation; coord=8:90052293..90060254:-1; parent_transcript=GRMZM2G063676_T01; parent_gene=GRMZM2G063676	GRMZM2G063676_P01	TRUE	TRUE	eAALSSDQk	94%	n+304 (+304), K+304 (+304)	30.99	26.30
2265	seq=translation; coord=8:90052293..90060254:-1; parent_transcript=GRMZM2G063676_T01; parent_gene=GRMZM2G063676	GRMZM2G063676_P01	TRUE	TRUE	eFQVNDGFPFSIALSWk	95%	n+304 (+304), K+304 (+304)	29.33	25.00
2266	seq=translation; coord=8:90052293..90060254:-1; parent_transcript=GRMZM2G063676_T01; parent_gene=GRMZM2G063676	GRMZM2G063676_P01	TRUE	TRUE	fGHIDISEk	95%	n+304 (+304), K+304 (+304)	38.93	25.05
2267	seq=translation; coord=8:90052293..90060254:-1; parent_transcript=GRMZM2G063676_T01; parent_gene=GRMZM2G063676	GRMZM2G063676_P01	TRUE	TRUE	hTDPVLLVSDLk	95%	n+304 (+304), K+304 (+304)	28.81	25.00
2268	seq=translation; coord=8:90052293..90060254:-1; parent_transcript=GRMZM2G063676_T01; parent_gene=GRMZM2G063676	GRMZM2G063676_P01	TRUE	TRUE	kFSDPELQSDLASFPFR	95%	K+304 (+304), n+304 (+304)	50.61	25.73
2269	seq=translation; coord=8:90052293..90060254:-1; parent_transcript=GRMZM2G063676_T01; parent_gene=GRMZM2G063676	GRMZM2G063676_P01	TRUE	TRUE	vINEcSEVENWLR	95%	n+304 (+304), Carbamidomethyl (+57)	66.22	25.00
2270	seq=translation; coord=7:137455248..137460051:1; parent_transcript=GRMZM2G115757_T01; parent_gene=GRMZM2G115757	GRMZM2G115757_P01,GRMZM2G115757_P02	TRUE	TRUE	gDPIEFELGTGQVIk	95%	n+304 (+304), K+304 (+304)	51.54	25.29
2271	seq=translation; coord=7:137455248..137460051:1; parent_transcript=GRMZM2G115757_T01; parent_gene=GRMZM2G115757	GRMZM2G115757_P01,GRMZM2G115757_P02	TRUE	TRUE	lTDGTVFDSSYER	95%	n+304 (+304)	66.76	25.00

2272	seq=translation; coord=7:137455248..137460051:1; parent_transcript=GRMZM2G115757_T01; parent_gene=GRMZM2G115757	GRMZM2G115757_P01,GRMZM2G115757_P02	TRUE	TRUE	sGDVSELQIGVk	95%	n+304 (+304), K+304 (+304)	67.43	26.23
2273	seq=translation; coord=1:66023613..66026784:-1; parent_transcript=GRMZM2G078876_T01; parent_gene=GRMZM2G078876	GRMZM2G078876_P01	TRUE	TRUE	rPSAAccGEVk	94%	n+304 (+304), Carbamidomethyl (+57), Carbamidomethyl (+57), K+304 (+304)	27.36	25.00
2274	seq=translation; coord=1:66023613..66026784:-1; parent_transcript=GRMZM2G078876_T01; parent_gene=GRMZM2G078876	GRMZM2G078876_P01	TRUE	TRUE	rVLALPGAcGASNAAFSk	95%	n+304 (+304), Carbamidomethyl (+57), K+304 (+304)	48.98	25.39
2275	seq=translation; coord=1:66023613..66026784:-1; parent_transcript=GRMZM2G078876_T01; parent_gene=GRMZM2G078876	GRMZM2G078876_P01	TRUE	TRUE	tAVASPVIVGcLcSLAGSNSSNLGFPIDMk	95%	n+304 (+304), Carbamidomethyl (+57), Carbamidomethyl (+57), K+304 (+304)	49.31	25.00
2276	seq=translation; coord=1:66023613..66026784:-1; parent_transcript=GRMZM2G078876_T01; parent_gene=GRMZM2G078876	GRMZM2G078876_P01	TRUE	TRUE	vLALPGAcGASNAAFSk	95%	n+304 (+304), Carbamidomethyl (+57), K+304 (+304)	68.55	25.85
2277	seq=translation; coord=10:145479598..145483674:-1; parent_transcript=GRMZM2G073465_T03; parent_gene=GRMZM2G073465	GRMZM2G073465_P03	TRUE	TRUE	aVANQPISVAIEAGGR	95%	n+304 (+304)	67.62	25.61
2278	seq=translation; coord=10:145479598..145483674:-1; parent_transcript=GRMZM2G073465_T03; parent_gene=GRMZM2G073465	GRMZM2G073465_P03	TRUE	TRUE	vVTIDSYEDVPANSEk	95%	n+304 (+304), K+304 (+304)	42.72	25.00
2279	seq=translation; coord=6:9203729..9206286:-1; parent_transcript=GRMZM2G066460_T01; parent_gene=GRMZM2G066460	GRMZM2G066460_P01	TRUE	TRUE	fAVAAPVAAGDSGAAAAPk	95%	n+304 (+304), K+304 (+304)	61.88	25.26
2280	seq=translation; coord=1:180306606..180308510:1; parent_transcript=GRMZM2G161335_T01; parent_gene=GRMZM2G161335	GRMZM2G161335_P01	TRUE	TRUE	gFESGALPDGVEDEVR	90%	n+304 (+304)	25.37	25.00
2281	seq=translation; coord=1:180306606..180308510:1; parent_transcript=GRMZM2G161335_T01; parent_gene=GRMZM2G161335	GRMZM2G161335_P01	TRUE	TRUE	gIGIGVDVDETASPR	95%	n+304 (+304)	66.56	25.00
2282	seq=translation; coord=1:180306606..180308510:1; parent_transcript=GRMZM2G161335_T01; parent_gene=GRMZM2G161335	GRMZM2G161335_P01	TRUE	TRUE	gLAITVFHSGALDPADYPADYR	95%	n+304 (+304)	49.23	25.00
2283	seq=translation; coord=1:180306606..180308510:1; parent_transcript=GRMZM2G161335_T01; parent_gene=GRMZM2G161335	GRMZM2G161335_P01	TRUE	TRUE	tDLTDLVDLIk	95%	n+304 (+304), K+304 (+304)	37.44	25.80
2284	seq=translation; coord=1:180306606..180308510:1; parent_transcript=GRMZM2G161335_T01; parent_gene=GRMZM2G161335	GRMZM2G161335_P01	TRUE	TRUE	vDTSDLEEFAELLAR	95%	n+304 (+304)	74.65	25.00
2285	seq=translation; coord=1:180306606..180308510:1; parent_transcript=GRMZM2G161335_T01; parent_gene=GRMZM2G161335	GRMZM2G161335_P01	TRUE	TRUE	vGTELVGEQLER	92%	n+304 (+304)	29.00	25.30
2286	seq=translation; coord=1:203587258..203590599:-1; parent_transcript=GRMZM2G106928_T01; parent_gene=GRMZM2G106928	GRMZM2G106928_P01	TRUE	TRUE	aFVVHELEDDLGk	95%	n+304 (+304), K+304 (+304)	33.36	26.33
2287	seq=translation; coord=1:203587258..203590599:-1; parent_transcript=GRMZM2G106928_T01; parent_gene=GRMZM2G106928	GRMZM2G106928_P01	TRUE	TRUE	gASEVEGVVTLTQDDDGPTTVNVR	91%	n+304 (+304)	27.72	25.00
2288	seq=translation; coord=1:203587258..203590599:-1; parent_transcript=GRMZM2G106928_T01; parent_gene=GRMZM2G106928	GRMZM2G106928_P01	TRUE	TRUE	hAGDLGNIVANAEGIAEATIVDTQIPLTGPNSVVGR	95%	n+304 (+304)	33.49	25.00

2289	seq=translation; coord=1:203587258..203590599:-1; parent_transcript=GRMZM2G106928_T01; parent_gene=GRMZM2G106928	GRMZM2G106928_P01	TRUE	TRUE	lAcGVVGLTPL	95%	n+304 (+304), Carbamidomethyl (+57)	72.81	27.13
2290	seq=translation; coord=7:160032302..160036034:1; parent_transcript=GRMZM2G083243_T01; parent_gene=GRMZM2G083243	GRMZM2G083243_P01,GRMZM2G099167_P01	TRUE	TRUE	aYHEQLSVPEITNAVFEPSSMMAk	95%	n+304 (+304), K+304 (+304)	26.47	25.00
2291	seq=translation; coord=7:160032302..160036034:1; parent_transcript=GRMZM2G083243_T01; parent_gene=GRMZM2G083243	GRMZM2G083243_P01,GRMZM2G099167_P01	TRUE	TRUE	lISQIISSLTTSLR	95%	n+304 (+304)	34.42	25.00
2292	seq=translation; coord=7:160032302..160036034:1; parent_transcript=GRMZM2G083243_T01; parent_gene=GRMZM2G083243	GRMZM2G083243_P01,GRMZM2G099167_P01	TRUE	TRUE	tVQFVDWcPTGFk	95%	n+304 (+304), Carbamidomethyl (+57), K+304 (+304)	35.77	25.00
2293	seq=translation; coord=3:24095213..24095931:-1; parent_transcript=GRMZM2G011523_T01; parent_gene=GRMZM2G011523	GRMZM2G011523_P01,GRMZM2G011523_P02, GRMZM2G011523_P04	TRUE	TRUE	dFLPENcGck	95%	n+304 (+304), Carbamidomethyl (+57), Carbamidomethyl (+57), K+304 (+304)	30.48	25.00
2294	seq=translation; coord=3:24095213..24095931:-1; parent_transcript=GRMZM2G011523_T01; parent_gene=GRMZM2G011523	GRMZM2G011523_P01,GRMZM2G011523_P02, GRMZM2G011523_P04	TRUE	TRUE	ncVPVSTDk	90%	n+304 (+304), Carbamidomethyl (+57), K+304 (+304)	27.44	25.00
2295	seq=translation; coord=3:24095213..24095931:-1; parent_transcript=GRMZM2G011523_T01; parent_gene=GRMZM2G011523	GRMZM2G011523_P01,GRMZM2G011523_P02, GRMZM2G011523_P04	TRUE	TRUE	qcWcGEcTSWSGVWTcDDLLTk	95%	n+304 (+304), Carbamidomethyl (+57), Carbamidomethyl (+57), Carbamidomethyl (+57), Carbamidomethyl (+57), K+304 (+304)	46.79	25.00
2296	seq=translation; coord=6:161966268..161967646:1; parent_transcript=GRMZM2G096475_T01; parent_gene=GRMZM2G096475	GRMZM2G096475_P01	TRUE	TRUE	aADAAGHAAGk	89%	n+304 (+304), K+304 (+304)	26.08	25.91
2297	seq=translation; coord=6:161966268..161967646:1; parent_transcript=GRMZM2G096475_T01; parent_gene=GRMZM2G096475	GRMZM2G096475_P01	TRUE	TRUE	aADAMEAAk	95%	n+304 (+304), K+304 (+304)	49.35	25.00
2298	seq=translation; coord=6:161966268..161967646:1; parent_transcript=GRMZM2G096475_T01; parent_gene=GRMZM2G096475	GRMZM2G096475_P01	TRUE	TRUE	aAEAGQYAk	95%	n+304 (+304), K+304 (+304)	52.66	26.09
2299	seq=translation; coord=6:161966268..161967646:1; parent_transcript=GRMZM2G096475_T01; parent_gene=GRMZM2G096475	GRMZM2G096475_P01	TRUE	TRUE	aGETTEAAk	92%	n+304 (+304), K+304 (+304)	27.03	25.59
2300	seq=translation; coord=6:161966268..161967646:1; parent_transcript=GRMZM2G096475_T01; parent_gene=GRMZM2G096475	GRMZM2G096475_P01	TRUE	TRUE	aSDTGSYLGk	95%	n+304 (+304), K+304 (+304)	55.84	25.54
2301	seq=translation; coord=6:161966268..161967646:1; parent_transcript=GRMZM2G096475_T01; parent_gene=GRMZM2G096475	GRMZM2G096475_P01	TRUE	TRUE	aSYQAGETk	95%	n+304 (+304), K+304 (+304)	37.63	26.39
2302	seq=translation; coord=6:161966268..161967646:1; parent_transcript=GRMZM2G096475_T01; parent_gene=GRMZM2G096475	GRMZM2G096475_P01	TRUE	TRUE	dAVMSTLGMGGDDk	95%	n+304 (+304), K+304 (+304)	61.04	25.00
2303	seq=translation; coord=6:161966268..161967646:1; parent_transcript=GRMZM2G096475_T01; parent_gene=GRMZM2G096475	GRMZM2G096475_P01	TRUE	TRUE	dAVMSTLGmGGDDk	95%	n+304 (+304), Oxidation (+16), K+304 (+304)	38.64	25.00
2304	seq=translation; coord=6:161966268..161967646:1; parent_transcript=GRMZM2G096475_T01; parent_gene=GRMZM2G096475	GRMZM2G096475_P01	TRUE	TRUE	sGGVIQQATEQVk	95%	n+304 (+304), K+304 (+304)	43.42	26.18

2305	seq=translation; coord=6:161966268..161967646:1; parent_transcript=GRMZM2G096475_T01; parent_gene=GRMZM2G096475	GRMZM2G096475_P01	TRUE	TRUE	tGQAVGATk	95%	n+304 (+304), K+304 (+304)	45.84	27.63
2306	seq=translation; coord=2:86767115..86790532:1; parent_transcript=GRMZM2G028313_T01; parent_gene=GRMZM2G028313	GRMZM2G028313_P01,GRMZM2G028313_P03	TRUE	TRUE	dVQFLPISGLVGTNMk	92%	n+304 (+304), K+304 (+304)	27.00	25.11
2307	seq=translation; coord=2:86767115..86790532:1; parent_transcript=GRMZM2G028313_T01; parent_gene=GRMZM2G028313	GRMZM2G028313_P01,GRMZM2G028313_P03	TRUE	TRUE	eGDSLLIMPNk	95%	n+304 (+304), K+304 (+304)	31.29	25.53
2308	seq=translation; coord=2:86767115..86790532:1; parent_transcript=GRMZM2G028313_T01; parent_gene=GRMZM2G028313	GRMZM2G028313_P01,GRMZM2G028313_P03	TRUE	TRUE	eIQSSLQSLELk	95%	n+304 (+304), K+304 (+304)	40.53	25.84
2309	seq=translation; coord=2:86767115..86790532:1; parent_transcript=GRMZM2G028313_T01; parent_gene=GRMZM2G028313	GRMZM2G028313_P01,GRMZM2G028313_P03	TRUE	TRUE	fTILDAPGHk	95%	n+304 (+304), K+304 (+304)	56.94	25.16
2310	seq=translation; coord=2:86767115..86790532:1; parent_transcript=GRMZM2G028313_T01; parent_gene=GRMZM2G028313	GRMZM2G028313_P01,GRMZM2G028313_P03	TRUE	TRUE	hLNVVFIGHVDAGk	95%	n+304 (+304), K+304 (+304)	39.80	25.20
2311	seq=translation; coord=2:86767115..86790532:1; parent_transcript=GRMZM2G028313_T01; parent_gene=GRMZM2G028313	GRMZM2G028313_P01,GRMZM2G028313_P03	TRUE	TRUE	iQVNNLIcIEk	92%	n+304 (+304), Carbamidomethyl (+57), K+304 (+304)	26.67	25.21
2312	seq=translation; coord=2:86767115..86790532:1; parent_transcript=GRMZM2G028313_T01; parent_gene=GRMZM2G028313	GRMZM2G028313_P01,GRMZM2G028313_P03	TRUE	TRUE	lVVVINk	93%	n+304 (+304), K+304 (+304)	26.83	25.00
2313	seq=translation; coord=2:86767115..86790532:1; parent_transcript=GRMZM2G028313_T01; parent_gene=GRMZM2G028313	GRMZM2G028313_P01,GRMZM2G028313_P03	TRUE	TRUE	sIcSWWDGPcLFEVLDR	95%	n+304 (+304), Carbamidomethyl (+57), Carbamidomethyl (+57)	61.82	25.00
2314	seq=translation; coord=2:86767115..86790532:1; parent_transcript=GRMZM2G028313_T01; parent_gene=GRMZM2G028313	GRMZM2G028313_P01,GRMZM2G028313_P03	TRUE	TRUE	sTTGGQILFLSGQVDDR	95%	n+304 (+304)	83.47	25.00
2315	seq=translation; coord=2:86767115..86790532:1; parent_transcript=GRMZM2G028313_T01; parent_gene=GRMZM2G028313	GRMZM2G028313_P01,GRMZM2G028313_P03	TRUE	TRUE	vIGLNLDESk	95%	n+304 (+304), K+304 (+304)	43.74	26.28
2316	seq=translation; coord=1:94680007..94684730:-1; parent_transcript=GRMZM2G178576_T02; parent_gene=GRMZM2G178576	GRMZM2G178576_P02,GRMZM2G178576_P03	TRUE	TRUE	eIALWFPEGPADWQSSQHPWIYEk	95%	n+304 (+304), K+304 (+304)	29.82	25.00
2317	seq=translation; coord=1:94680007..94684730:-1; parent_transcript=GRMZM2G178576_T02; parent_gene=GRMZM2G178576	GRMZM2G178576_P02,GRMZM2G178576_P03	TRUE	TRUE	gDFAVDIGR	95%	n+304 (+304)	39.05	25.00
2318	seq=translation; coord=1:94680007..94684730:-1; parent_transcript=GRMZM2G178576_T02; parent_gene=GRMZM2G178576	GRMZM2G178576_P02,GRMZM2G178576_P03	TRUE	TRUE	gLIGEIISR	95%	n+304 (+304)	34.71	25.00
2319	seq=translation; coord=1:94680007..94684730:-1; parent_transcript=GRMZM2G178576_T02; parent_gene=GRMZM2G178576	GRMZM2G178576_P02,GRMZM2G178576_P03	TRUE	TRUE	iIGATNPLASEPGTIR	95%	n+304 (+304)	59.34	26.54
2320	seq=translation; coord=1:94680007..94684730:-1; parent_transcript=GRMZM2G178576_T02; parent_gene=GRMZM2G178576	GRMZM2G178576_P02,GRMZM2G178576_P03	TRUE	TRUE	kIIGATNPLASEPGTIR	95%	K+304 (+304), n+304 (+304)	51.16	25.00
2321	seq=translation; coord=10:75695843..75698244:1; parent_transcript=GRMZM2G099352_T03; parent_gene=GRMZM2G099352	GRMZM2G099352_P03	TRUE	TRUE	dEDEPRPPVLAPPEV	95%	n+304 (+304)	48.04	25.00
2322	seq=translation; coord=10:75695843..75698244:1; parent_transcript=GRMZM2G099352_T03; parent_gene=GRMZM2G099352	GRMZM2G099352_P03	TRUE	TRUE	eLAEDGYSGVEVR	95%	n+304 (+304)	40.47	25.00

2323	seq=translation; coord=10:75695843..75698244:1; parent_transcript=GRMZM2G099352_T03; parent_gene=GRMZM2G099352	GRMZM2G099352_P03	TRUE	TRUE	fkDGYMISSGQPVNEYIDSAVR	95%	n+304 (+304), K+304 (+304)	34.57	25.04
2324	seq=translation; coord=10:75695843..75698244:1; parent_transcript=GRMZM2G099352_T03; parent_gene=GRMZM2G099352	GRMZM2G099352_P03	TRUE	TRUE	fVSDGVFYAELNEMLTR	95%	n+304 (+304)	65.24	25.00
2325	seq=translation; coord=10:75695843..75698244:1; parent_transcript=GRMZM2G099352_T03; parent_gene=GRMZM2G099352	GRMZM2G099352_P03	TRUE	TRUE	gLcAIAQAESLR	95%	n+304 (+304), Carbamidomethyl (+57)	50.56	26.09
2326	seq=translation; coord=10:75695843..75698244:1; parent_transcript=GRMZM2G099352_T03; parent_gene=GRMZM2G099352	GRMZM2G099352_P03	TRUE	TRUE	iMLDWDPk	91%	n+304 (+304), K+304 (+304)	29.05	25.89
2327	seq=translation; coord=3:19924648..19927549:1; parent_transcript=GRMZM2G169182_T01; parent_gene=GRMZM2G169182	GRMZM2G169182_P01,GRMZM2G169182_P02	TRUE	TRUE	gGDSWDEPFPSSAAAAAAAAGGGGR	95%	n+304 (+304)	80.48	25.00
2328	seq=translation; coord=3:19924648..19927549:1; parent_transcript=GRMZM2G169182_T01; parent_gene=GRMZM2G169182	GRMZM2G169182_P01,GRMZM2G169182_P02	TRUE	TRUE	lQAGAAAAAVGTGR	95%	n+304 (+304)	52.51	26.39
2329	seq=translation; coord=3:19924648..19927549:1; parent_transcript=GRMZM2G169182_T01; parent_gene=GRMZM2G169182	GRMZM2G169182_P01,GRMZM2G169182_P02	TRUE	TRUE	qDVPPVVDPTNR	95%	n+304 (+304)	41.24	25.00
2330	seq=translation; coord=3:19924648..19927549:1; parent_transcript=GRMZM2G169182_T01; parent_gene=GRMZM2G169182	GRMZM2G169182_P01,GRMZM2G169182_P02	TRUE	TRUE	sGVTNSAPTEDQR	95%	n+304 (+304)	43.01	25.00
2331	seq=translation; coord=8:168745391..168749427:1; parent_transcript=GRMZM2G146206_T04; parent_gene=GRMZM2G146206	GRMZM2G146206_P04	TRUE	TRUE	aLLGESSDFVADk	95%	n+304 (+304), K+304 (+304)	58.08	25.88
2332	seq=translation; coord=8:168745391..168749427:1; parent_transcript=GRMZM2G146206_T04; parent_gene=GRMZM2G146206	GRMZM2G146206_P04	TRUE	TRUE	eAGTTMDVVAAQTk	89%	n+304 (+304), K+304 (+304)	27.56	25.89
2333	seq=translation; coord=8:168745391..168749427:1; parent_transcript=GRMZM2G146206_T04; parent_gene=GRMZM2G146206	GRMZM2G146206_P04	TRUE	TRUE	eAGTTmDVVAAQTk	95%	n+304 (+304), Oxidation (+16), K+304 (+304)	29.71	25.44
2334	seq=translation; coord=8:168745391..168749427:1; parent_transcript=GRMZM2G146206_T04; parent_gene=GRMZM2G146206	GRMZM2G146206_P04	TRUE	TRUE	iSDWTNIVLAYEPVWAIGTGk	95%	n+304 (+304), K+304 (+304)	38.88	25.16
2335	seq=translation; coord=8:168745391..168749427:1; parent_transcript=GRMZM2G146206_T04; parent_gene=GRMZM2G146206	GRMZM2G146206_P04	TRUE	TRUE	kGGAFTGEISAEMLVNLQVPWVILGHSER	95%	K+304 (+304), n+304 (+304)	28.12	25.00
2336	seq=translation; coord=8:168745391..168749427:1; parent_transcript=GRMZM2G146206_T04; parent_gene=GRMZM2G146206	GRMZM2G146206_P04	TRUE	TRUE	vATPAQAQEVHDGLR	95%	n+304 (+304)	52.56	25.25
2337	seq=translation; coord=8:168745391..168749427:1; parent_transcript=GRMZM2G146206_T04; parent_gene=GRMZM2G146206	GRMZM2G146206_P04	TRUE	TRUE	vAYALTQGLk	95%	n+304 (+304), K+304 (+304)	28.28	25.12
2338	seq=translation; coord=8:168745391..168749427:1; parent_transcript=GRMZM2G146206_T04; parent_gene=GRMZM2G146206	GRMZM2G146206_P04	TRUE	TRUE	vIAcIGETLEQR	95%	n+304 (+304), Carbamidomethyl (+57)	43.08	25.38
2339	seq=translation; coord=8:168745391..168749427:1; parent_transcript=GRMZM2G146206_T04; parent_gene=GRMZM2G146206	GRMZM2G146206_P04	TRUE	TRUE	wLHSNVSPAVAELTR	95%	n+304 (+304)	50.87	25.49
2340	seq=translation; coord=2:48112888..48133114:1; parent_transcript=GRMZM2G053019_T01; parent_gene=GRMZM2G053019	GRMZM2G053019_P01	TRUE	TRUE	dLQSSLEMVDVk	95%	n+304 (+304), K+304 (+304)	51.45	26.28

2341	seq=translation; coord=2:48112888..48133114:1; parent_transcript=GRMZM2G053019_T01; parent_gene=GRMZM2G053019	GRMZM2G053019_P01	TRUE	TRUE	gPTESSQSGPMLSVETIEk	95%	n+304 (+304), K+304 (+304)	44.23	25.17
2342	seq=translation; coord=2:48112888..48133114:1; parent_transcript=GRMZM2G053019_T01; parent_gene=GRMZM2G053019	GRMZM2G053019_P01	TRUE	TRUE	nMGGSPDQWDNR	95%	n+304 (+304)	46.45	25.00
2343	seq=translation; coord=10:4694280..4699187:-1; parent_transcript=GRMZM2G004534_T01; parent_gene=GRMZM2G004534	GRMZM2G004534_P01	TRUE	TRUE	iENVEGLNHFDEILAEADGIILSR	95%	n+304 (+304)	43.20	25.33
2344	seq=translation; coord=10:4694280..4699187:-1; parent_transcript=GRMZM2G004534_T01; parent_gene=GRMZM2G004534	GRMZM2G004534_P01	TRUE	TRUE	lGDLSQTQIFAk	95%	n+304 (+304), K+304 (+304)	31.63	25.21
2345	seq=translation; coord=10:4694280..4699187:-1; parent_transcript=GRMZM2G004534_T01; parent_gene=GRMZM2G004534	GRMZM2G004534_P01	TRUE	TRUE	yRPSMPVLSVVIPR	95%	n+304 (+304)	32.02	25.00
2346	seq=translation; coord=4:37224780..37227724:1; parent_transcript=GRMZM2G064133_T01; parent_gene=GRMZM2G064133	GRMZM2G064133_P01	TRUE	TRUE	aEESAASGDPLAMASk	94%	n+304 (+304), K+304 (+304)	27.98	25.00
2347	seq=translation; coord=4:37224780..37227724:1; parent_transcript=GRMZM2G064133_T01; parent_gene=GRMZM2G064133	GRMZM2G064133_P01	TRUE	TRUE	ePDLLELFR	90%	n+304 (+304)	28.33	25.97
2348	seq=translation; coord=4:37224780..37227724:1; parent_transcript=GRMZM2G064133_T01; parent_gene=GRMZM2G064133	GRMZM2G064133_P01	TRUE	TRUE	gFGFVNFVHR	95%	n+304 (+304)	37.82	25.00
2349	seq=translation; coord=4:37224780..37227724:1; parent_transcript=GRMZM2G064133_T01; parent_gene=GRMZM2G064133	GRMZM2G064133_P01	TRUE	TRUE	gGAVLMVcR	86%	n+304 (+304), Carbamidomethyl (+57)	25.30	25.00
2350	seq=translation; coord=4:37224780..37227724:1; parent_transcript=GRMZM2G064133_T01; parent_gene=GRMZM2G064133	GRMZM2G064133_P01	TRUE	TRUE	lTMVSTEEILLERPR	95%	n+304 (+304)	37.31	25.80
2351	seq=translation; coord=4:37224780..37227724:1; parent_transcript=GRMZM2G064133_T01; parent_gene=GRMZM2G064133	GRMZM2G064133_P01	TRUE	TRUE	vEWATPRPN	95%	n+304 (+304)	35.25	25.00
2352	seq=translation; coord=4:37224780..37227724:1; parent_transcript=GRMZM2G064133_T01; parent_gene=GRMZM2G064133	GRMZM2G064133_P01	TRUE	TRUE	vTNLSEDTREPDLLELFR	91%	n+304 (+304)	26.35	25.34
2353	seq=translation; coord=4:37224780..37227724:1; parent_transcript=GRMZM2G064133_T01; parent_gene=GRMZM2G064133	GRMZM2G064133_P01	TRUE	TRUE	wGELEEDDGGDLDFLLPPR	95%	n+304 (+304)	46.81	25.00
2354	seq=translation; coord=6:90306950..90312408:-1; parent_transcript=GRMZM2G094074_T01; parent_gene=GRMZM2G094074	GRMZM2G094074_P01,GRMZM2G094074_P02, GRMZM2G100462_P02,GRMZM2G100462_P03, GRMZM2G120432_P01,GRMZM2G120432_P02, GRMZM5G805627_P02	TRUE	TRUE	aDRDESSPYAAMLAAQDVAQR	95%	n+304 (+304)	57.24	25.00
2355	seq=translation; coord=6:90306950..90312408:-1; parent_transcript=GRMZM2G094074_T01; parent_gene=GRMZM2G094074	GRMZM2G094074_P01,GRMZM2G094074_P02, GRMZM2G100462_P02,GRMZM2G100462_P03, GRMZM2G120432_P01,GRMZM2G120432_P02, GRMZM5G805627_P02	TRUE	TRUE	eENVTLGPTVR	95%	n+304 (+304)	35.08	26.45
2356	seq=translation; coord=6:90306950..90312408:-1; parent_transcript=GRMZM2G094074_T01; parent_gene=GRMZM2G094074	GRMZM2G094074_P01,GRMZM2G094074_P02, GRMZM2G100462_P02,GRMZM2G100462_P03, GRMZM2G120432_P01,GRMZM2G120432_P02, GRMZM5G805627_P02	TRUE	TRUE	eLGITALHIk	95%	n+304 (+304), K+304 (+304)	30.13	25.00

2357	seq=translation; coord=6:90306950..90312408:-1; parent_transcript=GRMZM2G094074_T01; parent_gene=GRMZM2G094074	GRMZM2G094074_P01,GRMZM2G094074_P02, GRMZM2G100462_P02,GRMZM2G100462_P03, GRMZM2G120432_P01,GRMZM2G120432_P02, GRMZM5G805627_P02	TRUE	TRUE	iEDVTPVPTDSTR	95%	n+304 (+304)	43.60	25.00
2358	seq=translation; coord=6:90306950..90312408:-1; parent_transcript=GRMZM2G094074_T01; parent_gene=GRMZM2G094074	GRMZM2G094074_P01,GRMZM2G094074_P02, GRMZM2G100462_P02,GRMZM2G100462_P03, GRMZM2G120432_P01,GRMZM2G120432_P02, GRMZM5G805627_P02	TRUE	TRUE	tPGPGAQSALR	93%	n+304 (+304)	30.58	25.79
2359	seq=translation; coord=7:171631430..171634129:1; parent_transcript=GRMZM2G439201_T02; parent_gene=GRMZM2G439201	GRMZM2G439201_P02	TRUE	TRUE	sSVLMDVkPWDDETDMk	95%	n+304 (+304), K+304 (+304), K+304 (+304)	35.69	25.35
2360	seq=translation; coord=7:171631430..171634129:1; parent_transcript=GRMZM2G439201_T02; parent_gene=GRMZM2G439201	GRMZM2G439201_P02	TRUE	TRUE	sVQMEGLTWGASk	95%	n+304 (+304), K+304 (+304)	37.60	26.09
2361	seq=translation; coord=7:171631430..171634129:1; parent_transcript=GRMZM2G439201_T02; parent_gene=GRMZM2G439201	GRMZM2G439201_P02	TRUE	TRUE	tYVSGDSITk	95%	n+304 (+304), K+304 (+304)	49.78	25.91
2362	seq=translation; coord=7:171631430..171634129:1; parent_transcript=GRMZM2G439201_T02; parent_gene=GRMZM2G439201	GRMZM2G439201_P02	TRUE	TRUE	vFAAVPSkPGAEFPNAAR	95%	n+304 (+304), K+304 (+304)	31.46	25.00
2363	seq=translation; coord=7:171631430..171634129:1; parent_transcript=GRMZM2G439201_T02; parent_gene=GRMZM2G439201	GRMZM2G439201_P02	TRUE	TRUE	wYETVSAAVASR	93%	n+304 (+304)	29.87	25.00
2364	seq=translation; coord=8:100394854..100398658:-1; parent_transcript=GRMZM2G126010_T01; parent_gene=GRMZM2G126010	GRMZM2G126010_P01,GRMZM2G126010_P02, GRMZM2G126010_P03	TRUE	TRUE	dAYVGDEAQAk	95%	n+304 (+304), K+304 (+304)	67.82	25.00
2365	seq=translation; coord=8:100394854..100398658:-1; parent_transcript=GRMZM2G126010_T01; parent_gene=GRMZM2G126010	GRMZM2G126010_P01,GRMZM2G126010_P02, GRMZM2G126010_P03	TRUE	TRUE	gYSLTTSAER	95%	n+304 (+304)	31.68	25.00
2366	seq=translation; coord=8:100394854..100398658:-1; parent_transcript=GRMZM2G126010_T01; parent_gene=GRMZM2G126010	GRMZM2G126010_P01,GRMZM2G126010_P02, GRMZM2G126010_P03	TRUE	TRUE	lAYVALDYEQELETAR	95%	n+304 (+304)	70.07	25.00
2367	seq=translation; coord=8:100394854..100398658:-1; parent_transcript=GRMZM2G126010_T01; parent_gene=GRMZM2G126010	GRMZM2G126010_P01,GRMZM2G126010_P02, GRMZM2G126010_P03	TRUE	TRUE	lAyVALDYEQELETAR	95%	n+304 (+304), iTRAQ8plex (+304)	45.45	25.13
2368	seq=translation; coord=8:100394854..100398658:-1; parent_transcript=GRMZM2G126010_T01; parent_gene=GRMZM2G126010	GRMZM2G126010_P01,GRMZM2G126010_P02, GRMZM2G126010_P03	TRUE	TRUE	sYEMPDGQVITIGSER	95%	n+304 (+304)	54.00	25.00
2369	seq=translation; coord=8:100394854..100398658:-1; parent_transcript=GRMZM2G126010_T01; parent_gene=GRMZM2G126010	GRMZM2G126010_P01,GRMZM2G126010_P02, GRMZM2G126010_P03	TRUE	TRUE	syEMPDGQVITIGSER	95%	n+304 (+304), iTRAQ8plex (+304)	47.12	25.00
2370	seq=translation; coord=1:198230317..198233618:-1; parent_transcript=GRMZM2G017110_T02; parent_gene=GRMZM2G017110	GRMZM2G017110_P02,GRMZM2G017110_P03	TRUE	TRUE	dAGVPLVAFSLR	95%	n+304 (+304)	47.86	25.00
2371	seq=translation; coord=1:198230317..198233618:-1; parent_transcript=GRMZM2G017110_T02; parent_gene=GRMZM2G017110	GRMZM2G017110_P02,GRMZM2G017110_P03	TRUE	TRUE	eAAQQIISDELMLDGNPR	95%	n+304 (+304)	80.51	25.00
2372	seq=translation; coord=1:198230317..198233618:-1; parent_transcript=GRMZM2G017110_T02; parent_gene=GRMZM2G017110	GRMZM2G017110_P02,GRMZM2G017110_P03	TRUE	TRUE	fGWIVPAYTMPADAEHVAVLR	95%	n+304 (+304)	65.29	25.04
2373	seq=translation; coord=1:198230317..198233618:-1; parent_transcript=GRMZM2G017110_T02; parent_gene=GRMZM2G017110	GRMZM2G017110_P02,GRMZM2G017110_P03	TRUE	TRUE	fSVFDVSENLR	95%	n+304 (+304)	41.67	25.00

2374	seq=translation; coord=1:198230317..198233618:-1; parent_transcript=GRMZM2G017110_T02; parent_gene=GRMZM2G017110	GRMZM2G017110_P02,GRMZM2G017110_P03	TRUE	TRUE	gScQIIAQYYQLIR	95%	n+304 (+304), Carbamidomethyl (+57)	46.71	25.98
2375	seq=translation; coord=1:198230317..198233618:-1; parent_transcript=GRMZM2G017110_T02; parent_gene=GRMZM2G017110	GRMZM2G017110_P02,GRMZM2G017110_P03	TRUE	TRUE	nIMENcQENAAILR	95%	n+304 (+304), Carbamidomethyl (+57)	58.78	25.00
2376	seq=translation; coord=1:56160677..56162858:-1; parent_transcript=GRMZM2G054916_T01; parent_gene=GRMZM2G054916	GRMZM2G054916_P01	TRUE	TRUE	aGPFEFFGFTTSAR	95%	n+304 (+304)	35.61	25.00
2377	seq=translation; coord=1:56160677..56162858:-1; parent_transcript=GRMZM2G054916_T01; parent_gene=GRMZM2G054916	GRMZM2G054916_P01	TRUE	TRUE	eGLMHIGFITMEPk	94%	n+304 (+304), K+304 (+304)	29.26	25.53
2378	seq=translation; coord=1:56160677..56162858:-1; parent_transcript=GRMZM2G054916_T01; parent_gene=GRMZM2G054916	GRMZM2G054916_P01	TRUE	TRUE	gLFLLHR	95%	n+304 (+304)	36.43	27.04
2379	seq=translation; coord=1:56160677..56162858:-1; parent_transcript=GRMZM2G054916_T01; parent_gene=GRMZM2G054916	GRMZM2G054916_P01	TRUE	TRUE	mGDVLHIDAGSTFYMVNPGk	95%	n+304 (+304), K+304 (+304)	32.03	25.00
2380	seq=translation; coord=1:56160677..56162858:-1; parent_transcript=GRMZM2G054916_T01; parent_gene=GRMZM2G054916	GRMZM2G054916_P01	TRUE	TRUE	nRPQFLVGASSVLR	95%	n+304 (+304)	44.56	25.02
2381	seq=translation; coord=1:56160677..56162858:-1; parent_transcript=GRMZM2G054916_T01; parent_gene=GRMZM2G054916	GRMZM2G054916_P01	TRUE	TRUE	tLFVPQYLDSSITLFVQR	95%	n+304 (+304)	64.76	25.53
2382	seq=translation; coord=1:56160677..56162858:-1; parent_transcript=GRMZM2G054916_T01; parent_gene=GRMZM2G054916	GRMZM2G054916_P01	TRUE	TRUE	tMLGPEIAAAFGAR	95%	n+304 (+304)	49.36	25.54
2383	seq=translation; coord=1:56160677..56162858:-1; parent_transcript=GRMZM2G054916_T01; parent_gene=GRMZM2G054916	GRMZM2G054916_P01	TRUE	TRUE	vVESEGGQVR	88%	n+304 (+304)	26.70	25.59
2384	seq=translation; coord=4:26893142..26901172:1; parent_transcript=GRMZM2G093900_T01; parent_gene=GRMZM2G093900	GRMZM2G093900_P01	TRUE	TRUE	aFSGSPIVGEIR	95%	n+304 (+304)	44.46	25.04
2385	seq=translation; coord=4:26893142..26901172:1; parent_transcript=GRMZM2G093900_T01; parent_gene=GRMZM2G093900	GRMZM2G093900_P01	TRUE	TRUE	eGPETIAAFIAEPVMGAGGVILPPk	93%	n+304 (+304), K+304 (+304)	26.39	25.00
2386	seq=translation; coord=4:26893142..26901172:1; parent_transcript=GRMZM2G093900_T01; parent_gene=GRMZM2G093900	GRMZM2G093900_P01	TRUE	TRUE	gHSMLAPFTAGWQSTDVHPLVIER	95%	n+304 (+304)	56.87	25.00
2387	seq=translation; coord=4:26893142..26901172:1; parent_transcript=GRMZM2G093900_T01; parent_gene=GRMZM2G093900	GRMZM2G093900_P01	TRUE	TRUE	gLGLILGTEFVDNk	95%	n+304 (+304), K+304 (+304)	29.58	25.00
2388	seq=translation; coord=4:26893142..26901172:1; parent_transcript=GRMZM2G093900_T01; parent_gene=GRMZM2G093900	GRMZM2G093900_P01	TRUE	TRUE	kYDILLIADEVITAFGR	95%	K+304 (+304), n+304 (+304)	27.40	25.00
2389	seq=translation; coord=4:26893142..26901172:1; parent_transcript=GRMZM2G093900_T01; parent_gene=GRMZM2G093900	GRMZM2G093900_P01	TRUE	TRUE	lPDETEEEFSTR	95%	n+304 (+304)	40.16	25.00
2390	seq=translation; coord=4:26893142..26901172:1; parent_transcript=GRMZM2G093900_T01; parent_gene=GRMZM2G093900	GRMZM2G093900_P01	TRUE	TRUE	sPNDPFPAEWGVGSIFGAEcEk	95%	n+304 (+304), Carbamidomethyl (+57), K+304 (+304)	43.24	25.00
2391	seq=translation; coord=4:26893142..26901172:1; parent_transcript=GRMZM2G093900_T01; parent_gene=GRMZM2G093900	GRMZM2G093900_P01	TRUE	TRUE	tTkPSLDLANDILSMFTAR	95%	n+304 (+304), K+304 (+304)	29.04	25.00

2392	seq=translation; coord=4:26893142..26901172:1; parent_transcript=GRMZM2G093900_T01; parent_gene=GRMZM2G093900	GRMZM2G093900_P01	TRUE	TRUE	vAGDSIMLSPPLIMTPNEVEEIISk	95%	n+304 (+304), K+304 (+304)	29.75	25.02
2393	seq=translation; coord=8:37409326..37412866:1; parent_transcript=GRMZM2G002416_T01; parent_gene=GRMZM2G002416	GRMZM2G002416_P01	TRUE	TRUE	aGkDLVSSLVSGLLTIGPR	95%	n+304 (+304), K+304 (+304)	43.59	25.00
2394	seq=translation; coord=8:37409326..37412866:1; parent_transcript=GRMZM2G002416_T01; parent_gene=GRMZM2G002416	GRMZM2G002416_P01	TRUE	TRUE	dLVSSLVSGLLTIGPR	95%	n+304 (+304)	42.26	25.00
2395	seq=translation; coord=8:37409326..37412866:1; parent_transcript=GRMZM2G002416_T01; parent_gene=GRMZM2G002416	GRMZM2G002416_P01	TRUE	TRUE	fGGAIDDAAR	95%	n+304 (+304)	40.35	25.00
2396	seq=translation; coord=8:37409326..37412866:1; parent_transcript=GRMZM2G002416_T01; parent_gene=GRMZM2G002416	GRMZM2G002416_P01	TRUE	TRUE	mMVVLGELGGkDEYSLVEALk	95%	n+304 (+304), K+304 (+304), K+304 (+304)	38.16	25.00
2397	seq=translation; coord=8:37409326..37412866:1; parent_transcript=GRMZM2G002416_T01; parent_gene=GRMZM2G002416	GRMZM2G002416_P01	TRUE	TRUE	sGGELESAQAk	94%	n+304 (+304), K+304 (+304)	28.12	25.43
2398	seq=translation; coord=8:37409326..37412866:1; parent_transcript=GRMZM2G002416_T01; parent_gene=GRMZM2G002416	GRMZM2G002416_P01	TRUE	TRUE	vQkPVVAWVSGTcAR	90%	n+304 (+304), K+304 (+304), Carbamidomethyl (+57)	26.48	25.79
2399	seq=translation; coord=2:58586007..58596234:1; parent_transcript=GRMZM2G073054_T01; parent_gene=GRMZM2G073054	GRMZM2G073054_P01,GRMZM2G073054_P02	TRUE	TRUE	aAIDQHEGGLDAFSR	95%	n+304 (+304)	33.01	25.00
2400	seq=translation; coord=2:58586007..58596234:1; parent_transcript=GRMZM2G073054_T01; parent_gene=GRMZM2G073054	GRMZM2G073054_P01,GRMZM2G073054_P02	TRUE	TRUE	aPLVEEkPR	90%	n+304 (+304), K+304 (+304)	25.54	25.00
2401	seq=translation; coord=2:58586007..58596234:1; parent_transcript=GRMZM2G073054_T01; parent_gene=GRMZM2G073054	GRMZM2G073054_P01,GRMZM2G073054_P02	TRUE	TRUE	gMQEFDQAMQHLEGk	95%	n+304 (+304), K+304 (+304)	31.07	25.00
2402	seq=translation; coord=2:58586007..58596234:1; parent_transcript=GRMZM2G073054_T01; parent_gene=GRMZM2G073054	GRMZM2G073054_P01,GRMZM2G073054_P02	TRUE	TRUE	iVLDSDDGLFGGFSR	95%	n+304 (+304)	48.64	25.00
2403	seq=translation; coord=2:58586007..58596234:1; parent_transcript=GRMZM2G073054_T01; parent_gene=GRMZM2G073054	GRMZM2G073054_P01,GRMZM2G073054_P02	TRUE	TRUE	iYEIDPMLEGFR	95%	n+304 (+304)	55.37	25.00
2404	seq=translation; coord=2:58586007..58596234:1; parent_transcript=GRMZM2G073054_T01; parent_gene=GRMZM2G073054	GRMZM2G073054_P01,GRMZM2G073054_P02	TRUE	TRUE	qSDEYWEMGDIVHTLTNR	95%	n+304 (+304)	61.18	25.00
2405	seq=translation; coord=2:58586007..58596234:1; parent_transcript=GRMZM2G073054_T01; parent_gene=GRMZM2G073054	GRMZM2G073054_P01,GRMZM2G073054_P02	TRUE	TRUE	vIIFER	90%	n+304 (+304)	27.44	25.00
2406	seq=translation; coord=2:58586007..58596234:1; parent_transcript=GRMZM2G073054_T01; parent_gene=GRMZM2G073054	GRMZM2G073054_P01,GRMZM2G073054_P02	TRUE	TRUE	vIPPPGDGQR	95%	n+304 (+304)	31.48	25.00
2407	seq=translation; coord=1:2801080..2803977:-1; parent_transcript=GRMZM2G041881_T01; parent_gene=GRMZM2G041881	GRMZM2G041881_P01	TRUE	TRUE	dDIVIQFQNPk	95%	n+304 (+304), K+304 (+304)	48.95	25.76
2408	seq=translation; coord=1:2801080..2803977:-1; parent_transcript=GRMZM2G041881_T01; parent_gene=GRMZM2G041881	GRMZM2G041881_P01	TRUE	TRUE	eVPEAEPEEk	90%	n+304 (+304), K+304 (+304)	27.70	25.53
2409	seq=translation; coord=1:2801080..2803977:-1; parent_transcript=GRMZM2G041881_T01; parent_gene=GRMZM2G041881	GRMZM2G041881_P01	TRUE	TRUE	sLQDLLPTIINQLGPDNLENLk	95%	n+304 (+304), K+304 (+304)	39.55	25.00

2410	seq=translation; coord=1:2801080..2803977:-1; parent_transcript=GRMZM2G041881_T01; parent_gene=GRMZM2G041881	GRMZM2G041881_P01	TRUE	TRUE	sLQDLLPTIINQLGPDNLENLkR	92%	n+304 (+304), K+304 (+304)	26.85	25.00
2411	seq=translation; coord=1:2801080..2803977:-1; parent_transcript=GRMZM2G041881_T01; parent_gene=GRMZM2G041881	GRMZM2G041881_P01	TRUE	TRUE	vQASIPANTWVVSGVPQTk	93%	n+304 (+304), K+304 (+304)	27.64	25.11
2412	seq=translation; coord=10:110621503..110626726:1; parent_transcript=GRMZM2G079538_T01; parent_gene=GRMZM2G079538	GRMZM2G079538_P01,GRMZM2G079538_P02, GRMZM2G079538_P03,GRMZM5G807639_P03	TRUE	TRUE	dADTMNFADIEk	95%	n+304 (+304), K+304 (+304)	44.07	25.00
2413	seq=translation; coord=10:110621503..110626726:1; parent_transcript=GRMZM2G079538_T01; parent_gene=GRMZM2G079538	GRMZM2G079538_P01,GRMZM2G079538_P02, GRMZM2G079538_P03,GRMZM5G807639_P03	TRUE	TRUE	dSQNTFAMLSTFNEVDMTNLMk	95%	n+304 (+304), K+304 (+304)	54.82	25.00
2414	seq=translation; coord=10:110621503..110626726:1; parent_transcript=GRMZM2G079538_T01; parent_gene=GRMZM2G079538	GRMZM2G079538_P01,GRMZM2G079538_P02, GRMZM2G079538_P03,GRMZM5G807639_P03	TRUE	TRUE	gLVVPVIR	87%	n+304 (+304)	25.80	25.00
2415	seq=translation; coord=10:110621503..110626726:1; parent_transcript=GRMZM2G079538_T01; parent_gene=GRMZM2G079538	GRMZM2G079538_P01,GRMZM2G079538_P02, GRMZM2G079538_P03,GRMZM5G807639_P03	TRUE	TRUE	lGLMScFVk	93%	n+304 (+304), Carbamidomethyl (+57), K+304 (+304)	27.56	25.00
2416	seq=translation; coord=10:110621503..110626726:1; parent_transcript=GRMZM2G079538_T01; parent_gene=GRMZM2G079538	GRMZM2G079538_P01,GRMZM2G079538_P02, GRMZM2G079538_P03,GRMZM5G807639_P03	TRUE	TRUE	lIASEGDTVTPGTk	95%	n+304 (+304), K+304 (+304)	51.97	26.02
2417	seq=translation; coord=10:110621503..110626726:1; parent_transcript=GRMZM2G079538_T01; parent_gene=GRMZM2G079538	GRMZM2G079538_P01,GRMZM2G079538_P02, GRMZM2G079538_P03,GRMZM5G807639_P03	TRUE	TRUE	lLLDI	90%	n+304 (+304)	29.99	28.69
2418	seq=translation; coord=10:110621503..110626726:1; parent_transcript=GRMZM2G079538_T01; parent_gene=GRMZM2G079538	GRMZM2G079538_P01,GRMZM2G079538_P02, GRMZM2G079538_P03,GRMZM5G807639_P03	TRUE	TRUE	tSPSEPQLPPk	95%	n+304 (+304), K+304 (+304)	36.61	26.11
2419	seq=translation; coord=8:9901033..9904862:-1; parent_transcript=GRMZM2G069195_T01; parent_gene=GRMZM2G069195	GRMZM2G069195_P01	TRUE	TRUE	aAAGSYSDELVSTAk	95%	n+304 (+304), K+304 (+304)	48.74	25.00
2420	seq=translation; coord=8:9901033..9904862:-1; parent_transcript=GRMZM2G069195_T01; parent_gene=GRMZM2G069195	GRMZM2G069195_P01	TRUE	TRUE	lSSIGLDNTEPNR	95%	n+304 (+304)	44.82	26.01
2421	seq=translation; coord=8:9901033..9904862:-1; parent_transcript=GRMZM2G069195_T01; parent_gene=GRMZM2G069195	GRMZM2G069195_P01	TRUE	TRUE	yTGEGESDEAk	95%	n+304 (+304), K+304 (+304)	53.49	25.00
2422	seq=translation; coord=1:56785163..56792250:-1; parent_transcript=GRMZM2G318780_T02; parent_gene=GRMZM2G318780	GRMZM2G318780_P02	TRUE	TRUE	aLAEGPFLDVLR	95%	n+304 (+304)	34.61	25.53
2423	seq=translation; coord=1:56785163..56792250:-1; parent_transcript=GRMZM2G318780_T02; parent_gene=GRMZM2G318780	GRMZM2G318780_P02	TRUE	TRUE	aLENEMVLR	88%	n+304 (+304)	26.13	25.69
2424	seq=translation; coord=1:56785163..56792250:-1; parent_transcript=GRMZM2G318780_T02; parent_gene=GRMZM2G318780	GRMZM2G318780_P02	TRUE	TRUE	dcLEPLLDFLR	94%	n+304 (+304), Carbamidomethyl (+57)	28.45	25.00
2425	seq=translation; coord=1:56785163..56792250:-1; parent_transcript=GRMZM2G318780_T02; parent_gene=GRMZM2G318780	GRMZM2G318780_P02	TRUE	TRUE	fNIVSPGADMSIYFPHTEk	95%	n+304 (+304), K+304 (+304)	56.98	25.00
2426	seq=translation; coord=1:56785163..56792250:-1; parent_transcript=GRMZM2G318780_T02; parent_gene=GRMZM2G318780	GRMZM2G318780_P02	TRUE	TRUE	gILQPHHILDALDEVQGSGGR	95%	n+304 (+304)	47.48	25.40
2427	seq=translation; coord=1:56785163..56792250:-1; parent_transcript=GRMZM2G318780_T02; parent_gene=GRMZM2G318780	GRMZM2G318780_P02	TRUE	TRUE	lMTLAGVYGFWk	95%	n+304 (+304), K+304 (+304)	34.76	25.39

2428	seq=translation; coord=1:56785163..56792250:-1; parent_transcript=GRMZM2G318780_T02; parent_gene=GRMZM2G318780	GRMZM2G318780_P02	TRUE	TRUE	lPADTPYSQFAYk	95%	n+304 (+304), K+304 (+304)	35.29	25.17
2429	seq=translation; coord=1:56785163..56792250:-1; parent_transcript=GRMZM2G318780_T02; parent_gene=GRMZM2G318780	GRMZM2G318780_P02	TRUE	TRUE	nELVALLSk	95%	n+304 (+304), K+304 (+304)	45.27	25.00
2430	seq=translation; coord=1:56785163..56792250:-1; parent_transcript=GRMZM2G318780_T02; parent_gene=GRMZM2G318780	GRMZM2G318780_P02	TRUE	TRUE	nITGLVEAFAk	95%	n+304 (+304), K+304 (+304)	34.69	25.00
2431	seq=translation; coord=1:56785163..56792250:-1; parent_transcript=GRMZM2G318780_T02; parent_gene=GRMZM2G318780	GRMZM2G318780_P02	TRUE	TRUE	nTVGQYESHTAFTLPGLYR	95%	n+304 (+304)	57.94	25.00
2432	seq=translation; coord=1:222203896..222206239:-1; parent_transcript=GRMZM2G320269_T01; parent_gene=GRMZM2G320269	GRMZM2G320269_P01	TRUE	TRUE	aLFHSDEALLR	95%	n+304 (+304)	32.39	26.51
2433	seq=translation; coord=1:222203896..222206239:-1; parent_transcript=GRMZM2G320269_T01; parent_gene=GRMZM2G320269	GRMZM2G320269_P01	TRUE	TRUE	dGNLYQVETGR	95%	n+304 (+304)	43.23	25.00
2434	seq=translation; coord=1:222203896..222206239:-1; parent_transcript=GRMZM2G320269_T01; parent_gene=GRMZM2G320269	GRMZM2G320269_P01	TRUE	TRUE	dScPDAEAVVR	95%	n+304 (+304), Carbamidomethyl (+57)	52.15	25.00
2435	seq=translation; coord=1:222203896..222206239:-1; parent_transcript=GRMZM2G320269_T01; parent_gene=GRMZM2G320269	GRMZM2G320269_P01	TRUE	TRUE	dSDPTLDGAYAAELR	95%	n+304 (+304)	41.54	25.00
2436	seq=translation; coord=1:222203896..222206239:-1; parent_transcript=GRMZM2G320269_T01; parent_gene=GRMZM2G320269	GRMZM2G320269_P01	TRUE	TRUE	nLPDSMDGIR	95%	n+304 (+304)	33.44	25.00
2437	seq=translation; coord=10:132619525..132621315:1; parent_transcript=GRMZM2G468855_T02; parent_gene=GRMZM2G468855	GRMZM2G468855_P02	TRUE	TRUE	dFEAGALk	93%	n+304 (+304), K+304 (+304)	30.47	27.12
2438	seq=translation; coord=10:132619525..132621315:1; parent_transcript=GRMZM2G468855_T02; parent_gene=GRMZM2G468855	GRMZM2G468855_P02	TRUE	TRUE	gAAAVEELADAGLSGVVFHQLEVTDAQSIAR	95%	n+304 (+304)	73.78	25.00
2439	seq=translation; coord=10:132619525..132621315:1; parent_transcript=GRMZM2G468855_T02; parent_gene=GRMZM2G468855	GRMZM2G468855_P02	TRUE	TRUE	gMDAFQMAELMR	95%	n+304 (+304)	69.08	25.00
2440	seq=translation; coord=10:132619525..132621315:1; parent_transcript=GRMZM2G468855_T02; parent_gene=GRMZM2G468855	GRMZM2G468855_P02	TRUE	TRUE	gWPTEFAAYk	95%	n+304 (+304), K+304 (+304)	38.18	25.38
2441	seq=translation; coord=10:132619525..132621315:1; parent_transcript=GRMZM2G468855_T02; parent_gene=GRMZM2G468855	GRMZM2G468855_P02	TRUE	TRUE	lDELLSTFLR	88%	n+304 (+304)	27.89	26.59
2442	seq=translation; coord=10:132619525..132621315:1; parent_transcript=GRMZM2G468855_T02; parent_gene=GRMZM2G468855	GRMZM2G468855_P02	TRUE	TRUE	nVTEALLPLLLQASSSGGGR	92%	n+304 (+304)	27.83	25.94
2443	seq=translation; coord=10:132619525..132621315:1; parent_transcript=GRMZM2G468855_T02; parent_gene=GRMZM2G468855	GRMZM2G468855_P02	TRUE	TRUE	vALLPEGGPTGAFFALGk	95%	n+304 (+304), K+304 (+304)	57.43	25.00
2444	seq=translation; coord=10:132619525..132621315:1; parent_transcript=GRMZM2G468855_T02; parent_gene=GRMZM2G468855	GRMZM2G468855_P02	TRUE	TRUE	vVNVSSDFGLLR	95%	n+304 (+304)	50.24	25.74
2445	seq=translation; coord=8:162019785..162023951:1; parent_transcript=GRMZM2G053898_T01; parent_gene=GRMZM2G053898	GRMZM2G053898_P01,GRMZM2G053898_P02, GRMZM2G082390_P01,GRMZM2G082390_P02	TRUE	TRUE	gQDGNEVFFR	95%	n+304 (+304)	34.62	25.00

2446	seq=translation; coord=8:162019785..162023951:1; parent_transcript=GRMZM2G053898_T01; parent_gene=GRMZM2G053898	GRMZM2G053898_P01,GRMZM2G053898_P02, GRMZM2G082390_P01,GRMZM2G082390_P02	TRUE	TRUE	lMNAYcDR	86%	n+304 (+304), Carbamidomethyl (+57)	25.43	25.00
2447	seq=translation; coord=8:162019785..162023951:1; parent_transcript=GRMZM2G053898_T01; parent_gene=GRMZM2G053898	GRMZM2G053898_P01,GRMZM2G053898_P02, GRMZM2G082390_P01,GRMZM2G082390_P02	TRUE	TRUE	qSVDMNAIAFLFDGR	95%	n+304 (+304)	89.83	25.00
2448	seq=translation; coord=8:173117675..173120538:1; parent_transcript=GRMZM2G116273_T01; parent_gene=GRMZM2G116273	GRMZM2G116273_P01	TRUE	TRUE	lYGAVMSWNVTR	95%	n+304 (+304)	38.40	25.00
2449	seq=translation; coord=8:173117675..173120538:1; parent_transcript=GRMZM2G116273_T01; parent_gene=GRMZM2G116273	GRMZM2G116273_P01	TRUE	TRUE	nPFGQVPALQDGDLYLFESR	95%	n+304 (+304)	56.21	25.00
2450	seq=translation; coord=8:173117675..173120538:1; parent_transcript=GRMZM2G116273_T01; parent_gene=GRMZM2G116273	GRMZM2G116273_P01	TRUE	TRUE	nPFGQVPALQDGDLyLFESR	95%	n+304 (+304), iTRAQ8plex (+304)	34.47	25.00
2451	seq=translation; coord=1:250061761..250067450:1; parent_transcript=GRMZM2G157470_T01; parent_gene=GRMZM2G157470	GRMZM2G157470_P01	TRUE	TRUE	aANcDEGQIQPGASAVR	95%	n+304 (+304), Carbamidomethyl (+57)	64.80	25.00
2452	seq=translation; coord=1:250061761..250067450:1; parent_transcript=GRMZM2G157470_T01; parent_gene=GRMZM2G157470	GRMZM2G157470_P01	TRUE	TRUE	aASTTELPAPSGWTk	95%	n+304 (+304), K+304 (+304)	64.66	25.82
2453	seq=translation; coord=1:250061761..250067450:1; parent_transcript=GRMZM2G157470_T01; parent_gene=GRMZM2G157470	GRMZM2G157470_P01	TRUE	TRUE	aHPGGPASSEFDWGTSDTPR	95%	n+304 (+304)	92.13	25.00
2454	seq=translation; coord=1:250061761..250067450:1; parent_transcript=GRMZM2G157470_T01; parent_gene=GRMZM2G157470	GRMZM2G157470_P01	TRUE	TRUE	aTESPEGEk	95%	n+304 (+304), K+304 (+304)	30.66	25.00
2455	seq=translation; coord=1:250061761..250067450:1; parent_transcript=GRMZM2G157470_T01; parent_gene=GRMZM2G157470	GRMZM2G157470_P01	TRUE	TRUE	eVAPAVDATEETEk	95%	n+304 (+304), K+304 (+304)	52.53	25.49
2456	seq=translation; coord=1:250061761..250067450:1; parent_transcript=GRMZM2G157470_T01; parent_gene=GRMZM2G157470	GRMZM2G157470_P01	TRUE	TRUE	eVPNADAAEk	95%	n+304 (+304), K+304 (+304)	37.15	25.60
2457	seq=translation; coord=1:250061761..250067450:1; parent_transcript=GRMZM2G157470_T01; parent_gene=GRMZM2G157470	GRMZM2G157470_P01	TRUE	TRUE	fEVIFVSPTGEEVk	95%	n+304 (+304), K+304 (+304)	60.15	25.63
2458	seq=translation; coord=1:250061761..250067450:1; parent_transcript=GRMZM2G157470_T01; parent_gene=GRMZM2G157470	GRMZM2G157470_P01	TRUE	TRUE	gAENSGQPNTGSQEPk	95%	n+304 (+304), K+304 (+304)	36.41	25.00
2459	seq=translation; coord=1:250061761..250067450:1; parent_transcript=GRMZM2G157470_T01; parent_gene=GRMZM2G157470	GRMZM2G157470_P01	TRUE	TRUE	gTDVEMk	91%	n+304 (+304), K+304 (+304)	28.95	26.22
2460	seq=translation; coord=1:250061761..250067450:1; parent_transcript=GRMZM2G157470_T01; parent_gene=GRMZM2G157470	GRMZM2G157470_P01	TRUE	TRUE	qLTQYLk	92%	n+304 (+304), K+304 (+304)	27.52	25.00
2461	seq=translation; coord=9:130484606..130489460:-1; parent_transcript=GRMZM2G436092_T01; parent_gene=GRMZM2G436092	GRMZM2G436092_P01,GRMZM2G436092_P02, GRMZM2G436092_P03,GRMZM2G436092_P05	TRUE	TRUE	aGDVcFSEVYR	95%	n+304 (+304), Carbamidomethyl (+57)	42.74	25.00
2462	seq=translation; coord=9:130484606..130489460:-1; parent_transcript=GRMZM2G436092_T01; parent_gene=GRMZM2G436092	GRMZM2G436092_P01,GRMZM2G436092_P02, GRMZM2G436092_P03,GRMZM2G436092_P05	TRUE	TRUE	dAEEAIAGR	95%	n+304 (+304)	32.09	25.00
2463	seq=translation; coord=9:130484606..130489460:-1; parent_transcript=GRMZM2G436092_T01; parent_gene=GRMZM2G436092	GRMZM2G436092_P01,GRMZM2G436092_P02, GRMZM2G436092_P03,GRMZM2G436092_P05	TRUE	TRUE	eGGGTVGIVDYTNYDDMk	95%	n+304 (+304), K+304 (+304)	57.16	25.00

2464	seq=translation; coord=9:130484606..130489460:-1; parent_transcript=GRMZM2G436092_T01; parent_gene=GRMZM2G436092	GRMZM2G436092_P01,GRMZM2G436092_P02, GRMZM2G436092_P03,GRMZM2G436092_P05	TRUE	TRUE	eVEDLFYk	95%	n+304 (+304), K+304 (+304)	35.76	25.62
2465	seq=translation; coord=9:130484606..130489460:-1; parent_transcript=GRMZM2G436092_T01; parent_gene=GRMZM2G436092	GRMZM2G436092_P01,GRMZM2G436092_P02, GRMZM2G436092_P03,GRMZM2G436092_P05	TRUE	TRUE	iVDIDLk	92%	n+304 (+304), K+304 (+304)	26.45	25.00
2466	seq=translation; coord=9:130484606..130489460:-1; parent_transcript=GRMZM2G436092_T01; parent_gene=GRMZM2G436092	GRMZM2G436092_P01,GRMZM2G436092_P02, GRMZM2G436092_P03,GRMZM2G436092_P05	TRUE	TRUE	kAGDVcFSEVYR	95%	K+304 (+304), n+304 (+304), Carbamidomethyl (+57)	28.99	25.01
2467	seq=translation; coord=9:130484606..130489460:-1; parent_transcript=GRMZM2G436092_T01; parent_gene=GRMZM2G436092	GRMZM2G436092_P01,GRMZM2G436092_P02, GRMZM2G436092_P03,GRMZM2G436092_P05	TRUE	TRUE	tIYVGNLPGDIR	95%	n+304 (+304)	40.07	25.89
2468	seq=translation; coord=9:130484606..130489460:-1; parent_transcript=GRMZM2G436092_T01; parent_gene=GRMZM2G436092	GRMZM2G436092_P01,GRMZM2G436092_P02, GRMZM2G436092_P03,GRMZM2G436092_P05	TRUE	TRUE	vLVTGLPSSASWQDLk	95%	n+304 (+304), K+304 (+304)	49.18	25.00
2469	seq=translation; coord=4:236362229..236373144:1; parent_transcript=GRMZM2G168629_T01; parent_gene=GRMZM2G168629	GRMZM2G168629_P01,GRMZM2G168629_P04	TRUE	TRUE	aTLQVVQLLk	95%	n+304 (+304), K+304 (+304)	31.90	25.00
2470	seq=translation; coord=4:236362229..236373144:1; parent_transcript=GRMZM2G168629_T01; parent_gene=GRMZM2G168629	GRMZM2G168629_P01,GRMZM2G168629_P04	TRUE	TRUE	aTVAIAIk	95%	n+304 (+304), K+304 (+304)	41.37	25.29
2471	seq=translation; coord=4:236362229..236373144:1; parent_transcript=GRMZM2G168629_T01; parent_gene=GRMZM2G168629	GRMZM2G168629_P01,GRMZM2G168629_P04	TRUE	TRUE	cPSAVLAVLDSIVEPIEk	95%	Carbamidomethyl (+57), n+304 (+304), K+304 (+304)	38.44	25.00
2472	seq=translation; coord=4:236362229..236373144:1; parent_transcript=GRMZM2G168629_T01; parent_gene=GRMZM2G168629	GRMZM2G168629_P01,GRMZM2G168629_P04	TRUE	TRUE	eYSLQALESFMLR	95%	n+304 (+304)	50.10	25.71
2473	seq=translation; coord=4:236362229..236373144:1; parent_transcript=GRMZM2G168629_T01; parent_gene=GRMZM2G168629	GRMZM2G168629_P01,GRMZM2G168629_P04	TRUE	TRUE	gLLPEILPLLYDQTVIk	95%	n+304 (+304), K+304 (+304)	64.16	25.00
2474	seq=translation; coord=4:236362229..236373144:1; parent_transcript=GRMZM2G168629_T01; parent_gene=GRMZM2G168629	GRMZM2G168629_P01,GRMZM2G168629_P04	TRUE	TRUE	iLLSLAPQLIk	95%	n+304 (+304), K+304 (+304)	38.05	25.00
2475	seq=translation; coord=4:236362229..236373144:1; parent_transcript=GRMZM2G168629_T01; parent_gene=GRMZM2G168629	GRMZM2G168629_P01,GRMZM2G168629_P04	TRUE	TRUE	lTITVLQQLEDASGDVSGLAVk	95%	n+304 (+304), K+304 (+304)	43.66	25.00
2476	seq=translation; coord=4:236362229..236373144:1; parent_transcript=GRMZM2G168629_T01; parent_gene=GRMZM2G168629	GRMZM2G168629_P01,GRMZM2G168629_P04	TRUE	TRUE	mDIFNTFIELLR	95%	n+304 (+304)	48.00	26.09
2477	seq=translation; coord=4:236362229..236373144:1; parent_transcript=GRMZM2G168629_T01; parent_gene=GRMZM2G168629	GRMZM2G168629_P01,GRMZM2G168629_P04	TRUE	TRUE	yLPFILDQIDNQQk	95%	n+304 (+304), K+304 (+304)	34.33	25.35
2478	seq=translation; coord=4:236362229..236373144:1; parent_transcript=GRMZM2G168629_T01; parent_gene=GRMZM2G168629	GRMZM2G168629_P01,GRMZM2G168629_P04	TRUE	TRUE	yMATSDLLSELNk	95%	n+304 (+304), K+304 (+304)	39.45	25.09
2479	seq=translation; coord=7:131783728..131787563:1; parent_transcript=GRMZM2G069651_T01; parent_gene=GRMZM2G069651	GRMZM2G069651_P01	TRUE	TRUE	aSNTLSIIDSGIGMTk	95%	n+304 (+304), K+304 (+304)	38.15	25.72
2480	seq=translation; coord=7:131783728..131787563:1; parent_transcript=GRMZM2G069651_T01; parent_gene=GRMZM2G069651	GRMZM2G069651_P01	TRUE	TRUE	eDQLEYLEER	95%	n+304 (+304)	41.07	25.00
2481	seq=translation; coord=8:171565757..171566487:-1; parent_transcript=GRMZM2G091054_T01; parent_gene=GRMZM2G091054	GRMZM2G091054_P01	TRUE	TRUE	tYVMFPANPk	93%	n+304 (+304), K+304 (+304)	30.28	25.66

2482	seq=translation; coord=8:171565757..171566487:-1; parent_transcript=GRMZM2G091054_T01; parent_gene=GRMZM2G091054	GRMZM2G091054_P01	TRUE	TRUE	vPPSDAccGVIk	88%	n+304 (+304), Carbamidomethyl (+57), Carbamidomethyl (+57), K+304 (+304)	26.41	25.07
2483	seq=translation; coord=8:171565757..171566487:-1; parent_transcript=GRMZM2G091054_T01; parent_gene=GRMZM2G091054	GRMZM2G091054_P01	TRUE	TRUE	vVcMEk	91%	n+304 (+304), Carbamidomethyl (+57), K+304 (+304)	26.08	25.82
2484	seq=translation; coord=8:171565757..171566487:-1; parent_transcript=GRMZM2G091054_T01; parent_gene=GRMZM2G091054	GRMZM2G091054_P01	TRUE	TRUE	vVYVAEQck	95%	n+304 (+304), Carbamidomethyl (+57), K+304 (+304)	33.12	26.35
2485	seq=translation; coord=5:63317874..63324803:-1; parent_transcript=GRMZM2G088753_T01; parent_gene=GRMZM2G088753	GRMZM2G088753_P01,GRMZM2G088753_P02	TRUE	TRUE	eFADNVLPR	95%	n+304 (+304)	31.05	25.69
2486	seq=translation; coord=5:63317874..63324803:-1; parent_transcript=GRMZM2G088753_T01; parent_gene=GRMZM2G088753	GRMZM2G088753_P01,GRMZM2G088753_P02	TRUE	TRUE	fGAPYDGVHWDPPASER	95%	n+304 (+304)	40.08	25.00
2487	seq=translation; coord=5:63317874..63324803:-1; parent_transcript=GRMZM2G088753_T01; parent_gene=GRMZM2G088753	GRMZM2G088753_P01,GRMZM2G088753_P02	TRUE	TRUE	fGVWSIk	94%	n+304 (+304), K+304 (+304)	28.91	26.79
2488	seq=translation; coord=5:63317874..63324803:-1; parent_transcript=GRMZM2G088753_T01; parent_gene=GRMZM2G088753	GRMZM2G088753_P01,GRMZM2G088753_P02	TRUE	TRUE	fLHGGVWVDR	95%	n+304 (+304)	33.17	25.19
2489	seq=translation; coord=5:63317874..63324803:-1; parent_transcript=GRMZM2G088753_T01; parent_gene=GRMZM2G088753	GRMZM2G088753_P01,GRMZM2G088753_P02	TRUE	TRUE	gDLVFVFNFHPk	95%	n+304 (+304), K+304 (+304)	38.84	25.65
2490	seq=translation; coord=5:63317874..63324803:-1; parent_transcript=GRMZM2G088753_T01; parent_gene=GRMZM2G088753	GRMZM2G088753_P01,GRMZM2G088753_P02	TRUE	TRUE	gDVDHLPIYDLDPk	91%	n+304 (+304), K+304 (+304)	26.14	25.87
2491	seq=translation; coord=5:63317874..63324803:-1; parent_transcript=GRMZM2G088753_T01; parent_gene=GRMZM2G088753	GRMZM2G088753_P01,GRMZM2G088753_P02	TRUE	TRUE	gSIEENEGSLESFSk	95%	n+304 (+304), K+304 (+304)	55.38	25.00
2492	seq=translation; coord=5:63317874..63324803:-1; parent_transcript=GRMZM2G088753_T01; parent_gene=GRMZM2G088753	GRMZM2G088753_P01,GRMZM2G088753_P02	TRUE	TRUE	qWSLVDTDHLR	95%	n+304 (+304)	48.72	25.00
2493	seq=translation; coord=5:63317874..63324803:-1; parent_transcript=GRMZM2G088753_T01; parent_gene=GRMZM2G088753	GRMZM2G088753_P01,GRMZM2G088753_P02	TRUE	TRUE	rQWSLVDTDHLR	95%	n+304 (+304)	33.97	25.00
2494	seq=translation; coord=5:63317874..63324803:-1; parent_transcript=GRMZM2G088753_T01; parent_gene=GRMZM2G088753	GRMZM2G088753_P01,GRMZM2G088753_P02	TRUE	TRUE	vALDSDALVFGGHGR	95%	n+304 (+304)	49.03	25.37
2495	seq=translation; coord=5:63317874..63324803:-1; parent_transcript=GRMZM2G088753_T01; parent_gene=GRMZM2G088753	GRMZM2G088753_P01,GRMZM2G088753_P02	TRUE	TRUE	yMNAFDQAMNALDER	95%	n+304 (+304)	56.09	25.00
2496	seq=translation; coord=1:233228509..233231671:-1; parent_transcript=GRMZM2G134747_T01; parent_gene=GRMZM2G134747	GRMZM2G134747_P01	TRUE	TRUE	aALPTLTDDLIELMMQPPFR	95%	n+304 (+304)	33.20	25.45
2497	seq=translation; coord=1:233228509..233231671:-1; parent_transcript=GRMZM2G134747_T01; parent_gene=GRMZM2G134747	GRMZM2G134747_P01	TRUE	TRUE	aALPTLTDDLIELMmQPPFR	95%	n+304 (+304), Oxidation (+16)	41.30	25.65
2498	seq=translation; coord=1:233228509..233231671:-1; parent_transcript=GRMZM2G134747_T01; parent_gene=GRMZM2G134747	GRMZM2G134747_P01	TRUE	TRUE	aDTVPALR	95%	n+304 (+304)	36.06	25.42

2499	seq=translation; coord=1:233228509..233231671:-1; parent_transcript=GRMZM2G134747_T01; parent_gene=GRMZM2G134747	GRMZM2G134747_P01	TRUE	TRUE	tFSGIAAETGLTNVYVAQLLR	93%	n+304 (+304)	28.14	25.87
2500	seq=translation; coord=6:92813319..92815990:-1; parent_transcript=GRMZM2G043822_T02; parent_gene=GRMZM2G043822	GRMZM2G043822_P02,GRMZM2G043822_P03	TRUE	TRUE	aLTVPELTQQMWDSk	95%	n+304 (+304), K+304 (+304)	59.15	26.75
2501	seq=translation; coord=6:92813319..92815990:-1; parent_transcript=GRMZM2G043822_T02; parent_gene=GRMZM2G043822	GRMZM2G043822_P02,GRMZM2G043822_P03	TRUE	TRUE	iNVYYNEASGGR	95%	n+304 (+304)	46.53	25.00
2502	seq=translation; coord=6:92813319..92815990:-1; parent_transcript=GRMZM2G043822_T02; parent_gene=GRMZM2G043822	GRMZM2G043822_P02,GRMZM2G043822_P03	TRUE	TRUE	lSTPTFGDLNHLISATMSGVTccLR	95%	n+304 (+304), Carbamidomethyl (+57), Carbamidomethyl (+57)	33.19	25.00
2503	seq=translation; coord=6:92813319..92815990:-1; parent_transcript=GRMZM2G043822_T02; parent_gene=GRMZM2G043822	GRMZM2G043822_P02,GRMZM2G043822_P03	TRUE	TRUE	sGPFGQIFRPDNFVFGQSGAGNNWAk	95%	n+304 (+304), K+304 (+304)	33.94	25.00
2504	seq=translation; coord=6:92813319..92815990:-1; parent_transcript=GRMZM2G043822_T02; parent_gene=GRMZM2G043822	GRMZM2G043822_P02,GRMZM2G043822_P03	TRUE	TRUE	sSVcDIPPIGLk	92%	n+304 (+304), Carbamidomethyl (+57), K+304 (+304)	28.61	25.00
2505	seq=translation; coord=9:59153476..59160133:1; parent_transcript=GRMZM2G104481_T01; parent_gene=GRMZM2G104481	GRMZM2G104481_P01,GRMZM2G104481_P04	TRUE	TRUE	gFGFVTFESEDAVER	95%	n+304 (+304)	63.87	25.00
2506	seq=translation; coord=9:59153476..59160133:1; parent_transcript=GRMZM2G104481_T01; parent_gene=GRMZM2G104481	GRMZM2G104481_P01,GRMZM2G104481_P04	TRUE	TRUE	gFGFVTFSDPSVLDR	95%	n+304 (+304)	47.32	25.00
2507	seq=translation; coord=9:59153476..59160133:1; parent_transcript=GRMZM2G104481_T01; parent_gene=GRMZM2G104481	GRMZM2G104481_P01,GRMZM2G104481_P04	TRUE	TRUE	gLAEAVGNVDAGGEDER	95%	n+304 (+304)	100.09	25.00
2508	seq=translation; coord=9:59153476..59160133:1; parent_transcript=GRMZM2G104481_T01; parent_gene=GRMZM2G104481	GRMZM2G104481_P01,GRMZM2G104481_P04	TRUE	TRUE	gLAEAVGNVDAGGEDERDADSSGGDASGk	95%	n+304 (+304), K+304 (+304)	31.99	25.00
2509	seq=translation; coord=9:59153476..59160133:1; parent_transcript=GRMZM2G104481_T01; parent_gene=GRMZM2G104481	GRMZM2G104481_P01,GRMZM2G104481_P04	TRUE	TRUE	iFVGGIPPSLTEDk	95%	n+304 (+304), K+304 (+304)	33.20	25.53
2510	seq=translation; coord=9:59153476..59160133:1; parent_transcript=GRMZM2G104481_T01; parent_gene=GRMZM2G104481	GRMZM2G104481_P01,GRMZM2G104481_P04	TRUE	TRUE	vFVGGVAWETTEETFTk	95%	n+304 (+304), K+304 (+304)	60.12	25.25
2511	seq=translation; coord=1:264798453..264802516:-1; parent_transcript=GRMZM2G118003_T03; parent_gene=GRMZM2G118003	GRMZM2G118003_P03	TRUE	TRUE	aSAYWFR	90%	n+304 (+304)	26.41	25.00
2512	seq=translation; coord=1:264798453..264802516:-1; parent_transcript=GRMZM2G118003_T03; parent_gene=GRMZM2G118003	GRMZM2G118003_P03	TRUE	TRUE	fGIVYVDFNTLER	95%	n+304 (+304)	38.99	25.97
2513	seq=translation; coord=1:264798453..264802516:-1; parent_transcript=GRMZM2G118003_T03; parent_gene=GRMZM2G118003	GRMZM2G118003_P03	TRUE	TRUE	gANWLGGLSR	93%	n+304 (+304)	29.81	25.33
2514	seq=translation; coord=1:264798453..264802516:-1; parent_transcript=GRMZM2G118003_T03; parent_gene=GRMZM2G118003	GRMZM2G118003_P03	TRUE	TRUE	gSADYIGINEYTSSYMk	95%	n+304 (+304), K+304 (+304)	41.98	25.00
2515	seq=translation; coord=1:264798453..264802516:-1; parent_transcript=GRMZM2G118003_T03; parent_gene=GRMZM2G118003	GRMZM2G118003_P03	TRUE	TRUE	hWFTFNEPR	95%	n+304 (+304)	47.81	25.00
2516	seq=translation; coord=1:264798453..264802516:-1; parent_transcript=GRMZM2G118003_T03; parent_gene=GRMZM2G118003	GRMZM2G118003_P03	TRUE	TRUE	iVALLGYDTGSNPPQR	95%	n+304 (+304)	59.41	25.89

2517	seq=translation; coord=1:264798453..264802516:-1; parent_transcript=GRMZM2G118003_T03; parent_gene=GRMZM2G118003	GRMZM2G118003_P03	TRUE	TRUE	mADLFTDYADFcFk	95%	n+304 (+304), Carbamidomethyl (+57), K+304 (+304)	52.04	25.00
2518	seq=translation; coord=1:264798453..264802516:-1; parent_transcript=GRMZM2G118003_T03; parent_gene=GRMZM2G118003	GRMZM2G118003_P03	TRUE	TRUE	mADLFTDyADFcFk	95%	n+304 (+304), iTRAQ8plex (+304), Carbamidomethyl (+57), K+304 (+304)	27.38	25.00
2519	seq=translation; coord=1:264798453..264802516:-1; parent_transcript=GRMZM2G118003_T03; parent_gene=GRMZM2G118003	GRMZM2G118003_P03	TRUE	TRUE	sYIGQLk	91%	n+304 (+304), K+304 (+304)	25.73	25.69
2520	seq=translation; coord=1:35455268..35465683:-1; parent_transcript=GRMZM2G164562_T01; parent_gene=GRMZM2G164562	GRMZM2G164562_P01	TRUE	TRUE	aMLSLPASk	95%	n+304 (+304), K+304 (+304)	43.48	26.93
2521	seq=translation; coord=1:35455268..35465683:-1; parent_transcript=GRMZM2G164562_T01; parent_gene=GRMZM2G164562	GRMZM2G164562_P01	TRUE	TRUE	eHEDVELLAR	95%	n+304 (+304)	47.40	25.00
2522	seq=translation; coord=1:35455268..35465683:-1; parent_transcript=GRMZM2G164562_T01; parent_gene=GRMZM2G164562	GRMZM2G164562_P01	TRUE	TRUE	gDSIGGVVTcIAR	95%	n+304 (+304), Carbamidomethyl (+57)	43.24	25.00
2523	seq=translation; coord=1:35455268..35465683:-1; parent_transcript=GRMZM2G164562_T01; parent_gene=GRMZM2G164562	GRMZM2G164562_P01	TRUE	TRUE	gLGSPVFDk	94%	n+304 (+304), K+304 (+304)	33.10	26.98
2524	seq=translation; coord=1:35455268..35465683:-1; parent_transcript=GRMZM2G164562_T01; parent_gene=GRMZM2G164562	GRMZM2G164562_P01	TRUE	TRUE	iPLTEADMQVELDR	95%	n+304 (+304)	43.34	25.43
2525	seq=translation; coord=1:35455268..35465683:-1; parent_transcript=GRMZM2G164562_T01; parent_gene=GRMZM2G164562	GRMZM2G164562_P01	TRUE	TRUE	mIAAIDTVR	95%	n+304 (+304)	31.17	25.00
2526	seq=translation; coord=1:35455268..35465683:-1; parent_transcript=GRMZM2G164562_T01; parent_gene=GRMZM2G164562	GRMZM2G164562_P01	TRUE	TRUE	sGVEILAFVSk	95%	n+304 (+304), K+304 (+304)	29.48	25.00
2527	seq=translation; coord=1:35455268..35465683:-1; parent_transcript=GRMZM2G164562_T01; parent_gene=GRMZM2G164562	GRMZM2G164562_P01	TRUE	TRUE	vHQVVLPEDAVDYETVTLEHIESNIVR	95%	n+304 (+304)	43.55	25.26
2528	seq=translation; coord=5:77434003..77436424:1; parent_transcript=GRMZM5G805485_T01; parent_gene=GRMZM5G805485	GRMZM5G805485_P01	TRUE	TRUE	aSYAALQAWk	95%	n+304 (+304), K+304 (+304)	45.38	25.71
2529	seq=translation; coord=5:77434003..77436424:1; parent_transcript=GRMZM5G805485_T01; parent_gene=GRMZM5G805485	GRMZM5G805485_P01	TRUE	TRUE	fVGGFPEVVLSLPALR	95%	n+304 (+304)	48.07	25.04
2530	seq=translation; coord=5:77434003..77436424:1; parent_transcript=GRMZM5G805485_T01; parent_gene=GRMZM5G805485	GRMZM5G805485_P01	TRUE	TRUE	lEGAVPAGVcALASLR	95%	n+304 (+304), Carbamidomethyl (+57)	47.14	26.55
2531	seq=translation; coord=5:77434003..77436424:1; parent_transcript=GRMZM5G805485_T01; parent_gene=GRMZM5G805485	GRMZM5G805485_P01	TRUE	TRUE	lQGQLPSAIANMAAVQELDVAR	95%	n+304 (+304)	60.72	26.09
2532	seq=translation; coord=3:220506759..220509488:1; parent_transcript=GRMZM2G116135_T01; parent_gene=GRMZM2G116135	GRMZM2G116135_P01	TRUE	TRUE	ePTAPVAAPAPSTGVPk	95%	n+304 (+304), K+304 (+304)	35.57	25.00
2533	seq=translation; coord=3:220506759..220509488:1; parent_transcript=GRMZM2G116135_T01; parent_gene=GRMZM2G116135	GRMZM2G116135_P01	TRUE	TRUE	vWLDPNEVSEISMANSR	95%	n+304 (+304)	49.02	25.00
2534	seq=translation; coord=6:39092081..39092901:1; parent_transcript=GRMZM2G012806_T01; parent_gene=GRMZM2G012806	GRMZM2G012806_P01	TRUE	TRUE	dMPNAYIQVLPVGSPVTLDLRPDR	95%	n+304 (+304)	30.44	25.66

2535	seq=translation; coord=6:39092081..39092901:1; parent_transcript=GRMZM2G012806_T01; parent_gene=GRMZM2G012806	GRMZM2G012806_P01	TRUE	TRUE	dmPNAYIQVLPVGSPVTLDLRPDR	95%	n+304 (+304), Oxidation (+16)	30.05	25.00
2536	seq=translation; coord=6:39092081..39092901:1; parent_transcript=GRMZM2G012806_T01; parent_gene=GRMZM2G012806	GRMZM2G012806_P01	TRUE	TRUE	eATETILk	95%	n+304 (+304), K+304 (+304)	40.19	25.04
2537	seq=translation; coord=6:39092081..39092901:1; parent_transcript=GRMZM2G012806_T01; parent_gene=GRMZM2G012806	GRMZM2G012806_P01	TRUE	TRUE	iFVDTVAMTPTVG	95%	n+304 (+304)	52.03	25.00
2538	seq=translation; coord=6:39092081..39092901:1; parent_transcript=GRMZM2G012806_T01; parent_gene=GRMZM2G012806	GRMZM2G012806_P01	TRUE	TRUE	kTSWPEVVGMSIk	95%	K+304 (+304), n+304 (+304), K+304 (+304)	33.60	25.00
2539	seq=translation; coord=10:55753301..55756434:-1; parent_transcript=GRMZM2G127087_T01; parent_gene=GRMZM2G127087	GRMZM2G127087_P01,GRMZM2G127087_P03	TRUE	TRUE	dFYMYLLk	93%	n+304 (+304), K+304 (+304)	28.37	26.19
2540	seq=translation; coord=10:55753301..55756434:-1; parent_transcript=GRMZM2G127087_T01; parent_gene=GRMZM2G127087	GRMZM2G127087_P01,GRMZM2G127087_P03	TRUE	TRUE	dQYGNFIEVDTR	95%	n+304 (+304)	55.02	25.00
2541	seq=translation; coord=10:55753301..55756434:-1; parent_transcript=GRMZM2G127087_T01; parent_gene=GRMZM2G127087	GRMZM2G127087_P01,GRMZM2G127087_P03	TRUE	TRUE	dYIPIYEk	91%	n+304 (+304), K+304 (+304)	28.88	25.72
2542	seq=translation; coord=10:55753301..55756434:-1; parent_transcript=GRMZM2G127087_T01; parent_gene=GRMZM2G127087	GRMZM2G127087_P01,GRMZM2G127087_P03	TRUE	TRUE	eLTEDSWk	88%	n+304 (+304), K+304 (+304)	26.35	25.12
2543	seq=translation; coord=10:55753301..55756434:-1; parent_transcript=GRMZM2G127087_T01; parent_gene=GRMZM2G127087	GRMZM2G127087_P01,GRMZM2G127087_P03	TRUE	TRUE	fMIEQGLALk	95%	n+304 (+304), K+304 (+304)	30.72	25.55
2544	seq=translation; coord=10:55753301..55756434:-1; parent_transcript=GRMZM2G127087_T01; parent_gene=GRMZM2G127087	GRMZM2G127087_P01,GRMZM2G127087_P03	TRUE	TRUE	gSHDVPEIVDLIITLQR	95%	n+304 (+304)	49.95	26.60
2545	seq=translation; coord=10:55753301..55756434:-1; parent_transcript=GRMZM2G127087_T01; parent_gene=GRMZM2G127087	GRMZM2G127087_P01,GRMZM2G127087_P03	TRUE	TRUE	iMEWVMSDAELVk	95%	n+304 (+304), K+304 (+304)	44.77	25.39
2546	seq=translation; coord=10:55753301..55756434:-1; parent_transcript=GRMZM2G127087_T01; parent_gene=GRMZM2G127087	GRMZM2G127087_P01,GRMZM2G127087_P03	TRUE	TRUE	lEHLESPIAEEVSSALDTPLFR	95%	n+304 (+304)	45.71	25.44
2547	seq=translation; coord=10:55753301..55756434:-1; parent_transcript=GRMZM2G127087_T01; parent_gene=GRMZM2G127087	GRMZM2G127087_P01,GRMZM2G127087_P03	TRUE	TRUE	lNFNLLQLLYSSELk	95%	n+304 (+304), K+304 (+304)	39.02	25.00
2548	seq=translation; coord=10:55753301..55756434:-1; parent_transcript=GRMZM2G127087_T01; parent_gene=GRMZM2G127087	GRMZM2G127087_P01,GRMZM2G127087_P03	TRUE	TRUE	sILEFAk	93%	n+304 (+304), K+304 (+304)	27.33	25.00
2549	seq=translation; coord=10:55753301..55756434:-1; parent_transcript=GRMZM2G127087_T01; parent_gene=GRMZM2G127087	GRMZM2G127087_P01,GRMZM2G127087_P03	TRUE	TRUE	sLLSLYNAAYLR	95%	n+304 (+304)	43.35	26.02
2550	seq=translation; coord=10:55753301..55756434:-1; parent_transcript=GRMZM2G127087_T01; parent_gene=GRMZM2G127087	GRMZM2G127087_P01,GRMZM2G127087_P03	TRUE	TRUE	tVDYMYk	86%	n+304 (+304), K+304 (+304)	25.34	25.00
2551	seq=translation; coord=10:55753301..55756434:-1; parent_transcript=GRMZM2G127087_T01; parent_gene=GRMZM2G127087	GRMZM2G127087_P01,GRMZM2G127087_P03	TRUE	TRUE	tVLEFAR	91%	n+304 (+304)	28.37	25.00
2552	seq=translation; coord=8:142871869..142875661:-1; parent_transcript=GRMZM2G134738_T01; parent_gene=GRMZM2G134738	GRMZM2G134738_P01,GRMZM2G134738_P02	TRUE	TRUE	eELEAELQGk	95%	n+304 (+304), K+304 (+304)	45.01	25.04

2553	seq=translation; coord=8:142871869..142875661:-1; parent_transcript=GRMZM2G134738_T01; parent_gene=GRMZM2G134738	GRMZM2G134738_P01,GRMZM2G134738_P02	TRUE	TRUE	eEPAVIESYYNk	95%	n+304 (+304), K+304 (+304)	37.80	25.38
2554	seq=translation; coord=8:142871869..142875661:-1; parent_transcript=GRMZM2G134738_T01; parent_gene=GRMZM2G134738	GRMZM2G134738_P01,GRMZM2G134738_P02	TRUE	TRUE	fDMDSPVGPFGTk	95%	n+304 (+304), K+304 (+304)	51.09	25.00
2555	seq=translation; coord=8:142871869..142875661:-1; parent_transcript=GRMZM2G134738_T01; parent_gene=GRMZM2G134738	GRMZM2G134738_P01,GRMZM2G134738_P02	TRUE	TRUE	rFDMDSPVGPFGTk	95%	n+304 (+304), K+304 (+304)	39.16	25.91
2556	seq=translation; coord=8:142871869..142875661:-1; parent_transcript=GRMZM2G134738_T01; parent_gene=GRMZM2G134738	GRMZM2G134738_P01,GRMZM2G134738_P02	TRUE	TRUE	vEDVMPIATGLER	95%	n+304 (+304)	44.99	25.94
2557	seq=translation; coord=8:142871869..142875661:-1; parent_transcript=GRMZM2G134738_T01; parent_gene=GRMZM2G134738	GRMZM2G134738_P01,GRMZM2G134738_P02	TRUE	TRUE	vEDVMPIATGLEREELEAELQGk	95%	n+304 (+304), K+304 (+304)	33.03	25.09
2558	seq=translation; coord=2:3098932..3104990:1; parent_transcript=GRMZM2G071333_T01; parent_gene=GRMZM2G071333	GRMZM2G071333_P01	TRUE	TRUE	sFGLDDEEIAR	89%	n+304 (+304)	26.85	25.00
2559	seq=translation; coord=2:3098932..3104990:1; parent_transcript=GRMZM2G071333_T01; parent_gene=GRMZM2G071333	GRMZM2G071333_P01	TRUE	TRUE	yQWEIMk	93%	n+304 (+304), K+304 (+304)	29.02	25.77
2560	seq=translation; coord=1:177045380..177050536:-1; parent_transcript=GRMZM2G094742_T03; parent_gene=GRMZM2G094742	GRMZM2G094742_P03	TRUE	TRUE	gNVTPDAVLQTVSk	95%	n+304 (+304), K+304 (+304)	48.13	26.14
2561	seq=translation; coord=1:177045380..177050536:-1; parent_transcript=GRMZM2G094742_T03; parent_gene=GRMZM2G094742	GRMZM2G094742_P03	TRUE	TRUE	mEGVESYDVDIMEQk	95%	n+304 (+304), K+304 (+304)	60.92	25.00
2562	seq=translation; coord=1:177045380..177050536:-1; parent_transcript=GRMZM2G094742_T03; parent_gene=GRMZM2G094742	GRMZM2G094742_P03	TRUE	TRUE	mEGVESYDVDIMEQk	95%	Oxidation (+16), n+304 (+304), K+304 (+304)	41.30	25.00
2563	seq=translation; coord=1:177045380..177050536:-1; parent_transcript=GRMZM2G094742_T03; parent_gene=GRMZM2G094742	GRMZM2G094742_P03	TRUE	TRUE	mEGVESYDVDImEQk	95%	n+304 (+304), Oxidation (+16), K+304 (+304)	38.03	25.00
2564	seq=translation; coord=1:177045380..177050536:-1; parent_transcript=GRMZM2G094742_T03; parent_gene=GRMZM2G094742	GRMZM2G094742_P03	TRUE	TRUE	tSFWEAEAVTSESATPAGATA	95%	n+304 (+304)	62.45	25.00
2565	seq=translation; coord=1:177045380..177050536:-1; parent_transcript=GRMZM2G094742_T03; parent_gene=GRMZM2G094742	GRMZM2G094742_P03	TRUE	TRUE	vGMScEGcVGAVk	95%	n+304 (+304), Carbamidomethyl (+57), Carbamidomethyl (+57), K+304 (+304)	50.27	25.00
2566	seq=translation; coord=2:29943554..29946951:-1; parent_transcript=GRMZM2G150295_T01; parent_gene=GRMZM2G150295	GRMZM2G150295_P01	TRUE	TRUE	eIVASAPLVVFSk	95%	n+304 (+304), K+304 (+304)	48.02	25.00
2567	seq=translation; coord=2:29943554..29946951:-1; parent_transcript=GRMZM2G150295_T01; parent_gene=GRMZM2G150295	GRMZM2G150295_P01	TRUE	TRUE	gITSSTSSPTSSPESR	95%	n+304 (+304)	87.93	25.00
2568	seq=translation; coord=2:29943554..29946951:-1; parent_transcript=GRMZM2G150295_T01; parent_gene=GRMZM2G150295	GRMZM2G150295_P01	TRUE	TRUE	hIGGcDDTMALNNDGk	95%	n+304 (+304), Carbamidomethyl (+57), K+304 (+304)	54.91	25.00
2569	seq=translation; coord=2:29943554..29946951:-1; parent_transcript=GRMZM2G150295_T01; parent_gene=GRMZM2G150295	GRMZM2G150295_P01	TRUE	TRUE	lVPLLTEAGAIAGSASk	95%	n+304 (+304), K+304 (+304)	34.08	25.00

2570	seq=translation; coord=2:29943554..29946951:-1; parent_transcript=GRMZM2G150295_T01; parent_gene=GRMZM2G150295	GRMZM2G150295_P01	TRUE	TRUE	tScPFcVR	88%	n+304 (+304), Carbamidomethyl (+57), Carbamidomethyl (+57)	26.38	25.00
2571	seq=translation; coord=4:191578887..191581169:1; parent_transcript=GRMZM2G004699_T01; parent_gene=GRMZM2G004699	GRMZM2G004699_P01	TRUE	TRUE	gFHVDGcEASAEAR	95%	n+304 (+304), Carbamidomethyl (+57)	48.25	25.00
2572	seq=translation; coord=4:191578887..191581169:1; parent_transcript=GRMZM2G004699_T01; parent_gene=GRMZM2G004699	GRMZM2G004699_P01	TRUE	TRUE	lYSSLWNADDWATR	95%	n+304 (+304)	67.12	25.00
2573	seq=translation; coord=4:191578887..191581169:1; parent_transcript=GRMZM2G004699_T01; parent_gene=GRMZM2G004699	GRMZM2G004699_P01	TRUE	TRUE	nYVPTWAQDHIHYIDGGR	95%	n+304 (+304)	38.85	25.00
2574	seq=translation; coord=4:191578887..191581169:1; parent_transcript=GRMZM2G004699_T01; parent_gene=GRMZM2G004699	GRMZM2G004699_P01	TRUE	TRUE	tDWSNAPFVASYR	92%	n+304 (+304)	29.02	25.00
2575	seq=translation; coord=4:191578887..191581169:1; parent_transcript=GRMZM2G004699_T01; parent_gene=GRMZM2G004699	GRMZM2G004699_P01	TRUE	TRUE	yGAAVPPEcAR	93%	n+304 (+304), Carbamidomethyl (+57)	29.52	25.00
2576	seq=translation; coord=4:191578887..191581169:1; parent_transcript=GRMZM2G004699_T01; parent_gene=GRMZM2G004699	GRMZM2G004699_P01	TRUE	TRUE	yTIYNYcTDR	93%	n+304 (+304), Carbamidomethyl (+57)	29.73	25.00
2577	seq=translation; coord=3:1711864..1714237:1; parent_transcript=GRMZM2G123558_T01; parent_gene=GRMZM2G123558	GRMZM2G123558_P01,GRMZM2G123558_P02	TRUE	TRUE	eVAVEEk	95%	n+304 (+304), K+304 (+304)	38.35	25.73
2578	seq=translation; coord=3:1711864..1714237:1; parent_transcript=GRMZM2G123558_T01; parent_gene=GRMZM2G123558	GRMZM2G123558_P01,GRMZM2G123558_P02	TRUE	TRUE	sGTSPLLPAITFILDk	95%	n+304 (+304), K+304 (+304)	32.92	25.00
2579	seq=translation; coord=3:1711864..1714237:1; parent_transcript=GRMZM2G123558_T01; parent_gene=GRMZM2G123558	GRMZM2G123558_P01,GRMZM2G123558_P02	TRUE	TRUE	vVEAYEASPPEVk	94%	n+304 (+304), K+304 (+304)	28.59	25.55
2580	seq=translation; coord=4:230101062..230104782:-1; parent_transcript=GRMZM2G134582_T01; parent_gene=GRMZM2G134582	GRMZM2G134582_P01,GRMZM2G320497_P01	TRUE	TRUE	iPVLETPDGPVFESNAIAR	95%	n+304 (+304)	37.96	26.03
2581	seq=translation; coord=4:230101062..230104782:-1; parent_transcript=GRMZM2G134582_T01; parent_gene=GRMZM2G134582	GRMZM2G134582_P01,GRMZM2G320497_P01	TRUE	TRUE	mILDDWk	95%	n+304 (+304), K+304 (+304)	30.61	25.21
2582	seq=translation; coord=4:230101062..230104782:-1; parent_transcript=GRMZM2G134582_T01; parent_gene=GRMZM2G134582	GRMZM2G134582_P01,GRMZM2G320497_P01	TRUE	TRUE	qAEAVPLVPQk	95%	n+304 (+304), K+304 (+304)	37.25	25.00
2583	seq=translation; coord=4:230101062..230104782:-1; parent_transcript=GRMZM2G134582_T01; parent_gene=GRMZM2G134582	GRMZM2G134582_P01,GRMZM2G320497_P01	TRUE	TRUE	tLIAAEYSGVk	95%	n+304 (+304), K+304 (+304)	53.32	25.71
2584	seq=translation; coord=7:124381997..124386923:1; parent_transcript=GRMZM2G389173_T01; parent_gene=GRMZM2G389173	GRMZM2G389173_P01	TRUE	TRUE	dEIIEFVk	95%	n+304 (+304), K+304 (+304)	45.98	26.17
2585	seq=translation; coord=7:124381997..124386923:1; parent_transcript=GRMZM2G389173_T01; parent_gene=GRMZM2G389173	GRMZM2G389173_P01	TRUE	TRUE	dLWIVEFFAPWcGHck	95%	n+304 (+304), Carbamidomethyl (+57), Carbamidomethyl (+57), K+304 (+304)	33.19	25.00
2586	seq=translation; coord=7:124381997..124386923:1; parent_transcript=GRMZM2G389173_T01; parent_gene=GRMZM2G389173	GRMZM2G389173_P01	TRUE	TRUE	dVkPIVEFALSQVk	95%	n+304 (+304), K+304 (+304), K+304 (+304)	27.76	25.00

2587	seq=translation; coord=7:124381997..124386923:1; parent_transcript=GRMZM2G389173_T01; parent_gene=GRMZM2G389173	GRMZM2G389173_P01	TRUE	TRUE	gNLPLNDAPTVVASEPWDGk	95%	n+304 (+304), K+304 (+304)	32.08	25.77
2588	seq=translation; coord=7:124381997..124386923:1; parent_transcript=GRMZM2G389173_T01; parent_gene=GRMZM2G389173	GRMZM2G389173_P01	TRUE	TRUE	gVATVAALDADAHQALAQEYGIk	95%	n+304 (+304), K+304 (+304)	41.03	25.24
2589	seq=translation; coord=7:124381997..124386923:1; parent_transcript=GRMZM2G389173_T01; parent_gene=GRMZM2G389173	GRMZM2G389173_P01	TRUE	TRUE	nFDELVVk	88%	n+304 (+304), K+304 (+304)	27.56	26.34
2590	seq=translation; coord=7:124381997..124386923:1; parent_transcript=GRMZM2G389173_T01; parent_gene=GRMZM2G389173	GRMZM2G389173_P01	TRUE	TRUE	qLAPAWEk	95%	n+304 (+304), K+304 (+304)	32.04	25.00
2591	seq=translation; coord=7:124381997..124386923:1; parent_transcript=GRMZM2G389173_T01; parent_gene=GRMZM2G389173	GRMZM2G389173_P01	TRUE	TRUE	vEGFPTILVFGADk	95%	n+304 (+304), K+304 (+304)	33.91	25.00
2592	seq=translation; coord=7:124381997..124386923:1; parent_transcript=GRMZM2G389173_T01; parent_gene=GRMZM2G389173	GRMZM2G389173_P01	TRUE	TRUE	vEGFPTILVFGADkESPFPYQGAR	95%	n+304 (+304), K+304 (+304)	27.32	25.00
2593	seq=translation; coord=7:124381997..124386923:1; parent_transcript=GRMZM2G389173_T01; parent_gene=GRMZM2G389173	GRMZM2G389173_P01	TRUE	TRUE	yLELLLSVAEk	95%	n+304 (+304), K+304 (+304)	31.01	25.00
2594	seq=translation; coord=2:7091727..7098725:-1; parent_transcript=GRMZM2G379758_T01; parent_gene=GRMZM2G379758	GRMZM2G379758_P01	TRUE	TRUE	aFSVINSLEIWk	91%	n+304 (+304), K+304 (+304)	25.84	25.00
2595	seq=translation; coord=2:7091727..7098725:-1; parent_transcript=GRMZM2G379758_T01; parent_gene=GRMZM2G379758	GRMZM2G379758_P01	TRUE	TRUE	aGLINEAHGcLTELYSTGR	95%	n+304 (+304), Carbamidomethyl (+57)	52.61	25.00
2596	seq=translation; coord=2:7091727..7098725:-1; parent_transcript=GRMZM2G379758_T01; parent_gene=GRMZM2G379758	GRMZM2G379758_P01	TRUE	TRUE	eEPLFLVVAQNVQDYLER	95%	n+304 (+304)	73.83	25.44
2597	seq=translation; coord=2:7091727..7098725:-1; parent_transcript=GRMZM2G379758_T01; parent_gene=GRMZM2G379758	GRMZM2G379758_P01	TRUE	TRUE	eEPLFLVVAQNVQDyLER	95%	n+304 (+304), iTRAQ8plex (+304)	38.64	25.00
2598	seq=translation; coord=2:7091727..7098725:-1; parent_transcript=GRMZM2G379758_T01; parent_gene=GRMZM2G379758	GRMZM2G379758_P01	TRUE	TRUE	eHVLEMLELk	95%	n+304 (+304), K+304 (+304)	35.48	25.71
2599	seq=translation; coord=2:7091727..7098725:-1; parent_transcript=GRMZM2G379758_T01; parent_gene=GRMZM2G379758	GRMZM2G379758_P01	TRUE	TRUE	eLLAQGVQQSR	95%	n+304 (+304)	33.16	26.88
2600	seq=translation; coord=2:7091727..7098725:-1; parent_transcript=GRMZM2G379758_T01; parent_gene=GRMZM2G379758	GRMZM2G379758_P01	TRUE	TRUE	gPSPFVVIPEAVHR	95%	n+304 (+304)	39.47	25.33
2601	seq=translation; coord=2:7091727..7098725:-1; parent_transcript=GRMZM2G379758_T01; parent_gene=GRMZM2G379758	GRMZM2G379758_P01	TRUE	TRUE	lQGLLFQMADk	95%	n+304 (+304), K+304 (+304)	36.50	25.98
2602	seq=translation; coord=2:7091727..7098725:-1; parent_transcript=GRMZM2G379758_T01; parent_gene=GRMZM2G379758	GRMZM2G379758_P01	TRUE	TRUE	lREEPLFLVVAQNVQDYLER	95%	n+304 (+304)	32.79	25.39
2603	seq=translation; coord=2:7091727..7098725:-1; parent_transcript=GRMZM2G379758_T01; parent_gene=GRMZM2G379758	GRMZM2G379758_P01	TRUE	TRUE	lREEPLFLVVAQNVQDyLER	94%	n+304 (+304), iTRAQ8plex (+304)	25.17	25.00
2604	seq=translation; coord=2:7091727..7098725:-1; parent_transcript=GRMZM2G379758_T01; parent_gene=GRMZM2G379758	GRMZM2G379758_P01	TRUE	TRUE	tLMDGLMFLIYk	95%	n+304 (+304), K+304 (+304)	35.62	26.28

2605	seq=translation; coord=2:7091727..7098725:-1; parent_transcript=GRMZM2G379758_T01; parent_gene=GRMZM2G379758	GRMZM2G379758_P01	TRUE	TRUE	wQDNFVSSQGR	94%	n+304 (+304)	29.11	25.00
2606	seq=translation; coord=8:150358554..150362799:-1; parent_transcript=GRMZM2G055936_T01; parent_gene=GRMZM2G055936	GRMZM2G055936_P01	TRUE	TRUE	aLDLELDLTDk	95%	n+304 (+304), K+304 (+304)	53.58	25.79
2607	seq=translation; coord=8:150358554..150362799:-1; parent_transcript=GRMZM2G055936_T01; parent_gene=GRMZM2G055936	GRMZM2G055936_P01	TRUE	TRUE	fLADGSGAYTk	95%	n+304 (+304), K+304 (+304)	67.55	25.12
2608	seq=translation; coord=8:150358554..150362799:-1; parent_transcript=GRMZM2G055936_T01; parent_gene=GRMZM2G055936	GRMZM2G055936_P01	TRUE	TRUE	gVDEILLISVNDPFVMk	95%	n+304 (+304), K+304 (+304)	41.36	25.21
2609	seq=translation; coord=8:150358554..150362799:-1; parent_transcript=GRMZM2G055936_T01; parent_gene=GRMZM2G055936	GRMZM2G055936_P01	TRUE	TRUE	vILFGVPGAFTPTcSNQHVPGFITQAEQLk	95%	n+304 (+304), Carbamidomethyl (+57), K+304 (+304)	26.97	25.00
2610	seq=translation; coord=7:172711231..172713259:1; parent_transcript=GRMZM2G063617_T01; parent_gene=GRMZM2G063617	GRMZM2G063617_P01	TRUE	TRUE	aGNLGDSVTISR	95%	n+304 (+304)	55.42	25.13
2611	seq=translation; coord=7:172711231..172713259:1; parent_transcript=GRMZM2G063617_T01; parent_gene=GRMZM2G063617	GRMZM2G063617_P01	TRUE	TRUE	iMEVASLEk	95%	n+304 (+304), K+304 (+304)	29.97	25.22
2612	seq=translation; coord=5:84934242..84936493:1; parent_transcript=GRMZM2G010321_T01; parent_gene=GRMZM2G010321	GRMZM2G010321_P01	TRUE	TRUE	fcEEEDNH	95%	n+304 (+304), Carbamidomethyl (+57)	50.89	25.00
2613	seq=translation; coord=5:84934242..84936493:1; parent_transcript=GRMZM2G010321_T01; parent_gene=GRMZM2G010321	GRMZM2G010321_P01	TRUE	TRUE	lSNSGDLNSLMDDDk	95%	n+304 (+304), K+304 (+304)	34.05	25.00
2614	seq=translation; coord=5:84934242..84936493:1; parent_transcript=GRMZM2G010321_T01; parent_gene=GRMZM2G010321	GRMZM2G010321_P01	TRUE	TRUE	lSNSGDLNSLMDDDkYSk	95%	n+304 (+304), K+304 (+304), K+304 (+304)	40.00	25.00
2615	seq=translation; coord=10:132006574..132009975:-1; parent_transcript=GRMZM2G164714_T02; parent_gene=GRMZM2G164714	GRMZM2G164714_P02	TRUE	TRUE	aFADAGADVLFIDALASR	95%	n+304 (+304)	80.95	25.11
2616	seq=translation; coord=10:132006574..132009975:-1; parent_transcript=GRMZM2G164714_T02; parent_gene=GRMZM2G164714	GRMZM2G164714_P02	TRUE	TRUE	gFINAGFAGIILEDQVSPk	95%	n+304 (+304), K+304 (+304)	52.34	25.20
2617	seq=translation; coord=10:132006574..132009975:-1; parent_transcript=GRMZM2G164714_T02; parent_gene=GRMZM2G164714	GRMZM2G164714_P02	TRUE	TRUE	iIAYPLSLIGVSMR	95%	n+304 (+304)	40.31	25.42
2618	seq=translation; coord=10:132006574..132009975:-1; parent_transcript=GRMZM2G164714_T02; parent_gene=GRMZM2G164714	GRMZM2G164714_P02	TRUE	TRUE	iIPGLAGANIMER	95%	n+304 (+304)	33.23	26.62
2619	seq=translation; coord=10:132006574..132009975:-1; parent_transcript=GRMZM2G164714_T02; parent_gene=GRMZM2G164714	GRMZM2G164714_P02	TRUE	TRUE	iPAGFLEGMTR	95%	n+304 (+304)	31.23	25.00
2620	seq=translation; coord=10:132006574..132009975:-1; parent_transcript=GRMZM2G164714_T02; parent_gene=GRMZM2G164714	GRMZM2G164714_P02	TRUE	TRUE	iPPPSSLPTFEEIk	95%	n+304 (+304), K+304 (+304)	32.82	25.30
2621	seq=translation; coord=10:132006574..132009975:-1; parent_transcript=GRMZM2G164714_T02; parent_gene=GRMZM2G164714	GRMZM2G164714_P02	TRUE	TRUE	lGLPDVGLISYGEMIDQGR	95%	n+304 (+304)	36.84	25.55
2622	seq=translation; coord=10:132006574..132009975:-1; parent_transcript=GRMZM2G164714_T02; parent_gene=GRMZM2G164714	GRMZM2G164714_P02	TRUE	TRUE	tPILSPVELEEIGYk	95%	n+304 (+304), K+304 (+304)	39.38	25.67

2623	seq=translation; coord=10:132006574..132009975:-1; parent_transcript=GRMZM2G164714_T02; parent_gene=GRMZM2G164714	GRMZM2G164714_P02	TRUE	TRUE	vLEMPGAHQAPAcYDALSAR	95%	n+304 (+304), Carbamidomethyl (+57)	38.36	25.00
2624	seq=translation; coord=2:71086139..71088448:-1; parent_transcript=GRMZM2G112176_T01; parent_gene=GRMZM2G112176	GRMZM2G112176_P01,GRMZM2G112176_P02	TRUE	TRUE	iLATPGAHQAPccYDALGAR	95%	n+304 (+304), Carbamidomethyl (+57), Carbamidomethyl (+57)	30.69	25.00
2625	seq=translation; coord=2:71086139..71088448:-1; parent_transcript=GRMZM2G112176_T01; parent_gene=GRMZM2G112176	GRMZM2G112176_P01,GRMZM2G112176_P02	TRUE	TRUE	lGLPDVGLISYGEMVDQGR	95%	n+304 (+304)	66.66	25.00
2626	seq=translation; coord=2:71086139..71088448:-1; parent_transcript=GRMZM2G112176_T01; parent_gene=GRMZM2G112176	GRMZM2G112176_P01,GRMZM2G112176_P02	TRUE	TRUE	lGLPDVGLISyGEMVDQGR	91%	n+304 (+304), iTRAQ8plex (+304)	26.01	25.58
2627	seq=translation; coord=2:71086139..71088448:-1; parent_transcript=GRMZM2G112176_T01; parent_gene=GRMZM2G112176	GRMZM2G112176_P01,GRMZM2G112176_P02	TRUE	TRUE	lGLPDVGLISYGEmVDQGR	95%	n+304 (+304), Oxidation (+16)	54.38	25.22
2628	seq=translation; coord=2:71086139..71088448:-1; parent_transcript=GRMZM2G112176_T01; parent_gene=GRMZM2G112176	GRMZM2G112176_P01,GRMZM2G112176_P02	TRUE	TRUE	sGESPAAALR	88%	n+304 (+304)	25.69	25.00
2629	seq=translation; coord=1:76732179..76733770:-1; parent_transcript=GRMZM2G077316_T01; parent_gene=GRMZM2G077316	GRMZM2G077316_P01	TRUE	TRUE	vQELYVYEINER	95%	n+304 (+304)	59.17	25.00
2630	seq=translation; coord=8:157452514..157453996:-1; parent_transcript=GRMZM2G059299_T01; parent_gene=GRMZM2G059299	GRMZM2G059299_P01	TRUE	TRUE	dGNLLLLDAR	95%	n+304 (+304)	52.97	26.91
2631	seq=translation; coord=8:157452514..157453996:-1; parent_transcript=GRMZM2G059299_T01; parent_gene=GRMZM2G059299	GRMZM2G059299_P01	TRUE	TRUE	gSPVGEGAAAELTAAGDLVLR	95%	n+304 (+304)	74.27	26.42
2632	seq=translation; coord=8:157452514..157453996:-1; parent_transcript=GRMZM2G059299_T01; parent_gene=GRMZM2G059299	GRMZM2G059299_P01	TRUE	TRUE	lVSLPGLAYFNDPATSLR	95%	n+304 (+304)	37.59	25.89
2633	seq=translation; coord=2:197582100..197595530:1; parent_transcript=GRMZM2G117870_T01; parent_gene=GRMZM2G117870	GRMZM2G117870_P01	TRUE	TRUE	eAFTMDEQR	87%	n+304 (+304)	25.23	25.00
2634	seq=translation; coord=2:197582100..197595530:1; parent_transcript=GRMZM2G117870_T01; parent_gene=GRMZM2G117870	GRMZM2G117870_P01	TRUE	TRUE	lENEMDIVLDAVNk	89%	n+304 (+304), K+304 (+304)	25.84	25.71
2635	seq=translation; coord=2:197582100..197595530:1; parent_transcript=GRMZM2G117870_T01; parent_gene=GRMZM2G117870	GRMZM2G117870_P01	TRUE	TRUE	lLHPFMPFVTEELWQR	95%	n+304 (+304)	79.97	25.53
2636	seq=translation; coord=2:197582100..197595530:1; parent_transcript=GRMZM2G117870_T01; parent_gene=GRMZM2G117870	GRMZM2G117870_P01	TRUE	TRUE	nESDANLEAQk	95%	n+304 (+304), K+304 (+304)	37.85	25.00
2637	seq=translation; coord=2:197582100..197595530:1; parent_transcript=GRMZM2G117870_T01; parent_gene=GRMZM2G117870	GRMZM2G117870_P01	TRUE	TRUE	vPAWYVTLEDDLDk	89%	n+304 (+304), K+304 (+304)	26.14	26.05
2638	seq=translation; coord=4:233024312..233031203:1; parent_transcript=GRMZM2G165817_T01; parent_gene=GRMZM2G165817	GRMZM2G165817_P01,GRMZM2G171604_P01	TRUE	TRUE	dHQPcIIFMDEIDAIGGR	95%	n+304 (+304), Carbamidomethyl (+57)	29.38	25.00
2639	seq=translation; coord=4:233024312..233031203:1; parent_transcript=GRMZM2G165817_T01; parent_gene=GRMZM2G165817	GRMZM2G165817_P01,GRMZM2G171604_P01	TRUE	TRUE	eSIELPLMNPELFLR	94%	n+304 (+304)	29.97	26.32
2640	seq=translation; coord=4:233024312..233031203:1; parent_transcript=GRMZM2G165817_T01; parent_gene=GRMZM2G165817	GRMZM2G165817_P01,GRMZM2G171604_P01	TRUE	TRUE	nVcTEAGMAAIR	87%	n+304 (+304), Carbamidomethyl (+57)	25.76	25.00

2641	seq=translation; coord=4:233024312..233031203:1; parent_transcript=GRMZM2G165817_T01; parent_gene=GRMZM2G165817	GRMZM2G165817_P01,GRMZM2G171604_P01	TRUE	TRUE	sLQSVGQIIGEVLRPLDNER	95%	n+304 (+304)	35.08	25.00
2642	seq=translation; coord=4:233024312..233031203:1; parent_transcript=GRMZM2G165817_T01; parent_gene=GRMZM2G165817	GRMZM2G165817_P01,GRMZM2G171604_P01	TRUE	TRUE	tLMELLNQLDGFDELGk	95%	n+304 (+304), K+304 (+304)	44.07	25.62
2643	seq=translation; coord=4:233024312..233031203:1; parent_transcript=GRMZM2G165817_T01; parent_gene=GRMZM2G165817	GRMZM2G165817_P01,GRMZM2G171604_P01	TRUE	TRUE	vVLDMTTLTIMR	95%	n+304 (+304)	35.98	25.29
2644	seq=translation; coord=1:216050547..216055495:-1; parent_transcript=GRMZM2G016189_T01; parent_gene=GRMZM2G016189	GRMZM2G016189_P01	TRUE	TRUE	eLGEGGIDNYLSVk	95%	n+304 (+304), K+304 (+304)	44.59	26.12
2645	seq=translation; coord=1:216050547..216055495:-1; parent_transcript=GRMZM2G016189_T01; parent_gene=GRMZM2G016189	GRMZM2G016189_P01	TRUE	TRUE	lGPVVSEGQYEk	95%	n+304 (+304), K+304 (+304)	50.98	26.52
2646	seq=translation; coord=1:216050547..216055495:-1; parent_transcript=GRMZM2G016189_T01; parent_gene=GRMZM2G016189	GRMZM2G016189_P01	TRUE	TRUE	rEPIGVVGLITPWNYPLLMATWk	95%	n+304 (+304), K+304 (+304)	36.07	25.00
2647	seq=translation; coord=1:216050547..216055495:-1; parent_transcript=GRMZM2G016189_T01; parent_gene=GRMZM2G016189	GRMZM2G016189_P01	TRUE	TRUE	rLPVVNPTTEAHIGEIPAGTAEDVDAAVAAAR	95%	n+304 (+304)	61.49	25.00
2648	seq=translation; coord=8:38410669..38412926:1; parent_transcript=GRMZM2G054123_T01; parent_gene=GRMZM2G054123	GRMZM2G054123_P01,GRMZM2G054123_P02, GRMZM2G054123_P03,GRMZM2G054123_P04, GRMZM2G054123_P05	TRUE	TRUE	eIGFTSDDVGLDADR	95%	n+304 (+304)	79.55	25.00
2649	seq=translation; coord=8:38410669..38412926:1; parent_transcript=GRMZM2G054123_T01; parent_gene=GRMZM2G054123	GRMZM2G054123_P01,GRMZM2G054123_P02, GRMZM2G054123_P03,GRMZM2G054123_P04, GRMZM2G054123_P05	TRUE	TRUE	lcDQVSDAVLDAcLAQDPDSk	95%	n+304 (+304), Carbamidomethyl (+57), Carbamidomethyl (+57), K+304 (+304)	44.80	25.00
2650	seq=translation; coord=8:38410669..38412926:1; parent_transcript=GRMZM2G054123_T01; parent_gene=GRMZM2G054123	GRMZM2G054123_P01,GRMZM2G054123_P02, GRMZM2G054123_P03,GRMZM2G054123_P04, GRMZM2G054123_P05	TRUE	TRUE	tQVTVEYVNEGGAMVPVR	95%	n+304 (+304)	52.30	25.00
2651	seq=translation; coord=9:139187765..139188857:1; parent_transcript=GRMZM2G305046_T01; parent_gene=GRMZM2G305046	GRMZM2G305046_P01,GRMZM2G305046_P02	TRUE	TRUE	lLGGVTIAHGGVLPNINPVLLPk	95%	n+304 (+304), K+304 (+304)	37.48	25.00
2652	seq=translation; coord=8:160396937..160401812:1; parent_transcript=GRMZM2G134176_T01; parent_gene=GRMZM2G134176	GRMZM2G134176_P01	TRUE	TRUE	dQWSPALTLk	95%	n+304 (+304), K+304 (+304)	35.98	26.09
2653	seq=translation; coord=8:160396937..160401812:1; parent_transcript=GRMZM2G134176_T01; parent_gene=GRMZM2G134176	GRMZM2G134176_P01	TRUE	TRUE	dYPTFAATAR	95%	n+304 (+304)	33.31	25.00
2654	seq=translation; coord=8:160396937..160401812:1; parent_transcript=GRMZM2G134176_T01; parent_gene=GRMZM2G134176	GRMZM2G134176_P01	TRUE	TRUE	lVEMGFPEDQVR	94%	n+304 (+304)	28.00	25.00
2655	seq=translation; coord=8:160396937..160401812:1; parent_transcript=GRMZM2G134176_T01; parent_gene=GRMZM2G134176	GRMZM2G134176_P01	TRUE	TRUE	sASTGMEEk	95%	n+304 (+304), K+304 (+304)	27.88	25.00
2656	seq=translation; coord=8:160396937..160401812:1; parent_transcript=GRMZM2G134176_T01; parent_gene=GRMZM2G134176	GRMZM2G134176_P01	TRUE	TRUE	sVDGDENMALEk	95%	n+304 (+304), K+304 (+304)	38.90	25.00
2657	seq=translation; coord=8:135138380..135140767:-1; parent_transcript=GRMZM2G076544_T01; parent_gene=GRMZM2G076544	GRMZM2G076544_P01	TRUE	TRUE	hVVFGQVLEGMDVVSLIESQETDR	95%	n+304 (+304)	99.64	25.00

2658	seq=translation; coord=8:135138380..135140767:-1; parent_transcript=GRMZM2G076544_T01; parent_gene=GRMZM2G076544	GRMZM2G076544_P01	TRUE	TRUE	iVIGLYGDDVPQTTENFR	95%	n+304 (+304)	38.96	25.00
2659	seq=translation; coord=5:169454598..169459090:1; parent_transcript=GRMZM2G139300_T01; parent_gene=GRMZM2G139300	GRMZM2G139300_P01	TRUE	TRUE	aEAFDPAYDDDAQk	95%	n+304 (+304), K+304 (+304)	30.73	25.00
2660	seq=translation; coord=5:169454598..169459090:1; parent_transcript=GRMZM2G139300_T01; parent_gene=GRMZM2G139300	GRMZM2G139300_P01	TRUE	TRUE	dASDPLLR	86%	n+304 (+304)	25.24	25.00
2661	seq=translation; coord=5:169454598..169459090:1; parent_transcript=GRMZM2G139300_T01; parent_gene=GRMZM2G139300	GRMZM2G139300_P01	TRUE	TRUE	gMALVYR	87%	n+304 (+304)	26.29	25.54
2662	seq=translation; coord=5:169454598..169459090:1; parent_transcript=GRMZM2G139300_T01; parent_gene=GRMZM2G139300	GRMZM2G139300_P01	TRUE	TRUE	gWAGIHAIPR	95%	n+304 (+304)	33.95	25.00
2663	seq=translation; coord=5:169454598..169459090:1; parent_transcript=GRMZM2G139300_T01; parent_gene=GRMZM2G139300	GRMZM2G139300_P01	TRUE	TRUE	gWYHLFYQYNPk	95%	n+304 (+304), K+304 (+304)	35.53	25.44
2664	seq=translation; coord=5:169454598..169459090:1; parent_transcript=GRMZM2G139300_T01; parent_gene=GRMZM2G139300	GRMZM2G139300_P01	TRUE	TRUE	qLLQWPIHEVEk	90%	Pyro-cmC (-17), n+304 (+304), K+304 (+304)	27.75	25.31
2665	seq=translation; coord=5:169454598..169459090:1; parent_transcript=GRMZM2G139300_T01; parent_gene=GRMZM2G139300	GRMZM2G139300_P01	TRUE	TRUE	tFYDPVER	90%	n+304 (+304)	27.61	25.00
2666	seq=translation; coord=5:169454598..169459090:1; parent_transcript=GRMZM2G139300_T01; parent_gene=GRMZM2G139300	GRMZM2G139300_P01	TRUE	TRUE	vLMcTDPTk	95%	n+304 (+304), Carbamidomethyl (+57), K+304 (+304)	38.14	25.00
2667	seq=translation; coord=3:173015163..173022969:-1; parent_transcript=GRMZM2G038281_T01; parent_gene=GRMZM2G038281	GRMZM2G038281_P01,GRMZM2G038281_P02	TRUE	TRUE	acLNTNScTVSLTDESFGk	95%	n+304 (+304), Carbamidomethyl (+57), Carbamidomethyl (+57), K+304 (+304)	34.00	25.00
2668	seq=translation; coord=3:173015163..173022969:-1; parent_transcript=GRMZM2G038281_T01; parent_gene=GRMZM2G038281	GRMZM2G038281_P01,GRMZM2G038281_P02	TRUE	TRUE	dAGLLLILR	92%	n+304 (+304)	28.57	25.00
2669	seq=translation; coord=3:173015163..173022969:-1; parent_transcript=GRMZM2G038281_T01; parent_gene=GRMZM2G038281	GRMZM2G038281_P01,GRMZM2G038281_P02	TRUE	TRUE	dLcPGVTk	91%	n+304 (+304), Carbamidomethyl (+57), K+304 (+304)	29.02	25.71
2670	seq=translation; coord=3:173015163..173022969:-1; parent_transcript=GRMZM2G038281_T01; parent_gene=GRMZM2G038281	GRMZM2G038281_P01,GRMZM2G038281_P02	TRUE	TRUE	fASLGNPSGTcR	86%	n+304 (+304), Carbamidomethyl (+57)	25.22	25.00
2671	seq=translation; coord=3:173015163..173022969:-1; parent_transcript=GRMZM2G038281_T01; parent_gene=GRMZM2G038281	GRMZM2G038281_P01,GRMZM2G038281_P02	TRUE	TRUE	gGSVQNYYVYHGGTNFGR	95%	n+304 (+304)	43.46	25.00
2672	seq=translation; coord=3:173015163..173022969:-1; parent_transcript=GRMZM2G038281_T01; parent_gene=GRMZM2G038281	GRMZM2G038281_P01,GRMZM2G038281_P02	TRUE	TRUE	iWTENWPGWFQTFGESNPHRPPEDVAFAVAR	95%	n+304 (+304)	29.43	25.00
2673	seq=translation; coord=3:173015163..173022969:-1; parent_transcript=GRMZM2G038281_T01; parent_gene=GRMZM2G038281	GRMZM2G038281_P01,GRMZM2G038281_P02	TRUE	TRUE	nQPLTWYk	91%	n+304 (+304), K+304 (+304)	29.15	25.84
2674	seq=translation; coord=3:173015163..173022969:-1; parent_transcript=GRMZM2G038281_T01; parent_gene=GRMZM2G038281	GRMZM2G038281_P01,GRMZM2G038281_P02	TRUE	TRUE	sFTTYIVDMMk	95%	n+304 (+304), K+304 (+304)	35.18	25.02

2675	seq=translation; coord=3:173015163..173022969:-1; parent_transcript=GRMZM2G038281_T01; parent_gene=GRMZM2G038281	GRMZM2G038281_P01,GRMZM2G038281_P02	TRUE	TRUE	vNVDVPQGDDPVGIDMQSMGk	95%	n+304 (+304), K+304 (+304)	46.95	25.00
2676	seq=translation; coord=2:82394515..82407013:1; parent_transcript=GRMZM5G858094_T01; parent_gene=GRMZM5G858094	GRMZM5G858094_P01,GRMZM5G858094_P02	TRUE	TRUE	dIIEENLSYGSEk	95%	n+304 (+304), K+304 (+304)	43.59	25.15
2677	seq=translation; coord=2:82394515..82407013:1; parent_transcript=GRMZM5G858094_T01; parent_gene=GRMZM5G858094	GRMZM5G858094_P01,GRMZM5G858094_P02	TRUE	TRUE	eDAFFETVTNLAcER	95%	n+304 (+304), Carbamidomethyl (+57)	34.80	25.00
2678	seq=translation; coord=8:74938636..74939549:-1; parent_transcript=GRMZM2G045664_T01; parent_gene=GRMZM2G045664	GRMZM2G045664_P01	TRUE	TRUE	eVASGTMPDPGWIAASGSTALEIPAk	95%	n+304 (+304), K+304 (+304)	55.20	25.47
2679	seq=translation; coord=8:74938636..74939549:-1; parent_transcript=GRMZM2G045664_T01; parent_gene=GRMZM2G045664	GRMZM2G045664_P01	TRUE	TRUE	lPIcEVTYTLR	90%	n+304 (+304), Carbamidomethyl (+57)	27.63	25.00
2680	seq=translation; coord=8:74938636..74939549:-1; parent_transcript=GRMZM2G045664_T01; parent_gene=GRMZM2G045664	GRMZM2G045664_P01	TRUE	TRUE	vPYDFLVSLVR	92%	n+304 (+304)	30.52	26.58
2681	seq=translation; coord=3:26352719..26355885:1; parent_transcript=GRMZM2G410916_T02; parent_gene=GRMZM2G410916	GRMZM2G410916_P02	TRUE	TRUE	dTVIVEER	95%	n+304 (+304)	34.76	25.66
2682	seq=translation; coord=3:26352719..26355885:1; parent_transcript=GRMZM2G410916_T02; parent_gene=GRMZM2G410916	GRMZM2G410916_P02	TRUE	TRUE	gDVQLVMANAAPWWLVk	95%	n+304 (+304), K+304 (+304)	46.02	25.55
2683	seq=translation; coord=3:26352719..26355885:1; parent_transcript=GRMZM2G410916_T02; parent_gene=GRMZM2G410916	GRMZM2G410916_P02	TRUE	TRUE	iTEVTVR	93%	n+304 (+304)	29.57	25.00
2684	seq=translation; coord=3:26352719..26355885:1; parent_transcript=GRMZM2G410916_T02; parent_gene=GRMZM2G410916	GRMZM2G410916_P02	TRUE	TRUE	lLLNLDAVVR	95%	n+304 (+304)	32.77	25.00
2685	seq=translation; coord=3:26352719..26355885:1; parent_transcript=GRMZM2G410916_T02; parent_gene=GRMZM2G410916	GRMZM2G410916_P02	TRUE	TRUE	sDIcDLAGDVR	95%	n+304 (+304), Carbamidomethyl (+57)	40.42	25.00
2686	seq=translation; coord=3:26352719..26355885:1; parent_transcript=GRMZM2G410916_T02; parent_gene=GRMZM2G410916	GRMZM2G410916_P02	TRUE	TRUE	sLDGLATPDFTR	95%	n+304 (+304)	45.23	25.00
2687	seq=translation; coord=6:165704660..165707098:-1; parent_transcript=GRMZM2G354604_T01; parent_gene=GRMZM2G354604	GRMZM2G354604_P01,GRMZM2G354604_P02, GRMZM2G354604_P03	TRUE	TRUE	dGYYIHGQcAIIMFDVTSR	95%	n+304 (+304), Carbamidomethyl (+57)	55.10	25.00
2688	seq=translation; coord=6:165704660..165707098:-1; parent_transcript=GRMZM2G354604_T01; parent_gene=GRMZM2G354604	GRMZM2G354604_P01,GRMZM2G354604_P02, GRMZM2G354604_P03	TRUE	TRUE	dGYyIHGQcAIIMFDVTSR	95%	n+304 (+304), iTRAQ8plex (+304), Carbamidomethyl (+57)	44.26	25.00
2689	seq=translation; coord=6:165704660..165707098:-1; parent_transcript=GRMZM2G354604_T01; parent_gene=GRMZM2G354604	GRMZM2G354604_P01,GRMZM2G354604_P02, GRMZM2G354604_P03	TRUE	TRUE	fYcWDTAGQEk	95%	n+304 (+304), Carbamidomethyl (+57), K+304 (+304)	36.63	25.00
2690	seq=translation; coord=6:165704660..165707098:-1; parent_transcript=GRMZM2G354604_T01; parent_gene=GRMZM2G354604	GRMZM2G354604_P01,GRMZM2G354604_P02, GRMZM2G354604_P03	TRUE	TRUE	hLTGEFEk	95%	n+304 (+304), K+304 (+304)	32.33	25.79
2691	seq=translation; coord=6:165704660..165707098:-1; parent_transcript=GRMZM2G354604_T01; parent_gene=GRMZM2G354604	GRMZM2G354604_P01,GRMZM2G354604_P02, GRMZM2G354604_P03	TRUE	TRUE	lVIVGDGGTGk	95%	n+304 (+304), K+304 (+304)	33.06	25.48
2692	seq=translation; coord=6:165704660..165707098:-1; parent_transcript=GRMZM2G354604_T01; parent_gene=GRMZM2G354604	GRMZM2G354604_P01,GRMZM2G354604_P02, GRMZM2G354604_P03	TRUE	TRUE	nLQYYEISAk	95%	n+304 (+304), K+304 (+304)	32.09	25.16

2693	seq=translation; coord=6:165704660..165707098:-1; parent_transcript=GRMZM2G354604_T01; parent_gene=GRMZM2G354604	GRMZM2G354604_P01,GRMZM2G354604_P02, GRMZM2G354604_P03	TRUE	TRUE	sNYNFEkPFLYLAR	95%	n+304 (+304), K+304 (+304)	31.50	25.85
2694	seq=translation; coord=6:165704660..165707098:-1; parent_transcript=GRMZM2G354604_T01; parent_gene=GRMZM2G354604	GRMZM2G354604_P01,GRMZM2G354604_P02, GRMZM2G354604_P03	TRUE	TRUE	vcENIPIVLcGNk	95%	n+304 (+304), Carbamidomethyl (+57), Carbamidomethyl (+57), K+304 (+304)	44.09	26.25
2695	seq=translation; coord=7:169267359..169269362:1; parent_transcript=GRMZM2G013652_T01; parent_gene=GRMZM2G013652	GRMZM2G013652_P01,GRMZM2G013652_P03	TRUE	TRUE	aGNLIPVALk	95%	n+304 (+304), K+304 (+304)	39.27	25.00
2696	seq=translation; coord=7:169267359..169269362:1; parent_transcript=GRMZM2G013652_T01; parent_gene=GRMZM2G013652	GRMZM2G013652_P01,GRMZM2G013652_P03	TRUE	TRUE	eGDTVLLPEYGGTEVk	95%	n+304 (+304), K+304 (+304)	35.35	25.25
2697	seq=translation; coord=7:169267359..169269362:1; parent_transcript=GRMZM2G013652_T01; parent_gene=GRMZM2G013652	GRMZM2G013652_P01,GRMZM2G013652_P03	TRUE	TRUE	eHDILGTLVD	95%	n+304 (+304)	43.18	25.00
2698	seq=translation; coord=7:169267359..169269362:1; parent_transcript=GRMZM2G013652_T01; parent_gene=GRMZM2G013652	GRMZM2G013652_P01,GRMZM2G013652_P03	TRUE	TRUE	eYLLFR	87%	n+304 (+304)	25.54	25.00
2699	seq=translation; coord=7:169267359..169269362:1; parent_transcript=GRMZM2G013652_T01; parent_gene=GRMZM2G013652	GRMZM2G013652_P01,GRMZM2G013652_P03	TRUE	TRUE	vVAVGPGER	88%	n+304 (+304)	28.28	27.28
2700	seq=translation; coord=6:31411848..31415600:-1; parent_transcript=GRMZM2G099186_T02; parent_gene=GRMZM2G099186	GRMZM2G099186_P02,GRMZM2G175419_P01	TRUE	TRUE	dADDAQYNLDGR	95%	n+304 (+304)	50.99	25.00
2701	seq=translation; coord=6:31411848..31415600:-1; parent_transcript=GRMZM2G099186_T02; parent_gene=GRMZM2G099186	GRMZM2G099186_P02,GRMZM2G175419_P01	TRUE	TRUE	dLEYLFSk	95%	n+304 (+304), K+304 (+304)	35.61	25.45
2702	seq=translation; coord=6:31411848..31415600:-1; parent_transcript=GRMZM2G099186_T02; parent_gene=GRMZM2G099186	GRMZM2G099186_P02,GRMZM2G175419_P01	TRUE	TRUE	dYAFIEFSDPR	94%	n+304 (+304)	30.42	25.00
2703	seq=translation; coord=6:31411848..31415600:-1; parent_transcript=GRMZM2G099186_T02; parent_gene=GRMZM2G099186	GRMZM2G099186_P02,GRMZM2G175419_P01	TRUE	TRUE	iIVEFAk	92%	n+304 (+304), K+304 (+304)	28.98	25.00
2704	seq=translation; coord=10:148535505..148536885:1; parent_transcript=GRMZM2G011513_T01; parent_gene=GRMZM2G011513	GRMZM2G011513_P01	TRUE	TRUE	gLEDTDFLNNMDPFVILTcR	95%	n+304 (+304), Carbamidomethyl (+57)	40.60	25.00
2705	seq=translation; coord=10:148535505..148536885:1; parent_transcript=GRMZM2G011513_T01; parent_gene=GRMZM2G011513	GRMZM2G011513_P01	TRUE	TRUE	gLEDTDFLNNmDPFVILTcR	95%	n+304 (+304), Oxidation (+16), Carbamidomethyl (+57)	43.09	25.00
2706	seq=translation; coord=10:148535505..148536885:1; parent_transcript=GRMZM2G011513_T01; parent_gene=GRMZM2G011513	GRMZM2G011513_P01	TRUE	TRUE	lALTFTPAAETR	95%	n+304 (+304)	53.05	25.49
2707	seq=translation; coord=10:148535505..148536885:1; parent_transcript=GRMZM2G011513_T01; parent_gene=GRMZM2G011513	GRMZM2G011513_P01	TRUE	TRUE	lEVLLVSAk	95%	n+304 (+304), K+304 (+304)	41.49	25.00
2708	seq=translation; coord=10:148535505..148536885:1; parent_transcript=GRMZM2G011513_T01; parent_gene=GRMZM2G011513	GRMZM2G011513_P01	TRUE	TRUE	rPDDNEEGPPYSSWS	95%	n+304 (+304)	47.05	25.00
2709	seq=translation; coord=2:61490885..61494711:-1; parent_transcript=GRMZM2G134668_T02; parent_gene=GRMZM2G134668	GRMZM2G134668_P02	TRUE	TRUE	dGTVVLQFEVR	95%	n+304 (+304)	46.39	25.65

2710	seq=translation; coord=2:61490885..61494711:-1; parent_transcript=GRMZM2G134668_T02; parent_gene=GRMZM2G134668	GRMZM2G134668_P02	TRUE	TRUE	eLDEPVTLk	95%	n+304 (+304), K+304 (+304)	42.90	25.55
2711	seq=translation; coord=2:61490885..61494711:-1; parent_transcript=GRMZM2G134668_T02; parent_gene=GRMZM2G134668	GRMZM2G134668_P02	TRUE	TRUE	iLVDGEEk	95%	n+304 (+304), K+304 (+304)	30.65	26.59
2712	seq=translation; coord=1:58418770..58420264:-1; parent_transcript=GRMZM2G075290_T01; parent_gene=GRMZM2G075290	GRMZM2G075290_P01	TRUE	TRUE	iVAEFGLQEVGHIR	95%	n+304 (+304)	78.05	26.95
2713	seq=translation; coord=1:58418770..58420264:-1; parent_transcript=GRMZM2G075290_T01; parent_gene=GRMZM2G075290	GRMZM2G075290_P01	TRUE	TRUE	lALGGPPPVGAR	89%	n+304 (+304)	26.56	25.00
2714	seq=translation; coord=1:58418770..58420264:-1; parent_transcript=GRMZM2G075290_T01; parent_gene=GRMZM2G075290	GRMZM2G075290_P01	TRUE	TRUE	lGAEGAIcTNVLSADR	89%	n+304 (+304), Carbamidomethyl (+57)	25.14	25.00
2715	seq=translation; coord=1:58418770..58420264:-1; parent_transcript=GRMZM2G075290_T01; parent_gene=GRMZM2G075290	GRMZM2G075290_P01	TRUE	TRUE	lLAGLLGVEAAQDAVFR	95%	n+304 (+304)	54.27	25.31
2716	seq=translation; coord=1:58418770..58420264:-1; parent_transcript=GRMZM2G075290_T01; parent_gene=GRMZM2G075290	GRMZM2G075290_P01	TRUE	TRUE	rLGAEGAIcTNVLSADR	95%	n+304 (+304), Carbamidomethyl (+57)	46.89	25.61
2717	seq=translation; coord=1:58418770..58420264:-1; parent_transcript=GRMZM2G075290_T01; parent_gene=GRMZM2G075290	GRMZM2G075290_P01	TRUE	TRUE	tPAELLSILYLTGDER	92%	n+304 (+304)	28.41	26.48
2718	seq=translation; coord=1:58418770..58420264:-1; parent_transcript=GRMZM2G075290_T01; parent_gene=GRMZM2G075290	GRMZM2G075290_P01	TRUE	TRUE	vMDEAFGTR	91%	n+304 (+304)	27.91	25.00
2719	seq=translation; coord=7:171733893..171737098:-1; parent_transcript=GRMZM2G025992_T01; parent_gene=GRMZM2G025992	GRMZM2G025992_P01,GRMZM2G025992_P03, GRMZM2G025992_P04,GRMZM2G025992_P05	TRUE	TRUE	aVAVLAGTDVk	95%	n+304 (+304), K+304 (+304)	54.69	25.29
2720	seq=translation; coord=7:171733893..171737098:-1; parent_transcript=GRMZM2G025992_T01; parent_gene=GRMZM2G025992	GRMZM2G025992_P01,GRMZM2G025992_P03, GRMZM2G025992_P04,GRMZM2G025992_P05	TRUE	TRUE	hAGDLGNVTAGEDGVVNVNITDSQIPLAGPHSIIGR	95%	n+304 (+304)	31.87	25.00
2721	seq=translation; coord=4:217021920..217028774:-1; parent_transcript=GRMZM2G180578_T01; parent_gene=GRMZM2G180578	GRMZM2G180578_P01	TRUE	TRUE	dELGQETLLNLLLR	95%	n+304 (+304)	70.62	26.05
2722	seq=translation; coord=4:217021920..217028774:-1; parent_transcript=GRMZM2G180578_T01; parent_gene=GRMZM2G180578	GRMZM2G180578_P01	TRUE	TRUE	hMAEDDDDDF	95%	n+304 (+304)	38.36	25.00
2723	seq=translation; coord=4:217021920..217028774:-1; parent_transcript=GRMZM2G180578_T01; parent_gene=GRMZM2G180578	GRMZM2G180578_P01	TRUE	TRUE	iSLADIAk	93%	n+304 (+304), K+304 (+304)	27.07	25.00
2724	seq=translation; coord=4:217021920..217028774:-1; parent_transcript=GRMZM2G180578_T01; parent_gene=GRMZM2G180578	GRMZM2G180578_P01	TRUE	TRUE	vGDLELFR	92%	n+304 (+304)	29.07	25.00
2725	seq=translation; coord=4:217021920..217028774:-1; parent_transcript=GRMZM2G180578_T01; parent_gene=GRMZM2G180578	GRMZM2G180578_P01	TRUE	TRUE	yLFYLGk	95%	n+304 (+304), K+304 (+304)	30.36	25.00
2726	seq=translation; coord=3:226624022..226632817:-1; parent_transcript=GRMZM2G017086_T01; parent_gene=GRMZM2G017086	GRMZM2G017086_P01,GRMZM2G017086_P03	TRUE	TRUE	dDADWSTLGVk	95%	n+304 (+304), K+304 (+304)	61.44	25.00
2727	seq=translation; coord=3:226624022..226632817:-1; parent_transcript=GRMZM2G017086_T01; parent_gene=GRMZM2G017086	GRMZM2G017086_P01,GRMZM2G017086_P03	TRUE	TRUE	eLFPGIEIDTSQPPIVFk	95%	n+304 (+304), K+304 (+304)	30.48	25.48

2728	seq=translation; coord=3:226624022..226632817:-1; parent_transcript=GRMZM2G017086_T01; parent_gene=GRMZM2G017086	GRMZM2G017086_P01,GRMZM2G017086_P03	TRUE	TRUE	lMMIGTADEIVk	95%	n+304 (+304), K+304 (+304)	30.43	25.51
2729	seq=translation; coord=3:226624022..226632817:-1; parent_transcript=GRMZM2G017086_T01; parent_gene=GRMZM2G017086	GRMZM2G017086_P01,GRMZM2G017086_P03	TRUE	TRUE	qLFGIDLVSR	94%	n+304 (+304)	31.13	25.00
2730	seq=translation; coord=3:226624022..226632817:-1; parent_transcript=GRMZM2G017086_T01; parent_gene=GRMZM2G017086	GRMZM2G017086_P01,GRMZM2G017086_P03	TRUE	TRUE	qLTGVYDLVAVLTHk	95%	n+304 (+304), K+304 (+304)	59.63	25.00
2731	seq=translation; coord=3:226624022..226632817:-1; parent_transcript=GRMZM2G017086_T01; parent_gene=GRMZM2G017086	GRMZM2G017086_P01,GRMZM2G017086_P03	TRUE	TRUE	qLTGVYDLVAVLTHk	91%	Pyro-cmC (-17), n+304 (+304), K+304 (+304)	28.66	26.60
2732	seq=translation; coord=3:226624022..226632817:-1; parent_transcript=GRMZM2G017086_T01; parent_gene=GRMZM2G017086	GRMZM2G017086_P01,GRMZM2G017086_P03	TRUE	TRUE	sALLSYSDTVR	95%	n+304 (+304)	38.68	26.05
2733	seq=translation; coord=3:226624022..226632817:-1; parent_transcript=GRMZM2G017086_T01; parent_gene=GRMZM2G017086	GRMZM2G017086_P01,GRMZM2G017086_P03	TRUE	TRUE	wIEFDDDNPNIR	95%	n+304 (+304)	44.66	25.00
2734	seq=translation; coord=2:183929119..183934914:-1; parent_transcript=GRMZM2G399284_T01; parent_gene=GRMZM2G399284	GRMZM2G399284_P01,GRMZM2G399284_P03	TRUE	TRUE	eDDIIGILDSDDVk	95%	n+304 (+304), K+304 (+304)	50.95	25.01
2735	seq=translation; coord=2:183929119..183934914:-1; parent_transcript=GRMZM2G399284_T01; parent_gene=GRMZM2G399284	GRMZM2G399284_P01,GRMZM2G399284_P03	TRUE	TRUE	gEDGEYIVLR	94%	n+304 (+304)	30.31	25.00
2736	seq=translation; coord=2:183929119..183934914:-1; parent_transcript=GRMZM2G399284_T01; parent_gene=GRMZM2G399284	GRMZM2G399284_P01,GRMZM2G399284_P03	TRUE	TRUE	iEISVPVGAQVVYSk	94%	n+304 (+304), K+304 (+304)	30.73	25.00
2737	seq=translation; coord=2:183929119..183934914:-1; parent_transcript=GRMZM2G399284_T01; parent_gene=GRMZM2G399284	GRMZM2G399284_P01,GRMZM2G399284_P03	TRUE	TRUE	nPLSITPGSNVMYSk	95%	n+304 (+304), K+304 (+304)	36.39	26.74
2738	seq=translation; coord=2:183929119..183934914:-1; parent_transcript=GRMZM2G399284_T01; parent_gene=GRMZM2G399284	GRMZM2G399284_P01,GRMZM2G399284_P03	TRUE	TRUE	sDGGILLPVSVQTRPQGGEIVAVGEGR	92%	n+304 (+304)	26.79	25.00
2739	seq=translation; coord=2:183929119..183934914:-1; parent_transcript=GRMZM2G399284_T01; parent_gene=GRMZM2G399284	GRMZM2G399284_P01,GRMZM2G399284_P03	TRUE	TRUE	yAGSEFkGEDGEYIVLR	93%	n+304 (+304), K+304 (+304)	27.66	25.26
2740	seq=translation; coord=4:170332797..170339707:-1; parent_transcript=GRMZM2G008410_T01; parent_gene=GRMZM2G008410	GRMZM2G008410_P01	TRUE	TRUE	aMELILEk	95%	n+304 (+304), K+304 (+304)	36.53	25.82
2741	seq=translation; coord=4:170332797..170339707:-1; parent_transcript=GRMZM2G008410_T01; parent_gene=GRMZM2G008410	GRMZM2G008410_P01	TRUE	TRUE	fLVSNTAAGcIIGk	95%	n+304 (+304), Carbamidomethyl (+57), K+304 (+304)	46.86	25.65
2742	seq=translation; coord=4:170332797..170339707:-1; parent_transcript=GRMZM2G008410_T01; parent_gene=GRMZM2G008410	GRMZM2G008410_P01	TRUE	TRUE	gDYISGTSDR	90%	n+304 (+304)	26.57	25.00
2743	seq=translation; coord=4:170332797..170339707:-1; parent_transcript=GRMZM2G008410_T01; parent_gene=GRMZM2G008410	GRMZM2G008410_P01	TRUE	TRUE	iIMVSGLFDEVMk	95%	n+304 (+304), K+304 (+304)	60.31	25.20
2744	seq=translation; coord=4:170332797..170339707:-1; parent_transcript=GRMZM2G008410_T01; parent_gene=GRMZM2G008410	GRMZM2G008410_P01	TRUE	TRUE	sPASNEAQESLTIGIADEHIGAVVGR	95%	n+304 (+304)	90.16	25.00
2745	seq=translation; coord=4:170332797..170339707:-1; parent_transcript=GRMZM2G008410_T01; parent_gene=GRMZM2G008410	GRMZM2G008410_P01	TRUE	TRUE	vTITGTPEAIR	91%	n+304 (+304)	27.86	25.00

2746	seq=translation; coord=3:218785554..218795824:-1; parent_transcript=GRMZM2G090779_T01; parent_gene=GRMZM2G090779	GRMZM2G090779_P01	TRUE	TRUE	aDMDALPLQESVEWEHk	95%	n+304 (+304), K+304 (+304)	54.21	25.00
2747	seq=translation; coord=3:218785554..218795824:-1; parent_transcript=GRMZM2G090779_T01; parent_gene=GRMZM2G090779	GRMZM2G090779_P01	TRUE	TRUE	dRPFFPPTINSPELHDFFVNVAGEMVGSR	95%	n+304 (+304)	38.35	25.00
2748	seq=translation; coord=3:218785554..218795824:-1; parent_transcript=GRMZM2G090779_T01; parent_gene=GRMZM2G090779	GRMZM2G090779_P01	TRUE	TRUE	eADPLDSQVVTVGk	95%	n+304 (+304), K+304 (+304)	54.78	26.30
2749	seq=translation; coord=3:218785554..218795824:-1; parent_transcript=GRMZM2G090779_T01; parent_gene=GRMZM2G090779	GRMZM2G090779_P01	TRUE	TRUE	ePAFADWMVGVR	95%	n+304 (+304)	48.21	25.00
2750	seq=translation; coord=3:218785554..218795824:-1; parent_transcript=GRMZM2G090779_T01; parent_gene=GRMZM2G090779	GRMZM2G090779_P01	TRUE	TRUE	fQGGGAFNVIPDSVTIGGTFR	95%	n+304 (+304)	35.09	25.40
2751	seq=translation; coord=3:218785554..218795824:-1; parent_transcript=GRMZM2G090779_T01; parent_gene=GRMZM2G090779	GRMZM2G090779_P01	TRUE	TRUE	hPFAVTGVVATVGTGGPPFVALR	95%	n+304 (+304)	43.85	25.00
2752	seq=translation; coord=3:218785554..218795824:-1; parent_transcript=GRMZM2G090779_T01; parent_gene=GRMZM2G090779	GRMZM2G090779_P01	TRUE	TRUE	iEEVIVSQASVQR	95%	n+304 (+304)	30.54	25.51
2753	seq=translation; coord=3:218785554..218795824:-1; parent_transcript=GRMZM2G090779_T01; parent_gene=GRMZM2G090779	GRMZM2G090779_P01	TRUE	TRUE	iHENPELGYEEFQTSELVR	95%	n+304 (+304)	53.97	25.00
2754	seq=translation; coord=3:218785554..218795824:-1; parent_transcript=GRMZM2G090779_T01; parent_gene=GRMZM2G090779	GRMZM2G090779_P01	TRUE	TRUE	qQPAAATADk	95%	Pyro-cmC (-17), n+304 (+304), K+304 (+304)	35.78	25.00
2755	seq=translation; coord=3:218785554..218795824:-1; parent_transcript=GRMZM2G090779_T01; parent_gene=GRMZM2G090779	GRMZM2G090779_P01	TRUE	TRUE	qQPAAATADkAETHDEL	91%	Pyro-cmC (-17), n+304 (+304), K+304 (+304)	26.98	25.00
2756	seq=translation; coord=8:171852196..171855863:-1; parent_transcript=GRMZM2G159643_T01; parent_gene=GRMZM2G159643	GRMZM2G159643_P01	TRUE	TRUE	lAEEIQk	86%	n+304 (+304), K+304 (+304)	27.33	27.19
2757	seq=translation; coord=8:171852196..171855863:-1; parent_transcript=GRMZM2G159643_T01; parent_gene=GRMZM2G159643	GRMZM2G159643_P01	TRUE	TRUE	tWQVPETLPEEVLGk	95%	n+304 (+304), K+304 (+304)	40.54	25.84
2758	seq=translation; coord=2:10210584..10215840:-1; parent_transcript=GRMZM5G817886_T02; parent_gene=GRMZM5G817886	GRMZM5G817886_P02	TRUE	TRUE	aLADSPIIGEIR	95%	n+304 (+304)	45.31	26.05
2759	seq=translation; coord=2:10210584..10215840:-1; parent_transcript=GRMZM5G817886_T02; parent_gene=GRMZM5G817886	GRMZM5G817886_P02	TRUE	TRUE	kYDILFIADEVITAFGR	95%	K+304 (+304), n+304 (+304)	42.05	25.00
2760	seq=translation; coord=2:10210584..10215840:-1; parent_transcript=GRMZM5G817886_T02; parent_gene=GRMZM5G817886	GRMZM5G817886_P02	TRUE	TRUE	lATNLENLILk	95%	n+304 (+304), K+304 (+304)	31.60	25.00
2761	seq=translation; coord=2:10210584..10215840:-1; parent_transcript=GRMZM5G817886_T02; parent_gene=GRMZM5G817886	GRMZM5G817886_P02	TRUE	TRUE	sPTDLFPAEWGVGAIFGEEcQk	95%	n+304 (+304), Carbamidomethyl (+57), K+304 (+304)	52.84	25.00
2762	seq=translation; coord=2:10210584..10215840:-1; parent_transcript=GRMZM5G817886_T02; parent_gene=GRMZM5G817886	GRMZM5G817886_P02	TRUE	TRUE	tTkPSLDLAQEILSMFTAR	95%	n+304 (+304), K+304 (+304)	34.14	25.00
2763	seq=translation; coord=2:10210584..10215840:-1; parent_transcript=GRMZM5G817886_T02; parent_gene=GRMZM5G817886	GRMZM5G817886_P02	TRUE	TRUE	yHLPGETEEDFATR	95%	n+304 (+304), iTRAQ8plex (+304)	39.39	25.00

2764	seq=translation; coord=2:10210584..10215840:-1; parent_transcript=GRMZM5G817886_T02; parent_gene=GRMZM5G817886	GRMZM5G817886_P02	TRUE	TRUE	yLDSLAGLWcTALGGSEPR	95%	n+304 (+304), Carbamidomethyl (+57)	29.59	25.00
2765	seq=translation; coord=1:246702682..246710172:-1; parent_transcript=GRMZM2G146589_T01; parent_gene=GRMZM2G146589	GRMZM2G146589_P01,GRMZM2G146589_P02, GRMZM2G146589_P03,GRMZM2G146589_P04	TRUE	TRUE	aSAADDDDMDPTQYYENR	95%	n+304 (+304)	34.52	25.00
2766	seq=translation; coord=1:246702682..246710172:-1; parent_transcript=GRMZM2G146589_T01; parent_gene=GRMZM2G146589	GRMZM2G146589_P01,GRMZM2G146589_P02, GRMZM2G146589_P03,GRMZM2G146589_P04	TRUE	TRUE	fPVGISVPEYIEk	90%	n+304 (+304), K+304 (+304)	25.37	25.00
2767	seq=translation; coord=1:246702682..246710172:-1; parent_transcript=GRMZM2G146589_T01; parent_gene=GRMZM2G146589	GRMZM2G146589_P01,GRMZM2G146589_P02, GRMZM2G146589_P03,GRMZM2G146589_P04	TRUE	TRUE	gEGSAVPVPWTPGMGR	95%	n+304 (+304)	44.46	25.00
2768	seq=translation; coord=1:246702682..246710172:-1; parent_transcript=GRMZM2G146589_T01; parent_gene=GRMZM2G146589	GRMZM2G146589_P01,GRMZM2G146589_P02, GRMZM2G146589_P03,GRMZM2G146589_P04	TRUE	TRUE	lFFYDLYGGGMk	95%	n+304 (+304), K+304 (+304)	49.76	25.26
2769	seq=translation; coord=1:246702682..246710172:-1; parent_transcript=GRMZM2G146589_T01; parent_gene=GRMZM2G146589	GRMZM2G146589_P01,GRMZM2G146589_P02, GRMZM2G146589_P03,GRMZM2G146589_P04	TRUE	TRUE	lTDVAEcLAGR	94%	n+304 (+304), Carbamidomethyl (+57)	31.32	25.00
2770	seq=translation; coord=1:246702682..246710172:-1; parent_transcript=GRMZM2G146589_T01; parent_gene=GRMZM2G146589	GRMZM2G146589_P01,GRMZM2G146589_P02, GRMZM2G146589_P03,GRMZM2G146589_P04	TRUE	TRUE	lTMLLTDSQNIk	95%	n+304 (+304), K+304 (+304)	30.69	25.00
2771	seq=translation; coord=1:246702682..246710172:-1; parent_transcript=GRMZM2G146589_T01; parent_gene=GRMZM2G146589	GRMZM2G146589_P01,GRMZM2G146589_P02, GRMZM2G146589_P03,GRMZM2G146589_P04	TRUE	TRUE	tSELDEAEFSk	93%	n+304 (+304), K+304 (+304)	26.36	25.00
2772	seq=translation; coord=7:123475099..123479587:1; parent_transcript=GRMZM5G824944_T01; parent_gene=GRMZM5G824944	GRMZM5G824944_P01,GRMZM5G824944_P02	TRUE	TRUE	hFADIMFR	95%	n+304 (+304)	32.15	25.00
2773	seq=translation; coord=7:123475099..123479587:1; parent_transcript=GRMZM5G824944_T01; parent_gene=GRMZM5G824944	GRMZM5G824944_P01,GRMZM5G824944_P02	TRUE	TRUE	lDIDPESITWR	95%	n+304 (+304)	31.86	25.01
2774	seq=translation; coord=7:123475099..123479587:1; parent_transcript=GRMZM5G824944_T01; parent_gene=GRMZM5G824944	GRMZM5G824944_P01,GRMZM5G824944_P02	TRUE	TRUE	sGEPITADDLGVGGALTVLMk	95%	n+304 (+304), K+304 (+304)	49.19	25.00
2775	seq=translation; coord=7:123475099..123479587:1; parent_transcript=GRMZM5G824944_T01; parent_gene=GRMZM5G824944	GRMZM5G824944_P01,GRMZM5G824944_P02	TRUE	TRUE	tDPNELTPDEIR	95%	n+304 (+304)	53.38	25.00
2776	seq=translation; coord=7:123475099..123479587:1; parent_transcript=GRMZM5G824944_T01; parent_gene=GRMZM5G824944	GRMZM5G824944_P01,GRMZM5G824944_P02	TRUE	TRUE	vLLSVLDELk	95%	n+304 (+304), K+304 (+304)	43.96	25.00
2777	seq=translation; coord=1:220892398..220897302:-1; parent_transcript=GRMZM2G078022_T03; parent_gene=GRMZM2G078022	GRMZM2G078022_P01,GRMZM2G078022_P02, GRMZM2G078022_P03	TRUE	TRUE	aPHFVk	95%	n+304 (+304), K+304 (+304)	37.84	25.00
2778	seq=translation; coord=1:220892398..220897302:-1; parent_transcript=GRMZM2G078022_T03; parent_gene=GRMZM2G078022	GRMZM2G078022_P01,GRMZM2G078022_P02, GRMZM2G078022_P03	TRUE	TRUE	aVEAEETESAETk	95%	n+304 (+304), K+304 (+304)	77.29	25.00
2779	seq=translation; coord=1:220892398..220897302:-1; parent_transcript=GRMZM2G078022_T03; parent_gene=GRMZM2G078022	GRMZM2G078022_P01,GRMZM2G078022_P02, GRMZM2G078022_P03	TRUE	TRUE	sIFcIVEk	95%	n+304 (+304), Carbamidomethyl (+57), K+304 (+304)	34.36	26.88
2780	seq=translation; coord=1:220892398..220897302:-1; parent_transcript=GRMZM2G078022_T03; parent_gene=GRMZM2G078022	GRMZM2G078022_P01,GRMZM2G078022_P02, GRMZM2G078022_P03	TRUE	TRUE	vNLDPDGVFTFSGSAGTNLyELk	95%	n+304 (+304), iTRAQ8plex (+304), K+304 (+304)	26.11	25.00
2781	seq=translation; coord=1:220892398..220897302:-1; parent_transcript=GRMZM2G078022_T03; parent_gene=GRMZM2G078022	GRMZM2G078022_P01,GRMZM2G078022_P02, GRMZM2G078022_P03	TRUE	TRUE	vYITVQLPDAk	95%	n+304 (+304), K+304 (+304)	39.89	25.00

2782	seq=translation; coord=7:136570996..136574421:1; parent_transcript=GRMZM2G074386_T01; parent_gene=GRMZM2G074386	GRMZM2G074386_P01	TRUE	TRUE	dVFASATER	93%	n+304 (+304)	30.01	25.00
2783	seq=translation; coord=7:136570996..136574421:1; parent_transcript=GRMZM2G074386_T01; parent_gene=GRMZM2G074386	GRMZM2G074386_P01	TRUE	TRUE	eYIDLR	95%	n+304 (+304)	30.64	25.00
2784	seq=translation; coord=7:136570996..136574421:1; parent_transcript=GRMZM2G074386_T01; parent_gene=GRMZM2G074386	GRMZM2G074386_P01	TRUE	TRUE	fFPYYAFNVLGGLDSEGk	95%	n+304 (+304), K+304 (+304)	61.99	25.17
2785	seq=translation; coord=7:136570996..136574421:1; parent_transcript=GRMZM2G074386_T01; parent_gene=GRMZM2G074386	GRMZM2G074386_P01	TRUE	TRUE	lSVGYSILTR	95%	n+304 (+304)	34.71	27.23
2786	seq=translation; coord=7:136570996..136574421:1; parent_transcript=GRMZM2G074386_T01; parent_gene=GRMZM2G074386	GRMZM2G074386_P01	TRUE	TRUE	mScPAMAQLLSNTLYYk	95%	n+304 (+304), Carbamidomethyl (+57), K+304 (+304)	47.19	25.22
2787	seq=translation; coord=1:20418930..20420739:-1; parent_transcript=GRMZM2G134367_T01; parent_gene=GRMZM2G134367	GRMZM2G134367_P01	TRUE	TRUE	vAGAAADLLHAASQYGk	95%	n+304 (+304), K+304 (+304)	34.49	25.00
2788	seq=translation; coord=1:20418930..20420739:-1; parent_transcript=GRMZM2G134367_T01; parent_gene=GRMZM2G134367	GRMZM2G134367_P01	TRUE	TRUE	yEDEDDYR	90%	n+304 (+304)	27.34	25.00
2789	seq=translation; coord=1:20418930..20420739:-1; parent_transcript=GRMZM2G134367_T01; parent_gene=GRMZM2G134367	GRMZM2G134367_P01	TRUE	TRUE	yEEDDEYR	95%	n+304 (+304)	31.69	25.00
2790	seq=translation; coord=1:20418930..20420739:-1; parent_transcript=GRMZM2G134367_T01; parent_gene=GRMZM2G134367	GRMZM2G134367_P01	TRUE	TRUE	yEEEEGYk	95%	n+304 (+304), K+304 (+304)	41.95	25.00
2791	seq=translation; coord=1:20418930..20420739:-1; parent_transcript=GRMZM2G134367_T01; parent_gene=GRMZM2G134367	GRMZM2G134367_P01	TRUE	TRUE	yEQEDNYk	93%	n+304 (+304), K+304 (+304)	29.38	25.00
2792	seq=translation; coord=8:79300397..79307702:1; parent_transcript=GRMZM2G073774_T01; parent_gene=GRMZM2G073774	GRMZM2G073774_P01,GRMZM2G152958_P01, GRMZM2G152958_P02	TRUE	TRUE	aLGVDILTGVGTIVGk	95%	n+304 (+304), K+304 (+304)	60.34	25.00
2793	seq=translation; coord=8:79300397..79307702:1; parent_transcript=GRMZM2G073774_T01; parent_gene=GRMZM2G073774	GRMZM2G073774_P01,GRMZM2G152958_P01, GRMZM2G152958_P02	TRUE	TRUE	gFVPVDER	95%	n+304 (+304)	41.58	25.55
2794	seq=translation; coord=8:79300397..79307702:1; parent_transcript=GRMZM2G073774_T01; parent_gene=GRMZM2G073774	GRMZM2G073774_P01,GRMZM2G152958_P01, GRMZM2G152958_P02	TRUE	TRUE	gLGLENINVVTQR	95%	n+304 (+304)	65.56	26.11
2795	seq=translation; coord=8:79300397..79307702:1; parent_transcript=GRMZM2G073774_T01; parent_gene=GRMZM2G073774	GRMZM2G073774_P01,GRMZM2G152958_P01, GRMZM2G152958_P02	TRUE	TRUE	lMLAHAASAQGISVVEQISGk	95%	n+304 (+304), K+304 (+304)	27.25	25.00
2796	seq=translation; coord=6:75746632..75749530:-1; parent_transcript=GRMZM2G130528_T01; parent_gene=GRMZM2G130528	GRMZM2G130528_P01	TRUE	TRUE	eGANMAITGATAMDAPFFR	95%	n+304 (+304)	43.89	25.00
2797	seq=translation; coord=6:75746632..75749530:-1; parent_transcript=GRMZM2G130528_T01; parent_gene=GRMZM2G130528	GRMZM2G130528_P01	TRUE	TRUE	eGANmAITGATAMDAPFFR	95%	n+304 (+304), Oxidation (+16)	73.73	25.00
2798	seq=translation; coord=6:75746632..75749530:-1; parent_transcript=GRMZM2G130528_T01; parent_gene=GRMZM2G130528	GRMZM2G130528_P01	TRUE	TRUE	eGANMAITGATAmDAPFFR	95%	n+304 (+304), Oxidation (+16)	33.47	25.00

2799	seq=translation; coord=6:75746632..75749530:-1; parent_transcript=GRMZM2G130528_T01; parent_gene=GRMZM2G130528	GRMZM2G130528_P01	TRUE	TRUE	nPGSYGFSTAFQTccGSGGGk	95%	n+304 (+304), Carbamidomethyl (+57), Carbamidomethyl (+57), K+304 (+304)	54.83	25.00
2800	seq=translation; coord=6:75746632..75749530:-1; parent_transcript=GRMZM2G130528_T01; parent_gene=GRMZM2G130528	GRMZM2G130528_P01	TRUE	TRUE	vIPDFLcSk	95%	n+304 (+304), Carbamidomethyl (+57), K+304 (+304)	36.40	26.05
2801	seq=translation; coord=1:200480447..200483616:-1; parent_transcript=GRMZM2G157018_T01; parent_gene=GRMZM2G157018	GRMZM2G157018_P01	TRUE	TRUE	eAFESIEIPk	95%	n+304 (+304), K+304 (+304)	45.02	25.34
2802	seq=translation; coord=1:200480447..200483616:-1; parent_transcript=GRMZM2G157018_T01; parent_gene=GRMZM2G157018	GRMZM2G157018_P01	TRUE	TRUE	eLAEMEEMR	87%	n+304 (+304)	25.87	25.00
2803	seq=translation; coord=1:200480447..200483616:-1; parent_transcript=GRMZM2G157018_T01; parent_gene=GRMZM2G157018	GRMZM2G157018_P01	TRUE	TRUE	fDAVIAELk	95%	n+304 (+304), K+304 (+304)	31.60	25.07
2804	seq=translation; coord=1:200480447..200483616:-1; parent_transcript=GRMZM2G157018_T01; parent_gene=GRMZM2G157018	GRMZM2G157018_P01	TRUE	TRUE	fSQEPQPIDWEYYR	95%	n+304 (+304)	33.74	25.00
2805	seq=translation; coord=1:200480447..200483616:-1; parent_transcript=GRMZM2G157018_T01; parent_gene=GRMZM2G157018	GRMZM2G157018_P01	TRUE	TRUE	iSTMTADDYFEk	95%	n+304 (+304), K+304 (+304)	34.88	25.00
2806	seq=translation; coord=1:200480447..200483616:-1; parent_transcript=GRMZM2G157018_T01; parent_gene=GRMZM2G157018	GRMZM2G157018_P01	TRUE	TRUE	tIIDWDGMAk	95%	n+304 (+304), K+304 (+304)	69.12	25.08
2807	seq=translation; coord=9:48437559..48441823:-1; parent_transcript=GRMZM2G049811_T01; parent_gene=GRMZM2G049811	GRMZM2G049811_P01	TRUE	TRUE	fEEDLDAMLPk	95%	n+304 (+304), K+304 (+304)	54.89	25.59
2808	seq=translation; coord=9:48437559..48441823:-1; parent_transcript=GRMZM2G049811_T01; parent_gene=GRMZM2G049811	GRMZM2G049811_P01	TRUE	TRUE	gEDFPVQNYIVk	95%	n+304 (+304), K+304 (+304)	76.26	25.98
2809	seq=translation; coord=9:48437559..48441823:-1; parent_transcript=GRMZM2G049811_T01; parent_gene=GRMZM2G049811	GRMZM2G049811_P01	TRUE	TRUE	hIEDMHVLITTPFHPAYVTAER	95%	n+304 (+304)	27.20	25.00
2810	seq=translation; coord=9:48437559..48441823:-1; parent_transcript=GRMZM2G049811_T01; parent_gene=GRMZM2G049811	GRMZM2G049811_P01	TRUE	TRUE	lQIDPELEk	95%	n+304 (+304), K+304 (+304)	36.40	25.15
2811	seq=translation; coord=9:48437559..48441823:-1; parent_transcript=GRMZM2G049811_T01; parent_gene=GRMZM2G049811	GRMZM2G049811_P01	TRUE	TRUE	nFLPGYQQVVQGEWNVAGIAHR	95%	n+304 (+304)	61.57	25.39
2812	seq=translation; coord=9:48437559..48441823:-1; parent_transcript=GRMZM2G049811_T01; parent_gene=GRMZM2G049811	GRMZM2G049811_P01	TRUE	TRUE	nPNFVGcVEGALGIR	95%	n+304 (+304), Carbamidomethyl (+57)	67.25	25.51
2813	seq=translation; coord=1:180400813..180406565:-1; parent_transcript=GRMZM2G085474_T01; parent_gene=GRMZM2G085474	GRMZM2G085474_P01,GRMZM2G085474_P04	TRUE	TRUE	dSGAFAWEIVPIEVPVGR	95%	n+304 (+304)	50.87	26.14
2814	seq=translation; coord=1:180400813..180406565:-1; parent_transcript=GRMZM2G085474_T01; parent_gene=GRMZM2G085474	GRMZM2G085474_P01,GRMZM2G085474_P04	TRUE	TRUE	eDQDAFAIQSNQR	95%	n+304 (+304)	55.17	25.00
2815	seq=translation; coord=1:180400813..180406565:-1; parent_transcript=GRMZM2G085474_T01; parent_gene=GRMZM2G085474	GRMZM2G085474_P01,GRMZM2G085474_P04	TRUE	TRUE	tPLGGFLGALSSLPATk	95%	n+304 (+304), K+304 (+304)	44.72	25.00

2816	seq=translation; coord=9:139511132..139514364:1; parent_transcript=GRMZM2G366532_T01; parent_gene=GRMZM2G366532	GRMZM2G366532_P01,GRMZM2G366532_P02	TRUE	TRUE	dAGVIAGLNVTR	93%	n+304 (+304)	31.72	26.51
2817	seq=translation; coord=9:139511132..139514364:1; parent_transcript=GRMZM2G366532_T01; parent_gene=GRMZM2G366532	GRMZM2G366532_P01,GRMZM2G366532_P02	TRUE	TRUE	fSDPSVQADMk	95%	n+304 (+304), K+304 (+304)	40.85	25.00
2818	seq=translation; coord=9:139511132..139514364:1; parent_transcript=GRMZM2G366532_T01; parent_gene=GRMZM2G366532	GRMZM2G366532_P01,GRMZM2G366532_P02	TRUE	TRUE	sINPDEAVAYGAAVQAAILSGEGNQk	95%	n+304 (+304), K+304 (+304)	71.85	25.43
2819	seq=translation; coord=9:139511132..139514364:1; parent_transcript=GRMZM2G366532_T01; parent_gene=GRMZM2G366532	GRMZM2G366532_P01,GRMZM2G366532_P02	TRUE	TRUE	sQIHDVVLVGGSTR	95%	n+304 (+304)	30.62	26.59
2820	seq=translation; coord=9:139511132..139514364:1; parent_transcript=GRMZM2G366532_T01; parent_gene=GRMZM2G366532	GRMZM2G366532_P01,GRMZM2G366532_P02	TRUE	TRUE	wLDGNQLAEAEEFEDk	95%	n+304 (+304), K+304 (+304)	51.16	25.00
2821	seq=translation; coord=4:38181649..38185320:-1; parent_transcript=GRMZM2G103430_T01; parent_gene=GRMZM2G103430	GRMZM2G103430_P01,GRMZM2G103430_P02, GRMZM2G103430_P03,GRMZM2G103430_P04, GRMZM2G103430_P05	TRUE	TRUE	dITEEDLR	95%	n+304 (+304)	31.97	25.00
2822	seq=translation; coord=4:38181649..38185320:-1; parent_transcript=GRMZM2G103430_T01; parent_gene=GRMZM2G103430	GRMZM2G103430_P01,GRMZM2G103430_P02, GRMZM2G103430_P03,GRMZM2G103430_P04, GRMZM2G103430_P05	TRUE	TRUE	gFAFVTFMDk	95%	n+304 (+304), K+304 (+304)	33.57	25.00
2823	seq=translation; coord=4:38181649..38185320:-1; parent_transcript=GRMZM2G103430_T01; parent_gene=GRMZM2G103430	GRMZM2G103430_P01,GRMZM2G103430_P02, GRMZM2G103430_P03,GRMZM2G103430_P04, GRMZM2G103430_P05	TRUE	TRUE	gPDVAGGGPEDEEER	95%	n+304 (+304)	92.52	25.00
2824	seq=translation; coord=4:38181649..38185320:-1; parent_transcript=GRMZM2G103430_T01; parent_gene=GRMZM2G103430	GRMZM2G103430_P01,GRMZM2G103430_P02, GRMZM2G103430_P03,GRMZM2G103430_P04, GRMZM2G103430_P05	TRUE	TRUE	gPGVINIEMFk	95%	n+304 (+304), K+304 (+304)	52.68	26.28
2825	seq=translation; coord=4:38181649..38185320:-1; parent_transcript=GRMZM2G103430_T01; parent_gene=GRMZM2G103430	GRMZM2G103430_P01,GRMZM2G103430_P02, GRMZM2G103430_P03,GRMZM2G103430_P04, GRMZM2G103430_P05	TRUE	TRUE	mVPMVLPDGR	95%	n+304 (+304)	31.15	25.07
2826	seq=translation; coord=4:38181649..38185320:-1; parent_transcript=GRMZM2G103430_T01; parent_gene=GRMZM2G103430	GRMZM2G103430_P01,GRMZM2G103430_P02, GRMZM2G103430_P03,GRMZM2G103430_P04, GRMZM2G103430_P05	TRUE	TRUE	vDGSQLTVSWAEPk	95%	n+304 (+304), K+304 (+304)	45.51	25.98
2827	seq=translation; coord=4:53954699..53962275:1; parent_transcript=GRMZM2G423027_T01; parent_gene=GRMZM2G423027	GRMZM2G423027_P01,GRMZM2G423027_P07	TRUE	TRUE	mVIPVGTVFQELk	95%	n+304 (+304), K+304 (+304)	51.13	25.00
2828	seq=translation; coord=4:53954699..53962275:1; parent_transcript=GRMZM2G423027_T01; parent_gene=GRMZM2G423027	GRMZM2G423027_P01,GRMZM2G423027_P07	TRUE	TRUE	vAEVMEAIDR	95%	n+304 (+304)	36.30	25.50
2829	seq=translation; coord=4:237508371..237511930:-1; parent_transcript=GRMZM2G166035_T01; parent_gene=GRMZM2G166035	GRMZM2G166035_P01,GRMZM2G166035_P02	TRUE	TRUE	aSMEDFYEAR	95%	n+304 (+304)	43.11	25.00
2830	seq=translation; coord=4:237508371..237511930:-1; parent_transcript=GRMZM2G166035_T01; parent_gene=GRMZM2G166035	GRMZM2G166035_P01,GRMZM2G166035_P02	TRUE	TRUE	vGMFGVYDGHGGVR	95%	n+304 (+304)	57.79	25.00
2831	seq=translation; coord=5:199376854..199379060:1; parent_transcript=GRMZM2G027741_T01; parent_gene=GRMZM2G027741	GRMZM2G027741_P01,GRMZM2G027741_P02, GRMZM2G027741_P03,GRMZM2G027741_P04	TRUE	TRUE	aFSEVAAER	95%	n+304 (+304)	31.98	25.00
2832	seq=translation; coord=5:199376854..199379060:1; parent_transcript=GRMZM2G027741_T01; parent_gene=GRMZM2G027741	GRMZM2G027741_P01,GRMZM2G027741_P02, GRMZM2G027741_P03,GRMZM2G027741_P04	TRUE	TRUE	aLMLIEAWGESGDELR	93%	n+304 (+304)	29.68	25.00
2833	seq=translation; coord=5:199376854..199379060:1; parent_transcript=GRMZM2G027741_T01; parent_gene=GRMZM2G027741	GRMZM2G027741_P01,GRMZM2G027741_P02, GRMZM2G027741_P03,GRMZM2G027741_P04	TRUE	TRUE	vDSVELIR	91%	n+304 (+304)	28.95	25.69

2834	seq=translation; coord=5:199376854..199379060:1; parent_transcript=GRMZM2G027741_T01; parent_gene=GRMZM2G027741	GRMZM2G027741_P01,GRMZM2G027741_P02, GRMZM2G027741_P03,GRMZM2G027741_P04	TRUE	TRUE	vQYLSLVLLETIVk	93%	n+304 (+304), K+304 (+304)	27.20	25.00
2835	seq=translation; coord=3:161953049..161955801:1; parent_transcript=GRMZM2G153991_T01; parent_gene=GRMZM2G153991	GRMZM2G153991_P01	TRUE	TRUE	gTLTDGSVFDSSYDR	95%	n+304 (+304)	73.86	25.00
2836	seq=translation; coord=3:161953049..161955801:1; parent_transcript=GRMZM2G153991_T01; parent_gene=GRMZM2G153991	GRMZM2G153991_P01	TRUE	TRUE	sGDVTELQIGVk	95%	n+304 (+304), K+304 (+304)	34.39	26.09
2837	seq=translation; coord=6:161454531..161462066:-1; parent_transcript=GRMZM2G090087_T02; parent_gene=GRMZM2G090087	GRMZM2G090087_P02,GRMZM2G090087_P03	TRUE	TRUE	aAHMLGQPAVLDFFAk	95%	n+304 (+304), K+304 (+304)	29.82	25.87
2838	seq=translation; coord=6:161454531..161462066:-1; parent_transcript=GRMZM2G090087_T02; parent_gene=GRMZM2G090087	GRMZM2G090087_P02,GRMZM2G090087_P03	TRUE	TRUE	fLENFcGDQVR	93%	n+304 (+304), Carbamidomethyl (+57)	30.13	25.00
2839	seq=translation; coord=6:161454531..161462066:-1; parent_transcript=GRMZM2G090087_T02; parent_gene=GRMZM2G090087	GRMZM2G090087_P02,GRMZM2G090087_P03	TRUE	TRUE	gAGIGTPEAIk	95%	n+304 (+304), K+304 (+304)	56.30	25.17
2840	seq=translation; coord=6:161454531..161462066:-1; parent_transcript=GRMZM2G090087_T02; parent_gene=GRMZM2G090087	GRMZM2G090087_P02,GRMZM2G090087_P03	TRUE	TRUE	lEDAGFDWk	95%	n+304 (+304), K+304 (+304)	29.54	25.00
2841	seq=translation; coord=6:161454531..161462066:-1; parent_transcript=GRMZM2G090087_T02; parent_gene=GRMZM2G090087	GRMZM2G090087_P02,GRMZM2G090087_P03	TRUE	TRUE	lLHDcGLPAEDMDFINSDGAVMNk	95%	n+304 (+304), Carbamidomethyl (+57), K+304 (+304)	38.23	25.00
2842	seq=translation; coord=6:161454531..161462066:-1; parent_transcript=GRMZM2G090087_T02; parent_gene=GRMZM2G090087	GRMZM2G090087_P02,GRMZM2G090087_P03	TRUE	TRUE	lLLEANPk	91%	n+304 (+304), K+304 (+304)	27.75	25.00
2843	seq=translation; coord=6:161454531..161462066:-1; parent_transcript=GRMZM2G090087_T02; parent_gene=GRMZM2G090087	GRMZM2G090087_P02,GRMZM2G090087_P03	TRUE	TRUE	sGNFELVTk	95%	n+304 (+304), K+304 (+304)	33.78	26.58
2844	seq=translation; coord=6:161454531..161462066:-1; parent_transcript=GRMZM2G090087_T02; parent_gene=GRMZM2G090087	GRMZM2G090087_P02,GRMZM2G090087_P03	TRUE	TRUE	sYQQALAEVQVSQk	95%	n+304 (+304), K+304 (+304)	36.02	25.58
2845	seq=translation; coord=6:161454531..161462066:-1; parent_transcript=GRMZM2G090087_T02; parent_gene=GRMZM2G090087	GRMZM2G090087_P02,GRMZM2G090087_P03	TRUE	TRUE	yLMYGDISAk	95%	n+304 (+304), K+304 (+304)	34.46	25.51
2846	seq=translation; coord=7:23715448..23717527:-1; parent_transcript=GRMZM2G097030_T01; parent_gene=GRMZM2G097030	GRMZM2G097030_P01	TRUE	TRUE	aDGAATLLSMLATPR	95%	n+304 (+304)	29.85	25.15
2847	seq=translation; coord=7:23715448..23717527:-1; parent_transcript=GRMZM2G097030_T01; parent_gene=GRMZM2G097030	GRMZM2G097030_P01	TRUE	TRUE	eVAcVVVDGQWYk	95%	n+304 (+304), Carbamidomethyl (+57), K+304 (+304)	44.18	25.30
2848	seq=translation; coord=7:23715448..23717527:-1; parent_transcript=GRMZM2G097030_T01; parent_gene=GRMZM2G097030	GRMZM2G097030_P01	TRUE	TRUE	lDEVVPGLEPLR	95%	n+304 (+304)	41.40	26.56
2849	seq=translation; coord=7:23715448..23717527:-1; parent_transcript=GRMZM2G097030_T01; parent_gene=GRMZM2G097030	GRMZM2G097030_P01	TRUE	TRUE	lMAGELGPQGPR	90%	n+304 (+304)	27.48	25.85
2850	seq=translation; coord=7:23715448..23717527:-1; parent_transcript=GRMZM2G097030_T01; parent_gene=GRMZM2G097030	GRMZM2G097030_P01	TRUE	TRUE	vAVPALVLR	95%	n+304 (+304)	31.33	25.00
2851	seq=translation; coord=7:23715448..23717527:-1; parent_transcript=GRMZM2G097030_T01; parent_gene=GRMZM2G097030	GRMZM2G097030_P01	TRUE	TRUE	vDGSDDETVLR	95%	n+304 (+304)	46.03	25.00

2852	seq=translation; coord=7:23715448..23717527:-1; parent_transcript=GRMZM2G097030_T01; parent_gene=GRMZM2G097030	GRMZM2G097030_P01	TRUE	TRUE	vVMFPFPFWSHINQMLQLGk	95%	n+304 (+304), K+304 (+304)	31.23	25.00
2853	seq=translation; coord=6:31868128..31869802:-1; parent_transcript=GRMZM2G163406_T01; parent_gene=GRMZM2G163406	GRMZM2G163406_P01	TRUE	TRUE	eVSGTYGAFEGATTLTSLR	95%	n+304 (+304)	68.81	25.17
2854	seq=translation; coord=6:31868128..31869802:-1; parent_transcript=GRMZM2G163406_T01; parent_gene=GRMZM2G163406	GRMZM2G163406_P01	TRUE	TRUE	gPDASLVAR	95%	n+304 (+304)	33.09	25.51
2855	seq=translation; coord=6:31868128..31869802:-1; parent_transcript=GRMZM2G163406_T01; parent_gene=GRMZM2G163406	GRMZM2G163406_P01	TRUE	TRUE	iGLWGGPGGSAQDITAERPPQR	95%	n+304 (+304)	34.31	25.00
2856	seq=translation; coord=6:31868128..31869802:-1; parent_transcript=GRMZM2G163406_T01; parent_gene=GRMZM2G163406	GRMZM2G163406_P01	TRUE	TRUE	lVDAIGVYLR	95%	n+304 (+304)	39.07	25.00
2857	seq=translation; coord=6:31868128..31869802:-1; parent_transcript=GRMZM2G163406_T01; parent_gene=GRMZM2G163406	GRMZM2G163406_P01	TRUE	TRUE	vQGITSEIGNAHQLFVVVFDTDR	95%	n+304 (+304)	77.45	25.30
2858	seq=translation; coord=6:31868128..31869802:-1; parent_transcript=GRMZM2G163406_T01; parent_gene=GRMZM2G163406	GRMZM2G163406_P01	TRUE	TRUE	yVSDFPVYDGR	95%	n+304 (+304)	46.13	25.00
2859	seq=translation; coord=8:95715355..95726032:1; parent_transcript=GRMZM2G375504_T01; parent_gene=GRMZM2G375504	GRMZM2G375504_P01,GRMZM2G375504_P02, GRMZM2G375504_P03	TRUE	TRUE	aDLLVLLSDVDGLYSGPPSEPGSk	95%	n+304 (+304), K+304 (+304)	47.78	25.19
2860	seq=translation; coord=8:95715355..95726032:1; parent_transcript=GRMZM2G375504_T01; parent_gene=GRMZM2G375504	GRMZM2G375504_P01,GRMZM2G375504_P02, GRMZM2G375504_P03	TRUE	TRUE	aPGLDDILLSLk	94%	n+304 (+304), K+304 (+304)	28.58	25.00
2861	seq=translation; coord=8:95715355..95726032:1; parent_transcript=GRMZM2G375504_T01; parent_gene=GRMZM2G375504	GRMZM2G375504_P01,GRMZM2G375504_P02, GRMZM2G375504_P03	TRUE	TRUE	iLLDIADALEQNEDLIR	95%	n+304 (+304)	38.82	25.62
2862	seq=translation; coord=8:95715355..95726032:1; parent_transcript=GRMZM2G375504_T01; parent_gene=GRMZM2G375504	GRMZM2G375504_P01,GRMZM2G375504_P02, GRMZM2G375504_P03	TRUE	TRUE	lcETVESLLDLk	95%	n+304 (+304), Carbamidomethyl (+57), K+304 (+304)	32.10	25.50
2863	seq=translation; coord=8:95715355..95726032:1; parent_transcript=GRMZM2G375504_T01; parent_gene=GRMZM2G375504	GRMZM2G375504_P01,GRMZM2G375504_P02, GRMZM2G375504_P03	TRUE	TRUE	lDDVIDLVIPR	95%	n+304 (+304)	50.16	26.34
2864	seq=translation; coord=7:147132517..147134161:-1; parent_transcript=GRMZM2G168330_T02; parent_gene=GRMZM2G168330	GRMZM2G168330_P02	TRUE	TRUE	aALSDFDR	95%	n+304 (+304)	37.87	25.00
2865	seq=translation; coord=7:147132517..147134161:-1; parent_transcript=GRMZM2G168330_T02; parent_gene=GRMZM2G168330	GRMZM2G168330_P02	TRUE	TRUE	aLVDAPDMVR	93%	n+304 (+304)	29.76	25.00
2866	seq=translation; coord=7:147132517..147134161:-1; parent_transcript=GRMZM2G168330_T02; parent_gene=GRMZM2G168330	GRMZM2G168330_P02	TRUE	TRUE	aMEEADVk	95%	n+304 (+304), K+304 (+304)	37.29	25.42
2867	seq=translation; coord=7:147132517..147134161:-1; parent_transcript=GRMZM2G168330_T02; parent_gene=GRMZM2G168330	GRMZM2G168330_P02	TRUE	TRUE	lVVIVDVVDQNR	95%	n+304 (+304)	78.08	25.69
2868	seq=translation; coord=7:147132517..147134161:-1; parent_transcript=GRMZM2G168330_T02; parent_gene=GRMZM2G168330	GRMZM2G168330_P02	TRUE	TRUE	wENSSWGk	89%	n+304 (+304), K+304 (+304)	25.81	25.00
2869	seq=translation; coord=1:60163114..60165406:1; parent_transcript=GRMZM2G000923_T01; parent_gene=GRMZM2G000923	GRMZM2G000923_P01,GRMZM2G000923_P02, GRMZM2G465333_P01	TRUE	TRUE	dIELVMTQASVSRPk	93%	n+304 (+304), K+304 (+304)	27.85	25.24

2870	seq=translation; coord=1:60163114..60165406:1; parent_transcript=GRMZM2G000923_T01; parent_gene=GRMZM2G000923	GRMZM2G000923_P01,GRMZM2G000923_P02, GRMZM2G465333_P01	TRUE	TRUE	iEDLSSQLQTQAAEQFk	95%	n+304 (+304), K+304 (+304)	44.20	25.80
2871	seq=translation; coord=1:60163114..60165406:1; parent_transcript=GRMZM2G000923_T01; parent_gene=GRMZM2G000923	GRMZM2G000923_P01,GRMZM2G000923_P02, GRMZM2G465333_P01	TRUE	TRUE	sPNSDTYVIFGEAk	95%	n+304 (+304), K+304 (+304)	38.31	25.74
2872	seq=translation; coord=1:60163114..60165406:1; parent_transcript=GRMZM2G000923_T01; parent_gene=GRMZM2G000923	GRMZM2G000923_P01,GRMZM2G000923_P02, GRMZM2G465333_P01	TRUE	TRUE	tAQTAEELATQIEQQk	95%	n+304 (+304), K+304 (+304)	65.30	26.31
2873	seq=translation; coord=9:107209042..107212141:1; parent_transcript=GRMZM2G172357_T01; parent_gene=GRMZM2G172357	GRMZM2G172357_P01,GRMZM2G172357_P02, GRMZM2G172357_P05,GRMZM2G172357_P06	TRUE	TRUE	eDGSEIQDALLEIVGR	95%	n+304 (+304)	52.86	25.01
2874	seq=translation; coord=9:107209042..107212141:1; parent_transcript=GRMZM2G172357_T01; parent_gene=GRMZM2G172357	GRMZM2G172357_P01,GRMZM2G172357_P02, GRMZM2G172357_P05,GRMZM2G172357_P06	TRUE	TRUE	lLNIGVk	93%	n+304 (+304), K+304 (+304)	27.22	25.00
2875	seq=translation; coord=9:107209042..107212141:1; parent_transcript=GRMZM2G172357_T01; parent_gene=GRMZM2G172357	GRMZM2G172357_P01,GRMZM2G172357_P02, GRMZM2G172357_P05,GRMZM2G172357_P06	TRUE	TRUE	tVPQVFVHGk	95%	n+304 (+304), K+304 (+304)	30.83	25.00
2876	seq=translation; coord=1:293135180..293138761:1; parent_transcript=GRMZM2G061900_T01; parent_gene=GRMZM2G061900	GRMZM2G061900_P01,GRMZM2G061900_P02, GRMZM2G156476_P01,GRMZM2G156476_P02	TRUE	TRUE	aDYDYLIk	90%	n+304 (+304), K+304 (+304)	25.53	25.00
2877	seq=translation; coord=1:293135180..293138761:1; parent_transcript=GRMZM2G061900_T01; parent_gene=GRMZM2G061900	GRMZM2G061900_P01,GRMZM2G061900_P02, GRMZM2G156476_P01,GRMZM2G156476_P02	TRUE	TRUE	gQALADEYGIk	95%	n+304 (+304), K+304 (+304)	44.31	25.49
2878	seq=translation; coord=1:293135180..293138761:1; parent_transcript=GRMZM2G061900_T01; parent_gene=GRMZM2G061900	GRMZM2G061900_P01,GRMZM2G061900_P02, GRMZM2G156476_P01,GRMZM2G156476_P02	TRUE	TRUE	tNLNVEQVFFSIAR	95%	n+304 (+304)	42.70	25.25
2879	seq=translation; coord=1:234307913..234320907:1; parent_transcript=GRMZM2G008607_T01; parent_gene=GRMZM2G008607	GRMZM2G008607_P01	TRUE	TRUE	aLWFSPPTNDGYSGTGTyGPLPR	95%	n+304 (+304), iTRAQ8plex (+304)	28.78	25.00
2880	seq=translation; coord=1:234307913..234320907:1; parent_transcript=GRMZM2G008607_T01; parent_gene=GRMZM2G008607	GRMZM2G008607_P01	TRUE	TRUE	aPTDPLPk	95%	n+304 (+304), K+304 (+304)	32.76	25.76
2881	seq=translation; coord=1:234307913..234320907:1; parent_transcript=GRMZM2G008607_T01; parent_gene=GRMZM2G008607	GRMZM2G008607_P01	TRUE	TRUE	aVISNMEk	95%	n+304 (+304), K+304 (+304)	34.37	26.58
2882	seq=translation; coord=1:234307913..234320907:1; parent_transcript=GRMZM2G008607_T01; parent_gene=GRMZM2G008607	GRMZM2G008607_P01	TRUE	TRUE	gADLFFR	95%	n+304 (+304)	33.18	25.00
2883	seq=translation; coord=1:234307913..234320907:1; parent_transcript=GRMZM2G008607_T01; parent_gene=GRMZM2G008607	GRMZM2G008607_P01	TRUE	TRUE	iFISELLVDELTTQSQEIIHk	95%	n+304 (+304), K+304 (+304)	38.19	25.00
2884	seq=translation; coord=1:234307913..234320907:1; parent_transcript=GRMZM2G008607_T01; parent_gene=GRMZM2G008607	GRMZM2G008607_P01	TRUE	TRUE	lLLPQFk	93%	n+304 (+304), K+304 (+304)	28.36	25.00
2885	seq=translation; coord=1:234307913..234320907:1; parent_transcript=GRMZM2G008607_T01; parent_gene=GRMZM2G008607	GRMZM2G008607_P01	TRUE	TRUE	lNSEGGILk	95%	n+304 (+304), K+304 (+304)	41.50	25.49
2886	seq=translation; coord=1:234307913..234320907:1; parent_transcript=GRMZM2G008607_T01; parent_gene=GRMZM2G008607	GRMZM2G008607_P01	TRUE	TRUE	sYIEFAER	92%	n+304 (+304)	27.48	25.00
2887	seq=translation; coord=6:79193305..79195490:1; parent_transcript=GRMZM2G127948_T01; parent_gene=GRMZM2G127948	GRMZM2G127948_P01	TRUE	TRUE	aLAADDRVEIcQLPVGDGVTLcR	95%	n+304 (+304), Carbamidomethyl (+57), Carbamidomethyl (+57)	31.64	25.44

2888	seq=translation; coord=6:79193305..79195490:1; parent_transcript=GRMZM2G127948_T01; parent_gene=GRMZM2G127948	GRMZM2G127948_P01	TRUE	TRUE	eGPALPVLDDLIAEEk	95%	n+304 (+304), K+304 (+304)	39.83	25.71
2889	seq=translation; coord=6:79193305..79195490:1; parent_transcript=GRMZM2G127948_T01; parent_gene=GRMZM2G127948	GRMZM2G127948_P01	TRUE	TRUE	hPWNLMTTSADEGQFLNMLIk	95%	n+304 (+304), K+304 (+304)	29.19	25.00
2890	seq=translation; coord=6:79193305..79195490:1; parent_transcript=GRMZM2G127948_T01; parent_gene=GRMZM2G127948	GRMZM2G127948_P01	TRUE	TRUE	sDDLYQYILDTSVYPR	95%	n+304 (+304)	53.92	25.00
2891	seq=translation; coord=6:79193305..79195490:1; parent_transcript=GRMZM2G127948_T01; parent_gene=GRMZM2G127948	GRMZM2G127948_P01	TRUE	TRUE	sDDLYQYILDTSVyPR	95%	n+304 (+304), iTRAQ8plex (+304)	30.36	25.21
2892	seq=translation; coord=6:79193305..79195490:1; parent_transcript=GRMZM2G127948_T01; parent_gene=GRMZM2G127948	GRMZM2G127948_P01	TRUE	TRUE	vEIcQLPVGDGVTLcR	95%	n+304 (+304), Carbamidomethyl (+57), Carbamidomethyl (+57)	66.78	25.77
2893	seq=translation; coord=3:231823510..231824603:1; parent_transcript=GRMZM2G107839_T01; parent_gene=GRMZM2G107839	GRMZM2G107839_P01,GRMZM2G107839_P02	TRUE	TRUE	aSALPASccSGVk	95%	n+304 (+304), Carbamidomethyl (+57), Carbamidomethyl (+57), K+304 (+304)	41.37	25.25
2894	seq=translation; coord=3:231823510..231824603:1; parent_transcript=GRMZM2G107839_T01; parent_gene=GRMZM2G107839	GRMZM2G107839_P01,GRMZM2G107839_P02	TRUE	TRUE	cGVSVGFPISMSTDcNk	95%	Carbamidomethyl (+57), n+304 (+304), Carbamidomethyl (+57), K+304 (+304)	59.85	25.00
2895	seq=translation; coord=3:231823510..231824603:1; parent_transcript=GRMZM2G107839_T01; parent_gene=GRMZM2G107839	GRMZM2G107839_P01,GRMZM2G107839_P02	TRUE	TRUE	cGVSVGFPISmSTDcNk	95%	Carbamidomethyl (+57), n+304 (+304), Oxidation (+16), Carbamidomethyl (+57), K+304 (+304)	41.13	25.00
2896	seq=translation; coord=3:231823510..231824603:1; parent_transcript=GRMZM2G107839_T01; parent_gene=GRMZM2G107839	GRMZM2G107839_P01,GRMZM2G107839_P02	TRUE	TRUE	sLANSVk	87%	n+304 (+304), K+304 (+304)	26.41	25.85
2897	seq=translation; coord=3:231823510..231824603:1; parent_transcript=GRMZM2G107839_T01; parent_gene=GRMZM2G107839	GRMZM2G107839_P01,GRMZM2G107839_P02	TRUE	TRUE	sVNMGTVATIPGk	92%	n+304 (+304), K+304 (+304)	27.73	26.15
2898	seq=translation; coord=3:231823510..231824603:1; parent_transcript=GRMZM2G107839_T01; parent_gene=GRMZM2G107839	GRMZM2G107839_P01,GRMZM2G107839_P02	TRUE	TRUE	sVNmGTVATIPGk	95%	n+304 (+304), Oxidation (+16), K+304 (+304)	43.79	26.00
2899	seq=translation; coord=4:216707047..216710020:-1; parent_transcript=GRMZM2G091715_T01; parent_gene=GRMZM2G091715	GRMZM2G091715_P01,GRMZM2G091715_P03, GRMZM2G175818_P02	TRUE	TRUE	qLALPDHTEVcGESk	95%	n+304 (+304), Carbamidomethyl (+57), K+304 (+304)	49.50	25.51
2900	seq=translation; coord=4:216707047..216710020:-1; parent_transcript=GRMZM2G091715_T01; parent_gene=GRMZM2G091715	GRMZM2G091715_P01,GRMZM2G091715_P03, GRMZM2G175818_P02	TRUE	TRUE	qLALPDHTEVcGESk	95%	Pyro-cmC (-17), n+304 (+304), Carbamidomethyl (+57), K+304 (+304)	38.17	25.00
2901	seq=translation; coord=4:239151749..239155703:-1; parent_transcript=GRMZM2G024310_T01; parent_gene=GRMZM2G024310	GRMZM2G024310_P01	TRUE	TRUE	nMATLMAEFDk	95%	n+304 (+304), K+304 (+304)	48.29	25.00
2902	seq=translation; coord=9:145346839..145349218:-1; parent_transcript=GRMZM2G067225_T01; parent_gene=GRMZM2G067225	GRMZM2G067225_P01	TRUE	TRUE	dRLDFYYFQGQDk	95%	n+304 (+304), K+304 (+304)	27.25	25.00
2903	seq=translation; coord=9:145346839..145349218:-1; parent_transcript=GRMZM2G067225_T01; parent_gene=GRMZM2G067225	GRMZM2G067225_P01	TRUE	TRUE	dVPGSYGLPLVGAVR	95%	n+304 (+304)	36.90	25.99
2904	seq=translation; coord=9:145346839..145349218:-1; parent_transcript=GRMZM2G067225_T01; parent_gene=GRMZM2G067225	GRMZM2G067225_P01	TRUE	TRUE	kDLLVESHDAVFQVR	95%	K+304 (+304), n+304 (+304)	37.18	25.30

2905	seq=translation; coord=9:145346839..145349218:-1; parent_transcript=GRMZM2G067225_T01; parent_gene=GRMZM2G067225	GRMZM2G067225_P01	TRUE	TRUE	kGEMLFGYQPcATk	95%	K+304 (+304), n+304 (+304), Carbamidomethyl (+57), K+304 (+304)	36.27	26.62
2906	seq=translation; coord=9:145346839..145349218:-1; parent_transcript=GRMZM2G067225_T01; parent_gene=GRMZM2G067225	GRMZM2G067225_P01	TRUE	TRUE	mNVPPGPFMAR	89%	n+304 (+304)	26.63	25.00
2907	seq=translation; coord=9:145346839..145349218:-1; parent_transcript=GRMZM2G067225_T01; parent_gene=GRMZM2G067225	GRMZM2G067225_P01	TRUE	TRUE	sFPVLFDMDk	95%	n+304 (+304), K+304 (+304)	38.77	25.55
2908	seq=translation; coord=9:145346839..145349218:-1; parent_transcript=GRMZM2G067225_T01; parent_gene=GRMZM2G067225	GRMZM2G067225_P01	TRUE	TRUE	sNFSSLLATVEAELAk	95%	n+304 (+304), K+304 (+304)	36.10	25.80
2909	seq=translation; coord=9:145346839..145349218:-1; parent_transcript=GRMZM2G067225_T01; parent_gene=GRMZM2G067225	GRMZM2G067225_P01	TRUE	TRUE	sVVWESLR	94%	n+304 (+304)	32.11	25.72
2910	seq=translation; coord=9:145346839..145349218:-1; parent_transcript=GRMZM2G067225_T01; parent_gene=GRMZM2G067225	GRMZM2G067225_P01	TRUE	TRUE	vFGDTAGDFVPDR	95%	n+304 (+304)	51.01	25.00
2911	seq=translation; coord=9:145346839..145349218:-1; parent_transcript=GRMZM2G067225_T01; parent_gene=GRMZM2G067225	GRMZM2G067225_P01	TRUE	TRUE	vVAVLDAk	88%	n+304 (+304), K+304 (+304)	28.58	27.10
2912	seq=translation; coord=5:163984090..163986394:1; parent_transcript=GRMZM2G143008_T01; parent_gene=GRMZM2G143008	GRMZM2G143008_P01	TRUE	TRUE	aDLLLALGVR	95%	n+304 (+304)	31.91	25.00
2913	seq=translation; coord=5:163984090..163986394:1; parent_transcript=GRMZM2G143008_T01; parent_gene=GRMZM2G143008	GRMZM2G143008_P01	TRUE	TRUE	dIQQQMAVPVWDkPMSLPGYIAR	95%	n+304 (+304), K+304 (+304)	30.74	25.26
2914	seq=translation; coord=5:163984090..163986394:1; parent_transcript=GRMZM2G143008_T01; parent_gene=GRMZM2G143008	GRMZM2G143008_P01	TRUE	TRUE	dVFAYPGGASMEIHQALTR	95%	n+304 (+304)	72.80	25.00
2915	seq=translation; coord=5:163984090..163986394:1; parent_transcript=GRMZM2G143008_T01; parent_gene=GRMZM2G143008	GRMZM2G143008_P01	TRUE	TRUE	hNYLVLDVDDIPR	95%	n+304 (+304)	58.19	25.43
2916	seq=translation; coord=5:163984090..163986394:1; parent_transcript=GRMZM2G143008_T01; parent_gene=GRMZM2G143008	GRMZM2G143008_P01	TRUE	TRUE	iVHVDIDPAEIGk	94%	n+304 (+304), K+304 (+304)	25.53	25.00
2917	seq=translation; coord=5:163984090..163986394:1; parent_transcript=GRMZM2G143008_T01; parent_gene=GRMZM2G143008	GRMZM2G143008_P01	TRUE	TRUE	kGADILVESLER	95%	K+304 (+304), n+304 (+304)	29.82	25.97
2918	seq=translation; coord=5:163984090..163986394:1; parent_transcript=GRMZM2G143008_T01; parent_gene=GRMZM2G143008	GRMZM2G143008_P01	TRUE	TRUE	lALQGMNALLEGSTSk	95%	n+304 (+304), K+304 (+304)	36.98	25.72
2919	seq=translation; coord=5:163984090..163986394:1; parent_transcript=GRMZM2G143008_T01; parent_gene=GRMZM2G143008	GRMZM2G143008_P01	TRUE	TRUE	lPkPPATELLEQVLR	95%	n+304 (+304), K+304 (+304)	39.71	25.00
2920	seq=translation; coord=5:163984090..163986394:1; parent_transcript=GRMZM2G143008_T01; parent_gene=GRMZM2G143008	GRMZM2G143008_P01	TRUE	TRUE	mIGTDAFQETPIVEVTR	95%	n+304 (+304)	65.82	25.01
2921	seq=translation; coord=5:163984090..163986394:1; parent_transcript=GRMZM2G143008_T01; parent_gene=GRMZM2G143008	GRMZM2G143008_P01	TRUE	TRUE	mLETPGPYLLDIIVPHQEHVLPMIPSGGAFk	95%	n+304 (+304), K+304 (+304)	26.17	25.00
2922	seq=translation; coord=5:163984090..163986394:1; parent_transcript=GRMZM2G143008_T01; parent_gene=GRMZM2G143008	GRMZM2G143008_P01	TRUE	TRUE	sPVIANHLFR	95%	n+304 (+304)	57.29	25.73

2923	seq=translation; coord=5:163984090..163986394:1; parent_transcript=GRMZM2G143008_T01; parent_gene=GRMZM2G143008	GRMZM2G143008_P01	TRUE	TRUE	vFVLNNQHLGMVVQWEDR	95%	n+304 (+304)	67.71	25.45
2924	seq=translation; coord=2:232101270..232109417:-1; parent_transcript=GRMZM2G036034_T01; parent_gene=GRMZM2G036034	GRMZM2G036034_P01,GRMZM2G178618_P01	TRUE	TRUE	aIVDSIIILIR	95%	n+304 (+304)	36.77	25.00
2925	seq=translation; coord=2:232101270..232109417:-1; parent_transcript=GRMZM2G036034_T01; parent_gene=GRMZM2G036034	GRMZM2G036034_P01,GRMZM2G178618_P01	TRUE	TRUE	eLSPSADEVIIVTSSLMk	95%	n+304 (+304), K+304 (+304)	37.63	25.00
2926	seq=translation; coord=2:232101270..232109417:-1; parent_transcript=GRMZM2G036034_T01; parent_gene=GRMZM2G036034	GRMZM2G036034_P01,GRMZM2G178618_P01	TRUE	TRUE	eLTPAITVLQLFLSSSkPVLR	95%	n+304 (+304), K+304 (+304)	39.88	25.00
2927	seq=translation; coord=2:232101270..232109417:-1; parent_transcript=GRMZM2G036034_T01; parent_gene=GRMZM2G036034	GRMZM2G036034_P01,GRMZM2G178618_P01	TRUE	TRUE	iIDSTLLTQIER	95%	n+304 (+304)	59.16	25.00
2928	seq=translation; coord=2:232101270..232109417:-1; parent_transcript=GRMZM2G036034_T01; parent_gene=GRMZM2G036034	GRMZM2G036034_P01,GRMZM2G178618_P01	TRUE	TRUE	lLSSIPEFADFGk	95%	n+304 (+304), K+304 (+304)	39.81	25.81
2929	seq=translation; coord=2:232101270..232109417:-1; parent_transcript=GRMZM2G036034_T01; parent_gene=GRMZM2G036034	GRMZM2G036034_P01,GRMZM2G178618_P01	TRUE	TRUE	lLYLLNQGDTFTk	95%	n+304 (+304), K+304 (+304)	43.14	25.00
2930	seq=translation; coord=2:232101270..232109417:-1; parent_transcript=GRMZM2G036034_T01; parent_gene=GRMZM2G036034	GRMZM2G036034_P01,GRMZM2G178618_P01	TRUE	TRUE	nAWENMDPETER	95%	n+304 (+304)	32.19	25.00
2931	seq=translation; coord=2:232101270..232109417:-1; parent_transcript=GRMZM2G036034_T01; parent_gene=GRMZM2G036034	GRMZM2G036034_P01,GRMZM2G178618_P01	TRUE	TRUE	sSAPVELTEAETEYSVNVVk	95%	n+304 (+304), K+304 (+304)	32.25	25.29
2932	seq=translation; coord=1:26684241..26696594:-1; parent_transcript=GRMZM2G099628_T02; parent_gene=GRMZM2G099628	GRMZM2G099628_P02	TRUE	TRUE	dTDHLFLELPLLR	95%	n+304 (+304)	50.67	26.26
2933	seq=translation; coord=1:26684241..26696594:-1; parent_transcript=GRMZM2G099628_T02; parent_gene=GRMZM2G099628	GRMZM2G099628_P02	TRUE	TRUE	gIGVFGNDAk	95%	n+304 (+304), K+304 (+304)	30.79	25.99
2934	seq=translation; coord=1:26684241..26696594:-1; parent_transcript=GRMZM2G099628_T02; parent_gene=GRMZM2G099628	GRMZM2G099628_P02	TRUE	TRUE	hPDADSLYVEEIDVGEDTPR	95%	n+304 (+304)	42.37	25.00
2935	seq=translation; coord=1:26684241..26696594:-1; parent_transcript=GRMZM2G099628_T02; parent_gene=GRMZM2G099628	GRMZM2G099628_P02	TRUE	TRUE	sPWDFVPAGHR	95%	n+304 (+304)	37.77	25.00
2936	seq=translation; coord=1:26684241..26696594:-1; parent_transcript=GRMZM2G099628_T02; parent_gene=GRMZM2G099628	GRMZM2G099628_P02	TRUE	TRUE	vELVEPPESAAVGER	95%	n+304 (+304)	63.27	25.67
2937	seq=translation; coord=1:26684241..26696594:-1; parent_transcript=GRMZM2G099628_T02; parent_gene=GRMZM2G099628	GRMZM2G099628_P02	TRUE	TRUE	vTFAGYSGEPEASLSGk	95%	n+304 (+304), K+304 (+304)	49.11	25.75
2938	seq=translation; coord=1:26684241..26696594:-1; parent_transcript=GRMZM2G099628_T02; parent_gene=GRMZM2G099628	GRMZM2G099628_P02	TRUE	TRUE	wGVPVPHEk	95%	n+304 (+304), K+304 (+304)	28.72	25.71
2939	seq=translation; coord=3:12189233..12194178:1; parent_transcript=GRMZM2G054559_T01; parent_gene=GRMZM2G054559	GRMZM2G054559_P01,GRMZM2G054559_P08	TRUE	TRUE	eTWNVQLFR	90%	n+304 (+304)	27.26	25.00
2940	seq=translation; coord=3:12189233..12194178:1; parent_transcript=GRMZM2G054559_T01; parent_gene=GRMZM2G054559	GRMZM2G054559_P01,GRMZM2G054559_P08	TRUE	TRUE	iVSFIGGIDLcDGR	95%	n+304 (+304), Carbamidomethyl (+57)	31.98	25.00

2941	seq=translation; coord=3:12189233..12194178:1; parent_transcript=GRMZM2G054559_T01; parent_gene=GRMZM2G054559	GRMZM2G054559_P01,GRMZM2G054559_P08	TRUE	TRUE	qEGEYEPEEHPEPDTDYIR	95%	n+304 (+304)	39.28	25.00
2942	seq=translation; coord=3:12189233..12194178:1; parent_transcript=GRMZM2G054559_T01; parent_gene=GRMZM2G054559	GRMZM2G054559_P01,GRMZM2G054559_P08	TRUE	TRUE	sIDGGAAFGFPETPEEAAR	95%	n+304 (+304)	56.64	25.00
2943	seq=translation; coord=2:31644513..31646214:-1; parent_transcript=GRMZM2G083253_T02; parent_gene=GRMZM2G083253	GRMZM2G083253_P02,GRMZM2G083253_P04, GRMZM2G083253_P05,GRMZM2G083253_P06, GRMZM2G166659_P01,GRMZM2G166659_P04, GRMZM2G166659_P05	TRUE	TRUE	iEDNNTLVFIVDLk	95%	n+304 (+304), K+304 (+304)	32.50	25.09
2944	seq=translation; coord=2:31644513..31646214:-1; parent_transcript=GRMZM2G083253_T02; parent_gene=GRMZM2G083253	GRMZM2G083253_P02,GRMZM2G083253_P04, GRMZM2G083253_P05,GRMZM2G083253_P06, GRMZM2G166659_P01,GRMZM2G166659_P04, GRMZM2G166659_P05	TRUE	TRUE	lDQYQILk	95%	n+304 (+304), K+304 (+304)	32.72	25.00
2945	seq=translation; coord=2:31644513..31646214:-1; parent_transcript=GRMZM2G083253_T02; parent_gene=GRMZM2G083253	GRMZM2G083253_P02,GRMZM2G083253_P04, GRMZM2G083253_P05,GRMZM2G083253_P06, GRMZM2G166659_P01,GRMZM2G166659_P04, GRMZM2G166659_P05	TRUE	TRUE	lTPDYDALDVANk	95%	n+304 (+304), K+304 (+304)	47.28	25.77
2946	seq=translation; coord=7:150542721..150550925:1; parent_transcript=GRMZM2G138220_T01; parent_gene=GRMZM2G138220	GRMZM2G138220_P01,GRMZM2G171628_P01	TRUE	TRUE	aEAQIGVDVHSALNAALTG	95%	n+304 (+304)	44.76	25.00
2947	seq=translation; coord=7:150542721..150550925:1; parent_transcript=GRMZM2G138220_T01; parent_gene=GRMZM2G138220	GRMZM2G138220_P01,GRMZM2G171628_P01	TRUE	TRUE	gLAEFTAk	93%	n+304 (+304), K+304 (+304)	29.39	26.80
2948	seq=translation; coord=7:150542721..150550925:1; parent_transcript=GRMZM2G138220_T01; parent_gene=GRMZM2G138220	GRMZM2G138220_P01,GRMZM2G171628_P01	TRUE	TRUE	lGSASTDLEk	95%	n+304 (+304), K+304 (+304)	41.85	26.23
2949	seq=translation; coord=7:150542721..150550925:1; parent_transcript=GRMZM2G138220_T01; parent_gene=GRMZM2G138220	GRMZM2G138220_P01,GRMZM2G171628_P01	TRUE	TRUE	lTVNFVLPYk	95%	n+304 (+304), K+304 (+304)	31.77	25.00
2950	seq=translation; coord=9:37790950..37793111:1; parent_transcript=GRMZM2G118873_T01; parent_gene=GRMZM2G118873	GRMZM2G118873_P01,GRMZM2G118873_P02, GRMZM2G121308_P02	TRUE	TRUE	sGSTNLQAPLSVR	95%	n+304 (+304)	58.20	25.89
2951	seq=translation; coord=9:37790950..37793111:1; parent_transcript=GRMZM2G118873_T01; parent_gene=GRMZM2G118873	GRMZM2G118873_P01,GRMZM2G118873_P02, GRMZM2G121308_P02	TRUE	TRUE	sWGAVWQYk	91%	n+304 (+304), K+304 (+304)	27.37	26.30
2952	seq=translation; coord=9:37790950..37793111:1; parent_transcript=GRMZM2G118873_T01; parent_gene=GRMZM2G118873	GRMZM2G118873_P01,GRMZM2G118873_P02, GRMZM2G121308_P02	TRUE	TRUE	vLVASNVIPAGWQPGR	95%	n+304 (+304)	69.77	25.77
2953	seq=translation; coord=2:220832499..220836411:-1; parent_transcript=GRMZM2G148769_T01; parent_gene=GRMZM2G148769	GRMZM2G148769_P01,GRMZM2G148769_P02	TRUE	TRUE	gAFTAVLSGGSLIEALR	95%	n+304 (+304)	90.91	25.37
2954	seq=translation; coord=2:220832499..220836411:-1; parent_transcript=GRMZM2G148769_T01; parent_gene=GRMZM2G148769	GRMZM2G148769_P01,GRMZM2G148769_P02	TRUE	TRUE	wHVFWVDER	95%	n+304 (+304)	40.90	25.00
2955	seq=translation; coord=7:153098952..153101687:1; parent_transcript=GRMZM2G117642_T02; parent_gene=GRMZM2G117642	GRMZM2G117642_P02	TRUE	TRUE	aNNLVFVSGVLGLNPETR	95%	n+304 (+304)	30.12	25.55
2956	seq=translation; coord=7:153098952..153101687:1; parent_transcript=GRMZM2G117642_T02; parent_gene=GRMZM2G117642	GRMZM2G117642_P02	TRUE	TRUE	aPPALGPYSQAIk	95%	n+304 (+304), K+304 (+304)	33.34	25.00
2957	seq=translation; coord=7:153098952..153101687:1; parent_transcript=GRMZM2G117642_T02; parent_gene=GRMZM2G117642	GRMZM2G117642_P02	TRUE	TRUE	sTYQVAALPLNAR	95%	n+304 (+304)	50.00	25.25

2958	seq=translation; coord=7:153098952..153101687:1; parent_transcript=GRMZM2G117642_T02; parent_gene=GRMZM2G117642	GRMZM2G117642_P02	TRUE	TRUE	tTIMLADLQDFNk	90%	n+304 (+304), K+304 (+304)	26.05	25.49
2959	seq=translation; coord=7:153098952..153101687:1; parent_transcript=GRMZM2G117642_T02; parent_gene=GRMZM2G117642	GRMZM2G117642_P02	TRUE	TRUE	yFPVPAPAR	87%	n+304 (+304)	25.58	25.47
2960	seq=translation; coord=4:197363864..197368446:-1; parent_transcript=GRMZM2G002220_T01; parent_gene=GRMZM2G002220	GRMZM2G002220_P01	TRUE	TRUE	aQTLGDTSSLEFMR	95%	n+304 (+304)	48.49	25.00
2961	seq=translation; coord=4:197363864..197368446:-1; parent_transcript=GRMZM2G002220_T01; parent_gene=GRMZM2G002220	GRMZM2G002220_P01	TRUE	TRUE	aVELLYEAALISSGYTPESPAELGGk	95%	n+304 (+304), K+304 (+304)	34.28	25.00
2962	seq=translation; coord=4:197363864..197368446:-1; parent_transcript=GRMZM2G002220_T01; parent_gene=GRMZM2G002220	GRMZM2G002220_P01	TRUE	TRUE	dIEVLYLIEPIDEVAIQNLQTYk	95%	n+304 (+304), K+304 (+304)	31.82	25.00
2963	seq=translation; coord=4:197363864..197368446:-1; parent_transcript=GRMZM2G002220_T01; parent_gene=GRMZM2G002220	GRMZM2G002220_P01	TRUE	TRUE	gVVDSNDLPLNVSR	95%	n+304 (+304)	49.65	25.49
2964	seq=translation; coord=4:197363864..197368446:-1; parent_transcript=GRMZM2G002220_T01; parent_gene=GRMZM2G002220	GRMZM2G002220_P01	TRUE	TRUE	iFEINPDHPIIk	91%	n+304 (+304), K+304 (+304)	25.50	25.00
2965	seq=translation; coord=4:197363864..197368446:-1; parent_transcript=GRMZM2G002220_T01; parent_gene=GRMZM2G002220	GRMZM2G002220_P01	TRUE	TRUE	iYEMMAIALGGR	95%	n+304 (+304)	42.55	25.00
2966	seq=translation; coord=4:197363864..197368446:-1; parent_transcript=GRMZM2G002220_T01; parent_gene=GRMZM2G002220	GRMZM2G002220_P01	TRUE	TRUE	tAPFLEk	95%	n+304 (+304), K+304 (+304)	32.57	26.22
2967	seq=translation; coord=4:197363864..197368446:-1; parent_transcript=GRMZM2G002220_T01; parent_gene=GRMZM2G002220	GRMZM2G002220_P01	TRUE	TRUE	vFISDDFDGELFPR	95%	n+304 (+304)	50.52	25.00
2968	seq=translation; coord=4:197363864..197368446:-1; parent_transcript=GRMZM2G002220_T01; parent_gene=GRMZM2G002220	GRMZM2G002220_P01	TRUE	TRUE	yWDWELANETkPIWMR	93%	n+304 (+304), K+304 (+304)	26.66	25.00
2969	seq=translation; coord=10:34232717..34238135:1; parent_transcript=GRMZM2G016890_T01; parent_gene=GRMZM2G016890	GRMZM2G016890_P01	TRUE	TRUE	eMGMDAYR	88%	n+304 (+304)	26.22	25.00
2970	seq=translation; coord=10:34232717..34238135:1; parent_transcript=GRMZM2G016890_T01; parent_gene=GRMZM2G016890	GRMZM2G016890_P01	TRUE	TRUE	iGLAFDVMGR	95%	n+304 (+304)	43.50	25.00
2971	seq=translation; coord=10:34232717..34238135:1; parent_transcript=GRMZM2G016890_T01; parent_gene=GRMZM2G016890	GRMZM2G016890_P01	TRUE	TRUE	iGLAFDVmGR	87%	n+304 (+304), Oxidation (+16)	25.93	25.00
2972	seq=translation; coord=10:34232717..34238135:1; parent_transcript=GRMZM2G016890_T01; parent_gene=GRMZM2G016890	GRMZM2G016890_P01	TRUE	TRUE	sIVEDYTYFAk	95%	n+304 (+304), K+304 (+304)	36.95	25.00
2973	seq=translation; coord=10:34232717..34238135:1; parent_transcript=GRMZM2G016890_T01; parent_gene=GRMZM2G016890	GRMZM2G016890_P01	TRUE	TRUE	vPYGTSFLDk	95%	n+304 (+304), K+304 (+304)	30.48	26.03
2974	seq=translation; coord=10:34232717..34238135:1; parent_transcript=GRMZM2G016890_T01; parent_gene=GRMZM2G016890	GRMZM2G016890_P01	TRUE	TRUE	yGIVYVDR	89%	n+304 (+304)	26.81	25.00
2975	seq=translation; coord=8:162756043..162756672:-1; parent_transcript=GRMZM2G170969_T01; parent_gene=GRMZM2G170969	GRMZM2G170969_P01	TRUE	TRUE	aIPSAcNLPNAk	95%	n+304 (+304), Carbamidomethyl (+57), K+304 (+304)	54.00	25.50

2976	seq=translation; coord=8:162756043..162756672:-1; parent_transcript=GRMZM2G170969_T01; parent_gene=GRMZM2G170969	GRMZM2G170969_P01	TRUE	TRUE	nADFGcLcSNYWNk	95%	n+304 (+304), Carbamidomethyl (+57), Carbamidomethyl (+57), K+304 (+304)	53.99	25.00
2977	seq=translation; coord=8:162756043..162756672:-1; parent_transcript=GRMZM2G170969_T01; parent_gene=GRMZM2G170969	GRMZM2G170969_P01	TRUE	TRUE	nTPYAScAk	93%	n+304 (+304), Carbamidomethyl (+57), K+304 (+304)	29.31	25.00
2978	seq=translation; coord=8:162756043..162756672:-1; parent_transcript=GRMZM2G170969_T01; parent_gene=GRMZM2G170969	GRMZM2G170969_P01	TRUE	TRUE	qcAScSSGAPSQGccDALR	95%	n+304 (+304), Carbamidomethyl (+57), Carbamidomethyl (+57), Carbamidomethyl (+57), Carbamidomethyl (+57)	70.74	25.00
2979	seq=translation; coord=9:24144173..24150987:-1; parent_transcript=GRMZM2G122135_T03; parent_gene=GRMZM2G122135	GRMZM2G122135_P03	TRUE	TRUE	eTDIVDWFIPVVk	95%	n+304 (+304), K+304 (+304)	34.47	26.00
2980	seq=translation; coord=9:24144173..24150987:-1; parent_transcript=GRMZM2G122135_T03; parent_gene=GRMZM2G122135	GRMZM2G122135_P03	TRUE	TRUE	lAEEFGPEWAMQHIIPQVLEk	95%	n+304 (+304), K+304 (+304)	42.05	25.62
2981	seq=translation; coord=9:24144173..24150987:-1; parent_transcript=GRMZM2G122135_T03; parent_gene=GRMZM2G122135	GRMZM2G122135_P03	TRUE	TRUE	lGALcMQWLEDk	92%	n+304 (+304), Carbamidomethyl (+57), K+304 (+304)	27.46	25.94
2982	seq=translation; coord=9:24144173..24150987:-1; parent_transcript=GRMZM2G122135_T03; parent_gene=GRMZM2G122135	GRMZM2G122135_P03	TRUE	TRUE	mTILQAISLLAPVMGAEITcQk	95%	n+304 (+304), Carbamidomethyl (+57), K+304 (+304)	52.54	25.00
2983	seq=translation; coord=9:24144173..24150987:-1; parent_transcript=GRMZM2G122135_T03; parent_gene=GRMZM2G122135	GRMZM2G122135_P03	TRUE	TRUE	sALASVIMGMAPVLGk	95%	n+304 (+304), K+304 (+304)	35.70	25.20
2984	seq=translation; coord=9:24144173..24150987:-1; parent_transcript=GRMZM2G122135_T03; parent_gene=GRMZM2G122135	GRMZM2G122135_P03	TRUE	TRUE	sLVPILDQSVVEk	95%	n+304 (+304), K+304 (+304)	32.23	25.00
2985	seq=translation; coord=9:24144173..24150987:-1; parent_transcript=GRMZM2G122135_T03; parent_gene=GRMZM2G122135	GRMZM2G122135_P03	TRUE	TRUE	tEIMSIFDDLTQDDQDSVR	95%	n+304 (+304)	35.05	25.00
2986	seq=translation; coord=8:163654067..163656422:-1; parent_transcript=GRMZM2G333861_T01; parent_gene=GRMZM2G333861	GRMZM2G333861_P01	TRUE	TRUE	aSGATWYTAVPTIHQIILDR	95%	n+304 (+304)	40.20	25.34
2987	seq=translation; coord=8:163654067..163656422:-1; parent_transcript=GRMZM2G333861_T01; parent_gene=GRMZM2G333861	GRMZM2G333861_P01	TRUE	TRUE	aVGQELAVLDEEGR	95%	n+304 (+304)	78.39	25.00
2988	seq=translation; coord=8:163654067..163656422:-1; parent_transcript=GRMZM2G333861_T01; parent_gene=GRMZM2G333861	GRMZM2G333861_P01	TRUE	TRUE	fGWFHTGDIGVVDDQGYVR	95%	n+304 (+304)	43.10	25.00
2989	seq=translation; coord=8:163654067..163656422:-1; parent_transcript=GRMZM2G333861_T01; parent_gene=GRMZM2G333861	GRMZM2G333861_P01	TRUE	TRUE	fGWFHTGDIGVVDDQGyVR	95%	n+304 (+304), iTRAQ8plex (+304)	27.27	25.00
2990	seq=translation; coord=8:163654067..163656422:-1; parent_transcript=GRMZM2G333861_T01; parent_gene=GRMZM2G333861	GRMZM2G333861_P01	TRUE	TRUE	fSASTFWADMR	95%	n+304 (+304)	40.01	25.00
2991	seq=translation; coord=8:163654067..163656422:-1; parent_transcript=GRMZM2G333861_T01; parent_gene=GRMZM2G333861	GRMZM2G333861_P01	TRUE	TRUE	gNPEANEAAFR	95%	n+304 (+304)	48.79	25.00
2992	seq=translation; coord=8:163654067..163656422:-1; parent_transcript=GRMZM2G333861_T01; parent_gene=GRMZM2G333861	GRMZM2G333861_P01	TRUE	TRUE	lELTHAALDALVDAAAAR	95%	n+304 (+304)	64.30	26.16

2993	seq=translation; coord=8:163654067..163656422:-1; parent_transcript=GRMZM2G333861_T01; parent_gene=GRMZM2G333861	GRMZM2G333861_P01	TRUE	TRUE	scSASLAPAILER	95%	n+304 (+304), Carbamidomethyl (+57)	48.20	25.00
2994	seq=translation; coord=3:127004080..127006073:-1; parent_transcript=GRMZM2G067456_T01; parent_gene=GRMZM2G067456	GRMZM2G067456_P01,GRMZM2G072729_P01, GRMZM2G072729_P02,GRMZM2G077851_P01	TRUE	TRUE	aMIGQVAGGGR	95%	n+304 (+304)	34.21	25.50
2995	seq=translation; coord=3:127004080..127006073:-1; parent_transcript=GRMZM2G067456_T01; parent_gene=GRMZM2G067456	GRMZM2G067456_P01,GRMZM2G072729_P01, GRMZM2G072729_P02,GRMZM2G077851_P01	TRUE	TRUE	aTLSIGNVLPLR	95%	n+304 (+304)	39.12	25.00
2996	seq=translation; coord=3:127004080..127006073:-1; parent_transcript=GRMZM2G067456_T01; parent_gene=GRMZM2G067456	GRMZM2G067456_P01,GRMZM2G072729_P01, GRMZM2G072729_P02,GRMZM2G077851_P01	TRUE	TRUE	gAPLAk	92%	n+304 (+304), K+304 (+304)	25.86	25.00
2997	seq=translation; coord=3:127004080..127006073:-1; parent_transcript=GRMZM2G067456_T01; parent_gene=GRMZM2G067456	GRMZM2G067456_P01,GRMZM2G072729_P01, GRMZM2G072729_P02,GRMZM2G077851_P01	TRUE	TRUE	gIPEGAVVcNVEHHVGDR	95%	n+304 (+304), Carbamidomethyl (+57)	71.59	25.00
2998	seq=translation; coord=3:127004080..127006073:-1; parent_transcript=GRMZM2G067456_T01; parent_gene=GRMZM2G067456	GRMZM2G067456_P01,GRMZM2G072729_P01, GRMZM2G072729_P02,GRMZM2G077851_P01	TRUE	TRUE	gVVTDVIHDPGR	95%	n+304 (+304)	46.42	25.11
2999	seq=translation; coord=3:127004080..127006073:-1; parent_transcript=GRMZM2G067456_T01; parent_gene=GRMZM2G067456	GRMZM2G067456_P01,GRMZM2G072729_P01, GRMZM2G072729_P02,GRMZM2G077851_P01	TRUE	TRUE	rATLSIGNVLPLR	95%	n+304 (+304)	52.67	25.00
3000	seq=translation; coord=3:208332821..208344531:1; parent_transcript=GRMZM2G085078_T01; parent_gene=GRMZM2G085078	GRMZM2G085078_P01,GRMZM2G085078_P02	TRUE	TRUE	aLPNPGDYHWR	93%	n+304 (+304)	27.53	25.00
3001	seq=translation; coord=3:208332821..208344531:1; parent_transcript=GRMZM2G085078_T01; parent_gene=GRMZM2G085078	GRMZM2G085078_P01,GRMZM2G085078_P02	TRUE	TRUE	dILSDFDNLLPk	95%	n+304 (+304), K+304 (+304)	47.02	26.61
3002	seq=translation; coord=3:208332821..208344531:1; parent_transcript=GRMZM2G085078_T01; parent_gene=GRMZM2G085078	GRMZM2G085078_P01,GRMZM2G085078_P02	TRUE	TRUE	eGYTILVLSDR	95%	n+304 (+304)	50.34	25.67
3003	seq=translation; coord=3:208332821..208344531:1; parent_transcript=GRMZM2G085078_T01; parent_gene=GRMZM2G085078	GRMZM2G085078_P01,GRMZM2G085078_P02	TRUE	TRUE	iPEFNELVHQNR	95%	n+304 (+304)	60.64	25.00
3004	seq=translation; coord=3:208332821..208344531:1; parent_transcript=GRMZM2G085078_T01; parent_gene=GRMZM2G085078	GRMZM2G085078_P01,GRMZM2G085078_P02	TRUE	TRUE	mEPLPDGSMNPR	87%	n+304 (+304)	25.61	25.00
3005	seq=translation; coord=3:208332821..208344531:1; parent_transcript=GRMZM2G085078_T01; parent_gene=GRMZM2G085078	GRMZM2G085078_P01,GRMZM2G085078_P02	TRUE	TRUE	mGHFVTVFER	95%	n+304 (+304)	39.18	25.00
3006	seq=translation; coord=3:208332821..208344531:1; parent_transcript=GRMZM2G085078_T01; parent_gene=GRMZM2G085078	GRMZM2G085078_P01,GRMZM2G085078_P02	TRUE	TRUE	sLLDSNLEDGk	91%	n+304 (+304), K+304 (+304)	26.38	25.44
3007	seq=translation; coord=3:208332821..208344531:1; parent_transcript=GRMZM2G085078_T01; parent_gene=GRMZM2G085078	GRMZM2G085078_P01,GRMZM2G085078_P02	TRUE	TRUE	vAESLGHVILGWR	92%	n+304 (+304)	27.59	25.68
3008	seq=translation; coord=5:154983595..154988021:1; parent_transcript=GRMZM2G167356_T01; parent_gene=GRMZM2G167356	GRMZM2G167356_P01,GRMZM2G167356_P02	TRUE	TRUE	dGASGYGDAGGEDVR	92%	n+304 (+304)	28.70	25.00
3009	seq=translation; coord=5:154983595..154988021:1; parent_transcript=GRMZM2G167356_T01; parent_gene=GRMZM2G167356	GRMZM2G167356_P01,GRMZM2G167356_P02	TRUE	TRUE	gFGFVTFESEDSVER	95%	n+304 (+304)	74.87	25.00
3010	seq=translation; coord=4:28340661..28347586:1; parent_transcript=GRMZM2G426591_T01; parent_gene=GRMZM2G426591	GRMZM2G426591_P01,GRMZM2G426591_P03, GRMZM2G426591_P04	TRUE	TRUE	qVFTPYGDVVHVk	95%	n+304 (+304), K+304 (+304)	40.11	25.80

3011	seq=translation; coord=4:28340661..28347586:1; parent_transcript=GRMZM2G426591_T01; parent_gene=GRMZM2G426591	GRMZM2G426591_P01,GRMZM2G426591_P03, GRMZM2G426591_P04	TRUE	TRUE	qVFTPYGDVVHVk	95%	Pyro-cmC (-17), n+304 (+304), K+304 (+304)	48.23	25.30
3012	seq=translation; coord=4:28340661..28347586:1; parent_transcript=GRMZM2G426591_T01; parent_gene=GRMZM2G426591	GRMZM2G426591_P01,GRMZM2G426591_P03, GRMZM2G426591_P04	TRUE	TRUE	sSAEEALVILQGTLVGGQNVR	95%	n+304 (+304)	62.41	25.85
3013	seq=translation; coord=3:135594596..135597030:-1; parent_transcript=GRMZM2G108348_T01; parent_gene=GRMZM2G108348	GRMZM2G108348_P01,GRMZM2G108348_P03	TRUE	TRUE	hIDFSLSSPFGGGPAGR	95%	n+304 (+304)	46.43	25.00
3014	seq=translation; coord=3:135594596..135597030:-1; parent_transcript=GRMZM2G108348_T01; parent_gene=GRMZM2G108348	GRMZM2G108348_P01,GRMZM2G108348_P03	TRUE	TRUE	iFEGEALLR	90%	n+304 (+304)	27.56	25.00
3015	seq=translation; coord=3:135594596..135597030:-1; parent_transcript=GRMZM2G108348_T01; parent_gene=GRMZM2G108348	GRMZM2G108348_P01,GRMZM2G108348_P03	TRUE	TRUE	kASGGGDAGDEDEE	95%	K+304 (+304), n+304 (+304)	54.89	25.00
3016	seq=translation; coord=3:135594596..135597030:-1; parent_transcript=GRMZM2G108348_T01; parent_gene=GRMZM2G108348	GRMZM2G108348_P01,GRMZM2G108348_P03	TRUE	TRUE	lDYVLALTAENFLAR	95%	n+304 (+304)	31.76	25.42
3017	seq=translation; coord=3:135594596..135597030:-1; parent_transcript=GRMZM2G108348_T01; parent_gene=GRMZM2G108348	GRMZM2G108348_P01,GRMZM2G108348_P03	TRUE	TRUE	qIVNVPSFMVR	91%	n+304 (+304)	28.40	25.19
3018	seq=translation; coord=3:197717130..197718439:-1; parent_transcript=GRMZM2G043521_T01; parent_gene=GRMZM2G043521	GRMZM2G043521_P01	TRUE	TRUE	aDVEAPAEEHPGQADYWLR	95%	n+304 (+304)	54.21	25.00
3019	seq=translation; coord=3:197717130..197718439:-1; parent_transcript=GRMZM2G043521_T01; parent_gene=GRMZM2G043521	GRMZM2G043521_P01	TRUE	TRUE	gFAVDVVPHEMk	95%	n+304 (+304), K+304 (+304)	31.34	25.91
3020	seq=translation; coord=3:197717130..197718439:-1; parent_transcript=GRMZM2G043521_T01; parent_gene=GRMZM2G043521	GRMZM2G043521_P01	TRUE	TRUE	lIGVEYIVSR	95%	n+304 (+304)	36.38	25.00
3021	seq=translation; coord=3:197717130..197718439:-1; parent_transcript=GRMZM2G043521_T01; parent_gene=GRMZM2G043521	GRMZM2G043521_P01	TRUE	TRUE	lPLGAPALMVSPQADPAAAVRPDLVR	90%	n+304 (+304)	25.23	25.00
3022	seq=translation; coord=3:197717130..197718439:-1; parent_transcript=GRMZM2G043521_T01; parent_gene=GRMZM2G043521	GRMZM2G043521_P01	TRUE	TRUE	vLDMGAAAMQSLRPVk	95%	n+304 (+304), K+304 (+304)	28.41	25.65
3023	seq=translation; coord=3:197717130..197718439:-1; parent_transcript=GRMZM2G043521_T01; parent_gene=GRMZM2G043521	GRMZM2G043521_P01	TRUE	TRUE	yGLSTEELR	90%	n+304 (+304)	26.64	25.00
3024	seq=translation; coord=5:72919285..72923425:-1; parent_transcript=GRMZM2G370852_T01; parent_gene=GRMZM2G370852	GRMZM2G370852_P01,GRMZM2G370852_P02, GRMZM2G370852_P03	TRUE	TRUE	dFcLEQNIk	95%	n+304 (+304), Carbamidomethyl (+57), K+304 (+304)	37.82	25.26
3025	seq=translation; coord=5:72919285..72923425:-1; parent_transcript=GRMZM2G370852_T01; parent_gene=GRMZM2G370852	GRMZM2G370852_P01,GRMZM2G370852_P02, GRMZM2G370852_P03	TRUE	TRUE	eGQIYLASPyTAAASALTGYVTDPR	95%	n+304 (+304), iTRAQ8plex (+304)	28.48	25.00
3026	seq=translation; coord=5:72919285..72923425:-1; parent_transcript=GRMZM2G370852_T01; parent_gene=GRMZM2G370852	GRMZM2G370852_P01,GRMZM2G370852_P02, GRMZM2G370852_P03	TRUE	TRUE	fVLDGEMPPYLLAk	95%	n+304 (+304), K+304 (+304)	31.64	25.12
3027	seq=translation; coord=5:72919285..72923425:-1; parent_transcript=GRMZM2G370852_T01; parent_gene=GRMZM2G370852	GRMZM2G370852_P01,GRMZM2G370852_P02, GRMZM2G370852_P03	TRUE	TRUE	sMEFVGSTVESLTMEER	95%	n+304 (+304)	90.94	25.00
3028	seq=translation; coord=5:72919285..72923425:-1; parent_transcript=GRMZM2G370852_T01; parent_gene=GRMZM2G370852	GRMZM2G370852_P01,GRMZM2G370852_P02, GRMZM2G370852_P03	TRUE	TRUE	tEDFLAAAk	95%	n+304 (+304), K+304 (+304)	37.28	26.40

3029	seq=translation; coord=5:72919285..72923425:-1; parent_transcript=GRMZM2G370852_T01; parent_gene=GRMZM2G370852	GRMZM2G370852_P01,GRMZM2G370852_P02, GRMZM2G370852_P03	TRUE	TRUE	tSVDYQPVYSDAEAR	95%	n+304 (+304)	72.66	25.00
3030	seq=translation; coord=5:72919285..72923425:-1; parent_transcript=GRMZM2G370852_T01; parent_gene=GRMZM2G370852	GRMZM2G370852_P01,GRMZM2G370852_P02, GRMZM2G370852_P03	TRUE	TRUE	vPTFLVPATQk	95%	n+304 (+304), K+304 (+304)	31.40	25.00
3031	seq=translation; coord=5:72919285..72923425:-1; parent_transcript=GRMZM2G370852_T01; parent_gene=GRMZM2G370852	GRMZM2G370852_P01,GRMZM2G370852_P02, GRMZM2G370852_P03	TRUE	TRUE	vVIIPDHYIFTSDER	95%	n+304 (+304)	55.21	25.00
3032	seq=translation; coord=5:72919285..72923425:-1; parent_transcript=GRMZM2G370852_T01; parent_gene=GRMZM2G370852	GRMZM2G370852_P01,GRMZM2G370852_P02, GRMZM2G370852_P03	TRUE	TRUE	vWMDVYSLPVPGSGGk	95%	n+304 (+304), K+304 (+304)	40.45	25.84
3033	seq=translation; coord=4:175134158..175138628:1; parent_transcript=GRMZM2G070239_T01; parent_gene=GRMZM2G070239	GRMZM2G070239_P01,GRMZM2G070239_P02	TRUE	TRUE	kPPGFAFIDFDDR	95%	K+304 (+304), n+304 (+304)	35.23	25.83
3034	seq=translation; coord=4:175134158..175138628:1; parent_transcript=GRMZM2G070239_T01; parent_gene=GRMZM2G070239	GRMZM2G070239_P01,GRMZM2G070239_P02	TRUE	TRUE	vTSGELEDEFR	95%	n+304 (+304)	60.44	25.00
3035	seq=translation; coord=4:175134158..175138628:1; parent_transcript=GRMZM2G070239_T01; parent_gene=GRMZM2G070239	GRMZM2G070239_P01,GRMZM2G070239_P02	TRUE	TRUE	vYVGNLDAR	90%	n+304 (+304)	27.68	25.00
3036	seq=translation; coord=4:179822113..179823382:-1; parent_transcript=GRMZM2G007729_T02; parent_gene=GRMZM2G007729	GRMZM2G007729_P02	TRUE	TRUE	dYAVPSLFSDIFR	95%	n+304 (+304)	39.40	25.00
3037	seq=translation; coord=4:179822113..179823382:-1; parent_transcript=GRMZM2G007729_T02; parent_gene=GRMZM2G007729	GRMZM2G007729_P02	TRUE	TRUE	dYAVPSLFSDIFRDPLSAPHSIGR	95%	n+304 (+304)	32.94	25.07
3038	seq=translation; coord=4:179822113..179823382:-1; parent_transcript=GRMZM2G007729_T02; parent_gene=GRMZM2G007729	GRMZM2G007729_P02	TRUE	TRUE	lLNLVDDLAVAAPGR	95%	n+304 (+304)	49.90	25.85
3039	seq=translation; coord=4:179822113..179823382:-1; parent_transcript=GRMZM2G007729_T02; parent_gene=GRMZM2G007729	GRMZM2G007729_P02	TRUE	TRUE	vDMPGLGk	95%	n+304 (+304), K+304 (+304)	30.81	25.15
3040	seq=translation; coord=5:68020016..68022909:-1; parent_transcript=GRMZM2G108474_T01; parent_gene=GRMZM2G108474	GRMZM2G108474_P01	TRUE	TRUE	dDASVVFAYYk	95%	n+304 (+304), K+304 (+304)	42.56	25.38
3041	seq=translation; coord=5:68020016..68022909:-1; parent_transcript=GRMZM2G108474_T01; parent_gene=GRMZM2G108474	GRMZM2G108474_P01	TRUE	TRUE	dLQFFVGESMk	95%	n+304 (+304), K+304 (+304)	39.43	25.45
3042	seq=translation; coord=5:68020016..68022909:-1; parent_transcript=GRMZM2G108474_T01; parent_gene=GRMZM2G108474	GRMZM2G108474_P01	TRUE	TRUE	mLVYQDLLSGDELLSDSFTYk	95%	n+304 (+304), K+304 (+304)	49.58	25.00
3043	seq=translation; coord=5:68020016..68022909:-1; parent_transcript=GRMZM2G108474_T01; parent_gene=GRMZM2G108474	GRMZM2G108474_P01	TRUE	TRUE	mLVYQDLLSGDELLSDSFTyk	95%	n+304 (+304), iTRAQ8plex (+304), K+304 (+304)	28.07	25.00
3044	seq=translation; coord=5:68020016..68022909:-1; parent_transcript=GRMZM2G108474_T01; parent_gene=GRMZM2G108474	GRMZM2G108474_P01	TRUE	TRUE	nLTAVLEPEk	95%	n+304 (+304), K+304 (+304)	32.74	25.04
3045	seq=translation; coord=5:68020016..68022909:-1; parent_transcript=GRMZM2G108474_T01; parent_gene=GRMZM2G108474	GRMZM2G108474_P01	TRUE	TRUE	sFVSYIk	95%	n+304 (+304), K+304 (+304)	37.42	25.00
3046	seq=translation; coord=5:68020016..68022909:-1; parent_transcript=GRMZM2G108474_T01; parent_gene=GRMZM2G108474	GRMZM2G108474_P01	TRUE	TRUE	vVDIVDTFR	95%	n+304 (+304)	38.65	25.00

3047	seq=translation; coord=5:213336713..213337573:1; parent_transcript=GRMZM2G119782_T01; parent_gene=GRMZM2G119782	GRMZM2G119782_P01	TRUE	TRUE	aYDGTDPSkPIYVSVR	95%	n+304 (+304), K+304 (+304)	42.03	25.91
3048	seq=translation; coord=5:213336713..213337573:1; parent_transcript=GRMZM2G119782_T01; parent_gene=GRMZM2G119782	GRMZM2G119782_P01	TRUE	TRUE	dEADVSGDLSGLTDk	95%	n+304 (+304), K+304 (+304)	55.21	25.00
3049	seq=translation; coord=5:213336713..213337573:1; parent_transcript=GRMZM2G119782_T01; parent_gene=GRMZM2G119782	GRMZM2G119782_P01	TRUE	TRUE	gFYGPGGAYAVFAGR	95%	n+304 (+304)	54.23	25.00
3050	seq=translation; coord=5:213336713..213337573:1; parent_transcript=GRMZM2G119782_T01; parent_gene=GRMZM2G119782	GRMZM2G119782_P01	TRUE	TRUE	gFyGPGGAYAVFAGR	95%	n+304 (+304), iTRAQ8plex (+304)	47.88	25.74
3051	seq=translation; coord=5:213336713..213337573:1; parent_transcript=GRMZM2G119782_T01; parent_gene=GRMZM2G119782	GRMZM2G119782_P01	TRUE	TRUE	yPVVAR	87%	n+304 (+304)	25.04	25.00
3052	seq=translation; coord=3:215654139..215663757:-1; parent_transcript=GRMZM2G125193_T01; parent_gene=GRMZM2G125193	GRMZM2G125193_P01	TRUE	TRUE	eAMDkGELVSDNLVVGIIDEAMk	95%	n+304 (+304), K+304 (+304), K+304 (+304)	29.95	25.00
3053	seq=translation; coord=3:215654139..215663757:-1; parent_transcript=GRMZM2G125193_T01; parent_gene=GRMZM2G125193	GRMZM2G125193_P01	TRUE	TRUE	gELVSDNLVVGIIDEAMk	95%	n+304 (+304), K+304 (+304)	57.31	25.90
3054	seq=translation; coord=3:215654139..215663757:-1; parent_transcript=GRMZM2G125193_T01; parent_gene=GRMZM2G125193	GRMZM2G125193_P01	TRUE	TRUE	vLNFAIDDAILEER	95%	n+304 (+304)	74.86	25.35
3055	seq=translation; coord=7:134970635..134972839:-1; parent_transcript=GRMZM2G052266_T01; parent_gene=GRMZM2G052266	GRMZM2G052266_P01,GRMZM2G052266_P02	TRUE	TRUE	aVAMFQALAVk	95%	n+304 (+304), K+304 (+304)	52.83	25.00
3056	seq=translation; coord=7:134970635..134972839:-1; parent_transcript=GRMZM2G052266_T01; parent_gene=GRMZM2G052266	GRMZM2G052266_P01,GRMZM2G052266_P02	TRUE	TRUE	dDSALVASMR	95%	n+304 (+304)	33.07	25.00
3057	seq=translation; coord=7:134970635..134972839:-1; parent_transcript=GRMZM2G052266_T01; parent_gene=GRMZM2G052266	GRMZM2G052266_P01,GRMZM2G052266_P02	TRUE	TRUE	dWSTGAGDLVEPVAR	95%	n+304 (+304)	66.62	25.00
3058	seq=translation; coord=7:134970635..134972839:-1; parent_transcript=GRMZM2G052266_T01; parent_gene=GRMZM2G052266	GRMZM2G052266_P01,GRMZM2G052266_P02	TRUE	TRUE	lWDEAQADAGYTVLTPTPHLDLGPR	95%	n+304 (+304)	73.51	25.01
3059	seq=translation; coord=7:134970635..134972839:-1; parent_transcript=GRMZM2G052266_T01; parent_gene=GRMZM2G052266	GRMZM2G052266_P01,GRMZM2G052266_P02	TRUE	TRUE	sVTGLELPVQPVHTLIcYWk	95%	n+304 (+304), Carbamidomethyl (+57), K+304 (+304)	28.08	25.33
3060	seq=translation; coord=7:134970635..134972839:-1; parent_transcript=GRMZM2G052266_T01; parent_gene=GRMZM2G052266	GRMZM2G052266_P01,GRMZM2G052266_P02	TRUE	TRUE	vLLLER	95%	n+304 (+304)	29.99	25.00
3061	seq=translation; coord=3:202362837..202365659:1; parent_transcript=GRMZM2G161868_T01; parent_gene=GRMZM2G161868	GRMZM2G161868_P01	TRUE	TRUE	fDYILTQQAFVAVDk	95%	n+304 (+304), K+304 (+304)	34.18	25.95
3062	seq=translation; coord=3:202362837..202365659:1; parent_transcript=GRMZM2G161868_T01; parent_gene=GRMZM2G161868	GRMZM2G161868_P01	TRUE	TRUE	gILLGAVHGIVEALFR	95%	n+304 (+304)	74.97	25.00
3063	seq=translation; coord=3:202362837..202365659:1; parent_transcript=GRMZM2G161868_T01; parent_gene=GRMZM2G161868	GRMZM2G161868_P01	TRUE	TRUE	gILLGAVHGIVEALFRR	94%	n+304 (+304)	25.28	25.00
3064	seq=translation; coord=3:202362837..202365659:1; parent_transcript=GRMZM2G161868_T01; parent_gene=GRMZM2G161868	GRMZM2G161868_P01	TRUE	TRUE	rYTEQGMDEESAYk	95%	n+304 (+304), K+304 (+304)	36.00	25.00

3065	seq=translation; coord=3:202362837..202365659:1; parent_transcript=GRMZM2G161868_T01; parent_gene=GRMZM2G161868	GRMZM2G161868_P01	TRUE	TRUE	yTEQGMDEESAYk	95%	n+304 (+304), K+304 (+304)	49.78	25.00
3066	seq=translation; coord=8:60632430..60633244:-1; parent_transcript=GRMZM2G050607_T01; parent_gene=GRMZM2G050607	GRMZM2G050607_P01	TRUE	TRUE	dAGRDWDIDYEMR	91%	n+304 (+304)	25.22	25.00
3067	seq=translation; coord=8:60632430..60633244:-1; parent_transcript=GRMZM2G050607_T01; parent_gene=GRMZM2G050607	GRMZM2G050607_P01	TRUE	TRUE	dWDIDYEMR	95%	n+304 (+304)	39.86	25.00
3068	seq=translation; coord=8:60632430..60633244:-1; parent_transcript=GRMZM2G050607_T01; parent_gene=GRMZM2G050607	GRMZM2G050607_P01	TRUE	TRUE	tVASGTVPDPGSLAGDGATTR	95%	n+304 (+304)	47.03	25.00
3069	seq=translation; coord=8:60632430..60633244:-1; parent_transcript=GRMZM2G050607_T01; parent_gene=GRMZM2G050607	GRMZM2G050607_P01	TRUE	TRUE	vGLTVDLPVVGk	95%	n+304 (+304), K+304 (+304)	60.08	25.00
3070	seq=translation; coord=8:60632430..60633244:-1; parent_transcript=GRMZM2G050607_T01; parent_gene=GRMZM2G050607	GRMZM2G050607_P01	TRUE	TRUE	vPYDFLVSLAk	95%	n+304 (+304), K+304 (+304)	34.99	25.00
3071	seq=translation; coord=1:15773796..15778609:-1; parent_transcript=GRMZM2G119852_T01; parent_gene=GRMZM2G119852	GRMZM2G119852_P01	TRUE	TRUE	aHLLFDLHQAVDGLR	94%	n+304 (+304)	25.90	25.35
3072	seq=translation; coord=1:15773796..15778609:-1; parent_transcript=GRMZM2G119852_T01; parent_gene=GRMZM2G119852	GRMZM2G119852_P01	TRUE	TRUE	aIGDLIGVVk	95%	n+304 (+304), K+304 (+304)	38.19	25.00
3073	seq=translation; coord=1:15773796..15778609:-1; parent_transcript=GRMZM2G119852_T01; parent_gene=GRMZM2G119852	GRMZM2G119852_P01	TRUE	TRUE	iEELVGVPVHYIGVGPGR	95%	n+304 (+304)	56.66	25.47
3074	seq=translation; coord=1:15773796..15778609:-1; parent_transcript=GRMZM2G119852_T01; parent_gene=GRMZM2G119852	GRMZM2G119852_P01	TRUE	TRUE	lDVLSGLSEIk	95%	n+304 (+304), K+304 (+304)	37.50	25.00
3075	seq=translation; coord=1:15773796..15778609:-1; parent_transcript=GRMZM2G119852_T01; parent_gene=GRMZM2G119852	GRMZM2G119852_P01	TRUE	TRUE	lEPFIADTVHVLNESIk	95%	n+304 (+304), K+304 (+304)	33.37	25.63
3076	seq=translation; coord=1:15773796..15778609:-1; parent_transcript=GRMZM2G119852_T01; parent_gene=GRMZM2G119852	GRMZM2G119852_P01	TRUE	TRUE	lVDVLAPR	90%	n+304 (+304)	28.63	25.99
3077	seq=translation; coord=1:15773796..15778609:-1; parent_transcript=GRMZM2G119852_T01; parent_gene=GRMZM2G119852	GRMZM2G119852_P01	TRUE	TRUE	vSSLTQVSGVLGSQWGDEGk	95%	n+304 (+304), K+304 (+304)	65.53	25.25
3078	seq=translation; coord=8:170316262..170329055:1; parent_transcript=GRMZM2G166345_T02; parent_gene=GRMZM2G166345	GRMZM2G166345_P02	TRUE	TRUE	fcSDFTASDGk	95%	n+304 (+304), Carbamidomethyl (+57), K+304 (+304)	33.85	25.00
3079	seq=translation; coord=8:170316262..170329055:1; parent_transcript=GRMZM2G166345_T02; parent_gene=GRMZM2G166345	GRMZM2G166345_P02	TRUE	TRUE	gVPFTTSAGPcR	95%	n+304 (+304), Carbamidomethyl (+57)	52.17	25.00
3080	seq=translation; coord=8:170316262..170329055:1; parent_transcript=GRMZM2G166345_T02; parent_gene=GRMZM2G166345	GRMZM2G166345_P02	TRUE	TRUE	iADkESEcDISILNIQVGLIR	95%	n+304 (+304), K+304 (+304), Carbamidomethyl (+57)	42.61	25.09
3081	seq=translation; coord=8:170316262..170329055:1; parent_transcript=GRMZM2G166345_T02; parent_gene=GRMZM2G166345	GRMZM2G166345_P02	TRUE	TRUE	lSAADNDVFATVHPFVIR	95%	n+304 (+304)	76.20	25.84
3082	seq=translation; coord=8:170316262..170329055:1; parent_transcript=GRMZM2G166345_T02; parent_gene=GRMZM2G166345	GRMZM2G166345_P02	TRUE	TRUE	qHALLDNLNQELSQk	95%	n+304 (+304), K+304 (+304)	48.82	25.22

3083	seq=translation; coord=8:170316262..170329055:1; parent_transcript=GRMZM2G166345_T02; parent_gene=GRMZM2G166345	GRMZM2G166345_P02	TRUE	TRUE	qHALLDNLNQELSQk	95%	Pyro-cmC (-17), n+304 (+304), K+304 (+304)	45.83	25.43
3084	seq=translation; coord=8:170316262..170329055:1; parent_transcript=GRMZM2G166345_T02; parent_gene=GRMZM2G166345	GRMZM2G166345_P02	TRUE	TRUE	sVLLGDGFk	95%	n+304 (+304), K+304 (+304)	33.34	26.16
3085	seq=translation; coord=8:170316262..170329055:1; parent_transcript=GRMZM2G166345_T02; parent_gene=GRMZM2G166345	GRMZM2G166345_P02	TRUE	TRUE	wMDYIQNTVGAGTTLQk	94%	n+304 (+304), K+304 (+304)	29.14	26.04
3086	seq=translation; coord=8:170316262..170329055:1; parent_transcript=GRMZM2G166345_T02; parent_gene=GRMZM2G166345	GRMZM2G166345_P02	TRUE	TRUE	ycSPDDLTNR	94%	n+304 (+304), Carbamidomethyl (+57)	30.94	25.00
3087	seq=translation; coord=5:75935116..75939320:-1; parent_transcript=GRMZM2G019121_T01; parent_gene=GRMZM2G019121	GRMZM2G019121_P01	TRUE	TRUE	aIPTLPINLEDAAR	95%	n+304 (+304)	38.40	26.56
3088	seq=translation; coord=5:75935116..75939320:-1; parent_transcript=GRMZM2G019121_T01; parent_gene=GRMZM2G019121	GRMZM2G019121_P01	TRUE	TRUE	aTTQQVEIQVR	95%	n+304 (+304)	43.86	26.51
3089	seq=translation; coord=5:75935116..75939320:-1; parent_transcript=GRMZM2G019121_T01; parent_gene=GRMZM2G019121	GRMZM2G019121_P01	TRUE	TRUE	gEEIISGAQR	88%	n+304 (+304)	27.64	26.16
3090	seq=translation; coord=5:75935116..75939320:-1; parent_transcript=GRMZM2G019121_T01; parent_gene=GRMZM2G019121	GRMZM2G019121_P01	TRUE	TRUE	qMAISGGFER	90%	n+304 (+304)	26.38	25.00
3091	seq=translation; coord=6:158451653..158457625:-1; parent_transcript=GRMZM2G176396_T01; parent_gene=GRMZM2G176396	GRMZM2G176396_P01,GRMZM2G176396_P02, GRMZM2G176396_P03	TRUE	TRUE	aWPEAEFk	89%	n+304 (+304), K+304 (+304)	26.82	25.19
3092	seq=translation; coord=6:158451653..158457625:-1; parent_transcript=GRMZM2G176396_T01; parent_gene=GRMZM2G176396	GRMZM2G176396_P01,GRMZM2G176396_P02, GRMZM2G176396_P03	TRUE	TRUE	dFIPEDER	89%	n+304 (+304)	25.79	25.00
3093	seq=translation; coord=6:158451653..158457625:-1; parent_transcript=GRMZM2G176396_T01; parent_gene=GRMZM2G176396	GRMZM2G176396_P01,GRMZM2G176396_P02, GRMZM2G176396_P03	TRUE	TRUE	gEDDEFSLAFAR	95%	n+304 (+304)	43.61	25.00
3094	seq=translation; coord=6:158451653..158457625:-1; parent_transcript=GRMZM2G176396_T01; parent_gene=GRMZM2G176396	GRMZM2G176396_P01,GRMZM2G176396_P02, GRMZM2G176396_P03	TRUE	TRUE	gFLPSDSFLLDNVDk	95%	n+304 (+304), K+304 (+304)	38.75	25.12
3095	seq=translation; coord=6:158451653..158457625:-1; parent_transcript=GRMZM2G176396_T01; parent_gene=GRMZM2G176396	GRMZM2G176396_P01,GRMZM2G176396_P02, GRMZM2G176396_P03	TRUE	TRUE	rGEDDEFSLAFAR	95%	n+304 (+304)	61.19	25.00
3096	seq=translation; coord=5:163209220..163228678:1; parent_transcript=GRMZM2G012690_T01; parent_gene=GRMZM2G012690	GRMZM2G012690_P01	TRUE	TRUE	eVLTDLEGMGFSTAR	95%	n+304 (+304)	49.71	25.00
3097	seq=translation; coord=5:163209220..163228678:1; parent_transcript=GRMZM2G012690_T01; parent_gene=GRMZM2G012690	GRMZM2G012690_P01	TRUE	TRUE	lEGDEFLFLAR	95%	n+304 (+304)	40.64	25.56
3098	seq=translation; coord=5:163209220..163228678:1; parent_transcript=GRMZM2G012690_T01; parent_gene=GRMZM2G012690	GRMZM2G012690_P01	TRUE	TRUE	vGNLHGGIEFLELcGFEk	95%	n+304 (+304), Carbamidomethyl (+57), K+304 (+304)	32.71	25.31
3099	seq=translation; coord=4:161070624..161077204:-1; parent_transcript=GRMZM2G015875_T01; parent_gene=GRMZM2G015875	GRMZM2G015875_P01	TRUE	TRUE	dFELELER	95%	n+304 (+304)	33.94	25.00
3100	seq=translation; coord=4:161070624..161077204:-1; parent_transcript=GRMZM2G015875_T01; parent_gene=GRMZM2G015875	GRMZM2G015875_P01	TRUE	TRUE	eQEESLQEWEk	95%	n+304 (+304), K+304 (+304)	32.46	25.00

3101	seq=translation; coord=4:161070624..161077204:-1; parent_transcript=GRMZM2G015875_T01; parent_gene=GRMZM2G015875	GRMZM2G015875_P01	TRUE	TRUE	gWTGWSTPTPANQR	95%	n+304 (+304)	46.24	25.00
3102	seq=translation; coord=4:161070624..161077204:-1; parent_transcript=GRMZM2G015875_T01; parent_gene=GRMZM2G015875	GRMZM2G015875_P01	TRUE	TRUE	kLWSFFTT	93%	K+304 (+304), n+304 (+304)	28.72	26.19
3103	seq=translation; coord=4:161070624..161077204:-1; parent_transcript=GRMZM2G015875_T01; parent_gene=GRMZM2G015875	GRMZM2G015875_P01	TRUE	TRUE	mILGEVFEEk	95%	n+304 (+304), K+304 (+304)	40.77	26.54
3104	seq=translation; coord=4:161070624..161077204:-1; parent_transcript=GRMZM2G015875_T01; parent_gene=GRMZM2G015875	GRMZM2G015875_P01	TRUE	TRUE	qTAGAVTDAPGER	95%	n+304 (+304)	50.99	25.00
3105	seq=translation; coord=4:161070624..161077204:-1; parent_transcript=GRMZM2G015875_T01; parent_gene=GRMZM2G015875	GRMZM2G015875_P01	TRUE	TRUE	tDDQGDTVk	95%	n+304 (+304), K+304 (+304)	38.27	25.00
3106	seq=translation; coord=6:71732900..71733812:-1; parent_transcript=GRMZM2G101859_T01; parent_gene=GRMZM2G101859	GRMZM2G101859_P01	TRUE	TRUE	aYGELPDTLR	95%	n+304 (+304)	33.85	25.00
3107	seq=translation; coord=6:71732900..71733812:-1; parent_transcript=GRMZM2G101859_T01; parent_gene=GRMZM2G101859	GRMZM2G101859_P01	TRUE	TRUE	eDGQEYAQVTR	95%	n+304 (+304)	42.16	25.00
3108	seq=translation; coord=6:71732900..71733812:-1; parent_transcript=GRMZM2G101859_T01; parent_gene=GRMZM2G101859	GRMZM2G101859_P01	TRUE	TRUE	kVWIAAGDIVLVGLR	95%	K+304 (+304), n+304 (+304)	48.57	25.00
3109	seq=translation; coord=6:71732900..71733812:-1; parent_transcript=GRMZM2G101859_T01; parent_gene=GRMZM2G101859	GRMZM2G101859_P01	TRUE	TRUE	vWIAAGDIVLVGLR	87%	n+304 (+304)	25.88	25.00
3110	seq=translation; coord=6:71732900..71733812:-1; parent_transcript=GRMZM2G101859_T01; parent_gene=GRMZM2G101859	GRMZM2G101859_P01	TRUE	TRUE	yMNDEAR	89%	n+304 (+304)	26.09	25.00
3111	seq=translation; coord=7:165243551..165244574:-1; parent_transcript=GRMZM2G030731_T01; parent_gene=GRMZM2G030731	GRMZM2G030731_P01,GRMZM2G096123_P01	TRUE	TRUE	aAGGAVVLTA	88%	n+304 (+304)	28.25	27.12
3112	seq=translation; coord=7:165243551..165244574:-1; parent_transcript=GRMZM2G030731_T01; parent_gene=GRMZM2G030731	GRMZM2G030731_P01,GRMZM2G096123_P01	TRUE	TRUE	aPQIDVTQFGYFk	95%	n+304 (+304), K+304 (+304)	53.11	25.66
3113	seq=translation; coord=7:165243551..165244574:-1; parent_transcript=GRMZM2G030731_T01; parent_gene=GRMZM2G030731	GRMZM2G030731_P01,GRMZM2G096123_P01	TRUE	TRUE	fYcPAVNIER	95%	n+304 (+304), Carbamidomethyl (+57)	32.31	25.00
3114	seq=translation; coord=2:192576240..192582957:1; parent_transcript=GRMZM2G097226_T01; parent_gene=GRMZM2G097226	GRMZM2G097226_P01,GRMZM2G097226_P02	TRUE	TRUE	mVGYALQAAEILSk	95%	n+304 (+304), K+304 (+304)	45.31	25.21
3115	seq=translation; coord=2:192576240..192582957:1; parent_transcript=GRMZM2G097226_T01; parent_gene=GRMZM2G097226	GRMZM2G097226_P01,GRMZM2G097226_P02	TRUE	TRUE	vLTPYSSEDAR	86%	n+304 (+304)	25.39	25.00
3116	seq=translation; coord=4:230189571..230193924:-1; parent_transcript=GRMZM2G161969_T01; parent_gene=GRMZM2G161969	GRMZM2G161969_P01	TRUE	TRUE	aDIEDYLAk	95%	n+304 (+304), K+304 (+304)	39.40	26.39
3117	seq=translation; coord=4:230189571..230193924:-1; parent_transcript=GRMZM2G161969_T01; parent_gene=GRMZM2G161969	GRMZM2G161969_P01	TRUE	TRUE	aQPEPSQPk	95%	n+304 (+304), K+304 (+304)	35.72	26.61
3118	seq=translation; coord=4:230189571..230193924:-1; parent_transcript=GRMZM2G161969_T01; parent_gene=GRMZM2G161969	GRMZM2G161969_P01	TRUE	TRUE	eAFAAPGLGYVDIPNAQIR	95%	n+304 (+304)	31.76	25.37

3119	seq=translation; coord=4:230189571..230193924:-1; parent_transcript=GRMZM2G161969_T01; parent_gene=GRMZM2G161969	GRMZM2G161969_P01	TRUE	TRUE	gLGTIAEEVk	95%	n+304 (+304), K+304 (+304)	58.27	25.42
3120	seq=translation; coord=1:46852658..46861936:1; parent_transcript=GRMZM2G010328_T01; parent_gene=GRMZM2G010328	GRMZM2G010328_P01	TRUE	TRUE	aAHLLk	93%	n+304 (+304), K+304 (+304)	26.52	25.00
3121	seq=translation; coord=1:46852658..46861936:1; parent_transcript=GRMZM2G010328_T01; parent_gene=GRMZM2G010328	GRMZM2G010328_P01	TRUE	TRUE	aNVVTTPGTGFGPGGEGFVR	95%	n+304 (+304)	91.29	25.01
3122	seq=translation; coord=1:46852658..46861936:1; parent_transcript=GRMZM2G010328_T01; parent_gene=GRMZM2G010328	GRMZM2G010328_P01	TRUE	TRUE	lQAGYLFPEIAR	95%	n+304 (+304)	41.01	26.23
3123	seq=translation; coord=1:46852658..46861936:1; parent_transcript=GRMZM2G010328_T01; parent_gene=GRMZM2G010328	GRMZM2G010328_P01	TRUE	TRUE	nAPYVWVHFPGR	95%	n+304 (+304)	55.93	25.00
3124	seq=translation; coord=1:46852658..46861936:1; parent_transcript=GRMZM2G010328_T01; parent_gene=GRMZM2G010328	GRMZM2G010328_P01	TRUE	TRUE	nSWDVFAEILEk	95%	n+304 (+304), K+304 (+304)	51.14	25.60
3125	seq=translation; coord=5:741455..752529:1; parent_transcript=GRMZM2G022258_T01; parent_gene=GRMZM2G022258	GRMZM2G022258_P01,GRMZM2G022258_P02	TRUE	TRUE	dLSQPIDVTLLDATVAAFYGTGSk	95%	n+304 (+304), K+304 (+304)	32.39	25.00
3126	seq=translation; coord=5:741455..752529:1; parent_transcript=GRMZM2G022258_T01; parent_gene=GRMZM2G022258	GRMZM2G022258_P01,GRMZM2G022258_P02	TRUE	TRUE	dLYAEEAAAQR	95%	n+304 (+304)	47.47	25.00
3127	seq=translation; coord=5:741455..752529:1; parent_transcript=GRMZM2G022258_T01; parent_gene=GRMZM2G022258	GRMZM2G022258_P01,GRMZM2G022258_P02	TRUE	TRUE	eSEVLSLFATIINk	95%	n+304 (+304), K+304 (+304)	40.46	25.00
3128	seq=translation; coord=5:741455..752529:1; parent_transcript=GRMZM2G022258_T01; parent_gene=GRMZM2G022258	GRMZM2G022258_P01,GRMZM2G022258_P02	TRUE	TRUE	fFALQVLESVIk	95%	n+304 (+304), K+304 (+304)	37.02	25.00
3129	seq=translation; coord=5:741455..752529:1; parent_transcript=GRMZM2G022258_T01; parent_gene=GRMZM2G022258	GRMZM2G022258_P01,GRMZM2G022258_P02	TRUE	TRUE	fLVMVIR	87%	n+304 (+304)	25.17	25.00
3130	seq=translation; coord=5:741455..752529:1; parent_transcript=GRMZM2G022258_T01; parent_gene=GRMZM2G022258	GRMZM2G022258_P01,GRMZM2G022258_P02	TRUE	TRUE	lLSEEIFDFSR	95%	n+304 (+304)	46.37	25.00
3131	seq=translation; coord=5:741455..752529:1; parent_transcript=GRMZM2G022258_T01; parent_gene=GRMZM2G022258	GRMZM2G022258_P01,GRMZM2G022258_P02	TRUE	TRUE	lNIILVQVLk	95%	n+304 (+304), K+304 (+304)	49.72	25.00
3132	seq=translation; coord=5:741455..752529:1; parent_transcript=GRMZM2G022258_T01; parent_gene=GRMZM2G022258	GRMZM2G022258_P01,GRMZM2G022258_P02	TRUE	TRUE	lVIDSINWAFR	95%	n+304 (+304)	31.78	25.98
3133	seq=translation; coord=5:741455..752529:1; parent_transcript=GRMZM2G022258_T01; parent_gene=GRMZM2G022258	GRMZM2G022258_P01,GRMZM2G022258_P02	TRUE	TRUE	nIAETGLSLLLEILk	95%	n+304 (+304), K+304 (+304)	32.57	25.00
3134	seq=translation; coord=2:133839420..133842192:1; parent_transcript=GRMZM2G111143_T01; parent_gene=GRMZM2G111143	GRMZM2G111143_P01,GRMZM2G111143_P02, GRMZM2G111143_P03	TRUE	TRUE	aEAGQVGVGPEEAR	95%	n+304 (+304)	39.06	25.00
3135	seq=translation; coord=2:133839420..133842192:1; parent_transcript=GRMZM2G111143_T01; parent_gene=GRMZM2G111143	GRMZM2G111143_P01,GRMZM2G111143_P02, GRMZM2G111143_P03	TRUE	TRUE	nFGIFNTDLTPk	93%	n+304 (+304), K+304 (+304)	29.22	26.80
3136	seq=translation; coord=2:133839420..133842192:1; parent_transcript=GRMZM2G111143_T01; parent_gene=GRMZM2G111143	GRMZM2G111143_P01,GRMZM2G111143_P02, GRMZM2G111143_P03	TRUE	TRUE	tFETYVFSLFDENQkPGPVAER	95%	n+304 (+304), K+304 (+304)	30.27	25.00

3137	seq=translation; coord=2:133839420..133842192:1; parent_transcript=GRMZM2G111143_T01; parent_gene=GRMZM2G111143	GRMZM2G111143_P01,GRMZM2G111143_P02, GRMZM2G111143_P03	TRUE	TRUE	yDLGLLR	90%	n+304 (+304)	27.25	25.00
3138	seq=translation; coord=2:4174237..4178923:-1; parent_transcript=GRMZM2G039588_T03; parent_gene=GRMZM2G039588	GRMZM2G039588_P03	TRUE	TRUE	gGIPIcFPQFSNFGNLEPHGFAR	95%	n+304 (+304), Carbamidomethyl (+57)	78.13	25.00
3139	seq=translation; coord=2:4174237..4178923:-1; parent_transcript=GRMZM2G039588_T03; parent_gene=GRMZM2G039588	GRMZM2G039588_P03	TRUE	TRUE	iSDISEVR	94%	n+304 (+304)	30.82	25.25
3140	seq=translation; coord=2:4174237..4178923:-1; parent_transcript=GRMZM2G039588_T03; parent_gene=GRMZM2G039588	GRMZM2G039588_P03	TRUE	TRUE	lELSAVPSSYYSGQLDPDR	95%	n+304 (+304)	31.42	25.00
3141	seq=translation; coord=2:4174237..4178923:-1; parent_transcript=GRMZM2G039588_T03; parent_gene=GRMZM2G039588	GRMZM2G039588_P03	TRUE	TRUE	sTEQGDAIVFESELDR	95%	n+304 (+304)	60.90	25.00
3142	seq=translation; coord=2:4174237..4178923:-1; parent_transcript=GRMZM2G039588_T03; parent_gene=GRMZM2G039588	GRMZM2G039588_P03	TRUE	TRUE	vALSPGGDLMLTSR	95%	n+304 (+304)	59.92	25.40
3143	seq=translation; coord=2:4174237..4178923:-1; parent_transcript=GRMZM2G039588_T03; parent_gene=GRMZM2G039588	GRMZM2G039588_P03	TRUE	TRUE	vEGLETLDYLDNLQDR	95%	n+304 (+304)	76.88	25.00
3144	seq=translation; coord=4:155907661..155908892:-1; parent_transcript=GRMZM2G044627_T01; parent_gene=GRMZM2G044627	GRMZM2G044627_P01	TRUE	TRUE	eVDLPASTTAGAGR	95%	n+304 (+304)	53.48	25.00
3145	seq=translation; coord=4:155907661..155908892:-1; parent_transcript=GRMZM2G044627_T01; parent_gene=GRMZM2G044627	GRMZM2G044627_P01	TRUE	TRUE	gDALPLGLPQIMMVLTR	95%	n+304 (+304)	37.17	25.65
3146	seq=translation; coord=4:155907661..155908892:-1; parent_transcript=GRMZM2G044627_T01; parent_gene=GRMZM2G044627	GRMZM2G044627_P01	TRUE	TRUE	gGVLFMPGVPGVVER	95%	n+304 (+304)	39.05	26.00
3147	seq=translation; coord=4:155907661..155908892:-1; parent_transcript=GRMZM2G044627_T01; parent_gene=GRMZM2G044627	GRMZM2G044627_P01	TRUE	TRUE	qcLIFDGPGAGAR	95%	n+304 (+304), Carbamidomethyl (+57)	40.96	25.00
3148	seq=translation; coord=4:155907661..155908892:-1; parent_transcript=GRMZM2G044627_T01; parent_gene=GRMZM2G044627	GRMZM2G044627_P01	TRUE	TRUE	tIHFWQVDR	95%	n+304 (+304)	32.82	25.00
3149	seq=translation; coord=3:132396184..132400373:-1; parent_transcript=GRMZM2G063949_T01; parent_gene=GRMZM2G063949	GRMZM2G063949_P01,GRMZM2G167872_P01, GRMZM2G167872_P02	TRUE	TRUE	dAIEAVVLMIENPAR	95%	n+304 (+304)	45.16	25.39
3150	seq=translation; coord=3:132396184..132400373:-1; parent_transcript=GRMZM2G063949_T01; parent_gene=GRMZM2G063949	GRMZM2G063949_P01,GRMZM2G167872_P01, GRMZM2G167872_P02	TRUE	TRUE	mDFIPGVDGPSEGVPR	95%	n+304 (+304)	48.68	25.00
3151	seq=translation; coord=6:162010397..162015714:1; parent_transcript=GRMZM2G079263_T01; parent_gene=GRMZM2G079263	GRMZM2G079263_P01,GRMZM2G079263_P02, GRMZM2G079263_P03	TRUE	TRUE	gTSATPQAISIAcSDAVPcR	95%	n+304 (+304), Carbamidomethyl (+57), Carbamidomethyl (+57)	73.77	25.00
3152	seq=translation; coord=6:162010397..162015714:1; parent_transcript=GRMZM2G079263_T01; parent_gene=GRMZM2G079263	GRMZM2G079263_P01,GRMZM2G079263_P02, GRMZM2G079263_P03	TRUE	TRUE	sAGTVVPPScLAR	95%	n+304 (+304), Carbamidomethyl (+57)	41.86	26.13
3153	seq=translation; coord=6:162010397..162015714:1; parent_transcript=GRMZM2G079263_T01; parent_gene=GRMZM2G079263	GRMZM2G079263_P01,GRMZM2G079263_P02, GRMZM2G079263_P03	TRUE	TRUE	sWQGGTGFAR	90%	n+304 (+304)	27.53	25.00
3154	seq=translation; coord=6:162010397..162015714:1; parent_transcript=GRMZM2G079263_T01; parent_gene=GRMZM2G079263	GRMZM2G079263_P01,GRMZM2G079263_P02, GRMZM2G079263_P03	TRUE	TRUE	tTDMVEDVk	95%	n+304 (+304), K+304 (+304)	35.21	25.99

3155	seq=translation; coord=6:162010397..162015714:1; parent_transcript=GRMZM2G079263_T01; parent_gene=GRMZM2G079263	GRMZM2G079263_P01,GRMZM2G079263_P02, GRMZM2G079263_P03	TRUE	TRUE	vYQIWPVQLSGPck	95%	n+304 (+304), Carbamidomethyl (+57), K+304 (+304)	51.28	25.79
3156	seq=translation; coord=5:142403718..142405631:1; parent_transcript=GRMZM2G412436_T01; parent_gene=GRMZM2G412436	GRMZM2G412436_P01	TRUE	TRUE	aAGATADYASAk	95%	n+304 (+304), K+304 (+304)	52.92	25.59
3157	seq=translation; coord=5:142403718..142405631:1; parent_transcript=GRMZM2G412436_T01; parent_gene=GRMZM2G412436	GRMZM2G412436_P01	TRUE	TRUE	aEDVTWR	90%	n+304 (+304)	26.26	25.00
3158	seq=translation; coord=5:142403718..142405631:1; parent_transcript=GRMZM2G412436_T01; parent_gene=GRMZM2G412436	GRMZM2G412436_P01	TRUE	TRUE	dLTAGAAGTTMEYAk	95%	n+304 (+304), K+304 (+304)	58.24	25.31
3159	seq=translation; coord=5:142403718..142405631:1; parent_transcript=GRMZM2G412436_T01; parent_gene=GRMZM2G412436	GRMZM2G412436_P01	TRUE	TRUE	dTAWDAAGGMAQR	95%	n+304 (+304)	41.59	25.00
3160	seq=translation; coord=5:142403718..142405631:1; parent_transcript=GRMZM2G412436_T01; parent_gene=GRMZM2G412436	GRMZM2G412436_P01	TRUE	TRUE	dVTLATGETAAEYAk	95%	n+304 (+304), K+304 (+304)	28.84	25.97
3161	seq=translation; coord=5:142403718..142405631:1; parent_transcript=GRMZM2G412436_T01; parent_gene=GRMZM2G412436	GRMZM2G412436_P01	TRUE	TRUE	dVTLSTGETAAEYAk	95%	n+304 (+304), K+304 (+304)	72.84	25.04
3162	seq=translation; coord=5:142403718..142405631:1; parent_transcript=GRMZM2G412436_T01; parent_gene=GRMZM2G412436	GRMZM2G412436_P01	TRUE	TRUE	eTGAAAGQGVk	95%	n+304 (+304), K+304 (+304)	67.10	26.12
3163	seq=translation; coord=5:142403718..142405631:1; parent_transcript=GRMZM2G412436_T01; parent_gene=GRMZM2G412436	GRMZM2G412436_P01	TRUE	TRUE	gGGDENTTVVGDVLEAVGATVVGLAk	95%	n+304 (+304), K+304 (+304)	39.80	25.00
3164	seq=translation; coord=5:142403718..142405631:1; parent_transcript=GRMZM2G412436_T01; parent_gene=GRMZM2G412436	GRMZM2G412436_P01	TRUE	TRUE	gIVAGEEELVPVGGEk	95%	n+304 (+304), K+304 (+304)	71.18	25.00
3165	seq=translation; coord=3:47448537..47463984:-1; parent_transcript=GRMZM2G011101_T01; parent_gene=GRMZM2G011101	GRMZM2G011101_P01,GRMZM2G378906_P01	TRUE	TRUE	aLPEMILIcQNLR	95%	n+304 (+304), Carbamidomethyl (+57)	45.76	25.98
3166	seq=translation; coord=3:47448537..47463984:-1; parent_transcript=GRMZM2G011101_T01; parent_gene=GRMZM2G011101	GRMZM2G011101_P01,GRMZM2G378906_P01	TRUE	TRUE	dVMVGVVMDVLR	90%	n+304 (+304)	27.33	25.00
3167	seq=translation; coord=3:47448537..47463984:-1; parent_transcript=GRMZM2G011101_T01; parent_gene=GRMZM2G011101	GRMZM2G011101_P01,GRMZM2G378906_P01	TRUE	TRUE	kVLDLVLDLLTPR	95%	K+304 (+304), n+304 (+304)	61.99	25.00
3168	seq=translation; coord=3:47448537..47463984:-1; parent_transcript=GRMZM2G011101_T01; parent_gene=GRMZM2G011101	GRMZM2G011101_P01,GRMZM2G378906_P01	TRUE	TRUE	lPHGDQLIPDAPELVER	95%	n+304 (+304)	42.78	25.63
3169	seq=translation; coord=3:47448537..47463984:-1; parent_transcript=GRMZM2G011101_T01; parent_gene=GRMZM2G011101	GRMZM2G011101_P01,GRMZM2G378906_P01	TRUE	TRUE	lSEPEVLEPLVPSVLANLEHR	95%	n+304 (+304)	67.48	25.00
3170	seq=translation; coord=9:143165107..143170341:1; parent_transcript=GRMZM2G147671_T01; parent_gene=GRMZM2G147671	GRMZM2G147671_P01,GRMZM2G147671_P02, GRMZM2G165926_P01,GRMZM2G165926_P02	TRUE	TRUE	dLLASLHSQGEQEk	95%	n+304 (+304), K+304 (+304)	28.54	25.20
3171	seq=translation; coord=9:143165107..143170341:1; parent_transcript=GRMZM2G147671_T01; parent_gene=GRMZM2G147671	GRMZM2G147671_P01,GRMZM2G147671_P02, GRMZM2G165926_P01,GRMZM2G165926_P02	TRUE	TRUE	fQAQADAVNLIcGAk	95%	n+304 (+304), Carbamidomethyl (+57), K+304 (+304)	44.20	26.54
3172	seq=translation; coord=9:143165107..143170341:1; parent_transcript=GRMZM2G147671_T01; parent_gene=GRMZM2G147671	GRMZM2G147671_P01,GRMZM2G147671_P02, GRMZM2G165926_P01,GRMZM2G165926_P02	TRUE	TRUE	iIAFIGSPVk	95%	n+304 (+304), K+304 (+304)	37.08	25.00

3173	seq=translation; coord=9:143165107..143170341:1; parent_transcript=GRMZM2G147671_T01; parent_gene=GRMZM2G147671	GRMZM2G147671_P01,GRMZM2G147671_P02, GRMZM2G165926_P01,GRMZM2G165926_P02	TRUE	TRUE	lSMEEER	86%	n+304 (+304)	25.09	25.00
3174	seq=translation; coord=9:143165107..143170341:1; parent_transcript=GRMZM2G147671_T01; parent_gene=GRMZM2G147671	GRMZM2G147671_P01,GRMZM2G147671_P02, GRMZM2G165926_P01,GRMZM2G165926_P02	TRUE	TRUE	tQSNPENTVGVMTMAGk	93%	n+304 (+304), K+304 (+304)	27.56	25.00
3175	seq=translation; coord=9:143165107..143170341:1; parent_transcript=GRMZM2G147671_T01; parent_gene=GRMZM2G147671	GRMZM2G147671_P01,GRMZM2G147671_P02, GRMZM2G165926_P01,GRMZM2G165926_P02	TRUE	TRUE	vLEATMIcIDNSEWMR	95%	n+304 (+304), Carbamidomethyl (+57)	29.04	25.00
3176	seq=translation; coord=9:143165107..143170341:1; parent_transcript=GRMZM2G147671_T01; parent_gene=GRMZM2G147671	GRMZM2G147671_P01,GRMZM2G147671_P02, GRMZM2G165926_P01,GRMZM2G165926_P02	TRUE	TRUE	vLVTPTSDLGk	95%	n+304 (+304), K+304 (+304)	36.64	25.11
3177	seq=translation; coord=1:202752380..202764238:1; parent_transcript=GRMZM2G389233_T03; parent_gene=GRMZM2G389233	GRMZM2G389233_P03	TRUE	TRUE	aITLPGQGEIMDMMEVADPDAVHAVR	95%	n+304 (+304)	84.75	25.00
3178	seq=translation; coord=1:202752380..202764238:1; parent_transcript=GRMZM2G389233_T03; parent_gene=GRMZM2G389233	GRMZM2G389233_P03	TRUE	TRUE	dDALLDFYNk	95%	n+304 (+304), K+304 (+304)	37.82	25.00
3179	seq=translation; coord=1:202752380..202764238:1; parent_transcript=GRMZM2G389233_T03; parent_gene=GRMZM2G389233	GRMZM2G389233_P03	TRUE	TRUE	dQEFSSDLGcR	89%	n+304 (+304), Carbamidomethyl (+57)	26.10	25.00
3180	seq=translation; coord=1:202752380..202764238:1; parent_transcript=GRMZM2G389233_T03; parent_gene=GRMZM2G389233	GRMZM2G389233_P03	TRUE	TRUE	dWFQLTLk	93%	n+304 (+304), K+304 (+304)	27.78	25.71
3181	seq=translation; coord=1:202752380..202764238:1; parent_transcript=GRMZM2G389233_T03; parent_gene=GRMZM2G389233	GRMZM2G389233_P03	TRUE	TRUE	eLAVQLk	92%	n+304 (+304), K+304 (+304)	29.47	26.73
3182	seq=translation; coord=1:202752380..202764238:1; parent_transcript=GRMZM2G389233_T03; parent_gene=GRMZM2G389233	GRMZM2G389233_P03	TRUE	TRUE	eLAVQLkDDLLAAVk	95%	n+304 (+304), K+304 (+304), K+304 (+304)	42.84	25.00
3183	seq=translation; coord=1:202752380..202764238:1; parent_transcript=GRMZM2G389233_T03; parent_gene=GRMZM2G389233	GRMZM2G389233_P03	TRUE	TRUE	fLGEIVLQLDk	95%	n+304 (+304), K+304 (+304)	39.57	25.00
3184	seq=translation; coord=1:202752380..202764238:1; parent_transcript=GRMZM2G389233_T03; parent_gene=GRMZM2G389233	GRMZM2G389233_P03	TRUE	TRUE	iWTPAQDLPk	93%	n+304 (+304), K+304 (+304)	26.81	25.00
3185	seq=translation; coord=1:202752380..202764238:1; parent_transcript=GRMZM2G389233_T03; parent_gene=GRMZM2G389233	GRMZM2G389233_P03	TRUE	TRUE	kPSYLFALVAGQLGcR	95%	K+304 (+304), n+304 (+304), Carbamidomethyl (+57)	29.75	25.09
3186	seq=translation; coord=1:202752380..202764238:1; parent_transcript=GRMZM2G389233_T03; parent_gene=GRMZM2G389233	GRMZM2G389233_P03	TRUE	TRUE	lLSHPAFDLR	95%	n+304 (+304)	44.51	25.01
3187	seq=translation; coord=1:202752380..202764238:1; parent_transcript=GRMZM2G389233_T03; parent_gene=GRMZM2G389233	GRMZM2G389233_P03	TRUE	TRUE	lVLASPETATDGDYAAILGVVGHEYFHNWTGNR	95%	n+304 (+304)	47.29	25.00
3188	seq=translation; coord=1:202752380..202764238:1; parent_transcript=GRMZM2G389233_T03; parent_gene=GRMZM2G389233	GRMZM2G389233_P03	TRUE	TRUE	tLVLNPk	89%	n+304 (+304), K+304 (+304)	27.09	26.15
3189	seq=translation; coord=1:202752380..202764238:1; parent_transcript=GRMZM2G389233_T03; parent_gene=GRMZM2G389233	GRMZM2G389233_P03	TRUE	TRUE	wFALQATSEIPGNVANVQk	95%	n+304 (+304), K+304 (+304)	45.01	25.47
3190	seq=translation; coord=1:290265297..290286811:1; parent_transcript=GRMZM2G082664_T01; parent_gene=GRMZM2G082664	GRMZM2G082664_P01	TRUE	TRUE	dDTYEDIPNSQIR	95%	n+304 (+304)	35.95	25.00

3191	seq=translation; coord=1:290265297..290286811:1; parent_transcript=GRMZM2G082664_T01; parent_gene=GRMZM2G082664	GRMZM2G082664_P01	TRUE	TRUE	dVQVGQPIAVTVEDVEDIk	95%	n+304 (+304), K+304 (+304)	29.57	25.48
3192	seq=translation; coord=1:290265297..290286811:1; parent_transcript=GRMZM2G082664_T01; parent_gene=GRMZM2G082664	GRMZM2G082664_P01	TRUE	TRUE	dVVLDPLLAFR	95%	n+304 (+304)	38.87	25.16
3193	seq=translation; coord=1:290265297..290286811:1; parent_transcript=GRMZM2G082664_T01; parent_gene=GRMZM2G082664	GRMZM2G082664_P01	TRUE	TRUE	eEQSTESAPQNk	95%	n+304 (+304), K+304 (+304)	32.50	25.00
3194	seq=translation; coord=1:290265297..290286811:1; parent_transcript=GRMZM2G082664_T01; parent_gene=GRMZM2G082664	GRMZM2G082664_P01	TRUE	TRUE	gDVLAALk	95%	n+304 (+304), K+304 (+304)	33.19	26.56
3195	seq=translation; coord=1:290265297..290286811:1; parent_transcript=GRMZM2G082664_T01; parent_gene=GRMZM2G082664	GRMZM2G082664_P01	TRUE	TRUE	vVNVSEQSSTVSR	95%	n+304 (+304)	45.90	25.00
3196	seq=translation; coord=5:10855539..10861668:-1; parent_transcript=GRMZM2G109383_T01; parent_gene=GRMZM2G109383	GRMZM2G109383_P01	TRUE	TRUE	aTGAFILTASHNPGGPk	95%	n+304 (+304), K+304 (+304)	46.92	25.26
3197	seq=translation; coord=5:10855539..10861668:-1; parent_transcript=GRMZM2G109383_T01; parent_gene=GRMZM2G109383	GRMZM2G109383_P01	TRUE	TRUE	dSQDALAPLVDVALk	95%	n+304 (+304), K+304 (+304)	42.25	25.38
3198	seq=translation; coord=5:10855539..10861668:-1; parent_transcript=GRMZM2G109383_T01; parent_gene=GRMZM2G109383	GRMZM2G109383_P01	TRUE	TRUE	vTVFQQPHYLQNFVQSTFNALPVDQVR	95%	n+304 (+304)	41.22	25.13
3199	seq=translation; coord=5:10855539..10861668:-1; parent_transcript=GRMZM2G109383_T01; parent_gene=GRMZM2G109383	GRMZM2G109383_P01	TRUE	TRUE	vYIEQYER	94%	n+304 (+304)	29.07	25.00
3200	seq=translation; coord=2:160150779..160152270:1; parent_transcript=GRMZM2G304548_T01; parent_gene=GRMZM2G304548	GRMZM2G304548_P01	TRUE	TRUE	eLADIPAYcR	95%	n+304 (+304), Carbamidomethyl (+57)	39.56	25.00
3201	seq=translation; coord=2:160150779..160152270:1; parent_transcript=GRMZM2G304548_T01; parent_gene=GRMZM2G304548	GRMZM2G304548_P01	TRUE	TRUE	eLADIPAycR	89%	n+304 (+304), iTRAQ8plex (+304), Carbamidomethyl (+57)	27.20	26.03
3202	seq=translation; coord=2:160150779..160152270:1; parent_transcript=GRMZM2G304548_T01; parent_gene=GRMZM2G304548	GRMZM2G304548_P01	TRUE	TRUE	lEDLPGcPR	89%	n+304 (+304), Carbamidomethyl (+57)	26.20	25.00
3203	seq=translation; coord=2:160150779..160152270:1; parent_transcript=GRMZM2G304548_T01; parent_gene=GRMZM2G304548	GRMZM2G304548_P01	TRUE	TRUE	lPWPELk	87%	n+304 (+304), K+304 (+304)	25.25	25.00
3204	seq=translation; coord=1:192404194..192408778:-1; parent_transcript=GRMZM2G096806_T01; parent_gene=GRMZM2G096806	GRMZM2G096806_P01	TRUE	TRUE	dGSGQQDWNSPAR	95%	n+304 (+304)	57.70	25.00
3205	seq=translation; coord=1:192404194..192408778:-1; parent_transcript=GRMZM2G096806_T01; parent_gene=GRMZM2G096806	GRMZM2G096806_P01	TRUE	TRUE	eLAVDPTSSIGTMGNIGNAEDk	95%	n+304 (+304), K+304 (+304)	28.88	25.00
3206	seq=translation; coord=1:192404194..192408778:-1; parent_transcript=GRMZM2G096806_T01; parent_gene=GRMZM2G096806	GRMZM2G096806_P01	TRUE	TRUE	lASEEIIFTSNR	95%	n+304 (+304)	31.08	25.17
3207	seq=translation; coord=1:192404194..192408778:-1; parent_transcript=GRMZM2G096806_T01; parent_gene=GRMZM2G096806	GRMZM2G096806_P01	TRUE	TRUE	mcEETMSGPTLDk	94%	n+304 (+304), Carbamidomethyl (+57), K+304 (+304)	27.69	25.00
3208	seq=translation; coord=1:192404194..192408778:-1; parent_transcript=GRMZM2G096806_T01; parent_gene=GRMZM2G096806	GRMZM2G096806_P01	TRUE	TRUE	mHDEEVNEDLDSR	95%	n+304 (+304)	43.66	25.00

3209	seq=translation; coord=1:192404194..192408778:-1; parent_transcript=GRMZM2G096806_T01; parent_gene=GRMZM2G096806	GRMZM2G096806_P01	TRUE	TRUE	qNENTNVMEEER	95%	n+304 (+304)	65.54	25.00
3210	seq=translation; coord=1:192404194..192408778:-1; parent_transcript=GRMZM2G096806_T01; parent_gene=GRMZM2G096806	GRMZM2G096806_P01	TRUE	TRUE	sSADLASYEVk	95%	n+304 (+304), K+304 (+304)	44.50	25.54
3211	seq=translation; coord=1:192404194..192408778:-1; parent_transcript=GRMZM2G096806_T01; parent_gene=GRMZM2G096806	GRMZM2G096806_P01	TRUE	TRUE	sVENTTVEGk	95%	n+304 (+304), K+304 (+304)	29.44	26.21
3212	seq=translation; coord=1:192404194..192408778:-1; parent_transcript=GRMZM2G096806_T01; parent_gene=GRMZM2G096806	GRMZM2G096806_P01	TRUE	TRUE	sVGTGDDLLQSGk	95%	n+304 (+304), K+304 (+304)	62.06	26.11
3213	seq=translation; coord=1:192404194..192408778:-1; parent_transcript=GRMZM2G096806_T01; parent_gene=GRMZM2G096806	GRMZM2G096806_P01	TRUE	TRUE	tDDGTLTTVATQYSEk	93%	n+304 (+304), K+304 (+304)	26.37	25.00
3214	seq=translation; coord=1:192404194..192408778:-1; parent_transcript=GRMZM2G096806_T01; parent_gene=GRMZM2G096806	GRMZM2G096806_P01	TRUE	TRUE	vLLADk	94%	n+304 (+304), K+304 (+304)	29.33	26.88
3215	seq=translation; coord=1:192404194..192408778:-1; parent_transcript=GRMZM2G096806_T01; parent_gene=GRMZM2G096806	GRMZM2G096806_P01	TRUE	TRUE	vTVEDTTSR	95%	n+304 (+304)	32.84	25.00
3216	seq=translation; coord=2:139185152..139187472:-1; parent_transcript=GRMZM2G047292_T01; parent_gene=GRMZM2G047292	GRMZM2G047292_P01	TRUE	TRUE	aPPNVNLRPAGAGVADGGAASGAAFLGAR	95%	n+304 (+304)	31.21	25.00
3217	seq=translation; coord=2:139185152..139187472:-1; parent_transcript=GRMZM2G047292_T01; parent_gene=GRMZM2G047292	GRMZM2G047292_P01	TRUE	TRUE	aVNPSGIEEGLQSLR	95%	n+304 (+304)	59.38	26.29
3218	seq=translation; coord=2:139185152..139187472:-1; parent_transcript=GRMZM2G047292_T01; parent_gene=GRMZM2G047292	GRMZM2G047292_P01	TRUE	TRUE	fLIIDDGWQETVDEIk	95%	n+304 (+304), K+304 (+304)	51.36	26.01
3219	seq=translation; coord=2:139185152..139187472:-1; parent_transcript=GRMZM2G047292_T01; parent_gene=GRMZM2G047292	GRMZM2G047292_P01	TRUE	TRUE	gSLYALVLPVLDGGFR	95%	n+304 (+304)	35.00	26.45
3220	seq=translation; coord=2:139185152..139187472:-1; parent_transcript=GRMZM2G047292_T01; parent_gene=GRMZM2G047292	GRMZM2G047292_P01	TRUE	TRUE	vDVQNVLETLGR	95%	n+304 (+304)	40.33	26.57
3221	seq=translation; coord=5:47578824..47582423:1; parent_transcript=GRMZM2G085967_T01; parent_gene=GRMZM2G085967	GRMZM2G085967_P01,GRMZM2G085967_P02	TRUE	TRUE	aHIPHAPDVASTLLR	95%	n+304 (+304)	49.44	26.10
3222	seq=translation; coord=5:47578824..47582423:1; parent_transcript=GRMZM2G085967_T01; parent_gene=GRMZM2G085967	GRMZM2G085967_P01,GRMZM2G085967_P02	TRUE	TRUE	dSVGVIGGPFWSVPTGR	95%	n+304 (+304)	72.27	25.95
3223	seq=translation; coord=5:47578824..47582423:1; parent_transcript=GRMZM2G085967_T01; parent_gene=GRMZM2G085967	GRMZM2G085967_P01,GRMZM2G085967_P02	TRUE	TRUE	eGFYDYScPQAEk	95%	n+304 (+304), Carbamidomethyl (+57), K+304 (+304)	33.54	25.00
3224	seq=translation; coord=5:47578824..47582423:1; parent_transcript=GRMZM2G085967_T01; parent_gene=GRMZM2G085967	GRMZM2G085967_P01,GRMZM2G085967_P02	TRUE	TRUE	gGPDDADPSLDPLYAAk	94%	n+304 (+304), K+304 (+304)	27.96	25.08
3225	seq=translation; coord=5:47578824..47582423:1; parent_transcript=GRMZM2G085967_T01; parent_gene=GRMZM2G085967	GRMZM2G085967_P01,GRMZM2G085967_P02	TRUE	TRUE	gLFQSDAALITDAASk	95%	n+304 (+304), K+304 (+304)	60.18	25.59
3226	seq=translation; coord=10:4165442..4166593:-1; parent_transcript=GRMZM2G101958_T01; parent_gene=GRMZM2G101958	GRMZM2G101958_P01	TRUE	TRUE	nAAAGVSGLNAGNAASIPSk	95%	n+304 (+304), K+304 (+304)	99.23	25.66

3227	seq=translation; coord=6:1338452..1339388:-1; parent_transcript=GRMZM2G122357_T01; parent_gene=GRMZM2G122357	GRMZM2G122357_P01	TRUE	TRUE	iADSQPVDLFAAAR	95%	n+304 (+304)	64.33	25.39
3228	seq=translation; coord=6:1338452..1339388:-1; parent_transcript=GRMZM2G122357_T01; parent_gene=GRMZM2G122357	GRMZM2G122357_P01	TRUE	TRUE	nAVIAESEPVDLPASAR	95%	n+304 (+304)	81.31	25.19
3229	seq=translation; coord=6:1338452..1339388:-1; parent_transcript=GRMZM2G122357_T01; parent_gene=GRMZM2G122357	GRMZM2G122357_P01	TRUE	TRUE	qGMAEPTAGGR	95%	n+304 (+304)	32.46	25.00
3230	seq=translation; coord=6:1338452..1339388:-1; parent_transcript=GRMZM2G122357_T01; parent_gene=GRMZM2G122357	GRMZM2G122357_P01	TRUE	TRUE	vAGDDEMLR	95%	n+304 (+304)	44.02	25.00
3231	seq=translation; coord=1:25381004..25387870:-1; parent_transcript=GRMZM2G069765_T01; parent_gene=GRMZM2G069765	GRMZM2G069765_P01	TRUE	TRUE	acGAVIVNRPEELQESDVGTR	94%	n+304 (+304), Carbamidomethyl (+57)	28.00	25.00
3232	seq=translation; coord=1:25381004..25387870:-1; parent_transcript=GRMZM2G069765_T01; parent_gene=GRMZM2G069765	GRMZM2G069765_P01	TRUE	TRUE	aLDDAIAVLDk	95%	n+304 (+304), K+304 (+304)	35.97	26.46
3233	seq=translation; coord=1:25381004..25387870:-1; parent_transcript=GRMZM2G069765_T01; parent_gene=GRMZM2G069765	GRMZM2G069765_P01	TRUE	TRUE	gLSDLAIHYLSk	95%	n+304 (+304), K+304 (+304)	32.29	25.98
3234	seq=translation; coord=1:25381004..25387870:-1; parent_transcript=GRMZM2G069765_T01; parent_gene=GRMZM2G069765	GRMZM2G069765_P01	TRUE	TRUE	iDDIVSGIk	94%	n+304 (+304), K+304 (+304)	27.20	25.00
3235	seq=translation; coord=1:25381004..25387870:-1; parent_transcript=GRMZM2G069765_T01; parent_gene=GRMZM2G069765	GRMZM2G069765_P01	TRUE	TRUE	kIGDEFFSFIVDck	95%	K+304 (+304), n+304 (+304), Carbamidomethyl (+57), K+304 (+304)	33.22	25.00
3236	seq=translation; coord=1:25381004..25387870:-1; parent_transcript=GRMZM2G069765_T01; parent_gene=GRMZM2G069765	GRMZM2G069765_P01	TRUE	TRUE	nLQDAMSVAR	86%	n+304 (+304)	25.06	25.00
3237	seq=translation; coord=1:25381004..25387870:-1; parent_transcript=GRMZM2G069765_T01; parent_gene=GRMZM2G069765	GRMZM2G069765_P01	TRUE	TRUE	wPYEAAALAFEAIPR	95%	n+304 (+304)	75.58	25.79
3238	seq=translation; coord=4:125089612..125098586:-1; parent_transcript=GRMZM2G157019_T01; parent_gene=GRMZM2G157019	GRMZM2G157019_P01	TRUE	TRUE	tYIFADDGTTTIk	95%	n+304 (+304), K+304 (+304)	46.85	25.43
3239	seq=translation; coord=5:186082222..186092865:1; parent_transcript=GRMZM2G064695_T04; parent_gene=GRMZM2G064695	GRMZM2G064695_P04	TRUE	TRUE	aILVNIFGGIMk	95%	n+304 (+304), K+304 (+304)	53.88	25.00
3240	seq=translation; coord=5:186082222..186092865:1; parent_transcript=GRMZM2G064695_T04; parent_gene=GRMZM2G064695	GRMZM2G064695_P04	TRUE	TRUE	aILVNIFGGImk	95%	n+304 (+304), Oxidation (+16), K+304 (+304)	42.05	25.00
3241	seq=translation; coord=5:186082222..186092865:1; parent_transcript=GRMZM2G064695_T04; parent_gene=GRMZM2G064695	GRMZM2G064695_P04	TRUE	TRUE	gITDEDAAk	94%	n+304 (+304), K+304 (+304)	28.14	25.00
3242	seq=translation; coord=5:186082222..186092865:1; parent_transcript=GRMZM2G064695_T04; parent_gene=GRMZM2G064695	GRMZM2G064695_P04	TRUE	TRUE	lHGGTPANFLDVGGSASEGQVVEAFk	95%	n+304 (+304), K+304 (+304)	69.73	25.00
3243	seq=translation; coord=5:186082222..186092865:1; parent_transcript=GRMZM2G064695_T04; parent_gene=GRMZM2G064695	GRMZM2G064695_P04	TRUE	TRUE	lNFDDNAAFR	95%	n+304 (+304)	36.20	25.00
3244	seq=translation; coord=5:186082222..186092865:1; parent_transcript=GRMZM2G064695_T04; parent_gene=GRMZM2G064695	GRMZM2G064695_P04	TRUE	TRUE	lNIHEYQGAELMGk	95%	n+304 (+304), K+304 (+304)	52.83	25.72

3245	seq=translation; coord=3:3887574..3888713:1; parent_transcript=GRMZM2G093405_T01; parent_gene=GRMZM2G093405	GRMZM2G093405_P01,GRMZM2G093405_P02	TRUE	TRUE	fGGFVADADVGEQAAR	95%	n+304 (+304)	68.85	25.00
3246	seq=translation; coord=3:3887574..3888713:1; parent_transcript=GRMZM2G093405_T01; parent_gene=GRMZM2G093405	GRMZM2G093405_P01,GRMZM2G093405_P02	TRUE	TRUE	rADPASPYTVAQYNSPFEFTGR	95%	n+304 (+304)	66.03	25.00
3247	seq=translation; coord=3:3887574..3888713:1; parent_transcript=GRMZM2G093405_T01; parent_gene=GRMZM2G093405	GRMZM2G093405_P01,GRMZM2G093405_P02	TRUE	TRUE	rFGGFVADADVGEQAAR	95%	n+304 (+304)	57.53	25.00
3248	seq=translation; coord=3:3887574..3888713:1; parent_transcript=GRMZM2G093405_T01; parent_gene=GRMZM2G093405	GRMZM2G093405_P01,GRMZM2G093405_P02	TRUE	TRUE	tGFLQLFDYIQGk	95%	n+304 (+304), K+304 (+304)	51.40	25.51
3249	seq=translation; coord=5:157277567..157288178:1; parent_transcript=GRMZM2G124886_T01; parent_gene=GRMZM2G124886	GRMZM2G124886_P01,GRMZM2G124886_P02, GRMZM2G124886_P03,GRMZM2G124886_P04, GRMZM2G141587_P01,GRMZM2G141587_P02, GRMZM2G141587_P03,GRMZM2G141587_P04, GRMZM2G141587_P05	TRUE	TRUE	eLGScDLYPQSLR	95%	n+304 (+304), Carbamidomethyl (+57)	40.78	25.00
3250	seq=translation; coord=5:157277567..157288178:1; parent_transcript=GRMZM2G124886_T01; parent_gene=GRMZM2G124886	GRMZM2G124886_P01,GRMZM2G124886_P02, GRMZM2G124886_P03,GRMZM2G124886_P04, GRMZM2G141587_P01,GRMZM2G141587_P02, GRMZM2G141587_P03,GRMZM2G141587_P04, GRMZM2G141587_P05	TRUE	TRUE	gMLEEALEIATDTNYR	95%	n+304 (+304)	47.42	25.00
3251	seq=translation; coord=5:157277567..157288178:1; parent_transcript=GRMZM2G124886_T01; parent_gene=GRMZM2G124886	GRMZM2G124886_P01,GRMZM2G124886_P02, GRMZM2G124886_P03,GRMZM2G124886_P04, GRMZM2G141587_P01,GRMZM2G141587_P02, GRMZM2G141587_P03,GRMZM2G141587_P04, GRMZM2G141587_P05	TRUE	TRUE	gMLEEALEIATDTNyR	95%	n+304 (+304), iTRAQ8plex (+304)	36.50	25.75
3252	seq=translation; coord=5:157277567..157288178:1; parent_transcript=GRMZM2G124886_T01; parent_gene=GRMZM2G124886	GRMZM2G124886_P01,GRMZM2G124886_P02, GRMZM2G124886_P03,GRMZM2G124886_P04, GRMZM2G141587_P01,GRMZM2G141587_P02, GRMZM2G141587_P03,GRMZM2G141587_P04, GRMZM2G141587_P05	TRUE	TRUE	kQWVVAGADDMFIR	95%	K+304 (+304), n+304 (+304)	36.64	25.17
3253	seq=translation; coord=5:157277567..157288178:1; parent_transcript=GRMZM2G124886_T01; parent_gene=GRMZM2G124886	GRMZM2G124886_P01,GRMZM2G124886_P02, GRMZM2G124886_P03,GRMZM2G124886_P04, GRMZM2G141587_P01,GRMZM2G141587_P02, GRMZM2G141587_P03,GRMZM2G141587_P04, GRMZM2G141587_P05	TRUE	TRUE	sFEVTDLPVR	94%	n+304 (+304)	31.33	25.34
3254	seq=translation; coord=5:157277567..157288178:1; parent_transcript=GRMZM2G124886_T01; parent_gene=GRMZM2G124886	GRMZM2G124886_P01,GRMZM2G124886_P02, GRMZM2G124886_P03,GRMZM2G124886_P04, GRMZM2G141587_P01,GRMZM2G141587_P02, GRMZM2G141587_P03,GRMZM2G141587_P04, GRMZM2G141587_P05	TRUE	TRUE	vPEIVALWk	95%	n+304 (+304), K+304 (+304)	33.96	25.00
3255	seq=translation; coord=7:174582270..174585306:-1; parent_transcript=GRMZM2G056569_T01; parent_gene=GRMZM2G056569	GRMZM2G056569_P01	TRUE	TRUE	aIcTEAGLLALR	95%	n+304 (+304), Carbamidomethyl (+57)	55.76	25.99
3256	seq=translation; coord=7:174582270..174585306:-1; parent_transcript=GRMZM2G056569_T01; parent_gene=GRMZM2G056569	GRMZM2G056569_P01	TRUE	TRUE	lPNVAPLSk	92%	n+304 (+304), K+304 (+304)	28.95	25.00
3257	seq=translation; coord=7:174582270..174585306:-1; parent_transcript=GRMZM2G056569_T01; parent_gene=GRMZM2G056569	GRMZM2G056569_P01	TRUE	TRUE	mTLADDVNLEEFVMTk	95%	n+304 (+304), K+304 (+304)	48.89	25.00
3258	seq=translation; coord=7:174582270..174585306:-1; parent_transcript=GRMZM2G056569_T01; parent_gene=GRMZM2G056569	GRMZM2G056569_P01	TRUE	TRUE	tMLELLNQLDGFDSR	95%	n+304 (+304)	34.75	25.00

3259	seq=translation; coord=7:174582270..174585306:-1; parent_transcript=GRMZM2G056569_T01; parent_gene=GRMZM2G056569	GRMZM2G056569_P01	TRUE	TRUE	vLSVVGILQDEVDPMVSVMk	95%	n+304 (+304), K+304 (+304)	30.81	25.00
3260	seq=translation; coord=8:116533013..116535098:1; parent_transcript=GRMZM2G009936_T01; parent_gene=GRMZM2G009936	GRMZM2G009936_P01,GRMZM2G009936_P02, GRMZM2G009936_P03	TRUE	TRUE	eVAGFAPYEk	91%	n+304 (+304), K+304 (+304)	30.01	26.88
3261	seq=translation; coord=8:116533013..116535098:1; parent_transcript=GRMZM2G009936_T01; parent_gene=GRMZM2G009936	GRMZM2G009936_P01,GRMZM2G009936_P02, GRMZM2G009936_P03	TRUE	TRUE	sGLFVGINk	94%	n+304 (+304), K+304 (+304)	28.13	25.00
3262	seq=translation; coord=1:62530194..62532719:1; parent_transcript=GRMZM2G577677_T01; parent_gene=GRMZM2G577677	GRMZM2G577677_P01	TRUE	TRUE	fYGDGVYNcAGcGTPLYk	95%	n+304 (+304), Carbamidomethyl (+57), Carbamidomethyl (+57), K+304 (+304)	48.32	25.00
3263	seq=translation; coord=1:62530194..62532719:1; parent_transcript=GRMZM2G577677_T01; parent_gene=GRMZM2G577677	GRMZM2G577677_P01	TRUE	TRUE	gTELPGTGEYNk	95%	n+304 (+304), K+304 (+304)	51.07	25.83
3264	seq=translation; coord=1:62530194..62532719:1; parent_transcript=GRMZM2G577677_T01; parent_gene=GRMZM2G577677	GRMZM2G577677_P01	TRUE	TRUE	vVLSPEQFR	95%	n+304 (+304)	43.83	25.44
3265	seq=translation; coord=9:106627644..106628551:-1; parent_transcript=GRMZM2G361699_T01; parent_gene=GRMZM2G361699	GRMZM2G361699_P01	TRUE	TRUE	dGSVEALLSGDPAk	95%	n+304 (+304), K+304 (+304)	29.83	25.69
3266	seq=translation; coord=9:106627644..106628551:-1; parent_transcript=GRMZM2G361699_T01; parent_gene=GRMZM2G361699	GRMZM2G361699_P01	TRUE	TRUE	dWTASTAESLGLAGWVR	95%	n+304 (+304)	61.59	25.00
3267	seq=translation; coord=9:106627644..106628551:-1; parent_transcript=GRMZM2G361699_T01; parent_gene=GRMZM2G361699	GRMZM2G361699_P01	TRUE	TRUE	iEDMITR	87%	n+304 (+304)	25.61	25.37
3268	seq=translation; coord=9:106627644..106628551:-1; parent_transcript=GRMZM2G361699_T01; parent_gene=GRMZM2G361699	GRMZM2G361699_P01	TRUE	TRUE	vTGVGFR	91%	n+304 (+304)	26.82	25.00
3269	seq=translation; coord=7:116376688..116380988:1; parent_transcript=GRMZM2G116689_T01; parent_gene=GRMZM2G116689	GRMZM2G116689_P01,GRMZM2G116689_P03	TRUE	TRUE	eIEIDIEPTDSIDR	95%	n+304 (+304)	45.70	25.00
3270	seq=translation; coord=7:116376688..116380988:1; parent_transcript=GRMZM2G116689_T01; parent_gene=GRMZM2G116689	GRMZM2G116689_P01,GRMZM2G116689_P03	TRUE	TRUE	tITLEVESSDTVDNVk	95%	n+304 (+304), K+304 (+304)	32.18	25.51
3271	seq=translation; coord=8:4232302..4235855:1; parent_transcript=GRMZM2G015361_T01; parent_gene=GRMZM2G015361	GRMZM2G015361_P01,GRMZM2G015361_P03, GRMZM2G015361_P05,GRMZM2G015361_P06, GRMZM2G015361_P07,GRMZM2G105996_P01, GRMZM2G157596_P01,GRMZM2G157596_P02, GRMZM2G157596_P03,GRMZM2G157596_P04, GRMZM2G157596_P05,GRMZM2G357399_P01, GRMZM2G357399_P02,GRMZM2G395844_P01, GRMZM2G395844_P02,GRMZM2G395844_P03, GRMZM2G395844_P04,GRMZM5G836182_P01	TRUE	TRUE	dAVLLVFANk	95%	n+304 (+304), K+304 (+304)	49.25	25.00

3272	seq=translation; coord=8:4232302..4235855:1; parent_transcript=GRMZM2G015361_T01; parent_gene=GRMZM2G015361	GRMZM2G015361_P01,GRMZM2G015361_P03, GRMZM2G015361_P05,GRMZM2G015361_P06, GRMZM2G015361_P07,GRMZM2G105996_P01, GRMZM2G157596_P01,GRMZM2G157596_P02, GRMZM2G157596_P03,GRMZM2G157596_P04, GRMZM2G157596_P05,GRMZM2G357399_P01, GRMZM2G357399_P02,GRMZM2G395844_P01, GRMZM2G395844_P02,GRMZM2G395844_P03, GRMZM2G395844_P04,GRMZM5G836182_P01	TRUE	TRUE	iLMVGLDAAGk	95%	n+304 (+304), K+304 (+304)	52.87	25.20
3273	seq=translation; coord=8:4232302..4235855:1; parent_transcript=GRMZM2G015361_T01; parent_gene=GRMZM2G015361	GRMZM2G015361_P01,GRMZM2G015361_P03, GRMZM2G015361_P05,GRMZM2G015361_P06, GRMZM2G015361_P07,GRMZM2G105996_P01, GRMZM2G157596_P01,GRMZM2G157596_P02, GRMZM2G157596_P03,GRMZM2G157596_P04, GRMZM2G157596_P05,GRMZM2G357399_P01, GRMZM2G357399_P02,GRMZM2G395844_P01, GRMZM2G395844_P02,GRMZM2G395844_P03, GRMZM2G395844_P04,GRMZM5G836182_P01	TRUE	TRUE	lGEIVTTIPTIGFNVETVEYk	95%	n+304 (+304), K+304 (+304)	36.61	25.00
3274	seq=translation; coord=8:4232302..4235855:1; parent_transcript=GRMZM2G015361_T01; parent_gene=GRMZM2G015361	GRMZM2G015361_P01,GRMZM2G015361_P03, GRMZM2G015361_P05,GRMZM2G015361_P06, GRMZM2G015361_P07,GRMZM2G105996_P01, GRMZM2G157596_P01,GRMZM2G157596_P02, GRMZM2G157596_P03,GRMZM2G157596_P04, GRMZM2G157596_P05,GRMZM2G357399_P01, GRMZM2G357399_P02,GRMZM2G395844_P01, GRMZM2G395844_P02,GRMZM2G395844_P03, GRMZM2G395844_P04,GRMZM5G836182_P01	TRUE	TRUE	mLNEDELR	91%	n+304 (+304)	28.24	25.00
3275	seq=translation; coord=8:4232302..4235855:1; parent_transcript=GRMZM2G015361_T01; parent_gene=GRMZM2G015361	GRMZM2G015361_P01,GRMZM2G015361_P03, GRMZM2G015361_P05,GRMZM2G015361_P06, GRMZM2G015361_P07,GRMZM2G105996_P01, GRMZM2G157596_P01,GRMZM2G157596_P02, GRMZM2G157596_P03,GRMZM2G157596_P04, GRMZM2G157596_P05,GRMZM2G357399_P01, GRMZM2G357399_P02,GRMZM2G395844_P01, GRMZM2G395844_P02,GRMZM2G395844_P03, GRMZM2G395844_P04,GRMZM5G836182_P01	TRUE	TRUE	nISFTVWDVGGQDk	95%	n+304 (+304), K+304 (+304)	36.61	25.59
3276	seq=translation; coord=3:228250718..228270546:-1; parent_transcript=GRMZM2G035620_T01; parent_gene=GRMZM2G035620	GRMZM2G035620_P01	TRUE	TRUE	aLIPDLYR	87%	n+304 (+304)	25.25	25.00
3277	seq=translation; coord=3:228250718..228270546:-1; parent_transcript=GRMZM2G035620_T01; parent_gene=GRMZM2G035620	GRMZM2G035620_P01	TRUE	TRUE	eDTTFDAYVVGk	95%	n+304 (+304), K+304 (+304)	29.19	25.00
3278	seq=translation; coord=3:228250718..228270546:-1; parent_transcript=GRMZM2G035620_T01; parent_gene=GRMZM2G035620	GRMZM2G035620_P01	TRUE	TRUE	gMGLTDENQEAVDLAWSR	95%	n+304 (+304)	79.62	25.00
3279	seq=translation; coord=3:228250718..228270546:-1; parent_transcript=GRMZM2G035620_T01; parent_gene=GRMZM2G035620	GRMZM2G035620_P01	TRUE	TRUE	vALDVAEAQHLMEGLDWQGAVk	95%	n+304 (+304), K+304 (+304)	64.22	25.00

3280	seq=translation; coord=3:228250718..228270546:-1; parent_transcript=GRMZM2G035620_T01; parent_gene=GRMZM2G035620	GRMZM2G035620_P01	TRUE	TRUE	vALDVAEAQHLmEGLDWQGAVk	95%	n+304 (+304), Oxidation (+16), K+304 (+304)	49.74	25.00
3281	seq=translation; coord=1:38637065..38637541:1; parent_transcript=AC208204.3_FGT006; parent_gene=AC208204.3_FG006	AC208204.3_FGP006,GRMZM2G046382_P01	TRUE	TRUE	tSSETAAFAGAR	95%	n+304 (+304)	33.38	25.00
3282	seq=translation; coord=1:38637065..38637541:1; parent_transcript=AC208204.3_FGT006; parent_gene=AC208204.3_FG006	AC208204.3_FGP006,GRMZM2G046382_P01	TRUE	TRUE	vEVEDGNVLQISGER	95%	n+304 (+304)	70.45	25.00
3283	seq=translation; coord=7:130426898..130428254:1; parent_transcript=GRMZM2G133053_T01; parent_gene=GRMZM2G133053	GRMZM2G133053_P01	TRUE	TRUE	gDVVTIATAASAPAR	95%	n+304 (+304)	71.91	26.21
3284	seq=translation; coord=3:168695539..168699190:1; parent_transcript=GRMZM2G156861_T02; parent_gene=GRMZM2G156861	GRMZM2G156861_P02	TRUE	TRUE	aWMSDEEFAR	95%	n+304 (+304)	35.98	25.00
3285	seq=translation; coord=3:168695539..168699190:1; parent_transcript=GRMZM2G156861_T02; parent_gene=GRMZM2G156861	GRMZM2G156861_P02	TRUE	TRUE	dTMNINALAR	86%	n+304 (+304)	25.25	25.00
3286	seq=translation; coord=3:168695539..168699190:1; parent_transcript=GRMZM2G156861_T02; parent_gene=GRMZM2G156861	GRMZM2G156861_P02	TRUE	TRUE	eTLAGVNPLIIR	95%	n+304 (+304)	33.33	25.00
3287	seq=translation; coord=3:168695539..168699190:1; parent_transcript=GRMZM2G156861_T02; parent_gene=GRMZM2G156861	GRMZM2G156861_P02	TRUE	TRUE	lPDIPALEEFR	94%	n+304 (+304)	31.41	25.59
3288	seq=translation; coord=4:193146957..193151153:1; parent_transcript=GRMZM5G821551_T02; parent_gene=GRMZM5G821551	GRMZM5G821551_P02,GRMZM5G821551_P03	TRUE	TRUE	fVDVADLEFLMFPR	95%	n+304 (+304)	37.39	25.07
3289	seq=translation; coord=4:193146957..193151153:1; parent_transcript=GRMZM5G821551_T02; parent_gene=GRMZM5G821551	GRMZM5G821551_P02,GRMZM5G821551_P03	TRUE	TRUE	iLAILDDLSAEELGAk	95%	n+304 (+304), K+304 (+304)	69.72	25.00
3290	seq=translation; coord=4:193146957..193151153:1; parent_transcript=GRMZM5G821551_T02; parent_gene=GRMZM5G821551	GRMZM5G821551_P02,GRMZM5G821551_P03	TRUE	TRUE	lFYIPSFk	93%	n+304 (+304), K+304 (+304)	27.49	26.11
3291	seq=translation; coord=4:193146957..193151153:1; parent_transcript=GRMZM5G821551_T02; parent_gene=GRMZM5G821551	GRMZM5G821551_P02,GRMZM5G821551_P03	TRUE	TRUE	mVLEALEAMSEk	95%	n+304 (+304), K+304 (+304)	34.93	25.56
3292	seq=translation; coord=4:193146957..193151153:1; parent_transcript=GRMZM5G821551_T02; parent_gene=GRMZM5G821551	GRMZM5G821551_P02,GRMZM5G821551_P03	TRUE	TRUE	sEEEQLNVFR	93%	n+304 (+304)	28.71	25.00
3293	seq=translation; coord=4:193146957..193151153:1; parent_transcript=GRMZM5G821551_T02; parent_gene=GRMZM5G821551	GRMZM5G821551_P02,GRMZM5G821551_P03	TRUE	TRUE	vYLFLTSLGIDk	90%	n+304 (+304), K+304 (+304)	25.41	25.00
3294	seq=translation; coord=1:221739264..221743951:-1; parent_transcript=GRMZM2G450163_T01; parent_gene=GRMZM2G450163	GRMZM2G450163_P01	TRUE	TRUE	aSSFLLDDFYR	95%	n+304 (+304)	40.10	25.00
3295	seq=translation; coord=1:221739264..221743951:-1; parent_transcript=GRMZM2G450163_T01; parent_gene=GRMZM2G450163	GRMZM2G450163_P01	TRUE	TRUE	aYNPQSLLYGFVGGTEGLFANk	95%	n+304 (+304), K+304 (+304)	35.88	25.37
3296	seq=translation; coord=1:221739264..221743951:-1; parent_transcript=GRMZM2G450163_T01; parent_gene=GRMZM2G450163	GRMZM2G450163_P01	TRUE	TRUE	gSTPSNFDcDYAYALGR	95%	n+304 (+304), Carbamidomethyl (+57)	57.92	25.00
3297	seq=translation; coord=1:221739264..221743951:-1; parent_transcript=GRMZM2G450163_T01; parent_gene=GRMZM2G450163	GRMZM2G450163_P01	TRUE	TRUE	lPPcLQGPTVR	88%	n+304 (+304), Carbamidomethyl (+57)	26.56	25.53

3298	seq=translation; coord=1:221739264..221743951:-1; parent_transcript=GRMZM2G450163_T01; parent_gene=GRMZM2G450163	GRMZM2G450163_P01	TRUE	TRUE	lTLMEIISk	95%	n+304 (+304), K+304 (+304)	32.93	25.00
3299	seq=translation; coord=1:221739264..221743951:-1; parent_transcript=GRMZM2G450163_T01; parent_gene=GRMZM2G450163	GRMZM2G450163_P01	TRUE	TRUE	tLEITDDVLASYk	95%	n+304 (+304), K+304 (+304)	63.46	25.50
3300	seq=translation; coord=1:221739264..221743951:-1; parent_transcript=GRMZM2G450163_T01; parent_gene=GRMZM2G450163	GRMZM2G450163_P01	TRUE	TRUE	vGVVFSGR	91%	n+304 (+304)	28.42	25.00
3301	seq=translation; coord=6:4896839..4897613:-1; parent_transcript=GRMZM2G391364_T01; parent_gene=GRMZM2G391364	GRMZM2G391364_P01	TRUE	TRUE	dLTEDQIASMR	95%	n+304 (+304)	34.99	25.00
3302	seq=translation; coord=6:4896839..4897613:-1; parent_transcript=GRMZM2G391364_T01; parent_gene=GRMZM2G391364	GRMZM2G391364_P01	TRUE	TRUE	iAPSELGVLMR	93%	n+304 (+304)	31.69	26.74
3303	seq=translation; coord=6:4896839..4897613:-1; parent_transcript=GRMZM2G391364_T01; parent_gene=GRMZM2G391364	GRMZM2G391364_P01	TRUE	TRUE	lEAHEFDEWIR	95%	n+304 (+304)	56.34	25.00
3304	seq=translation; coord=6:4896839..4897613:-1; parent_transcript=GRMZM2G391364_T01; parent_gene=GRMZM2G391364	GRMZM2G391364_P01	TRUE	TRUE	yDDFILR	91%	n+304 (+304)	28.15	25.00
3305	seq=translation; coord=5:185405145..185408787:1; parent_transcript=GRMZM2G148387_T01; parent_gene=GRMZM2G148387	GRMZM2G148387_P01	TRUE	TRUE	eIIASSPVDLALR	95%	n+304 (+304)	39.83	25.51
3306	seq=translation; coord=5:185405145..185408787:1; parent_transcript=GRMZM2G148387_T01; parent_gene=GRMZM2G148387	GRMZM2G148387_P01	TRUE	TRUE	lVPLLTEAGAIVTAR	95%	n+304 (+304)	59.09	25.00
3307	seq=translation; coord=5:185405145..185408787:1; parent_transcript=GRMZM2G148387_T01; parent_gene=GRMZM2G148387	GRMZM2G148387_P01	TRUE	TRUE	tVPNVFVk	90%	n+304 (+304), K+304 (+304)	26.50	25.00
3308	seq=translation; coord=3:141261688..141325830:1; parent_transcript=GRMZM2G073584_T02; parent_gene=GRMZM2G073584	GRMZM2G073584_P02	TRUE	TRUE	aTPEMWPSLIAk	95%	n+304 (+304), K+304 (+304)	35.29	26.33
3309	seq=translation; coord=3:141261688..141325830:1; parent_transcript=GRMZM2G073584_T02; parent_gene=GRMZM2G073584	GRMZM2G073584_P02	TRUE	TRUE	dISDYLSYTTR	95%	n+304 (+304)	37.77	25.00
3310	seq=translation; coord=3:141261688..141325830:1; parent_transcript=GRMZM2G073584_T02; parent_gene=GRMZM2G073584	GRMZM2G073584_P02	TRUE	TRUE	dVPGIEFR	95%	n+304 (+304)	30.99	25.35
3311	seq=translation; coord=3:141261688..141325830:1; parent_transcript=GRMZM2G073584_T02; parent_gene=GRMZM2G073584	GRMZM2G073584_P02	TRUE	TRUE	gFLPSLTIDQIR	95%	n+304 (+304)	38.41	25.17
3312	seq=translation; coord=3:141261688..141325830:1; parent_transcript=GRMZM2G073584_T02; parent_gene=GRMZM2G073584	GRMZM2G073584_P02	TRUE	TRUE	lVAAEGLFLFLR	95%	n+304 (+304)	43.14	25.00
3313	seq=translation; coord=10:148228962..148233420:1; parent_transcript=GRMZM2G008714_T01; parent_gene=GRMZM2G008714	GRMZM2G008714_P01,GRMZM2G150098_P01	TRUE	TRUE	aALIVVLTR	95%	n+304 (+304)	33.20	25.00
3314	seq=translation; coord=10:148228962..148233420:1; parent_transcript=GRMZM2G008714_T01; parent_gene=GRMZM2G008714	GRMZM2G008714_P01,GRMZM2G150098_P01	TRUE	TRUE	aTDSESTEVILEAALk	92%	n+304 (+304), K+304 (+304)	27.75	25.99
3315	seq=translation; coord=10:148228962..148233420:1; parent_transcript=GRMZM2G008714_T01; parent_gene=GRMZM2G008714	GRMZM2G008714_P01,GRMZM2G150098_P01	TRUE	TRUE	eTDAFMVAR	86%	n+304 (+304)	25.37	25.00

3316	seq=translation; coord=10:148228962..148233420:1; parent_transcript=GRMZM2G008714_T01; parent_gene=GRMZM2G008714	GRMZM2G008714_P01,GRMZM2G150098_P01	TRUE	TRUE	gLIPLLAEGSAk	95%	n+304 (+304), K+304 (+304)	53.66	25.50
3317	seq=translation; coord=10:148228962..148233420:1; parent_transcript=GRMZM2G008714_T01; parent_gene=GRMZM2G008714	GRMZM2G008714_P01,GRMZM2G150098_P01	TRUE	TRUE	iGVASVIk	93%	n+304 (+304), K+304 (+304)	27.60	25.00
3318	seq=translation; coord=10:148228962..148233420:1; parent_transcript=GRMZM2G008714_T01; parent_gene=GRMZM2G008714	GRMZM2G008714_P01,GRMZM2G150098_P01	TRUE	TRUE	iLADLDR	93%	n+304 (+304)	31.40	27.07
3319	seq=translation; coord=10:148228962..148233420:1; parent_transcript=GRMZM2G008714_T01; parent_gene=GRMZM2G008714	GRMZM2G008714_P01,GRMZM2G150098_P01	TRUE	TRUE	sAPLPMSPLESLASSAVR	95%	n+304 (+304)	62.37	25.74
3320	seq=translation; coord=5:213904655..213909590:1; parent_transcript=GRMZM5G836910_T01; parent_gene=GRMZM5G836910	GRMZM5G836910_P01	TRUE	TRUE	eYLPIEGLAAFNk	95%	n+304 (+304), K+304 (+304)	42.47	26.22
3321	seq=translation; coord=5:213904655..213909590:1; parent_transcript=GRMZM5G836910_T01; parent_gene=GRMZM5G836910	GRMZM5G836910_P01	TRUE	TRUE	fEGVPMAPPDPILGVSEAFk	95%	n+304 (+304), K+304 (+304)	30.07	25.91
3322	seq=translation; coord=5:213904655..213909590:1; parent_transcript=GRMZM5G836910_T01; parent_gene=GRMZM5G836910	GRMZM5G836910_P01	TRUE	TRUE	iADVIQEk	95%	n+304 (+304), K+304 (+304)	38.19	25.00
3323	seq=translation; coord=5:213904655..213909590:1; parent_transcript=GRMZM5G836910_T01; parent_gene=GRMZM5G836910	GRMZM5G836910_P01	TRUE	TRUE	iSLAGLSLAk	95%	n+304 (+304), K+304 (+304)	45.92	25.00
3324	seq=translation; coord=5:213904655..213909590:1; parent_transcript=GRMZM5G836910_T01; parent_gene=GRMZM5G836910	GRMZM5G836910_P01	TRUE	TRUE	iVANVVGDPTMFGEWk	95%	n+304 (+304), K+304 (+304)	69.51	25.63
3325	seq=translation; coord=5:213904655..213909590:1; parent_transcript=GRMZM5G836910_T01; parent_gene=GRMZM5G836910	GRMZM5G836910_P01	TRUE	TRUE	qEMELMAGR	94%	n+304 (+304)	28.97	25.00
3326	seq=translation; coord=1:91875822..91877199:-1; parent_transcript=GRMZM2G176595_T01; parent_gene=GRMZM2G176595	GRMZM2G176595_P01	TRUE	TRUE	gTNQYPFSSMTScGNEPIFk	95%	n+304 (+304), Carbamidomethyl (+57), K+304 (+304)	36.28	25.00
3327	seq=translation; coord=1:91875822..91877199:-1; parent_transcript=GRMZM2G176595_T01; parent_gene=GRMZM2G176595	GRMZM2G176595_P01	TRUE	TRUE	hAGIIDMQFR	95%	n+304 (+304)	44.79	25.00
3328	seq=translation; coord=1:91875822..91877199:-1; parent_transcript=GRMZM2G176595_T01; parent_gene=GRMZM2G176595	GRMZM2G176595_P01	TRUE	TRUE	nVIPANYIPDVDYR	95%	n+304 (+304)	46.09	25.00
3329	seq=translation; coord=10:23213183..23215664:1; parent_transcript=GRMZM2G036427_T01; parent_gene=GRMZM2G036427	GRMZM2G036427_P01,GRMZM2G036427_P04	TRUE	TRUE	gLcTQASDAVFEALEGDAR	95%	n+304 (+304), Carbamidomethyl (+57)	74.10	25.00
3330	seq=translation; coord=10:23213183..23215664:1; parent_transcript=GRMZM2G036427_T01; parent_gene=GRMZM2G036427	GRMZM2G036427_P01,GRMZM2G036427_P04	TRUE	TRUE	nVLcEAVER	95%	n+304 (+304), Carbamidomethyl (+57)	37.34	25.00
3331	seq=translation; coord=9:136016928..136020802:-1; parent_transcript=GRMZM2G152417_T01; parent_gene=GRMZM2G152417	GRMZM2G152417_P01	TRUE	TRUE	aNAEAFAGGWYHSGDLGVk	95%	n+304 (+304), K+304 (+304)	27.91	25.02
3332	seq=translation; coord=9:136016928..136020802:-1; parent_transcript=GRMZM2G152417_T01; parent_gene=GRMZM2G152417	GRMZM2G152417_P01	TRUE	TRUE	dIDDLPR	90%	n+304 (+304)	27.55	25.00
3333	seq=translation; coord=9:136016928..136020802:-1; parent_transcript=GRMZM2G152417_T01; parent_gene=GRMZM2G152417	GRMZM2G152417_P01	TRUE	TRUE	nDANYTALTPLWFLER	92%	n+304 (+304)	26.81	25.00

3334	seq=translation; coord=9:136016928..136020802:-1; parent_transcript=GRMZM2G152417_T01; parent_gene=GRMZM2G152417	GRMZM2G152417_P01	TRUE	TRUE	sVIFGPLPk	95%	n+304 (+304), K+304 (+304)	35.40	25.00
3335	seq=translation; coord=9:136016928..136020802:-1; parent_transcript=GRMZM2G152417_T01; parent_gene=GRMZM2G152417	GRMZM2G152417_P01	TRUE	TRUE	vLcAHPAVLEVSVVAR	95%	n+304 (+304), Carbamidomethyl (+57)	45.21	25.74
3336	seq=translation; coord=9:136016928..136020802:-1; parent_transcript=GRMZM2G152417_T01; parent_gene=GRMZM2G152417	GRMZM2G152417_P01	TRUE	TRUE	wGESPcAFVTLk	87%	n+304 (+304), Carbamidomethyl (+57), K+304 (+304)	26.31	25.53
3337	seq=translation; coord=9:136016928..136020802:-1; parent_transcript=GRMZM2G152417_T01; parent_gene=GRMZM2G152417	GRMZM2G152417_P01	TRUE	TRUE	yIAMEGLDVVDPk	95%	n+304 (+304), K+304 (+304)	70.37	25.93
3338	seq=translation; coord=8:77429944..77433319:1; parent_transcript=GRMZM2G464401_T01; parent_gene=GRMZM2G464401	GRMZM2G464401_P01	TRUE	TRUE	dFGDADAHGFEGGYGGGGGGFGDGGVAR	95%	n+304 (+304)	66.84	25.00
3339	seq=translation; coord=8:77429944..77433319:1; parent_transcript=GRMZM2G464401_T01; parent_gene=GRMZM2G464401	GRMZM2G464401_P01	TRUE	TRUE	dGDEVFIk	95%	n+304 (+304), K+304 (+304)	34.99	26.24
3340	seq=translation; coord=8:77429944..77433319:1; parent_transcript=GRMZM2G464401_T01; parent_gene=GRMZM2G464401	GRMZM2G464401_P01	TRUE	TRUE	eMTLEEYEk	95%	n+304 (+304), K+304 (+304)	48.52	25.00
3341	seq=translation; coord=8:77429944..77433319:1; parent_transcript=GRMZM2G464401_T01; parent_gene=GRMZM2G464401	GRMZM2G464401_P01	TRUE	TRUE	gPAAAPAIEDQAQFPALA	95%	n+304 (+304)	45.73	25.00
3342	seq=translation; coord=8:77429944..77433319:1; parent_transcript=GRMZM2G464401_T01; parent_gene=GRMZM2G464401	GRMZM2G464401_P01	TRUE	TRUE	rDFGDADAHGFEGGYGGGGGGFGDGGVAR	95%	n+304 (+304)	64.89	25.00
3343	seq=translation; coord=8:77429944..77433319:1; parent_transcript=GRMZM2G464401_T01; parent_gene=GRMZM2G464401	GRMZM2G464401_P01	TRUE	TRUE	sLSINEFLkPAEGER	95%	n+304 (+304), K+304 (+304)	46.29	25.89
3344	seq=translation; coord=3:43991720..43996960:1; parent_transcript=GRMZM2G018566_T01; parent_gene=GRMZM2G018566	GRMZM2G018566_P01,GRMZM2G018566_P02	TRUE	TRUE	aTLFPGDGIGPEIAESVk	95%	n+304 (+304), K+304 (+304)	56.55	25.42
3345	seq=translation; coord=3:43991720..43996960:1; parent_transcript=GRMZM2G018566_T01; parent_gene=GRMZM2G018566	GRMZM2G018566_P01,GRMZM2G018566_P02	TRUE	TRUE	nLANPTALMLSAVMMLR	95%	n+304 (+304)	50.17	26.03
3346	seq=translation; coord=3:43991720..43996960:1; parent_transcript=GRMZM2G018566_T01; parent_gene=GRMZM2G018566	GRMZM2G018566_P01,GRMZM2G018566_P02	TRUE	TRUE	qVFNVAGVPIEWEEHYVGTEVDPR	95%	n+304 (+304)	59.85	25.00
3347	seq=translation; coord=3:43991720..43996960:1; parent_transcript=GRMZM2G018566_T01; parent_gene=GRMZM2G018566	GRMZM2G018566_P01,GRMZM2G018566_P02	TRUE	TRUE	tESFLTWESLESVR	95%	n+304 (+304)	44.45	25.00
3348	seq=translation; coord=3:43991720..43996960:1; parent_transcript=GRMZM2G018566_T01; parent_gene=GRMZM2G018566	GRMZM2G018566_P01,GRMZM2G018566_P02	TRUE	TRUE	yDDVNLVTIR	95%	n+304 (+304)	32.69	25.31
3349	seq=translation; coord=2:176644918..176648601:1; parent_transcript=GRMZM2G150616_T01; parent_gene=GRMZM2G150616	GRMZM2G150616_P01	TRUE	TRUE	aVAVGADISLDTATGNLTk	95%	n+304 (+304), K+304 (+304)	48.83	25.90
3350	seq=translation; coord=4:154624996..154627067:1; parent_transcript=GRMZM2G156785_T01; parent_gene=GRMZM2G156785	GRMZM2G156785_P01,GRMZM2G156785_P03, GRMZM2G156785_P04	TRUE	TRUE	qEYQAQHPGNk	95%	n+304 (+304), K+304 (+304)	37.69	25.79
3351	seq=translation; coord=4:154624996..154627067:1; parent_transcript=GRMZM2G156785_T01; parent_gene=GRMZM2G156785	GRMZM2G156785_P01,GRMZM2G156785_P03, GRMZM2G156785_P04	TRUE	TRUE	sMSEQEk	90%	n+304 (+304), K+304 (+304)	25.22	25.00

3352	seq=translation; coord=4:154624996..154627067:1; parent_transcript=GRMZM2G156785_T01; parent_gene=GRMZM2G156785	GRMZM2G156785_P01,GRMZM2G156785_P03, GRMZM2G156785_P04	TRUE	TRUE	sVAAVSk	95%	n+304 (+304), K+304 (+304)	29.20	26.07
3353	seq=translation; coord=9:23256308..23260236:-1; parent_transcript=GRMZM2G024993_T01; parent_gene=GRMZM2G024993	GRMZM2G024993_P01,GRMZM2G024993_P06	TRUE	TRUE	eALQAEVGLPVDR	95%	n+304 (+304)	59.36	26.22
3354	seq=translation; coord=9:23256308..23260236:-1; parent_transcript=GRMZM2G024993_T01; parent_gene=GRMZM2G024993	GRMZM2G024993_P01,GRMZM2G024993_P06	TRUE	TRUE	fAFSDYPELNLPER	95%	n+304 (+304)	58.89	25.00
3355	seq=translation; coord=9:23256308..23260236:-1; parent_transcript=GRMZM2G024993_T01; parent_gene=GRMZM2G024993	GRMZM2G024993_P01,GRMZM2G024993_P06	TRUE	TRUE	fNAALAHHIMAGADVLAVTSR	95%	n+304 (+304)	43.63	26.07
3356	seq=translation; coord=9:23256308..23260236:-1; parent_transcript=GRMZM2G024993_T01; parent_gene=GRMZM2G024993	GRMZM2G024993_P01,GRMZM2G024993_P06	TRUE	TRUE	fSLLcQAALEAPR	91%	n+304 (+304), Carbamidomethyl (+57)	28.73	25.61
3357	seq=translation; coord=9:23256308..23260236:-1; parent_transcript=GRMZM2G024993_T01; parent_gene=GRMZM2G024993	GRMZM2G024993_P01,GRMZM2G024993_P06	TRUE	TRUE	sSFDFIDGYEkPVEGR	95%	n+304 (+304), K+304 (+304)	26.68	25.00
3358	seq=translation; coord=9:23256308..23260236:-1; parent_transcript=GRMZM2G024993_T01; parent_gene=GRMZM2G024993	GRMZM2G024993_P01,GRMZM2G024993_P06	TRUE	TRUE	vFVDHPLFLER	95%	n+304 (+304)	49.02	26.50
3359	seq=translation; coord=9:23256308..23260236:-1; parent_transcript=GRMZM2G024993_T01; parent_gene=GRMZM2G024993	GRMZM2G024993_P01,GRMZM2G024993_P06	TRUE	TRUE	vLTVSPYYAEELISGIAR	95%	n+304 (+304)	43.06	26.06
3360	seq=translation; coord=9:23256308..23260236:-1; parent_transcript=GRMZM2G024993_T01; parent_gene=GRMZM2G024993	GRMZM2G024993_P01,GRMZM2G024993_P06	TRUE	TRUE	vVGTPAYEEMVR	95%	n+304 (+304)	37.12	25.00
3361	seq=translation; coord=4:37762302..37763543:-1; parent_transcript=GRMZM2G112050_T01; parent_gene=GRMZM2G112050	GRMZM2G112050_P01,GRMZM2G158034_P01, GRMZM2G158034_P04	TRUE	TRUE	aHGLAPEIPEDLYFLIk	95%	n+304 (+304), K+304 (+304)	42.89	25.00
3362	seq=translation; coord=4:37762302..37763543:-1; parent_transcript=GRMZM2G112050_T01; parent_gene=GRMZM2G112050	GRMZM2G112050_P01,GRMZM2G158034_P01, GRMZM2G158034_P04	TRUE	TRUE	dQHGIPLVk	90%	n+304 (+304), K+304 (+304)	25.32	25.00
3363	seq=translation; coord=4:37762302..37763543:-1; parent_transcript=GRMZM2G112050_T01; parent_gene=GRMZM2G112050	GRMZM2G112050_P01,GRMZM2G158034_P01, GRMZM2G158034_P04	TRUE	TRUE	gISSSALPYk	92%	n+304 (+304), K+304 (+304)	28.65	25.00
3364	seq=translation; coord=4:37762302..37763543:-1; parent_transcript=GRMZM2G112050_T01; parent_gene=GRMZM2G112050	GRMZM2G112050_P01,GRMZM2G158034_P01, GRMZM2G158034_P04	TRUE	TRUE	kGQMPSQIGVLLR	95%	K+304 (+304), n+304 (+304)	36.74	25.00
3365	seq=translation; coord=4:37762302..37763543:-1; parent_transcript=GRMZM2G112050_T01; parent_gene=GRMZM2G112050	GRMZM2G112050_P01,GRMZM2G158034_P01, GRMZM2G158034_P04	TRUE	TRUE	tPPTWLk	90%	n+304 (+304), K+304 (+304)	28.08	25.99
3366	seq=translation; coord=9:28603999..28607933:1; parent_transcript=GRMZM2G153969_T01; parent_gene=GRMZM2G153969	GRMZM2G153969_P01	TRUE	TRUE	iEVTQPADFk	95%	n+304 (+304), K+304 (+304)	33.91	26.38
3367	seq=translation; coord=9:28603999..28607933:1; parent_transcript=GRMZM2G153969_T01; parent_gene=GRMZM2G153969	GRMZM2G153969_P01	TRUE	TRUE	nEQVDLLkPDTTVIFR	95%	n+304 (+304), K+304 (+304)	31.97	25.00
3368	seq=translation; coord=9:28603999..28607933:1; parent_transcript=GRMZM2G153969_T01; parent_gene=GRMZM2G153969	GRMZM2G153969_P01	TRUE	TRUE	vAEcLVGDSTGTVLVTAR	95%	n+304 (+304), Carbamidomethyl (+57)	96.56	25.84
3369	seq=translation; coord=3:197406823..197407784:-1; parent_transcript=GRMZM2G010762_T01; parent_gene=GRMZM2G010762	GRMZM2G010762_P01	TRUE	TRUE	dGWVLDPTESYNHWAGR	95%	n+304 (+304)	37.25	25.00

3370	seq=translation; coord=3:197406823..197407784:-1; parent_transcript=GRMZM2G010762_T01; parent_gene=GRMZM2G010762	GRMZM2G010762_P01	TRUE	TRUE	lYIIVMAVR	89%	n+304 (+304)	27.54	25.93
3371	seq=translation; coord=3:197406823..197407784:-1; parent_transcript=GRMZM2G010762_T01; parent_gene=GRMZM2G010762	GRMZM2G010762_P01	TRUE	TRUE	sGPFFFISSDEER	95%	n+304 (+304)	66.88	25.00
3372	seq=translation; coord=1:7192641..7198439:-1; parent_transcript=GRMZM2G057158_T01; parent_gene=GRMZM2G057158	GRMZM2G057158_P01,GRMZM2G057158_P02	TRUE	TRUE	aDLGATVGTFk	95%	n+304 (+304), K+304 (+304)	49.00	25.93
3373	seq=translation; coord=1:7192641..7198439:-1; parent_transcript=GRMZM2G057158_T01; parent_gene=GRMZM2G057158	GRMZM2G057158_P01,GRMZM2G057158_P02	TRUE	TRUE	aIVADScDVPAPQQR	95%	n+304 (+304), Carbamidomethyl (+57)	57.06	25.00
3374	seq=translation; coord=1:7192641..7198439:-1; parent_transcript=GRMZM2G057158_T01; parent_gene=GRMZM2G057158	GRMZM2G057158_P01,GRMZM2G057158_P02	TRUE	TRUE	eIMNMPLMQNLMNSPELIR	95%	n+304 (+304)	67.44	25.00
3375	seq=translation; coord=1:7192641..7198439:-1; parent_transcript=GRMZM2G057158_T01; parent_gene=GRMZM2G057158	GRMZM2G057158_P01,GRMZM2G057158_P02	TRUE	TRUE	eMIQNPEFIR	95%	n+304 (+304)	30.06	25.00
3376	seq=translation; coord=1:7192641..7198439:-1; parent_transcript=GRMZM2G057158_T01; parent_gene=GRMZM2G057158	GRMZM2G057158_P01,GRMZM2G057158_P02	TRUE	TRUE	nPDLAHVLNDPSIMR	95%	n+304 (+304)	29.63	25.00
3377	seq=translation; coord=2:110896551..110900469:-1; parent_transcript=GRMZM2G033894_T01; parent_gene=GRMZM2G033894	GRMZM2G033894_P01	TRUE	TRUE	aAQAAAAHPAVTR	95%	n+304 (+304)	52.21	25.00
3378	seq=translation; coord=2:110896551..110900469:-1; parent_transcript=GRMZM2G033894_T01; parent_gene=GRMZM2G033894	GRMZM2G033894_P01	TRUE	TRUE	aTSDPEIWk	95%	n+304 (+304), K+304 (+304)	34.71	25.00
3379	seq=translation; coord=2:110896551..110900469:-1; parent_transcript=GRMZM2G033894_T01; parent_gene=GRMZM2G033894	GRMZM2G033894_P01	TRUE	TRUE	eEALELYEDMVLGR	95%	n+304 (+304)	73.77	25.00
3380	seq=translation; coord=2:110896551..110900469:-1; parent_transcript=GRMZM2G033894_T01; parent_gene=GRMZM2G033894	GRMZM2G033894_P01	TRUE	TRUE	gFGIGPDGk	95%	n+304 (+304), K+304 (+304)	29.43	26.34
3381	seq=translation; coord=2:110896551..110900469:-1; parent_transcript=GRMZM2G033894_T01; parent_gene=GRMZM2G033894	GRMZM2G033894_P01	TRUE	TRUE	kIDDVVEEAVEFADASPHPPR	95%	K+304 (+304), n+304 (+304)	84.54	25.25
3382	seq=translation; coord=2:110896551..110900469:-1; parent_transcript=GRMZM2G033894_T01; parent_gene=GRMZM2G033894	GRMZM2G033894_P01	TRUE	TRUE	lPIVFVVENNLWAIGMSHIR	95%	n+304 (+304)	35.84	25.00
3383	seq=translation; coord=2:110896551..110900469:-1; parent_transcript=GRMZM2G033894_T01; parent_gene=GRMZM2G033894	GRMZM2G033894_P01	TRUE	TRUE	rGEGPTLVEcETYR	95%	n+304 (+304), Carbamidomethyl (+57)	44.73	25.00
3384	seq=translation; coord=2:110896551..110900469:-1; parent_transcript=GRMZM2G033894_T01; parent_gene=GRMZM2G033894	GRMZM2G033894_P01	TRUE	TRUE	sVMAELFGk	90%	n+304 (+304), K+304 (+304)	27.30	26.68
3385	seq=translation; coord=2:110896551..110900469:-1; parent_transcript=GRMZM2G033894_T01; parent_gene=GRMZM2G033894	GRMZM2G033894_P01	TRUE	TRUE	yIIEQNLATESELk	95%	n+304 (+304), K+304 (+304)	57.66	25.43
3386	seq=translation; coord=8:116617793..116620719:-1; parent_transcript=GRMZM5G802801_T01; parent_gene=GRMZM5G802801	GRMZM5G802801_P01	TRUE	TRUE	mYQGAGADMGAAAGMDEDAPAAGGSAAGPk	95%	n+304 (+304), K+304 (+304)	65.62	25.00
3387	seq=translation; coord=8:116617793..116620719:-1; parent_transcript=GRMZM5G802801_T01; parent_gene=GRMZM5G802801	GRMZM5G802801_P01	TRUE	TRUE	myQGAGADMGAAAGMDEDAPAAGGSAAGPk	95%	n+304 (+304), iTRAQ8plex (+304), K+304 (+304)	54.08	25.00

3388	seq=translation; coord=8:116617793..116620719:-1; parent_transcript=GRMZM5G802801_T01; parent_gene=GRMZM5G802801	GRMZM5G802801_P01	TRUE	TRUE	qFSAEEISSMVLTk	95%	n+304 (+304), K+304 (+304)	39.08	26.09
3389	seq=translation; coord=2:125696253..125710943:1; parent_transcript=GRMZM5G877316_T02; parent_gene=GRMZM5G877316	GRMZM5G877316_P02,GRMZM5G877316_P03	TRUE	TRUE	eNTLDDLIALLk	95%	n+304 (+304), K+304 (+304)	39.67	25.00
3390	seq=translation; coord=2:125696253..125710943:1; parent_transcript=GRMZM5G877316_T02; parent_gene=GRMZM5G877316	GRMZM5G877316_P02,GRMZM5G877316_P03	TRUE	TRUE	gIVLSFITEFFk	95%	n+304 (+304), K+304 (+304)	35.42	25.28
3391	seq=translation; coord=2:125696253..125710943:1; parent_transcript=GRMZM5G877316_T02; parent_gene=GRMZM5G877316	GRMZM5G877316_P02,GRMZM5G877316_P03	TRUE	TRUE	lAIFTALAFSQk	95%	n+304 (+304), K+304 (+304)	27.97	25.00
3392	seq=translation; coord=2:125696253..125710943:1; parent_transcript=GRMZM5G877316_T02; parent_gene=GRMZM5G877316	GRMZM5G877316_P02,GRMZM5G877316_P03	TRUE	TRUE	lSGLPPETVFQPLLk	95%	n+304 (+304), K+304 (+304)	30.11	25.00
3393	seq=translation; coord=2:125696253..125710943:1; parent_transcript=GRMZM5G877316_T02; parent_gene=GRMZM5G877316	GRMZM5G877316_P02,GRMZM5G877316_P03	TRUE	TRUE	mEDNLLDFFPSAk	95%	n+304 (+304), K+304 (+304)	51.53	25.00
3394	seq=translation; coord=2:125696253..125710943:1; parent_transcript=GRMZM5G877316_T02; parent_gene=GRMZM5G877316	GRMZM5G877316_P02,GRMZM5G877316_P03	TRUE	TRUE	sIESSDLNFSR	93%	n+304 (+304)	29.53	25.00
3395	seq=translation; coord=6:120018887..120020772:1; parent_transcript=GRMZM2G383404_T01; parent_gene=GRMZM2G383404	GRMZM2G383404_P01	TRUE	TRUE	aEQGSDIGDVDIPGVcR	95%	n+304 (+304), Carbamidomethyl (+57)	67.37	25.00
3396	seq=translation; coord=6:120018887..120020772:1; parent_transcript=GRMZM2G383404_T01; parent_gene=GRMZM2G383404	GRMZM2G383404_P01	TRUE	TRUE	aWVDQEALLk	95%	n+304 (+304), K+304 (+304)	38.44	26.19
3397	seq=translation; coord=6:120018887..120020772:1; parent_transcript=GRMZM2G383404_T01; parent_gene=GRMZM2G383404	GRMZM2G383404_P01	TRUE	TRUE	eIAAGLEASNcR	95%	n+304 (+304), Carbamidomethyl (+57)	32.29	25.00
3398	seq=translation; coord=6:120018887..120020772:1; parent_transcript=GRMZM2G383404_T01; parent_gene=GRMZM2G383404	GRMZM2G383404_P01	TRUE	TRUE	kAEQGSDIGDVDIPGVcR	95%	K+304 (+304), n+304 (+304), Carbamidomethyl (+57)	43.32	25.00
3399	seq=translation; coord=6:120018887..120020772:1; parent_transcript=GRMZM2G383404_T01; parent_gene=GRMZM2G383404	GRMZM2G383404_P01	TRUE	TRUE	sAHLLGPLIAGAAPR	95%	n+304 (+304)	54.91	25.00
3400	seq=translation; coord=6:120018887..120020772:1; parent_transcript=GRMZM2G383404_T01; parent_gene=GRMZM2G383404	GRMZM2G383404_P01	TRUE	TRUE	sVVYVAFGNR	88%	n+304 (+304)	26.22	25.24
3401	seq=translation; coord=2:60415730..60417474:1; parent_transcript=GRMZM2G085260_T01; parent_gene=GRMZM2G085260	GRMZM2G085260_P01	TRUE	TRUE	gFPRPLLDISAANFGk	95%	n+304 (+304), K+304 (+304)	32.50	25.24
3402	seq=translation; coord=2:60415730..60417474:1; parent_transcript=GRMZM2G085260_T01; parent_gene=GRMZM2G085260	GRMZM2G085260_P01	TRUE	TRUE	kLLAGLLAVESAQDAVIR	95%	K+304 (+304), n+304 (+304)	83.27	25.00
3403	seq=translation; coord=2:60415730..60417474:1; parent_transcript=GRMZM2G085260_T01; parent_gene=GRMZM2G085260	GRMZM2G085260_P01	TRUE	TRUE	lLAGLLAVESAQDAVIR	95%	n+304 (+304)	81.84	25.31
3404	seq=translation; coord=2:60415730..60417474:1; parent_transcript=GRMZM2G085260_T01; parent_gene=GRMZM2G085260	GRMZM2G085260_P01	TRUE	TRUE	vPSYAGGVAEITAR	95%	n+304 (+304)	50.64	25.39
3405	seq=translation; coord=5:56842476..56843934:-1; parent_transcript=GRMZM2G077208_T02; parent_gene=GRMZM2G077208	GRMZM2G077208_P02,GRMZM2G378770_P01	TRUE	TRUE	qHSGEIEASAATPYELQR	95%	n+304 (+304)	62.17	25.00

3406	seq=translation; coord=5:56842476..56843934:-1; parent_transcript=GRMZM2G077208_T02; parent_gene=GRMZM2G077208	GRMZM2G077208_P02,GRMZM2G378770_P01	TRUE	TRUE	qHSGEIEASAATPYELQR	95%	Pyro-cmC (-17), n+304 (+304)	75.41	25.00
3407	seq=translation; coord=5:56842476..56843934:-1; parent_transcript=GRMZM2G077208_T02; parent_gene=GRMZM2G077208	GRMZM2G077208_P02,GRMZM2G378770_P01	TRUE	TRUE	qHSGEIEASAATPyELQR	94%	n+304 (+304), iTRAQ8plex (+304)	26.23	25.00
3408	seq=translation; coord=5:56842476..56843934:-1; parent_transcript=GRMZM2G077208_T02; parent_gene=GRMZM2G077208	GRMZM2G077208_P02,GRMZM2G378770_P01	TRUE	TRUE	qHSGEIEASAATPyELQR	95%	Pyro-cmC (-17), n+304 (+304), iTRAQ8plex (+304)	37.95	25.00
3409	seq=translation; coord=10:22319764..22321055:-1; parent_transcript=GRMZM2G075283_T01; parent_gene=GRMZM2G075283	GRMZM2G075283_P01	TRUE	TRUE	acAGYVDAVEVDGDGGPGSVTTMk	95%	n+304 (+304), Carbamidomethyl (+57), K+304 (+304)	52.35	25.00
3410	seq=translation; coord=10:22319764..22321055:-1; parent_transcript=GRMZM2G075283_T01; parent_gene=GRMZM2G075283	GRMZM2G075283_P01	TRUE	TRUE	lDGAPLAPEDEAR	95%	n+304 (+304)	53.32	25.00
3411	seq=translation; coord=10:22319764..22321055:-1; parent_transcript=GRMZM2G075283_T01; parent_gene=GRMZM2G075283	GRMZM2G075283_P01	TRUE	TRUE	mVEAYLVAHPDEFA	95%	n+304 (+304)	32.71	25.00
3412	seq=translation; coord=10:22319764..22321055:-1; parent_transcript=GRMZM2G075283_T01; parent_gene=GRMZM2G075283	GRMZM2G075283_P01	TRUE	TRUE	tEVLQGGTVSAQLR	94%	n+304 (+304)	31.63	26.17
3413	seq=translation; coord=10:22319764..22321055:-1; parent_transcript=GRMZM2G075283_T01; parent_gene=GRMZM2G075283	GRMZM2G075283_P01	TRUE	TRUE	vEAAGEGAcVAk	95%	n+304 (+304), Carbamidomethyl (+57), K+304 (+304)	79.17	25.00
3414	seq=translation; coord=10:22319764..22321055:-1; parent_transcript=GRMZM2G075283_T01; parent_gene=GRMZM2G075283	GRMZM2G075283_P01	TRUE	TRUE	vVALDAAAR	95%	n+304 (+304)	35.41	28.73
3415	seq=translation; coord=5:208194750..208197747:1; parent_transcript=GRMZM2G061950_T01; parent_gene=GRMZM2G061950	GRMZM2G061950_P01	TRUE	TRUE	aYSDEEIIR	92%	n+304 (+304)	29.24	25.00
3416	seq=translation; coord=5:208194750..208197747:1; parent_transcript=GRMZM2G061950_T01; parent_gene=GRMZM2G061950	GRMZM2G061950_P01	TRUE	TRUE	lALGGMGTDEDDLTR	95%	n+304 (+304)	81.15	25.00
3417	seq=translation; coord=5:208194750..208197747:1; parent_transcript=GRMZM2G061950_T01; parent_gene=GRMZM2G061950	GRMZM2G061950_P01	TRUE	TRUE	lIISILAHR	95%	n+304 (+304)	45.12	25.00
3418	seq=translation; coord=5:208194750..208197747:1; parent_transcript=GRMZM2G061950_T01; parent_gene=GRMZM2G061950	GRMZM2G061950_P01	TRUE	TRUE	lLVPLVSAYR	89%	n+304 (+304)	26.93	25.00
3419	seq=translation; coord=5:208194750..208197747:1; parent_transcript=GRMZM2G061950_T01; parent_gene=GRMZM2G061950	GRMZM2G061950_P01	TRUE	TRUE	sLEEDVAAHVTGDFR	95%	n+304 (+304)	51.41	25.00
3420	seq=translation; coord=10:101520598..101521474:1; parent_transcript=GRMZM2G166141_T01; parent_gene=GRMZM2G166141	GRMZM2G166141_P01	TRUE	TRUE	dPMAVTPDDFFNPAMIIGk	95%	n+304 (+304), K+304 (+304)	62.40	25.00
3421	seq=translation; coord=10:101520598..101521474:1; parent_transcript=GRMZM2G166141_T01; parent_gene=GRMZM2G166141	GRMZM2G166141_P01	TRUE	TRUE	dPmAVTPDDFFNPAMIIGk	95%	n+304 (+304), Oxidation (+16), K+304 (+304)	32.48	25.00
3422	seq=translation; coord=10:101520598..101521474:1; parent_transcript=GRMZM2G166141_T01; parent_gene=GRMZM2G166141	GRMZM2G166141_P01	TRUE	TRUE	dPMAVTPDDFFNPAmIIGk	92%	n+304 (+304), Oxidation (+16), K+304 (+304)	26.90	25.00
3423	seq=translation; coord=10:101520598..101521474:1; parent_transcript=GRMZM2G166141_T01; parent_gene=GRMZM2G166141	GRMZM2G166141_P01	TRUE	TRUE	dPmAVTPDDFFNPAmIIGk	95%	n+304 (+304), Oxidation (+16), Oxidation (+16), K+304 (+304)	32.07	25.00

3424	seq=translation; coord=2:1436938..1441569:-1; parent_transcript=GRMZM2G019500_T01; parent_gene=GRMZM2G019500	GRMZM2G019500_P01	TRUE	TRUE	dGAAAPANDAk	93%	n+304 (+304), K+304 (+304)	27.37	25.13
3425	seq=translation; coord=2:1436938..1441569:-1; parent_transcript=GRMZM2G019500_T01; parent_gene=GRMZM2G019500	GRMZM2G019500_P01	TRUE	TRUE	eEGILLR	95%	n+304 (+304)	32.68	25.09
3426	seq=translation; coord=3:126628409..126636305:-1; parent_transcript=GRMZM2G066650_T01; parent_gene=GRMZM2G066650	GRMZM2G066650_P01	TRUE	TRUE	gLLLQTLTSSVASAR	95%	n+304 (+304)	30.05	25.00
3427	seq=translation; coord=3:126628409..126636305:-1; parent_transcript=GRMZM2G066650_T01; parent_gene=GRMZM2G066650	GRMZM2G066650_P01	TRUE	TRUE	iLPFcDGIMTQLLk	95%	n+304 (+304), Carbamidomethyl (+57), K+304 (+304)	49.94	26.16
3428	seq=translation; coord=3:126628409..126636305:-1; parent_transcript=GRMZM2G066650_T01; parent_gene=GRMZM2G066650	GRMZM2G066650_P01	TRUE	TRUE	lATDMQDIFNITAk	95%	n+304 (+304), K+304 (+304)	43.61	25.37
3429	seq=translation; coord=3:126628409..126636305:-1; parent_transcript=GRMZM2G066650_T01; parent_gene=GRMZM2G066650	GRMZM2G066650_P01	TRUE	TRUE	qALPALVPMLLETLLk	95%	n+304 (+304), K+304 (+304)	33.32	25.00
3430	seq=translation; coord=3:126628409..126636305:-1; parent_transcript=GRMZM2G066650_T01; parent_gene=GRMZM2G066650	GRMZM2G066650_P01	TRUE	TRUE	tQLLMPFAPHIIQFLDALYNGk	95%	n+304 (+304), K+304 (+304)	31.55	25.00
3431	seq=translation; coord=3:126628409..126636305:-1; parent_transcript=GRMZM2G066650_T01; parent_gene=GRMZM2G066650	GRMZM2G066650_P01	TRUE	TRUE	tVGDDIVPLVMPFVEENITk	95%	n+304 (+304), K+304 (+304)	40.92	25.40
3432	seq=translation; coord=1:236088923..236128384:-1; parent_transcript=GRMZM2G053764_T01; parent_gene=GRMZM2G053764	GRMZM2G053764_P01,GRMZM2G053764_P02, GRMZM2G053764_P04,GRMZM2G146374_P02	TRUE	TRUE	gPAESIYQGGVWk	95%	n+304 (+304), K+304 (+304)	36.27	25.92
3433	seq=translation; coord=1:236088923..236128384:-1; parent_transcript=GRMZM2G053764_T01; parent_gene=GRMZM2G053764	GRMZM2G053764_P01,GRMZM2G053764_P02, GRMZM2G053764_P04,GRMZM2G146374_P02	TRUE	TRUE	sPSIGFINk	92%	n+304 (+304), K+304 (+304)	27.46	25.00
3434	seq=translation; coord=1:236088923..236128384:-1; parent_transcript=GRMZM2G053764_T01; parent_gene=GRMZM2G053764	GRMZM2G053764_P01,GRMZM2G053764_P02, GRMZM2G053764_P04,GRMZM2G146374_P02	TRUE	TRUE	vELPDAYPYk	95%	n+304 (+304), K+304 (+304)	48.10	26.19
3435	seq=translation; coord=4:233821047..233828196:1; parent_transcript=GRMZM2G064023_T01; parent_gene=GRMZM2G064023	GRMZM2G064023_P01,GRMZM2G064023_P02, GRMZM2G064023_P03,GRMZM2G064023_P04	TRUE	TRUE	gMIGMLWETSLLDPEEGIR	95%	n+304 (+304)	35.67	25.00
3436	seq=translation; coord=8:6456722..6458297:1; parent_transcript=GRMZM2G328094_T01; parent_gene=GRMZM2G328094	GRMZM2G328094_P01	TRUE	TRUE	aLIGLFHGR	95%	n+304 (+304)	43.66	25.53
3437	seq=translation; coord=8:6456722..6458297:1; parent_transcript=GRMZM2G328094_T01; parent_gene=GRMZM2G328094	GRMZM2G328094_P01	TRUE	TRUE	eSYTAAFVPGDTITGYGVAR	95%	n+304 (+304)	87.00	25.00
3438	seq=translation; coord=8:6456722..6458297:1; parent_transcript=GRMZM2G328094_T01; parent_gene=GRMZM2G328094	GRMZM2G328094_P01	TRUE	TRUE	eYVEGYPR	88%	n+304 (+304)	26.35	25.00
3439	seq=translation; coord=8:6456722..6458297:1; parent_transcript=GRMZM2G328094_T01; parent_gene=GRMZM2G328094	GRMZM2G328094_P01	TRUE	TRUE	iAVcGLISQYNLADGEk	95%	n+304 (+304), Carbamidomethyl (+57), K+304 (+304)	36.55	25.02
3440	seq=translation; coord=8:6456722..6458297:1; parent_transcript=GRMZM2G328094_T01; parent_gene=GRMZM2G328094	GRMZM2G328094_P01	TRUE	TRUE	kGETVFVSAASGAVGQLVGQFAR	95%	K+304 (+304), n+304 (+304)	43.29	25.00
3441	seq=translation; coord=8:6456722..6458297:1; parent_transcript=GRMZM2G328094_T01; parent_gene=GRMZM2G328094	GRMZM2G328094_P01	TRUE	TRUE	lTGAEPAGSVLVR	95%	n+304 (+304)	45.46	26.95

3442	seq=translation; coord=8:6456722..6458297:1; parent_transcript=GRMZM2G328094_T01; parent_gene=GRMZM2G328094	GRMZM2G328094_P01	TRUE	TRUE	vVDSSDPR	89%	n+304 (+304)	26.86	25.00
3443	seq=translation; coord=5:215477924..215482853:1; parent_transcript=GRMZM2G078396_T01; parent_gene=GRMZM2G078396	GRMZM2G078396_P01,GRMZM2G102230_P01, GRMZM2G123495_P01,GRMZM2G150058_P01, GRMZM2G171181_P01	TRUE	TRUE	gSAITGPIGk	95%	n+304 (+304), K+304 (+304)	50.82	25.51
3444	seq=translation; coord=5:215477924..215482853:1; parent_transcript=GRMZM2G078396_T01; parent_gene=GRMZM2G078396	GRMZM2G078396_P01,GRMZM2G102230_P01, GRMZM2G123495_P01,GRMZM2G150058_P01, GRMZM2G171181_P01	TRUE	TRUE	mSLGLPVAATVNcADNTGAk	95%	n+304 (+304), Carbamidomethyl (+57), K+304 (+304)	56.27	25.13
3445	seq=translation; coord=5:215477924..215482853:1; parent_transcript=GRMZM2G078396_T01; parent_gene=GRMZM2G078396	GRMZM2G078396_P01,GRMZM2G102230_P01, GRMZM2G123495_P01,GRMZM2G150058_P01, GRMZM2G171181_P01	TRUE	TRUE	nLYIISVk	95%	n+304 (+304), K+304 (+304)	34.12	25.00
3446	seq=translation; coord=5:215477924..215482853:1; parent_transcript=GRMZM2G078396_T01; parent_gene=GRMZM2G078396	GRMZM2G078396_P01,GRMZM2G102230_P01, GRMZM2G123495_P01,GRMZM2G150058_P01, GRMZM2G171181_P01	TRUE	TRUE	vMPAVIVR	93%	n+304 (+304)	28.38	25.00
3447	seq=translation; coord=3:6926705..6931400:-1; parent_transcript=GRMZM2G166646_T01; parent_gene=GRMZM2G166646	GRMZM2G166646_P01	TRUE	TRUE	dITSVAEk	95%	n+304 (+304), K+304 (+304)	34.04	26.71
3448	seq=translation; coord=3:6926705..6931400:-1; parent_transcript=GRMZM2G166646_T01; parent_gene=GRMZM2G166646	GRMZM2G166646_P01	TRUE	TRUE	lNADVLEQFITENYTASR	95%	n+304 (+304)	59.47	25.00
3449	seq=translation; coord=3:6926705..6931400:-1; parent_transcript=GRMZM2G166646_T01; parent_gene=GRMZM2G166646	GRMZM2G166646_P01	TRUE	TRUE	lVNEFDQIk	95%	n+304 (+304), K+304 (+304)	36.71	26.78
3450	seq=translation; coord=3:6926705..6931400:-1; parent_transcript=GRMZM2G166646_T01; parent_gene=GRMZM2G166646	GRMZM2G166646_P01	TRUE	TRUE	sSGGFWTWLTGAR	95%	n+304 (+304)	50.79	25.00
3451	seq=translation; coord=3:6926705..6931400:-1; parent_transcript=GRMZM2G166646_T01; parent_gene=GRMZM2G166646	GRMZM2G166646_P01	TRUE	TRUE	tMAFTTTANR	88%	n+304 (+304)	26.23	25.00
3452	seq=translation; coord=3:6926705..6931400:-1; parent_transcript=GRMZM2G166646_T01; parent_gene=GRMZM2G166646	GRMZM2G166646_P01	TRUE	TRUE	tYMPEMVEVLIDcVR	95%	n+304 (+304), Carbamidomethyl (+57)	32.21	25.00
3453	seq=translation; coord=3:6926705..6931400:-1; parent_transcript=GRMZM2G166646_T01; parent_gene=GRMZM2G166646	GRMZM2G166646_P01	TRUE	TRUE	tyMPEMVEVLIDcVR	95%	n+304 (+304), iTRAQ8plex (+304), Carbamidomethyl (+57)	50.17	25.00
3454	seq=translation; coord=7:19072056..19090250:-1; parent_transcript=GRMZM2G462325_T01; parent_gene=GRMZM2G462325	GRMZM2G462325_P01	TRUE	TRUE	lASPPSLTGSAISLVSVMQAR	95%	n+304 (+304)	71.27	25.00
3455	seq=translation; coord=1:273865741..273870770:-1; parent_transcript=GRMZM2G416388_T01; parent_gene=GRMZM2G416388	GRMZM2G416388_P01,GRMZM2G416388_P02, GRMZM2G416388_P03,GRMZM2G416388_P06	TRUE	TRUE	gFETATVADVLk	95%	n+304 (+304), K+304 (+304)	44.63	26.24
3456	seq=translation; coord=1:273865741..273870770:-1; parent_transcript=GRMZM2G416388_T01; parent_gene=GRMZM2G416388	GRMZM2G416388_P01,GRMZM2G416388_P02, GRMZM2G416388_P03,GRMZM2G416388_P06	TRUE	TRUE	hIPVIDGTGMLGMVSIGDVVR	95%	n+304 (+304)	56.63	25.43
3457	seq=translation; coord=1:273865741..273870770:-1; parent_transcript=GRMZM2G416388_T01; parent_gene=GRMZM2G416388	GRMZM2G416388_P01,GRMZM2G416388_P02, GRMZM2G416388_P03,GRMZM2G416388_P06	TRUE	TRUE	sIAGIVTER	92%	n+304 (+304)	29.85	25.93
3458	seq=translation; coord=1:273865741..273870770:-1; parent_transcript=GRMZM2G416388_T01; parent_gene=GRMZM2G416388	GRMZM2G416388_P01,GRMZM2G416388_P02, GRMZM2G416388_P03,GRMZM2G416388_P06	TRUE	TRUE	vLQAMQLMTENR	95%	n+304 (+304)	55.70	25.17
3459	seq=translation; coord=1:52807627..52810251:-1; parent_transcript=GRMZM2G018728_T01; parent_gene=GRMZM2G018728	GRMZM2G018728_P01	TRUE	TRUE	dINPQAPTHILIIPk	95%	n+304 (+304), K+304 (+304)	31.79	25.00

3460	seq=translation; coord=1:52807627..52810251:-1; parent_transcript=GRMZM2G018728_T01; parent_gene=GRMZM2G018728	GRMZM2G018728_P01	TRUE	TRUE	eAALAAVLDDSPTIFDk	95%	n+304 (+304), K+304 (+304)	72.28	26.10
3461	seq=translation; coord=1:52807627..52810251:-1; parent_transcript=GRMZM2G018728_T01; parent_gene=GRMZM2G018728	GRMZM2G018728_P01	TRUE	TRUE	eIPSTVVYEDEk	95%	n+304 (+304), K+304 (+304)	41.62	25.90
3462	seq=translation; coord=1:52807627..52810251:-1; parent_transcript=GRMZM2G018728_T01; parent_gene=GRMZM2G018728	GRMZM2G018728_P01	TRUE	TRUE	qEGLEDGYR	92%	n+304 (+304)	27.52	25.00
3463	seq=translation; coord=1:79131877..79132681:-1; parent_transcript=GRMZM2G401328_T01; parent_gene=GRMZM2G401328	GRMZM2G401328_P01	TRUE	TRUE	fGEVTGGEEQVVSGMNYk	95%	n+304 (+304), K+304 (+304)	43.19	25.00
3464	seq=translation; coord=1:79131877..79132681:-1; parent_transcript=GRMZM2G401328_T01; parent_gene=GRMZM2G401328	GRMZM2G401328_P01	TRUE	TRUE	fGEVTGGEEQVVSGmNYk	95%	n+304 (+304), Oxidation (+16), K+304 (+304)	56.21	25.00
3465	seq=translation; coord=1:79131877..79132681:-1; parent_transcript=GRMZM2G401328_T01; parent_gene=GRMZM2G401328	GRMZM2G401328_P01	TRUE	TRUE	fGEVTGGEEQVVSGMNyk	95%	n+304 (+304), iTRAQ8plex (+304), K+304 (+304)	29.95	25.17
3466	seq=translation; coord=1:79131877..79132681:-1; parent_transcript=GRMZM2G401328_T01; parent_gene=GRMZM2G401328	GRMZM2G401328_P01	TRUE	TRUE	lVLDATDADGk	95%	n+304 (+304), K+304 (+304)	55.17	25.87
3467	seq=translation; coord=1:79131877..79132681:-1; parent_transcript=GRMZM2G401328_T01; parent_gene=GRMZM2G401328	GRMZM2G401328_P01	TRUE	TRUE	vAAYGAFVYEQSWTNTR	95%	n+304 (+304)	57.30	25.00
3468	seq=translation; coord=1:79131877..79132681:-1; parent_transcript=GRMZM2G401328_T01; parent_gene=GRMZM2G401328	GRMZM2G401328_P01	TRUE	TRUE	vAAyGAFVYEQSWTNTR	95%	n+304 (+304), iTRAQ8plex (+304)	37.11	25.49
3469	seq=translation; coord=1:79131877..79132681:-1; parent_transcript=GRMZM2G401328_T01; parent_gene=GRMZM2G401328	GRMZM2G401328_P01	TRUE	TRUE	vAAYGAFVyEQSWTNTR	95%	n+304 (+304), iTRAQ8plex (+304)	31.60	25.47
3470	seq=translation; coord=8:162579348..162580103:-1; parent_transcript=GRMZM2G030717_T01; parent_gene=GRMZM2G030717	GRMZM2G030717_P01	TRUE	TRUE	dVEGDGEVQELGR	95%	n+304 (+304)	39.12	25.00
3471	seq=translation; coord=8:162579348..162580103:-1; parent_transcript=GRMZM2G030717_T01; parent_gene=GRMZM2G030717	GRMZM2G030717_P01	TRUE	TRUE	vFDAVVVVkPWLDSR	95%	n+304 (+304), K+304 (+304)	40.69	25.00
3472	seq=translation; coord=8:133178452..133180059:1; parent_transcript=GRMZM2G005552_T01; parent_gene=GRMZM2G005552	GRMZM2G005552_P01	TRUE	TRUE	aWDLPEADAAALVSSQPASGIVR	95%	n+304 (+304)	30.69	25.09
3473	seq=translation; coord=8:133178452..133180059:1; parent_transcript=GRMZM2G005552_T01; parent_gene=GRMZM2G005552	GRMZM2G005552_P01	TRUE	TRUE	dREGVALNcLEAPLDVDIPGGGR	95%	n+304 (+304), Carbamidomethyl (+57)	42.94	25.00
3474	seq=translation; coord=8:133178452..133180059:1; parent_transcript=GRMZM2G005552_T01; parent_gene=GRMZM2G005552	GRMZM2G005552_P01	TRUE	TRUE	iADASGMEWFSIITTPNPIFSHLAGk	95%	n+304 (+304), K+304 (+304)	32.04	25.00
3475	seq=translation; coord=8:133178452..133180059:1; parent_transcript=GRMZM2G005552_T01; parent_gene=GRMZM2G005552	GRMZM2G005552_P01	TRUE	TRUE	rLDSEIFFAPPSN	95%	n+304 (+304)	38.17	25.00
3476	seq=translation; coord=8:133178452..133180059:1; parent_transcript=GRMZM2G005552_T01; parent_gene=GRMZM2G005552	GRMZM2G005552_P01	TRUE	TRUE	vEGGFLFIVPR	95%	n+304 (+304)	63.99	25.00
3477	seq=translation; coord=8:133178452..133180059:1; parent_transcript=GRMZM2G005552_T01; parent_gene=GRMZM2G005552	GRMZM2G005552_P01	TRUE	TRUE	vVVLNTANLPLVk	95%	n+304 (+304), K+304 (+304)	41.08	25.00

3478	seq=translation; coord=7:168744978..168747272:1; parent_transcript=GRMZM2G039757_T01; parent_gene=GRMZM2G039757	GRMZM2G039757_P01	TRUE	TRUE	aADDGWWSVFR	93%	n+304 (+304)	29.29	25.00
3479	seq=translation; coord=7:168744978..168747272:1; parent_transcript=GRMZM2G039757_T01; parent_gene=GRMZM2G039757	GRMZM2G039757_P01	TRUE	TRUE	aLETEVAVFR	90%	n+304 (+304)	27.55	25.00
3480	seq=translation; coord=7:168744978..168747272:1; parent_transcript=GRMZM2G039757_T01; parent_gene=GRMZM2G039757	GRMZM2G039757_P01	TRUE	TRUE	ePDLFYTTWSPTER	95%	n+304 (+304)	52.99	25.00
3481	seq=translation; coord=7:168744978..168747272:1; parent_transcript=GRMZM2G039757_T01; parent_gene=GRMZM2G039757	GRMZM2G039757_P01	TRUE	TRUE	gGWPAWHGDAALFFHR	95%	n+304 (+304)	43.78	25.00
3482	seq=translation; coord=7:168744978..168747272:1; parent_transcript=GRMZM2G039757_T01; parent_gene=GRMZM2G039757	GRMZM2G039757_P01	TRUE	TRUE	nLYVVDTAR	91%	n+304 (+304)	27.86	25.00
3483	seq=translation; coord=4:131010547..131027664:-1; parent_transcript=GRMZM2G006672_T02; parent_gene=GRMZM2G006672	GRMZM2G006672_P02	TRUE	TRUE	aNPVALLLSSAMMLR	95%	n+304 (+304)	42.27	25.20
3484	seq=translation; coord=4:131010547..131027664:-1; parent_transcript=GRMZM2G006672_T02; parent_gene=GRMZM2G006672	GRMZM2G006672_P02	TRUE	TRUE	aNPVALLLSSAmMLR	91%	n+304 (+304), Oxidation (+16)	26.22	25.21
3485	seq=translation; coord=4:131010547..131027664:-1; parent_transcript=GRMZM2G006672_T02; parent_gene=GRMZM2G006672	GRMZM2G006672_P02	TRUE	TRUE	aNPVALLLSSAMmLR	90%	n+304 (+304), Oxidation (+16)	25.56	25.24
3486	seq=translation; coord=4:131010547..131027664:-1; parent_transcript=GRMZM2G006672_T02; parent_gene=GRMZM2G006672	GRMZM2G006672_P02	TRUE	TRUE	hDNVDIVVIR	95%	n+304 (+304)	44.77	25.93
3487	seq=translation; coord=7:159130478..159136959:-1; parent_transcript=GRMZM2G107562_T01; parent_gene=GRMZM2G107562	GRMZM2G107562_P01	TRUE	TRUE	qFHNVLAVSk	95%	n+304 (+304), K+304 (+304)	38.70	25.95
3488	seq=translation; coord=7:159130478..159136959:-1; parent_transcript=GRMZM2G107562_T01; parent_gene=GRMZM2G107562	GRMZM2G107562_P01	TRUE	TRUE	qFHNVLAVSk	95%	Pyro-cmC (-17), n+304 (+304), K+304 (+304)	41.74	25.01
3489	seq=translation; coord=7:159130478..159136959:-1; parent_transcript=GRMZM2G107562_T01; parent_gene=GRMZM2G107562	GRMZM2G107562_P01	TRUE	TRUE	qIVIGDTVVFTYNk	95%	n+304 (+304), K+304 (+304)	49.65	25.30
3490	seq=translation; coord=2:22990515..22995670:1; parent_transcript=GRMZM2G122937_T01; parent_gene=GRMZM2G122937	GRMZM2G122937_P01,GRMZM2G122937_P03	TRUE	TRUE	aEYAEk	89%	n+304 (+304), K+304 (+304)	28.06	26.57
3491	seq=translation; coord=2:22990515..22995670:1; parent_transcript=GRMZM2G122937_T01; parent_gene=GRMZM2G122937	GRMZM2G122937_P01,GRMZM2G122937_P03	TRUE	TRUE	aLAIVEk	95%	n+304 (+304), K+304 (+304)	30.77	25.00
3492	seq=translation; coord=2:22990515..22995670:1; parent_transcript=GRMZM2G122937_T01; parent_gene=GRMZM2G122937	GRMZM2G122937_P01,GRMZM2G122937_P03	TRUE	TRUE	aNIEAELk	91%	n+304 (+304), K+304 (+304)	30.94	27.59
3493	seq=translation; coord=2:22990515..22995670:1; parent_transcript=GRMZM2G122937_T01; parent_gene=GRMZM2G122937	GRMZM2G122937_P01,GRMZM2G122937_P03	TRUE	TRUE	aWEENEk	95%	n+304 (+304), K+304 (+304)	40.26	25.00
3494	seq=translation; coord=2:22990515..22995670:1; parent_transcript=GRMZM2G122937_T01; parent_gene=GRMZM2G122937	GRMZM2G122937_P01,GRMZM2G122937_P03	TRUE	TRUE	eDVADDk	95%	n+304 (+304), K+304 (+304)	36.75	25.00
3495	seq=translation; coord=2:22990515..22995670:1; parent_transcript=GRMZM2G122937_T01; parent_gene=GRMZM2G122937	GRMZM2G122937_P01,GRMZM2G122937_P03	TRUE	TRUE	lIGcFGA	86%	n+304 (+304), Carbamidomethyl (+57)	25.47	25.00

3496	seq=translation; coord=2:22990515..22995670:1; parent_transcript=GRMZM2G122937_T01; parent_gene=GRMZM2G122937	GRMZM2G122937_P01,GRMZM2G122937_P03	TRUE	TRUE	vSAILSWENTk	95%	n+304 (+304), K+304 (+304)	39.45	26.12
3497	seq=translation; coord=5:210169906..210174618:-1; parent_transcript=GRMZM2G015401_T01; parent_gene=GRMZM2G015401	GRMZM2G015401_P01,GRMZM2G152827_P01	TRUE	TRUE	fASFETIVELIYk	95%	n+304 (+304), K+304 (+304)	35.76	25.12
3498	seq=translation; coord=5:210169906..210174618:-1; parent_transcript=GRMZM2G015401_T01; parent_gene=GRMZM2G015401	GRMZM2G015401_P01,GRMZM2G152827_P01	TRUE	TRUE	gATVGDAVk	94%	n+304 (+304), K+304 (+304)	32.21	26.23
3499	seq=translation; coord=5:210169906..210174618:-1; parent_transcript=GRMZM2G015401_T01; parent_gene=GRMZM2G015401	GRMZM2G015401_P01,GRMZM2G152827_P01	TRUE	TRUE	gLSDGLPk	92%	n+304 (+304), K+304 (+304)	29.08	25.00
3500	seq=translation; coord=5:210169906..210174618:-1; parent_transcript=GRMZM2G015401_T01; parent_gene=GRMZM2G015401	GRMZM2G015401_P01,GRMZM2G152827_P01	TRUE	TRUE	gWVPTLLGYSAQGAck	95%	n+304 (+304), Carbamidomethyl (+57), K+304 (+304)	51.92	26.03
3501	seq=translation; coord=5:210169906..210174618:-1; parent_transcript=GRMZM2G015401_T01; parent_gene=GRMZM2G015401	GRMZM2G015401_P01,GRMZM2G152827_P01	TRUE	TRUE	sEGVLGLYk	95%	n+304 (+304), K+304 (+304)	45.01	25.61
3502	seq=translation; coord=5:210169906..210174618:-1; parent_transcript=GRMZM2G015401_T01; parent_gene=GRMZM2G015401	GRMZM2G015401_P01,GRMZM2G152827_P01	TRUE	TRUE	sISSGFGILLk	95%	n+304 (+304), K+304 (+304)	42.14	25.00
3503	seq=translation; coord=7:127859191..127864034:-1; parent_transcript=GRMZM2G140614_T01; parent_gene=GRMZM2G140614	GRMZM2G140614_P01,GRMZM2G140614_P02	TRUE	TRUE	aAGEVLALQk	95%	n+304 (+304), K+304 (+304)	37.91	25.99
3504	seq=translation; coord=7:127859191..127864034:-1; parent_transcript=GRMZM2G140614_T01; parent_gene=GRMZM2G140614	GRMZM2G140614_P01,GRMZM2G140614_P02	TRUE	TRUE	dRPAGHDWELEPGVTcGDYLFGMLQGTR	95%	n+304 (+304), Carbamidomethyl (+57)	39.78	25.00
3505	seq=translation; coord=7:127859191..127864034:-1; parent_transcript=GRMZM2G140614_T01; parent_gene=GRMZM2G140614	GRMZM2G140614_P01,GRMZM2G140614_P02	TRUE	TRUE	eGVHNFFVTFIEVLR	95%	n+304 (+304)	35.89	26.11
3506	seq=translation; coord=7:127859191..127864034:-1; parent_transcript=GRMZM2G140614_T01; parent_gene=GRMZM2G140614	GRMZM2G140614_P01,GRMZM2G140614_P02	TRUE	TRUE	iLAFSQDVVSGk	95%	n+304 (+304), K+304 (+304)	37.99	25.79
3507	seq=translation; coord=7:127859191..127864034:-1; parent_transcript=GRMZM2G140614_T01; parent_gene=GRMZM2G140614	GRMZM2G140614_P01,GRMZM2G140614_P02	TRUE	TRUE	mGFTEDFLR	95%	n+304 (+304)	32.06	25.00
3508	seq=translation; coord=7:127859191..127864034:-1; parent_transcript=GRMZM2G140614_T01; parent_gene=GRMZM2G140614	GRMZM2G140614_P01,GRMZM2G140614_P02	TRUE	TRUE	tSEMSAVGLLPAALQGIDIk	95%	n+304 (+304), K+304 (+304)	67.94	25.00
3509	seq=translation; coord=1:217021624..217059348:1; parent_transcript=GRMZM2G090542_T01; parent_gene=GRMZM2G090542	GRMZM2G090542_P01	TRUE	TRUE	aVEVTEFFAGQVkPSFER	95%	n+304 (+304), K+304 (+304)	29.05	25.42
3510	seq=translation; coord=1:217021624..217059348:1; parent_transcript=GRMZM2G090542_T01; parent_gene=GRMZM2G090542	GRMZM2G090542_P01	TRUE	TRUE	fALDVAVk	91%	n+304 (+304), K+304 (+304)	25.52	25.00
3511	seq=translation; coord=1:217021624..217059348:1; parent_transcript=GRMZM2G090542_T01; parent_gene=GRMZM2G090542	GRMZM2G090542_P01	TRUE	TRUE	fHIFFEDGk	94%	n+304 (+304), K+304 (+304)	28.97	25.77
3512	seq=translation; coord=1:217021624..217059348:1; parent_transcript=GRMZM2G090542_T01; parent_gene=GRMZM2G090542	GRMZM2G090542_P01	TRUE	TRUE	fLVLNAAELDVDR	95%	n+304 (+304)	67.04	25.00
3513	seq=translation; coord=1:217021624..217059348:1; parent_transcript=GRMZM2G090542_T01; parent_gene=GRMZM2G090542	GRMZM2G090542_P01	TRUE	TRUE	lTLEVPSDLVALSNMPVAk	95%	n+304 (+304), K+304 (+304)	29.06	25.00

3514	seq=translation; coord=1:217021624..217059348:1; parent_transcript=GRMZM2G090542_T01; parent_gene=GRMZM2G090542	GRMZM2G090542_P01	TRUE	TRUE	nMAVTQFEAADAR	95%	n+304 (+304)	36.20	25.00
3515	seq=translation; coord=1:217021624..217059348:1; parent_transcript=GRMZM2G090542_T01; parent_gene=GRMZM2G090542	GRMZM2G090542_P01	TRUE	TRUE	sSSLISDFIESIVPR	95%	n+304 (+304)	62.17	25.15
3516	seq=translation; coord=3:171894936..171898201:1; parent_transcript=GRMZM2G167637_T01; parent_gene=GRMZM2G167637	GRMZM2G167637_P01	TRUE	TRUE	dLTIANTAGPDAHQAVAFR	95%	n+304 (+304)	80.26	25.00
3517	seq=translation; coord=3:171894936..171898201:1; parent_transcript=GRMZM2G167637_T01; parent_gene=GRMZM2G167637	GRMZM2G167637_P01	TRUE	TRUE	eAVAAAPDYGDGAFVVHVk	95%	n+304 (+304), K+304 (+304)	80.40	25.09
3518	seq=translation; coord=3:171894936..171898201:1; parent_transcript=GRMZM2G167637_T01; parent_gene=GRMZM2G167637	GRMZM2G167637_P01	TRUE	TRUE	eTVSVPWEk	93%	n+304 (+304), K+304 (+304)	30.59	25.58
3519	seq=translation; coord=3:171894936..171898201:1; parent_transcript=GRMZM2G167637_T01; parent_gene=GRMZM2G167637	GRMZM2G167637_P01	TRUE	TRUE	lPPAVSTAk	95%	n+304 (+304), K+304 (+304)	39.57	25.00
3520	seq=translation; coord=3:171894936..171898201:1; parent_transcript=GRMZM2G167637_T01; parent_gene=GRMZM2G167637	GRMZM2G167637_P01	TRUE	TRUE	tDPAQPTGIVLSR	94%	n+304 (+304)	32.59	26.43
3521	seq=translation; coord=3:171894936..171898201:1; parent_transcript=GRMZM2G167637_T01; parent_gene=GRMZM2G167637	GRMZM2G167637_P01	TRUE	TRUE	tNVVLVGDGMGk	87%	n+304 (+304), K+304 (+304)	26.94	26.43
3522	seq=translation; coord=3:171894936..171898201:1; parent_transcript=GRMZM2G167637_T01; parent_gene=GRMZM2G167637	GRMZM2G167637_P01	TRUE	TRUE	vHVDAYSVASFIQGHEWIPR	95%	n+304 (+304)	26.98	25.53
3523	seq=translation; coord=1:267609498..267614894:-1; parent_transcript=GRMZM2G010054_T01; parent_gene=GRMZM2G010054	GRMZM2G010054_P01,GRMZM2G149406_P01	TRUE	TRUE	dVScPYcGSR	88%	n+304 (+304), Carbamidomethyl (+57), Carbamidomethyl (+57)	26.38	25.00
3524	seq=translation; coord=1:267609498..267614894:-1; parent_transcript=GRMZM2G010054_T01; parent_gene=GRMZM2G010054	GRMZM2G010054_P01,GRMZM2G149406_P01	TRUE	TRUE	gFPEVALHFVk	95%	n+304 (+304), K+304 (+304)	49.60	26.19
3525	seq=translation; coord=1:267609498..267614894:-1; parent_transcript=GRMZM2G010054_T01; parent_gene=GRMZM2G010054	GRMZM2G010054_P01,GRMZM2G149406_P01	TRUE	TRUE	gIFEGGLDATGR	95%	n+304 (+304)	34.73	25.00
3526	seq=translation; coord=1:267609498..267614894:-1; parent_transcript=GRMZM2G010054_T01; parent_gene=GRMZM2G010054	GRMZM2G010054_P01,GRMZM2G149406_P01	TRUE	TRUE	gWNESASPNVR	89%	n+304 (+304)	26.69	25.00
3527	seq=translation; coord=1:267609498..267614894:-1; parent_transcript=GRMZM2G010054_T01; parent_gene=GRMZM2G010054	GRMZM2G010054_P01,GRMZM2G149406_P01	TRUE	TRUE	lVLASAMALcFk	95%	n+304 (+304), Carbamidomethyl (+57), K+304 (+304)	33.93	25.59
3528	seq=translation; coord=1:267609498..267614894:-1; parent_transcript=GRMZM2G010054_T01; parent_gene=GRMZM2G010054	GRMZM2G010054_P01,GRMZM2G149406_P01	TRUE	TRUE	sALFVAPTPGIPVSQIWTQR	95%	n+304 (+304)	41.55	25.00
3529	seq=translation; coord=1:267609498..267614894:-1; parent_transcript=GRMZM2G010054_T01; parent_gene=GRMZM2G010054	GRMZM2G010054_P01,GRMZM2G149406_P01	TRUE	TRUE	tVSPADDILR	95%	n+304 (+304)	39.85	26.62
3530	seq=translation; coord=5:202811453..202814808:1; parent_transcript=GRMZM2G180930_T02; parent_gene=GRMZM2G180930	GRMZM2G180930_P02,GRMZM2G180930_P03	TRUE	TRUE	gGPTSVVIYDIHALQER	95%	n+304 (+304)	46.90	25.33
3531	seq=translation; coord=5:202811453..202814808:1; parent_transcript=GRMZM2G180930_T02; parent_gene=GRMZM2G180930	GRMZM2G180930_P02,GRMZM2G180930_P03	TRUE	TRUE	hVVIVDDLVQSGGTLR	95%	n+304 (+304)	47.82	26.34

3532	seq=translation; coord=5:202811453..202814808:1; parent_transcript=GRMZM2G180930_T02; parent_gene=GRMZM2G180930	GRMZM2G180930_P02,GRMZM2G180930_P03	TRUE	TRUE	sFDDGFPNLFINk	95%	n+304 (+304), K+304 (+304)	30.31	25.51
3533	seq=translation; coord=5:202811453..202814808:1; parent_transcript=GRMZM2G180930_T02; parent_gene=GRMZM2G180930	GRMZM2G180930_P02,GRMZM2G180930_P03	TRUE	TRUE	vEEEGDVATAFTLAR	95%	n+304 (+304)	56.17	25.00
3534	seq=translation; coord=2:233428481..233430995:-1; parent_transcript=GRMZM2G018607_T01; parent_gene=GRMZM2G018607	GRMZM2G018607_P01	TRUE	TRUE	sALEAVVAADEELTALR	95%	n+304 (+304)	40.60	26.21
3535	seq=translation; coord=2:233428481..233430995:-1; parent_transcript=GRMZM2G018607_T01; parent_gene=GRMZM2G018607	GRMZM2G018607_P01	TRUE	TRUE	vPGSSADDGAVDDNVR	95%	n+304 (+304)	49.15	25.00
3536	seq=translation; coord=6:56216948..56219146:-1; parent_transcript=GRMZM2G385287_T01; parent_gene=GRMZM2G385287	GRMZM2G385287_P01	TRUE	TRUE	hLNLDFQLQEGGR	95%	n+304 (+304)	58.07	25.22
3537	seq=translation; coord=7:150418464..150423441:1; parent_transcript=GRMZM2G075719_T01; parent_gene=GRMZM2G075719	GRMZM2G075719_P01,GRMZM2G075719_P02	TRUE	TRUE	dLGVMFIETSAk	95%	n+304 (+304), K+304 (+304)	42.46	26.01
3538	seq=translation; coord=7:150418464..150423441:1; parent_transcript=GRMZM2G075719_T01; parent_gene=GRMZM2G075719	GRMZM2G075719_P01,GRMZM2G075719_P02	TRUE	TRUE	dSSVAVIVFDVASR	95%	n+304 (+304)	38.31	25.85
3539	seq=translation; coord=7:150418464..150423441:1; parent_transcript=GRMZM2G075719_T01; parent_gene=GRMZM2G075719	GRMZM2G075719_P01,GRMZM2G075719_P02	TRUE	TRUE	iAAALPGMETLSSAk	95%	n+304 (+304), K+304 (+304)	39.73	25.00
3540	seq=translation; coord=7:150418464..150423441:1; parent_transcript=GRMZM2G075719_T01; parent_gene=GRMZM2G075719	GRMZM2G075719_P01,GRMZM2G075719_P02	TRUE	TRUE	qEDMVDVNLR	93%	n+304 (+304)	30.12	25.00
3541	seq=translation; coord=7:150418464..150423441:1; parent_transcript=GRMZM2G075719_T01; parent_gene=GRMZM2G075719	GRMZM2G075719_P01,GRMZM2G075719_P02	TRUE	TRUE	wIDEVR	89%	n+304 (+304)	26.82	25.13
3542	seq=translation; coord=8:173702854..173715423:1; parent_transcript=GRMZM2G110714_T01; parent_gene=GRMZM2G110714	GRMZM2G110714_P01,GRMZM2G110714_P02	TRUE	TRUE	fLDILQDLHGEDIR	95%	n+304 (+304)	40.55	25.77
3543	seq=translation; coord=8:173702854..173715423:1; parent_transcript=GRMZM2G110714_T01; parent_gene=GRMZM2G110714	GRMZM2G110714_P01,GRMZM2G110714_P02	TRUE	TRUE	hTDVMDAITEYLGIGSYR	95%	n+304 (+304)	56.29	25.00
3544	seq=translation; coord=8:173702854..173715423:1; parent_transcript=GRMZM2G110714_T01; parent_gene=GRMZM2G110714	GRMZM2G110714_P01,GRMZM2G110714_P02	TRUE	TRUE	lLVSEDLWALGAR	95%	n+304 (+304)	44.85	25.24
3545	seq=translation; coord=8:173702854..173715423:1; parent_transcript=GRMZM2G110714_T01; parent_gene=GRMZM2G110714	GRMZM2G110714_P01,GRMZM2G110714_P02	TRUE	TRUE	lSDDDkLVEYDALLLDR	95%	n+304 (+304), K+304 (+304)	30.80	25.55
3546	seq=translation; coord=8:173702854..173715423:1; parent_transcript=GRMZM2G110714_T01; parent_gene=GRMZM2G110714	GRMZM2G110714_P01,GRMZM2G110714_P02	TRUE	TRUE	sVADGSLLDFLR	95%	n+304 (+304)	55.72	25.95
3547	seq=translation; coord=6:138057377..138059762:-1; parent_transcript=GRMZM2G078985_T01; parent_gene=GRMZM2G078985	GRMZM2G078985_P01,GRMZM2G156673_P01, GRMZM2G156673_P02	TRUE	TRUE	hTLEIIHLLTDANPIQVVVDAIINSGPR	95%	n+304 (+304)	37.64	25.00
3548	seq=translation; coord=6:138057377..138059762:-1; parent_transcript=GRMZM2G078985_T01; parent_gene=GRMZM2G078985	GRMZM2G078985_P01,GRMZM2G156673_P01, GRMZM2G156673_P02	TRUE	TRUE	qAVDISPLR	91%	n+304 (+304)	30.46	27.16
3549	seq=translation; coord=6:138057377..138059762:-1; parent_transcript=GRMZM2G078985_T01; parent_gene=GRMZM2G078985	GRMZM2G078985_P01,GRMZM2G156673_P01, GRMZM2G156673_P02	TRUE	TRUE	tIAEcLADELINAAk	95%	n+304 (+304), Carbamidomethyl (+57), K+304 (+304)	51.63	26.78

3550	seq=translation; coord=6:138057377..138059762:-1; parent_transcript=GRMZM2G078985_T01; parent_gene=GRMZM2G078985	GRMZM2G078985_P01,GRMZM2G156673_P01, GRMZM2G156673_P02	TRUE	TRUE	vNQAIYLLTTGAR	95%	n+304 (+304)	36.23	25.00
3551	seq=translation; coord=6:108069110..108070157:1; parent_transcript=GRMZM2G073150_T01; parent_gene=GRMZM2G073150	GRMZM2G073150_P01	TRUE	TRUE	qAEVSSVPAYGRPQYGGPR	88%	Pyro-cmC (-17), n+304 (+304)	25.98	25.00
3552	seq=translation; coord=6:108069110..108070157:1; parent_transcript=GRMZM2G073150_T01; parent_gene=GRMZM2G073150	GRMZM2G073150_P01	TRUE	TRUE	sALEVEEIR	95%	n+304 (+304)	39.27	26.14
3553	seq=translation; coord=6:108069110..108070157:1; parent_transcript=GRMZM2G073150_T01; parent_gene=GRMZM2G073150	GRMZM2G073150_P01	TRUE	TRUE	vDkETMEMLAALGMADLPGVER	95%	n+304 (+304), K+304 (+304)	45.62	25.00
3554	seq=translation; coord=6:108069110..108070157:1; parent_transcript=GRMZM2G073150_T01; parent_gene=GRMZM2G073150	GRMZM2G073150_P01	TRUE	TRUE	vLEEVSILPSk	95%	n+304 (+304), K+304 (+304)	33.18	25.05
3555	seq=translation; coord=8:63271714..63275991:-1; parent_transcript=GRMZM2G168681_T01; parent_gene=GRMZM2G168681	GRMZM2G168681_P01	TRUE	TRUE	gkPYYADLLDLFNEVEFk	95%	n+304 (+304), K+304 (+304), K+304 (+304)	26.14	25.08
3556	seq=translation; coord=8:63271714..63275991:-1; parent_transcript=GRMZM2G168681_T01; parent_gene=GRMZM2G168681	GRMZM2G168681_P01	TRUE	TRUE	lIADIEAQPSIAVQk	95%	n+304 (+304), K+304 (+304)	54.64	25.00
3557	seq=translation; coord=8:63271714..63275991:-1; parent_transcript=GRMZM2G168681_T01; parent_gene=GRMZM2G168681	GRMZM2G168681_P01	TRUE	TRUE	mVDYNVLGGk	95%	n+304 (+304), K+304 (+304)	36.08	26.32
3558	seq=translation; coord=8:63271714..63275991:-1; parent_transcript=GRMZM2G168681_T01; parent_gene=GRMZM2G168681	GRMZM2G168681_P01	TRUE	TRUE	tLkEELLTDPAFEFTEESR	95%	n+304 (+304), K+304 (+304)	43.92	25.53
3559	seq=translation; coord=8:63271714..63275991:-1; parent_transcript=GRMZM2G168681_T01; parent_gene=GRMZM2G168681	GRMZM2G168681_P01	TRUE	TRUE	vPQVGLIAANDGIILR	95%	n+304 (+304)	70.96	25.00
3560	seq=translation; coord=3:186781963..186783499:-1; parent_transcript=GRMZM2G460860_T01; parent_gene=GRMZM2G460860	GRMZM2G460860_P01	TRUE	TRUE	gGILGLLLAGWAAR	95%	n+304 (+304)	44.39	25.00
3561	seq=translation; coord=3:186781963..186783499:-1; parent_transcript=GRMZM2G460860_T01; parent_gene=GRMZM2G460860	GRMZM2G460860_P01	TRUE	TRUE	lIAVGNAAADAINEAR	95%	n+304 (+304)	65.81	26.01
3562	seq=translation; coord=3:186781963..186783499:-1; parent_transcript=GRMZM2G460860_T01; parent_gene=GRMZM2G460860	GRMZM2G460860_P01	TRUE	TRUE	vSVVALR	95%	n+304 (+304)	36.13	25.00
3563	seq=translation; coord=8:69592559..69594672:1; parent_transcript=GRMZM2G001514_T01; parent_gene=GRMZM2G001514	GRMZM2G001514_P01	TRUE	TRUE	dDQFSEFk	95%	n+304 (+304), K+304 (+304)	38.24	25.00
3564	seq=translation; coord=8:69592559..69594672:1; parent_transcript=GRMZM2G001514_T01; parent_gene=GRMZM2G001514	GRMZM2G001514_P01	TRUE	TRUE	fYSLSMLGTLDGk	95%	n+304 (+304), K+304 (+304)	33.63	25.49
3565	seq=translation; coord=8:69592559..69594672:1; parent_transcript=GRMZM2G001514_T01; parent_gene=GRMZM2G001514	GRMZM2G001514_P01	TRUE	TRUE	fYSLSmLGTLDGk	95%	n+304 (+304), Oxidation (+16), K+304 (+304)	36.76	26.19
3566	seq=translation; coord=6:142310594..142315324:-1; parent_transcript=GRMZM2G137535_T01; parent_gene=GRMZM2G137535	GRMZM2G137535_P01,GRMZM2G137535_P02	TRUE	TRUE	eSGVEQNWGLFYPNMQHVyPISF	95%	n+304 (+304), iTRAQ8plex (+304)	33.92	25.00
3567	seq=translation; coord=6:142310594..142315324:-1; parent_transcript=GRMZM2G137535_T01; parent_gene=GRMZM2G137535	GRMZM2G137535_P01,GRMZM2G137535_P02	TRUE	TRUE	gYMGPVLQFLAR	95%	n+304 (+304)	37.82	25.00

3568	seq=translation; coord=6:142310594..142315324:-1; parent_transcript=GRMZM2G137535_T01; parent_gene=GRMZM2G137535	GRMZM2G137535_P01,GRMZM2G137535_P02	TRUE	TRUE	hPGAIETYLFSMFNENQk	95%	n+304 (+304), K+304 (+304)	44.06	25.00
3569	seq=translation; coord=5:169817842..169818381:1; parent_transcript=AC233850.1_FGT002; parent_gene=AC233850.1_FG002	AC233850.1_FGP002	TRUE	TRUE	lYDAAEMAFAEAYDGINR	95%	n+304 (+304)	56.04	25.00
3570	seq=translation; coord=5:169817842..169818381:1; parent_transcript=AC233850.1_FGT002; parent_gene=AC233850.1_FG002	AC233850.1_FGP002	TRUE	TRUE	lyDAAEMAFAEAYDGINR	95%	n+304 (+304), iTRAQ8plex (+304)	72.37	25.00
3571	seq=translation; coord=5:169817842..169818381:1; parent_transcript=AC233850.1_FGT002; parent_gene=AC233850.1_FG002	AC233850.1_FGP002	TRUE	TRUE	lYDAAEMAFAEAyDGINR	95%	n+304 (+304), iTRAQ8plex (+304)	46.59	25.00
3572	seq=translation; coord=5:169817842..169818381:1; parent_transcript=AC233850.1_FGT002; parent_gene=AC233850.1_FG002	AC233850.1_FGP002	TRUE	TRUE	vAADVGVATAGDAVYDIk	95%	n+304 (+304), K+304 (+304)	95.70	25.87
3573	seq=translation; coord=6:69568276..69571793:-1; parent_transcript=GRMZM2G152775_T04; parent_gene=GRMZM2G152775	GRMZM2G152775_P04	TRUE	TRUE	eGMSQEEAEk	95%	n+304 (+304), K+304 (+304)	41.62	25.00
3574	seq=translation; coord=6:69568276..69571793:-1; parent_transcript=GRMZM2G152775_T04; parent_gene=GRMZM2G152775	GRMZM2G152775_P04	TRUE	TRUE	iTQLTDNVYVcR	95%	n+304 (+304), Carbamidomethyl (+57)	41.79	25.00
3575	seq=translation; coord=6:69568276..69571793:-1; parent_transcript=GRMZM2G152775_T04; parent_gene=GRMZM2G152775	GRMZM2G152775_P04	TRUE	TRUE	lLAYQNk	91%	n+304 (+304), K+304 (+304)	27.77	25.00
3576	seq=translation; coord=6:69568276..69571793:-1; parent_transcript=GRMZM2G152775_T04; parent_gene=GRMZM2G152775	GRMZM2G152775_P04	TRUE	TRUE	nMLQAGMIVGGWDk	95%	n+304 (+304), K+304 (+304)	47.02	25.61
3577	seq=translation; coord=6:69568276..69571793:-1; parent_transcript=GRMZM2G152775_T04; parent_gene=GRMZM2G152775	GRMZM2G152775_P04	TRUE	TRUE	sGSAADTQVISDYVR	95%	n+304 (+304)	33.85	25.00
3578	seq=translation; coord=4:193735481..193760758:1; parent_transcript=AC215244.3_FGT002; parent_gene=AC215244.3_FG002	AC215244.3_FGP002	TRUE	TRUE	qGLPPAGFSNPFDFGSMQSLLNDPSIk	95%	n+304 (+304), K+304 (+304)	48.99	25.00
3579	seq=translation; coord=4:193735481..193760758:1; parent_transcript=AC215244.3_FGT002; parent_gene=AC215244.3_FG002	AC215244.3_FGP002	TRUE	TRUE	vMENPQFMTMAER	95%	n+304 (+304)	57.81	25.00
3580	seq=translation; coord=4:193735481..193760758:1; parent_transcript=AC215244.3_FGT002; parent_gene=AC215244.3_FG002	AC215244.3_FGP002	TRUE	TRUE	yWNDPDTLQk	95%	n+304 (+304), K+304 (+304)	38.72	25.00
3581	seq=translation; coord=2:2326285..2329820:1; parent_transcript=GRMZM2G077541_T01; parent_gene=GRMZM2G077541	GRMZM2G077541_P01,GRMZM2G077541_P02	TRUE	TRUE	aPEYLAMDEk	93%	n+304 (+304), K+304 (+304)	27.62	25.00
3582	seq=translation; coord=2:2326285..2329820:1; parent_transcript=GRMZM2G077541_T01; parent_gene=GRMZM2G077541	GRMZM2G077541_P01,GRMZM2G077541_P02	TRUE	TRUE	gHNFTLNASFDEITASEYDGLVIPGGR	95%	n+304 (+304)	42.09	25.00
3583	seq=translation; coord=2:2326285..2329820:1; parent_transcript=GRMZM2G077541_T01; parent_gene=GRMZM2G077541	GRMZM2G077541_P01,GRMZM2G077541_P02	TRUE	TRUE	gHNFTLNASFDEITASEyDGLVIPGGR	95%	n+304 (+304), iTRAQ8plex (+304)	27.01	25.00
3584	seq=translation; coord=2:2326285..2329820:1; parent_transcript=GRMZM2G077541_T01; parent_gene=GRMZM2G077541	GRMZM2G077541_P01,GRMZM2G077541_P02	TRUE	TRUE	lNVLLGGGTWLEPDPIHR	95%	n+304 (+304)	74.32	25.89
3585	seq=translation; coord=2:2326285..2329820:1; parent_transcript=GRMZM2G077541_T01; parent_gene=GRMZM2G077541	GRMZM2G077541_P01,GRMZM2G077541_P02	TRUE	TRUE	wEEPDTMAk	95%	n+304 (+304), K+304 (+304)	32.95	25.00

3586	seq=translation; coord=7:41731651..41795285:-1; parent_transcript=GRMZM2G069676_T02; parent_gene=GRMZM2G069676	GRMZM2G069676_P02,GRMZM2G069676_P03	TRUE	TRUE	aDLGIIFDTDVDR	95%	n+304 (+304)	32.77	25.00
3587	seq=translation; coord=7:41731651..41795285:-1; parent_transcript=GRMZM2G069676_T02; parent_gene=GRMZM2G069676	GRMZM2G069676_P02,GRMZM2G069676_P03	TRUE	TRUE	aIAAAFAAWLLNk	95%	n+304 (+304), K+304 (+304)	31.47	25.00
3588	seq=translation; coord=7:41731651..41795285:-1; parent_transcript=GRMZM2G069676_T02; parent_gene=GRMZM2G069676	GRMZM2G069676_P02,GRMZM2G069676_P03	TRUE	TRUE	gVVTNVDYMSIYASDLVQAVR	95%	n+304 (+304)	30.47	25.29
3589	seq=translation; coord=7:41731651..41795285:-1; parent_transcript=GRMZM2G069676_T02; parent_gene=GRMZM2G069676	GRMZM2G069676_P02,GRMZM2G069676_P03	TRUE	TRUE	lGLAVLAAVNEFPALDVTALNk	95%	n+304 (+304), K+304 (+304)	31.70	25.00
3590	seq=translation; coord=6:165533613..165537241:1; parent_transcript=GRMZM5G829778_T01; parent_gene=GRMZM5G829778	GRMZM5G829778_P01	TRUE	TRUE	lIFPFVDLDIk	95%	n+304 (+304), K+304 (+304)	38.92	25.00
3591	seq=translation; coord=6:165533613..165537241:1; parent_transcript=GRMZM5G829778_T01; parent_gene=GRMZM5G829778	GRMZM5G829778_P01	TRUE	TRUE	sHYLNTEEFIDAVADELR	95%	n+304 (+304)	44.08	25.00
3592	seq=translation; coord=6:165533613..165537241:1; parent_transcript=GRMZM5G829778_T01; parent_gene=GRMZM5G829778	GRMZM5G829778_P01	TRUE	TRUE	sHyLNTEEFIDAVADELR	95%	n+304 (+304), iTRAQ8plex (+304)	27.18	25.00
3593	seq=translation; coord=6:165533613..165537241:1; parent_transcript=GRMZM5G829778_T01; parent_gene=GRMZM5G829778	GRMZM5G829778_P01	TRUE	TRUE	yFDLGLPHR	95%	n+304 (+304)	48.44	25.00
3594	seq=translation; coord=10:106428641..106433063:1; parent_transcript=GRMZM2G139407_T01; parent_gene=GRMZM2G139407	GRMZM2G139407_P01,GRMZM2G139407_P02	TRUE	TRUE	eFVQSFFLAPQEk	95%	n+304 (+304), K+304 (+304)	29.41	25.53
3595	seq=translation; coord=10:106428641..106433063:1; parent_transcript=GRMZM2G139407_T01; parent_gene=GRMZM2G139407	GRMZM2G139407_P01,GRMZM2G139407_P02	TRUE	TRUE	gYFVLNDILR	95%	n+304 (+304)	42.19	26.43
3596	seq=translation; coord=10:106428641..106433063:1; parent_transcript=GRMZM2G139407_T01; parent_gene=GRMZM2G139407	GRMZM2G139407_P01,GRMZM2G139407_P02	TRUE	TRUE	lGRPSGTGDDGMETVTSMDAINDk	95%	n+304 (+304), K+304 (+304)	32.06	25.00
3597	seq=translation; coord=10:106428641..106433063:1; parent_transcript=GRMZM2G139407_T01; parent_gene=GRMZM2G139407	GRMZM2G139407_P01,GRMZM2G139407_P02	TRUE	TRUE	sLPLNATPQQLEEEFk	90%	n+304 (+304), K+304 (+304)	27.45	25.93
3598	seq=translation; coord=10:106428641..106433063:1; parent_transcript=GRMZM2G139407_T01; parent_gene=GRMZM2G139407	GRMZM2G139407_P01,GRMZM2G139407_P02	TRUE	TRUE	sLPLNATPQQLEEEFkR	90%	n+304 (+304), K+304 (+304)	26.73	26.32
3599	seq=translation; coord=4:83505768..83508091:1; parent_transcript=GRMZM2G068455_T01; parent_gene=GRMZM2G068455	GRMZM2G068455_P01,GRMZM2G068455_P02, GRMZM2G068455_P03	TRUE	TRUE	gIAFVNk	90%	n+304 (+304), K+304 (+304)	27.82	26.20
3600	seq=translation; coord=4:83505768..83508091:1; parent_transcript=GRMZM2G068455_T01; parent_gene=GRMZM2G068455	GRMZM2G068455_P01,GRMZM2G068455_P02, GRMZM2G068455_P03	TRUE	TRUE	vAVLGAAGGIGQPLGLLVk	95%	n+304 (+304), K+304 (+304)	47.56	25.00
3601	seq=translation; coord=3:7404885..7413183:1; parent_transcript=GRMZM2G093050_T01; parent_gene=GRMZM2G093050	GRMZM2G093050_P01,GRMZM2G093050_P02	TRUE	TRUE	aPDSAPVAQPPR	95%	n+304 (+304)	46.93	25.15
3602	seq=translation; coord=3:7404885..7413183:1; parent_transcript=GRMZM2G093050_T01; parent_gene=GRMZM2G093050	GRMZM2G093050_P01,GRMZM2G093050_P02	TRUE	TRUE	dLQLIASSVLLAALSVSPYDkk	95%	n+304 (+304), K+304 (+304), K+304 (+304)	36.16	25.00
3603	seq=translation; coord=3:7404885..7413183:1; parent_transcript=GRMZM2G093050_T01; parent_gene=GRMZM2G093050	GRMZM2G093050_P01,GRMZM2G093050_P02	TRUE	TRUE	dLYNLLEHEFLPLDLASk	94%	n+304 (+304), K+304 (+304)	26.81	26.48

3604	seq=translation; coord=3:7404885..7413183:1; parent_transcript=GRMZM2G093050_T01; parent_gene=GRMZM2G093050	GRMZM2G093050_P01,GRMZM2G093050_P02	TRUE	TRUE	dRPDLTAPESLQLYLDTR	95%	n+304 (+304)	77.43	25.13
3605	seq=translation; coord=3:7404885..7413183:1; parent_transcript=GRMZM2G093050_T01; parent_gene=GRMZM2G093050	GRMZM2G093050_P01,GRMZM2G093050_P02	TRUE	TRUE	qSALQALHDLITSk	95%	Pyro-cmC (-17), n+304 (+304), K+304 (+304)	28.92	25.58
3606	seq=translation; coord=3:7404885..7413183:1; parent_transcript=GRMZM2G093050_T01; parent_gene=GRMZM2G093050	GRMZM2G093050_P01,GRMZM2G093050_P02	TRUE	TRUE	tMDYLER	94%	n+304 (+304)	30.58	25.00
3607	seq=translation; coord=1:215329050..215330533:1; parent_transcript=GRMZM2G106133_T02; parent_gene=GRMZM2G106133	GRMZM2G106133_P02	TRUE	TRUE	aGAPDAPPk	94%	n+304 (+304), K+304 (+304)	32.27	26.65
3608	seq=translation; coord=1:215329050..215330533:1; parent_transcript=GRMZM2G106133_T02; parent_gene=GRMZM2G106133	GRMZM2G106133_P02	TRUE	TRUE	dPNAPAPAPk	95%	n+304 (+304), K+304 (+304)	35.51	26.25
3609	seq=translation; coord=1:215329050..215330533:1; parent_transcript=GRMZM2G106133_T02; parent_gene=GRMZM2G106133	GRMZM2G106133_P02	TRUE	TRUE	eSGELVFLk	95%	n+304 (+304), K+304 (+304)	29.27	25.04
3610	seq=translation; coord=1:215329050..215330533:1; parent_transcript=GRMZM2G106133_T02; parent_gene=GRMZM2G106133	GRMZM2G106133_P02	TRUE	TRUE	sPLEAAVk	95%	n+304 (+304), K+304 (+304)	37.67	25.00
3611	seq=translation; coord=1:215329050..215330533:1; parent_transcript=GRMZM2G106133_T02; parent_gene=GRMZM2G106133	GRMZM2G106133_P02	TRUE	TRUE	vRPAVPSETAAA	88%	n+304 (+304)	27.73	26.50
3612	seq=translation; coord=5:215493131..215497186:1; parent_transcript=GRMZM5G824600_T03; parent_gene=GRMZM5G824600	GRMZM5G824600_P03	TRUE	TRUE	gTAFGGFk	90%	n+304 (+304), K+304 (+304)	27.14	25.00
3613	seq=translation; coord=5:215493131..215497186:1; parent_transcript=GRMZM5G824600_T03; parent_gene=GRMZM5G824600	GRMZM5G824600_P03	TRUE	TRUE	gWGTSVIVGVAASGQEIATRPFQLVTGR	95%	n+304 (+304)	28.12	25.00
3614	seq=translation; coord=5:215493131..215497186:1; parent_transcript=GRMZM5G824600_T03; parent_gene=GRMZM5G824600	GRMZM5G824600_P03	TRUE	TRUE	nFGVTEFVNPk	95%	n+304 (+304), K+304 (+304)	57.90	25.93
3615	seq=translation; coord=5:215493131..215497186:1; parent_transcript=GRMZM5G824600_T03; parent_gene=GRMZM5G824600	GRMZM5G824600_P03	TRUE	TRUE	vIGIDIDNk	95%	n+304 (+304), K+304 (+304)	34.07	27.23
3616	seq=translation; coord=7:46478537..46482054:1; parent_transcript=GRMZM2G011129_T01; parent_gene=GRMZM2G011129	GRMZM2G011129_P01	TRUE	TRUE	gFGFVTMASQDELDDAIAALDGQSLDGR	95%	n+304 (+304)	36.92	25.00
3617	seq=translation; coord=7:46478537..46482054:1; parent_transcript=GRMZM2G011129_T01; parent_gene=GRMZM2G011129	GRMZM2G011129_P01	TRUE	TRUE	gFGFVTMSTVEEAEk	95%	n+304 (+304), K+304 (+304)	36.44	25.25
3618	seq=translation; coord=7:46478537..46482054:1; parent_transcript=GRMZM2G011129_T01; parent_gene=GRMZM2G011129	GRMZM2G011129_P01	TRUE	TRUE	iYVGNLPWQVDDSR	95%	n+304 (+304)	42.81	25.00
3619	seq=translation; coord=7:46478537..46482054:1; parent_transcript=GRMZM2G011129_T01; parent_gene=GRMZM2G011129	GRMZM2G011129_P01	TRUE	TRUE	lAQLFDQAGVVEVAEVIyNR	95%	n+304 (+304), iTRAQ8plex (+304)	43.77	25.00
3620	seq=translation; coord=7:46478537..46482054:1; parent_transcript=GRMZM2G011129_T01; parent_gene=GRMZM2G011129	GRMZM2G011129_P01	TRUE	TRUE	lVELFSEHGk	95%	n+304 (+304), K+304 (+304)	43.95	26.33
3621	seq=translation; coord=7:46478537..46482054:1; parent_transcript=GRMZM2G011129_T01; parent_gene=GRMZM2G011129	GRMZM2G011129_P01	TRUE	TRUE	vYVGNLPYDVDSER	95%	n+304 (+304)	66.99	25.00

3622	seq=translation; coord=1:10991438..10998924:-1; parent_transcript=GRMZM2G056870_T01; parent_gene=GRMZM2G056870	GRMZM2G056870_P01	TRUE	TRUE	eLTLQEAETIALSILk	95%	n+304 (+304), K+304 (+304)	41.09	25.00
3623	seq=translation; coord=3:223089120..223091094:-1; parent_transcript=GRMZM2G042008_T01; parent_gene=GRMZM2G042008	GRMZM2G042008_P01	TRUE	TRUE	gGLSFPTQISR	95%	n+304 (+304)	37.35	25.00
3624	seq=translation; coord=3:223089120..223091094:-1; parent_transcript=GRMZM2G042008_T01; parent_gene=GRMZM2G042008	GRMZM2G042008_P01	TRUE	TRUE	gGVIVDSGTSVTR	95%	n+304 (+304)	53.21	25.00
3625	seq=translation; coord=3:223089120..223091094:-1; parent_transcript=GRMZM2G042008_T01; parent_gene=GRMZM2G042008	GRMZM2G042008_P01	TRUE	TRUE	vALGcGHDNEGLFVAAAGLLGLGR	95%	n+304 (+304), Carbamidomethyl (+57)	55.80	25.22
3626	seq=translation; coord=3:190774183..190777585:1; parent_transcript=GRMZM2G062373_T01; parent_gene=GRMZM2G062373	GRMZM2G062373_P01,GRMZM2G062373_P02	TRUE	TRUE	aQDLAQPLVDAQIPFk	94%	n+304 (+304), K+304 (+304)	31.45	25.55
3627	seq=translation; coord=3:190774183..190777585:1; parent_transcript=GRMZM2G062373_T01; parent_gene=GRMZM2G062373	GRMZM2G062373_P01,GRMZM2G062373_P02	TRUE	TRUE	lSTSADAPASVR	92%	n+304 (+304)	29.72	25.90
3628	seq=translation; coord=9:136867378..136869080:1; parent_transcript=GRMZM2G030293_T01; parent_gene=GRMZM2G030293	GRMZM2G030293_P01,GRMZM2G030293_P02, GRMZM2G124143_P01,GRMZM2G124143_P02, GRMZM2G124143_P03,GRMZM2G455828_P01, GRMZM2G455828_P02,GRMZM2G455828_P03, GRMZM2G455828_P04	TRUE	TRUE	eGDILTLLESER	95%	n+304 (+304)	79.55	25.00
3629	seq=translation; coord=7:148082947..148083258:1; parent_transcript=AC196961.2_FGT003; parent_gene=AC196961.2_FG003	AC196961.2_FGP003,AC212565.3_FGP001,AC233865.1_FGP001,GRMZM2G016232_P01,GRMZM2G016232_P02,GRMZM2G063896_P01,GRMZM2G063896_P02,GRMZM2G072855_P01,GRMZM2G073275_P01,GRMZM2G084195_P01,GRMZM2G143780_P01,GRMZM2G143780_P02,GRMZM2G143780_P03,GRMZM2G149178_P01,GRMZM2G181153_P01,GRMZM2G332838_P01,GRMZM2G332838_P02,GRMZM2G349651_P01,GRMZM2G349651_P02,GRMZM2G421279_P01,GRMZM2G479684_P01	TRUE	TRUE	dAVTYTEHAR	95%	n+304 (+304)	49.18	25.00
3630	seq=translation; coord=7:148082947..148083258:1; parent_transcript=AC196961.2_FGT003; parent_gene=AC196961.2_FG003	AC196961.2_FGP003,AC212565.3_FGP001,AC233865.1_FGP001,GRMZM2G016232_P01,GRMZM2G016232_P02,GRMZM2G063896_P01,GRMZM2G063896_P02,GRMZM2G072855_P01,GRMZM2G073275_P01,GRMZM2G084195_P01,GRMZM2G143780_P01,GRMZM2G143780_P02,GRMZM2G143780_P03,GRMZM2G149178_P01,GRMZM2G181153_P01,GRMZM2G332838_P01,GRMZM2G332838_P02,GRMZM2G349651_P01,GRMZM2G349651_P02,GRMZM2G421279_P01,GRMZM2G479684_P01	TRUE	TRUE	dNIQGITkPAIR	95%	n+304 (+304), K+304 (+304)	37.66	25.54

3631	seq=translation; coord=7:148082947..148083258:1; parent_transcript=AC196961.2_FGT003; parent_gene=AC196961.2_FG003	AC196961.2_FGP003,AC212565.3_FGP001,AC233865.1_FGP001,GRMZM2G016232_P01,GRMZM2G016232_P02,GRMZM2G063896_P01,GRMZM2G063896_P02,GRMZM2G072855_P01,GRMZM2G073275_P01,GRMZM2G084195_P01,GRMZM2G143780_P01,GRMZM2G143780_P02,GRMZM2G143780_P03,GRMZM2G149178_P01,GRMZM2G181153_P01,GRMZM2G332838_P01,GRMZM2G332838_P02,GRMZM2G349651_P01,GRMZM2G349651_P02,GRMZM2G421279_P01,GRMZM2G479684_P01	TRUE	TRUE	iSGLIYEETR	95%	n+304 (+304)	34.44	25.81
3632	seq=translation; coord=7:148082947..148083258:1; parent_transcript=AC196961.2_FGT003; parent_gene=AC196961.2_FG003	AC196961.2_FGP003,AC212565.3_FGP001,AC233865.1_FGP001,GRMZM2G016232_P01,GRMZM2G016232_P02,GRMZM2G063896_P01,GRMZM2G063896_P02,GRMZM2G072855_P01,GRMZM2G073275_P01,GRMZM2G084195_P01,GRMZM2G143780_P01,GRMZM2G143780_P02,GRMZM2G143780_P03,GRMZM2G149178_P01,GRMZM2G181153_P01,GRMZM2G332838_P01,GRMZM2G332838_P02,GRMZM2G349651_P01,GRMZM2G349651_P02,GRMZM2G421279_P01,GRMZM2G479684_P01	TRUE	TRUE	tLYGFGG	95%	n+304 (+304)	32.21	25.00
3633	seq=translation; coord=7:148082947..148083258:1; parent_transcript=AC196961.2_FGT003; parent_gene=AC196961.2_FG003	AC196961.2_FGP003,AC212565.3_FGP001,AC233865.1_FGP001,GRMZM2G016232_P01,GRMZM2G016232_P02,GRMZM2G063896_P01,GRMZM2G063896_P02,GRMZM2G072855_P01,GRMZM2G073275_P01,GRMZM2G084195_P01,GRMZM2G143780_P01,GRMZM2G143780_P02,GRMZM2G143780_P03,GRMZM2G149178_P01,GRMZM2G181153_P01,GRMZM2G332838_P01,GRMZM2G332838_P02,GRMZM2G349651_P01,GRMZM2G349651_P02,GRMZM2G421279_P01,GRMZM2G479684_P01	TRUE	TRUE	tVTAMDVVYALk	95%	n+304 (+304), K+304 (+304)	67.98	26.84
3634	seq=translation; coord=10:85640717..85645627:1; parent_transcript=GRMZM2G394500_T01; parent_gene=GRMZM2G394500	GRMZM2G394500_P01,GRMZM2G394500_P02	TRUE	TRUE	aVAAAFPSFLGk	95%	n+304 (+304), K+304 (+304)	44.67	25.00
3635	seq=translation; coord=10:85640717..85645627:1; parent_transcript=GRMZM2G394500_T01; parent_gene=GRMZM2G394500	GRMZM2G394500_P01,GRMZM2G394500_P02	TRUE	TRUE	dLPDSTFTVSELIR	95%	n+304 (+304)	71.76	25.05
3636	seq=translation; coord=10:85640717..85645627:1; parent_transcript=GRMZM2G394500_T01; parent_gene=GRMZM2G394500	GRMZM2G394500_P01,GRMZM2G394500_P02	TRUE	TRUE	iVTFNSDWQLLTEk	95%	n+304 (+304), K+304 (+304)	35.48	25.66
3637	seq=translation; coord=10:85640717..85645627:1; parent_transcript=GRMZM2G394500_T01; parent_gene=GRMZM2G394500	GRMZM2G394500_P01,GRMZM2G394500_P02	TRUE	TRUE	lSAPPAQIVPAYR	95%	n+304 (+304)	42.32	25.69
3638	seq=translation; coord=10:85640717..85645627:1; parent_transcript=GRMZM2G394500_T01; parent_gene=GRMZM2G394500	GRMZM2G394500_P01,GRMZM2G394500_P02	TRUE	TRUE	tAPINIGLAAFEVIDEIk	95%	n+304 (+304), K+304 (+304)	54.69	25.00
3639	seq=translation; coord=1:41376931..41378234:-1; parent_transcript=GRMZM2G172574_T01; parent_gene=GRMZM2G172574	GRMZM2G172574_P01	TRUE	TRUE	aASFYDAAFGYTVR	95%	n+304 (+304)	54.12	25.00
3640	seq=translation; coord=1:41376931..41378234:-1; parent_transcript=GRMZM2G172574_T01; parent_gene=GRMZM2G172574	GRMZM2G172574_P01	TRUE	TRUE	dMDGNIVR	91%	n+304 (+304)	26.74	25.00

3641	seq=translation; coord=1:41376931..41378234:-1; parent_transcript=GRMZM2G172574_T01; parent_gene=GRMZM2G172574	GRMZM2G172574_P01	TRUE	TRUE	eTDELSGAVQLPDSSAAGR	95%	n+304 (+304)	110.85	25.00
3642	seq=translation; coord=1:41376931..41378234:-1; parent_transcript=GRMZM2G172574_T01; parent_gene=GRMZM2G172574	GRMZM2G172574_P01	TRUE	TRUE	lAYIILYVR	88%	n+304 (+304)	25.73	25.00
3643	seq=translation; coord=1:41376931..41378234:-1; parent_transcript=GRMZM2G172574_T01; parent_gene=GRMZM2G172574	GRMZM2G172574_P01	TRUE	TRUE	wAELESGATTIAFTPLHQR	95%	n+304 (+304)	61.46	25.50
3644	seq=translation; coord=1:96834561..96839284:-1; parent_transcript=GRMZM2G174757_T01; parent_gene=GRMZM2G174757	GRMZM2G174757_P01	TRUE	TRUE	dIPIEVLELENk	95%	n+304 (+304), K+304 (+304)	39.77	25.34
3645	seq=translation; coord=1:96834561..96839284:-1; parent_transcript=GRMZM2G174757_T01; parent_gene=GRMZM2G174757	GRMZM2G174757_P01	TRUE	TRUE	eVEVEEVVDVR	95%	n+304 (+304)	37.44	25.00
3646	seq=translation; coord=1:96834561..96839284:-1; parent_transcript=GRMZM2G174757_T01; parent_gene=GRMZM2G174757	GRMZM2G174757_P01	TRUE	TRUE	iIAFAWEPk	95%	n+304 (+304), K+304 (+304)	37.04	25.77
3647	seq=translation; coord=1:96834561..96839284:-1; parent_transcript=GRMZM2G174757_T01; parent_gene=GRMZM2G174757	GRMZM2G174757_P01	TRUE	TRUE	kYEQEDQDAFNQLSEQDR	95%	K+304 (+304), n+304 (+304)	33.26	25.00
3648	seq=translation; coord=1:96834561..96839284:-1; parent_transcript=GRMZM2G174757_T01; parent_gene=GRMZM2G174757	GRMZM2G174757_P01	TRUE	TRUE	sHIFAVNLLDDFEk	95%	n+304 (+304), K+304 (+304)	29.03	26.03
3649	seq=translation; coord=1:96834561..96839284:-1; parent_transcript=GRMZM2G174757_T01; parent_gene=GRMZM2G174757	GRMZM2G174757_P01	TRUE	TRUE	vVLNIFDVR	95%	n+304 (+304)	37.34	25.00
3650	seq=translation; coord=6:131159543..131163430:-1; parent_transcript=GRMZM2G051630_T01; parent_gene=GRMZM2G051630	GRMZM2G051630_P01	TRUE	TRUE	aMALNMGMLASYDQSVELFR	95%	n+304 (+304)	47.91	25.00
3651	seq=translation; coord=6:131159543..131163430:-1; parent_transcript=GRMZM2G051630_T01; parent_gene=GRMZM2G051630	GRMZM2G051630_P01	TRUE	TRUE	gLSAGLLR	93%	n+304 (+304)	30.02	25.00
3652	seq=translation; coord=6:131159543..131163430:-1; parent_transcript=GRMZM2G051630_T01; parent_gene=GRMZM2G051630	GRMZM2G051630_P01	TRUE	TRUE	iAPHVMMTWIFLNQIQk	95%	n+304 (+304), K+304 (+304)	45.45	25.00
3653	seq=translation; coord=6:131159543..131163430:-1; parent_transcript=GRMZM2G051630_T01; parent_gene=GRMZM2G051630	GRMZM2G051630_P01	TRUE	TRUE	iQLGEGSAGQVTR	95%	n+304 (+304)	38.79	25.99
3654	seq=translation; coord=6:131159543..131163430:-1; parent_transcript=GRMZM2G051630_T01; parent_gene=GRMZM2G051630	GRMZM2G051630_P01	TRUE	TRUE	iSADEGVLALWk	95%	n+304 (+304), K+304 (+304)	35.55	26.01
3655	seq=translation; coord=6:131159543..131163430:-1; parent_transcript=GRMZM2G051630_T01; parent_gene=GRMZM2G051630	GRMZM2G051630_P01	TRUE	TRUE	mQADSTLPAAQR	91%	n+304 (+304)	26.75	25.00
3656	seq=translation; coord=6:131159543..131163430:-1; parent_transcript=GRMZM2G051630_T01; parent_gene=GRMZM2G051630	GRMZM2G051630_P01	TRUE	TRUE	nMLANEGVR	94%	n+304 (+304)	29.47	25.00
3657	seq=translation; coord=4:19423579..19439397:-1; parent_transcript=GRMZM2G019236_T01; parent_gene=GRMZM2G019236	GRMZM2G019236_P01,GRMZM2G019236_P03, GRMZM2G019236_P05	TRUE	TRUE	lLPLYTLALVk	95%	n+304 (+304), K+304 (+304)	28.87	25.00
3658	seq=translation; coord=4:19423579..19439397:-1; parent_transcript=GRMZM2G019236_T01; parent_gene=GRMZM2G019236	GRMZM2G019236_P01,GRMZM2G019236_P03, GRMZM2G019236_P05	TRUE	TRUE	lLVFQSVLPSLGIGSLSAR	95%	n+304 (+304)	34.51	25.00

3659	seq=translation; coord=4:19423579..19439397:-1; parent_transcript=GRMZM2G019236_T01; parent_gene=GRMZM2G019236	GRMZM2G019236_P01,GRMZM2G019236_P03, GRMZM2G019236_P05	TRUE	TRUE	rVPTDIDLPAIDSDk	95%	n+304 (+304), K+304 (+304)	30.66	25.53
3660	seq=translation; coord=4:19423579..19439397:-1; parent_transcript=GRMZM2G019236_T01; parent_gene=GRMZM2G019236	GRMZM2G019236_P01,GRMZM2G019236_P03, GRMZM2G019236_P05	TRUE	TRUE	yADLETQFAcFLk	95%	n+304 (+304), Carbamidomethyl (+57), K+304 (+304)	45.66	25.31
3661	seq=translation; coord=7:41420177..41423983:-1; parent_transcript=GRMZM2G014914_T01; parent_gene=GRMZM2G014914	GRMZM2G014914_P01,GRMZM2G014914_P02	TRUE	TRUE	aFQSAYFDR	92%	n+304 (+304)	28.57	25.00
3662	seq=translation; coord=7:41420177..41423983:-1; parent_transcript=GRMZM2G014914_T01; parent_gene=GRMZM2G014914	GRMZM2G014914_P01,GRMZM2G014914_P02	TRUE	TRUE	dSHVPVLAPLPIGFAVFMVHLATIPVTGTGINPAR	94%	n+304 (+304)	28.23	25.00
3663	seq=translation; coord=7:41420177..41423983:-1; parent_transcript=GRMZM2G014914_T01; parent_gene=GRMZM2G014914	GRMZM2G014914_P01,GRMZM2G014914_P02	TRUE	TRUE	sLGAAVIYNk	95%	n+304 (+304), K+304 (+304)	41.70	25.00
3664	seq=translation; coord=5:205338436..205344337:-1; parent_transcript=GRMZM2G181505_T01; parent_gene=GRMZM2G181505	GRMZM2G181505_P01	TRUE	TRUE	aAWHELIER	95%	n+304 (+304)	32.85	25.15
3665	seq=translation; coord=5:205338436..205344337:-1; parent_transcript=GRMZM2G181505_T01; parent_gene=GRMZM2G181505	GRMZM2G181505_P01	TRUE	TRUE	aTVPVWAk	95%	n+304 (+304), K+304 (+304)	33.64	25.00
3666	seq=translation; coord=5:205338436..205344337:-1; parent_transcript=GRMZM2G181505_T01; parent_gene=GRMZM2G181505	GRMZM2G181505_P01	TRUE	TRUE	aVHPIALAk	90%	n+304 (+304), K+304 (+304)	25.67	25.00
3667	seq=translation; coord=5:205338436..205344337:-1; parent_transcript=GRMZM2G181505_T01; parent_gene=GRMZM2G181505	GRMZM2G181505_P01	TRUE	TRUE	eHNFSSIEEFR	91%	n+304 (+304)	26.18	25.00
3668	seq=translation; coord=5:205338436..205344337:-1; parent_transcript=GRMZM2G181505_T01; parent_gene=GRMZM2G181505	GRMZM2G181505_P01	TRUE	TRUE	iLIGSIMEEYNk	95%	n+304 (+304), K+304 (+304)	35.64	25.73
3669	seq=translation; coord=5:205338436..205344337:-1; parent_transcript=GRMZM2G181505_T01; parent_gene=GRMZM2G181505	GRMZM2G181505_P01	TRUE	TRUE	lcAELQDFMR	95%	n+304 (+304), Carbamidomethyl (+57)	31.91	25.00
3670	seq=translation; coord=5:205338436..205344337:-1; parent_transcript=GRMZM2G181505_T01; parent_gene=GRMZM2G181505	GRMZM2G181505_P01	TRUE	TRUE	mGAAVGQDcDLLEEVcGWINEk	95%	n+304 (+304), Carbamidomethyl (+57), Carbamidomethyl (+57), K+304 (+304)	42.42	25.00
3671	seq=translation; coord=5:205338436..205344337:-1; parent_transcript=GRMZM2G181505_T01; parent_gene=GRMZM2G181505	GRMZM2G181505_P01	TRUE	TRUE	mTPNITDITQPAR	95%	n+304 (+304)	46.19	25.33
3672	seq=translation; coord=5:205338436..205344337:-1; parent_transcript=GRMZM2G181505_T01; parent_gene=GRMZM2G181505	GRMZM2G181505_P01	TRUE	TRUE	sGcEGVSAINTIMSVMGINLk	89%	n+304 (+304), Carbamidomethyl (+57), K+304 (+304)	26.15	25.97
3673	seq=translation; coord=8:12989704..12991151:-1; parent_transcript=GRMZM2G097900_T01; parent_gene=GRMZM2G097900	GRMZM2G097900_P01	TRUE	TRUE	aAFAVYDADGDGR	95%	n+304 (+304)	69.88	25.00
3674	seq=translation; coord=8:12989704..12991151:-1; parent_transcript=GRMZM2G097900_T01; parent_gene=GRMZM2G097900	GRMZM2G097900_P01	TRUE	TRUE	dGFVDLGEFR	95%	n+304 (+304)	44.16	25.00
3675	seq=translation; coord=8:12989704..12991151:-1; parent_transcript=GRMZM2G097900_T01; parent_gene=GRMZM2G097900	GRMZM2G097900_P01	TRUE	TRUE	iSPSELAAVSR	95%	n+304 (+304)	36.86	25.29

3676	seq=translation; coord=8:12989704..12991151:-1; parent_transcript=GRMZM2G097900_T01; parent_gene=GRMZM2G097900	GRMZM2G097900_P01	TRUE	TRUE	iTAAELGSVLAR	95%	n+304 (+304)	34.78	25.00
3677	seq=translation; coord=1:224236124..224244611:-1; parent_transcript=GRMZM2G031572_T01; parent_gene=GRMZM2G031572	GRMZM2G031572_P01	TRUE	TRUE	aALQEITGLVDGQLQSGR	95%	n+304 (+304)	58.80	25.16
3678	seq=translation; coord=1:224236124..224244611:-1; parent_transcript=GRMZM2G031572_T01; parent_gene=GRMZM2G031572	GRMZM2G031572_P01	TRUE	TRUE	nVFGEGVYPDVFMTNLYYGGTGIAGSk	95%	n+304 (+304), K+304 (+304)	36.00	25.00
3679	seq=translation; coord=1:224236124..224244611:-1; parent_transcript=GRMZM2G031572_T01; parent_gene=GRMZM2G031572	GRMZM2G031572_P01	TRUE	TRUE	qALFDLAVFR	87%	n+304 (+304)	26.84	25.95
3680	seq=translation; coord=1:224236124..224244611:-1; parent_transcript=GRMZM2G031572_T01; parent_gene=GRMZM2G031572	GRMZM2G031572_P01	TRUE	TRUE	yYEFLDYYR	95%	n+304 (+304)	33.87	25.00
3681	seq=translation; coord=8:101408887..101413622:1; parent_transcript=GRMZM2G033799_T01; parent_gene=GRMZM2G033799	GRMZM2G033799_P01,GRMZM2G033799_P02, GRMZM2G033799_P03	TRUE	TRUE	eLLEEIAAIVR	88%	n+304 (+304)	26.39	25.22
3682	seq=translation; coord=8:101408887..101413622:1; parent_transcript=GRMZM2G033799_T01; parent_gene=GRMZM2G033799	GRMZM2G033799_P01,GRMZM2G033799_P02, GRMZM2G033799_P03	TRUE	TRUE	tMAITDQATALR	86%	n+304 (+304)	25.50	25.25
3683	seq=translation; coord=1:8135964..8147853:-1; parent_transcript=GRMZM2G176397_T01; parent_gene=GRMZM2G176397	GRMZM2G176397_P01,GRMZM2G176397_P02	TRUE	TRUE	mFVDHSQVDAER	95%	n+304 (+304)	53.61	25.00
3684	seq=translation; coord=1:8135964..8147853:-1; parent_transcript=GRMZM2G176397_T01; parent_gene=GRMZM2G176397	GRMZM2G176397_P01,GRMZM2G176397_P02	TRUE	TRUE	sNLAGMGIIPLcYk	91%	n+304 (+304), Carbamidomethyl (+57), K+304 (+304)	26.44	25.51
3685	seq=translation; coord=3:136407818..136421472:1; parent_transcript=GRMZM2G168510_T02; parent_gene=GRMZM2G168510	GRMZM2G168510_P02	TRUE	TRUE	eSNcPVVADVTHALQQPAGR	95%	n+304 (+304), Carbamidomethyl (+57)	67.53	25.00
3686	seq=translation; coord=3:136407818..136421472:1; parent_transcript=GRMZM2G168510_T02; parent_gene=GRMZM2G168510	GRMZM2G168510_P02	TRUE	TRUE	lAGNPNVMVcER	95%	n+304 (+304), Carbamidomethyl (+57)	35.79	25.00
3687	seq=translation; coord=3:136407818..136421472:1; parent_transcript=GRMZM2G168510_T02; parent_gene=GRMZM2G168510	GRMZM2G168510_P02	TRUE	TRUE	lGIPLVFk	95%	n+304 (+304), K+304 (+304)	35.90	25.00
3688	seq=translation; coord=3:136407818..136421472:1; parent_transcript=GRMZM2G168510_T02; parent_gene=GRMZM2G168510	GRMZM2G168510_P02	TRUE	TRUE	vADIIQIPAFLcR	95%	n+304 (+304), Carbamidomethyl (+57)	42.54	26.28
3689	seq=translation; coord=8:21615163..21616013:1; parent_transcript=GRMZM2G034157_T01; parent_gene=GRMZM2G034157	GRMZM2G034157_P01	TRUE	TRUE	eLPGAYAFVVDMPGLGTGDIR	94%	n+304 (+304)	28.28	25.00
3690	seq=translation; coord=8:21615163..21616013:1; parent_transcript=GRMZM2G034157_T01; parent_gene=GRMZM2G034157	GRMZM2G034157_P01	TRUE	TRUE	eLPGAyAFVVDMPGLGTGDIR	95%	n+304 (+304), iTRAQ8plex (+304)	35.86	25.00
3691	seq=translation; coord=8:21615163..21616013:1; parent_transcript=GRMZM2G034157_T01; parent_gene=GRMZM2G034157	GRMZM2G034157_P01	TRUE	TRUE	vLVVSGER	89%	n+304 (+304)	26.84	25.33
3692	seq=translation; coord=5:125802058..125817596:1; parent_transcript=GRMZM2G038126_T01; parent_gene=GRMZM2G038126	GRMZM2G038126_P01,GRMZM2G038126_P02, GRMZM2G090904_P01,GRMZM2G090904_P02	TRUE	TRUE	eNAPAIIFIDEVDAIATAR	95%	n+304 (+304)	42.82	25.00
3693	seq=translation; coord=5:125802058..125817596:1; parent_transcript=GRMZM2G038126_T01; parent_gene=GRMZM2G038126	GRMZM2G038126_P01,GRMZM2G038126_P02, GRMZM2G090904_P01,GRMZM2G090904_P02	TRUE	TRUE	mNLSDEVDLEDYVSRPDk	95%	n+304 (+304), K+304 (+304)	31.30	25.00

3694	seq=translation; coord=1:14449794..14451164:-1; parent_transcript=GRMZM2G063287_T01; parent_gene=GRMZM2G063287	GRMZM2G063287_P01	TRUE	TRUE	aQETLSQTADAAAEk	95%	n+304 (+304), K+304 (+304)	39.73	25.28
3695	seq=translation; coord=1:14449794..14451164:-1; parent_transcript=GRMZM2G063287_T01; parent_gene=GRMZM2G063287	GRMZM2G063287_P01	TRUE	TRUE	eAAEAASESGAEAHER	95%	n+304 (+304)	84.94	25.00
3696	seq=translation; coord=1:14449794..14451164:-1; parent_transcript=GRMZM2G063287_T01; parent_gene=GRMZM2G063287	GRMZM2G063287_P01	TRUE	TRUE	eTAGDAAAGASNk	95%	n+304 (+304), K+304 (+304)	59.72	25.00
3697	seq=translation; coord=1:14449794..14451164:-1; parent_transcript=GRMZM2G063287_T01; parent_gene=GRMZM2G063287	GRMZM2G063287_P01	TRUE	TRUE	iSEGLGLk	93%	n+304 (+304), K+304 (+304)	28.64	25.47
3698	seq=translation; coord=1:14449794..14451164:-1; parent_transcript=GRMZM2G063287_T01; parent_gene=GRMZM2G063287	GRMZM2G063287_P01	TRUE	TRUE	vAGPDVAAVDGk	95%	n+304 (+304), K+304 (+304)	35.95	25.05
3699	seq=translation; coord=1:19327055..19329850:-1; parent_transcript=GRMZM2G154007_T01; parent_gene=GRMZM2G154007	GRMZM2G154007_P01	TRUE	TRUE	aAGEPLTIEEIVVDPPk	95%	n+304 (+304), K+304 (+304)	35.01	25.00
3700	seq=translation; coord=1:19327055..19329850:-1; parent_transcript=GRMZM2G154007_T01; parent_gene=GRMZM2G154007	GRMZM2G154007_P01	TRUE	TRUE	aFDLLLQGk	93%	n+304 (+304), K+304 (+304)	27.09	25.00
3701	seq=translation; coord=1:19327055..19329850:-1; parent_transcript=GRMZM2G154007_T01; parent_gene=GRMZM2G154007	GRMZM2G154007_P01	TRUE	TRUE	dSDEVcLPSLELLFGR	95%	n+304 (+304), Carbamidomethyl (+57)	53.00	25.00
3702	seq=translation; coord=1:19327055..19329850:-1; parent_transcript=GRMZM2G154007_T01; parent_gene=GRMZM2G154007	GRMZM2G154007_P01	TRUE	TRUE	lAcLLScGAGTGVGAAWR	93%	n+304 (+304), Carbamidomethyl (+57), Carbamidomethyl (+57)	27.53	25.00
3703	seq=translation; coord=1:19327055..19329850:-1; parent_transcript=GRMZM2G154007_T01; parent_gene=GRMZM2G154007	GRMZM2G154007_P01	TRUE	TRUE	vAPVFPR	90%	n+304 (+304)	27.22	25.00
3704	seq=translation; coord=1:19327055..19329850:-1; parent_transcript=GRMZM2G154007_T01; parent_gene=GRMZM2G154007	GRMZM2G154007_P01	TRUE	TRUE	vEPGSTVAIFGLGSVGLAVVQGAk	95%	n+304 (+304), K+304 (+304)	47.33	25.00
3705	seq=translation; coord=5:46946530..46947446:-1; parent_transcript=AC209987.4_FGT010; parent_gene=AC209987.4_FG010	AC209987.4_FGP010	TRUE	TRUE	fDDGSTVFTFDR	95%	n+304 (+304)	38.07	25.00
3706	seq=translation; coord=5:46946530..46947446:-1; parent_transcript=AC209987.4_FGT010; parent_gene=AC209987.4_FG010	AC209987.4_FGP010	TRUE	TRUE	fQIGDQLLFVYPk	95%	n+304 (+304), K+304 (+304)	35.67	25.42
3707	seq=translation; coord=5:46946530..46947446:-1; parent_transcript=AC209987.4_FGT010; parent_gene=AC209987.4_FG010	AC209987.4_FGP010	TRUE	TRUE	sGAFFFVSGNEAScR	95%	n+304 (+304), Carbamidomethyl (+57)	72.12	25.00
3708	seq=translation; coord=10:137458157..137460537:-1; parent_transcript=AC209206.3_FGT014; parent_gene=AC209206.3_FG014	AC209206.3_FGP014	TRUE	TRUE	aQQIDQNLMIMYR	95%	n+304 (+304)	41.16	25.00
3709	seq=translation; coord=10:137458157..137460537:-1; parent_transcript=AC209206.3_FGT014; parent_gene=AC209206.3_FG014	AC209206.3_FGP014	TRUE	TRUE	dPLFFAHHGNIDR	95%	n+304 (+304)	29.63	25.00
3710	seq=translation; coord=10:137458157..137460537:-1; parent_transcript=AC209206.3_FGT014; parent_gene=AC209206.3_FG014	AC209206.3_FGP014	TRUE	TRUE	fGIcDLLDDIGADGDk	95%	n+304 (+304), Carbamidomethyl (+57), K+304 (+304)	52.93	25.00
3711	seq=translation; coord=10:137458157..137460537:-1; parent_transcript=AC209206.3_FGT014; parent_gene=AC209206.3_FG014	AC209206.3_FGP014	TRUE	TRUE	fYLYFHER	95%	n+304 (+304)	30.43	25.00

3712	seq=translation; coord=10:137458157..137460537:-1; parent_transcript=AC209206.3_FGT014; parent_gene=AC209206.3_FG014	AC209206.3_FGP014	TRUE	TRUE	rDPAHQPPFTLDLDYDGTEPTIPR	95%	n+304 (+304)	27.98	25.00
3713	seq=translation; coord=1:228643379..228644379:-1; parent_transcript=GRMZM2G153208_T01; parent_gene=GRMZM2G153208	GRMZM2G153208_P01	TRUE	TRUE	dGYTDDFFAQILGk	95%	n+304 (+304), K+304 (+304)	29.46	25.00
3714	seq=translation; coord=1:228643379..228644379:-1; parent_transcript=GRMZM2G153208_T01; parent_gene=GRMZM2G153208	GRMZM2G153208_P01	TRUE	TRUE	fDQAVGLDYAk	95%	n+304 (+304), K+304 (+304)	70.06	25.59
3715	seq=translation; coord=1:228643379..228644379:-1; parent_transcript=GRMZM2G153208_T01; parent_gene=GRMZM2G153208	GRMZM2G153208_P01	TRUE	TRUE	tVQQLWQDYk	95%	n+304 (+304), K+304 (+304)	44.76	25.29
3716	seq=translation; coord=1:228643379..228644379:-1; parent_transcript=GRMZM2G153208_T01; parent_gene=GRMZM2G153208	GRMZM2G153208_P01	TRUE	TRUE	wDQGYDVTAR	91%	n+304 (+304)	28.25	25.00
3717	seq=translation; coord=4:231475847..231481286:1; parent_transcript=GRMZM2G107362_T02; parent_gene=GRMZM2G107362	GRMZM2G107362_P02,GRMZM2G107362_P04, GRMZM2G137528_P01,GRMZM2G137528_P02	TRUE	TRUE	aVcTEAGMFALR	90%	n+304 (+304), Carbamidomethyl (+57)	26.37	25.00
3718	seq=translation; coord=4:231475847..231481286:1; parent_transcript=GRMZM2G107362_T02; parent_gene=GRMZM2G107362	GRMZM2G107362_P02,GRMZM2G107362_P04, GRMZM2G137528_P01,GRMZM2G137528_P02	TRUE	TRUE	eHAPSIIFMDEIDSIGSAR	95%	n+304 (+304)	62.29	25.00
3719	seq=translation; coord=4:231475847..231481286:1; parent_transcript=GRMZM2G107362_T02; parent_gene=GRMZM2G107362	GRMZM2G107362_P02,GRMZM2G107362_P04, GRMZM2G137528_P01,GRMZM2G137528_P02	TRUE	TRUE	eLFVMAR	94%	n+304 (+304)	30.30	25.00
3720	seq=translation; coord=4:231475847..231481286:1; parent_transcript=GRMZM2G107362_T02; parent_gene=GRMZM2G107362	GRMZM2G107362_P02,GRMZM2G107362_P04, GRMZM2G137528_P01,GRMZM2G137528_P02	TRUE	TRUE	iDILDQALLRPGR	92%	n+304 (+304)	26.16	25.00
3721	seq=translation; coord=4:231475847..231481286:1; parent_transcript=GRMZM2G107362_T02; parent_gene=GRMZM2G107362	GRMZM2G107362_P02,GRMZM2G107362_P04, GRMZM2G137528_P01,GRMZM2G137528_P02	TRUE	TRUE	tMLELLNQLDGFEASNk	95%	n+304 (+304), K+304 (+304)	74.14	25.88
3722	seq=translation; coord=4:231475847..231481286:1; parent_transcript=GRMZM2G107362_T02; parent_gene=GRMZM2G107362	GRMZM2G107362_P02,GRMZM2G107362_P04, GRMZM2G137528_P01,GRMZM2G137528_P02	TRUE	TRUE	vDPLVNLMk	95%	n+304 (+304), K+304 (+304)	40.00	25.00
3723	seq=translation; coord=4:231475847..231481286:1; parent_transcript=GRMZM2G107362_T02; parent_gene=GRMZM2G107362	GRMZM2G107362_P02,GRMZM2G107362_P04, GRMZM2G137528_P01,GRMZM2G137528_P02	TRUE	TRUE	vPDSTYDMIGGLDQQIk	95%	n+304 (+304), K+304 (+304)	38.22	25.58
3724	seq=translation; coord=5:18452211..18455290:-1; parent_transcript=GRMZM2G066996_T01; parent_gene=GRMZM2G066996	GRMZM2G066996_P01	TRUE	TRUE	aDTQSVDFQk	95%	n+304 (+304), K+304 (+304)	31.87	25.68
3725	seq=translation; coord=5:18452211..18455290:-1; parent_transcript=GRMZM2G066996_T01; parent_gene=GRMZM2G066996	GRMZM2G066996_P01	TRUE	TRUE	eDLTGVVLFVGHVVNPLLAP	95%	n+304 (+304)	33.48	25.00
3726	seq=translation; coord=5:18452211..18455290:-1; parent_transcript=GRMZM2G066996_T01; parent_gene=GRMZM2G066996	GRMZM2G066996_P01	TRUE	TRUE	iSFGFEASELLk	95%	n+304 (+304), K+304 (+304)	54.00	25.31
3727	seq=translation; coord=5:18452211..18455290:-1; parent_transcript=GRMZM2G066996_T01; parent_gene=GRMZM2G066996	GRMZM2G066996_P01	TRUE	TRUE	lVLGNALYFk	95%	n+304 (+304), K+304 (+304)	45.80	25.00
3728	seq=translation; coord=5:18452211..18455290:-1; parent_transcript=GRMZM2G066996_T01; parent_gene=GRMZM2G066996	GRMZM2G066996_P01	TRUE	TRUE	sFTMPQDFVADHPFMFLIR	95%	n+304 (+304)	46.53	25.00
3729	seq=translation; coord=5:18452211..18455290:-1; parent_transcript=GRMZM2G066996_T01; parent_gene=GRMZM2G066996	GRMZM2G066996_P01	TRUE	TRUE	sFVEVNEEGTEAAAASAATVVLR	95%	n+304 (+304)	50.70	25.00

3730	seq=translation; coord=5:18452211..18455290:-1; parent_transcript=GRMZM2G066996_T01; parent_gene=GRMZM2G066996	GRMZM2G066996_P01	TRUE	TRUE	vAFADGVFVDSSLk	95%	n+304 (+304), K+304 (+304)	54.07	26.17
3731	seq=translation; coord=9:8196377..8197429:-1; parent_transcript=GRMZM2G150656_T03; parent_gene=GRMZM2G150656	GRMZM2G150656_P03	TRUE	TRUE	iGVDEEIFVVDLk	95%	n+304 (+304), K+304 (+304)	43.44	25.75
3732	seq=translation; coord=9:8196377..8197429:-1; parent_transcript=GRMZM2G150656_T03; parent_gene=GRMZM2G150656	GRMZM2G150656_P03	TRUE	TRUE	kIGVDEEIFVVDLk	95%	K+304 (+304), n+304 (+304), K+304 (+304)	34.39	25.00
3733	seq=translation; coord=9:8196377..8197429:-1; parent_transcript=GRMZM2G150656_T03; parent_gene=GRMZM2G150656	GRMZM2G150656_P03	TRUE	TRUE	sAQLLELMR	92%	n+304 (+304)	29.01	25.43
3734	seq=translation; coord=9:8196377..8197429:-1; parent_transcript=GRMZM2G150656_T03; parent_gene=GRMZM2G150656	GRMZM2G150656_P03	TRUE	TRUE	vGLVYQLNIAPk	95%	n+304 (+304), K+304 (+304)	33.89	25.00
3735	seq=translation; coord=7:165014609..165016387:1; parent_transcript=GRMZM2G393671_T01; parent_gene=GRMZM2G393671	GRMZM2G393671_P01	TRUE	TRUE	aRPEAVASGAAAAMVSSLQALATLVPGLAYIHAGPVLR	95%	n+304 (+304)	44.29	25.00
3736	seq=translation; coord=7:165014609..165016387:1; parent_transcript=GRMZM2G393671_T01; parent_gene=GRMZM2G393671	GRMZM2G393671_P01	TRUE	TRUE	eGVEVAQLVEk	95%	n+304 (+304), K+304 (+304)	67.34	26.32
3737	seq=translation; coord=7:165014609..165016387:1; parent_transcript=GRMZM2G393671_T01; parent_gene=GRMZM2G393671	GRMZM2G393671_P01	TRUE	TRUE	vAAATQAAGDAk	91%	n+304 (+304), K+304 (+304)	26.92	25.98
3738	seq=translation; coord=7:165014609..165016387:1; parent_transcript=GRMZM2G393671_T01; parent_gene=GRMZM2G393671	GRMZM2G393671_P01	TRUE	TRUE	vkEGVEVAQLVEk	94%	n+304 (+304), K+304 (+304), K+304 (+304)	27.96	25.00
3739	seq=translation; coord=7:165014609..165016387:1; parent_transcript=GRMZM2G393671_T01; parent_gene=GRMZM2G393671	GRMZM2G393671_P01	TRUE	TRUE	vSFGENFSPAR	95%	n+304 (+304)	33.16	25.00
3740	seq=translation; coord=8:158113092..158118053:-1; parent_transcript=GRMZM2G109472_T01; parent_gene=GRMZM2G109472	GRMZM2G109472_P01,GRMZM2G109472_P03	TRUE	TRUE	aEGLVVADAVVVVDR	95%	n+304 (+304)	51.27	26.15
3741	seq=translation; coord=8:158113092..158118053:-1; parent_transcript=GRMZM2G109472_T01; parent_gene=GRMZM2G109472	GRMZM2G109472_P01,GRMZM2G109472_P03	TRUE	TRUE	lFELMETk	95%	n+304 (+304), K+304 (+304)	30.35	25.50
3742	seq=translation; coord=8:158113092..158118053:-1; parent_transcript=GRMZM2G109472_T01; parent_gene=GRMZM2G109472	GRMZM2G109472_P01,GRMZM2G109472_P03	TRUE	TRUE	tHVDILSDFTPDFGSk	95%	n+304 (+304), K+304 (+304)	27.53	25.00
3743	seq=translation; coord=7:170244063..170247650:1; parent_transcript=GRMZM2G024484_T01; parent_gene=GRMZM2G024484	GRMZM2G024484_P01,GRMZM2G040209_P01, GRMZM2G040209_P02	TRUE	TRUE	eGTPWLWMIMER	95%	n+304 (+304)	35.79	25.00
3744	seq=translation; coord=7:170244063..170247650:1; parent_transcript=GRMZM2G024484_T01; parent_gene=GRMZM2G024484	GRMZM2G024484_P01,GRMZM2G040209_P01, GRMZM2G040209_P02	TRUE	TRUE	gGWDNLLAVIPGGSSVPLLPk	95%	n+304 (+304), K+304 (+304)	31.60	25.00
3745	seq=translation; coord=7:170244063..170247650:1; parent_transcript=GRMZM2G024484_T01; parent_gene=GRMZM2G024484	GRMZM2G024484_P01,GRMZM2G040209_P01, GRMZM2G040209_P02	TRUE	TRUE	hIcDDVLMDYDALk	95%	n+304 (+304), Carbamidomethyl (+57), K+304 (+304)	34.58	25.00
3746	seq=translation; coord=7:170244063..170247650:1; parent_transcript=GRMZM2G024484_T01; parent_gene=GRMZM2G024484	GRMZM2G024484_P01,GRMZM2G040209_P01, GRMZM2G040209_P02	TRUE	TRUE	rGPEWFASFGR	95%	n+304 (+304)	43.99	25.00
3747	seq=translation; coord=9:7615635..7616809:-1; parent_transcript=GRMZM5G898880_T01; parent_gene=GRMZM5G898880	GRMZM5G898880_P01	TRUE	TRUE	aSAAGALPDGVTIR	95%	n+304 (+304)	49.14	26.61

3748	seq=translation; coord=9:7615635..7616809:-1; parent_transcript=GRMZM5G898880_T01; parent_gene=GRMZM5G898880	GRMZM5G898880_P01	TRUE	TRUE	fVVSDTVGLDDPR	95%	n+304 (+304)	50.51	25.00
3749	seq=translation; coord=9:7615635..7616809:-1; parent_transcript=GRMZM5G898880_T01; parent_gene=GRMZM5G898880	GRMZM5G898880_P01	TRUE	TRUE	lPAAYDDAWAALR	95%	n+304 (+304)	41.43	25.39
3750	seq=translation; coord=9:7615635..7616809:-1; parent_transcript=GRMZM5G898880_T01; parent_gene=GRMZM5G898880	GRMZM5G898880_P01	TRUE	TRUE	vLVcVAENDFLLk	95%	n+304 (+304), Carbamidomethyl (+57), K+304 (+304)	45.18	25.00
3751	seq=translation; coord=9:7615635..7616809:-1; parent_transcript=GRMZM5G898880_T01; parent_gene=GRMZM5G898880	GRMZM5G898880_P01	TRUE	TRUE	vNPFVDDAAR	95%	n+304 (+304)	37.58	25.00
3752	seq=translation; coord=2:1928487..1932811:-1; parent_transcript=GRMZM2G407347_T01; parent_gene=GRMZM2G407347	GRMZM2G407347_P01,GRMZM2G407347_P04	TRUE	TRUE	aVGDLAAAAEVSR	95%	n+304 (+304)	49.79	25.85
3753	seq=translation; coord=2:1928487..1932811:-1; parent_transcript=GRMZM2G407347_T01; parent_gene=GRMZM2G407347	GRMZM2G407347_P01,GRMZM2G407347_P04	TRUE	TRUE	eQLVAEIR	95%	n+304 (+304)	34.53	26.32
3754	seq=translation; coord=2:1928487..1932811:-1; parent_transcript=GRMZM2G407347_T01; parent_gene=GRMZM2G407347	GRMZM2G407347_P01,GRMZM2G407347_P04	TRUE	TRUE	lELTAEQINNPQSAcGScGLGDAFR	95%	n+304 (+304), Carbamidomethyl (+57), Carbamidomethyl (+57)	35.05	25.00
3755	seq=translation; coord=2:1928487..1932811:-1; parent_transcript=GRMZM2G407347_T01; parent_gene=GRMZM2G407347	GRMZM2G407347_P01,GRMZM2G407347_P04	TRUE	TRUE	lPFEDASVGAVLAVIk	95%	n+304 (+304), K+304 (+304)	48.14	25.00
3756	seq=translation; coord=1:68430654..68436197:1; parent_transcript=GRMZM5G866758_T02; parent_gene=GRMZM5G866758	GRMZM5G866758_P02	TRUE	TRUE	dSGAFAWEIIPVQVPVGR	89%	n+304 (+304)	27.46	25.97
3757	seq=translation; coord=1:68430654..68436197:1; parent_transcript=GRMZM5G866758_T02; parent_gene=GRMZM5G866758	GRMZM5G866758_P02	TRUE	TRUE	eDQDAFAIQSNER	95%	n+304 (+304)	34.75	25.00
3758	seq=translation; coord=1:68430654..68436197:1; parent_transcript=GRMZM5G866758_T02; parent_gene=GRMZM5G866758	GRMZM5G866758_P02	TRUE	TRUE	tPMGGFLGALSPLPATk	95%	n+304 (+304), K+304 (+304)	31.88	25.00
3759	seq=translation; coord=4:14930698..14950684:-1; parent_transcript=GRMZM2G151050_T01; parent_gene=GRMZM2G151050	GRMZM2G151050_P01,GRMZM2G151050_P02	TRUE	TRUE	aPWEAQAAAGSLPPPPAR	95%	n+304 (+304)	97.35	25.35
3760	seq=translation; coord=4:14930698..14950684:-1; parent_transcript=GRMZM2G151050_T01; parent_gene=GRMZM2G151050	GRMZM2G151050_P01,GRMZM2G151050_P02	TRUE	TRUE	dLVDFAk	93%	n+304 (+304), K+304 (+304)	28.28	26.27
3761	seq=translation; coord=4:14930698..14950684:-1; parent_transcript=GRMZM2G151050_T01; parent_gene=GRMZM2G151050	GRMZM2G151050_P01,GRMZM2G151050_P02	TRUE	TRUE	gIVDVLDEMLNALDHR	95%	n+304 (+304)	51.08	25.53
3762	seq=translation; coord=4:14930698..14950684:-1; parent_transcript=GRMZM2G151050_T01; parent_gene=GRMZM2G151050	GRMZM2G151050_P01,GRMZM2G151050_P02	TRUE	TRUE	iLSLIDTWQVAFGGPSGk	95%	n+304 (+304), K+304 (+304)	69.96	25.00
3763	seq=translation; coord=4:14930698..14950684:-1; parent_transcript=GRMZM2G151050_T01; parent_gene=GRMZM2G151050	GRMZM2G151050_P01,GRMZM2G151050_P02	TRUE	TRUE	rPVYTEASSVDYLSGDSYk	95%	n+304 (+304), K+304 (+304)	32.29	25.00
3764	seq=translation; coord=4:14930698..14950684:-1; parent_transcript=GRMZM2G151050_T01; parent_gene=GRMZM2G151050	GRMZM2G151050_P01,GRMZM2G151050_P02	TRUE	TRUE	vQILTLYVLETLSk	95%	n+304 (+304), K+304 (+304)	33.20	25.00
3765	seq=translation; coord=1:39213217..39219522:-1; parent_transcript=GRMZM2G141799_T01; parent_gene=GRMZM2G141799	GRMZM2G141799_P01	TRUE	TRUE	aNSVAPWYITTSLTEGILANk	95%	n+304 (+304), K+304 (+304)	45.52	25.00

3766	seq=translation; coord=1:39213217..39219522:-1; parent_transcript=GRMZM2G141799_T01; parent_gene=GRMZM2G141799	GRMZM2G141799_P01	TRUE	TRUE	aVVEELAALGAAVHTcSR	95%	n+304 (+304), Carbamidomethyl (+57)	68.87	25.04
3767	seq=translation; coord=1:39213217..39219522:-1; parent_transcript=GRMZM2G141799_T01; parent_gene=GRMZM2G141799	GRMZM2G141799_P01	TRUE	TRUE	gFSVTGSVcDLSER	95%	n+304 (+304), Carbamidomethyl (+57)	53.94	25.00
3768	seq=translation; coord=1:39213217..39219522:-1; parent_transcript=GRMZM2G141799_T01; parent_gene=GRMZM2G141799	GRMZM2G141799_P01	TRUE	TRUE	nFEEQVVSR	95%	n+304 (+304)	32.54	25.00
3769	seq=translation; coord=4:53078446..53083109:-1; parent_transcript=GRMZM2G134708_T01; parent_gene=GRMZM2G134708	GRMZM2G134708_P01	TRUE	TRUE	aQPPVADVQALk	95%	n+304 (+304), K+304 (+304)	31.63	25.00
3770	seq=translation; coord=4:53078446..53083109:-1; parent_transcript=GRMZM2G134708_T01; parent_gene=GRMZM2G134708	GRMZM2G134708_P01	TRUE	TRUE	eSGESVAEYDYLPyFYSR	95%	n+304 (+304), iTRAQ8plex (+304)	37.43	25.00
3771	seq=translation; coord=4:53078446..53083109:-1; parent_transcript=GRMZM2G134708_T01; parent_gene=GRMZM2G134708	GRMZM2G134708_P01	TRUE	TRUE	gYLFPQNAAR	93%	n+304 (+304)	29.69	25.00
3772	seq=translation; coord=4:53078446..53083109:-1; parent_transcript=GRMZM2G134708_T01; parent_gene=GRMZM2G134708	GRMZM2G134708_P01	TRUE	TRUE	lFIGQAADEk	95%	n+304 (+304), K+304 (+304)	46.06	25.87
3773	seq=translation; coord=4:53078446..53083109:-1; parent_transcript=GRMZM2G134708_T01; parent_gene=GRMZM2G134708	GRMZM2G134708_P01	TRUE	TRUE	lTDFGVQGAESNNILYLR	95%	n+304 (+304)	39.64	25.00
3774	seq=translation; coord=4:53078446..53083109:-1; parent_transcript=GRMZM2G134708_T01; parent_gene=GRMZM2G134708	GRMZM2G134708_P01	TRUE	TRUE	tLTSAAAETFTYETLLIATGSSVIk	95%	n+304 (+304), K+304 (+304)	30.48	25.00
3775	seq=translation; coord=4:53078446..53083109:-1; parent_transcript=GRMZM2G134708_T01; parent_gene=GRMZM2G134708	GRMZM2G134708_P01	TRUE	TRUE	vVGVFLEGGSAEENQAIAR	95%	n+304 (+304)	77.15	25.58
3776	seq=translation; coord=8:83335717..83339016:1; parent_transcript=GRMZM2G104025_T01; parent_gene=GRMZM2G104025	GRMZM2G104025_P01,GRMZM5G803952_P03	TRUE	TRUE	aPLGENTVLLR	95%	n+304 (+304)	33.03	25.85
3777	seq=translation; coord=8:83335717..83339016:1; parent_transcript=GRMZM2G104025_T01; parent_gene=GRMZM2G104025	GRMZM2G104025_P01,GRMZM5G803952_P03	TRUE	TRUE	iVNAGGEcLTFDQLALR	95%	n+304 (+304), Carbamidomethyl (+57)	46.35	25.28
3778	seq=translation; coord=5:67403566..67405443:1; parent_transcript=GRMZM2G128929_T01; parent_gene=GRMZM2G128929	GRMZM2G128929_P01	TRUE	TRUE	aGVQGVAEMELTEEEAk	95%	n+304 (+304), K+304 (+304)	68.65	25.00
3779	seq=translation; coord=5:67403566..67405443:1; parent_transcript=GRMZM2G128929_T01; parent_gene=GRMZM2G128929	GRMZM2G128929_P01	TRUE	TRUE	gEMLDLQHAAAFLPR	95%	n+304 (+304)	62.44	25.00
3780	seq=translation; coord=5:67403566..67405443:1; parent_transcript=GRMZM2G128929_T01; parent_gene=GRMZM2G128929	GRMZM2G128929_P01	TRUE	TRUE	gFHGIPDGNDVFLSLPAR	95%	n+304 (+304)	44.35	25.50
3781	seq=translation; coord=5:67403566..67405443:1; parent_transcript=GRMZM2G128929_T01; parent_gene=GRMZM2G128929	GRMZM2G128929_P01	TRUE	TRUE	gSDLVIVTAGAR	95%	n+304 (+304)	31.90	25.20
3782	seq=translation; coord=5:67403566..67405443:1; parent_transcript=GRMZM2G128929_T01; parent_gene=GRMZM2G128929	GRMZM2G128929_P01	TRUE	TRUE	gyTSWAIGYSVASLAASLLR	95%	n+304 (+304), iTRAQ8plex (+304)	31.66	25.00
3783	seq=translation; coord=5:67403566..67405443:1; parent_transcript=GRMZM2G128929_T01; parent_gene=GRMZM2G128929	GRMZM2G128929_P01	TRUE	TRUE	vIGSGTNLDSSR	94%	n+304 (+304)	30.88	25.20

3784	seq=translation; coord=5:67403566..67405443:1;<br>parent_transcript=GRMZM2G128929_T01;<br>parent_gene=GRMZM2G128929	GRMZM2G128929_P01	TRUE	TRUE	vSVIGAGNVGMAIAQTILTR	95%	n+304 (+304)	37.47	25.00
3785	seq=translation; coord=2:4834198..4840631:-1;<br>parent_transcript=GRMZM2G033219_T01;<br>parent_gene=GRMZM2G033219	GRMZM2G033219_P01,GRMZM2G033219_P02,<br>GRMZM2G143480_P01,GRMZM2G143480_P02,<br>GRMZM2G143480_P03	TRUE	TRUE	gAEAALTAIEMASLFR	95%	n+304 (+304)	56.85	25.00
3786	seq=translation; coord=7:159911588..159920834:-1;<br>parent_transcript=GRMZM2G305211_T02;<br>parent_gene=GRMZM2G305211	GRMZM2G305211_P02	TRUE	TRUE	tNVSPEVAESTR	95%	n+304 (+304)	50.01	25.00
3787	seq=translation; coord=9:133421526..133422671:1;<br>parent_transcript=GRMZM2G704475_T02;<br>parent_gene=GRMZM2G704475	GRMZM2G704475_P02	TRUE	TRUE	aASVYFEEk	95%	n+304 (+304), K+304 (+304)	38.46	25.49
3788	seq=translation; coord=9:133421526..133422671:1;<br>parent_transcript=GRMZM2G704475_T02;<br>parent_gene=GRMZM2G704475	GRMZM2G704475_P02	TRUE	TRUE	dYTADk	92%	n+304 (+304), K+304 (+304)	28.36	25.68
3789	seq=translation; coord=9:133421526..133422671:1;<br>parent_transcript=GRMZM2G704475_T02;<br>parent_gene=GRMZM2G704475	GRMZM2G704475_P02	TRUE	TRUE	eQGQGLLGALGNVTGAIk	95%	n+304 (+304), K+304 (+304)	56.21	25.00
3790	seq=translation; coord=9:133421526..133422671:1;<br>parent_transcript=GRMZM2G704475_T02;<br>parent_gene=GRMZM2G704475	GRMZM2G704475_P02	TRUE	TRUE	gAAGGGGILESVQEGAR	95%	n+304 (+304)	100.38	25.00
3791	seq=translation; coord=9:133421526..133422671:1;<br>parent_transcript=GRMZM2G704475_T02;<br>parent_gene=GRMZM2G704475	GRMZM2G704475_P02	TRUE	TRUE	lTLGQGQHVDVR	95%	n+304 (+304)	65.35	26.15
3792	seq=translation; coord=9:133421526..133422671:1;<br>parent_transcript=GRMZM2G704475_T02;<br>parent_gene=GRMZM2G704475	GRMZM2G704475_P02	TRUE	TRUE	tSETAEATk	95%	n+304 (+304), K+304 (+304)	29.99	25.11
3793	seq=translation; coord=1:293793122..293796213:1;<br>parent_transcript=GRMZM2G459811_T02;<br>parent_gene=GRMZM2G459811	GRMZM2G459811_P02,GRMZM2G459811_P03	TRUE	TRUE	aAEVVSTIVQNNPk	95%	n+304 (+304), K+304 (+304)	29.44	25.54
3794	seq=translation; coord=1:293793122..293796213:1;<br>parent_transcript=GRMZM2G459811_T02;<br>parent_gene=GRMZM2G459811	GRMZM2G459811_P02,GRMZM2G459811_P03	TRUE	TRUE	aLGAISSLIR	95%	n+304 (+304)	36.35	25.00
3795	seq=translation; coord=1:293793122..293796213:1;<br>parent_transcript=GRMZM2G459811_T02;<br>parent_gene=GRMZM2G459811	GRMZM2G459811_P02,GRMZM2G459811_P03	TRUE	TRUE	aLNLIQYLLHNYk	95%	n+304 (+304), K+304 (+304)	28.54	25.00
3796	seq=translation; coord=1:293793122..293796213:1;<br>parent_transcript=GRMZM2G459811_T02;<br>parent_gene=GRMZM2G459811	GRMZM2G459811_P02,GRMZM2G459811_P03	TRUE	TRUE	eAALGGLLELAR	95%	n+304 (+304)	73.99	25.90
3797	seq=translation; coord=1:293793122..293796213:1;<br>parent_transcript=GRMZM2G459811_T02;<br>parent_gene=GRMZM2G459811	GRMZM2G459811_P02,GRMZM2G459811_P03	TRUE	TRUE	gLVVLPGEDAPQQPPPDVAGk	93%	n+304 (+304), K+304 (+304)	27.04	25.00
3798	seq=translation; coord=5:85252669..85257667:1;<br>parent_transcript=GRMZM2G104632_T01;<br>parent_gene=GRMZM2G104632	GRMZM2G104632_P01	TRUE	TRUE	dDIEVVAVNDPFIDAk	95%	n+304 (+304), K+304 (+304)	44.58	25.55
3799	seq=translation; coord=5:85252669..85257667:1;<br>parent_transcript=GRMZM2G104632_T01;<br>parent_gene=GRMZM2G104632	GRMZM2G104632_P01	TRUE	TRUE	gAAQNIIPSSTGAAk	95%	n+304 (+304), K+304 (+304)	58.73	25.00
3800	seq=translation; coord=5:85252669..85257667:1;<br>parent_transcript=GRMZM2G104632_T01;<br>parent_gene=GRMZM2G104632	GRMZM2G104632_P01	TRUE	TRUE	vLDLIAHMALVSAk	95%	n+304 (+304), K+304 (+304)	33.32	25.00
3801	seq=translation; coord=10:123440513..123444639:1;<br>parent_transcript=GRMZM2G134539_T01;<br>parent_gene=GRMZM2G134539	GRMZM2G134539_P01	TRUE	TRUE	eLVPTDGEHVDASVIR	95%	n+304 (+304)	48.45	25.80

3802	seq=translation; coord=10:123440513..123444639:1; parent_transcript=GRMZM2G134539_T01; parent_gene=GRMZM2G134539	GRMZM2G134539_P01	TRUE	TRUE	lLTGETISSAAPQEPVPIR	92%	n+304 (+304)	26.91	26.01
3803	seq=translation; coord=10:123440513..123444639:1; parent_transcript=GRMZM2G134539_T01; parent_gene=GRMZM2G134539	GRMZM2G134539_P01	TRUE	TRUE	nDVDAIFEQAR	95%	n+304 (+304)	35.51	25.00
3804	seq=translation; coord=10:123440513..123444639:1; parent_transcript=GRMZM2G134539_T01; parent_gene=GRMZM2G134539	GRMZM2G134539_P01	TRUE	TRUE	sAGVVVDDSQPFTSIQLR	95%	n+304 (+304)	78.29	25.01
3805	seq=translation; coord=10:123440513..123444639:1; parent_transcript=GRMZM2G134539_T01; parent_gene=GRMZM2G134539	GRMZM2G134539_P01	TRUE	TRUE	sGMLVQDPTR	92%	n+304 (+304)	29.06	25.43
3806	seq=translation; coord=10:123440513..123444639:1; parent_transcript=GRMZM2G134539_T01; parent_gene=GRMZM2G134539	GRMZM2G134539_P01	TRUE	TRUE	tLGGGPSPDESATAAPASAAPAASR	95%	n+304 (+304)	48.11	25.00
3807	seq=translation; coord=6:75510288..75518697:1; parent_transcript=GRMZM2G060870_T01; parent_gene=GRMZM2G060870	GRMZM2G060870_P01,GRMZM2G060870_P02	TRUE	TRUE	aMDFLVELFR	95%	n+304 (+304)	35.79	26.05
3808	seq=translation; coord=6:75510288..75518697:1; parent_transcript=GRMZM2G060870_T01; parent_gene=GRMZM2G060870	GRMZM2G060870_P01,GRMZM2G060870_P02	TRUE	TRUE	eNHEFLASVGLDDMk	95%	n+304 (+304), K+304 (+304)	31.68	25.00
3809	seq=translation; coord=6:75510288..75518697:1; parent_transcript=GRMZM2G060870_T01; parent_gene=GRMZM2G060870	GRMZM2G060870_P01,GRMZM2G060870_P02	TRUE	TRUE	fGAAMAIVk	95%	n+304 (+304), K+304 (+304)	29.75	25.76
3810	seq=translation; coord=6:75510288..75518697:1; parent_transcript=GRMZM2G060870_T01; parent_gene=GRMZM2G060870	GRMZM2G060870_P01,GRMZM2G060870_P02	TRUE	TRUE	qILPVLDk	95%	n+304 (+304), K+304 (+304)	36.53	25.00
3811	seq=translation; coord=8:14389329..14393515:-1; parent_transcript=GRMZM2G018177_T01; parent_gene=GRMZM2G018177	GRMZM2G018177_P01	TRUE	TRUE	dWSNVVLAYEPVWAIGTGk	95%	n+304 (+304), K+304 (+304)	32.93	25.01
3812	seq=translation; coord=8:14389329..14393515:-1; parent_transcript=GRMZM2G018177_T01; parent_gene=GRMZM2G018177	GRMZM2G018177_P01	TRUE	TRUE	iNVSPEVSESTR	95%	n+304 (+304)	38.08	25.60
3813	seq=translation; coord=8:14389329..14393515:-1; parent_transcript=GRMZM2G018177_T01; parent_gene=GRMZM2G018177	GRMZM2G018177_P01	TRUE	TRUE	qEFQVAAQNcWVk	95%	n+304 (+304), Carbamidomethyl (+57), K+304 (+304)	36.73	26.05
3814	seq=translation; coord=8:14389329..14393515:-1; parent_transcript=GRMZM2G018177_T01; parent_gene=GRMZM2G018177	GRMZM2G018177_P01	TRUE	TRUE	vAFALSQGLk	95%	n+304 (+304), K+304 (+304)	35.46	25.00
3815	seq=translation; coord=1:3000161..3000884:-1; parent_transcript=GRMZM2G137329_T01; parent_gene=GRMZM2G137329	GRMZM2G137329_P01	TRUE	TRUE	aQQGcFcQFVk	95%	n+304 (+304), Carbamidomethyl (+57), Carbamidomethyl (+57), K+304 (+304)	58.31	25.00
3816	seq=translation; coord=1:3000161..3000884:-1; parent_transcript=GRMZM2G137329_T01; parent_gene=GRMZM2G137329	GRMZM2G137329_P01	TRUE	TRUE	kVVAScGVSVPR	92%	K+304 (+304), n+304 (+304), Carbamidomethyl (+57)	26.76	25.21
3817	seq=translation; coord=1:3000161..3000884:-1; parent_transcript=GRMZM2G137329_T01; parent_gene=GRMZM2G137329	GRMZM2G137329_P01	TRUE	TRUE	vVAScGVSVPR	95%	n+304 (+304), Carbamidomethyl (+57)	47.54	25.67
3818	seq=translation; coord=4:217521316..217523853:1; parent_transcript=GRMZM2G066222_T01; parent_gene=GRMZM2G066222	GRMZM2G066222_P01,GRMZM2G132121_P02, GRMZM2G377600_P01	TRUE	TRUE	lTEGcSFR	87%	n+304 (+304), Carbamidomethyl (+57)	25.06	25.00

3819	seq=translation; coord=4:217521316..217523853:1; parent_transcript=GRMZM2G066222_T01; parent_gene=GRMZM2G066222	GRMZM2G066222_P01,GRMZM2G132121_P02, GRMZM2G377600_P01	TRUE	TRUE	lVQSPNSFFMDVk	95%	n+304 (+304), K+304 (+304)	38.99	25.76
3820	seq=translation; coord=4:217521316..217523853:1; parent_transcript=GRMZM2G066222_T01; parent_gene=GRMZM2G066222	GRMZM2G066222_P01,GRMZM2G132121_P02, GRMZM2G377600_P01	TRUE	TRUE	vLQNDIDLLNPPAELEk	95%	n+304 (+304), K+304 (+304)	48.60	25.00
3821	seq=translation; coord=8:148264071..148273363:1; parent_transcript=GRMZM2G030167_T01; parent_gene=GRMZM2G030167	GRMZM2G030167_P01	TRUE	TRUE	dLIIQGLLR	95%	n+304 (+304)	32.21	25.00
3822	seq=translation; coord=8:148264071..148273363:1; parent_transcript=GRMZM2G030167_T01; parent_gene=GRMZM2G030167	GRMZM2G030167_P01	TRUE	TRUE	iVFESTLDAR	95%	n+304 (+304)	35.41	25.00
3823	seq=translation; coord=8:148264071..148273363:1; parent_transcript=GRMZM2G030167_T01; parent_gene=GRMZM2G030167	GRMZM2G030167_P01	TRUE	TRUE	vLQAQDDLVNk	95%	n+304 (+304), K+304 (+304)	73.00	26.01
3824	seq=translation; coord=3:11529201..11534274:1; parent_transcript=GRMZM2G061830_T02; parent_gene=GRMZM2G061830	GRMZM2G061830_P02	TRUE	TRUE	aPIGEEAEGR	95%	n+304 (+304)	57.51	25.00
3825	seq=translation; coord=3:11529201..11534274:1; parent_transcript=GRMZM2G061830_T02; parent_gene=GRMZM2G061830	GRMZM2G061830_P02	TRUE	TRUE	dLGYTAEMVYk	95%	n+304 (+304), K+304 (+304)	47.37	25.00
3826	seq=translation; coord=3:11529201..11534274:1; parent_transcript=GRMZM2G061830_T02; parent_gene=GRMZM2G061830	GRMZM2G061830_P02	TRUE	TRUE	gYFDLLIk	89%	n+304 (+304), K+304 (+304)	25.20	25.00
3827	seq=translation; coord=3:11529201..11534274:1; parent_transcript=GRMZM2G061830_T02; parent_gene=GRMZM2G061830	GRMZM2G061830_P02	TRUE	TRUE	lGLDVAScLITR	95%	n+304 (+304), Carbamidomethyl (+57)	41.37	26.06
3828	seq=translation; coord=3:11529201..11534274:1; parent_transcript=GRMZM2G061830_T02; parent_gene=GRMZM2G061830	GRMZM2G061830_P02	TRUE	TRUE	nPNDNTQVSLIYANVSPDDILLk	95%	n+304 (+304), K+304 (+304)	29.74	25.56
3829	seq=translation; coord=3:11529201..11534274:1; parent_transcript=GRMZM2G061830_T02; parent_gene=GRMZM2G061830	GRMZM2G061830_P02	TRUE	TRUE	vYPDGk	90%	n+304 (+304), K+304 (+304)	29.16	26.92
3830	seq=translation; coord=9:124034244..124040497:-1; parent_transcript=GRMZM2G162968_T01; parent_gene=GRMZM2G162968	GRMZM2G162968_P01	TRUE	TRUE	iTQQEFTEMAWQAIVSSPEVAk	95%	n+304 (+304), K+304 (+304)	36.11	25.72
3831	seq=translation; coord=9:124034244..124040497:-1; parent_transcript=GRMZM2G162968_T01; parent_gene=GRMZM2G162968	GRMZM2G162968_P01	TRUE	TRUE	lIALDMGALIAGAk	95%	n+304 (+304), K+304 (+304)	31.89	25.00
3832	seq=translation; coord=7:1284259..1285978:1; parent_transcript=GRMZM2G120652_T01; parent_gene=GRMZM2G120652	GRMZM2G120652_P01	TRUE	TRUE	gGVIMDVVTPEQAR	95%	n+304 (+304)	51.90	25.48
3833	seq=translation; coord=7:1284259..1285978:1; parent_transcript=GRMZM2G120652_T01; parent_gene=GRMZM2G120652	GRMZM2G120652_P01	TRUE	TRUE	iAAPYDLVMQTk	95%	n+304 (+304), K+304 (+304)	50.66	26.33
3834	seq=translation; coord=7:1284259..1285978:1; parent_transcript=GRMZM2G120652_T01; parent_gene=GRMZM2G120652	GRMZM2G120652_P01	TRUE	TRUE	lAEEAGAcAVMALER	95%	n+304 (+304), Carbamidomethyl (+57)	55.37	25.00
3835	seq=translation; coord=7:1284259..1285978:1; parent_transcript=GRMZM2G120652_T01; parent_gene=GRMZM2G120652	GRMZM2G120652_P01	TRUE	TRUE	mSDPGLIR	87%	n+304 (+304)	25.75	25.72
3836	seq=translation; coord=7:1284259..1285978:1; parent_transcript=GRMZM2G120652_T01; parent_gene=GRMZM2G120652	GRMZM2G120652_P01	TRUE	TRUE	sMDDDEVFAYAk	95%	n+304 (+304), K+304 (+304)	42.84	25.00

3837	seq=translation; coord=4:69841345..69843586:1; parent_transcript=GRMZM2G100225_T01; parent_gene=GRMZM2G100225	GRMZM2G100225_P01,GRMZM2G100225_P02	TRUE	TRUE	aAAVPESVLR	92%	n+304 (+304)	30.68	27.26
3838	seq=translation; coord=6:157770836..157775279:1; parent_transcript=GRMZM2G424053_T02; parent_gene=GRMZM2G424053	GRMZM2G424053_P02	TRUE	TRUE	aMAPIFADMAk	95%	n+304 (+304), K+304 (+304)	44.51	25.43
3839	seq=translation; coord=6:157770836..157775279:1; parent_transcript=GRMZM2G424053_T02; parent_gene=GRMZM2G424053	GRMZM2G424053_P02	TRUE	TRUE	tIAEQFSVEAMPTFLFMR	95%	n+304 (+304)	33.21	25.00
3840	seq=translation; coord=6:157770836..157775279:1; parent_transcript=GRMZM2G424053_T02; parent_gene=GRMZM2G424053	GRMZM2G424053_P02	TRUE	TRUE	vDVDEMk	94%	n+304 (+304), K+304 (+304)	30.63	25.19
3841	seq=translation; coord=2:190158113..190161171:-1; parent_transcript=GRMZM2G365374_T01; parent_gene=GRMZM2G365374	GRMZM2G365374_P01	TRUE	TRUE	eIDEVLLVGGMTR	91%	n+304 (+304)	27.82	25.00
3842	seq=translation; coord=2:190158113..190161171:-1; parent_transcript=GRMZM2G365374_T01; parent_gene=GRMZM2G365374	GRMZM2G365374_P01	TRUE	TRUE	eIESAVSDLR	89%	n+304 (+304)	26.79	25.00
3843	seq=translation; coord=2:190158113..190161171:-1; parent_transcript=GRMZM2G365374_T01; parent_gene=GRMZM2G365374	GRMZM2G365374_P01	TRUE	TRUE	fESLVSNLIER	95%	n+304 (+304)	37.26	25.22
3844	seq=translation; coord=1:167851430..167863929:-1; parent_transcript=GRMZM2G130034_T01; parent_gene=GRMZM2G130034	GRMZM2G130034_P01,GRMZM2G130034_P02	TRUE	TRUE	eDAVGNIFGR	95%	n+304 (+304)	31.30	25.00
3845	seq=translation; coord=1:167851430..167863929:-1; parent_transcript=GRMZM2G130034_T01; parent_gene=GRMZM2G130034	GRMZM2G130034_P01,GRMZM2G130034_P02	TRUE	TRUE	lMAGIEELAQSLR	94%	n+304 (+304)	31.44	26.19
3846	seq=translation; coord=1:167851430..167863929:-1; parent_transcript=GRMZM2G130034_T01; parent_gene=GRMZM2G130034	GRMZM2G130034_P01,GRMZM2G130034_P02	TRUE	TRUE	lNLEDLHSVFLk	95%	n+304 (+304), K+304 (+304)	30.00	25.47
3847	seq=translation; coord=1:167851430..167863929:-1; parent_transcript=GRMZM2G130034_T01; parent_gene=GRMZM2G130034	GRMZM2G130034_P01,GRMZM2G130034_P02	TRUE	TRUE	sLEVIMFTSEEPTR	95%	n+304 (+304)	45.40	25.00
3848	seq=translation; coord=1:233560736..233562244:-1; parent_transcript=GRMZM2G001850_T01; parent_gene=GRMZM2G001850	GRMZM2G001850_P01	TRUE	TRUE	gFDTSGGEDQIR	95%	n+304 (+304)	38.72	25.00
3849	seq=translation; coord=1:233560736..233562244:-1; parent_transcript=GRMZM2G001850_T01; parent_gene=GRMZM2G001850	GRMZM2G001850_P01	TRUE	TRUE	iDMATER	94%	n+304 (+304)	31.32	25.00
3850	seq=translation; coord=6:43621161..43627397:1; parent_transcript=GRMZM2G025214_T02; parent_gene=GRMZM2G025214	GRMZM2G025214_P02	TRUE	TRUE	dSQLAAQFQEIPLDLk	95%	n+304 (+304), K+304 (+304)	38.80	25.91
3851	seq=translation; coord=6:43621161..43627397:1; parent_transcript=GRMZM2G025214_T02; parent_gene=GRMZM2G025214	GRMZM2G025214_P02	TRUE	TRUE	vIGTLLGSLLSDGTVHVR	95%	n+304 (+304)	71.61	25.00
3852	seq=translation; coord=5:1033053..1037664:1; parent_transcript=GRMZM2G313678_T01; parent_gene=GRMZM2G313678	GRMZM2G313678_P01,GRMZM2G313678_P02	TRUE	TRUE	aFLMPIEDVFSIQGR	95%	n+304 (+304)	36.16	25.00
3853	seq=translation; coord=5:10781545..10785536:1; parent_transcript=GRMZM2G145854_T01; parent_gene=GRMZM2G145854	GRMZM2G145854_P01	TRUE	TRUE	aLSEVAGALLPYDSLSAVR	94%	n+304 (+304)	28.83	25.42
3854	seq=translation; coord=5:10781545..10785536:1; parent_transcript=GRMZM2G145854_T01; parent_gene=GRMZM2G145854	GRMZM2G145854_P01	TRUE	TRUE	aNVILPSSAFSEk	95%	n+304 (+304), K+304 (+304)	44.48	25.38

	seq=translation; coord=5:10781545..10785536:1;								
3855	parent_transcript=GRMZM2G145854_T01; parent_gene=GRMZM2G145854	GRMZM2G145854_P01	TRUE	TRUE	fATEVAGVQDLGMLGR	95%	n+304 (+304)	38.85	25.34
3856	seq=translation; coord=5:10781545..10785536:1; parent_transcript=GRMZM2G145854_T01; parent_gene=GRMZM2G145854	GRMZM2G145854_P01	TRUE	TRUE	gTETIDVTDAVGSNIR	95%	n+304 (+304)	44.83	25.00
3857	seq=translation; coord=5:10781545..10785536:1; parent_transcript=GRMZM2G145854_T01; parent_gene=GRMZM2G145854	GRMZM2G145854_P01	TRUE	TRUE	vELPPNPEDALEVFVDGHAVR	95%	n+304 (+304)	53.89	25.00
3858	seq=translation; coord=9:94249084..94255465:1; parent_transcript=AC211394.4_FGT004; parent_gene=AC211394.4_FG004	AC211394.4_FGP004	TRUE	TRUE	fYNPQPDYSAFR	95%	n+304 (+304)	38.43	25.00
3859	seq=translation; coord=9:94249084..94255465:1; parent_transcript=AC211394.4_FGT004; parent_gene=AC211394.4_FG004	AC211394.4_FGP004	TRUE	TRUE	sAPVYITVGDGGNQEGLASR	95%	n+304 (+304)	90.86	25.00
3860	seq=translation; coord=9:94249084..94255465:1; parent_transcript=AC211394.4_FGT004; parent_gene=AC211394.4_FG004	AC211394.4_FGP004	TRUE	TRUE	tHAIYQWNR	95%	n+304 (+304)	29.21	25.00
3861	seq=translation; coord=9:94249084..94255465:1; parent_transcript=AC211394.4_FGT004; parent_gene=AC211394.4_FG004	AC211394.4_FGP004	TRUE	TRUE	vDLVFAGHVHAYER	95%	n+304 (+304)	27.75	25.67
3862	seq=translation; coord=6:115546691..115548383:1; parent_transcript=GRMZM2G156310_T01; parent_gene=GRMZM2G156310	GRMZM2G156310_P01	TRUE	TRUE	aLEWVAASGGDPWLSR	94%	n+304 (+304)	27.89	25.00
3863	seq=translation; coord=6:115546691..115548383:1; parent_transcript=GRMZM2G156310_T01; parent_gene=GRMZM2G156310	GRMZM2G156310_P01	TRUE	TRUE	dVVIDSDAGLYVR	95%	n+304 (+304)	48.26	25.00
3864	seq=translation; coord=6:115546691..115548383:1; parent_transcript=GRMZM2G156310_T01; parent_gene=GRMZM2G156310	GRMZM2G156310_P01	TRUE	TRUE	vLVcGAELDSLLPR	95%	n+304 (+304), Carbamidomethyl (+57)	57.37	25.76
3865	seq=translation; coord=10:96759871..96761276:-1; parent_transcript=GRMZM2G330635_T01; parent_gene=GRMZM2G330635	GRMZM2G330635_P01	TRUE	TRUE	aVFGLPPLRP	93%	n+304 (+304)	26.75	25.02
3866	seq=translation; coord=10:96759871..96761276:-1; parent_transcript=GRMZM2G330635_T01; parent_gene=GRMZM2G330635	GRMZM2G330635_P01	TRUE	TRUE	eLTLLGLWASPFVLR	95%	n+304 (+304)	38.20	25.00
3867	seq=translation; coord=10:96759871..96761276:-1; parent_transcript=GRMZM2G330635_T01; parent_gene=GRMZM2G330635	GRMZM2G330635_P01	TRUE	TRUE	qAVAALETLEQAFR	95%	n+304 (+304)	64.71	25.77
3868	seq=translation; coord=10:96759871..96761276:-1; parent_transcript=GRMZM2G330635_T01; parent_gene=GRMZM2G330635	GRMZM2G330635_P01	TRUE	TRUE	tAEEMAEAAR	94%	n+304 (+304)	29.01	25.00
3869	seq=translation; coord=6:107821435..107825807:-1; parent_transcript=GRMZM2G135498_T01; parent_gene=GRMZM2G135498	GRMZM2G135498_P01	TRUE	TRUE	dGFVMGEGAGVLVMESLEHAMk	95%	n+304 (+304), K+304 (+304)	51.45	25.17
3870	seq=translation; coord=6:107821435..107825807:-1; parent_transcript=GRMZM2G135498_T01; parent_gene=GRMZM2G135498	GRMZM2G135498_P01	TRUE	TRUE	lLVGESGIGPIDR	95%	n+304 (+304)	42.89	25.91
3871	seq=translation; coord=4:201542946..201547734:-1; parent_transcript=GRMZM2G381744_T01; parent_gene=GRMZM2G381744	GRMZM2G381744_P01	TRUE	TRUE	gSTDSILDDLER	95%	n+304 (+304)	78.55	25.00
3872	seq=translation; coord=4:201542946..201547734:-1; parent_transcript=GRMZM2G381744_T01; parent_gene=GRMZM2G381744	GRMZM2G381744_P01	TRUE	TRUE	nPANFNVDNVR	95%	n+304 (+304)	49.50	25.00

3873	seq=translation; coord=4:201542946..201547734:-1; parent_transcript=GRMZM2G381744_T01; parent_gene=GRMZM2G381744	GRMZM2G381744_P01	TRUE	TRUE	tIEILEDLVEk	95%	n+304 (+304), K+304 (+304)	35.26	26.06
3874	seq=translation; coord=4:201542946..201547734:-1; parent_transcript=GRMZM2G381744_T01; parent_gene=GRMZM2G381744	GRMZM2G381744_P01	TRUE	TRUE	vIVSGAAVGDMALHFcER	95%	n+304 (+304), Carbamidomethyl (+57)	48.89	25.00
3875	seq=translation; coord=1:247547660..247549896:1; parent_transcript=GRMZM2G027451_T02; parent_gene=GRMZM2G027451	GRMZM2G027451_P02,GRMZM2G027451_P03, GRMZM2G027451_P04,GRMZM2G087233_P01	TRUE	TRUE	dAFHLR	91%	n+304 (+304)	25.29	25.00
3876	seq=translation; coord=1:247547660..247549896:1; parent_transcript=GRMZM2G027451_T02; parent_gene=GRMZM2G027451	GRMZM2G027451_P02,GRMZM2G027451_P03, GRMZM2G027451_P04,GRMZM2G087233_P01	TRUE	TRUE	eNVSSEALEAAR	95%	n+304 (+304)	44.59	25.00
3877	seq=translation; coord=1:247547660..247549896:1; parent_transcript=GRMZM2G027451_T02; parent_gene=GRMZM2G027451	GRMZM2G027451_P02,GRMZM2G027451_P03, GRMZM2G027451_P04,GRMZM2G087233_P01	TRUE	TRUE	vDIGQVLLSVR	95%	n+304 (+304)	33.93	26.22
3878	seq=translation; coord=4:39092198..39097412:-1; parent_transcript=GRMZM2G158153_T01; parent_gene=GRMZM2G158153	GRMZM2G158153_P01,GRMZM2G158153_P02	TRUE	TRUE	gVSPQPGGGAAAyGAGGQWTA	93%	n+304 (+304), iTRAQ8plex (+304)	26.32	25.00
3879	seq=translation; coord=4:39092198..39097412:-1; parent_transcript=GRMZM2G158153_T01; parent_gene=GRMZM2G158153	GRMZM2G158153_P01,GRMZM2G158153_P02	TRUE	TRUE	iQLIPQHPPEGVTLTER	94%	n+304 (+304)	28.73	25.58
3880	seq=translation; coord=4:39092198..39097412:-1; parent_transcript=GRMZM2G158153_T01; parent_gene=GRMZM2G158153	GRMZM2G158153_P01,GRMZM2G158153_P02	TRUE	TRUE	sVIAEAEAGGSPALIAR	95%	n+304 (+304)	77.70	25.84
3881	seq=translation; coord=1:99538428..99539066:-1; parent_transcript=AC194914.3_FGT002; parent_gene=AC194914.3_FG002	AC194914.3_FGP002	TRUE	TRUE	eVSVISGLPASTSTER	95%	n+304 (+304)	72.80	25.49
3882	seq=translation; coord=1:99538428..99539066:-1; parent_transcript=AC194914.3_FGT002; parent_gene=AC194914.3_FG002	AC194914.3_FGP002	TRUE	TRUE	iHAPPEAVWAVVR	95%	n+304 (+304)	54.03	25.47
3883	seq=translation; coord=1:99538428..99539066:-1; parent_transcript=AC194914.3_FGT002; parent_gene=AC194914.3_FG002	AC194914.3_FGP002	TRUE	TRUE	lDLLDDAAR	94%	n+304 (+304)	29.70	25.77
3884	seq=translation; coord=1:99538428..99539066:-1; parent_transcript=AC194914.3_FGT002; parent_gene=AC194914.3_FG002	AC194914.3_FGP002	TRUE	TRUE	lFADTVIR	93%	n+304 (+304)	29.61	25.00
3885	seq=translation; coord=4:236107257..236109295:-1; parent_transcript=GRMZM2G051208_T01; parent_gene=GRMZM2G051208	GRMZM2G051208_P01	TRUE	TRUE	fcEEEDGH	95%	n+304 (+304), Carbamidomethyl (+57)	33.60	25.00
3886	seq=translation; coord=4:236107257..236109295:-1; parent_transcript=GRMZM2G051208_T01; parent_gene=GRMZM2G051208	GRMZM2G051208_P01	TRUE	TRUE	lSDSGELSSLMDGDk	95%	n+304 (+304), K+304 (+304)	48.00	25.00
3887	seq=translation; coord=5:168671806..168673715:1; parent_transcript=GRMZM2G113332_T01; parent_gene=GRMZM2G113332	GRMZM2G113332_P01	TRUE	TRUE	mEGVETFDIDLk	95%	n+304 (+304), K+304 (+304)	49.72	25.09
3888	seq=translation; coord=5:168671806..168673715:1; parent_transcript=GRMZM2G113332_T01; parent_gene=GRMZM2G113332	GRMZM2G113332_P01	TRUE	TRUE	vAMScEGcAGAVR	95%	n+304 (+304), Carbamidomethyl (+57), Carbamidomethyl (+57)	44.88	25.00
3889	seq=translation; coord=4:194761789..194765861:1; parent_transcript=GRMZM2G149150_T01; parent_gene=GRMZM2G149150	GRMZM2G149150_P01	TRUE	TRUE	aGSAAESSENEAESQSTSADR	90%	n+304 (+304)	25.28	25.00
3890	seq=translation; coord=4:194761789..194765861:1; parent_transcript=GRMZM2G149150_T01; parent_gene=GRMZM2G149150	GRMZM2G149150_P01	TRUE	TRUE	lAEIALTDPk	92%	n+304 (+304), K+304 (+304)	27.93	25.44

3891	seq=translation; coord=4:194761789..194765861:1; parent_transcript=GRMZM2G149150_T01; parent_gene=GRMZM2G149150	GRMZM2G149150_P01	TRUE	TRUE	vQVLQTEATTLSAQLTMLQR	95%	n+304 (+304)	72.17	25.17
3892	seq=translation; coord=3:18219856..18221974:1; parent_transcript=AC233872.1_FGT003; parent_gene=AC233872.1_FG003	AC233872.1_FGP003	TRUE	TRUE	dMIAYEGPDFNDLDENLQR	95%	n+304 (+304)	80.35	25.00
3893	seq=translation; coord=3:18219856..18221974:1; parent_transcript=AC233872.1_FGT003; parent_gene=AC233872.1_FG003	AC233872.1_FGP003	TRUE	TRUE	dMIAyEGPDFNDLDENLQR	95%	n+304 (+304), iTRAQ8plex (+304)	40.53	25.00
3894	seq=translation; coord=9:25922353..25927460:1; parent_transcript=GRMZM2G083716_T01; parent_gene=GRMZM2G083716	GRMZM2G083716_P01,GRMZM2G083716_P02, GRMZM2G083716_P03,GRMZM2G083716_P04	TRUE	TRUE	aLVEELR	95%	n+304 (+304)	36.47	25.09
3895	seq=translation; coord=9:25922353..25927460:1; parent_transcript=GRMZM2G083716_T01; parent_gene=GRMZM2G083716	GRMZM2G083716_P01,GRMZM2G083716_P02, GRMZM2G083716_P03,GRMZM2G083716_P04	TRUE	TRUE	dLINVLEEAIR	95%	n+304 (+304)	47.80	26.07
3896	seq=translation; coord=7:86433388..86439128:-1; parent_transcript=GRMZM2G473001_T01; parent_gene=GRMZM2G473001	GRMZM2G473001_P01	TRUE	TRUE	aLLDEMAVVATEEYR	95%	n+304 (+304)	82.37	25.00
3897	seq=translation; coord=7:86433388..86439128:-1; parent_transcript=GRMZM2G473001_T01; parent_gene=GRMZM2G473001	GRMZM2G473001_P01	TRUE	TRUE	fLDILQDLHGDDLk	95%	n+304 (+304), K+304 (+304)	49.73	25.00
3898	seq=translation; coord=7:86433388..86439128:-1; parent_transcript=GRMZM2G473001_T01; parent_gene=GRMZM2G473001	GRMZM2G473001_P01	TRUE	TRUE	lATPETEYGR	95%	n+304 (+304)	42.65	25.00
3899	seq=translation; coord=7:86433388..86439128:-1; parent_transcript=GRMZM2G473001_T01; parent_gene=GRMZM2G473001	GRMZM2G473001_P01	TRUE	TRUE	lNPGSEYAPGLEDTLILTMk	90%	n+304 (+304), K+304 (+304)	26.26	25.76
3900	seq=translation; coord=3:179988293..179993427:-1; parent_transcript=GRMZM2G014136_T01; parent_gene=GRMZM2G014136	GRMZM2G014136_P01	TRUE	TRUE	eNLLNLVADVLPDGR	95%	n+304 (+304)	50.71	25.72
3901	seq=translation; coord=3:179988293..179993427:-1; parent_transcript=GRMZM2G014136_T01; parent_gene=GRMZM2G014136	GRMZM2G014136_P01	TRUE	TRUE	fAAFLSPLTLGR	95%	n+304 (+304)	37.16	26.65
3902	seq=translation; coord=3:179988293..179993427:-1; parent_transcript=GRMZM2G014136_T01; parent_gene=GRMZM2G014136	GRMZM2G014136_P01	TRUE	TRUE	gIETIATYDSETR	95%	n+304 (+304)	47.03	25.00
3903	seq=translation; coord=5:14853348..14857185:-1; parent_transcript=GRMZM2G066191_T01; parent_gene=GRMZM2G066191	GRMZM2G066191_P01,GRMZM2G066191_P03	TRUE	TRUE	aVLMDLEPGTMESIR	95%	n+304 (+304)	62.10	25.00
3904	seq=translation; coord=5:14853348..14857185:-1; parent_transcript=GRMZM2G066191_T01; parent_gene=GRMZM2G066191	GRMZM2G066191_P01,GRMZM2G066191_P03	TRUE	TRUE	fWEVIcGEHcVDSTGR	95%	n+304 (+304), Carbamidomethyl (+57), Carbamidomethyl (+57)	66.59	25.00
3905	seq=translation; coord=5:14853348..14857185:-1; parent_transcript=GRMZM2G066191_T01; parent_gene=GRMZM2G066191	GRMZM2G066191_P01,GRMZM2G066191_P03	TRUE	TRUE	ySGTSSQQLELER	87%	n+304 (+304)	25.62	25.00
3906	seq=translation; coord=7:25903581..25911265:1; parent_transcript=GRMZM2G016958_T01; parent_gene=GRMZM2G016958	GRMZM2G016958_P01	TRUE	TRUE	aDMDALPLQELVDWEHk	95%	n+304 (+304), K+304 (+304)	28.89	25.02
3907	seq=translation; coord=7:25903581..25911265:1; parent_transcript=GRMZM2G016958_T01; parent_gene=GRMZM2G016958	GRMZM2G016958_P01	TRUE	TRUE	eIDPLQAAVVSVTFMk	92%	n+304 (+304), K+304 (+304)	26.68	25.00
3908	seq=translation; coord=7:25903581..25911265:1; parent_transcript=GRMZM2G016958_T01; parent_gene=GRMZM2G016958	GRMZM2G016958_P01	TRUE	TRUE	eVAEAMLGQDk	95%	n+304 (+304), K+304 (+304)	48.46	25.74

3909	seq=translation; coord=7:25903581..25911265:1; parent_transcript=GRMZM2G016958_T01; parent_gene=GRMZM2G016958	GRMZM2G016958_P01	TRUE	TRUE	gGDAYNVIPESASFGGTFR	95%	n+304 (+304)	76.08	25.00
3910	seq=translation; coord=7:25903581..25911265:1; parent_transcript=GRMZM2G016958_T01; parent_gene=GRMZM2G016958	GRMZM2G016958_P01	TRUE	TRUE	gGHAAGPQDAVDPIVAASSAIVSLQLLVAR	95%	n+304 (+304)	44.37	25.00
3911	seq=translation; coord=7:25903581..25911265:1; parent_transcript=GRMZM2G016958_T01; parent_gene=GRMZM2G016958	GRMZM2G016958_P01	TRUE	TRUE	lRPYPATVNDEGMYR	95%	n+304 (+304)	55.24	25.00
3912	seq=translation; coord=7:25903581..25911265:1; parent_transcript=GRMZM2G016958_T01; parent_gene=GRMZM2G016958	GRMZM2G016958_P01	TRUE	TRUE	lVFQPGEEGYGGAYHVLR	95%	n+304 (+304)	51.90	25.00
3913	seq=translation; coord=10:93684085..93687059:-1; parent_transcript=GRMZM5G833699_T01; parent_gene=GRMZM5G833699	GRMZM5G833699_P01	TRUE	TRUE	aILFVPR	93%	n+304 (+304)	29.27	25.91
3914	seq=translation; coord=10:93684085..93687059:-1; parent_transcript=GRMZM5G833699_T01; parent_gene=GRMZM5G833699	GRMZM5G833699_P01	TRUE	TRUE	aVENSPFLER	95%	n+304 (+304)	34.12	25.00
3915	seq=translation; coord=10:93684085..93687059:-1; parent_transcript=GRMZM5G833699_T01; parent_gene=GRMZM5G833699	GRMZM5G833699_P01	TRUE	TRUE	dIYYITGESR	90%	n+304 (+304)	26.56	25.00
3916	seq=translation; coord=10:93684085..93687059:-1; parent_transcript=GRMZM5G833699_T01; parent_gene=GRMZM5G833699	GRMZM5G833699_P01	TRUE	TRUE	eVSHEWVQINk	95%	n+304 (+304), K+304 (+304)	30.61	25.93
3917	seq=translation; coord=10:93684085..93687059:-1; parent_transcript=GRMZM5G833699_T01; parent_gene=GRMZM5G833699	GRMZM5G833699_P01	TRUE	TRUE	gVVDSDDLPLNISR	95%	n+304 (+304)	49.60	25.00
3918	seq=translation; coord=10:93684085..93687059:-1; parent_transcript=GRMZM5G833699_T01; parent_gene=GRMZM5G833699	GRMZM5G833699_P01	TRUE	TRUE	tLSIIDSGVGMTk	95%	n+304 (+304), K+304 (+304)	53.44	25.85
3919	seq=translation; coord=7:39873483..39874398:1; parent_transcript=GRMZM5G896560_T01; parent_gene=GRMZM5G896560	GRMZM5G896560_P01	TRUE	TRUE	gFGFGFGk	95%	n+304 (+304), K+304 (+304)	31.87	25.08
3920	seq=translation; coord=7:39873483..39874398:1; parent_transcript=GRMZM5G896560_T01; parent_gene=GRMZM5G896560	GRMZM5G896560_P01	TRUE	TRUE	gGGIGHGMGGGFGk	95%	n+304 (+304), K+304 (+304)	40.70	25.12
3921	seq=translation; coord=7:39873483..39874398:1; parent_transcript=GRMZM5G896560_T01; parent_gene=GRMZM5G896560	GRMZM5G896560_P01	TRUE	TRUE	gGGIGHGmGGGFGk	94%	n+304 (+304), Oxidation (+16), K+304 (+304)	28.51	25.00
3922	seq=translation; coord=7:39873483..39874398:1; parent_transcript=GRMZM5G896560_T01; parent_gene=GRMZM5G896560	GRMZM5G896560_P01	TRUE	TRUE	gGGLGGGIGHGLGGGYGk	95%	n+304 (+304), K+304 (+304)	43.76	25.24
3923	seq=translation; coord=7:39873483..39874398:1; parent_transcript=GRMZM5G896560_T01; parent_gene=GRMZM5G896560	GRMZM5G896560_P01	TRUE	TRUE	gGGLGGGYGk	95%	n+304 (+304), K+304 (+304)	67.06	25.83
3924	seq=translation; coord=2:220539596..220543690:1; parent_transcript=GRMZM2G163421_T01; parent_gene=GRMZM2G163421	GRMZM2G163421_P01	TRUE	TRUE	eFSGTEFTNFMELAEk	95%	n+304 (+304), K+304 (+304)	52.68	25.00
3925	seq=translation; coord=2:220539596..220543690:1; parent_transcript=GRMZM2G163421_T01; parent_gene=GRMZM2G163421	GRMZM2G163421_P01	TRUE	TRUE	gDASVEGPLIR	95%	n+304 (+304)	34.46	26.50
3926	seq=translation; coord=2:220539596..220543690:1; parent_transcript=GRMZM2G163421_T01; parent_gene=GRMZM2G163421	GRMZM2G163421_P01	TRUE	TRUE	iYIVGIFk	95%	n+304 (+304), K+304 (+304)	34.85	25.00

3927	seq=translation; coord=2:220539596..220543690:1; parent_transcript=GRMZM2G163421_T01; parent_gene=GRMZM2G163421	GRMZM2G163421_P01	TRUE	TRUE	vVVADNIHDVVFk	95%	n+304 (+304), K+304 (+304)	38.44	25.00
3928	seq=translation; coord=10:119822322..119823346:-1; parent_transcript=GRMZM2G096695_T01; parent_gene=GRMZM2G096695	GRMZM2G096695_P01	TRUE	TRUE	vYPEGHGEEFVEIGAGDLVVFPk	95%	n+304 (+304), K+304 (+304)	41.36	25.38
3929	seq=translation; coord=4:65004377..65017210:-1; parent_transcript=GRMZM2G002440_T01; parent_gene=GRMZM2G002440	GRMZM2G002440_P01,GRMZM2G002440_P02	TRUE	TRUE	sLLDVADNLSR	95%	n+304 (+304)	41.49	25.74
3930	seq=translation; coord=4:65004377..65017210:-1; parent_transcript=GRMZM2G002440_T01; parent_gene=GRMZM2G002440	GRMZM2G002440_P01,GRMZM2G002440_P02	TRUE	TRUE	sVGASTGASSEANNVPGTEk	95%	n+304 (+304), K+304 (+304)	54.40	25.17
3931	seq=translation; coord=4:65004377..65017210:-1; parent_transcript=GRMZM2G002440_T01; parent_gene=GRMZM2G002440	GRMZM2G002440_P01,GRMZM2G002440_P02	TRUE	TRUE	sYAEMENVLAR	95%	n+304 (+304)	40.41	25.00
3932	seq=translation; coord=4:65004377..65017210:-1; parent_transcript=GRMZM2G002440_T01; parent_gene=GRMZM2G002440	GRMZM2G002440_P01,GRMZM2G002440_P02	TRUE	TRUE	vGYMLHDR	95%	n+304 (+304)	44.46	25.00
3933	seq=translation; coord=4:65004377..65017210:-1; parent_transcript=GRMZM2G002440_T01; parent_gene=GRMZM2G002440	GRMZM2G002440_P01,GRMZM2G002440_P02	TRUE	TRUE	vLRPAEVGVTEGGPTEEEPEE	95%	n+304 (+304)	43.16	25.00
3934	seq=translation; coord=2:207032350..207038547:1; parent_transcript=GRMZM2G039251_T01; parent_gene=GRMZM2G039251	GRMZM2G039251_P01,GRMZM2G072054_P01	TRUE	TRUE	eAGVTVVESPAk	95%	n+304 (+304), K+304 (+304)	48.44	26.40
3935	seq=translation; coord=2:207032350..207038547:1; parent_transcript=GRMZM2G039251_T01; parent_gene=GRMZM2G039251	GRMZM2G039251_P01,GRMZM2G072054_P01	TRUE	TRUE	iGSTMFEIFk	95%	n+304 (+304), K+304 (+304)	38.53	25.43
3936	seq=translation; coord=2:207032350..207038547:1; parent_transcript=GRMZM2G039251_T01; parent_gene=GRMZM2G039251	GRMZM2G039251_P01,GRMZM2G072054_P01	TRUE	TRUE	vIcQGITGk	91%	n+304 (+304), Carbamidomethyl (+57), K+304 (+304)	29.32	25.93
3937	seq=translation; coord=7:116285614..116288886:-1; parent_transcript=GRMZM2G160770_T01; parent_gene=GRMZM2G160770	GRMZM2G160770_P01	TRUE	TRUE	fGNVEAGDQLQER	95%	n+304 (+304)	68.61	25.00
3938	seq=translation; coord=7:116285614..116288886:-1; parent_transcript=GRMZM2G160770_T01; parent_gene=GRMZM2G160770	GRMZM2G160770_P01	TRUE	TRUE	gALEAEMDAIIAR	95%	n+304 (+304)	56.18	25.63
3939	seq=translation; coord=7:116285614..116288886:-1; parent_transcript=GRMZM2G160770_T01; parent_gene=GRMZM2G160770	GRMZM2G160770_P01	TRUE	TRUE	gLLGcHFR	95%	n+304 (+304), Carbamidomethyl (+57)	48.50	26.25
3940	seq=translation; coord=7:116285614..116288886:-1; parent_transcript=GRMZM2G160770_T01; parent_gene=GRMZM2G160770	GRMZM2G160770_P01	TRUE	TRUE	sDIDIPNVLAQR	95%	n+304 (+304)	33.93	26.55
3941	seq=translation; coord=1:296201923..296204120:1; parent_transcript=GRMZM5G874478_T01; parent_gene=GRMZM5G874478	GRMZM5G874478_P01,GRMZM5G874478_P02, GRMZM5G874478_P03	TRUE	TRUE	cFIGNLSWSTTDESLk	95%	Carbamidomethyl (+57), n+304 (+304), K+304 (+304)	65.28	25.00
3942	seq=translation; coord=1:296201923..296204120:1; parent_transcript=GRMZM5G874478_T01; parent_gene=GRMZM5G874478	GRMZM5G874478_P01,GRMZM5G874478_P02, GRMZM5G874478_P03	TRUE	TRUE	fGNLTEAk	95%	n+304 (+304), K+304 (+304)	34.37	26.62
3943	seq=translation; coord=1:296201923..296204120:1; parent_transcript=GRMZM5G874478_T01; parent_gene=GRMZM5G874478	GRMZM5G874478_P01,GRMZM5G874478_P02, GRMZM5G874478_P03	TRUE	TRUE	gFGFVTFDEk	95%	n+304 (+304), K+304 (+304)	50.44	25.00
3944	seq=translation; coord=2:189276282..189278019:-1; parent_transcript=GRMZM2G004138_T01; parent_gene=GRMZM2G004138	GRMZM2G004138_P01	TRUE	TRUE	eFNAAVAADPR	95%	n+304 (+304)	48.25	25.00

3945	seq=translation; coord=2:189276282..189278019:-1; parent_transcript=GRMZM2G004138_T01; parent_gene=GRMZM2G004138	GRMZM2G004138_P01	TRUE	TRUE	sQPLYQYILESTVFPR	95%	n+304 (+304)	60.61	25.69
3946	seq=translation; coord=2:189276282..189278019:-1; parent_transcript=GRMZM2G004138_T01; parent_gene=GRMZM2G004138	GRMZM2G004138_P01	TRUE	TRUE	vGLALPVLDQMVAEEGNk	95%	n+304 (+304), K+304 (+304)	34.17	25.00
3947	seq=translation; coord=1:204681269..204683434:-1; parent_transcript=GRMZM2G057608_T01; parent_gene=GRMZM2G057608	GRMZM2G057608_P01,GRMZM2G057608_P04, GRMZM2G057608_P05,GRMZM2G084868_P02, GRMZM2G084868_P03,GRMZM2G139349_P01	TRUE	TRUE	qITPSILSER	95%	n+304 (+304)	40.43	26.33
3948	seq=translation; coord=1:204681269..204683434:-1; parent_transcript=GRMZM2G057608_T01; parent_gene=GRMZM2G057608	GRMZM2G057608_P01,GRMZM2G057608_P04, GRMZM2G057608_P05,GRMZM2G084868_P02, GRMZM2G084868_P03,GRMZM2G139349_P01	TRUE	TRUE	vNNAVLFDQATYDk	95%	n+304 (+304), K+304 (+304)	50.76	25.79
3949	seq=translation; coord=4:241233055..241239106:-1; parent_transcript=GRMZM2G134889_T01; parent_gene=GRMZM2G134889	GRMZM2G134889_P01	TRUE	TRUE	aSAIAGFVSANk	95%	n+304 (+304), K+304 (+304)	36.67	25.67
3950	seq=translation; coord=4:241233055..241239106:-1; parent_transcript=GRMZM2G134889_T01; parent_gene=GRMZM2G134889	GRMZM2G134889_P01	TRUE	TRUE	aVLAFLDTLSGAHSDELAAASR	95%	n+304 (+304)	61.28	25.25
3951	seq=translation; coord=4:241233055..241239106:-1; parent_transcript=GRMZM2G134889_T01; parent_gene=GRMZM2G134889	GRMZM2G134889_P01	TRUE	TRUE	lFHIDAAAk	91%	n+304 (+304), K+304 (+304)	25.10	25.00
3952	seq=translation; coord=4:241233055..241239106:-1; parent_transcript=GRMZM2G134889_T01; parent_gene=GRMZM2G134889	GRMZM2G134889_P01	TRUE	TRUE	lLFVFVER	95%	n+304 (+304)	32.07	26.60
3953	seq=translation; coord=4:241233055..241239106:-1; parent_transcript=GRMZM2G134889_T01; parent_gene=GRMZM2G134889	GRMZM2G134889_P01	TRUE	TRUE	yDVQGFPTILFFIDGVPR	95%	n+304 (+304)	35.79	25.13
3954	seq=translation; coord=4:63419630..63429299:-1; parent_transcript=GRMZM2G075775_T01; parent_gene=GRMZM2G075775	GRMZM2G075775_P01	TRUE	TRUE	aSSVSDNQGLGcIALQYIk	95%	n+304 (+304), Carbamidomethyl (+57), K+304 (+304)	83.94	25.37
3955	seq=translation; coord=4:63419630..63429299:-1; parent_transcript=GRMZM2G075775_T01; parent_gene=GRMZM2G075775	GRMZM2G075775_P01	TRUE	TRUE	lVAcQSQYLAPAEAFGVLLVNDAk	95%	n+304 (+304), Carbamidomethyl (+57), K+304 (+304)	51.38	25.00
3956	seq=translation; coord=1:193791442..193794419:-1; parent_transcript=GRMZM2G148744_T01; parent_gene=GRMZM2G148744	GRMZM2G148744_P01,GRMZM2G148744_P02, GRMZM2G702426_P01	TRUE	TRUE	gLDVDNLYVSHIQVNQAQk	95%	n+304 (+304), K+304 (+304)	56.34	25.00
3957	seq=translation; coord=1:193791442..193794419:-1; parent_transcript=GRMZM2G148744_T01; parent_gene=GRMZM2G148744	GRMZM2G148744_P01,GRMZM2G148744_P02, GRMZM2G702426_P01	TRUE	TRUE	yLEDVIAHk	95%	n+304 (+304), K+304 (+304)	34.32	25.98
3958	seq=translation; coord=7:21276565..21279263:-1; parent_transcript=GRMZM2G063340_T01; parent_gene=GRMZM2G063340	GRMZM2G063340_P01,GRMZM2G063340_P03, GRMZM2G063340_P04,GRMZM2G139900_P01, GRMZM2G139900_P02	TRUE	TRUE	aLcAEHNVHLVTVPAAk	95%	n+304 (+304), Carbamidomethyl (+57), K+304 (+304)	30.59	25.00
3959	seq=translation; coord=7:21276565..21279263:-1; parent_transcript=GRMZM2G063340_T01; parent_gene=GRMZM2G063340	GRMZM2G063340_P01,GRMZM2G063340_P03, GRMZM2G063340_P04,GRMZM2G139900_P01, GRMZM2G139900_P02	TRUE	TRUE	dYGEESEGLNIVQEYVk	95%	n+304 (+304), K+304 (+304)	30.55	25.00
3960	seq=translation; coord=7:21276565..21279263:-1; parent_transcript=GRMZM2G063340_T01; parent_gene=GRMZM2G063340	GRMZM2G063340_P01,GRMZM2G063340_P03, GRMZM2G063340_P04,GRMZM2G139900_P01, GRMZM2G139900_P02	TRUE	TRUE	hAAQLcVLAEDcDQPDYVk	94%	n+304 (+304), Carbamidomethyl (+57), Carbamidomethyl (+57), K+304 (+304)	25.47	25.00
3961	seq=translation; coord=7:21276565..21279263:-1; parent_transcript=GRMZM2G063340_T01; parent_gene=GRMZM2G063340	GRMZM2G063340_P01,GRMZM2G063340_P03, GRMZM2G063340_P04,GRMZM2G139900_P01, GRMZM2G139900_P02	TRUE	TRUE	tLGEWAGLck	95%	n+304 (+304), Carbamidomethyl (+57), K+304 (+304)	47.16	26.22

3962	seq=translation; coord=7:21276565..21279263:-1; parent_transcript=GRMZM2G063340_T01; parent_gene=GRMZM2G063340	GRMZM2G063340_P01,GRMZM2G063340_P03, GRMZM2G063340_P04,GRMZM2G139900_P01, GRMZM2G139900_P02	TRUE	TRUE	vVGcScVVVk	95%	n+304 (+304), Carbamidomethyl (+57), Carbamidomethyl (+57), K+304 (+304)	52.10	26.51
3963	seq=translation; coord=3:33565292..33570884:-1; parent_transcript=GRMZM2G328893_T01; parent_gene=GRMZM2G328893	GRMZM2G328893_P01	TRUE	TRUE	aFGAELVLTDAAk	95%	n+304 (+304), K+304 (+304)	39.45	25.00
3964	seq=translation; coord=3:33565292..33570884:-1; parent_transcript=GRMZM2G328893_T01; parent_gene=GRMZM2G328893	GRMZM2G328893_P01	TRUE	TRUE	iQGIGAGFVPR	95%	n+304 (+304)	46.19	25.82
3965	seq=translation; coord=3:33565292..33570884:-1; parent_transcript=GRMZM2G328893_T01; parent_gene=GRMZM2G328893	GRMZM2G328893_P01	TRUE	TRUE	lIVVVFPSFGER	95%	n+304 (+304)	45.03	25.83
3966	seq=translation; coord=3:33565292..33570884:-1; parent_transcript=GRMZM2G328893_T01; parent_gene=GRMZM2G328893	GRMZM2G328893_P01	TRUE	TRUE	vHYETTGPEIWEDSk	95%	n+304 (+304), K+304 (+304)	37.50	25.00
3967	seq=translation; coord=9:92916278..92921897:-1; parent_transcript=GRMZM2G326472_T01; parent_gene=GRMZM2G326472	GRMZM2G326472_P01,GRMZM2G326472_P02, GRMZM2G326472_P03	TRUE	TRUE	gVSLPDLLGR	92%	n+304 (+304)	29.91	26.13
3968	seq=translation; coord=9:92916278..92921897:-1; parent_transcript=GRMZM2G326472_T01; parent_gene=GRMZM2G326472	GRMZM2G326472_P01,GRMZM2G326472_P02, GRMZM2G326472_P03	TRUE	TRUE	lADAYGGATGPHFAVk	95%	n+304 (+304), K+304 (+304)	39.12	26.32
3969	seq=translation; coord=9:92916278..92921897:-1; parent_transcript=GRMZM2G326472_T01; parent_gene=GRMZM2G326472	GRMZM2G326472_P01,GRMZM2G326472_P02, GRMZM2G326472_P03	TRUE	TRUE	vFIFPILDAAGGSGAAGGEDLETGNFDSGLR	95%	n+304 (+304)	31.17	25.00
3970	seq=translation; coord=5:146886556..146888542:-1; parent_transcript=GRMZM2G014444_T01; parent_gene=GRMZM2G014444	GRMZM2G014444_P01,GRMZM2G014444_P02, GRMZM2G014444_P04,GRMZM2G018770_P01, GRMZM2G018770_P02	TRUE	TRUE	aMQLLESGLk	95%	n+304 (+304), K+304 (+304)	57.37	25.20
3971	seq=translation; coord=5:146886556..146888542:-1; parent_transcript=GRMZM2G014444_T01; parent_gene=GRMZM2G014444	GRMZM2G014444_P01,GRMZM2G014444_P02, GRMZM2G014444_P04,GRMZM2G018770_P01, GRMZM2G018770_P02	TRUE	TRUE	lVLNISVGESGDR	95%	n+304 (+304)	43.36	26.36
3972	seq=translation; coord=5:146886556..146888542:-1; parent_transcript=GRMZM2G014444_T01; parent_gene=GRMZM2G014444	GRMZM2G014444_P01,GRMZM2G014444_P02, GRMZM2G014444_P04,GRMZM2G018770_P01, GRMZM2G018770_P02	TRUE	TRUE	vLEQLSGQTPVFSk	95%	n+304 (+304), K+304 (+304)	45.56	25.07
3973	seq=translation; coord=1:35919768..35937067:-1; parent_transcript=GRMZM2G174589_T01; parent_gene=GRMZM2G174589	GRMZM2G174589_P01	TRUE	TRUE	dFPLGDLPMLEVLR	95%	n+304 (+304)	37.34	26.42
3974	seq=translation; coord=1:35919768..35937067:-1; parent_transcript=GRMZM2G174589_T01; parent_gene=GRMZM2G174589	GRMZM2G174589_P01	TRUE	TRUE	dVQSPEDIVAAER	95%	n+304 (+304)	43.17	25.00
3975	seq=translation; coord=1:35919768..35937067:-1; parent_transcript=GRMZM2G174589_T01; parent_gene=GRMZM2G174589	GRMZM2G174589_P01	TRUE	TRUE	lVFPGVGAFGSAMDVLNR	95%	n+304 (+304)	56.10	25.77
3976	seq=translation; coord=4:183698889..183701902:1; parent_transcript=GRMZM2G088847_T01; parent_gene=GRMZM2G088847	GRMZM2G088847_P01,GRMZM2G088847_P02, GRMZM2G088847_P03,GRMZM2G088847_P04, GRMZM2G088847_P05,GRMZM2G088847_P07	TRUE	TRUE	lSAGIASILESk	95%	n+304 (+304), K+304 (+304)	41.66	25.00
3977	seq=translation; coord=4:183698889..183701902:1; parent_transcript=GRMZM2G088847_T01; parent_gene=GRMZM2G088847	GRMZM2G088847_P01,GRMZM2G088847_P02, GRMZM2G088847_P03,GRMZM2G088847_P04, GRMZM2G088847_P05,GRMZM2G088847_P07	TRUE	TRUE	sVANIIGkPEAYVMVVLk	95%	n+304 (+304), K+304 (+304), K+304 (+304)	27.13	25.00
3978	seq=translation; coord=5:191366452..191369776:1; parent_transcript=GRMZM5G840002_T01; parent_gene=GRMZM5G840002	GRMZM5G840002_P01,GRMZM5G840002_P02, GRMZM5G840002_P03	TRUE	TRUE	lkPIGDAGPVIEAGGIFAYAR	95%	n+304 (+304), K+304 (+304)	52.61	25.00

3979	seq=translation; coord=5:191366452..191369776:1; parent_transcript=GRMZM5G840002_T01; parent_gene=GRMZM5G840002	GRMZM5G840002_P01,GRMZM5G840002_P02, GRMZM5G840002_P03	TRUE	TRUE	nSVATGEVYPLELTDVGAWk	95%	n+304 (+304), K+304 (+304)	39.56	25.39
3980	seq=translation; coord=5:191366452..191369776:1; parent_transcript=GRMZM5G840002_T01; parent_gene=GRMZM5G840002	GRMZM5G840002_P01,GRMZM5G840002_P02, GRMZM5G840002_P03	TRUE	TRUE	yAIIVGGANFGcGSSR	95%	n+304 (+304), Carbamidomethyl (+57)	47.76	25.00
3981	seq=translation; coord=3:213298401..213303662:1; parent_transcript=GRMZM2G033515_T01; parent_gene=GRMZM2G033515	GRMZM2G033515_P01	TRUE	TRUE	lIFPFLDLDIk	95%	n+304 (+304), K+304 (+304)	34.91	25.00
3982	seq=translation; coord=3:158348059..158355046:1; parent_transcript=GRMZM2G139617_T01; parent_gene=GRMZM2G139617	GRMZM2G139617_P01	TRUE	TRUE	eVDFNTATLADILR	95%	n+304 (+304)	61.70	25.00
3983	seq=translation; coord=3:158348059..158355046:1; parent_transcript=GRMZM2G139617_T01; parent_gene=GRMZM2G139617	GRMZM2G139617_P01	TRUE	TRUE	lLEFLESPcVTR	95%	n+304 (+304), Carbamidomethyl (+57)	49.53	25.00
3984	seq=translation; coord=1:268251834..268259468:1; parent_transcript=GRMZM5G881950_T01; parent_gene=GRMZM5G881950	GRMZM5G881950_P01,GRMZM5G881950_P02	TRUE	TRUE	eQVAALVSAQVQQMLAAyAANR	95%	n+304 (+304), iTRAQ8plex (+304)	41.98	25.34
3985	seq=translation; coord=1:268251834..268259468:1; parent_transcript=GRMZM5G881950_T01; parent_gene=GRMZM5G881950	GRMZM5G881950_P01,GRMZM5G881950_P02	TRUE	TRUE	lLcESAVLLDAAAAQLWGk	95%	n+304 (+304), Carbamidomethyl (+57), K+304 (+304)	40.55	25.00
3986	seq=translation; coord=1:268251834..268259468:1; parent_transcript=GRMZM5G881950_T01; parent_gene=GRMZM5G881950	GRMZM5G881950_P01,GRMZM5G881950_P02	TRUE	TRUE	lLDSIVTLLSR	90%	n+304 (+304)	26.39	25.00
3987	seq=translation; coord=1:268251834..268259468:1; parent_transcript=GRMZM5G881950_T01; parent_gene=GRMZM5G881950	GRMZM5G881950_P01,GRMZM5G881950_P02	TRUE	TRUE	lRDEDEELFEcNWVEYVR	95%	n+304 (+304), Carbamidomethyl (+57)	66.32	25.00
3988	seq=translation; coord=1:268251834..268259468:1; parent_transcript=GRMZM5G881950_T01; parent_gene=GRMZM5G881950	GRMZM5G881950_P01,GRMZM5G881950_P02	TRUE	TRUE	nPDFNHYLFEALAAVIGR	95%	n+304 (+304)	37.64	25.68
3989	seq=translation; coord=1:268251834..268259468:1; parent_transcript=GRMZM5G881950_T01; parent_gene=GRMZM5G881950	GRMZM5G881950_P01,GRMZM5G881950_P02	TRUE	TRUE	tGEQDPALLPAFEASLFPVLQR	95%	n+304 (+304)	31.69	25.53
3990	seq=translation; coord=1:268251834..268259468:1; parent_transcript=GRMZM5G881950_T01; parent_gene=GRMZM5G881950	GRMZM5G881950_P01,GRMZM5G881950_P02	TRUE	TRUE	vLGIANIAGQIVHEITAR	95%	n+304 (+304)	75.73	25.00
3991	seq=translation; coord=2:2487249..2492988:-1; parent_transcript=GRMZM2G104430_T01; parent_gene=GRMZM2G104430	GRMZM2G104430_P01,GRMZM2G104430_P02	TRUE	TRUE	fAPPPAAALGDAMLHGFGVGEDDDVR	95%	n+304 (+304)	83.84	25.00
3992	seq=translation; coord=2:2487249..2492988:-1; parent_transcript=GRMZM2G104430_T01; parent_gene=GRMZM2G104430	GRMZM2G104430_P01,GRMZM2G104430_P02	TRUE	TRUE	iAVEDEDEELAR	95%	n+304 (+304)	38.50	25.00
3993	seq=translation; coord=2:2487249..2492988:-1; parent_transcript=GRMZM2G104430_T01; parent_gene=GRMZM2G104430	GRMZM2G104430_P01,GRMZM2G104430_P02	TRUE	TRUE	lLEDLLPYLDk	95%	n+304 (+304), K+304 (+304)	38.78	25.00
3994	seq=translation; coord=4:110069837..110073965:1; parent_transcript=GRMZM2G139680_T01; parent_gene=GRMZM2G139680	GRMZM2G139680_P01	TRUE	TRUE	aFGVLIPDQGIALR	94%	n+304 (+304)	29.55	25.04
3995	seq=translation; coord=4:110069837..110073965:1; parent_transcript=GRMZM2G139680_T01; parent_gene=GRMZM2G139680	GRMZM2G139680_P01	TRUE	TRUE	aPDFEAEAVFDQEFINVk	95%	n+304 (+304), K+304 (+304)	44.65	25.00
3996	seq=translation; coord=4:110069837..110073965:1; parent_transcript=GRMZM2G139680_T01; parent_gene=GRMZM2G139680	GRMZM2G139680_P01	TRUE	TRUE	aSVVDDLPLVGNk	95%	n+304 (+304), K+304 (+304)	35.64	25.90

3997	seq=translation; coord=7:13844415..13850805:1; parent_transcript=GRMZM2G001898_T01; parent_gene=GRMZM2G001898	GRMZM2G001898_P01	TRUE	TRUE	aDEHVDVTNPATQEVVSR	95%	n+304 (+304)	61.94	25.00
3998	seq=translation; coord=7:13844415..13850805:1; parent_transcript=GRMZM2G001898_T01; parent_gene=GRMZM2G001898	GRMZM2G001898_P01	TRUE	TRUE	dAWGDVFR	95%	n+304 (+304)	36.02	25.00
3999	seq=translation; coord=7:13844415..13850805:1; parent_transcript=GRMZM2G001898_T01; parent_gene=GRMZM2G001898	GRMZM2G001898_P01	TRUE	TRUE	dPGAAMMLAELAMEAGLPk	95%	n+304 (+304), K+304 (+304)	50.23	25.00
4000	seq=translation; coord=7:13844415..13850805:1; parent_transcript=GRMZM2G001898_T01; parent_gene=GRMZM2G001898	GRMZM2G001898_P01	TRUE	TRUE	iPLTTADEFk	95%	n+304 (+304), K+304 (+304)	39.51	26.64
4001	seq=translation; coord=7:13844415..13850805:1; parent_transcript=GRMZM2G001898_T01; parent_gene=GRMZM2G001898	GRMZM2G001898_P01	TRUE	TRUE	lLIGGEFVESR	91%	n+304 (+304)	28.24	25.00
4002	seq=translation; coord=7:13844415..13850805:1; parent_transcript=GRMZM2G001898_T01; parent_gene=GRMZM2G001898	GRMZM2G001898_P01	TRUE	TRUE	nHAIILPDADRDATLNALIAAGFGAAGQR	95%	n+304 (+304)	37.96	25.19
4003	seq=translation; coord=3:218835389..218837093:-1; parent_transcript=GRMZM2G074097_T01; parent_gene=GRMZM2G074097	GRMZM2G074097_P01	TRUE	TRUE	aLDMNAAEDEIVR	95%	n+304 (+304)	66.24	25.00
4004	seq=translation; coord=3:218835389..218837093:-1; parent_transcript=GRMZM2G074097_T01; parent_gene=GRMZM2G074097	GRMZM2G074097_P01	TRUE	TRUE	eVVPGMIVTGMEVAEIDGAPR	95%	n+304 (+304)	81.00	25.00
4005	seq=translation; coord=1:7566145..7569296:1; parent_transcript=GRMZM2G410991_T01; parent_gene=GRMZM2G410991	GRMZM2G410991_P01	TRUE	TRUE	gDDPPGIAAR	89%	n+304 (+304)	26.80	25.00
4006	seq=translation; coord=1:7566145..7569296:1; parent_transcript=GRMZM2G410991_T01; parent_gene=GRMZM2G410991	GRMZM2G410991_P01	TRUE	TRUE	iPLPcTcDQVDGADVMHFAYSVAk	92%	n+304 (+304), Carbamidomethyl (+57), Carbamidomethyl (+57), K+304 (+304)	26.51	25.00
4007	seq=translation; coord=1:7566145..7569296:1; parent_transcript=GRMZM2G410991_T01; parent_gene=GRMZM2G410991	GRMZM2G410991_P01	TRUE	TRUE	tSLQQGQILDVPLPVck	95%	n+304 (+304), Carbamidomethyl (+57), K+304 (+304)	50.07	25.47
4008	seq=translation; coord=1:43678296..43681497:1; parent_transcript=GRMZM2G137839_T01; parent_gene=GRMZM2G137839	GRMZM2G137839_P01	TRUE	TRUE	nYPTVSAEYSEAVDk	95%	n+304 (+304), K+304 (+304)	40.12	25.00
4009	seq=translation; coord=1:8972308..8976833:-1; parent_transcript=GRMZM2G046932_T01; parent_gene=GRMZM2G046932	GRMZM2G046932_P01	TRUE	TRUE	eALNEVVAAFVGk	95%	n+304 (+304), K+304 (+304)	33.56	25.97
4010	seq=translation; coord=1:8972308..8976833:-1; parent_transcript=GRMZM2G046932_T01; parent_gene=GRMZM2G046932	GRMZM2G046932_P01	TRUE	TRUE	lMEMAESLIk	95%	n+304 (+304), K+304 (+304)	39.30	25.51
4011	seq=translation; coord=1:8972308..8976833:-1; parent_transcript=GRMZM2G046932_T01; parent_gene=GRMZM2G046932	GRMZM2G046932_P01	TRUE	TRUE	lNMVYTLLSk	95%	n+304 (+304), K+304 (+304)	30.77	25.00
4012	seq=translation; coord=1:8972308..8976833:-1; parent_transcript=GRMZM2G046932_T01; parent_gene=GRMZM2G046932	GRMZM2G046932_P01	TRUE	TRUE	vDALIQFILQQGASk	91%	n+304 (+304), K+304 (+304)	26.30	25.00
4013	seq=translation; coord=1:8972308..8976833:-1; parent_transcript=GRMZM2G046932_T01; parent_gene=GRMZM2G046932	GRMZM2G046932_P01	TRUE	TRUE	vYPTYDFAcPFVDALEGVTHALR	95%	n+304 (+304), Carbamidomethyl (+57)	42.07	25.00

4014	seq=translation; coord=4:237041435..237046635:-1; parent_transcript=GRMZM2G009871_T01; parent_gene=GRMZM2G009871	GRMZM2G009871_P01,GRMZM2G009871_P02, GRMZM2G009871_P03,GRMZM2G009871_P04, GRMZM2G009871_P05	TRUE	TRUE	eLAVSIEGk	95%	n+304 (+304), K+304 (+304)	32.72	26.28
4015	seq=translation; coord=4:237041435..237046635:-1; parent_transcript=GRMZM2G009871_T01; parent_gene=GRMZM2G009871	GRMZM2G009871_P01,GRMZM2G009871_P02, GRMZM2G009871_P03,GRMZM2G009871_P04, GRMZM2G009871_P05	TRUE	TRUE	gGVTISNDGATIMR	94%	n+304 (+304)	30.63	25.08
4016	seq=translation; coord=4:237041435..237046635:-1; parent_transcript=GRMZM2G009871_T01; parent_gene=GRMZM2G009871	GRMZM2G009871_P01,GRMZM2G009871_P02, GRMZM2G009871_P03,GRMZM2G009871_P04, GRMZM2G009871_P05	TRUE	TRUE	iLVDIAk	95%	n+304 (+304), K+304 (+304)	29.84	25.00
4017	seq=translation; coord=4:237041435..237046635:-1; parent_transcript=GRMZM2G009871_T01; parent_gene=GRMZM2G009871	GRMZM2G009871_P01,GRMZM2G009871_P02, GRMZM2G009871_P03,GRMZM2G009871_P04, GRMZM2G009871_P05	TRUE	TRUE	lAIGDLGTQYFADR	95%	n+304 (+304)	34.21	25.00
4018	seq=translation; coord=4:237041435..237046635:-1; parent_transcript=GRMZM2G009871_T01; parent_gene=GRMZM2G009871	GRMZM2G009871_P01,GRMZM2G009871_P02, GRMZM2G009871_P03,GRMZM2G009871_P04, GRMZM2G009871_P05	TRUE	TRUE	lLDIVHPAAk	95%	n+304 (+304), K+304 (+304)	31.47	25.00
4019	seq=translation; coord=4:237041435..237046635:-1; parent_transcript=GRMZM2G009871_T01; parent_gene=GRMZM2G009871	GRMZM2G009871_P01,GRMZM2G009871_P02, GRMZM2G009871_P03,GRMZM2G009871_P04, GRMZM2G009871_P05	TRUE	TRUE	nSTVVPGGGAIDMEISk	95%	n+304 (+304), K+304 (+304)	37.26	25.47
4020	seq=translation; coord=4:237041435..237046635:-1; parent_transcript=GRMZM2G009871_T01; parent_gene=GRMZM2G009871	GRMZM2G009871_P01,GRMZM2G009871_P02, GRMZM2G009871_P03,GRMZM2G009871_P04, GRMZM2G009871_P05	TRUE	TRUE	vTEEDLQR	95%	n+304 (+304)	33.90	25.00
4021	seq=translation; coord=9:22779373..22783918:1; parent_transcript=GRMZM2G033208_T01; parent_gene=GRMZM2G033208	GRMZM2G033208_P01,GRMZM2G033208_P02, GRMZM2G033208_P03	TRUE	TRUE	eSVLPAAVTAR	94%	n+304 (+304)	32.69	26.50
4022	seq=translation; coord=9:22779373..22783918:1; parent_transcript=GRMZM2G033208_T01; parent_gene=GRMZM2G033208	GRMZM2G033208_P01,GRMZM2G033208_P02, GRMZM2G033208_P03	TRUE	TRUE	fEALGWHTIWVk	95%	n+304 (+304), K+304 (+304)	30.95	25.49
4023	seq=translation; coord=9:22779373..22783918:1; parent_transcript=GRMZM2G033208_T01; parent_gene=GRMZM2G033208	GRMZM2G033208_P01,GRMZM2G033208_P02, GRMZM2G033208_P03	TRUE	TRUE	qNLGWPYDTFFVPEDVk	95%	n+304 (+304), K+304 (+304)	36.87	25.00
4024	seq=translation; coord=9:22779373..22783918:1; parent_transcript=GRMZM2G033208_T01; parent_gene=GRMZM2G033208	GRMZM2G033208_P01,GRMZM2G033208_P02, GRMZM2G033208_P03	TRUE	TRUE	sIITGELPTGWVDALPk	95%	n+304 (+304), K+304 (+304)	59.33	25.00
4025	seq=translation; coord=9:22779373..22783918:1; parent_transcript=GRMZM2G033208_T01; parent_gene=GRMZM2G033208	GRMZM2G033208_P01,GRMZM2G033208_P02, GRMZM2G033208_P03	TRUE	TRUE	yTPESPGDATR	95%	n+304 (+304)	39.59	25.00
4026	seq=translation; coord=9:138752306..138754760:1; parent_transcript=GRMZM2G162486_T01; parent_gene=GRMZM2G162486	GRMZM2G162486_P01	TRUE	TRUE	eVLPSPLTSASEPPPLFDGTTR	95%	n+304 (+304)	42.68	26.07
4027	seq=translation; coord=9:138752306..138754760:1; parent_transcript=GRMZM2G162486_T01; parent_gene=GRMZM2G162486	GRMZM2G162486_P01	TRUE	TRUE	fQILYSNIk	95%	n+304 (+304), K+304 (+304)	35.58	25.00
4028	seq=translation; coord=9:138752306..138754760:1; parent_transcript=GRMZM2G162486_T01; parent_gene=GRMZM2G162486	GRMZM2G162486_P01	TRUE	TRUE	gESLDLVk	94%	n+304 (+304), K+304 (+304)	30.44	26.77
4029	seq=translation; coord=9:138752306..138754760:1; parent_transcript=GRMZM2G162486_T01; parent_gene=GRMZM2G162486	GRMZM2G162486_P01	TRUE	TRUE	iDAYTQTk	95%	n+304 (+304), K+304 (+304)	36.06	26.07
4030	seq=translation; coord=9:138752306..138754760:1; parent_transcript=GRMZM2G162486_T01; parent_gene=GRMZM2G162486	GRMZM2G162486_P01	TRUE	TRUE	iVAIDLADRPAWYk	95%	n+304 (+304), K+304 (+304)	27.50	25.60
4031	seq=translation; coord=9:138752306..138754760:1; parent_transcript=GRMZM2G162486_T01; parent_gene=GRMZM2G162486	GRMZM2G162486_P01	TRUE	TRUE	lDPQFLLEQTk	95%	n+304 (+304), K+304 (+304)	36.60	25.05

4032	seq=translation; coord=3:230900751..230906058:-1; parent_transcript=GRMZM2G152688_T01; parent_gene=GRMZM2G152688	GRMZM2G152688_P01,GRMZM2G152688_P02, GRMZM2G152688_P03	TRUE	TRUE	eLSAEAASLVEELNR	95%	n+304 (+304)	42.84	25.77
4033	seq=translation; coord=3:230900751..230906058:-1; parent_transcript=GRMZM2G152688_T01; parent_gene=GRMZM2G152688	GRMZM2G152688_P01,GRMZM2G152688_P02, GRMZM2G152688_P03	TRUE	TRUE	gELANFEVRPR	91%	n+304 (+304)	26.90	25.81
4034	seq=translation; coord=3:230900751..230906058:-1; parent_transcript=GRMZM2G152688_T01; parent_gene=GRMZM2G152688	GRMZM2G152688_P01,GRMZM2G152688_P02, GRMZM2G152688_P03	TRUE	TRUE	gNLELLEQR	95%	n+304 (+304)	35.15	26.30
4035	seq=translation; coord=3:230900751..230906058:-1; parent_transcript=GRMZM2G152688_T01; parent_gene=GRMZM2G152688	GRMZM2G152688_P01,GRMZM2G152688_P02, GRMZM2G152688_P03	TRUE	TRUE	gYPDTHLLEEPR	91%	n+304 (+304)	25.82	25.00
4036	seq=translation; coord=3:230900751..230906058:-1; parent_transcript=GRMZM2G152688_T01; parent_gene=GRMZM2G152688	GRMZM2G152688_P01,GRMZM2G152688_P02, GRMZM2G152688_P03	TRUE	TRUE	nMLSMAQEIEQMR	95%	n+304 (+304)	61.85	25.00
4037	seq=translation; coord=1:214719298..214723588:1; parent_transcript=GRMZM2G058702_T01; parent_gene=GRMZM2G058702	GRMZM2G058702_P01,GRMZM2G117786_P01	TRUE	TRUE	iVSWSAGEGDR	95%	n+304 (+304)	33.50	25.00
4038	seq=translation; coord=1:214719298..214723588:1; parent_transcript=GRMZM2G058702_T01; parent_gene=GRMZM2G058702	GRMZM2G058702_P01,GRMZM2G117786_P01	TRUE	TRUE	iVYGADLAAFLQTFAk	95%	n+304 (+304), K+304 (+304)	48.60	25.00
4039	seq=translation; coord=1:214719298..214723588:1; parent_transcript=GRMZM2G058702_T01; parent_gene=GRMZM2G058702	GRMZM2G058702_P01,GRMZM2G117786_P01	TRUE	TRUE	lDIYLLSQNWk	95%	n+304 (+304), K+304 (+304)	35.90	25.42
4040	seq=translation; coord=1:214719298..214723588:1; parent_transcript=GRMZM2G058702_T01; parent_gene=GRMZM2G058702	GRMZM2G058702_P01,GRMZM2G117786_P01	TRUE	TRUE	nMVESLAVPAFR	95%	n+304 (+304)	42.10	25.56
4041	seq=translation; coord=2:190424050..190431552:1; parent_transcript=GRMZM2G446050_T01; parent_gene=GRMZM2G446050	GRMZM2G446050_P01	TRUE	TRUE	dLAFADEANVAPLHLQR	95%	n+304 (+304)	83.77	25.00
4042	seq=translation; coord=2:190424050..190431552:1; parent_transcript=GRMZM2G446050_T01; parent_gene=GRMZM2G446050	GRMZM2G446050_P01	TRUE	TRUE	scSAAVESEVIR	95%	n+304 (+304), Carbamidomethyl (+57)	47.60	25.00
4043	seq=translation; coord=2:190424050..190431552:1; parent_transcript=GRMZM2G446050_T01; parent_gene=GRMZM2G446050	GRMZM2G446050_P01	TRUE	TRUE	tVDFIDEITAESNFk	95%	n+304 (+304), K+304 (+304)	33.12	25.28
4044	seq=translation; coord=9:150809669..150813518:1; parent_transcript=GRMZM2G169384_T01; parent_gene=GRMZM2G169384	GRMZM2G169384_P01	TRUE	TRUE	gGSPPGPPPPYR	95%	n+304 (+304)	33.09	25.00
4045	seq=translation; coord=9:150809669..150813518:1; parent_transcript=GRMZM2G169384_T01; parent_gene=GRMZM2G169384	GRMZM2G169384_P01	TRUE	TRUE	tLAQVVGSEEEAR	95%	n+304 (+304)	57.11	25.66
4046	seq=translation; coord=4:54122481..54127439:-1; parent_transcript=GRMZM2G070422_T01; parent_gene=GRMZM2G070422	GRMZM2G070422_P01,GRMZM2G070422_P02	TRUE	TRUE	aISEEcGPQGLPIDLR	95%	n+304 (+304), Carbamidomethyl (+57)	54.04	25.00
4047	seq=translation; coord=4:54122481..54127439:-1; parent_transcript=GRMZM2G070422_T01; parent_gene=GRMZM2G070422	GRMZM2G070422_P01,GRMZM2G070422_P02	TRUE	TRUE	eVVESVR	88%	n+304 (+304)	26.34	25.00
4048	seq=translation; coord=4:54122481..54127439:-1; parent_transcript=GRMZM2G070422_T01; parent_gene=GRMZM2G070422	GRMZM2G070422_P01,GRMZM2G070422_P02	TRUE	TRUE	mIEYSYVNAPLQk	95%	n+304 (+304), K+304 (+304)	31.72	25.15
4049	seq=translation; coord=4:54122481..54127439:-1; parent_transcript=GRMZM2G070422_T01; parent_gene=GRMZM2G070422	GRMZM2G070422_P01,GRMZM2G070422_P02	TRUE	TRUE	vNTISAGPLGSR	93%	n+304 (+304)	30.78	26.43

4050	seq=translation; coord=6:161725359..161731090:-1; parent_transcript=GRMZM2G074790_T01; parent_gene=GRMZM2G074790	GRMZM2G074790_P01,GRMZM2G074790_P02, GRMZM2G074790_P03	TRUE	TRUE	aAMLQGVNDLADAVk	95%	n+304 (+304), K+304 (+304)	62.58	25.65
4051	seq=translation; coord=6:161725359..161731090:-1; parent_transcript=GRMZM2G074790_T01; parent_gene=GRMZM2G074790	GRMZM2G074790_P01,GRMZM2G074790_P02, GRMZM2G074790_P03	TRUE	TRUE	aPLMTIAANAGIDGAIVIGk	95%	n+304 (+304), K+304 (+304)	33.39	25.00
4052	seq=translation; coord=6:161725359..161731090:-1; parent_transcript=GRMZM2G074790_T01; parent_gene=GRMZM2G074790	GRMZM2G074790_P01,GRMZM2G074790_P02, GRMZM2G074790_P03	TRUE	TRUE	eIGDLISk	95%	n+304 (+304), K+304 (+304)	35.72	26.70
4053	seq=translation; coord=6:161725359..161731090:-1; parent_transcript=GRMZM2G074790_T01; parent_gene=GRMZM2G074790	GRMZM2G074790_P01,GRMZM2G074790_P02, GRMZM2G074790_P03	TRUE	TRUE	eVSFGVGAR	89%	n+304 (+304)	26.14	25.00
4054	seq=translation; coord=6:161725359..161731090:-1; parent_transcript=GRMZM2G074790_T01; parent_gene=GRMZM2G074790	GRMZM2G074790_P01,GRMZM2G074790_P02, GRMZM2G074790_P03	TRUE	TRUE	hNLDDMAVMTGGEVISEER	95%	n+304 (+304)	42.87	25.00
4055	seq=translation; coord=6:161725359..161731090:-1; parent_transcript=GRMZM2G074790_T01; parent_gene=GRMZM2G074790	GRMZM2G074790_P01,GRMZM2G074790_P02, GRMZM2G074790_P03	TRUE	TRUE	kPLLIVAEDVEGDALSMLVLNk	95%	K+304 (+304), n+304 (+304), K+304 (+304)	30.32	25.00
4056	seq=translation; coord=6:161725359..161731090:-1; parent_transcript=GRMZM2G074790_T01; parent_gene=GRMZM2G074790	GRMZM2G074790_P01,GRMZM2G074790_P02, GRMZM2G074790_P03	TRUE	TRUE	lIEQEDLSLGYDAAk	95%	n+304 (+304), K+304 (+304)	31.23	26.30
4057	seq=translation; coord=6:161725359..161731090:-1; parent_transcript=GRMZM2G074790_T01; parent_gene=GRMZM2G074790	GRMZM2G074790_P01,GRMZM2G074790_P02, GRMZM2G074790_P03	TRUE	TRUE	tALQDAASVSLLMATTEAAVSELPATk	95%	n+304 (+304), K+304 (+304)	35.98	25.00
4058	seq=translation; coord=6:161725359..161731090:-1; parent_transcript=GRMZM2G074790_T01; parent_gene=GRMZM2G074790	GRMZM2G074790_P01,GRMZM2G074790_P02, GRMZM2G074790_P03	TRUE	TRUE	tLDNELEAVQGMk	95%	n+304 (+304), K+304 (+304)	29.25	26.26
4059	seq=translation; coord=6:113343612..113346307:1; parent_transcript=GRMZM2G168119_T01; parent_gene=GRMZM2G168119	GRMZM2G168119_P01	TRUE	TRUE	fVVFDLk	95%	n+304 (+304), K+304 (+304)	32.63	25.00
4060	seq=translation; coord=6:113343612..113346307:1; parent_transcript=GRMZM2G168119_T01; parent_gene=GRMZM2G168119	GRMZM2G168119_P01	TRUE	TRUE	qMGLPTSDEMQk	95%	n+304 (+304), K+304 (+304)	37.95	25.00
4061	seq=translation; coord=6:113343612..113346307:1; parent_transcript=GRMZM2G168119_T01; parent_gene=GRMZM2G168119	GRMZM2G168119_P01	TRUE	TRUE	vDDcFWSIEDGR	95%	n+304 (+304), Carbamidomethyl (+57)	49.53	25.00
4062	seq=translation; coord=6:113343612..113346307:1; parent_transcript=GRMZM2G168119_T01; parent_gene=GRMZM2G168119	GRMZM2G168119_P01	TRUE	TRUE	vTAMAAAVR	95%	n+304 (+304)	36.66	25.00
4063	seq=translation; coord=10:29873986..29885675:1; parent_transcript=GRMZM2G042089_T01; parent_gene=GRMZM2G042089	GRMZM2G042089_P01,GRMZM2G042089_P03, GRMZM2G143354_P01	TRUE	TRUE	iEGLLAAFPk	95%	n+304 (+304), K+304 (+304)	35.86	25.00
4064	seq=translation; coord=10:29873986..29885675:1; parent_transcript=GRMZM2G042089_T01; parent_gene=GRMZM2G042089	GRMZM2G042089_P01,GRMZM2G042089_P03, GRMZM2G143354_P01	TRUE	TRUE	nSVLEWSILLIDQSNR	94%	n+304 (+304)	30.30	26.30
4065	seq=translation; coord=10:29873986..29885675:1; parent_transcript=GRMZM2G042089_T01; parent_gene=GRMZM2G042089	GRMZM2G042089_P01,GRMZM2G042089_P03, GRMZM2G143354_P01	TRUE	TRUE	qSNILEDLDTLR	95%	n+304 (+304)	59.29	25.00
4066	seq=translation; coord=8:40257571..40264535:-1; parent_transcript=GRMZM2G132903_T01; parent_gene=GRMZM2G132903	GRMZM2G132903_P01	TRUE	TRUE	aMSLVTGVLDYER	95%	n+304 (+304)	78.78	25.00
4067	seq=translation; coord=8:40257571..40264535:-1; parent_transcript=GRMZM2G132903_T01; parent_gene=GRMZM2G132903	GRMZM2G132903_P01	TRUE	TRUE	dIVEMVFFPVINEAcR	95%	n+304 (+304), Carbamidomethyl (+57)	28.57	25.00

4068	seq=translation; coord=8:40257571..40264535:-1; parent_transcript=GRMZM2G132903_T01; parent_gene=GRMZM2G132903	GRMZM2G132903_P01	TRUE	TRUE	eATSFQELLFSDTck	95%	n+304 (+304), Carbamidomethyl (+57), K+304 (+304)	56.74	25.00
4069	seq=translation; coord=8:40257571..40264535:-1; parent_transcript=GRMZM2G132903_T01; parent_gene=GRMZM2G132903	GRMZM2G132903_P01	TRUE	TRUE	gIPLSAPTk	95%	n+304 (+304), K+304 (+304)	35.90	25.00
4070	seq=translation; coord=8:40257571..40264535:-1; parent_transcript=GRMZM2G132903_T01; parent_gene=GRMZM2G132903	GRMZM2G132903_P01	TRUE	TRUE	lSEkDIVEMVFFPVINEAcR	95%	n+304 (+304), K+304 (+304), Carbamidomethyl (+57)	30.23	25.72
4071	seq=translation; coord=8:40257571..40264535:-1; parent_transcript=GRMZM2G132903_T01; parent_gene=GRMZM2G132903	GRMZM2G132903_P01	TRUE	TRUE	sMAGVTPDPELMk	94%	n+304 (+304), K+304 (+304)	28.44	25.00
4072	seq=translation; coord=8:40257571..40264535:-1; parent_transcript=GRMZM2G132903_T01; parent_gene=GRMZM2G132903	GRMZM2G132903_P01	TRUE	TRUE	vAILGGGLMGSGIATAMVLSNYPVLLk	95%	n+304 (+304), K+304 (+304)	68.12	25.00
4073	seq=translation; coord=9:129405892..129410419:1; parent_transcript=GRMZM2G125148_T01; parent_gene=GRMZM2G125148	GRMZM2G125148_P01,GRMZM2G125148_P03, GRMZM2G125148_P04	TRUE	TRUE	eAPAGEGDGEk	95%	n+304 (+304), K+304 (+304)	56.77	25.37
4074	seq=translation; coord=9:129405892..129410419:1; parent_transcript=GRMZM2G125148_T01; parent_gene=GRMZM2G125148	GRMZM2G125148_P01,GRMZM2G125148_P03, GRMZM2G125148_P04	TRUE	TRUE	eVEEEPVVAAASEVk	95%	n+304 (+304), K+304 (+304)	29.05	25.68
4075	seq=translation; coord=7:13173972..13176516:-1; parent_transcript=GRMZM2G144653_T02; parent_gene=GRMZM2G144653	GRMZM2G144653_P02	TRUE	TRUE	aIAPLFVEHAk	95%	n+304 (+304), K+304 (+304)	56.87	25.00
4076	seq=translation; coord=7:13173972..13176516:-1; parent_transcript=GRMZM2G144653_T02; parent_gene=GRMZM2G144653	GRMZM2G144653_P02	TRUE	TRUE	eVTAAYEVEAMPTFHFVk	95%	n+304 (+304), K+304 (+304)	52.65	25.65
4077	seq=translation; coord=7:13173972..13176516:-1; parent_transcript=GRMZM2G144653_T02; parent_gene=GRMZM2G144653	GRMZM2G144653_P02	TRUE	TRUE	fTQVVFLk	94%	n+304 (+304), K+304 (+304)	27.71	25.00
4078	seq=translation; coord=1:154077677..154081800:1; parent_transcript=GRMZM2G056039_T01; parent_gene=GRMZM2G056039	GRMZM2G056039_P01	TRUE	TRUE	kIEDAIDAAISWLDANQLAEADEFDDk	95%	K+304 (+304), n+304 (+304), K+304 (+304)	31.73	25.00
4079	seq=translation; coord=1:154077677..154081800:1; parent_transcript=GRMZM2G056039_T01; parent_gene=GRMZM2G056039	GRMZM2G056039_P01	TRUE	TRUE	mYQGAGADMGGSGGMDEDAPAGSGGPGPk	95%	n+304 (+304), K+304 (+304)	104.53	25.00
4080	seq=translation; coord=4:9677072..9677727:-1; parent_transcript=GRMZM2G419675_T01; parent_gene=GRMZM2G419675	GRMZM2G419675_P01,GRMZM2G454556_P01	TRUE	TRUE	gYQMGHLDVDyQFVGc	95%	n+304 (+304), iTRAQ8plex (+304), Carbamidomethyl (+57)	32.65	25.00
4081	seq=translation; coord=4:9677072..9677727:-1; parent_transcript=GRMZM2G419675_T01; parent_gene=GRMZM2G419675	GRMZM2G419675_P01,GRMZM2G454556_P01	TRUE	TRUE	qQYGWTAFcGPSGPk	95%	n+304 (+304), Carbamidomethyl (+57), K+304 (+304)	33.22	25.00
4082	seq=translation; coord=4:9677072..9677727:-1; parent_transcript=GRMZM2G419675_T01; parent_gene=GRMZM2G419675	GRMZM2G419675_P01,GRMZM2G454556_P01	TRUE	TRUE	qQYGWTAFcGPSGPk	95%	Pyro-cmC (-17), n+304 (+304), Carbamidomethyl (+57), K+304 (+304)	36.39	25.00
4083	seq=translation; coord=4:9677072..9677727:-1; parent_transcript=GRMZM2G419675_T01; parent_gene=GRMZM2G419675	GRMZM2G419675_P01,GRMZM2G454556_P01	TRUE	TRUE	qQyGWTAFcGPSGPk	95%	Pyro-cmC (-17), n+304 (+304), iTRAQ8plex (+304), Carbamidomethyl (+57), K+304 (+304)	60.24	25.00
4084	seq=translation; coord=4:9677072..9677727:-1; parent_transcript=GRMZM2G419675_T01; parent_gene=GRMZM2G419675	GRMZM2G419675_P01,GRMZM2G454556_P01	TRUE	TRUE	vGAYcATWDAGkPPSWR	95%	n+304 (+304), Carbamidomethyl (+57), K+304 (+304)	37.43	25.00

4085	seq=translation; coord=1:25625764..25630712:-1; parent_transcript=GRMZM2G091563_T01; parent_gene=GRMZM2G091563	GRMZM2G091563_P01	TRUE	TRUE	aMEVLSDILQNSNLDQAR	95%	n+304 (+304)	30.33	25.00
4086	seq=translation; coord=5:23016144..23019861:1; parent_transcript=GRMZM2G030169_T01; parent_gene=GRMZM2G030169	GRMZM2G030169_P01,GRMZM2G030169_P02, GRMZM2G030169_P03,GRMZM2G082484_P01	TRUE	TRUE	lAYVALDYEQELETAk	95%	n+304 (+304), K+304 (+304)	42.74	25.88
4087	seq=translation; coord=8:8351675..8357336:1; parent_transcript=GRMZM2G132796_T01; parent_gene=GRMZM2G132796	GRMZM2G132796_P01	TRUE	TRUE	vAEPLLPDPLLPDPPSR	95%	n+304 (+304)	40.29	25.63
4088	seq=translation; coord=4:12082697..12084089:1; parent_transcript=GRMZM2G358153_T01; parent_gene=GRMZM2G358153	GRMZM2G358153_P01	TRUE	TRUE	lPGLFIWSADSSk	93%	n+304 (+304), K+304 (+304)	28.33	25.90
4089	seq=translation; coord=4:12082697..12084089:1; parent_transcript=GRMZM2G358153_T01; parent_gene=GRMZM2G358153	GRMZM2G358153_P01	TRUE	TRUE	tGNVTGLLSPDQGISGAk	95%	n+304 (+304), K+304 (+304)	42.94	25.75
4090	seq=translation; coord=4:12082697..12084089:1; parent_transcript=GRMZM2G358153_T01; parent_gene=GRMZM2G358153	GRMZM2G358153_P01	TRUE	TRUE	yETEAQQIIANH	95%	n+304 (+304)	67.12	25.00
4091	seq=translation; coord=4:12082697..12084089:1; parent_transcript=GRMZM2G358153_T01; parent_gene=GRMZM2G358153	GRMZM2G358153_P01	TRUE	TRUE	yGLDGVDVDyEHFADGADVNTFVEcIGR	95%	n+304 (+304), iTRAQ8plex (+304), Carbamidomethyl (+57)	39.50	25.00
4092	seq=translation; coord=3:128521980..128525993:1; parent_transcript=GRMZM2G034083_T01; parent_gene=GRMZM2G034083	GRMZM2G034083_P01	TRUE	TRUE	eEEVLQYAR	92%	n+304 (+304)	28.50	25.00
4093	seq=translation; coord=3:128521980..128525993:1; parent_transcript=GRMZM2G034083_T01; parent_gene=GRMZM2G034083	GRMZM2G034083_P01	TRUE	TRUE	sGLVALNLDLAQVR	95%	n+304 (+304)	53.24	25.12
4094	seq=translation; coord=3:128521980..128525993:1; parent_transcript=GRMZM2G034083_T01; parent_gene=GRMZM2G034083	GRMZM2G034083_P01	TRUE	TRUE	wGNIVFPLPFGR	95%	n+304 (+304)	32.57	25.12
4095	seq=translation; coord=1:298687651..298689984:1; parent_transcript=GRMZM5G821637_T05; parent_gene=GRMZM5G821637	GRMZM5G821637_P05	TRUE	TRUE	aGPSAPGGAPPNPFDFSTMMNLLNDPSIk	95%	n+304 (+304), K+304 (+304)	32.51	25.00
4096	seq=translation; coord=1:298687651..298689984:1; parent_transcript=GRMZM5G821637_T05; parent_gene=GRMZM5G821637	GRMZM5G821637_P05	TRUE	TRUE	dcVALLLESGAAVTLQNLDGk	95%	n+304 (+304), Carbamidomethyl (+57), K+304 (+304)	61.55	25.00
4097	seq=translation; coord=1:298687651..298689984:1; parent_transcript=GRMZM5G821637_T05; parent_gene=GRMZM5G821637	GRMZM5G821637_P05	TRUE	TRUE	dPAFTEMAEQLQk	95%	n+304 (+304), K+304 (+304)	53.25	25.00
4098	seq=translation; coord=1:298687651..298689984:1; parent_transcript=GRMZM5G821637_T05; parent_gene=GRMZM5G821637	GRMZM5G821637_P05	TRUE	TRUE	lGSALMQDPAMSAMLGGLTNPAHk	95%	n+304 (+304), K+304 (+304)	38.63	25.25
4099	seq=translation; coord=1:298687651..298689984:1; parent_transcript=GRMZM5G821637_T05; parent_gene=GRMZM5G821637	GRMZM5G821637_P05	TRUE	TRUE	lNNQDDVLk	95%	n+304 (+304), K+304 (+304)	46.97	25.42
4100	seq=translation; coord=1:298687651..298689984:1; parent_transcript=GRMZM5G821637_T05; parent_gene=GRMZM5G821637	GRMZM5G821637_P05	TRUE	TRUE	qQQQQLDPQk	92%	Pyro-cmC (-17), n+304 (+304), K+304 (+304)	28.44	25.00
4101	seq=translation; coord=1:298687651..298689984:1; parent_transcript=GRMZM5G821637_T05; parent_gene=GRMZM5G821637	GRMZM5G821637_P05	TRUE	TRUE	yVATMQQLMQNPQFVAMAER	95%	n+304 (+304)	49.97	25.00
4102	seq=translation; coord=1:298687651..298689984:1; parent_transcript=GRMZM5G821637_T05; parent_gene=GRMZM5G821637	GRMZM5G821637_P05	TRUE	TRUE	yWNDPEALQk	88%	n+304 (+304), K+304 (+304)	26.81	25.67

4103	seq=translation; coord=7:1266505..1269350:-1; parent_transcript=GRMZM2G420743_T01; parent_gene=GRMZM2G420743	GRMZM2G420743_P01	TRUE	TRUE	eLAVVGGTGGLR	95%	n+304 (+304)	33.01	25.83
4104	seq=translation; coord=7:1266505..1269350:-1; parent_transcript=GRMZM2G420743_T01; parent_gene=GRMZM2G420743	GRMZM2G420743_P01	TRUE	TRUE	gASGYVLWTTAk	95%	n+304 (+304), K+304 (+304)	29.95	26.05
4105	seq=translation; coord=7:1266505..1269350:-1; parent_transcript=GRMZM2G420743_T01; parent_gene=GRMZM2G420743	GRMZM2G420743_P01	TRUE	TRUE	tFGDTVAIDDLVTDGPGVGADSRPVGR	95%	n+304 (+304)	30.46	25.00
4106	seq=translation; coord=1:172122450..172128491:-1; parent_transcript=GRMZM2G029262_T01; parent_gene=GRMZM2G029262	GRMZM2G029262_P01,GRMZM2G029262_P03, GRMZM2G029262_P04	TRUE	TRUE	aFSAAVSALGIYNk	95%	n+304 (+304), K+304 (+304)	49.82	25.67
4107	seq=translation; coord=1:172122450..172128491:-1; parent_transcript=GRMZM2G029262_T01; parent_gene=GRMZM2G029262	GRMZM2G029262_P01,GRMZM2G029262_P03, GRMZM2G029262_P04	TRUE	TRUE	aSGYDVESSASSDAILk	95%	n+304 (+304), K+304 (+304)	29.75	25.55
4108	seq=translation; coord=1:172122450..172128491:-1; parent_transcript=GRMZM2G029262_T01; parent_gene=GRMZM2G029262	GRMZM2G029262_P01,GRMZM2G029262_P03, GRMZM2G029262_P04	TRUE	TRUE	eNINTQTHQLIDAR	95%	n+304 (+304)	54.74	25.00
4109	seq=translation; coord=1:172122450..172128491:-1; parent_transcript=GRMZM2G029262_T01; parent_gene=GRMZM2G029262	GRMZM2G029262_P01,GRMZM2G029262_P03, GRMZM2G029262_P04	TRUE	TRUE	lQPHLLWSLDQVk	95%	n+304 (+304), K+304 (+304)	31.50	25.00
4110	seq=translation; coord=1:172122450..172128491:-1; parent_transcript=GRMZM2G029262_T01; parent_gene=GRMZM2G029262	GRMZM2G029262_P01,GRMZM2G029262_P03, GRMZM2G029262_P04	TRUE	TRUE	nPLQEYQVAHIPGALFFDVDGISDR	95%	n+304 (+304)	50.49	25.00
4111	seq=translation; coord=1:172122450..172128491:-1; parent_transcript=GRMZM2G029262_T01; parent_gene=GRMZM2G029262	GRMZM2G029262_P01,GRMZM2G029262_P03, GRMZM2G029262_P04	TRUE	TRUE	vLDASWYMPAEQR	95%	n+304 (+304)	46.14	25.00
4112	seq=translation; coord=1:172122450..172128491:-1; parent_transcript=GRMZM2G029262_T01; parent_gene=GRMZM2G029262	GRMZM2G029262_P01,GRMZM2G029262_P03, GRMZM2G029262_P04	TRUE	TRUE	vWVLDGGLPQWR	95%	n+304 (+304)	34.12	26.04
4113	seq=translation; coord=1:167864280..167870604:1; parent_transcript=GRMZM2G130095_T01; parent_gene=GRMZM2G130095	GRMZM2G130095_P01,GRMZM2G130095_P03	TRUE	TRUE	aVELIASHLR	95%	n+304 (+304)	50.47	26.16
4114	seq=translation; coord=1:167864280..167870604:1; parent_transcript=GRMZM2G130095_T01; parent_gene=GRMZM2G130095	GRMZM2G130095_P01,GRMZM2G130095_P03	TRUE	TRUE	dEPDTELPPAVDGLLR	95%	n+304 (+304)	68.60	25.00
4115	seq=translation; coord=1:167864280..167870604:1; parent_transcript=GRMZM2G130095_T01; parent_gene=GRMZM2G130095	GRMZM2G130095_P01,GRMZM2G130095_P03	TRUE	TRUE	iTDGLETETDQPQR	91%	n+304 (+304)	28.10	25.00
4116	seq=translation; coord=1:167864280..167870604:1; parent_transcript=GRMZM2G130095_T01; parent_gene=GRMZM2G130095	GRMZM2G130095_P01,GRMZM2G130095_P03	TRUE	TRUE	lLVPASQAGSLIGk	95%	n+304 (+304), K+304 (+304)	52.58	25.00
4117	seq=translation; coord=1:167864280..167870604:1; parent_transcript=GRMZM2G130095_T01; parent_gene=GRMZM2G130095	GRMZM2G130095_P01,GRMZM2G130095_P03	TRUE	TRUE	sVLPLFETQIk	95%	n+304 (+304), K+304 (+304)	41.90	25.00
4118	seq=translation; coord=2:212180089..212183936:-1; parent_transcript=GRMZM2G365160_T01; parent_gene=GRMZM2G365160	GRMZM2G365160_P01,GRMZM2G365160_P02	TRUE	TRUE	gVANPLGIk	95%	n+304 (+304), K+304 (+304)	42.49	25.00
4119	seq=translation; coord=2:212180089..212183936:-1; parent_transcript=GRMZM2G365160_T01; parent_gene=GRMZM2G365160	GRMZM2G365160_P01,GRMZM2G365160_P02	TRUE	TRUE	tRPFDSILAEVR	95%	n+304 (+304)	42.86	25.17
4120	seq=translation; coord=2:212180089..212183936:-1; parent_transcript=GRMZM2G365160_T01; parent_gene=GRMZM2G365160	GRMZM2G365160_P01,GRMZM2G365160_P02	TRUE	TRUE	tVTFDDLSDR	95%	n+304 (+304)	31.75	25.00

4121	seq=translation; coord=2:212180089..212183936:-1; parent_transcript=GRMZM2G365160_T01; parent_gene=GRMZM2G365160	GRMZM2G365160_P01,GRMZM2G365160_P02	TRUE	TRUE	vTQWNLDFMDHNEQGDR	95%	n+304 (+304)	58.21	25.00
4122	seq=translation; coord=4:33112293..33117947:1; parent_transcript=GRMZM2G104907_T01; parent_gene=GRMZM2G104907	GRMZM2G104907_P01	TRUE	TRUE	fLVQSVVAPAGITVk	95%	n+304 (+304), K+304 (+304)	42.66	25.00
4123	seq=translation; coord=4:33112293..33117947:1; parent_transcript=GRMZM2G104907_T01; parent_gene=GRMZM2G104907	GRMZM2G104907_P01	TRUE	TRUE	sTcDVVVTMQAQR	95%	n+304 (+304), Carbamidomethyl (+57)	59.11	25.00
4124	seq=translation; coord=4:33112293..33117947:1; parent_transcript=GRMZM2G104907_T01; parent_gene=GRMZM2G104907	GRMZM2G104907_P01	TRUE	TRUE	vDPLELR	93%	n+304 (+304)	33.06	28.24
4125	seq=translation; coord=3:168523033..168526394:1; parent_transcript=GRMZM2G086845_T01; parent_gene=GRMZM2G086845	GRMZM2G086845_P01	TRUE	TRUE	gAIPSLPTEAEVLQLIEk	95%	n+304 (+304), K+304 (+304)	48.37	25.00
4126	seq=translation; coord=3:168523033..168526394:1; parent_transcript=GRMZM2G086845_T01; parent_gene=GRMZM2G086845	GRMZM2G086845_P01	TRUE	TRUE	lGDDEFGR	93%	n+304 (+304)	29.38	25.00
4127	seq=translation; coord=3:168523033..168526394:1; parent_transcript=GRMZM2G086845_T01; parent_gene=GRMZM2G086845	GRMZM2G086845_P01	TRUE	TRUE	lGGGAAFVGk	95%	n+304 (+304), K+304 (+304)	39.21	27.03
4128	seq=translation; coord=3:168523033..168526394:1; parent_transcript=GRMZM2G086845_T01; parent_gene=GRMZM2G086845	GRMZM2G086845_P01	TRUE	TRUE	tQILSIWDQADIVk	95%	n+304 (+304), K+304 (+304)	37.57	25.00
4129	seq=translation; coord=3:168523033..168526394:1; parent_transcript=GRMZM2G086845_T01; parent_gene=GRMZM2G086845	GRMZM2G086845_P01	TRUE	TRUE	vQQVDTTGAGDAFVGALLQR	95%	n+304 (+304)	37.08	25.48
4130	seq=translation; coord=4:63703567..63707582:1; parent_transcript=GRMZM2G024959_T01; parent_gene=GRMZM2G024959	GRMZM2G024959_P01,GRMZM2G102927_P01	TRUE	TRUE	fNLIADMMLDEAIk	95%	n+304 (+304), K+304 (+304)	45.70	25.62
4131	seq=translation; coord=4:63703567..63707582:1; parent_transcript=GRMZM2G024959_T01; parent_gene=GRMZM2G024959	GRMZM2G024959_P01,GRMZM2G102927_P01	TRUE	TRUE	nAITSQGPGTAVEFALALVEk	95%	n+304 (+304), K+304 (+304)	37.78	25.01
4132	seq=translation; coord=4:63703567..63707582:1; parent_transcript=GRMZM2G024959_T01; parent_gene=GRMZM2G024959	GRMZM2G024959_P01,GRMZM2G102927_P01	TRUE	TRUE	vLVPVAAGTEPIEAATTADVLNR	93%	n+304 (+304)	27.21	25.15
4133	seq=translation; coord=4:63703567..63707582:1; parent_transcript=GRMZM2G024959_T01; parent_gene=GRMZM2G024959	GRMZM2G024959_P01,GRMZM2G102927_P01	TRUE	TRUE	vVVDGNLITSR	92%	n+304 (+304)	29.22	25.68
4134	seq=translation; coord=4:165966482..165969746:-1; parent_transcript=GRMZM2G125268_T01; parent_gene=GRMZM2G125268	GRMZM2G125268_P01	TRUE	TRUE	dGVEQGPQIDGEQFNk	95%	n+304 (+304), K+304 (+304)	71.36	25.00
4135	seq=translation; coord=4:165966482..165969746:-1; parent_transcript=GRMZM2G125268_T01; parent_gene=GRMZM2G125268	GRMZM2G125268_P01	TRUE	TRUE	fADLIER	87%	n+304 (+304)	25.53	25.00
4136	seq=translation; coord=4:165966482..165969746:-1; parent_transcript=GRMZM2G125268_T01; parent_gene=GRMZM2G125268	GRMZM2G125268_P01	TRUE	TRUE	vAEGDSEDIDR	95%	n+304 (+304)	66.66	25.00
4137	seq=translation; coord=10:142067983..142070677:1; parent_transcript=GRMZM2G019325_T01; parent_gene=GRMZM2G019325	GRMZM2G019325_P01,GRMZM2G044800_P01	TRUE	TRUE	eAIEGTYIDk	95%	n+304 (+304), K+304 (+304)	59.37	26.19
4138	seq=translation; coord=10:142067983..142070677:1; parent_transcript=GRMZM2G019325_T01; parent_gene=GRMZM2G019325	GRMZM2G019325_P01,GRMZM2G044800_P01	TRUE	TRUE	sIGLGFk	87%	n+304 (+304), K+304 (+304)	25.65	25.00

4139	seq=translation; coord=10:142067983..142070677:1; parent_transcript=GRMZM2G019325_T01; parent_gene=GRMZM2G019325	GRMZM2G019325_P01,GRMZM2G044800_P01	TRUE	TRUE	vIPAGSAAAGk	95%	n+304 (+304), K+304 (+304)	51.42	25.00
4140	seq=translation; coord=2:196050685..196097541:1; parent_transcript=GRMZM2G058105_T01; parent_gene=GRMZM2G058105	GRMZM2G058105_P01	TRUE	TRUE	aLQDAEELEVAAR	95%	n+304 (+304)	49.16	25.34
4141	seq=translation; coord=2:196050685..196097541:1; parent_transcript=GRMZM2G058105_T01; parent_gene=GRMZM2G058105	GRMZM2G058105_P01	TRUE	TRUE	gLSADALASLPSVTYk	95%	n+304 (+304), K+304 (+304)	29.63	25.00
4142	seq=translation; coord=2:196050685..196097541:1; parent_transcript=GRMZM2G058105_T01; parent_gene=GRMZM2G058105	GRMZM2G058105_P01	TRUE	TRUE	lMALAGLSDWR	93%	n+304 (+304)	30.10	25.39
4143	seq=translation; coord=10:4243574..4244555:-1; parent_transcript=GRMZM5G898755_T01; parent_gene=GRMZM5G898755	GRMZM5G898755_P01,GRMZM5G898755_P02	TRUE	TRUE	cGVSVGVPISPTVDcTk	95%	Carbamidomethyl (+57), n+304 (+304), Carbamidomethyl (+57), K+304 (+304)	39.62	25.40
4144	seq=translation; coord=10:4243574..4244555:-1; parent_transcript=GRMZM5G898755_T01; parent_gene=GRMZM5G898755	GRMZM5G898755_P01,GRMZM5G898755_P02	TRUE	TRUE	lGGGVSMANAANIPSk	95%	n+304 (+304), K+304 (+304)	78.63	25.85
4145	seq=translation; coord=10:4243574..4244555:-1; parent_transcript=GRMZM5G898755_T01; parent_gene=GRMZM5G898755	GRMZM5G898755_P01,GRMZM5G898755_P02	TRUE	TRUE	qATcNcLk	95%	n+304 (+304), Carbamidomethyl (+57), Carbamidomethyl (+57), K+304 (+304)	31.69	25.00
4146	seq=translation; coord=10:76402669..76405224:1; parent_transcript=GRMZM2G079908_T01; parent_gene=GRMZM2G079908	GRMZM2G079908_P01,GRMZM2G079908_P02, GRMZM2G079908_P04	TRUE	TRUE	gLQEEFEEHAEk	95%	n+304 (+304), K+304 (+304)	71.70	25.00
4147	seq=translation; coord=10:76402669..76405224:1; parent_transcript=GRMZM2G079908_T01; parent_gene=GRMZM2G079908	GRMZM2G079908_P01,GRMZM2G079908_P02, GRMZM2G079908_P04	TRUE	TRUE	lILYGLYk	95%	n+304 (+304), K+304 (+304)	41.67	25.00
4148	seq=translation; coord=10:76402669..76405224:1; parent_transcript=GRMZM2G079908_T01; parent_gene=GRMZM2G079908	GRMZM2G079908_P01,GRMZM2G079908_P02, GRMZM2G079908_P04	TRUE	TRUE	qATVGDVNTDRPGIFYQk	90%	Pyro-cmC (-17), n+304 (+304), K+304 (+304)	25.68	25.00
4149	seq=translation; coord=10:14302293..14305526:1; parent_transcript=GRMZM2G106622_T01; parent_gene=GRMZM2G106622	GRMZM2G106622_P01	TRUE	TRUE	aAPSMSDAAMGk	95%	n+304 (+304), K+304 (+304)	54.80	25.00
4150	seq=translation; coord=10:14302293..14305526:1; parent_transcript=GRMZM2G106622_T01; parent_gene=GRMZM2G106622	GRMZM2G106622_P01	TRUE	TRUE	aLSEGGFEk	94%	n+304 (+304), K+304 (+304)	31.01	25.73
4151	seq=translation; coord=10:14302293..14305526:1; parent_transcript=GRMZM2G106622_T01; parent_gene=GRMZM2G106622	GRMZM2G106622_P01	TRUE	TRUE	iATVEPVTMk	95%	n+304 (+304), K+304 (+304)	29.29	25.00
4152	seq=translation; coord=10:14302293..14305526:1; parent_transcript=GRMZM2G106622_T01; parent_gene=GRMZM2G106622	GRMZM2G106622_P01	TRUE	TRUE	qMGPPAAPGAHPENQEAAR	95%	Pyro-cmC (-17), n+304 (+304)	74.39	25.00
4153	seq=translation; coord=10:14302293..14305526:1; parent_transcript=GRMZM2G106622_T01; parent_gene=GRMZM2G106622	GRMZM2G106622_P01	TRUE	TRUE	qmGPPAAPGAHPENQEAAR	95%	Pyro-cmC (-17), n+304 (+304), Oxidation (+16)	76.31	25.00
4154	seq=translation; coord=4:229408072..229414150:-1; parent_transcript=GRMZM2G162992_T02; parent_gene=GRMZM2G162992	GRMZM2G162992_P02	TRUE	TRUE	eVLAEADAGSSGAGSGGR	95%	n+304 (+304)	58.73	25.00
4155	seq=translation; coord=4:229408072..229414150:-1; parent_transcript=GRMZM2G162992_T02; parent_gene=GRMZM2G162992	GRMZM2G162992_P02	TRUE	TRUE	qLISEVTSENR	86%	n+304 (+304)	25.66	25.29

4156	seq=translation; coord=3:230006629..230017669:-1; parent_transcript=GRMZM2G006130_T01; parent_gene=GRMZM2G006130	GRMZM2G006130_P01,GRMZM2G006130_P02, GRMZM2G006130_P03,GRMZM2G006130_P04, GRMZM2G006130_P06,GRMZM2G006130_P07, GRMZM2G006130_P08	TRUE	TRUE	aFVSEYAVWR	95%	n+304 (+304)	40.06	25.00
4157	seq=translation; coord=3:230006629..230017669:-1; parent_transcript=GRMZM2G006130_T01; parent_gene=GRMZM2G006130	GRMZM2G006130_P01,GRMZM2G006130_P02, GRMZM2G006130_P03,GRMZM2G006130_P04, GRMZM2G006130_P06,GRMZM2G006130_P07, GRMZM2G006130_P08	TRUE	TRUE	dVLDSLEFAR	95%	n+304 (+304)	33.70	25.00
4158	seq=translation; coord=3:230006629..230017669:-1; parent_transcript=GRMZM2G006130_T01; parent_gene=GRMZM2G006130	GRMZM2G006130_P01,GRMZM2G006130_P02, GRMZM2G006130_P03,GRMZM2G006130_P04, GRMZM2G006130_P06,GRMZM2G006130_P07, GRMZM2G006130_P08	TRUE	TRUE	gSLLASLAEAAFLTGLEk	95%	n+304 (+304), K+304 (+304)	49.35	25.00
4159	seq=translation; coord=3:230006629..230017669:-1; parent_transcript=GRMZM2G006130_T01; parent_gene=GRMZM2G006130	GRMZM2G006130_P01,GRMZM2G006130_P02, GRMZM2G006130_P03,GRMZM2G006130_P04, GRMZM2G006130_P06,GRMZM2G006130_P07, GRMZM2G006130_P08	TRUE	TRUE	gVVWLDQVSLMPEDTYk	95%	n+304 (+304), K+304 (+304)	36.62	26.41
4160	seq=translation; coord=3:230006629..230017669:-1; parent_transcript=GRMZM2G006130_T01; parent_gene=GRMZM2G006130	GRMZM2G006130_P01,GRMZM2G006130_P02, GRMZM2G006130_P03,GRMZM2G006130_P04, GRMZM2G006130_P06,GRMZM2G006130_P07, GRMZM2G006130_P08	TRUE	TRUE	tELISMLLDLkPR	95%	n+304 (+304), K+304 (+304)	28.85	25.00
4161	seq=translation; coord=1:214899491..214901246:-1; parent_transcript=AC217050.4_FGT006; parent_gene=AC217050.4_FG006	AC217050.4_FGP006	TRUE	TRUE	eSAESTMMAYk	95%	n+304 (+304), K+304 (+304)	31.88	25.00
4162	seq=translation; coord=1:214899491..214901246:-1; parent_transcript=AC217050.4_FGT006; parent_gene=AC217050.4_FG006	AC217050.4_FGP006	TRUE	TRUE	tVDVEELTVEER	95%	n+304 (+304)	65.02	25.00
4163	seq=translation; coord=UNKNOWN:5070241..5074601:-1; parent_transcript=GRMZM2G351125_T01; parent_gene=GRMZM2G351125	GRMZM2G351125_P01	TRUE	TRUE	eAPEESTk	95%	n+304 (+304), K+304 (+304)	27.86	25.01
4164	seq=translation; coord=UNKNOWN:5070241..5074601:-1; parent_transcript=GRMZM2G351125_T01; parent_gene=GRMZM2G351125	GRMZM2G351125_P01	TRUE	TRUE	eVEVFSLR	95%	n+304 (+304)	43.05	25.00
4165	seq=translation; coord=UNKNOWN:5070241..5074601:-1; parent_transcript=GRMZM2G351125_T01; parent_gene=GRMZM2G351125	GRMZM2G351125_P01	TRUE	TRUE	sPPLPFLLSSLLQSR	95%	n+304 (+304)	51.81	25.00
4166	seq=translation; coord=4:197713557..197717259:-1; parent_transcript=GRMZM2G050218_T01; parent_gene=GRMZM2G050218	GRMZM2G050218_P01	TRUE	TRUE	dTTDATFVR	95%	n+304 (+304)	31.68	25.00
4167	seq=translation; coord=4:197713557..197717259:-1; parent_transcript=GRMZM2G050218_T01; parent_gene=GRMZM2G050218	GRMZM2G050218_P01	TRUE	TRUE	gFGFITYSDPAVVDk	94%	n+304 (+304), K+304 (+304)	29.01	25.82
4168	seq=translation; coord=4:197713557..197717259:-1; parent_transcript=GRMZM2G050218_T01; parent_gene=GRMZM2G050218	GRMZM2G050218_P01	TRUE	TRUE	lSSYGGVGDFGGAYGR	95%	n+304 (+304)	33.75	25.00
4169	seq=translation; coord=9:14322326..14327546:-1; parent_transcript=GRMZM2G014240_T01; parent_gene=GRMZM2G014240	GRMZM2G014240_P01	TRUE	TRUE	aADVGADLVGk	95%	n+304 (+304), K+304 (+304)	53.19	26.21
4170	seq=translation; coord=9:14322326..14327546:-1; parent_transcript=GRMZM2G014240_T01; parent_gene=GRMZM2G014240	GRMZM2G014240_P01	TRUE	TRUE	aGYADSLIEEEEGLNDHNVVVk	95%	n+304 (+304), K+304 (+304)	60.16	25.00
4171	seq=translation; coord=9:14322326..14327546:-1; parent_transcript=GRMZM2G014240_T01; parent_gene=GRMZM2G014240	GRMZM2G014240_P01	TRUE	TRUE	nIPEDDPR	95%	n+304 (+304)	37.80	25.00

4172	seq=translation; coord=10:1152679..1158648:-1; parent_transcript=GRMZM2G057441_T01; parent_gene=GRMZM2G057441	GRMZM2G057441_P01	TRUE	TRUE	aETFGIPIPGWAk	95%	n+304 (+304), K+304 (+304)	51.71	25.83
4173	seq=translation; coord=10:1152679..1158648:-1; parent_transcript=GRMZM2G057441_T01; parent_gene=GRMZM2G057441	GRMZM2G057441_P01	TRUE	TRUE	aVLNPMAAMFGGIVGQEVVk	95%	n+304 (+304), K+304 (+304)	60.85	25.97
4174	seq=translation; coord=10:1152679..1158648:-1; parent_transcript=GRMZM2G057441_T01; parent_gene=GRMZM2G057441	GRMZM2G057441_P01	TRUE	TRUE	lIDLAISINETLGDSk	95%	n+304 (+304), K+304 (+304)	59.08	25.00
4175	seq=translation; coord=5:193849196..193850728:-1; parent_transcript=GRMZM2G147014_T01; parent_gene=GRMZM2G147014	GRMZM2G147014_P01	TRUE	TRUE	sSAEEADHk	93%	n+304 (+304), K+304 (+304)	27.08	25.00
4176	seq=translation; coord=5:193849196..193850728:-1; parent_transcript=GRMZM2G147014_T01; parent_gene=GRMZM2G147014	GRMZM2G147014_P01	TRUE	TRUE	vTVSEPEk	89%	n+304 (+304), K+304 (+304)	27.67	26.05
4177	seq=translation; coord=8:25334083..25343016:1; parent_transcript=GRMZM2G373928_T01; parent_gene=GRMZM2G373928	GRMZM2G373928_P01,GRMZM2G373928_P02	TRUE	TRUE	iIGDVVDPFVR	95%	n+304 (+304)	33.17	25.85
4178	seq=translation; coord=8:25334083..25343016:1; parent_transcript=GRMZM2G373928_T01; parent_gene=GRMZM2G373928	GRMZM2G373928_P01,GRMZM2G373928_P02	TRUE	TRUE	vVFLLFQQLGR	95%	n+304 (+304)	36.78	25.20
4179	seq=translation; coord=4:147218569..147222484:-1; parent_transcript=GRMZM2G070343_T01; parent_gene=GRMZM2G070343	GRMZM2G070343_P01,GRMZM2G070343_P02, GRMZM2G070343_P03	TRUE	TRUE	dSVIGAVPESTLVTcIDk	95%	n+304 (+304), Carbamidomethyl (+57), K+304 (+304)	41.29	26.25
4180	seq=translation; coord=4:147218569..147222484:-1; parent_transcript=GRMZM2G070343_T01; parent_gene=GRMZM2G070343	GRMZM2G070343_P01,GRMZM2G070343_P02, GRMZM2G070343_P03	TRUE	TRUE	lNTDENPDIATQFGIR	95%	n+304 (+304)	83.42	25.00
4181	seq=translation; coord=4:147218569..147222484:-1; parent_transcript=GRMZM2G070343_T01; parent_gene=GRMZM2G070343	GRMZM2G070343_P01,GRMZM2G070343_P02, GRMZM2G070343_P03	TRUE	TRUE	sIPTMMIFk	93%	n+304 (+304), K+304 (+304)	27.34	25.00
4182	seq=translation; coord=2:2803488..2806329:1; parent_transcript=GRMZM2G102471_T01; parent_gene=GRMZM2G102471	GRMZM2G102471_P01,GRMZM2G102471_P03, GRMZM2G102471_P04,GRMZM2G102471_P06, GRMZM2G132759_P01,GRMZM2G132759_P02, GRMZM2G132759_P03,GRMZM2G132759_P04, GRMZM2G132759_P06,GRMZM2G165511_P01, GRMZM5G866947_P03,GRMZM5G866947_P04, GRMZM5G866947_P05	TRUE	TRUE	eQWSPALTISk	94%	n+304 (+304), K+304 (+304)	33.02	26.68
4183	seq=translation; coord=8:136129273..136131879:-1; parent_transcript=GRMZM2G100146_T01; parent_gene=GRMZM2G100146	GRMZM2G100146_P01	TRUE	TRUE	aVAATASk	95%	n+304 (+304), K+304 (+304)	36.93	25.44
4184	seq=translation; coord=8:136129273..136131879:-1; parent_transcript=GRMZM2G100146_T01; parent_gene=GRMZM2G100146	GRMZM2G100146_P01	TRUE	TRUE	lAIGTLSIDk	95%	n+304 (+304), K+304 (+304)	37.29	25.00
4185	seq=translation; coord=8:136129273..136131879:-1; parent_transcript=GRMZM2G100146_T01; parent_gene=GRMZM2G100146	GRMZM2G100146_P01	TRUE	TRUE	tTSVFFSGYk	92%	n+304 (+304), K+304 (+304)	28.25	25.68
4186	seq=translation; coord=8:136129273..136131879:-1; parent_transcript=GRMZM2G100146_T01; parent_gene=GRMZM2G100146	GRMZM2G100146_P01	TRUE	TRUE	vATPPAQk	91%	n+304 (+304), K+304 (+304)	26.93	25.00
4187	seq=translation; coord=6:6317365..6323357:-1; parent_transcript=GRMZM2G412470_T02; parent_gene=GRMZM2G412470	GRMZM2G412470_P02	TRUE	TRUE	aLTLGMESPAAEALIVk	95%	n+304 (+304), K+304 (+304)	59.01	25.00

4188	seq=translation; coord=6:6317365..6323357:-1; parent_transcript=GRMZM2G412470_T02; parent_gene=GRMZM2G412470	GRMZM2G412470_P02	TRUE	TRUE	aVVDDILPPAFLSNAk	95%	n+304 (+304), K+304 (+304)	33.79	25.00
4189	seq=translation; coord=6:6317365..6323357:-1; parent_transcript=GRMZM2G412470_T02; parent_gene=GRMZM2G412470	GRMZM2G412470_P02	TRUE	TRUE	dGLDDLALDIPDAR	95%	n+304 (+304)	67.72	25.00
4190	seq=translation; coord=6:6317365..6323357:-1; parent_transcript=GRMZM2G412470_T02; parent_gene=GRMZM2G412470	GRMZM2G412470_P02	TRUE	TRUE	eASEEcLVSSSQMMk	95%	n+304 (+304), Carbamidomethyl (+57), K+304 (+304)	28.94	25.00
4191	seq=translation; coord=6:6317365..6323357:-1; parent_transcript=GRMZM2G412470_T02; parent_gene=GRMZM2G412470	GRMZM2G412470_P02	TRUE	TRUE	eMASVLLPSLSMELFSSEDIAk	95%	n+304 (+304), K+304 (+304)	37.57	25.00
4192	seq=translation; coord=6:6317365..6323357:-1; parent_transcript=GRMZM2G412470_T02; parent_gene=GRMZM2G412470	GRMZM2G412470_P02	TRUE	TRUE	iAVQNAETQSLASPR	95%	n+304 (+304)	36.93	26.09
4193	seq=translation; coord=6:6317365..6323357:-1; parent_transcript=GRMZM2G412470_T02; parent_gene=GRMZM2G412470	GRMZM2G412470_P02	TRUE	TRUE	lIETDAEAcLDR	95%	n+304 (+304), Carbamidomethyl (+57)	45.39	25.00
4194	seq=translation; coord=6:6317365..6323357:-1; parent_transcript=GRMZM2G412470_T02; parent_gene=GRMZM2G412470	GRMZM2G412470_P02	TRUE	TRUE	tLNLLQEcFGEGIITINQMTk	95%	n+304 (+304), Carbamidomethyl (+57), K+304 (+304)	67.16	25.40
4195	seq=translation; coord=6:6317365..6323357:-1; parent_transcript=GRMZM2G412470_T02; parent_gene=GRMZM2G412470	GRMZM2G412470_P02	TRUE	TRUE	vAESLDDLILDIPSAk	95%	n+304 (+304), K+304 (+304)	52.92	25.00
4196	seq=translation; coord=6:6317365..6323357:-1; parent_transcript=GRMZM2G412470_T02; parent_gene=GRMZM2G412470	GRMZM2G412470_P02	TRUE	TRUE	vRDGLDDLALDIPDAR	95%	n+304 (+304)	51.26	25.65
4197	seq=translation; coord=10:82038497..82043288:1; parent_transcript=GRMZM5G828229_T02; parent_gene=GRMZM5G828229	GRMZM5G828229_P02	TRUE	TRUE	dVADADALVSSLGSAk	95%	n+304 (+304), K+304 (+304)	35.83	25.95
4198	seq=translation; coord=10:82038497..82043288:1; parent_transcript=GRMZM5G828229_T02; parent_gene=GRMZM5G828229	GRMZM5G828229_P02	TRUE	TRUE	kVWWQFYGDNVGEAVEVGNFDPk	95%	K+304 (+304), n+304 (+304), K+304 (+304)	53.46	25.04
4199	seq=translation; coord=10:82038497..82043288:1; parent_transcript=GRMZM5G828229_T02; parent_gene=GRMZM5G828229	GRMZM5G828229_P02	TRUE	TRUE	lQAATSVEDALEIAR	95%	n+304 (+304)	66.86	25.38
4200	seq=translation; coord=10:82038497..82043288:1; parent_transcript=GRMZM5G828229_T02; parent_gene=GRMZM5G828229	GRMZM5G828229_P02	TRUE	TRUE	tSVPGIFAIGDVAAFPLk	95%	n+304 (+304), K+304 (+304)	42.68	25.00
4201	seq=translation; coord=10:82038497..82043288:1; parent_transcript=GRMZM5G828229_T02; parent_gene=GRMZM5G828229	GRMZM5G828229_P02	TRUE	TRUE	vATFWIDSDSR	90%	n+304 (+304)	27.28	25.00
4202	seq=translation; coord=8:118103124..118103722:-1; parent_transcript=AC197705.4_FGT003; parent_gene=AC197705.4_FG003	AC197705.4_FGP003	TRUE	TRUE	dMSPPLEWYGVPEGAR	95%	n+304 (+304)	54.46	25.00
4203	seq=translation; coord=8:118103124..118103722:-1; parent_transcript=AC197705.4_FGT003; parent_gene=AC197705.4_FG003	AC197705.4_FGP003	TRUE	TRUE	dQLGGLQEGVNDWk	95%	n+304 (+304), K+304 (+304)	66.96	25.28
4204	seq=translation; coord=8:118103124..118103722:-1; parent_transcript=AC197705.4_FGT003; parent_gene=AC197705.4_FG003	AC197705.4_FGP003	TRUE	TRUE	kDMSPPLEWYGVPEGAR	95%	K+304 (+304), n+304 (+304)	61.85	25.00
4205	seq=translation; coord=3:59325157..59327502:1; parent_transcript=GRMZM2G107228_T01; parent_gene=GRMZM2G107228	GRMZM2G107228_P01	TRUE	TRUE	aAVEQScPQTVScADIVAFAAR	93%	n+304 (+304), Carbamidomethyl (+57), Carbamidomethyl (+57)	30.09	25.00

4206	seq=translation; coord=3:59325157..59327502:1; parent_transcript=GRMZM2G107228_T01; parent_gene=GRMZM2G107228	GRMZM2G107228_P01	TRUE	TRUE	lLPLNLGLFFSDNQLR	95%	n+304 (+304)	60.34	25.59
4207	seq=translation; coord=3:59325157..59327502:1; parent_transcript=GRMZM2G107228_T01; parent_gene=GRMZM2G107228	GRMZM2G107228_P01	TRUE	TRUE	mGNIQVLTGTQGQIR	95%	n+304 (+304)	47.92	25.69
4208	seq=translation; coord=3:5595065..5596815:-1; parent_transcript=GRMZM2G143165_T01; parent_gene=GRMZM2G143165	GRMZM2G143165_P01	TRUE	TRUE	gMFEDVNIILVGDHGMVGTcDR	95%	n+304 (+304), Carbamidomethyl (+57)	75.36	25.00
4209	seq=translation; coord=3:5595065..5596815:-1; parent_transcript=GRMZM2G143165_T01; parent_gene=GRMZM2G143165	GRMZM2G143165_P01	TRUE	TRUE	iPPIIGLVGEGYk	95%	n+304 (+304), K+304 (+304)	41.64	25.00
4210	seq=translation; coord=3:5595065..5596815:-1; parent_transcript=GRMZM2G143165_T01; parent_gene=GRMZM2G143165	GRMZM2G143165_P01	TRUE	TRUE	kLVFLEELAPWIELk	95%	K+304 (+304), n+304 (+304), K+304 (+304)	31.56	25.00
4211	seq=translation; coord=3:5595065..5596815:-1; parent_transcript=GRMZM2G143165_T01; parent_gene=GRMZM2G143165	GRMZM2G143165_P01	TRUE	TRUE	lSkPVVLLISSDGFR	95%	n+304 (+304), K+304 (+304)	37.14	25.00
4212	seq=translation; coord=3:5595065..5596815:-1; parent_transcript=GRMZM2G143165_T01; parent_gene=GRMZM2G143165	GRMZM2G143165_P01	TRUE	TRUE	lVFLEELAPWIELk	95%	n+304 (+304), K+304 (+304)	47.25	25.00
4213	seq=translation; coord=3:5595065..5596815:-1; parent_transcript=GRMZM2G143165_T01; parent_gene=GRMZM2G143165	GRMZM2G143165_P01	TRUE	TRUE	sDWVLSVTPLLAIRPPDGVSPAEVVAk	95%	n+304 (+304), K+304 (+304)	33.74	25.00
4214	seq=translation; coord=3:5595065..5596815:-1; parent_transcript=GRMZM2G143165_T01; parent_gene=GRMZM2G143165	GRMZM2G143165_P01	TRUE	TRUE	tIFAAHGPR	95%	n+304 (+304)	45.73	25.89
4215	seq=translation; coord=6:49040773..49051761:1; parent_transcript=GRMZM2G700926_T01; parent_gene=GRMZM2G700926	GRMZM2G700926_P01	TRUE	TRUE	fLHIIENSPVYPVIYDSNR	95%	n+304 (+304)	67.13	25.28
4216	seq=translation; coord=6:49040773..49051761:1; parent_transcript=GRMZM2G700926_T01; parent_gene=GRMZM2G700926	GRMZM2G700926_P01	TRUE	TRUE	iFEVGDVVMLDSSR	94%	n+304 (+304)	31.33	25.00
4217	seq=translation; coord=6:49040773..49051761:1; parent_transcript=GRMZM2G700926_T01; parent_gene=GRMZM2G700926	GRMZM2G700926_P01	TRUE	TRUE	mDVLLSDILGPIGISQDEk	95%	n+304 (+304), K+304 (+304)	57.25	25.21
4218	seq=translation; coord=6:49040773..49051761:1; parent_transcript=GRMZM2G700926_T01; parent_gene=GRMZM2G700926	GRMZM2G700926_P01	TRUE	TRUE	vSGFEEIMGLVDSIVk	95%	n+304 (+304), K+304 (+304)	38.79	25.00
4219	seq=translation; coord=6:49040773..49051761:1; parent_transcript=GRMZM2G700926_T01; parent_gene=GRMZM2G700926	GRMZM2G700926_P01	TRUE	TRUE	yDLLcLEGLAR	95%	n+304 (+304), Carbamidomethyl (+57)	33.12	25.00
4220	seq=translation; coord=3:106336887..106353717:1; parent_transcript=GRMZM2G014805_T01; parent_gene=GRMZM2G014805	GRMZM2G014805_P01	TRUE	TRUE	dPNQNQQIQNFGDPFLMVIR	95%	n+304 (+304)	39.00	25.00
4221	seq=translation; coord=3:106336887..106353717:1; parent_transcript=GRMZM2G014805_T01; parent_gene=GRMZM2G014805	GRMZM2G014805_P01	TRUE	TRUE	fTWTIESFSR	92%	n+304 (+304)	28.72	25.00
4222	seq=translation; coord=3:106336887..106353717:1; parent_transcript=GRMZM2G014805_T01; parent_gene=GRMZM2G014805	GRMZM2G014805_P01	TRUE	TRUE	lLEVFYHk	95%	n+304 (+304), K+304 (+304)	32.16	25.20
4223	seq=translation; coord=3:106336887..106353717:1; parent_transcript=GRMZM2G014805_T01; parent_gene=GRMZM2G014805	GRMZM2G014805_P01	TRUE	TRUE	vELSNPDTELR	95%	n+304 (+304)	34.17	25.00

4224	seq=translation; coord=1:175294828..175302644:-1; parent_transcript=GRMZM2G022365_T01; parent_gene=GRMZM2G022365	GRMZM2G022365_P01	TRUE	TRUE	aHPGGPAASDFDWGTGDTPR	95%	n+304 (+304)	85.81	25.00
4225	seq=translation; coord=1:175294828..175302644:-1; parent_transcript=GRMZM2G022365_T01; parent_gene=GRMZM2G022365	GRMZM2G022365_P01	TRUE	TRUE	dAETGQDAPSEDGTk	95%	n+304 (+304), K+304 (+304)	43.33	25.00
4226	seq=translation; coord=1:175294828..175302644:-1; parent_transcript=GRMZM2G022365_T01; parent_gene=GRMZM2G022365	GRMZM2G022365_P01	TRUE	TRUE	eAPETEEAk	95%	n+304 (+304), K+304 (+304)	29.24	25.00
4227	seq=translation; coord=1:175294828..175302644:-1; parent_transcript=GRMZM2G022365_T01; parent_gene=GRMZM2G022365	GRMZM2G022365_P01	TRUE	TRUE	lVAESAADAVAAPAPETk	95%	n+304 (+304), K+304 (+304)	39.72	25.22
4228	seq=translation; coord=1:175294828..175302644:-1; parent_transcript=GRMZM2G022365_T01; parent_gene=GRMZM2G022365	GRMZM2G022365_P01	TRUE	TRUE	sEIVFVSPTGEEIk	95%	n+304 (+304), K+304 (+304)	31.64	25.95
4229	seq=translation; coord=1:175294828..175302644:-1; parent_transcript=GRMZM2G022365_T01; parent_gene=GRMZM2G022365	GRMZM2G022365_P01	TRUE	TRUE	vFDSPEGEk	91%	n+304 (+304), K+304 (+304)	29.40	26.07
4230	seq=translation; coord=7:155957641..155960914:1; parent_transcript=GRMZM2G173863_T01; parent_gene=GRMZM2G173863	GRMZM2G173863_P01,GRMZM2G173863_P02	TRUE	TRUE	gILVQDQEPGVILFDIGLAHk	95%	n+304 (+304), K+304 (+304)	27.99	25.00
4231	seq=translation; coord=7:155957641..155960914:1; parent_transcript=GRMZM2G173863_T01; parent_gene=GRMZM2G173863	GRMZM2G173863_P01,GRMZM2G173863_P02	TRUE	TRUE	gVEGLSQEELFVWLPVk	95%	n+304 (+304), K+304 (+304)	42.15	25.05
4232	seq=translation; coord=7:155957641..155960914:1; parent_transcript=GRMZM2G173863_T01; parent_gene=GRMZM2G173863	GRMZM2G173863_P01,GRMZM2G173863_P02	TRUE	TRUE	gVESYTLDESTGLLEAR	95%	n+304 (+304)	43.99	25.00
4233	seq=translation; coord=5:9900674..9908640:1; parent_transcript=GRMZM2G096596_T01; parent_gene=GRMZM2G096596	GRMZM2G096596_P01	TRUE	TRUE	sDPAIMAYDEFVAEAVGR	95%	n+304 (+304)	32.09	25.00
4234	seq=translation; coord=5:9900674..9908640:1; parent_transcript=GRMZM2G096596_T01; parent_gene=GRMZM2G096596	GRMZM2G096596_P01	TRUE	TRUE	sVADSLPALAWVAFLGk	95%	n+304 (+304), K+304 (+304)	34.53	25.00
4235	seq=translation; coord=5:9900674..9908640:1; parent_transcript=GRMZM2G096596_T01; parent_gene=GRMZM2G096596	GRMZM2G096596_P01	TRUE	TRUE	tLAIDDcDSR	95%	n+304 (+304), Carbamidomethyl (+57)	50.48	25.00
4236	seq=translation; coord=5:9900674..9908640:1; parent_transcript=GRMZM2G096596_T01; parent_gene=GRMZM2G096596	GRMZM2G096596_P01	TRUE	TRUE	wVVENQVGk	94%	n+304 (+304), K+304 (+304)	29.31	25.83
4237	seq=translation; coord=2:222875577..222883048:1; parent_transcript=GRMZM2G172101_T01; parent_gene=GRMZM2G172101	GRMZM2G172101_P01,GRMZM2G172101_P02, GRMZM2G172101_P03	TRUE	TRUE	aLQPYMGGIEFLPFk	95%	n+304 (+304), K+304 (+304)	32.61	25.87
4238	seq=translation; coord=2:222875577..222883048:1; parent_transcript=GRMZM2G172101_T01; parent_gene=GRMZM2G172101	GRMZM2G172101_P01,GRMZM2G172101_P02, GRMZM2G172101_P03	TRUE	TRUE	fASVELVDEVIALDk	95%	n+304 (+304), K+304 (+304)	37.96	25.65
4239	seq=translation; coord=2:222875577..222883048:1; parent_transcript=GRMZM2G172101_T01; parent_gene=GRMZM2G172101	GRMZM2G172101_P01,GRMZM2G172101_P02, GRMZM2G172101_P03	TRUE	TRUE	iYPAELPIk	90%	n+304 (+304), K+304 (+304)	26.70	25.44
4240	seq=translation; coord=8:138510107..138515042:1; parent_transcript=GRMZM2G700683_T01; parent_gene=GRMZM2G700683	GRMZM2G700683_P01	TRUE	TRUE	aLAEEGYEVVLVNSNPATIMTDPGLAHR	95%	n+304 (+304)	85.63	25.00
4241	seq=translation; coord=8:138510107..138515042:1; parent_transcript=GRMZM2G700683_T01; parent_gene=GRMZM2G700683	GRMZM2G700683_P01	TRUE	TRUE	vPIITTVDGAR	95%	n+304 (+304)	40.43	26.13

4242	seq=translation; coord=7:6064236..6068299:1; parent_transcript=GRMZM2G025977_T01; parent_gene=GRMZM2G025977	GRMZM2G025977_P01,GRMZM2G025977_P02, GRMZM2G025977_P03	TRUE	TRUE	aVLAASGAEVVIGEVER	95%	n+304 (+304)	57.67	26.50
4243	seq=translation; coord=7:6064236..6068299:1; parent_transcript=GRMZM2G025977_T01; parent_gene=GRMZM2G025977	GRMZM2G025977_P01,GRMZM2G025977_P02, GRMZM2G025977_P03	TRUE	TRUE	dENPPDDPATITGEPDSIAMLHk	95%	n+304 (+304), K+304 (+304)	75.06	25.00
4244	seq=translation; coord=7:6064236..6068299:1; parent_transcript=GRMZM2G025977_T01; parent_gene=GRMZM2G025977	GRMZM2G025977_P01,GRMZM2G025977_P02, GRMZM2G025977_P03	TRUE	TRUE	dGVLDADAVVLALGPWSGR	95%	n+304 (+304)	44.40	26.46
4245	seq=translation; coord=7:6064236..6068299:1; parent_transcript=GRMZM2G025977_T01; parent_gene=GRMZM2G025977	GRMZM2G025977_P01,GRMZM2G025977_P02, GRMZM2G025977_P03	TRUE	TRUE	iVDLEPFSPAR	95%	n+304 (+304)	33.11	26.50
4246	seq=translation; coord=1:12146189..12150797:1; parent_transcript=GRMZM2G000823_T01; parent_gene=GRMZM2G000823	GRMZM2G000823_P01	TRUE	TRUE	iQAITLPMILTPPYk	95%	n+304 (+304), K+304 (+304)	29.88	25.00
4247	seq=translation; coord=1:12146189..12150797:1; parent_transcript=GRMZM2G000823_T01; parent_gene=GRMZM2G000823	GRMZM2G000823_P01	TRUE	TRUE	sWADVEEEEEAk	95%	n+304 (+304), K+304 (+304)	47.45	25.00
4248	seq=translation; coord=1:12146189..12150797:1; parent_transcript=GRMZM2G000823_T01; parent_gene=GRMZM2G000823	GRMZM2G000823_P01	TRUE	TRUE	vLISTDVLAR	95%	n+304 (+304)	31.70	25.00
4249	seq=translation; coord=8:116620898..116625716:1; parent_transcript=GRMZM5G874500_T02; parent_gene=GRMZM5G874500	GRMZM5G874500_P02,GRMZM5G874500_P03	TRUE	TRUE	aGLTEEQLQEQIEQR	95%	n+304 (+304)	83.58	25.00
4250	seq=translation; coord=8:116620898..116625716:1; parent_transcript=GRMZM5G874500_T02; parent_gene=GRMZM5G874500	GRMZM5G874500_P02,GRMZM5G874500_P03	TRUE	TRUE	lSILGLMPSSSLAEVLk	95%	n+304 (+304), K+304 (+304)	57.06	25.00
4251	seq=translation; coord=8:116620898..116625716:1; parent_transcript=GRMZM5G874500_T02; parent_gene=GRMZM5G874500	GRMZM5G874500_P02,GRMZM5G874500_P03	TRUE	TRUE	nPADFALWk	95%	n+304 (+304), K+304 (+304)	30.27	25.93
4252	seq=translation; coord=8:116620898..116625716:1; parent_transcript=GRMZM5G874500_T02; parent_gene=GRMZM5G874500	GRMZM5G874500_P02,GRMZM5G874500_P03	TRUE	TRUE	qPEDYIQALIALETELk	95%	n+304 (+304), K+304 (+304)	34.26	25.29
4253	seq=translation; coord=2:233433323..233435947:-1; parent_transcript=GRMZM2G018197_T01; parent_gene=GRMZM2G018197	GRMZM2G018197_P01	TRUE	TRUE	fVIWTESAFk	95%	n+304 (+304), K+304 (+304)	29.33	25.42
4254	seq=translation; coord=2:233433323..233435947:-1; parent_transcript=GRMZM2G018197_T01; parent_gene=GRMZM2G018197	GRMZM2G018197_P01	TRUE	TRUE	iETVPEMPLVISDSAESIEk	95%	n+304 (+304), K+304 (+304)	36.18	25.00
4255	seq=translation; coord=3:8746797..8754263:1; parent_transcript=GRMZM2G120271_T01; parent_gene=GRMZM2G120271	GRMZM2G120271_P01	TRUE	TRUE	dGIPEcGLLQNcLEELATR	95%	n+304 (+304), Carbamidomethyl (+57), Carbamidomethyl (+57)	50.06	25.00
4256	seq=translation; coord=3:8746797..8754263:1; parent_transcript=GRMZM2G120271_T01; parent_gene=GRMZM2G120271	GRMZM2G120271_P01	TRUE	TRUE	dVEGASTQWDDIQR	95%	n+304 (+304)	64.55	25.00
4257	seq=translation; coord=3:8746797..8754263:1; parent_transcript=GRMZM2G120271_T01; parent_gene=GRMZM2G120271	GRMZM2G120271_P01	TRUE	TRUE	iISTDcIPNYPDR	95%	n+304 (+304), Carbamidomethyl (+57)	54.76	25.00
4258	seq=translation; coord=1:288732967..288734700:1; parent_transcript=GRMZM2G164020_T01; parent_gene=GRMZM2G164020	GRMZM2G164020_P01,GRMZM2G164020_P02	TRUE	TRUE	aNPTHPPYAEMISEAVTSLk	95%	n+304 (+304), K+304 (+304)	35.46	25.00
4259	seq=translation; coord=1:288732967..288734700:1; parent_transcript=GRMZM2G164020_T01; parent_gene=GRMZM2G164020	GRMZM2G164020_P01,GRMZM2G164020_P02	TRUE	TRUE	lLLGQLk	94%	n+304 (+304), K+304 (+304)	30.30	25.00

4260	seq=translation; coord=1:288732967..288734700:1; parent_transcript=GRMZM2G164020_T01; parent_gene=GRMZM2G164020	GRMZM2G164020_P01,GRMZM2G164020_P02	TRUE	TRUE	tGSSQYAIAk	95%	n+304 (+304), K+304 (+304)	60.63	25.88
4261	seq=translation; coord=7:39219675..39225654:-1; parent_transcript=GRMZM2G471269_T04; parent_gene=GRMZM2G471269	GRMZM2G471269_P04	TRUE	TRUE	iVPFLGQMISEGIELGR	95%	n+304 (+304)	37.21	25.37
4262	seq=translation; coord=7:39219675..39225654:-1; parent_transcript=GRMZM2G471269_T04; parent_gene=GRMZM2G471269	GRMZM2G471269_P04	TRUE	TRUE	sLNFPVVDDWR	95%	n+304 (+304)	35.73	25.50
4263	seq=translation; coord=7:39219675..39225654:-1; parent_transcript=GRMZM2G471269_T04; parent_gene=GRMZM2G471269	GRMZM2G471269_P04	TRUE	TRUE	vSHILFVDSPVGAGFSFSR	95%	n+304 (+304)	36.93	26.33
4264	seq=translation; coord=7:39219675..39225654:-1; parent_transcript=GRMZM2G471269_T04; parent_gene=GRMZM2G471269	GRMZM2G471269_P04	TRUE	TRUE	wILDRPL	87%	n+304 (+304)	28.21	27.34
4265	seq=translation; coord=2:176000645..176001838:1; parent_transcript=GRMZM2G046961_T01; parent_gene=GRMZM2G046961	GRMZM2G046961_P01	TRUE	TRUE	aEEEDTVVVVGEQPR	95%	n+304 (+304)	38.64	25.00
4266	seq=translation; coord=2:176000645..176001838:1; parent_transcript=GRMZM2G046961_T01; parent_gene=GRMZM2G046961	GRMZM2G046961_P01	TRUE	TRUE	gGGYEDAVVVGGR	95%	n+304 (+304)	52.99	25.00
4267	seq=translation; coord=3:135219277..135223126:-1; parent_transcript=GRMZM2G150521_T01; parent_gene=GRMZM2G150521	GRMZM2G150521_P01	TRUE	TRUE	gFGFVTYTSSEEASAAITALDGk	94%	n+304 (+304), K+304 (+304)	25.65	25.00
4268	seq=translation; coord=3:135219277..135223126:-1; parent_transcript=GRMZM2G150521_T01; parent_gene=GRMZM2G150521	GRMZM2G150521_P01	TRUE	TRUE	lFVGGISYQTDDHSLR	95%	n+304 (+304)	75.20	25.00
4269	seq=translation; coord=3:135219277..135223126:-1; parent_transcript=GRMZM2G150521_T01; parent_gene=GRMZM2G150521	GRMZM2G150521_P01	TRUE	TRUE	yGQVIEAR	88%	n+304 (+304)	26.06	25.00
4270	seq=translation; coord=8:78643850..78648764:1; parent_transcript=GRMZM5G892645_T01; parent_gene=GRMZM5G892645	GRMZM5G892645_P01,GRMZM5G892645_P02, GRMZM5G892645_P04,GRMZM5G892645_P05	TRUE	TRUE	fMQDPMEIYVDDEAk	95%	n+304 (+304), K+304 (+304)	30.24	25.00
4271	seq=translation; coord=8:78643850..78648764:1; parent_transcript=GRMZM5G892645_T01; parent_gene=GRMZM5G892645	GRMZM5G892645_P01,GRMZM5G892645_P02, GRMZM5G892645_P04,GRMZM5G892645_P05	TRUE	TRUE	gLAITFVSSASDSDVLNQVQER	95%	n+304 (+304)	43.84	25.00
4272	seq=translation; coord=8:78643850..78648764:1; parent_transcript=GRMZM5G892645_T01; parent_gene=GRMZM5G892645	GRMZM5G892645_P01,GRMZM5G892645_P02, GRMZM5G892645_P04,GRMZM5G892645_P05	TRUE	TRUE	iLVATDLVGR	95%	n+304 (+304)	38.00	25.00
4273	seq=translation; coord=2:108630218..108681539:-1; parent_transcript=GRMZM2G158043_T02; parent_gene=GRMZM2G158043	GRMZM2G158043_P02	TRUE	TRUE	gSYASNPDGPSR	95%	n+304 (+304)	30.47	25.00
4274	seq=translation; coord=2:108630218..108681539:-1; parent_transcript=GRMZM2G158043_T02; parent_gene=GRMZM2G158043	GRMZM2G158043_P02	TRUE	TRUE	hDIIAALDk	94%	n+304 (+304), K+304 (+304)	26.54	25.65
4275	seq=translation; coord=2:108630218..108681539:-1; parent_transcript=GRMZM2G158043_T02; parent_gene=GRMZM2G158043	GRMZM2G158043_P02	TRUE	TRUE	iYLYGEGWNFGEVAENQR	94%	n+304 (+304)	27.33	25.00
4276	seq=translation; coord=2:108630218..108681539:-1; parent_transcript=GRMZM2G158043_T02; parent_gene=GRMZM2G158043	GRMZM2G158043_P02	TRUE	TRUE	lIVDDLLNWAVNYk	94%	n+304 (+304), K+304 (+304)	29.07	25.00
4277	seq=translation; coord=2:108630218..108681539:-1; parent_transcript=GRMZM2G158043_T02; parent_gene=GRMZM2G158043	GRMZM2G158043_P02	TRUE	TRUE	lIVDDLLNWAVNyk	94%	n+304 (+304), iTRAQ8plex (+304), K+304 (+304)	26.17	25.00

4278	seq=translation; coord=2:108630218..108681539:-1; parent_transcript=GRMZM2G158043_T02; parent_gene=GRMZM2G158043	GRMZM2G158043_P02	TRUE	TRUE	lTTASDIVQR	95%	n+304 (+304)	41.37	25.15
4279	seq=translation; coord=2:108630218..108681539:-1; parent_transcript=GRMZM2G158043_T02; parent_gene=GRMZM2G158043	GRMZM2G158043_P02	TRUE	TRUE	tPMDLSIDER	95%	n+304 (+304)	45.13	25.00
4280	seq=translation; coord=2:108630218..108681539:-1; parent_transcript=GRMZM2G158043_T02; parent_gene=GRMZM2G158043	GRMZM2G158043_P02	TRUE	TRUE	vELQPENDGLPSSVTQk	95%	n+304 (+304), K+304 (+304)	31.20	25.35
4281	seq=translation; coord=10:124299211..124301978:1; parent_transcript=GRMZM2G173878_T01; parent_gene=GRMZM2G173878	GRMZM2G173878_P01,GRMZM2G330430_P02	TRUE	TRUE	eTFNHLASWLEDAR	93%	n+304 (+304)	29.43	25.00
4282	seq=translation; coord=10:124299211..124301978:1; parent_transcript=GRMZM2G173878_T01; parent_gene=GRMZM2G173878	GRMZM2G173878_P01,GRMZM2G330430_P02	TRUE	TRUE	fQPVHDLTIGVEFGAR	95%	n+304 (+304)	35.65	26.16
4283	seq=translation; coord=10:124299211..124301978:1; parent_transcript=GRMZM2G173878_T01; parent_gene=GRMZM2G173878	GRMZM2G173878_P01,GRMZM2G330430_P02	TRUE	TRUE	gAAGALLVYDITR	95%	n+304 (+304)	34.12	25.48
4284	seq=translation; coord=10:124299211..124301978:1; parent_transcript=GRMZM2G173878_T01; parent_gene=GRMZM2G173878	GRMZM2G173878_P01,GRMZM2G330430_P02	TRUE	TRUE	lQIWDTAGQESFR	95%	n+304 (+304)	49.51	25.00
4285	seq=translation; coord=3:150265213..150269134:1; parent_transcript=GRMZM2G068665_T01; parent_gene=GRMZM2G068665	GRMZM2G068665_P01	TRUE	TRUE	dLALGLASQTVLGAATMVSQTGk	95%	n+304 (+304), K+304 (+304)	83.24	25.00
4286	seq=translation; coord=3:150265213..150269134:1; parent_transcript=GRMZM2G068665_T01; parent_gene=GRMZM2G068665	GRMZM2G068665_P01	TRUE	TRUE	gTLISAVVAAAk	95%	n+304 (+304), K+304 (+304)	33.94	25.00
4287	seq=translation; coord=3:150265213..150269134:1; parent_transcript=GRMZM2G068665_T01; parent_gene=GRMZM2G068665	GRMZM2G068665_P01	TRUE	TRUE	gVAASGVLPASAIR	95%	n+304 (+304)	36.43	25.50
4288	seq=translation; coord=3:150265213..150269134:1; parent_transcript=GRMZM2G068665_T01; parent_gene=GRMZM2G068665	GRMZM2G068665_P01	TRUE	TRUE	mQDLQDWSGQR	95%	n+304 (+304)	34.44	25.00
4289	seq=translation; coord=3:150265213..150269134:1; parent_transcript=GRMZM2G068665_T01; parent_gene=GRMZM2G068665	GRMZM2G068665_P01	TRUE	TRUE	qVLLQLRPR	95%	n+304 (+304)	29.66	25.00
4290	seq=translation; coord=3:150265213..150269134:1; parent_transcript=GRMZM2G068665_T01; parent_gene=GRMZM2G068665	GRMZM2G068665_P01	TRUE	TRUE	qVLLQLRPR	93%	Pyro-cmC (-17), n+304 (+304)	28.11	25.00
4291	seq=translation; coord=5:203926164..203934465:-1; parent_transcript=GRMZM2G150172_T01; parent_gene=GRMZM2G150172	GRMZM2G150172_P01	TRUE	TRUE	fPASQccAALk	95%	n+304 (+304), Carbamidomethyl (+57), Carbamidomethyl (+57), K+304 (+304)	42.47	25.00
4292	seq=translation; coord=5:203926164..203934465:-1; parent_transcript=GRMZM2G150172_T01; parent_gene=GRMZM2G150172	GRMZM2G150172_P01	TRUE	TRUE	mGLScADVPR	95%	n+304 (+304), Carbamidomethyl (+57)	47.04	25.00
4293	seq=translation; coord=8:65969534..65973268:1; parent_transcript=GRMZM2G021331_T01; parent_gene=GRMZM2G021331	GRMZM2G021331_P01	TRUE	TRUE	yDEGLPPILTALEVLDNDIR	95%	n+304 (+304)	61.64	25.63
4294	seq=translation; coord=8:65969534..65973268:1; parent_transcript=GRMZM2G021331_T01; parent_gene=GRMZM2G021331	GRMZM2G021331_P01	TRUE	TRUE	yDEGLPPILTALEVLDNDIR	95%	n+304 (+304), iTRAQ8plex (+304)	44.84	25.04

4295	seq=translation; coord=5:112928080..112930204:-1; parent_transcript=GRMZM2G144081_T01; parent_gene=GRMZM2G144081	GRMZM2G144081_P01,GRMZM2G144081_P02	TRUE	TRUE	dEGPSELYR	95%	n+304 (+304)	44.89	25.00
4296	seq=translation; coord=5:112928080..112930204:-1; parent_transcript=GRMZM2G144081_T01; parent_gene=GRMZM2G144081	GRMZM2G144081_P01,GRMZM2G144081_P02	TRUE	TRUE	dVYDNVAHAFVk	95%	n+304 (+304), K+304 (+304)	43.41	26.32
4297	seq=translation; coord=5:112928080..112930204:-1; parent_transcript=GRMZM2G144081_T01; parent_gene=GRMZM2G144081	GRMZM2G144081_P01,GRMZM2G144081_P02	TRUE	TRUE	iPIPTPLVAGALAGFASTLcTYPMELIk	95%	n+304 (+304), Carbamidomethyl (+57), K+304 (+304)	31.25	25.00
4298	seq=translation; coord=5:112928080..112930204:-1; parent_transcript=GRMZM2G144081_T01; parent_gene=GRMZM2G144081	GRMZM2G144081_P01,GRMZM2G144081_P02	TRUE	TRUE	qQQLGDLSLR	95%	n+304 (+304)	38.37	26.41
4299	seq=translation; coord=5:112928080..112930204:-1; parent_transcript=GRMZM2G144081_T01; parent_gene=GRMZM2G144081	GRMZM2G144081_P01,GRMZM2G144081_P02	TRUE	TRUE	rPGADVGPVATLLIGSAAGAIASSATFPLEVAR	95%	n+304 (+304)	35.34	25.00
4300	seq=translation; coord=3:8884623..8885815:-1; parent_transcript=GRMZM2G083810_T01; parent_gene=GRMZM2G083810	GRMZM2G083810_P01	TRUE	TRUE	eLPGAYAFVVDMPGLGTGDIk	93%	n+304 (+304), K+304 (+304)	27.13	25.43
4301	seq=translation; coord=3:8884623..8885815:-1; parent_transcript=GRMZM2G083810_T01; parent_gene=GRMZM2G083810	GRMZM2G083810_P01	TRUE	TRUE	fVLPDNADMDk	93%	n+304 (+304), K+304 (+304)	28.31	25.01
4302	seq=translation; coord=3:8884623..8885815:-1; parent_transcript=GRMZM2G083810_T01; parent_gene=GRMZM2G083810	GRMZM2G083810_P01	TRUE	TRUE	kFVLPDNADMDk	95%	K+304 (+304), n+304 (+304), K+304 (+304)	35.65	25.40
4303	seq=translation; coord=3:8884623..8885815:-1; parent_transcript=GRMZM2G083810_T01; parent_gene=GRMZM2G083810	GRMZM2G083810_P01	TRUE	TRUE	mFGLETPLMVALQHLLDVPDGDAGAGGDk	95%	n+304 (+304), K+304 (+304)	28.14	25.00
4304	seq=translation; coord=3:8884623..8885815:-1; parent_transcript=GRMZM2G083810_T01; parent_gene=GRMZM2G083810	GRMZM2G083810_P01	TRUE	TRUE	mFGLETPLMVALQHLLDVPDGDAGAGGDk	95%	Oxidation (+16), n+304 (+304), K+304 (+304)	27.66	25.00
4305	seq=translation; coord=3:8884623..8885815:-1; parent_transcript=GRMZM2G083810_T01; parent_gene=GRMZM2G083810	GRMZM2G083810_P01	TRUE	TRUE	mFGLETPLmVALQHLLDVPDGDAGAGGDk	95%	n+304 (+304), Oxidation (+16), K+304 (+304)	29.92	25.00
4306	seq=translation; coord=10:102318701..102320375:-1; parent_transcript=AC204711.3_FGT003; parent_gene=AC204711.3_FG003	AC204711.3_FGP003	TRUE	TRUE	nIEGGYTAWVESGLAVk	95%	n+304 (+304), K+304 (+304)	66.57	25.65
4307	seq=translation; coord=10:102318701..102320375:-1; parent_transcript=AC204711.3_FGT003; parent_gene=AC204711.3_FG003	AC204711.3_FGP003	TRUE	TRUE	sELAcADLMAAGFR	95%	n+304 (+304), Carbamidomethyl (+57)	54.43	25.00
4308	seq=translation; coord=10:102318701..102320375:-1; parent_transcript=AC204711.3_FGT003; parent_gene=AC204711.3_FG003	AC204711.3_FGP003	TRUE	TRUE	sGHVEDSLNVPYLFFTSQGR	95%	n+304 (+304)	42.14	25.00
4309	seq=translation; coord=6:31707585..31709218:-1; parent_transcript=GRMZM2G050412_T01; parent_gene=GRMZM2G050412	GRMZM2G050412_P01	TRUE	TRUE	aGVAVDSIEFTYTDDAGQR	95%	n+304 (+304)	92.10	25.00
4310	seq=translation; coord=6:31707585..31709218:-1; parent_transcript=GRMZM2G050412_T01; parent_gene=GRMZM2G050412	GRMZM2G050412_P01	TRUE	TRUE	eVSGTYGAFEGAITLTSLR	95%	n+304 (+304)	59.59	25.69
4311	seq=translation; coord=6:31707585..31709218:-1; parent_transcript=GRMZM2G050412_T01; parent_gene=GRMZM2G050412	GRMZM2G050412_P01	TRUE	TRUE	vVTDSPVYDGR	95%	n+304 (+304)	37.29	25.00
4312	seq=translation; coord=10:99379756..99383298:1; parent_transcript=GRMZM2G177203_T02; parent_gene=GRMZM2G177203	GRMZM2G177203_P02	TRUE	TRUE	nLVVPIIGETNLAAFR	95%	n+304 (+304)	38.76	25.00

4313	seq=translation; coord=9:128714706..128721565:1; parent_transcript=GRMZM2G095124_T01; parent_gene=GRMZM2G095124	GRMZM2G095124_P01	TRUE	TRUE	aSFLEAWk	94%	n+304 (+304), K+304 (+304)	29.64	26.33
4314	seq=translation; coord=9:128714706..128721565:1; parent_transcript=GRMZM2G095124_T01; parent_gene=GRMZM2G095124	GRMZM2G095124_P01	TRUE	TRUE	eMVPLFFEAMEALTk	95%	n+304 (+304), K+304 (+304)	42.77	25.98
4315	seq=translation; coord=9:128714706..128721565:1; parent_transcript=GRMZM2G095124_T01; parent_gene=GRMZM2G095124	GRMZM2G095124_P01	TRUE	TRUE	iPLHVFFGEDGk	95%	n+304 (+304), K+304 (+304)	38.09	26.02
4316	seq=translation; coord=9:128714706..128721565:1; parent_transcript=GRMZM2G095124_T01; parent_gene=GRMZM2G095124	GRMZM2G095124_P01	TRUE	TRUE	lAASNVFFIAk	95%	n+304 (+304), K+304 (+304)	33.49	25.00
4317	seq=translation; coord=9:128714706..128721565:1; parent_transcript=GRMZM2G095124_T01; parent_gene=GRMZM2G095124	GRMZM2G095124_P01	TRUE	TRUE	mAPPLVTLLSAEPEIQYVALR	95%	n+304 (+304)	29.00	25.00
4318	seq=translation; coord=9:128714706..128721565:1; parent_transcript=GRMZM2G095124_T01; parent_gene=GRMZM2G095124	GRMZM2G095124_P01	TRUE	TRUE	sQPDLAILAVNTFVk	95%	n+304 (+304), K+304 (+304)	49.47	25.00
4319	seq=translation; coord=2:214832665..214852459:1; parent_transcript=GRMZM2G035985_T01; parent_gene=GRMZM2G035985	GRMZM2G035985_P01,GRMZM2G035985_P02	TRUE	TRUE	iGIYNLEFSGLYATGDAVGAPVRPR	95%	n+304 (+304)	62.79	25.26
4320	seq=translation; coord=2:214832665..214852459:1; parent_transcript=GRMZM2G035985_T01; parent_gene=GRMZM2G035985	GRMZM2G035985_P01,GRMZM2G035985_P02	TRUE	TRUE	lLNVGDTLAATLcYIcAGNIDk	95%	n+304 (+304), Carbamidomethyl (+57), Carbamidomethyl (+57), K+304 (+304)	43.48	25.02
4321	seq=translation; coord=2:214832665..214852459:1; parent_transcript=GRMZM2G035985_T01; parent_gene=GRMZM2G035985	GRMZM2G035985_P01,GRMZM2G035985_P02	TRUE	TRUE	tYVDLLQDLMEk	95%	n+304 (+304), K+304 (+304)	28.85	25.00
4322	seq=translation; coord=4:234901650..234908817:1; parent_transcript=GRMZM2G143462_T01; parent_gene=GRMZM2G143462	GRMZM2G143462_P01,GRMZM2G143462_P02, GRMZM2G143462_P03,GRMZM2G143462_P04, GRMZM2G143462_P05,GRMZM2G143462_P06	TRUE	TRUE	nIEEIQGk	94%	n+304 (+304), K+304 (+304)	31.03	25.62
4323	seq=translation; coord=4:234901650..234908817:1; parent_transcript=GRMZM2G143462_T01; parent_gene=GRMZM2G143462	GRMZM2G143462_P01,GRMZM2G143462_P02, GRMZM2G143462_P03,GRMZM2G143462_P04, GRMZM2G143462_P05,GRMZM2G143462_P06	TRUE	TRUE	nVDTIINQLMEMGGGSWDkDk	95%	n+304 (+304), K+304 (+304), K+304 (+304)	60.87	25.53
4324	seq=translation; coord=4:234901650..234908817:1; parent_transcript=GRMZM2G143462_T01; parent_gene=GRMZM2G143462	GRMZM2G143462_P01,GRMZM2G143462_P02, GRMZM2G143462_P03,GRMZM2G143462_P04, GRMZM2G143462_P05,GRMZM2G143462_P06	TRUE	TRUE	vNEDGFLVVMLSk	95%	n+304 (+304), K+304 (+304)	37.12	25.60
4325	seq=translation; coord=4:234901650..234908817:1; parent_transcript=GRMZM2G143462_T01; parent_gene=GRMZM2G143462	GRMZM2G143462_P01,GRMZM2G143462_P02, GRMZM2G143462_P03,GRMZM2G143462_P04, GRMZM2G143462_P05,GRMZM2G143462_P06	TRUE	TRUE	vQPNDTIMAVk	95%	n+304 (+304), K+304 (+304)	48.20	26.02
4326	seq=translation; coord=2:212405484..212408361:-1; parent_transcript=GRMZM5G879278_T02; parent_gene=GRMZM5G879278	GRMZM5G879278_P02,GRMZM5G879278_P03, GRMZM5G879278_P04	TRUE	TRUE	eLSGTLLSEDDSk	95%	n+304 (+304), K+304 (+304)	34.20	26.17
4327	seq=translation; coord=2:212405484..212408361:-1; parent_transcript=GRMZM5G879278_T02; parent_gene=GRMZM5G879278	GRMZM5G879278_P02,GRMZM5G879278_P03, GRMZM5G879278_P04	TRUE	TRUE	fTDLAGNIFk	95%	n+304 (+304), K+304 (+304)	43.59	25.01
4328	seq=translation; coord=2:212405484..212408361:-1; parent_transcript=GRMZM5G879278_T02; parent_gene=GRMZM5G879278	GRMZM5G879278_P02,GRMZM5G879278_P03, GRMZM5G879278_P04	TRUE	TRUE	gDETPPGTAEk	93%	n+304 (+304), K+304 (+304)	26.68	25.00

4329	seq=translation; coord=6:157814627..157816731:-1; parent_transcript=GRMZM2G097040_T01; parent_gene=GRMZM2G097040	GRMZM2G097040_P01	TRUE	TRUE	eIEETLLEHLGVk	95%	n+304 (+304), K+304 (+304)	30.67	25.00
4330	seq=translation; coord=6:157814627..157816731:-1; parent_transcript=GRMZM2G097040_T01; parent_gene=GRMZM2G097040	GRMZM2G097040_P01	TRUE	TRUE	rVVEIVEMLR	95%	n+304 (+304)	42.54	25.00
4331	seq=translation; coord=6:157814627..157816731:-1; parent_transcript=GRMZM2G097040_T01; parent_gene=GRMZM2G097040	GRMZM2G097040_P01	TRUE	TRUE	vVEIVEMLR	91%	n+304 (+304)	28.71	25.33
4332	seq=translation; coord=5:120784559..120792367:1; parent_transcript=GRMZM2G093359_T01; parent_gene=GRMZM2G093359	GRMZM2G093359_P01,GRMZM2G093359_P02	TRUE	TRUE	sFVEVAAGSHFPIQNLPFGVFR	95%	n+304 (+304)	56.96	25.38
4333	seq=translation; coord=3:185834707..185839417:-1; parent_transcript=GRMZM2G003385_T01; parent_gene=GRMZM2G003385	GRMZM2G003385_P01,GRMZM2G003385_P02	TRUE	TRUE	aLQIAEk	89%	n+304 (+304), K+304 (+304)	29.89	28.03
4334	seq=translation; coord=3:185834707..185839417:-1; parent_transcript=GRMZM2G003385_T01; parent_gene=GRMZM2G003385	GRMZM2G003385_P01,GRMZM2G003385_P02	TRUE	TRUE	eEYVEVPSDSGITFNVAPk	95%	n+304 (+304), K+304 (+304)	37.78	25.80
4335	seq=translation; coord=5:188243871..188290615:-1; parent_transcript=GRMZM5G824831_T01; parent_gene=GRMZM5G824831	GRMZM5G824831_P01,GRMZM5G824831_P02, GRMZM5G824831_P03,GRMZM5G824831_P04, GRMZM5G824831_P05,GRMZM5G824831_P06, GRMZM5G824831_P07,GRMZM5G824831_P08	TRUE	TRUE	eGYEGQISSNNIEIGIIR	95%	n+304 (+304)	64.44	25.00
4336	seq=translation; coord=5:188243871..188290615:-1; parent_transcript=GRMZM5G824831_T01; parent_gene=GRMZM5G824831	GRMZM5G824831_P01,GRMZM5G824831_P02, GRMZM5G824831_P03,GRMZM5G824831_P04, GRMZM5G824831_P05,GRMZM5G824831_P06, GRMZM5G824831_P07,GRMZM5G824831_P08	TRUE	TRUE	lVQIEHALTAVGSGQTSLGIk	95%	n+304 (+304), K+304 (+304)	26.98	25.00
4337	seq=translation; coord=5:188243871..188290615:-1; parent_transcript=GRMZM5G824831_T01; parent_gene=GRMZM5G824831	GRMZM5G824831_P01,GRMZM5G824831_P02, GRMZM5G824831_P03,GRMZM5G824831_P04, GRMZM5G824831_P05,GRMZM5G824831_P06, GRMZM5G824831_P07,GRMZM5G824831_P08	TRUE	TRUE	vLSPAEIkDFLEEVE	90%	n+304 (+304), K+304 (+304)	26.21	25.93
4338	seq=translation; coord=5:188243871..188290615:-1; parent_transcript=GRMZM5G824831_T01; parent_gene=GRMZM5G824831	GRMZM5G824831_P01,GRMZM5G824831_P02, GRMZM5G824831_P03,GRMZM5G824831_P04, GRMZM5G824831_P05,GRMZM5G824831_P06, GRMZM5G824831_P07,GRMZM5G824831_P08	TRUE	TRUE	yTEDMELDDAIHTAILTLk	95%	n+304 (+304), K+304 (+304)	36.28	25.95
4339	seq=translation; coord=2:32257322..32262715:1; parent_transcript=GRMZM2G157061_T01; parent_gene=GRMZM2G157061	GRMZM2G157061_P01	TRUE	TRUE	eIVDVSEEILR	95%	n+304 (+304)	39.05	26.78
4340	seq=translation; coord=2:32257322..32262715:1; parent_transcript=GRMZM2G157061_T01; parent_gene=GRMZM2G157061	GRMZM2G157061_P01	TRUE	TRUE	fEALEVILk	95%	n+304 (+304), K+304 (+304)	36.90	25.00
4341	seq=translation; coord=2:32257322..32262715:1; parent_transcript=GRMZM2G157061_T01; parent_gene=GRMZM2G157061	GRMZM2G157061_P01	TRUE	TRUE	lLPQGSGAALIGk	92%	n+304 (+304), K+304 (+304)	25.87	25.00
4342	seq=translation; coord=2:32257322..32262715:1; parent_transcript=GRMZM2G157061_T01; parent_gene=GRMZM2G157061	GRMZM2G157061_P01	TRUE	TRUE	nDSTVQGQAQAQTQAQNR	95%	n+304 (+304)	28.32	25.00
4343	seq=translation; coord=6:112268596..112280791:-1; parent_transcript=GRMZM2G553687_T01; parent_gene=GRMZM2G553687	GRMZM2G553687_P01	TRUE	TRUE	acVQSADAVPALLWLLk	95%	n+304 (+304), Carbamidomethyl (+57), K+304 (+304)	52.89	25.00

4344	seq=translation; coord=6:112268596..112280791:-1; parent_transcript=GRMZM2G553687_T01; parent_gene=GRMZM2G553687	GRMZM2G553687_P01	TRUE	TRUE	aDMGPEGQYSTLQVLVNILEHPEcR	95%	n+304 (+304), Carbamidomethyl (+57)	38.80	25.00
4345	seq=translation; coord=6:112268596..112280791:-1; parent_transcript=GRMZM2G553687_T01; parent_gene=GRMZM2G553687	GRMZM2G553687_P01	TRUE	TRUE	dTAFGAVGSHSQAVPILVSLLR	95%	n+304 (+304)	38.06	25.00
4346	seq=translation; coord=6:112268596..112280791:-1; parent_transcript=GRMZM2G553687_T01; parent_gene=GRMZM2G553687	GRMZM2G553687_P01	TRUE	TRUE	lLAALALGDLFQNEGLAR	95%	n+304 (+304)	31.61	25.00
4347	seq=translation; coord=6:112268596..112280791:-1; parent_transcript=GRMZM2G553687_T01; parent_gene=GRMZM2G553687	GRMZM2G553687_P01	TRUE	TRUE	vVQPLFSLLSk	95%	n+304 (+304), K+304 (+304)	39.31	25.00
4348	seq=translation; coord=3:34547144..34558771:1; parent_transcript=EF517601.1_FGT012; parent_gene=EF517601.1_FG012	EF517601.1_FGP012	TRUE	TRUE	iHPMTIIAGYR	95%	n+304 (+304)	30.50	26.71
4349	seq=translation; coord=3:34547144..34558771:1; parent_transcript=EF517601.1_FGT012; parent_gene=EF517601.1_FG012	EF517601.1_FGP012	TRUE	TRUE	iLVANTAMDTDk	93%	n+304 (+304), K+304 (+304)	27.95	26.67
4350	seq=translation; coord=3:34547144..34558771:1; parent_transcript=EF517601.1_FGT012; parent_gene=EF517601.1_FG012	EF517601.1_FGP012	TRUE	TRUE	qAILLSATEAAEMILR	95%	n+304 (+304)	59.96	25.02
4351	seq=translation; coord=3:34547144..34558771:1; parent_transcript=EF517601.1_FGT012; parent_gene=EF517601.1_FG012	EF517601.1_FGP012	TRUE	TRUE	sDLMNIAMTTLSSk	95%	n+304 (+304), K+304 (+304)	39.88	25.74
4352	seq=translation; coord=3:34547144..34558771:1; parent_transcript=EF517601.1_FGT012; parent_gene=EF517601.1_FG012	EF517601.1_FGP012	TRUE	TRUE	vLFGGGWPEMVMAk	95%	n+304 (+304), K+304 (+304)	31.31	25.98
4353	seq=translation; coord=3:162825591..162831818:-1; parent_transcript=GRMZM2G133173_T01; parent_gene=GRMZM2G133173	GRMZM2G133173_P01	TRUE	TRUE	aEIGISDNDAGAVESVQk	95%	n+304 (+304), K+304 (+304)	45.34	25.31
4354	seq=translation; coord=3:162825591..162831818:-1; parent_transcript=GRMZM2G133173_T01; parent_gene=GRMZM2G133173	GRMZM2G133173_P01	TRUE	TRUE	aEISHIFR	95%	n+304 (+304)	45.29	26.63
4355	seq=translation; coord=3:162825591..162831818:-1; parent_transcript=GRMZM2G133173_T01; parent_gene=GRMZM2G133173	GRMZM2G133173_P01	TRUE	TRUE	vDkAEIGISDNDAGAVESVQk	95%	n+304 (+304), K+304 (+304), K+304 (+304)	35.21	25.00
4356	seq=translation; coord=1:143622295..143626633:1; parent_transcript=GRMZM2G152526_T01; parent_gene=GRMZM2G152526	GRMZM2G152526_P01	TRUE	TRUE	dAGGYGGSSYGR	95%	n+304 (+304)	47.55	25.00
4357	seq=translation; coord=1:143622295..143626633:1; parent_transcript=GRMZM2G152526_T01; parent_gene=GRMZM2G152526	GRMZM2G152526_P01	TRUE	TRUE	gFGFITFADPTVVDR	93%	n+304 (+304)	29.51	25.00
4358	seq=translation; coord=1:143622295..143626633:1; parent_transcript=GRMZM2G152526_T01; parent_gene=GRMZM2G152526	GRMZM2G152526_P01	TRUE	TRUE	gFGFIVFDAEk	95%	n+304 (+304), K+304 (+304)	34.44	25.33
4359	seq=translation; coord=1:143622295..143626633:1; parent_transcript=GRMZM2G152526_T01; parent_gene=GRMZM2G152526	GRMZM2G152526_P01	TRUE	TRUE	iFIGGLSk	95%	n+304 (+304), K+304 (+304)	32.24	25.00
4360	seq=translation; coord=1:143622295..143626633:1; parent_transcript=GRMZM2G152526_T01; parent_gene=GRMZM2G152526	GRMZM2G152526_P01	TRUE	TRUE	yGDIIDAVIMk	95%	n+304 (+304), K+304 (+304)	32.13	25.58
4361	seq=translation; coord=10:90200523..90201913:-1; parent_transcript=GRMZM2G132093_T01; parent_gene=GRMZM2G132093	GRMZM2G132093_P01	TRUE	TRUE	aLLALEEAGVDYELVPMSR	95%	n+304 (+304)	46.69	25.05

4362	seq=translation; coord=10:90200523..90201913:-1; parent_transcript=GRMZM2G132093_T01; parent_gene=GRMZM2G132093	GRMZM2G132093_P01	TRUE	TRUE	vPVLEDGDLTLFESR	95%	n+304 (+304)	41.25	25.40
4363	seq=translation; coord=5:12565068..12568390:1; parent_transcript=GRMZM2G111566_T01; parent_gene=GRMZM2G111566	GRMZM2G111566_P01	TRUE	TRUE	dFAVVAADTSAVQSILVHk	95%	n+304 (+304), K+304 (+304)	44.82	25.00
4364	seq=translation; coord=5:12565068..12568390:1; parent_transcript=GRMZM2G111566_T01; parent_gene=GRMZM2G111566	GRMZM2G111566_P01	TRUE	TRUE	gELATALR	90%	n+304 (+304)	27.03	25.71
4365	seq=translation; coord=5:12565068..12568390:1; parent_transcript=GRMZM2G111566_T01; parent_gene=GRMZM2G111566	GRMZM2G111566_P01	TRUE	TRUE	lVVAPQNFVIk	95%	n+304 (+304), K+304 (+304)	44.89	25.00
4366	seq=translation; coord=5:12565068..12568390:1; parent_transcript=GRMZM2G111566_T01; parent_gene=GRMZM2G111566	GRMZM2G111566_P01	TRUE	TRUE	nTIPLSTAAAANFTR	95%	n+304 (+304)	51.97	25.26
4367	seq=translation; coord=5:12565068..12568390:1; parent_transcript=GRMZM2G111566_T01; parent_gene=GRMZM2G111566	GRMZM2G111566_P01	TRUE	TRUE	vQFTEFIQk	95%	n+304 (+304), K+304 (+304)	37.36	25.68
4368	seq=translation; coord=9:22655251..22657461:1; parent_transcript=GRMZM2G092296_T01; parent_gene=GRMZM2G092296	GRMZM2G092296_P01	TRUE	TRUE	aGFEAGMMDPQHR	95%	n+304 (+304)	36.94	25.00
4369	seq=translation; coord=9:22655251..22657461:1; parent_transcript=GRMZM2G092296_T01; parent_gene=GRMZM2G092296	GRMZM2G092296_P01	TRUE	TRUE	aGFEAGMmDPQHR	95%	n+304 (+304), Oxidation (+16)	48.18	25.00
4370	seq=translation; coord=9:22655251..22657461:1; parent_transcript=GRMZM2G092296_T01; parent_gene=GRMZM2G092296	GRMZM2G092296_P01	TRUE	TRUE	vIDLVSTPDVVk	95%	n+304 (+304), K+304 (+304)	33.09	25.00
4371	seq=translation; coord=9:6307326..6312829:-1; parent_transcript=GRMZM2G082198_T01; parent_gene=GRMZM2G082198	GRMZM2G082198_P01	TRUE	TRUE	eMEEEAAALR	95%	n+304 (+304)	32.83	25.00
4372	seq=translation; coord=9:6307326..6312829:-1; parent_transcript=GRMZM2G082198_T01; parent_gene=GRMZM2G082198	GRMZM2G082198_P01	TRUE	TRUE	gFAYVEFVEVEAVQEAIk	95%	n+304 (+304), K+304 (+304)	61.72	25.42
4373	seq=translation; coord=9:6307326..6312829:-1; parent_transcript=GRMZM2G082198_T01; parent_gene=GRMZM2G082198	GRMZM2G082198_P01	TRUE	TRUE	vTILTDk	91%	n+304 (+304), K+304 (+304)	26.94	25.00
4374	seq=translation; coord=7:120200894..120203011:1; parent_transcript=GRMZM2G138727_T01; parent_gene=GRMZM2G138727	GRMZM2G138727_P01	TRUE	TRUE	hQcSPTATPYcSPQcQSLR	95%	n+304 (+304), Carbamidomethyl (+57), Carbamidomethyl (+57), Carbamidomethyl (+57)	40.95	25.00
4375	seq=translation; coord=7:120200894..120203011:1; parent_transcript=GRMZM2G138727_T01; parent_gene=GRMZM2G138727	GRMZM2G138727_P01	TRUE	TRUE	qQccQQLR	95%	n+304 (+304), Carbamidomethyl (+57), Carbamidomethyl (+57)	34.33	25.00
4376	seq=translation; coord=7:120200894..120203011:1; parent_transcript=GRMZM2G138727_T01; parent_gene=GRMZM2G138727	GRMZM2G138727_P01	TRUE	TRUE	qVEPQHR	95%	n+304 (+304)	28.95	25.00
4377	seq=translation; coord=2:34985296..34990180:-1; parent_transcript=GRMZM2G021219_T01; parent_gene=GRMZM2G021219	GRMZM2G021219_P01	TRUE	TRUE	aAATDSVQPEAAATETAAAEPANR	95%	n+304 (+304)	67.66	25.00
4378	seq=translation; coord=2:34985296..34990180:-1; parent_transcript=GRMZM2G021219_T01; parent_gene=GRMZM2G021219	GRMZM2G021219_P01	TRUE	TRUE	iVLIPIVVAVDcPFPPSDk	95%	n+304 (+304), Carbamidomethyl (+57), K+304 (+304)	52.75	25.00

4379	seq=translation; coord=2:34985296..34990180:-1; parent_transcript=GRMZM2G021219_T01; parent_gene=GRMZM2G021219	GRMZM2G021219_P01	TRUE	TRUE	mAWVPYVPLEDR	92%	n+304 (+304)	28.46	25.00
4380	seq=translation; coord=2:34985296..34990180:-1; parent_transcript=GRMZM2G021219_T01; parent_gene=GRMZM2G021219	GRMZM2G021219_P01	TRUE	TRUE	nLEDLWLSAFPVGTEWENIDk	95%	n+304 (+304), K+304 (+304)	70.41	25.04
4381	seq=translation; coord=2:17796828..17799226:-1; parent_transcript=GRMZM2G101408_T01; parent_gene=GRMZM2G101408	GRMZM2G101408_P01,GRMZM2G101408_P02, GRMZM2G101408_P03	TRUE	TRUE	aFEEAELPk	95%	n+304 (+304), K+304 (+304)	51.32	25.67
4382	seq=translation; coord=2:17796828..17799226:-1; parent_transcript=GRMZM2G101408_T01; parent_gene=GRMZM2G101408	GRMZM2G101408_P01,GRMZM2G101408_P02, GRMZM2G101408_P03	TRUE	TRUE	vAEEEEYER	94%	n+304 (+304)	31.17	25.00
4383	seq=translation; coord=6:86343600..86346720:-1; parent_transcript=GRMZM2G082823_T01; parent_gene=GRMZM2G082823	GRMZM2G082823_P01	TRUE	TRUE	fVGAFPAVVLALPSLR	95%	n+304 (+304)	47.62	25.00
4384	seq=translation; coord=6:86343600..86346720:-1; parent_transcript=GRMZM2G082823_T01; parent_gene=GRMZM2G082823	GRMZM2G082823_P01	TRUE	TRUE	gPVPPAVcGLQGTLR	95%	n+304 (+304), Carbamidomethyl (+57)	48.73	25.69
4385	seq=translation; coord=8:160297692..160299701:-1; parent_transcript=GRMZM2G105005_T01; parent_gene=GRMZM2G105005	GRMZM2G105005_P01,GRMZM2G105005_P02, GRMZM2G105005_P03	TRUE	TRUE	sALDEVTDTGAFDR	95%	n+304 (+304)	52.04	25.00
4386	seq=translation; coord=8:160297692..160299701:-1; parent_transcript=GRMZM2G105005_T01; parent_gene=GRMZM2G105005	GRMZM2G105005_P01,GRMZM2G105005_P02, GRMZM2G105005_P03	TRUE	TRUE	yIcGDQLTEADIR	95%	n+304 (+304), Carbamidomethyl (+57)	34.67	25.00
4387	seq=translation; coord=5:14072358..14073760:-1; parent_transcript=GRMZM2G165901_T01; parent_gene=GRMZM2G165901	GRMZM2G165901_P01	TRUE	TRUE	dGGYGGGGGYGGR	95%	n+304 (+304)	38.13	25.00
4388	seq=translation; coord=5:14072358..14073760:-1; parent_transcript=GRMZM2G165901_T01; parent_gene=GRMZM2G165901	GRMZM2G165901_P01	TRUE	TRUE	eGGGGGYGGGGGYGGR	95%	n+304 (+304)	47.42	25.00
4389	seq=translation; coord=7:5095194..5100761:-1; parent_transcript=GRMZM2G011858_T01; parent_gene=GRMZM2G011858	GRMZM2G011858_P01	TRUE	TRUE	ePYTATIVSVER	95%	n+304 (+304)	35.12	25.00
4390	seq=translation; coord=4:197062103..197070522:-1; parent_transcript=GRMZM2G016296_T01; parent_gene=GRMZM2G016296	GRMZM2G016296_P01,GRMZM2G016296_P02, GRMZM2G016296_P03,GRMZM2G016296_P04, GRMZM2G016296_P05,GRMZM2G016296_P06, GRMZM2G110143_P01,GRMZM2G110143_P02, GRMZM2G110143_P04,GRMZM2G110143_P05	TRUE	TRUE	tTADDLFPLFER	94%	n+304 (+304)	30.76	25.00
4391	seq=translation; coord=4:136936001..136939330:1; parent_transcript=GRMZM2G044684_T01; parent_gene=GRMZM2G044684	GRMZM2G044684_P01	TRUE	TRUE	eESSEFEEGIILQEFR	95%	n+304 (+304)	64.88	25.00
4392	seq=translation; coord=4:136936001..136939330:1; parent_transcript=GRMZM2G044684_T01; parent_gene=GRMZM2G044684	GRMZM2G044684_P01	TRUE	TRUE	fGDDDPTAVEGSVAPQk	92%	n+304 (+304), K+304 (+304)	25.81	25.00
4393	seq=translation; coord=4:136936001..136939330:1; parent_transcript=GRMZM2G044684_T01; parent_gene=GRMZM2G044684	GRMZM2G044684_P01	TRUE	TRUE	vSAGPGPEk	95%	n+304 (+304), K+304 (+304)	34.11	25.49
4394	seq=translation; coord=9:64028902..64031969:1; parent_transcript=GRMZM2G469898_T01; parent_gene=GRMZM2G469898	GRMZM2G469898_P01,GRMZM2G469898_P02	TRUE	TRUE	aALQFVVNPGk	95%	n+304 (+304), K+304 (+304)	35.16	25.00
4395	seq=translation; coord=9:64028902..64031969:1; parent_transcript=GRMZM2G469898_T01; parent_gene=GRMZM2G469898	GRMZM2G469898_P01,GRMZM2G469898_P02	TRUE	TRUE	gANFAITGATALEYSFFk	95%	n+304 (+304), K+304 (+304)	67.36	25.37

4396	seq=translation; coord=9:64028902..64031969:1; parent_transcript=GRMZM2G469898_T01; parent_gene=GRMZM2G469898	GRMZM2G469898_P01,GRMZM2G469898_P02	TRUE	TRUE	lVVDFLAEHFGLPLPPPSQAk	95%	n+304 (+304), K+304 (+304)	45.53	25.00
4397	seq=translation; coord=9:64028902..64031969:1; parent_transcript=GRMZM2G469898_T01; parent_gene=GRMZM2G469898	GRMZM2G469898_P01,GRMZM2G469898_P02	TRUE	TRUE	sLFVVGEFGGNDYNAPLFSGVPFSDVk	92%	n+304 (+304), K+304 (+304)	26.55	25.00
4398	seq=translation; coord=9:64028902..64031969:1; parent_transcript=GRMZM2G469898_T01; parent_gene=GRMZM2G469898	GRMZM2G469898_P01,GRMZM2G469898_P02	TRUE	TRUE	tYVPLVAk	94%	n+304 (+304), K+304 (+304)	30.41	25.00
4399	seq=translation; coord=3:202766518..202768685:-1; parent_transcript=GRMZM2G181135_T01; parent_gene=GRMZM2G181135	GRMZM2G181135_P01	TRUE	TRUE	aYEYVQSSIMGASR	95%	n+304 (+304)	63.57	25.00
4400	seq=translation; coord=3:202766518..202768685:-1; parent_transcript=GRMZM2G181135_T01; parent_gene=GRMZM2G181135	GRMZM2G181135_P01	TRUE	TRUE	lSYATTDGLFQSVELENHIR	95%	n+304 (+304)	66.86	25.00
4401	seq=translation; coord=3:202766518..202768685:-1; parent_transcript=GRMZM2G181135_T01; parent_gene=GRMZM2G181135	GRMZM2G181135_P01	TRUE	TRUE	sDDDFEVLVER	95%	n+304 (+304)	50.49	25.00
4402	seq=translation; coord=3:202766518..202768685:-1; parent_transcript=GRMZM2G181135_T01; parent_gene=GRMZM2G181135	GRMZM2G181135_P01	TRUE	TRUE	vMLANLHGEEDIFAFGHDVMR	95%	n+304 (+304)	39.10	25.00
4403	seq=translation; coord=2:189488589..189490512:-1; parent_transcript=GRMZM2G081017_T01; parent_gene=GRMZM2G081017	GRMZM2G081017_P01,GRMZM2G174799_P01, GRMZM2G174799_P02,GRMZM2G174799_P03	TRUE	TRUE	fYSLAEFk	93%	n+304 (+304), K+304 (+304)	29.02	26.41
4404	seq=translation; coord=2:189488589..189490512:-1; parent_transcript=GRMZM2G081017_T01; parent_gene=GRMZM2G081017	GRMZM2G081017_P01,GRMZM2G174799_P01, GRMZM2G174799_P02,GRMZM2G174799_P03	TRUE	TRUE	vGITVFVPk	94%	n+304 (+304), K+304 (+304)	30.84	25.00
4405	seq=translation; coord=2:189488589..189490512:-1; parent_transcript=GRMZM2G081017_T01; parent_gene=GRMZM2G081017	GRMZM2G081017_P01,GRMZM2G174799_P01, GRMZM2G174799_P02,GRMZM2G174799_P03	TRUE	TRUE	vLLPMQLFSk	95%	n+304 (+304), K+304 (+304)	35.25	25.00
4406	seq=translation; coord=8:118165588..118167724:1; parent_transcript=AC197705.4_FGT001; parent_gene=AC197705.4_FG001	AC197705.4_FGP001	TRUE	TRUE	aNLEYAVEAAADFLNk	95%	n+304 (+304), K+304 (+304)	57.05	25.28
4407	seq=translation; coord=8:118165588..118167724:1; parent_transcript=AC197705.4_FGT001; parent_gene=AC197705.4_FG001	AC197705.4_FGP001	TRUE	TRUE	eAFAAVADASGYPFAVMPAAk	95%	n+304 (+304), K+304 (+304)	54.68	25.26
4408	seq=translation; coord=8:118165588..118167724:1; parent_transcript=AC197705.4_FGT001; parent_gene=AC197705.4_FG001	AC197705.4_FGP001	TRUE	TRUE	mVVGDGPAFGcILMPEFLR	93%	n+304 (+304), Carbamidomethyl (+57)	28.39	25.00
4409	seq=translation; coord=8:87034106..87038095:-1; parent_transcript=GRMZM2G007404_T01; parent_gene=GRMZM2G007404	GRMZM2G007404_P01,GRMZM2G007404_P03	TRUE	TRUE	eGLPLMVTDFR	92%	n+304 (+304)	28.91	25.00
4410	seq=translation; coord=8:87034106..87038095:-1; parent_transcript=GRMZM2G007404_T01; parent_gene=GRMZM2G007404	GRMZM2G007404_P01,GRMZM2G007404_P03	TRUE	TRUE	gDSVIVVDNFFTGR	95%	n+304 (+304)	53.92	25.00
4411	seq=translation; coord=8:87034106..87038095:-1; parent_transcript=GRMZM2G007404_T01; parent_gene=GRMZM2G007404	GRMZM2G007404_P01,GRMZM2G007404_P03	TRUE	TRUE	sFQYVSDLVEGLMk	95%	n+304 (+304), K+304 (+304)	67.96	25.83
4412	seq=translation; coord=8:87034106..87038095:-1; parent_transcript=GRMZM2G007404_T01; parent_gene=GRMZM2G007404	GRMZM2G007404_P01,GRMZM2G007404_P03	TRUE	TRUE	vLVTGGAGFVGSHLVDR	95%	n+304 (+304)	64.33	25.60
4413	seq=translation; coord=1:262889860..262892469:1; parent_transcript=GRMZM2G024131_T01; parent_gene=GRMZM2G024131	GRMZM2G024131_P01,GRMZM2G024131_P02	TRUE	TRUE	aIGPcVPLPTAGR	91%	n+304 (+304), Carbamidomethyl (+57)	28.06	26.13

4414	seq=translation; coord=1:262889860..262892469:1;<br>parent_transcript=GRMZM2G024131_T01;<br>parent_gene=GRMZM2G024131	GRMZM2G024131_P01,GRMZM2G024131_P02	TRUE	TRUE	gLLAAGkPFLWVVR	95%	n+304 (+304), K+304 (+304)	28.09	25.00
4415	seq=translation; coord=1:262889860..262892469:1;<br>parent_transcript=GRMZM2G024131_T01;<br>parent_gene=GRMZM2G024131	GRMZM2G024131_P01,GRMZM2G024131_P02	TRUE	TRUE	nLDEFVQFVR	95%	n+304 (+304)	34.97	25.19
4416	seq=translation; coord=3:204689100..204690354:1;<br>parent_transcript=GRMZM2G425629_T02;<br>parent_gene=GRMZM2G425629	GRMZM2G425629_P02	TRUE	TRUE	dGAQQTGSYVAQTAEAAR	95%	n+304 (+304)	111.94	25.00
4417	seq=translation; coord=3:204689100..204690354:1;<br>parent_transcript=GRMZM2G425629_T02;<br>parent_gene=GRMZM2G425629	GRMZM2G425629_P02	TRUE	TRUE	dTASDAAGAAGDR	95%	n+304 (+304)	57.18	25.00
4418	seq=translation; coord=3:204689100..204690354:1;<br>parent_transcript=GRMZM2G425629_T02;<br>parent_gene=GRMZM2G425629	GRMZM2G425629_P02	TRUE	TRUE	tGALLQQAGEk	92%	n+304 (+304), K+304 (+304)	26.85	25.75
4419	seq=translation; coord=2:223290295..223311152:1;<br>parent_transcript=GRMZM2G172369_T03;<br>parent_gene=GRMZM2G172369	GRMZM2G172369_P03	TRUE	TRUE	fIYVEQAFFQR	95%	n+304 (+304)	32.77	25.00
4420	seq=translation; coord=2:223290295..223311152:1;<br>parent_transcript=GRMZM2G172369_T03;<br>parent_gene=GRMZM2G172369	GRMZM2G172369_P03	TRUE	TRUE	tLGSSADIFAGIFPk	95%	n+304 (+304), K+304 (+304)	44.22	25.75
4421	seq=translation; coord=9:151682948..151683563:1;<br>parent_transcript=GRMZM2G428040_T01;<br>parent_gene=GRMZM2G428040	GRMZM2G428040_P01	TRUE	TRUE	aSATQSADGPEEQR	95%	n+304 (+304)	47.49	25.00
4422	seq=translation; coord=9:151682948..151683563:1;<br>parent_transcript=GRMZM2G428040_T01;<br>parent_gene=GRMZM2G428040	GRMZM2G428040_P01	TRUE	TRUE	eAYGGGMYAAEEEGGR	95%	n+304 (+304)	81.50	25.00
4423	seq=translation; coord=10:87090618..87092722:1;<br>parent_transcript=GRMZM2G123029_T01;<br>parent_gene=GRMZM2G123029	GRMZM2G123029_P01,GRMZM2G123029_P02,<br>GRMZM2G123029_P03,GRMZM2G123029_P04,<br>GRMZM2G123029_P05	TRUE	TRUE	eLILQYANQLcER	95%	n+304 (+304),<br>Carbamidomethyl (+57)	50.05	25.28
4424	seq=translation; coord=10:87090618..87092722:1;<br>parent_transcript=GRMZM2G123029_T01;<br>parent_gene=GRMZM2G123029	GRMZM2G123029_P01,GRMZM2G123029_P02,<br>GRMZM2G123029_P03,GRMZM2G123029_P04,<br>GRMZM2G123029_P05	TRUE	TRUE	lPSPNGESTVScQEISk	89%	n+304 (+304),<br>Carbamidomethyl (+57),<br>K+304 (+304)	25.21	25.00
4425	seq=translation; coord=6:136910575..136913807:-1;<br>parent_transcript=GRMZM2G093574_T01;<br>parent_gene=GRMZM2G093574	GRMZM2G093574_P01,GRMZM2G093574_P02,<br>GRMZM2G125300_P01,GRMZM2G125300_P02,<br>GRMZM2G125300_P03,GRMZM2G134109_P01,<br>GRMZM2G134109_P02,GRMZM2G157300_P01,<br>GRMZM2G421795_P02,GRMZM5G831474_P01	TRUE	TRUE	tVDLYVPR	91%	n+304 (+304)	28.13	25.00
4426	seq=translation; coord=4:171375805..171380259:-1;<br>parent_transcript=GRMZM2G142661_T01;<br>parent_gene=GRMZM2G142661	GRMZM2G142661_P01,GRMZM2G356938_P01	TRUE	TRUE	aLDVIPLLTPEVVDR	95%	n+304 (+304)	62.96	25.35
4427	seq=translation; coord=4:233120026..233126298:-1;<br>parent_transcript=GRMZM2G049839_T01;<br>parent_gene=GRMZM2G049839	GRMZM2G049839_P01,GRMZM2G049839_P02	TRUE	TRUE	gIAVGVGQSEILGR	95%	n+304 (+304)	68.51	25.77
4428	seq=translation; coord=4:233120026..233126298:-1;<br>parent_transcript=GRMZM2G049839_T01;<br>parent_gene=GRMZM2G049839	GRMZM2G049839_P01,GRMZM2G049839_P02	TRUE	TRUE	gIDENWEAALEHNPEAFAR	95%	n+304 (+304)	94.76	25.00
4429	seq=translation; coord=4:233120026..233126298:-1;<br>parent_transcript=GRMZM2G049839_T01;<br>parent_gene=GRMZM2G049839	GRMZM2G049839_P01,GRMZM2G049839_P02	TRUE	TRUE	lSAAGVQDGDLVMMFSTSERPSQDGLR	95%	n+304 (+304)	35.72	25.00
4430	seq=translation; coord=4:233120026..233126298:-1;<br>parent_transcript=GRMZM2G049839_T01;<br>parent_gene=GRMZM2G049839	GRMZM2G049839_P01,GRMZM2G049839_P02	TRUE	TRUE	vNPDGTAVNPQAFQQHAR	95%	n+304 (+304)	40.08	25.00

4431	seq=translation; coord=6:141280696..141285603:-1; parent_transcript=GRMZM2G084767_T01; parent_gene=GRMZM2G084767	GRMZM2G084767_P01	TRUE	TRUE	aAVAVLGDLADTLGQSSk	95%	n+304 (+304), K+304 (+304)	80.76	25.42
4432	seq=translation; coord=6:141280696..141285603:-1; parent_transcript=GRMZM2G084767_T01; parent_gene=GRMZM2G084767	GRMZM2G084767_P01	TRUE	TRUE	aLPLLVPMLLETLLk	95%	n+304 (+304), K+304 (+304)	30.99	25.00
4433	seq=translation; coord=6:141280696..141285603:-1; parent_transcript=GRMZM2G084767_T01; parent_gene=GRMZM2G084767	GRMZM2G084767_P01	TRUE	TRUE	aLPLLVPmLLETLLk	93%	n+304 (+304), Oxidation (+16), K+304 (+304)	26.25	25.00
4434	seq=translation; coord=6:141280696..141285603:-1; parent_transcript=GRMZM2G084767_T01; parent_gene=GRMZM2G084767	GRMZM2G084767_P01	TRUE	TRUE	aQLMIPYATHLLQFTEAVFk	95%	n+304 (+304), K+304 (+304)	33.34	25.00
4435	seq=translation; coord=6:141280696..141285603:-1; parent_transcript=GRMZM2G084767_T01; parent_gene=GRMZM2G084767	GRMZM2G084767_P01	TRUE	TRUE	gIFEAYSGILQGIk	95%	n+304 (+304), K+304 (+304)	54.34	25.00
4436	seq=translation; coord=6:141280696..141285603:-1; parent_transcript=GRMZM2G084767_T01; parent_gene=GRMZM2G084767	GRMZM2G084767_P01	TRUE	TRUE	tHLFHVEFLR	95%	n+304 (+304)	35.82	26.33
4437	seq=translation; coord=6:141280696..141285603:-1; parent_transcript=GRMZM2G084767_T01; parent_gene=GRMZM2G084767	GRMZM2G084767_P01	TRUE	TRUE	vSNIPETSGIIGQLLQEIMR	95%	n+304 (+304)	76.55	25.48
4438	seq=translation; coord=3:217464401..217471380:-1; parent_transcript=GRMZM2G105019_T01; parent_gene=GRMZM2G105019	GRMZM2G105019_P01	TRUE	TRUE	aALELGSSYASGDSR	95%	n+304 (+304)	39.08	25.00
4439	seq=translation; coord=3:217464401..217471380:-1; parent_transcript=GRMZM2G105019_T01; parent_gene=GRMZM2G105019	GRMZM2G105019_P01	TRUE	TRUE	aEQVTWTTLDYSGRPk	95%	n+304 (+304), K+304 (+304)	32.06	26.60
4440	seq=translation; coord=3:217464401..217471380:-1; parent_transcript=GRMZM2G105019_T01; parent_gene=GRMZM2G105019	GRMZM2G105019_P01	TRUE	TRUE	aIELDPMMHk	95%	n+304 (+304), K+304 (+304)	29.58	25.58
4441	seq=translation; coord=3:111553578..111570930:1; parent_transcript=GRMZM2G411536_T01; parent_gene=GRMZM2G411536	GRMZM2G411536_P01,GRMZM2G411536_P03	TRUE	TRUE	fQALDMTSQPTEMITDHR	95%	n+304 (+304)	55.56	25.00
4442	seq=translation; coord=3:111553578..111570930:1; parent_transcript=GRMZM2G411536_T01; parent_gene=GRMZM2G411536	GRMZM2G411536_P01,GRMZM2G411536_P03	TRUE	TRUE	gVGADTIDPTFLEALPEDLR	95%	n+304 (+304)	47.97	25.25
4443	seq=translation; coord=3:111553578..111570930:1; parent_transcript=GRMZM2G411536_T01; parent_gene=GRMZM2G411536	GRMZM2G411536_P01,GRMZM2G411536_P03	TRUE	TRUE	hILEDPHTLQQAMELEIR	95%	n+304 (+304)	62.40	25.54
4444	seq=translation; coord=3:111553578..111570930:1; parent_transcript=GRMZM2G411536_T01; parent_gene=GRMZM2G411536	GRMZM2G411536_P01,GRMZM2G411536_P03	TRUE	TRUE	lDAIFR	95%	n+304 (+304)	31.57	25.00
4445	seq=translation; coord=3:111553578..111570930:1; parent_transcript=GRMZM2G411536_T01; parent_gene=GRMZM2G411536	GRMZM2G411536_P01,GRMZM2G411536_P03	TRUE	TRUE	lLLFLEFPcPSR	95%	n+304 (+304), Carbamidomethyl (+57)	35.82	25.72
4446	seq=translation; coord=5:167061160..167068865:-1; parent_transcript=GRMZM2G136296_T01; parent_gene=GRMZM2G136296	GRMZM2G136296_P01,GRMZM2G136296_P02	TRUE	TRUE	fYVQTVGDQR	95%	n+304 (+304)	31.99	25.00
4447	seq=translation; coord=5:167061160..167068865:-1; parent_transcript=GRMZM2G136296_T01; parent_gene=GRMZM2G136296	GRMZM2G136296_P01,GRMZM2G136296_P02	TRUE	TRUE	gEIVLSQFSLDNSWNR	95%	n+304 (+304)	59.01	25.00
4448	seq=translation; coord=5:167061160..167068865:-1; parent_transcript=GRMZM2G136296_T01; parent_gene=GRMZM2G136296	GRMZM2G136296_P01,GRMZM2G136296_P02	TRUE	TRUE	gGEQNPHLGELLR	94%	n+304 (+304)	28.47	25.00

4449	seq=translation; coord=5:167061160..167068865:-1; parent_transcript=GRMZM2G136296_T01; parent_gene=GRMZM2G136296	GRMZM2G136296_P01,GRMZM2G136296_P02	TRUE	TRUE	yLPPSAIGEASGFDAk	95%	n+304 (+304), K+304 (+304)	49.12	26.09
4450	seq=translation; coord=8:115478841..115482432:1; parent_transcript=GRMZM2G111411_T01; parent_gene=GRMZM2G111411	GRMZM2G111411_P01	TRUE	TRUE	eLVEEIAESLTk	95%	n+304 (+304), K+304 (+304)	47.30	26.44
4451	seq=translation; coord=8:115478841..115482432:1; parent_transcript=GRMZM2G111411_T01; parent_gene=GRMZM2G111411	GRMZM2G111411_P01	TRUE	TRUE	fkELVEEIAESLTk	95%	n+304 (+304), K+304 (+304), K+304 (+304)	29.69	25.00
4452	seq=translation; coord=8:115478841..115482432:1; parent_transcript=GRMZM2G111411_T01; parent_gene=GRMZM2G111411	GRMZM2G111411_P01	TRUE	TRUE	lEEVAVTTGEEDEDVLLDMk	95%	n+304 (+304), K+304 (+304)	72.65	25.00
4453	seq=translation; coord=3:1479593..1481033:-1; parent_transcript=GRMZM5G825437_T04; parent_gene=GRMZM5G825437	GRMZM5G825437_P04	TRUE	TRUE	iAEcLVGDETGVIVFTAR	95%	n+304 (+304), Carbamidomethyl (+57)	42.35	25.00
4454	seq=translation; coord=3:1479593..1481033:-1; parent_transcript=GRMZM5G825437_T04; parent_gene=GRMZM5G825437	GRMZM5G825437_P04	TRUE	TRUE	iEATEPASFTVk	95%	n+304 (+304), K+304 (+304)	31.11	25.88
4455	seq=translation; coord=3:1479593..1481033:-1; parent_transcript=GRMZM5G825437_T04; parent_gene=GRMZM5G825437	GRMZM5G825437_P04	TRUE	TRUE	nDQVDLLkPDATVILR	95%	n+304 (+304), K+304 (+304)	29.30	25.00
4456	seq=translation; coord=8:16994084..16999444:-1; parent_transcript=GRMZM2G025215_T01; parent_gene=GRMZM2G025215	GRMZM2G025215_P01	TRUE	TRUE	eVQAGVQILR	92%	n+304 (+304)	29.78	25.63
4457	seq=translation; coord=8:16994084..16999444:-1; parent_transcript=GRMZM2G025215_T01; parent_gene=GRMZM2G025215	GRMZM2G025215_P01	TRUE	TRUE	sNFPTVTIADIDR	95%	n+304 (+304)	32.33	25.00
4458	seq=translation; coord=8:16994084..16999444:-1; parent_transcript=GRMZM2G025215_T01; parent_gene=GRMZM2G025215	GRMZM2G025215_P01	TRUE	TRUE	vPLDDVVEk	87%	n+304 (+304), K+304 (+304)	26.34	25.43
4459	seq=translation; coord=4:127851768..127857102:1; parent_transcript=GRMZM2G018416_T01; parent_gene=GRMZM2G018416	GRMZM2G018416_P01	TRUE	TRUE	aPAVVAk	93%	n+304 (+304), K+304 (+304)	29.46	25.00
4460	seq=translation; coord=4:127851768..127857102:1; parent_transcript=GRMZM2G018416_T01; parent_gene=GRMZM2G018416	GRMZM2G018416_P01	TRUE	TRUE	vDGVITDFPGTAHR	95%	n+304 (+304)	43.83	25.00
4461	seq=translation; coord=4:127851768..127857102:1; parent_transcript=GRMZM2G018416_T01; parent_gene=GRMZM2G018416	GRMZM2G018416_P01	TRUE	TRUE	yNLVYMIEESVR	86%	n+304 (+304)	25.40	25.00
4462	seq=translation; coord=4:127851768..127857102:1; parent_transcript=GRMZM2G018416_T01; parent_gene=GRMZM2G018416	GRMZM2G018416_P01	TRUE	TRUE	yNLVYMIEESVR	90%	n+304 (+304), iTRAQ8plex (+304)	26.82	26.22
4463	seq=translation; coord=9:18896527..18899443:1; parent_transcript=GRMZM2G102596_T01; parent_gene=GRMZM2G102596	GRMZM2G102596_P01,GRMZM2G102596_P02, GRMZM2G102596_P03	TRUE	TRUE	aSMGGYISSQTVR	95%	n+304 (+304)	35.10	25.00
4464	seq=translation; coord=9:18896527..18899443:1; parent_transcript=GRMZM2G102596_T01; parent_gene=GRMZM2G102596	GRMZM2G102596_P01,GRMZM2G102596_P02, GRMZM2G102596_P03	TRUE	TRUE	gMGLSIGTMIAGWDEk	90%	n+304 (+304), K+304 (+304)	25.89	25.31
4465	seq=translation; coord=9:18896527..18899443:1; parent_transcript=GRMZM2G102596_T01; parent_gene=GRMZM2G102596	GRMZM2G102596_P01,GRMZM2G102596_P02, GRMZM2G102596_P03	TRUE	TRUE	gPGLYYVDSEGAR	95%	n+304 (+304)	46.04	25.00
4466	seq=translation; coord=9:18896527..18899443:1; parent_transcript=GRMZM2G102596_T01; parent_gene=GRMZM2G102596	GRMZM2G102596_P01,GRMZM2G102596_P02, GRMZM2G102596_P03	TRUE	TRUE	iIEINPYMLGTMAGGAADcQFWHR	95%	n+304 (+304), Carbamidomethyl (+57)	80.55	25.00

4467	seq=translation; coord=1:5952987..5954672:1; parent_transcript=GRMZM2G065718_T01; parent_gene=GRMZM2G065718	GRMZM2G065718_P01	TRUE	TRUE	aDVTVVAk	95%	n+304 (+304), K+304 (+304)	38.01	26.49
4468	seq=translation; coord=1:5952987..5954672:1; parent_transcript=GRMZM2G065718_T01; parent_gene=GRMZM2G065718	GRMZM2G065718_P01	TRUE	TRUE	gDDVSLDTGk	95%	n+304 (+304), K+304 (+304)	40.28	25.00
4469	seq=translation; coord=1:5952987..5954672:1; parent_transcript=GRMZM2G065718_T01; parent_gene=GRMZM2G065718	GRMZM2G065718_P01	TRUE	TRUE	gLTLFAVNDDAFQAk	95%	n+304 (+304), K+304 (+304)	55.33	25.63
4470	seq=translation; coord=1:5952987..5954672:1; parent_transcript=GRMZM2G065718_T01; parent_gene=GRMZM2G065718	GRMZM2G065718_P01	TRUE	TRUE	vVDQEDkNYGFSAATPGAR	95%	n+304 (+304), K+304 (+304)	30.17	25.50
4471	seq=translation; coord=3:133888888..133893638:1; parent_transcript=GRMZM2G116204_T01; parent_gene=GRMZM2G116204	GRMZM2G116204_P01	TRUE	TRUE	eVEVWLQTISPGQR	91%	n+304 (+304)	26.86	25.85
4472	seq=translation; coord=3:133888888..133893638:1; parent_transcript=GRMZM2G116204_T01; parent_gene=GRMZM2G116204	GRMZM2G116204_P01	TRUE	TRUE	fPFVWDEDcFEAAk	95%	n+304 (+304), Carbamidomethyl (+57), K+304 (+304)	45.89	25.00
4473	seq=translation; coord=3:133888888..133893638:1; parent_transcript=GRMZM2G116204_T01; parent_gene=GRMZM2G116204	GRMZM2G116204_P01	TRUE	TRUE	gTLLMGSSSLk	95%	n+304 (+304), K+304 (+304)	33.49	25.00
4474	seq=translation; coord=3:133888888..133893638:1; parent_transcript=GRMZM2G116204_T01; parent_gene=GRMZM2G116204	GRMZM2G116204_P01	TRUE	TRUE	iFLYDDWSMPHTAAVLk	95%	n+304 (+304), K+304 (+304)	48.44	26.17
4475	seq=translation; coord=3:9805845..9808735:-1; parent_transcript=GRMZM2G083418_T01; parent_gene=GRMZM2G083418	GRMZM2G083418_P01	TRUE	TRUE	eLFWAPAPLTTk	95%	n+304 (+304), K+304 (+304)	30.04	25.29
4476	seq=translation; coord=3:9805845..9808735:-1; parent_transcript=GRMZM2G083418_T01; parent_gene=GRMZM2G083418	GRMZM2G083418_P01	TRUE	TRUE	eTQEALDGSEETASAGPDEDTSAVDVk	95%	n+304 (+304), K+304 (+304)	59.84	25.00
4477	seq=translation; coord=3:9805845..9808735:-1; parent_transcript=GRMZM2G083418_T01; parent_gene=GRMZM2G083418	GRMZM2G083418_P01	TRUE	TRUE	lNTGNVFAALESLk	95%	n+304 (+304), K+304 (+304)	40.28	25.13
4478	seq=translation; coord=10:148849233..148855901:1; parent_transcript=GRMZM2G406074_T01; parent_gene=GRMZM2G406074	GRMZM2G406074_P01	TRUE	TRUE	hFAPYLQSDPAASS	95%	n+304 (+304)	42.23	25.00
4479	seq=translation; coord=10:148849233..148855901:1; parent_transcript=GRMZM2G406074_T01; parent_gene=GRMZM2G406074	GRMZM2G406074_P01	TRUE	TRUE	iDIPSAWQMPQGGIDAGEEPR	95%	n+304 (+304)	33.64	25.00
4480	seq=translation; coord=10:148849233..148855901:1; parent_transcript=GRMZM2G406074_T01; parent_gene=GRMZM2G406074	GRMZM2G406074_P01	TRUE	TRUE	tNVGIcLADPSLTk	95%	n+304 (+304), Carbamidomethyl (+57), K+304 (+304)	43.66	25.77
4481	seq=translation; coord=2:146194741..146200435:-1; parent_transcript=GRMZM2G537291_T01; parent_gene=GRMZM2G537291	GRMZM2G537291_P01	TRUE	TRUE	aIDQIIQIPTVLR	95%	n+304 (+304)	42.49	25.00
4482	seq=translation; coord=9:93447494..93469121:-1; parent_transcript=GRMZM2G348666_T01; parent_gene=GRMZM2G348666	GRMZM2G348666_P01,GRMZM2G348666_P03	TRUE	TRUE	fGWDcHGLPVEFEIDk	94%	n+304 (+304), Carbamidomethyl (+57), K+304 (+304)	25.95	25.00
4483	seq=translation; coord=9:93447494..93469121:-1; parent_transcript=GRMZM2G348666_T01; parent_gene=GRMZM2G348666	GRMZM2G348666_P01,GRMZM2G348666_P03	TRUE	TRUE	fHNWLEGAR	95%	n+304 (+304)	31.01	25.00
4484	seq=translation; coord=9:93447494..93469121:-1; parent_transcript=GRMZM2G348666_T01; parent_gene=GRMZM2G348666	GRMZM2G348666_P01,GRMZM2G348666_P03	TRUE	TRUE	iPGVSLVGLk	92%	n+304 (+304), K+304 (+304)	25.80	25.00

4485	seq=translation; coord=9:93447494..93469121:-1; parent_transcript=GRMZM2G348666_T01; parent_gene=GRMZM2G348666	GRMZM2G348666_P01,GRMZM2G348666_P03	TRUE	TRUE	tALSNFEAALDYR	95%	n+304 (+304)	51.60	25.00
4486	seq=translation; coord=2:8906086..8909067:-1; parent_transcript=GRMZM5G876898_T01; parent_gene=GRMZM5G876898	GRMZM5G876898_P01,GRMZM5G876898_P02	TRUE	TRUE	gAIPFLESLVVADVAALR	94%	n+304 (+304)	28.70	25.00
4487	seq=translation; coord=2:8906086..8909067:-1; parent_transcript=GRMZM5G876898_T01; parent_gene=GRMZM5G876898	GRMZM5G876898_P01,GRMZM5G876898_P02	TRUE	TRUE	sLLALQGPLAAPTLQLLTk	95%	n+304 (+304), K+304 (+304)	42.80	25.00
4488	seq=translation; coord=1:67644786..67647420:1; parent_transcript=GRMZM2G051879_T01; parent_gene=GRMZM2G051879	GRMZM2G051879_P01,GRMZM2G051879_P02, GRMZM2G051879_P03,GRMZM2G051879_P05, GRMZM2G118355_P01,GRMZM2G118355_P02, GRMZM2G118355_P03,GRMZM2G118355_P04, GRMZM2G171387_P01,GRMZM2G171387_P02, GRMZM2G176358_P01,GRMZM2G176358_P02, GRMZM2G176358_P03,GRMZM2G176358_P04	TRUE	TRUE	eIAQDFk	95%	n+304 (+304), K+304 (+304)	33.84	27.12
4489	seq=translation; coord=1:67644786..67647420:1; parent_transcript=GRMZM2G051879_T01; parent_gene=GRMZM2G051879	GRMZM2G051879_P01,GRMZM2G051879_P02, GRMZM2G051879_P03,GRMZM2G051879_P05, GRMZM2G118355_P01,GRMZM2G118355_P02, GRMZM2G118355_P03,GRMZM2G118355_P04, GRMZM2G171387_P01,GRMZM2G171387_P02, GRMZM2G176358_P01,GRMZM2G176358_P02, GRMZM2G176358_P03,GRMZM2G176358_P04	TRUE	TRUE	sTELLIR	89%	n+304 (+304)	26.66	25.00
4490	seq=translation; coord=1:68543805..68546269:-1; parent_transcript=GRMZM2G077991_T01; parent_gene=GRMZM2G077991	GRMZM2G077991_P01,GRMZM2G077991_P02, GRMZM2G077991_P03,GRMZM2G168674_P01	TRUE	TRUE	eGTEAAPk	95%	n+304 (+304), K+304 (+304)	31.43	25.00
4491	seq=translation; coord=1:68543805..68546269:-1; parent_transcript=GRMZM2G077991_T01; parent_gene=GRMZM2G077991	GRMZM2G077991_P01,GRMZM2G077991_P02, GRMZM2G077991_P03,GRMZM2G168674_P01	TRUE	TRUE	sTcSScGYPAAR	95%	n+304 (+304), Carbamidomethyl (+57), Carbamidomethyl (+57)	48.83	25.00
4492	seq=translation; coord=10:8848146..8849526:-1; parent_transcript=GRMZM2G136910_T01; parent_gene=GRMZM2G136910	GRMZM2G136910_P01	TRUE	TRUE	eAEEAGGEk	95%	n+304 (+304), K+304 (+304)	36.65	25.00
4493	seq=translation; coord=9:28003772..28008138:1; parent_transcript=GRMZM2G049693_T01; parent_gene=GRMZM2G049693	GRMZM2G049693_P01,GRMZM2G049693_P02	TRUE	TRUE	dQIGSFFYFPPLGMQR	95%	n+304 (+304)	30.63	25.00
4494	seq=translation; coord=9:28003772..28008138:1; parent_transcript=GRMZM2G049693_T01; parent_gene=GRMZM2G049693	GRMZM2G049693_P01,GRMZM2G049693_P02	TRUE	TRUE	dVYTLDFPTMPIDGPPVIR	95%	n+304 (+304)	49.55	25.00
4495	seq=translation; coord=9:28003772..28008138:1; parent_transcript=GRMZM2G049693_T01; parent_gene=GRMZM2G049693	GRMZM2G049693_P01,GRMZM2G049693_P02	TRUE	TRUE	tTLSGISYSPPDTPLR	95%	n+304 (+304)	68.12	26.13
4496	seq=translation; coord=6:132760746..132763409:1; parent_transcript=GRMZM2G160925_T01; parent_gene=GRMZM2G160925	GRMZM2G160925_P01	TRUE	TRUE	dAGVDIDAGAELVR	95%	n+304 (+304)	55.71	25.02
4497	seq=translation; coord=6:132760746..132763409:1; parent_transcript=GRMZM2G160925_T01; parent_gene=GRMZM2G160925	GRMZM2G160925_P01	TRUE	TRUE	nDGITTTVGEALMAPTFIYVk	89%	n+304 (+304), K+304 (+304)	25.95	25.89
4498	seq=translation; coord=6:132760746..132763409:1; parent_transcript=GRMZM2G160925_T01; parent_gene=GRMZM2G160925	GRMZM2G160925_P01	TRUE	TRUE	sGLSLDDQLPR	95%	n+304 (+304)	41.79	26.04

4499	seq=translation; coord=1:279565504..279568174:1; parent_transcript=GRMZM2G047456_T02; parent_gene=GRMZM2G047456	GRMZM2G047456_P02,GRMZM2G047456_P03, GRMZM2G047456_P04	TRUE	TRUE	dVIELAGGPSYAVELGR	95%	n+304 (+304)	80.36	25.21
4500	seq=translation; coord=1:279565504..279568174:1; parent_transcript=GRMZM2G047456_T02; parent_gene=GRMZM2G047456	GRMZM2G047456_P02,GRMZM2G047456_P03, GRMZM2G047456_P04	TRUE	TRUE	gLFASDQVLFSDAR	95%	n+304 (+304)	77.45	25.00
4501	seq=translation; coord=1:279565504..279568174:1; parent_transcript=GRMZM2G047456_T02; parent_gene=GRMZM2G047456	GRMZM2G047456_P02,GRMZM2G047456_P03, GRMZM2G047456_P04	TRUE	TRUE	kVQAQPVAVGATIR	91%	K+304 (+304), n+304 (+304)	25.78	25.00
4502	seq=translation; coord=8:143279539..143283510:-1; parent_transcript=GRMZM2G050270_T01; parent_gene=GRMZM2G050270	GRMZM2G050270_P01	TRUE	TRUE	aLAVDFAPDAEVR	95%	n+304 (+304)	56.91	25.00
4503	seq=translation; coord=8:143279539..143283510:-1; parent_transcript=GRMZM2G050270_T01; parent_gene=GRMZM2G050270	GRMZM2G050270_P01	TRUE	TRUE	eASLVEIVTAIR	95%	n+304 (+304)	47.68	25.00
4504	seq=translation; coord=8:143279539..143283510:-1; parent_transcript=GRMZM2G050270_T01; parent_gene=GRMZM2G050270	GRMZM2G050270_P01	TRUE	TRUE	fLDWLSGGAk	95%	n+304 (+304), K+304 (+304)	34.68	26.13
4505	seq=translation; coord=8:143279539..143283510:-1; parent_transcript=GRMZM2G050270_T01; parent_gene=GRMZM2G050270	GRMZM2G050270_P01	TRUE	TRUE	sVVEIALDSIk	95%	n+304 (+304), K+304 (+304)	38.67	25.00
4506	seq=translation; coord=5:193157657..193165368:1; parent_transcript=AC211737.3_FGT012; parent_gene=AC211737.3_FG012	AC211737.3_FGP012	TRUE	TRUE	aDADGAIVGASGAR	95%	n+304 (+304)	52.25	25.00
4507	seq=translation; coord=5:193157657..193165368:1; parent_transcript=AC211737.3_FGT012; parent_gene=AC211737.3_FG012	AC211737.3_FGP012	TRUE	TRUE	fLAAHPEYDGR	95%	n+304 (+304)	39.58	25.00
4508	seq=translation; coord=5:193157657..193165368:1; parent_transcript=AC211737.3_FGT012; parent_gene=AC211737.3_FG012	AC211737.3_FGP012	TRUE	TRUE	lIYELFTDTLTSR	90%	n+304 (+304)	25.94	25.72
4509	seq=translation; coord=5:193157657..193165368:1; parent_transcript=AC211737.3_FGT012; parent_gene=AC211737.3_FG012	AC211737.3_FGP012	TRUE	TRUE	lVINPSWk	86%	n+304 (+304), K+304 (+304)	25.22	25.00
4510	seq=translation; coord=4:237521017..237524915:-1; parent_transcript=GRMZM2G165917_T01; parent_gene=GRMZM2G165917	GRMZM2G165917_P01,GRMZM2G165917_P02, GRMZM2G165917_P03,GRMZM2G369939_P01	TRUE	TRUE	eVPVVLk	94%	n+304 (+304), K+304 (+304)	30.71	25.00
4511	seq=translation; coord=4:237521017..237524915:-1; parent_transcript=GRMZM2G165917_T01; parent_gene=GRMZM2G165917	GRMZM2G165917_P01,GRMZM2G165917_P02, GRMZM2G165917_P03,GRMZM2G369939_P01	TRUE	TRUE	lAGLLEVFIk	95%	n+304 (+304), K+304 (+304)	34.61	25.00
4512	seq=translation; coord=5:69096740..69101545:-1; parent_transcript=GRMZM2G020146_T01; parent_gene=GRMZM2G020146	GRMZM2G020146_P01	TRUE	TRUE	aALEMLR	91%	n+304 (+304)	27.88	25.00
4513	seq=translation; coord=5:69096740..69101545:-1; parent_transcript=GRMZM2G020146_T01; parent_gene=GRMZM2G020146	GRMZM2G020146_P01	TRUE	TRUE	lcYDFSNLER	95%	n+304 (+304), Carbamidomethyl (+57)	47.00	25.00
4514	seq=translation; coord=5:69096740..69101545:-1; parent_transcript=GRMZM2G020146_T01; parent_gene=GRMZM2G020146	GRMZM2G020146_P01	TRUE	TRUE	nDFFITGESyAGHYIPAFASR	93%	n+304 (+304), iTRAQ8plex (+304)	25.07	25.00
4515	seq=translation; coord=1:254659295..254663896:1; parent_transcript=GRMZM2G038848_T01; parent_gene=GRMZM2G038848	GRMZM2G038848_P01,GRMZM2G038848_P02	TRUE	TRUE	aIIISVIDNLVk	95%	n+304 (+304), K+304 (+304)	48.16	25.00
4516	seq=translation; coord=10:96835856..96839456:-1; parent_transcript=GRMZM2G021170_T01; parent_gene=GRMZM2G021170	GRMZM2G021170_P01	TRUE	TRUE	gEPTVEELAGLLDVR	95%	n+304 (+304)	44.44	25.63

4517	seq=translation; coord=10:96835856..96839456:-1; parent_transcript=GRMZM2G021170_T01; parent_gene=GRMZM2G021170	GRMZM2G021170_P01	TRUE	TRUE	hPEADTLYVEEVDVGEAEPR	95%	n+304 (+304)	36.77	25.00
4518	seq=translation; coord=6:164377053..164379535:-1; parent_transcript=GRMZM2G039886_T01; parent_gene=GRMZM2G039886	GRMZM2G039886_P01	TRUE	TRUE	aVYDQYGEEGLk	95%	n+304 (+304), K+304 (+304)	51.82	25.00
4519	seq=translation; coord=6:164377053..164379535:-1; parent_transcript=GRMZM2G039886_T01; parent_gene=GRMZM2G039886	GRMZM2G039886_P01	TRUE	TRUE	iPLAEALTGYTAHVTTLDGR	95%	n+304 (+304)	57.85	25.59
4520	seq=translation; coord=6:164377053..164379535:-1; parent_transcript=GRMZM2G039886_T01; parent_gene=GRMZM2G039886	GRMZM2G039886_P01	TRUE	TRUE	iPLAEALTGyTAHVTTLDGR	95%	n+304 (+304), iTRAQ8plex (+304)	26.79	25.17
4521	seq=translation; coord=6:164377053..164379535:-1; parent_transcript=GRMZM2G039886_T01; parent_gene=GRMZM2G039886	GRMZM2G039886_P01	TRUE	TRUE	qISEAYEVLSDPQk	95%	n+304 (+304), K+304 (+304)	59.27	26.63
4522	seq=translation; coord=6:164377053..164379535:-1; parent_transcript=GRMZM2G039886_T01; parent_gene=GRMZM2G039886	GRMZM2G039886_P01	TRUE	TRUE	sLTVPISSVIHPGYEEVVR	95%	n+304 (+304)	49.06	25.30
4523	seq=translation; coord=6:153256043..153259238:1; parent_transcript=GRMZM2G169943_T01; parent_gene=GRMZM2G169943	GRMZM2G169943_P01	TRUE	TRUE	dLQLDYLDLYLIHWPFR	95%	n+304 (+304)	55.68	25.00
4524	seq=translation; coord=6:153256043..153259238:1; parent_transcript=GRMZM2G169943_T01; parent_gene=GRMZM2G169943	GRMZM2G169943_P01	TRUE	TRUE	dLQLDYLDLyLIHWPFR	95%	n+304 (+304), iTRAQ8plex (+304)	32.72	25.05
4525	seq=translation; coord=6:153256043..153259238:1; parent_transcript=GRMZM2G169943_T01; parent_gene=GRMZM2G169943	GRMZM2G169943_P01	TRUE	TRUE	eMEGLVk	95%	n+304 (+304), K+304 (+304)	31.58	25.85
4526	seq=translation; coord=6:153256043..153259238:1; parent_transcript=GRMZM2G169943_T01; parent_gene=GRMZM2G169943	GRMZM2G169943_P01	TRUE	TRUE	sATEVWDHED	95%	n+304 (+304)	30.50	25.00
4527	seq=translation; coord=6:153256043..153259238:1; parent_transcript=GRMZM2G169943_T01; parent_gene=GRMZM2G169943	GRMZM2G169943_P01	TRUE	TRUE	vLTGEELFVNk	95%	n+304 (+304), K+304 (+304)	47.96	26.39
4528	seq=translation; coord=6:156534704..156535855:-1; parent_transcript=GRMZM2G410134_T01; parent_gene=GRMZM2G410134	GRMZM2G410134_P01	TRUE	TRUE	dLQcWQEVQESPLDAcR	95%	n+304 (+304), Carbamidomethyl (+57), Carbamidomethyl (+57)	69.91	25.00
4529	seq=translation; coord=6:156534704..156535855:-1; parent_transcript=GRMZM2G410134_T01; parent_gene=GRMZM2G410134	GRMZM2G410134_P01	TRUE	TRUE	eYAAGLPMMcR	95%	n+304 (+304), Carbamidomethyl (+57)	35.65	25.00
4530	seq=translation; coord=6:156534704..156535855:-1; parent_transcript=GRMZM2G410134_T01; parent_gene=GRMZM2G410134	GRMZM2G410134_P01	TRUE	TRUE	gYEEAMPPLEk	95%	n+304 (+304), K+304 (+304)	36.67	25.30
4531	seq=translation; coord=6:156534704..156535855:-1; parent_transcript=GRMZM2G410134_T01; parent_gene=GRMZM2G410134	GRMZM2G410134_P01	TRUE	TRUE	lSEPQEcSIFSGGDQy	95%	n+304 (+304), Carbamidomethyl (+57), iTRAQ8plex (+304)	59.69	25.00
4532	seq=translation; coord=6:157582700..157589894:1; parent_transcript=GRMZM2G006377_T01; parent_gene=GRMZM2G006377	GRMZM2G006377_P01	TRUE	TRUE	dQGScGAcWSFSATGAMEGINk	95%	n+304 (+304), Carbamidomethyl (+57), Carbamidomethyl (+57), K+304 (+304)	35.46	25.00
4533	seq=translation; coord=6:157582700..157589894:1; parent_transcript=GRMZM2G006377_T01; parent_gene=GRMZM2G006377	GRMZM2G006377_P01	TRUE	TRUE	tGSLVSLSEQELIDcDR	95%	n+304 (+304), Carbamidomethyl (+57)	89.34	25.00

4534	seq=translation; coord=6:63919594..63950390:1; parent_transcript=GRMZM2G052812_T02; parent_gene=GRMZM2G052812	GRMZM2G052812_P02,GRMZM2G052812_P05	TRUE	TRUE	lVDSLSEQDFR	87%	n+304 (+304)	25.75	25.00
4535	seq=translation; coord=6:63919594..63950390:1; parent_transcript=GRMZM2G052812_T02; parent_gene=GRMZM2G052812	GRMZM2G052812_P02,GRMZM2G052812_P05	TRUE	TRUE	yIGLSEASASTIR	95%	n+304 (+304)	40.13	25.56
4536	seq=translation; coord=1:41468889..41473581:1; parent_transcript=GRMZM2G044027_T01; parent_gene=GRMZM2G044027	GRMZM2G044027_P01,GRMZM2G044027_P02	TRUE	TRUE	eLINPDITVTMTENTPDDPR	94%	n+304 (+304)	30.48	25.00
4537	seq=translation; coord=1:41468889..41473581:1; parent_transcript=GRMZM2G044027_T01; parent_gene=GRMZM2G044027	GRMZM2G044027_P01,GRMZM2G044027_P02	TRUE	TRUE	sFcYVADMVDGLIR	95%	n+304 (+304), Carbamidomethyl (+57)	33.52	25.00
4538	seq=translation; coord=8:17872894..17877539:1; parent_transcript=GRMZM2G080722_T01; parent_gene=GRMZM2G080722	GRMZM2G080722_P01	TRUE	TRUE	aYSNWPTFPQIFIk	90%	n+304 (+304), K+304 (+304)	25.37	25.00
4539	seq=translation; coord=8:17872894..17877539:1; parent_transcript=GRMZM2G080722_T01; parent_gene=GRMZM2G080722	GRMZM2G080722_P01	TRUE	TRUE	eLLGDIAQk	95%	n+304 (+304), K+304 (+304)	36.97	26.90
4540	seq=translation; coord=8:17872894..17877539:1; parent_transcript=GRMZM2G080722_T01; parent_gene=GRMZM2G080722	GRMZM2G080722_P01	TRUE	TRUE	gEFVGGSDIILTMHQk	95%	n+304 (+304), K+304 (+304)	43.21	26.17
4541	seq=translation; coord=8:17872894..17877539:1; parent_transcript=GRMZM2G080722_T01; parent_gene=GRMZM2G080722	GRMZM2G080722_P01	TRUE	TRUE	sSDMSFSDIVAQDVk	95%	n+304 (+304), K+304 (+304)	30.99	25.00
4542	seq=translation; coord=1:290521787..290523917:1; parent_transcript=GRMZM2G012224_T01; parent_gene=GRMZM2G012224	GRMZM2G012224_P01	TRUE	TRUE	dATSPLPGWIIESLk	95%	n+304 (+304), K+304 (+304)	31.93	25.00
4543	seq=translation; coord=1:290521787..290523917:1; parent_transcript=GRMZM2G012224_T01; parent_gene=GRMZM2G012224	GRMZM2G012224_P01	TRUE	TRUE	eSAIAQVIR	95%	n+304 (+304)	33.45	26.51
4544	seq=translation; coord=1:290521787..290523917:1; parent_transcript=GRMZM2G012224_T01; parent_gene=GRMZM2G012224	GRMZM2G012224_P01	TRUE	TRUE	nDGFEIGPQVLR	93%	n+304 (+304)	29.01	25.56
4545	seq=translation; coord=1:290521787..290523917:1; parent_transcript=GRMZM2G012224_T01; parent_gene=GRMZM2G012224	GRMZM2G012224_P01	TRUE	TRUE	vAVAPSLSAPADVAVR	95%	n+304 (+304)	73.82	25.99
4546	seq=translation; coord=1:290521787..290523917:1; parent_transcript=GRMZM2G012224_T01; parent_gene=GRMZM2G012224	GRMZM2G012224_P01	TRUE	TRUE	vTDGVSLcGPVLDQVk	95%	n+304 (+304), Carbamidomethyl (+57), K+304 (+304)	47.67	26.41
4547	seq=translation; coord=6:148590842..148593721:1; parent_transcript=GRMZM2G033641_T01; parent_gene=GRMZM2G033641	GRMZM2G033641_P01	TRUE	TRUE	aAEAEAGAEEk	95%	n+304 (+304), K+304 (+304)	65.15	25.00
4548	seq=translation; coord=6:148590842..148593721:1; parent_transcript=GRMZM2G033641_T01; parent_gene=GRMZM2G033641	GRMZM2G033641_P01	TRUE	TRUE	eAMAMLk	91%	n+304 (+304), K+304 (+304)	30.40	27.04
4549	seq=translation; coord=6:148590842..148593721:1; parent_transcript=GRMZM2G033641_T01; parent_gene=GRMZM2G033641	GRMZM2G033641_P01	TRUE	TRUE	eELIWGVPLAGDDER	95%	n+304 (+304)	54.73	25.00
4550	seq=translation; coord=6:148590842..148593721:1; parent_transcript=GRMZM2G033641_T01; parent_gene=GRMZM2G033641	GRMZM2G033641_P01	TRUE	TRUE	qEAAAPAAEVVVTEEEk	93%	n+304 (+304), K+304 (+304)	28.29	25.62
4551	seq=translation; coord=6:168917945..168919116:1; parent_transcript=GRMZM5G833747_T01; parent_gene=GRMZM5G833747	GRMZM5G833747_P01,GRMZM5G833747_P02, GRMZM5G833747_P03	TRUE	TRUE	gDLGVVVGR	95%	n+304 (+304)	34.97	27.40

4552	seq=translation; coord=6:168917945..168919116:1; parent_transcript=GRMZM5G833747_T01; parent_gene=GRMZM5G833747	GRMZM5G833747_P01,GRMZM5G833747_P02, GRMZM5G833747_P03	TRUE	TRUE	tFIAIkPDGVQR	95%	n+304 (+304), K+304 (+304)	31.17	25.00
4553	seq=translation; coord=1:41535916..41540223:-1; parent_transcript=GRMZM2G157329_T01; parent_gene=GRMZM2G157329	GRMZM2G157329_P01,GRMZM2G157329_P02	TRUE	TRUE	aPDAAWQHDMYPAVAAGGGGGGR	91%	n+304 (+304)	25.38	25.00
4554	seq=translation; coord=1:41535916..41540223:-1; parent_transcript=GRMZM2G157329_T01; parent_gene=GRMZM2G157329	GRMZM2G157329_P01,GRMZM2G157329_P02	TRUE	TRUE	eLFSELGDLk	95%	n+304 (+304), K+304 (+304)	40.31	26.52
4555	seq=translation; coord=1:41535916..41540223:-1; parent_transcript=GRMZM2G157329_T01; parent_gene=GRMZM2G157329	GRMZM2G157329_P01,GRMZM2G157329_P02	TRUE	TRUE	gTAEVVFAR	90%	n+304 (+304)	27.41	25.00
4556	seq=translation; coord=1:41535916..41540223:-1; parent_transcript=GRMZM2G157329_T01; parent_gene=GRMZM2G157329	GRMZM2G157329_P01,GRMZM2G157329_P02	TRUE	TRUE	lYISNLDFGVSNDDIk	95%	n+304 (+304), K+304 (+304)	41.86	26.06
4557	seq=translation; coord=1:41535916..41540223:-1; parent_transcript=GRMZM2G157329_T01; parent_gene=GRMZM2G157329	GRMZM2G157329_P01,GRMZM2G157329_P02	TRUE	TRUE	sAEELDADLEk	95%	n+304 (+304), K+304 (+304)	69.75	25.00
4558	seq=translation; coord=5:19123690..19126862:1; parent_transcript=GRMZM2G074898_T01; parent_gene=GRMZM2G074898	GRMZM2G074898_P01,GRMZM2G074898_P02, GRMZM2G074898_P03,GRMZM2G110328_P01, GRMZM2G110328_P02,GRMZM2G110328_P03, GRMZM2G142640_P01,GRMZM2G142640_P02, GRMZM2G142640_P03	TRUE	TRUE	aDSQVFLFANSk	94%	n+304 (+304), K+304 (+304)	29.33	26.02
4559	seq=translation; coord=5:19123690..19126862:1; parent_transcript=GRMZM2G074898_T01; parent_gene=GRMZM2G074898	GRMZM2G074898_P01,GRMZM2G074898_P02, GRMZM2G074898_P03,GRMZM2G110328_P01, GRMZM2G110328_P02,GRMZM2G110328_P03, GRMZM2G142640_P01,GRMZM2G142640_P02, GRMZM2G142640_P03	TRUE	TRUE	sIVGASLEVIQk	95%	n+304 (+304), K+304 (+304)	50.98	25.00
4560	seq=translation; coord=2:38192410..38194128:1; parent_transcript=GRMZM2G121700_T01; parent_gene=GRMZM2G121700	GRMZM2G121700_P01	TRUE	TRUE	aLFDLPDDAk	95%	n+304 (+304), K+304 (+304)	38.31	25.59
4561	seq=translation; coord=2:38192410..38194128:1; parent_transcript=GRMZM2G121700_T01; parent_gene=GRMZM2G121700	GRMZM2G121700_P01	TRUE	TRUE	iSIAMFLLAPk	95%	n+304 (+304), K+304 (+304)	32.73	25.00
4562	seq=translation; coord=2:38192410..38194128:1; parent_transcript=GRMZM2G121700_T01; parent_gene=GRMZM2G121700	GRMZM2G121700_P01	TRUE	TRUE	lAGSSPDESAR	95%	n+304 (+304)	43.96	25.00
4563	seq=translation; coord=2:17348097..17355999:-1; parent_transcript=GRMZM2G145968_T01; parent_gene=GRMZM2G145968	GRMZM2G145968_P01	TRUE	TRUE	eGDEAEGSNk	95%	n+304 (+304), K+304 (+304)	31.96	25.00
4564	seq=translation; coord=2:17348097..17355999:-1; parent_transcript=GRMZM2G145968_T01; parent_gene=GRMZM2G145968	GRMZM2G145968_P01	TRUE	TRUE	gLTPFFAFLAEFRPQYLEk	94%	n+304 (+304), K+304 (+304)	26.28	25.00
4565	seq=translation; coord=2:17348097..17355999:-1; parent_transcript=GRMZM2G145968_T01; parent_gene=GRMZM2G145968	GRMZM2G145968_P01	TRUE	TRUE	skSEVEDDEQDGNEDEDE	95%	n+304 (+304), K+304 (+304)	86.75	25.00
4566	seq=translation; coord=2:17348097..17355999:-1; parent_transcript=GRMZM2G145968_T01; parent_gene=GRMZM2G145968	GRMZM2G145968_P01	TRUE	TRUE	sMSDEEk	95%	n+304 (+304), K+304 (+304)	29.40	25.00
4567	seq=translation; coord=5:180669231..180673418:1; parent_transcript=GRMZM2G139614_T01; parent_gene=GRMZM2G139614	GRMZM2G139614_P01	TRUE	TRUE	dASATPTPPAEAGR	95%	n+304 (+304)	31.78	25.00

4568	seq=translation; coord=5:180669231..180673418:1; parent_transcript=GRMZM2G139614_T01; parent_gene=GRMZM2G139614	GRMZM2G139614_P01	TRUE	TRUE	iTQMEEQLELLR	95%	n+304 (+304)	40.20	25.85
4569	seq=translation; coord=7:10575103..10576227:-1; parent_transcript=GRMZM2G328171_T01; parent_gene=GRMZM2G328171	GRMZM2G328171_P01	TRUE	TRUE	rPFGDAYVDGLDFFLDR	95%	n+304 (+304)	47.79	25.00
4570	seq=translation; coord=7:10575103..10576227:-1; parent_transcript=GRMZM2G328171_T01; parent_gene=GRMZM2G328171	GRMZM2G328171_P01	TRUE	TRUE	tPVQLSATPR	90%	n+304 (+304)	29.18	26.99
4571	seq=translation; coord=4:199076122..199077395:-1; parent_transcript=GRMZM2G177720_T01; parent_gene=GRMZM2G177720	GRMZM2G177720_P01	TRUE	TRUE	dIEGWTAR	90%	n+304 (+304)	27.60	25.00
4572	seq=translation; coord=4:199076122..199077395:-1; parent_transcript=GRMZM2G177720_T01; parent_gene=GRMZM2G177720	GRMZM2G177720_P01	TRUE	TRUE	qFGYIVLTTSAGIMDHEEAR	95%	n+304 (+304)	71.01	25.00
4573	seq=translation; coord=4:199076122..199077395:-1; parent_transcript=GRMZM2G177720_T01; parent_gene=GRMZM2G177720	GRMZM2G177720_P01	TRUE	TRUE	qFGYIVLTTSAGIMDHEEAR	95%	Pyro-cmC (-17), n+304 (+304)	133.31	25.00
4574	seq=translation; coord=4:199076122..199077395:-1; parent_transcript=GRMZM2G177720_T01; parent_gene=GRMZM2G177720	GRMZM2G177720_P01	TRUE	TRUE	sMYNAEk	92%	n+304 (+304), K+304 (+304)	25.91	25.00
4575	seq=translation; coord=4:199076122..199077395:-1; parent_transcript=GRMZM2G177720_T01; parent_gene=GRMZM2G177720	GRMZM2G177720_P01	TRUE	TRUE	vSVLNDALk	95%	n+304 (+304), K+304 (+304)	31.53	25.29
4576	seq=translation; coord=2:24608141..24615659:1; parent_transcript=GRMZM2G061928_T01; parent_gene=GRMZM2G061928	GRMZM2G061928_P01,GRMZM2G061928_P02, GRMZM2G061928_P03	TRUE	TRUE	aIGLMPMIDQGEk	95%	n+304 (+304), K+304 (+304)	30.01	25.80
4577	seq=translation; coord=1:46126597..46129448:1; parent_transcript=GRMZM2G030016_T01; parent_gene=GRMZM2G030016	GRMZM2G030016_P01,GRMZM2G030016_P02	TRUE	TRUE	dLYINNAIQMDVTGSR	95%	n+304 (+304)	86.34	25.00
4578	seq=translation; coord=1:46126597..46129448:1; parent_transcript=GRMZM2G030016_T01; parent_gene=GRMZM2G030016	GRMZM2G030016_P01,GRMZM2G030016_P02	TRUE	TRUE	tLTAVHDGILEDVVYPAEIVGk	95%	n+304 (+304), K+304 (+304)	61.76	25.00
4579	seq=translation; coord=4:195054129..195060931:1; parent_transcript=GRMZM2G084406_T02; parent_gene=GRMZM2G084406	GRMZM2G084406_P02	TRUE	TRUE	aRPPQEAIDTFVSITGADEVTAVR	95%	n+304 (+304)	44.32	25.99
4580	seq=translation; coord=4:195054129..195060931:1; parent_transcript=GRMZM2G084406_T02; parent_gene=GRMZM2G084406	GRMZM2G084406_P02	TRUE	TRUE	ePLQNDEGAVTVVVR	95%	n+304 (+304)	56.55	25.01
4581	seq=translation; coord=4:195054129..195060931:1; parent_transcript=GRMZM2G084406_T02; parent_gene=GRMZM2G084406	GRMZM2G084406_P02	TRUE	TRUE	lQYLFDFIDISR	95%	n+304 (+304)	35.82	25.55
4582	seq=translation; coord=4:195054129..195060931:1; parent_transcript=GRMZM2G084406_T02; parent_gene=GRMZM2G084406	GRMZM2G084406_P02	TRUE	TRUE	lQyLFDFIDISR	95%	n+304 (+304), iTRAQ8plex (+304)	28.80	25.29
4583	seq=translation; coord=4:195054129..195060931:1; parent_transcript=GRMZM2G084406_T02; parent_gene=GRMZM2G084406	GRMZM2G084406_P02	TRUE	TRUE	tIEPAETVQR	90%	n+304 (+304)	27.62	26.23
4584	seq=translation; coord=6:98313412..98316578:1; parent_transcript=GRMZM2G110402_T01; parent_gene=GRMZM2G110402	GRMZM2G110402_P01,GRMZM2G110402_P02, GRMZM2G110402_P03	TRUE	TRUE	iSVDASLAGAIIGk	95%	n+304 (+304), K+304 (+304)	38.95	25.00
4585	seq=translation; coord=6:98313412..98316578:1; parent_transcript=GRMZM2G110402_T01; parent_gene=GRMZM2G110402	GRMZM2G110402_P01,GRMZM2G110402_P02, GRMZM2G110402_P03	TRUE	TRUE	mPMGPGGPDGPPSPAVk	95%	n+304 (+304), K+304 (+304)	39.05	25.65

4586	seq=translation; coord=6:98313412..98316578:1; parent_transcript=GRMZM2G110402_T01; parent_gene=GRMZM2G110402	GRMZM2G110402_P01,GRMZM2G110402_P02, GRMZM2G110402_P03	TRUE	TRUE	mTNLGGPPVPAPPGR	95%	n+304 (+304)	47.59	26.38
4587	seq=translation; coord=6:98313412..98316578:1; parent_transcript=GRMZM2G110402_T01; parent_gene=GRMZM2G110402	GRMZM2G110402_P01,GRMZM2G110402_P02, GRMZM2G110402_P03	TRUE	TRUE	nIELEGTFDQIk	95%	n+304 (+304), K+304 (+304)	47.48	26.09
4588	seq=translation; coord=3:151293748..151299116:1; parent_transcript=GRMZM2G030902_T01; parent_gene=GRMZM2G030902	GRMZM2G030902_P01,GRMZM2G030902_P02, GRMZM2G030902_P03,GRMZM2G060977_P01, GRMZM2G060977_P02,GRMZM2G060977_P03, GRMZM2G060977_P04,GRMZM2G060977_P05	TRUE	TRUE	iPQSVDESMFR	95%	n+304 (+304)	36.66	25.00
4589	seq=translation; coord=3:151293748..151299116:1; parent_transcript=GRMZM2G030902_T01; parent_gene=GRMZM2G030902	GRMZM2G030902_P01,GRMZM2G030902_P02, GRMZM2G030902_P03,GRMZM2G060977_P01, GRMZM2G060977_P02,GRMZM2G060977_P03, GRMZM2G060977_P04,GRMZM2G060977_P05	TRUE	TRUE	lVVLGIPWDVDTEGLR	95%	n+304 (+304)	36.67	26.57
4590	seq=translation; coord=1:293644603..293647117:-1; parent_transcript=GRMZM2G068244_T01; parent_gene=GRMZM2G068244	GRMZM2G068244_P01	TRUE	TRUE	iGAAGDVYVADIR	95%	n+304 (+304)	31.38	25.50
4591	seq=translation; coord=1:293644603..293647117:-1; parent_transcript=GRMZM2G068244_T01; parent_gene=GRMZM2G068244	GRMZM2G068244_P01	TRUE	TRUE	lAPAVQGVDALIILTSAAPk	95%	n+304 (+304), K+304 (+304)	61.78	25.00
4592	seq=translation; coord=10:103630422..103635991:-1; parent_transcript=GRMZM2G359298_T01; parent_gene=GRMZM2G359298	GRMZM2G359298_P01	TRUE	TRUE	gEDTLDTWSER	95%	n+304 (+304)	34.30	25.00
4593	seq=translation; coord=10:103630422..103635991:-1; parent_transcript=GRMZM2G359298_T01; parent_gene=GRMZM2G359298	GRMZM2G359298_P01	TRUE	TRUE	lDMDVDGADNSFVR	95%	n+304 (+304)	56.22	25.00
4594	seq=translation; coord=8:171328319..171331663:-1; parent_transcript=GRMZM2G172932_T01; parent_gene=GRMZM2G172932	GRMZM2G172932_P01,GRMZM2G172932_P02, GRMZM2G172932_P03	TRUE	TRUE	gLSMASTFVGNSTSIQEMFR	95%	n+304 (+304)	31.32	25.00
4595	seq=translation; coord=8:171328319..171331663:-1; parent_transcript=GRMZM2G172932_T01; parent_gene=GRMZM2G172932	GRMZM2G172932_P01,GRMZM2G172932_P02, GRMZM2G172932_P03	TRUE	TRUE	yTGTSDLQLER	95%	n+304 (+304)	48.82	25.00
4596	seq=translation; coord=1:16603599..16606666:-1; parent_transcript=GRMZM2G016511_T01; parent_gene=GRMZM2G016511	GRMZM2G016511_P01	TRUE	TRUE	aLTTEEIDQHLTAISERD	95%	n+304 (+304)	55.25	25.00
4597	seq=translation; coord=5:212248891..212249987:1; parent_transcript=GRMZM2G098167_T01; parent_gene=GRMZM2G098167	GRMZM2G098167_P01	TRUE	TRUE	eYAFVLDVPGLSk	95%	n+304 (+304), K+304 (+304)	40.64	25.26
4598	seq=translation; coord=5:212248891..212249987:1; parent_transcript=GRMZM2G098167_T01; parent_gene=GRMZM2G098167	GRMZM2G098167_P01	TRUE	TRUE	lPEDADTGGVAAR	93%	n+304 (+304)	29.33	25.00
4599	seq=translation; coord=5:212248891..212249987:1; parent_transcript=GRMZM2G098167_T01; parent_gene=GRMZM2G098167	GRMZM2G098167_P01	TRUE	TRUE	sDIQVTLEEDR	95%	n+304 (+304)	55.98	25.00
4600	seq=translation; coord=7:7118087..7119389:-1; parent_transcript=GRMZM2G320298_T01; parent_gene=GRMZM2G320298	GRMZM2G320298_P01	TRUE	TRUE	fGGSPADLAPVAVR	95%	n+304 (+304)	45.87	26.05
4601	seq=translation; coord=7:7118087..7119389:-1; parent_transcript=GRMZM2G320298_T01; parent_gene=GRMZM2G320298	GRMZM2G320298_P01	TRUE	TRUE	lPAAIDDGAAAVLWLAR	89%	n+304 (+304)	26.03	25.82

4602	seq=translation; coord=6:147724800..147731064:1; parent_transcript=GRMZM2G070199_T01; parent_gene=GRMZM2G070199	GRMZM2G070199_P01	TRUE	TRUE	aDLIAYLk	93%	n+304 (+304), K+304 (+304)	28.08	25.31
4603	seq=translation; coord=6:147724800..147731064:1; parent_transcript=GRMZM2G070199_T01; parent_gene=GRMZM2G070199	GRMZM2G070199_P01	TRUE	TRUE	qSGTTAGYSYSAGNk	95%	n+304 (+304), K+304 (+304)	70.48	25.00
4604	seq=translation; coord=4:7172367..7173014:-1; parent_transcript=GRMZM2G132162_T01; parent_gene=GRMZM2G132162	GRMZM2G132162_P01	TRUE	TRUE	vLVSSNcETPTR	95%	n+304 (+304), Carbamidomethyl (+57)	42.69	25.00
4605	seq=translation; coord=4:7172367..7173014:-1; parent_transcript=GRMZM2G132162_T01; parent_gene=GRMZM2G132162	GRMZM2G132162_P01	TRUE	TRUE	vSIAcDTGcNAAISR	95%	n+304 (+304), Carbamidomethyl (+57), Carbamidomethyl (+57)	54.63	25.00
4606	seq=translation; coord=7:152391333..152401606:1; parent_transcript=GRMZM2G325008_T01; parent_gene=GRMZM2G325008	GRMZM2G325008_P01,GRMZM2G325008_P02, GRMZM2G325008_P03,GRMZM2G325008_P04, GRMZM2G325008_P05	TRUE	TRUE	lYDADPR	95%	n+304 (+304)	37.07	25.00
4607	seq=translation; coord=7:152391333..152401606:1; parent_transcript=GRMZM2G325008_T01; parent_gene=GRMZM2G325008	GRMZM2G325008_P01,GRMZM2G325008_P02, GRMZM2G325008_P03,GRMZM2G325008_P04, GRMZM2G325008_P05	TRUE	TRUE	yTNVFDAMLDAVR	95%	n+304 (+304)	38.51	25.00
4608	seq=translation; coord=5:31178435..31182856:-1; parent_transcript=GRMZM2G102639_T01; parent_gene=GRMZM2G102639	GRMZM2G102639_P01,GRMZM2G170699_P01	TRUE	TRUE	lLYDTFSAFGVIVTNPk	95%	n+304 (+304), K+304 (+304)	51.86	25.51
4609	seq=translation; coord=5:31178435..31182856:-1; parent_transcript=GRMZM2G102639_T01; parent_gene=GRMZM2G102639	GRMZM2G102639_P01,GRMZM2G170699_P01	TRUE	TRUE	vTNLHQGYGFVEFR	94%	n+304 (+304)	28.56	25.00
4610	seq=translation; coord=3:221273481..221276486:-1; parent_transcript=GRMZM2G051764_T01; parent_gene=GRMZM2G051764	GRMZM2G051764_P01,GRMZM2G051764_P03, GRMZM2G051764_P04	TRUE	TRUE	dGLTAADHLGEDFYk	93%	n+304 (+304), K+304 (+304)	27.47	25.00
4611	seq=translation; coord=3:221273481..221276486:-1; parent_transcript=GRMZM2G051764_T01; parent_gene=GRMZM2G051764	GRMZM2G051764_P01,GRMZM2G051764_P03, GRMZM2G051764_P04	TRUE	TRUE	vIEIPGDTSVIDAVR	95%	n+304 (+304)	40.57	25.94
4612	seq=translation; coord=1:14103040..14104304:1; parent_transcript=GRMZM5G850455_T01; parent_gene=GRMZM5G850455	GRMZM5G850455_P01,GRMZM5G850455_P02, GRMZM5G850455_P03	TRUE	TRUE	aPPPDccGGLR	91%	n+304 (+304), Carbamidomethyl (+57), Carbamidomethyl (+57)	28.13	25.00
4613	seq=translation; coord=1:14103040..14104304:1; parent_transcript=GRMZM5G850455_T01; parent_gene=GRMZM5G850455	GRMZM5G850455_P01,GRMZM5G850455_P02, GRMZM5G850455_P03	TRUE	TRUE	tEcADQLVGLAPcLQYVQGQAR	95%	n+304 (+304), Carbamidomethyl (+57), Carbamidomethyl (+57)	59.86	25.00
4614	seq=translation; coord=7:24151509..24152227:1; parent_transcript=GRMZM2G046532_T01; parent_gene=GRMZM2G046532	GRMZM2G046532_P01	TRUE	TRUE	dANcANVcLTEGFTGGVck	95%	n+304 (+304), Carbamidomethyl (+57), Carbamidomethyl (+57), Carbamidomethyl (+57), K+304 (+304)	72.20	25.00
4615	seq=translation; coord=2:5269563..5273858:1; parent_transcript=GRMZM2G166281_T01; parent_gene=GRMZM2G166281	GRMZM2G166281_P01	TRUE	TRUE	vVTIDSYEDVPANDEk	95%	n+304 (+304), K+304 (+304)	45.96	25.00
4616	seq=translation; coord=7:10513154..10517482:1; parent_transcript=GRMZM2G066290_T01; parent_gene=GRMZM2G066290	GRMZM2G066290_P01,GRMZM2G066290_P02	TRUE	TRUE	aEDGEVWTFSVR	95%	n+304 (+304)	29.97	25.00
4617	seq=translation; coord=7:10513154..10517482:1; parent_transcript=GRMZM2G066290_T01; parent_gene=GRMZM2G066290	GRMZM2G066290_P01,GRMZM2G066290_P02	TRUE	TRUE	aEVADVSEAVR	95%	n+304 (+304)	31.62	25.00
4618	seq=translation; coord=7:10513154..10517482:1; parent_transcript=GRMZM2G066290_T01; parent_gene=GRMZM2G066290	GRMZM2G066290_P01,GRMZM2G066290_P02	TRUE	TRUE	gDMGAQVPLEQVPSIQQk	92%	n+304 (+304), K+304 (+304)	26.49	25.61

4619	seq=translation; coord=7:10513154..10517482:1; parent_transcript=GRMZM2G066290_T01; parent_gene=GRMZM2G066290	GRMZM2G066290_P01,GRMZM2G066290_P02	TRUE	TRUE	lNLQWGLIPFR	95%	n+304 (+304)	53.23	25.35
4620	seq=translation; coord=7:10513154..10517482:1; parent_transcript=GRMZM2G066290_T01; parent_gene=GRMZM2G066290	GRMZM2G066290_P01,GRMZM2G066290_P02	TRUE	TRUE	qLNkPVIVASQLLESMIEYPTPTR	95%	n+304 (+304), K+304 (+304)	57.58	25.00
4621	seq=translation; coord=7:10513154..10517482:1; parent_transcript=GRMZM2G066290_T01; parent_gene=GRMZM2G066290	GRMZM2G066290_P01,GRMZM2G066290_P02	TRUE	TRUE	rLNLQWGLIPFR	95%	n+304 (+304)	37.72	25.00
4622	seq=translation; coord=7:10513154..10517482:1; parent_transcript=GRMZM2G066290_T01; parent_gene=GRMZM2G066290	GRMZM2G066290_P01,GRMZM2G066290_P02	TRUE	TRUE	tINVNYDGFAEDVR	95%	n+304 (+304)	40.20	25.00
4623	seq=translation; coord=2:36456199..36464613:-1; parent_transcript=GRMZM2G130230_T01; parent_gene=GRMZM2G130230	GRMZM2G130230_P01,GRMZM2G130230_P02, GRMZM2G130230_P03	TRUE	TRUE	dLDSAEELSAQLGELFEEHQLYR	95%	n+304 (+304)	50.52	25.00
4624	seq=translation; coord=2:36456199..36464613:-1; parent_transcript=GRMZM2G130230_T01; parent_gene=GRMZM2G130230	GRMZM2G130230_P01,GRMZM2G130230_P02, GRMZM2G130230_P03	TRUE	TRUE	dNIDNIQIVFR	92%	n+304 (+304)	29.49	25.77
4625	seq=translation; coord=2:36456199..36464613:-1; parent_transcript=GRMZM2G130230_T01; parent_gene=GRMZM2G130230	GRMZM2G130230_P01,GRMZM2G130230_P02, GRMZM2G130230_P03	TRUE	TRUE	gAPEDLSEFLQLIk	93%	n+304 (+304), K+304 (+304)	27.54	25.33
4626	seq=translation; coord=9:149239142..149244150:1; parent_transcript=GRMZM2G004528_T03; parent_gene=GRMZM2G004528	GRMZM2G004528_P03,GRMZM2G155242_P01	TRUE	TRUE	acVGLAPENNMILEYk	95%	n+304 (+304), Carbamidomethyl (+57), K+304 (+304)	34.99	25.39
4627	seq=translation; coord=9:149239142..149244150:1; parent_transcript=GRMZM2G004528_T03; parent_gene=GRMZM2G004528	GRMZM2G004528_P03,GRMZM2G155242_P01	TRUE	TRUE	aMDEYTSEIFMGGk	95%	n+304 (+304), K+304 (+304)	46.89	25.00
4628	seq=translation; coord=9:149239142..149244150:1; parent_transcript=GRMZM2G004528_T03; parent_gene=GRMZM2G004528	GRMZM2G004528_P03,GRMZM2G155242_P01	TRUE	TRUE	eGISWATk	95%	n+304 (+304), K+304 (+304)	32.08	27.31
4629	seq=translation; coord=9:149239142..149244150:1; parent_transcript=GRMZM2G004528_T03; parent_gene=GRMZM2G004528	GRMZM2G004528_P03,GRMZM2G155242_P01	TRUE	TRUE	vLDIDLQk	95%	n+304 (+304), K+304 (+304)	37.50	25.00
4630	seq=translation; coord=9:149239142..149244150:1; parent_transcript=GRMZM2G004528_T03; parent_gene=GRMZM2G004528	GRMZM2G004528_P03,GRMZM2G155242_P01	TRUE	TRUE	yGPTEIESEYR	95%	n+304 (+304)	41.74	25.00
4631	seq=translation; coord=4:237868446..237932504:1; parent_transcript=GRMZM2G092627_T01; parent_gene=GRMZM2G092627	GRMZM2G092627_P01	TRUE	TRUE	gAAFGLAGVVk	95%	n+304 (+304), K+304 (+304)	49.91	25.00
4632	seq=translation; coord=4:237868446..237932504:1; parent_transcript=GRMZM2G092627_T01; parent_gene=GRMZM2G092627	GRMZM2G092627_P01	TRUE	TRUE	lLDVLNTPSEAVQR	94%	n+304 (+304)	30.84	26.36
4633	seq=translation; coord=4:237868446..237932504:1; parent_transcript=GRMZM2G092627_T01; parent_gene=GRMZM2G092627	GRMZM2G092627_P01	TRUE	TRUE	lVLPSLLk	93%	n+304 (+304), K+304 (+304)	26.23	25.00
4634	seq=translation; coord=4:237868446..237932504:1; parent_transcript=GRMZM2G092627_T01; parent_gene=GRMZM2G092627	GRMZM2G092627_P01	TRUE	TRUE	qGIALALHSIADVLASk	95%	Pyro-cmC (-17), n+304 (+304), K+304 (+304)	43.09	25.00
4635	seq=translation; coord=5:128105440..128107392:-1; parent_transcript=GRMZM2G071089_T01; parent_gene=GRMZM2G071089	GRMZM2G071089_P01	TRUE	TRUE	aAAAAELVk	95%	n+304 (+304), K+304 (+304)	41.12	26.92
4636	seq=translation; coord=5:128105440..128107392:-1; parent_transcript=GRMZM2G071089_T01; parent_gene=GRMZM2G071089	GRMZM2G071089_P01	TRUE	TRUE	aGPALLSGPIFATFEk	95%	n+304 (+304), K+304 (+304)	51.01	25.00

4637	seq=translation; coord=5:128105440..128107392:-1; parent_transcript=GRMZM2G071089_T01; parent_gene=GRMZM2G071089	GRMZM2G071089_P01	TRUE	TRUE	eGIDGEFQEk	95%	n+304 (+304), K+304 (+304)	50.88	25.56
4638	seq=translation; coord=5:128105440..128107392:-1; parent_transcript=GRMZM2G071089_T01; parent_gene=GRMZM2G071089	GRMZM2G071089_P01	TRUE	TRUE	vSTLLPVVAAEEPAk	95%	n+304 (+304), K+304 (+304)	37.64	25.00
4639	seq=translation; coord=3:52892215..52895069:-1; parent_transcript=GRMZM2G033226_T01; parent_gene=GRMZM2G033226	GRMZM2G033226_P01	TRUE	TRUE	mLDFDFLcGR	95%	n+304 (+304), Carbamidomethyl (+57)	33.61	25.00
4640	seq=translation; coord=3:52892215..52895069:-1; parent_transcript=GRMZM2G033226_T01; parent_gene=GRMZM2G033226	GRMZM2G033226_P01	TRUE	TRUE	sAAASSMSALk	95%	n+304 (+304), K+304 (+304)	42.07	26.54
4641	seq=translation; coord=3:52892215..52895069:-1; parent_transcript=GRMZM2G033226_T01; parent_gene=GRMZM2G033226	GRMZM2G033226_P01	TRUE	TRUE	vIIGPATVGGIQAGAFk	95%	n+304 (+304), K+304 (+304)	59.63	25.00
4642	seq=translation; coord=2:224904656..224907978:1; parent_transcript=GRMZM2G408768_T01; parent_gene=GRMZM2G408768	GRMZM2G408768_P01	TRUE	TRUE	aAQDIALADLAPTHPIR	95%	n+304 (+304)	64.37	25.54
4643	seq=translation; coord=2:224904656..224907978:1; parent_transcript=GRMZM2G408768_T01; parent_gene=GRMZM2G408768	GRMZM2G408768_P01	TRUE	TRUE	sAGGAGGGEELSVEER	95%	n+304 (+304)	61.83	25.00
4644	seq=translation; coord=2:224904656..224907978:1; parent_transcript=GRMZM2G408768_T01; parent_gene=GRMZM2G408768	GRMZM2G408768_P01	TRUE	TRUE	yEEMVEFMER	91%	n+304 (+304)	28.05	25.00
4645	seq=translation; coord=1:298431972..298436349:1; parent_transcript=GRMZM2G021742_T01; parent_gene=GRMZM2G021742	GRMZM2G021742_P01	TRUE	TRUE	lQFLSLLDNTVTk	94%	n+304 (+304), K+304 (+304)	28.24	25.00
4646	seq=translation; coord=1:298431972..298436349:1; parent_transcript=GRMZM2G021742_T01; parent_gene=GRMZM2G021742	GRMZM2G021742_P01	TRUE	TRUE	lTADLIWk	95%	n+304 (+304), K+304 (+304)	27.83	25.00
4647	seq=translation; coord=1:298431972..298436349:1; parent_transcript=GRMZM2G021742_T01; parent_gene=GRMZM2G021742	GRMZM2G021742_P01	TRUE	TRUE	lTNLAEIDPLASLPk	92%	n+304 (+304), K+304 (+304)	25.87	25.00
4648	seq=translation; coord=1:298431972..298436349:1; parent_transcript=GRMZM2G021742_T01; parent_gene=GRMZM2G021742	GRMZM2G021742_P01	TRUE	TRUE	mDTDGENQEGGEADGQk	95%	n+304 (+304), K+304 (+304)	83.85	25.00
4649	seq=translation; coord=4:100154058..100157650:1; parent_transcript=GRMZM2G091995_T01; parent_gene=GRMZM2G091995	GRMZM2G091995_P01,GRMZM2G091995_P02	TRUE	TRUE	eVSLPAPAPPTR	89%	n+304 (+304)	27.34	25.55
4650	seq=translation; coord=4:100154058..100157650:1; parent_transcript=GRMZM2G091995_T01; parent_gene=GRMZM2G091995	GRMZM2G091995_P01,GRMZM2G091995_P02	TRUE	TRUE	gYFVLNDMFR	95%	n+304 (+304)	38.19	25.00
4651	seq=translation; coord=4:100154058..100157650:1; parent_transcript=GRMZM2G091995_T01; parent_gene=GRMZM2G091995	GRMZM2G091995_P01,GRMZM2G091995_P02	TRUE	TRUE	lGRPASAAGAAGMDSVTTMEAIGEk	95%	n+304 (+304), K+304 (+304)	56.20	25.54
4652	seq=translation; coord=4:100154058..100157650:1; parent_transcript=GRMZM2G091995_T01; parent_gene=GRMZM2G091995	GRMZM2G091995_P01,GRMZM2G091995_P02	TRUE	TRUE	nLPLNATETQLEDEFk	95%	n+304 (+304), K+304 (+304)	68.66	25.05
4653	seq=translation; coord=4:100154058..100157650:1; parent_transcript=GRMZM2G091995_T01; parent_gene=GRMZM2G091995	GRMZM2G091995_P01,GRMZM2G091995_P02	TRUE	TRUE	qSPALAPVk	89%	Pyro-cmC (-17), n+304 (+304), K+304 (+304)	25.84	25.00
4654	seq=translation; coord=9:86866692..86874101:-1; parent_transcript=GRMZM2G022275_T01; parent_gene=GRMZM2G022275	GRMZM2G022275_P01,GRMZM2G022275_P02	TRUE	TRUE	gAVWSccLDR	91%	n+304 (+304), Carbamidomethyl (+57), Carbamidomethyl (+57)	28.22	25.00

4655	seq=translation; coord=9:86866692..86874101:-1; parent_transcript=GRMZM2G022275_T01; parent_gene=GRMZM2G022275	GRMZM2G022275_P01,GRMZM2G022275_P02	TRUE	TRUE	lWDVR	92%	n+304 (+304)	30.61	26.79
4656	seq=translation; coord=9:86866692..86874101:-1; parent_transcript=GRMZM2G022275_T01; parent_gene=GRMZM2G022275	GRMZM2G022275_P01,GRMZM2G022275_P02	TRUE	TRUE	vWDALTGDELHSFEHk	95%	n+304 (+304), K+304 (+304)	29.54	25.00
4657	seq=translation; coord=8:155644343..155646847:-1; parent_transcript=GRMZM2G151252_T01; parent_gene=GRMZM2G151252	GRMZM2G151252_P01,GRMZM2G151252_P02, GRMZM2G151252_P04	TRUE	TRUE	sTGFGLIYDNLESAk	95%	n+304 (+304), K+304 (+304)	32.55	25.81
4658	seq=translation; coord=7:108281609..108388418:-1; parent_transcript=GRMZM2G700188_T04; parent_gene=GRMZM2G700188	GRMZM2G700188_P04,GRMZM2G700188_P05	TRUE	TRUE	dDSGVLSPYSFSR	92%	n+304 (+304)	28.84	25.00
4659	seq=translation; coord=7:108281609..108388418:-1; parent_transcript=GRMZM2G700188_T04; parent_gene=GRMZM2G700188	GRMZM2G700188_P04,GRMZM2G700188_P05	TRUE	TRUE	gGFSDVLVASEHYVVR	95%	n+304 (+304)	29.76	25.29
4660	seq=translation; coord=7:108281609..108388418:-1; parent_transcript=GRMZM2G700188_T04; parent_gene=GRMZM2G700188	GRMZM2G700188_P04,GRMZM2G700188_P05	TRUE	TRUE	hLGVVGLGGLGHVAVk	95%	n+304 (+304), K+304 (+304)	30.94	25.00
4661	seq=translation; coord=7:108281609..108388418:-1; parent_transcript=GRMZM2G700188_T04; parent_gene=GRMZM2G700188	GRMZM2G700188_P04,GRMZM2G700188_P05	TRUE	TRUE	mDYVNTAMER	91%	n+304 (+304)	26.77	25.00
4662	seq=translation; coord=7:108281609..108388418:-1; parent_transcript=GRMZM2G700188_T04; parent_gene=GRMZM2G700188	GRMZM2G700188_P04,GRMZM2G700188_P05	TRUE	TRUE	rQEAIENLGADEFLISR	95%	n+304 (+304)	54.83	25.91
4663	seq=translation; coord=4:2112375..2118463:-1; parent_transcript=GRMZM2G057450_T01; parent_gene=GRMZM2G057450	GRMZM2G057450_P01,GRMZM2G057450_P02, GRMZM2G057450_P03,GRMZM2G057450_P04, GRMZM2G057450_P06,GRMZM5G813627_P01, GRMZM5G813627_P02	TRUE	TRUE	fAFVEMR	95%	n+304 (+304)	29.93	25.00
4664	seq=translation; coord=4:2112375..2118463:-1; parent_transcript=GRMZM2G057450_T01; parent_gene=GRMZM2G057450	GRMZM2G057450_P01,GRMZM2G057450_P02, GRMZM2G057450_P03,GRMZM2G057450_P04, GRMZM2G057450_P06,GRMZM5G813627_P01, GRMZM5G813627_P02	TRUE	TRUE	iFVGGLPYYFTEAQVR	95%	n+304 (+304)	46.22	25.00
4665	seq=translation; coord=4:2112375..2118463:-1; parent_transcript=GRMZM2G057450_T01; parent_gene=GRMZM2G057450	GRMZM2G057450_P01,GRMZM2G057450_P02, GRMZM2G057450_P03,GRMZM2G057450_P04, GRMZM2G057450_P06,GRMZM5G813627_P01, GRMZM5G813627_P02	TRUE	TRUE	sVEEASNAMALDGIMFEGAPVk	95%	n+304 (+304), K+304 (+304)	35.41	25.00
4666	seq=translation; coord=7:166387724..166390583:1; parent_transcript=GRMZM2G054136_T01; parent_gene=GRMZM2G054136	GRMZM2G054136_P01,GRMZM2G054136_P03, GRMZM2G054136_P04,GRMZM2G054136_P05, GRMZM2G054136_P07,GRMZM2G054136_P09, GRMZM5G851698_P02	TRUE	TRUE	fNIANPSTGcQk	90%	n+304 (+304), Carbamidomethyl (+57), K+304 (+304)	28.43	25.77
4667	seq=translation; coord=7:166387724..166390583:1; parent_transcript=GRMZM2G054136_T01; parent_gene=GRMZM2G054136	GRMZM2G054136_P01,GRMZM2G054136_P03, GRMZM2G054136_P04,GRMZM2G054136_P05, GRMZM2G054136_P07,GRMZM2G054136_P09, GRMZM5G851698_P02	TRUE	TRUE	gcIVSQDLSVINLVIVk	95%	n+304 (+304), Carbamidomethyl (+57), K+304 (+304)	37.67	25.00
4668	seq=translation; coord=7:166387724..166390583:1; parent_transcript=GRMZM2G054136_T01; parent_gene=GRMZM2G054136	GRMZM2G054136_P01,GRMZM2G054136_P03, GRMZM2G054136_P04,GRMZM2G054136_P05, GRMZM2G054136_P07,GRMZM2G054136_P09, GRMZM5G851698_P02	TRUE	TRUE	iSQEVSGDALGEEFk	95%	n+304 (+304), K+304 (+304)	52.47	25.50
4669	seq=translation; coord=7:166387724..166390583:1; parent_transcript=GRMZM2G054136_T01; parent_gene=GRMZM2G054136	GRMZM2G054136_P01,GRMZM2G054136_P03, GRMZM2G054136_P04,GRMZM2G054136_P05, GRMZM2G054136_P07,GRMZM2G054136_P09, GRMZM5G851698_P02	TRUE	TRUE	sEAAEYQk	92%	n+304 (+304), K+304 (+304)	27.24	25.75

4670	seq=translation; coord=6:167112832..167116884:-1; parent_transcript=GRMZM2G144372_T01; parent_gene=GRMZM2G144372	GRMZM2G144372_P01	TRUE	TRUE	dFEYSPEAQESR	95%	n+304 (+304)	42.22	25.00
4671	seq=translation; coord=6:167112832..167116884:-1; parent_transcript=GRMZM2G144372_T01; parent_gene=GRMZM2G144372	GRMZM2G144372_P01	TRUE	TRUE	dWLSSYETLDTFVVPR	95%	n+304 (+304)	75.59	25.26
4672	seq=translation; coord=6:167112832..167116884:-1; parent_transcript=GRMZM2G144372_T01; parent_gene=GRMZM2G144372	GRMZM2G144372_P01	TRUE	TRUE	qIEDLER	95%	n+304 (+304)	34.18	25.16
4673	seq=translation; coord=6:167112832..167116884:-1; parent_transcript=GRMZM2G144372_T01; parent_gene=GRMZM2G144372	GRMZM2G144372_P01	TRUE	TRUE	vGTLDSLLALSDDLVk	95%	n+304 (+304), K+304 (+304)	59.79	25.00
4674	seq=translation; coord=3:230441023..230443000:1; parent_transcript=GRMZM2G353103_T01; parent_gene=GRMZM2G353103	GRMZM2G353103_P01,GRMZM2G377797_P01	TRUE	TRUE	aFEPILLAGR	95%	n+304 (+304)	35.78	25.92
4675	seq=translation; coord=3:230441023..230443000:1; parent_transcript=GRMZM2G353103_T01; parent_gene=GRMZM2G353103	GRMZM2G353103_P01,GRMZM2G377797_P01	TRUE	TRUE	gLVAYYQk	95%	n+304 (+304), K+304 (+304)	32.69	25.00
4676	seq=translation; coord=3:230441023..230443000:1; parent_transcript=GRMZM2G353103_T01; parent_gene=GRMZM2G353103	GRMZM2G353103_P01,GRMZM2G377797_P01	TRUE	TRUE	tLLVADPR	87%	n+304 (+304)	27.01	26.50
4677	seq=translation; coord=8:5159968..5162055:1; parent_transcript=GRMZM2G044128_T01; parent_gene=GRMZM2G044128	GRMZM2G044128_P01	TRUE	TRUE	gEAIAIGIAEMTTAVMATcDHGAVSk	95%	n+304 (+304), Carbamidomethyl (+57), K+304 (+304)	35.08	25.00
4678	seq=translation; coord=2:235441754..235443878:-1; parent_transcript=GRMZM2G082974_T01; parent_gene=GRMZM2G082974	GRMZM2G082974_P01	TRUE	TRUE	dLLLTVMSSMGEEQIcAVk	94%	n+304 (+304), Carbamidomethyl (+57), K+304 (+304)	28.76	25.49
4679	seq=translation; coord=2:235441754..235443878:-1; parent_transcript=GRMZM2G082974_T01; parent_gene=GRMZM2G082974	GRMZM2G082974_P01	TRUE	TRUE	lPTDEALEGQIk	95%	n+304 (+304), K+304 (+304)	31.90	25.47
4680	seq=translation; coord=2:235441754..235443878:-1; parent_transcript=GRMZM2G082974_T01; parent_gene=GRMZM2G082974	GRMZM2G082974_P01	TRUE	TRUE	tEYQLIDITEDGFVSLLTDSGNTk	93%	n+304 (+304), K+304 (+304)	26.60	25.00
4681	seq=translation; coord=2:235441754..235443878:-1; parent_transcript=GRMZM2G082974_T01; parent_gene=GRMZM2G082974	GRMZM2G082974_P01	TRUE	TRUE	tYPQQAGTIR	94%	n+304 (+304)	31.00	25.00
4682	seq=translation; coord=1:275223498..275227310:-1; parent_transcript=GRMZM2G169671_T01; parent_gene=GRMZM2G169671	GRMZM2G169671_P01	TRUE	TRUE	fAFILYDTLSk	95%	n+304 (+304), K+304 (+304)	40.56	26.01
4683	seq=translation; coord=1:275223498..275227310:-1; parent_transcript=GRMZM2G169671_T01; parent_gene=GRMZM2G169671	GRMZM2G169671_P01	TRUE	TRUE	sPPVAGAAAGAGGAGAGALMk	95%	n+304 (+304), K+304 (+304)	81.50	25.84
4684	seq=translation; coord=5:115978543..115983314:-1; parent_transcript=GRMZM2G452633_T01; parent_gene=GRMZM2G452633	GRMZM2G452633_P01,GRMZM2G452633_P02, GRMZM2G452633_P03	TRUE	TRUE	aTPMPSFYk	95%	n+304 (+304), K+304 (+304)	45.14	26.11
4685	seq=translation; coord=5:115978543..115983314:-1; parent_transcript=GRMZM2G452633_T01; parent_gene=GRMZM2G452633	GRMZM2G452633_P01,GRMZM2G452633_P02, GRMZM2G452633_P03	TRUE	TRUE	lPSEETcPLDATSR	95%	n+304 (+304), Carbamidomethyl (+57)	40.46	25.00
4686	seq=translation; coord=5:115978543..115983314:-1; parent_transcript=GRMZM2G452633_T01; parent_gene=GRMZM2G452633	GRMZM2G452633_P01,GRMZM2G452633_P02, GRMZM2G452633_P03	TRUE	TRUE	sISETEENTTTDQPAR	95%	n+304 (+304)	75.98	25.00
4687	seq=translation; coord=7:16096163..16099019:1; parent_transcript=GRMZM2G170044_T01; parent_gene=GRMZM2G170044	GRMZM2G170044_P01	TRUE	TRUE	dAQVFEQYAk	90%	n+304 (+304), K+304 (+304)	26.10	25.83

4688	seq=translation; coord=7:16096163..16099019:1; parent_transcript=GRMZM2G170044_T01; parent_gene=GRMZM2G170044	GRMZM2G170044_P01	TRUE	TRUE	lLDLPAGSk	95%	n+304 (+304), K+304 (+304)	31.08	25.65
4689	seq=translation; coord=7:16096163..16099019:1; parent_transcript=GRMZM2G170044_T01; parent_gene=GRMZM2G170044	GRMZM2G170044_P01	TRUE	TRUE	sSAAASTTAGAQk	95%	n+304 (+304), K+304 (+304)	39.09	26.46
4690	seq=translation; coord=3:188328660..188331609:-1; parent_transcript=GRMZM2G047274_T01; parent_gene=GRMZM2G047274	GRMZM2G047274_P01	TRUE	TRUE	fGFDEADIR	95%	n+304 (+304)	33.00	25.00
4691	seq=translation; coord=3:188328660..188331609:-1; parent_transcript=GRMZM2G047274_T01; parent_gene=GRMZM2G047274	GRMZM2G047274_P01	TRUE	TRUE	sLPLSTLIEMLk	95%	n+304 (+304), K+304 (+304)	36.46	25.00
4692	seq=translation; coord=10:38522742..38534648:1; parent_transcript=GRMZM2G010406_T01; parent_gene=GRMZM2G010406	GRMZM2G010406_P01,GRMZM2G010406_P03, GRMZM2G010406_P06	TRUE	TRUE	dLkEEFVSEYIYPcLR	95%	n+304 (+304), K+304 (+304), Carbamidomethyl (+57)	37.45	25.00
4693	seq=translation; coord=10:38522742..38534648:1; parent_transcript=GRMZM2G010406_T01; parent_gene=GRMZM2G010406	GRMZM2G010406_P01,GRMZM2G010406_P03, GRMZM2G010406_P06	TRUE	TRUE	dLSPASLLAELNEIGGk	95%	n+304 (+304), K+304 (+304)	47.14	25.17
4694	seq=translation; coord=10:38522742..38534648:1; parent_transcript=GRMZM2G010406_T01; parent_gene=GRMZM2G010406	GRMZM2G010406_P01,GRMZM2G010406_P03, GRMZM2G010406_P06	TRUE	TRUE	eDAIEYAk	95%	n+304 (+304), K+304 (+304)	34.80	26.35
4695	seq=translation; coord=10:38522742..38534648:1; parent_transcript=GRMZM2G010406_T01; parent_gene=GRMZM2G010406	GRMZM2G010406_P01,GRMZM2G010406_P03, GRMZM2G010406_P06	TRUE	TRUE	fELTFYALNPELk	91%	n+304 (+304), K+304 (+304)	26.42	25.91
4696	seq=translation; coord=10:38522742..38534648:1; parent_transcript=GRMZM2G010406_T01; parent_gene=GRMZM2G010406	GRMZM2G010406_P01,GRMZM2G010406_P03, GRMZM2G010406_P06	TRUE	TRUE	gVYETPGGTIMAAAVR	95%	n+304 (+304)	46.75	25.02
4697	seq=translation; coord=10:3062645..3066771:-1; parent_transcript=GRMZM2G068465_T01; parent_gene=GRMZM2G068465	GRMZM2G068465_P01,GRMZM2G068465_P02, GRMZM5G899349_P01,GRMZM5G899349_P02	TRUE	TRUE	aIWTHLLR	95%	n+304 (+304)	34.74	25.00
4698	seq=translation; coord=10:3062645..3066771:-1; parent_transcript=GRMZM2G068465_T01; parent_gene=GRMZM2G068465	GRMZM2G068465_P01,GRMZM2G068465_P02, GRMZM5G899349_P01,GRMZM5G899349_P02	TRUE	TRUE	iDHDILAEMDVLYR	95%	n+304 (+304)	48.37	25.04
4699	seq=translation; coord=10:3062645..3066771:-1; parent_transcript=GRMZM2G068465_T01; parent_gene=GRMZM2G068465	GRMZM2G068465_P01,GRMZM2G068465_P02, GRMZM5G899349_P01,GRMZM5G899349_P02	TRUE	TRUE	lAEIFATIIk	95%	n+304 (+304), K+304 (+304)	36.49	25.00
4700	seq=translation; coord=2:21258086..21262803:1; parent_transcript=GRMZM2G019919_T01; parent_gene=GRMZM2G019919	GRMZM2G019919_P01,GRMZM2G019919_P02, GRMZM2G019919_P03	TRUE	TRUE	lMMNPVTk	95%	n+304 (+304), K+304 (+304)	31.95	26.48
4701	seq=translation; coord=2:21258086..21262803:1; parent_transcript=GRMZM2G019919_T01; parent_gene=GRMZM2G019919	GRMZM2G019919_P01,GRMZM2G019919_P02, GRMZM2G019919_P03	TRUE	TRUE	tVFLDGLPPSWDEDR	95%	n+304 (+304)	58.73	25.00
4702	seq=translation; coord=2:21258086..21262803:1; parent_transcript=GRMZM2G019919_T01; parent_gene=GRMZM2G019919	GRMZM2G019919_P01,GRMZM2G019919_P02, GRMZM2G019919_P03	TRUE	TRUE	vFGEVGEITEVR	95%	n+304 (+304)	40.56	25.00
4703	seq=translation; coord=2:21258086..21262803:1; parent_transcript=GRMZM2G019919_T01; parent_gene=GRMZM2G019919	GRMZM2G019919_P01,GRMZM2G019919_P02, GRMZM2G019919_P03	TRUE	TRUE	vSFADSYPQVDDEIMAQVR	95%	n+304 (+304)	36.35	25.00
4704	seq=translation; coord=6:150509782..150519705:1; parent_transcript=GRMZM2G118037_T01; parent_gene=GRMZM2G118037	GRMZM2G118037_P01	TRUE	TRUE	nAASGFSAk	88%	n+304 (+304), K+304 (+304)	27.64	26.93
4705	seq=translation; coord=6:150509782..150519705:1; parent_transcript=GRMZM2G118037_T01; parent_gene=GRMZM2G118037	GRMZM2G118037_P01	TRUE	TRUE	qAAPAAQETAVHIVYVDRPEDADPEEFHIR	95%	Pyro-cmC (-17), n+304 (+304)	29.02	25.00

4706	seq=translation; coord=6:150509782..150519705:1; parent_transcript=GRMZM2G118037_T01; parent_gene=GRMZM2G118037	GRMZM2G118037_P01	TRUE	TRUE	tLTPVLGSEQk	90%	n+304 (+304), K+304 (+304)	28.13	25.87
4707	seq=translation; coord=8:134463276..134464095:1; parent_transcript=GRMZM2G028393_T01; parent_gene=GRMZM2G028393	GRMZM2G028393_P01,GRMZM2G028393_P02	TRUE	TRUE	iFVDIVAQTPHIG	95%	n+304 (+304)	33.13	26.79
4708	seq=translation; coord=8:134463276..134464095:1; parent_transcript=GRMZM2G028393_T01; parent_gene=GRMZM2G028393	GRMZM2G028393_P01,GRMZM2G028393_P02	TRUE	TRUE	tSWPEVVGLSVEDAk	95%	n+304 (+304), K+304 (+304)	35.69	26.20
4709	seq=translation; coord=1:292879723..292882219:-1; parent_transcript=AC225147.4_FGT002; parent_gene=AC225147.4_FG002	AC225147.4_FGP002,GRMZM2G084465_P01,GRMZM2G084465_P03,GRMZM2G110952_P12,GRMZM2G140609_P01,GRMZM2G163561_P01	TRUE	TRUE	gIVLEk	90%	n+304 (+304), K+304 (+304)	28.27	26.70
4710	seq=translation; coord=1:292879723..292882219:-1; parent_transcript=AC225147.4_FGT002; parent_gene=AC225147.4_FG002	AC225147.4_FGP002,GRMZM2G084465_P01,GRMZM2G084465_P03,GRMZM2G110952_P12,GRMZM2G140609_P01,GRMZM2G163561_P01	TRUE	TRUE	vSGVSLLALFk	95%	n+304 (+304), K+304 (+304)	35.56	25.00
4711	seq=translation; coord=8:15778520..15799328:1; parent_transcript=GRMZM2G097775_T01; parent_gene=GRMZM2G097775	GRMZM2G097775_P01,GRMZM2G097775_P02	TRUE	TRUE	eGDWVcPDAScGNVNFAR	95%	n+304 (+304), Carbamidomethyl (+57), Carbamidomethyl (+57)	77.00	25.00
4712	seq=translation; coord=8:15778520..15799328:1; parent_transcript=GRMZM2G097775_T01; parent_gene=GRMZM2G097775	GRMZM2G097775_P01,GRMZM2G097775_P02	TRUE	TRUE	iSVVMAEk	94%	n+304 (+304), K+304 (+304)	30.27	26.87
4713	seq=translation; coord=8:15778520..15799328:1; parent_transcript=GRMZM2G097775_T01; parent_gene=GRMZM2G097775	GRMZM2G097775_P01,GRMZM2G097775_P02	TRUE	TRUE	iyISNLPPDVTVEELQELFGGIGQVGR	95%	n+304 (+304), iTRAQ8plex (+304)	27.92	25.00
4714	seq=translation; coord=8:113008265..113010860:-1; parent_transcript=GRMZM2G044947_T01; parent_gene=GRMZM2G044947	GRMZM2G044947_P01,GRMZM2G044947_P02, GRMZM2G044947_P03	TRUE	TRUE	gANMAIIGATTMDFDFFk	95%	n+304 (+304), K+304 (+304)	55.17	25.00
4715	seq=translation; coord=8:113008265..113010860:-1; parent_transcript=GRMZM2G044947_T01; parent_gene=GRMZM2G044947	GRMZM2G044947_P01,GRMZM2G044947_P02, GRMZM2G044947_P03	TRUE	TRUE	iMYADFYTQVTHMIR	95%	n+304 (+304)	74.87	25.00
4716	seq=translation; coord=8:113008265..113010860:-1; parent_transcript=GRMZM2G044947_T01; parent_gene=GRMZM2G044947	GRMZM2G044947_P01,GRMZM2G044947_P02, GRMZM2G044947_P03	TRUE	TRUE	vIVDFLAEHFGLPLLPASk	93%	n+304 (+304), K+304 (+304)	25.08	25.00
4717	seq=translation; coord=6:129086004..129087221:1; parent_transcript=GRMZM2G447795_T01; parent_gene=GRMZM2G447795	GRMZM2G447795_P01	TRUE	TRUE	aPNYGGVMVWNR	93%	n+304 (+304)	29.69	25.00
4718	seq=translation; coord=6:129086004..129087221:1; parent_transcript=GRMZM2G447795_T01; parent_gene=GRMZM2G447795	GRMZM2G447795_P01	TRUE	TRUE	tGELTVFWGR	95%	n+304 (+304)	47.93	25.00
4719	seq=translation; coord=6:129086004..129087221:1; parent_transcript=GRMZM2G447795_T01; parent_gene=GRMZM2G447795	GRMZM2G447795_P01	TRUE	TRUE	yLYYDLLPk	95%	n+304 (+304), K+304 (+304)	30.18	26.06
4720	seq=translation; coord=1:84413344..84417967:1; parent_transcript=GRMZM2G083173_T01; parent_gene=GRMZM2G083173	GRMZM2G083173_P01,GRMZM2G083173_P02	TRUE	TRUE	gMPLEVITEFFSVGAk	95%	n+304 (+304), K+304 (+304)	33.76	25.02
4721	seq=translation; coord=1:84413344..84417967:1; parent_transcript=GRMZM2G083173_T01; parent_gene=GRMZM2G083173	GRMZM2G083173_P01,GRMZM2G083173_P02	TRUE	TRUE	gmPLEVITEFFSVGAk	95%	n+304 (+304), Oxidation (+16), K+304 (+304)	32.53	25.54
4722	seq=translation; coord=1:84413344..84417967:1; parent_transcript=GRMZM2G083173_T01; parent_gene=GRMZM2G083173	GRMZM2G083173_P01,GRMZM2G083173_P02	TRUE	TRUE	gREDVSGEMALLVEGLGVGk	95%	n+304 (+304), K+304 (+304)	29.98	25.94

4723	seq=translation; coord=1:84413344..84417967:1; parent_transcript=GRMZM2G083173_T01; parent_gene=GRMZM2G083173	GRMZM2G083173_P01,GRMZM2G083173_P02	TRUE	TRUE	sTLFPNFGSMFSVADQQQVk	95%	n+304 (+304), K+304 (+304)	51.85	25.00
4724	seq=translation; coord=4:14570220..14574637:-1; parent_transcript=GRMZM5G840928_T01; parent_gene=GRMZM5G840928	GRMZM5G840928_P01,GRMZM5G840928_P03, GRMZM5G840928_P04	TRUE	TRUE	dPFLLEcVSNIMR	95%	n+304 (+304), Carbamidomethyl (+57)	39.40	25.00
4725	seq=translation; coord=4:14570220..14574637:-1; parent_transcript=GRMZM5G840928_T01; parent_gene=GRMZM5G840928	GRMZM5G840928_P01,GRMZM5G840928_P03, GRMZM5G840928_P04	TRUE	TRUE	vNPMTLGcIAASLLLR	95%	n+304 (+304), Carbamidomethyl (+57)	58.49	25.00
4726	seq=translation; coord=6:88903392..88908316:-1; parent_transcript=GRMZM2G150256_T01; parent_gene=GRMZM2G150256	GRMZM2G150256_P01	TRUE	TRUE	aFQLYSSGIFDGR	95%	n+304 (+304)	57.66	25.00
4727	seq=translation; coord=6:88903392..88908316:-1; parent_transcript=GRMZM2G150256_T01; parent_gene=GRMZM2G150256	GRMZM2G150256_P01	TRUE	TRUE	dQGQcGAcWAFSAVAAVEGINk	95%	n+304 (+304), Carbamidomethyl (+57), Carbamidomethyl (+57), K+304 (+304)	46.58	25.00
4728	seq=translation; coord=6:88903392..88908316:-1; parent_transcript=GRMZM2G150256_T01; parent_gene=GRMZM2G150256	GRMZM2G150256_P01	TRUE	TRUE	nSWGTQWGEAGYVR	95%	n+304 (+304)	53.60	25.00
4729	seq=translation; coord=6:88903392..88908316:-1; parent_transcript=GRMZM2G150256_T01; parent_gene=GRMZM2G150256	GRMZM2G150256_P01	TRUE	TRUE	vVSIDSFER	95%	n+304 (+304)	36.93	25.00
4730	seq=translation; coord=1:198219532..198222746:1; parent_transcript=GRMZM2G318475_T02; parent_gene=GRMZM2G318475	GRMZM2G318475_P02,GRMZM2G318475_P03	TRUE	TRUE	eGQPTAIVEDMR	95%	n+304 (+304)	43.23	25.00
4731	seq=translation; coord=2:210443691..210447027:-1; parent_transcript=GRMZM5G830681_T01; parent_gene=GRMZM5G830681	GRMZM5G830681_P01	TRUE	TRUE	nLDFLEMWR	95%	n+304 (+304)	38.06	25.00
4732	seq=translation; coord=2:13403335..13406342:-1; parent_transcript=GRMZM2G354053_T01; parent_gene=GRMZM2G354053	GRMZM2G354053_P01	TRUE	TRUE	aMEGLASALQEMSAESR	95%	n+304 (+304)	34.84	25.00
4733	seq=translation; coord=2:13403335..13406342:-1; parent_transcript=GRMZM2G354053_T01; parent_gene=GRMZM2G354053	GRMZM2G354053_P01	TRUE	TRUE	aQEVEHLIAEVTR	92%	n+304 (+304)	27.35	25.94
4734	seq=translation; coord=2:13403335..13406342:-1; parent_transcript=GRMZM2G354053_T01; parent_gene=GRMZM2G354053	GRMZM2G354053_P01	TRUE	TRUE	lQQLESQLMEANR	95%	n+304 (+304)	53.62	25.04
4735	seq=translation; coord=2:13403335..13406342:-1; parent_transcript=GRMZM2G354053_T01; parent_gene=GRMZM2G354053	GRMZM2G354053_P01	TRUE	TRUE	nEALSQVDDAVR	95%	n+304 (+304)	33.81	25.26
4736	seq=translation; coord=2:13403335..13406342:-1; parent_transcript=GRMZM2G354053_T01; parent_gene=GRMZM2G354053	GRMZM2G354053_P01	TRUE	TRUE	vLAELER	90%	n+304 (+304)	28.02	25.73
4737	seq=translation; coord=8:171412337..171415029:-1; parent_transcript=GRMZM2G012160_T01; parent_gene=GRMZM2G012160	GRMZM2G012160_P01	TRUE	TRUE	aNALLGFEk	95%	n+304 (+304), K+304 (+304)	41.97	25.85
4738	seq=translation; coord=8:171412337..171415029:-1; parent_transcript=GRMZM2G012160_T01; parent_gene=GRMZM2G012160	GRMZM2G012160_P01	TRUE	TRUE	dVPGNENDLHLQELAR	95%	n+304 (+304)	76.55	25.00
4739	seq=translation; coord=8:171412337..171415029:-1; parent_transcript=GRMZM2G012160_T01; parent_gene=GRMZM2G012160	GRMZM2G012160_P01	TRUE	TRUE	fAVDEHNk	95%	n+304 (+304), K+304 (+304)	42.65	25.00

4740	seq=translation; coord=8:171412337..171415029:-1; parent_transcript=GRMZM2G012160_T01; parent_gene=GRMZM2G012160	GRMZM2G012160_P01	TRUE	TRUE	tQVVAGTMYYLTIEVk	95%	n+304 (+304), K+304 (+304)	41.27	26.00
4741	seq=translation; coord=3:213775068..213778961:1; parent_transcript=GRMZM2G047298_T01; parent_gene=GRMZM2G047298	GRMZM2G047298_P01	TRUE	TRUE	aALAAMSkPVYLLDVTLLSQLR	95%	n+304 (+304), K+304 (+304)	51.54	25.00
4742	seq=translation; coord=3:213775068..213778961:1; parent_transcript=GRMZM2G047298_T01; parent_gene=GRMZM2G047298	GRMZM2G047298_P01	TRUE	TRUE	sTYLVDIVEESVGR	95%	n+304 (+304)	42.34	25.74
4743	seq=translation; coord=3:213775068..213778961:1; parent_transcript=GRMZM2G047298_T01; parent_gene=GRMZM2G047298	GRMZM2G047298_P01	TRUE	TRUE	wVDSSVDTSR	95%	n+304 (+304)	33.18	25.00
4744	seq=translation; coord=2:33498879..33500909:-1; parent_transcript=GRMZM2G051898_T01; parent_gene=GRMZM2G051898	GRMZM2G051898_P01	TRUE	TRUE	aVELVSGMETALVQNVATVPGQLyR	95%	n+304 (+304), iTRAQ8plex (+304)	40.82	25.00
4745	seq=translation; coord=2:33498879..33500909:-1; parent_transcript=GRMZM2G051898_T01; parent_gene=GRMZM2G051898	GRMZM2G051898_P01	TRUE	TRUE	gVLDFAAVADQTR	95%	n+304 (+304)	54.79	25.00
4746	seq=translation; coord=2:33498879..33500909:-1; parent_transcript=GRMZM2G051898_T01; parent_gene=GRMZM2G051898	GRMZM2G051898_P01	TRUE	TRUE	nAIPHWEISGYVEYIGSGQk	95%	n+304 (+304), K+304 (+304)	33.70	25.00
4747	seq=translation; coord=4:182566741..182604691:1; parent_transcript=GRMZM2G439339_T04; parent_gene=GRMZM2G439339	GRMZM2G439339_P04,GRMZM2G439339_P05	TRUE	TRUE	ePSSNPAQAGASSEQR	95%	n+304 (+304)	29.74	25.00
4748	seq=translation; coord=4:182566741..182604691:1; parent_transcript=GRMZM2G439339_T04; parent_gene=GRMZM2G439339	GRMZM2G439339_P04,GRMZM2G439339_P05	TRUE	TRUE	nTSTVVEAAETR	95%	n+304 (+304)	40.99	25.00
4749	seq=translation; coord=4:182566741..182604691:1; parent_transcript=GRMZM2G439339_T04; parent_gene=GRMZM2G439339	GRMZM2G439339_P04,GRMZM2G439339_P05	TRUE	TRUE	sEAIIESFDDDR	95%	n+304 (+304)	38.87	25.00
4750	seq=translation; coord=7:173600066..173601522:1; parent_transcript=GRMZM2G348125_T01; parent_gene=GRMZM2G348125	GRMZM2G348125_P01,GRMZM2G410766_P01	TRUE	TRUE	aLPPQPTVk	90%	n+304 (+304), K+304 (+304)	27.07	25.00
4751	seq=translation; coord=7:173600066..173601522:1; parent_transcript=GRMZM2G348125_T01; parent_gene=GRMZM2G348125	GRMZM2G348125_P01,GRMZM2G410766_P01	TRUE	TRUE	aNPNYAMSVR	94%	n+304 (+304)	30.95	25.00
4752	seq=translation; coord=7:173600066..173601522:1; parent_transcript=GRMZM2G348125_T01; parent_gene=GRMZM2G348125	GRMZM2G348125_P01,GRMZM2G410766_P01	TRUE	TRUE	lVPYNPDFLDESVLWTESR	92%	n+304 (+304)	26.91	25.00
4753	seq=translation; coord=1:240863375..240866448:1; parent_transcript=GRMZM2G124434_T01; parent_gene=GRMZM2G124434	GRMZM2G124434_P01	TRUE	TRUE	eSDAWPAEPQDAYGLEk	95%	n+304 (+304), K+304 (+304)	44.37	25.00
4754	seq=translation; coord=1:240863375..240866448:1; parent_transcript=GRMZM2G124434_T01; parent_gene=GRMZM2G124434	GRMZM2G124434_P01	TRUE	TRUE	iSITGAGGFIGSHIAR	95%	n+304 (+304)	81.48	26.82
4755	seq=translation; coord=1:240863375..240866448:1; parent_transcript=GRMZM2G124434_T01; parent_gene=GRMZM2G124434	GRMZM2G124434_P01	TRUE	TRUE	lATEELck	91%	n+304 (+304), Carbamidomethyl (+57), K+304 (+304)	29.38	26.33
4756	seq=translation; coord=1:240863375..240866448:1; parent_transcript=GRMZM2G124434_T01; parent_gene=GRMZM2G124434	GRMZM2G124434_P01	TRUE	TRUE	nEHMTEDMFcHEFHLVDLR	95%	n+304 (+304), Carbamidomethyl (+57)	33.66	25.00
4757	seq=translation; coord=1:240863375..240866448:1; parent_transcript=GRMZM2G124434_T01; parent_gene=GRMZM2G124434	GRMZM2G124434_P01	TRUE	TRUE	sFTFIDEcVEGVLR	95%	n+304 (+304), Carbamidomethyl (+57)	49.46	25.00

4758	seq=translation; coord=1:240863375..240866448:1; parent_transcript=GRMZM2G124434_T01; parent_gene=GRMZM2G124434	GRMZM2G124434_P01	TRUE	TRUE	tVTAYGEYTYAELER	95%	n+304 (+304)	67.44	25.00
4759	seq=translation; coord=1:240863375..240866448:1; parent_transcript=GRMZM2G124434_T01; parent_gene=GRMZM2G124434	GRMZM2G124434_P01	TRUE	TRUE	vVSTQAPVQLGSLR	95%	n+304 (+304)	38.96	25.00
4760	seq=translation; coord=3:38743793..38746868:-1; parent_transcript=GRMZM2G033626_T01; parent_gene=GRMZM2G033626	GRMZM2G033626_P01,GRMZM2G033626_P02, GRMZM2G467059_P03	TRUE	TRUE	aGVPMEVMGLMLGEFVDDyTVR	95%	n+304 (+304), iTRAQ8plex (+304)	38.20	25.00
4761	seq=translation; coord=3:38743793..38746868:-1; parent_transcript=GRMZM2G033626_T01; parent_gene=GRMZM2G033626	GRMZM2G033626_P01,GRMZM2G033626_P02, GRMZM2G467059_P03	TRUE	TRUE	lAIANVGR	87%	n+304 (+304)	25.79	25.00
4762	seq=translation; coord=3:38743793..38746868:-1; parent_transcript=GRMZM2G033626_T01; parent_gene=GRMZM2G033626	GRMZM2G033626_P01,GRMZM2G033626_P02, GRMZM2G467059_P03	TRUE	TRUE	lINPQTMMLGQEPR	95%	n+304 (+304)	32.23	25.42
4763	seq=translation; coord=3:38743793..38746868:-1; parent_transcript=GRMZM2G033626_T01; parent_gene=GRMZM2G033626	GRMZM2G033626_P01,GRMZM2G033626_P02, GRMZM2G467059_P03	TRUE	TRUE	mLLNLHk	95%	n+304 (+304), K+304 (+304)	32.73	25.00
4764	seq=translation; coord=3:38743793..38746868:-1; parent_transcript=GRMZM2G033626_T01; parent_gene=GRMZM2G033626	GRMZM2G033626_P01,GRMZM2G033626_P02, GRMZM2G467059_P03	TRUE	TRUE	tNEQTVQEMLNLAIk	95%	n+304 (+304), K+304 (+304)	29.80	25.35
4765	seq=translation; coord=3:71260323..71320829:1; parent_transcript=GRMZM2G314652_T02; parent_gene=GRMZM2G314652	GRMZM2G314652_P02	TRUE	TRUE	aLGFYDVVFDAVYAPk	95%	n+304 (+304), K+304 (+304)	40.88	25.31
4766	seq=translation; coord=3:71260323..71320829:1; parent_transcript=GRMZM2G314652_T02; parent_gene=GRMZM2G314652	GRMZM2G314652_P02	TRUE	TRUE	eHLPPLLR	95%	n+304 (+304)	31.45	25.00
4767	seq=translation; coord=3:71260323..71320829:1; parent_transcript=GRMZM2G314652_T02; parent_gene=GRMZM2G314652	GRMZM2G314652_P02	TRUE	TRUE	iLGLIANPVk	95%	n+304 (+304), K+304 (+304)	31.57	25.00
4768	seq=translation; coord=3:71260323..71320829:1; parent_transcript=GRMZM2G314652_T02; parent_gene=GRMZM2G314652	GRMZM2G314652_P02	TRUE	TRUE	lDFIQGFRPR	93%	n+304 (+304)	27.55	25.19
4769	seq=translation; coord=3:71260323..71320829:1; parent_transcript=GRMZM2G314652_T02; parent_gene=GRMZM2G314652	GRMZM2G314652_P02	TRUE	TRUE	lVGYNTDYIGAISAIEDGIGGPGSk	95%	n+304 (+304), K+304 (+304)	29.48	25.00
4770	seq=translation; coord=1:226551729..226556786:-1; parent_transcript=GRMZM2G131769_T01; parent_gene=GRMZM2G131769	GRMZM2G131769_P01	TRUE	TRUE	eVGSAAELQAAVAGAR	95%	n+304 (+304)	62.03	25.28
4771	seq=translation; coord=1:226551729..226556786:-1; parent_transcript=GRMZM2G131769_T01; parent_gene=GRMZM2G131769	GRMZM2G131769_P01	TRUE	TRUE	nEEAGEPESATEk	95%	n+304 (+304), K+304 (+304)	43.10	25.00
4772	seq=translation; coord=2:224332394..224344331:1; parent_transcript=GRMZM2G169095_T01; parent_gene=GRMZM2G169095	GRMZM2G169095_P01	TRUE	TRUE	eALLWTDGR	92%	n+304 (+304)	29.21	25.09
4773	seq=translation; coord=2:224332394..224344331:1; parent_transcript=GRMZM2G169095_T01; parent_gene=GRMZM2G169095	GRMZM2G169095_P01	TRUE	TRUE	eFISGFTGSAGLALITMk	95%	n+304 (+304), K+304 (+304)	29.51	25.00
4774	seq=translation; coord=2:224332394..224344331:1; parent_transcript=GRMZM2G169095_T01; parent_gene=GRMZM2G169095	GRMZM2G169095_P01	TRUE	TRUE	iLEPHLNEQEk	95%	n+304 (+304), K+304 (+304)	35.22	26.54
4775	seq=translation; coord=2:224332394..224344331:1; parent_transcript=GRMZM2G169095_T01; parent_gene=GRMZM2G169095	GRMZM2G169095_P01	TRUE	TRUE	nPTELDGLR	89%	n+304 (+304)	26.11	25.02

4776	seq=translation; coord=6:134096678..134101001:-1; parent_transcript=GRMZM2G339091_T01; parent_gene=GRMZM2G339091	GRMZM2G339091_P01	TRUE	TRUE	gSSYDPcTER	86%	n+304 (+304), Carbamidomethyl (+57)	25.44	25.00
4777	seq=translation; coord=6:134096678..134101001:-1; parent_transcript=GRMZM2G339091_T01; parent_gene=GRMZM2G339091	GRMZM2G339091_P01	TRUE	TRUE	iRPDGATLFLNAYR	95%	n+304 (+304)	34.36	25.66
4778	seq=translation; coord=6:134096678..134101001:-1; parent_transcript=GRMZM2G339091_T01; parent_gene=GRMZM2G339091	GRMZM2G339091_P01	TRUE	TRUE	iWVFSGDTDAVVPLTATR	95%	n+304 (+304)	67.56	25.31
4779	seq=translation; coord=6:134096678..134101001:-1; parent_transcript=GRMZM2G339091_T01; parent_gene=GRMZM2G339091	GRMZM2G339091_P01	TRUE	TRUE	qALILFR	85%	n+304 (+304)	25.04	25.00
4780	seq=translation; coord=6:134096678..134101001:-1; parent_transcript=GRMZM2G339091_T01; parent_gene=GRMZM2G339091	GRMZM2G339091_P01	TRUE	TRUE	ySIDALNLPTVVSWYPWYDAk	95%	n+304 (+304), K+304 (+304)	34.97	25.08
4781	seq=translation; coord=1:22629310..22633408:1; parent_transcript=GRMZM2G055025_T01; parent_gene=GRMZM2G055025	GRMZM2G055025_P01	TRUE	TRUE	aSAVAELTR	94%	n+304 (+304)	31.06	25.75
4782	seq=translation; coord=1:22629310..22633408:1; parent_transcript=GRMZM2G055025_T01; parent_gene=GRMZM2G055025	GRMZM2G055025_P01	TRUE	TRUE	fGLALALkP	95%	n+304 (+304), K+304 (+304)	28.92	25.00
4783	seq=translation; coord=1:22629310..22633408:1; parent_transcript=GRMZM2G055025_T01; parent_gene=GRMZM2G055025	GRMZM2G055025_P01	TRUE	TRUE	gPGLFSDIGk	92%	n+304 (+304), K+304 (+304)	29.99	25.75
4784	seq=translation; coord=1:22629310..22633408:1; parent_transcript=GRMZM2G055025_T01; parent_gene=GRMZM2G055025	GRMZM2G055025_P01	TRUE	TRUE	lTISTASASGLGLTSTAVk	95%	n+304 (+304), K+304 (+304)	62.82	25.00
4785	seq=translation; coord=1:5345118..5348832:1; parent_transcript=GRMZM2G119494_T01; parent_gene=GRMZM2G119494	GRMZM2G119494_P01	TRUE	TRUE	aEDSVAINTESAVR	95%	n+304 (+304)	68.50	25.00
4786	seq=translation; coord=1:5345118..5348832:1; parent_transcript=GRMZM2G119494_T01; parent_gene=GRMZM2G119494	GRMZM2G119494_P01	TRUE	TRUE	hWPLIAPLPSYGR	95%	n+304 (+304)	36.78	25.49
4787	seq=translation; coord=1:5345118..5348832:1; parent_transcript=GRMZM2G119494_T01; parent_gene=GRMZM2G119494	GRMZM2G119494_P01	TRUE	TRUE	lTcVSPTSAMIALGSEMSGGIR	95%	n+304 (+304), Carbamidomethyl (+57)	58.69	25.00
4788	seq=translation; coord=1:5345118..5348832:1; parent_transcript=GRMZM2G119494_T01; parent_gene=GRMZM2G119494	GRMZM2G119494_P01	TRUE	TRUE	vTEYGAVGDGR	93%	n+304 (+304)	30.04	25.00
4789	seq=translation; coord=5:175584484..175585836:1; parent_transcript=GRMZM2G049675_T01; parent_gene=GRMZM2G049675	GRMZM2G049675_P01,GRMZM2G128219_P01	TRUE	TRUE	gAVLIHPWFWGSEAVGEEPR	95%	n+304 (+304)	42.15	25.00
4790	seq=translation; coord=5:175584484..175585836:1; parent_transcript=GRMZM2G049675_T01; parent_gene=GRMZM2G049675	GRMZM2G049675_P01,GRMZM2G128219_P01	TRUE	TRUE	lAPEHPLPAGYDDcLAALk	95%	n+304 (+304), Carbamidomethyl (+57), K+304 (+304)	28.01	25.72
4791	seq=translation; coord=5:175584484..175585836:1; parent_transcript=GRMZM2G049675_T01; parent_gene=GRMZM2G049675	GRMZM2G049675_P01,GRMZM2G128219_P01	TRUE	TRUE	wVLSAADPWVAAHGDLAR	95%	n+304 (+304)	92.01	25.48
4792	seq=translation; coord=1:250092343..250095992:-1; parent_transcript=GRMZM2G364069_T01; parent_gene=GRMZM2G364069	GRMZM2G364069_P01	TRUE	TRUE	qAHAAQEAYEEDDEMPGGAQR	95%	n+304 (+304)	58.38	25.00
4793	seq=translation; coord=1:250092343..250095992:-1; parent_transcript=GRMZM2G364069_T01; parent_gene=GRMZM2G364069	GRMZM2G364069_P01	TRUE	TRUE	vLEVHVEk	95%	n+304 (+304), K+304 (+304)	31.14	25.00

4794	seq=translation; coord=1:65994545..65996015:-1; parent_transcript=GRMZM2G004298_T01; parent_gene=GRMZM2G004298	GRMZM2G004298_P01,GRMZM2G004298_P02, GRMZM2G004298_P03,GRMZM2G096100_P01, GRMZM2G096100_P02,GRMZM2G096100_P03	TRUE	TRUE	gDVFVFPR	95%	n+304 (+304)	31.72	25.00
4795	seq=translation; coord=1:65994545..65996015:-1; parent_transcript=GRMZM2G004298_T01; parent_gene=GRMZM2G004298	GRMZM2G004298_P01,GRMZM2G004298_P02, GRMZM2G004298_P03,GRMZM2G096100_P01, GRMZM2G096100_P02,GRMZM2G096100_P03	TRUE	TRUE	nPGNTNNPAGSVVTAANVEk	95%	n+304 (+304), K+304 (+304)	32.07	25.58
4796	seq=translation; coord=1:274494460..274510748:-1; parent_transcript=GRMZM2G396451_T02; parent_gene=GRMZM2G396451	GRMZM2G396451_P02,GRMZM2G396451_P03	TRUE	TRUE	dSVETSPLLQYR	95%	n+304 (+304)	44.48	25.01
4797	seq=translation; coord=1:274494460..274510748:-1; parent_transcript=GRMZM2G396451_T02; parent_gene=GRMZM2G396451	GRMZM2G396451_P02,GRMZM2G396451_P03	TRUE	TRUE	eDYGELSSIAR	95%	n+304 (+304)	40.56	25.00
4798	seq=translation; coord=1:274494460..274510748:-1; parent_transcript=GRMZM2G396451_T02; parent_gene=GRMZM2G396451	GRMZM2G396451_P02,GRMZM2G396451_P03	TRUE	TRUE	vVVDEGQALIDSVTGLPk	95%	n+304 (+304), K+304 (+304)	55.88	25.00
4799	seq=translation; coord=7:159001810..159027357:-1; parent_transcript=GRMZM2G081155_T01; parent_gene=GRMZM2G081155	GRMZM2G081155_P01,GRMZM2G081155_P02, GRMZM2G081155_P03	TRUE	TRUE	fSPDAIQNVLNELTPETVR	95%	n+304 (+304)	71.41	25.49
4800	seq=translation; coord=7:159001810..159027357:-1; parent_transcript=GRMZM2G081155_T01; parent_gene=GRMZM2G081155	GRMZM2G081155_P01,GRMZM2G081155_P02, GRMZM2G081155_P03	TRUE	TRUE	qNVLLQLLALVGk	95%	n+304 (+304), K+304 (+304)	28.95	25.00
4801	seq=translation; coord=7:159001810..159027357:-1; parent_transcript=GRMZM2G081155_T01; parent_gene=GRMZM2G081155	GRMZM2G081155_P01,GRMZM2G081155_P02, GRMZM2G081155_P03	TRUE	TRUE	sVEQLGYIALLR	95%	n+304 (+304)	39.12	26.01
4802	seq=translation; coord=2:62991591..62998602:-1; parent_transcript=GRMZM2G171688_T01; parent_gene=GRMZM2G171688	GRMZM2G171688_P01	TRUE	TRUE	lVVESLEAMTETEALDMk	95%	n+304 (+304), K+304 (+304)	67.51	25.48
4803	seq=translation; coord=2:62991591..62998602:-1; parent_transcript=GRMZM2G171688_T01; parent_gene=GRMZM2G171688	GRMZM2G171688_P01	TRUE	TRUE	vLAILDDLTAEQLGAk	95%	n+304 (+304), K+304 (+304)	45.02	25.00
4804	seq=translation; coord=5:13253262..13257697:-1; parent_transcript=GRMZM2G076524_T01; parent_gene=GRMZM2G076524	GRMZM2G076524_P01,GRMZM2G076524_P02	TRUE	TRUE	aAEAcEEFSGTLVTLR	95%	n+304 (+304), Carbamidomethyl (+57)	86.74	25.00
4805	seq=translation; coord=5:13253262..13257697:-1; parent_transcript=GRMZM2G076524_T01; parent_gene=GRMZM2G076524	GRMZM2G076524_P01,GRMZM2G076524_P02	TRUE	TRUE	dLLAASWNELPDSLVAEAk	95%	n+304 (+304), K+304 (+304)	67.46	25.75
4806	seq=translation; coord=5:13253262..13257697:-1; parent_transcript=GRMZM2G076524_T01; parent_gene=GRMZM2G076524	GRMZM2G076524_P01,GRMZM2G076524_P02	TRUE	TRUE	mIHLk	92%	n+304 (+304), K+304 (+304)	26.16	25.00
4807	seq=translation; coord=8:2052922..2061036:1; parent_transcript=GRMZM2G052875_T01; parent_gene=GRMZM2G052875	GRMZM2G052875_P01,GRMZM2G052875_P02	TRUE	TRUE	dkEFYSFYDQcFETk	95%	n+304 (+304), K+304 (+304), Carbamidomethyl (+57), K+304 (+304)	32.84	25.00
4808	seq=translation; coord=8:2052922..2061036:1; parent_transcript=GRMZM2G052875_T01; parent_gene=GRMZM2G052875	GRMZM2G052875_P01,GRMZM2G052875_P02	TRUE	TRUE	eQLNDDFcDcPDGTDEPGTSAcPEAk	95%	n+304 (+304), Carbamidomethyl (+57), Carbamidomethyl (+57), Carbamidomethyl (+57), K+304 (+304)	48.57	25.00
4809	seq=translation; coord=8:2052922..2061036:1; parent_transcript=GRMZM2G052875_T01; parent_gene=GRMZM2G052875	GRMZM2G052875_P01,GRMZM2G052875_P02	TRUE	TRUE	tPVDLSEASR	89%	n+304 (+304)	26.86	25.00

4810	seq=translation; coord=1:194657095..194659202:-1; parent_transcript=GRMZM2G083725_T01; parent_gene=GRMZM2G083725	GRMZM2G083725_P01,GRMZM2G083725_P02	TRUE	TRUE	aRPEcLcYIIQQTHTGR	95%	n+304 (+304), Carbamidomethyl (+57), Carbamidomethyl (+57)	39.57	25.00
4811	seq=translation; coord=1:194657095..194659202:-1; parent_transcript=GRMZM2G083725_T01; parent_gene=GRMZM2G083725	GRMZM2G083725_P01,GRMZM2G083725_P02	TRUE	TRUE	nQVQSLGLR	90%	n+304 (+304)	29.71	27.37
4812	seq=translation; coord=3:154973863..154975444:-1; parent_transcript=GRMZM2G146246_T02; parent_gene=GRMZM2G146246	GRMZM2G146246_P02	TRUE	TRUE	gLDFEIVPVDLTTGAHk	95%	n+304 (+304), K+304 (+304)	55.75	25.48
4813	seq=translation; coord=3:154973863..154975444:-1; parent_transcript=GRMZM2G146246_T02; parent_gene=GRMZM2G146246	GRMZM2G146246_P02	TRUE	TRUE	lYGMPLSPNVVR	95%	n+304 (+304)	32.57	25.45
4814	seq=translation; coord=3:154973863..154975444:-1; parent_transcript=GRMZM2G146246_T02; parent_gene=GRMZM2G146246	GRMZM2G146246_P02	TRUE	TRUE	vATVLNEk	92%	n+304 (+304), K+304 (+304)	31.32	27.36
4815	seq=translation; coord=3:154973863..154975444:-1; parent_transcript=GRMZM2G146246_T02; parent_gene=GRMZM2G146246	GRMZM2G146246_P02	TRUE	TRUE	vLDVYEAHLAR	95%	n+304 (+304)	65.50	26.30
4816	seq=translation; coord=3:154973863..154975444:-1; parent_transcript=GRMZM2G146246_T02; parent_gene=GRMZM2G146246	GRMZM2G146246_P02	TRUE	TRUE	vLDVyEAHLAR	94%	n+304 (+304), iTRAQ8plex (+304)	25.69	25.16
4817	seq=translation; coord=1:174313373..174317518:1; parent_transcript=GRMZM2G088565_T01; parent_gene=GRMZM2G088565	GRMZM2G088565_P01	TRUE	TRUE	aDEAAAAAGSk	95%	n+304 (+304), K+304 (+304)	54.97	25.21
4818	seq=translation; coord=1:174313373..174317518:1; parent_transcript=GRMZM2G088565_T01; parent_gene=GRMZM2G088565	GRMZM2G088565_P01	TRUE	TRUE	gLAEMFGDLR	95%	n+304 (+304)	41.00	25.00
4819	seq=translation; coord=1:174313373..174317518:1; parent_transcript=GRMZM2G088565_T01; parent_gene=GRMZM2G088565	GRMZM2G088565_P01	TRUE	TRUE	gYDPEVIDIR	93%	n+304 (+304)	28.77	25.00
4820	seq=translation; coord=1:174313373..174317518:1; parent_transcript=GRMZM2G088565_T01; parent_gene=GRMZM2G088565	GRMZM2G088565_P01	TRUE	TRUE	sENPVVLFEHVLLYNLk	95%	n+304 (+304), K+304 (+304)	40.60	25.00
4821	seq=translation; coord=1:174313373..174317518:1; parent_transcript=GRMZM2G088565_T01; parent_gene=GRMZM2G088565	GRMZM2G088565_P01	TRUE	TRUE	sGGHELLMFEALR	95%	n+304 (+304)	29.03	25.13
4822	seq=translation; coord=1:174313373..174317518:1; parent_transcript=GRMZM2G088565_T01; parent_gene=GRMZM2G088565	GRMZM2G088565_P01	TRUE	TRUE	vLIVEEcMR	95%	n+304 (+304), Carbamidomethyl (+57)	38.92	25.00
4823	seq=translation; coord=7:137275571..137279379:1; parent_transcript=GRMZM2G458164_T01; parent_gene=GRMZM2G458164	GRMZM2G458164_P01	TRUE	TRUE	gPIWcVLSAAASNk	95%	n+304 (+304), Carbamidomethyl (+57), K+304 (+304)	47.44	26.19
4824	seq=translation; coord=7:137275571..137279379:1; parent_transcript=GRMZM2G458164_T01; parent_gene=GRMZM2G458164	GRMZM2G458164_P01	TRUE	TRUE	mPVFVFSLYNEDLkPGPGTER	95%	n+304 (+304), K+304 (+304)	29.39	25.44
4825	seq=translation; coord=7:137275571..137279379:1; parent_transcript=GRMZM2G458164_T01; parent_gene=GRMZM2G458164	GRMZM2G458164_P01	TRUE	TRUE	vADDIPSPWR	93%	n+304 (+304)	29.26	25.00
4826	seq=translation; coord=7:137275571..137279379:1; parent_transcript=GRMZM2G458164_T01; parent_gene=GRMZM2G458164	GRMZM2G458164_P01	TRUE	TRUE	wVAENLVPYYPATR	95%	n+304 (+304)	36.99	25.31
4827	seq=translation; coord=1:296874949..296877709:-1; parent_transcript=GRMZM2G035017_T01; parent_gene=GRMZM2G035017	GRMZM2G035017_P01	TRUE	TRUE	gGFDTIHVEDSMGHQFATR	95%	n+304 (+304)	34.38	25.00

4828	seq=translation; coord=2:160538409..160543692:1; parent_transcript=GRMZM2G151549_T01; parent_gene=GRMZM2G151549	GRMZM2G151549_P01,GRMZM2G151549_P02	TRUE	TRUE	hLVFPLLEFLQER	95%	n+304 (+304)	35.19	25.80
4829	seq=translation; coord=2:160538409..160543692:1; parent_transcript=GRMZM2G151549_T01; parent_gene=GRMZM2G151549	GRMZM2G151549_P01,GRMZM2G151549_P02	TRUE	TRUE	lIEcEQVILNDPFLGk	95%	n+304 (+304), Carbamidomethyl (+57), K+304 (+304)	56.42	25.39
4830	seq=translation; coord=2:160538409..160543692:1; parent_transcript=GRMZM2G151549_T01; parent_gene=GRMZM2G151549	GRMZM2G151549_P01,GRMZM2G151549_P02	TRUE	TRUE	sLEEAAAPLVAFLQNPQLVQEFRPDk	95%	n+304 (+304), K+304 (+304)	29.51	25.00
4831	seq=translation; coord=2:160538409..160543692:1; parent_transcript=GRMZM2G151549_T01; parent_gene=GRMZM2G151549	GRMZM2G151549_P01,GRMZM2G151549_P02	TRUE	TRUE	yQIGPDQIEALYQYAk	95%	n+304 (+304), K+304 (+304)	59.92	25.01
4832	seq=translation; coord=9:26618156..26621708:1; parent_transcript=GRMZM2G051890_T01; parent_gene=GRMZM2G051890	GRMZM2G051890_P01	TRUE	TRUE	aEDVVSTMLAk	95%	n+304 (+304), K+304 (+304)	31.71	26.60
4833	seq=translation; coord=8:135054057..135061548:1; parent_transcript=GRMZM2G098039_T01; parent_gene=GRMZM2G098039	GRMZM2G098039_P01	TRUE	TRUE	aAGFDGELDASNIR	91%	n+304 (+304)	27.81	25.00
4834	seq=translation; coord=3:205620317..205627720:1; parent_transcript=GRMZM2G157317_T01; parent_gene=GRMZM2G157317	GRMZM2G157317_P01,GRMZM2G157317_P03	TRUE	TRUE	aVFSADSALYEYLDGGWk	95%	n+304 (+304), K+304 (+304)	61.28	25.00
4835	seq=translation; coord=3:205620317..205627720:1; parent_transcript=GRMZM2G157317_T01; parent_gene=GRMZM2G157317	GRMZM2G157317_P01,GRMZM2G157317_P03	TRUE	TRUE	dNPEPDDEGTEQEMGTFk	95%	n+304 (+304), K+304 (+304)	33.79	25.00
4836	seq=translation; coord=3:205620317..205627720:1; parent_transcript=GRMZM2G157317_T01; parent_gene=GRMZM2G157317	GRMZM2G157317_P01,GRMZM2G157317_P03	TRUE	TRUE	gQTPSATPLFSFk	95%	n+304 (+304), K+304 (+304)	32.16	25.68
4837	seq=translation; coord=3:205620317..205627720:1; parent_transcript=GRMZM2G157317_T01; parent_gene=GRMZM2G157317	GRMZM2G157317_P01,GRMZM2G157317_P03	TRUE	TRUE	mFAMTEGPVETGEENEk	95%	n+304 (+304), K+304 (+304)	39.45	25.00
4838	seq=translation; coord=3:205620317..205627720:1; parent_transcript=GRMZM2G157317_T01; parent_gene=GRMZM2G157317	GRMZM2G157317_P01,GRMZM2G157317_P03	TRUE	TRUE	vVVGEPNEGScMPSEVEGk	95%	n+304 (+304), Carbamidomethyl (+57), K+304 (+304)	74.56	25.00
4839	seq=translation; coord=10:121538895..121542946:-1; parent_transcript=GRMZM2G381267_T01; parent_gene=GRMZM2G381267	GRMZM2G381267_P01,GRMZM2G381267_P02	TRUE	TRUE	eLSGSDIFGPPPEIPARPLAAR	95%	n+304 (+304)	38.38	25.25
4840	seq=translation; coord=10:121538895..121542946:-1; parent_transcript=GRMZM2G381267_T01; parent_gene=GRMZM2G381267	GRMZM2G381267_P01,GRMZM2G381267_P02	TRUE	TRUE	eLSGTLEDADAk	95%	n+304 (+304), K+304 (+304)	45.73	25.22
4841	seq=translation; coord=10:121538895..121542946:-1; parent_transcript=GRMZM2G381267_T01; parent_gene=GRMZM2G381267	GRMZM2G381267_P01,GRMZM2G381267_P02	TRUE	TRUE	eMGGSGIFAEk	95%	n+304 (+304), K+304 (+304)	36.10	25.93
4842	seq=translation; coord=10:121538895..121542946:-1; parent_transcript=GRMZM2G381267_T01; parent_gene=GRMZM2G381267	GRMZM2G381267_P01,GRMZM2G381267_P02	TRUE	TRUE	eMSGSDIFADGTPAPR	95%	n+304 (+304)	59.06	25.00
4843	seq=translation; coord=10:121538895..121542946:-1; parent_transcript=GRMZM2G381267_T01; parent_gene=GRMZM2G381267	GRMZM2G381267_P01,GRMZM2G381267_P02	TRUE	TRUE	nMELQGNVDFSLPQR	95%	n+304 (+304)	55.59	25.00
4844	seq=translation; coord=10:121538895..121542946:-1; parent_transcript=GRMZM2G381267_T01; parent_gene=GRMZM2G381267	GRMZM2G381267_P01,GRMZM2G381267_P02	TRUE	TRUE	vSNPAGGPSNISFSEEPVVk	95%	n+304 (+304), K+304 (+304)	44.04	25.22
4845	seq=translation; coord=7:155559747..155562921:1; parent_transcript=GRMZM2G108285_T01; parent_gene=GRMZM2G108285	GRMZM2G108285_P01	TRUE	TRUE	eSELIQENYLGVR	95%	n+304 (+304)	47.45	25.25

4846	seq=translation; coord=7:155559747..155562921:1; parent_transcript=GRMZM2G108285_T01; parent_gene=GRMZM2G108285	GRMZM2G108285_P01	TRUE	TRUE	gTLGEAVDMETIHk	95%	n+304 (+304), K+304 (+304)	47.23	25.22
4847	seq=translation; coord=7:155559747..155562921:1; parent_transcript=GRMZM2G108285_T01; parent_gene=GRMZM2G108285	GRMZM2G108285_P01	TRUE	TRUE	lVVDNFLMGWWDHVEk	95%	n+304 (+304), K+304 (+304)	56.82	25.44
4848	seq=translation; coord=7:155559747..155562921:1; parent_transcript=GRMZM2G108285_T01; parent_gene=GRMZM2G108285	GRMZM2G108285_P01	TRUE	TRUE	wGEHELDYLLFIVR	95%	n+304 (+304)	42.83	25.44
4849	seq=translation; coord=8:13369483..13372340:-1; parent_transcript=GRMZM2G119361_T01; parent_gene=GRMZM2G119361	GRMZM2G119361_P01,GRMZM2G119361_P02	TRUE	TRUE	gGDSWDEPFPSSAAAAAGGGGR	95%	n+304 (+304)	98.87	25.00
4850	seq=translation; coord=8:13369483..13372340:-1; parent_transcript=GRMZM2G119361_T01; parent_gene=GRMZM2G119361	GRMZM2G119361_P01,GRMZM2G119361_P02	TRUE	TRUE	lFLFPATPESSSSLGSLLDDSSk	95%	n+304 (+304), K+304 (+304)	38.73	25.00
4851	seq=translation; coord=8:13369483..13372340:-1; parent_transcript=GRMZM2G119361_T01; parent_gene=GRMZM2G119361	GRMZM2G119361_P01,GRMZM2G119361_P02	TRUE	TRUE	qEVPAIVDPTNR	95%	n+304 (+304)	31.61	25.81
4852	seq=translation; coord=8:13369483..13372340:-1; parent_transcript=GRMZM2G119361_T01; parent_gene=GRMZM2G119361	GRMZM2G119361_P01,GRMZM2G119361_P02	TRUE	TRUE	vFSDDDk	93%	n+304 (+304), K+304 (+304)	28.35	25.00
4853	seq=translation; coord=1:296017136..296022027:1; parent_transcript=GRMZM2G053458_T01; parent_gene=GRMZM2G053458	GRMZM2G053458_P01,GRMZM2G053458_P02	TRUE	TRUE	aGAcSTcAGk	95%	n+304 (+304), Carbamidomethyl (+57), Carbamidomethyl (+57), K+304 (+304)	34.02	25.00
4854	seq=translation; coord=1:296017136..296022027:1; parent_transcript=GRMZM2G053458_T01; parent_gene=GRMZM2G053458	GRMZM2G053458_P01,GRMZM2G053458_P02	TRUE	TRUE	lVGPEGEEHEFDAPDDAYILDAAETAGVELPYScR	95%	n+304 (+304), Carbamidomethyl (+57)	31.35	25.00
4855	seq=translation; coord=4:1508758..1509325:-1; parent_transcript=GRMZM2G107302_T01; parent_gene=GRMZM2G107302	GRMZM2G107302_P01	TRUE	TRUE	aEGSSNYASAcEPAVAPFk	95%	n+304 (+304), Carbamidomethyl (+57), K+304 (+304)	50.30	25.00
4856	seq=translation; coord=4:1508758..1509325:-1; parent_transcript=GRMZM2G107302_T01; parent_gene=GRMZM2G107302	GRMZM2G107302_P01	TRUE	TRUE	ecTDVNYDcNR	95%	n+304 (+304), Carbamidomethyl (+57), Carbamidomethyl (+57)	36.75	25.00
4857	seq=translation; coord=10:4071331..4073937:-1; parent_transcript=GRMZM2G129804_T01; parent_gene=GRMZM2G129804	GRMZM2G129804_P01	TRUE	TRUE	iIDFDLPITVR	95%	n+304 (+304)	36.40	25.00
4858	seq=translation; coord=10:4071331..4073937:-1; parent_transcript=GRMZM2G129804_T01; parent_gene=GRMZM2G129804	GRMZM2G129804_P01	TRUE	TRUE	vGTNVEVDGAPWk	95%	n+304 (+304), K+304 (+304)	44.18	25.00
4859	seq=translation; coord=9:25038179..25042320:-1; parent_transcript=GRMZM2G051247_T01; parent_gene=GRMZM2G051247	GRMZM2G051247_P01	TRUE	TRUE	dTESLNASYER	95%	n+304 (+304)	36.59	25.00
4860	seq=translation; coord=1:216692560..216696036:1; parent_transcript=GRMZM2G167669_T01; parent_gene=GRMZM2G167669	GRMZM2G167669_P01	TRUE	TRUE	aILFFEGQR	92%	n+304 (+304)	27.59	25.93
4861	seq=translation; coord=1:216692560..216696036:1; parent_transcript=GRMZM2G167669_T01; parent_gene=GRMZM2G167669	GRMZM2G167669_P01	TRUE	TRUE	sAIQWGADFLLR	95%	n+304 (+304)	41.14	25.00
4862	seq=translation; coord=1:216692560..216696036:1; parent_transcript=GRMZM2G167669_T01; parent_gene=GRMZM2G167669	GRMZM2G167669_P01	TRUE	TRUE	sSPGSEAAGEAAAALAAAYLVFR	95%	n+304 (+304)	35.83	25.00

4863	seq=translation; coord=1:216692560..216696036:1; parent_transcript=GRMZM2G167669_T01; parent_gene=GRMZM2G167669	GRMZM2G167669_P01	TRUE	TRUE	tLATQLLAASR	95%	n+304 (+304)	32.72	25.00
4864	seq=translation; coord=1:262391932..262394279:1; parent_transcript=GRMZM2G063503_T02; parent_gene=GRMZM2G063503	GRMZM2G063503_P02	TRUE	TRUE	cANIFEALVGTLR	95%	Carbamidomethyl (+57), n+304 (+304)	46.32	25.00
4865	seq=translation; coord=1:262391932..262394279:1; parent_transcript=GRMZM2G063503_T02; parent_gene=GRMZM2G063503	GRMZM2G063503_P02	TRUE	TRUE	fGVLFNDDR	91%	n+304 (+304)	27.87	25.00
4866	seq=translation; coord=1:262391932..262394279:1; parent_transcript=GRMZM2G063503_T02; parent_gene=GRMZM2G063503	GRMZM2G063503_P02	TRUE	TRUE	lGQQQPDGSYk	95%	n+304 (+304), K+304 (+304)	49.99	25.84
4867	seq=translation; coord=5:62197397..62202236:1; parent_transcript=GRMZM2G152111_T01; parent_gene=GRMZM2G152111	GRMZM2G152111_P01,GRMZM2G152111_P02, GRMZM2G152111_P03,GRMZM2G152111_P04, GRMZM2G152111_P06	TRUE	TRUE	gFAFVSMDTVEEAER	95%	n+304 (+304)	62.04	25.00
4868	seq=translation; coord=5:62197397..62202236:1; parent_transcript=GRMZM2G152111_T01; parent_gene=GRMZM2G152111	GRMZM2G152111_P01,GRMZM2G152111_P02, GRMZM2G152111_P03,GRMZM2G152111_P04, GRMZM2G152111_P06	TRUE	TRUE	gNTLYVTGLSSR	95%	n+304 (+304)	41.15	25.00
4869	seq=translation; coord=8:39126474..39127702:1; parent_transcript=GRMZM2G047732_T01; parent_gene=GRMZM2G047732	GRMZM2G047732_P01,GRMZM2G047732_P02	TRUE	TRUE	ecPNAEcGAGVFMANHFDR	95%	n+304 (+304), Carbamidomethyl (+57), Carbamidomethyl (+57)	75.01	25.00
4870	seq=translation; coord=8:39126474..39127702:1; parent_transcript=GRMZM2G047732_T01; parent_gene=GRMZM2G047732	GRMZM2G047732_P01,GRMZM2G047732_P02	TRUE	TRUE	lAVLQFYk	91%	n+304 (+304), K+304 (+304)	25.93	25.00
4871	seq=translation; coord=5:1122734..1125202:1; parent_transcript=GRMZM2G034326_T01; parent_gene=GRMZM2G034326	GRMZM2G034326_P01,GRMZM2G034326_P02, GRMZM2G034326_P03,GRMZM2G034326_P04	TRUE	TRUE	fNMVIAPTLNLDGTPDTGyYTQAGR	95%	n+304 (+304), iTRAQ8plex (+304)	33.36	25.13
4872	seq=translation; coord=5:1122734..1125202:1; parent_transcript=GRMZM2G034326_T01; parent_gene=GRMZM2G034326	GRMZM2G034326_P01,GRMZM2G034326_P02, GRMZM2G034326_P03,GRMZM2G034326_P04	TRUE	TRUE	gDPSTAASFELDQR	95%	n+304 (+304)	63.46	25.00
4873	seq=translation; coord=5:202050924..202059781:1; parent_transcript=GRMZM2G020040_T01; parent_gene=GRMZM2G020040	GRMZM2G020040_P01,GRMZM2G020040_P02	TRUE	TRUE	aIGDPILFR	95%	n+304 (+304)	34.89	25.00
4874	seq=translation; coord=5:202050924..202059781:1; parent_transcript=GRMZM2G020040_T01; parent_gene=GRMZM2G020040	GRMZM2G020040_P01,GRMZM2G020040_P02	TRUE	TRUE	aYELPPGIPSHk	92%	n+304 (+304), K+304 (+304)	27.27	25.53
4875	seq=translation; coord=5:202050924..202059781:1; parent_transcript=GRMZM2G020040_T01; parent_gene=GRMZM2G020040	GRMZM2G020040_P01,GRMZM2G020040_P02	TRUE	TRUE	fAEYSVSGLTDASEk	94%	n+304 (+304), K+304 (+304)	28.49	25.00
4876	seq=translation; coord=5:202050924..202059781:1; parent_transcript=GRMZM2G020040_T01; parent_gene=GRMZM2G020040	GRMZM2G020040_P01,GRMZM2G020040_P02	TRUE	TRUE	iDDIYAVELIGGATR	95%	n+304 (+304)	34.53	25.00
4877	seq=translation; coord=5:202050924..202059781:1; parent_transcript=GRMZM2G020040_T01; parent_gene=GRMZM2G020040	GRMZM2G020040_P01,GRMZM2G020040_P02	TRUE	TRUE	lSEVQDWLYMDGEDAQANEFk	94%	n+304 (+304), K+304 (+304)	28.03	25.00
4878	seq=translation; coord=5:202050924..202059781:1; parent_transcript=GRMZM2G020040_T01; parent_gene=GRMZM2G020040	GRMZM2G020040_P01,GRMZM2G020040_P02	TRUE	TRUE	lYLDELQk	95%	n+304 (+304), K+304 (+304)	40.87	25.00
4879	seq=translation; coord=5:202050924..202059781:1; parent_transcript=GRMZM2G020040_T01; parent_gene=GRMZM2G020040	GRMZM2G020040_P01,GRMZM2G020040_P02	TRUE	TRUE	vDEVVSEAEk	95%	n+304 (+304), K+304 (+304)	59.35	25.15
4880	seq=translation; coord=1:221648603..221653196:1; parent_transcript=GRMZM2G163437_T01; parent_gene=GRMZM2G163437	GRMZM2G163437_P01,GRMZM2G163437_P02	TRUE	TRUE	eTDGYFIk	95%	n+304 (+304), K+304 (+304)	34.09	25.34

4881	seq=translation; coord=1:221648603..221653196:1; parent_transcript=GRMZM2G163437_T01; parent_gene=GRMZM2G163437	GRMZM2G163437_P01,GRMZM2G163437_P02	TRUE	TRUE	iLNADNVQEAAR	95%	n+304 (+304)	60.29	25.45
4882	seq=translation; coord=10:125428223..125432495:1; parent_transcript=GRMZM2G066815_T01; parent_gene=GRMZM2G066815	GRMZM2G066815_P01	TRUE	TRUE	gLTHDEMLLLPTGPR	95%	n+304 (+304)	71.49	25.00
4883	seq=translation; coord=10:125428223..125432495:1; parent_transcript=GRMZM2G066815_T01; parent_gene=GRMZM2G066815	GRMZM2G066815_P01	TRUE	TRUE	iSQLESQLEQLSR	95%	n+304 (+304)	51.05	26.37
4884	seq=translation; coord=10:125428223..125432495:1; parent_transcript=GRMZM2G066815_T01; parent_gene=GRMZM2G066815	GRMZM2G066815_P01	TRUE	TRUE	kIDIELEHR	95%	K+304 (+304), n+304 (+304)	40.81	25.00
4885	seq=translation; coord=10:125428223..125432495:1; parent_transcript=GRMZM2G066815_T01; parent_gene=GRMZM2G066815	GRMZM2G066815_P01	TRUE	TRUE	sYGGGSGFGGPGGER	95%	n+304 (+304)	49.15	25.00
4886	seq=translation; coord=10:125428223..125432495:1; parent_transcript=GRMZM2G066815_T01; parent_gene=GRMZM2G066815	GRMZM2G066815_P01	TRUE	TRUE	yPSLGSGGGFR	95%	n+304 (+304)	29.72	25.00
4887	seq=translation; coord=10:116860755..116863190:1; parent_transcript=GRMZM2G043737_T01; parent_gene=GRMZM2G043737	GRMZM2G043737_P01,GRMZM2G043737_P05, GRMZM2G050925_P01	TRUE	TRUE	sGAPAEFQPSFR	95%	n+304 (+304)	40.48	25.00
4888	seq=translation; coord=5:23763272..23765390:-1; parent_transcript=GRMZM2G376743_T01; parent_gene=GRMZM2G376743	GRMZM2G376743_P01	TRUE	TRUE	aLSEAISGAVQR	95%	n+304 (+304)	34.91	26.68
4889	seq=translation; coord=5:23763272..23765390:-1; parent_transcript=GRMZM2G376743_T01; parent_gene=GRMZM2G376743	GRMZM2G376743_P01	TRUE	TRUE	eVEGDGVSPR	95%	n+304 (+304)	33.07	25.00
4890	seq=translation; coord=5:23763272..23765390:-1; parent_transcript=GRMZM2G376743_T01; parent_gene=GRMZM2G376743	GRMZM2G376743_P01	TRUE	TRUE	nIGVGEAEDDGAGGQAQLHR	95%	n+304 (+304)	106.64	25.00
4891	seq=translation; coord=5:23763272..23765390:-1; parent_transcript=GRMZM2G376743_T01; parent_gene=GRMZM2G376743	GRMZM2G376743_P01	TRUE	TRUE	qLASTVYEk	95%	n+304 (+304), K+304 (+304)	36.01	25.00
4892	seq=translation; coord=5:23763272..23765390:-1; parent_transcript=GRMZM2G376743_T01; parent_gene=GRMZM2G376743	GRMZM2G376743_P01	TRUE	TRUE	qLASTVYEk	95%	Pyro-cmC (-17), n+304 (+304), K+304 (+304)	35.86	25.00
4893	seq=translation; coord=9:77151999..77153846:1; parent_transcript=GRMZM2G080839_T01; parent_gene=GRMZM2G080839	GRMZM2G080839_P01	TRUE	TRUE	dLYAFMAPYVSSNPR	90%	n+304 (+304)	25.25	25.00
4894	seq=translation; coord=9:77151999..77153846:1; parent_transcript=GRMZM2G080839_T01; parent_gene=GRMZM2G080839	GRMZM2G080839_P01	TRUE	TRUE	dVWANIFSR	95%	n+304 (+304)	36.42	25.00
4895	seq=translation; coord=9:77151999..77153846:1; parent_transcript=GRMZM2G080839_T01; parent_gene=GRMZM2G080839	GRMZM2G080839_P01	TRUE	TRUE	kYGLAIDNVLDAVLVDAR	95%	K+304 (+304), n+304 (+304)	52.97	25.00
4896	seq=translation; coord=9:77151999..77153846:1; parent_transcript=GRMZM2G080839_T01; parent_gene=GRMZM2G080839	GRMZM2G080839_P01	TRUE	TRUE	wQQVAPALPDDLFIR	87%	n+304 (+304)	26.73	26.00
4897	seq=translation; coord=4:9657544..9658965:-1; parent_transcript=GRMZM2G117989_T01; parent_gene=GRMZM2G117989	GRMZM2G117989_P01,GRMZM2G117989_P02, GRMZM2G117989_P03,GRMZM2G117989_P04, GRMZM2G117989_P05,GRMZM2G117989_P06	TRUE	TRUE	aTGASIVAR	95%	n+304 (+304)	37.08	25.02

4898	seq=translation; coord=4:9657544..9658965:-1; parent_transcript=GRMZM2G117989_T01; parent_gene=GRMZM2G117989	GRMZM2G117989_P01,GRMZM2G117989_P02, GRMZM2G117989_P03,GRMZM2G117989_P04, GRMZM2G117989_P05,GRMZM2G117989_P06	TRUE	TRUE	hGWTAFcGPAGQk	95%	n+304 (+304), Carbamidomethyl (+57), K+304 (+304)	47.83	25.00
4899	seq=translation; coord=4:9657544..9658965:-1; parent_transcript=GRMZM2G117989_T01; parent_gene=GRMZM2G117989	GRMZM2G117989_P01,GRMZM2G117989_P02, GRMZM2G117989_P03,GRMZM2G117989_P04, GRMZM2G117989_P05,GRMZM2G117989_P06	TRUE	TRUE	vSAYcATWDADkPLSWR	95%	n+304 (+304), Carbamidomethyl (+57), K+304 (+304)	37.67	25.00
4900	seq=translation; coord=5:6444041..6445037:1; parent_transcript=GRMZM2G077034_T01; parent_gene=GRMZM2G077034	GRMZM2G077034_P01	TRUE	TRUE	hDDcLk	93%	n+304 (+304), Carbamidomethyl (+57), K+304 (+304)	27.70	25.00
4901	seq=translation; coord=5:6444041..6445037:1; parent_transcript=GRMZM2G077034_T01; parent_gene=GRMZM2G077034	GRMZM2G077034_P01	TRUE	TRUE	vSMAGSGFcDGk	95%	n+304 (+304), Carbamidomethyl (+57), K+304 (+304)	52.48	25.00
4902	seq=translation; coord=5:6444041..6445037:1; parent_transcript=GRMZM2G077034_T01; parent_gene=GRMZM2G077034	GRMZM2G077034_P01	TRUE	TRUE	vSmAGSGFcDGk	95%	n+304 (+304), Oxidation (+16), Carbamidomethyl (+57), K+304 (+304)	38.00	25.00
4903	seq=translation; coord=5:6444041..6445037:1; parent_transcript=GRMZM2G077034_T01; parent_gene=GRMZM2G077034	GRMZM2G077034_P01	TRUE	TRUE	ycGIccATcNcVPSGTAGNk	95%	n+304 (+304), Carbamidomethyl (+57), Carbamidomethyl (+57), Carbamidomethyl (+57), Carbamidomethyl (+57), Carbamidomethyl (+57), K+304 (+304)	31.25	25.00
4904	seq=translation; coord=5:193174093..193205154:1; parent_transcript=GRMZM5G883741_T02; parent_gene=GRMZM5G883741	GRMZM5G883741_P02	TRUE	TRUE	eLVLFIER	92%	n+304 (+304)	28.44	25.00
4905	seq=translation; coord=5:193174093..193205154:1; parent_transcript=GRMZM5G883741_T02; parent_gene=GRMZM5G883741	GRMZM5G883741_P02	TRUE	TRUE	sIPEQLDEEVR	95%	n+304 (+304)	53.71	25.00
4906	seq=translation; coord=1:27178033..27184068:-1; parent_transcript=GRMZM5G891739_T01; parent_gene=GRMZM5G891739	GRMZM5G891739_P01	TRUE	TRUE	aVVVHADPDDLGR	95%	n+304 (+304)	36.30	25.43
4907	seq=translation; coord=1:27178033..27184068:-1; parent_transcript=GRMZM5G891739_T01; parent_gene=GRMZM5G891739	GRMZM5G891739_P01	TRUE	TRUE	gVALIGGSANSTVAGVIHFFEDPSTR	95%	n+304 (+304)	56.26	25.65
4908	seq=translation; coord=1:27178033..27184068:-1; parent_transcript=GRMZM5G891739_T01; parent_gene=GRMZM5G891739	GRMZM5G891739_P01	TRUE	TRUE	hLGDLGNIVANEDGDAEVFIR	95%	n+304 (+304)	66.71	25.00
4909	seq=translation; coord=9:11741082..11743899:1; parent_transcript=GRMZM2G177098_T03; parent_gene=GRMZM2G177098	GRMZM2G177098_P03	TRUE	TRUE	eDVAWYAGk	95%	n+304 (+304), K+304 (+304)	47.50	25.88
4910	seq=translation; coord=9:11741082..11743899:1; parent_transcript=GRMZM2G177098_T03; parent_gene=GRMZM2G177098	GRMZM2G177098_P03	TRUE	TRUE	sNQYETTSLVQIEGVNTk	95%	n+304 (+304), K+304 (+304)	41.91	25.38
4911	seq=translation; coord=8:126595171..126599674:1; parent_transcript=GRMZM2G389118_T01; parent_gene=GRMZM2G389118	GRMZM2G389118_P01	TRUE	TRUE	dADGDDVVAQGGEEALEESTk	95%	n+304 (+304), K+304 (+304)	33.88	25.00
4912	seq=translation; coord=8:126595171..126599674:1; parent_transcript=GRMZM2G389118_T01; parent_gene=GRMZM2G389118	GRMZM2G389118_P01	TRUE	TRUE	fESAAAAAAEEEVDDR	95%	n+304 (+304)	35.46	25.00
4913	seq=translation; coord=8:126595171..126599674:1; parent_transcript=GRMZM2G389118_T01; parent_gene=GRMZM2G389118	GRMZM2G389118_P01	TRUE	TRUE	gAAVEVNVVDk	94%	n+304 (+304), K+304 (+304)	28.53	25.22

4914	seq=translation; coord=8:126595171..126599674:1; parent_transcript=GRMZM2G389118_T01; parent_gene=GRMZM2G389118	GRMZM2G389118_P01	TRUE	TRUE	gELQEEEQHDEER	95%	n+304 (+304)	67.91	25.00
4915	seq=translation; coord=8:126595171..126599674:1; parent_transcript=GRMZM2G389118_T01; parent_gene=GRMZM2G389118	GRMZM2G389118_P01	TRUE	TRUE	lVPEVEk	90%	n+304 (+304), K+304 (+304)	26.30	25.00
4916	seq=translation; coord=8:126595171..126599674:1; parent_transcript=GRMZM2G389118_T01; parent_gene=GRMZM2G389118	GRMZM2G389118_P01	TRUE	TRUE	qVVADVGEREEEEEEVR	95%	Pyro-cmC (-17), n+304 (+304)	37.53	25.00
4917	seq=translation; coord=2:164667085..164668830:-1; parent_transcript=AC210204.3_FGT002; parent_gene=AC210204.3_FG002	AC210204.3_FGP002	TRUE	TRUE	dVLDDAGVGGk	95%	n+304 (+304), K+304 (+304)	68.20	25.11
4918	seq=translation; coord=2:164667085..164668830:-1; parent_transcript=AC210204.3_FGT002; parent_gene=AC210204.3_FG002	AC210204.3_FGP002	TRUE	TRUE	lDDAVLYVDVVHANAAQGVkPLVGSAR	95%	n+304 (+304), K+304 (+304)	38.78	25.00
4919	seq=translation; coord=2:164667085..164668830:-1; parent_transcript=AC210204.3_FGT002; parent_gene=AC210204.3_FG002	AC210204.3_FGP002	TRUE	TRUE	lPLRDVLDDAGVGGk	95%	n+304 (+304), K+304 (+304)	28.97	25.00
4920	seq=translation; coord=5:171688070..171693270:-1; parent_transcript=GRMZM2G000278_T01; parent_gene=GRMZM2G000278	GRMZM2G000278_P01,GRMZM2G000278_P03, GRMZM2G000278_P04,GRMZM2G130018_P01, GRMZM2G130018_P03	TRUE	TRUE	dIGSGNFGVAR	95%	n+304 (+304)	39.96	25.00
4921	seq=translation; coord=5:171688070..171693270:-1; parent_transcript=GRMZM2G000278_T01; parent_gene=GRMZM2G000278	GRMZM2G000278_P01,GRMZM2G000278_P03, GRMZM2G000278_P04,GRMZM2G130018_P01, GRMZM2G130018_P03	TRUE	TRUE	sTVGTPAYIAPEVLSR	95%	n+304 (+304)	69.34	25.44
4922	seq=translation; coord=10:5718777..5728532:-1; parent_transcript=GRMZM2G020446_T01; parent_gene=GRMZM2G020446	GRMZM2G020446_P01	TRUE	TRUE	lQWFLDSIk	94%	n+304 (+304), K+304 (+304)	27.88	25.00
4923	seq=translation; coord=1:67436204..67440532:-1; parent_transcript=GRMZM2G107473_T01; parent_gene=GRMZM2G107473	GRMZM2G107473_P01	TRUE	TRUE	eLSGHDIFADTLDSR	95%	n+304 (+304)	41.47	25.00
4924	seq=translation; coord=1:67436204..67440532:-1; parent_transcript=GRMZM2G107473_T01; parent_gene=GRMZM2G107473	GRMZM2G107473_P01	TRUE	TRUE	kVVFGGQVTEEEADSLTk	95%	K+304 (+304), n+304 (+304), K+304 (+304)	51.54	25.25
4925	seq=translation; coord=1:67436204..67440532:-1; parent_transcript=GRMZM2G107473_T01; parent_gene=GRMZM2G107473	GRMZM2G107473_P01	TRUE	TRUE	nTNASTFSFGDANTDSTPk	95%	n+304 (+304), K+304 (+304)	78.20	25.00
4926	seq=translation; coord=1:67436204..67440532:-1; parent_transcript=GRMZM2G107473_T01; parent_gene=GRMZM2G107473	GRMZM2G107473_P01	TRUE	TRUE	vVFGGQVTEEEADSLTk	95%	n+304 (+304), K+304 (+304)	30.38	25.29
4927	seq=translation; coord=7:157907062..157937456:-1; parent_transcript=GRMZM2G335287_T01; parent_gene=GRMZM2G335287	GRMZM2G335287_P01	TRUE	TRUE	aMLTQMISIVFR	95%	n+304 (+304)	41.43	26.30
4928	seq=translation; coord=7:157907062..157937456:-1; parent_transcript=GRMZM2G335287_T01; parent_gene=GRMZM2G335287	GRMZM2G335287_P01	TRUE	TRUE	eLLEEIYDSIVk	95%	n+304 (+304), K+304 (+304)	42.91	25.82
4929	seq=translation; coord=7:157907062..157937456:-1; parent_transcript=GRMZM2G335287_T01; parent_gene=GRMZM2G335287	GRMZM2G335287_P01	TRUE	TRUE	gLIVDcIVQLIk	95%	n+304 (+304), Carbamidomethyl (+57), K+304 (+304)	29.64	25.00
4930	seq=translation; coord=7:157907062..157937456:-1; parent_transcript=GRMZM2G335287_T01; parent_gene=GRMZM2G335287	GRMZM2G335287_P01	TRUE	TRUE	lVNILNLALPR	95%	n+304 (+304)	38.14	25.00
4931	seq=translation; coord=7:157907062..157937456:-1; parent_transcript=GRMZM2G335287_T01; parent_gene=GRMZM2G335287	GRMZM2G335287_P01	TRUE	TRUE	vLFPIFDHVR	95%	n+304 (+304)	37.88	25.44

4932	seq=translation; coord=1:300990204..300994789:1; parent_transcript=GRMZM2G122267_T01; parent_gene=GRMZM2G122267	GRMZM2G122267_P01,GRMZM2G122267_P02	TRUE	TRUE	dAAFQEAHMWR	95%	n+304 (+304)	47.76	25.00
4933	seq=translation; coord=1:300990204..300994789:1; parent_transcript=GRMZM2G122267_T01; parent_gene=GRMZM2G122267	GRMZM2G122267_P01,GRMZM2G122267_P02	TRUE	TRUE	ekEELLALVGVLQSQVLR	95%	n+304 (+304), K+304 (+304)	37.25	25.00
4934	seq=translation; coord=1:300990204..300994789:1; parent_transcript=GRMZM2G122267_T01; parent_gene=GRMZM2G122267	GRMZM2G122267_P01,GRMZM2G122267_P02	TRUE	TRUE	lTETAEAAEAAASAAHTMDEQR	95%	n+304 (+304)	88.18	25.00
4935	seq=translation; coord=4:162075480..162081835:-1; parent_transcript=GRMZM2G042502_T01; parent_gene=GRMZM2G042502	GRMZM2G042502_P01,GRMZM2G123371_P01, GRMZM2G123371_P02	TRUE	TRUE	lIGFLGGSDGLLAQR	95%	n+304 (+304)	75.23	25.47
4936	seq=translation; coord=3:158520150..158525636:-1; parent_transcript=GRMZM2G081102_T01; parent_gene=GRMZM2G081102	GRMZM2G081102_P01,GRMZM2G081102_P02, GRMZM2G081102_P03,GRMZM2G145280_P01, GRMZM2G145280_P02,GRMZM2G409407_P01, GRMZM2G409407_P02	TRUE	TRUE	gFTLEELk	95%	n+304 (+304), K+304 (+304)	45.80	26.22
4937	seq=translation; coord=3:158520150..158525636:-1; parent_transcript=GRMZM2G081102_T01; parent_gene=GRMZM2G081102	GRMZM2G081102_P01,GRMZM2G081102_P02, GRMZM2G081102_P03,GRMZM2G145280_P01, GRMZM2G145280_P02,GRMZM2G409407_P01, GRMZM2G409407_P02	TRUE	TRUE	sLEGLQANVQR	95%	n+304 (+304)	33.42	26.45
4938	seq=translation; coord=5:4422815..4424855:1; parent_transcript=GRMZM2G130544_T02; parent_gene=GRMZM2G130544	GRMZM2G130544_P02	TRUE	TRUE	aGELTPDELER	95%	n+304 (+304)	40.45	25.00
4939	seq=translation; coord=10:17606896..17610628:1; parent_transcript=GRMZM2G009448_T01; parent_gene=GRMZM2G009448	GRMZM2G009448_P01	TRUE	TRUE	gFGFITYTSSEEASAAITAMDGk	95%	n+304 (+304), K+304 (+304)	37.57	25.00
4940	seq=translation; coord=10:17606896..17610628:1; parent_transcript=GRMZM2G009448_T01; parent_gene=GRMZM2G009448	GRMZM2G009448_P01	TRUE	TRUE	yGQVIEAk	95%	n+304 (+304), K+304 (+304)	35.30	26.68
4941	seq=translation; coord=1:213095235..213096735:1; parent_transcript=GRMZM2G161274_T02; parent_gene=GRMZM2G161274	GRMZM2G161274_P02	TRUE	TRUE	fWGHEWEk	95%	n+304 (+304), K+304 (+304)	35.06	25.00
4942	seq=translation; coord=1:213095235..213096735:1; parent_transcript=GRMZM2G161274_T02; parent_gene=GRMZM2G161274	GRMZM2G161274_P02	TRUE	TRUE	vSDLLSSLR	94%	n+304 (+304)	30.27	25.00
4943	seq=translation; coord=2:49721009..49733282:-1; parent_transcript=GRMZM2G120517_T02; parent_gene=GRMZM2G120517	GRMZM2G120517_P02,GRMZM2G120517_P03	TRUE	TRUE	dQDLLVLPTDAALFEDPk	91%	n+304 (+304), K+304 (+304)	27.49	26.64
4944	seq=translation; coord=2:73288608..73291178:1; parent_transcript=GRMZM2G016928_T01; parent_gene=GRMZM2G016928	GRMZM2G016928_P01,GRMZM2G016928_P02, GRMZM2G088880_P01,GRMZM2G088880_P02, GRMZM2G088880_P03,GRMZM2G303374_P01, GRMZM2G303374_P03,GRMZM2G303374_P04, GRMZM2G303374_P05	TRUE	TRUE	aGGAYTMNTASAVTVR	95%	n+304 (+304)	51.55	25.00
4945	seq=translation; coord=10:128106169..128112118:-1; parent_transcript=GRMZM2G098577_T01; parent_gene=GRMZM2G098577	GRMZM2G098577_P01	TRUE	TRUE	dVINLIFEk	94%	n+304 (+304), K+304 (+304)	27.01	25.00
4946	seq=translation; coord=10:128106169..128112118:-1; parent_transcript=GRMZM2G098577_T01; parent_gene=GRMZM2G098577	GRMZM2G098577_P01	TRUE	TRUE	eLVDVSEDILR	95%	n+304 (+304)	44.86	25.91
4947	seq=translation; coord=10:128106169..128112118:-1; parent_transcript=GRMZM2G098577_T01; parent_gene=GRMZM2G098577	GRMZM2G098577_P01	TRUE	TRUE	sAQPPSANEEk	95%	n+304 (+304), K+304 (+304)	32.63	25.19

4948	seq=translation; coord=1:289108489..289111192:-1; parent_transcript=GRMZM2G053023_T01; parent_gene=GRMZM2G053023	GRMZM2G053023_P01,GRMZM2G053023_P02	TRUE	TRUE	vDVDELAEVAR	95%	n+304 (+304)	40.95	26.02
4949	seq=translation; coord=1:289108489..289111192:-1; parent_transcript=GRMZM2G053023_T01; parent_gene=GRMZM2G053023	GRMZM2G053023_P01,GRMZM2G053023_P02	TRUE	TRUE	vEAMPTFVLVk	95%	n+304 (+304), K+304 (+304)	69.88	25.00
4950	seq=translation; coord=9:1634017..1638812:-1; parent_transcript=GRMZM2G143862_T01; parent_gene=GRMZM2G143862	GRMZM2G143862_P01	TRUE	TRUE	eLFEFLPk	95%	n+304 (+304), K+304 (+304)	32.36	25.00
4951	seq=translation; coord=9:1634017..1638812:-1; parent_transcript=GRMZM2G143862_T01; parent_gene=GRMZM2G143862	GRMZM2G143862_P01	TRUE	TRUE	tIQEQLMLER	95%	n+304 (+304)	36.11	26.16
4952	seq=translation; coord=9:126016122..126022567:1; parent_transcript=GRMZM2G170843_T01; parent_gene=GRMZM2G170843	GRMZM2G170843_P01,GRMZM2G170843_P02	TRUE	TRUE	eVLSGScGVPAPQQR	95%	n+304 (+304), Carbamidomethyl (+57)	47.23	25.00
4953	seq=translation; coord=9:126016122..126022567:1; parent_transcript=GRMZM2G170843_T01; parent_gene=GRMZM2G170843	GRMZM2G170843_P01,GRMZM2G170843_P02	TRUE	TRUE	fTVQTDLGATVGAFk	95%	n+304 (+304), K+304 (+304)	55.37	25.38
4954	seq=translation; coord=5:70685080..70691262:-1; parent_transcript=GRMZM2G120578_T01; parent_gene=GRMZM2G120578	GRMZM2G120578_P01,GRMZM2G120578_P02	TRUE	TRUE	dGPPVNFLTPVNPIEEVEGATk	95%	n+304 (+304), K+304 (+304)	48.05	25.68
4955	seq=translation; coord=5:70685080..70691262:-1; parent_transcript=GRMZM2G120578_T01; parent_gene=GRMZM2G120578	GRMZM2G120578_P01,GRMZM2G120578_P02	TRUE	TRUE	sPYQEVLVFESSTYGk	95%	n+304 (+304), K+304 (+304)	30.24	25.60
4956	seq=translation; coord=5:70685080..70691262:-1; parent_transcript=GRMZM2G120578_T01; parent_gene=GRMZM2G120578	GRMZM2G120578_P01,GRMZM2G120578_P02	TRUE	TRUE	vQLHVGDAVEFLR	95%	n+304 (+304)	29.50	25.67
4957	seq=translation; coord=1:190997916..191004927:1; parent_transcript=GRMZM2G084149_T01; parent_gene=GRMZM2G084149	GRMZM2G084149_P01	TRUE	TRUE	aITSMLDEIASLQAk	95%	n+304 (+304), K+304 (+304)	49.74	25.63
4958	seq=translation; coord=1:190997916..191004927:1; parent_transcript=GRMZM2G084149_T01; parent_gene=GRMZM2G084149	GRMZM2G084149_P01	TRUE	TRUE	iSALESDIEER	95%	n+304 (+304)	49.21	25.00
4959	seq=translation; coord=5:83557023..83560095:1; parent_transcript=AC234515.1_FGT003; parent_gene=AC234515.1_FG003	AC234515.1_FGP003	TRUE	TRUE	lATPTFGDLNHLISATMSGVTccLR	95%	n+304 (+304), Carbamidomethyl (+57), Carbamidomethyl (+57)	33.76	25.00
4960	seq=translation; coord=8:79074521..79075661:-1; parent_transcript=GRMZM2G125893_T01; parent_gene=GRMZM2G125893	GRMZM2G125893_P01	TRUE	TRUE	gDLAIVVGR	91%	n+304 (+304)	31.32	27.97
4961	seq=translation; coord=8:79074521..79075661:-1; parent_transcript=GRMZM2G125893_T01; parent_gene=GRMZM2G125893	GRMZM2G125893_P01	TRUE	TRUE	wIYGVN	87%	n+304 (+304)	26.73	25.90
4962	seq=translation; coord=5:4038554..4040410:1; parent_transcript=GRMZM2G121221_T01; parent_gene=GRMZM2G121221	GRMZM2G121221_P01,GRMZM2G121221_P02, GRMZM2G121221_P03	TRUE	TRUE	lLNVQLk	93%	n+304 (+304), K+304 (+304)	29.69	25.00
4963	seq=translation; coord=2:19443026..19444255:1; parent_transcript=GRMZM2G132777_T01; parent_gene=GRMZM2G132777	GRMZM2G132777_P01	TRUE	TRUE	lLLLTDVSGILADR	93%	n+304 (+304)	29.37	25.00
4964	seq=translation; coord=2:19443026..19444255:1; parent_transcript=GRMZM2G132777_T01; parent_gene=GRMZM2G132777	GRMZM2G132777_P01	TRUE	TRUE	vTDALTMEVVEMVLVGk	95%	n+304 (+304), K+304 (+304)	42.55	25.20
4965	seq=translation; coord=3:224922109..224926422:1; parent_transcript=GRMZM2G064753_T01; parent_gene=GRMZM2G064753	GRMZM2G064753_P01	TRUE	TRUE	eELEAELk	94%	n+304 (+304), K+304 (+304)	30.50	27.08

4966	seq=translation; coord=3:224922109..224926422:1; parent_transcript=GRMZM2G064753_T01; parent_gene=GRMZM2G064753	GRMZM2G064753_P01	TRUE	TRUE	eEPSVVESYYNk	95%	n+304 (+304), K+304 (+304)	58.02	25.00
4967	seq=translation; coord=3:224922109..224926422:1; parent_transcript=GRMZM2G064753_T01; parent_gene=GRMZM2G064753	GRMZM2G064753_P01	TRUE	TRUE	fDMDPLVGPFGTk	92%	n+304 (+304), K+304 (+304)	27.37	25.93
4968	seq=translation; coord=4:1494566..1495635:-1; parent_transcript=GRMZM2G107286_T01; parent_gene=GRMZM2G107286	GRMZM2G107286_P01	TRUE	TRUE	gNFLVGVGVIR	95%	n+304 (+304)	48.62	25.00
4969	seq=translation; coord=2:23473629..23477257:1; parent_transcript=GRMZM2G177937_T01; parent_gene=GRMZM2G177937	GRMZM2G177937_P01,GRMZM2G177937_P03	TRUE	TRUE	lNTDESPDIASQYGVR	95%	n+304 (+304)	78.54	25.00
4970	seq=translation; coord=1:50087976..50089098:1; parent_transcript=GRMZM2G394203_T01; parent_gene=GRMZM2G394203	GRMZM2G394203_P01	TRUE	TRUE	gGGFGGGAGGGAGGGLGGGGGLGGGEGGGYGGGAGR	95%	n+304 (+304)	59.35	25.00
4971	seq=translation; coord=1:50087976..50089098:1; parent_transcript=GRMZM2G394203_T01; parent_gene=GRMZM2G394203	GRMZM2G394203_P01	TRUE	TRUE	gGGFGGGAGGGAGGGLGGGGGLGGGEGGGyGGGAGR	95%	n+304 (+304), iTRAQ8plex (+304)	85.75	25.00
4972	seq=translation; coord=7:104173693..104176328:-1; parent_transcript=GRMZM2G032160_T01; parent_gene=GRMZM2G032160	GRMZM2G032160_P01	TRUE	TRUE	aDVAGLASTAITTTSSYFYGk	95%	n+304 (+304), K+304 (+304)	38.57	25.79
4973	seq=translation; coord=7:104173693..104176328:-1; parent_transcript=GRMZM2G032160_T01; parent_gene=GRMZM2G032160	GRMZM2G032160_P01	TRUE	TRUE	aTSTVVPDPSR	92%	n+304 (+304)	29.66	25.53
4974	seq=translation; coord=7:104173693..104176328:-1; parent_transcript=GRMZM2G032160_T01; parent_gene=GRMZM2G032160	GRMZM2G032160_P01	TRUE	TRUE	dGPGELLMLAHPLHLR	95%	n+304 (+304)	42.57	26.06
4975	seq=translation; coord=7:104173693..104176328:-1; parent_transcript=GRMZM2G032160_T01; parent_gene=GRMZM2G032160	GRMZM2G032160_P01	TRUE	TRUE	vAYLPDASMEPVLDR	95%	n+304 (+304)	36.22	25.00
4976	seq=translation; coord=7:104173693..104176328:-1; parent_transcript=GRMZM2G032160_T01; parent_gene=GRMZM2G032160	GRMZM2G032160_P01	TRUE	TRUE	wGGLVTLQGLk	89%	n+304 (+304), K+304 (+304)	25.01	25.00
4977	seq=translation; coord=2:6820716..6853179:1; parent_transcript=GRMZM2G138782_T01; parent_gene=GRMZM2G138782	GRMZM2G138782_P01	TRUE	TRUE	dLTIAGAVEQk	95%	n+304 (+304), K+304 (+304)	33.89	25.71
4978	seq=translation; coord=2:6820716..6853179:1; parent_transcript=GRMZM2G138782_T01; parent_gene=GRMZM2G138782	GRMZM2G138782_P01	TRUE	TRUE	eLMYAEEAAILDk	95%	n+304 (+304), K+304 (+304)	43.87	25.40
4979	seq=translation; coord=2:6820716..6853179:1; parent_transcript=GRMZM2G138782_T01; parent_gene=GRMZM2G138782	GRMZM2G138782_P01	TRUE	TRUE	iVLDIIEAIHAAEk	95%	n+304 (+304), K+304 (+304)	44.74	25.00
4980	seq=translation; coord=2:6820716..6853179:1; parent_transcript=GRMZM2G138782_T01; parent_gene=GRMZM2G138782	GRMZM2G138782_P01	TRUE	TRUE	sLAETYLLQDEPGLSk	95%	n+304 (+304), K+304 (+304)	42.00	27.33
4981	seq=translation; coord=2:24834408..24836108:-1; parent_transcript=GRMZM2G108153_T01; parent_gene=GRMZM2G108153	GRMZM2G108153_P01	TRUE	TRUE	dAVSLSGGPSFTVPLGR	95%	n+304 (+304)	63.22	25.79
4982	seq=translation; coord=2:24834408..24836108:-1; parent_transcript=GRMZM2G108153_T01; parent_gene=GRMZM2G108153	GRMZM2G108153_P01	TRUE	TRUE	gLPPNVGLQQEAVQLVEDIR	95%	n+304 (+304)	81.01	25.75
4983	seq=translation; coord=2:24834408..24836108:-1; parent_transcript=GRMZM2G108153_T01; parent_gene=GRMZM2G108153	GRMZM2G108153_P01	TRUE	TRUE	gVMLPSDQGLVSDPR	95%	n+304 (+304)	34.62	25.17

4984	seq=translation; coord=2:24834408..24836108:-1; parent_transcript=GRMZM2G108153_T01; parent_gene=GRMZM2G108153	GRMZM2G108153_P01	TRUE	TRUE	vHAAcGPTVScADITVLATR	95%	n+304 (+304), Carbamidomethyl (+57), Carbamidomethyl (+57)	47.29	25.00
4985	seq=translation; coord=8:125902255..125906710:1; parent_transcript=GRMZM2G059693_T01; parent_gene=GRMZM2G059693	GRMZM2G059693_P01,GRMZM2G059693_P02, GRMZM2G059693_P04	TRUE	TRUE	aTGLLVPGIASHSPR	95%	n+304 (+304)	39.46	25.17
4986	seq=translation; coord=8:125902255..125906710:1; parent_transcript=GRMZM2G059693_T01; parent_gene=GRMZM2G059693	GRMZM2G059693_P01,GRMZM2G059693_P02, GRMZM2G059693_P04	TRUE	TRUE	eGAVSLLYR	95%	n+304 (+304)	38.62	26.68
4987	seq=translation; coord=8:125902255..125906710:1; parent_transcript=GRMZM2G059693_T01; parent_gene=GRMZM2G059693	GRMZM2G059693_P01,GRMZM2G059693_P02, GRMZM2G059693_P04	TRUE	TRUE	gAFLWAk	95%	n+304 (+304), K+304 (+304)	38.37	25.56
4988	seq=translation; coord=7:151190045..151192101:1; parent_transcript=GRMZM2G155911_T01; parent_gene=GRMZM2G155911	GRMZM2G155911_P01	TRUE	TRUE	aPTEQLAADLDAHIR	95%	n+304 (+304)	74.18	25.74
4989	seq=translation; coord=7:151190045..151192101:1; parent_transcript=GRMZM2G155911_T01; parent_gene=GRMZM2G155911	GRMZM2G155911_P01	TRUE	TRUE	lPAVQPPTDFHGIEEFISR	95%	n+304 (+304)	34.06	25.80
4990	seq=translation; coord=7:151190045..151192101:1; parent_transcript=GRMZM2G155911_T01; parent_gene=GRMZM2G155911	GRMZM2G155911_P01	TRUE	TRUE	nAVEEGGSSYSSLR	95%	n+304 (+304)	32.85	25.00
4991	seq=translation; coord=7:129358205..129362749:-1; parent_transcript=GRMZM5G843748_T02; parent_gene=GRMZM5G843748	GRMZM5G843748_P02	TRUE	TRUE	aFIEGMIk	91%	n+304 (+304), K+304 (+304)	27.44	26.70
4992	seq=translation; coord=7:129358205..129362749:-1; parent_transcript=GRMZM5G843748_T02; parent_gene=GRMZM5G843748	GRMZM5G843748_P02	TRUE	TRUE	dQDAFFR	86%	n+304 (+304)	25.16	25.00
4993	seq=translation; coord=7:129358205..129362749:-1; parent_transcript=GRMZM5G843748_T02; parent_gene=GRMZM5G843748	GRMZM5G843748_P02	TRUE	TRUE	sFEVVDEIkDALEER	95%	n+304 (+304), K+304 (+304)	34.15	25.00
4994	seq=translation; coord=7:129358205..129362749:-1; parent_transcript=GRMZM5G843748_T02; parent_gene=GRMZM5G843748	GRMZM5G843748_P02	TRUE	TRUE	vFDNQYFEDLVALR	95%	n+304 (+304)	68.58	25.00
4995	seq=translation; coord=5:77916981..77922073:1; parent_transcript=GRMZM2G097207_T01; parent_gene=GRMZM2G097207	GRMZM2G097207_P01	TRUE	TRUE	kSGLEVMVGIPNDLLSTMATSMk	95%	K+304 (+304), n+304 (+304), K+304 (+304)	32.04	25.00
4996	seq=translation; coord=5:77916981..77922073:1; parent_transcript=GRMZM2G097207_T01; parent_gene=GRMZM2G097207	GRMZM2G097207_P01	TRUE	TRUE	lFDAEEGTMSALR	95%	n+304 (+304)	48.71	25.00
4997	seq=translation; coord=5:77916981..77922073:1; parent_transcript=GRMZM2G097207_T01; parent_gene=GRMZM2G097207	GRMZM2G097207_P01	TRUE	TRUE	sGLEVMVGIPNDLLSTMATSMk	95%	n+304 (+304), K+304 (+304)	49.15	25.13
4998	seq=translation; coord=10:86984445..86986672:1; parent_transcript=GRMZM2G028104_T01; parent_gene=GRMZM2G028104	GRMZM2G028104_P01	TRUE	TRUE	aSGEVAAADVVDVVR	95%	n+304 (+304)	61.71	25.79
4999	seq=translation; coord=10:86984445..86986672:1; parent_transcript=GRMZM2G028104_T01; parent_gene=GRMZM2G028104	GRMZM2G028104_P01	TRUE	TRUE	fVVVDAEGGGETR	95%	n+304 (+304)	46.82	25.00
5000	seq=translation; coord=10:86984445..86986672:1; parent_transcript=GRMZM2G028104_T01; parent_gene=GRMZM2G028104	GRMZM2G028104_P01	TRUE	TRUE	lDLLDLR	87%	n+304 (+304)	26.51	25.77
5001	seq=translation; coord=7:137518004..137519265:-1; parent_transcript=GRMZM2G066202_T01; parent_gene=GRMZM2G066202	GRMZM2G066202_P01	TRUE	TRUE	aAELAADESGGGEVR	95%	n+304 (+304)	36.60	25.00

5002	seq=translation; coord=7:137518004..137519265:-1; parent_transcript=GRMZM2G066202_T01; parent_gene=GRMZM2G066202	GRMZM2G066202_P01	TRUE	TRUE	gAQALLALk	95%	n+304 (+304), K+304 (+304)	37.47	25.00
5003	seq=translation; coord=9:41048627..41058528:-1; parent_transcript=GRMZM2G538535_T01; parent_gene=GRMZM2G538535	GRMZM2G538535_P01	TRUE	TRUE	aPAFSILDTcFEGQASQLR	95%	n+304 (+304), Carbamidomethyl (+57)	53.35	25.00
5004	seq=translation; coord=9:41048627..41058528:-1; parent_transcript=GRMZM2G538535_T01; parent_gene=GRMZM2G538535	GRMZM2G538535_P01	TRUE	TRUE	sAGLIGLAR	88%	n+304 (+304)	26.24	25.21
5005	seq=translation; coord=3:212009199..212010553:-1; parent_transcript=GRMZM2G453805_T01; parent_gene=GRMZM2G453805	GRMZM2G453805_P01	TRUE	TRUE	gIGNYGGIMVWDR	95%	n+304 (+304)	57.24	25.00
5006	seq=translation; coord=1:232041908..232053896:-1; parent_transcript=GRMZM2G148323_T01; parent_gene=GRMZM2G148323	GRMZM2G148323_P01	TRUE	TRUE	dAAALDPELLQLAELAPGALR	95%	n+304 (+304)	48.06	25.00
5007	seq=translation; coord=1:232041908..232053896:-1; parent_transcript=GRMZM2G148323_T01; parent_gene=GRMZM2G148323	GRMZM2G148323_P01	TRUE	TRUE	eVIPQFYFk	95%	n+304 (+304), K+304 (+304)	27.95	25.22
5008	seq=translation; coord=2:2338643..2346346:-1; parent_transcript=GRMZM2G077744_T01; parent_gene=GRMZM2G077744	GRMZM2G077744_P01,GRMZM2G077744_P02, GRMZM2G167262_P01,GRMZM2G167262_P02	TRUE	TRUE	acPDPTcIEIADccSHLk	95%	n+304 (+304), Carbamidomethyl (+57), Carbamidomethyl (+57), Carbamidomethyl (+57), Carbamidomethyl (+57), K+304 (+304)	54.92	25.00
5009	seq=translation; coord=2:2338643..2346346:-1; parent_transcript=GRMZM2G077744_T01; parent_gene=GRMZM2G077744	GRMZM2G077744_P01,GRMZM2G077744_P02, GRMZM2G167262_P01,GRMZM2G167262_P02	TRUE	TRUE	iPHAIELDk	95%	n+304 (+304), K+304 (+304)	42.80	25.00
5010	seq=translation; coord=2:2338643..2346346:-1; parent_transcript=GRMZM2G077744_T01; parent_gene=GRMZM2G077744	GRMZM2G077744_P01,GRMZM2G077744_P02, GRMZM2G167262_P01,GRMZM2G167262_P02	TRUE	TRUE	qLMIQVAELVPk	95%	n+304 (+304), K+304 (+304)	40.30	25.00
5011	seq=translation; coord=2:196218998..196222283:1; parent_transcript=GRMZM2G042604_T01; parent_gene=GRMZM2G042604	GRMZM2G042604_P01,GRMZM2G138128_P01, GRMZM2G138128_P02,GRMZM2G138128_P03	TRUE	TRUE	aLLEVVESGGk	95%	n+304 (+304), K+304 (+304)	47.46	25.53
5012	seq=translation; coord=2:196218998..196222283:1; parent_transcript=GRMZM2G042604_T01; parent_gene=GRMZM2G042604	GRMZM2G042604_P01,GRMZM2G138128_P01, GRMZM2G138128_P02,GRMZM2G138128_P03	TRUE	TRUE	iAcLDTHIALAcAGLk	94%	n+304 (+304), Carbamidomethyl (+57), Carbamidomethyl (+57), K+304 (+304)	26.37	25.53
5013	seq=translation; coord=2:196218998..196222283:1; parent_transcript=GRMZM2G042604_T01; parent_gene=GRMZM2G042604	GRMZM2G042604_P01,GRMZM2G138128_P01, GRMZM2G138128_P02,GRMZM2G138128_P03	TRUE	TRUE	lTVEDPVTVEYITR	95%	n+304 (+304)	42.24	25.49
5014	seq=translation; coord=8:18214060..18227316:1; parent_transcript=GRMZM2G107696_T01; parent_gene=GRMZM2G107696	GRMZM2G107696_P01,GRMZM2G107696_P02	TRUE	TRUE	aAINTSNTGVEEAMNWLLSHMDDPDINDPISk	95%	n+304 (+304), K+304 (+304)	37.42	25.00
5015	seq=translation; coord=8:18214060..18227316:1; parent_transcript=GRMZM2G107696_T01; parent_gene=GRMZM2G107696	GRMZM2G107696_P01,GRMZM2G107696_P02	TRUE	TRUE	eLDANTNYDWNR	95%	n+304 (+304)	31.73	25.00
5016	seq=translation; coord=8:18214060..18227316:1; parent_transcript=GRMZM2G107696_T01; parent_gene=GRMZM2G107696	GRMZM2G107696_P01,GRMZM2G107696_P02	TRUE	TRUE	eLNPFSGFk	95%	n+304 (+304), K+304 (+304)	32.43	26.48
5017	seq=translation; coord=8:18214060..18227316:1; parent_transcript=GRMZM2G107696_T01; parent_gene=GRMZM2G107696	GRMZM2G107696_P01,GRMZM2G107696_P02	TRUE	TRUE	qQDVLEFFLHLIDR	95%	n+304 (+304)	45.38	26.14

5018	seq=translation; coord=9:115668526..115674221:1; parent_transcript=GRMZM5G818887_T01; parent_gene=GRMZM5G818887	GRMZM5G818887_P01	TRUE	TRUE	gVLSVPEFILAGDNLVAk	95%	n+304 (+304), K+304 (+304)	36.15	25.00
5019	seq=translation; coord=9:115668526..115674221:1; parent_transcript=GRMZM5G818887_T01; parent_gene=GRMZM5G818887	GRMZM5G818887_P01	TRUE	TRUE	mPLMPELVFEDISQDHAR	95%	n+304 (+304)	48.05	25.00
5020	seq=translation; coord=9:115668526..115674221:1; parent_transcript=GRMZM5G818887_T01; parent_gene=GRMZM5G818887	GRMZM5G818887_P01	TRUE	TRUE	tEEEEDIPSMDTLDIGR	95%	n+304 (+304)	31.61	25.00
5021	seq=translation; coord=1:248590498..248595499:-1; parent_transcript=GRMZM5G830403_T02; parent_gene=GRMZM5G830403	GRMZM5G830403_P02,GRMZM5G830403_P03, GRMZM5G830403_P04	TRUE	TRUE	ePAANQDADDQNk	95%	n+304 (+304), K+304 (+304)	42.62	25.00
5022	seq=translation; coord=1:248590498..248595499:-1; parent_transcript=GRMZM5G830403_T02; parent_gene=GRMZM5G830403	GRMZM5G830403_P02,GRMZM5G830403_P03, GRMZM5G830403_P04	TRUE	TRUE	lVEVIEk	92%	n+304 (+304), K+304 (+304)	28.69	25.00
5023	seq=translation; coord=7:155961862..155967544:-1; parent_transcript=GRMZM2G173910_T01; parent_gene=GRMZM2G173910	GRMZM2G173910_P01	TRUE	TRUE	aPYNIQINVk	91%	n+304 (+304), K+304 (+304)	25.98	25.05
5024	seq=translation; coord=7:155961862..155967544:-1; parent_transcript=GRMZM2G173910_T01; parent_gene=GRMZM2G173910	GRMZM2G173910_P01	TRUE	TRUE	dFFVEEQPk	89%	n+304 (+304), K+304 (+304)	27.80	25.88
5025	seq=translation; coord=7:155961862..155967544:-1; parent_transcript=GRMZM2G173910_T01; parent_gene=GRMZM2G173910	GRMZM2G173910_P01	TRUE	TRUE	vNDAAAMAVVR	95%	n+304 (+304)	34.36	26.47
5026	seq=translation; coord=5:15703665..15718967:1; parent_transcript=AC197122.3_FGT003; parent_gene=AC197122.3_FG003	AC197122.3_FGP003	TRUE	TRUE	dFLDDYVER	95%	n+304 (+304)	32.43	25.00
5027	seq=translation; coord=5:15703665..15718967:1; parent_transcript=AC197122.3_FGT003; parent_gene=AC197122.3_FG003	AC197122.3_FGP003	TRUE	TRUE	vEALEDMVSLAPSSLVk	95%	n+304 (+304), K+304 (+304)	47.35	25.00
5028	seq=translation; coord=6:117850410..117855773:-1; parent_transcript=GRMZM2G074454_T01; parent_gene=GRMZM2G074454	GRMZM2G074454_P01	TRUE	TRUE	iGFIGLGAMGFGMASHLLk	95%	n+304 (+304), K+304 (+304)	33.45	25.00
5029	seq=translation; coord=6:117850410..117855773:-1; parent_transcript=GRMZM2G074454_T01; parent_gene=GRMZM2G074454	GRMZM2G074454_P01	TRUE	TRUE	lLSEDPLLIDFLk	95%	n+304 (+304), K+304 (+304)	40.17	25.00
5030	seq=translation; coord=8:83396432..83400437:1; parent_transcript=GRMZM2G131249_T02; parent_gene=GRMZM2G131249	GRMZM2G131249_P02	TRUE	TRUE	gFDPETGTVk	95%	n+304 (+304), K+304 (+304)	33.09	26.02
5031	seq=translation; coord=8:83396432..83400437:1; parent_transcript=GRMZM2G131249_T02; parent_gene=GRMZM2G131249	GRMZM2G131249_P02	TRUE	TRUE	sGIENMLMHYVPEVk	90%	n+304 (+304), K+304 (+304)	27.55	26.90
5032	seq=translation; coord=8:83396432..83400437:1; parent_transcript=GRMZM2G131249_T02; parent_gene=GRMZM2G131249	GRMZM2G131249_P02	TRUE	TRUE	sLFAIDGVTR	93%	n+304 (+304)	29.54	25.12
5033	seq=translation; coord=8:83396432..83400437:1; parent_transcript=GRMZM2G131249_T02; parent_gene=GRMZM2G131249	GRMZM2G131249_P02	TRUE	TRUE	vFFGSDFVTVTk	95%	n+304 (+304), K+304 (+304)	42.35	26.15
5034	seq=translation; coord=6:167006647..167019258:1; parent_transcript=AC233870.1_FGT006; parent_gene=AC233870.1_FG006	AC233870.1_FGP006	TRUE	TRUE	aGNLVESMTADLAGEVTSAAGPVR	95%	n+304 (+304)	34.99	25.00
5035	seq=translation; coord=6:167006647..167019258:1; parent_transcript=AC233870.1_FGT006; parent_gene=AC233870.1_FG006	AC233870.1_FGP006	TRUE	TRUE	sLDAEADLR	94%	n+304 (+304)	31.79	25.42

5036	seq=translation; coord=8:3347680..3350552:1; parent_transcript=GRMZM2G013461_T01; parent_gene=GRMZM2G013461	GRMZM2G013461_P01,GRMZM2G013461_P02	TRUE	TRUE	eNALLEFVR	94%	n+304 (+304)	31.04	25.54
5037	seq=translation; coord=8:3347680..3350552:1; parent_transcript=GRMZM2G013461_T01; parent_gene=GRMZM2G013461	GRMZM2G013461_P01,GRMZM2G013461_P02	TRUE	TRUE	eQVVAGTLHHLTLEAVEAGR	95%	n+304 (+304)	50.54	25.62
5038	seq=translation; coord=8:3347680..3350552:1; parent_transcript=GRMZM2G013461_T01; parent_gene=GRMZM2G013461	GRMZM2G013461_P01,GRMZM2G013461_P02	TRUE	TRUE	sNSLFPYELLEILR	95%	n+304 (+304)	41.25	26.62
5039	seq=translation; coord=9:134504096..134514418:-1; parent_transcript=GRMZM2G051613_T01; parent_gene=GRMZM2G051613	GRMZM2G051613_P01,GRMZM2G051613_P02	TRUE	TRUE	eTTGIVGLEVAPNAR	95%	n+304 (+304)	76.26	26.02
5040	seq=translation; coord=9:134504096..134514418:-1; parent_transcript=GRMZM2G051613_T01; parent_gene=GRMZM2G051613	GRMZM2G051613_P01,GRMZM2G051613_P02	TRUE	TRUE	lQIcQEEDDWk	95%	n+304 (+304), Carbamidomethyl (+57), K+304 (+304)	28.93	25.00
5041	seq=translation; coord=2:232093952..232099171:1; parent_transcript=GRMZM2G035807_T01; parent_gene=GRMZM2G035807	GRMZM2G035807_P01,GRMZM2G092327_P01, GRMZM2G092327_P02,GRMZM2G092327_P03	TRUE	TRUE	lMDLLER	93%	n+304 (+304)	30.46	25.67
5042	seq=translation; coord=2:232093952..232099171:1; parent_transcript=GRMZM2G035807_T01; parent_gene=GRMZM2G035807	GRMZM2G035807_P01,GRMZM2G092327_P01, GRMZM2G092327_P02,GRMZM2G092327_P03	TRUE	TRUE	tAAFcFPIISGILk	95%	n+304 (+304), Carbamidomethyl (+57), K+304 (+304)	37.68	25.00
5043	seq=translation; coord=1:66948065..66952666:-1; parent_transcript=GRMZM2G152925_T01; parent_gene=GRMZM2G152925	GRMZM2G152925_P01	TRUE	TRUE	gEDAPEcDk	95%	n+304 (+304), Carbamidomethyl (+57), K+304 (+304)	31.87	25.00
5044	seq=translation; coord=1:66948065..66952666:-1; parent_transcript=GRMZM2G152925_T01; parent_gene=GRMZM2G152925	GRMZM2G152925_P01	TRUE	TRUE	sLcPGEWVDR	92%	n+304 (+304), Carbamidomethyl (+57)	29.10	25.00
5045	seq=translation; coord=8:143277512..143279395:-1; parent_transcript=GRMZM2G050072_T01; parent_gene=GRMZM2G050072	GRMZM2G050072_P01	TRUE	TRUE	aLVPYYPVAGR	86%	n+304 (+304)	25.27	25.00
5046	seq=translation; coord=8:143277512..143279395:-1; parent_transcript=GRMZM2G050072_T01; parent_gene=GRMZM2G050072	GRMZM2G050072_P01	TRUE	TRUE	dATGQTcSTFDAVTAVVFk	95%	n+304 (+304), Carbamidomethyl (+57), K+304 (+304)	34.77	25.00
5047	seq=translation; coord=8:143277512..143279395:-1; parent_transcript=GRMZM2G050072_T01; parent_gene=GRMZM2G050072	GRMZM2G050072_P01	TRUE	TRUE	eASLPEVVGVMR	95%	n+304 (+304)	33.90	26.10
5048	seq=translation; coord=8:143277512..143279395:-1; parent_transcript=GRMZM2G050072_T01; parent_gene=GRMZM2G050072	GRMZM2G050072_P01	TRUE	TRUE	fTDWMR	87%	n+304 (+304)	25.04	25.00
5049	seq=translation; coord=8:143277512..143279395:-1; parent_transcript=GRMZM2G050072_T01; parent_gene=GRMZM2G050072	GRMZM2G050072_P01	TRUE	TRUE	lEDLVLMAQVTk	95%	n+304 (+304), K+304 (+304)	38.07	25.00
5050	seq=translation; coord=10:25975417..25980229:-1; parent_transcript=GRMZM2G014376_T01; parent_gene=GRMZM2G014376	GRMZM2G014376_P01,GRMZM2G014376_P02, GRMZM2G014376_P03	TRUE	TRUE	eMAAELAAAAALGADVAELR	92%	n+304 (+304)	27.18	25.55
5051	seq=translation; coord=10:25975417..25980229:-1; parent_transcript=GRMZM2G014376_T01; parent_gene=GRMZM2G014376	GRMZM2G014376_P01,GRMZM2G014376_P02, GRMZM2G014376_P03	TRUE	TRUE	lFVVIGAGGAGk	95%	n+304 (+304), K+304 (+304)	48.27	25.00
5052	seq=translation; coord=10:25975417..25980229:-1; parent_transcript=GRMZM2G014376_T01; parent_gene=GRMZM2G014376	GRMZM2G014376_P01,GRMZM2G014376_P02, GRMZM2G014376_P03	TRUE	TRUE	sVGFNAVYVPFLVDDLAk	95%	n+304 (+304), K+304 (+304)	33.53	25.07
5053	seq=translation; coord=1:264743594..264751569:1; parent_transcript=GRMZM2G002687_T01; parent_gene=GRMZM2G002687	GRMZM2G002687_P01	TRUE	TRUE	aIGIFGFNLEDHIGk	95%	n+304 (+304), K+304 (+304)	27.76	26.03

5054	seq=translation; coord=1:264743594..264751569:1; parent_transcript=GRMZM2G002687_T01; parent_gene=GRMZM2G002687	GRMZM2G002687_P01	TRUE	TRUE	lIAVLSEIVAR	94%	n+304 (+304)	30.27	25.00
5055	seq=translation; coord=4:170517685..170521446:-1; parent_transcript=GRMZM2G150648_T03; parent_gene=GRMZM2G150648	GRMZM2G150648_P03,GRMZM2G150648_P04, GRMZM2G150648_P05	TRUE	TRUE	aDQFkPAEQVTLEPYER	93%	n+304 (+304), K+304 (+304)	28.09	25.62
5056	seq=translation; coord=4:170517685..170521446:-1; parent_transcript=GRMZM2G150648_T03; parent_gene=GRMZM2G150648	GRMZM2G150648_P03,GRMZM2G150648_P04, GRMZM2G150648_P05	TRUE	TRUE	mLVGMVDVIFSDVAQPDQAR	95%	n+304 (+304)	89.36	25.00
5057	seq=translation; coord=4:174901704..174907176:-1; parent_transcript=GRMZM2G039263_T01; parent_gene=GRMZM2G039263	GRMZM2G039263_P01,GRMZM2G039263_P02, GRMZM2G110626_P01	TRUE	TRUE	eQLAIAEFAESLLIIPk	95%	n+304 (+304), K+304 (+304)	33.58	25.00
5058	seq=translation; coord=4:174901704..174907176:-1; parent_transcript=GRMZM2G039263_T01; parent_gene=GRMZM2G039263	GRMZM2G039263_P01,GRMZM2G039263_P02, GRMZM2G110626_P01	TRUE	TRUE	eVGDGTTSVVIIAAELLk	91%	n+304 (+304), K+304 (+304)	25.37	25.00
5059	seq=translation; coord=4:174901704..174907176:-1; parent_transcript=GRMZM2G039263_T01; parent_gene=GRMZM2G039263	GRMZM2G039263_P01,GRMZM2G039263_P02, GRMZM2G110626_P01	TRUE	TRUE	iADDDVILVk	87%	n+304 (+304), K+304 (+304)	25.10	25.00
5060	seq=translation; coord=5:176387128..176391570:1; parent_transcript=GRMZM2G075637_T01; parent_gene=GRMZM2G075637	GRMZM2G075637_P01	TRUE	TRUE	rPLVVESFFSWFSDTELk	95%	n+304 (+304), K+304 (+304)	30.76	25.00
5061	seq=translation; coord=1:226171571..226174821:1; parent_transcript=GRMZM5G882228_T02; parent_gene=GRMZM5G882228	GRMZM5G882228_P02	TRUE	TRUE	eEPETEPLPPPVQMGEVSEEDLR	94%	n+304 (+304)	27.97	25.00
5062	seq=translation; coord=1:226171571..226174821:1; parent_transcript=GRMZM5G882228_T02; parent_gene=GRMZM5G882228	GRMZM5G882228_P02	TRUE	TRUE	gQIYDVTQSR	93%	n+304 (+304)	29.95	25.00
5063	seq=translation; coord=1:226171571..226174821:1; parent_transcript=GRMZM5G882228_T02; parent_gene=GRMZM5G882228	GRMZM5G882228_P02	TRUE	TRUE	mFYGPGGPYALFAGk	95%	n+304 (+304), K+304 (+304)	39.76	25.76
5064	seq=translation; coord=3:19453334..19462209:1; parent_transcript=GRMZM2G088834_T03; parent_gene=GRMZM2G088834	GRMZM2G088834_P03,GRMZM2G126860_P01	TRUE	TRUE	aFPLYMNALEYFk	95%	n+304 (+304), K+304 (+304)	53.15	26.79
5065	seq=translation; coord=3:19453334..19462209:1; parent_transcript=GRMZM2G088834_T03; parent_gene=GRMZM2G088834	GRMZM2G088834_P03,GRMZM2G126860_P01	TRUE	TRUE	eNAPSIIFIDEIDSLcGQR	95%	n+304 (+304), Carbamidomethyl (+57)	73.90	25.00
5066	seq=translation; coord=2:170263508..170267458:-1; parent_transcript=GRMZM2G074687_T01; parent_gene=GRMZM2G074687	GRMZM2G074687_P01	TRUE	TRUE	fGGDTTYSIMFGPDIcGYSTk	91%	n+304 (+304), Carbamidomethyl (+57), K+304 (+304)	26.05	25.00
5067	seq=translation; coord=2:170263508..170267458:-1; parent_transcript=GRMZM2G074687_T01; parent_gene=GRMZM2G074687	GRMZM2G074687_P01	TRUE	TRUE	lLGGDVDQk	94%	n+304 (+304), K+304 (+304)	30.24	27.29
5068	seq=translation; coord=1:94486908..94489534:1; parent_transcript=GRMZM2G052666_T01; parent_gene=GRMZM2G052666	GRMZM2G052666_P01	TRUE	TRUE	vQYDPAAck	95%	n+304 (+304), Carbamidomethyl (+57), K+304 (+304)	36.96	25.00
5069	seq=translation; coord=1:94486908..94489534:1; parent_transcript=GRMZM2G052666_T01; parent_gene=GRMZM2G052666	GRMZM2G052666_P01	TRUE	TRUE	yDDLLDTFWAR	95%	n+304 (+304)	37.05	25.00
5070	seq=translation; coord=3:47497754..47499644:-1; parent_transcript=GRMZM2G123407_T01; parent_gene=GRMZM2G123407	GRMZM2G123407_P01	TRUE	TRUE	aYFFSGNYDGEQcESGQR	95%	n+304 (+304), Carbamidomethyl (+57)	33.26	25.00
5071	seq=translation; coord=3:47497754..47499644:-1; parent_transcript=GRMZM2G123407_T01; parent_gene=GRMZM2G123407	GRMZM2G123407_P01	TRUE	TRUE	dAVTVAVPLLDEGR	95%	n+304 (+304)	65.63	26.37

5072	seq=translation; coord=1:277739114..277741150:1; parent_transcript=GRMZM2G090422_T01; parent_gene=GRMZM2G090422	GRMZM2G090422_P01	TRUE	TRUE	lGSQLEILAPIkY	95%	n+304 (+304), K+304 (+304)	63.58	25.00
5073	seq=translation; coord=1:277739114..277741150:1; parent_transcript=GRMZM2G090422_T01; parent_gene=GRMZM2G090422	GRMZM2G090422_P01	TRUE	TRUE	mVIPDALk	90%	n+304 (+304), K+304 (+304)	25.93	25.47
5074	seq=translation; coord=1:277739114..277741150:1; parent_transcript=GRMZM2G090422_T01; parent_gene=GRMZM2G090422	GRMZM2G090422_P01	TRUE	TRUE	ycLLGELSk	93%	n+304 (+304), Carbamidomethyl (+57), K+304 (+304)	28.26	26.02
5075	seq=translation; coord=2:208079590..208081944:1; parent_transcript=GRMZM2G034764_T01; parent_gene=GRMZM2G034764	GRMZM2G034764_P01	TRUE	TRUE	aASETADAEVDSSR	95%	n+304 (+304)	62.37	25.00
5076	seq=translation; coord=4:229500502..229510053:1; parent_transcript=GRMZM2G069631_T01; parent_gene=GRMZM2G069631	GRMZM2G069631_P01,GRMZM2G069631_P02, GRMZM2G165418_P01,GRMZM2G165418_P02, GRMZM2G165418_P03,GRMZM2G165418_P04	TRUE	TRUE	lIDLR	86%	n+304 (+304)	25.44	25.00
5077	seq=translation; coord=4:229500502..229510053:1; parent_transcript=GRMZM2G069631_T01; parent_gene=GRMZM2G069631	GRMZM2G069631_P01,GRMZM2G069631_P02, GRMZM2G165418_P01,GRMZM2G165418_P02, GRMZM2G165418_P03,GRMZM2G165418_P04	TRUE	TRUE	vVLFER	90%	n+304 (+304)	27.38	25.00
5078	seq=translation; coord=2:232371777..232375068:-1; parent_transcript=GRMZM2G008919_T01; parent_gene=GRMZM2G008919	GRMZM2G008919_P01,GRMZM2G008919_P02, GRMZM2G008919_P03,GRMZM2G008919_P05, GRMZM2G104505_P01	TRUE	TRUE	gVMMENIEk	95%	n+304 (+304), K+304 (+304)	40.85	25.30
5079	seq=translation; coord=2:30905783..30911404:-1; parent_transcript=GRMZM2G352129_T01; parent_gene=GRMZM2G352129	GRMZM2G352129_P01	TRUE	TRUE	dAALSPAAQIGTLTSALANANPEQQR	95%	n+304 (+304)	44.12	25.71
5080	seq=translation; coord=2:30905783..30911404:-1; parent_transcript=GRMZM2G352129_T01; parent_gene=GRMZM2G352129	GRMZM2G352129_P01	TRUE	TRUE	nLDDSIGDDQLR	95%	n+304 (+304)	45.42	25.00
5081	seq=translation; coord=9:44374790..44375756:-1; parent_transcript=GRMZM2G382673_T01; parent_gene=GRMZM2G382673	GRMZM2G382673_P01	TRUE	TRUE	dPAHPWR	95%	n+304 (+304)	44.76	25.00
5082	seq=translation; coord=1:84814341..84815071:1; parent_transcript=GRMZM2G402564_T01; parent_gene=GRMZM2G402564	GRMZM2G402564_P01	TRUE	TRUE	aNcScGAScNcAScASA	95%	n+304 (+304), Carbamidomethyl (+57), Carbamidomethyl (+57), Carbamidomethyl (+57), Carbamidomethyl (+57), Carbamidomethyl (+57)	45.23	25.00
5083	seq=translation; coord=1:84814341..84815071:1; parent_transcript=GRMZM2G402564_T01; parent_gene=GRMZM2G402564	GRMZM2G402564_P01	TRUE	TRUE	cGcAVPcPGGk	95%	Carbamidomethyl (+57), n+304 (+304), Carbamidomethyl (+57), Carbamidomethyl (+57), K+304 (+304)	31.00	25.00
5084	seq=translation; coord=1:84814341..84815071:1; parent_transcript=GRMZM2G402564_T01; parent_gene=GRMZM2G402564	GRMZM2G402564_P01	TRUE	TRUE	eHTTcGcGEHcEcSPcTcGR	92%	n+304 (+304), Carbamidomethyl (+57), Carbamidomethyl (+57), Carbamidomethyl (+57), Carbamidomethyl (+57), Carbamidomethyl (+57), Carbamidomethyl (+57)	26.12	25.00
5085	seq=translation; coord=1:248861739..248864579:-1; parent_transcript=GRMZM2G073725_T01; parent_gene=GRMZM2G073725	GRMZM2G073725_P01	TRUE	TRUE	lGTIDPYFVk	95%	n+304 (+304), K+304 (+304)	37.06	25.07

5086	seq=translation; coord=10:143716436..143719458:-1; parent_transcript=GRMZM2G023387_T01; parent_gene=GRMZM2G023387	GRMZM2G023387_P01	TRUE	TRUE	aMPTFFLIk	93%	n+304 (+304), K+304 (+304)	27.02	25.00
5087	seq=translation; coord=2:53844493..53846005:1; parent_transcript=GRMZM2G044132_T01; parent_gene=GRMZM2G044132	GRMZM2G044132_P01	TRUE	TRUE	eHLGEMGALAAGAFALYER	95%	n+304 (+304)	62.51	25.00
5088	seq=translation; coord=2:53844493..53846005:1; parent_transcript=GRMZM2G044132_T01; parent_gene=GRMZM2G044132	GRMZM2G044132_P01	TRUE	TRUE	gTGGGGYNk	95%	n+304 (+304), K+304 (+304)	38.05	25.24
5089	seq=translation; coord=2:53844493..53846005:1; parent_transcript=GRMZM2G044132_T01; parent_gene=GRMZM2G044132	GRMZM2G044132_P01	TRUE	TRUE	sGAGDDYDSGYNSk	88%	n+304 (+304), K+304 (+304)	26.22	25.00
5090	seq=translation; coord=2:53844493..53846005:1; parent_transcript=GRMZM2G044132_T01; parent_gene=GRMZM2G044132	GRMZM2G044132_P01	TRUE	TRUE	sGNDGYDSGYNR	90%	n+304 (+304)	27.39	25.00
5091	seq=translation; coord=5:183144788..183149469:1; parent_transcript=GRMZM2G007647_T01; parent_gene=GRMZM2G007647	GRMZM2G007647_P01	TRUE	TRUE	eILDIAEIFR	95%	n+304 (+304)	44.02	25.00
5092	seq=translation; coord=5:183144788..183149469:1; parent_transcript=GRMZM2G007647_T01; parent_gene=GRMZM2G007647	GRMZM2G007647_P01	TRUE	TRUE	iAGVFAR	89%	n+304 (+304)	26.14	25.00
5093	seq=translation; coord=5:183144788..183149469:1; parent_transcript=GRMZM2G007647_T01; parent_gene=GRMZM2G007647	GRMZM2G007647_P01	TRUE	TRUE	lIDVHEVHDITHSPFAER	95%	n+304 (+304)	38.24	25.53
5094	seq=translation; coord=5:183144788..183149469:1; parent_transcript=GRMZM2G007647_T01; parent_gene=GRMZM2G007647	GRMZM2G007647_P01	TRUE	TRUE	rEILDIAEIFR	95%	n+304 (+304)	38.81	25.15
5095	seq=translation; coord=5:6445850..6453287:-1; parent_transcript=GRMZM2G076885_T01; parent_gene=GRMZM2G076885	GRMZM2G076885_P01	TRUE	TRUE	aAPGVTIIDDR	95%	n+304 (+304)	36.82	25.90
5096	seq=translation; coord=5:6445850..6453287:-1; parent_transcript=GRMZM2G076885_T01; parent_gene=GRMZM2G076885	GRMZM2G076885_P01	TRUE	TRUE	eDGPSVAIVGATGAVGQEFLR	95%	n+304 (+304)	45.15	25.51
5097	seq=translation; coord=5:6445850..6453287:-1; parent_transcript=GRMZM2G076885_T01; parent_gene=GRMZM2G076885	GRMZM2G076885_P01	TRUE	TRUE	fPTPLEVSDkDDVAVGR	95%	n+304 (+304), K+304 (+304)	40.33	25.72
5098	seq=translation; coord=5:6445850..6453287:-1; parent_transcript=GRMZM2G076885_T01; parent_gene=GRMZM2G076885	GRMZM2G076885_P01	TRUE	TRUE	gAALNAVQIAEMLLk	95%	n+304 (+304), K+304 (+304)	37.00	25.00
5099	seq=translation; coord=8:144270470..144273473:-1; parent_transcript=GRMZM2G024315_T01; parent_gene=GRMZM2G024315	GRMZM2G024315_P01	TRUE	TRUE	eLGIGIVPYSPLGR	95%	n+304 (+304)	33.67	25.00
5100	seq=translation; coord=8:144270470..144273473:-1; parent_transcript=GRMZM2G024315_T01; parent_gene=GRMZM2G024315	GRMZM2G024315_P01	TRUE	TRUE	fAPENLEk	94%	n+304 (+304), K+304 (+304)	31.60	26.19
5101	seq=translation; coord=8:144270470..144273473:-1; parent_transcript=GRMZM2G024315_T01; parent_gene=GRMZM2G024315	GRMZM2G024315_P01	TRUE	TRUE	gVTLFDTSDVYGPLTNEILLGk	95%	n+304 (+304), K+304 (+304)	71.07	25.00
5102	seq=translation; coord=3:30996380..30997528:1; parent_transcript=AC226235.2_FGT001; parent_gene=AC226235.2_FG001	AC226235.2_FGP001	TRUE	TRUE	aVFVEEGDIATYTVLAADDPR	95%	n+304 (+304)	33.82	25.00
5103	seq=translation; coord=3:30996380..30997528:1; parent_transcript=AC226235.2_FGT001; parent_gene=AC226235.2_FG001	AC226235.2_FGP001	TRUE	TRUE	fFPSEFGLDVDR	95%	n+304 (+304)	54.56	25.00

5104	seq=translation; coord=3:30996380..30997528:1; parent_transcript=AC226235.2_FGT001; parent_gene=AC226235.2_FG001	AC226235.2_FGP001	TRUE	TRUE	gDLYDQASLVSAVk	95%	n+304 (+304), K+304 (+304)	34.86	25.84
5105	seq=translation; coord=5:65417067..65419686:1; parent_transcript=GRMZM2G112805_T01; parent_gene=GRMZM2G112805	GRMZM2G112805_P01	TRUE	TRUE	aISELIPHEIANIEk	94%	n+304 (+304), K+304 (+304)	26.24	25.00
5106	seq=translation; coord=5:65417067..65419686:1; parent_transcript=GRMZM2G112805_T01; parent_gene=GRMZM2G112805	GRMZM2G112805_P01	TRUE	TRUE	sQIIVDAEEFk	95%	n+304 (+304), K+304 (+304)	55.20	25.80
5107	seq=translation; coord=2:34740703..34741540:1; parent_transcript=AC155352.2_FGT010; parent_gene=AC155352.2_FG010	AC155352.2_FGP010,GRMZM2G379898_P01,GRMZM2G379898_P02	TRUE	TRUE	aNVLGINLNLPINLSLLVNycGR	95%	n+304 (+304), iTRAQ8plex (+304), Carbamidomethyl (+57)	34.01	25.00
5108	seq=translation; coord=2:34740703..34741540:1; parent_transcript=AC155352.2_FGT010; parent_gene=AC155352.2_FG010	AC155352.2_FGP010,GRMZM2G379898_P01,GRMZM2G379898_P02	TRUE	TRUE	cPVDALk	90%	Carbamidomethyl (+57), n+304 (+304), K+304 (+304)	25.66	25.00
5109	seq=translation; coord=10:4776409..4777145:-1; parent_transcript=GRMZM2G031033_T01; parent_gene=GRMZM2G031033	GRMZM2G031033_P01	TRUE	TRUE	eVVVGEVEVAk	95%	n+304 (+304), K+304 (+304)	28.71	25.62
5110	seq=translation; coord=10:4776409..4777145:-1; parent_transcript=GRMZM2G031033_T01; parent_gene=GRMZM2G031033	GRMZM2G031033_P01	TRUE	TRUE	vDVAPGLLQDQk	95%	n+304 (+304), K+304 (+304)	45.32	25.20
5111	seq=translation; coord=6:14560251..14563314:-1; parent_transcript=GRMZM2G076943_T01; parent_gene=GRMZM2G076943	GRMZM2G076943_P01	TRUE	TRUE	gATLYDDPcILR	95%	n+304 (+304), Carbamidomethyl (+57)	34.01	25.00
5112	seq=translation; coord=6:14560251..14563314:-1; parent_transcript=GRMZM2G076943_T01; parent_gene=GRMZM2G076943	GRMZM2G076943_P01	TRUE	TRUE	sIDSLLFDLPALR	95%	n+304 (+304)	42.53	26.03
5113	seq=translation; coord=4:1482217..1485577:-1; parent_transcript=GRMZM2G107082_T01; parent_gene=GRMZM2G107082	GRMZM2G107082_P01	TRUE	TRUE	vVLDcATADPDGR	95%	n+304 (+304), Carbamidomethyl (+57)	35.67	25.00
5114	seq=translation; coord=4:1482217..1485577:-1; parent_transcript=GRMZM2G107082_T01; parent_gene=GRMZM2G107082	GRMZM2G107082_P01	TRUE	TRUE	wGGIEFPLPFGR	95%	n+304 (+304)	33.06	25.19
5115	seq=translation; coord=2:233468750..233475308:1; parent_transcript=GRMZM2G117226_T01; parent_gene=GRMZM2G117226	GRMZM2G117226_P01,GRMZM2G117226_P02, GRMZM2G117226_P03	TRUE	TRUE	iTEVLk	91%	n+304 (+304), K+304 (+304)	25.15	25.00
5116	seq=translation; coord=5:181383017..181386545:1; parent_transcript=GRMZM2G029583_T02; parent_gene=GRMZM2G029583	GRMZM2G029583_P02,GRMZM2G029583_P03	TRUE	TRUE	iADAEENLGESEVR	95%	n+304 (+304)	67.08	25.00
5117	seq=translation; coord=5:181383017..181386545:1; parent_transcript=GRMZM2G029583_T02; parent_gene=GRMZM2G029583	GRMZM2G029583_P02,GRMZM2G029583_P03	TRUE	TRUE	sFFVAFSGLTEQIk	95%	n+304 (+304), K+304 (+304)	44.12	25.66
5118	seq=translation; coord=5:181383017..181386545:1; parent_transcript=GRMZM2G029583_T02; parent_gene=GRMZM2G029583	GRMZM2G029583_P02,GRMZM2G029583_P03	TRUE	TRUE	sLYFIR	86%	n+304 (+304)	25.07	25.00
5119	seq=translation; coord=5:181383017..181386545:1; parent_transcript=GRMZM2G029583_T02; parent_gene=GRMZM2G029583	GRMZM2G029583_P02,GRMZM2G029583_P03	TRUE	TRUE	vVDAPEILAVIGk	95%	n+304 (+304), K+304 (+304)	46.17	25.00
5120	seq=translation; coord=1:297509820..297513009:-1; parent_transcript=GRMZM5G898915_T01; parent_gene=GRMZM5G898915	GRMZM5G898915_P01	TRUE	TRUE	aTYSLPEATTLR	95%	n+304 (+304)	32.63	25.45
5121	seq=translation; coord=1:297509820..297513009:-1; parent_transcript=GRMZM5G898915_T01; parent_gene=GRMZM5G898915	GRMZM5G898915_P01	TRUE	TRUE	gMSPYFGcIVGR	95%	n+304 (+304), Carbamidomethyl (+57)	45.03	25.00

5122	seq=translation; coord=1:297509820..297513009:-1; parent_transcript=GRMZM5G898915_T01; parent_gene=GRMZM5G898915	GRMZM5G898915_P01	TRUE	TRUE	hITPVDENTIPTGEIMPVDGTPFDFTTER	95%	n+304 (+304)	35.14	25.00
5123	seq=translation; coord=1:297509820..297513009:-1; parent_transcript=GRMZM5G898915_T01; parent_gene=GRMZM5G898915	GRMZM5G898915_P01	TRUE	TRUE	vVWDVVEHk	94%	n+304 (+304), K+304 (+304)	28.27	25.35
5124	seq=translation; coord=4:26128851..26131595:1; parent_transcript=GRMZM2G024482_T02; parent_gene=GRMZM2G024482	GRMZM2G024482_P02	TRUE	TRUE	dNFFFAGIDk	94%	n+304 (+304), K+304 (+304)	28.30	25.15
5125	seq=translation; coord=4:26128851..26131595:1; parent_transcript=GRMZM2G024482_T02; parent_gene=GRMZM2G024482	GRMZM2G024482_P02	TRUE	TRUE	fPFLLVDR	94%	n+304 (+304)	31.28	25.00
5126	seq=translation; coord=4:26128851..26131595:1; parent_transcript=GRMZM2G024482_T02; parent_gene=GRMZM2G024482	GRMZM2G024482_P02	TRUE	TRUE	kPVIAGDTLIMR	95%	K+304 (+304), n+304 (+304)	32.13	25.00
5127	seq=translation; coord=4:26128851..26131595:1; parent_transcript=GRMZM2G024482_T02; parent_gene=GRMZM2G024482	GRMZM2G024482_P02	TRUE	TRUE	rFPPFPSVMDINQIR	95%	n+304 (+304)	65.26	26.25
5128	seq=translation; coord=2:82246568..82258715:1; parent_transcript=GRMZM2G125310_T01; parent_gene=GRMZM2G125310	GRMZM2G125310_P01,GRMZM2G125310_P02	TRUE	TRUE	aTLELTHNWGTENDPEFk	95%	n+304 (+304), K+304 (+304)	35.87	25.00
5129	seq=translation; coord=2:82246568..82258715:1; parent_transcript=GRMZM2G125310_T01; parent_gene=GRMZM2G125310	GRMZM2G125310_P01,GRMZM2G125310_P02	TRUE	TRUE	gFGHIGVTVDDVHk	95%	n+304 (+304), K+304 (+304)	27.69	25.72
5130	seq=translation; coord=2:82246568..82258715:1; parent_transcript=GRMZM2G125310_T01; parent_gene=GRMZM2G125310	GRMZM2G125310_P01,GRMZM2G125310_P02	TRUE	TRUE	gYFLQQTMLR	89%	n+304 (+304)	26.92	25.00
5131	seq=translation; coord=10:70222469..70228198:-1; parent_transcript=GRMZM2G103287_T01; parent_gene=GRMZM2G103287	GRMZM2G103287_P01,GRMZM2G103287_P02	TRUE	TRUE	aMQDFGDAEYk	95%	n+304 (+304), K+304 (+304)	32.49	25.00
5132	seq=translation; coord=10:70222469..70228198:-1; parent_transcript=GRMZM2G103287_T01; parent_gene=GRMZM2G103287	GRMZM2G103287_P01,GRMZM2G103287_P02	TRUE	TRUE	fTEQGDAIVFESEVDk	95%	n+304 (+304), K+304 (+304)	41.73	25.38
5133	seq=translation; coord=10:70222469..70228198:-1; parent_transcript=GRMZM2G103287_T01; parent_gene=GRMZM2G103287	GRMZM2G103287_P01,GRMZM2G103287_P02	TRUE	TRUE	iWPHSFEFR	91%	n+304 (+304)	25.84	25.00
5134	seq=translation; coord=10:70222469..70228198:-1; parent_transcript=GRMZM2G103287_T01; parent_gene=GRMZM2G103287	GRMZM2G103287_P01,GRMZM2G103287_P02	TRUE	TRUE	vALGPSGDLSLTSR	95%	n+304 (+304)	61.96	25.48
5135	seq=translation; coord=7:8520216..8522520:-1; parent_transcript=GRMZM2G024354_T01; parent_gene=GRMZM2G024354	GRMZM2G024354_P01	TRUE	TRUE	yFEIILVDVAHk	95%	n+304 (+304), K+304 (+304)	65.95	25.00
5136	seq=translation; coord=1:225492875..225495179:1; parent_transcript=GRMZM2G056431_T01; parent_gene=GRMZM2G056431	GRMZM2G056431_P01,GRMZM2G056431_P02	TRUE	TRUE	aIEAVADIYGIDSIAADLk	95%	n+304 (+304), K+304 (+304)	47.46	25.34
5137	seq=translation; coord=1:225492875..225495179:1; parent_transcript=GRMZM2G056431_T01; parent_gene=GRMZM2G056431	GRMZM2G056431_P01,GRMZM2G056431_P02	TRUE	TRUE	mTIIGEMDAVAIAk	95%	n+304 (+304), K+304 (+304)	66.27	26.29
5138	seq=translation; coord=1:225492875..225495179:1; parent_transcript=GRMZM2G056431_T01; parent_gene=GRMZM2G056431	GRMZM2G056431_P01,GRMZM2G056431_P02	TRUE	TRUE	vPTMTDDk	93%	n+304 (+304), K+304 (+304)	30.39	25.59
5139	seq=translation; coord=10:69423694..69433754:-1; parent_transcript=GRMZM2G145870_T01; parent_gene=GRMZM2G145870	GRMZM2G145870_P01,GRMZM2G145870_P02	TRUE	TRUE	aIVGLFGk	95%	n+304 (+304), K+304 (+304)	29.29	25.00

5140	seq=translation; coord=10:69423694..69433754:-1; parent_transcript=GRMZM2G145870_T01; parent_gene=GRMZM2G145870	GRMZM2G145870_P01,GRMZM2G145870_P02	TRUE	TRUE	dFYGALEAAYk	95%	n+304 (+304), K+304 (+304)	66.05	25.22
5141	seq=translation; coord=10:69423694..69433754:-1; parent_transcript=GRMZM2G145870_T01; parent_gene=GRMZM2G145870	GRMZM2G145870_P01,GRMZM2G145870_P02	TRUE	TRUE	eIEEELGAPLNILEk	95%	n+304 (+304), K+304 (+304)	47.70	25.00
5142	seq=translation; coord=4:5375612..5380694:1; parent_transcript=GRMZM2G154169_T01; parent_gene=GRMZM2G154169	GRMZM2G154169_P01	TRUE	TRUE	aAAELQQAAPIPADGR	92%	n+304 (+304)	27.28	25.87
5143	seq=translation; coord=4:5375612..5380694:1; parent_transcript=GRMZM2G154169_T01; parent_gene=GRMZM2G154169	GRMZM2G154169_P01	TRUE	TRUE	qLILAILENQNLGk	95%	n+304 (+304), K+304 (+304)	62.68	25.00
5144	seq=translation; coord=4:5375612..5380694:1; parent_transcript=GRMZM2G154169_T01; parent_gene=GRMZM2G154169	GRMZM2G154169_P01	TRUE	TRUE	qLILAILENQNLGk	92%	Pyro-cmC (-17), n+304 (+304), K+304 (+304)	28.51	25.91
5145	seq=translation; coord=4:5375612..5380694:1; parent_transcript=GRMZM2G154169_T01; parent_gene=GRMZM2G154169	GRMZM2G154169_P01	TRUE	TRUE	sATGADTEAGGDVAEk	95%	n+304 (+304), K+304 (+304)	39.73	25.00
5146	seq=translation; coord=9:110247484..110252966:-1; parent_transcript=GRMZM2G174444_T01; parent_gene=GRMZM2G174444	GRMZM2G174444_P01,GRMZM2G174444_P02	TRUE	TRUE	aIAPLVSk	94%	n+304 (+304), K+304 (+304)	27.05	25.00
5147	seq=translation; coord=9:110247484..110252966:-1; parent_transcript=GRMZM2G174444_T01; parent_gene=GRMZM2G174444	GRMZM2G174444_P01,GRMZM2G174444_P02	TRUE	TRUE	lPAVFYYTAVWcGPcR	95%	n+304 (+304), Carbamidomethyl (+57), Carbamidomethyl (+57)	28.84	25.00
5148	seq=translation; coord=9:110247484..110252966:-1; parent_transcript=GRMZM2G174444_T01; parent_gene=GRMZM2G174444	GRMZM2G174444_P01,GRMZM2G174444_P02	TRUE	TRUE	vDIELEGLGNk	95%	n+304 (+304), K+304 (+304)	54.40	26.15
5149	seq=translation; coord=5:29321627..29325204:1; parent_transcript=GRMZM2G158277_T01; parent_gene=GRMZM2G158277	GRMZM2G158277_P01,GRMZM2G158277_P02, GRMZM2G158277_P03	TRUE	TRUE	eSAVETVLDSSR	95%	n+304 (+304)	53.55	25.00
5150	seq=translation; coord=5:29321627..29325204:1; parent_transcript=GRMZM2G158277_T01; parent_gene=GRMZM2G158277	GRMZM2G158277_P01,GRMZM2G158277_P02, GRMZM2G158277_P03	TRUE	TRUE	lEDPATGELFAAcFVLPGQR	95%	n+304 (+304), Carbamidomethyl (+57)	35.73	25.00
5151	seq=translation; coord=5:29321627..29325204:1; parent_transcript=GRMZM2G158277_T01; parent_gene=GRMZM2G158277	GRMZM2G158277_P01,GRMZM2G158277_P02, GRMZM2G158277_P03	TRUE	TRUE	sPLPPPPNDPAAAR	95%	n+304 (+304)	33.07	25.98
5152	seq=translation; coord=8:21874081..21876830:1; parent_transcript=GRMZM2G033283_T01; parent_gene=GRMZM2G033283	GRMZM2G033283_P01	TRUE	TRUE	fATGASVVk	95%	n+304 (+304), K+304 (+304)	28.89	25.00
5153	seq=translation; coord=8:21874081..21876830:1; parent_transcript=GRMZM2G033283_T01; parent_gene=GRMZM2G033283	GRMZM2G033283_P01	TRUE	TRUE	gLELFGVk	95%	n+304 (+304), K+304 (+304)	34.25	25.00
5154	seq=translation; coord=8:21874081..21876830:1; parent_transcript=GRMZM2G033283_T01; parent_gene=GRMZM2G033283	GRMZM2G033283_P01	TRUE	TRUE	vLycGVcGLPAEYcEFGPDFER	95%	n+304 (+304), iTRAQ8plex (+304), Carbamidomethyl (+57), Carbamidomethyl (+57), Carbamidomethyl (+57)	38.93	25.00
5155	seq=translation; coord=6:158946942..158950117:1; parent_transcript=GRMZM2G044368_T01; parent_gene=GRMZM2G044368	GRMZM2G044368_P01,GRMZM2G158887_P01, GRMZM5G811797_P04,GRMZM5G811797_P05	TRUE	TRUE	aTIGADFLTk	95%	n+304 (+304), K+304 (+304)	33.73	25.00
5156	seq=translation; coord=6:158946942..158950117:1; parent_transcript=GRMZM2G044368_T01; parent_gene=GRMZM2G044368	GRMZM2G044368_P01,GRMZM2G158887_P01, GRMZM5G811797_P04,GRMZM5G811797_P05	TRUE	TRUE	eVQFEDR	94%	n+304 (+304)	30.87	25.00

5157	seq=translation; coord=6:158946942..158950117:1; parent_transcript=GRMZM2G044368_T01; parent_gene=GRMZM2G044368	GRMZM2G044368_P01,GRMZM2G158887_P01, GRMZM5G811797_P04,GRMZM5G811797_P05	TRUE	TRUE	vIILGDSGVGk	89%	n+304 (+304), K+304 (+304)	25.15	25.00
5158	seq=translation; coord=2:46344346..46345626:-1; parent_transcript=AC206968.3_FGT003; parent_gene=AC206968.3_FG003	AC206968.3_FGP003	TRUE	TRUE	iMVNQADDVAGIISSk	94%	n+304 (+304), K+304 (+304)	29.49	26.86
5159	seq=translation; coord=2:46344346..46345626:-1; parent_transcript=AC206968.3_FGT003; parent_gene=AC206968.3_FG003	AC206968.3_FGP003	TRUE	TRUE	lLLVDIDLLESk	95%	n+304 (+304), K+304 (+304)	58.06	25.00
5160	seq=translation; coord=6:130963745..130976059:-1; parent_transcript=GRMZM2G361605_T01; parent_gene=GRMZM2G361605	GRMZM2G361605_P01,GRMZM2G361605_P02	TRUE	TRUE	aVLDAATIAGLRPLR	92%	n+304 (+304)	26.38	25.00
5161	seq=translation; coord=6:130963745..130976059:-1; parent_transcript=GRMZM2G361605_T01; parent_gene=GRMZM2G361605	GRMZM2G361605_P01,GRMZM2G361605_P02	TRUE	TRUE	vINEcSEAENWLR	87%	n+304 (+304), Carbamidomethyl (+57)	25.60	25.00
5162	seq=translation; coord=5:160443383..160458679:1; parent_transcript=GRMZM2G052435_T03; parent_gene=GRMZM2G052435	GRMZM2G052435_P03	TRUE	TRUE	eNVLGFIVQVPPLLR	95%	n+304 (+304)	60.95	25.00
5163	seq=translation; coord=5:160443383..160458679:1; parent_transcript=GRMZM2G052435_T03; parent_gene=GRMZM2G052435	GRMZM2G052435_P03	TRUE	TRUE	gNVDQWVEHYLR	95%	n+304 (+304)	40.05	25.00
5164	seq=translation; coord=7:170026036..170029198:1; parent_transcript=GRMZM2G055527_T01; parent_gene=GRMZM2G055527	GRMZM2G055527_P01	TRUE	TRUE	sTYFIQcEPTETALSIk	95%	n+304 (+304), Carbamidomethyl (+57), K+304 (+304)	35.75	25.01
5165	seq=translation; coord=7:170026036..170029198:1; parent_transcript=GRMZM2G055527_T01; parent_gene=GRMZM2G055527	GRMZM2G055527_P01	TRUE	TRUE	vDNDSIVALALR	95%	n+304 (+304)	57.62	27.25
5166	seq=translation; coord=4:45262758..45263990:-1; parent_transcript=GRMZM2G071575_T01; parent_gene=GRMZM2G071575	GRMZM2G071575_P01	TRUE	TRUE	aRPEcLcYIIQQVHAGR	95%	n+304 (+304), Carbamidomethyl (+57), Carbamidomethyl (+57)	29.54	25.35
5167	seq=translation; coord=4:45262758..45263990:-1; parent_transcript=GRMZM2G071575_T01; parent_gene=GRMZM2G071575	GRMZM2G071575_P01	TRUE	TRUE	dQVQSLGLR	87%	n+304 (+304)	26.69	25.97
5168	seq=translation; coord=3:57145425..57150481:1; parent_transcript=GRMZM2G033644_T01; parent_gene=GRMZM2G033644	GRMZM2G033644_P01	TRUE	TRUE	fGSPLLNLPEVAIIALGR	95%	n+304 (+304)	33.86	25.00
5169	seq=translation; coord=3:57145425..57150481:1; parent_transcript=GRMZM2G033644_T01; parent_gene=GRMZM2G033644	GRMZM2G033644_P01	TRUE	TRUE	hTFLPFLIk	95%	n+304 (+304), K+304 (+304)	30.50	25.00
5170	seq=translation; coord=10:79271116..79273839:-1; parent_transcript=GRMZM2G032564_T01; parent_gene=GRMZM2G032564	GRMZM2G032564_P01,GRMZM2G152552_P01, GRMZM2G152573_P04,GRMZM2G166963_P01, GRMZM5G820996_P01,GRMZM5G820996_P02	TRUE	TRUE	aFLVEEQk	95%	n+304 (+304), K+304 (+304)	37.81	26.48
5171	seq=translation; coord=10:79271116..79273839:-1; parent_transcript=GRMZM2G032564_T01; parent_gene=GRMZM2G032564	GRMZM2G032564_P01,GRMZM2G152552_P01, GRMZM2G152573_P04,GRMZM2G166963_P01, GRMZM5G820996_P01,GRMZM5G820996_P02	TRUE	TRUE	lVYQYTk	93%	n+304 (+304), K+304 (+304)	28.19	25.00
5172	seq=translation; coord=2:217631963..217637147:1; parent_transcript=GRMZM2G109425_T01; parent_gene=GRMZM2G109425	GRMZM2G109425_P01,GRMZM2G109425_P02	TRUE	TRUE	aQLGVEAFAEALLVIPk	95%	n+304 (+304), K+304 (+304)	49.98	25.00
5173	seq=translation; coord=2:217631963..217637147:1; parent_transcript=GRMZM2G109425_T01; parent_gene=GRMZM2G109425	GRMZM2G109425_P01,GRMZM2G109425_P02	TRUE	TRUE	fLVDGFDVAk	95%	n+304 (+304), K+304 (+304)	40.33	25.89

5174	seq=translation; coord=2:217631963..217637147:1; parent_transcript=GRMZM2G109425_T01; parent_gene=GRMZM2G109425	GRMZM2G109425_P01,GRMZM2G109425_P02	TRUE	TRUE	kPDEPIDLFMVEIMHMR	95%	K+304 (+304), n+304 (+304)	43.63	25.77
5175	seq=translation; coord=2:217631963..217637147:1; parent_transcript=GRMZM2G109425_T01; parent_gene=GRMZM2G109425	GRMZM2G109425_P01,GRMZM2G109425_P02	TRUE	TRUE	mLVGGAGDLk	95%	n+304 (+304), K+304 (+304)	30.92	26.80
5176	seq=translation; coord=2:217631963..217637147:1; parent_transcript=GRMZM2G109425_T01; parent_gene=GRMZM2G109425	GRMZM2G109425_P01,GRMZM2G109425_P02	TRUE	TRUE	tLAENSGLDTQDVIVSLQNEHDR	95%	n+304 (+304)	70.33	25.00
5177	seq=translation; coord=9:136083370..136087677:1; parent_transcript=GRMZM2G124059_T01; parent_gene=GRMZM2G124059	GRMZM2G124059_P01	TRUE	TRUE	aQPVLLPHLLLSYVEQLER	95%	n+304 (+304)	44.13	25.00
5178	seq=translation; coord=9:136083370..136087677:1; parent_transcript=GRMZM2G124059_T01; parent_gene=GRMZM2G124059	GRMZM2G124059_P01	TRUE	TRUE	aQPVLLPHLLLSyVEQLER	94%	n+304 (+304), iTRAQ8plex (+304)	25.87	25.00
5179	seq=translation; coord=8:122751582..122753805:1; parent_transcript=GRMZM2G154936_T01; parent_gene=GRMZM2G154936	GRMZM2G154936_P01,GRMZM2G154936_P02, GRMZM2G154936_P03	TRUE	TRUE	gADcASASk	95%	n+304 (+304), Carbamidomethyl (+57), K+304 (+304)	28.47	25.00
5180	seq=translation; coord=8:122751582..122753805:1; parent_transcript=GRMZM2G154936_T01; parent_gene=GRMZM2G154936	GRMZM2G154936_P01,GRMZM2G154936_P02, GRMZM2G154936_P03	TRUE	TRUE	tGVPDQAMQAAIDYAcAk	95%	n+304 (+304), Carbamidomethyl (+57), K+304 (+304)	63.91	25.00
5181	seq=translation; coord=8:122751582..122753805:1; parent_transcript=GRMZM2G154936_T01; parent_gene=GRMZM2G154936	GRMZM2G154936_P01,GRMZM2G154936_P02, GRMZM2G154936_P03	TRUE	TRUE	vAVcSYIcNSYYQSR	95%	n+304 (+304), Carbamidomethyl (+57), Carbamidomethyl (+57)	60.67	25.00
5182	seq=translation; coord=10:35583589..35590383:1; parent_transcript=GRMZM2G009443_T01; parent_gene=GRMZM2G009443	GRMZM2G009443_P01,GRMZM2G123922_P01	TRUE	TRUE	aHPDVFNMMLQILEDGR	95%	n+304 (+304)	45.30	25.00
5183	seq=translation; coord=10:35583589..35590383:1; parent_transcript=GRMZM2G009443_T01; parent_gene=GRMZM2G009443	GRMZM2G009443_P01,GRMZM2G123922_P01	TRUE	TRUE	lGHNFVGTEQILLGLIGEGTGIAAk	95%	n+304 (+304), K+304 (+304)	53.14	25.00
5184	seq=translation; coord=8:1370962..1373972:-1; parent_transcript=GRMZM2G042636_T01; parent_gene=GRMZM2G042636	GRMZM2G042636_P01,GRMZM2G042636_P02, GRMZM2G042636_P03,GRMZM2G042636_P04, GRMZM2G133802_P01,GRMZM2G133802_P02, GRMZM2G133802_P03	TRUE	TRUE	yVGTSDLQLER	95%	n+304 (+304)	53.70	25.28
5185	seq=translation; coord=4:93237748..93253880:1; parent_transcript=GRMZM2G451443_T01; parent_gene=GRMZM2G451443	GRMZM2G451443_P01	TRUE	TRUE	eNILGFIVQVPPLLR	93%	n+304 (+304)	28.34	25.00
5186	seq=translation; coord=4:93237748..93253880:1; parent_transcript=GRMZM2G451443_T01; parent_gene=GRMZM2G451443	GRMZM2G451443_P01	TRUE	TRUE	tAAMDFVSELVR	95%	n+304 (+304)	33.67	25.00
5187	seq=translation; coord=5:24616889..24618887:-1; parent_transcript=GRMZM2G032910_T01; parent_gene=GRMZM2G032910	GRMZM2G032910_P01,GRMZM2G032910_P02	TRUE	TRUE	dFGAMPAYEFALQHPGR	95%	n+304 (+304)	63.27	25.00
5188	seq=translation; coord=5:24616889..24618887:-1; parent_transcript=GRMZM2G032910_T01; parent_gene=GRMZM2G032910	GRMZM2G032910_P01,GRMZM2G032910_P02	TRUE	TRUE	fPGFETAMR	93%	n+304 (+304)	30.11	25.00
5189	seq=translation; coord=5:24616889..24618887:-1; parent_transcript=GRMZM2G032910_T01; parent_gene=GRMZM2G032910	GRMZM2G032910_P01,GRMZM2G032910_P02	TRUE	TRUE	fQVPVFMVMGEk	95%	n+304 (+304), K+304 (+304)	32.02	25.63
5190	seq=translation; coord=5:24616889..24618887:-1; parent_transcript=GRMZM2G032910_T01; parent_gene=GRMZM2G032910	GRMZM2G032910_P01,GRMZM2G032910_P02	TRUE	TRUE	tIYVLFAGAEIPIAk	95%	n+304 (+304), K+304 (+304)	47.04	25.00

5191	seq=translation; coord=1:12987102..12993471:1; parent_transcript=GRMZM2G172322_T01; parent_gene=GRMZM2G172322	GRMZM2G172322_P01,GRMZM2G172322_P02	TRUE	TRUE	aTLSGLPDR	92%	n+304 (+304)	30.34	26.51
5192	seq=translation; coord=1:12987102..12993471:1; parent_transcript=GRMZM2G172322_T01; parent_gene=GRMZM2G172322	GRMZM2G172322_P01,GRMZM2G172322_P02	TRUE	TRUE	gITFHTEQSPQAITk	94%	n+304 (+304), K+304 (+304)	28.06	25.00
5193	seq=translation; coord=1:12987102..12993471:1; parent_transcript=GRMZM2G172322_T01; parent_gene=GRMZM2G172322	GRMZM2G172322_P01,GRMZM2G172322_P02	TRUE	TRUE	tSVDSIWAVGDVTNR	92%	n+304 (+304)	26.99	25.00
5194	seq=translation; coord=1:12987102..12993471:1; parent_transcript=GRMZM2G172322_T01; parent_gene=GRMZM2G172322	GRMZM2G172322_P01,GRMZM2G172322_P02	TRUE	TRUE	vNLTPVALMEGGAFAk	95%	n+304 (+304), K+304 (+304)	37.53	25.45
5195	seq=translation; coord=4:54600217..54609072:-1; parent_transcript=GRMZM2G098496_T01; parent_gene=GRMZM2G098496	GRMZM2G098496_P01,GRMZM2G098496_P02	TRUE	TRUE	aTEIFEAIAR	93%	n+304 (+304)	29.89	25.01
5196	seq=translation; coord=4:54600217..54609072:-1; parent_transcript=GRMZM2G098496_T01; parent_gene=GRMZM2G098496	GRMZM2G098496_P01,GRMZM2G098496_P02	TRUE	TRUE	lLADLAASMDEGDVAk	95%	n+304 (+304), K+304 (+304)	41.93	25.08
5197	seq=translation; coord=4:54600217..54609072:-1; parent_transcript=GRMZM2G098496_T01; parent_gene=GRMZM2G098496	GRMZM2G098496_P01,GRMZM2G098496_P02	TRUE	TRUE	vAEIAAQLEQYPk	95%	n+304 (+304), K+304 (+304)	38.22	25.00
5198	seq=translation; coord=3:148834092..148846161:-1; parent_transcript=GRMZM2G047310_T02; parent_gene=GRMZM2G047310	GRMZM2G047310_P02	TRUE	TRUE	eAYLSDSEFQTVFGMTk	95%	n+304 (+304), K+304 (+304)	45.60	25.00
5199	seq=translation; coord=3:148834092..148846161:-1; parent_transcript=GRMZM2G047310_T02; parent_gene=GRMZM2G047310	GRMZM2G047310_P02	TRUE	TRUE	ePPQFVALFQPMVILk	95%	n+304 (+304), K+304 (+304)	38.22	25.00
5200	seq=translation; coord=3:148834092..148846161:-1; parent_transcript=GRMZM2G047310_T02; parent_gene=GRMZM2G047310	GRMZM2G047310_P02	TRUE	TRUE	tVSDDDVVLETTAPk	92%	n+304 (+304), K+304 (+304)	27.10	25.61
5201	seq=translation; coord=5:155530969..155538363:-1; parent_transcript=GRMZM2G015132_T01; parent_gene=GRMZM2G015132	GRMZM2G015132_P01	TRUE	TRUE	dNSLkPADYEGGTFTVSNLGGPFGIk	95%	n+304 (+304), K+304 (+304), K+304 (+304)	27.94	25.00
5202	seq=translation; coord=5:155530969..155538363:-1; parent_transcript=GRMZM2G015132_T01; parent_gene=GRMZM2G015132	GRMZM2G015132_P01	TRUE	TRUE	gLGAIAEEVk	90%	n+304 (+304), K+304 (+304)	27.03	26.64
5203	seq=translation; coord=5:155530969..155538363:-1; parent_transcript=GRMZM2G015132_T01; parent_gene=GRMZM2G015132	GRMZM2G015132_P01	TRUE	TRUE	vIDGAIGAEFLk	95%	n+304 (+304), K+304 (+304)	60.13	25.00
5204	seq=translation; coord=8:98594898..98599276:-1; parent_transcript=GRMZM2G114954_T01; parent_gene=GRMZM2G114954	GRMZM2G114954_P01,GRMZM2G114954_P02, GRMZM2G114954_P03,GRMZM2G114954_P04	TRUE	TRUE	lEFLLTELk	95%	n+304 (+304), K+304 (+304)	29.45	25.00
5205	seq=translation; coord=8:98594898..98599276:-1; parent_transcript=GRMZM2G114954_T01; parent_gene=GRMZM2G114954	GRMZM2G114954_P01,GRMZM2G114954_P02, GRMZM2G114954_P03,GRMZM2G114954_P04	TRUE	TRUE	vIAAYLLAVLGGNTSPTADDVk	95%	n+304 (+304), K+304 (+304)	29.98	25.00
5206	seq=translation; coord=8:9919345..9920090:-1; parent_transcript=AC204893.3_FGT001; parent_gene=AC204893.3_FG001	AC204893.3_FGP001,GRMZM2G322618_P01	TRUE	TRUE	nAAcLAEQLAR	95%	n+304 (+304), Carbamidomethyl (+57)	50.03	25.00
5207	seq=translation; coord=8:9919345..9920090:-1; parent_transcript=AC204893.3_FGT001; parent_gene=AC204893.3_FG001	AC204893.3_FGP001,GRMZM2G322618_P01	TRUE	TRUE	vPALSENk	95%	n+304 (+304), K+304 (+304)	32.78	25.11
5208	seq=translation; coord=8:159703050..159707801:1; parent_transcript=GRMZM2G122607_T01; parent_gene=GRMZM2G122607	GRMZM2G122607_P01	TRUE	TRUE	dLAADGGGAGGGGIGVVEMLFR	95%	n+304 (+304)	58.05	25.00

5209	seq=translation; coord=8:159703050..159707801:1; parent_transcript=GRMZM2G122607_T01; parent_gene=GRMZM2G122607	GRMZM2G122607_P01	TRUE	TRUE	iIVVLENk	95%	n+304 (+304), K+304 (+304)	36.11	25.00
5210	seq=translation; coord=4:161027635..161031592:-1; parent_transcript=GRMZM2G156068_T01; parent_gene=GRMZM2G156068	GRMZM2G156068_P01,GRMZM2G156068_P02	TRUE	TRUE	aIAEIFADAGFSDVTk	95%	n+304 (+304), K+304 (+304)	51.63	26.63
5211	seq=translation; coord=4:161027635..161031592:-1; parent_transcript=GRMZM2G156068_T01; parent_gene=GRMZM2G156068	GRMZM2G156068_P01,GRMZM2G156068_P02	TRUE	TRUE	aLFGGTGNYASALFLTAAk	95%	n+304 (+304), K+304 (+304)	60.12	25.16
5212	seq=translation; coord=3:88528132..88533870:-1; parent_transcript=GRMZM2G476933_T01; parent_gene=GRMZM2G476933	GRMZM2G476933_P01	TRUE	TRUE	aTAADPAPGAPAENSTDk	95%	n+304 (+304), K+304 (+304)	52.23	25.12
5213	seq=translation; coord=3:88528132..88533870:-1; parent_transcript=GRMZM2G476933_T01; parent_gene=GRMZM2G476933	GRMZM2G476933_P01	TRUE	TRUE	sDAEPAAAAAPVPGTNSDAAATDPAPGTk	95%	n+304 (+304), K+304 (+304)	33.39	25.00
5214	seq=translation; coord=3:88528132..88533870:-1; parent_transcript=GRMZM2G476933_T01; parent_gene=GRMZM2G476933	GRMZM2G476933_P01	TRUE	TRUE	sDAEPAAVAAPAPETk	95%	n+304 (+304), K+304 (+304)	64.13	25.89
5215	seq=translation; coord=1:54927760..54932392:-1; parent_transcript=GRMZM2G031825_T01; parent_gene=GRMZM2G031825	GRMZM2G031825_P01,GRMZM2G031825_P02, GRMZM2G031825_P05	TRUE	TRUE	vDLPEIQAk	89%	n+304 (+304), K+304 (+304)	26.71	25.00
5216	seq=translation; coord=5:181390095..181395720:1; parent_transcript=GRMZM2G031107_T01; parent_gene=GRMZM2G031107	GRMZM2G031107_P01,GRMZM2G031107_P02	TRUE	TRUE	aAWEIFTPLLHDIDGGk	95%	n+304 (+304), K+304 (+304)	54.45	25.26
5217	seq=translation; coord=10:112583884..112587345:-1; parent_transcript=GRMZM2G022180_T02; parent_gene=GRMZM2G022180	GRMZM2G022180_P02	TRUE	TRUE	iLIDGEEk	91%	n+304 (+304), K+304 (+304)	28.86	25.84
5218	seq=translation; coord=5:66839986..66842997:-1; parent_transcript=GRMZM2G115176_T01; parent_gene=GRMZM2G115176	GRMZM2G115176_P01,GRMZM2G115176_P03, GRMZM2G115925_P01,GRMZM5G884325_P01, GRMZM5G884325_P02	TRUE	TRUE	fMIIPDMLk	90%	n+304 (+304), K+304 (+304)	26.12	25.45
5219	seq=translation; coord=6:167429613..167438752:-1; parent_transcript=GRMZM2G402002_T01; parent_gene=GRMZM2G402002	GRMZM2G402002_P01	TRUE	TRUE	iFSLPDLELIR	91%	n+304 (+304)	27.93	25.01
5220	seq=translation; coord=6:167429613..167438752:-1; parent_transcript=GRMZM2G402002_T01; parent_gene=GRMZM2G402002	GRMZM2G402002_P01	TRUE	TRUE	nFLDGDLIESFLDLSR	95%	n+304 (+304)	41.13	25.00
5221	seq=translation; coord=1:56223520..56226018:1; parent_transcript=GRMZM2G062425_T01; parent_gene=GRMZM2G062425	GRMZM2G062425_P01,GRMZM2G062425_P02	TRUE	TRUE	eTAQQLLSQFEk	92%	n+304 (+304), K+304 (+304)	27.00	26.16
5222	seq=translation; coord=1:56223520..56226018:1; parent_transcript=GRMZM2G062425_T01; parent_gene=GRMZM2G062425	GRMZM2G062425_P01,GRMZM2G062425_P02	TRUE	TRUE	gDITLWSVDcATDAIVNAANER	95%	n+304 (+304), Carbamidomethyl (+57)	65.72	25.00
5223	seq=translation; coord=1:56223520..56226018:1; parent_transcript=GRMZM2G062425_T01; parent_gene=GRMZM2G062425	GRMZM2G062425_P01,GRMZM2G062425_P02	TRUE	TRUE	lSAAPGAGLLk	95%	n+304 (+304), K+304 (+304)	45.94	25.00
5224	seq=translation; coord=2:207265904..207268967:1; parent_transcript=AC194970.5_FGT009; parent_gene=AC194970.5_FG009	AC194970.5_FGP009	TRUE	TRUE	aAGLILQLIR	94%	n+304 (+304)	29.19	25.00
5225	seq=translation; coord=2:207265904..207268967:1; parent_transcript=AC194970.5_FGT009; parent_gene=AC194970.5_FG009	AC194970.5_FGP009	TRUE	TRUE	iLDIR	86%	n+304 (+304)	25.44	25.00
5226	seq=translation; coord=3:224163656..224168718:-1; parent_transcript=GRMZM2G339540_T04; parent_gene=GRMZM2G339540	GRMZM2G339540_P04,GRMZM2G339540_P05	TRUE	TRUE	aLFLQQNLLSGPIPTGIQk	95%	n+304 (+304), K+304 (+304)	61.97	25.00

5227	seq=translation; coord=3:224163656..224168718:-1; parent_transcript=GRMZM2G339540_T04; parent_gene=GRMZM2G339540	GRMZM2G339540_P04,GRMZM2G339540_P05	TRUE	TRUE	lPGVGLVGAIPPGTLGR	95%	n+304 (+304)	41.79	25.00
5228	seq=translation; coord=3:224163656..224168718:-1; parent_transcript=GRMZM2G339540_T04; parent_gene=GRMZM2G339540	GRMZM2G339540_P04,GRMZM2G339540_P05	TRUE	TRUE	sALLAFLTATPHER	95%	n+304 (+304)	52.35	26.01
5229	seq=translation; coord=7:154714410..154723867:1; parent_transcript=GRMZM2G375593_T01; parent_gene=GRMZM2G375593	GRMZM2G375593_P01	TRUE	TRUE	eGSETDQFWSLLGGk	93%	n+304 (+304), K+304 (+304)	26.84	25.00
5230	seq=translation; coord=7:154714410..154723867:1; parent_transcript=GRMZM2G375593_T01; parent_gene=GRMZM2G375593	GRMZM2G375593_P01	TRUE	TRUE	fIVLDFLMENLAR	95%	n+304 (+304)	29.51	26.25
5231	seq=translation; coord=7:154714410..154723867:1; parent_transcript=GRMZM2G375593_T01; parent_gene=GRMZM2G375593	GRMZM2G375593_P01	TRUE	TRUE	iALQLF	95%	n+304 (+304)	31.03	25.00
5232	seq=translation; coord=7:7507005..7510800:-1; parent_transcript=GRMZM2G143330_T01; parent_gene=GRMZM2G143330	GRMZM2G143330_P01	TRUE	TRUE	iWDSETGQLLk	95%	n+304 (+304), K+304 (+304)	52.57	25.44
5233	seq=translation; coord=7:7507005..7510800:-1; parent_transcript=GRMZM2G143330_T01; parent_gene=GRMZM2G143330	GRMZM2G143330_P01	TRUE	TRUE	tIITAGEDATVR	95%	n+304 (+304)	39.93	25.35
5234	seq=translation; coord=7:124863398..124868735:1; parent_transcript=GRMZM2G067265_T01; parent_gene=GRMZM2G067265	GRMZM2G067265_P01,GRMZM2G067265_P02	TRUE	TRUE	aPDFAVPLEELEAAVSk	95%	n+304 (+304), K+304 (+304)	41.37	25.66
5235	seq=translation; coord=7:124863398..124868735:1; parent_transcript=GRMZM2G067265_T01; parent_gene=GRMZM2G067265	GRMZM2G067265_P01,GRMZM2G067265_P02	TRUE	TRUE	hGAINLGQGFPNFDGPDFVk	95%	n+304 (+304), K+304 (+304)	47.80	25.71
5236	seq=translation; coord=7:124863398..124868735:1; parent_transcript=GRMZM2G067265_T01; parent_gene=GRMZM2G067265	GRMZM2G067265_P01,GRMZM2G067265_P02	TRUE	TRUE	tTIFTQMSMLAIk	94%	n+304 (+304), K+304 (+304)	27.65	25.00
5237	seq=translation; coord=5:136560253..136565466:1; parent_transcript=GRMZM2G147210_T01; parent_gene=GRMZM2G147210	GRMZM2G147210_P01	TRUE	TRUE	sPEDAAALIDDk	95%	n+304 (+304), K+304 (+304)	31.10	25.00
5238	seq=translation; coord=3:15212255..15222203:-1; parent_transcript=GRMZM2G334628_T01; parent_gene=GRMZM2G334628	GRMZM2G334628_P01,GRMZM2G334628_P02, GRMZM2G334628_P07	TRUE	TRUE	aNDQYLPPFVIVDDSGk	95%	n+304 (+304), K+304 (+304)	33.53	25.00
5239	seq=translation; coord=5:23838144..23838818:1; parent_transcript=GRMZM2G387360_T01; parent_gene=GRMZM2G387360	GRMZM2G387360_P01	TRUE	TRUE	aQEPcFcQYAR	87%	n+304 (+304), Carbamidomethyl (+57), Carbamidomethyl (+57)	25.49	25.00
5240	seq=translation; coord=1:289786005..289788499:1; parent_transcript=GRMZM2G168833_T01; parent_gene=GRMZM2G168833	GRMZM2G168833_P01	TRUE	TRUE	aMDLPSLcSNPATFSDcPk	95%	n+304 (+304), Carbamidomethyl (+57), Carbamidomethyl (+57), K+304 (+304)	28.85	25.00
5241	seq=translation; coord=1:289786005..289788499:1; parent_transcript=GRMZM2G168833_T01; parent_gene=GRMZM2G168833	GRMZM2G168833_P01	TRUE	TRUE	amDLPSLcSNPATFSDcPk	95%	n+304 (+304), Oxidation (+16), Carbamidomethyl (+57), Carbamidomethyl (+57), K+304 (+304)	29.81	25.00
5242	seq=translation; coord=1:289786005..289788499:1; parent_transcript=GRMZM2G168833_T01; parent_gene=GRMZM2G168833	GRMZM2G168833_P01	TRUE	TRUE	aPTPDccAGLk	95%	n+304 (+304), Carbamidomethyl (+57), Carbamidomethyl (+57), K+304 (+304)	55.27	25.00
5243	seq=translation; coord=1:289786005..289788499:1; parent_transcript=GRMZM2G168833_T01; parent_gene=GRMZM2G168833	GRMZM2G168833_P01	TRUE	TRUE	iLGMSPDAPEAEIFk	95%	n+304 (+304), K+304 (+304)	38.76	25.93

5244	seq=translation; coord=1:289786005..289788499:1; parent_transcript=GRMZM2G168833_T01; parent_gene=GRMZM2G168833	GRMZM2G168833_P01	TRUE	TRUE	lmGLATcLTFVQDk	95%	n+304 (+304), Oxidation (+16), Carbamidomethyl (+57), K+304 (+304)	44.21	25.42
5245	seq=translation; coord=5:159290030..159322686:-1; parent_transcript=GRMZM2G386155_T01; parent_gene=GRMZM2G386155	GRMZM2G386155_P01,GRMZM2G386155_P02	TRUE	TRUE	gAGHVPSDYLPFEVFAAYQR	95%	n+304 (+304)	56.18	25.00
5246	seq=translation; coord=5:159290030..159322686:-1; parent_transcript=GRMZM2G386155_T01; parent_gene=GRMZM2G386155	GRMZM2G386155_P01,GRMZM2G386155_P02	TRUE	TRUE	mGITNQFIEYDSGQMFESLSk	95%	n+304 (+304), K+304 (+304)	59.56	25.00
5247	seq=translation; coord=5:159290030..159322686:-1; parent_transcript=GRMZM2G386155_T01; parent_gene=GRMZM2G386155	GRMZM2G386155_P01,GRMZM2G386155_P02	TRUE	TRUE	vADLYDFLSk	95%	n+304 (+304), K+304 (+304)	33.46	25.35
5248	seq=translation; coord=5:159290030..159322686:-1; parent_transcript=GRMZM2G386155_T01; parent_gene=GRMZM2G386155	GRMZM2G386155_P01,GRMZM2G386155_P02	TRUE	TRUE	vAVNPYSWTk	92%	n+304 (+304), K+304 (+304)	27.69	25.19
5249	seq=translation; coord=6:103857605..103858686:-1; parent_transcript=GRMZM2G335618_T01; parent_gene=GRMZM2G335618	GRMZM2G335618_P01	TRUE	TRUE	kGLVLLDFWVSPFGQR	94%	K+304 (+304), n+304 (+304)	25.74	25.00
5250	seq=translation; coord=6:103857605..103858686:-1; parent_transcript=GRMZM2G335618_T01; parent_gene=GRMZM2G335618	GRMZM2G335618_P01	TRUE	TRUE	lLPDAAYAR	91%	n+304 (+304)	27.77	25.00
5251	seq=translation; coord=6:103857605..103858686:-1; parent_transcript=GRMZM2G335618_T01; parent_gene=GRMZM2G335618	GRMZM2G335618_P01	TRUE	TRUE	sDILLR	93%	n+304 (+304)	30.65	25.43
5252	seq=translation; coord=6:103857605..103858686:-1; parent_transcript=GRMZM2G335618_T01; parent_gene=GRMZM2G335618	GRMZM2G335618_P01	TRUE	TRUE	vDEFINLLk	95%	n+304 (+304), K+304 (+304)	35.49	25.00
5253	seq=translation; coord=2:42784980..42792494:1; parent_transcript=GRMZM2G065073_T02; parent_gene=GRMZM2G065073	GRMZM2G065073_P02	TRUE	TRUE	lADGTAVVYDSR	95%	n+304 (+304)	36.70	25.00
5254	seq=translation; coord=2:42784980..42792494:1; parent_transcript=GRMZM2G065073_T02; parent_gene=GRMZM2G065073	GRMZM2G065073_P02	TRUE	TRUE	nMDPLASVMAPR	94%	n+304 (+304)	30.75	25.00
5255	seq=translation; coord=2:42784980..42792494:1; parent_transcript=GRMZM2G065073_T02; parent_gene=GRMZM2G065073	GRMZM2G065073_P02	TRUE	TRUE	qGDLTAVSDPR	95%	n+304 (+304)	45.48	25.00
5256	seq=translation; coord=8:607069..619737:1; parent_transcript=GRMZM2G077233_T01; parent_gene=GRMZM2G077233	GRMZM2G077233_P01,GRMZM2G077233_P02, GRMZM2G077233_P03,GRMZM2G077233_P04, GRMZM2G077233_P06	TRUE	TRUE	dVFSFWLNR	95%	n+304 (+304)	37.32	25.00
5257	seq=translation; coord=8:607069..619737:1; parent_transcript=GRMZM2G077233_T01; parent_gene=GRMZM2G077233	GRMZM2G077233_P01,GRMZM2G077233_P02, GRMZM2G077233_P03,GRMZM2G077233_P04, GRMZM2G077233_P06	TRUE	TRUE	eLILNYANQLcER	94%	n+304 (+304), Carbamidomethyl (+57)	31.25	25.00
5258	seq=translation; coord=8:607069..619737:1; parent_transcript=GRMZM2G077233_T01; parent_gene=GRMZM2G077233	GRMZM2G077233_P01,GRMZM2G077233_P02, GRMZM2G077233_P03,GRMZM2G077233_P04, GRMZM2G077233_P06	TRUE	TRUE	tFTLTPEQYIVk	95%	n+304 (+304), K+304 (+304)	31.82	25.30
5259	seq=translation; coord=3:184434264..184435351:1; parent_transcript=GRMZM2G126397_T01; parent_gene=GRMZM2G126397	GRMZM2G126397_P01	TRUE	TRUE	sLLQQANNTPDR	95%	n+304 (+304)	47.89	25.00
5260	seq=translation; coord=3:184434264..184435351:1; parent_transcript=GRMZM2G126397_T01; parent_gene=GRMZM2G126397	GRMZM2G126397_P01	TRUE	TRUE	tIcGcLk	95%	n+304 (+304), Carbamidomethyl (+57), Carbamidomethyl (+57), K+304 (+304)	32.22	25.82

5261	seq=translation; coord=5:168890072..168891220:-1; parent_transcript=GRMZM2G115491_T01; parent_gene=GRMZM2G115491	GRMZM2G115491_P01	TRUE	TRUE	aFGLEPEELR	95%	n+304 (+304)	35.92	25.00
5262	seq=translation; coord=5:168890072..168891220:-1; parent_transcript=GRMZM2G115491_T01; parent_gene=GRMZM2G115491	GRMZM2G115491_P01	TRUE	TRUE	aTETALVLAGR	95%	n+304 (+304)	49.11	25.72
5263	seq=translation; coord=8:5023676..5024789:1; parent_transcript=GRMZM2G095164_T01; parent_gene=GRMZM2G095164	GRMZM2G095164_P01,GRMZM2G332259_P01	TRUE	TRUE	gASYYNcQPGAEANPYSR	93%	n+304 (+304), Carbamidomethyl (+57)	29.43	25.00
5264	seq=translation; coord=8:5023676..5024789:1; parent_transcript=GRMZM2G095164_T01; parent_gene=GRMZM2G095164	GRMZM2G095164_P01,GRMZM2G332259_P01	TRUE	TRUE	gcSAITQcR	92%	n+304 (+304), Carbamidomethyl (+57), Carbamidomethyl (+57)	29.23	25.00
5265	seq=translation; coord=4:35209593..35215535:1; parent_transcript=GRMZM2G145972_T02; parent_gene=GRMZM2G145972	GRMZM2G145972_P02	TRUE	TRUE	iSLIEAADHILTMFDk	95%	n+304 (+304), K+304 (+304)	31.55	26.06
5266	seq=translation; coord=4:35209593..35215535:1; parent_transcript=GRMZM2G145972_T02; parent_gene=GRMZM2G145972	GRMZM2G145972_P02	TRUE	TRUE	vMEDISEIFR	94%	n+304 (+304)	31.39	25.00
5267	seq=translation; coord=9:143036859..143040005:-1; parent_transcript=GRMZM2G149809_T01; parent_gene=GRMZM2G149809	GRMZM2G149809_P01,GRMZM2G149809_P02	TRUE	TRUE	gASAAVDAPAGWSGR	95%	n+304 (+304)	73.99	25.00
5268	seq=translation; coord=9:143036859..143040005:-1; parent_transcript=GRMZM2G149809_T01; parent_gene=GRMZM2G149809	GRMZM2G149809_P01,GRMZM2G149809_P02	TRUE	TRUE	tLcATDGATGR	95%	n+304 (+304), Carbamidomethyl (+57)	41.05	25.00
5269	seq=translation; coord=3:147969444..147972018:1; parent_transcript=GRMZM2G701801_T02; parent_gene=GRMZM2G701801	GRMZM2G701801_P02	TRUE	TRUE	aAAEVcEDSIPGLR	95%	n+304 (+304), Carbamidomethyl (+57)	48.71	25.00
5270	seq=translation; coord=3:147969444..147972018:1; parent_transcript=GRMZM2G701801_T02; parent_gene=GRMZM2G701801	GRMZM2G701801_P02	TRUE	TRUE	vLLSLQAFTLR	88%	n+304 (+304)	26.88	25.48
5271	seq=translation; coord=2:222675442..222682154:1; parent_transcript=GRMZM2G015933_T01; parent_gene=GRMZM2G015933	GRMZM2G015933_P01	TRUE	TRUE	vTAVIQDMR	95%	n+304 (+304)	32.88	25.11
5272	seq=translation; coord=4:20506351..20509895:1; parent_transcript=GRMZM2G112795_T01; parent_gene=GRMZM2G112795	GRMZM2G112795_P01	TRUE	TRUE	mIYDVNSPLFR	95%	n+304 (+304)	39.42	25.00
5273	seq=translation; coord=1:219892621..219901471:1; parent_transcript=GRMZM2G015925_T01; parent_gene=GRMZM2G015925	GRMZM2G015925_P01,GRMZM2G015925_P02	TRUE	TRUE	eVAELIk	87%	n+304 (+304), K+304 (+304)	26.02	25.47
5274	seq=translation; coord=8:159254849..159263818:-1; parent_transcript=GRMZM2G046558_T02; parent_gene=GRMZM2G046558	GRMZM2G046558_P02	TRUE	TRUE	aAAAHGIALVAPDTSPR	95%	n+304 (+304)	38.60	26.09
5275	seq=translation; coord=8:159254849..159263818:-1; parent_transcript=GRMZM2G046558_T02; parent_gene=GRMZM2G046558	GRMZM2G046558_P02	TRUE	TRUE	aAGAALTLR	90%	n+304 (+304)	26.53	25.00
5276	seq=translation; coord=8:159254849..159263818:-1; parent_transcript=GRMZM2G046558_T02; parent_gene=GRMZM2G046558	GRMZM2G046558_P02	TRUE	TRUE	sDWEEYDATcLIk	95%	n+304 (+304), Carbamidomethyl (+57), K+304 (+304)	39.91	25.00
5277	seq=translation; coord=8:159254849..159263818:-1; parent_transcript=GRMZM2G046558_T02; parent_gene=GRMZM2G046558	GRMZM2G046558_P02	TRUE	TRUE	vPTTILIDQGEADk	91%	n+304 (+304), K+304 (+304)	26.09	25.88
5278	seq=translation; coord=9:64132094..64145762:1; parent_transcript=GRMZM2G072909_T01; parent_gene=GRMZM2G072909	GRMZM2G072909_P01	TRUE	TRUE	aNVVVASLEDkLEIVASR	95%	n+304 (+304), K+304 (+304)	61.60	25.00

	seq=translation; coord=9:64132094..64145762:1;								
5279	parent_transcript=GRMZM2G072909_T01; parent_gene=GRMZM2G072909	GRMZM2G072909_P01	TRUE	TRUE	gPGTAMEYSVILVEQLYGk	95%	n+304 (+304), K+304 (+304)	45.58	25.26
5280	seq=translation; coord=9:64132094..64145762:1; parent_transcript=GRMZM2G072909_T01; parent_gene=GRMZM2G072909	GRMZM2G072909_P01	TRUE	TRUE	mIADVLLDDALk	95%	n+304 (+304), K+304 (+304)	63.18	26.26
5281	seq=translation; coord=4:194605171..194610906:-1; parent_transcript=GRMZM2G097043_T01; parent_gene=GRMZM2G097043	GRMZM2G097043_P01	TRUE	TRUE	lDDFVASRPELFVIEGDFIHLR	95%	n+304 (+304)	47.33	25.37
5282	seq=translation; coord=4:194605171..194610906:-1; parent_transcript=GRMZM2G097043_T01; parent_gene=GRMZM2G097043	GRMZM2G097043_P01	TRUE	TRUE	qIHDLQELLR	95%	Pyro-cmC (-17), n+304 (+304)	38.34	25.00
5283	seq=translation; coord=4:194605171..194610906:-1; parent_transcript=GRMZM2G097043_T01; parent_gene=GRMZM2G097043	GRMZM2G097043_P01	TRUE	TRUE	sLLHTIQQLQQELAEAR	95%	n+304 (+304)	56.67	25.75
5284	seq=translation; coord=1:3830541..3834833:1; parent_transcript=GRMZM2G085909_T01; parent_gene=GRMZM2G085909	GRMZM2G085909_P01	TRUE	TRUE	aVEILHAMAIPIELSDR	95%	n+304 (+304)	54.41	26.07
5285	seq=translation; coord=1:3830541..3834833:1; parent_transcript=GRMZM2G085909_T01; parent_gene=GRMZM2G085909	GRMZM2G085909_P01	TRUE	TRUE	gSNQLVIDEAER	95%	n+304 (+304)	73.15	25.13
5286	seq=translation; coord=1:3830541..3834833:1; parent_transcript=GRMZM2G085909_T01; parent_gene=GRMZM2G085909	GRMZM2G085909_P01	TRUE	TRUE	iAVIQFQISPPk	95%	n+304 (+304), K+304 (+304)	48.19	25.00
5287	seq=translation; coord=1:3830541..3834833:1; parent_transcript=GRMZM2G085909_T01; parent_gene=GRMZM2G085909	GRMZM2G085909_P01	TRUE	TRUE	lGGTVDDTELIPGLIFDk	95%	n+304 (+304), K+304 (+304)	36.92	25.24
5288	seq=translation; coord=2:212802942..212807188:1; parent_transcript=GRMZM2G141510_T01; parent_gene=GRMZM2G141510	GRMZM2G141510_P01	TRUE	TRUE	dHEILPPLPVGFHR	94%	n+304 (+304)	27.05	25.79
5289	seq=translation; coord=2:212802942..212807188:1; parent_transcript=GRMZM2G141510_T01; parent_gene=GRMZM2G141510	GRMZM2G141510_P01	TRUE	TRUE	sALEATAPGLEAk	95%	n+304 (+304), K+304 (+304)	31.72	25.99
5290	seq=translation; coord=2:212802942..212807188:1; parent_transcript=GRMZM2G141510_T01; parent_gene=GRMZM2G141510	GRMZM2G141510_P01	TRUE	TRUE	yTVENLNDVSSDALLER	95%	n+304 (+304)	52.42	25.00
5291	seq=translation; coord=8:10870907..10877056:1; parent_transcript=GRMZM2G030384_T01; parent_gene=GRMZM2G030384	GRMZM2G030384_P01	TRUE	TRUE	aFVWDSGSTVGEFDGHSk	95%	n+304 (+304), K+304 (+304)	52.01	25.00
5292	seq=translation; coord=8:10870907..10877056:1; parent_transcript=GRMZM2G030384_T01; parent_gene=GRMZM2G030384	GRMZM2G030384_P01	TRUE	TRUE	gILLAGDPk	95%	n+304 (+304), K+304 (+304)	41.57	25.00
5293	seq=translation; coord=8:10870907..10877056:1; parent_transcript=GRMZM2G030384_T01; parent_gene=GRMZM2G030384	GRMZM2G030384_P01	TRUE	TRUE	tILSTSYDGVIMR	95%	n+304 (+304)	46.25	25.63
5294	seq=translation; coord=8:10870907..10877056:1; parent_transcript=GRMZM2G030384_T01; parent_gene=GRMZM2G030384	GRMZM2G030384_P01	TRUE	TRUE	vWDIFEDASGk	95%	n+304 (+304), K+304 (+304)	38.91	25.99
5295	seq=translation; coord=10:141573795..141580886:-1; parent_transcript=GRMZM2G156145_T01; parent_gene=GRMZM2G156145	GRMZM2G156145_P01	TRUE	TRUE	aTEEASGSTSSk	95%	n+304 (+304), K+304 (+304)	34.66	25.00
5296	seq=translation; coord=10:141573795..141580886:-1; parent_transcript=GRMZM2G156145_T01; parent_gene=GRMZM2G156145	GRMZM2G156145_P01	TRUE	TRUE	aTEEEENIDPR	95%	n+304 (+304)	56.10	25.00

5297	seq=translation; coord=10:141573795..141580886:-1; parent_transcript=GRMZM2G156145_T01; parent_gene=GRMZM2G156145	GRMZM2G156145_P01	TRUE	TRUE	lEAIVER	95%	n+304 (+304)	31.40	25.73
5298	seq=translation; coord=6:161231386..161234113:-1; parent_transcript=GRMZM2G159032_T01; parent_gene=GRMZM2G159032	GRMZM2G159032_P01,GRMZM2G159032_P03, GRMZM2G159032_P04	TRUE	TRUE	lAIGTLSVDk	95%	n+304 (+304), K+304 (+304)	30.25	25.00
5299	seq=translation; coord=6:161231386..161234113:-1; parent_transcript=GRMZM2G159032_T01; parent_gene=GRMZM2G159032	GRMZM2G159032_P01,GRMZM2G159032_P03, GRMZM2G159032_P04	TRUE	TRUE	sFISETALQAHSk	95%	n+304 (+304), K+304 (+304)	37.34	26.27
5300	seq=translation; coord=6:161231386..161234113:-1; parent_transcript=GRMZM2G159032_T01; parent_gene=GRMZM2G159032	GRMZM2G159032_P01,GRMZM2G159032_P03, GRMZM2G159032_P04	TRUE	TRUE	tTSVFFTGYk	92%	n+304 (+304), K+304 (+304)	27.85	26.40
5301	seq=translation; coord=5:183882422..183889051:-1; parent_transcript=GRMZM2G142088_T01; parent_gene=GRMZM2G142088	GRMZM2G142088_P01,GRMZM2G142088_P02	TRUE	TRUE	dVFSLIVEAFk	95%	n+304 (+304), K+304 (+304)	31.54	25.00
5302	seq=translation; coord=5:183882422..183889051:-1; parent_transcript=GRMZM2G142088_T01; parent_gene=GRMZM2G142088	GRMZM2G142088_P01,GRMZM2G142088_P02	TRUE	TRUE	vAYDDGDVEVLLLR	95%	n+304 (+304)	49.25	25.45
5303	seq=translation; coord=5:204089494..204100246:-1; parent_transcript=GRMZM2G029186_T01; parent_gene=GRMZM2G029186	GRMZM2G029186_P01,GRMZM2G029186_P02, GRMZM2G029186_P03,GRMZM2G029186_P04	TRUE	TRUE	eISSLGPVVAR	94%	n+304 (+304)	32.80	26.58
5304	seq=translation; coord=5:204089494..204100246:-1; parent_transcript=GRMZM2G029186_T01; parent_gene=GRMZM2G029186	GRMZM2G029186_P01,GRMZM2G029186_P02, GRMZM2G029186_P03,GRMZM2G029186_P04	TRUE	TRUE	sSLDQTVEMISER	95%	n+304 (+304)	36.75	25.00
5305	seq=translation; coord=5:74450298..74452700:-1; parent_transcript=GRMZM5G831200_T01; parent_gene=GRMZM5G831200	GRMZM5G831200_P01	TRUE	TRUE	gGAFAAVADVALDR	93%	n+304 (+304)	27.03	25.47
5306	seq=translation; coord=5:74450298..74452700:-1; parent_transcript=GRMZM5G831200_T01; parent_gene=GRMZM5G831200	GRMZM5G831200_P01	TRUE	TRUE	lWLLFSGLAGAR	95%	n+304 (+304)	41.52	25.24
5307	seq=translation; coord=5:74450298..74452700:-1; parent_transcript=GRMZM5G831200_T01; parent_gene=GRMZM5G831200	GRMZM5G831200_P01	TRUE	TRUE	rADDDGEVQTVcNcAVGLDYGR	95%	n+304 (+304), Carbamidomethyl (+57), Carbamidomethyl (+57)	67.46	25.00
5308	seq=translation; coord=3:133205499..133209390:-1; parent_transcript=GRMZM2G136262_T01; parent_gene=GRMZM2G136262	GRMZM2G136262_P01	TRUE	TRUE	eQLALPDGTAITGESk	95%	n+304 (+304), K+304 (+304)	49.74	25.88
5309	seq=translation; coord=3:133205499..133209390:-1; parent_transcript=GRMZM2G136262_T01; parent_gene=GRMZM2G136262	GRMZM2G136262_P01	TRUE	TRUE	lMLEAPPk	94%	n+304 (+304), K+304 (+304)	27.92	25.00
5310	seq=translation; coord=6:131028969..131032495:1; parent_transcript=GRMZM2G133631_T01; parent_gene=GRMZM2G133631	GRMZM2G133631_P01,GRMZM2G165567_P01, GRMZM2G165567_P02,GRMZM2G165567_P03, GRMZM2G165567_P04,GRMZM2G405064_P01	TRUE	TRUE	aPVHLVWELVR	95%	n+304 (+304)	49.83	25.56
5311	seq=translation; coord=6:131028969..131032495:1; parent_transcript=GRMZM2G133631_T01; parent_gene=GRMZM2G133631	GRMZM2G133631_P01,GRMZM2G165567_P01, GRMZM2G165567_P02,GRMZM2G165567_P03, GRMZM2G165567_P04,GRMZM2G405064_P01	TRUE	TRUE	gDQLEVGSLR	86%	n+304 (+304)	25.81	25.74
5312	seq=translation; coord=3:153925171..153929056:-1; parent_transcript=GRMZM2G145715_T01; parent_gene=GRMZM2G145715	GRMZM2G145715_P01	TRUE	TRUE	nPNLANLLVDPEFAQEIIDR	95%	n+304 (+304)	48.35	25.48
5313	seq=translation; coord=6:140465764..140469654:1; parent_transcript=AC213884.3_FGT001; parent_gene=AC213884.3_FG001	AC213884.3_FGP001	TRUE	TRUE	icANHLVAPTTR	95%	n+304 (+304), Carbamidomethyl (+57)	36.81	25.43

5314	seq=translation; coord=6:140465764..140469654:1; parent_transcript=AC213884.3_FGT001; parent_gene=AC213884.3_FG001	AC213884.3_FGP001	TRUE	TRUE	lEEVLVTTGEEDEDVLLDMk	95%	n+304 (+304), K+304 (+304)	45.13	25.00
5315	seq=translation; coord=10:23245983..23251624:-1; parent_transcript=GRMZM2G013478_T01; parent_gene=GRMZM2G013478	GRMZM2G013478_P01	TRUE	TRUE	eIELWFk	92%	n+304 (+304), K+304 (+304)	25.75	25.00
5316	seq=translation; coord=10:23245983..23251624:-1; parent_transcript=GRMZM2G013478_T01; parent_gene=GRMZM2G013478	GRMZM2G013478_P01	TRUE	TRUE	kLIGATNPLQAEPGTIR	95%	K+304 (+304), n+304 (+304)	49.73	25.00
5317	seq=translation; coord=9:153591547..153607432:1; parent_transcript=GRMZM2G051219_T01; parent_gene=GRMZM2G051219	GRMZM2G051219_P01,GRMZM2G051219_P02	TRUE	TRUE	aINTLESWIk	95%	n+304 (+304), K+304 (+304)	43.42	25.25
5318	seq=translation; coord=9:153591547..153607432:1; parent_transcript=GRMZM2G051219_T01; parent_gene=GRMZM2G051219	GRMZM2G051219_P01,GRMZM2G051219_P02	TRUE	TRUE	gAIADALVLNAAASLLVSGk	95%	n+304 (+304), K+304 (+304)	29.72	25.00
5319	seq=translation; coord=9:153591547..153607432:1; parent_transcript=GRMZM2G051219_T01; parent_gene=GRMZM2G051219	GRMZM2G051219_P01,GRMZM2G051219_P02	TRUE	TRUE	iSAFLVLLR	94%	n+304 (+304)	35.95	30.23
5320	seq=translation; coord=4:235606747..235623515:-1; parent_transcript=GRMZM2G119482_T01; parent_gene=GRMZM2G119482	GRMZM2G119482_P01	TRUE	TRUE	aFIQAVQSLk	95%	n+304 (+304), K+304 (+304)	47.98	25.00
5321	seq=translation; coord=4:235606747..235623515:-1; parent_transcript=GRMZM2G119482_T01; parent_gene=GRMZM2G119482	GRMZM2G119482_P01	TRUE	TRUE	eEVFGPVAPLIPFk	95%	n+304 (+304), K+304 (+304)	36.69	25.00
5322	seq=translation; coord=4:235606747..235623515:-1; parent_transcript=GRMZM2G119482_T01; parent_gene=GRMZM2G119482	GRMZM2G119482_P01	TRUE	TRUE	qPVGVVGAITPWNFPLAMITR	95%	n+304 (+304)	52.35	25.11
5323	seq=translation; coord=5:214424827..214427180:1; parent_transcript=GRMZM2G030523_T01; parent_gene=GRMZM2G030523	GRMZM2G030523_P01	TRUE	TRUE	aDDGSDTVTFMFESPk	95%	n+304 (+304), K+304 (+304)	33.33	25.00
5324	seq=translation; coord=6:102399027..102412128:1; parent_transcript=GRMZM2G106427_T01; parent_gene=GRMZM2G106427	GRMZM2G106427_P01,GRMZM2G106427_P02, GRMZM2G106427_P03,GRMZM2G106427_P04	TRUE	TRUE	qDAAQAIALVLWk	95%	n+304 (+304), K+304 (+304)	30.59	25.04
5325	seq=translation; coord=6:102399027..102412128:1; parent_transcript=GRMZM2G106427_T01; parent_gene=GRMZM2G106427	GRMZM2G106427_P01,GRMZM2G106427_P02, GRMZM2G106427_P03,GRMZM2G106427_P04	TRUE	TRUE	sIVAEDDLDQR	95%	n+304 (+304)	46.22	25.00
5326	seq=translation; coord=6:102399027..102412128:1; parent_transcript=GRMZM2G106427_T01; parent_gene=GRMZM2G106427	GRMZM2G106427_P01,GRMZM2G106427_P02, GRMZM2G106427_P03,GRMZM2G106427_P04	TRUE	TRUE	wQEATGESVSEVIFLSk	95%	n+304 (+304), K+304 (+304)	44.61	25.99
5327	seq=translation; coord=1:46061768..46066293:1; parent_transcript=GRMZM2G058138_T01; parent_gene=GRMZM2G058138	GRMZM2G058138_P01,GRMZM2G058138_P02, GRMZM2G058138_P03	TRUE	TRUE	lIVADPDAILDVLTYEEk	95%	n+304 (+304), K+304 (+304)	39.72	25.00
5328	seq=translation; coord=1:46061768..46066293:1; parent_transcript=GRMZM2G058138_T01; parent_gene=GRMZM2G058138	GRMZM2G058138_P01,GRMZM2G058138_P02, GRMZM2G058138_P03	TRUE	TRUE	vVPAVTPEVk	95%	n+304 (+304), K+304 (+304)	38.24	25.00
5329	seq=translation; coord=4:102065832..102092090:1; parent_transcript=GRMZM2G028766_T01; parent_gene=GRMZM2G028766	GRMZM2G028766_P01,GRMZM2G028766_P02	TRUE	TRUE	eMLTDLEGMGFSTAR	95%	n+304 (+304)	76.77	25.00
5330	seq=translation; coord=4:102065832..102092090:1; parent_transcript=GRMZM2G028766_T01; parent_gene=GRMZM2G028766	GRMZM2G028766_P01,GRMZM2G028766_P02	TRUE	TRUE	vGNLHVGIEFLELcGFEk	95%	n+304 (+304), Carbamidomethyl (+57), K+304 (+304)	31.23	25.72
5331	seq=translation; coord=5:176347442..176349538:1; parent_transcript=GRMZM2G056629_T01; parent_gene=GRMZM2G056629	GRMZM2G056629_P01	TRUE	TRUE	rGTGGVAGYDTNYAVDGAASPSLRPVAR	95%	n+304 (+304)	48.94	25.00

5332	seq=translation; coord=5:176347442..176349538:1; parent_transcript=GRMZM2G056629_T01; parent_gene=GRMZM2G056629	GRMZM2G056629_P01	TRUE	TRUE	vTNWGATLMSVILPDSk	95%	n+304 (+304), K+304 (+304)	40.13	26.23
5333	seq=translation; coord=6:160040980..160045154:-1; parent_transcript=GRMZM2G005036_T01; parent_gene=GRMZM2G005036	GRMZM2G005036_P01	TRUE	TRUE	aVDLAAGELLEIATPGk	95%	n+304 (+304), K+304 (+304)	53.62	25.00
5334	seq=translation; coord=6:160040980..160045154:-1; parent_transcript=GRMZM2G005036_T01; parent_gene=GRMZM2G005036	GRMZM2G005036_P01	TRUE	TRUE	eQLQNIk	95%	n+304 (+304), K+304 (+304)	34.00	25.00
5335	seq=translation; coord=6:160040980..160045154:-1; parent_transcript=GRMZM2G005036_T01; parent_gene=GRMZM2G005036	GRMZM2G005036_P01	TRUE	TRUE	lDVSSLEEFVAEHyTAPR	95%	n+304 (+304), iTRAQ8plex (+304)	41.26	25.25
5336	seq=translation; coord=6:160040980..160045154:-1; parent_transcript=GRMZM2G005036_T01; parent_gene=GRMZM2G005036	GRMZM2G005036_P01	TRUE	TRUE	qSSGGLFSWLLGGk	95%	n+304 (+304), K+304 (+304)	37.15	25.99
5337	seq=translation; coord=6:160040980..160045154:-1; parent_transcript=GRMZM2G005036_T01; parent_gene=GRMZM2G005036	GRMZM2G005036_P01	TRUE	TRUE	sSELPPLDVPLPGISIPSPLPDFVEPSk	95%	n+304 (+304), K+304 (+304)	30.38	25.00
5338	seq=translation; coord=6:160040980..160045154:-1; parent_transcript=GRMZM2G005036_T01; parent_gene=GRMZM2G005036	GRMZM2G005036_P01	TRUE	TRUE	sYTPEMVEVLIDSVR	94%	n+304 (+304)	31.05	25.00
5339	seq=translation; coord=1:274275100..274280175:1; parent_transcript=GRMZM2G135283_T03; parent_gene=GRMZM2G135283	GRMZM2G135283_P03,GRMZM2G135283_P04	TRUE	TRUE	gFVEEDFAk	95%	n+304 (+304), K+304 (+304)	32.99	25.56
5340	seq=translation; coord=1:274275100..274280175:1; parent_transcript=GRMZM2G135283_T03; parent_gene=GRMZM2G135283	GRMZM2G135283_P03,GRMZM2G135283_P04	TRUE	TRUE	gLELIPSENFTSVSVMQAVGSVMTNk	95%	n+304 (+304), K+304 (+304)	38.86	25.00
5341	seq=translation; coord=1:274275100..274280175:1; parent_transcript=GRMZM2G135283_T03; parent_gene=GRMZM2G135283	GRMZM2G135283_P03,GRMZM2G135283_P04	TRUE	TRUE	gYELVSGGTDNHLVLVNLk	94%	n+304 (+304), K+304 (+304)	26.28	25.24
5342	seq=translation; coord=1:274275100..274280175:1; parent_transcript=GRMZM2G135283_T03; parent_gene=GRMZM2G135283	GRMZM2G135283_P03,GRMZM2G135283_P04	TRUE	TRUE	iSATSIFFETMPYR	95%	n+304 (+304)	43.41	25.00
5343	seq=translation; coord=1:274275100..274280175:1; parent_transcript=GRMZM2G135283_T03; parent_gene=GRMZM2G135283	GRMZM2G135283_P03,GRMZM2G135283_P04	TRUE	TRUE	vADFFDAAVNLALk	92%	n+304 (+304), K+304 (+304)	26.79	25.00
5344	seq=translation; coord=1:191893507..191894902:1; parent_transcript=GRMZM2G427451_T01; parent_gene=GRMZM2G427451	GRMZM2G427451_P01	TRUE	TRUE	aSTPLDFPATDLAADAAGGk	95%	n+304 (+304), K+304 (+304)	49.57	25.00
5345	seq=translation; coord=1:191893507..191894902:1; parent_transcript=GRMZM2G427451_T01; parent_gene=GRMZM2G427451	GRMZM2G427451_P01	TRUE	TRUE	aYAScEDLPk	95%	n+304 (+304), Carbamidomethyl (+57), K+304 (+304)	37.22	25.00
5346	seq=translation; coord=1:191893507..191894902:1; parent_transcript=GRMZM2G427451_T01; parent_gene=GRMZM2G427451	GRMZM2G427451_P01	TRUE	TRUE	hAFQADNLGAk	95%	n+304 (+304), K+304 (+304)	50.37	25.00
5347	seq=translation; coord=1:56746484..56752553:1; parent_transcript=GRMZM2G010823_T01; parent_gene=GRMZM2G010823	GRMZM2G010823_P01	TRUE	TRUE	eAALDLGVLTEQEFHELVVPEk	95%	n+304 (+304), K+304 (+304)	48.01	25.00
5348	seq=translation; coord=1:56746484..56752553:1; parent_transcript=GRMZM2G010823_T01; parent_gene=GRMZM2G010823	GRMZM2G010823_P01	TRUE	TRUE	mYQLAQGGTAVGTGLNTk	94%	n+304 (+304), K+304 (+304)	29.57	25.92
5349	seq=translation; coord=1:56746484..56752553:1; parent_transcript=GRMZM2G010823_T01; parent_gene=GRMZM2G010823	GRMZM2G010823_P01	TRUE	TRUE	sLQNFDIGGER	95%	n+304 (+304)	39.35	25.00

5350	seq=translation; coord=8:116304892..116311147:-1; parent_transcript=GRMZM2G055489_T01; parent_gene=GRMZM2G055489	GRMZM2G055489_P01,GRMZM2G055489_P02, GRMZM2G055489_P04	TRUE	TRUE	fDLWESEGDVR	95%	n+304 (+304)	34.06	25.00
5351	seq=translation; coord=8:116304892..116311147:-1; parent_transcript=GRMZM2G055489_T01; parent_gene=GRMZM2G055489	GRMZM2G055489_P01,GRMZM2G055489_P02, GRMZM2G055489_P04	TRUE	TRUE	gQALAYLLk	95%	n+304 (+304), K+304 (+304)	35.96	25.00
5352	seq=translation; coord=5:56740173..56751799:1; parent_transcript=GRMZM2G115939_T01; parent_gene=GRMZM2G115939	GRMZM2G115939_P01,GRMZM2G115939_P02, GRMZM2G115939_P04	TRUE	TRUE	aMTGGYVGQHYFQR	94%	n+304 (+304)	28.49	25.00
5353	seq=translation; coord=5:56740173..56751799:1; parent_transcript=GRMZM2G115939_T01; parent_gene=GRMZM2G115939	GRMZM2G115939_P01,GRMZM2G115939_P02, GRMZM2G115939_P04	TRUE	TRUE	dISELNLPk	95%	n+304 (+304), K+304 (+304)	36.25	25.00
5354	seq=translation; coord=4:163464001..163467089:-1; parent_transcript=GRMZM2G072612_T01; parent_gene=GRMZM2G072612	GRMZM2G072612_P01,GRMZM2G072612_P02	TRUE	TRUE	dTGALLVMDPR	95%	n+304 (+304)	34.21	25.00
5355	seq=translation; coord=4:163464001..163467089:-1; parent_transcript=GRMZM2G072612_T01; parent_gene=GRMZM2G072612	GRMZM2G072612_P01,GRMZM2G072612_P02	TRUE	TRUE	fLDVVER	95%	n+304 (+304)	37.79	25.00
5356	seq=translation; coord=10:106434010..106436707:-1; parent_transcript=GRMZM2G139512_T01; parent_gene=GRMZM2G139512	GRMZM2G139512_P01,GRMZM2G139512_P02, GRMZM2G139512_P03,GRMZM2G139512_P04	TRUE	TRUE	aDLEFLVGLLk	95%	n+304 (+304), K+304 (+304)	59.88	25.00
5357	seq=translation; coord=10:106434010..106436707:-1; parent_transcript=GRMZM2G139512_T01; parent_gene=GRMZM2G139512	GRMZM2G139512_P01,GRMZM2G139512_P02, GRMZM2G139512_P03,GRMZM2G139512_P04	TRUE	TRUE	rPPEVSAAEGAGLPIAAGTALQALR	95%	n+304 (+304)	35.09	25.00
5358	seq=translation; coord=9:146914705..146918889:-1; parent_transcript=GRMZM2G321725_T02; parent_gene=GRMZM2G321725	GRMZM2G321725_P02	TRUE	TRUE	gLWQPFTALLGDAPSVDVk	95%	n+304 (+304), K+304 (+304)	54.48	26.03
5359	seq=translation; coord=3:223741308..223745402:-1; parent_transcript=GRMZM2G157588_T01; parent_gene=GRMZM2G157588	GRMZM2G157588_P01	TRUE	TRUE	lETWSDSFQEAFDR	95%	n+304 (+304)	35.58	25.00
5360	seq=translation; coord=7:164069905..164076083:1; parent_transcript=GRMZM2G136918_T01; parent_gene=GRMZM2G136918	GRMZM2G136918_P01,GRMZM2G136918_P02, GRMZM2G136918_P03,GRMZM2G136918_P04, GRMZM2G136918_P05	TRUE	TRUE	aVHIAVSDMAEGLDAPTSLPAR	95%	n+304 (+304)	29.92	25.21
5361	seq=translation; coord=7:164069905..164076083:1; parent_transcript=GRMZM2G136918_T01; parent_gene=GRMZM2G136918	GRMZM2G136918_P01,GRMZM2G136918_P02, GRMZM2G136918_P03,GRMZM2G136918_P04, GRMZM2G136918_P05	TRUE	TRUE	lVWFLDk	93%	n+304 (+304), K+304 (+304)	30.14	25.00
5362	seq=translation; coord=8:161837476..161841019:1; parent_transcript=GRMZM2G180399_T01; parent_gene=GRMZM2G180399	GRMZM2G180399_P01,GRMZM5G852502_P01	TRUE	TRUE	dYADILEFLVDR	93%	n+304 (+304)	29.57	25.00
5363	seq=translation; coord=10:148101348..148102945:1; parent_transcript=GRMZM5G892522_T01; parent_gene=GRMZM5G892522	GRMZM5G892522_P01	TRUE	TRUE	lIAPVVDWASEEYDGR	95%	n+304 (+304)	53.52	25.00
5364	seq=translation; coord=10:148101348..148102945:1; parent_transcript=GRMZM5G892522_T01; parent_gene=GRMZM5G892522	GRMZM5G892522_P01	TRUE	TRUE	vYGLPTLIFFk	92%	n+304 (+304), K+304 (+304)	26.87	25.00
5365	seq=translation; coord=4:154310162..154311883:-1; parent_transcript=GRMZM2G373522_T01; parent_gene=GRMZM2G373522	GRMZM2G373522_P01	TRUE	TRUE	iEDDDTk	94%	n+304 (+304), K+304 (+304)	31.13	25.00
5366	seq=translation; coord=4:154310162..154311883:-1; parent_transcript=GRMZM2G373522_T01; parent_gene=GRMZM2G373522	GRMZM2G373522_P01	TRUE	TRUE	nTQQHQGGEQAQDQENEVk	95%	n+304 (+304), K+304 (+304)	29.44	25.00
5367	seq=translation; coord=5:113730181..113780006:1; parent_transcript=GRMZM2G056661_T01; parent_gene=GRMZM2G056661	GRMZM2G056661_P01,GRMZM2G056661_P03	TRUE	TRUE	eLLSVIEAALLGSTAPSPAQR	95%	n+304 (+304)	37.05	25.00

5368	seq=translation; coord=5:113730181..113780006:1; parent_transcript=GRMZM2G056661_T01; parent_gene=GRMZM2G056661	GRMZM2G056661_P01,GRMZM2G056661_P03	TRUE	TRUE	vLDLLEVLQFR	95%	n+304 (+304)	42.26	25.00
5369	seq=translation; coord=7:170419371..170421238:-1; parent_transcript=GRMZM2G111672_T01; parent_gene=GRMZM2G111672	GRMZM2G111672_P01	TRUE	TRUE	dLAEFGTGLR	95%	n+304 (+304)	31.43	25.00
5370	seq=translation; coord=8:173103213..173107748:1; parent_transcript=GRMZM2G124365_T01; parent_gene=GRMZM2G124365	GRMZM2G124365_P01	TRUE	TRUE	eVEVAYLLk	95%	n+304 (+304), K+304 (+304)	32.49	25.00
5371	seq=translation; coord=8:173103213..173107748:1; parent_transcript=GRMZM2G124365_T01; parent_gene=GRMZM2G124365	GRMZM2G124365_P01	TRUE	TRUE	lEDSIIFGLLER	95%	n+304 (+304)	44.95	25.26
5372	seq=translation; coord=5:4495054..4499934:-1; parent_transcript=GRMZM2G007260_T01; parent_gene=GRMZM2G007260	GRMZM2G007260_P01,GRMZM2G007260_P02, GRMZM2G007260_P03,GRMZM2G007260_P04, GRMZM2G022206_P02,GRMZM2G074479_P01, GRMZM2G074479_P02,GRMZM2G074479_P03	TRUE	TRUE	lTLQFTEDYPNkPPTVR	95%	n+304 (+304), K+304 (+304)	31.18	25.65
5373	seq=translation; coord=8:28159275..28161158:-1; parent_transcript=GRMZM2G032348_T04; parent_gene=GRMZM2G032348	GRMZM2G032348_P04,GRMZM2G032348_P05	TRUE	TRUE	eMVAALADGR	95%	n+304 (+304)	42.25	25.00
5374	seq=translation; coord=2:147034275..147039199:-1; parent_transcript=GRMZM2G095392_T01; parent_gene=GRMZM2G095392	GRMZM2G095392_P01,GRMZM2G095392_P02, GRMZM2G140047_P01	TRUE	TRUE	aFDLLDMLk	95%	n+304 (+304), K+304 (+304)	35.69	25.63
5375	seq=translation; coord=5:202307973..202315571:-1; parent_transcript=GRMZM2G164948_T01; parent_gene=GRMZM2G164948	GRMZM2G164948_P01,GRMZM2G164948_P02, GRMZM2G164948_P03	TRUE	TRUE	vSADSTTVLADAVIVATGAVAR	95%	n+304 (+304)	54.49	25.40
5376	seq=translation; coord=1:60216405..60219476:-1; parent_transcript=GRMZM2G144995_T01; parent_gene=GRMZM2G144995	GRMZM2G144995_P01,GRMZM2G144995_P02	TRUE	TRUE	aSMGQDLSPVDLINVQQFAQR	95%	n+304 (+304)	72.12	25.00
5377	seq=translation; coord=1:60216405..60219476:-1; parent_transcript=GRMZM2G144995_T01; parent_gene=GRMZM2G144995	GRMZM2G144995_P01,GRMZM2G144995_P02	TRUE	TRUE	mNDIAPNLTSLIGEVVGAR	95%	n+304 (+304)	61.73	25.71
5378	seq=translation; coord=5:64107900..64117086:1; parent_transcript=GRMZM2G145088_T01; parent_gene=GRMZM2G145088	GRMZM2G145088_P01	TRUE	TRUE	gSGLPQPVLAQIWTYADk	95%	n+304 (+304), K+304 (+304)	56.79	25.67
5379	seq=translation; coord=5:64107900..64117086:1; parent_transcript=GRMZM2G145088_T01; parent_gene=GRMZM2G145088	GRMZM2G145088_P01	TRUE	TRUE	iSGQEAVAFFk	93%	n+304 (+304), K+304 (+304)	27.28	25.19
5380	seq=translation; coord=5:64107900..64117086:1; parent_transcript=GRMZM2G145088_T01; parent_gene=GRMZM2G145088	GRMZM2G145088_P01	TRUE	TRUE	lTMDEATFR	94%	n+304 (+304)	31.02	25.00
5381	seq=translation; coord=4:238325251..238328383:-1; parent_transcript=GRMZM2G060567_T01; parent_gene=GRMZM2G060567	GRMZM2G060567_P01	TRUE	TRUE	dISSSVTAIANEk	95%	n+304 (+304), K+304 (+304)	44.56	26.02
5382	seq=translation; coord=4:238325251..238328383:-1; parent_transcript=GRMZM2G060567_T01; parent_gene=GRMZM2G060567	GRMZM2G060567_P01	TRUE	TRUE	gDDDFIPGR	88%	n+304 (+304)	26.29	25.00
5383	seq=translation; coord=4:238325251..238328383:-1; parent_transcript=GRMZM2G060567_T01; parent_gene=GRMZM2G060567	GRMZM2G060567_P01	TRUE	TRUE	lVEEADFk	95%	n+304 (+304), K+304 (+304)	34.30	26.43
5384	seq=translation; coord=4:238325251..238328383:-1; parent_transcript=GRMZM2G060567_T01; parent_gene=GRMZM2G060567	GRMZM2G060567_P01	TRUE	TRUE	sESDFAEYAELIANk	95%	n+304 (+304), K+304 (+304)	43.36	25.00

5385	seq=translation; coord=7:106230652..106231840:1; parent_transcript=GRMZM2G025646_T01; parent_gene=GRMZM2G025646	GRMZM2G025646_P01,GRMZM2G025646_P02	TRUE	TRUE	sFGEVQGILEHNR	95%	n+304 (+304)	60.80	25.00
5386	seq=translation; coord=7:106230652..106231840:1; parent_transcript=GRMZM2G025646_T01; parent_gene=GRMZM2G025646	GRMZM2G025646_P01,GRMZM2G025646_P02	TRUE	TRUE	vLIQEISQNQETR	95%	n+304 (+304)	45.10	25.37
5387	seq=translation; coord=7:106230652..106231840:1; parent_transcript=GRMZM2G025646_T01; parent_gene=GRMZM2G025646	GRMZM2G025646_P01,GRMZM2G025646_P02	TRUE	TRUE	vVDLYGDMSGSFAR	95%	n+304 (+304)	66.87	25.00
5388	seq=translation; coord=3:22814532..22820273:-1; parent_transcript=GRMZM2G009282_T01; parent_gene=GRMZM2G009282	GRMZM2G009282_P01	TRUE	TRUE	dPFWFAVHR	95%	n+304 (+304)	48.23	25.00
5389	seq=translation; coord=3:22814532..22820273:-1; parent_transcript=GRMZM2G009282_T01; parent_gene=GRMZM2G009282	GRMZM2G009282_P01	TRUE	TRUE	lLAFLDk	91%	n+304 (+304), K+304 (+304)	26.27	25.00
5390	seq=translation; coord=3:22814532..22820273:-1; parent_transcript=GRMZM2G009282_T01; parent_gene=GRMZM2G009282	GRMZM2G009282_P01	TRUE	TRUE	wIELGAFYPFSR	90%	n+304 (+304)	25.28	25.00
5391	seq=translation; coord=2:130291275..130295558:-1; parent_transcript=GRMZM2G118800_T01; parent_gene=GRMZM2G118800	GRMZM2G118800_P01	TRUE	TRUE	fYGEDPLQSADLSR	95%	n+304 (+304)	61.71	25.00
5392	seq=translation; coord=2:130291275..130295558:-1; parent_transcript=GRMZM2G118800_T01; parent_gene=GRMZM2G118800	GRMZM2G118800_P01	TRUE	TRUE	sFAPELVASLk	95%	n+304 (+304), K+304 (+304)	34.55	25.65
5393	seq=translation; coord=4:172748163..172754261:1; parent_transcript=GRMZM2G178398_T01; parent_gene=GRMZM2G178398	GRMZM2G178398_P01	TRUE	TRUE	aNPHADIGVDFDSINR	95%	n+304 (+304)	47.29	25.00
5394	seq=translation; coord=4:172748163..172754261:1; parent_transcript=GRMZM2G178398_T01; parent_gene=GRMZM2G178398	GRMZM2G178398_P01	TRUE	TRUE	gLIVLDYLVAHGTER	95%	n+304 (+304)	54.93	25.19
5395	seq=translation; coord=4:172748163..172754261:1; parent_transcript=GRMZM2G178398_T01; parent_gene=GRMZM2G178398	GRMZM2G178398_P01	TRUE	TRUE	gLVNLDISGPk	95%	n+304 (+304), K+304 (+304)	40.15	25.09
5396	seq=translation; coord=5:182124005..182130631:-1; parent_transcript=GRMZM2G137409_T01; parent_gene=GRMZM2G137409	GRMZM2G137409_P01	TRUE	TRUE	aIAMEGATDALIQLIk	95%	n+304 (+304), K+304 (+304)	59.39	25.00
5397	seq=translation; coord=5:182124005..182130631:-1; parent_transcript=GRMZM2G137409_T01; parent_gene=GRMZM2G137409	GRMZM2G137409_P01	TRUE	TRUE	sAIGIGTLLMDGLGDTIR	95%	n+304 (+304)	42.93	25.00
5398	seq=translation; coord=5:182124005..182130631:-1; parent_transcript=GRMZM2G137409_T01; parent_gene=GRMZM2G137409	GRMZM2G137409_P01	TRUE	TRUE	tLFDLQEISAEIR	95%	n+304 (+304)	39.31	25.37
5399	seq=translation; coord=1:293041177..293042705:-1; parent_transcript=GRMZM2G155329_T01; parent_gene=GRMZM2G155329	GRMZM2G155329_P01	TRUE	TRUE	fTAIGVYLEDAAVPALAk	95%	n+304 (+304), K+304 (+304)	66.08	25.00
5400	seq=translation; coord=1:293041177..293042705:-1; parent_transcript=GRMZM2G155329_T01; parent_gene=GRMZM2G155329	GRMZM2G155329_P01	TRUE	TRUE	lcEAVLESIIGER	95%	n+304 (+304), Carbamidomethyl (+57)	40.64	25.90
5401	seq=translation; coord=1:293041177..293042705:-1; parent_transcript=GRMZM2G155329_T01; parent_gene=GRMZM2G155329	GRMZM2G155329_P01	TRUE	TRUE	tADELASDAAFFR	95%	n+304 (+304)	65.25	25.00
5402	seq=translation; coord=7:156569238..156573041:1; parent_transcript=GRMZM2G153162_T02; parent_gene=GRMZM2G153162	GRMZM2G153162_P02	TRUE	TRUE	iSSEPPNSASSVEDk	95%	n+304 (+304), K+304 (+304)	40.36	25.21

5403	seq=translation; coord=7:156569238..156573041:1; parent_transcript=GRMZM2G153162_T02; parent_gene=GRMZM2G153162	GRMZM2G153162_P02	TRUE	TRUE	tSSAPPNLDEQk	94%	n+304 (+304), K+304 (+304)	27.14	25.15
5404	seq=translation; coord=3:123772202..123775285:1; parent_transcript=GRMZM2G164868_T01; parent_gene=GRMZM2G164868	GRMZM2G164868_P01,GRMZM2G164868_P02	TRUE	TRUE	aAVVASVAAFLESAGLSR	95%	n+304 (+304)	46.76	25.73
5405	seq=translation; coord=3:123772202..123775285:1; parent_transcript=GRMZM2G164868_T01; parent_gene=GRMZM2G164868	GRMZM2G164868_P01,GRMZM2G164868_P02	TRUE	TRUE	aELAILNk	92%	n+304 (+304), K+304 (+304)	27.98	27.15
5406	seq=translation; coord=3:123772202..123775285:1; parent_transcript=GRMZM2G164868_T01; parent_gene=GRMZM2G164868	GRMZM2G164868_P01,GRMZM2G164868_P02	TRUE	TRUE	tDAGSAPAEADGAk	95%	n+304 (+304), K+304 (+304)	37.78	25.85
5407	seq=translation; coord=4:163261154..163272206:1; parent_transcript=GRMZM2G014994_T01; parent_gene=GRMZM2G014994	GRMZM2G014994_P01	TRUE	TRUE	fPAGPccAALk	95%	n+304 (+304), Carbamidomethyl (+57), Carbamidomethyl (+57), K+304 (+304)	35.57	25.00
5408	seq=translation; coord=4:163261154..163272206:1; parent_transcript=GRMZM2G014994_T01; parent_gene=GRMZM2G014994	GRMZM2G014994_P01	TRUE	TRUE	qGLScADVLER	95%	n+304 (+304), Carbamidomethyl (+57)	46.26	25.00
5409	seq=translation; coord=10:12167272..12169103:-1; parent_transcript=GRMZM2G094375_T01; parent_gene=GRMZM2G094375	GRMZM2G094375_P01,GRMZM2G094375_P02	TRUE	TRUE	aTVYGALVLRPR	95%	n+304 (+304)	37.70	25.24
5410	seq=translation; coord=10:12167272..12169103:-1; parent_transcript=GRMZM2G094375_T01; parent_gene=GRMZM2G094375	GRMZM2G094375_P01,GRMZM2G094375_P02	TRUE	TRUE	iINAALNTPLFFk	95%	n+304 (+304), K+304 (+304)	50.11	25.00
5411	seq=translation; coord=3:231890529..231892088:-1; parent_transcript=GRMZM2G175076_T01; parent_gene=GRMZM2G175076	GRMZM2G175076_P01	TRUE	TRUE	gSQYGVQLESSVR	95%	n+304 (+304)	50.82	25.00
5412	seq=translation; coord=3:231890529..231892088:-1; parent_transcript=GRMZM2G175076_T01; parent_gene=GRMZM2G175076	GRMZM2G175076_P01	TRUE	TRUE	lAAADLYEDDEEEALEk	95%	n+304 (+304), K+304 (+304)	78.59	25.00
5413	seq=translation; coord=6:167749462..167750447:-1; parent_transcript=GRMZM5G849087_T01; parent_gene=GRMZM5G849087	GRMZM5G849087_P01	TRUE	TRUE	aAAAAYDYEGDAR	95%	n+304 (+304)	59.24	25.00
5414	seq=translation; coord=1:208529333..208544044:-1; parent_transcript=GRMZM2G050583_T01; parent_gene=GRMZM2G050583	GRMZM2G050583_P01	TRUE	TRUE	aVSTAIYFLLPAGSVSR	95%	n+304 (+304)	31.12	25.68
5415	seq=translation; coord=1:41101054..41103817:1; parent_transcript=GRMZM2G428391_T01; parent_gene=GRMZM2G428391	GRMZM2G428391_P01	TRUE	TRUE	sINPDEAVAYGAAVQAAILTGEGNEk	95%	n+304 (+304), K+304 (+304)	49.57	25.00
5416	seq=translation; coord=3:203752020..203753539:1; parent_transcript=GRMZM2G380088_T01; parent_gene=GRMZM2G380088	GRMZM2G380088_P01	TRUE	TRUE	aAAAAGVATVQDLLPLLVPSAR	95%	n+304 (+304)	39.90	25.00
5417	seq=translation; coord=2:36927268..36929809:1; parent_transcript=GRMZM2G022413_T01; parent_gene=GRMZM2G022413	GRMZM2G022413_P01	TRUE	TRUE	eWQDAIVTEVLPAR	95%	n+304 (+304)	39.13	25.29
5418	seq=translation; coord=7:134007495..134008927:1; parent_transcript=GRMZM2G101711_T01; parent_gene=GRMZM2G101711	GRMZM2G101711_P01,GRMZM2G101711_P02	TRUE	TRUE	sSASMESEVIR	89%	n+304 (+304)	26.69	25.00
5419	seq=translation; coord=7:167020090..167021470:-1; parent_transcript=GRMZM2G171597_T01; parent_gene=GRMZM2G171597	GRMZM2G171597_P01	TRUE	TRUE	aAGLPAAcGEDPAAFSR	95%	n+304 (+304), Carbamidomethyl (+57)	75.64	25.00

5420	seq=translation; coord=4:211079286..211080981:-1; parent_transcript=GRMZM5G889769_T01; parent_gene=GRMZM5G889769	GRMZM5G889769_P01	TRUE	TRUE	sTTVLADAVIVATGAVAR	95%	n+304 (+304)	54.14	25.00
5421	AC196426.3_FGP007-R	AC196426.3_FGP007-R	TRUE	TRUE	mVVER	87%	n+304 (+304)	25.82	25.21
5422	seq=translation; coord=6:166587013..166592611:1; parent_transcript=GRMZM2G027955_T01; parent_gene=GRMZM2G027955	GRMZM2G027955_P01,GRMZM2G027955_P02, GRMZM2G027955_P03	TRUE	TRUE	aIEHILILSGDQLYR	95%	n+304 (+304)	33.04	25.15
5423	seq=translation; coord=6:166587013..166592611:1; parent_transcript=GRMZM2G027955_T01; parent_gene=GRMZM2G027955	GRMZM2G027955_P01,GRMZM2G027955_P02, GRMZM2G027955_P03	TRUE	TRUE	dVLLDLLk	95%	n+304 (+304), K+304 (+304)	30.47	25.00
5424	seq=translation; coord=6:166587013..166592611:1; parent_transcript=GRMZM2G027955_T01; parent_gene=GRMZM2G027955	GRMZM2G027955_P01,GRMZM2G027955_P02, GRMZM2G027955_P03	TRUE	TRUE	fIWVLEDYYk	95%	n+304 (+304), K+304 (+304)	31.70	25.85
5425	seq=translation; coord=2:36317292..36319768:1; parent_transcript=GRMZM2G102183_T01; parent_gene=GRMZM2G102183	GRMZM2G102183_P01,GRMZM2G102183_P02, GRMZM2G102183_P03	TRUE	TRUE	aTVLVETLPAVFQMNEILHELR	95%	n+304 (+304)	79.79	25.26
5426	seq=translation; coord=2:36317292..36319768:1; parent_transcript=GRMZM2G102183_T01; parent_gene=GRMZM2G102183	GRMZM2G102183_P01,GRMZM2G102183_P02, GRMZM2G102183_P03	TRUE	TRUE	dAVAGTISFR	91%	n+304 (+304)	27.78	25.00
5427	seq=translation; coord=4:173177749..173179720:-1; parent_transcript=GRMZM2G054012_T01; parent_gene=GRMZM2G054012	GRMZM2G054012_P01,GRMZM2G096690_P01, GRMZM2G328168_P01,GRMZM5G805526_P01	TRUE	TRUE	fVTAVVGFGk	95%	n+304 (+304), K+304 (+304)	33.34	25.00
5428	seq=translation; coord=4:173177749..173179720:-1; parent_transcript=GRMZM2G054012_T01; parent_gene=GRMZM2G054012	GRMZM2G054012_P01,GRMZM2G096690_P01, GRMZM2G328168_P01,GRMZM5G805526_P01	TRUE	TRUE	rFVTAVVGFGk	95%	n+304 (+304), K+304 (+304)	32.60	25.00
5429	seq=translation; coord=10:63619774..63622627:-1; parent_transcript=GRMZM2G368861_T01; parent_gene=GRMZM2G368861	GRMZM2G368861_P01	TRUE	TRUE	eDGGWTAGYcHLR	95%	n+304 (+304), Carbamidomethyl (+57)	36.61	25.00
5430	seq=translation; coord=10:63619774..63622627:-1; parent_transcript=GRMZM2G368861_T01; parent_gene=GRMZM2G368861	GRMZM2G368861_P01	TRUE	TRUE	lcSNEcVk	95%	n+304 (+304), Carbamidomethyl (+57), Carbamidomethyl (+57), K+304 (+304)	35.65	25.00
5431	seq=translation; coord=1:191987932..191990561:1; parent_transcript=AC197246.3_FGT003; parent_gene=AC197246.3_FG003	AC197246.3_FGP003,GRMZM2G155931_P01,G RMZM2G155931_P03,GRMZM2G155931_P04,G RMZM2G155931_P05	TRUE	TRUE	aVPLLTk	91%	n+304 (+304), K+304 (+304)	27.04	25.00
5432	seq=translation; coord=1:191987932..191990561:1; parent_transcript=AC197246.3_FGT003; parent_gene=AC197246.3_FG003	AC197246.3_FGP003,GRMZM2G155931_P01,G RMZM2G155931_P03,GRMZM2G155931_P04,G RMZM2G155931_P05	TRUE	TRUE	fVVHNVSELELLMMHNR	95%	n+304 (+304)	59.10	25.00
5433	seq=translation; coord=10:127861555..127867760:-1; parent_transcript=GRMZM2G096705_T01; parent_gene=GRMZM2G096705	GRMZM2G096705_P01	TRUE	TRUE	gLILAFk	95%	n+304 (+304), K+304 (+304)	37.44	25.00
5434	seq=translation; coord=10:127861555..127867760:-1; parent_transcript=GRMZM2G096705_T01; parent_gene=GRMZM2G096705	GRMZM2G096705_P01	TRUE	TRUE	gNVSEEQADAVEELk	95%	n+304 (+304), K+304 (+304)	54.29	25.00
5435	seq=translation; coord=7:82007091..82010522:1; parent_transcript=GRMZM2G056501_T01; parent_gene=GRMZM2G056501	GRMZM2G056501_P01,GRMZM2G056501_P02, GRMZM2G056501_P06	TRUE	TRUE	dYSMEYILTHLk	95%	n+304 (+304), K+304 (+304)	36.12	26.72
5436	seq=translation; coord=7:82007091..82010522:1; parent_transcript=GRMZM2G056501_T01; parent_gene=GRMZM2G056501	GRMZM2G056501_P01,GRMZM2G056501_P02, GRMZM2G056501_P06	TRUE	TRUE	fSVLGNWQR	89%	n+304 (+304)	26.85	25.00
5437	seq=translation; coord=2:189443283..189447454:1; parent_transcript=GRMZM2G112165_T01; parent_gene=GRMZM2G112165	GRMZM2G112165_P01	TRUE	TRUE	aTNTLSIIDSGIGMTk	95%	n+304 (+304), K+304 (+304)	40.45	25.85

5438	seq=translation; coord=2:189443283..189447454:1; parent_transcript=GRMZM2G112165_T01; parent_gene=GRMZM2G112165	GRMZM2G112165_P01	TRUE	TRUE	rAPFDLFDTk	91%	n+304 (+304), K+304 (+304)	26.05	25.95
5439	seq=translation; coord=8:127487910..127490998:1; parent_transcript=GRMZM2G150485_T01; parent_gene=GRMZM2G150485	GRMZM2G150485_P01,GRMZM2G150485_P03, GRMZM2G150485_P06	TRUE	TRUE	sNIVGLPVSLLLLk	95%	n+304 (+304), K+304 (+304)	32.32	25.00
5440	seq=translation; coord=8:127487910..127490998:1; parent_transcript=GRMZM2G150485_T01; parent_gene=GRMZM2G150485	GRMZM2G150485_P01,GRMZM2G150485_P03, GRMZM2G150485_P06	TRUE	TRUE	vAGYLTPVPGGVGPMTVAMLLk	94%	n+304 (+304), K+304 (+304)	28.21	25.00
5441	seq=translation; coord=7:166680013..166686364:1; parent_transcript=GRMZM2G025281_T01; parent_gene=GRMZM2G025281	GRMZM2G025281_P01,GRMZM2G025281_P02, GRMZM2G025281_P03,GRMZM2G077823_P01, GRMZM2G077823_P02,GRMZM2G077823_P03, GRMZM2G077823_P04,GRMZM2G077823_P05	TRUE	TRUE	eIMVQFAk	93%	n+304 (+304), K+304 (+304)	27.86	26.33
5442	seq=translation; coord=7:166680013..166686364:1; parent_transcript=GRMZM2G025281_T01; parent_gene=GRMZM2G025281	GRMZM2G025281_P01,GRMZM2G025281_P02, GRMZM2G025281_P03,GRMZM2G077823_P01, GRMZM2G077823_P02,GRMZM2G077823_P03, GRMZM2G077823_P04,GRMZM2G077823_P05	TRUE	TRUE	tTADDLFPLFDk	95%	n+304 (+304), K+304 (+304)	31.12	25.00
5443	seq=translation; coord=2:217093149..217097528:-1; parent_transcript=GRMZM2G030646_T01; parent_gene=GRMZM2G030646	GRMZM2G030646_P01	TRUE	TRUE	aGFVAQVGR	91%	n+304 (+304)	27.00	25.00
5444	seq=translation; coord=2:217093149..217097528:-1; parent_transcript=GRMZM2G030646_T01; parent_gene=GRMZM2G030646	GRMZM2G030646_P01	TRUE	TRUE	eNNPDLAGDR	95%	n+304 (+304)	35.91	25.00
5445	seq=translation; coord=2:217093149..217097528:-1; parent_transcript=GRMZM2G030646_T01; parent_gene=GRMZM2G030646	GRMZM2G030646_P01	TRUE	TRUE	qPEHVMMFLLAEMGTSGSLDGQQR	95%	n+304 (+304)	37.96	25.00
5446	seq=translation; coord=2:217093149..217097528:-1; parent_transcript=GRMZM2G030646_T01; parent_gene=GRMZM2G030646	GRMZM2G030646_P01	TRUE	TRUE	tESLAVAEPAELNFTGMk	95%	n+304 (+304), K+304 (+304)	40.41	25.79
5447	seq=translation; coord=2:217093149..217097528:-1; parent_transcript=GRMZM2G030646_T01; parent_gene=GRMZM2G030646	GRMZM2G030646_P01	TRUE	TRUE	tVFVNFMDLck	95%	n+304 (+304), Carbamidomethyl (+57), K+304 (+304)	34.66	25.15
5448	seq=translation; coord=1:27094498..27098727:1; parent_transcript=GRMZM2G163129_T01; parent_gene=GRMZM2G163129	GRMZM2G163129_P01	TRUE	TRUE	fHVPDVLLPDLEAR	95%	n+304 (+304)	47.81	25.65
5449	seq=translation; coord=1:27094498..27098727:1; parent_transcript=GRMZM2G163129_T01; parent_gene=GRMZM2G163129	GRMZM2G163129_P01	TRUE	TRUE	fVFADNk	91%	n+304 (+304), K+304 (+304)	26.02	25.66
5450	seq=translation; coord=1:27094498..27098727:1; parent_transcript=GRMZM2G163129_T01; parent_gene=GRMZM2G163129	GRMZM2G163129_P01	TRUE	TRUE	yGAVWTGDNSADWDHLk	95%	n+304 (+304), K+304 (+304)	27.76	25.00
5451	seq=translation; coord=5:210594434..210598555:-1; parent_transcript=GRMZM2G090718_T01; parent_gene=GRMZM2G090718	GRMZM2G090718_P01	TRUE	TRUE	aGDGVFHFVVEIPk	91%	n+304 (+304), K+304 (+304)	26.96	25.67
5452	seq=translation; coord=6:109292609..109296377:1; parent_transcript=GRMZM2G048194_T01; parent_gene=GRMZM2G048194	GRMZM2G048194_P01,GRMZM2G048194_P02	TRUE	TRUE	tTIEPcSGADAcPALLGYk	95%	n+304 (+304), Carbamidomethyl (+57), Carbamidomethyl (+57), K+304 (+304)	54.84	25.00
5453	seq=translation; coord=6:109292609..109296377:1; parent_transcript=GRMZM2G048194_T01; parent_gene=GRMZM2G048194	GRMZM2G048194_P01,GRMZM2G048194_P02	TRUE	TRUE	vSEVAALFGADPAAVLAANALDFASPGAANR	95%	n+304 (+304)	31.37	25.35

5454	seq=translation; coord=8:78073258..78079706:1; parent_transcript=GRMZM2G023418_T01; parent_gene=GRMZM2G023418	GRMZM2G023418_P01	TRUE	TRUE	aEDAAPETk	95%	n+304 (+304), K+304 (+304)	30.94	25.00
5455	seq=translation; coord=8:78073258..78079706:1; parent_transcript=GRMZM2G023418_T01; parent_gene=GRMZM2G023418	GRMZM2G023418_P01	TRUE	TRUE	dEGTAATAALVDDAEGk	95%	n+304 (+304), K+304 (+304)	32.57	25.00
5456	seq=translation; coord=8:78073258..78079706:1; parent_transcript=GRMZM2G023418_T01; parent_gene=GRMZM2G023418	GRMZM2G023418_P01	TRUE	TRUE	iSAFDEEEAkPIVk	95%	n+304 (+304), K+304 (+304), K+304 (+304)	26.45	25.08
5457	seq=translation; coord=8:78073258..78079706:1; parent_transcript=GRMZM2G023418_T01; parent_gene=GRMZM2G023418	GRMZM2G023418_P01	TRUE	TRUE	kDPIVLGVDVLEGIAk	95%	K+304 (+304), n+304 (+304), K+304 (+304)	39.39	25.00
5458	seq=translation; coord=8:78073258..78079706:1; parent_transcript=GRMZM2G023418_T01; parent_gene=GRMZM2G023418	GRMZM2G023418_P01	TRUE	TRUE	lTDIVTQFk	95%	n+304 (+304), K+304 (+304)	30.00	25.00
5459	seq=translation; coord=9:150816519..150821326:-1; parent_transcript=GRMZM2G169365_T01; parent_gene=GRMZM2G169365	GRMZM2G169365_P01	TRUE	TRUE	aADNQLFVATcAPAR	95%	n+304 (+304), Carbamidomethyl (+57)	69.19	25.00
5460	seq=translation; coord=9:150816519..150821326:-1; parent_transcript=GRMZM2G169365_T01; parent_gene=GRMZM2G169365	GRMZM2G169365_P01	TRUE	TRUE	iHLFDIDIPGk	95%	n+304 (+304), K+304 (+304)	28.45	25.02
5461	seq=translation; coord=9:150816519..150821326:-1; parent_transcript=GRMZM2G169365_T01; parent_gene=GRMZM2G169365	GRMZM2G169365_P01	TRUE	TRUE	rGDLYQLVDVQR	95%	n+304 (+304)	44.01	25.73
5462	seq=translation; coord=9:150816519..150821326:-1; parent_transcript=GRMZM2G169365_T01; parent_gene=GRMZM2G169365	GRMZM2G169365_P01	TRUE	TRUE	tLTAGQSPTVVDTDVGR	95%	n+304 (+304)	35.85	25.48
5463	seq=translation; coord=2:193416339..193419729:-1; parent_transcript=GRMZM2G023080_T01; parent_gene=GRMZM2G023080	GRMZM2G023080_P01,GRMZM2G023080_P02, GRMZM2G040164_P01,GRMZM2G040164_P02	TRUE	TRUE	fDPLWNSLVLGGVk	95%	n+304 (+304), K+304 (+304)	30.22	25.29
5464	seq=translation; coord=2:193416339..193419729:-1; parent_transcript=GRMZM2G023080_T01; parent_gene=GRMZM2G023080	GRMZM2G023080_P01,GRMZM2G023080_P02, GRMZM2G040164_P01,GRMZM2G040164_P02	TRUE	TRUE	hSIIGASGEFSDFQEILR	95%	n+304 (+304)	80.56	25.00
5465	seq=translation; coord=5:30771033..30772796:1; parent_transcript=GRMZM2G026216_T01; parent_gene=GRMZM2G026216	GRMZM2G026216_P01,GRMZM2G026216_P02, GRMZM2G089421_P01,GRMZM2G149257_P01	TRUE	TRUE	aIGVEINk	95%	n+304 (+304), K+304 (+304)	36.43	27.12
5466	seq=translation; coord=5:30771033..30772796:1; parent_transcript=GRMZM2G026216_T01; parent_gene=GRMZM2G026216	GRMZM2G026216_P01,GRMZM2G026216_P02, GRMZM2G089421_P01,GRMZM2G149257_P01	TRUE	TRUE	iGDYVDVk	89%	n+304 (+304), K+304 (+304)	26.82	25.88
5467	seq=translation; coord=5:215656610..215661825:1; parent_transcript=GRMZM2G143651_T01; parent_gene=GRMZM2G143651	GRMZM2G143651_P01	TRUE	TRUE	iQPVYVIDVAAAIVNSLk	95%	n+304 (+304), K+304 (+304)	66.10	25.00
5468	seq=translation; coord=5:215656610..215661825:1; parent_transcript=GRMZM2G143651_T01; parent_gene=GRMZM2G143651	GRMZM2G143651_P01	TRUE	TRUE	sSVSGIVATVFGATGFLGR	95%	n+304 (+304)	41.26	25.91
5469	seq=translation; coord=6:28127232..28129613:1; parent_transcript=GRMZM2G165815_T02; parent_gene=GRMZM2G165815	GRMZM2G165815_P02	TRUE	TRUE	lDLDIER	95%	n+304 (+304)	33.35	25.29
5470	seq=translation; coord=6:28127232..28129613:1; parent_transcript=GRMZM2G165815_T02; parent_gene=GRMZM2G165815	GRMZM2G165815_P02	TRUE	TRUE	sMEEMANEANEYR	95%	n+304 (+304)	66.35	25.00
5471	seq=translation; coord=8:154456893..154470550:1; parent_transcript=GRMZM2G316113_T01; parent_gene=GRMZM2G316113	GRMZM2G316113_P01,GRMZM2G316113_P02, GRMZM2G316113_P05	TRUE	TRUE	aSVFGSSAEDGFLNVWDHEk	95%	n+304 (+304), K+304 (+304)	36.29	25.00

5472	seq=translation; coord=8:154456893..154470550:1; parent_transcript=GRMZM2G316113_T01; parent_gene=GRMZM2G316113	GRMZM2G316113_P01,GRMZM2G316113_P02, GRMZM2G316113_P05	TRUE	TRUE	iIATHTDSPDVLIWDVEAQPNR	95%	n+304 (+304)	33.32	25.45
5473	seq=translation; coord=6:24549898..24554957:-1; parent_transcript=GRMZM2G111579_T01; parent_gene=GRMZM2G111579	GRMZM2G111579_P01	TRUE	TRUE	aGLALQPIATAIFANSPFk	95%	n+304 (+304), K+304 (+304)	58.23	25.00
5474	seq=translation; coord=6:24549898..24554957:-1; parent_transcript=GRMZM2G111579_T01; parent_gene=GRMZM2G111579	GRMZM2G111579_P01	TRUE	TRUE	wALSDIPIMPk	91%	n+304 (+304), K+304 (+304)	25.31	25.00
5475	seq=translation; coord=5:163610246..163620614:1; parent_transcript=GRMZM2G360589_T01; parent_gene=GRMZM2G360589	GRMZM2G360589_P01	TRUE	TRUE	dMSAEVVDNNPYSR	95%	n+304 (+304)	49.26	25.00
5476	seq=translation; coord=5:163610246..163620614:1; parent_transcript=GRMZM2G360589_T01; parent_gene=GRMZM2G360589	GRMZM2G360589_P01	TRUE	TRUE	gFETFLASLk	94%	n+304 (+304), K+304 (+304)	28.99	25.42
5477	seq=translation; coord=2:76993674..76995064:1; parent_transcript=GRMZM2G069523_T01; parent_gene=GRMZM2G069523	GRMZM2G069523_P01	TRUE	TRUE	aMVTLTENAAAELGQHGIR	95%	n+304 (+304)	86.18	25.00
5478	seq=translation; coord=2:76993674..76995064:1; parent_transcript=GRMZM2G069523_T01; parent_gene=GRMZM2G069523	GRMZM2G069523_P01	TRUE	TRUE	lFVEHGAHVVVADIQDEAGAR	95%	n+304 (+304)	61.61	25.22
5479	seq=translation; coord=2:76993674..76995064:1; parent_transcript=GRMZM2G069523_T01; parent_gene=GRMZM2G069523	GRMZM2G069523_P01	TRUE	TRUE	vHDVAAAVLFLASDDAR	91%	n+304 (+304)	26.72	25.69
5480	seq=translation; coord=2:76993674..76995064:1; parent_transcript=GRMZM2G069523_T01; parent_gene=GRMZM2G069523	GRMZM2G069523_P01	TRUE	TRUE	vLAVNLVGPFLGTk	95%	n+304 (+304), K+304 (+304)	48.73	25.00
5481	seq=translation; coord=4:240105137..240108983:1; parent_transcript=GRMZM2G079348_T01; parent_gene=GRMZM2G079348	GRMZM2G079348_P01,GRMZM2G079348_P02, GRMZM2G079348_P03,GRMZM2G079348_P04	TRUE	TRUE	cILTDEEAALVGGR	95%	Carbamidomethyl (+57), n+304 (+304)	69.28	25.00
5482	seq=translation; coord=4:240105137..240108983:1; parent_transcript=GRMZM2G079348_T01; parent_gene=GRMZM2G079348	GRMZM2G079348_P01,GRMZM2G079348_P02, GRMZM2G079348_P03,GRMZM2G079348_P04	TRUE	TRUE	gFFEcTHDVTSLTcADFLR	95%	n+304 (+304), Carbamidomethyl (+57), Carbamidomethyl (+57)	39.06	25.00
5483	seq=translation; coord=4:240105137..240108983:1; parent_transcript=GRMZM2G079348_T01; parent_gene=GRMZM2G079348	GRMZM2G079348_P01,GRMZM2G079348_P02, GRMZM2G079348_P03,GRMZM2G079348_P04	TRUE	TRUE	sIWIDLLAk	95%	n+304 (+304), K+304 (+304)	40.79	25.00
5484	seq=translation; coord=5:3195909..3207226:1; parent_transcript=GRMZM2G071071_T01; parent_gene=GRMZM2G071071	GRMZM2G071071_P01	TRUE	TRUE	aPDQTLELTSEVVDFLR	95%	n+304 (+304)	43.50	25.60
5485	seq=translation; coord=5:3195909..3207226:1; parent_transcript=GRMZM2G071071_T01; parent_gene=GRMZM2G071071	GRMZM2G071071_P01	TRUE	TRUE	lIAEcQAADAVVLTYAcDRPATLER	95%	n+304 (+304), Carbamidomethyl (+57), Carbamidomethyl (+57)	29.25	25.00
5486	seq=translation; coord=5:3195909..3207226:1; parent_transcript=GRMZM2G071071_T01; parent_gene=GRMZM2G071071	GRMZM2G071071_P01	TRUE	TRUE	lSSFWLPELR	95%	n+304 (+304)	50.57	25.00
5487	seq=translation; coord=5:3195909..3207226:1; parent_transcript=GRMZM2G071071_T01; parent_gene=GRMZM2G071071	GRMZM2G071071_P01	TRUE	TRUE	sSLIVALATEQFPENVPR	95%	n+304 (+304)	43.71	25.17
5488	seq=translation; coord=8:94750778..94753384:1; parent_transcript=GRMZM2G147221_T01; parent_gene=GRMZM2G147221	GRMZM2G147221_P01	TRUE	TRUE	lLAVELLNEPLAPGATLDSLTR	95%	n+304 (+304)	37.48	25.00
5489	seq=translation; coord=8:94750778..94753384:1; parent_transcript=GRMZM2G147221_T01; parent_gene=GRMZM2G147221	GRMZM2G147221_P01	TRUE	TRUE	tQASDWETFk	95%	n+304 (+304), K+304 (+304)	37.14	25.00

	seq=translation; coord=6:6269340..6271805:-1;								
5490	parent_transcript=GRMZM2G134134_T02; parent_gene=GRMZM2G134134	GRMZM2G134134_P02	TRUE	TRUE	dLVVDMTNFYNQYk	95%	n+304 (+304), K+304 (+304)	37.58	25.04
5491	seq=translation; coord=9:19034931..19038665:-1; parent_transcript=GRMZM5G877388_T02; parent_gene=GRMZM5G877388	GRMZM5G877388_P02	TRUE	TRUE	kTGMSLIIGIYDEPMTPGQcNMVVER	95%	K+304 (+304), n+304 (+304), Carbamidomethyl (+57)	41.44	25.00
5492	seq=translation; coord=9:19034931..19038665:-1; parent_transcript=GRMZM5G877388_T02; parent_gene=GRMZM5G877388	GRMZM5G877388_P02	TRUE	TRUE	tGMSLIIGIyDEPMTPGQcNMVVER	95%	n+304 (+304), iTRAQ8plex (+304), Carbamidomethyl (+57)	27.52	25.00
5493	seq=translation; coord=1:34664608..34673432:1; parent_transcript=GRMZM2G058870_T01; parent_gene=GRMZM2G058870	GRMZM2G058870_P01	TRUE	TRUE	iIGDFDGALTPLAR	92%	n+304 (+304)	29.70	26.22
5494	seq=translation; coord=1:34664608..34673432:1; parent_transcript=GRMZM2G058870_T01; parent_gene=GRMZM2G058870	GRMZM2G058870_P01	TRUE	TRUE	lVEIFSPFLEk	95%	n+304 (+304), K+304 (+304)	31.21	25.11
5495	seq=translation; coord=5:6688332..6691221:1; parent_transcript=GRMZM5G862540_T01; parent_gene=GRMZM5G862540	GRMZM5G862540_P01,GRMZM5G862540_P02	TRUE	TRUE	gAHGVcILTEWDEFk	95%	n+304 (+304), Carbamidomethyl (+57), K+304 (+304)	33.91	25.00
5496	seq=translation; coord=5:6688332..6691221:1; parent_transcript=GRMZM5G862540_T01; parent_gene=GRMZM5G862540	GRMZM5G862540_P01,GRMZM5G862540_P02	TRUE	TRUE	vVASMFNTVAGk	91%	n+304 (+304), K+304 (+304)	26.63	25.77
5497	seq=translation; coord=4:84106470..84111628:-1; parent_transcript=GRMZM2G035268_T01; parent_gene=GRMZM2G035268	GRMZM2G035268_P01,GRMZM2G035268_P03	TRUE	TRUE	vVLIMESIADAVVQk	95%	n+304 (+304), K+304 (+304)	47.34	25.00
5498	seq=translation; coord=9:57980157..57982564:1; parent_transcript=GRMZM2G066111_T01; parent_gene=GRMZM2G066111	GRMZM2G066111_P01,GRMZM2G109418_P01, GRMZM2G470035_P01,GRMZM5G884912_P01	TRUE	TRUE	eAFLLEPR	91%	n+304 (+304)	28.66	25.77
5499	seq=translation; coord=9:57980157..57982564:1; parent_transcript=GRMZM2G066111_T01; parent_gene=GRMZM2G066111	GRMZM2G066111_P01,GRMZM2G109418_P01, GRMZM2G470035_P01,GRMZM5G884912_P01	TRUE	TRUE	eGFIVLR	88%	n+304 (+304)	25.98	25.00
5500	seq=translation; coord=3:87798819..87808953:-1; parent_transcript=GRMZM2G009593_T01; parent_gene=GRMZM2G009593	GRMZM2G009593_P01,GRMZM2G028700_P01, GRMZM2G070323_P01	TRUE	TRUE	gNHEAADINALFGFR	95%	n+304 (+304)	46.52	25.00
5501	seq=translation; coord=3:87798819..87808953:-1; parent_transcript=GRMZM2G009593_T01; parent_gene=GRMZM2G009593	GRMZM2G009593_P01,GRMZM2G028700_P01, GRMZM2G070323_P01	TRUE	TRUE	iFGDLHGQFGDLMR	95%	n+304 (+304)	27.79	25.00
5502	seq=translation; coord=3:87798819..87808953:-1; parent_transcript=GRMZM2G009593_T01; parent_gene=GRMZM2G009593	GRMZM2G009593_P01,GRMZM2G028700_P01, GRMZM2G070323_P01	TRUE	TRUE	lFNWLPLAALIEk	92%	n+304 (+304), K+304 (+304)	26.97	25.00
5503	seq=translation; coord=6:126146579..126150071:1; parent_transcript=GRMZM2G341729_T01; parent_gene=GRMZM2G341729	GRMZM2G341729_P01	TRUE	TRUE	wVGLATDFSEGSR	95%	n+304 (+304)	40.35	25.00
5504	seq=translation; coord=2:196359627..196363692:-1; parent_transcript=GRMZM2G573867_T01; parent_gene=GRMZM2G573867	GRMZM2G573867_P01,GRMZM2G573867_P02	TRUE	TRUE	gVNFIQIPTTLMAQVDSSVGGk	95%	n+304 (+304), K+304 (+304)	41.00	25.00
5505	seq=translation; coord=2:196359627..196363692:-1; parent_transcript=GRMZM2G573867_T01; parent_gene=GRMZM2G573867	GRMZM2G573867_P01,GRMZM2G573867_P02	TRUE	TRUE	vSTVVDVDLGDR	95%	n+304 (+304)	39.77	25.87
5506	seq=translation; coord=1:49207907..49216831:1; parent_transcript=GRMZM2G140432_T01; parent_gene=GRMZM2G140432	GRMZM2G140432_P01	TRUE	TRUE	eLcDLPLAEDIPITMSSVR	95%	n+304 (+304), Carbamidomethyl (+57)	38.93	25.00
5507	seq=translation; coord=1:49207907..49216831:1; parent_transcript=GRMZM2G140432_T01; parent_gene=GRMZM2G140432	GRMZM2G140432_P01	TRUE	TRUE	gSPNEFPGIGATSPLLWLAR	95%	n+304 (+304)	36.94	25.16

5508	seq=translation; coord=5:214501921..214505061:1; parent_transcript=GRMZM2G008728_T02; parent_gene=GRMZM2G008728	GRMZM2G008728_P02	TRUE	TRUE	eVEDHPALGEGEVLLEVAAAGVNR	95%	n+304 (+304)	50.30	25.00
5509	seq=translation; coord=5:214501921..214505061:1; parent_transcript=GRMZM2G008728_T02; parent_gene=GRMZM2G008728	GRMZM2G008728_P02	TRUE	TRUE	gVDVILDNIGGSYLQR	95%	n+304 (+304)	34.56	26.18
5510	seq=translation; coord=9:147281001..147286520:-1; parent_transcript=GRMZM2G100511_T01; parent_gene=GRMZM2G100511	GRMZM2G100511_P01	TRUE	TRUE	aMEVLADILQNSNLDQAR	95%	n+304 (+304)	32.47	25.00
5511	seq=translation; coord=1:298585659..298587727:-1; parent_transcript=GRMZM2G058568_T03; parent_gene=GRMZM2G058568	GRMZM2G058568_P03,GRMZM2G058568_P04	TRUE	TRUE	eLYLGDR	90%	n+304 (+304)	27.24	25.00
5512	seq=translation; coord=1:298585659..298587727:-1; parent_transcript=GRMZM2G058568_T03; parent_gene=GRMZM2G058568	GRMZM2G058568_P03,GRMZM2G058568_P04	TRUE	TRUE	lGAGDPFPGSPPGAAR	86%	n+304 (+304)	25.68	25.21
5513	seq=translation; coord=1:298585659..298587727:-1; parent_transcript=GRMZM2G058568_T03; parent_gene=GRMZM2G058568	GRMZM2G058568_P03,GRMZM2G058568_P04	TRUE	TRUE	vTDADLYAPAk	95%	n+304 (+304), K+304 (+304)	38.56	26.73
5514	seq=translation; coord=9:116241684..116244580:-1; parent_transcript=GRMZM2G013002_T01; parent_gene=GRMZM2G013002	GRMZM2G013002_P01	TRUE	TRUE	eSWGSVWR	90%	n+304 (+304)	27.21	25.00
5515	seq=translation; coord=9:116241684..116244580:-1; parent_transcript=GRMZM2G013002_T01; parent_gene=GRMZM2G013002	GRMZM2G013002_P01	TRUE	TRUE	vPcEFPGLk	90%	n+304 (+304), Carbamidomethyl (+57), K+304 (+304)	28.51	26.21
5516	seq=translation; coord=9:116241684..116244580:-1; parent_transcript=GRMZM2G013002_T01; parent_gene=GRMZM2G013002	GRMZM2G013002_P01	TRUE	TRUE	yHFDLSGTAFGR	95%	n+304 (+304), iTRAQ8plex (+304)	34.71	25.00
5517	seq=translation; coord=9:107790531..107797125:1; parent_transcript=GRMZM2G055320_T01; parent_gene=GRMZM2G055320	GRMZM2G055320_P01	TRUE	TRUE	gDVVMALLR	95%	n+304 (+304)	41.44	25.15
5518	seq=translation; coord=9:107790531..107797125:1; parent_transcript=GRMZM2G055320_T01; parent_gene=GRMZM2G055320	GRMZM2G055320_P01	TRUE	TRUE	gFQVPPAELEALLITHPEIk	95%	n+304 (+304), K+304 (+304)	64.58	25.00
5519	seq=translation; coord=9:107790531..107797125:1; parent_transcript=GRMZM2G055320_T01; parent_gene=GRMZM2G055320	GRMZM2G055320_P01	TRUE	TRUE	ncPEFALVFLGAAR	95%	n+304 (+304), Carbamidomethyl (+57)	62.11	25.98
5520	seq=translation; coord=8:143676986..143682414:1; parent_transcript=GRMZM2G107757_T01; parent_gene=GRMZM2G107757	GRMZM2G107757_P01,GRMZM2G107757_P02, GRMZM2G107757_P03	TRUE	TRUE	aTTETVDALR	90%	n+304 (+304)	27.80	25.50
5521	seq=translation; coord=8:143676986..143682414:1; parent_transcript=GRMZM2G107757_T01; parent_gene=GRMZM2G107757	GRMZM2G107757_P01,GRMZM2G107757_P02, GRMZM2G107757_P03	TRUE	TRUE	lNETLDMLEk	95%	n+304 (+304), K+304 (+304)	36.64	26.46
5522	seq=translation; coord=8:143676986..143682414:1; parent_transcript=GRMZM2G107757_T01; parent_gene=GRMZM2G107757	GRMZM2G107757_P01,GRMZM2G107757_P02, GRMZM2G107757_P03	TRUE	TRUE	lYEQQIEQLGNFQLR	95%	n+304 (+304)	54.77	25.68
5523	seq=translation; coord=3:147676601..147679180:-1; parent_transcript=GRMZM2G479112_T01; parent_gene=GRMZM2G479112	GRMZM2G479112_P01	TRUE	TRUE	eLVSQFAVcDR	93%	n+304 (+304), Carbamidomethyl (+57)	30.14	25.00
5524	seq=translation; coord=3:147676601..147679180:-1; parent_transcript=GRMZM2G479112_T01; parent_gene=GRMZM2G479112	GRMZM2G479112_P01	TRUE	TRUE	gFNPADVAVYR	93%	n+304 (+304)	30.05	25.00
5525	seq=translation; coord=3:147676601..147679180:-1; parent_transcript=GRMZM2G479112_T01; parent_gene=GRMZM2G479112	GRMZM2G479112_P01	TRUE	TRUE	vPAILVSPWIDR	95%	n+304 (+304)	42.60	25.90

5526	seq=translation; coord=8:8472359..8474789:1; parent_transcript=GRMZM2G021614_T01; parent_gene=GRMZM2G021614	GRMZM2G021614_P01,GRMZM2G021614_P02	TRUE	TRUE	eYLHWIVINIPGGTDATk	95%	n+304 (+304), K+304 (+304)	31.14	25.00
5527	seq=translation; coord=8:8472359..8474789:1; parent_transcript=GRMZM2G021614_T01; parent_gene=GRMZM2G021614	GRMZM2G021614_P01,GRMZM2G021614_P02	TRUE	TRUE	yVLVLFEQk	95%	n+304 (+304), K+304 (+304)	42.31	25.00
5528	seq=translation; coord=6:123432811..123435820:-1; parent_transcript=GRMZM2G127160_T01; parent_gene=GRMZM2G127160	GRMZM2G127160_P01,GRMZM2G127160_P02	TRUE	TRUE	eSGGMAAALR	95%	n+304 (+304)	34.18	25.00
5529	seq=translation; coord=6:123432811..123435820:-1; parent_transcript=GRMZM2G127160_T01; parent_gene=GRMZM2G127160	GRMZM2G127160_P01,GRMZM2G127160_P02	TRUE	TRUE	vSMVDTDQAFNVFINR	95%	n+304 (+304)	62.55	25.00
5530	seq=translation; coord=6:86773351..86783757:1; parent_transcript=GRMZM2G161493_T01; parent_gene=GRMZM2G161493	GRMZM2G161493_P01	TRUE	TRUE	vGFTVMDTWLAYAPVk	95%	n+304 (+304), K+304 (+304)	42.02	25.02
5531	seq=translation; coord=9:7614057..7616811:1; parent_transcript=GRMZM2G079949_T01; parent_gene=GRMZM2G079949	GRMZM2G079949_P01	TRUE	TRUE	dVVVDPATGLWAR	89%	n+304 (+304)	27.46	25.54
5532	seq=translation; coord=9:7614057..7616811:1; parent_transcript=GRMZM2G079949_T01; parent_gene=GRMZM2G079949	GRMZM2G079949_P01	TRUE	TRUE	vFLAGASAGATIAHFVAVR	95%	n+304 (+304)	100.92	25.67
5533	seq=translation; coord=5:188857165..188858944:1; parent_transcript=GRMZM2G170017_T01; parent_gene=GRMZM2G170017	GRMZM2G170017_P01	TRUE	TRUE	lDELSALFLEDYR	92%	n+304 (+304)	26.73	25.00
5534	seq=translation; coord=5:188857165..188858944:1; parent_transcript=GRMZM2G170017_T01; parent_gene=GRMZM2G170017	GRMZM2G170017_P01	TRUE	TRUE	tTQSAEQAEEcVR	95%	n+304 (+304), Carbamidomethyl (+57)	59.58	25.00
5535	seq=translation; coord=6:160034201..160039816:-1; parent_transcript=GRMZM2G004932_T01; parent_gene=GRMZM2G004932	GRMZM2G004932_P01	TRUE	TRUE	lLLDIGLWLTHk	95%	n+304 (+304), K+304 (+304)	33.94	25.00
5536	seq=translation; coord=6:160034201..160039816:-1; parent_transcript=GRMZM2G004932_T01; parent_gene=GRMZM2G004932	GRMZM2G004932_P01	TRUE	TRUE	sFGFDTAVEEAQR	95%	n+304 (+304)	62.44	25.00
5537	seq=translation; coord=1:38548724..38550734:-1; parent_transcript=GRMZM2G012566_T02; parent_gene=GRMZM2G012566	GRMZM2G012566_P02	TRUE	TRUE	eYYDDAAPk	95%	n+304 (+304), K+304 (+304)	34.64	25.00
5538	seq=translation; coord=1:38548724..38550734:-1; parent_transcript=GRMZM2G012566_T02; parent_gene=GRMZM2G012566	GRMZM2G012566_P02	TRUE	TRUE	lSEDPNAEER	95%	n+304 (+304)	33.82	25.00
5539	seq=translation; coord=4:53673036..53676735:-1; parent_transcript=GRMZM2G032367_T01; parent_gene=GRMZM2G032367	GRMZM2G032367_P01,GRMZM2G032367_P02, GRMZM2G032367_P03,GRMZM2G158479_P02, GRMZM2G158479_P03,GRMZM2G158479_P04	TRUE	TRUE	dLVGVAYTEEETk	95%	n+304 (+304), K+304 (+304)	42.26	26.09
5540	seq=translation; coord=4:53673036..53676735:-1; parent_transcript=GRMZM2G032367_T01; parent_gene=GRMZM2G032367	GRMZM2G032367_P01,GRMZM2G032367_P02, GRMZM2G032367_P03,GRMZM2G158479_P02, GRMZM2G158479_P03,GRMZM2G158479_P04	TRUE	TRUE	dVVSFLSWAAEPEMEER	89%	n+304 (+304)	27.01	25.00
5541	seq=translation; coord=1:258447609..258448792:1; parent_transcript=GRMZM2G039639_T01; parent_gene=GRMZM2G039639	GRMZM2G039639_P01	TRUE	TRUE	cDADVTSQcPAALQAPGGcDNPcTVFk	95%	Carbamidomethyl (+57), n+304 (+304), Carbamidomethyl (+57), Carbamidomethyl (+57), Carbamidomethyl (+57), K+304 (+304)	43.38	25.00

5542	seq=translation; coord=1:258447609..258448792:1; parent_transcript=GRMZM2G039639_T01; parent_gene=GRMZM2G039639	GRMZM2G039639_P01	TRUE	TRUE	gLcPDAYSYPk	93%	n+304 (+304), Carbamidomethyl (+57), K+304 (+304)	27.43	25.00
5543	seq=translation; coord=1:7406938..7408184:1; parent_transcript=GRMZM2G099454_T02; parent_gene=GRMZM2G099454	GRMZM2G099454_P01,GRMZM2G099454_P02, GRMZM2G099454_P03,GRMZM2G099454_P04	TRUE	TRUE	fPGFGTTGDEQTR	95%	n+304 (+304)	33.26	25.00
5544	seq=translation; coord=1:7406938..7408184:1; parent_transcript=GRMZM2G099454_T02; parent_gene=GRMZM2G099454	GRMZM2G099454_P01,GRMZM2G099454_P02, GRMZM2G099454_P03,GRMZM2G099454_P04	TRUE	TRUE	gDSGcQGAFYTYDAFIEAASk	95%	n+304 (+304), Carbamidomethyl (+57), K+304 (+304)	59.49	25.00
5545	seq=translation; coord=5:49897243..49899430:-1; parent_transcript=GRMZM2G177828_T01; parent_gene=GRMZM2G177828	GRMZM2G177828_P01	TRUE	TRUE	eYDETDLAYLQk	95%	n+304 (+304), K+304 (+304)	67.83	25.00
5546	seq=translation; coord=5:49897243..49899430:-1; parent_transcript=GRMZM2G177828_T01; parent_gene=GRMZM2G177828	GRMZM2G177828_P01	TRUE	TRUE	gAIGGSGLk	95%	n+304 (+304), K+304 (+304)	30.17	25.68
5547	seq=translation; coord=8:2199781..2200562:-1; parent_transcript=GRMZM2G353266_T01; parent_gene=GRMZM2G353266	GRMZM2G353266_P01	TRUE	TRUE	aGAAESEEDVMLR	95%	n+304 (+304)	65.28	25.00
5548	seq=translation; coord=8:2199781..2200562:-1; parent_transcript=GRMZM2G353266_T01; parent_gene=GRMZM2G353266	GRMZM2G353266_P01	TRUE	TRUE	gGGGILGSLQEGk	95%	n+304 (+304), K+304 (+304)	51.02	26.37
5549	seq=translation; coord=7:3483732..3484794:1; parent_transcript=GRMZM2G465226_T01; parent_gene=GRMZM2G465226	GRMZM2G465226_P01	TRUE	TRUE	aDVGVGPVSWDDTVAAYAQSYAAQR	95%	n+304 (+304)	45.88	25.00
5550	seq=translation; coord=7:3483732..3484794:1; parent_transcript=GRMZM2G465226_T01; parent_gene=GRMZM2G465226	GRMZM2G465226_P01	TRUE	TRUE	aDVGVGPVSWDDTVAAyAQSYAAQR	95%	n+304 (+304), iTRAQ8plex (+304)	48.41	25.08
5551	seq=translation; coord=7:3483732..3484794:1; parent_transcript=GRMZM2G465226_T01; parent_gene=GRMZM2G465226	GRMZM2G465226_P01	TRUE	TRUE	dSTAIGcAR	95%	n+304 (+304), Carbamidomethyl (+57)	35.92	25.00
5552	seq=translation; coord=7:29542055..29544356:-1; parent_transcript=GRMZM5G872934_T01; parent_gene=GRMZM5G872934	GRMZM5G872934_P01	TRUE	TRUE	aSGEPALVLASSVHcALR	95%	n+304 (+304), Carbamidomethyl (+57)	61.00	25.50
5553	seq=translation; coord=7:29542055..29544356:-1; parent_transcript=GRMZM5G872934_T01; parent_gene=GRMZM5G872934	GRMZM5G872934_P01	TRUE	TRUE	lWPDADGGGEcLLGR	95%	n+304 (+304), Carbamidomethyl (+57)	51.41	25.00
5554	seq=translation; coord=7:168251112..168255445:1; parent_transcript=GRMZM2G160569_T01; parent_gene=GRMZM2G160569	GRMZM2G160569_P01,GRMZM2G160569_P02, GRMZM2G160569_P03	TRUE	TRUE	iADAVGDWFFER	95%	n+304 (+304)	52.15	25.00
5555	seq=translation; coord=5:192259829..192267449:-1; parent_transcript=GRMZM2G004768_T01; parent_gene=GRMZM2G004768	GRMZM2G004768_P01,GRMZM2G102502_P01	TRUE	TRUE	eALEELTR	89%	n+304 (+304)	27.68	25.67
5556	seq=translation; coord=2:12351628..12357486:-1; parent_transcript=GRMZM2G125728_T01; parent_gene=GRMZM2G125728	GRMZM2G125728_P01,GRMZM2G125728_P02	TRUE	TRUE	dPFDLSSSANVGk	95%	n+304 (+304), K+304 (+304)	31.09	26.00
5557	seq=translation; coord=2:12351628..12357486:-1; parent_transcript=GRMZM2G125728_T01; parent_gene=GRMZM2G125728	GRMZM2G125728_P01,GRMZM2G125728_P02	TRUE	TRUE	gLIDLNITAPk	95%	n+304 (+304), K+304 (+304)	45.26	25.00
5558	seq=translation; coord=2:12351628..12357486:-1; parent_transcript=GRMZM2G125728_T01; parent_gene=GRMZM2G125728	GRMZM2G125728_P01,GRMZM2G125728_P02	TRUE	TRUE	kVNLADIGIVGGLGDGSDEk	95%	K+304 (+304), n+304 (+304), K+304 (+304)	39.46	25.00
5559	seq=translation; coord=2:12351628..12357486:-1; parent_transcript=GRMZM2G125728_T01; parent_gene=GRMZM2G125728	GRMZM2G125728_P01,GRMZM2G125728_P02	TRUE	TRUE	sGIWADSLSR	89%	n+304 (+304)	26.56	25.00

5560	seq=translation; coord=2:12351628..12357486:-1; parent_transcript=GRMZM2G125728_T01; parent_gene=GRMZM2G125728	GRMZM2G125728_P01,GRMZM2G125728_P02	TRUE	TRUE	vNVDLFAEk	95%	n+304 (+304), K+304 (+304)	34.55	26.20
5561	seq=translation; coord=2:236283794..236288865:1; parent_transcript=GRMZM2G103955_T02; parent_gene=GRMZM2G103955	GRMZM2G103955_P02	TRUE	TRUE	aIEDEDFVDAVDcLSR	95%	n+304 (+304), Carbamidomethyl (+57)	66.74	25.00
5562	seq=translation; coord=2:236283794..236288865:1; parent_transcript=GRMZM2G103955_T02; parent_gene=GRMZM2G103955	GRMZM2G103955_P02	TRUE	TRUE	aLSMLEHLVEPDHR	95%	n+304 (+304)	43.01	25.25
5563	seq=translation; coord=2:236283794..236288865:1; parent_transcript=GRMZM2G103955_T02; parent_gene=GRMZM2G103955	GRMZM2G103955_P02	TRUE	TRUE	icLVYELVSk	92%	n+304 (+304), Carbamidomethyl (+57), K+304 (+304)	26.65	25.00
5564	seq=translation; coord=1:194210583..194219751:1; parent_transcript=GRMZM2G171111_T02; parent_gene=GRMZM2G171111	GRMZM2G171111_P02,GRMZM2G171111_P04	TRUE	TRUE	lSTGDSLDMSPQDEk	95%	n+304 (+304), K+304 (+304)	46.80	25.00
5565	seq=translation; coord=1:194210583..194219751:1; parent_transcript=GRMZM2G171111_T02; parent_gene=GRMZM2G171111	GRMZM2G171111_P02,GRMZM2G171111_P04	TRUE	TRUE	rIPIPVFDMYGIGAR	95%	n+304 (+304)	54.09	25.20
5566	seq=translation; coord=2:115064269..115065362:-1; parent_transcript=GRMZM2G008649_T01; parent_gene=GRMZM2G008649	GRMZM2G008649_P01,GRMZM2G506062_P01	TRUE	TRUE	aGEEDATAILGR	95%	n+304 (+304)	36.23	25.00
5567	seq=translation; coord=3:193091877..193119714:1; parent_transcript=GRMZM2G071441_T01; parent_gene=GRMZM2G071441	GRMZM2G071441_P01,GRMZM2G071441_P02	TRUE	TRUE	lFLEVHFR	95%	n+304 (+304)	44.08	25.00
5568	seq=translation; coord=3:193091877..193119714:1; parent_transcript=GRMZM2G071441_T01; parent_gene=GRMZM2G071441	GRMZM2G071441_P01,GRMZM2G071441_P02	TRUE	TRUE	sNSLLDLIEDLk	95%	n+304 (+304), K+304 (+304)	36.65	25.43
5569	seq=translation; coord=5:1526460..1528840:-1; parent_transcript=GRMZM2G448001_T01; parent_gene=GRMZM2G448001	GRMZM2G448001_P01	TRUE	TRUE	aLAVPDGGWPTWADDTTVFFHR	95%	n+304 (+304)	72.05	25.00
5570	seq=translation; coord=5:1526460..1528840:-1; parent_transcript=GRMZM2G448001_T01; parent_gene=GRMZM2G448001	GRMZM2G448001_P01	TRUE	TRUE	lTFTDDDVASVVDR	95%	n+304 (+304)	70.47	25.00
5571	seq=translation; coord=5:14752854..14761623:1; parent_transcript=GRMZM2G553314_T01; parent_gene=GRMZM2G553314	GRMZM2G553314_P01	TRUE	TRUE	iDIGVFDALk	95%	n+304 (+304), K+304 (+304)	36.01	25.00
5572	seq=translation; coord=5:14752854..14761623:1; parent_transcript=GRMZM2G553314_T01; parent_gene=GRMZM2G553314	GRMZM2G553314_P01	TRUE	TRUE	lNIGGEVIk	94%	n+304 (+304), K+304 (+304)	29.46	25.90
5573	seq=translation; coord=1:67523715..67533727:-1; parent_transcript=GRMZM2G410479_T01; parent_gene=GRMZM2G410479	GRMZM2G410479_P01	TRUE	TRUE	aILESIR	93%	n+304 (+304)	29.31	25.00
5574	seq=translation; coord=1:67523715..67533727:-1; parent_transcript=GRMZM2G410479_T01; parent_gene=GRMZM2G410479	GRMZM2G410479_P01	TRUE	TRUE	lNLQFLTLHDYLLR	95%	n+304 (+304)	40.53	25.00
5575	seq=translation; coord=1:67523715..67533727:-1; parent_transcript=GRMZM2G410479_T01; parent_gene=GRMZM2G410479	GRMZM2G410479_P01	TRUE	TRUE	nLIEEFVEILNSk	93%	n+304 (+304), K+304 (+304)	27.27	25.00
5576	seq=translation; coord=7:156205011..156210949:-1; parent_transcript=GRMZM5G890190_T01; parent_gene=GRMZM5G890190	GRMZM5G890190_P01,GRMZM5G890190_P02	TRUE	TRUE	aDDFASPLk	95%	n+304 (+304), K+304 (+304)	39.92	26.09
5577	seq=translation; coord=7:156205011..156210949:-1; parent_transcript=GRMZM5G890190_T01; parent_gene=GRMZM5G890190	GRMZM5G890190_P01,GRMZM5G890190_P02	TRUE	TRUE	lcLEVER	87%	n+304 (+304), Carbamidomethyl (+57)	26.66	25.98

5578	seq=translation; coord=7:156205011..156210949:-1; parent_transcript=GRMZM5G890190_T01; parent_gene=GRMZM5G890190	GRMZM5G890190_P01,GRMZM5G890190_P02	TRUE	TRUE	lSLSAVIMGSk		93%	n+304 (+304), K+304 (+304)	27.29	25.00
5579	seq=translation; coord=4:201936215..201938145:1; parent_transcript=GRMZM2G325118_T01; parent_gene=GRMZM2G325118	GRMZM2G325118_P01	TRUE	TRUE	qAcEWQYGk		95%	n+304 (+304), Carbamidomethyl (+57), K+304 (+304)	41.91	25.00
5580	seq=translation; coord=4:201936215..201938145:1; parent_transcript=GRMZM2G325118_T01; parent_gene=GRMZM2G325118	GRMZM2G325118_P01	TRUE	TRUE	qQcVQAcER		91%	n+304 (+304), Carbamidomethyl (+57), Carbamidomethyl (+57)	28.17	25.00
5581	seq=translation; coord=9:130498884..130500159:1; parent_transcript=GRMZM2G134107_T01; parent_gene=GRMZM2G134107	GRMZM2G134107_P01	TRUE	TRUE	aGQLGEGVTVTR		95%	n+304 (+304)	42.38	26.34
5582	seq=translation; coord=9:130498884..130500159:1; parent_transcript=GRMZM2G134107_T01; parent_gene=GRMZM2G134107	GRMZM2G134107_P01	TRUE	TRUE	iMEIATLEk		94%	n+304 (+304), K+304 (+304)	28.44	25.17
5583	seq=translation; coord=2:8578099..8582568:-1; parent_transcript=GRMZM2G012628_T01; parent_gene=GRMZM2G012628	GRMZM2G012628_P01	TRUE	TRUE	dkNSGQLQGYGFVEFTSR		95%	n+304 (+304), K+304 (+304)	33.48	25.00
5584	seq=translation; coord=2:8578099..8582568:-1; parent_transcript=GRMZM2G012628_T01; parent_gene=GRMZM2G012628	GRMZM2G012628_P01	TRUE	TRUE	qVFSPYGEVVHVk		95%	n+304 (+304), K+304 (+304)	41.67	25.92
5585	seq=translation; coord=2:8578099..8582568:-1; parent_transcript=GRMZM2G012628_T01; parent_gene=GRMZM2G012628	GRMZM2G012628_P01	TRUE	TRUE	qVFSPYGEVVHVk		95%	Pyro-cmC (-17), n+304 (+304), K+304 (+304)	35.22	25.75
5586	seq=translation; coord=7:11441042..11445373:-1; parent_transcript=GRMZM2G401308_T02; parent_gene=GRMZM2G401308	GRMZM2G401308_P02	TRUE	TRUE	mIVEAIAALk		95%	n+304 (+304), K+304 (+304)	43.02	25.00
5587	seq=translation; coord=7:11441042..11445373:-1; parent_transcript=GRMZM2G401308_T02; parent_gene=GRMZM2G401308	GRMZM2G401308_P02	TRUE	TRUE	qLTVQLk		95%	n+304 (+304), K+304 (+304)	37.87	25.33
5588	seq=translation; coord=3:44223365..44228905:-1; parent_transcript=GRMZM2G003883_T01; parent_gene=GRMZM2G003883	GRMZM2G003883_P01	TRUE	TRUE	aALILVLTR		92%	n+304 (+304)	28.56	25.00
5589	seq=translation; coord=3:44223365..44228905:-1; parent_transcript=GRMZM2G003883_T01; parent_gene=GRMZM2G003883	GRMZM2G003883_P01	TRUE	TRUE	iDMIALSFVR		86%	n+304 (+304)	26.38	25.97
5590	seq=translation; coord=1:267894143..267897525:-1; parent_transcript=GRMZM2G024104_T01; parent_gene=GRMZM2G024104	GRMZM2G024104_P01,GRMZM2G024104_P02, GRMZM2G024104_P03,GRMZM2G024104_P04	TRUE	TRUE	aEEPWFGIEQEYTLLQk		95%	n+304 (+304), K+304 (+304)	70.93	25.91
5591	seq=translation; coord=8:29610407..29612711:1; parent_transcript=GRMZM2G067522_T01; parent_gene=GRMZM2G067522	GRMZM2G067522_P01	TRUE	TRUE	aRPGAPAAAPSR		95%	n+304 (+304)	35.61	25.66
5592	seq=translation; coord=8:29610407..29612711:1; parent_transcript=GRMZM2G067522_T01; parent_gene=GRMZM2G067522	GRMZM2G067522_P01	TRUE	TRUE	iAEcLVGDETGAIVFTAR		95%	n+304 (+304), Carbamidomethyl (+57)	55.95	25.00
5593	seq=translation; coord=3:1297208..1299271:1; parent_transcript=GRMZM5G884242_T01; parent_gene=GRMZM5G884242	GRMZM5G884242_P01,GRMZM5G884242_P02, GRMZM5G884242_P03,GRMZM5G884242_P04, GRMZM5G884242_P05	TRUE	TRUE	aYFDSADWALGk		93%	n+304 (+304), K+304 (+304)	26.50	25.00
5594	seq=translation; coord=4:17087184..17096848:1; parent_transcript=GRMZM2G045987_T01; parent_gene=GRMZM2G045987	GRMZM2G045987_P01	TRUE	TRUE	iIALLGGPcTEGPGMIVSk		95%	n+304 (+304), Carbamidomethyl (+57), K+304 (+304)	66.26	25.43
5595	seq=translation; coord=4:17087184..17096848:1; parent_transcript=GRMZM2G045987_T01; parent_gene=GRMZM2G045987	GRMZM2G045987_P01	TRUE	TRUE	lTWNTWPR		93%	n+304 (+304)	29.32	25.63

5596	seq=translation; coord=4:17087184..17096848:1; parent_transcript=GRMZM2G045987_T01; parent_gene=GRMZM2G045987	GRMZM2G045987_P01	TRUE	TRUE	qLVSQGHVLDVFASALDQVGLAEMk	95%	n+304 (+304), K+304 (+304)	33.82	25.00
5597	seq=translation; coord=4:17087184..17096848:1; parent_transcript=GRMZM2G045987_T01; parent_gene=GRMZM2G045987	GRMZM2G045987_P01	TRUE	TRUE	vQGIIGPcTSLEk	95%	n+304 (+304), Carbamidomethyl (+57), K+304 (+304)	36.33	25.42
5598	seq=translation; coord=10:11749519..11756094:-1; parent_transcript=GRMZM2G036534_T01; parent_gene=GRMZM2G036534	GRMZM2G036534_P01	TRUE	TRUE	dVEAALAAPk	95%	n+304 (+304), K+304 (+304)	45.12	25.00
5599	seq=translation; coord=10:11749519..11756094:-1; parent_transcript=GRMZM2G036534_T01; parent_gene=GRMZM2G036534	GRMZM2G036534_P01	TRUE	TRUE	vIYGADLAAFLQTLAk	95%	n+304 (+304), K+304 (+304)	35.04	25.00
5600	seq=translation; coord=4:187884467..187890415:-1; parent_transcript=GRMZM2G119175_T01; parent_gene=GRMZM2G119175	GRMZM2G119175_P01,GRMZM2G119175_P02	TRUE	TRUE	lGDLSQTLIFAk	95%	n+304 (+304), K+304 (+304)	31.77	25.00
5601	seq=translation; coord=4:187884467..187890415:-1; parent_transcript=GRMZM2G119175_T01; parent_gene=GRMZM2G119175	GRMZM2G119175_P01,GRMZM2G119175_P02	TRUE	TRUE	yRPTMPVLSVVIPR	95%	n+304 (+304)	40.92	25.00
5602	seq=translation; coord=7:154188635..154191068:-1; parent_transcript=GRMZM2G056369_T01; parent_gene=GRMZM2G056369	GRMZM2G056369_P01	TRUE	TRUE	eLQAIEDEWLAAAQLk	95%	n+304 (+304), K+304 (+304)	59.01	25.84
5603	seq=translation; coord=7:154188635..154191068:-1; parent_transcript=GRMZM2G056369_T01; parent_gene=GRMZM2G056369	GRMZM2G056369_P01	TRUE	TRUE	gQSLAAVLSAGMSAGk	95%	n+304 (+304), K+304 (+304)	42.36	25.97
5604	seq=translation; coord=7:154188635..154191068:-1; parent_transcript=GRMZM2G056369_T01; parent_gene=GRMZM2G056369	GRMZM2G056369_P01	TRUE	TRUE	vEHLFFAQLYHDR	95%	n+304 (+304)	27.46	25.00
5605	seq=translation; coord=5:30994200..30995891:-1; parent_transcript=GRMZM2G111477_T01; parent_gene=GRMZM2G111477	GRMZM2G111477_P01	TRUE	TRUE	gEYVDMVk	87%	n+304 (+304), K+304 (+304)	25.96	25.66
5606	seq=translation; coord=5:30994200..30995891:-1; parent_transcript=GRMZM2G111477_T01; parent_gene=GRMZM2G111477	GRMZM2G111477_P01	TRUE	TRUE	lLEQENTDLGYDAAk	95%	n+304 (+304), K+304 (+304)	53.20	25.59
5607	seq=translation; coord=10:94095731..94103354:-1; parent_transcript=GRMZM2G174671_T01; parent_gene=GRMZM2G174671	GRMZM2G174671_P01,GRMZM2G174671_P04	TRUE	TRUE	lMHVISESVk	95%	n+304 (+304), K+304 (+304)	33.54	25.99
5608	seq=translation; coord=4:140347561..140350401:1; parent_transcript=GRMZM2G079817_T01; parent_gene=GRMZM2G079817	GRMZM2G079817_P01	TRUE	TRUE	aAPDLAEcLAVMQSLk	95%	n+304 (+304), Carbamidomethyl (+57), K+304 (+304)	39.93	25.60
5609	seq=translation; coord=4:140347561..140350401:1; parent_transcript=GRMZM2G079817_T01; parent_gene=GRMZM2G079817	GRMZM2G079817_P01	TRUE	TRUE	aLSELIPATEk	95%	n+304 (+304), K+304 (+304)	29.53	25.16
5610	seq=translation; coord=4:140347561..140350401:1; parent_transcript=GRMZM2G079817_T01; parent_gene=GRMZM2G079817	GRMZM2G079817_P01	TRUE	TRUE	lPDLVELYLSDLNLENk	94%	n+304 (+304), K+304 (+304)	27.87	25.00
5611	seq=translation; coord=4:140347561..140350401:1; parent_transcript=GRMZM2G079817_T01; parent_gene=GRMZM2G079817	GRMZM2G079817_P01	TRUE	TRUE	tIEELcFALADEHFR	95%	n+304 (+304), Carbamidomethyl (+57)	55.27	25.00
5612	seq=translation; coord=1:189145769..189150346:-1; parent_transcript=GRMZM2G088627_T01; parent_gene=GRMZM2G088627	GRMZM2G088627_P01	TRUE	TRUE	aEGDVVSVNFAYLk	95%	n+304 (+304), K+304 (+304)	40.99	25.43
5613	seq=translation; coord=1:189145769..189150346:-1; parent_transcript=GRMZM2G088627_T01; parent_gene=GRMZM2G088627	GRMZM2G088627_P01	TRUE	TRUE	gIGIYESIIR	95%	n+304 (+304)	33.46	25.99

5614	seq=translation; coord=1:189145769..189150346:-1; parent_transcript=GRMZM2G088627_T01; parent_gene=GRMZM2G088627	GRMZM2G088627_P01	TRUE	TRUE	lYDGSAMENLMk	94%	n+304 (+304), K+304 (+304)	28.66	25.00
5615	seq=translation; coord=6:151454748..151457344:-1; parent_transcript=GRMZM2G059314_T01; parent_gene=GRMZM2G059314	GRMZM2G059314_P01	TRUE	TRUE	aIGVSNFSSk	95%	n+304 (+304), K+304 (+304)	36.43	25.89
5616	seq=translation; coord=6:151454748..151457344:-1; parent_transcript=GRMZM2G059314_T01; parent_gene=GRMZM2G059314	GRMZM2G059314_P01	TRUE	TRUE	aNLDVFGWSLPEDLLAk	95%	n+304 (+304), K+304 (+304)	29.74	25.28
5617	seq=translation; coord=6:151454748..151457344:-1; parent_transcript=GRMZM2G059314_T01; parent_gene=GRMZM2G059314	GRMZM2G059314_P01	TRUE	TRUE	dLQTDYVDLYLMHWPVR	95%	n+304 (+304)	63.56	25.00
5618	seq=translation; coord=6:151454748..151457344:-1; parent_transcript=GRMZM2G059314_T01; parent_gene=GRMZM2G059314	GRMZM2G059314_P01	TRUE	TRUE	lADLLAVAR	95%	n+304 (+304)	40.28	25.00
5619	seq=translation; coord=5:13726531..13727525:-1; parent_transcript=GRMZM2G129083_T01; parent_gene=GRMZM2G129083	GRMZM2G129083_P01	TRUE	TRUE	cFSSYSGAGk	95%	Carbamidomethyl (+57), n+304 (+304), K+304 (+304)	41.68	25.00
5620	seq=translation; coord=5:13726531..13727525:-1; parent_transcript=GRMZM2G129083_T01; parent_gene=GRMZM2G129083	GRMZM2G129083_P01	TRUE	TRUE	gGVVAPTVTcSEk	95%	n+304 (+304), Carbamidomethyl (+57), K+304 (+304)	33.16	26.15
5621	seq=translation; coord=5:13726531..13727525:-1; parent_transcript=GRMZM2G129083_T01; parent_gene=GRMZM2G129083	GRMZM2G129083_P01	TRUE	TRUE	gYGGGGGGGScTVDck	95%	n+304 (+304), Carbamidomethyl (+57), Carbamidomethyl (+57), K+304 (+304)	55.76	25.00
5622	seq=translation; coord=9:22677939..22681262:-1; parent_transcript=GRMZM5G877500_T01; parent_gene=GRMZM5G877500	GRMZM5G877500_P01	TRUE	TRUE	lNVTAIDTYDDHR	95%	n+304 (+304)	48.14	25.00
5623	seq=translation; coord=9:22677939..22681262:-1; parent_transcript=GRMZM5G877500_T01; parent_gene=GRMZM5G877500	GRMZM5G877500_P01	TRUE	TRUE	tFPDYFDVLSTFVk	95%	n+304 (+304), K+304 (+304)	40.16	26.51
5624	seq=translation; coord=3:160397311..160408336:1; parent_transcript=GRMZM2G129155_T01; parent_gene=GRMZM2G129155	GRMZM2G129155_P01,GRMZM2G180335_P01	TRUE	TRUE	iFSPNVLNITLVDLPGITk	95%	n+304 (+304), K+304 (+304)	41.16	25.00
5625	seq=translation; coord=3:160397311..160408336:1; parent_transcript=GRMZM2G129155_T01; parent_gene=GRMZM2G129155	GRMZM2G129155_P01,GRMZM2G180335_P01	TRUE	TRUE	tLDEIPLDAEAVER	95%	n+304 (+304)	35.08	25.44
5626	seq=translation; coord=1:132810230..132856187:1; parent_transcript=GRMZM2G019673_T01; parent_gene=GRMZM2G019673	GRMZM2G019673_P01,GRMZM2G019673_P04, GRMZM2G019673_P05	TRUE	TRUE	eVFSALILSADILGk	95%	n+304 (+304), K+304 (+304)	46.80	25.00
5627	seq=translation; coord=1:132810230..132856187:1; parent_transcript=GRMZM2G019673_T01; parent_gene=GRMZM2G019673	GRMZM2G019673_P01,GRMZM2G019673_P04, GRMZM2G019673_P05	TRUE	TRUE	fLLDWLIEGHR	95%	n+304 (+304)	51.76	26.51
5628	seq=translation; coord=4:237676754..237682663:-1; parent_transcript=GRMZM2G117346_T01; parent_gene=GRMZM2G117346	GRMZM2G117346_P01,GRMZM2G117346_P02, GRMZM2G410393_P01,GRMZM2G410393_P03, GRMZM2G410393_P04,GRMZM2G410393_P05	TRUE	TRUE	aIGPQDVLATLLNNLk	95%	n+304 (+304), K+304 (+304)	31.53	25.00
5629	seq=translation; coord=4:237676754..237682663:-1; parent_transcript=GRMZM2G117346_T01; parent_gene=GRMZM2G117346	GRMZM2G117346_P01,GRMZM2G117346_P02, GRMZM2G410393_P01,GRMZM2G410393_P03, GRMZM2G410393_P04,GRMZM2G410393_P05	TRUE	TRUE	tSSDWDAPDATPGIGR	95%	n+304 (+304)	78.79	25.00
5630	seq=translation; coord=2:45890416..45891705:-1; parent_transcript=GRMZM2G152141_T01; parent_gene=GRMZM2G152141	GRMZM2G152141_P01	TRUE	TRUE	gYVSDMEVVGk	95%	n+304 (+304), K+304 (+304)	57.11	25.07

5631	seq=translation; coord=2:45890416..45891705:-1; parent_transcript=GRMZM2G152141_T01; parent_gene=GRMZM2G152141	GRMZM2G152141_P01	TRUE	TRUE	yIEALNVVHSAVHALATGNLR	95%	n+304 (+304)	59.20	25.56
5632	seq=translation; coord=8:135417381..135418239:1; parent_transcript=GRMZM2G175273_T01; parent_gene=GRMZM2G175273	GRMZM2G175273_P01	TRUE	TRUE	aQQAAAGGTGTSTDNNk	95%	n+304 (+304), K+304 (+304)	73.24	25.07
5633	seq=translation; coord=8:135417381..135418239:1; parent_transcript=GRMZM2G175273_T01; parent_gene=GRMZM2G175273	GRMZM2G175273_P01	TRUE	TRUE	nSQDGLTPEQR	95%	n+304 (+304)	39.30	25.00
5634	seq=translation; coord=4:183588190..183591209:-1; parent_transcript=GRMZM2G325575_T01; parent_gene=GRMZM2G325575	GRMZM2G325575_P01	TRUE	TRUE	gDALYAMELALALEk	95%	n+304 (+304), K+304 (+304)	38.90	25.72
5635	seq=translation; coord=4:183588190..183591209:-1; parent_transcript=GRMZM2G325575_T01; parent_gene=GRMZM2G325575	GRMZM2G325575_P01	TRUE	TRUE	gHGVWHFDQMLLEEEA	95%	n+304 (+304)	31.07	25.00
5636	seq=translation; coord=6:115639609..115647217:-1; parent_transcript=GRMZM2G008556_T01; parent_gene=GRMZM2G008556	GRMZM2G008556_P01,GRMZM2G039325_P01, GRMZM5G832989_P01,GRMZM5G832989_P02	TRUE	TRUE	dDDMPPIEAILAPLEAELk	95%	n+304 (+304), K+304 (+304)	42.17	25.42
5637	seq=translation; coord=6:115639609..115647217:-1; parent_transcript=GRMZM2G008556_T01; parent_gene=GRMZM2G008556	GRMZM2G008556_P01,GRMZM2G039325_P01, GRMZM5G832989_P01,GRMZM5G832989_P02	TRUE	TRUE	eAALLEAER	89%	n+304 (+304)	27.51	25.89
5638	seq=translation; coord=8:12273514..12274222:-1; parent_transcript=GRMZM2G156632_T01; parent_gene=GRMZM2G156632	GRMZM2G156632_P01	TRUE	TRUE	aIDINPGQLk	95%	n+304 (+304), K+304 (+304)	35.41	25.43
5639	seq=translation; coord=1:296022541..296026398:-1; parent_transcript=GRMZM2G053299_T01; parent_gene=GRMZM2G053299	GRMZM2G053299_P01	TRUE	TRUE	lAFVALDYEQELETAk	95%	n+304 (+304), K+304 (+304)	37.29	25.80
5640	seq=translation; coord=3:196019180..196024451:1; parent_transcript=GRMZM2G324886_T01; parent_gene=GRMZM2G324886	GRMZM2G324886_P01	TRUE	TRUE	dPMSGIMDLMk	93%	n+304 (+304), K+304 (+304)	27.72	25.00
5641	seq=translation; coord=3:196019180..196024451:1; parent_transcript=GRMZM2G324886_T01; parent_gene=GRMZM2G324886	GRMZM2G324886_P01	TRUE	TRUE	lELDELR	95%	n+304 (+304)	34.32	27.40
5642	seq=translation; coord=4:168987228..168990822:1; parent_transcript=GRMZM2G018947_T01; parent_gene=GRMZM2G018947	GRMZM2G018947_P01,GRMZM2G133764_P01, GRMZM2G133764_P02	TRUE	TRUE	lLVLDEADEMLSR	93%	n+304 (+304)	29.71	25.00
5643	seq=translation; coord=4:168987228..168990822:1; parent_transcript=GRMZM2G018947_T01; parent_gene=GRMZM2G018947	GRMZM2G018947_P01,GRMZM2G133764_P01, GRMZM2G133764_P02	TRUE	TRUE	vLITTDVWAR	95%	n+304 (+304)	32.54	25.04
5644	seq=translation; coord=4:168987228..168990822:1; parent_transcript=GRMZM2G018947_T01; parent_gene=GRMZM2G018947	GRMZM2G018947_P01,GRMZM2G133764_P01, GRMZM2G133764_P02	TRUE	TRUE	yLPPELQVVLISATLPHEILEMTSk	95%	n+304 (+304), K+304 (+304)	47.96	25.00
5645	seq=translation; coord=8:157966228..157972830:-1; parent_transcript=GRMZM2G016827_T02; parent_gene=GRMZM2G016827	GRMZM2G016827_P02	TRUE	TRUE	mGMEIIETGDPVAFk	92%	n+304 (+304), K+304 (+304)	26.61	25.87
5646	seq=translation; coord=8:157966228..157972830:-1; parent_transcript=GRMZM2G016827_T02; parent_gene=GRMZM2G016827	GRMZM2G016827_P02	TRUE	TRUE	vAGASGEEDkQD	95%	n+304 (+304), K+304 (+304)	54.83	25.00
5647	seq=translation; coord=8:157966228..157972830:-1; parent_transcript=GRMZM2G016827_T02; parent_gene=GRMZM2G016827	GRMZM2G016827_P02	TRUE	TRUE	vVPILVGALSSQNEALYGQLLSk	95%	n+304 (+304), K+304 (+304)	40.25	25.00
5648	seq=translation; coord=8:2349948..2353138:1; parent_transcript=GRMZM2G027728_T01; parent_gene=GRMZM2G027728	GRMZM2G027728_P01,GRMZM2G050460_P01	TRUE	TRUE	lQLVMk	90%	n+304 (+304), K+304 (+304)	27.62	25.00

5649	seq=translation; coord=8:2349948..2353138:1; parent_transcript=GRMZM2G027728_T01; parent_gene=GRMZM2G027728	GRMZM2G027728_P01,GRMZM2G050460_P01	TRUE	TRUE	vccLSIIDPGDSDIIk	95%	n+304 (+304), Carbamidomethyl (+57), Carbamidomethyl (+57), K+304 (+304)	32.28	25.19
5650	seq=translation; coord=7:1306578..1314769:-1; parent_transcript=GRMZM2G120563_T06; parent_gene=GRMZM2G120563	GRMZM2G120563_P06,GRMZM2G120563_P07	TRUE	TRUE	eGVFHLR	91%	n+304 (+304)	27.04	26.01
5651	seq=translation; coord=7:1306578..1314769:-1; parent_transcript=GRMZM2G120563_T06; parent_gene=GRMZM2G120563	GRMZM2G120563_P06,GRMZM2G120563_P07	TRUE	TRUE	gVMQILNTIIR	95%	n+304 (+304)	32.64	25.00
5652	seq=translation; coord=1:197854710..197858129:-1; parent_transcript=GRMZM2G021816_T01; parent_gene=GRMZM2G021816	GRMZM2G021816_P01	TRUE	TRUE	dIYEILMDENR	95%	n+304 (+304)	45.23	25.00
5653	seq=translation; coord=1:197854710..197858129:-1; parent_transcript=GRMZM2G021816_T01; parent_gene=GRMZM2G021816	GRMZM2G021816_P01	TRUE	TRUE	eAVLAPAPAVEk	95%	n+304 (+304), K+304 (+304)	29.02	25.00
5654	seq=translation; coord=1:197854710..197858129:-1; parent_transcript=GRMZM2G021816_T01; parent_gene=GRMZM2G021816	GRMZM2G021816_P01	TRUE	TRUE	gDETPLALR	88%	n+304 (+304)	27.09	25.77
5655	seq=translation; coord=3:134285297..134289508:1; parent_transcript=GRMZM2G047434_T01; parent_gene=GRMZM2G047434	GRMZM2G047434_P01	TRUE	TRUE	aLDVLFETAMISSGFSPDNPAELSGk	95%	n+304 (+304), K+304 (+304)	34.96	25.00
5656	seq=translation; coord=3:134285297..134289508:1; parent_transcript=GRMZM2G047434_T01; parent_gene=GRMZM2G047434	GRMZM2G047434_P01	TRUE	TRUE	fVDISkEDLDLGDNNEER	95%	n+304 (+304), K+304 (+304)	41.28	25.00
5657	seq=translation; coord=3:134285297..134289508:1; parent_transcript=GRMZM2G047434_T01; parent_gene=GRMZM2G047434	GRMZM2G047434_P01	TRUE	TRUE	qEFSQAcDWIk	95%	n+304 (+304), Carbamidomethyl (+57), K+304 (+304)	38.03	25.00
5658	seq=translation; coord=7:161685025..161693219:-1; parent_transcript=GRMZM2G396397_T01; parent_gene=GRMZM2G396397	GRMZM2G396397_P01	TRUE	TRUE	vLIYSPAR	89%	n+304 (+304)	26.05	25.00
5659	seq=translation; coord=6:76236848..76238660:1; parent_transcript=GRMZM2G051338_T01; parent_gene=GRMZM2G051338	GRMZM2G051338_P01	TRUE	TRUE	fVLVLFQQLGR	95%	n+304 (+304)	46.18	25.45
5660	seq=translation; coord=6:76236848..76238660:1; parent_transcript=GRMZM2G051338_T01; parent_gene=GRMZM2G051338	GRMZM2G051338_P01	TRUE	TRUE	vVGDVLDPFVR	88%	n+304 (+304)	28.13	26.92
5661	seq=translation; coord=2:192412235..192414620:-1; parent_transcript=GRMZM2G178968_T01; parent_gene=GRMZM2G178968	GRMZM2G178968_P01,GRMZM5G870752_P01	TRUE	TRUE	vTNPLFEk	95%	n+304 (+304), K+304 (+304)	37.09	25.00
5662	seq=translation; coord=2:20765568..20767537:1; parent_transcript=GRMZM2G337229_T01; parent_gene=GRMZM2G337229	GRMZM2G337229_P01	TRUE	TRUE	gATGGGGGYGDLQR	95%	n+304 (+304)	54.26	25.00
5663	seq=translation; coord=2:20765568..20767537:1; parent_transcript=GRMZM2G337229_T01; parent_gene=GRMZM2G337229	GRMZM2G337229_P01	TRUE	TRUE	qGAMMTALk	95%	n+304 (+304), K+304 (+304)	30.61	26.25
5664	seq=translation; coord=3:202257167..202260653:-1; parent_transcript=GRMZM2G116282_T02; parent_gene=GRMZM2G116282	GRMZM2G116282_P02	TRUE	TRUE	sLQPLSNk	91%	n+304 (+304), K+304 (+304)	26.33	25.93
5665	seq=translation; coord=3:221235623..221241542:1; parent_transcript=GRMZM2G149321_T01; parent_gene=GRMZM2G149321	GRMZM2G149321_P01	TRUE	TRUE	dVAEcNEQPNccTEDER	95%	n+304 (+304), Carbamidomethyl (+57), Carbamidomethyl (+57), Carbamidomethyl (+57)	59.76	25.00

5666	seq=translation; coord=3:221235623..221241542:1; parent_transcript=GRMZM2G149321_T01; parent_gene=GRMZM2G149321	GRMZM2G149321_P01	TRUE	TRUE	tVDEPSEAER	95%	n+304 (+304)	32.45	25.00
5667	seq=translation; coord=3:226173114..226176070:1; parent_transcript=GRMZM2G015364_T01; parent_gene=GRMZM2G015364	GRMZM2G015364_P01,GRMZM2G015364_P02, GRMZM2G094595_P01,GRMZM2G094595_P02, GRMZM2G094595_P03,GRMZM2G094595_P04	TRUE	TRUE	eIDPSESGIATTAR	95%	n+304 (+304)	43.11	25.00
5668	seq=translation; coord=1:237087363..237092641:-1; parent_transcript=GRMZM2G036765_T01; parent_gene=GRMZM2G036765	GRMZM2G036765_P01	TRUE	TRUE	dYSTAILER	95%	n+304 (+304)	36.06	25.00
5669	seq=translation; coord=8:24719639..24721093:1; parent_transcript=GRMZM2G047139_T01; parent_gene=GRMZM2G047139	GRMZM2G047139_P01,GRMZM2G047139_P02	TRUE	TRUE	lVVDFIAQELGVPLLPPSk	95%	n+304 (+304), K+304 (+304)	32.20	25.00
5670	seq=translation; coord=2:220468734..220469483:-1; parent_transcript=GRMZM2G081464_T01; parent_gene=GRMZM2G081464	GRMZM2G081464_P01	TRUE	TRUE	lFAIcNLPMPR	95%	n+304 (+304), Carbamidomethyl (+57)	35.93	26.02
5671	seq=translation; coord=8:154379685..154384907:1; parent_transcript=GRMZM2G102404_T01; parent_gene=GRMZM2G102404	GRMZM2G102404_P01,GRMZM2G102404_P02	TRUE	TRUE	sAGLAALVDALVAAGR	95%	n+304 (+304)	48.72	25.00
5672	seq=translation; coord=6:99761240..99767447:-1; parent_transcript=GRMZM2G112337_T03; parent_gene=GRMZM2G112337	GRMZM2G112337_P03,GRMZM2G112337_P04	TRUE	TRUE	iPAIVDTLLAk	95%	n+304 (+304), K+304 (+304)	32.13	25.00
5673	seq=translation; coord=2:204757521..204759565:-1; parent_transcript=GRMZM2G142922_T01; parent_gene=GRMZM2G142922	GRMZM2G142922_P01	TRUE	TRUE	aAASFAALVAALAk	95%	n+304 (+304), K+304 (+304)	34.22	25.00
5674	seq=translation; coord=2:135017876..135044554:1; parent_transcript=GRMZM2G162286_T01; parent_gene=GRMZM2G162286	GRMZM2G162286_P01	TRUE	TRUE	aLPLPPDLLDPLLLSSLR	95%	n+304 (+304)	50.50	25.00
5675	seq=translation; coord=1:264855610..264864472:-1; parent_transcript=AC217401.3_FGT002; parent_gene=AC217401.3_FG002	AC217401.3_FGP002	TRUE	TRUE	fGIVYVDFATLk	95%	n+304 (+304), K+304 (+304)	51.89	25.90
5676	seq=translation; coord=1:264855610..264864472:-1; parent_transcript=AC217401.3_FGT002; parent_gene=AC217401.3_FG002	AC217401.3_FGP002	TRUE	TRUE	gFVFGTATSAYQVEGAATSGGR	95%	n+304 (+304)	55.90	25.00
5677	seq=translation; coord=1:264855610..264864472:-1; parent_transcript=AC217401.3_FGT002; parent_gene=AC217401.3_FG002	AC217401.3_FGP002	TRUE	TRUE	iVAFLGYDk	95%	n+304 (+304), K+304 (+304)	37.52	25.00
5678	seq=translation; coord=1:264855610..264864472:-1; parent_transcript=AC217401.3_FGT002; parent_gene=AC217401.3_FG002	AC217401.3_FGP002	TRUE	TRUE	iVDIFADYADFcFk	95%	n+304 (+304), Carbamidomethyl (+57), K+304 (+304)	38.16	25.00
5679	seq=translation; coord=1:269493908..269506409:1; parent_transcript=GRMZM5G826838_T01; parent_gene=GRMZM5G826838	GRMZM5G826838_P01	TRUE	TRUE	eAAYQIINDELMLDGNPR	92%	n+304 (+304)	26.55	25.00
5680	seq=translation; coord=1:269493908..269506409:1; parent_transcript=GRMZM5G826838_T01; parent_gene=GRMZM5G826838	GRMZM5G826838_P01	TRUE	TRUE	gSSQVIAQYYQLIR	95%	n+304 (+304)	47.95	26.66
5681	seq=translation; coord=1:269493908..269506409:1; parent_transcript=GRMZM5G826838_T01; parent_gene=GRMZM5G826838	GRMZM5G826838_P01	TRUE	TRUE	lLNDLLTk	95%	n+304 (+304), K+304 (+304)	31.38	25.00
5682	seq=translation; coord=9:12103632..12110868:-1; parent_transcript=GRMZM2G467169_T01; parent_gene=GRMZM2G467169	GRMZM2G467169_P01,GRMZM2G467169_P02	TRUE	TRUE	dDSVAILEQFFGNVLSk	95%	n+304 (+304), K+304 (+304)	57.99	25.33

5683	seq=translation; coord=9:12103632..12110868:-1; parent_transcript=GRMZM2G467169_T01; parent_gene=GRMZM2G467169	GRMZM2G467169_P01,GRMZM2G467169_P02	TRUE	TRUE	dLTLEALFGAAFMNELHSk	95%	n+304 (+304), K+304 (+304)	71.62	25.72
5684	seq=translation; coord=9:12103632..12110868:-1; parent_transcript=GRMZM2G467169_T01; parent_gene=GRMZM2G467169	GRMZM2G467169_P01,GRMZM2G467169_P02	TRUE	TRUE	sLLSMIVk	92%	n+304 (+304), K+304 (+304)	26.12	25.00
5685	seq=translation; coord=1:278837830..278839720:1; parent_transcript=GRMZM2G118809_T01; parent_gene=GRMZM2G118809	GRMZM2G118809_P01,GRMZM2G154870_P01	TRUE	TRUE	lLGADDMPHLPYLQcVLTETLR	95%	n+304 (+304), Carbamidomethyl (+57)	46.79	25.00
5686	seq=translation; coord=1:278837830..278839720:1; parent_transcript=GRMZM2G118809_T01; parent_gene=GRMZM2G118809	GRMZM2G118809_P01,GRMZM2G154870_P01	TRUE	TRUE	sMMAVLLTLQk	95%	n+304 (+304), K+304 (+304)	31.01	25.00
5687	seq=translation; coord=9:138771755..138777249:1; parent_transcript=GRMZM2G162426_T01; parent_gene=GRMZM2G162426	GRMZM2G162426_P01	TRUE	TRUE	lADIGVAMGITGTEVAk	95%	n+304 (+304), K+304 (+304)	48.87	25.72
5688	seq=translation; coord=9:138771755..138777249:1; parent_transcript=GRMZM2G162426_T01; parent_gene=GRMZM2G162426	GRMZM2G162426_P01	TRUE	TRUE	tPSEcLAELGVSADR	95%	n+304 (+304), Carbamidomethyl (+57)	67.41	25.00
5689	seq=translation; coord=9:138771755..138777249:1; parent_transcript=GRMZM2G162426_T01; parent_gene=GRMZM2G162426	GRMZM2G162426_P01	TRUE	TRUE	vLQLISSTLR	92%	n+304 (+304)	28.81	25.00
5690	seq=translation; coord=1:276433043..276437555:-1; parent_transcript=GRMZM2G100084_T01; parent_gene=GRMZM2G100084	GRMZM2G100084_P01	TRUE	TRUE	fIPYTMQAVk	95%	n+304 (+304), K+304 (+304)	35.51	25.47
5691	seq=translation; coord=1:276433043..276437555:-1; parent_transcript=GRMZM2G100084_T01; parent_gene=GRMZM2G100084	GRMZM2G100084_P01	TRUE	TRUE	gMGSLEAMTk	95%	n+304 (+304), K+304 (+304)	50.49	25.00
5692	seq=translation; coord=1:276433043..276437555:-1; parent_transcript=GRMZM2G100084_T01; parent_gene=GRMZM2G100084	GRMZM2G100084_P01	TRUE	TRUE	qGLQDLGADSVQSAHDLLR	95%	n+304 (+304)	45.65	25.00
5693	seq=translation; coord=4:177628395..177630803:-1; parent_transcript=GRMZM2G126732_T01; parent_gene=GRMZM2G126732	GRMZM2G126732_P01,GRMZM2G126732_P02	TRUE	TRUE	aHTDAGGLILLLQDDQVSGLQLLR	95%	n+304 (+304)	31.06	25.55
5694	seq=translation; coord=4:177628395..177630803:-1; parent_transcript=GRMZM2G126732_T01; parent_gene=GRMZM2G126732	GRMZM2G126732_P01,GRMZM2G126732_P02	TRUE	TRUE	gGDGGEWVDVPPLR	95%	n+304 (+304)	45.99	25.00
5695	seq=translation; coord=4:177628395..177630803:-1; parent_transcript=GRMZM2G126732_T01; parent_gene=GRMZM2G126732	GRMZM2G126732_P01,GRMZM2G126732_P02	TRUE	TRUE	vLDLLcENLGLEPGYLk	95%	n+304 (+304), Carbamidomethyl (+57), K+304 (+304)	64.17	25.00
5696	seq=translation; coord=9:26817670..26821498:1; parent_transcript=GRMZM2G475293_T01; parent_gene=GRMZM2G475293	GRMZM2G475293_P01,GRMZM2G475293_P02	TRUE	TRUE	aGVAAGMPVVGVATR	95%	n+304 (+304)	48.24	26.12
5697	seq=translation; coord=9:26817670..26821498:1; parent_transcript=GRMZM2G475293_T01; parent_gene=GRMZM2G475293	GRMZM2G475293_P01,GRMZM2G475293_P02	TRUE	TRUE	sDVEAAQNLFPDWELEk	95%	n+304 (+304), K+304 (+304)	34.46	25.04
5698	seq=translation; coord=9:26817670..26821498:1; parent_transcript=GRMZM2G475293_T01; parent_gene=GRMZM2G475293	GRMZM2G475293_P01,GRMZM2G475293_P02	TRUE	TRUE	sLVEAGAALLVk	95%	n+304 (+304), K+304 (+304)	52.59	25.00
5699	seq=translation; coord=3:166381506..166384595:-1; parent_transcript=GRMZM2G359397_T01; parent_gene=GRMZM2G359397	GRMZM2G359397_P01,GRMZM2G359397_P02, GRMZM2G359397_P03	TRUE	TRUE	aSQEFLDMR	95%	n+304 (+304)	38.67	25.00
5700	seq=translation; coord=3:166381506..166384595:-1; parent_transcript=GRMZM2G359397_T01; parent_gene=GRMZM2G359397	GRMZM2G359397_P01,GRMZM2G359397_P02, GRMZM2G359397_P03	TRUE	TRUE	vLPGVHNFVATLIEGR	95%	n+304 (+304)	62.17	25.63

5701	seq=translation; coord=3:166381506..166384595:-1; parent_transcript=GRMZM2G359397_T01; parent_gene=GRMZM2G359397	GRMZM2G359397_P01,GRMZM2G359397_P02, GRMZM2G359397_P03	TRUE	TRUE	vSPLEVIFR	91%	n+304 (+304)	27.14	25.00
5702	seq=translation; coord=2:199547128..199550293:1; parent_transcript=GRMZM5G868679_T01; parent_gene=GRMZM5G868679	GRMZM5G868679_P01	TRUE	TRUE	aSPLVLGHk	95%	n+304 (+304), K+304 (+304)	30.75	25.00
5703	seq=translation; coord=2:199547128..199550293:1; parent_transcript=GRMZM5G868679_T01; parent_gene=GRMZM5G868679	GRMZM5G868679_P01	TRUE	TRUE	lLAPAGTLTFDR	95%	n+304 (+304)	39.25	25.35
5704	seq=translation; coord=2:199547128..199550293:1; parent_transcript=GRMZM5G868679_T01; parent_gene=GRMZM5G868679	GRMZM5G868679_P01	TRUE	TRUE	qPWLIFLAHR	94%	Pyro-cmC (-17), n+304 (+304)	31.03	25.60
5705	seq=translation; coord=4:11466211..11470058:-1; parent_transcript=GRMZM2G085711_T02; parent_gene=GRMZM2G085711	GRMZM2G085711_P02	TRUE	TRUE	sILPLLEk	92%	n+304 (+304), K+304 (+304)	26.11	25.00
5706	seq=translation; coord=4:11466211..11470058:-1; parent_transcript=GRMZM2G085711_T02; parent_gene=GRMZM2G085711	GRMZM2G085711_P02	TRUE	TRUE	sIVPSWLMk	95%	n+304 (+304), K+304 (+304)	32.29	26.28
5707	seq=translation; coord=4:11466211..11470058:-1; parent_transcript=GRMZM2G085711_T02; parent_gene=GRMZM2G085711	GRMZM2G085711_P02	TRUE	TRUE	yAVIFDAGSTGSR	92%	n+304 (+304)	27.41	25.00
5708	seq=translation; coord=7:7954180..7955128:1; parent_transcript=GRMZM2G021149_T01; parent_gene=GRMZM2G021149	GRMZM2G021149_P01,GRMZM2G022041_P01	TRUE	TRUE	gEEVVSMTVEGPPPPDESR	91%	n+304 (+304)	25.75	25.00
5709	seq=translation; coord=7:7954180..7955128:1; parent_transcript=GRMZM2G021149_T01; parent_gene=GRMZM2G021149	GRMZM2G021149_P01,GRMZM2G022041_P01	TRUE	TRUE	hMNLVLGDcEEFR	95%	n+304 (+304), Carbamidomethyl (+57)	46.23	25.00
5710	seq=translation; coord=7:152772009..152776227:1; parent_transcript=GRMZM2G108849_T02; parent_gene=GRMZM2G108849	GRMZM2G108849_P02,GRMZM2G108849_P03, GRMZM2G108849_P04	TRUE	TRUE	gWGDDGYFk	95%	n+304 (+304), K+304 (+304)	39.08	25.00
5711	seq=translation; coord=7:152772009..152776227:1; parent_transcript=GRMZM2G108849_T02; parent_gene=GRMZM2G108849	GRMZM2G108849_P02,GRMZM2G108849_P03, GRMZM2G108849_P04	TRUE	TRUE	nGPVEVAFTVYEDFAHYk	95%	n+304 (+304), K+304 (+304)	28.26	25.00
5712	seq=translation; coord=10:144683393..144689170:-1; parent_transcript=GRMZM2G136895_T01; parent_gene=GRMZM2G136895	GRMZM2G136895_P01	TRUE	TRUE	aMHNVGLAGLTFWSPNINIFR	95%	n+304 (+304)	61.89	25.44
5713	seq=translation; coord=10:144683393..144689170:-1; parent_transcript=GRMZM2G136895_T01; parent_gene=GRMZM2G136895	GRMZM2G136895_P01	TRUE	TRUE	lGVPLYEWWSEALHGVSyVGPGTR	95%	n+304 (+304), iTRAQ8plex (+304)	41.57	25.00
5714	seq=translation; coord=3:91357080..91360381:-1; parent_transcript=GRMZM2G077415_T01; parent_gene=GRMZM2G077415	GRMZM2G077415_P01	TRUE	TRUE	aNTFVGEVLGLDPR	95%	n+304 (+304)	51.42	25.22
5715	seq=translation; coord=3:91357080..91360381:-1; parent_transcript=GRMZM2G077415_T01; parent_gene=GRMZM2G077415	GRMZM2G077415_P01	TRUE	TRUE	lLGVTTLDVVR	95%	n+304 (+304)	39.90	25.00
5716	seq=translation; coord=7:149132369..149134157:-1; parent_transcript=GRMZM2G077673_T01; parent_gene=GRMZM2G077673	GRMZM2G077673_P01	TRUE	TRUE	dGLIISDMDLNLcR	95%	n+304 (+304), Carbamidomethyl (+57)	62.26	25.00
5717	seq=translation; coord=7:149132369..149134157:-1; parent_transcript=GRMZM2G077673_T01; parent_gene=GRMZM2G077673	GRMZM2G077673_P01	TRUE	TRUE	yNMVIVSPILER	95%	n+304 (+304)	41.99	25.13
5718	seq=translation; coord=4:184547632..184552118:-1; parent_transcript=GRMZM2G347541_T01; parent_gene=GRMZM2G347541	GRMZM2G347541_P01,GRMZM2G389362_P01	TRUE	TRUE	akPGAFPTFLLVDR	95%	n+304 (+304), K+304 (+304)	34.39	25.00

5719	seq=translation; coord=4:184547632..184552118:-1; parent_transcript=GRMZM2G347541_T01; parent_gene=GRMZM2G347541	GRMZM2G347541_P01,GRMZM2G389362_P01	TRUE	TRUE	kPWLWWDYVTDFAIR	95%	K+304 (+304), n+304 (+304)	30.87	25.55
5720	seq=translation; coord=7:1269463..1270529:-1; parent_transcript=GRMZM2G420733_T01; parent_gene=GRMZM2G420733	GRMZM2G420733_P01	TRUE	TRUE	eIPVVGGTGTFR	94%	n+304 (+304)	29.27	25.85
5721	seq=translation; coord=7:1269463..1270529:-1; parent_transcript=GRMZM2G420733_T01; parent_gene=GRMZM2G420733	GRMZM2G420733_P01	TRUE	TRUE	mATGYVLWk	95%	n+304 (+304), K+304 (+304)	33.36	25.08
5722	seq=translation; coord=3:207473256..207484650:-1; parent_transcript=GRMZM2G073045_T01; parent_gene=GRMZM2G073045	GRMZM2G073045_P01	TRUE	TRUE	dASEIFR	86%	n+304 (+304)	25.30	25.00
5723	seq=translation; coord=3:207473256..207484650:-1; parent_transcript=GRMZM2G073045_T01; parent_gene=GRMZM2G073045	GRMZM2G073045_P01	TRUE	TRUE	gTLDAIGLHPDGR	95%	n+304 (+304)	46.91	25.47
5724	seq=translation; coord=9:154544227..154545597:-1; parent_transcript=GRMZM2G037327_T01; parent_gene=GRMZM2G037327	GRMZM2G037327_P01	TRUE	TRUE	aGAGALAAPLAVGLLAGANVLAcGALEGAVMNPAR	95%	n+304 (+304), Carbamidomethyl (+57)	50.23	25.00
5725	seq=translation; coord=6:31648456..31650307:1; parent_transcript=GRMZM2G025959_T01; parent_gene=GRMZM2G025959	GRMZM2G025959_P01	TRUE	TRUE	fVSDFPIYDGR	91%	n+304 (+304)	27.84	25.00
5726	seq=translation; coord=6:31648456..31650307:1; parent_transcript=GRMZM2G025959_T01; parent_gene=GRMZM2G025959	GRMZM2G025959_P01	TRUE	TRUE	tIDLGDAEVVR	95%	n+304 (+304)	34.99	25.58
5727	seq=translation; coord=5:3145791..3147542:-1; parent_transcript=GRMZM2G332976_T01; parent_gene=GRMZM2G332976	GRMZM2G332976_P01,GRMZM2G332976_P02, GRMZM2G332976_P03,GRMZM2G332976_P04, GRMZM2G332976_P05	TRUE	TRUE	vcIADIQDEAGQQLR	95%	n+304 (+304), Carbamidomethyl (+57)	69.03	25.00
5728	seq=translation; coord=10:130448044..130450386:1; parent_transcript=GRMZM2G018760_T01; parent_gene=GRMZM2G018760	GRMZM2G018760_P01	TRUE	TRUE	lSDMcGVDLGDAALR	95%	n+304 (+304), Carbamidomethyl (+57)	66.87	25.00
5729	seq=translation; coord=4:233220256..233225418:1; parent_transcript=GRMZM2G029566_T01; parent_gene=GRMZM2G029566	GRMZM2G029566_P01	TRUE	TRUE	sFVEVPAGSHFPIQNLPFGVFR	95%	n+304 (+304)	36.36	25.40
5730	seq=translation; coord=5:33171053..33173977:-1; parent_transcript=GRMZM2G042136_T01; parent_gene=GRMZM2G042136	GRMZM2G042136_P01,GRMZM2G042136_P02	TRUE	TRUE	eAPEEEAAAAVGR	95%	n+304 (+304)	32.60	25.00
5731	seq=translation; coord=9:31907012..31908736:-1; parent_transcript=GRMZM2G105364_T01; parent_gene=GRMZM2G105364	GRMZM2G105364_P01	TRUE	TRUE	acLTLcNk	95%	n+304 (+304), Carbamidomethyl (+57), Carbamidomethyl (+57), K+304 (+304)	46.90	25.00
5732	seq=translation; coord=2:186818557..186819984:1; parent_transcript=GRMZM2G323504_T01; parent_gene=GRMZM2G323504	GRMZM2G323504_P01	TRUE	TRUE	eLALVAEFVR	95%	n+304 (+304)	36.53	25.00
5733	seq=translation; coord=7:18139415..18147659:-1; parent_transcript=GRMZM2G122810_T01; parent_gene=GRMZM2G122810	GRMZM2G122810_P01,GRMZM2G122810_P02, GRMZM2G122810_P03,GRMZM2G122810_P05	TRUE	TRUE	eIFTPMYFFLVR	87%	n+304 (+304)	25.07	25.00
5734	seq=translation; coord=6:124736591..124745000:-1; parent_transcript=GRMZM2G148709_T01; parent_gene=GRMZM2G148709	GRMZM2G148709_P01,GRMZM2G148709_P02, GRMZM2G148709_P03,GRMZM2G148709_P04	TRUE	TRUE	gESFYNPYIPQVLEELSSk	95%	n+304 (+304), K+304 (+304)	35.79	25.00
5735	seq=translation; coord=6:124736591..124745000:-1; parent_transcript=GRMZM2G148709_T01; parent_gene=GRMZM2G148709	GRMZM2G148709_P01,GRMZM2G148709_P02, GRMZM2G148709_P03,GRMZM2G148709_P04	TRUE	TRUE	lVELLDEAk	95%	n+304 (+304), K+304 (+304)	36.58	26.13

5736	seq=translation; coord=6:124736591..124745000:-1; parent_transcript=GRMZM2G148709_T01; parent_gene=GRMZM2G148709	GRMZM2G148709_P01,GRMZM2G148709_P02, GRMZM2G148709_P03,GRMZM2G148709_P04	TRUE	TRUE	mFEFSNVDVLR	95%	n+304 (+304)	35.17	25.00
5737	seq=translation; coord=6:124736591..124745000:-1; parent_transcript=GRMZM2G148709_T01; parent_gene=GRMZM2G148709	GRMZM2G148709_P01,GRMZM2G148709_P02, GRMZM2G148709_P03,GRMZM2G148709_P04	TRUE	TRUE	tSHVGFGLVLGSDGk	95%	n+304 (+304), K+304 (+304)	31.91	25.58
5738	seq=translation; coord=7:160059330..160068443:-1; parent_transcript=GRMZM5G887631_T01; parent_gene=GRMZM5G887631	GRMZM5G887631_P01	TRUE	TRUE	aAVAAMPELLR	93%	n+304 (+304)	29.68	26.15
5739	seq=translation; coord=7:160059330..160068443:-1; parent_transcript=GRMZM5G887631_T01; parent_gene=GRMZM5G887631	GRMZM5G887631_P01	TRUE	TRUE	gSFLPFFEELSMyITPMLGk	95%	n+304 (+304), iTRAQ8plex (+304), K+304 (+304)	26.43	25.04
5740	seq=translation; coord=7:160059330..160068443:-1; parent_transcript=GRMZM5G887631_T01; parent_gene=GRMZM5G887631	GRMZM5G887631_P01	TRUE	TRUE	iAIcIFDDVAEQcR	95%	n+304 (+304), Carbamidomethyl (+57), Carbamidomethyl (+57)	30.45	25.00
5741	seq=translation; coord=7:160059330..160068443:-1; parent_transcript=GRMZM5G887631_T01; parent_gene=GRMZM5G887631	GRMZM5G887631_P01	TRUE	TRUE	lPDNIMAYDNAVSALGk	95%	n+304 (+304), K+304 (+304)	54.71	25.38
5742	seq=translation; coord=9:87461648..87475465:-1; parent_transcript=GRMZM2G047564_T03; parent_gene=GRMZM2G047564	GRMZM2G047564_P03,GRMZM2G047564_P04	TRUE	TRUE	aLLDEWETNPSVk	95%	n+304 (+304), K+304 (+304)	30.68	25.20
5743	seq=translation; coord=9:87461648..87475465:-1; parent_transcript=GRMZM2G047564_T03; parent_gene=GRMZM2G047564	GRMZM2G047564_P03,GRMZM2G047564_P04	TRUE	TRUE	gAPFSLcLTQk	89%	n+304 (+304), Carbamidomethyl (+57), K+304 (+304)	25.98	25.89
5744	seq=translation; coord=9:87461648..87475465:-1; parent_transcript=GRMZM2G047564_T03; parent_gene=GRMZM2G047564	GRMZM2G047564_P03,GRMZM2G047564_P04	TRUE	TRUE	lLPYIISSFSPDk	95%	n+304 (+304), K+304 (+304)	36.14	25.00
5745	seq=translation; coord=5:211206297..211209456:-1; parent_transcript=GRMZM2G151440_T01; parent_gene=GRMZM2G151440	GRMZM2G151440_P01,GRMZM2G151440_P02	TRUE	TRUE	iGSVALADLLk	95%	n+304 (+304), K+304 (+304)	54.97	25.00
5746	seq=translation; coord=5:211206297..211209456:-1; parent_transcript=GRMZM2G151440_T01; parent_gene=GRMZM2G151440	GRMZM2G151440_P01,GRMZM2G151440_P02	TRUE	TRUE	wNLPNLPEGTEVWIk	95%	n+304 (+304), K+304 (+304)	40.76	25.00
5747	seq=translation; coord=4:230556470..230569433:1; parent_transcript=GRMZM2G038195_T01; parent_gene=GRMZM2G038195	GRMZM2G038195_P01,GRMZM2G038195_P02, GRMZM2G119546_P01,GRMZM2G119546_P02	TRUE	TRUE	aILMEEWNVQPVR	95%	n+304 (+304)	50.97	25.84
5748	seq=translation; coord=4:230556470..230569433:1; parent_transcript=GRMZM2G038195_T01; parent_gene=GRMZM2G038195	GRMZM2G038195_P01,GRMZM2G038195_P02, GRMZM2G119546_P01,GRMZM2G119546_P02	TRUE	TRUE	iQEVPHEGPMcDLLWSDPDDR	95%	n+304 (+304), Carbamidomethyl (+57)	62.21	25.00
5749	seq=translation; coord=6:133205650..133225934:-1; parent_transcript=GRMZM2G076006_T03; parent_gene=GRMZM2G076006	GRMZM2G076006_P03	TRUE	TRUE	lLHVVYR	90%	n+304 (+304)	25.77	25.44
5750	seq=translation; coord=6:133205650..133225934:-1; parent_transcript=GRMZM2G076006_T03; parent_gene=GRMZM2G076006	GRMZM2G076006_P03	TRUE	TRUE	tVFVDNIDFLk	95%	n+304 (+304), K+304 (+304)	51.33	26.40
5751	seq=translation; coord=6:81686909..81693610:1; parent_transcript=GRMZM2G103266_T01; parent_gene=GRMZM2G103266	GRMZM2G103266_P01,GRMZM2G103266_P07, GRMZM2G103266_P09	TRUE	TRUE	gFIYVIAHIR	95%	n+304 (+304)	45.27	25.00
5752	seq=translation; coord=6:81686909..81693610:1; parent_transcript=GRMZM2G103266_T01; parent_gene=GRMZM2G103266	GRMZM2G103266_P01,GRMZM2G103266_P07, GRMZM2G103266_P09	TRUE	TRUE	sDPDVLAHLR	95%	n+304 (+304)	63.98	25.00
5753	seq=translation; coord=9:59939835..59941693:-1; parent_transcript=GRMZM2G471357_T01; parent_gene=GRMZM2G471357	GRMZM2G471357_P01,GRMZM2G471357_P03	TRUE	TRUE	gFEVIDTIk	95%	n+304 (+304), K+304 (+304)	45.72	25.04

5754	seq=translation; coord=9:59939835..59941693:-1; parent_transcript=GRMZM2G471357_T01; parent_gene=GRMZM2G471357	GRMZM2G471357_P01,GRMZM2G471357_P03	TRUE	TRUE	lFFHDcFVQGcDGSILLDAGGEk	95%	n+304 (+304), Carbamidomethyl (+57), Carbamidomethyl (+57), K+304 (+304)	27.65	25.00
5755	seq=translation; coord=9:59939835..59941693:-1; parent_transcript=GRMZM2G471357_T01; parent_gene=GRMZM2G471357	GRMZM2G471357_P01,GRMZM2G471357_P03	TRUE	TRUE	sGGDGNLAPIDVQTPVR	95%	n+304 (+304)	77.22	25.53
5756	seq=translation; coord=7:168913721..168922316:-1; parent_transcript=GRMZM2G026991_T01; parent_gene=GRMZM2G026991	GRMZM2G026991_P01	TRUE	TRUE	gAWTELSPLDVMQMLGR	95%	n+304 (+304)	41.33	25.00
5757	seq=translation; coord=7:168913721..168922316:-1; parent_transcript=GRMZM2G026991_T01; parent_gene=GRMZM2G026991	GRMZM2G026991_P01	TRUE	TRUE	lVMLLDYEk	90%	n+304 (+304), K+304 (+304)	25.48	25.00
5758	seq=translation; coord=2:179742429..179748543:1; parent_transcript=GRMZM2G105772_T01; parent_gene=GRMZM2G105772	GRMZM2G105772_P01,GRMZM2G105772_P02	TRUE	TRUE	aLVLEVFFAcNk	95%	n+304 (+304), Carbamidomethyl (+57), K+304 (+304)	43.95	25.00
5759	seq=translation; coord=2:179742429..179748543:1; parent_transcript=GRMZM2G105772_T01; parent_gene=GRMZM2G105772	GRMZM2G105772_P01,GRMZM2G105772_P02	TRUE	TRUE	gSSFQIEVEPTDk	95%	n+304 (+304), K+304 (+304)	50.28	25.68
5760	seq=translation; coord=3:225222066..225226693:-1; parent_transcript=GRMZM2G049866_T01; parent_gene=GRMZM2G049866	GRMZM2G049866_P01,GRMZM2G049866_P02	TRUE	TRUE	iFVGNVPADMASER	95%	n+304 (+304)	44.85	25.00
5761	seq=translation; coord=3:7599914..7604652:-1; parent_transcript=GRMZM2G146644_T01; parent_gene=GRMZM2G146644	GRMZM2G146644_P01	TRUE	TRUE	vPHPWLNMFVPR	95%	n+304 (+304)	41.69	25.19
5762	seq=translation; coord=3:7599914..7604652:-1; parent_transcript=GRMZM2G146644_T01; parent_gene=GRMZM2G146644	GRMZM2G146644_P01	TRUE	TRUE	yVDAGGEQVWIDVLR	95%	n+304 (+304)	32.81	25.00
5763	seq=translation; coord=5:194043178..194045076:1; parent_transcript=GRMZM2G013821_T01; parent_gene=GRMZM2G013821	GRMZM2G013821_P01,GRMZM2G013821_P02	TRUE	TRUE	aMSDQEk	95%	n+304 (+304), K+304 (+304)	30.85	25.30
5764	seq=translation; coord=5:194043178..194045076:1; parent_transcript=GRMZM2G013821_T01; parent_gene=GRMZM2G013821	GRMZM2G013821_P01,GRMZM2G013821_P02	TRUE	TRUE	qEYQALHPGNk	93%	n+304 (+304), K+304 (+304)	27.93	26.11
5765	seq=translation; coord=1:292988864..292997657:1; parent_transcript=GRMZM2G155580_T01; parent_gene=GRMZM2G155580	GRMZM2G155580_P01,GRMZM2G155580_P02, GRMZM2G155580_P03	TRUE	TRUE	fQSIDTETFLEFLk	95%	n+304 (+304), K+304 (+304)	40.01	26.37
5766	seq=translation; coord=1:292988864..292997657:1; parent_transcript=GRMZM2G155580_T01; parent_gene=GRMZM2G155580	GRMZM2G155580_P01,GRMZM2G155580_P02, GRMZM2G155580_P03	TRUE	TRUE	mVFLTPTVIk	95%	n+304 (+304), K+304 (+304)	32.03	25.00
5767	seq=translation; coord=1:292988864..292997657:1; parent_transcript=GRMZM2G155580_T01; parent_gene=GRMZM2G155580	GRMZM2G155580_P01,GRMZM2G155580_P02, GRMZM2G155580_P03	TRUE	TRUE	vYAEGGDTVLDEAAR	95%	n+304 (+304)	85.87	25.00
5768	seq=translation; coord=6:140312649..140313648:-1; parent_transcript=GRMZM2G162659_T01; parent_gene=GRMZM2G162659	GRMZM2G162659_P01	TRUE	TRUE	gGLSTMQESGGER	95%	n+304 (+304)	79.47	25.00
5769	seq=translation; coord=6:140312649..140313648:-1; parent_transcript=GRMZM2G162659_T01; parent_gene=GRMZM2G162659	GRMZM2G162659_P01	TRUE	TRUE	mAEEGQTVVPGGTGGk	93%	n+304 (+304), K+304 (+304)	26.93	25.48
5770	seq=translation; coord=6:140312649..140313648:-1; parent_transcript=GRMZM2G162659_T01; parent_gene=GRMZM2G162659	GRMZM2G162659_P01	TRUE	TRUE	tLEAQEHLAEGR	95%	n+304 (+304)	42.40	25.00

5771	seq=translation; coord=5:157517807..157521679:-1; parent_transcript=GRMZM2G057642_T02; parent_gene=GRMZM2G057642	GRMZM2G057642_P02,GRMZM2G057642_P03	TRUE	TRUE	dAFGYYGTIVSVk	95%	n+304 (+304), K+304 (+304)	54.94	25.81
5772	seq=translation; coord=5:157517807..157521679:-1; parent_transcript=GRMZM2G057642_T02; parent_gene=GRMZM2G057642	GRMZM2G057642_P02,GRMZM2G057642_P03	TRUE	TRUE	lEDELVAAR	94%	n+304 (+304)	30.40	25.11
5773	seq=translation; coord=3:1481217..1482997:-1; parent_transcript=GRMZM2G087103_T02; parent_gene=GRMZM2G087103	GRMZM2G087103_P02,GRMZM2G087103_P03, GRMZM2G087103_P04	TRUE	TRUE	gHGVFDTAMILDGALYELDAHLDR	95%	n+304 (+304)	85.25	25.00
5774	seq=translation; coord=3:1481217..1482997:-1; parent_transcript=GRMZM2G087103_T02; parent_gene=GRMZM2G087103	GRMZM2G087103_P02,GRMZM2G087103_P03, GRMZM2G087103_P04	TRUE	TRUE	lMPALSDLLWEDMk	95%	n+304 (+304), K+304 (+304)	52.72	25.97
5775	seq=translation; coord=1:74628405..74631787:1; parent_transcript=GRMZM2G130121_T01; parent_gene=GRMZM2G130121	GRMZM2G130121_P01	TRUE	TRUE	aHSDVFNVFLQILDDGR	95%	n+304 (+304)	57.29	25.42
5776	seq=translation; coord=1:74628405..74631787:1; parent_transcript=GRMZM2G130121_T01; parent_gene=GRMZM2G130121	GRMZM2G130121_P01	TRUE	TRUE	fQQVFVDQPSVEDTISILR	95%	n+304 (+304)	45.19	25.44
5777	seq=translation; coord=9:150085751..150089143:1; parent_transcript=GRMZM2G100120_T01; parent_gene=GRMZM2G100120	GRMZM2G100120_P01,GRMZM2G100120_P02, GRMZM2G100120_P03,GRMZM2G100120_P04, GRMZM2G100120_P06,GRMZM2G100120_P07	TRUE	TRUE	gGQSQVIVLPR	95%	n+304 (+304)	38.43	26.73
5778	seq=translation; coord=9:150085751..150089143:1; parent_transcript=GRMZM2G100120_T01; parent_gene=GRMZM2G100120	GRMZM2G100120_P01,GRMZM2G100120_P02, GRMZM2G100120_P03,GRMZM2G100120_P04, GRMZM2G100120_P06,GRMZM2G100120_P07	TRUE	TRUE	vLIVLSHYLETAR	94%	n+304 (+304)	27.58	25.00
5779	seq=translation; coord=8:157386210..157395665:-1; parent_transcript=GRMZM2G133926_T01; parent_gene=GRMZM2G133926	GRMZM2G133926_P01	TRUE	TRUE	aLLYMDGGQIDGNVVk	95%	n+304 (+304), K+304 (+304)	56.03	25.66
5780	seq=translation; coord=8:157386210..157395665:-1; parent_transcript=GRMZM2G133926_T01; parent_gene=GRMZM2G133926	GRMZM2G133926_P01	TRUE	TRUE	gYGYIEFk	93%	n+304 (+304), K+304 (+304)	27.67	25.83
5781	seq=translation; coord=3:39476674..39479631:1; parent_transcript=GRMZM2G132465_T01; parent_gene=GRMZM2G132465	GRMZM2G132465_P01,GRMZM2G132465_P02, GRMZM2G132465_P03,GRMZM2G132465_P04, GRMZM2G132465_P05	TRUE	TRUE	aAGLSEVLAPVEATMEk	92%	n+304 (+304), K+304 (+304)	26.97	25.81
5782	seq=translation; coord=3:39476674..39479631:1; parent_transcript=GRMZM2G132465_T01; parent_gene=GRMZM2G132465	GRMZM2G132465_P01,GRMZM2G132465_P02, GRMZM2G132465_P03,GRMZM2G132465_P04, GRMZM2G132465_P05	TRUE	TRUE	dAAIAHGDVLTTIR	95%	n+304 (+304)	47.51	26.40
5783	seq=translation; coord=3:39476674..39479631:1; parent_transcript=GRMZM2G132465_T01; parent_gene=GRMZM2G132465	GRMZM2G132465_P01,GRMZM2G132465_P02, GRMZM2G132465_P03,GRMZM2G132465_P04, GRMZM2G132465_P05	TRUE	TRUE	dQLVELLR	87%	n+304 (+304)	25.36	25.29
5784	seq=translation; coord=4:53185415..53193846:1; parent_transcript=GRMZM2G090241_T01; parent_gene=GRMZM2G090241	GRMZM2G090241_P01	TRUE	TRUE	lYDMIDVLR	93%	n+304 (+304)	30.06	25.00
5785	seq=translation; coord=3:182713971..182717776:-1; parent_transcript=GRMZM2G504401_T01; parent_gene=GRMZM2G504401	GRMZM2G504401_P01	TRUE	TRUE	aVEATPESFASFGQVIGASPDGDEFGPHDAQLDLSR	95%	n+304 (+304)	45.61	25.00
5786	seq=translation; coord=3:182713971..182717776:-1; parent_transcript=GRMZM2G504401_T01; parent_gene=GRMZM2G504401	GRMZM2G504401_P01	TRUE	TRUE	gTWHAGPLFk	94%	n+304 (+304), K+304 (+304)	28.57	25.00
5787	seq=translation; coord=7:165748301..165751201:-1; parent_transcript=GRMZM2G053999_T01; parent_gene=GRMZM2G053999	GRMZM2G053999_P01	TRUE	TRUE	aLEEAFNSLEGITcNk	95%	n+304 (+304), Carbamidomethyl (+57), K+304 (+304)	57.19	25.00

5788	seq=translation; coord=7:165748301..165751201:-1; parent_transcript=GRMZM2G053999_T01; parent_gene=GRMZM2G053999	GRMZM2G053999_P01	TRUE	TRUE	aLVVINPGNPTGQVLVEENQk	95%	n+304 (+304), K+304 (+304)	31.80	25.00
5789	seq=translation; coord=7:165748301..165751201:-1; parent_transcript=GRMZM2G053999_T01; parent_gene=GRMZM2G053999	GRMZM2G053999_P01	TRUE	TRUE	lLEATGIVVVPGSGFGQVPGTWHFR	95%	n+304 (+304)	75.44	25.66
5790	seq=translation; coord=1:273298297..273299437:-1; parent_transcript=GRMZM2G481194_T01; parent_gene=GRMZM2G481194	GRMZM2G481194_P01	TRUE	TRUE	aAVGVAPLR	88%	n+304 (+304)	26.07	25.00
5791	seq=translation; coord=1:273298297..273299437:-1; parent_transcript=GRMZM2G481194_T01; parent_gene=GRMZM2G481194	GRMZM2G481194_P01	TRUE	TRUE	aRPAEVVALWVAEGR	95%	n+304 (+304)	53.60	26.50
5792	seq=translation; coord=1:273298297..273299437:-1; parent_transcript=GRMZM2G481194_T01; parent_gene=GRMZM2G481194	GRMZM2G481194_P01	TRUE	TRUE	qcGTYTQVVWR	95%	n+304 (+304), Carbamidomethyl (+57)	51.10	25.00
5793	seq=translation; coord=4:162677644..162687691:1; parent_transcript=GRMZM2G020775_T01; parent_gene=GRMZM2G020775	GRMZM2G020775_P01	TRUE	TRUE	gLTHFDVDNFVEEVLVk	95%	n+304 (+304), K+304 (+304)	41.85	25.81
5794	seq=translation; coord=4:162677644..162687691:1; parent_transcript=GRMZM2G020775_T01; parent_gene=GRMZM2G020775	GRMZM2G020775_P01	TRUE	TRUE	hIGQGEVVDHLWR	95%	n+304 (+304)	48.91	25.73
5795	seq=translation; coord=1:269035042..269037767:1; parent_transcript=GRMZM2G047055_T01; parent_gene=GRMZM2G047055	GRMZM2G047055_P01,GRMZM2G047055_P02, GRMZM2G047055_P03,GRMZM2G152328_P01, GRMZM2G152328_P02	TRUE	TRUE	dLTDYLMk	95%	n+304 (+304), K+304 (+304)	42.19	25.20
5796	seq=translation; coord=1:269035042..269037767:1; parent_transcript=GRMZM2G047055_T01; parent_gene=GRMZM2G047055	GRMZM2G047055_P01,GRMZM2G047055_P02, GRMZM2G047055_P03,GRMZM2G152328_P01, GRMZM2G152328_P02	TRUE	TRUE	lAYIALDYDQEMETAk	95%	n+304 (+304), K+304 (+304)	57.64	25.00
5797	seq=translation; coord=3:186233588..186237469:1; parent_transcript=GRMZM2G133213_T01; parent_gene=GRMZM2G133213	GRMZM2G133213_P01	TRUE	TRUE	aFGMLAGGSGITPMFQVAR	95%	n+304 (+304)	32.79	25.16
5798	seq=translation; coord=3:186233588..186237469:1; parent_transcript=GRMZM2G133213_T01; parent_gene=GRMZM2G133213	GRMZM2G133213_P01	TRUE	TRUE	eMIQSHcPAPAEDIQILR	95%	n+304 (+304), Carbamidomethyl (+57)	35.80	25.00
5799	seq=translation; coord=4:197674970..197676500:1; parent_transcript=GRMZM2G301908_T01; parent_gene=GRMZM2G301908	GRMZM2G301908_P01	TRUE	TRUE	aFASLLDk	95%	n+304 (+304), K+304 (+304)	34.06	26.37
5800	seq=translation; coord=4:197674970..197676500:1; parent_transcript=GRMZM2G301908_T01; parent_gene=GRMZM2G301908	GRMZM2G301908_P01	TRUE	TRUE	aSLLLFHAVPVYYTLR	95%	n+304 (+304)	36.40	25.00
5801	seq=translation; coord=9:4832748..4841265:1; parent_transcript=GRMZM2G124288_T01; parent_gene=GRMZM2G124288	GRMZM2G124288_P01,GRMZM2G124288_P02	TRUE	TRUE	lFLYDFDLk	95%	n+304 (+304), K+304 (+304)	30.71	26.86
5802	seq=translation; coord=9:4832748..4841265:1; parent_transcript=GRMZM2G124288_T01; parent_gene=GRMZM2G124288	GRMZM2G124288_P01,GRMZM2G124288_P02	TRUE	TRUE	lPLLPIEEVR	95%	n+304 (+304)	36.33	25.69
5803	seq=translation; coord=9:4832748..4841265:1; parent_transcript=GRMZM2G124288_T01; parent_gene=GRMZM2G124288	GRMZM2G124288_P01,GRMZM2G124288_P02	TRUE	TRUE	sTTFVEASPVVQTQk	95%	n+304 (+304), K+304 (+304)	33.86	25.84
5804	seq=translation; coord=7:52251311..52252353:1; parent_transcript=GRMZM2G027198_T01; parent_gene=GRMZM2G027198	GRMZM2G027198_P01	TRUE	TRUE	fHPGDQTVFk	95%	n+304 (+304), K+304 (+304)	32.51	25.97
5805	seq=translation; coord=7:52251311..52252353:1; parent_transcript=GRMZM2G027198_T01; parent_gene=GRMZM2G027198	GRMZM2G027198_P01	TRUE	TRUE	tNYGTWVSSk	88%	n+304 (+304), K+304 (+304)	26.99	25.99

5806	seq=translation; coord=7:52251311..52252353:1; parent_transcript=GRMZM2G027198_T01; parent_gene=GRMZM2G027198	GRMZM2G027198_P01	TRUE	TRUE	yFIcGFPGHcDAGMk	95%	n+304 (+304), Carbamidomethyl (+57), Carbamidomethyl (+57), K+304 (+304)	35.47	25.00
5807	seq=translation; coord=3:2218397..2221027:-1; parent_transcript=GRMZM2G159388_T01; parent_gene=GRMZM2G159388	GRMZM2G159388_P01	TRUE	TRUE	qPSFSDAAATAAAEAk	95%	n+304 (+304), K+304 (+304)	31.09	25.47
5808	seq=translation; coord=1:235703411..235705133:-1; parent_transcript=GRMZM2G118433_T01; parent_gene=GRMZM2G118433	GRMZM2G118433_P01	TRUE	TRUE	lMEIVSAIASR	89%	n+304 (+304)	26.95	25.00
5809	seq=translation; coord=4:195793438..195797209:-1; parent_transcript=GRMZM2G163709_T01; parent_gene=GRMZM2G163709	GRMZM2G163709_P01,GRMZM2G163709_P02, GRMZM2G163709_P03,GRMZM2G163709_P04	TRUE	TRUE	gLMLDTYDFDNDVWLcHSFQGk	95%	n+304 (+304), Carbamidomethyl (+57), K+304 (+304)	44.00	25.00
5810	seq=translation; coord=4:195793438..195797209:-1; parent_transcript=GRMZM2G163709_T01; parent_gene=GRMZM2G163709	GRMZM2G163709_P01,GRMZM2G163709_P02, GRMZM2G163709_P03,GRMZM2G163709_P04	TRUE	TRUE	lLVFTSk	94%	n+304 (+304), K+304 (+304)	27.34	25.00
5811	seq=translation; coord=4:34337570..34341391:1; parent_transcript=GRMZM2G000481_T01; parent_gene=GRMZM2G000481	GRMZM2G000481_P01,GRMZM2G134756_P01, GRMZM2G134756_P02	TRUE	TRUE	iWIADWFAQLNNk	95%	n+304 (+304), K+304 (+304)	36.11	25.21
5812	seq=translation; coord=6:128560617..128567071:1; parent_transcript=GRMZM2G027723_T01; parent_gene=GRMZM2G027723	GRMZM2G027723_P01,GRMZM2G112336_P01, GRMZM2G112336_P02	TRUE	TRUE	eGNQccPQck	92%	n+304 (+304), Carbamidomethyl (+57), Carbamidomethyl (+57), Carbamidomethyl (+57), K+304 (+304)	25.72	25.00
5813	seq=translation; coord=6:128560617..128567071:1; parent_transcript=GRMZM2G027723_T01; parent_gene=GRMZM2G027723	GRMZM2G027723_P01,GRMZM2G112336_P01, GRMZM2G112336_P02	TRUE	TRUE	gPEWQLQGDDADLSSSAR	95%	n+304 (+304)	87.81	25.00
5814	seq=translation; coord=6:128560617..128567071:1; parent_transcript=GRMZM2G027723_T01; parent_gene=GRMZM2G027723	GRMZM2G027723_P01,GRMZM2G112336_P01, GRMZM2G112336_P02	TRUE	TRUE	vHGDDEEEDVDDLDNEFNYk	95%	n+304 (+304), K+304 (+304)	51.81	25.00
5815	seq=translation; coord=1:240997699..241001751:-1; parent_transcript=GRMZM2G071268_T01; parent_gene=GRMZM2G071268	GRMZM2G071268_P01	TRUE	TRUE	lLQEFVk	91%	n+304 (+304), K+304 (+304)	25.99	25.00
5816	seq=translation; coord=7:14459963..14466085:-1; parent_transcript=GRMZM2G104325_T01; parent_gene=GRMZM2G104325	GRMZM2G104325_P01	TRUE	TRUE	eSPGGPWQFIGLLPLFDPPR	95%	n+304 (+304)	31.53	25.94
5817	seq=translation; coord=7:14459963..14466085:-1; parent_transcript=GRMZM2G104325_T01; parent_gene=GRMZM2G104325	GRMZM2G104325_P01	TRUE	TRUE	lLEGDPLk	87%	n+304 (+304), K+304 (+304)	26.42	25.83
5818	seq=translation; coord=7:14459963..14466085:-1; parent_transcript=GRMZM2G104325_T01; parent_gene=GRMZM2G104325	GRMZM2G104325_P01	TRUE	TRUE	tALTYLDGEGR	95%	n+304 (+304)	40.55	25.00
5819	seq=translation; coord=7:158136732..158153170:1; parent_transcript=GRMZM2G107654_T01; parent_gene=GRMZM2G107654	GRMZM2G107654_P01	TRUE	TRUE	gEILMLNIGSMSTGAR	95%	n+304 (+304)	52.81	25.56
5820	seq=translation; coord=7:158136732..158153170:1; parent_transcript=GRMZM2G107654_T01; parent_gene=GRMZM2G107654	GRMZM2G107654_P01	TRUE	TRUE	iDLIQESAAMNQHEAIQk	95%	n+304 (+304), K+304 (+304)	39.81	25.49
5821	seq=translation; coord=7:158136732..158153170:1; parent_transcript=GRMZM2G107654_T01; parent_gene=GRMZM2G107654	GRMZM2G107654_P01	TRUE	TRUE	lHPLSPEVISR	92%	n+304 (+304)	26.24	25.19
5822	seq=translation; coord=7:158136732..158153170:1; parent_transcript=GRMZM2G107654_T01; parent_gene=GRMZM2G107654	GRMZM2G107654_P01	TRUE	TRUE	lQLTAPVcTSR	88%	n+304 (+304), Carbamidomethyl (+57)	26.47	25.40

5823	seq=translation; coord=8:157237785..157240528:-1; parent_transcript=GRMZM2G446960_T01; parent_gene=GRMZM2G446960	GRMZM2G446960_P01	TRUE	TRUE	sFLNLPSEVVPNTLk	95%	n+304 (+304), K+304 (+304)	52.59	25.00
5824	seq=translation; coord=5:31131578..31135872:-1; parent_transcript=GRMZM2G110201_T01; parent_gene=GRMZM2G110201	GRMZM2G110201_P01	TRUE	TRUE	aGLYDIGIAAGLESMTVNk	95%	n+304 (+304), K+304 (+304)	40.93	26.14
5825	seq=translation; coord=5:31131578..31135872:-1; parent_transcript=GRMZM2G110201_T01; parent_gene=GRMZM2G110201	GRMZM2G110201_P01	TRUE	TRUE	dTFAEDLLVPVFk	95%	n+304 (+304), K+304 (+304)	45.43	25.38
5826	seq=translation; coord=5:31131578..31135872:-1; parent_transcript=GRMZM2G110201_T01; parent_gene=GRMZM2G110201	GRMZM2G110201_P01	TRUE	TRUE	lDPSEVGDIVVGTVLAPGSQR	95%	n+304 (+304)	37.92	25.29
5827	seq=translation; coord=5:31131578..31135872:-1; parent_transcript=GRMZM2G110201_T01; parent_gene=GRMZM2G110201	GRMZM2G110201_P01	TRUE	TRUE	rPAFADDVVIVAAYR	93%	n+304 (+304)	28.25	25.73
5828	seq=translation; coord=6:2438893..2441433:1; parent_transcript=GRMZM2G024303_T01; parent_gene=GRMZM2G024303	GRMZM2G024303_P01	TRUE	TRUE	aFGASQVIAVDVLDEk	95%	n+304 (+304), K+304 (+304)	55.67	25.69
5829	seq=translation; coord=6:2438893..2441433:1; parent_transcript=GRMZM2G024303_T01; parent_gene=GRMZM2G024303	GRMZM2G024303_P01	TRUE	TRUE	gVDVAIEALGk	95%	n+304 (+304), K+304 (+304)	33.83	26.63
5830	seq=translation; coord=9:148077545..148089511:-1; parent_transcript=GRMZM2G030915_T01; parent_gene=GRMZM2G030915	GRMZM2G030915_P01,GRMZM2G030915_P02, GRMZM2G030915_P03,GRMZM2G030915_P04, GRMZM2G030915_P08	TRUE	TRUE	lERPVEGDEAVQEVAAAE	95%	n+304 (+304)	43.97	25.00
5831	seq=translation; coord=6:4593924..4606490:1; parent_transcript=GRMZM2G032315_T01; parent_gene=GRMZM2G032315	GRMZM2G032315_P01,GRMZM2G032315_P02, GRMZM2G157443_P01,GRMZM2G157443_P02, GRMZM2G157443_P05	TRUE	TRUE	vESYWPALFAk	95%	n+304 (+304), K+304 (+304)	48.50	26.17
5832	seq=translation; coord=6:97088034..97088919:1; parent_transcript=GRMZM2G045371_T01; parent_gene=GRMZM2G045371	GRMZM2G045371_P01,GRMZM2G045371_P02, GRMZM2G092975_P01,GRMZM2G117544_P01, GRMZM2G117544_P02	TRUE	TRUE	lYIGLSGLATDAQTLYQR	95%	n+304 (+304)	80.69	26.57
5833	seq=translation; coord=9:12571363..12580129:1; parent_transcript=GRMZM2G128056_T01; parent_gene=GRMZM2G128056	GRMZM2G128056_P01,GRMZM2G128056_P02	TRUE	TRUE	tATDSLAFLLk	95%	n+304 (+304), K+304 (+304)	35.12	25.22
5834	seq=translation; coord=4:71986473..72026506:-1; parent_transcript=GRMZM2G448456_T01; parent_gene=GRMZM2G448456	GRMZM2G448456_P01	TRUE	TRUE	iVVLPTLAIVk	95%	n+304 (+304), K+304 (+304)	34.93	25.00
5835	seq=translation; coord=1:227059090..227060674:-1; parent_transcript=GRMZM5G864178_T01; parent_gene=GRMZM5G864178	GRMZM5G864178_P01	TRUE	TRUE	lILPLVEAFESIk	90%	n+304 (+304), K+304 (+304)	25.55	25.00
5836	seq=translation; coord=10:23388783..23392102:-1; parent_transcript=GRMZM5G868647_T01; parent_gene=GRMZM5G868647	GRMZM5G868647_P01	TRUE	TRUE	eAPPAFDDLLGGFGk	95%	n+304 (+304), K+304 (+304)	48.44	26.00
5837	seq=translation; coord=5:209568609..209572480:-1; parent_transcript=GRMZM2G012178_T01; parent_gene=GRMZM2G012178	GRMZM2G012178_P01	TRUE	TRUE	fAEIGVAEVLVNALR	95%	n+304 (+304)	29.00	25.00
5838	seq=translation; coord=5:162834761..162837541:1; parent_transcript=GRMZM2G084279_T01; parent_gene=GRMZM2G084279	GRMZM2G084279_P01,GRMZM2G084279_P02	TRUE	TRUE	gYVQDAFFR	95%	n+304 (+304)	44.09	25.00
5839	seq=translation; coord=5:98993016..98997371:-1; parent_transcript=GRMZM5G844562_T01; parent_gene=GRMZM5G844562	GRMZM5G844562_P01,GRMZM5G844562_P03	TRUE	TRUE	gGILGLGGVGHMGVk	95%	n+304 (+304), K+304 (+304)	28.46	25.00
5840	seq=translation; coord=5:98993016..98997371:-1; parent_transcript=GRMZM5G844562_T01; parent_gene=GRMZM5G844562	GRMZM5G844562_P01,GRMZM5G844562_P03	TRUE	TRUE	hFGLTTPGLR	95%	n+304 (+304)	42.18	26.25

5841	seq=translation; coord=5:98993016..98997371:-1; parent_transcript=GRMZM5G844562_T01; parent_gene=GRMZM5G844562	GRMZM5G844562_P01,GRMZM5G844562_P03	TRUE	TRUE	mGYVNEALER	92%	n+304 (+304)	28.89	25.00
5842	seq=translation; coord=5:98993016..98997371:-1; parent_transcript=GRMZM5G844562_T01; parent_gene=GRMZM5G844562	GRMZM5G844562_P01,GRMZM5G844562_P03	TRUE	TRUE	yPMVPGHEVVGEVVEVGPEVAk	95%	n+304 (+304), K+304 (+304)	35.80	25.00
5843	seq=translation; coord=2:23469563..23473424:1; parent_transcript=GRMZM2G177923_T01; parent_gene=GRMZM2G177923	GRMZM2G177923_P01	TRUE	TRUE	aLFNLFSVQLR	92%	n+304 (+304)	27.91	25.95
5844	seq=translation; coord=2:23469563..23473424:1; parent_transcript=GRMZM2G177923_T01; parent_gene=GRMZM2G177923	GRMZM2G177923_P01	TRUE	TRUE	aLVSLLTLDEk	95%	n+304 (+304), K+304 (+304)	45.78	25.00
5845	seq=translation; coord=2:23469563..23473424:1; parent_transcript=GRMZM2G177923_T01; parent_gene=GRMZM2G177923	GRMZM2G177923_P01	TRUE	TRUE	eLAAEAVR	95%	n+304 (+304)	33.13	26.55
5846	seq=translation; coord=2:23469563..23473424:1; parent_transcript=GRMZM2G177923_T01; parent_gene=GRMZM2G177923	GRMZM2G177923_P01	TRUE	TRUE	gQETSGEDPAVAAAYSLEYVk	95%	n+304 (+304), K+304 (+304)	28.84	25.00
5847	seq=translation; coord=10:123811073..123815007:1; parent_transcript=GRMZM2G064159_T01; parent_gene=GRMZM2G064159	GRMZM2G064159_P01	TRUE	TRUE	aFPDHEVVMVEGGR	95%	n+304 (+304)	38.82	25.00
5848	seq=translation; coord=10:123811073..123815007:1; parent_transcript=GRMZM2G064159_T01; parent_gene=GRMZM2G064159	GRMZM2G064159_P01	TRUE	TRUE	eNAGPAQEVFAR	95%	n+304 (+304)	50.99	25.00
5849	seq=translation; coord=10:123811073..123815007:1; parent_transcript=GRMZM2G064159_T01; parent_gene=GRMZM2G064159	GRMZM2G064159_P01	TRUE	TRUE	iVEIER	90%	n+304 (+304)	27.42	25.00
5850	seq=translation; coord=3:168695539..168839001:1; parent_transcript=GRMZM2G156861_T03; parent_gene=GRMZM2G156861	GRMZM2G156861_P03	TRUE	TRUE	eILAGVNPMLVk	95%	n+304 (+304), K+304 (+304)	34.45	25.00
5851	seq=translation; coord=3:168695539..168839001:1; parent_transcript=GRMZM2G156861_T03; parent_gene=GRMZM2G156861	GRMZM2G156861_P03	TRUE	TRUE	gDGTLVPVAIELSLPELR	95%	n+304 (+304)	35.21	25.45
5852	seq=translation; coord=3:168695539..168839001:1; parent_transcript=GRMZM2G156861_T03; parent_gene=GRMZM2G156861	GRMZM2G156861_P03	TRUE	TRUE	lYILDHHDNFMPFLVR	95%	n+304 (+304)	33.36	25.05
5853	seq=translation; coord=6:143138266..143147623:1; parent_transcript=GRMZM2G305851_T01; parent_gene=GRMZM2G305851	GRMZM2G305851_P01	TRUE	TRUE	eALFVLAQLGR	95%	n+304 (+304)	33.36	25.51
5854	seq=translation; coord=6:143138266..143147623:1; parent_transcript=GRMZM2G305851_T01; parent_gene=GRMZM2G305851	GRMZM2G305851_P01	TRUE	TRUE	lFAVGTSASLIGTGVTNALIk	95%	n+304 (+304), K+304 (+304)	53.28	25.00
5855	seq=translation; coord=6:143138266..143147623:1; parent_transcript=GRMZM2G305851_T01; parent_gene=GRMZM2G305851	GRMZM2G305851_P01	TRUE	TRUE	lLADDLFLTk	95%	n+304 (+304), K+304 (+304)	38.01	25.00
5856	seq=translation; coord=4:18573473..18575395:-1; parent_transcript=GRMZM2G440208_T01; parent_gene=GRMZM2G440208	GRMZM2G440208_P01	TRUE	TRUE	gWNLNLAELAR	95%	n+304 (+304)	35.19	26.10
5857	seq=translation; coord=4:18573473..18575395:-1; parent_transcript=GRMZM2G440208_T01; parent_gene=GRMZM2G440208	GRMZM2G440208_P01	TRUE	TRUE	iGLAGLATMGQNLALNIAEk	95%	n+304 (+304), K+304 (+304)	67.92	25.00
5858	seq=translation; coord=4:18573473..18575395:-1; parent_transcript=GRMZM2G440208_T01; parent_gene=GRMZM2G440208	GRMZM2G440208_P01	TRUE	TRUE	wTVQQAAELAVAAPTIAASLDGR	95%	n+304 (+304)	38.15	25.38

5859	seq=translation; coord=7:143292623..143296263:1; parent_transcript=GRMZM2G109405_T01; parent_gene=GRMZM2G109405	GRMZM2G109405_P01,GRMZM2G109405_P02, GRMZM2G109405_P03	TRUE	TRUE	aAFVGHDHINDFcGk	94%	n+304 (+304), Carbamidomethyl (+57), K+304 (+304)	28.41	25.00
5860	seq=translation; coord=7:143292623..143296263:1; parent_transcript=GRMZM2G109405_T01; parent_gene=GRMZM2G109405	GRMZM2G109405_P01,GRMZM2G109405_P02, GRMZM2G109405_P03	TRUE	TRUE	lPWAAVIGNHDQEGTLSR	95%	n+304 (+304)	62.38	25.68
5861	seq=translation; coord=7:143292623..143296263:1; parent_transcript=GRMZM2G109405_T01; parent_gene=GRMZM2G109405	GRMZM2G109405_P01,GRMZM2G109405_P02, GRMZM2G109405_P03	TRUE	TRUE	sMDAAIAPAIDMk	95%	n+304 (+304), K+304 (+304)	30.89	25.00
5862	seq=translation; coord=9:89703050..89707450:1; parent_transcript=GRMZM2G442551_T01; parent_gene=GRMZM2G442551	GRMZM2G442551_P01,GRMZM2G442551_P02	TRUE	TRUE	dLIAGQEIER	86%	n+304 (+304)	25.37	25.00
5863	seq=translation; coord=9:89703050..89707450:1; parent_transcript=GRMZM2G442551_T01; parent_gene=GRMZM2G442551	GRMZM2G442551_P01,GRMZM2G442551_P02	TRUE	TRUE	fFPDTQALVLGALSk	95%	n+304 (+304), K+304 (+304)	46.61	25.00
5864	seq=translation; coord=9:89703050..89707450:1; parent_transcript=GRMZM2G442551_T01; parent_gene=GRMZM2G442551	GRMZM2G442551_P01,GRMZM2G442551_P02	TRUE	TRUE	iEGPAAWDVLYNFEQR	95%	n+304 (+304)	53.37	25.00
5865	seq=translation; coord=8:173066444..173072416:1; parent_transcript=GRMZM2G424857_T01; parent_gene=GRMZM2G424857	GRMZM2G424857_P01	TRUE	TRUE	fPGLTHPGIVGTAPSAELLNIWNER	95%	n+304 (+304)	32.13	25.21
5866	seq=translation; coord=8:173066444..173072416:1; parent_transcript=GRMZM2G424857_T01; parent_gene=GRMZM2G424857	GRMZM2G424857_P01	TRUE	TRUE	lVVPIDVk	91%	n+304 (+304), K+304 (+304)	25.45	25.00
5867	seq=translation; coord=8:173066444..173072416:1; parent_transcript=GRMZM2G424857_T01; parent_gene=GRMZM2G424857	GRMZM2G424857_P01	TRUE	TRUE	wHPDIPPVADVTEGELFR	95%	n+304 (+304)	57.66	25.00
5868	seq=translation; coord=5:123202556..123206585:1; parent_transcript=GRMZM2G009479_T01; parent_gene=GRMZM2G009479	GRMZM2G009479_P01	TRUE	TRUE	aLDDAGDFFGk	95%	n+304 (+304), K+304 (+304)	30.65	25.00
5869	seq=translation; coord=7:126805106..126806657:1; parent_transcript=GRMZM5G826321_T01; parent_gene=GRMZM5G826321	GRMZM5G826321_P01	TRUE	TRUE	dVVVDSDAGVYVR	95%	n+304 (+304)	34.17	25.00
5870	seq=translation; coord=7:126805106..126806657:1; parent_transcript=GRMZM5G826321_T01; parent_gene=GRMZM5G826321	GRMZM5G826321_P01	TRUE	TRUE	iNPLAAAAAPSLR	94%	n+304 (+304)	31.28	25.26
5871	seq=translation; coord=7:131436732..131438757:1; parent_transcript=GRMZM2G018108_T01; parent_gene=GRMZM2G018108	GRMZM2G018108_P01	TRUE	TRUE	gVPLQVGWGk	95%	n+304 (+304), K+304 (+304)	44.20	25.00
5872	seq=translation; coord=7:131436732..131438757:1; parent_transcript=GRMZM2G018108_T01; parent_gene=GRMZM2G018108	GRMZM2G018108_P01	TRUE	TRUE	tLAVIYDVDNQk	95%	n+304 (+304), K+304 (+304)	31.69	25.98
5873	seq=translation; coord=5:201664982..201667877:1; parent_transcript=GRMZM2G170602_T01; parent_gene=GRMZM2G170602	GRMZM2G170602_P01	TRUE	TRUE	nPSFVGcAEHALGIR	95%	n+304 (+304), Carbamidomethyl (+57)	85.59	25.00
5874	seq=translation; coord=5:201664982..201667877:1; parent_transcript=GRMZM2G170602_T01; parent_gene=GRMZM2G170602	GRMZM2G170602_P01	TRUE	TRUE	qAVGGGWDVAAVAHR	95%	Pyro-cmC (-17), n+304 (+304)	75.65	25.00
5875	seq=translation; coord=5:3285681..3289943:1; parent_transcript=GRMZM2G017532_T01; parent_gene=GRMZM2G017532	GRMZM2G017532_P01	TRUE	TRUE	aAVFALHVk	94%	n+304 (+304), K+304 (+304)	27.44	25.55
5876	seq=translation; coord=5:3285681..3289943:1; parent_transcript=GRMZM2G017532_T01; parent_gene=GRMZM2G017532	GRMZM2G017532_P01	TRUE	TRUE	eELNSWPEQSTR	95%	n+304 (+304)	32.87	25.00

5877	seq=translation; coord=4:180245812..180247211:1; parent_transcript=GRMZM2G448883_T01; parent_gene=GRMZM2G448883	GRMZM2G448883_P01	TRUE	TRUE	aINLVLTAADVAVDGFcASR	95%	n+304 (+304), Carbamidomethyl (+57)	45.03	25.91
5878	seq=translation; coord=4:180245812..180247211:1; parent_transcript=GRMZM2G448883_T01; parent_gene=GRMZM2G448883	GRMZM2G448883_P01	TRUE	TRUE	aVITDFVSSLSAAPR	95%	n+304 (+304)	73.50	25.47
5879	seq=translation; coord=4:238055009..238057832:-1; parent_transcript=AC233922.1_FGT005; parent_gene=AC233922.1_FG005	AC233922.1_FGP005	TRUE	TRUE	mkDVIGIVPGLAVILVGSR	95%	n+304 (+304), K+304 (+304)	51.98	25.00
5880	seq=translation; coord=4:238055009..238057832:-1; parent_transcript=AC233922.1_FGT005; parent_gene=AC233922.1_FG005	AC233922.1_FGP005	TRUE	TRUE	vAGAITPVPGGVGPMTIAMLLSNTLESAR	95%	n+304 (+304)	28.83	25.00
5881	seq=translation; coord=8:17966967..17971201:-1; parent_transcript=GRMZM2G175171_T01; parent_gene=GRMZM2G175171	GRMZM2G175171_P01	TRUE	TRUE	aTTIADIPR	95%	n+304 (+304)	33.44	26.32
5882	seq=translation; coord=8:17966967..17971201:-1; parent_transcript=GRMZM2G175171_T01; parent_gene=GRMZM2G175171	GRMZM2G175171_P01	TRUE	TRUE	lNWLLHFGEPPk	95%	n+304 (+304), K+304 (+304)	36.17	25.00
5883	seq=translation; coord=1:228560968..228564785:1; parent_transcript=GRMZM5G888696_T02; parent_gene=GRMZM5G888696	GRMZM5G888696_P02	TRUE	TRUE	aFVQQAEVILADGk	95%	n+304 (+304), K+304 (+304)	44.61	25.38
5884	seq=translation; coord=1:228560968..228564785:1; parent_transcript=GRMZM5G888696_T02; parent_gene=GRMZM5G888696	GRMZM5G888696_P02	TRUE	TRUE	lENSLVQAIALLR	95%	n+304 (+304)	64.95	25.00
5885	seq=translation; coord=6:161451718..161452634:-1; parent_transcript=GRMZM2G090245_T01; parent_gene=GRMZM2G090245	GRMZM2G090245_P01	TRUE	TRUE	dVTADDFHYGGLATPGTALkPFk	95%	n+304 (+304), K+304 (+304), K+304 (+304)	29.00	25.00
5886	seq=translation; coord=6:161451718..161452634:-1; parent_transcript=GRMZM2G090245_T01; parent_gene=GRMZM2G090245	GRMZM2G090245_P01	TRUE	TRUE	vTNLDELQIVk	95%	n+304 (+304), K+304 (+304)	48.93	25.07
5887	seq=translation; coord=2:43428306..43433991:1; parent_transcript=GRMZM2G031028_T02; parent_gene=GRMZM2G031028	GRMZM2G031028_P02	TRUE	TRUE	gDGSSIFVLFk	94%	n+304 (+304), K+304 (+304)	29.02	25.63
5888	seq=translation; coord=2:43428306..43433991:1; parent_transcript=GRMZM2G031028_T02; parent_gene=GRMZM2G031028	GRMZM2G031028_P02	TRUE	TRUE	vLELITQLETR	95%	n+304 (+304)	31.79	25.00
5889	seq=translation; coord=8:152747393..152749464:1; parent_transcript=GRMZM2G010987_T01; parent_gene=GRMZM2G010987	GRMZM2G010987_P01	TRUE	TRUE	eGGESEATLTGLVDEWk	95%	n+304 (+304), K+304 (+304)	28.90	25.00
5890	seq=translation; coord=9:24694136..24698929:-1; parent_transcript=GRMZM2G048557_T01; parent_gene=GRMZM2G048557	GRMZM2G048557_P01,GRMZM2G048557_P02	TRUE	TRUE	tAQNPLEEFFEVER	95%	n+304 (+304)	42.97	25.00
5891	seq=translation; coord=4:9697965..9698851:1; parent_transcript=GRMZM2G117942_T01; parent_gene=GRMZM2G117942	GRMZM2G117942_P01,GRMZM2G117971_P01	TRUE	TRUE	yGWTAFcGPAGPTGQAAcGQcLLVTNTATGASITVR	95%	n+304 (+304), iTRAQ8plex (+304), Carbamidomethyl (+57), Carbamidomethyl (+57), Carbamidomethyl (+57)	39.59	25.00
5892	seq=translation; coord=4:26293451..26297824:1; parent_transcript=GRMZM2G004997_T02; parent_gene=GRMZM2G004997	GRMZM2G004997_P02	TRUE	TRUE	dQILSGTENVTTGNAR	95%	n+304 (+304)	68.76	25.00
5893	seq=translation; coord=6:155589495..155593823:-1; parent_transcript=GRMZM2G116258_T01; parent_gene=GRMZM2G116258	GRMZM2G116258_P01	TRUE	TRUE	qDGALLVFDEVMTGFR	95%	n+304 (+304)	54.66	25.00

5894	seq=translation; coord=4:195759880..195760460:-1; parent_transcript=GRMZM2G406552_T01; parent_gene=GRMZM2G406552	GRMZM2G406552_P01	TRUE	TRUE	tFTScGIAIPk	95%	n+304 (+304), Carbamidomethyl (+57), K+304 (+304)	44.54	25.97
5895	seq=translation; coord=1:32051838..32054795:1; parent_transcript=GRMZM2G152477_T01; parent_gene=GRMZM2G152477	GRMZM2G152477_P01,GRMZM2G152477_P02, GRMZM2G152477_P03	TRUE	TRUE	gDALAQLSPVLR	95%	n+304 (+304)	53.25	26.16
5896	GRMZM2G057885_P01-R	GRMZM2G057885_P01-R	TRUE	TRUE	qTLEILk	92%	Pyro-cmC (-17), n+304 (+304), K+304 (+304)	28.82	25.00
5897	seq=translation; coord=5:2362323..2364777:-1; parent_transcript=GRMZM2G002825_T01; parent_gene=GRMZM2G002825	GRMZM2G002825_P01	TRUE	TRUE	aTSYEDFTNSLPENDcR	95%	n+304 (+304), Carbamidomethyl (+57)	37.51	25.00
5898	seq=translation; coord=10:116057034..116058081:-1; parent_transcript=GRMZM2G052100_T01; parent_gene=GRMZM2G052100	GRMZM2G052100_P01	TRUE	TRUE	hMEQLGQLGAIAAGAYALHEk	95%	n+304 (+304), K+304 (+304)	66.03	25.00
5899	seq=translation; coord=10:142505503..142509165:-1; parent_transcript=GRMZM2G054537_T01; parent_gene=GRMZM2G054537	GRMZM2G054537_P01	TRUE	TRUE	gLPGVLWVLPDSYLDVPNk	95%	n+304 (+304), K+304 (+304)	34.73	25.00
5900	seq=translation; coord=1:92464668..92465816:1; parent_transcript=GRMZM2G094639_T01; parent_gene=GRMZM2G094639	GRMZM2G094639_P01	TRUE	TRUE	lGVcANVLDLIk	89%	n+304 (+304), Carbamidomethyl (+57), K+304 (+304)	27.07	25.43
5901	seq=translation; coord=1:288165266..288170829:-1; parent_transcript=GRMZM2G015436_T01; parent_gene=GRMZM2G015436	GRMZM2G015436_P01,GRMZM2G015436_P02, GRMZM2G015436_P03,GRMZM2G046191_P01	TRUE	TRUE	gIAQASEGFLYLVSAVGVTGAR	95%	n+304 (+304)	62.31	26.12
5902	seq=translation; coord=6:75632814..75634590:1; parent_transcript=GRMZM5G873767_T01; parent_gene=GRMZM5G873767	GRMZM5G873767_P01	TRUE	TRUE	lVVDFLAQELGLPLLPPSk	95%	n+304 (+304), K+304 (+304)	32.40	25.00
5903	seq=translation; coord=3:206531676..206533642:1; parent_transcript=GRMZM2G050570_T01; parent_gene=GRMZM2G050570	GRMZM2G050570_P01	TRUE	TRUE	aDFGSVMDVLQk	95%	n+304 (+304), K+304 (+304)	34.08	26.21
5904	seq=translation; coord=3:206531676..206533642:1; parent_transcript=GRMZM2G050570_T01; parent_gene=GRMZM2G050570	GRMZM2G050570_P01	TRUE	TRUE	ySLDEVVYR	95%	n+304 (+304)	33.20	25.00
5905	seq=translation; coord=6:115289930..115298874:1; parent_transcript=GRMZM2G157462_T01; parent_gene=GRMZM2G157462	GRMZM2G157462_P01	TRUE	TRUE	aFVPPQHFIR	95%	n+304 (+304)	32.00	27.20
5906	seq=translation; coord=6:115289930..115298874:1; parent_transcript=GRMZM2G157462_T01; parent_gene=GRMZM2G157462	GRMZM2G157462_P01	TRUE	TRUE	eVVAIASNALETFk	95%	n+304 (+304), K+304 (+304)	37.84	26.16
5907	seq=translation; coord=6:115289930..115298874:1; parent_transcript=GRMZM2G157462_T01; parent_gene=GRMZM2G157462	GRMZM2G157462_P01	TRUE	TRUE	vPNLLSGLQGk	95%	n+304 (+304), K+304 (+304)	42.60	25.00
5908	seq=translation; coord=6:127249169..127252532:1; parent_transcript=GRMZM2G018020_T01; parent_gene=GRMZM2G018020	GRMZM2G018020_P01	TRUE	TRUE	gQAWVVFEDVASATEALk	95%	n+304 (+304), K+304 (+304)	47.46	26.14
5909	seq=translation; coord=6:127249169..127252532:1; parent_transcript=GRMZM2G018020_T01; parent_gene=GRMZM2G018020	GRMZM2G018020_P01	TRUE	TRUE	iLDVLAFk	90%	n+304 (+304), K+304 (+304)	25.42	25.00
5910	seq=translation; coord=6:126779719..126784379:1; parent_transcript=GRMZM2G159369_T01; parent_gene=GRMZM2G159369	GRMZM2G159369_P01	TRUE	TRUE	gHLNQEAGLVPSLNPLVk	92%	n+304 (+304), K+304 (+304)	26.77	25.00
5911	seq=translation; coord=6:126779719..126784379:1; parent_transcript=GRMZM2G159369_T01; parent_gene=GRMZM2G159369	GRMZM2G159369_P01	TRUE	TRUE	lASVFk	92%	n+304 (+304), K+304 (+304)	25.59	25.00

5912	seq=translation; coord=6:126779719..126784379:1; parent_transcript=GRMZM2G159369_T01; parent_gene=GRMZM2G159369	GRMZM2G159369_P01	TRUE	TRUE	sVEALAEFVNSEAGTNVk	95%	n+304 (+304), K+304 (+304)	42.66	25.35
5913	seq=translation; coord=1:218981641..218987465:-1; parent_transcript=GRMZM5G895175_T01; parent_gene=GRMZM5G895175	GRMZM5G895175_P01	TRUE	TRUE	iSNMIEEWLk	95%	n+304 (+304), K+304 (+304)	36.79	25.80
5914	seq=translation; coord=1:218981641..218987465:-1; parent_transcript=GRMZM5G895175_T01; parent_gene=GRMZM5G895175	GRMZM5G895175_P01	TRUE	TRUE	lGFGLIGLGk	95%	n+304 (+304), K+304 (+304)	34.28	25.00
5915	seq=translation; coord=6:164260122..164263444:1; parent_transcript=GRMZM2G007122_T02; parent_gene=GRMZM2G007122	GRMZM2G007122_P02,GRMZM2G015287_P01, GRMZM2G115828_P01,GRMZM2G115828_P02, GRMZM2G115828_P03,GRMZM5G862131_P01, GRMZM5G862131_P02	TRUE	TRUE	lELFLPEEYPMAAPk	95%	n+304 (+304), K+304 (+304)	41.79	26.54
5916	seq=translation; coord=6:164260122..164263444:1; parent_transcript=GRMZM2G007122_T02; parent_gene=GRMZM2G007122	GRMZM2G007122_P02,GRMZM2G015287_P01, GRMZM2G115828_P01,GRMZM2G115828_P02, GRMZM2G115828_P03,GRMZM5G862131_P01, GRMZM5G862131_P02	TRUE	TRUE	lLSEPAPGISASPSEENMR	95%	n+304 (+304)	89.15	25.00
5917	seq=translation; coord=7:10365682..10368158:1; parent_transcript=GRMZM2G018074_T01; parent_gene=GRMZM2G018074	GRMZM2G018074_P01	TRUE	TRUE	sALEAVVAADEELTALQAEQAR	95%	n+304 (+304)	73.17	25.82
5918	seq=translation; coord=7:10365682..10368158:1; parent_transcript=GRMZM2G018074_T01; parent_gene=GRMZM2G018074	GRMZM2G018074_P01	TRUE	TRUE	vPGSSAADGTVDDNVR	95%	n+304 (+304)	71.72	25.00
5919	seq=translation; coord=9:12323292..12327304:-1; parent_transcript=GRMZM2G151923_T01; parent_gene=GRMZM2G151923	GRMZM2G151923_P01,GRMZM2G151923_P02, GRMZM2G151923_P03,GRMZM2G151923_P04, GRMZM2G151923_P05	TRUE	TRUE	eLEDEFR	92%	n+304 (+304)	29.23	25.00
5920	seq=translation; coord=9:12323292..12327304:-1; parent_transcript=GRMZM2G151923_T01; parent_gene=GRMZM2G151923	GRMZM2G151923_P01,GRMZM2G151923_P02, GRMZM2G151923_P03,GRMZM2G151923_P04, GRMZM2G151923_P05	TRUE	TRUE	lYIGNLDPR	95%	n+304 (+304)	32.42	25.31
5921	seq=translation; coord=3:218727007..218745183:1; parent_transcript=GRMZM2G047093_T01; parent_gene=GRMZM2G047093	GRMZM2G047093_P01	TRUE	TRUE	aNILEQLFLk	93%	n+304 (+304), K+304 (+304)	27.75	25.00
5922	seq=translation; coord=6:164682301..164684550:1; parent_transcript=GRMZM2G022619_T01; parent_gene=GRMZM2G022619	GRMZM2G022619_P01,GRMZM2G113720_P01	TRUE	TRUE	aLPTPTDEHPk	95%	n+304 (+304), K+304 (+304)	32.38	26.33
5923	seq=translation; coord=6:164682301..164684550:1; parent_transcript=GRMZM2G022619_T01; parent_gene=GRMZM2G022619	GRMZM2G022619_P01,GRMZM2G113720_P01	TRUE	TRUE	sPcIQIIk	95%	n+304 (+304), Carbamidomethyl (+57), K+304 (+304)	29.91	26.13
5924	seq=translation; coord=8:171187220..171190525:-1; parent_transcript=GRMZM2G028346_T01; parent_gene=GRMZM2G028346	GRMZM2G028346_P01,GRMZM2G130398_P01, GRMZM2G130398_P02,GRMZM2G130398_P03	TRUE	TRUE	aAAQAALEEMDAD	95%	n+304 (+304)	34.20	25.00
5925	seq=translation; coord=8:171187220..171190525:-1; parent_transcript=GRMZM2G028346_T01; parent_gene=GRMZM2G028346	GRMZM2G028346_P01,GRMZM2G130398_P01, GRMZM2G130398_P02,GRMZM2G130398_P03	TRUE	TRUE	dGIVLGVEk	95%	n+304 (+304), K+304 (+304)	45.14	25.00
5926	seq=translation; coord=9:7578400..7585814:1; parent_transcript=GRMZM2G120302_T01; parent_gene=GRMZM2G120302	GRMZM2G120302_P01	TRUE	TRUE	gLPVALVEYEGEQHGFR	95%	n+304 (+304)	56.85	25.12
5927	seq=translation; coord=9:7578400..7585814:1; parent_transcript=GRMZM2G120302_T01; parent_gene=GRMZM2G120302	GRMZM2G120302_P01	TRUE	TRUE	sPITADVVSGADR	95%	n+304 (+304)	35.86	25.60
5928	seq=translation; coord=9:7578400..7585814:1; parent_transcript=GRMZM2G120302_T01; parent_gene=GRMZM2G120302	GRMZM2G120302_P01	TRUE	TRUE	tLAQEYGGGAFAVDk	95%	n+304 (+304), K+304 (+304)	35.10	25.91

5929	seq=translation; coord=UNKNOWN:5634502..5650723:1; parent_transcript=GRMZM2G011070_T01; parent_gene=GRMZM2G011070	GRMZM2G011070_P01	TRUE	TRUE	lAHEFWR	95%	n+304 (+304)	29.31	25.00
5930	seq=translation; coord=UNKNOWN:5634502..5650723:1; parent_transcript=GRMZM2G011070_T01; parent_gene=GRMZM2G011070	GRMZM2G011070_P01	TRUE	TRUE	vFHSEWIPR	95%	n+304 (+304)	33.43	25.28
5931	seq=translation; coord=8:156372419..156374495:-1; parent_transcript=GRMZM2G073622_T01; parent_gene=GRMZM2G073622	GRMZM2G073622_P01,GRMZM2G073622_P02, GRMZM2G073622_P03	TRUE	TRUE	aAESSAEWTVk	95%	n+304 (+304), K+304 (+304)	58.84	26.20
5932	seq=translation; coord=8:156372419..156374495:-1; parent_transcript=GRMZM2G073622_T01; parent_gene=GRMZM2G073622	GRMZM2G073622_P01,GRMZM2G073622_P02, GRMZM2G073622_P03	TRUE	TRUE	iAEcLVGDETGIIVFTAR	94%	n+304 (+304), Carbamidomethyl (+57)	28.21	26.18
5933	seq=translation; coord=4:175739965..175751055:-1; parent_transcript=GRMZM2G181362_T01; parent_gene=GRMZM2G181362	GRMZM2G181362_P01,GRMZM2G181362_P02	TRUE	TRUE	aGLIDFLHGLGQR	95%	n+304 (+304)	49.16	26.27
5934	seq=translation; coord=4:175739965..175751055:-1; parent_transcript=GRMZM2G181362_T01; parent_gene=GRMZM2G181362	GRMZM2G181362_P01,GRMZM2G181362_P02	TRUE	TRUE	eLPAFALEHLPNR	95%	n+304 (+304)	34.99	25.56
5935	seq=translation; coord=5:169232491..169237426:1; parent_transcript=GRMZM2G073950_T01; parent_gene=GRMZM2G073950	GRMZM2G073950_P01	TRUE	TRUE	iVLVQELFEGHYFcQAQR	95%	n+304 (+304), Carbamidomethyl (+57)	44.06	25.00
5936	seq=translation; coord=5:169232491..169237426:1; parent_transcript=GRMZM2G073950_T01; parent_gene=GRMZM2G073950	GRMZM2G073950_P01	TRUE	TRUE	vLLTLDGEk	95%	n+304 (+304), K+304 (+304)	33.90	25.54
5937	seq=translation; coord=5:39817839..39821668:1; parent_transcript=GRMZM2G048549_T01; parent_gene=GRMZM2G048549	GRMZM2G048549_P01	TRUE	TRUE	vLSDILLGMAAGLk	95%	n+304 (+304), K+304 (+304)	35.85	25.00
5938	seq=translation; coord=2:173380725..173391388:-1; parent_transcript=GRMZM2G106213_T01; parent_gene=GRMZM2G106213	GRMZM2G106213_P01,GRMZM2G106213_P02	TRUE	TRUE	eLPYIASMGIYVFSk	95%	n+304 (+304), K+304 (+304)	48.78	25.56
5939	seq=translation; coord=2:173380725..173391388:-1; parent_transcript=GRMZM2G106213_T01; parent_gene=GRMZM2G106213	GRMZM2G106213_P01,GRMZM2G106213_P02	TRUE	TRUE	eTDADITVAALPMDEQR	95%	n+304 (+304)	79.98	25.00
5940	seq=translation; coord=2:173380725..173391388:-1; parent_transcript=GRMZM2G106213_T01; parent_gene=GRMZM2G106213	GRMZM2G106213_P01,GRMZM2G106213_P02	TRUE	TRUE	iINFDNVQEAVR	95%	n+304 (+304)	39.37	25.30
5941	seq=translation; coord=2:173380725..173391388:-1; parent_transcript=GRMZM2G106213_T01; parent_gene=GRMZM2G106213	GRMZM2G106213_P01,GRMZM2G106213_P02	TRUE	TRUE	sMMVDTTILGLDPER	91%	n+304 (+304)	28.21	25.00
5942	seq=translation; coord=8:173029283..173035156:1; parent_transcript=GRMZM2G124047_T01; parent_gene=GRMZM2G124047	GRMZM2G124047_P01,GRMZM2G124047_P02	TRUE	TRUE	fAALVVFAEHR	95%	n+304 (+304)	53.59	25.89
5943	seq=translation; coord=6:148703816..148706201:1; parent_transcript=AC235543.1_FGT003; parent_gene=AC235543.1_FG003	AC235543.1_FGP003	TRUE	TRUE	lVLDGGGGDVDGGEDYVETR	95%	n+304 (+304)	47.52	25.00
5944	seq=translation; coord=6:148703816..148706201:1; parent_transcript=AC235543.1_FGT003; parent_gene=AC235543.1_FG003	AC235543.1_FGP003	TRUE	TRUE	sDPSVDTFAEPAAVDAGDHDLPELR	95%	n+304 (+304)	82.42	25.00
5945	seq=translation; coord=4:223661025..223669104:-1; parent_transcript=GRMZM2G068489_T01; parent_gene=GRMZM2G068489	GRMZM2G068489_P01,GRMZM2G109315_P01, GRMZM2G109315_P02	TRUE	TRUE	vLVLALGDLHIPHR	95%	n+304 (+304)	36.34	25.00
5946	seq=translation; coord=4:223661025..223669104:-1; parent_transcript=GRMZM2G068489_T01; parent_gene=GRMZM2G068489	GRMZM2G068489_P01,GRMZM2G109315_P01, GRMZM2G109315_P02	TRUE	TRUE	vVVYVYELIDGEVk	95%	n+304 (+304), K+304 (+304)	48.07	25.04

5947	seq=translation; coord=4:214239084..214241779:1; parent_transcript=GRMZM2G126190_T01; parent_gene=GRMZM2G126190	GRMZM2G126190_P01,GRMZM2G126190_P02, GRMZM2G126190_P03	TRUE	TRUE	lAYVALDYDQELENAk	95%	n+304 (+304), K+304 (+304)	64.62	25.40
5948	seq=translation; coord=2:25300018..25310642:1; parent_transcript=GRMZM5G827171_T01; parent_gene=GRMZM5G827171	GRMZM5G827171_P01,GRMZM5G827171_P02, GRMZM5G827171_P03	TRUE	TRUE	aSLVDIAESFk	95%	n+304 (+304), K+304 (+304)	32.22	25.37
5949	seq=translation; coord=2:25300018..25310642:1; parent_transcript=GRMZM5G827171_T01; parent_gene=GRMZM5G827171	GRMZM5G827171_P01,GRMZM5G827171_P02, GRMZM5G827171_P03	TRUE	TRUE	fYAALDEINFFk	95%	n+304 (+304), K+304 (+304)	53.76	26.32
5950	seq=translation; coord=7:33992878..33998025:-1; parent_transcript=GRMZM2G051771_T01; parent_gene=GRMZM2G051771	GRMZM2G051771_P01,GRMZM2G051771_P02	TRUE	TRUE	qFFPHLAVGFEDPR	95%	n+304 (+304)	35.56	25.00
5951	seq=translation; coord=7:33992878..33998025:-1; parent_transcript=GRMZM2G051771_T01; parent_gene=GRMZM2G051771	GRMZM2G051771_P01,GRMZM2G051771_P02	TRUE	TRUE	qFFPHLAVGFEDPR	93%	Pyro-cmC (-17), n+304 (+304)	29.78	25.00
5952	seq=translation; coord=7:33992878..33998025:-1; parent_transcript=GRMZM2G051771_T01; parent_gene=GRMZM2G051771	GRMZM2G051771_P01,GRMZM2G051771_P02	TRUE	TRUE	sDYQNVMVFQSSTYGk	95%	n+304 (+304), K+304 (+304)	50.60	25.00
5953	seq=translation; coord=7:94510636..94515443:1; parent_transcript=GRMZM2G053781_T01; parent_gene=GRMZM2G053781	GRMZM2G053781_P01,GRMZM2G053781_P02	TRUE	TRUE	aQSAGLcSSIEELDLTlk	95%	n+304 (+304), Carbamidomethyl (+57), K+304 (+304)	33.12	25.48
5954	seq=translation; coord=7:94510636..94515443:1; parent_transcript=GRMZM2G053781_T01; parent_gene=GRMZM2G053781	GRMZM2G053781_P01,GRMZM2G053781_P02	TRUE	TRUE	lVDAEIGLLQEDLDAATAR	95%	n+304 (+304)	43.32	25.42
5955	seq=translation; coord=6:89682252..89693247:1; parent_transcript=GRMZM2G044398_T01; parent_gene=GRMZM2G044398	GRMZM2G044398_P01,GRMZM2G044398_P02, GRMZM2G044398_P03,GRMZM2G044398_P04, GRMZM2G044398_P05,GRMZM2G044398_P06, GRMZM2G044398_P07	TRUE	TRUE	lEAILEk	93%	n+304 (+304), K+304 (+304)	27.24	25.00
5956	seq=translation; coord=9:126770546..126773822:1; parent_transcript=GRMZM2G426229_T01; parent_gene=GRMZM2G426229	GRMZM2G426229_P01	TRUE	TRUE	eIAVVFAEENR	88%	n+304 (+304)	26.38	25.40
5957	seq=translation; coord=9:126770546..126773822:1; parent_transcript=GRMZM2G426229_T01; parent_gene=GRMZM2G426229	GRMZM2G426229_P01	TRUE	TRUE	gFGFIQYFDPEDASDAk	95%	n+304 (+304), K+304 (+304)	61.47	25.00
5958	seq=translation; coord=2:2545896..2547904:1; parent_transcript=GRMZM2G010257_T01; parent_gene=GRMZM2G010257	GRMZM2G010257_P01,GRMZM2G075630_P01, GRMZM2G102891_P01,GRMZM2G102891_P02, GRMZM2G102891_P03,GRMZM2G105712_P01, GRMZM2G105712_P02,GRMZM2G105712_P03, GRMZM2G105712_P04,GRMZM2G105712_P05, GRMZM2G105712_P06,GRMZM2G119809_P01, GRMZM2G119809_P02,GRMZM2G119809_P03, GRMZM2G119809_P04	TRUE	TRUE	dITELIAAGR	91%	n+304 (+304)	29.39	26.13
5959	seq=translation; coord=2:2545896..2547904:1; parent_transcript=GRMZM2G010257_T01; parent_gene=GRMZM2G010257	GRMZM2G010257_P01,GRMZM2G075630_P01, GRMZM2G102891_P01,GRMZM2G102891_P02, GRMZM2G102891_P03,GRMZM2G105712_P01, GRMZM2G105712_P02,GRMZM2G105712_P03, GRMZM2G105712_P04,GRMZM2G105712_P05, GRMZM2G105712_P06,GRMZM2G119809_P01, GRMZM2G119809_P02,GRMZM2G119809_P03, GRMZM2G119809_P04	TRUE	TRUE	mELLLSQLSGk	95%	n+304 (+304), K+304 (+304)	34.55	25.68
5960	seq=translation; coord=8:70525339..70528822:1; parent_transcript=GRMZM2G413652_T01; parent_gene=GRMZM2G413652	GRMZM2G413652_P01,GRMZM2G413652_P06	TRUE	TRUE	aFTVGIGGPVGTGk	95%	n+304 (+304), K+304 (+304)	52.55	25.00

5961	seq=translation; coord=8:70525339..70528822:1; parent_transcript=GRMZM2G413652_T01; parent_gene=GRMZM2G413652	GRMZM2G413652_P01,GRMZM2G413652_P06	TRUE	TRUE	eLADYIIYIIDVSGGDk	94%	n+304 (+304), K+304 (+304)	27.16	25.25
5962	seq=translation; coord=8:70525339..70528822:1; parent_transcript=GRMZM2G413652_T01; parent_gene=GRMZM2G413652	GRMZM2G413652_P01,GRMZM2G413652_P06	TRUE	TRUE	hGVGVEGIVNHILQAWEIATGNk	95%	n+304 (+304), K+304 (+304)	31.73	25.00
5963	seq=translation; coord=1:289192462..289217741:1; parent_transcript=GRMZM5G825524_T01; parent_gene=GRMZM5G825524	GRMZM5G825524_P01,GRMZM5G844195_P02, GRMZM5G844195_P03	TRUE	TRUE	aQITAIHLIIGTLQR	95%	n+304 (+304)	45.24	25.00
5964	seq=translation; coord=1:289192462..289217741:1; parent_transcript=GRMZM5G825524_T01; parent_gene=GRMZM5G825524	GRMZM5G825524_P01,GRMZM5G844195_P02, GRMZM5G844195_P03	TRUE	TRUE	iEALFDLIk	91%	n+304 (+304), K+304 (+304)	25.43	25.00
5965	seq=translation; coord=1:289192462..289217741:1; parent_transcript=GRMZM5G825524_T01; parent_gene=GRMZM5G825524	GRMZM5G825524_P01,GRMZM5G844195_P02, GRMZM5G844195_P03	TRUE	TRUE	vMAVVIIQSIMk	95%	n+304 (+304), K+304 (+304)	30.10	25.00
5966	seq=translation; coord=1:2038186..2040079:1; parent_transcript=GRMZM2G164696_T01; parent_gene=GRMZM2G164696	GRMZM2G164696_P01,GRMZM2G164696_P03	TRUE	TRUE	fWEVVcAEHGIDATGR	95%	n+304 (+304), Carbamidomethyl (+57)	81.53	25.00
5967	seq=translation; coord=1:2038186..2040079:1; parent_transcript=GRMZM2G164696_T01; parent_gene=GRMZM2G164696	GRMZM2G164696_P01,GRMZM2G164696_P03	TRUE	TRUE	yGGDSDLQLER	90%	n+304 (+304)	27.17	25.00
5968	seq=translation; coord=5:56776697..56780711:-1; parent_transcript=GRMZM2G116034_T01; parent_gene=GRMZM2G116034	GRMZM2G116034_P01,GRMZM2G116034_P02	TRUE	TRUE	dQIYDIFQLLPAk	89%	n+304 (+304), K+304 (+304)	25.13	25.00
5969	seq=translation; coord=6:160675824..160684398:1; parent_transcript=GRMZM2G587327_T01; parent_gene=GRMZM2G587327	GRMZM2G587327_P01	TRUE	TRUE	iDINHFFSILSDIIR	95%	n+304 (+304)	37.80	25.00
5970	seq=translation; coord=6:160675824..160684398:1; parent_transcript=GRMZM2G587327_T01; parent_gene=GRMZM2G587327	GRMZM2G587327_P01	TRUE	TRUE	rFPNALAVVADSWVFTLR	95%	n+304 (+304)	39.33	25.59
5971	seq=translation; coord=7:162717116..162718128:1; parent_transcript=GRMZM2G309970_T01; parent_gene=GRMZM2G309970	GRMZM2G309970_P01,GRMZM2G378121_P01	TRUE	TRUE	aAEALLPSLSIWPPSQR	95%	n+304 (+304)	36.47	25.60
5972	seq=translation; coord=8:166372284..166377485:1; parent_transcript=GRMZM2G047214_T01; parent_gene=GRMZM2G047214	GRMZM2G047214_P01,GRMZM2G056495_P01, GRMZM2G056495_P02	TRUE	TRUE	mTMDDLIAR	94%	n+304 (+304)	28.91	25.00
5973	seq=translation; coord=9:139381433..139385689:-1; parent_transcript=GRMZM2G166767_T01; parent_gene=GRMZM2G166767	GRMZM2G166767_P01,GRMZM2G166767_P02	TRUE	TRUE	aMVEELLk	95%	n+304 (+304), K+304 (+304)	30.15	25.00
5974	seq=translation; coord=9:139381433..139385689:-1; parent_transcript=GRMZM2G166767_T01; parent_gene=GRMZM2G166767	GRMZM2G166767_P01,GRMZM2G166767_P02	TRUE	TRUE	mPISSDLSNPR	88%	n+304 (+304)	26.01	25.00
5975	seq=translation; coord=9:139381433..139385689:-1; parent_transcript=GRMZM2G166767_T01; parent_gene=GRMZM2G166767	GRMZM2G166767_P01,GRMZM2G166767_P02	TRUE	TRUE	nILITGAAGFIASHVANR	95%	n+304 (+304)	69.92	25.75
5976	seq=translation; coord=9:139381433..139385689:-1; parent_transcript=GRMZM2G166767_T01; parent_gene=GRMZM2G166767	GRMZM2G166767_P01,GRMZM2G166767_P02	TRUE	TRUE	sYLYcEDVAEAFEVVLHk	95%	n+304 (+304), Carbamidomethyl (+57), K+304 (+304)	78.01	25.00
5977	seq=translation; coord=5:181396172..181399390:-1; parent_transcript=GRMZM2G031496_T01; parent_gene=GRMZM2G031496	GRMZM2G031496_P01,GRMZM2G097190_P01	TRUE	TRUE	gPDPDVVAIVER	95%	n+304 (+304)	43.43	25.95
5978	seq=translation; coord=5:181396172..181399390:-1; parent_transcript=GRMZM2G031496_T01; parent_gene=GRMZM2G031496	GRMZM2G031496_P01,GRMZM2G097190_P01	TRUE	TRUE	vGVPVLGVVENMSGLR	95%	n+304 (+304)	45.62	25.79

5979	seq=translation; coord=1:268558195..268564501:-1; parent_transcript=GRMZM2G000622_T01; parent_gene=GRMZM2G000622	GRMZM2G000622_P01	TRUE	TRUE	dLWEETSFQLEELQR	95%	n+304 (+304)	50.39	25.00
5980	seq=translation; coord=1:268558195..268564501:-1; parent_transcript=GRMZM2G000622_T01; parent_gene=GRMZM2G000622	GRMZM2G000622_P01	TRUE	TRUE	gDPEIGMLVVk	95%	n+304 (+304), K+304 (+304)	34.02	25.45
5981	seq=translation; coord=1:268558195..268564501:-1; parent_transcript=GRMZM2G000622_T01; parent_gene=GRMZM2G000622	GRMZM2G000622_P01	TRUE	TRUE	lAVGEALTNLVWAk	95%	n+304 (+304), K+304 (+304)	51.74	25.00
5982	seq=translation; coord=2:46534345..46537526:-1; parent_transcript=AC202073.4_FGT001; parent_gene=AC202073.4_FG001	AC202073.4_FGP001,GRMZM2G113453_P01	TRUE	TRUE	vPALASVIVEALk	95%	n+304 (+304), K+304 (+304)	40.01	25.00
5983	seq=translation; coord=1:78701399..78707799:-1; parent_transcript=GRMZM2G050984_T01; parent_gene=GRMZM2G050984	GRMZM2G050984_P01	TRUE	TRUE	gFGGPQGLLIAENWIHHIATELQR	94%	n+304 (+304)	25.42	25.00
5984	seq=translation; coord=5:123629674..123636311:1; parent_transcript=GRMZM2G063851_T01; parent_gene=GRMZM2G063851	GRMZM2G063851_P01,GRMZM2G063851_P04	TRUE	TRUE	gMTGMLWETSLLDPEEGIR	95%	n+304 (+304)	29.06	25.00
5985	seq=translation; coord=3:117076600..117087785:1; parent_transcript=GRMZM2G069758_T01; parent_gene=GRMZM2G069758	GRMZM2G069758_P01,GRMZM2G069758_P02, GRMZM2G069758_P03,GRMZM2G069758_P05	TRUE	TRUE	aAELAVSSLQALLk	95%	n+304 (+304), K+304 (+304)	28.94	25.00
5986	seq=translation; coord=2:47087776..47091009:1; parent_transcript=GRMZM2G087192_T01; parent_gene=GRMZM2G087192	GRMZM2G087192_P01	TRUE	TRUE	fALEVTQAVVDEIGADk	95%	n+304 (+304), K+304 (+304)	41.54	25.69
5987	seq=translation; coord=3:17941693..17947153:-1; parent_transcript=GRMZM2G100881_T01; parent_gene=GRMZM2G100881	GRMZM2G100881_P01,GRMZM2G100881_P02	TRUE	TRUE	lPLLIEVLSk	95%	n+304 (+304), K+304 (+304)	33.55	25.00
5988	seq=translation; coord=5:207730826..207733163:-1; parent_transcript=GRMZM2G116981_T01; parent_gene=GRMZM2G116981	GRMZM2G116981_P01	TRUE	TRUE	lLPLPELLSSIASIk	95%	n+304 (+304), K+304 (+304)	35.96	25.00
5989	seq=translation; coord=4:36408206..36412230:1; parent_transcript=GRMZM2G131473_T01; parent_gene=GRMZM2G131473	GRMZM2G131473_P01,GRMZM2G131473_P02	TRUE	TRUE	sIIkPGMLMVDLcETLENMVR	95%	n+304 (+304), K+304 (+304), Carbamidomethyl (+57)	45.09	25.00
5990	seq=translation; coord=4:229898346..229903473:1; parent_transcript=GRMZM2G139643_T01; parent_gene=GRMZM2G139643	GRMZM2G139643_P01,GRMZM2G139643_P02, GRMZM2G139643_P03,GRMZM2G139643_P04, GRMZM2G139643_P05	TRUE	TRUE	vLEDDHVIDGR	95%	n+304 (+304)	55.67	25.00
5991	seq=translation; coord=5:74286010..74289403:1; parent_transcript=GRMZM2G152929_T03; parent_gene=GRMZM2G152929	GRMZM2G152929_P03	TRUE	TRUE	gEGLYDGLDWLASTLk	95%	n+304 (+304), K+304 (+304)	35.01	25.87
5992	seq=translation; coord=4:41369510..41378299:1; parent_transcript=GRMZM2G138060_T01; parent_gene=GRMZM2G138060	GRMZM2G138060_P01	TRUE	TRUE	dLVIYEMHLR	95%	n+304 (+304)	43.16	26.35
5993	seq=translation; coord=4:41369510..41378299:1; parent_transcript=GRMZM2G138060_T01; parent_gene=GRMZM2G138060	GRMZM2G138060_P01	TRUE	TRUE	eAPYDFLTDGLPDR	95%	n+304 (+304)	44.43	25.00
5994	seq=translation; coord=4:41369510..41378299:1; parent_transcript=GRMZM2G138060_T01; parent_gene=GRMZM2G138060	GRMZM2G138060_P01	TRUE	TRUE	gIEVIMDVVFNHTAEGNEk	95%	n+304 (+304), K+304 (+304)	33.10	25.00
5995	seq=translation; coord=4:41369510..41378299:1; parent_transcript=GRMZM2G138060_T01; parent_gene=GRMZM2G138060	GRMZM2G138060_P01	TRUE	TRUE	vTEEVPLDPLLNR	95%	n+304 (+304)	33.44	25.79
5996	seq=translation; coord=9:119710257..119715669:-1; parent_transcript=GRMZM2G151387_T01; parent_gene=GRMZM2G151387	GRMZM2G151387_P01,GRMZM2G151387_P02	TRUE	TRUE	aPPLQQTQSVPR	95%	n+304 (+304)	40.81	25.90

5997	seq=translation; coord=9:119710257..119715669:-1; parent_transcript=GRMZM2G151387_T01; parent_gene=GRMZM2G151387	GRMZM2G151387_P01,GRMZM2G151387_P02	TRUE	TRUE	aVQELLGk	95%	n+304 (+304), K+304 (+304)	28.49	25.28
5998	seq=translation; coord=9:119710257..119715669:-1; parent_transcript=GRMZM2G151387_T01; parent_gene=GRMZM2G151387	GRMZM2G151387_P01,GRMZM2G151387_P02	TRUE	TRUE	eVILPDSVVEASk	95%	n+304 (+304), K+304 (+304)	44.19	25.00
5999	seq=translation; coord=9:119710257..119715669:-1; parent_transcript=GRMZM2G151387_T01; parent_gene=GRMZM2G151387	GRMZM2G151387_P01,GRMZM2G151387_P02	TRUE	TRUE	fGATEASk	93%	n+304 (+304), K+304 (+304)	29.48	26.42
6000	seq=translation; coord=5:74640062..74641850:-1; parent_transcript=GRMZM2G017249_T01; parent_gene=GRMZM2G017249	GRMZM2G017249_P01	TRUE	TRUE	dIDISSVVLDNR	94%	n+304 (+304)	31.69	25.59
6001	seq=translation; coord=5:74640062..74641850:-1; parent_transcript=GRMZM2G017249_T01; parent_gene=GRMZM2G017249	GRMZM2G017249_P01	TRUE	TRUE	eVQQELPVSHFLMNLILGk	94%	n+304 (+304), K+304 (+304)	26.06	25.00
6002	seq=translation; coord=5:74640062..74641850:-1; parent_transcript=GRMZM2G017249_T01; parent_gene=GRMZM2G017249	GRMZM2G017249_P01	TRUE	TRUE	lVLEPLPEDk	95%	n+304 (+304), K+304 (+304)	33.53	25.00
6003	seq=translation; coord=4:5455601..5457606:1; parent_transcript=GRMZM2G154523_T01; parent_gene=GRMZM2G154523	GRMZM2G154523_P01	TRUE	TRUE	gLIPATIIAYLEAk	95%	n+304 (+304), K+304 (+304)	35.06	25.00
6004	seq=translation; coord=4:5455601..5457606:1; parent_transcript=GRMZM2G154523_T01; parent_gene=GRMZM2G154523	GRMZM2G154523_P01	TRUE	TRUE	vADTVTNVIVPAFDVk	95%	n+304 (+304), K+304 (+304)	45.99	25.00
6005	seq=translation; coord=2:3805095..3807781:-1; parent_transcript=GRMZM2G173341_T01; parent_gene=GRMZM2G173341	GRMZM2G173341_P01,GRMZM2G173341_P02, GRMZM2G173341_P03	TRUE	TRUE	aADAMVEWNNLWk	95%	n+304 (+304), K+304 (+304)	64.47	25.47
6006	seq=translation; coord=2:3805095..3807781:-1; parent_transcript=GRMZM2G173341_T01; parent_gene=GRMZM2G173341	GRMZM2G173341_P01,GRMZM2G173341_P02, GRMZM2G173341_P03	TRUE	TRUE	dVLGPAMFPGLR	95%	n+304 (+304)	42.61	26.00
6007	seq=translation; coord=2:3805095..3807781:-1; parent_transcript=GRMZM2G173341_T01; parent_gene=GRMZM2G173341	GRMZM2G173341_P01,GRMZM2G173341_P02, GRMZM2G173341_P03	TRUE	TRUE	vMDDVLPFFQSLk	95%	n+304 (+304), K+304 (+304)	48.40	25.00
6008	seq=translation; coord=3:213896741..213905427:1; parent_transcript=GRMZM2G466833_T01; parent_gene=GRMZM2G466833	GRMZM2G466833_P01	TRUE	TRUE	aNLPVTDVNVPVVGGHAGITILPLFSQATPATNALSDEDIk	95%	n+304 (+304), K+304 (+304)	29.32	25.00
6009	seq=translation; coord=3:213896741..213905427:1; parent_transcript=GRMZM2G466833_T01; parent_gene=GRMZM2G466833	GRMZM2G466833_P01	TRUE	TRUE	nGVEEVLGLGELSDFEk	95%	n+304 (+304), K+304 (+304)	31.35	25.09
6010	seq=translation; coord=3:213896741..213905427:1; parent_transcript=GRMZM2G466833_T01; parent_gene=GRMZM2G466833	GRMZM2G466833_P01	TRUE	TRUE	nLSTAIAk	95%	n+304 (+304), K+304 (+304)	39.26	26.33
6011	seq=translation; coord=10:19475272..19481312:1; parent_transcript=GRMZM2G181227_T01; parent_gene=GRMZM2G181227	GRMZM2G181227_P01	TRUE	TRUE	iDDPQWEDLNLPAR	95%	n+304 (+304)	61.64	25.00
6012	seq=translation; coord=10:19475272..19481312:1; parent_transcript=GRMZM2G181227_T01; parent_gene=GRMZM2G181227	GRMZM2G181227_P01	TRUE	TRUE	lEQVPDEAVEQYFLR	95%	n+304 (+304)	36.09	25.00
6013	seq=translation; coord=4:42919325..42920272:1; parent_transcript=GRMZM2G154578_T01; parent_gene=GRMZM2G154578	GRMZM2G154578_P01	TRUE	TRUE	aYAAcEDLPk	95%	n+304 (+304), Carbamidomethyl (+57), K+304 (+304)	38.14	25.00
6014	seq=translation; coord=4:42919325..42920272:1; parent_transcript=GRMZM2G154578_T01; parent_gene=GRMZM2G154578	GRMZM2G154578_P01	TRUE	TRUE	hAFQPDNLGAk	95%	n+304 (+304), K+304 (+304)	39.32	25.97

6015	seq=translation; coord=4:81250432..81251801:-1; parent_transcript=GRMZM2G004878_T01; parent_gene=GRMZM2G004878	GRMZM2G004878_P01	TRUE	TRUE	eSGELVFVk	95%	n+304 (+304), K+304 (+304)	35.05	26.71
6016	seq=translation; coord=4:81250432..81251801:-1; parent_transcript=GRMZM2G004878_T01; parent_gene=GRMZM2G004878	GRMZM2G004878_P01	TRUE	TRUE	vRPVVPSETAAA	89%	n+304 (+304)	28.50	26.47
6017	seq=translation; coord=6:142082329..142086400:1; parent_transcript=AC215201.3_FGT005; parent_gene=AC215201.3_FG005	AC215201.3_FGP005	TRUE	TRUE	lAGGVAVIk	95%	n+304 (+304), K+304 (+304)	35.46	25.00
6018	seq=translation; coord=6:142082329..142086400:1; parent_transcript=AC215201.3_FGT005; parent_gene=AC215201.3_FG005	AC215201.3_FGP005	TRUE	TRUE	vGAATETELEDR	95%	n+304 (+304)	64.41	25.00
6019	seq=translation; coord=1:34386070..34400519:-1; parent_transcript=GRMZM2G102483_T02; parent_gene=GRMZM2G102483	GRMZM2G102483_P02,GRMZM2G102483_P03	TRUE	TRUE	aEFQEALSDILLGMAAGLk	95%	n+304 (+304), K+304 (+304)	61.36	25.38
6020	seq=translation; coord=4:237941870..237948667:1; parent_transcript=GRMZM2G139341_T01; parent_gene=GRMZM2G139341	GRMZM2G139341_P01,GRMZM2G139341_P02	TRUE	TRUE	hQLLSFMDELIPTIR	95%	n+304 (+304)	68.86	26.17
6021	seq=translation; coord=4:237941870..237948667:1; parent_transcript=GRMZM2G139341_T01; parent_gene=GRMZM2G139341	GRMZM2G139341_P01,GRMZM2G139341_P02	TRUE	TRUE	sAGLQAIDEIVPTLLR	95%	n+304 (+304)	54.59	25.45
6022	seq=translation; coord=1:285124791..285129572:-1; parent_transcript=GRMZM2G058584_T01; parent_gene=GRMZM2G058584	GRMZM2G058584_P01,GRMZM2G058584_P02	TRUE	TRUE	eAFDVAYDNIyAFHVSQk	95%	n+304 (+304), iTRAQ8plex (+304), K+304 (+304)	26.37	25.00
6023	seq=translation; coord=1:285124791..285129572:-1; parent_transcript=GRMZM2G058584_T01; parent_gene=GRMZM2G058584	GRMZM2G058584_P01,GRMZM2G058584_P02	TRUE	TRUE	iDFSSIFGTVNPIVEDVR	95%	n+304 (+304)	49.43	25.17
6024	seq=translation; coord=6:154130608..154132782:-1; parent_transcript=GRMZM2G361064_T01; parent_gene=GRMZM2G361064	GRMZM2G361064_P01	TRUE	TRUE	aGIVPVAFR	94%	n+304 (+304)	30.92	25.00
6025	seq=translation; coord=6:154130608..154132782:-1; parent_transcript=GRMZM2G361064_T01; parent_gene=GRMZM2G361064	GRMZM2G361064_P01	TRUE	TRUE	qALSFQVTASDGR	95%	n+304 (+304)	50.50	25.00
6026	seq=translation; coord=6:39088029..39088716:1; parent_transcript=GRMZM2G012928_T01; parent_gene=GRMZM2G012928	GRMZM2G012928_P01	TRUE	TRUE	dMPNANIQVLPVGSLVTQDFRPDR	94%	n+304 (+304)	27.09	25.00
6027	seq=translation; coord=6:39088029..39088716:1; parent_transcript=GRMZM2G012928_T01; parent_gene=GRMZM2G012928	GRMZM2G012928_P01	TRUE	TRUE	eNkTSWPEVVGMSIk	95%	n+304 (+304), K+304 (+304), K+304 (+304)	26.62	25.00
6028	seq=translation; coord=5:205237019..205240533:-1; parent_transcript=GRMZM5G872068_T01; parent_gene=GRMZM5G872068	GRMZM5G872068_P01	TRUE	TRUE	dTNWPLGWPIGGFPGPQGPYYcGIGAEk	95%	n+304 (+304), Carbamidomethyl (+57), K+304 (+304)	36.18	25.00
6029	seq=translation; coord=1:292237691..292240994:1; parent_transcript=GRMZM2G129015_T01; parent_gene=GRMZM2G129015	GRMZM2G129015_P01,GRMZM2G129015_P02, GRMZM5G899149_P01	TRUE	TRUE	nIVEQAAVR	95%	n+304 (+304)	34.09	25.85
6030	seq=translation; coord=5:7724527..7726624:-1; parent_transcript=GRMZM2G050833_T01; parent_gene=GRMZM2G050833	GRMZM2G050833_P01,GRMZM2G050833_P02, GRMZM2G056231_P01,GRMZM2G149775_P01, GRMZM2G149775_P02	TRUE	TRUE	gDEELDTLIk	95%	n+304 (+304), K+304 (+304)	34.98	26.45
6031	seq=translation; coord=1:153349374..153351332:1; parent_transcript=GRMZM2G422631_T01; parent_gene=GRMZM2G422631	GRMZM2G422631_P01	TRUE	TRUE	fLSYQDTLLDATGR	95%	n+304 (+304)	43.94	25.00
6032	seq=translation; coord=5:158865959..158869458:1; parent_transcript=GRMZM2G098298_T01; parent_gene=GRMZM2G098298	GRMZM2G098298_P01	TRUE	TRUE	hLDHGVLLVGYGASGFAPIR	95%	n+304 (+304)	42.24	26.01

6033	seq=translation; coord=6:163821880..163829466:1; parent_transcript=GRMZM2G086132_T01; parent_gene=GRMZM2G086132	GRMZM2G086132_P01	TRUE	TRUE	lTPSALLLSVLQQDPDVR	95%	n+304 (+304)	70.76	25.24
6034	seq=translation; coord=1:261408681..261412620:1; parent_transcript=GRMZM2G028379_T01; parent_gene=GRMZM2G028379	GRMZM2G028379_P01,GRMZM2G028379_P02, GRMZM2G028379_P03,GRMZM2G028379_P04, GRMZM2G028379_P05	TRUE	TRUE	aVDAVLGPR	86%	n+304 (+304)	25.38	25.00
6035	seq=translation; coord=8:14287038..14292775:1; parent_transcript=GRMZM2G085254_T01; parent_gene=GRMZM2G085254	GRMZM2G085254_P01,GRMZM2G085254_P02, GRMZM2G085254_P03	TRUE	TRUE	lPLLIDVLSk	95%	n+304 (+304), K+304 (+304)	31.15	25.00
6036	seq=translation; coord=9:17633649..17644407:1; parent_transcript=GRMZM2G129451_T02; parent_gene=GRMZM2G129451	GRMZM2G129451_P02	TRUE	TRUE	gIDLIQLIIPDLMR	95%	n+304 (+304)	47.80	25.00
6037	seq=translation; coord=8:171439571..171440653:1; parent_transcript=AC204359.3_FGT005; parent_gene=AC204359.3_FG005	AC204359.3_FGP005,GRMZM2G130558_P01	TRUE	TRUE	aAQADEEAGSLLQER	95%	n+304 (+304)	59.26	25.00
6038	seq=translation; coord=2:37901719..37904325:1; parent_transcript=AC208221.3_FGT002; parent_gene=AC208221.3_FG002	AC208221.3_FGP002	TRUE	TRUE	vcEFVESDPPGYR	95%	n+304 (+304), Carbamidomethyl (+57)	33.04	25.00
6039	seq=translation; coord=2:221237698..221239030:-1; parent_transcript=GRMZM2G003852_T01; parent_gene=GRMZM2G003852	GRMZM2G003852_P01	TRUE	TRUE	vLPFSFLLPPR	93%	n+304 (+304)	29.75	25.25
6040	seq=translation; coord=8:109958740..109965366:-1; parent_transcript=GRMZM2G015906_T01; parent_gene=GRMZM2G015906	GRMZM2G015906_P01	TRUE	TRUE	lVDYYVPLFFTk	95%	n+304 (+304), K+304 (+304)	36.66	25.69
6041	seq=translation; coord=10:125302195..125306046:-1; parent_transcript=GRMZM2G025054_T01; parent_gene=GRMZM2G025054	GRMZM2G025054_P01,GRMZM2G025054_P02	TRUE	TRUE	sLPELLAELDGLR	95%	n+304 (+304)	60.14	25.85
6042	seq=translation; coord=10:10582274..10586991:-1; parent_transcript=GRMZM2G026231_T01; parent_gene=GRMZM2G026231	GRMZM2G026231_P01,GRMZM2G087212_P01, GRMZM2G420310_P01	TRUE	TRUE	eDDDLESLER	95%	n+304 (+304)	38.06	25.00
6043	seq=translation; coord=4:73093695..73100832:-1; parent_transcript=GRMZM2G031846_T01; parent_gene=GRMZM2G031846	GRMZM2G031846_P01,GRMZM2G031846_P02, GRMZM2G031846_P03,GRMZM2G031846_P04, GRMZM2G031846_P05	TRUE	TRUE	gFAFVTMDTLEGAR	95%	n+304 (+304)	63.74	25.00
6044	seq=translation; coord=4:5117703..5118736:1; parent_transcript=GRMZM2G044625_T01; parent_gene=GRMZM2G044625	GRMZM2G044625_P01,GRMZM2G088365_P01, GRMZM2G088441_P02,GRMZM2G346895_P01	TRUE	TRUE	qQPIVGGAIF	95%	n+304 (+304)	48.71	26.54
6045	seq=translation; coord=6:163005849..163012986:1; parent_transcript=GRMZM2G057251_T01; parent_gene=GRMZM2G057251	GRMZM2G057251_P01	TRUE	TRUE	sDLVAALPDR	86%	n+304 (+304)	26.45	26.24
6046	seq=translation; coord=9:30943220..30948556:1; parent_transcript=GRMZM2G058407_T01; parent_gene=GRMZM2G058407	GRMZM2G058407_P01,GRMZM2G348726_P01	TRUE	TRUE	vYVDSWDEFVER	95%	n+304 (+304)	74.32	25.00
6047	seq=translation; coord=2:233356514..233358701:1; parent_transcript=GRMZM2G080222_T01; parent_gene=GRMZM2G080222	GRMZM2G080222_P01,GRMZM2G080222_P02, GRMZM2G080222_P03,GRMZM2G102075_P01, GRMZM2G111172_P01,GRMZM2G111172_P02, GRMZM2G156110_P01,GRMZM2G156110_P02	TRUE	TRUE	nMIIVPEMIGSIVGVYNGk	95%	n+304 (+304), K+304 (+304)	54.92	25.00
6048	seq=translation; coord=7:136742676..136746945:1; parent_transcript=GRMZM2G083102_T01; parent_gene=GRMZM2G083102	GRMZM2G083102_P01,GRMZM2G083102_P02	TRUE	TRUE	iAPSMLSSDFANLASEAER	95%	n+304 (+304)	47.35	25.00
6049	seq=translation; coord=4:60172575..60178518:1; parent_transcript=GRMZM2G085573_T01; parent_gene=GRMZM2G085573	GRMZM2G085573_P01,GRMZM2G085573_P02, GRMZM2G146948_P01,GRMZM2G146948_P02	TRUE	TRUE	vAFDDILSILR	95%	n+304 (+304)	46.47	25.08

6050	seq=translation; coord=2:116778906..116783025:1; parent_transcript=GRMZM2G092160_T02; parent_gene=GRMZM2G092160	GRMZM2G092160_P02,GRMZM2G092160_P03	TRUE	TRUE	dMAEVFAVR	93%	n+304 (+304)	29.50	25.00
6051	seq=translation; coord=2:51230856..51233601:-1; parent_transcript=GRMZM2G131177_T01; parent_gene=GRMZM2G131177	GRMZM2G131177_P01	TRUE	TRUE	gITVSPDDYDSPR	95%	n+304 (+304)	63.32	25.00
6052	seq=translation; coord=9:28610042..28612570:1; parent_transcript=GRMZM2G154093_T01; parent_gene=GRMZM2G154093	GRMZM2G154093_P01,GRMZM2G154093_P02, GRMZM2G154093_P03,GRMZM2G154093_P04	TRUE	TRUE	lLVSPGEIVVIPQGLR	95%	n+304 (+304)	31.21	25.00
6053	seq=translation; coord=4:194515220..194519164:1; parent_transcript=GRMZM2G077253_T01; parent_gene=GRMZM2G077253	GRMZM2G077253_P01	TRUE	TRUE	dLSSVSQNQDPISVAQQFLEGFASR	95%	n+304 (+304)	75.79	25.00
6054	seq=translation; coord=6:70936697..70946158:1; parent_transcript=GRMZM2G080542_T01; parent_gene=GRMZM2G080542	GRMZM2G080542_P01	TRUE	TRUE	lSALAGWFDVHFR	95%	n+304 (+304)	44.42	25.00
6055	seq=translation; coord=2:18615584..18619086:-1; parent_transcript=GRMZM2G144153_T01; parent_gene=GRMZM2G144153	GRMZM2G144153_P01,GRMZM2G144153_P03	TRUE	TRUE	gGLFGDSIk	95%	n+304 (+304), K+304 (+304)	48.83	26.73
6056	seq=translation; coord=1:256085889..256086777:1; parent_transcript=GRMZM2G150688_T01; parent_gene=GRMZM2G150688	GRMZM2G150688_P01	TRUE	TRUE	dVNEcPcYR	93%	n+304 (+304), Carbamidomethyl (+57), Carbamidomethyl (+57)	29.85	25.00
6057	seq=translation; coord=10:114869410..114870649:1; parent_transcript=GRMZM2G045844_T01; parent_gene=GRMZM2G045844	GRMZM2G045844_P01,GRMZM5G800014_P01	TRUE	TRUE	fTHPADcTVDV	86%	n+304 (+304), Carbamidomethyl (+57)	25.07	25.00
6058	seq=translation; coord=4:12818434..12823201:-1; parent_transcript=GRMZM2G139441_T01; parent_gene=GRMZM2G139441	GRMZM2G139441_P01,GRMZM2G139441_P02	TRUE	TRUE	eEMIETYLQTLAk	95%	n+304 (+304), K+304 (+304)	31.79	26.05
6059	seq=translation; coord=4:12818434..12823201:-1; parent_transcript=GRMZM2G139441_T01; parent_gene=GRMZM2G139441	GRMZM2G139441_P01,GRMZM2G139441_P02	TRUE	TRUE	gYAPQQNYAQGGQDGR	95%	n+304 (+304)	43.45	25.00
6060	seq=translation; coord=6:114177100..114187293:-1; parent_transcript=GRMZM2G010960_T01; parent_gene=GRMZM2G010960	GRMZM2G010960_P01	TRUE	TRUE	gSEVSTEEMLELESNATk	95%	n+304 (+304), K+304 (+304)	45.07	25.00
6061	seq=translation; coord=6:114177100..114187293:-1; parent_transcript=GRMZM2G010960_T01; parent_gene=GRMZM2G010960	GRMZM2G010960_P01	TRUE	TRUE	lNkDQSELEGLSSGR	90%	n+304 (+304), K+304 (+304)	26.57	26.19
6062	seq=translation; coord=6:114177100..114187293:-1; parent_transcript=GRMZM2G010960_T01; parent_gene=GRMZM2G010960	GRMZM2G010960_P01	TRUE	TRUE	sLDNTTEVk	95%	n+304 (+304), K+304 (+304)	41.79	26.30
6063	seq=translation; coord=8:48994683..48999789:-1; parent_transcript=GRMZM2G083836_T01; parent_gene=GRMZM2G083836	GRMZM2G083836_P01,GRMZM2G083836_P03	TRUE	TRUE	kDDGSIAFENDFFk	95%	K+304 (+304), n+304 (+304), K+304 (+304)	31.43	25.35
6064	seq=translation; coord=8:48994683..48999789:-1; parent_transcript=GRMZM2G083836_T01; parent_gene=GRMZM2G083836	GRMZM2G083836_P01,GRMZM2G083836_P03	TRUE	TRUE	qEISTAVDELNR	95%	n+304 (+304)	47.12	25.00
6065	seq=translation; coord=8:48994683..48999789:-1; parent_transcript=GRMZM2G083836_T01; parent_gene=GRMZM2G083836	GRMZM2G083836_P01,GRMZM2G083836_P03	TRUE	TRUE	vGELIGGSQR	91%	n+304 (+304)	27.56	25.47
6066	seq=translation; coord=2:176238563..176244250:1; parent_transcript=GRMZM2G098076_T01; parent_gene=GRMZM2G098076	GRMZM2G098076_P01,GRMZM2G098076_P02	TRUE	TRUE	aYELLQELR	94%	n+304 (+304)	29.42	25.00
6067	seq=translation; coord=2:176238563..176244250:1; parent_transcript=GRMZM2G098076_T01; parent_gene=GRMZM2G098076	GRMZM2G098076_P01,GRMZM2G098076_P02	TRUE	TRUE	gMEVVGAEAAPAEIk	91%	n+304 (+304), K+304 (+304)	26.78	26.54

6068	seq=translation; coord=2:176238563..176244250:1; parent_transcript=GRMZM2G098076_T01; parent_gene=GRMZM2G098076	GRMZM2G098076_P01,GRMZM2G098076_P02	TRUE	TRUE	lVDAEIASLQEDLDAATAR	95%	n+304 (+304)	44.76	25.04
6069	seq=translation; coord=1:234254283..234259941:1; parent_transcript=GRMZM2G445905_T01; parent_gene=GRMZM2G445905	GRMZM2G445905_P01,GRMZM2G445905_P03	TRUE	TRUE	aYTFDTVEEFWGLYDHVFRPSk	95%	n+304 (+304), K+304 (+304)	30.87	25.00
6070	seq=translation; coord=1:234254283..234259941:1; parent_transcript=GRMZM2G445905_T01; parent_gene=GRMZM2G445905	GRMZM2G445905_P01,GRMZM2G445905_P03	TRUE	TRUE	iIYTFHDDSR	94%	n+304 (304)	27.09	25.00
6071	seq=translation; coord=3:217231435..217244458:-1; parent_transcript=AC198725.4_FGT007; parent_gene=AC198725.4_FG007	AC198725.4_FGP007	TRUE	TRUE	dGATAFFLVEcIVNTASAk	95%	n+304 (+304), Carbamidomethyl (+57), K+304 (+304)	29.00	25.65
6072	seq=translation; coord=3:217231435..217244458:-1; parent_transcript=AC198725.4_FGT007; parent_gene=AC198725.4_FG007	AC198725.4_FGP007	TRUE	TRUE	vPNLVEYLVTPLTTGLk	95%	n+304 (+304), K+304 (+304)	28.65	25.00
6073	seq=translation; coord=8:138088772..138094325:-1; parent_transcript=GRMZM2G018901_T01; parent_gene=GRMZM2G018901	GRMZM2G018901_P01,GRMZM2G018901_P02	TRUE	TRUE	gYEPVEFSFGTGWk	95%	n+304 (+304), K+304 (+304)	29.35	25.00
6074	seq=translation; coord=8:138088772..138094325:-1; parent_transcript=GRMZM2G018901_T01; parent_gene=GRMZM2G018901	GRMZM2G018901_P01,GRMZM2G018901_P02	TRUE	TRUE	sTYVTLSEQVDLPk	95%	n+304 (+304), K+304 (+304)	32.48	25.73
6075	seq=translation; coord=8:15548387..15551947:-1; parent_transcript=GRMZM2G038821_T01; parent_gene=GRMZM2G038821	GRMZM2G038821_P01	TRUE	TRUE	hPVPFFLSPR	95%	n+304 (+304)	34.83	25.92
6076	seq=translation; coord=8:15548387..15551947:-1; parent_transcript=GRMZM2G038821_T01; parent_gene=GRMZM2G038821	GRMZM2G038821_P01	TRUE	TRUE	lSNQMNLEAAVEAAAAFLNk	95%	n+304 (+304), K+304 (+304)	38.67	25.00
6077	seq=translation; coord=8:14541175..14542451:-1; parent_transcript=GRMZM2G052869_T02; parent_gene=GRMZM2G052869	GRMZM2G052869_P02,GRMZM2G052869_P03	TRUE	TRUE	cGGNcQcDPcNcGk	95%	Carbamidomethyl (+57), n+304 (+304), Carbamidomethyl (+57), Carbamidomethyl (+57), Carbamidomethyl (+57), Carbamidomethyl (+57), K+304 (+304)	42.45	25.00
6078	seq=translation; coord=8:14541175..14542451:-1; parent_transcript=GRMZM2G052869_T02; parent_gene=GRMZM2G052869	GRMZM2G052869_P02,GRMZM2G052869_P03	TRUE	TRUE	mYPDMAEQVTTTTQALIMGVAPSk	95%	n+304 (+304), K+304 (+304)	45.35	25.09
6079	seq=translation; coord=2:208530771..208535295:-1; parent_transcript=GRMZM2G090274_T01; parent_gene=GRMZM2G090274	GRMZM2G090274_P01,GRMZM2G090274_P02	TRUE	TRUE	mSLLEWFANEYk	95%	n+304 (+304), K+304 (+304)	28.86	25.68
6080	seq=translation; coord=2:208530771..208535295:-1; parent_transcript=GRMZM2G090274_T01; parent_gene=GRMZM2G090274	GRMZM2G090274_P01,GRMZM2G090274_P02	TRUE	TRUE	tELSQSDMFDQR	93%	n+304 (+304)	29.27	25.00
6081	seq=translation; coord=4:187295953..187301881:-1; parent_transcript=GRMZM2G055538_T01; parent_gene=GRMZM2G055538	GRMZM2G055538_P01,GRMZM2G055538_P02, GRMZM2G055538_P03	TRUE	TRUE	dALcQELLQAR	95%	n+304 (+304), Carbamidomethyl (+57)	30.87	25.75
6082	seq=translation; coord=4:187295953..187301881:-1; parent_transcript=GRMZM2G055538_T01; parent_gene=GRMZM2G055538	GRMZM2G055538_P01,GRMZM2G055538_P02, GRMZM2G055538_P03	TRUE	TRUE	lADEIVSLk	95%	n+304 (+304), K+304 (+304)	31.50	25.00
6083	seq=translation; coord=9:115327106..115330655:-1; parent_transcript=GRMZM2G119146_T01; parent_gene=GRMZM2G119146	GRMZM2G119146_P01,GRMZM2G180909_P01	TRUE	TRUE	rWPGWPGDSVFR	95%	n+304 (+304)	31.01	25.00

6084	seq=translation; coord=8:135950853..135955026:1; parent_transcript=GRMZM2G176707_T01; parent_gene=GRMZM2G176707	GRMZM2G176707_P01	TRUE	TRUE	lEFQFGTNPFFk	95%	n+304 (+304), K+304 (+304)	40.03	26.06
6085	seq=translation; coord=10:110956920..110958900:1; parent_transcript=GRMZM2G002921_T01; parent_gene=GRMZM2G002921	GRMZM2G002921_P01,GRMZM2G032878_P01, GRMZM2G032878_P02,GRMZM2G032878_P03	TRUE	TRUE	qLALPEGTEVcGSSk	95%	n+304 (+304), Carbamidomethyl (+57), K+304 (+304)	43.67	25.00
6086	seq=translation; coord=8:132520764..132524680:1; parent_transcript=GRMZM2G474658_T03; parent_gene=GRMZM2G474658	GRMZM2G474658_P03	TRUE	TRUE	dLEHLFGR	95%	n+304 (+304)	41.95	25.07
6087	seq=translation; coord=1:250554600..250557666:-1; parent_transcript=GRMZM2G020142_T01; parent_gene=GRMZM2G020142	GRMZM2G020142_P01,GRMZM5G895064_P01	TRUE	TRUE	lLFQELMAMAIAPR	95%	n+304 (+304)	37.90	25.26
6088	seq=translation; coord=1:250554600..250557666:-1; parent_transcript=GRMZM2G020142_T01; parent_gene=GRMZM2G020142	GRMZM2G020142_P01,GRMZM5G895064_P01	TRUE	TRUE	mTIGQLIEcIMGk	95%	n+304 (+304), Carbamidomethyl (+57), K+304 (+304)	29.86	26.07
6089	seq=translation; coord=7:170807791..170818840:1; parent_transcript=GRMZM2G152963_T01; parent_gene=GRMZM2G152963	GRMZM2G152963_P01,GRMZM2G152963_P02	TRUE	TRUE	iYVDLLQDLMEk	95%	n+304 (+304), K+304 (+304)	51.21	25.65
6090	seq=translation; coord=7:170807791..170818840:1; parent_transcript=GRMZM2G152963_T01; parent_gene=GRMZM2G152963	GRMZM2G152963_P01,GRMZM2G152963_P02	TRUE	TRUE	lDFQSDSPDLPLLAAAPSPDR	94%	n+304 (+304)	28.84	25.17
6091	seq=translation; coord=6:24318884..24321111:-1; parent_transcript=GRMZM2G119583_T01; parent_gene=GRMZM2G119583	GRMZM2G119583_P01,GRMZM2G119583_P02	TRUE	TRUE	aFFASTGTEANEAAIk	95%	n+304 (+304), K+304 (+304)	48.27	25.34
6092	seq=translation; coord=6:24318884..24321111:-1; parent_transcript=GRMZM2G119583_T01; parent_gene=GRMZM2G119583	GRMZM2G119583_P01,GRMZM2G119583_P02	TRUE	TRUE	lVEVSFADR	87%	n+304 (+304)	25.08	25.00
6093	seq=translation; coord=5:213944858..213947602:-1; parent_transcript=GRMZM2G469111_T01; parent_gene=GRMZM2G469111	GRMZM2G469111_P01	TRUE	TRUE	dSAENLGELLMGDR	95%	n+304 (+304)	64.96	25.00
6094	seq=translation; coord=5:213944858..213947602:-1; parent_transcript=GRMZM2G469111_T01; parent_gene=GRMZM2G469111	GRMZM2G469111_P01	TRUE	TRUE	sPPLGADAFSLLk	90%	n+304 (+304), K+304 (+304)	25.84	25.48
6095	seq=translation; coord=5:76210306..76210828:1; parent_transcript=AC192244.3_FGT001; parent_gene=AC192244.3_FG001	AC192244.3_FGP001	TRUE	TRUE	lFQVEYAMEAVk	95%	n+304 (+304), K+304 (+304)	62.55	25.11
6096	seq=translation; coord=5:76210306..76210828:1; parent_transcript=AC192244.3_FGT001; parent_gene=AC192244.3_FG001	AC192244.3_FGP001	TRUE	TRUE	nEcINHSFVYEAPLPVSR	95%	n+304 (+304), Carbamidomethyl (+57)	36.48	25.00
6097	seq=translation; coord=5:76210306..76210828:1; parent_transcript=AC192244.3_FGT001; parent_gene=AC192244.3_FG001	AC192244.3_FGP001	TRUE	TRUE	vADHAGVALAGLTADGR	95%	n+304 (+304)	55.66	25.92
6098	seq=translation; coord=6:87790147..87797085:1; parent_transcript=GRMZM2G156099_T01; parent_gene=GRMZM2G156099	GRMZM2G156099_P01,GRMZM2G156099_P02, GRMZM5G817439_P01	TRUE	TRUE	dkIDAAAAEVLEPLVR	95%	n+304 (+304), K+304 (+304)	44.14	25.34
6099	seq=translation; coord=6:87790147..87797085:1; parent_transcript=GRMZM2G156099_T01; parent_gene=GRMZM2G156099	GRMZM2G156099_P01,GRMZM2G156099_P02, GRMZM5G817439_P01	TRUE	TRUE	vQADIEQTLALVR	95%	n+304 (+304)	48.61	26.45
6100	seq=translation; coord=7:159777544..159783160:1; parent_transcript=GRMZM2G061156_T01; parent_gene=GRMZM2G061156	GRMZM2G061156_P01,GRMZM2G061156_P02, GRMZM2G061156_P03	TRUE	TRUE	iALTAEDNAPLVR	95%	n+304 (+304)	41.86	25.76
6101	seq=translation; coord=6:22918021..22921799:-1; parent_transcript=GRMZM2G143788_T01; parent_gene=GRMZM2G143788	GRMZM2G143788_P01	TRUE	TRUE	eFVGGAAELPR	95%	n+304 (+304)	40.78	25.49

6102	seq=translation; coord=6:22918021..22921799:-1; parent_transcript=GRMZM2G143788_T01; parent_gene=GRMZM2G143788	GRMZM2G143788_P01	TRUE	TRUE	lVWAHIk	95%	n+304 (+304), K+304 (+304)	32.11	25.33
6103	seq=translation; coord=1:51013925..51015964:1; parent_transcript=GRMZM2G018941_T01; parent_gene=GRMZM2G018941	GRMZM2G018941_P01,GRMZM2G018941_P02	TRUE	TRUE	aVPEDEGYR	92%	n+304 (+304)	28.66	25.00
6104	seq=translation; coord=1:51013925..51015964:1; parent_transcript=GRMZM2G018941_T01; parent_gene=GRMZM2G018941	GRMZM2G018941_P01,GRMZM2G018941_P02	TRUE	TRUE	eVLVGLYER	95%	n+304 (+304)	34.19	25.00
6105	seq=translation; coord=6:163428972..163435944:-1; parent_transcript=GRMZM2G149717_T01; parent_gene=GRMZM2G149717	GRMZM2G149717_P01,GRMZM2G314647_P01	TRUE	TRUE	fTDFAAVR	94%	n+304 (+304)	30.36	25.00
6106	seq=translation; coord=6:163428972..163435944:-1; parent_transcript=GRMZM2G149717_T01; parent_gene=GRMZM2G149717	GRMZM2G149717_P01,GRMZM2G314647_P01	TRUE	TRUE	gTDAVDILEGR	95%	n+304 (+304)	34.75	25.00
6107	seq=translation; coord=6:163428972..163435944:-1; parent_transcript=GRMZM2G149717_T01; parent_gene=GRMZM2G149717	GRMZM2G149717_P01,GRMZM2G314647_P01	TRUE	TRUE	sLLDHFFTELGAR	93%	n+304 (+304)	26.22	25.00
6108	seq=translation; coord=6:161877489..161879473:-1; parent_transcript=GRMZM2G394827_T01; parent_gene=GRMZM2G394827	GRMZM2G394827_P01,GRMZM2G394827_P02, GRMZM2G394827_P03,GRMZM2G394827_P04, GRMZM2G394827_P05,GRMZM2G394827_P06, GRMZM2G394827_P07,GRMZM2G394827_P08	TRUE	TRUE	ePNNLYNIHSFk	94%	n+304 (+304), K+304 (+304)	28.58	25.98
6109	seq=translation; coord=1:107847003..107851828:-1; parent_transcript=GRMZM2G179528_T01; parent_gene=GRMZM2G179528	GRMZM2G179528_P01,GRMZM2G179528_P02, GRMZM2G179528_P03	TRUE	TRUE	lSYIWADDPEVR	95%	n+304 (+304)	42.02	25.00
6110	seq=translation; coord=4:53963570..53966108:-1; parent_transcript=GRMZM2G123234_T01; parent_gene=GRMZM2G123234	GRMZM2G123234_P01	TRUE	TRUE	aYVGNLPWQVDDSR	95%	n+304 (+304)	57.76	25.00
6111	seq=translation; coord=4:53963570..53966108:-1; parent_transcript=GRMZM2G123234_T01; parent_gene=GRMZM2G123234	GRMZM2G123234_P01	TRUE	TRUE	gFGFVTMSTVEEADk	91%	n+304 (+304), K+304 (+304)	25.51	25.00
6112	seq=translation; coord=2:209110460..209113041:-1; parent_transcript=GRMZM2G378106_T01; parent_gene=GRMZM2G378106	GRMZM2G378106_P01,GRMZM2G378106_P02	TRUE	TRUE	aLHFLFVk	94%	n+304 (+304), K+304 (+304)	27.95	25.00
6113	seq=translation; coord=2:209110460..209113041:-1; parent_transcript=GRMZM2G378106_T01; parent_gene=GRMZM2G378106	GRMZM2G378106_P01,GRMZM2G378106_P02	TRUE	TRUE	vVRPGTFEELMDYAISR	95%	n+304 (+304)	31.67	25.01
6114	seq=translation; coord=10:125125070..125130397:1; parent_transcript=GRMZM2G065292_T01; parent_gene=GRMZM2G065292	GRMZM2G065292_P01	TRUE	TRUE	lDETHDAYEALLHEAESR	95%	n+304 (+304)	41.07	25.00
6115	seq=translation; coord=10:125125070..125130397:1; parent_transcript=GRMZM2G065292_T01; parent_gene=GRMZM2G065292	GRMZM2G065292_P01	TRUE	TRUE	lSGLEILNLSSNFSDLk	95%	n+304 (+304), K+304 (+304)	41.39	25.25
6116	seq=translation; coord=7:171699375..171703593:1; parent_transcript=GRMZM2G136803_T01; parent_gene=GRMZM2G136803	GRMZM2G136803_P01,GRMZM2G136803_P02	TRUE	TRUE	iDHILGFFR	95%	n+304 (+304)	35.43	26.31
6117	seq=translation; coord=6:112003443..112007827:-1; parent_transcript=GRMZM2G073700_T01; parent_gene=GRMZM2G073700	GRMZM2G073700_P01	TRUE	TRUE	vFVGGLAWETPSEGLR	95%	n+304 (+304)	67.22	25.62
6118	seq=translation; coord=6:112003443..112007827:-1; parent_transcript=GRMZM2G073700_T01; parent_gene=GRMZM2G073700	GRMZM2G073700_P01	TRUE	TRUE	yGDILEAVVITDR	95%	n+304 (+304)	30.24	25.00

6119	seq=translation; coord=7:162255747..162259720:1; parent_transcript=GRMZM2G167932_T01; parent_gene=GRMZM2G167932	GRMZM2G167932_P01,GRMZM2G167932_P02	TRUE	TRUE	eTGGYYAFVEFEELSGVHNALk	95%	n+304 (+304), K+304 (+304)	68.26	25.00
6120	seq=translation; coord=7:162255747..162259720:1; parent_transcript=GRMZM2G167932_T01; parent_gene=GRMZM2G167932	GRMZM2G167932_P01,GRMZM2G167932_P02	TRUE	TRUE	fIQMFFLAPQEk	95%	n+304 (+304), K+304 (+304)	40.70	25.00
6121	seq=translation; coord=7:162255747..162259720:1; parent_transcript=GRMZM2G167932_T01; parent_gene=GRMZM2G167932	GRMZM2G167932_P01,GRMZM2G167932_P02	TRUE	TRUE	sDVFGEGASHDDEESk	95%	n+304 (+304), K+304 (+304)	46.98	25.00
6122	seq=translation; coord=4:162903711..162910128:1; parent_transcript=GRMZM2G453424_T03; parent_gene=GRMZM2G453424	GRMZM2G453424_P03,GRMZM2G453424_P04, GRMZM2G453424_P05,GRMZM2G453424_P06, GRMZM2G453424_P07	TRUE	TRUE	iPESFDk	93%	n+304 (+304), K+304 (+304)	27.04	25.62
6123	seq=translation; coord=4:162903711..162910128:1; parent_transcript=GRMZM2G453424_T03; parent_gene=GRMZM2G453424	GRMZM2G453424_P03,GRMZM2G453424_P04, GRMZM2G453424_P05,GRMZM2G453424_P06, GRMZM2G453424_P07	TRUE	TRUE	mLMNcEAAELLQEIHEHMAILSEDPk	95%	n+304 (+304), Carbamidomethyl (+57), K+304 (+304)	28.07	25.00
6124	seq=translation; coord=9:152490486..152495067:-1; parent_transcript=AC149475.2_FGT003; parent_gene=AC149475.2_FG003	AC149475.2_FGP003	TRUE	TRUE	eLALEEAETIALSILk	93%	n+304 (+304), K+304 (+304)	27.16	25.00
6125	seq=translation; coord=2:21007540..21010895:1; parent_transcript=AC212835.3_FGT007; parent_gene=AC212835.3_FG007	AC212835.3_FGP007	TRUE	TRUE	gLVHVAIDAVEDAVR	95%	n+304 (+304)	57.64	25.00
6126	seq=translation; coord=1:81094071..81098200:1; parent_transcript=AC212979.2_FGT006; parent_gene=AC212979.2_FG006	AC212979.2_FGP006,GRMZM2G120973_P01,GRMZM2G120973_P02,GRMZM2G120973_P03,GRMZM2G120973_P04,GRMZM5G871419_P03,GRMZM5G871419_P04,GRMZM5G871419_P05	TRUE	TRUE	iLcASELIGSVIGk	95%	n+304 (+304), Carbamidomethyl (+57), K+304 (+304)	34.60	25.24
6127	seq=translation; coord=5:27393255..27396313:1; parent_transcript=GRMZM2G001887_T01; parent_gene=GRMZM2G001887	GRMZM2G001887_P01,GRMZM2G001887_P03	TRUE	TRUE	iGVNSIPGIEEVNIFk	95%	n+304 (+304), K+304 (+304)	40.24	25.00
6128	seq=translation; coord=4:105098023..105100902:1; parent_transcript=GRMZM2G010743_T01; parent_gene=GRMZM2G010743	GRMZM2G010743_P01,GRMZM2G473463_P01, GRMZM2G473463_P03	TRUE	TRUE	lLILDQIk	95%	n+304 (+304), K+304 (+304)	29.76	25.00
6129	seq=translation; coord=9:7186959..7210629:-1; parent_transcript=GRMZM2G017257_T01; parent_gene=GRMZM2G017257	GRMZM2G017257_P01,GRMZM2G017257_P02	TRUE	TRUE	gVFEQLPEAVSLLALAR	95%	n+304 (+304)	32.43	25.00
6130	seq=translation; coord=8:154154493..154157871:1; parent_transcript=GRMZM2G023667_T02; parent_gene=GRMZM2G023667	GRMZM2G023667_P02	TRUE	TRUE	tPLQQLMNDALIVAk	95%	n+304 (+304), K+304 (+304)	46.49	25.02
6131	seq=translation; coord=7:121981747..121986892:1; parent_transcript=GRMZM2G038636_T01; parent_gene=GRMZM2G038636	GRMZM2G038636_P01	TRUE	TRUE	dAVGLVRPVSVAFEVITGFR	95%	n+304 (+304)	32.09	25.00
6132	seq=translation; coord=8:113064531..113068162:-1; parent_transcript=GRMZM2G044775_T02; parent_gene=GRMZM2G044775	GRMZM2G044775_P02,GRMZM2G044775_P03, GRMZM2G044775_P04	TRUE	TRUE	aEVEDIFDAPLEMFLk	95%	n+304 (+304), K+304 (+304)	51.10	25.39
6133	seq=translation; coord=6:151440759..151444649:1; parent_transcript=GRMZM2G059121_T01; parent_gene=GRMZM2G059121	GRMZM2G059121_P01	TRUE	TRUE	aGLALAELASSVLPATk	95%	n+304 (+304), K+304 (+304)	32.82	25.00
6134	seq=translation; coord=9:27092199..27094638:1; parent_transcript=GRMZM2G084739_T01; parent_gene=GRMZM2G084739	GRMZM2G084739_P01	TRUE	TRUE	hLNLDFQLQEAGR	95%	n+304 (+304)	60.75	25.01
6135	seq=translation; coord=1:222151651..222155503:-1; parent_transcript=GRMZM2G096303_T02; parent_gene=GRMZM2G096303	GRMZM2G096303_P02	TRUE	TRUE	iSTADEWAEFQR	94%	n+304 (+304)	31.17	25.00

6136	seq=translation; coord=1:222151651..222155503:-1; parent_transcript=GRMZM2G096303_T02; parent_gene=GRMZM2G096303	GRMZM2G096303_P02	TRUE	TRUE	lADGLIYELSDGDNYFPHFyGPGR	95%	n+304 (+304), iTRAQ8plex (+304)	31.01	25.00
6137	seq=translation; coord=5:123087268..123104980:1; parent_transcript=GRMZM2G145376_T01; parent_gene=GRMZM2G145376	GRMZM2G145376_P01,GRMZM2G145376_P02	TRUE	TRUE	lIFPEGMLAQTLAQLk	95%	n+304 (+304), K+304 (+304)	46.54	25.00
6138	seq=translation; coord=4:25788335..25797883:-1; parent_transcript=GRMZM2G147726_T02; parent_gene=GRMZM2G147726	GRMZM2G147726_P02,GRMZM2G147726_P03	TRUE	TRUE	gEGLFEGLDWLSNTLk	95%	n+304 (+304), K+304 (+304)	36.31	25.21
6139	seq=translation; coord=2:12677824..12680299:-1; parent_transcript=GRMZM2G149649_T01; parent_gene=GRMZM2G149649	GRMZM2G149649_P01	TRUE	TRUE	hSGNISLDDVIEIAk	95%	n+304 (+304), K+304 (+304)	28.28	25.01
6140	seq=translation; coord=9:45186311..45202040:1; parent_transcript=GRMZM2G151414_T01; parent_gene=GRMZM2G151414	GRMZM2G151414_P01,GRMZM2G151414_P02	TRUE	TRUE	aMSLFEGVIk	95%	n+304 (+304), K+304 (+304)	35.01	25.55
6141	seq=translation; coord=6:136515921..136519080:-1; parent_transcript=GRMZM2G154218_T01; parent_gene=GRMZM2G154218	GRMZM2G154218_P01,GRMZM2G154218_P02	TRUE	TRUE	mIPTkPMVVETFSAYPPLGR	95%	n+304 (+304), K+304 (+304)	35.17	25.00
6142	seq=translation; coord=10:132962163..132965961:-1; parent_transcript=GRMZM2G155543_T01; parent_gene=GRMZM2G155543	GRMZM2G155543_P01,GRMZM2G155543_P02, GRMZM2G155543_P03	TRUE	TRUE	dLLEIEPLELR	95%	n+304 (+304)	40.32	25.80
6143	seq=translation; coord=5:214842953..214846166:1; parent_transcript=GRMZM2G168335_T01; parent_gene=GRMZM2G168335	GRMZM2G168335_P01	TRUE	TRUE	fQPVINADLDTDDDRk	95%	n+304 (+304), K+304 (+304)	33.42	25.00
6144	seq=translation; coord=5:214842953..214846166:1; parent_transcript=GRMZM2G168335_T01; parent_gene=GRMZM2G168335	GRMZM2G168335_P01	TRUE	TRUE	gSVDSLLQLLR	95%	n+304 (+304)	40.37	25.00
6145	seq=translation; coord=2:202916878..202922719:1; parent_transcript=GRMZM2G171707_T01; parent_gene=GRMZM2G171707	GRMZM2G171707_P01	TRUE	TRUE	dGVTATDLVLTVTHILR	95%	n+304 (+304)	53.67	25.28
6146	seq=translation; coord=2:202916878..202922719:1; parent_transcript=GRMZM2G171707_T01; parent_gene=GRMZM2G171707	GRMZM2G171707_P01	TRUE	TRUE	vLLQDFTGVPAIVDLASMR	95%	n+304 (+304)	50.05	25.51
6147	seq=translation; coord=1:287334203..287336597:-1; parent_transcript=GRMZM2G178341_T01; parent_gene=GRMZM2G178341	GRMZM2G178341_P01	TRUE	TRUE	aGELTPEELER	95%	n+304 (+304)	37.70	25.00
6148	seq=translation; coord=5:892571..893531:1; parent_transcript=GRMZM2G384338_T01; parent_gene=GRMZM2G384338	GRMZM2G384338_P01	TRUE	TRUE	aLAVLGLAAAESAAALR	95%	n+304 (+304)	37.34	25.00
6149	seq=translation; coord=5:182450024..182455917:1; parent_transcript=GRMZM2G095194_T01; parent_gene=GRMZM2G095194	GRMZM2G095194_P01	TRUE	TRUE	fTDIADLTTSLSTLk	94%	n+304 (+304), K+304 (+304)	29.17	26.22
6150	seq=translation; coord=5:182450024..182455917:1; parent_transcript=GRMZM2G095194_T01; parent_gene=GRMZM2G095194	GRMZM2G095194_P01	TRUE	TRUE	mMPNEIQFFDPk	95%	n+304 (+304), K+304 (+304)	28.18	25.00
6151	seq=translation; coord=2:225806982..225808719:-1; parent_transcript=GRMZM2G372068_T01; parent_gene=GRMZM2G372068	GRMZM2G372068_P01	TRUE	TRUE	sLPAIAAVLAELGETAR	95%	n+304 (+304)	30.72	25.00
6152	seq=translation; coord=7:168403976..168405995:-1; parent_transcript=GRMZM2G082365_T01; parent_gene=GRMZM2G082365	GRMZM2G082365_P01,GRMZM2G172956_P02	TRUE	TRUE	gEAIAIGIAEMTTAVMATcDHGAVAk	95%	n+304 (+304), Carbamidomethyl (+57), K+304 (+304)	26.45	25.00
6153	seq=translation; coord=9:79713849..79717604:-1; parent_transcript=GRMZM2G059580_T02; parent_gene=GRMZM2G059580	GRMZM2G059580_P02	TRUE	TRUE	aLIAAEYSGVk	94%	n+304 (+304), K+304 (+304)	30.76	25.48

6154	seq=translation; coord=5:183319186..183326822:-1; parent_transcript=GRMZM2G014750_T01; parent_gene=GRMZM2G014750	GRMZM2G014750_P01,GRMZM2G014750_P02	TRUE	TRUE	dHIVTLEPVTGEAEk	95%	n+304 (+304), K+304 (+304)	35.23	25.30
6155	seq=translation; coord=5:183319186..183326822:-1; parent_transcript=GRMZM2G014750_T01; parent_gene=GRMZM2G014750	GRMZM2G014750_P01,GRMZM2G014750_P02	TRUE	TRUE	iMETALVFAGANLEIRPk	95%	n+304 (+304), K+304 (+304)	31.87	25.00
6156	seq=translation; coord=5:183319186..183326822:-1; parent_transcript=GRMZM2G014750_T01; parent_gene=GRMZM2G014750	GRMZM2G014750_P01,GRMZM2G014750_P02	TRUE	TRUE	mSAVLADGGLIIk	95%	n+304 (+304), K+304 (+304)	55.24	25.00
6157	seq=translation; coord=5:183319186..183326822:-1; parent_transcript=GRMZM2G014750_T01; parent_gene=GRMZM2G014750	GRMZM2G014750_P01,GRMZM2G014750_P02	TRUE	TRUE	tIAVSPLPcNVk	95%	n+304 (+304), Carbamidomethyl (+57), K+304 (+304)	31.87	25.73
6158	seq=translation; coord=9:131582315..131589080:-1; parent_transcript=GRMZM2G006178_T01; parent_gene=GRMZM2G006178	GRMZM2G006178_P01,GRMZM2G109121_P01, GRMZM2G109121_P02,GRMZM2G109121_P03	TRUE	TRUE	fkNPPDWQEILTYFR	95%	n+304 (+304), K+304 (+304)	28.67	25.47
6159	seq=translation; coord=9:131582315..131589080:-1; parent_transcript=GRMZM2G006178_T01; parent_gene=GRMZM2G006178	GRMZM2G006178_P01,GRMZM2G109121_P01, GRMZM2G109121_P02,GRMZM2G109121_P03	TRUE	TRUE	fLSHLDITFR	95%	n+304 (+304)	47.11	25.00
6160	seq=translation; coord=9:131582315..131589080:-1; parent_transcript=GRMZM2G006178_T01; parent_gene=GRMZM2G006178	GRMZM2G006178_P01,GRMZM2G109121_P01, GRMZM2G109121_P02,GRMZM2G109121_P03	TRUE	TRUE	nVGDLSGGELQR	95%	n+304 (+304)	58.84	25.00
6161	seq=translation; coord=5:18992634..18995244:-1; parent_transcript=GRMZM2G045192_T01; parent_gene=GRMZM2G045192	GRMZM2G045192_P01,GRMZM2G045192_P02, GRMZM2G045192_P03	TRUE	TRUE	gLGLEYDMSGLTGDTMDSHR	95%	n+304 (+304)	85.61	25.00
6162	seq=translation; coord=5:18992634..18995244:-1; parent_transcript=GRMZM2G045192_T01; parent_gene=GRMZM2G045192	GRMZM2G045192_P01,GRMZM2G045192_P02, GRMZM2G045192_P03	TRUE	TRUE	gVDEVQEELkk	95%	n+304 (+304), K+304 (+304), K+304 (+304)	26.83	25.00
6163	seq=translation; coord=5:18992634..18995244:-1; parent_transcript=GRMZM2G045192_T01; parent_gene=GRMZM2G045192	GRMZM2G045192_P01,GRMZM2G045192_P02, GRMZM2G045192_P03	TRUE	TRUE	qNALVGELFLNYFcEGk	95%	n+304 (+304), Carbamidomethyl (+57), K+304 (+304)	72.47	25.43
6164	seq=translation; coord=9:30842366..30844467:1; parent_transcript=GRMZM2G060163_T01; parent_gene=GRMZM2G060163	GRMZM2G060163_P01	TRUE	TRUE	eIVADAVDIER	95%	n+304 (+304)	67.02	25.69
6165	seq=translation; coord=9:30842366..30844467:1; parent_transcript=GRMZM2G060163_T01; parent_gene=GRMZM2G060163	GRMZM2G060163_P01	TRUE	TRUE	fPQIWEFYk	95%	n+304 (+304), K+304 (+304)	28.23	25.45
6166	seq=translation; coord=9:30842366..30844467:1; parent_transcript=GRMZM2G060163_T01; parent_gene=GRMZM2G060163	GRMZM2G060163_P01	TRUE	TRUE	lLMALGck	95%	n+304 (+304), Carbamidomethyl (+57), K+304 (+304)	34.89	25.44
6167	seq=translation; coord=6:149942002..149944565:-1; parent_transcript=GRMZM2G161534_T01; parent_gene=GRMZM2G161534	GRMZM2G161534_P01	TRUE	TRUE	aLQQLLMSLGFk	95%	n+304 (+304), K+304 (+304)	45.07	25.00
6168	seq=translation; coord=6:149942002..149944565:-1; parent_transcript=GRMZM2G161534_T01; parent_gene=GRMZM2G161534	GRMZM2G161534_P01	TRUE	TRUE	lHEEMEVLDELLGk	95%	n+304 (+304), K+304 (+304)	27.13	26.09
6169	seq=translation; coord=6:149942002..149944565:-1; parent_transcript=GRMZM2G161534_T01; parent_gene=GRMZM2G161534	GRMZM2G161534_P01	TRUE	TRUE	wAESHDWTLEk	95%	n+304 (+304), K+304 (+304)	28.52	25.17
6170	seq=translation; coord=1:295450010..295457140:-1; parent_transcript=GRMZM2G458549_T01; parent_gene=GRMZM2G458549	GRMZM2G458549_P01,GRMZM2G458549_P02	TRUE	TRUE	ePIVPQFILk	95%	n+304 (+304), K+304 (+304)	29.87	25.00
6171	seq=translation; coord=1:295450010..295457140:-1; parent_transcript=GRMZM2G458549_T01; parent_gene=GRMZM2G458549	GRMZM2G458549_P01,GRMZM2G458549_P02	TRUE	TRUE	kEVAQFTcLSER	92%	K+304 (+304), n+304 (+304), Carbamidomethyl (+57)	27.02	25.50

6172	seq=translation; coord=1:295450010..295457140:-1; parent_transcript=GRMZM2G458549_T01; parent_gene=GRMZM2G458549	GRMZM2G458549_P01,GRMZM2G458549_P02	TRUE	TRUE	vNLIGEHIDYEGYSVLPMAIR	95%	n+304 (+304)	46.67	25.38
6173	seq=translation; coord=2:28117445..28120590:1; parent_transcript=GRMZM2G441347_T01; parent_gene=GRMZM2G441347	GRMZM2G441347_P01	TRUE	TRUE	aAGIEGGFFk	95%	n+304 (+304), K+304 (+304)	45.64	25.63
6174	seq=translation; coord=2:28117445..28120590:1; parent_transcript=GRMZM2G441347_T01; parent_gene=GRMZM2G441347	GRMZM2G441347_P01	TRUE	TRUE	aLLTAIDR	93%	n+304 (+304)	29.99	25.00
6175	seq=translation; coord=2:28117445..28120590:1; parent_transcript=GRMZM2G441347_T01; parent_gene=GRMZM2G441347	GRMZM2G441347_P01	TRUE	TRUE	dGAALQVELLR	95%	n+304 (+304)	36.99	26.91
6176	seq=translation; coord=6:164983203..164994562:1; parent_transcript=GRMZM2G101515_T01; parent_gene=GRMZM2G101515	GRMZM2G101515_P01	TRUE	TRUE	ecGMDPDETVSR	90%	n+304 (+304), Carbamidomethyl (+57)	26.44	25.00
6177	seq=translation; coord=6:164983203..164994562:1; parent_transcript=GRMZM2G101515_T01; parent_gene=GRMZM2G101515	GRMZM2G101515_P01	TRUE	TRUE	eESAPVDQIDAR	95%	n+304 (+304)	47.56	25.00
6178	seq=translation; coord=8:173177746..173180822:1; parent_transcript=GRMZM2G074028_T01; parent_gene=GRMZM2G074028	GRMZM2G074028_P01,GRMZM2G074028_P02, GRMZM2G074028_P03,GRMZM2G074028_P05, GRMZM5G898597_P01	TRUE	TRUE	aIVPTSDPETVYDIk	94%	n+304 (+304), K+304 (+304)	29.37	26.12
6179	seq=translation; coord=8:173177746..173180822:1; parent_transcript=GRMZM2G074028_T01; parent_gene=GRMZM2G074028	GRMZM2G074028_P01,GRMZM2G074028_P02, GRMZM2G074028_P03,GRMZM2G074028_P05, GRMZM5G898597_P01	TRUE	TRUE	kPWEITGPcASPEYR	95%	K+304 (+304), n+304 (+304), Carbamidomethyl (+57)	55.85	25.54
6180	seq=translation; coord=2:22504092..22513426:-1; parent_transcript=GRMZM2G070863_T01; parent_gene=GRMZM2G070863	GRMZM2G070863_P01	TRUE	TRUE	eLEQQEYYDPR	95%	n+304 (+304)	59.43	25.00
6181	seq=translation; coord=2:22504092..22513426:-1; parent_transcript=GRMZM2G070863_T01; parent_gene=GRMZM2G070863	GRMZM2G070863_P01	TRUE	TRUE	gLALEPSNEALk	95%	n+304 (+304), K+304 (+304)	38.61	25.58
6182	seq=translation; coord=7:13851997..13853595:1; parent_transcript=GRMZM2G108076_T01; parent_gene=GRMZM2G108076	GRMZM2G108076_P01	TRUE	TRUE	aESLEDAIQIINR	95%	n+304 (+304)	44.82	26.22
6183	seq=translation; coord=7:13851997..13853595:1; parent_transcript=GRMZM2G108076_T01; parent_gene=GRMZM2G108076	GRMZM2G108076_P01	TRUE	TRUE	aSFAGDLNFYGk	95%	n+304 (+304), K+304 (+304)	34.30	25.00
6184	seq=translation; coord=3:36465309..36469955:-1; parent_transcript=GRMZM2G038606_T01; parent_gene=GRMZM2G038606	GRMZM2G038606_P01,GRMZM2G038606_P02, GRMZM2G038606_P04,GRMZM2G095114_P01	TRUE	TRUE	mLPLSLLk	95%	n+304 (+304), K+304 (+304)	35.33	25.00
6185	seq=translation; coord=3:36465309..36469955:-1; parent_transcript=GRMZM2G038606_T01; parent_gene=GRMZM2G038606	GRMZM2G038606_P01,GRMZM2G038606_P02, GRMZM2G038606_P04,GRMZM2G095114_P01	TRUE	TRUE	vPDEVIDk	95%	n+304 (+304), K+304 (+304)	34.10	25.00
6186	seq=translation; coord=3:198588979..198593726:-1; parent_transcript=GRMZM2G042818_T01; parent_gene=GRMZM2G042818	GRMZM2G042818_P01	TRUE	TRUE	lPGYLGEYLALTGAR	95%	n+304 (+304)	43.67	25.56
6187	seq=translation; coord=3:198588979..198593726:-1; parent_transcript=GRMZM2G042818_T01; parent_gene=GRMZM2G042818	GRMZM2G042818_P01	TRUE	TRUE	vVYLLAQFLEk	95%	n+304 (+304), K+304 (+304)	32.03	25.00
6188	seq=translation; coord=2:196329725..196336472:-1; parent_transcript=GRMZM2G050193_T01; parent_gene=GRMZM2G050193	GRMZM2G050193_P01,GRMZM2G050193_P02	TRUE	TRUE	lHVEVISk	95%	n+304 (+304), K+304 (+304)	36.02	25.00
6189	seq=translation; coord=2:196329725..196336472:-1; parent_transcript=GRMZM2G050193_T01; parent_gene=GRMZM2G050193	GRMZM2G050193_P01,GRMZM2G050193_P02	TRUE	TRUE	sLEGILPEIPLWVk	92%	n+304 (+304), K+304 (+304)	26.83	25.00

6190	seq=translation; coord=4:176500156..176502218:1; parent_transcript=GRMZM2G091189_T01; parent_gene=GRMZM2G091189	GRMZM2G091189_P01	TRUE	TRUE	eDDVIGILETDDVk	95%	n+304 (+304), K+304 (+304)	45.77	25.79
6191	seq=translation; coord=4:176500156..176502218:1; parent_transcript=GRMZM2G091189_T01; parent_gene=GRMZM2G091189	GRMZM2G091189_P01	TRUE	TRUE	tPGGLLLTETAk	95%	n+304 (+304), K+304 (+304)	36.54	25.15
6192	seq=translation; coord=1:260939345..260957939:1; parent_transcript=GRMZM2G104542_T01; parent_gene=GRMZM2G104542	GRMZM2G104542_P01	TRUE	TRUE	aMPFIIGNETFEk	95%	n+304 (+304), K+304 (+304)	34.45	26.29
6193	seq=translation; coord=1:260939345..260957939:1; parent_transcript=GRMZM2G104542_T01; parent_gene=GRMZM2G104542	GRMZM2G104542_P01	TRUE	TRUE	vTPEGSPFTSVVQVLSAALk	95%	n+304 (+304), K+304 (+304)	47.88	25.00
6194	seq=translation; coord=5:216598113..216599835:1; parent_transcript=GRMZM2G145133_T01; parent_gene=GRMZM2G145133	GRMZM2G145133_P01,GRMZM2G145133_P03	TRUE	TRUE	lVHLFcVVPR	95%	n+304 (+304), Carbamidomethyl (+57)	54.87	26.16
6195	seq=translation; coord=5:216598113..216599835:1; parent_transcript=GRMZM2G145133_T01; parent_gene=GRMZM2G145133	GRMZM2G145133_P01,GRMZM2G145133_P03	TRUE	TRUE	yGEAQMQAEEGLR	95%	n+304 (+304)	34.54	25.00
6196	seq=translation; coord=3:39292213..39295100:1; parent_transcript=GRMZM2G145449_T01; parent_gene=GRMZM2G145449	GRMZM2G145449_P01	TRUE	TRUE	sFNVEEAPSDFk	93%	n+304 (+304), K+304 (+304)	27.09	25.00
6197	seq=translation; coord=3:39292213..39295100:1; parent_transcript=GRMZM2G145449_T01; parent_gene=GRMZM2G145449	GRMZM2G145449_P01	TRUE	TRUE	sWDEGVATk	95%	n+304 (+304), K+304 (+304)	32.79	25.35
6198	seq=translation; coord=9:150649384..150656114:-1; parent_transcript=GRMZM2G172523_T01; parent_gene=GRMZM2G172523	GRMZM2G172523_P01,GRMZM2G172523_P02, GRMZM2G172523_P03	TRUE	TRUE	iAPFLFMPEGk	95%	n+304 (+304), K+304 (+304)	32.86	25.44
6199	seq=translation; coord=9:150649384..150656114:-1; parent_transcript=GRMZM2G172523_T01; parent_gene=GRMZM2G172523	GRMZM2G172523_P01,GRMZM2G172523_P02, GRMZM2G172523_P03	TRUE	TRUE	iDETPVWyVMDELGSAMR	95%	n+304 (+304), iTRAQ8plex (+304)	29.15	25.00
6200	seq=translation; coord=4:185694322..185697081:1; parent_transcript=GRMZM5G840955_T01; parent_gene=GRMZM5G840955	GRMZM5G840955_P01	TRUE	TRUE	iVLLPEPWTPESGLVTAALk	93%	n+304 (+304), K+304 (+304)	27.55	25.00
6201	seq=translation; coord=4:185694322..185697081:1; parent_transcript=GRMZM5G840955_T01; parent_gene=GRMZM5G840955	GRMZM5G840955_P01	TRUE	TRUE	lVSWEEGGYk	95%	n+304 (+304), K+304 (+304)	32.82	25.79
6202	seq=translation; coord=8:157100232..157101209:-1; parent_transcript=GRMZM2G168552_T01; parent_gene=GRMZM2G168552	GRMZM2G168552_P01	TRUE	TRUE	rPAYGADDcGYGGR	95%	n+304 (+304), Carbamidomethyl (+57)	28.79	25.00
6203	seq=translation; coord=8:157100232..157101209:-1; parent_transcript=GRMZM2G168552_T01; parent_gene=GRMZM2G168552	GRMZM2G168552_P01	TRUE	TRUE	tDEYYDNVDDR	95%	n+304 (+304)	33.92	25.00
6204	seq=translation; coord=8:14793941..14796834:1; parent_transcript=GRMZM2G139600_T01; parent_gene=GRMZM2G139600	GRMZM2G139600_P01	TRUE	TRUE	gALAAGVPGELAGLHAAWSR	95%	n+304 (+304)	66.93	25.82
6205	seq=translation; coord=1:3714426..3715519:1; parent_transcript=GRMZM2G030173_T01; parent_gene=GRMZM2G030173	GRMZM2G030173_P01	TRUE	TRUE	fLPGSAVATTIR	91%	n+304 (+304)	28.61	25.22
6206	seq=translation; coord=1:3714426..3715519:1; parent_transcript=GRMZM2G030173_T01; parent_gene=GRMZM2G030173	GRMZM2G030173_P01	TRUE	TRUE	iAVDYcPEAcLHER	95%	n+304 (+304), Carbamidomethyl (+57), Carbamidomethyl (+57)	43.22	25.00
6207	seq=translation; coord=7:18222228..18224357:1; parent_transcript=GRMZM2G094928_T01; parent_gene=GRMZM2G094928	GRMZM2G094928_P01,GRMZM2G094928_P03	TRUE	TRUE	aQMEAEFQR	92%	n+304 (+304)	29.12	25.00

6208	seq=translation; coord=7:18222228..18224357:1; parent_transcript=GRMZM2G094928_T01; parent_gene=GRMZM2G094928	GRMZM2G094928_P01,GRMZM2G094928_P03	TRUE	TRUE	qSGIQQLLAAEQEAQQIVNAAR	95%	n+304 (+304)	96.44	25.55
6209	seq=translation; coord=3:148974556..148982804:-1; parent_transcript=GRMZM2G367026_T01; parent_gene=GRMZM2G367026	GRMZM2G367026_P01,GRMZM2G367026_P02, GRMZM2G367026_P03	TRUE	TRUE	tHVDILSDFTPDFGANLR	95%	n+304 (+304)	35.95	25.00
6210	seq=translation; coord=3:4757467..4758786:-1; parent_transcript=GRMZM2G055434_T01; parent_gene=GRMZM2G055434	GRMZM2G055434_P01	TRUE	TRUE	aGWAPNPAEPFNAWAER	95%	n+304 (+304)	39.17	25.00
6211	seq=translation; coord=3:4757467..4758786:-1; parent_transcript=GRMZM2G055434_T01; parent_gene=GRMZM2G055434	GRMZM2G055434_P01	TRUE	TRUE	lVVVVLAVR	89%	n+304 (+304)	26.15	25.00
6212	seq=translation; coord=2:5924234..5926085:1; parent_transcript=GRMZM2G372074_T01; parent_gene=GRMZM2G372074	GRMZM2G372074_P01	TRUE	TRUE	lGAcVDILGNEVHIGDANVk	95%	n+304 (+304), Carbamidomethyl (+57), K+304 (+304)	47.15	25.45
6213	seq=translation; coord=3:155457171..155458057:1; parent_transcript=GRMZM2G158407_T01; parent_gene=GRMZM2G158407	GRMZM2G158407_P01	TRUE	TRUE	dcAGScIQQNLGTSGYck	95%	n+304 (+304), Carbamidomethyl (+57), Carbamidomethyl (+57), Carbamidomethyl (+57), K+304 (+304)	66.29	25.00
6214	seq=translation; coord=1:88998110..89000472:-1; parent_transcript=GRMZM2G089121_T02; parent_gene=GRMZM2G089121	GRMZM2G089121_P02	TRUE	TRUE	vIPFPLFELQSk	95%	n+304 (+304), K+304 (+304)	50.71	25.00
6215	seq=translation; coord=3:132847200..132850927:-1; parent_transcript=GRMZM2G155096_T01; parent_gene=GRMZM2G155096	GRMZM2G155096_P01,GRMZM2G155096_P02, GRMZM2G155096_P03,GRMZM2G155096_P04, GRMZM2G155096_P05,GRMZM2G155096_P06, GRMZM2G155096_P07,GRMZM2G155096_P08	TRUE	TRUE	sALNNEIEQLR	95%	n+304 (+304)	45.74	26.11
6216	seq=translation; coord=10:47749003..47752985:1; parent_transcript=GRMZM2G146951_T01; parent_gene=GRMZM2G146951	GRMZM2G146951_P01	TRUE	TRUE	vNMILDNLPLVVPIQR	95%	n+304 (+304)	47.49	25.00
6217	seq=translation; coord=3:199293031..199294186:1; parent_transcript=GRMZM5G818186_T01; parent_gene=GRMZM5G818186	GRMZM5G818186_P01	TRUE	TRUE	aAAAAADVIFLMVGFPSDVR	95%	n+304 (+304)	67.71	25.56
6218	seq=translation; coord=3:134681400..134683771:-1; parent_transcript=GRMZM2G110369_T01; parent_gene=GRMZM2G110369	GRMZM2G110369_P01,GRMZM2G110369_P02	TRUE	TRUE	gLGVELLR	95%	n+304 (+304)	31.88	25.00
6219	seq=translation; coord=8:141648491..141651509:-1; parent_transcript=GRMZM2G351990_T01; parent_gene=GRMZM2G351990	GRMZM2G351990_P01	TRUE	TRUE	gYAALVGVVPSMk	95%	n+304 (+304), K+304 (+304)	37.34	25.02
6220	seq=translation; coord=8:144537174..144540536:1; parent_transcript=GRMZM2G052844_T01; parent_gene=GRMZM2G052844	GRMZM2G052844_P01,GRMZM2G052844_P02	TRUE	TRUE	vSVASFGGAGDGR	95%	n+304 (+304)	40.10	25.00
6221	seq=translation; coord=1:200401193..200409521:1; parent_transcript=GRMZM2G082640_T01; parent_gene=GRMZM2G082640	GRMZM2G082640_P01,GRMZM2G082640_P02, GRMZM2G082640_P03	TRUE	TRUE	sDYEDGYEEEDVNSk	95%	n+304 (+304), K+304 (+304)	29.45	25.00
6222	seq=translation; coord=1:199870010..199870993:1; parent_transcript=AC218900.3_FGT001; parent_gene=AC218900.3_FG001	AC218900.3_FGP001,GRMZM2G064193_P01	TRUE	TRUE	dVVISPNVSAR	87%	n+304 (+304)	27.50	26.97
6223	seq=translation; coord=1:260618711..260622985:-1; parent_transcript=GRMZM2G083783_T01; parent_gene=GRMZM2G083783	GRMZM2G083783_P01,GRMZM2G083783_P03, GRMZM2G161242_P01,GRMZM2G161242_P02	TRUE	TRUE	gFGFVTFEDR	93%	n+304 (+304)	30.02	25.00

6224	seq=translation; coord=9:149245864..149247159:1; parent_transcript=GRMZM2G004466_T01; parent_gene=GRMZM2G004466	GRMZM2G004466_P01,GRMZM2G004466_P02	TRUE	TRUE	aLQLPAAck	89%	n+304 (+304), Carbamidomethyl (+57), K+304 (+304)	28.12	26.14
6225	GRMZM2G017671_P02-R	GRMZM2G017671_P02-R	TRUE	TRUE	lILVER	91%	n+304 (+304)	28.21	25.00
6226	seq=translation; coord=5:193306422..193309271:1; parent_transcript=AC209208.3_FGT002; parent_gene=AC209208.3_FG002	AC209208.3_FGP002,GRMZM2G392975_P01,GRMZM2G392975_P02	TRUE	TRUE	sLGAAIIYNR	95%	n+304 (+304)	38.14	25.00
6227	seq=translation; coord=9:18623338..18625477:1; parent_transcript=GRMZM2G052562_T01; parent_gene=GRMZM2G052562	GRMZM2G052562_P01,GRMZM2G052562_P02, GRMZM5G835276_P01	TRUE	TRUE	lVVDFLAEk	95%	n+304 (+304), K+304 (+304)	50.32	25.00
6228	seq=translation; coord=2:74306561..74321272:-1; parent_transcript=GRMZM2G082207_T01; parent_gene=GRMZM2G082207	GRMZM2G082207_P01,GRMZM2G082207_P02, GRMZM2G082207_P03,GRMZM2G082207_P04	TRUE	TRUE	aESPLDSFLSSLGLEk	95%	n+304 (+304), K+304 (+304)	38.31	25.29
6229	seq=translation; coord=1:47177906..47178782:1; parent_transcript=GRMZM2G112488_T01; parent_gene=GRMZM2G112488	GRMZM2G112488_P01	TRUE	TRUE	qFNFTSAMPFGFVk	92%	n+304 (+304), K+304 (+304)	27.75	25.87
6230	seq=translation; coord=6:151821892..151823785:-1; parent_transcript=GRMZM2G130746_T01; parent_gene=GRMZM2G130746	GRMZM2G130746_P01,GRMZM2G130746_P02	TRUE	TRUE	lLAGVTIAHGGVLPNINPVLLPk	95%	n+304 (+304), K+304 (+304)	39.24	25.00
6231	seq=translation; coord=7:163688367..163691160:-1; parent_transcript=GRMZM2G131167_T01; parent_gene=GRMZM2G131167	GRMZM2G131167_P01,GRMZM2G131167_P03	TRUE	TRUE	dAFSEFGNVLEAR	95%	n+304 (+304)	56.61	25.00
6232	seq=translation; coord=2:23176274..23181034:-1; parent_transcript=GRMZM2G354909_T01; parent_gene=GRMZM2G354909	GRMZM2G354909_P01,GRMZM2G354909_P02	TRUE	TRUE	qVIETLLPLLQASSDGR	95%	n+304 (+304)	36.45	25.35
6233	seq=translation; coord=1:36497245..36514437:1; parent_transcript=GRMZM2G372398_T01; parent_gene=GRMZM2G372398	GRMZM2G372398_P01	TRUE	TRUE	aLELLGEIYAR	95%	n+304 (+304)	32.93	25.19
6234	seq=translation; coord=9:124917394..124918766:-1; parent_transcript=GRMZM2G415793_T01; parent_gene=GRMZM2G415793	GRMZM2G415793_P01,GRMZM2G415793_P02	TRUE	TRUE	vQELYVYEMNER	95%	n+304 (+304)	51.15	25.00
6235	seq=translation; coord=9:141474317..141477935:-1; parent_transcript=GRMZM2G446234_T01; parent_gene=GRMZM2G446234	GRMZM2G446234_P01	TRUE	TRUE	gMDLLQEILQHLR	95%	n+304 (+304)	36.79	25.77
6236	seq=translation; coord=5:215265820..215267607:1; parent_transcript=GRMZM5G862817_T01; parent_gene=GRMZM5G862817	GRMZM5G862817_P01,GRMZM5G862817_P04	TRUE	TRUE	lPDDLLSAALGR	95%	n+304 (+304)	34.60	26.46
6237	seq=translation; coord=3:23896367..23898018:-1; parent_transcript=GRMZM2G114552_T01; parent_gene=GRMZM2G114552	GRMZM2G114552_P01,GRMZM2G114552_P02, GRMZM2G114552_P03,GRMZM2G114552_P04	TRUE	TRUE	dEERPWGEccDLAVcVk	91%	n+304 (+304), Carbamidomethyl (+57), Carbamidomethyl (+57), Carbamidomethyl (+57), K+304 (+304)	25.80	25.00
6238	seq=translation; coord=7:22256701..22261965:-1; parent_transcript=GRMZM2G140352_T01; parent_gene=GRMZM2G140352	GRMZM2G140352_P01,GRMZM2G140352_P02, GRMZM2G140352_P03,GRMZM2G140352_P04	TRUE	TRUE	gTVEVVFAR	91%	n+304 (+304)	28.22	25.00
6239	seq=translation; coord=8:175624692..175630581:1; parent_transcript=AC211349.4_FGT001; parent_gene=AC211349.4_FG001	AC211349.4_FGP001,GRMZM2G078756_P01	TRUE	TRUE	lAMVLFDIPDIR	95%	n+304 (+304)	49.45	25.13
6240	seq=translation; coord=6:95841179..95843445:-1; parent_transcript=GRMZM2G014119_T01; parent_gene=GRMZM2G014119	GRMZM2G014119_P01	TRUE	TRUE	mQLFILTLTGk	95%	n+304 (+304), K+304 (+304)	32.54	25.00
6241	seq=translation; coord=8:162534879..162538967:1; parent_transcript=GRMZM2G031204_T01; parent_gene=GRMZM2G031204	GRMZM2G031204_P01	TRUE	TRUE	sSDVTELQIGVk	95%	n+304 (+304), K+304 (+304)	66.93	25.45

6242	seq=translation; coord=2:230608941..230617571:-1; parent_transcript=GRMZM2G052403_T01; parent_gene=GRMZM2G052403	GRMZM2G052403_P01,GRMZM2G126453_P01, GRMZM2G126453_P02	TRUE	TRUE	iAPSVIFIDEVDSMLGR	95%	n+304 (+304)	34.49	25.73
6243	seq=translation; coord=3:196137228..196147873:1; parent_transcript=GRMZM2G127665_T01; parent_gene=GRMZM2G127665	GRMZM2G127665_P01	TRUE	TRUE	lVQDLSVIEAALR	95%	n+304 (+304)	32.59	25.43
6244	seq=translation; coord=2:10717934..10719159:-1; parent_transcript=GRMZM2G371375_T01; parent_gene=GRMZM2G371375	GRMZM2G371375_P01	TRUE	TRUE	gVAEDAADTASR	95%	n+304 (+304)	44.12	25.00
6245	seq=translation; coord=5:67476538..67482854:1; parent_transcript=GRMZM2G472346_T01; parent_gene=GRMZM2G472346	GRMZM2G472346_P01,GRMZM2G472346_P02	TRUE	TRUE	sMLEELEGMLEIVEPQPPGkPR	89%	n+304 (+304), K+304 (+304)	25.79	25.77
6246	seq=translation; coord=9:44334178..44336694:1; parent_transcript=GRMZM2G074085_T01; parent_gene=GRMZM2G074085	GRMZM2G074085_P01,GRMZM2G132623_P01, GRMZM2G132623_P02,GRMZM2G132623_P03	TRUE	TRUE	aMGTTDIR	92%	n+304 (+304)	28.60	25.00
6247	seq=translation; coord=9:15975038..15976941:1; parent_transcript=GRMZM2G304378_T01; parent_gene=GRMZM2G304378	GRMZM2G304378_P01	TRUE	TRUE	tcPIDTLk	93%	n+304 (+304), Carbamidomethyl (+57), K+304 (+304)	31.79	26.85
6248	seq=translation; coord=1:299124462..299129776:-1; parent_transcript=GRMZM2G176612_T02; parent_gene=GRMZM2G176612	GRMZM2G176612_P02	TRUE	TRUE	aISSSQFFGTQNR	89%	n+304 (+304)	26.02	25.00
6249	seq=translation; coord=1:299124462..299129776:-1; parent_transcript=GRMZM2G176612_T02; parent_gene=GRMZM2G176612	GRMZM2G176612_P02	TRUE	TRUE	fEYVENEPSADSR	95%	n+304 (+304)	32.33	25.00
6250	seq=translation; coord=1:299124462..299129776:-1; parent_transcript=GRMZM2G176612_T02; parent_gene=GRMZM2G176612	GRMZM2G176612_P02	TRUE	TRUE	iSFQASQDLSSLk	95%	n+304 (+304), K+304 (+304)	49.54	26.07
6251	seq=translation; coord=10:16487570..16504336:1; parent_transcript=GRMZM2G028307_T02; parent_gene=GRMZM2G028307	GRMZM2G028307_P02	TRUE	TRUE	aQMALWSMTk	95%	n+304 (+304), K+304 (+304)	33.04	25.26
6252	seq=translation; coord=10:16487570..16504336:1; parent_transcript=GRMZM2G028307_T02; parent_gene=GRMZM2G028307	GRMZM2G028307_P02	TRUE	TRUE	gISVQAVNADTPILDIk	95%	n+304 (+304), K+304 (+304)	43.30	25.00
6253	seq=translation; coord=10:16487570..16504336:1; parent_transcript=GRMZM2G028307_T02; parent_gene=GRMZM2G028307	GRMZM2G028307_P02	TRUE	TRUE	sWPDLDMLPFGWLTDPSVNQGPHR	95%	n+304 (+304)	37.77	25.00
6254	seq=translation; coord=9:17315382..17321668:1; parent_transcript=GRMZM5G864319_T01; parent_gene=GRMZM5G864319	GRMZM5G864319_P01	TRUE	TRUE	lFPLLASAYAFR	95%	n+304 (+304)	63.86	25.42
6255	seq=translation; coord=9:17315382..17321668:1; parent_transcript=GRMZM5G864319_T01; parent_gene=GRMZM5G864319	GRMZM5G864319_P01	TRUE	TRUE	sPDLLEAAVAHIQLVIVTk	95%	n+304 (+304), K+304 (+304)	28.00	25.00
6256	seq=translation; coord=1:165814484..165965196:1; parent_transcript=GRMZM2G003595_T01; parent_gene=GRMZM2G003595	GRMZM2G003595_P01,GRMZM2G003595_P02, GRMZM2G003631_P01	TRUE	TRUE	kVEVQDQNEVGGMPSR	95%	K+304 (+304), n+304 (+304)	38.83	25.19
6257	seq=translation; coord=1:165814484..165965196:1; parent_transcript=GRMZM2G003595_T01; parent_gene=GRMZM2G003595	GRMZM2G003595_P01,GRMZM2G003595_P02, GRMZM2G003631_P01	TRUE	TRUE	qEEEAAIk	95%	n+304 (+304), K+304 (+304)	32.10	25.85
6258	seq=translation; coord=2:9503022..9507292:-1; parent_transcript=GRMZM2G040397_T01; parent_gene=GRMZM2G040397	GRMZM2G040397_P01,GRMZM2G145460_P01	TRUE	TRUE	iSGAcFDAALGIIPGLk	95%	n+304 (+304), Carbamidomethyl (+57), K+304 (+304)	50.52	25.39
6259	seq=translation; coord=2:9503022..9507292:-1; parent_transcript=GRMZM2G040397_T01; parent_gene=GRMZM2G040397	GRMZM2G040397_P01,GRMZM2G145460_P01	TRUE	TRUE	lQFIYADLGDAk	95%	n+304 (+304), K+304 (+304)	44.97	25.00

6260	seq=translation; coord=2:56806976..56810257:1; parent_transcript=GRMZM2G119689_T01; parent_gene=GRMZM2G119689	GRMZM2G119689_P01,GRMZM2G119689_P02, GRMZM2G119689_P03	TRUE	TRUE	dLINWVALEPALRPSIPGDR	95%	n+304 (+304)	30.74	25.00
6261	seq=translation; coord=2:56806976..56810257:1; parent_transcript=GRMZM2G119689_T01; parent_gene=GRMZM2G119689	GRMZM2G119689_P01,GRMZM2G119689_P02, GRMZM2G119689_P03	TRUE	TRUE	gWAGIQAIPR	95%	n+304 (+304)	39.23	26.13
6262	seq=translation; coord=2:56806976..56810257:1; parent_transcript=GRMZM2G119689_T01; parent_gene=GRMZM2G119689	GRMZM2G119689_P01,GRMZM2G119689_P02, GRMZM2G119689_P03	TRUE	TRUE	qLLQWPIEEVEALR	95%	n+304 (+304)	40.73	25.82
6263	seq=translation; coord=3:182159627..182165670:-1; parent_transcript=GRMZM2G032258_T01; parent_gene=GRMZM2G032258	GRMZM2G032258_P01	TRUE	TRUE	eESEANEESTNk	95%	n+304 (+304), K+304 (+304)	32.28	25.00
6264	seq=translation; coord=3:182159627..182165670:-1; parent_transcript=GRMZM2G032258_T01; parent_gene=GRMZM2G032258	GRMZM2G032258_P01	TRUE	TRUE	iAIEDEIEDQR	95%	n+304 (+304)	32.30	25.00
6265	seq=translation; coord=3:215590446..215594988:1; parent_transcript=GRMZM2G057942_T01; parent_gene=GRMZM2G057942	GRMZM2G057942_P01	TRUE	TRUE	iPTLAIHLDR	95%	n+304 (+304)	59.69	27.11
6266	seq=translation; coord=3:215590446..215594988:1; parent_transcript=GRMZM2G057942_T01; parent_gene=GRMZM2G057942	GRMZM2G057942_P01	TRUE	TRUE	mVALFDHEEVGSDSAQGAGSPAMLDALSR	95%	n+304 (+304)	48.80	25.00
6267	seq=translation; coord=7:164948659..164953684:1; parent_transcript=GRMZM2G009538_T01; parent_gene=GRMZM2G009538	GRMZM2G009538_P01,GRMZM2G009538_P02	TRUE	TRUE	iAGGLDHLVAAGLSSLR	92%	n+304 (+304)	26.07	25.37
6268	seq=translation; coord=7:164948659..164953684:1; parent_transcript=GRMZM2G009538_T01; parent_gene=GRMZM2G009538	GRMZM2G009538_P01,GRMZM2G009538_P02	TRUE	TRUE	iQDVDDLAPLADLR	95%	n+304 (+304)	67.36	25.50
6269	seq=translation; coord=9:99409919..99416720:1; parent_transcript=GRMZM2G033555_T01; parent_gene=GRMZM2G033555	GRMZM2G033555_P01,GRMZM2G033555_P02, GRMZM2G033555_P03	TRUE	TRUE	nFVEDDVNSVYSSEk	95%	n+304 (+304), K+304 (+304)	28.84	25.00
6270	seq=translation; coord=9:99409919..99416720:1; parent_transcript=GRMZM2G033555_T01; parent_gene=GRMZM2G033555	GRMZM2G033555_P01,GRMZM2G033555_P02, GRMZM2G033555_P03	TRUE	TRUE	sVcVTGAGGFVASELVk	95%	n+304 (+304), Carbamidomethyl (+57), K+304 (+304)	37.55	25.73
6271	seq=translation; coord=8:4108305..4111250:-1; parent_transcript=GRMZM2G028156_T01; parent_gene=GRMZM2G028156	GRMZM2G028156_P01,GRMZM2G431306_P01, GRMZM2G431306_P02,GRMZM2G431306_P03	TRUE	TRUE	eQIAGELQLPANk	95%	n+304 (+304), K+304 (+304)	30.24	25.00
6272	seq=translation; coord=8:4108305..4111250:-1; parent_transcript=GRMZM2G028156_T01; parent_gene=GRMZM2G028156	GRMZM2G028156_P01,GRMZM2G431306_P01, GRMZM2G431306_P02,GRMZM2G431306_P03	TRUE	TRUE	fVVSPITGELIPISEMEEHMR	95%	n+304 (+304)	31.35	25.25
6273	seq=translation; coord=8:4108305..4111250:-1; parent_transcript=GRMZM2G028156_T01; parent_gene=GRMZM2G028156	GRMZM2G028156_P01,GRMZM2G431306_P01, GRMZM2G431306_P02,GRMZM2G431306_P03	TRUE	TRUE	tIGIIHPPPDIR	95%	n+304 (+304)	33.73	25.00
6274	seq=translation; coord=2:198568661..198574971:1; parent_transcript=GRMZM2G081571_T04; parent_gene=GRMZM2G081571	GRMZM2G081571_P04,GRMZM2G081571_P05	TRUE	TRUE	aASFLSSLLGGGGGEPAATVk	95%	n+304 (+304), K+304 (+304)	63.75	25.17
6275	seq=translation; coord=2:198568661..198574971:1; parent_transcript=GRMZM2G081571_T04; parent_gene=GRMZM2G081571	GRMZM2G081571_P04,GRMZM2G081571_P05	TRUE	TRUE	gLAVSQAPITSTGFR	95%	n+304 (+304)	55.48	25.84
6276	seq=translation; coord=3:159873679..159879241:-1; parent_transcript=GRMZM2G105531_T01; parent_gene=GRMZM2G105531	GRMZM2G105531_P01	TRUE	TRUE	aPELHLEIQR	95%	n+304 (+304)	38.40	25.00
6277	seq=translation; coord=3:159873679..159879241:-1; parent_transcript=GRMZM2G105531_T01; parent_gene=GRMZM2G105531	GRMZM2G105531_P01	TRUE	TRUE	lFFFEMAck	95%	n+304 (+304), Carbamidomethyl (+57), K+304 (+304)	29.80	25.17

6278	seq=translation; coord=6:161446540..161449925:1; parent_transcript=GRMZM2G115049_T01; parent_gene=GRMZM2G115049	GRMZM2G115049_P01	TRUE	TRUE	aILcVPFPYQk	95%	n+304 (+304), Carbamidomethyl (+57), K+304 (+304)	34.22	25.00
6279	seq=translation; coord=6:161446540..161449925:1; parent_transcript=GRMZM2G115049_T01; parent_gene=GRMZM2G115049	GRMZM2G115049_P01	TRUE	TRUE	vGLSLVLkP	95%	n+304 (+304), K+304 (+304)	37.36	25.00
6280	seq=translation; coord=9:83796519..83798959:1; parent_transcript=GRMZM2G140179_T01; parent_gene=GRMZM2G140179	GRMZM2G140179_P01	TRUE	TRUE	aLAGSGPAEAVYIYLLDLGk	95%	n+304 (+304), K+304 (+304)	50.07	25.00
6281	seq=translation; coord=9:83796519..83798959:1; parent_transcript=GRMZM2G140179_T01; parent_gene=GRMZM2G140179	GRMZM2G140179_P01	TRUE	TRUE	aLLADLASLVLSAYk	95%	n+304 (+304), K+304 (+304)	30.17	25.00
6282	seq=translation; coord=7:169836954..169839970:-1; parent_transcript=GRMZM2G037177_T01; parent_gene=GRMZM2G037177	GRMZM2G037177_P01	TRUE	TRUE	iPSGEVWVGNPAk	95%	n+304 (+304), K+304 (+304)	30.67	25.00
6283	seq=translation; coord=7:169836954..169839970:-1; parent_transcript=GRMZM2G037177_T01; parent_gene=GRMZM2G037177	GRMZM2G037177_P01	TRUE	TRUE	sFDEIELEk	95%	n+304 (+304), K+304 (+304)	32.04	25.44
6284	seq=translation; coord=7:169836954..169839970:-1; parent_transcript=GRMZM2G037177_T01; parent_gene=GRMZM2G037177	GRMZM2G037177_P01	TRUE	TRUE	tIMNIFEk	91%	n+304 (+304), K+304 (+304)	27.80	26.58
6285	seq=translation; coord=1:285137082..285140683:-1; parent_transcript=GRMZM2G058760_T01; parent_gene=GRMZM2G058760	GRMZM2G058760_P01	TRUE	TRUE	iMLLPEEDPNATHIMIATGTGVAPFR	95%	n+304 (+304)	55.28	25.00
6286	seq=translation; coord=4:12777382..12783007:-1; parent_transcript=GRMZM2G139533_T01; parent_gene=GRMZM2G139533	GRMZM2G139533_P01,GRMZM2G139533_P02	TRUE	TRUE	iPATLIADSAAAALMk	95%	n+304 (+304), K+304 (+304)	45.01	25.00
6287	seq=translation; coord=2:16302477..16307549:-1; parent_transcript=GRMZM2G175728_T01; parent_gene=GRMZM2G175728	GRMZM2G175728_P01	TRUE	TRUE	sDPGIAAAVIAR	94%	n+304 (+304)	31.99	26.07
6288	seq=translation; coord=2:16302477..16307549:-1; parent_transcript=GRMZM2G175728_T01; parent_gene=GRMZM2G175728	GRMZM2G175728_P01	TRUE	TRUE	sIALYATEDR	95%	n+304 (+304)	40.23	25.00
6289	seq=translation; coord=1:268608810..268612288:-1; parent_transcript=GRMZM2G176735_T01; parent_gene=GRMZM2G176735	GRMZM2G176735_P01,GRMZM2G176735_P02	TRUE	TRUE	iAPPLVVPLLTGELILGk	93%	n+304 (+304), K+304 (+304)	27.31	25.00
6290	seq=translation; coord=1:28446186..28450646:-1; parent_transcript=GRMZM2G099860_T01; parent_gene=GRMZM2G099860	GRMZM2G099860_P01	TRUE	TRUE	vVMLGVDDMDIFk	95%	n+304 (+304), K+304 (+304)	58.87	25.75
6291	seq=translation; coord=6:164890857..164893003:-1; parent_transcript=GRMZM2G102779_T01; parent_gene=GRMZM2G102779	GRMZM2G102779_P01	TRUE	TRUE	wTAQALLAGADMMk	91%	n+304 (+304), K+304 (+304)	26.73	25.49
6292	seq=translation; coord=7:43705280..43706429:-1; parent_transcript=GRMZM2G113668_T01; parent_gene=GRMZM2G113668	GRMZM2G113668_P01	TRUE	TRUE	fLGVVR	91%	n+304 (+304)	26.76	25.00
6293	seq=translation; coord=7:43705280..43706429:-1; parent_transcript=GRMZM2G113668_T01; parent_gene=GRMZM2G113668	GRMZM2G113668_P01	TRUE	TRUE	gHLAALLWYQGEADTMR	95%	n+304 (+304)	41.58	25.00
6294	seq=translation; coord=10:18333983..18347671:-1; parent_transcript=GRMZM2G015238_T01; parent_gene=GRMZM2G015238	GRMZM2G015238_P01	TRUE	TRUE	vVPLHSLk	89%	n+304 (+304), K+304 (+304)	25.04	25.00
6295	seq=translation; coord=8:174419193..174424343:1; parent_transcript=GRMZM2G118462_T01; parent_gene=GRMZM2G118462	GRMZM2G118462_P01,GRMZM2G123277_P01, GRMZM2G304274_P01	TRUE	TRUE	lLAFENMLR	92%	n+304 (+304)	27.47	25.00

6296	seq=translation; coord=8:174419193..174424343:1; parent_transcript=GRMZM2G118462_T01; parent_gene=GRMZM2G118462	GRMZM2G118462_P01,GRMZM2G123277_P01, GRMZM2G304274_P01	TRUE	TRUE	tVLLGVDDMDIFk	95%	n+304 (+304), K+304 (+304)	40.32	25.99
6297	seq=translation; coord=4:186948838..186952130:-1; parent_transcript=GRMZM2G357595_T01; parent_gene=GRMZM2G357595	GRMZM2G357595_P01	TRUE	TRUE	aIEYDNFIEccLTVk	95%	n+304 (+304), Carbamidomethyl (+57), Carbamidomethyl (+57), K+304 (+304)	45.66	25.00
6298	seq=translation; coord=4:186948838..186952130:-1; parent_transcript=GRMZM2G357595_T01; parent_gene=GRMZM2G357595	GRMZM2G357595_P01	TRUE	TRUE	eFTSVFYSLQNWR	90%	n+304 (+304)	25.23	25.00
6299	seq=translation; coord=4:65261557..65280118:-1; parent_transcript=GRMZM2G055331_T01; parent_gene=GRMZM2G055331	GRMZM2G055331_P01	TRUE	TRUE	yFVEEVISGFDETDLYk	95%	n+304 (+304), iTRAQ8plex (+304), K+304 (+304)	40.29	25.48
6300	seq=translation; coord=6:90530607..90532293:-1; parent_transcript=GRMZM2G016250_T01; parent_gene=GRMZM2G016250	GRMZM2G016250_P01,GRMZM2G302712_P01, GRMZM2G326066_P01,GRMZM2G366077_P01	TRUE	TRUE	lVNFTHLMPTR	95%	n+304 (+304)	53.75	25.30
6301	seq=translation; coord=5:5433227..5435612:-1; parent_transcript=AC195340.3_FGT001; parent_gene=AC195340.3_FG001	AC195340.3_FGP001	TRUE	TRUE	aFHEQLSVAEITSSAFEPASMMVk	95%	n+304 (+304), K+304 (+304)	49.04	25.00
6302	seq=translation; coord=2:75419239..75423605:-1; parent_transcript=AC197056.3_FGT005; parent_gene=AC197056.3_FG005	AC197056.3_FGP005,GRMZM2G116693_P01	TRUE	TRUE	rWEVVSEFIGTSR	93%	n+304 (+304)	28.11	25.45
6303	seq=translation; coord=6:132657599..132658828:-1; parent_transcript=AC234575.1_FGT003; parent_gene=AC234575.1_FG003	AC234575.1_FGP003,GRMZM2G120833_P01,GRMZM2G179797_P03	TRUE	TRUE	aDFGAVMDVLk	95%	n+304 (+304), K+304 (+304)	48.29	26.31
6304	seq=translation; coord=5:183309974..183317753:1; parent_transcript=GRMZM2G012841_T01; parent_gene=GRMZM2G012841	GRMZM2G012841_P01,GRMZM2G012841_P02, GRMZM2G012841_P03,GRMZM2G012841_P04, GRMZM2G012841_P05,GRMZM2G012841_P07, GRMZM2G012841_P09	TRUE	TRUE	lPHQMNFLEELR	95%	n+304 (+304)	59.98	25.45
6305	seq=translation; coord=1:207168310..207172439:-1; parent_transcript=GRMZM2G037655_T01; parent_gene=GRMZM2G037655	GRMZM2G037655_P01,GRMZM2G138881_P01, GRMZM2G138881_P02	TRUE	TRUE	rPVAVVMAWVR	95%	n+304 (+304)	54.97	26.19
6306	seq=translation; coord=8:13554391..13555376:1; parent_transcript=GRMZM2G041238_T01; parent_gene=GRMZM2G041238	GRMZM2G041238_P01,GRMZM2G108871_P01, GRMZM2G307892_P01,GRMZM2G327564_P01, GRMZM2G412848_P01	TRUE	TRUE	wVIHVER	95%	n+304 (+304)	36.15	26.50
6307	seq=translation; coord=2:191350704..191353166:1; parent_transcript=GRMZM2G047971_T02; parent_gene=GRMZM2G047971	GRMZM2G047971_P02,GRMZM2G065868_P01, GRMZM2G065868_P02,GRMZM2G065868_P03	TRUE	TRUE	fVVHNVSELELLLMHNR	95%	n+304 (+304)	67.03	25.81
6308	seq=translation; coord=2:161045370..161050327:-1; parent_transcript=GRMZM2G051367_T01; parent_gene=GRMZM2G051367	GRMZM2G051367_P01	TRUE	TRUE	lSLPPSLTGSAISLVSVMQAR	95%	n+304 (+304)	35.66	25.34
6309	seq=translation; coord=2:3605933..3609654:-1; parent_transcript=GRMZM2G061745_T01; parent_gene=GRMZM2G061745	GRMZM2G061745_P01,GRMZM2G061745_P02, GRMZM2G061745_P03,GRMZM2G061745_P04, GRMZM2G368908_P01,GRMZM2G368908_P02	TRUE	TRUE	vVVHPLVLLSIVDHYNR	95%	n+304 (+304)	55.78	25.00
6310	seq=translation; coord=8:28291237..28300310:1; parent_transcript=GRMZM2G062953_T01; parent_gene=GRMZM2G062953	GRMZM2G062953_P01,GRMZM2G062953_P02	TRUE	TRUE	vLVLIDTWQDVFGGSHAR	95%	n+304 (+304)	48.13	25.65
6311	seq=translation; coord=3:127007479..127010593:-1; parent_transcript=GRMZM2G067476_T01; parent_gene=GRMZM2G067476	GRMZM2G067476_P01,GRMZM2G067476_P02, GRMZM2G067476_P03,GRMZM2G376727_P01, GRMZM2G376727_P02,GRMZM2G376727_P03, GRMZM2G376727_P04,GRMZM2G376727_P05	TRUE	TRUE	fQGSPAIITYR	95%	n+304 (+304)	42.02	26.31

6312	seq=translation; coord=3:38428236..38432826:1; parent_transcript=GRMZM2G067511_T01; parent_gene=GRMZM2G067511	GRMZM2G067511_P01	TRUE	TRUE	eADVDGDGQINYDEFVk	95%	n+304 (+304), K+304 (+304)	47.07	25.00
6313	seq=translation; coord=4:180790322..180791225:-1; parent_transcript=GRMZM2G087137_T01; parent_gene=GRMZM2G087137	GRMZM2G087137_P01,GRMZM2G087137_P02	TRUE	TRUE	vAEPEEGTEALGVLDR	95%	n+304 (+304)	46.14	25.33
6314	seq=translation; coord=5:191079735..191083805:1; parent_transcript=GRMZM2G097728_T02; parent_gene=GRMZM2G097728	GRMZM2G097728_P02,GRMZM2G097728_P03	TRUE	TRUE	nATNVEQAFMAMAASIk	95%	n+304 (+304), K+304 (+304)	34.65	25.56
6315	seq=translation; coord=6:120878064..120884267:-1; parent_transcript=GRMZM2G101874_T01; parent_gene=GRMZM2G101874	GRMZM2G101874_P01	TRUE	TRUE	iPALVETLVSk	95%	n+304 (+304), K+304 (+304)	52.45	25.00
6316	seq=translation; coord=3:147745196..147752548:1; parent_transcript=GRMZM2G109284_T01; parent_gene=GRMZM2G109284	GRMZM2G109284_P01	TRUE	TRUE	vDASGDAEQPAADELR	95%	n+304 (+304)	48.16	25.00
6317	seq=translation; coord=4:236889764..236896376:-1; parent_transcript=GRMZM2G119640_T01; parent_gene=GRMZM2G119640	GRMZM2G119640_P01,GRMZM2G119640_P02, GRMZM2G119640_P03,GRMZM2G119640_P04, GRMZM2G119640_P06,GRMZM2G119640_P07	TRUE	TRUE	sLLEIILDEk	95%	n+304 (+304), K+304 (+304)	41.91	25.00
6318	seq=translation; coord=6:147221316..147226156:1; parent_transcript=GRMZM2G121514_T01; parent_gene=GRMZM2G121514	GRMZM2G121514_P01,GRMZM2G121514_P02	TRUE	TRUE	gAPVLAGVHVAVR	95%	n+304 (+304)	49.22	25.00
6319	seq=translation; coord=6:131423962..131426896:1; parent_transcript=GRMZM2G126594_T01; parent_gene=GRMZM2G126594	GRMZM2G126594_P01,GRMZM2G126594_P02, GRMZM2G126594_P04,GRMZM2G127521_P01, GRMZM2G127521_P02	TRUE	TRUE	sYLEDVIAHk	95%	n+304 (+304), K+304 (+304)	38.94	26.33
6320	seq=translation; coord=7:144129492..144134147:-1; parent_transcript=GRMZM2G139210_T01; parent_gene=GRMZM2G139210	GRMZM2G139210_P01,GRMZM2G139210_P02, GRMZM2G139210_P03	TRUE	TRUE	aAIGIIDIVLk	95%	n+304 (+304), K+304 (+304)	53.16	25.00
6321	seq=translation; coord=2:38132179..38142127:1; parent_transcript=GRMZM2G147884_T01; parent_gene=GRMZM2G147884	GRMZM2G147884_P01,GRMZM2G147884_P02, GRMZM2G147884_P03	TRUE	TRUE	fVEAVLLEPEk	95%	n+304 (+304), K+304 (+304)	34.72	26.25
6322	seq=translation; coord=6:88898226..88901822:1; parent_transcript=GRMZM2G150209_T01; parent_gene=GRMZM2G150209	GRMZM2G150209_P01,GRMZM2G150209_P02	TRUE	TRUE	lYMVAPFLTGLk	95%	n+304 (+304), K+304 (+304)	30.04	25.00
6323	seq=translation; coord=7:167908789..167916594:-1; parent_transcript=GRMZM2G150683_T01; parent_gene=GRMZM2G150683	GRMZM2G150683_P01,GRMZM2G150683_P02, GRMZM2G150683_P03,GRMZM2G150683_P05, GRMZM2G150683_P06	TRUE	TRUE	qGADATALQAGLNWAcGQGR	95%	n+304 (+304), Carbamidomethyl (+57)	54.96	25.00
6324	seq=translation; coord=7:111657695..111660947:-1; parent_transcript=GRMZM2G159221_T01; parent_gene=GRMZM2G159221	GRMZM2G159221_P01,GRMZM2G159221_P02	TRUE	TRUE	eAAQDIIQEYLR	95%	n+304 (+304)	37.22	25.00
6325	seq=translation; coord=9:148206141..148211626:-1; parent_transcript=GRMZM2G181542_T01; parent_gene=GRMZM2G181542	GRMZM2G181542_P01	TRUE	TRUE	vAVDFIASHPQAk	95%	n+304 (+304), K+304 (+304)	31.22	25.28
6326	seq=translation; coord=5:79503885..79505592:1; parent_transcript=GRMZM2G314233_T01; parent_gene=GRMZM2G314233	GRMZM2G314233_P01	TRUE	TRUE	qNLAATFVNAFVNAGFGk	95%	n+304 (+304), K+304 (+304)	47.24	25.00
6327	seq=translation; coord=3:162599045..162600039:1; parent_transcript=GRMZM5G846152_T01; parent_gene=GRMZM5G846152	GRMZM5G846152_P01	TRUE	TRUE	aHQLPGGLLPAGITAFR	95%	n+304 (+304)	51.33	25.60
6328	seq=translation; coord=7:146895009..146896961:-1; parent_transcript=GRMZM2G066528_T01; parent_gene=GRMZM2G066528	GRMZM2G066528_P01,GRMZM2G066528_P02, GRMZM2G066528_P03,GRMZM2G066528_P04	TRUE	TRUE	rPPSAFFVFMEEFR	94%	n+304 (+304)	28.47	25.00

6329	seq=translation; coord=5:155243219..155246765:-1; parent_transcript=GRMZM2G029559_T02; parent_gene=GRMZM2G029559	GRMZM2G029559_P02	TRUE	TRUE	lILDDWk	94%	n+304 (+304), K+304 (+304)	29.29	26.05
6330	seq=translation; coord=2:4687656..4692442:1; parent_transcript=GRMZM2G086801_T01; parent_gene=GRMZM2G086801	GRMZM2G086801_P01,GRMZM2G086801_P02	TRUE	TRUE	iSALALLk	94%	n+304 (+304), K+304 (+304)	27.95	25.00
6331	seq=translation; coord=3:37865603..37869803:1; parent_transcript=GRMZM2G069208_T01; parent_gene=GRMZM2G069208	GRMZM2G049429_P01,GRMZM2G049429_P02, GRMZM2G069208_P01	TRUE	TRUE	lVDLPPQEEISGMGMSk	95%	n+304 (+304), K+304 (+304)	56.93	25.43
6332	seq=translation; coord=3:37865603..37869803:1; parent_transcript=GRMZM2G069208_T01; parent_gene=GRMZM2G069208	GRMZM2G049429_P01,GRMZM2G049429_P02, GRMZM2G069208_P01	TRUE	TRUE	qFHEMVGHFVDIMk	95%	Pyro-cmC (-17), n+304 (+304), K+304 (+304)	57.92	25.00
6333	seq=translation; coord=3:37865603..37869803:1; parent_transcript=GRMZM2G069208_T01; parent_gene=GRMZM2G069208	GRMZM2G049429_P01,GRMZM2G049429_P02, GRMZM2G069208_P01	TRUE	TRUE	sSIILPLSGLk	95%	n+304 (+304), K+304 (+304)	40.87	25.00
6334	seq=translation; coord=3:7078308..7082094:1; parent_transcript=GRMZM2G104081_T01; parent_gene=GRMZM2G104081	GRMZM2G104081_P01,GRMZM2G104081_P02, GRMZM2G104081_P03	TRUE	TRUE	aFSINDAVGEDVVAELQTAMEk	95%	n+304 (+304), K+304 (+304)	37.79	25.00
6335	seq=translation; coord=3:7078308..7082094:1; parent_transcript=GRMZM2G104081_T01; parent_gene=GRMZM2G104081	GRMZM2G104081_P01,GRMZM2G104081_P02, GRMZM2G104081_P03	TRUE	TRUE	lAAAGLAGILMk	95%	n+304 (+304), K+304 (+304)	29.47	25.00
6336	seq=translation; coord=3:7078308..7082094:1; parent_transcript=GRMZM2G104081_T01; parent_gene=GRMZM2G104081	GRMZM2G104081_P01,GRMZM2G104081_P02, GRMZM2G104081_P03	TRUE	TRUE	qGLDMHVAALINDAVGTLAGAR	95%	n+304 (+304)	30.39	25.82
6337	seq=translation; coord=10:61378667..61384801:1; parent_transcript=GRMZM2G143568_T01; parent_gene=GRMZM2G143568	GRMZM2G143568_P01	TRUE	TRUE	aLFGVSTIIYk	95%	n+304 (+304), K+304 (+304)	48.63	25.00
6338	seq=translation; coord=10:61378667..61384801:1; parent_transcript=GRMZM2G143568_T01; parent_gene=GRMZM2G143568	GRMZM2G143568_P01	TRUE	TRUE	iLcPDSLIGSVIGk	95%	n+304 (+304), Carbamidomethyl (+57), K+304 (+304)	32.59	25.00
6339	seq=translation; coord=10:61378667..61384801:1; parent_transcript=GRMZM2G143568_T01; parent_gene=GRMZM2G143568	GRMZM2G143568_P01	TRUE	TRUE	sAAVEAVLLLQAk	95%	n+304 (+304), K+304 (+304)	33.80	25.00
6340	seq=translation; coord=2:12023721..12027357:-1; parent_transcript=GRMZM2G156365_T02; parent_gene=GRMZM2G156365	GRMZM2G156365_P02,GRMZM2G156365_P03, GRMZM2G156365_P04,GRMZM2G156365_P05, GRMZM2G156365_P06	TRUE	TRUE	aQNALLSGcSAGGLAAILHcDR	95%	n+304 (+304), Carbamidomethyl (+57), Carbamidomethyl (+57)	80.08	25.00
6341	seq=translation; coord=2:12023721..12027357:-1; parent_transcript=GRMZM2G156365_T02; parent_gene=GRMZM2G156365	GRMZM2G156365_P02,GRMZM2G156365_P03, GRMZM2G156365_P04,GRMZM2G156365_P05, GRMZM2G156365_P06	TRUE	TRUE	aVIDDLLTVR	95%	n+304 (+304)	32.18	25.00
6342	seq=translation; coord=2:12023721..12027357:-1; parent_transcript=GRMZM2G156365_T02; parent_gene=GRMZM2G156365	GRMZM2G156365_P02,GRMZM2G156365_P03, GRMZM2G156365_P04,GRMZM2G156365_P05, GRMZM2G156365_P06	TRUE	TRUE	tPLFILNAAYDSWQVk	95%	n+304 (+304), K+304 (+304)	44.66	25.30
6343	seq=translation; coord=5:18170761..18172340:1; parent_transcript=GRMZM2G060940_T01; parent_gene=GRMZM2G060940	GRMZM2G060940_P01,GRMZM2G060940_P02, GRMZM2G060940_P03	TRUE	TRUE	dWGFFVVVGHGVPAETVAR	95%	n+304 (+304)	43.97	25.28
6344	seq=translation; coord=5:18170761..18172340:1; parent_transcript=GRMZM2G060940_T01; parent_gene=GRMZM2G060940	GRMZM2G060940_P01,GRMZM2G060940_P02, GRMZM2G060940_P03	TRUE	TRUE	eVYDLVPR	95%	n+304 (+304)	32.69	25.40
6345	seq=translation; coord=5:18170761..18172340:1; parent_transcript=GRMZM2G060940_T01; parent_gene=GRMZM2G060940	GRMZM2G060940_P01,GRMZM2G060940_P02, GRMZM2G060940_P03	TRUE	TRUE	lLELIAR	87%	n+304 (+304)	25.06	25.00
6346	seq=translation; coord=3:127303985..127310112:-1; parent_transcript=AC182617.3_FGT001; parent_gene=AC182617.3_FG001	AC182617.3_FGP001,GRMZM2G130332_P01,G RMZM2G130332_P02,GRMZM2G130332_P03,G RMZM2G130332_P04	TRUE	TRUE	aLHFVk	92%	n+304 (+304), K+304 (+304)	26.14	25.00

6347	seq=translation; coord=3:127303985..127310112:-1; parent_transcript=AC182617.3_FGT001; parent_gene=AC182617.3_FG001	AC182617.3_FGP001,GRMZM2G130332_P01,GRMZM2G130332_P02,GRMZM2G130332_P03,GRMZM2G130332_P04	TRUE	TRUE	yQGLGNDFIMVDNR	95%	n+304 (+304)	32.58	25.00
6348	seq=translation; coord=7:132157014..132162257:1; parent_transcript=GRMZM2G068862_T01; parent_gene=GRMZM2G068862	GRMZM2G068862_P01	TRUE	TRUE	mAEQTLSLLk	93%	n+304 (+304), K+304 (+304)	26.65	25.09
6349	seq=translation; coord=7:132157014..132162257:1; parent_transcript=GRMZM2G068862_T01; parent_gene=GRMZM2G068862	GRMZM2G068862_P01	TRUE	TRUE	vDVEYYAINVVVPQk	95%	n+304 (+304), K+304 (+304)	45.07	25.00
6350	seq=translation; coord=4:120394113..120399917:1; parent_transcript=GRMZM2G153984_T01; parent_gene=GRMZM2G153984	GRMZM2G153984_P01	TRUE	TRUE	gYVDMSTVDAATSTk	95%	n+304 (+304), K+304 (+304)	46.09	25.00
6351	seq=translation; coord=4:120394113..120399917:1; parent_transcript=GRMZM2G153984_T01; parent_gene=GRMZM2G153984	GRMZM2G153984_P01	TRUE	TRUE	sGLSPQTLLDVLDLGAIANPMFk	95%	n+304 (+304), K+304 (+304)	29.84	25.00
6352	seq=translation; coord=2:130522633..130525928:-1; parent_transcript=GRMZM2G128268_T01; parent_gene=GRMZM2G128268	GRMZM2G128268_P01	TRUE	TRUE	gSVSLVSQLk	95%	n+304 (+304), K+304 (+304)	46.79	25.00
6353	seq=translation; coord=2:130522633..130525928:-1; parent_transcript=GRMZM2G128268_T01; parent_gene=GRMZM2G128268	GRMZM2G128268_P01	TRUE	TRUE	nVVGHcLSTNGGGFLFFGDDVVPSSR	95%	n+304 (+304), Carbamidomethyl (+57)	57.56	25.00
6354	seq=translation; coord=3:173472565..173475730:-1; parent_transcript=GRMZM2G081652_T01; parent_gene=GRMZM2G081652	GRMZM2G081652_P01	TRUE	TRUE	aQVAADAPVEEVk	93%	n+304 (+304), K+304 (+304)	28.60	26.33
6355	seq=translation; coord=3:173472565..173475730:-1; parent_transcript=GRMZM2G081652_T01; parent_gene=GRMZM2G081652	GRMZM2G081652_P01	TRUE	TRUE	yIAPEQVPVQFGGLFk	95%	n+304 (+304), K+304 (+304)	47.60	25.00
6356	seq=translation; coord=1:205090665..205094372:1; parent_transcript=GRMZM2G144387_T01; parent_gene=GRMZM2G144387	GRMZM2G144387_P01,GRMZM2G144387_P03	TRUE	TRUE	dAISQIVGDAk	95%	n+304 (+304), K+304 (+304)	32.10	26.78
6357	seq=translation; coord=1:205090665..205094372:1; parent_transcript=GRMZM2G144387_T01; parent_gene=GRMZM2G144387	GRMZM2G144387_P01,GRMZM2G144387_P03	TRUE	TRUE	vcMLGDAQHVEEAEk	95%	n+304 (+304), Carbamidomethyl (+57), K+304 (+304)	30.86	25.00
6358	seq=translation; coord=9:25522193..25524594:-1; parent_transcript=GRMZM2G383540_T01; parent_gene=GRMZM2G383540	GRMZM2G383540_P01,GRMZM5G808811_P01	TRUE	TRUE	qQMIDcYIQTLAk	95%	n+304 (+304), Carbamidomethyl (+57), K+304 (+304)	37.76	25.49
6359	seq=translation; coord=10:85188397..85193819:-1; parent_transcript=GRMZM2G101875_T01; parent_gene=GRMZM2G101875	GRMZM2G101875_P01,GRMZM2G101875_P02	TRUE	TRUE	mTTDDVFLSFLPLAHILDR	95%	n+304 (+304)	34.58	25.00
6360	seq=translation; coord=5:38567212..38578529:-1; parent_transcript=GRMZM2G377341_T01; parent_gene=GRMZM2G377341	GRMZM2G377341_P01	TRUE	TRUE	sLAIANMVLGLk	94%	n+304 (+304), K+304 (+304)	28.22	25.00
6361	seq=translation; coord=10:98135181..98154139:1; parent_transcript=AC197672.3_FGT002; parent_gene=AC197672.3_FG002	AC197672.3_FGP002	TRUE	TRUE	aIHLIAMATPEDMR	95%	n+304 (+304)	31.25	25.24
6362	seq=translation; coord=10:98135181..98154139:1; parent_transcript=AC197672.3_FGT002; parent_gene=AC197672.3_FG002	AC197672.3_FGP002	TRUE	TRUE	dLFEGILQAGSAIVENLR	95%	n+304 (+304)	30.83	25.45
6363	seq=translation; coord=9:9638146..9648092:-1; parent_transcript=GRMZM2G033130_T01; parent_gene=GRMZM2G033130	GRMZM2G033130_P01,GRMZM2G033130_P03, GRMZM2G033130_P04,GRMZM2G033130_P06, GRMZM2G152768_P01	TRUE	TRUE	eSNMDPNETAQk	95%	n+304 (+304), K+304 (+304)	40.53	25.00
6364	seq=translation; coord=9:9638146..9648092:-1; parent_transcript=GRMZM2G033130_T01; parent_gene=GRMZM2G033130	GRMZM2G033130_P01,GRMZM2G033130_P03, GRMZM2G033130_P04,GRMZM2G033130_P06, GRMZM2G152768_P01	TRUE	TRUE	eVVVGHSDADILDTLR	95%	n+304 (+304)	50.82	25.45

6365	seq=translation; coord=9:99437276..99441674:1; parent_transcript=GRMZM2G034069_T01; parent_gene=GRMZM2G034069	GRMZM2G034069_P01	TRUE	TRUE	dVADALLLAYEkPEASGR	95%	n+304 (+304), K+304 (+304)	44.22	26.10
6366	seq=translation; coord=9:99437276..99441674:1; parent_transcript=GRMZM2G034069_T01; parent_gene=GRMZM2G034069	GRMZM2G034069_P01	TRUE	TRUE	vADMINILk	95%	n+304 (+304), K+304 (+304)	31.94	25.45
6367	seq=translation; coord=9:146299403..146305484:1; parent_transcript=GRMZM2G051782_T01; parent_gene=GRMZM2G051782	GRMZM2G051782_P01	TRUE	TRUE	aLFVDLEPTVIDEVk	95%	n+304 (+304), K+304 (+304)	59.33	25.69
6368	seq=translation; coord=9:146299403..146305484:1; parent_transcript=GRMZM2G051782_T01; parent_gene=GRMZM2G051782	GRMZM2G051782_P01	TRUE	TRUE	qLFHPEQLISHk	95%	Pyro-cmC (-17), n+304 (+304), K+304 (+304)	29.21	25.25
6369	seq=translation; coord=1:283086410..283088116:1; parent_transcript=GRMZM2G065083_T01; parent_gene=GRMZM2G065083	GRMZM2G065083_P01	TRUE	TRUE	gNRPSLSLLLPTLSAYEVGQLLSIYEHR	95%	n+304 (+304)	52.39	25.00
6370	seq=translation; coord=1:283086410..283088116:1; parent_transcript=GRMZM2G065083_T01; parent_gene=GRMZM2G065083	GRMZM2G065083_P01	TRUE	TRUE	vPENLIPHk	95%	n+304 (+304), K+304 (+304)	32.94	25.76
6371	seq=translation; coord=5:184168891..184173292:1; parent_transcript=GRMZM2G071846_T01; parent_gene=GRMZM2G071846	GRMZM2G071846_P01	TRUE	TRUE	gFIFGPAVALAIGAk	95%	n+304 (+304), K+304 (+304)	32.66	25.00
6372	seq=translation; coord=5:184168891..184173292:1; parent_transcript=GRMZM2G071846_T01; parent_gene=GRMZM2G071846	GRMZM2G071846_P01	TRUE	TRUE	hGIMFNDITTLLLRPR	95%	n+304 (+304)	30.67	25.24
6373	seq=translation; coord=2:189499850..189501644:1; parent_transcript=GRMZM2G080466_T01; parent_gene=GRMZM2G080466	GRMZM2G080466_P01,GRMZM2G080466_P02, GRMZM2G080466_P03,GRMZM2G080466_P04	TRUE	TRUE	aEPAAEVAEk	95%	n+304 (+304), K+304 (+304)	40.96	25.74
6374	seq=translation; coord=2:189499850..189501644:1; parent_transcript=GRMZM2G080466_T01; parent_gene=GRMZM2G080466	GRMZM2G080466_P01,GRMZM2G080466_P02, GRMZM2G080466_P03,GRMZM2G080466_P04	TRUE	TRUE	eAEPEPAAAGEVk	95%	n+304 (+304), K+304 (+304)	52.22	25.66
6375	seq=translation; coord=1:183983464..183984705:-1; parent_transcript=GRMZM2G080724_T01; parent_gene=GRMZM2G080724	GRMZM2G080724_P01	TRUE	TRUE	tIMDADDADTDTR	95%	n+304 (+304)	54.19	25.00
6376	seq=translation; coord=1:183983464..183984705:-1; parent_transcript=GRMZM2G080724_T01; parent_gene=GRMZM2G080724	GRMZM2G080724_P01	TRUE	TRUE	vELPENVDVER	95%	n+304 (+304)	45.90	25.00
6377	seq=translation; coord=2:29975938..29981822:1; parent_transcript=GRMZM2G082581_T01; parent_gene=GRMZM2G082581	GRMZM2G082581_P01	TRUE	TRUE	aFAESSMAMALSk	95%	n+304 (+304), K+304 (+304)	29.10	25.07
6378	seq=translation; coord=2:29975938..29981822:1; parent_transcript=GRMZM2G082581_T01; parent_gene=GRMZM2G082581	GRMZM2G082581_P01	TRUE	TRUE	eFyLSTEEFIDAVAQQLR	95%	n+304 (+304), iTRAQ8plex (+304)	40.26	25.13
6379	seq=translation; coord=8:137403695..137407159:-1; parent_transcript=GRMZM2G106960_T01; parent_gene=GRMZM2G106960	GRMZM2G106960_P01	TRUE	TRUE	aLADDIGIPFLETSAk	95%	n+304 (+304), K+304 (+304)	57.32	26.06
6380	seq=translation; coord=8:137403695..137407159:-1; parent_transcript=GRMZM2G106960_T01; parent_gene=GRMZM2G106960	GRMZM2G106960_P01	TRUE	TRUE	gAHGIIVVYDVTDQESFNNVk	95%	n+304 (+304), K+304 (+304)	33.11	25.54
6381	seq=translation; coord=5:13615570..13621897:1; parent_transcript=GRMZM2G161222_T03; parent_gene=GRMZM2G161222	GRMZM2G161222_P03	TRUE	TRUE	gVIFPIIFEALER	95%	n+304 (+304)	29.79	25.49
6382	seq=translation; coord=5:13615570..13621897:1; parent_transcript=GRMZM2G161222_T03; parent_gene=GRMZM2G161222	GRMZM2G161222_P03	TRUE	TRUE	lVAALFDLSDSLk	95%	n+304 (+304), K+304 (+304)	30.33	25.00

6383	seq=translation; coord=9:92025063..92027986:-1; parent_transcript=GRMZM2G316232_T01; parent_gene=GRMZM2G316232	GRMZM2G316232_P01	TRUE	TRUE	lADGAVLIVDAAEGVMVNTER	95%	n+304 (+304)	61.49	25.48
6384	seq=translation; coord=9:92025063..92027986:-1; parent_transcript=GRMZM2G316232_T01; parent_gene=GRMZM2G316232	GRMZM2G316232_P01	TRUE	TRUE	sFVEFILEPLYk	95%	n+304 (+304), K+304 (+304)	34.45	25.00
6385	seq=translation; coord=10:117993529..118000841:-1; parent_transcript=GRMZM2G353213_T01; parent_gene=GRMZM2G353213	GRMZM2G353213_P01,GRMZM2G353213_P02	TRUE	TRUE	fAFVDVSPEELQQk	95%	n+304 (+304), K+304 (+304)	32.40	25.89
6386	seq=translation; coord=10:117993529..118000841:-1; parent_transcript=GRMZM2G353213_T01; parent_gene=GRMZM2G353213	GRMZM2G353213_P01,GRMZM2G353213_P02	TRUE	TRUE	nLQSSPETVDVk	95%	n+304 (+304), K+304 (+304)	36.09	25.85
6387	seq=translation; coord=9:137069559..137072594:-1; parent_transcript=GRMZM2G053652_T01; parent_gene=GRMZM2G053652	GRMZM2G053652_P01,GRMZM2G053652_P02	TRUE	TRUE	lDTYTTVYR	95%	n+304 (+304)	30.31	25.08
6388	seq=translation; coord=9:137069559..137072594:-1; parent_transcript=GRMZM2G053652_T01; parent_gene=GRMZM2G053652	GRMZM2G053652_P01,GRMZM2G053652_P02	TRUE	TRUE	tLTAVHDGILEDVVFPAEIVGk	95%	n+304 (+304), K+304 (+304)	31.96	25.00
6389	seq=translation; coord=1:297376727..297379573:-1; parent_transcript=GRMZM2G369815_T01; parent_gene=GRMZM2G369815	GRMZM2G369815_P01,GRMZM2G369815_P02	TRUE	TRUE	aDDRDAVGcAASVNR	95%	n+304 (+304), Carbamidomethyl (+57)	29.18	25.00
6390	seq=translation; coord=1:297376727..297379573:-1; parent_transcript=GRMZM2G369815_T01; parent_gene=GRMZM2G369815	GRMZM2G369815_P01,GRMZM2G369815_P02	TRUE	TRUE	vTAFVEGLLER	95%	n+304 (+304)	43.81	25.00
6391	seq=translation; coord=3:221687099..221691249:1; parent_transcript=GRMZM2G071021_T01; parent_gene=GRMZM2G071021	GRMZM2G071021_P01	TRUE	TRUE	dIPGAAHLLR	95%	n+304 (+304)	59.41	25.00
6392	seq=translation; coord=4:159862612..159866537:1; parent_transcript=GRMZM2G050371_T01; parent_gene=GRMZM2G050371	GRMZM2G050371_P01	TRUE	TRUE	gFEPDANTVEIAR	95%	n+304 (+304)	29.82	25.00
6393	seq=translation; coord=4:159862612..159866537:1; parent_transcript=GRMZM2G050371_T01; parent_gene=GRMZM2G050371	GRMZM2G050371_P01	TRUE	TRUE	vFAHQDILDLAk	95%	n+304 (+304), K+304 (+304)	43.34	25.65
6394	seq=translation; coord=8:17388792..17393555:-1; parent_transcript=GRMZM2G061969_T01; parent_gene=GRMZM2G061969	GRMZM2G061969_P01	TRUE	TRUE	dLSDIIIPPSPVMFPEDR	95%	n+304 (+304)	48.59	25.04
6395	seq=translation; coord=8:17388792..17393555:-1; parent_transcript=GRMZM2G061969_T01; parent_gene=GRMZM2G061969	GRMZM2G061969_P01	TRUE	TRUE	sIQDAYINAIR	94%	n+304 (+304)	30.59	25.00
6396	seq=translation; coord=5:215652783..215654639:1; parent_transcript=GRMZM2G143627_T01; parent_gene=GRMZM2G143627	GRMZM2G143627_P01,GRMZM2G143627_P02	TRUE	TRUE	gADPYDLk	94%	n+304 (+304), K+304 (+304)	31.83	26.35
6397	seq=translation; coord=5:215652783..215654639:1; parent_transcript=GRMZM2G143627_T01; parent_gene=GRMZM2G143627	GRMZM2G143627_P01,GRMZM2G143627_P02	TRUE	TRUE	qQENVLAESR	89%	n+304 (+304)	26.86	25.00
6398	seq=translation; coord=1:286506118..286513080:-1; parent_transcript=GRMZM2G141473_T01; parent_gene=GRMZM2G141473	GRMZM2G141473_P01	TRUE	TRUE	lPGYDSGAVcTFPEFLk	93%	n+304 (+304), Carbamidomethyl (+57), K+304 (+304)	27.79	25.05
6399	seq=translation; coord=1:286506118..286513080:-1; parent_transcript=GRMZM2G141473_T01; parent_gene=GRMZM2G141473	GRMZM2G141473_P01	TRUE	TRUE	vGGGFGGk	86%	n+304 (+304), K+304 (+304)	25.97	25.85
6400	seq=translation; coord=5:18753627..18756558:1; parent_transcript=GRMZM2G138572_T01; parent_gene=GRMZM2G138572	GRMZM2G138572_P01	TRUE	TRUE	iLHAAFVPFGEVk	95%	n+304 (+304), K+304 (+304)	32.33	25.00

6401	seq=translation; coord=5:18753627..18756558:1; parent_transcript=GRMZM2G138572_T01; parent_gene=GRMZM2G138572	GRMZM2G138572_P01	TRUE	TRUE	sFGFVTFLER	89%	n+304 (+304)	27.32	25.71
6402	seq=translation; coord=4:26401952..26408946:-1; parent_transcript=GRMZM2G003814_T01; parent_gene=GRMZM2G003814	GRMZM2G003814_P01	TRUE	TRUE	aVVYDEHGLPEQVLR	92%	n+304 (+304)	27.37	25.56
6403	seq=translation; coord=1:35577459..35584230:-1; parent_transcript=GRMZM2G127123_T01; parent_gene=GRMZM2G127123	GRMZM2G127123_P01,GRMZM2G127123_P03	TRUE	TRUE	fTPPAGDDPVAIDFTGMGk	95%	n+304 (+304), K+304 (+304)	49.48	25.25
6404	seq=translation; coord=2:3969379..3974004:1; parent_transcript=GRMZM2G056572_T01; parent_gene=GRMZM2G056572	GRMZM2G056572_P01,GRMZM2G056572_P02, GRMZM2G056572_P03,GRMZM2G056572_P04, GRMZM2G056572_P05	TRUE	TRUE	iDcVDGQIVGLFGVFDGHGGAk	95%	n+304 (+304), Carbamidomethyl (+57), K+304 (+304)	97.74	25.09
6405	seq=translation; coord=7:155584542..155586999:1; parent_transcript=GRMZM2G041381_T01; parent_gene=GRMZM2G041381	GRMZM2G041381_P01,GRMZM2G151826_P01	TRUE	TRUE	aIGAGAAk	95%	n+304 (+304), K+304 (+304)	34.19	25.37
6406	seq=translation; coord=9:37699870..37701617:1; parent_transcript=GRMZM2G008539_T01; parent_gene=GRMZM2G008539	GRMZM2G008539_P01,GRMZM2G099740_P01	TRUE	TRUE	gAVVDRDDAGELSELLGEAFLR	95%	n+304 (+304)	46.14	25.00
6407	seq=translation; coord=4:24613100..24615973:-1; parent_transcript=GRMZM2G101480_T01; parent_gene=GRMZM2G101480	GRMZM2G101480_P01,GRMZM2G101480_P02, GRMZM2G101480_P04,GRMZM5G800112_P01, GRMZM5G800112_P02	TRUE	TRUE	rLDDDTENLAHER	95%	n+304 (+304)	38.39	25.00
6408	seq=translation; coord=7:165751276..165754416:-1; parent_transcript=GRMZM2G053939_T01; parent_gene=GRMZM2G053939	GRMZM2G053939_P01	TRUE	TRUE	eVLSLcDHPALLDk	95%	n+304 (+304), Carbamidomethyl (+57), K+304 (+304)	28.17	25.60
6409	seq=translation; coord=4:206497947..206499008:-1; parent_transcript=GRMZM2G059706_T01; parent_gene=GRMZM2G059706	GRMZM2G059706_P01,GRMZM2G178199_P01	TRUE	TRUE	eMSIVGGSGk	95%	n+304 (+304), K+304 (+304)	45.23	26.23
6410	seq=translation; coord=8:104902929..104904891:1; parent_transcript=GRMZM2G044440_T01; parent_gene=GRMZM2G044440	GRMZM2G044440_P01	TRUE	TRUE	mQIFVLMLTGk	95%	n+304 (+304), K+304 (+304)	31.47	25.45
6411	seq=translation; coord=10:2446094..2450352:1; parent_transcript=GRMZM2G151977_T01; parent_gene=GRMZM2G151977	GRMZM2G151977_P01	TRUE	TRUE	sAGSPLSSLLGR	94%	n+304 (+304)	29.64	25.19
6412	seq=translation; coord=2:26769801..26771736:1; parent_transcript=GRMZM2G474575_T01; parent_gene=GRMZM2G474575	GRMZM2G474575_P01	TRUE	TRUE	aHAVVFQk	91%	n+304 (+304), K+304 (+304)	25.13	25.00
6413	seq=translation; coord=2:26769801..26771736:1; parent_transcript=GRMZM2G474575_T01; parent_gene=GRMZM2G474575	GRMZM2G474575_P01	TRUE	TRUE	aWLGTTETDR	89%	n+304 (+304)	25.99	25.00
6414	seq=translation; coord=1:110974309..110976210:1; parent_transcript=GRMZM2G154216_T01; parent_gene=GRMZM2G154216	GRMZM2G154216_P01	TRUE	TRUE	iGEDELDLGWAER	95%	n+304 (+304)	33.04	25.00
6415	seq=translation; coord=3:3459125..3462998:1; parent_transcript=GRMZM2G102572_T01; parent_gene=GRMZM2G102572	GRMZM2G102572_P01,GRMZM2G102572_P02, GRMZM2G102572_P03	TRUE	TRUE	iLFEEVVk	94%	n+304 (+304), K+304 (+304)	28.59	25.00
6416	seq=translation; coord=9:101389917..101393912:1; parent_transcript=GRMZM2G000566_T01; parent_gene=GRMZM2G000566	GRMZM2G000566_P01	TRUE	TRUE	aEFDWLALYR	92%	n+304 (+304)	28.46	25.00
6417	seq=translation; coord=8:164932122..164937568:1; parent_transcript=GRMZM2G049269_T01; parent_gene=GRMZM2G049269	GRMZM2G049269_P01	TRUE	TRUE	lAAVFAEVDR	90%	n+304 (+304)	27.70	25.43
6418	seq=translation; coord=5:159269189..159288759:1; parent_transcript=GRMZM2G135691_T01; parent_gene=GRMZM2G135691	GRMZM2G135691_P01,GRMZM2G135691_P02	TRUE	TRUE	aYLTLEELR	95%	n+304 (+304)	42.37	25.51

6419	seq=translation; coord=4:149744485..149746284:1; parent_transcript=GRMZM2G065021_T01; parent_gene=GRMZM2G065021	GRMZM2G065021_P01	TRUE	TRUE	aAAEESTEQWVPFVR	95%	n+304 (+304)	72.73	25.00
6420	seq=translation; coord=4:143464625..143469700:-1; parent_transcript=GRMZM2G081582_T01; parent_gene=GRMZM2G081582	GRMZM2G081582_P01	TRUE	TRUE	lALAAIGk	92%	n+304 (+304), K+304 (+304)	26.46	25.00
6421	seq=translation; coord=10:84755864..84760724:-1; parent_transcript=GRMZM2G136341_T01; parent_gene=GRMZM2G136341	GRMZM2G136341_P01	TRUE	TRUE	lLEEALGk	95%	n+304 (+304), K+304 (+304)	28.53	25.00
6422	seq=translation; coord=1:5873406..5877213:1; parent_transcript=GRMZM2G105571_T01; parent_gene=GRMZM2G105571	GRMZM2G105571_P01,GRMZM2G105571_P02	TRUE	TRUE	aVAGAGVLAGYDk	95%	n+304 (+304), K+304 (+304)	33.11	25.47
6423	seq=translation; coord=1:5873406..5877213:1; parent_transcript=GRMZM2G105571_T01; parent_gene=GRMZM2G105571	GRMZM2G105571_P01,GRMZM2G105571_P02	TRUE	TRUE	dEGFVSLWR	89%	n+304 (+304)	26.56	25.00
6424	seq=translation; coord=3:59842989..59850482:-1; parent_transcript=GRMZM2G462140_T01; parent_gene=GRMZM2G462140	GRMZM2G462140_P01	TRUE	TRUE	sPFTSGIGLETLGVETDk	95%	n+304 (+304), K+304 (+304)	68.47	25.42
6425	seq=translation; coord=9:123408167..123411376:-1; parent_transcript=GRMZM2G124317_T01; parent_gene=GRMZM2G124317	GRMZM2G124317_P01,GRMZM2G124317_P02, GRMZM2G124317_P03,GRMZM2G124317_P07, GRMZM2G124317_P08,GRMZM2G124317_P09, GRMZM2G172244_P01,GRMZM2G172244_P02, GRMZM2G172244_P03	TRUE	TRUE	vHAAPGGGSSLGYLFGGN	95%	n+304 (+304)	33.97	25.12
6426	seq=translation; coord=9:123408167..123411376:-1; parent_transcript=GRMZM2G124317_T01; parent_gene=GRMZM2G124317	GRMZM2G124317_P01,GRMZM2G124317_P02, GRMZM2G124317_P03,GRMZM2G124317_P07, GRMZM2G124317_P08,GRMZM2G124317_P09, GRMZM2G172244_P01,GRMZM2G172244_P02, GRMZM2G172244_P03	TRUE	TRUE	vHAAPGGGSSLGyLFGGN	92%	n+304 (+304), iTRAQ8plex (+304)	26.28	25.38
6427	seq=translation; coord=4:172080242..172084381:1; parent_transcript=GRMZM2G075336_T01; parent_gene=GRMZM2G075336	GRMZM2G075336_P01,GRMZM2G075336_P02, GRMZM2G075336_P03	TRUE	TRUE	nLLSVVESWLR	95%	n+304 (+304)	39.15	25.28
6428	seq=translation; coord=5:179392458..179394688:1; parent_transcript=GRMZM2G003384_T01; parent_gene=GRMZM2G003384	GRMZM2G003384_P01	TRUE	TRUE	aVDAALIk	95%	n+304 (+304), K+304 (+304)	42.78	26.07
6429	seq=translation; coord=1:41759285..41762638:-1; parent_transcript=GRMZM2G119886_T01; parent_gene=GRMZM2G119886	GRMZM2G119886_P01	TRUE	TRUE	eTPGSGAPAPAR	86%	n+304 (+304)	25.18	25.00
6430	seq=translation; coord=5:13048033..13051225:1; parent_transcript=AC213463.3_FGT007; parent_gene=AC213463.3_FG007	AC213463.3_FGP007,GRMZM2G038217_P01,G RMZM2G038217_P02,GRMZM2G389510_P01	TRUE	TRUE	gAISDLSGVQAk	95%	n+304 (+304), K+304 (+304)	75.27	25.33
6431	seq=translation; coord=6:162690848..162694219:1; parent_transcript=GRMZM2G017269_T01; parent_gene=GRMZM2G017269	GRMZM2G017269_P01,GRMZM2G095631_P01	TRUE	TRUE	lQELLEPLVPLIVk	95%	n+304 (+304), K+304 (+304)	39.05	25.00
6432	seq=translation; coord=2:156178938..156194571:-1; parent_transcript=GRMZM2G057910_T01; parent_gene=GRMZM2G057910	GRMZM2G057910_P01	TRUE	TRUE	dVAVLEAMLESSAk	95%	n+304 (+304), K+304 (+304)	38.53	26.23
6433	seq=translation; coord=2:3556786..3558729:1; parent_transcript=GRMZM2G062396_T01; parent_gene=GRMZM2G062396	GRMZM2G062396_P01	TRUE	TRUE	hTDPGTITLLLQDLVGGLQATR	95%	n+304 (+304)	32.30	25.00
6434	seq=translation; coord=4:63536096..63539819:-1; parent_transcript=GRMZM2G082087_T02; parent_gene=GRMZM2G082087	GRMZM2G082087_P02,GRMZM2G148281_P01, GRMZM2G148281_P02	TRUE	TRUE	gAIFFcQLWHVGR	95%	n+304 (+304), Carbamidomethyl (+57)	63.88	25.00

6435	seq=translation; coord=2:4624358..4637151:1; parent_transcript=GRMZM2G086489_T01; parent_gene=GRMZM2G086489	GRMZM2G086489_P01,GRMZM2G086489_P02, GRMZM2G104294_P01,GRMZM2G104294_P02	TRUE	TRUE	sLLEVMAAFPSAkPPLGVFFAAIAPR	95%	n+304 (+304), K+304 (+304)	31.32	25.00
6436	seq=translation; coord=1:7341979..7348599:1; parent_transcript=GRMZM2G096020_T01; parent_gene=GRMZM2G096020	GRMZM2G096020_P01,GRMZM2G096020_P02, GRMZM2G096020_P03	TRUE	TRUE	sLISMVQIIMSR	95%	n+304 (+304)	33.97	25.92
6437	seq=translation; coord=5:9908227..9911250:-1; parent_transcript=GRMZM2G096591_T01; parent_gene=GRMZM2G096591	GRMZM2G096591_P01,GRMZM2G096591_P02	TRUE	TRUE	aFVSGLVSHLR	95%	n+304 (+304)	73.55	25.54
6438	seq=translation; coord=1:296260884..296265865:-1; parent_transcript=GRMZM2G113250_T01; parent_gene=GRMZM2G113250	GRMZM2G113250_P01	TRUE	TRUE	eQMIPLSEFEDkL	95%	n+304 (+304), K+304 (+304)	34.43	25.00
6439	seq=translation; coord=10:10551726..10552620:1; parent_transcript=GRMZM2G132273_T01; parent_gene=GRMZM2G132273	GRMZM2G132273_P01,GRMZM2G320023_P01	TRUE	TRUE	eMAVVGGTGAFR	94%	n+304 (+304)	30.44	25.00
6440	seq=translation; coord=6:109829372..109834694:1; parent_transcript=GRMZM2G136058_T01; parent_gene=GRMZM2G136058	GRMZM2G136058_P01,GRMZM2G414569_P01	TRUE	TRUE	iEGVELELR	95%	n+304 (+304)	41.42	26.94
6441	seq=translation; coord=1:6546908..6550733:-1; parent_transcript=GRMZM2G137312_T01; parent_gene=GRMZM2G137312	GRMZM2G137312_P01	TRUE	TRUE	vAEFYLPWk	94%	n+304 (+304), K+304 (+304)	28.64	25.71
6442	seq=translation; coord=1:107185706..107188803:1; parent_transcript=GRMZM2G144155_T01; parent_gene=GRMZM2G144155	GRMZM2G144155_P01,GRMZM2G144155_P02, GRMZM2G144155_P03,GRMZM2G144155_P04	TRUE	TRUE	gIPAILGIISTQR	95%	n+304 (+304)	42.36	25.00
6443	seq=translation; coord=6:130597982..130600487:-1; parent_transcript=GRMZM2G149201_T01; parent_gene=GRMZM2G149201	GRMZM2G149201_P01	TRUE	TRUE	lVGAFPAVVLELPALk	95%	n+304 (+304), K+304 (+304)	31.36	25.00
6444	seq=translation; coord=1:38008533..38011706:1; parent_transcript=GRMZM2G151564_T01; parent_gene=GRMZM2G151564	GRMZM2G151564_P01	TRUE	TRUE	gLSLLGLLR	95%	n+304 (+304)	33.17	25.00
6445	seq=translation; coord=5:74616842..74620998:-1; parent_transcript=GRMZM2G168762_T01; parent_gene=GRMZM2G168762	GRMZM2G168762_P01	TRUE	TRUE	nMATLLAEFDk	94%	n+304 (+304), K+304 (+304)	28.49	25.58
6446	seq=translation; coord=2:30874561..30879119:-1; parent_transcript=GRMZM2G170101_T01; parent_gene=GRMZM2G170101	GRMZM2G170101_P01,GRMZM2G170101_P03	TRUE	TRUE	gFIFGPAIALAIGAk	95%	n+304 (+304), K+304 (+304)	39.40	25.00
6447	seq=translation; coord=2:233597234..233599119:1; parent_transcript=GRMZM2G176394_T01; parent_gene=GRMZM2G176394	GRMZM2G176394_P01,GRMZM2G176394_P02, GRMZM2G176394_P03,GRMZM2G476294_P01	TRUE	TRUE	mAGTLAELVALNSGLR	95%	n+304 (+304)	50.48	25.99
6448	seq=translation; coord=6:157564314..157569386:1; parent_transcript=GRMZM2G305115_T01; parent_gene=GRMZM2G305115	GRMZM2G305115_P01,GRMZM2G305115_P03	TRUE	TRUE	fDDGWDSR	95%	n+304 (+304)	34.09	25.00
6449	seq=translation; coord=2:164472793..164474499:-1; parent_transcript=GRMZM2G341253_T01; parent_gene=GRMZM2G341253	GRMZM2G341253_P01,GRMZM2G436404_P01	TRUE	TRUE	sVVADPDDLR	95%	n+304 (+304)	32.90	25.00
6450	seq=translation; coord=5:99408290..99409902:-1; parent_transcript=GRMZM2G417945_T01; parent_gene=GRMZM2G417945	GRMZM2G417945_P01	TRUE	TRUE	lLVDAIIAQFHTIDR	95%	n+304 (+304)	73.57	25.38
6451	seq=translation; coord=3:1295431..1296371:1; parent_transcript=GRMZM5G860761_T02; parent_gene=GRMZM5G860761	GRMZM5G860761_P02	TRUE	TRUE	gGPcYYPDNLVSR	95%	n+304 (+304), Carbamidomethyl (+57)	36.87	25.00
6452	GRMZM2G155232_P01-R	GRMZM2G155232_P01-R,GRMZM2G301803_P03-R,GRMZM2G480712_P01-R	TRUE	TRUE	lEESLk	90%	n+304 (+304), K+304 (+304)	27.39	26.02

6453	seq=translation; coord=2:213386026..213389632:1; parent_transcript=GRMZM2G119675_T01; parent_gene=GRMZM2G119675	GRMZM2G119675_P01,GRMZM5G885744_P01, GRMZM5G885744_P02	TRUE	TRUE	tFDPWPVFFR	94%	n+304 (+304)	30.98	25.11
6454	seq=translation; coord=2:197756965..197760720:1; parent_transcript=AC233910.1_FGT005; parent_gene=AC233910.1_FG005	AC233910.1_FGP005	TRUE	TRUE	lEVPDGAVFENk	95%	n+304 (+304), K+304 (+304)	31.06	25.93
6455	seq=translation; coord=7:167230471..167241954:1; parent_transcript=GRMZM2G013811_T01; parent_gene=GRMZM2G013811	GRMZM2G013811_P01	TRUE	TRUE	gPHGIGDLGDEALAFLHWLR	95%	n+304 (+304)	66.62	25.09
6456	seq=translation; coord=7:33007300..33009101:-1; parent_transcript=GRMZM2G042692_T01; parent_gene=GRMZM2G042692	GRMZM2G042692_P01	TRUE	TRUE	lEGVGEVTVDSR	95%	n+304 (+304)	32.19	25.91
6457	seq=translation; coord=3:29472800..29479329:1; parent_transcript=GRMZM2G051622_T01; parent_gene=GRMZM2G051622	GRMZM2G051622_P01	TRUE	TRUE	iNTEESSIGLR	88%	n+304 (+304)	26.30	25.82
6458	seq=translation; coord=5:76624902..76632274:1; parent_transcript=GRMZM2G054765_T01; parent_gene=GRMZM2G054765	GRMZM2G054765_P01	TRUE	TRUE	yLTEPAVAVILDLVR	95%	n+304 (+304)	52.16	25.00
6459	seq=translation; coord=6:123603678..123607103:1; parent_transcript=GRMZM2G066612_T01; parent_gene=GRMZM2G066612	GRMZM2G066612_P01,GRMZM2G159145_P01, GRMZM2G159145_P02	TRUE	TRUE	aTPTFFFLk	95%	n+304 (+304), K+304 (+304)	28.71	25.00
6460	seq=translation; coord=2:124094006..124108029:-1; parent_transcript=GRMZM2G075680_T01; parent_gene=GRMZM2G075680	GRMZM2G075680_P01	TRUE	TRUE	eSSVIIYETR	95%	n+304 (+304)	30.96	25.08
6461	seq=translation; coord=10:145183272..145197508:1; parent_transcript=GRMZM2G086030_T01; parent_gene=GRMZM2G086030	GRMZM2G086030_P01	TRUE	TRUE	aFLLHNLFVDTAIFR	95%	n+304 (+304)	30.67	25.85
6462	seq=translation; coord=5:5616049..5616896:-1; parent_transcript=GRMZM2G091535_T02; parent_gene=GRMZM2G091535	GRMZM2G091535_P02	TRUE	TRUE	mVTPAPVAMTDPPGALEk	95%	n+304 (+304), K+304 (+304)	36.62	25.26
6463	seq=translation; coord=5:50092687..50097671:-1; parent_transcript=GRMZM2G113613_T01; parent_gene=GRMZM2G113613	GRMZM2G113613_P01	TRUE	TRUE	rDDLSSLNPPVMEGLDALLAk	95%	n+304 (+304), K+304 (+304)	41.04	25.56
6464	seq=translation; coord=1:277843773..277846597:-1; parent_transcript=GRMZM2G123714_T01; parent_gene=GRMZM2G123714	GRMZM2G123714_P01,GRMZM2G123714_P02	TRUE	TRUE	sDLADSALQk	95%	n+304 (+304), K+304 (+304)	31.95	25.47
6465	seq=translation; coord=8:91831315..91844753:1; parent_transcript=GRMZM2G124455_T01; parent_gene=GRMZM2G124455	GRMZM2G124455_P01	TRUE	TRUE	aLEQLDAAVAk	95%	n+304 (+304), K+304 (+304)	34.18	25.16
6466	seq=translation; coord=2:47422297..47424522:1; parent_transcript=GRMZM2G151021_T01; parent_gene=GRMZM2G151021	GRMZM2G151021_P01,GRMZM2G166484_P01	TRUE	TRUE	vAAQPQLVLAR	95%	n+304 (+304)	38.43	25.00
6467	seq=translation; coord=8:128348231..128358933:-1; parent_transcript=GRMZM2G170798_T01; parent_gene=GRMZM2G170798	GRMZM2G170798_P01	TRUE	TRUE	aIEGEGAIAVLDTAAGVWLDR	95%	n+304 (+304)	39.14	25.61
6468	seq=translation; coord=9:15978102..15983381:1; parent_transcript=GRMZM2G304362_T01; parent_gene=GRMZM2G304362	GRMZM2G304362_P01	TRUE	TRUE	sGLMAPLVAEDVYEIIMk	95%	n+304 (+304), K+304 (+304)	43.61	25.93
6469	seq=translation; coord=1:101830081..101832342:-1; parent_transcript=GRMZM2G456626_T01; parent_gene=GRMZM2G456626	GRMZM2G456626_P01	TRUE	TRUE	sLEDAVVPPAAFINQIAGGLR	95%	n+304 (+304)	66.26	26.20
6470	seq=translation; coord=8:143006137..143009764:-1; parent_transcript=GRMZM5G803433_T01; parent_gene=GRMZM5G803433	GRMZM5G803433_P01	TRUE	TRUE	dQLADIAAAAALVSAHALDAVR	95%	n+304 (+304)	88.15	25.60

6471	seq=translation; coord=1:194570543..194573682:-1; parent_transcript=GRMZM2G136534_T01; parent_gene=GRMZM2G136534	GRMZM2G136534_P01,GRMZM2G136534_P03	TRUE	TRUE	dScPGLESIVR	90%	n+304 (+304), Carbamidomethyl (+57)	27.08	25.00
6472	seq=translation; coord=4:198078696..198080533:-1; parent_transcript=GRMZM2G332522_T01; parent_gene=GRMZM2G332522	GRMZM2G332522_P01,GRMZM2G332522_P02	TRUE	TRUE	vIAFDVSR	89%	n+304 (+304)	27.00	25.00
6473	seq=translation; coord=7:55631624..55634493:-1; parent_transcript=GRMZM2G058484_T01; parent_gene=GRMZM2G058484	GRMZM2G058484_P01,GRMZM2G058484_P02, GRMZM2G058484_P03,GRMZM2G058484_P04	TRUE	TRUE	qMISIIk	87%	Pyro-cmC (-17), n+304 (+304), K+304 (+304)	27.15	26.64
6474	GRMZM2G322723_P02-R	GRMZM2G322723_P02-R	TRUE	TRUE	lLAPDEEGk	88%	n+304 (+304), K+304 (+304)	25.54	25.00
6475	GRMZM2G022787_P01-R	GRMZM2G022787_P01-R	TRUE	TRUE	eLLEGVR	87%	n+304 (+304)	25.12	25.00
6476	seq=translation; coord=7:110983097..110986540:-1; parent_transcript=GRMZM2G048277_T01; parent_gene=GRMZM2G048277	GRMZM2G048277_P01,GRMZM2G048277_P02, GRMZM2G048277_P03	TRUE	TRUE	kGGDLGWFPR	95%	K+304 (+304), n+304 (+304)	28.78	26.47
6477	seq=translation; coord=7:110983097..110986540:-1; parent_transcript=GRMZM2G048277_T01; parent_gene=GRMZM2G048277	GRMZM2G048277_P01,GRMZM2G048277_P02, GRMZM2G048277_P03	TRUE	TRUE	mAGPFQEVAFNTPEGTVSAPFk	95%	n+304 (+304), K+304 (+304)	30.67	25.00
6478	seq=translation; coord=7:110983097..110986540:-1; parent_transcript=GRMZM2G048277_T01; parent_gene=GRMZM2G048277	GRMZM2G048277_P01,GRMZM2G048277_P02, GRMZM2G048277_P03	TRUE	TRUE	sADGLGTcTYVk	95%	n+304 (+304), Carbamidomethyl (+57), K+304 (+304)	65.77	25.00
6479	seq=translation; coord=8:42281387..42289748:-1; parent_transcript=GRMZM2G145595_T01; parent_gene=GRMZM2G145595	GRMZM2G145595_P01	TRUE	TRUE	aLGVDILTGIGTIVGk	95%	n+304 (+304), K+304 (+304)	44.02	25.00
6480	seq=translation; coord=8:42281387..42289748:-1; parent_transcript=GRMZM2G145595_T01; parent_gene=GRMZM2G145595	GRMZM2G145595_P01	TRUE	TRUE	vGFPDNEITAR	95%	n+304 (+304)	37.70	25.00
6481	seq=translation; coord=10:107762378..107767126:1; parent_transcript=GRMZM2G028945_T01; parent_gene=GRMZM2G028945	GRMZM2G028945_P01,GRMZM2G104649_P03, GRMZM2G104649_P04	TRUE	TRUE	lSWVDIFEEIPIk	95%	n+304 (+304), K+304 (+304)	39.15	25.92
6482	seq=translation; coord=10:107762378..107767126:1; parent_transcript=GRMZM2G028945_T01; parent_gene=GRMZM2G028945	GRMZM2G028945_P01,GRMZM2G104649_P03, GRMZM2G104649_P04	TRUE	TRUE	vVQMEGLAVLk	95%	n+304 (+304), K+304 (+304)	33.57	25.00
6483	seq=translation; coord=1:31242217..31244585:-1; parent_transcript=GRMZM2G116846_T01; parent_gene=GRMZM2G116846	GRMZM2G116846_P01	TRUE	TRUE	aALEEAcPGVVScADILAIAAk	93%	n+304 (+304), Carbamidomethyl (+57), Carbamidomethyl (+57), K+304 (+304)	27.49	25.39
6484	seq=translation; coord=1:31242217..31244585:-1; parent_transcript=GRMZM2G116846_T01; parent_gene=GRMZM2G116846	GRMZM2G116846_P01	TRUE	TRUE	fGAVGLDDTDLVALSGAHTFGR	95%	n+304 (+304)	76.00	25.00
6485	seq=translation; coord=1:31242217..31244585:-1; parent_transcript=GRMZM2G116846_T01; parent_gene=GRMZM2G116846	GRMZM2G116846_P01	TRUE	TRUE	rGTLQSDQELLSTPGAPTAPIVGR	95%	n+304 (+304)	29.14	25.00
6486	seq=translation; coord=1:292354165..292360031:-1; parent_transcript=GRMZM2G151967_T01; parent_gene=GRMZM2G151967	GRMZM2G151967_P01	TRUE	TRUE	aSASAVAFGLGLLSGk	95%	n+304 (+304), K+304 (+304)	39.46	25.00
6487	seq=translation; coord=1:292354165..292360031:-1; parent_transcript=GRMZM2G151967_T01; parent_gene=GRMZM2G151967	GRMZM2G151967_P01	TRUE	TRUE	dVVQSMEEAIIAR	88%	n+304 (+304)	27.67	26.22
6488	seq=translation; coord=1:292354165..292360031:-1; parent_transcript=GRMZM2G151967_T01; parent_gene=GRMZM2G151967	GRMZM2G151967_P01	TRUE	TRUE	vSSFFLGLLSQk	95%	n+304 (+304), K+304 (+304)	30.46	25.17
6489	seq=translation; coord=2:110482584..110497021:-1; parent_transcript=GRMZM2G081745_T01; parent_gene=GRMZM2G081745	GRMZM2G081745_P01,GRMZM2G081745_P03, GRMZM2G143725_P01,GRMZM2G143725_P02	TRUE	TRUE	fLPLYILAIck	95%	n+304 (+304), Carbamidomethyl (+57), K+304 (+304)	34.12	25.00

6490	seq=translation; coord=2:110482584..110497021:-1; parent_transcript=GRMZM2G081745_T01; parent_gene=GRMZM2G081745	GRMZM2G081745_P01,GRMZM2G081745_P03, GRMZM2G143725_P01,GRMZM2G143725_P02	TRUE	TRUE	gGYADVSLDER	94%	n+304 (+304)	31.02	25.00
6491	seq=translation; coord=1:287631317..287634834:1; parent_transcript=GRMZM2G052471_T01; parent_gene=GRMZM2G052471	GRMZM2G052471_P01,GRMZM2G099474_P01	TRUE	TRUE	gPEEEGPk	94%	n+304 (+304), K+304 (+304)	29.28	25.90
6492	seq=translation; coord=3:35659936..35665816:1; parent_transcript=GRMZM2G091119_T01; parent_gene=GRMZM2G091119	GRMZM2G091119_P01	TRUE	TRUE	tMGATGDINVFAQMIDDAEGLEk	95%	n+304 (+304), K+304 (+304)	72.43	25.00
6493	seq=translation; coord=3:35659936..35665816:1; parent_transcript=GRMZM2G091119_T01; parent_gene=GRMZM2G091119	GRMZM2G091119_P01	TRUE	TRUE	tVGNIVTGDDQQTQcIIDHQALPR	95%	n+304 (+304), Carbamidomethyl (+57)	98.16	25.00
6494	seq=translation; coord=2:197623496..197625443:1; parent_transcript=GRMZM2G162284_T01; parent_gene=GRMZM2G162284	GRMZM2G162284_P01,GRMZM2G162284_P02, GRMZM2G162284_P03	TRUE	TRUE	rPPTAFFVFMEEFR	95%	n+304 (+304)	51.59	25.00
6495	seq=translation; coord=1:17933389..17954778:-1; parent_transcript=GRMZM2G059020_T01; parent_gene=GRMZM2G059020	GRMZM2G059020_P01	TRUE	TRUE	iLIFFQTk	94%	n+304 (+304), K+304 (+304)	27.97	25.00
6496	seq=translation; coord=1:17933389..17954778:-1; parent_transcript=GRMZM2G059020_T01; parent_gene=GRMZM2G059020	GRMZM2G059020_P01	TRUE	TRUE	tVMVVELIk	95%	n+304 (+304), K+304 (+304)	39.28	25.00
6497	seq=translation; coord=2:203135392..203142289:1; parent_transcript=GRMZM2G001820_T01; parent_gene=GRMZM2G001820	GRMZM2G001820_P01	TRUE	TRUE	eLLLFYk	95%	n+304 (+304), K+304 (+304)	27.74	25.00
6498	seq=translation; coord=2:177496379..177510526:-1; parent_transcript=GRMZM2G034848_T01; parent_gene=GRMZM2G034848	GRMZM2G034848_P01	TRUE	TRUE	iGMDVAASEFLTk	92%	n+304 (+304), K+304 (+304)	27.55	25.75
6499	seq=translation; coord=2:177496379..177510526:-1; parent_transcript=GRMZM2G034848_T01; parent_gene=GRMZM2G034848	GRMZM2G034848_P01	TRUE	TRUE	sGETEDNFIADLAVGLASGQIk	95%	n+304 (+304), K+304 (+304)	35.74	26.03
6500	seq=translation; coord=1:205410478..205412879:1; parent_transcript=GRMZM2G158462_T01; parent_gene=GRMZM2G158462	GRMZM2G158462_P01	TRUE	TRUE	eELDDAISALDGQELDGRPLR	90%	n+304 (+304)	25.36	25.00
6501	seq=translation; coord=7:4538433..4546019:1; parent_transcript=GRMZM2G480002_T01; parent_gene=GRMZM2G480002	GRMZM2G480002_P01	TRUE	TRUE	hDLTDDTEELFWGSPIR	95%	n+304 (+304)	32.29	25.00
6502	seq=translation; coord=7:4538433..4546019:1; parent_transcript=GRMZM2G480002_T01; parent_gene=GRMZM2G480002	GRMZM2G480002_P01	TRUE	TRUE	yTSVPSDDSTSLGFR	95%	n+304 (+304)	40.85	25.00
6503	seq=translation; coord=5:21571290..21578409:-1; parent_transcript=GRMZM2G073934_T01; parent_gene=GRMZM2G073934	GRMZM2G073934_P01	TRUE	TRUE	dALLEEAck	94%	n+304 (+304), Carbamidomethyl (+57), K+304 (+304)	28.85	25.25
6504	seq=translation; coord=5:21571290..21578409:-1; parent_transcript=GRMZM2G073934_T01; parent_gene=GRMZM2G073934	GRMZM2G073934_P01	TRUE	TRUE	sMLPLPYLk	95%	n+304 (+304), K+304 (+304)	36.40	25.00
6505	seq=translation; coord=4:194340222..194342295:-1; parent_transcript=AC190609.3_FGT002; parent_gene=AC190609.3_FG002	AC190609.3_FGP002,GRMZM2G069594_P01,G RMZM2G069594_P02,GRMZM2G069594_P03,G RMZM2G069594_P04,GRMZM2G069594_P06	TRUE	TRUE	dFLLTAR	95%	n+304 (+304)	33.65	25.00
6506	seq=translation; coord=4:194340222..194342295:-1; parent_transcript=AC190609.3_FGT002; parent_gene=AC190609.3_FG002	AC190609.3_FGP002,GRMZM2G069594_P01,G RMZM2G069594_P02,GRMZM2G069594_P03,G RMZM2G069594_P04,GRMZM2G069594_P06	TRUE	TRUE	yLYTLcVFDAEk	95%	n+304 (+304), Carbamidomethyl (+57), K+304 (+304)	36.63	25.05

6507	seq=translation; coord=2:63944481..63944981:-1; parent_transcript=AC196489.3_FGT002; parent_gene=AC196489.3_FG002	AC196489.3_FGP002	TRUE	TRUE	hSGNIGLDDVVEIAR	95%	n+304 (+304)	81.69	25.80
6508	seq=translation; coord=1:63573932..63578006:1; parent_transcript=AC216247.3_FGT005; parent_gene=AC216247.3_FG005	AC216247.3_FGP005,GRMZM2G117746_P01	TRUE	TRUE	mILGFAEGFPTMLk	95%	n+304 (+304), K+304 (+304)	37.98	25.50
6509	seq=translation; coord=5:82556313..82561535:-1; parent_transcript=GRMZM2G006144_T01; parent_gene=GRMZM2G006144	GRMZM2G006144_P01	TRUE	TRUE	nMVEFFDLLLSPASk	95%	n+304 (+304), K+304 (+304)	36.76	25.60
6510	seq=translation; coord=8:145082985..145087859:1; parent_transcript=GRMZM2G025387_T01; parent_gene=GRMZM2G025387	GRMZM2G025387_P01,GRMZM2G025387_P02, GRMZM2G058305_P01,GRMZM2G058305_P02, GRMZM5G856738_P01,GRMZM5G856738_P02, GRMZM5G856738_P03	TRUE	TRUE	eIIAEVDTDHDGR	95%	n+304 (+304)	53.28	25.00
6511	seq=translation; coord=8:145082985..145087859:1; parent_transcript=GRMZM2G025387_T01; parent_gene=GRMZM2G025387	GRMZM2G025387_P01,GRMZM2G025387_P02, GRMZM2G058305_P01,GRMZM2G058305_P02, GRMZM5G856738_P01,GRMZM5G856738_P02, GRMZM5G856738_P03	TRUE	TRUE	lTAAEILNHPWIR	95%	n+304 (+304)	70.26	26.12
6512	seq=translation; coord=6:162486424..162491946:-1; parent_transcript=GRMZM2G041694_T01; parent_gene=GRMZM2G041694	GRMZM2G041694_P01,GRMZM2G041694_P02, GRMZM2G041694_P03,GRMZM2G109743_P01, GRMZM2G109743_P02	TRUE	TRUE	aSILLIQDAAPHLR	95%	n+304 (+304)	33.49	26.38
6513	seq=translation; coord=8:1141430..1145642:1; parent_transcript=GRMZM2G063244_T01; parent_gene=GRMZM2G063244	GRMZM2G063244_P01,GRMZM2G167521_P01, GRMZM2G393515_P01,GRMZM2G398275_P01	TRUE	TRUE	iVIGLFGEVVPk	95%	n+304 (+304), K+304 (+304)	38.05	25.00
6514	seq=translation; coord=1:287818241..287821189:-1; parent_transcript=GRMZM2G068952_T01; parent_gene=GRMZM2G068952	GRMZM2G068952_P01	TRUE	TRUE	fVIWTEcAFk	95%	n+304 (+304), Carbamidomethyl (+57), K+304 (+304)	38.02	26.00
6515	seq=translation; coord=3:225893665..225898677:-1; parent_transcript=GRMZM2G090647_T01; parent_gene=GRMZM2G090647	GRMZM2G090647_P01,GRMZM2G090647_P02, GRMZM2G090647_P03,GRMZM2G090647_P05, GRMZM2G090647_P06	TRUE	TRUE	sISVVGGADGTSPSR	95%	n+304 (+304)	55.57	25.00
6516	seq=translation; coord=8:117297233..117299896:1; parent_transcript=GRMZM2G095905_T01; parent_gene=GRMZM2G095905	GRMZM2G095905_P01	TRUE	TRUE	gGFQVADLVWGk	95%	n+304 (+304), K+304 (+304)	56.04	26.21
6517	seq=translation; coord=4:143082898..143087846:-1; parent_transcript=GRMZM2G135132_T01; parent_gene=GRMZM2G135132	GRMZM2G135132_P01	TRUE	TRUE	gWETENVEEIALk	95%	n+304 (+304), K+304 (+304)	39.86	26.09
6518	seq=translation; coord=10:149089396..149092794:1; parent_transcript=GRMZM2G136283_T01; parent_gene=GRMZM2G136283	GRMZM2G136283_P01	TRUE	TRUE	eFIAVFDDFAHk	95%	n+304 (+304), K+304 (+304)	61.02	25.95
6519	seq=translation; coord=1:189637097..189642534:-1; parent_transcript=GRMZM2G141784_T01; parent_gene=GRMZM2G141784	GRMZM2G141784_P01,GRMZM2G141784_P02	TRUE	TRUE	lNVADIEATLSHVcQMVLQDSSAR	95%	n+304 (+304), Carbamidomethyl (+57)	44.23	25.00
6520	seq=translation; coord=10:148424594..148425826:-1; parent_transcript=GRMZM2G142802_T01; parent_gene=GRMZM2G142802	GRMZM2G142802_P01	TRUE	TRUE	dLAGAFAFVVFDSR	95%	n+304 (+304)	33.55	25.37
6521	seq=translation; coord=10:148424594..148425826:-1; parent_transcript=GRMZM2G142802_T01; parent_gene=GRMZM2G142802	GRMZM2G142802_P01	TRUE	TRUE	mFcGLDHIYcVFLGR	95%	n+304 (+304), Carbamidomethyl (+57), Carbamidomethyl (+57)	35.65	25.00
6522	seq=translation; coord=8:112147822..112150640:-1; parent_transcript=GRMZM2G153569_T01; parent_gene=GRMZM2G153569	GRMZM2G153569_P01,GRMZM2G153569_P02, GRMZM2G153569_P03	TRUE	TRUE	wFNHIDALLR	95%	n+304 (+304)	49.96	27.01
6523	seq=translation; coord=9:44480056..44486772:-1; parent_transcript=GRMZM2G158595_T01; parent_gene=GRMZM2G158595	GRMZM2G158595_P01,GRMZM2G158595_P02	TRUE	TRUE	lVAWSDSVADLLDLDHk	95%	n+304 (+304), K+304 (+304)	39.76	25.19

6524	seq=translation; coord=6:162189576..162190621:1; parent_transcript=GRMZM2G174906_T01; parent_gene=GRMZM2G174906	GRMZM2G174906_P01,GRMZM2G174906_P02	TRUE	TRUE	lHNLADEMEALR	95%	n+304 (+304)	47.34	25.00
6525	seq=translation; coord=4:37569961..37571464:1; parent_transcript=GRMZM2G324643_T01; parent_gene=GRMZM2G324643	GRMZM2G324643_P01,GRMZM2G324643_P02	TRUE	TRUE	dLTEEQIASMR	95%	n+304 (+304)	46.16	25.00
6526	seq=translation; coord=4:37569961..37571464:1; parent_transcript=GRMZM2G324643_T01; parent_gene=GRMZM2G324643	GRMZM2G324643_P01,GRMZM2G324643_P02	TRUE	TRUE	lEVHEFDEWIR	95%	n+304 (+304)	55.81	25.00
6527	seq=translation; coord=6:129879767..129880816:-1; parent_transcript=GRMZM2G441656_T01; parent_gene=GRMZM2G441656	GRMZM2G441656_P01	TRUE	TRUE	sASGMSGDPk	95%	n+304 (+304), K+304 (+304)	30.73	25.00
6528	seq=translation; coord=5:211975266..211979302:1; parent_transcript=GRMZM5G801436_T01; parent_gene=GRMZM5G801436	GRMZM5G801436_P01	TRUE	TRUE	iSPAFVLFFDcSEk	95%	n+304 (+304), Carbamidomethyl (+57), K+304 (+304)	61.55	25.95
6529	seq=translation; coord=1:22937896..22938573:-1; parent_transcript=GRMZM5G863656_T01; parent_gene=GRMZM5G863656	GRMZM5G863656_P01	TRUE	TRUE	lERPVEGDEAMQEVAAAE	95%	n+304 (+304)	44.39	25.00
6530	seq=translation; coord=2:230286713..230300365:-1; parent_transcript=GRMZM2G131443_T01; parent_gene=GRMZM2G131443	GRMZM2G131443_P01,GRMZM2G131443_P02, GRMZM2G131443_P03	TRUE	TRUE	iSPIVDMMTLLk	95%	n+304 (+304), K+304 (+304)	29.36	25.24
6531	seq=translation; coord=6:137824502..137828918:-1; parent_transcript=GRMZM2G170365_T01; parent_gene=GRMZM2G170365	GRMZM2G170365_P01,GRMZM2G170365_P02, GRMZM2G170365_P03,GRMZM2G170365_P04	TRUE	TRUE	aGDVcFTDVYR	95%	n+304 (+304), Carbamidomethyl (+57)	42.51	25.00
6532	seq=translation; coord=1:298331067..298333316:1; parent_transcript=GRMZM2G023242_T01; parent_gene=GRMZM2G023242	GRMZM2G023242_P01,GRMZM2G023242_P03, GRMZM2G023242_P04	TRUE	TRUE	dDLVIQFLNPk	95%	n+304 (+304), K+304 (+304)	49.71	25.99
6533	seq=translation; coord=1:298331067..298333316:1; parent_transcript=GRMZM2G023242_T01; parent_gene=GRMZM2G023242	GRMZM2G023242_P01,GRMZM2G023242_P03, GRMZM2G023242_P04	TRUE	TRUE	lQDVLPGIINQLGPDNMEHLk	95%	n+304 (+304), K+304 (+304)	30.50	25.31
6534	seq=translation; coord=1:298331067..298333316:1; parent_transcript=GRMZM2G023242_T01; parent_gene=GRMZM2G023242	GRMZM2G023242_P01,GRMZM2G023242_P03, GRMZM2G023242_P04	TRUE	TRUE	vQASIAANTWVVSGSPQTk	95%	n+304 (+304), K+304 (+304)	33.92	25.60
6535	seq=translation; coord=2:25913684..25932129:1; parent_transcript=GRMZM2G376731_T01; parent_gene=GRMZM2G376731	GRMZM2G376731_P01	TRUE	TRUE	iLyDALGTLADAVGAELNQAk	95%	n+304 (+304), iTRAQ8plex (+304), K+304 (+304)	40.76	25.00
6536	seq=translation; coord=2:25913684..25932129:1; parent_transcript=GRMZM2G376731_T01; parent_gene=GRMZM2G376731	GRMZM2G376731_P01	TRUE	TRUE	lQEFLTVAAk	93%	n+304 (+304), K+304 (+304)	26.83	25.00
6537	seq=translation; coord=2:25913684..25932129:1; parent_transcript=GRMZM2G376731_T01; parent_gene=GRMZM2G376731	GRMZM2G376731_P01	TRUE	TRUE	vAGWIELFQALHk	94%	n+304 (+304), K+304 (+304)	25.05	25.00
6538	seq=translation; coord=5:14176514..14178456:-1; parent_transcript=GRMZM5G858249_T01; parent_gene=GRMZM5G858249	GRMZM5G858249_P01	TRUE	TRUE	aHAAYAVNQLYQAAGR	89%	n+304 (+304)	25.06	25.00
6539	seq=translation; coord=5:14176514..14178456:-1; parent_transcript=GRMZM5G858249_T01; parent_gene=GRMZM5G858249	GRMZM5G858249_P01	TRUE	TRUE	aSATLTSDDPSYGAcVyTGGGQ	95%	n+304 (+304), Carbamidomethyl (+57), iTRAQ8plex (+304)	34.15	25.00
6540	seq=translation; coord=5:14176514..14178456:-1; parent_transcript=GRMZM5G858249_T01; parent_gene=GRMZM5G858249	GRMZM5G858249_P01	TRUE	TRUE	hPWNcDFR	95%	n+304 (+304), Carbamidomethyl (+57)	31.51	25.00
6541	seq=translation; coord=3:135084015..135105483:1; parent_transcript=GRMZM2G326116_T01; parent_gene=GRMZM2G326116	GRMZM2G326116_P01	TRUE	TRUE	fIPSEFGMDPSk	95%	n+304 (+304), K+304 (+304)	30.59	25.33

6542	seq=translation; coord=3:135084015..135105483:1; parent_transcript=GRMZM2G326116_T01; parent_gene=GRMZM2G326116	GRMZM2G326116_P01	TRUE	TRUE	iHIPADEFLASMk	95%	n+304 (+304), K+304 (+304)	29.18	26.17
6543	seq=translation; coord=5:145524164..145526605:-1; parent_transcript=GRMZM2G405459_T01; parent_gene=GRMZM2G405459	GRMZM2G405459_P01,GRMZM2G405459_P02	TRUE	TRUE	sQVEAAcPGTVScADILALAAR	95%	n+304 (+304), Carbamidomethyl (+57), Carbamidomethyl (+57)	36.78	25.00
6544	seq=translation; coord=6:110695290..110707260:1; parent_transcript=GRMZM2G412611_T01; parent_gene=GRMZM2G412611	GRMZM2G412611_P01	TRUE	TRUE	aPEAPESDESk	95%	n+304 (+304), K+304 (+304)	36.17	25.00
6545	seq=translation; coord=6:110695290..110707260:1; parent_transcript=GRMZM2G412611_T01; parent_gene=GRMZM2G412611	GRMZM2G412611_P01	TRUE	TRUE	iEIDDPGVHAIEFLIFDETQNk	95%	n+304 (+304), K+304 (+304)	27.20	25.59
6546	seq=translation; coord=3:1767353..1776209:-1; parent_transcript=GRMZM2G123499_T01; parent_gene=GRMZM2G123499	GRMZM2G123499_P01,GRMZM2G123499_P02	TRUE	TRUE	dALQIFFAEASk	94%	n+304 (+304), K+304 (+304)	28.59	25.43
6547	seq=translation; coord=3:1767353..1776209:-1; parent_transcript=GRMZM2G123499_T01; parent_gene=GRMZM2G123499	GRMZM2G123499_P01,GRMZM2G123499_P02	TRUE	TRUE	sFLSELSEVVGIGSASIk	95%	n+304 (+304), K+304 (+304)	35.23	25.17
6548	seq=translation; coord=6:47529651..47533455:-1; parent_transcript=GRMZM2G017671_T01; parent_gene=GRMZM2G017671	GRMZM2G017671_P01,GRMZM2G017671_P03	TRUE	TRUE	dLTLAAELVSELWNAGIk	95%	n+304 (+304), K+304 (+304)	47.59	25.00
6549	seq=translation; coord=1:122162499..122163689:1; parent_transcript=AC202185.4_FGT004; parent_gene=AC202185.4_FG004	AC202185.4_FGP004	TRUE	TRUE	sPAGGGVAEYGSQSQQQSR	95%	n+304 (+304)	36.80	25.00
6550	seq=translation; coord=1:122162499..122163689:1; parent_transcript=AC202185.4_FGT004; parent_gene=AC202185.4_FG004	AC202185.4_FGP004	TRUE	TRUE	tDLHPFNIPAFVLVHPVAPR	95%	n+304 (+304)	32.01	25.59
6551	seq=translation; coord=2:71785920..71790206:-1; parent_transcript=GRMZM2G000980_T01; parent_gene=GRMZM2G000980	GRMZM2G000980_P01,GRMZM2G034313_P01	TRUE	TRUE	dFAAEMDPEVHALNAR	95%	n+304 (+304)	48.65	25.00
6552	seq=translation; coord=2:71785920..71790206:-1; parent_transcript=GRMZM2G000980_T01; parent_gene=GRMZM2G000980	GRMZM2G000980_P01,GRMZM2G034313_P01	TRUE	TRUE	eYPGYNFIGLIIGPR	95%	n+304 (+304)	43.06	25.84
6553	seq=translation; coord=5:17058108..17064678:1; parent_transcript=GRMZM2G018943_T01; parent_gene=GRMZM2G018943	GRMZM2G018943_P01,GRMZM2G018943_P02	TRUE	TRUE	dVPQAASTLASEk	95%	n+304 (+304), K+304 (+304)	39.22	25.85
6554	seq=translation; coord=5:17058108..17064678:1; parent_transcript=GRMZM2G018943_T01; parent_gene=GRMZM2G018943	GRMZM2G018943_P01,GRMZM2G018943_P02	TRUE	TRUE	lDIVSEIGDDDSWSR	95%	n+304 (+304)	52.64	25.00
6555	seq=translation; coord=6:112137413..112140985:1; parent_transcript=GRMZM2G064993_T01; parent_gene=GRMZM2G064993	GRMZM2G064993_P01	TRUE	TRUE	aYAEAYGEELLR	95%	n+304 (+304)	51.11	25.00
6556	seq=translation; coord=6:112137413..112140985:1; parent_transcript=GRMZM2G064993_T01; parent_gene=GRMZM2G064993	GRMZM2G064993_P01	TRUE	TRUE	sITDEISGDFER	95%	n+304 (+304)	43.51	25.00
6557	seq=translation; coord=5:59290440..59291930:-1; parent_transcript=GRMZM2G084942_T01; parent_gene=GRMZM2G084942	GRMZM2G084942_P01,GRMZM2G084942_P02	TRUE	TRUE	aDAFLNIFER	95%	n+304 (+304)	35.95	25.00
6558	seq=translation; coord=5:59290440..59291930:-1; parent_transcript=GRMZM2G084942_T01; parent_gene=GRMZM2G084942	GRMZM2G084942_P01,GRMZM2G084942_P02	TRUE	TRUE	lFDGLHDVLR	95%	n+304 (+304)	51.05	25.58
6559	seq=translation; coord=5:209693887..209699045:1; parent_transcript=GRMZM2G101463_T01; parent_gene=GRMZM2G101463	GRMZM2G101463_P01	TRUE	TRUE	aILGDAFDVGMk	95%	n+304 (+304), K+304 (+304)	50.51	26.47

6560	seq=translation; coord=5:209693887..209699045:1; parent_transcript=GRMZM2G101463_T01; parent_gene=GRMZM2G101463	GRMZM2G101463_P01	TRUE	TRUE	gSPEAQLVAAISPEGASk	95%	n+304 (+304), K+304 (+304)	41.26	25.00
6561	seq=translation; coord=3:184059147..184065670:1; parent_transcript=GRMZM2G118363_T01; parent_gene=GRMZM2G118363	GRMZM2G118363_P01	TRUE	TRUE	iLLLLDSWQEAFGGPGSk	95%	n+304 (+304), K+304 (+304)	34.64	25.00
6562	seq=translation; coord=3:184059147..184065670:1; parent_transcript=GRMZM2G118363_T01; parent_gene=GRMZM2G118363	GRMZM2G118363_P01	TRUE	TRUE	nVTDLLNDMVYALNPSDR	95%	n+304 (+304)	41.22	25.00
6563	seq=translation; coord=9:140970479..140979075:-1; parent_transcript=GRMZM2G119627_T01; parent_gene=GRMZM2G119627	GRMZM2G119627_P01	TRUE	TRUE	aGDLTIADVESLLNSYk	95%	n+304 (+304), K+304 (+304)	42.60	25.07
6564	seq=translation; coord=9:140970479..140979075:-1; parent_transcript=GRMZM2G119627_T01; parent_gene=GRMZM2G119627	GRMZM2G119627_P01	TRUE	TRUE	aPDPEkDSTAIQEFLENMEGAFR	95%	n+304 (+304), K+304 (+304)	31.34	25.00
6565	seq=translation; coord=6:111182254..111183500:-1; parent_transcript=GRMZM2G122302_T01; parent_gene=GRMZM2G122302	GRMZM2G122302_P01	TRUE	TRUE	vGDSLEFk	95%	n+304 (+304), K+304 (+304)	35.56	26.28
6566	seq=translation; coord=6:111182254..111183500:-1; parent_transcript=GRMZM2G122302_T01; parent_gene=GRMZM2G122302	GRMZM2G122302_P01	TRUE	TRUE	yTVGDASGWTTTGDYATWASGk	95%	n+304 (+304), K+304 (+304)	34.83	25.00
6567	seq=translation; coord=8:85844666..85847518:-1; parent_transcript=GRMZM2G132130_T01; parent_gene=GRMZM2G132130	GRMZM2G132130_P01	TRUE	TRUE	fSVVGFGSLLSER	95%	n+304 (+304)	54.84	26.35
6568	seq=translation; coord=8:85844666..85847518:-1; parent_transcript=GRMZM2G132130_T01; parent_gene=GRMZM2G132130	GRMZM2G132130_P01	TRUE	TRUE	vFAHAAPIFFER	95%	n+304 (+304)	61.70	26.10
6569	seq=translation; coord=1:256071969..256085112:1; parent_transcript=GRMZM2G150772_T02; parent_gene=GRMZM2G150772	GRMZM2G150772_P02	TRUE	TRUE	fSALSAPGcVIcGMGSIk	95%	n+304 (+304), Carbamidomethyl (+57), Carbamidomethyl (+57), K+304 (+304)	39.03	25.21
6570	seq=translation; coord=1:256071969..256085112:1; parent_transcript=GRMZM2G150772_T02; parent_gene=GRMZM2G150772	GRMZM2G150772_P02	TRUE	TRUE	iAVALLQEIAR	95%	n+304 (+304)	33.57	25.00
6571	seq=translation; coord=6:111253708..111260162:-1; parent_transcript=GRMZM2G329306_T01; parent_gene=GRMZM2G329306	GRMZM2G329306_P01	TRUE	TRUE	fEDENFVLk	95%	n+304 (+304), K+304 (+304)	36.00	26.60
6572	seq=translation; coord=6:111253708..111260162:-1; parent_transcript=GRMZM2G329306_T01; parent_gene=GRMZM2G329306	GRMZM2G329306_P01	TRUE	TRUE	lGDSSGALLDADFALR	95%	n+304 (+304)	47.57	25.50
6573	seq=translation; coord=10:136292132..136296887:1; parent_transcript=GRMZM2G414915_T01; parent_gene=GRMZM2G414915	GRMZM2G414915_P01	TRUE	TRUE	lGIRPSPVVAAFSSR	95%	n+304 (+304)	31.96	25.40
6574	seq=translation; coord=10:136292132..136296887:1; parent_transcript=GRMZM2G414915_T01; parent_gene=GRMZM2G414915	GRMZM2G414915_P01	TRUE	TRUE	sLcLEGTLQPHEVAGk	95%	n+304 (+304), Carbamidomethyl (+57), K+304 (+304)	42.04	25.35
6575	seq=translation; coord=6:140865431..140872714:-1; parent_transcript=GRMZM2G442057_T01; parent_gene=GRMZM2G442057	GRMZM2G442057_P01	TRUE	TRUE	kYDTALEVLSGDLGSLLGR	95%	K+304 (+304), n+304 (+304)	51.72	25.37
6576	seq=translation; coord=6:140865431..140872714:-1; parent_transcript=GRMZM2G442057_T01; parent_gene=GRMZM2G442057	GRMZM2G442057_P01	TRUE	TRUE	vRPIWDAVDSR	91%	n+304 (+304)	26.96	26.52

6577	seq=translation; coord=10:10725550..10730964:1; parent_transcript=GRMZM2G014397_T02; parent_gene=GRMZM2G014397	GRMZM2G014397_P02,GRMZM2G014397_P03	TRUE	TRUE	dQDTFFEDYAEAHAk	95%	n+304 (+304), K+304 (+304)	55.85	25.00
6578	seq=translation; coord=10:10725550..10730964:1; parent_transcript=GRMZM2G014397_T02; parent_gene=GRMZM2G014397	GRMZM2G014397_P02,GRMZM2G014397_P03	TRUE	TRUE	vDVIAPEQcPPEGR	95%	n+304 (+304), Carbamidomethyl (+57)	31.55	25.00
6579	seq=translation; coord=1:257994567..257995838:-1; parent_transcript=GRMZM2G044762_T01; parent_gene=GRMZM2G044762	GRMZM2G044762_P01,GRMZM2G044762_P02	TRUE	TRUE	aLVPTDLSVAIPHGTyAR	94%	n+304 (+304), iTRAQ8plex (+304)	25.76	25.00
6580	seq=translation; coord=1:257994567..257995838:-1; parent_transcript=GRMZM2G044762_T01; parent_gene=GRMZM2G044762	GRMZM2G044762_P01,GRMZM2G044762_P02	TRUE	TRUE	hSIDVGAGVIDADYR	95%	n+304 (+304)	41.49	25.00
6581	seq=translation; coord=1:152164284..152165382:1; parent_transcript=GRMZM2G132077_T01; parent_gene=GRMZM2G132077	GRMZM2G132077_P01	TRUE	TRUE	iIYcNPHV	94%	n+304 (+304), Carbamidomethyl (+57)	30.66	25.00
6582	seq=translation; coord=1:152164284..152165382:1; parent_transcript=GRMZM2G132077_T01; parent_gene=GRMZM2G132077	GRMZM2G132077_P01	TRUE	TRUE	sHAEFPVcWWTGR	95%	n+304 (+304), Carbamidomethyl (+57)	43.38	25.00
6583	seq=translation; coord=3:124642615..124647335:-1; parent_transcript=GRMZM2G098397_T01; parent_gene=GRMZM2G098397	GRMZM2G098397_P01,GRMZM2G098397_P02, GRMZM2G098397_P03,GRMZM2G098397_P04	TRUE	TRUE	aSVLNLDLTNPLLAk	95%	n+304 (+304), K+304 (+304)	51.76	25.00
6584	seq=translation; coord=3:124642615..124647335:-1; parent_transcript=GRMZM2G098397_T01; parent_gene=GRMZM2G098397	GRMZM2G098397_P01,GRMZM2G098397_P02, GRMZM2G098397_P03,GRMZM2G098397_P04	TRUE	TRUE	eFIEYTVLk	92%	n+304 (+304), K+304 (+304)	26.86	25.00
6585	seq=translation; coord=2:45957407..45958283:1; parent_transcript=GRMZM2G028640_T01; parent_gene=GRMZM2G028640	GRMZM2G028640_P01	TRUE	TRUE	vYPDGHGEEFVEIAAGDLVVFPk	93%	n+304 (+304), K+304 (+304)	25.13	25.00
6586	seq=translation; coord=5:12291230..12291790:-1; parent_transcript=AC206941.2_FGT002; parent_gene=AC206941.2_FG002	AC206941.2_FGP002	TRUE	TRUE	tAQAGQAIQGR	95%	n+304 (+304)	47.82	25.98
6587	seq=translation; coord=9:80565232..80569476:-1; parent_transcript=GRMZM2G027385_T01; parent_gene=GRMZM2G027385	GRMZM2G027385_P01,GRMZM2G027385_P03, GRMZM2G081583_P02,GRMZM2G081583_P04	TRUE	TRUE	sTPEMWPGLLQk	89%	n+304 (+304), K+304 (+304)	26.36	26.30
6588	seq=translation; coord=8:23799793..23801891:-1; parent_transcript=GRMZM5G861077_T01; parent_gene=GRMZM5G861077	GRMZM5G861077_P01	TRUE	TRUE	iFGVWLSGAELLR	91%	n+304 (+304)	28.40	25.00
6589	seq=translation; coord=8:23799793..23801891:-1; parent_transcript=GRMZM5G861077_T01; parent_gene=GRMZM5G861077	GRMZM5G861077_P01	TRUE	TRUE	yVGTDGcYFR	93%	n+304 (+304), Carbamidomethyl (+57)	29.34	25.00
6590	seq=translation; coord=2:231282768..231284403:-1; parent_transcript=AC148152.3_FGT005; parent_gene=AC148152.3_FG005	AC148152.3_FGP005	TRUE	TRUE	sVTFPDFIAk	93%	n+304 (+304), K+304 (+304)	28.97	26.23
6591	seq=translation; coord=7:174339610..174341979:-1; parent_transcript=GRMZM2G424205_T01; parent_gene=GRMZM2G424205	GRMZM2G424205_P01	TRUE	TRUE	gGGFGGGAGGGFGGGk	95%	n+304 (+304), K+304 (+304)	38.30	25.00
6592	seq=translation; coord=7:174339610..174341979:-1; parent_transcript=GRMZM2G424205_T01; parent_gene=GRMZM2G424205	GRMZM2G424205_P01	TRUE	TRUE	gGGFGGGk	87%	n+304 (+304), K+304 (+304)	25.68	25.02
6593	seq=translation; coord=8:121435529..121437647:-1; parent_transcript=GRMZM2G042865_T01; parent_gene=GRMZM2G042865	GRMZM2G042865_P01,GRMZM2G082037_P01	TRUE	TRUE	fLEPGGPGEAAR	87%	n+304 (+304)	25.55	25.00
6594	seq=translation; coord=8:121435529..121437647:-1; parent_transcript=GRMZM2G042865_T01; parent_gene=GRMZM2G042865	GRMZM2G042865_P01,GRMZM2G082037_P01	TRUE	TRUE	lVTDVLGIGER	95%	n+304 (+304)	36.52	25.26

6595	seq=translation; coord=8:18448251..18451290:-1; parent_transcript=GRMZM2G336815_T01; parent_gene=GRMZM2G336815	GRMZM2G336815_P01	TRUE	TRUE	iFGMEHWHALR	95%	n+304 (+304)	26.09	25.00
6596	seq=translation; coord=1:11301152..11301835:-1; parent_transcript=AC209858.4_FGT004; parent_gene=AC209858.4_FG004	AC209858.4_FGP004	TRUE	TRUE	rLDLSASPLADPEAATFASPR	95%	n+304 (+304)	47.44	25.34
6597	seq=translation; coord=8:163002198..163002947:1; parent_transcript=GRMZM2G022958_T01; parent_gene=GRMZM2G022958	GRMZM2G022958_P01	TRUE	TRUE	vEEDINTcTGEVHER	95%	n+304 (+304), Carbamidomethyl (+57)	93.10	25.00
6598	seq=translation; coord=8:37807066..37810916:-1; parent_transcript=GRMZM2G061088_T01; parent_gene=GRMZM2G061088	GRMZM2G061088_P01	TRUE	TRUE	gFDVIDAAk	95%	n+304 (+304), K+304 (+304)	33.24	25.58
6599	seq=translation; coord=1:38124980..38130704:-1; parent_transcript=GRMZM2G161337_T01; parent_gene=GRMZM2G161337	GRMZM2G161337_P01,GRMZM2G161337_P02	TRUE	TRUE	eHIMAGDIFQIVLSQR	95%	n+304 (+304)	46.31	25.73
6600	seq=translation; coord=10:74848346..74854487:1; parent_transcript=GRMZM2G106974_T01; parent_gene=GRMZM2G106974	GRMZM2G106974_P01	TRUE	TRUE	lLHPFMPYVTEELWQR	95%	n+304 (+304)	47.14	25.00
6601	seq=translation; coord=1:193865451..193869209:1; parent_transcript=GRMZM2G114739_T01; parent_gene=GRMZM2G114739	GRMZM2G114739_P01	TRUE	TRUE	lSDVLELVGIPk	90%	n+304 (+304), K+304 (+304)	25.48	25.00
6602	seq=translation; coord=8:117552021..117572795:1; parent_transcript=GRMZM2G051050_T01; parent_gene=GRMZM2G051050	GRMZM2G051050_P01,GRMZM2G094951_P01, GRMZM2G096470_P01,GRMZM2G096470_P02	TRUE	TRUE	aDELETALMEMVk	94%	n+304 (+304), K+304 (+304)	29.30	25.56
6603	seq=translation; coord=1:46134883..46138910:-1; parent_transcript=GRMZM2G029543_T01; parent_gene=GRMZM2G029543	GRMZM2G029543_P01	TRUE	TRUE	vIAGILk	94%	n+304 (+304), K+304 (+304)	27.63	25.00
6604	seq=translation; coord=8:119902616..119903381:-1; parent_transcript=GRMZM2G005557_T01; parent_gene=GRMZM2G005557	GRMZM2G005557_P01	TRUE	TRUE	lTEEEADAMLR	94%	n+304 (+304)	30.58	25.00
6605	seq=translation; coord=7:2997300..3007755:-1; parent_transcript=GRMZM2G044060_T01; parent_gene=GRMZM2G044060	GRMZM2G044060_P01,GRMZM2G044060_P02	TRUE	TRUE	iGEVVDGPGEAAQGQVHDGVR	95%	n+304 (+304)	87.98	25.00
6606	seq=translation; coord=7:2997300..3007755:-1; parent_transcript=GRMZM2G044060_T01; parent_gene=GRMZM2G044060	GRMZM2G044060_P01,GRMZM2G044060_P02	TRUE	TRUE	lPAIVNVLk	93%	n+304 (+304), K+304 (+304)	26.21	25.00
6607	seq=translation; coord=3:90023131..90026648:1; parent_transcript=GRMZM2G135599_T01; parent_gene=GRMZM2G135599	GRMZM2G135599_P01,GRMZM2G135599_P02, GRMZM2G450424_P01,GRMZM2G450424_P03, GRMZM2G450424_P05	TRUE	TRUE	vILFPFFHR	92%	n+304 (+304)	26.52	25.00
6608	seq=translation; coord=5:210751187..210752748:-1; parent_transcript=GRMZM2G083886_T01; parent_gene=GRMZM2G083886	GRMZM2G083886_P01	TRUE	TRUE	lEHGALPQTDVLADALAGk	95%	n+304 (+304), K+304 (+304)	28.82	25.00
6609	seq=translation; coord=5:210751187..210752748:-1; parent_transcript=GRMZM2G083886_T01; parent_gene=GRMZM2G083886	GRMZM2G083886_P01	TRUE	TRUE	sVAPLPQk	92%	n+304 (+304), K+304 (+304)	28.58	25.00
6610	seq=translation; coord=4:79156649..79162538:-1; parent_transcript=GRMZM2G702490_T01; parent_gene=GRMZM2G702490	GRMZM2G702490_P01	TRUE	TRUE	hIPLIFLQNITGFMVGSk	95%	n+304 (+304), K+304 (+304)	44.74	25.00
6611	seq=translation; coord=7:2584958..2590700:-1; parent_transcript=GRMZM2G043383_T01; parent_gene=GRMZM2G043383	GRMZM2G043383_P01,GRMZM2G043383_P02, GRMZM2G070542_P01,GRMZM2G070542_P02	TRUE	TRUE	qQQILLATQVVk	93%	n+304 (+304), K+304 (+304)	26.69	25.00
6612	seq=translation; coord=7:2584958..2590700:-1; parent_transcript=GRMZM2G043383_T01; parent_gene=GRMZM2G043383	GRMZM2G043383_P01,GRMZM2G043383_P02, GRMZM2G070542_P01,GRMZM2G070542_P02	TRUE	TRUE	sQDYEIGDGTTGVVVMAGALLEQAEk	90%	n+304 (+304), K+304 (+304)	25.93	25.31

6613	seq=translation; coord=8:104770563..104774039:-1; parent_transcript=GRMZM2G334899_T01; parent_gene=GRMZM2G334899	GRMZM2G334899_P01	TRUE	TRUE	yMGTSDVQLER	95%	n+304 (+304)	34.09	25.00
6614	seq=translation; coord=8:10755838..10761776:-1; parent_transcript=GRMZM2G119345_T01; parent_gene=GRMZM2G119345	GRMZM2G119345_P01,GRMZM2G119345_P02	TRUE	TRUE	mGLLQVAAINTAMAALTk	94%	n+304 (+304), K+304 (+304)	28.24	25.00
6615	seq=translation; coord=2:60272199..60277574:-1; parent_transcript=GRMZM2G157269_T01; parent_gene=GRMZM2G157269	GRMZM2G157269_P01	TRUE	TRUE	gPIMIEWFk	95%	n+304 (+304), K+304 (+304)	36.61	26.22
6616	seq=translation; coord=2:183342461..183343536:1; parent_transcript=GRMZM2G060429_T01; parent_gene=GRMZM2G060429	GRMZM2G060429_P01	TRUE	TRUE	hQcSPAATPYGSPQcQALQQQccHQIR	95%	n+304 (+304), Carbamidomethyl (+57), Carbamidomethyl (+57), Carbamidomethyl (+57), Carbamidomethyl (+57)	31.55	25.00
6617	seq=translation; coord=5:206910875..206915210:-1; parent_transcript=GRMZM2G105539_T01; parent_gene=GRMZM2G105539	GRMZM2G105539_P01,GRMZM2G105539_P02	TRUE	TRUE	gVPALNFLGDILWk	95%	n+304 (+304), K+304 (+304)	32.50	25.00
6618	seq=translation; coord=3:196592864..196598555:-1; parent_transcript=GRMZM2G055857_T01; parent_gene=GRMZM2G055857	GRMZM2G055857_P01,GRMZM2G055857_P02, GRMZM2G055857_P03,GRMZM2G055857_P04	TRUE	TRUE	sIFEPLLPLIIGR	95%	n+304 (+304)	33.25	25.00
6619	seq=translation; coord=4:11823842..11828550:1; parent_transcript=GRMZM2G017804_T01; parent_gene=GRMZM2G017804	GRMZM2G017804_P01,GRMZM2G017804_P02	TRUE	TRUE	mLDLNPMIIAQLk	92%	n+304 (+304), K+304 (+304)	27.00	25.59
6620	seq=translation; coord=2:161123950..161130108:1; parent_transcript=GRMZM2G018241_T01; parent_gene=GRMZM2G018241	GRMZM2G018241_P01,GRMZM2G111642_P01, GRMZM2G111642_P02,GRMZM2G111642_P03, GRMZM2G111642_P04,GRMZM2G424832_P01, GRMZM2G424832_P02	TRUE	TRUE	dGTQAcPQck	91%	n+304 (+304), Carbamidomethyl (+57), Carbamidomethyl (+57), K+304 (+304)	25.86	25.00
6621	seq=translation; coord=1:111589370..111591480:1; parent_transcript=GRMZM2G033930_T01; parent_gene=GRMZM2G033930	GRMZM2G033930_P01	TRUE	TRUE	eMFGDDVLGALNIk	95%	n+304 (+304), K+304 (+304)	35.45	25.02
6622	seq=translation; coord=2:195920737..195924614:-1; parent_transcript=GRMZM2G002807_T01; parent_gene=GRMZM2G002807	GRMZM2G002807_P01,GRMZM5G852968_P02, GRMZM5G852968_P03	TRUE	TRUE	vIAcIGELLEER	86%	n+304 (+304), Carbamidomethyl (+57)	25.88	25.42
6623	seq=translation; coord=2:49056549..49061498:-1; parent_transcript=GRMZM2G003565_T03; parent_gene=GRMZM2G003565	GRMZM2G003565_P03	TRUE	TRUE	yAIVPDENVR	95%	n+304 (+304)	39.07	25.56
6624	seq=translation; coord=4:55258880..55260891:-1; parent_transcript=GRMZM2G005046_T01; parent_gene=GRMZM2G005046	GRMZM2G005046_P01,GRMZM2G129266_P01, GRMZM2G129266_P02	TRUE	TRUE	fDLLTGLLWk	95%	n+304 (+304), K+304 (+304)	29.37	25.00
6625	seq=translation; coord=9:151763726..151772001:-1; parent_transcript=GRMZM2G009808_T01; parent_gene=GRMZM2G009808	GRMZM2G009808_P01	TRUE	TRUE	sNLAGMGIIPLcFk	95%	n+304 (+304), Carbamidomethyl (+57), K+304 (+304)	63.74	25.79
6626	seq=translation; coord=8:172976483..172980420:-1; parent_transcript=GRMZM2G027535_T01; parent_gene=GRMZM2G027535	GRMZM2G027535_P01,GRMZM2G027535_P02	TRUE	TRUE	tWLDFPLFVDDR	95%	n+304 (+304)	32.50	25.00
6627	seq=translation; coord=1:88848897..88849748:-1; parent_transcript=GRMZM2G037255_T01; parent_gene=GRMZM2G037255	GRMZM2G037255_P01	TRUE	TRUE	lGVcANVLGLVk	95%	n+304 (+304), Carbamidomethyl (+57), K+304 (+304)	46.35	25.00
6628	seq=translation; coord=8:144588215..144595174:1; parent_transcript=GRMZM2G037694_T01; parent_gene=GRMZM2G037694	GRMZM2G037694_P01,GRMZM2G037694_P02	TRUE	TRUE	iVLEALPAVLLFEk	95%	n+304 (+304), K+304 (+304)	37.29	25.00
6629	seq=translation; coord=2:177646906..177651736:-1; parent_transcript=GRMZM2G039155_T01; parent_gene=GRMZM2G039155	GRMZM2G039155_P01,GRMZM2G039155_P02, GRMZM2G039155_P03,GRMZM2G039155_P05, GRMZM2G065355_P01	TRUE	TRUE	iDELEQSINDLk	95%	n+304 (+304), K+304 (+304)	33.39	26.04

6630	seq=translation; coord=6:144596360..144598543:-1; parent_transcript=GRMZM2G042797_T01; parent_gene=GRMZM2G042797	GRMZM2G042797_P01	TRUE	TRUE	vAAVWLDGAELLR	95%	n+304 (+304)	39.92	25.34
6631	seq=translation; coord=1:41956891..41966584:-1; parent_transcript=GRMZM2G044237_T01; parent_gene=GRMZM2G044237	GRMZM2G044237_P01,GRMZM2G044237_P02, GRMZM2G044237_P03,GRMZM2G044237_P04	TRUE	TRUE	lDISLGVIPLMk	95%	n+304 (+304), K+304 (+304)	42.40	25.00
6632	seq=translation; coord=2:35876928..35877532:1; parent_transcript=GRMZM2G054046_T01; parent_gene=GRMZM2G054046	GRMZM2G054046_P01,GRMZM2G096546_P01	TRUE	TRUE	gPTDVLVHDFQFEVEQVLSR	95%	n+304 (+304)	35.48	25.25
6633	seq=translation; coord=1:5530739..5532411:1; parent_transcript=GRMZM2G055704_T01; parent_gene=GRMZM2G055704	GRMZM2G055704_P01,GRMZM2G055704_P02	TRUE	TRUE	mTVIGTVDPVQVVSk	95%	n+304 (+304), K+304 (+304)	53.12	25.00
6634	seq=translation; coord=4:208244723..208251169:1; parent_transcript=GRMZM2G060800_T01; parent_gene=GRMZM2G060800	GRMZM2G060800_P01,GRMZM2G060800_P02	TRUE	TRUE	sFAPELVASFk	95%	n+304 (+304), K+304 (+304)	28.82	25.01
6635	seq=translation; coord=6:35595648..35597164:1; parent_transcript=GRMZM2G062197_T01; parent_gene=GRMZM2G062197	GRMZM2G062197_P01,GRMZM2G062197_P02, GRMZM2G137930_P01,GRMZM2G137930_P02, GRMZM2G137930_P03,GRMZM2G137930_P04	TRUE	TRUE	aIGLMPMIDQGEADDk	95%	n+304 (+304), K+304 (+304)	51.86	25.00
6636	seq=translation; coord=3:151971037..151972416:1; parent_transcript=GRMZM2G065585_T01; parent_gene=GRMZM2G065585	GRMZM2G065585_P01	TRUE	TRUE	rPGPIETYIFAMFNEDQk	94%	n+304 (+304), K+304 (+304)	26.30	25.00
6637	seq=translation; coord=7:105219462..105221560:-1; parent_transcript=GRMZM2G074781_T01; parent_gene=GRMZM2G074781	GRMZM2G074781_P01	TRUE	TRUE	eLTDWLLWLk	94%	n+304 (+304), K+304 (+304)	29.83	26.13
6638	seq=translation; coord=2:232964582..232968333:-1; parent_transcript=GRMZM2G082931_T01; parent_gene=GRMZM2G082931	GRMZM2G082931_P01	TRUE	TRUE	gFGFVTFSEPR	95%	n+304 (+304)	36.04	25.00
6639	seq=translation; coord=2:150834303..150835528:1; parent_transcript=GRMZM2G094589_T01; parent_gene=GRMZM2G094589	GRMZM2G094589_P01	TRUE	TRUE	iPGEVAIFGVFR	95%	n+304 (+304)	30.52	25.02
6640	seq=translation; coord=2:135956633..135961656:1; parent_transcript=GRMZM2G094699_T01; parent_gene=GRMZM2G094699	GRMZM2G094699_P01	TRUE	TRUE	lTEPELLEPLVPSILANLEHR	95%	n+304 (+304)	44.58	25.00
6641	seq=translation; coord=1:32308316..32321893:-1; parent_transcript=GRMZM2G099528_T01; parent_gene=GRMZM2G099528	GRMZM2G099528_P01,GRMZM2G099528_P02	TRUE	TRUE	lMcTSEIcTAEER	95%	n+304 (+304), Carbamidomethyl (+57), Carbamidomethyl (+57)	48.41	25.00
6642	seq=translation; coord=6:45613410..45627830:1; parent_transcript=GRMZM2G104377_T03; parent_gene=GRMZM2G104377	GRMZM2G104377_P03	TRUE	TRUE	fLDMWEPAILAIk	95%	n+304 (+304), K+304 (+304)	31.34	25.00
6643	seq=translation; coord=1:199112157..199114320:1; parent_transcript=GRMZM2G113191_T01; parent_gene=GRMZM2G113191	GRMZM2G113191_P01	TRUE	TRUE	mQSPIDLAR	95%	n+304 (+304)	39.53	26.10
6644	seq=translation; coord=4:235758732..235759487:-1; parent_transcript=GRMZM2G115340_T01; parent_gene=GRMZM2G115340	GRMZM2G115340_P01,GRMZM2G179248_P01	TRUE	TRUE	lGAcMcTk	95%	n+304 (+304), Carbamidomethyl (+57), Carbamidomethyl (+57), K+304 (+304)	32.98	25.00
6645	seq=translation; coord=5:59978801..59980128:1; parent_transcript=GRMZM2G116167_T01; parent_gene=GRMZM2G116167	GRMZM2G116167_P01,GRMZM2G116167_P02	TRUE	TRUE	aFGVAPEQGLVLAR	95%	n+304 (+304)	38.19	25.25
6646	seq=translation; coord=4:191950335..191955467:1; parent_transcript=GRMZM2G117614_T01; parent_gene=GRMZM2G117614	GRMZM2G117614_P01,GRMZM2G117614_P02, GRMZM2G117614_P03,GRMZM2G117614_P04	TRUE	TRUE	vcLGNLVGMPVVVVPTGLk	95%	n+304 (+304), Carbamidomethyl (+57), K+304 (+304)	30.50	25.00

6647	seq=translation; coord=4:74457132..74460979:1; parent_transcript=GRMZM2G117755_T01; parent_gene=GRMZM2G117755	GRMZM2G117755_P01,GRMZM2G117755_P02, GRMZM2G150762_P01,GRMZM2G150762_P02, GRMZM2G150762_P03,GRMZM2G150762_P04, GRMZM2G150762_P05	TRUE	TRUE	eQIQSYVFDVIR	95%	n+304 (+304)	43.07	25.24
6648	seq=translation; coord=5:34609536..34613247:-1; parent_transcript=GRMZM2G120579_T01; parent_gene=GRMZM2G120579	GRMZM2G120579_P01	TRUE	TRUE	lGDLLDTMSALk	95%	n+304 (+304), K+304 (+304)	31.33	25.94
6649	seq=translation; coord=1:222130457..222134768:1; parent_transcript=GRMZM2G149946_T01; parent_gene=GRMZM2G149946	GRMZM2G149946_P01,GRMZM2G149946_P02, GRMZM2G149946_P03	TRUE	TRUE	lADSMAEFIESR	95%	n+304 (+304)	36.11	25.00
6650	seq=translation; coord=6:104494543..104508487:-1; parent_transcript=GRMZM2G159744_T01; parent_gene=GRMZM2G159744	GRMZM2G159744_P01,GRMZM2G159744_P02	TRUE	TRUE	mGcVILSFLEk	95%	n+304 (+304), Carbamidomethyl (+57), K+304 (+304)	30.51	25.77
6651	GRMZM2G172322_P01-R	GRMZM2G172322_P01-R	TRUE	TRUE	aLAGQk	95%	n+304 (+304), K+304 (+304)	31.48	26.72
6652	seq=translation; coord=5:130658345..130666795:1; parent_transcript=GRMZM2G179981_T01; parent_gene=GRMZM2G179981	GRMZM2G179981_P01	TRUE	TRUE	sIGIELIPLETSVk	95%	n+304 (+304), K+304 (+304)	34.85	25.00
6653	seq=translation; coord=8:78358305..78366485:-1; parent_transcript=GRMZM2G451483_T01; parent_gene=GRMZM2G451483	GRMZM2G451483_P01	TRUE	TRUE	mTLNQLLVELDGFk	95%	n+304 (+304), K+304 (+304)	32.46	25.81
6654	seq=translation; coord=2:167362256..167363675:1; parent_transcript=GRMZM5G872070_T01; parent_gene=GRMZM5G872070	GRMZM5G872070_P01	TRUE	TRUE	gIFYGDSLScETVR	95%	n+304 (+304), Carbamidomethyl (+57)	59.11	25.00
6655	seq=translation; coord=3:224460656..224464191:-1; parent_transcript=GRMZM5G874955_T01; parent_gene=GRMZM5G874955	GRMZM5G874955_P01	TRUE	TRUE	gMILPFVPLSLTFDNIk	95%	n+304 (+304), K+304 (+304)	45.34	25.00
6656	seq=translation; coord=1:40222143..40226699:1; parent_transcript=GRMZM2G174644_T01; parent_gene=GRMZM2G174644	GRMZM2G174644_P01	TRUE	TRUE	sGVVLDLVPPLTk	90%	n+304 (+304), K+304 (+304)	25.33	25.00
6657	seq=translation; coord=1:50252957..50255269:1; parent_transcript=GRMZM2G033491_T01; parent_gene=GRMZM2G033491	GRMZM2G033491_P01	TRUE	TRUE	aIEVAEAIVR	87%	n+304 (+304)	26.44	25.71
6658	seq=translation; coord=6:109592211..109594652:-1; parent_transcript=GRMZM2G008456_T01; parent_gene=GRMZM2G008456	GRMZM2G008456_P01	TRUE	TRUE	eMIMQALTELR	95%	n+304 (+304)	31.51	25.00
6659	seq=translation; coord=6:6432070..6436373:-1; parent_transcript=GRMZM2G009661_T01; parent_gene=GRMZM2G009661	GRMZM2G009661_P01,GRMZM2G009661_P02, GRMZM2G369912_P01,GRMZM2G369912_P02	TRUE	TRUE	vILPIIFPALER	90%	n+304 (+304)	26.44	25.00
6660	seq=translation; coord=6:365874..371557:-1; parent_transcript=GRMZM2G021784_T01; parent_gene=GRMZM2G021784	GRMZM2G021784_P01,GRMZM2G021784_P02, GRMZM2G451366_P01,GRMZM2G451366_P02, GRMZM2G451366_P03	TRUE	TRUE	sALVAPLLDLPLSFLR	95%	n+304 (+304)	27.78	25.00
6661	seq=translation; coord=1:183447693..183452321:1; parent_transcript=GRMZM2G042371_T01; parent_gene=GRMZM2G042371	GRMZM2G042371_P01	TRUE	TRUE	vFEAPLSFLk	92%	n+304 (+304), K+304 (+304)	26.14	25.00
6662	seq=translation; coord=4:236083133..236088143:-1; parent_transcript=GRMZM2G051004_T01; parent_gene=GRMZM2G051004	GRMZM2G051004_P01	TRUE	TRUE	vLDLIAHMALVSAAAk	95%	n+304 (+304), K+304 (+304)	28.22	25.00
6663	seq=translation; coord=1:295996730..296001956:1; parent_transcript=GRMZM2G053916_T01; parent_gene=GRMZM2G053916	GRMZM2G053916_P01,GRMZM2G053916_P06	TRUE	TRUE	iVAPDLYMAFGVSGAIQHLAGMR	95%	n+304 (+304)	70.79	25.95
6664	seq=translation; coord=8:70944999..70971683:1; parent_transcript=GRMZM2G058913_T01; parent_gene=GRMZM2G058913	GRMZM2G058913_P01,GRMZM2G130586_P01, GRMZM2G130586_P02,GRMZM2G130586_P03	TRUE	TRUE	ecGMDPDEAVSR	95%	n+304 (+304), Carbamidomethyl (+57)	37.22	25.00

6665	seq=translation; coord=3:227412676..227417690:-1; parent_transcript=GRMZM2G066101_T01; parent_gene=GRMZM2G066101	GRMZM2G066101_P01,GRMZM2G327032_P01, GRMZM2G327032_P02	TRUE	TRUE	fDLIYLILDk	95%	n+304 (+304), K+304 (+304)	31.34	25.00
6666	seq=translation; coord=3:229845805..229849321:1; parent_transcript=GRMZM2G078592_T01; parent_gene=GRMZM2G078592	GRMZM2G078592_P01,GRMZM2G096972_P01	TRUE	TRUE	tNVDMVTGQLSDTR	95%	n+304 (+304)	45.50	25.00
6667	seq=translation; coord=1:183993561..184006197:1; parent_transcript=GRMZM2G080775_T01; parent_gene=GRMZM2G080775	GRMZM2G080775_P01,GRMZM2G115855_P01	TRUE	TRUE	gLLSEAVLALEAEVLk	94%	n+304 (+304), K+304 (+304)	27.58	25.00
6668	seq=translation; coord=7:120201746..120203011:1; parent_transcript=GRMZM2G138727_T02; parent_gene=GRMZM2G138727	GRMZM2G138727_P02	TRUE	TRUE	gAVGGADSAATDGDVRPAAADSMPLR	95%	n+304 (+304)	30.41	25.00
6669	seq=translation; coord=4:24085992..24091476:1; parent_transcript=GRMZM2G162859_T01; parent_gene=GRMZM2G162859	GRMZM2G162859_P01,GRMZM2G162859_P02, GRMZM5G812289_P01,GRMZM5G812289_P02	TRUE	TRUE	eADkPIDELLLALLR	95%	n+304 (+304), K+304 (+304)	26.82	25.00
6670	seq=translation; coord=3:176257536..176261095:1; parent_transcript=GRMZM2G164141_T01; parent_gene=GRMZM2G164141	GRMZM2G164141_P01,GRMZM2G164141_P02, GRMZM2G164141_P03	TRUE	TRUE	tTQSGFEGFVTDR	95%	n+304 (+304)	32.84	25.00
6671	seq=translation; coord=3:201894260..201901554:1; parent_transcript=GRMZM2G167856_T01; parent_gene=GRMZM2G167856	GRMZM2G167856_P01	TRUE	TRUE	wVGTLFALk	95%	n+304 (+304), K+304 (+304)	39.11	25.00
6672	seq=translation; coord=5:91582816..91615388:1; parent_transcript=GRMZM2G174785_T01; parent_gene=GRMZM2G174785	GRMZM2G133308_P01,GRMZM2G133308_P02, GRMZM2G174785_P01,GRMZM2G174785_P02	TRUE	TRUE	gNPLFDFLFNLk	95%	n+304 (+304), K+304 (+304)	31.31	25.00
6673	seq=translation; coord=4:228030455..228034349:-1; parent_transcript=GRMZM2G345055_T01; parent_gene=GRMZM2G345055	GRMZM2G345055_P01,GRMZM2G345055_P02	TRUE	TRUE	dALEEQGYTEDEIEER	95%	n+304 (+304)	34.40	25.00
6674	seq=translation; coord=6:134303791..134311555:1; parent_transcript=GRMZM2G424783_T01; parent_gene=GRMZM2G424783	GRMZM2G424783_P01,GRMZM2G424783_P02	TRUE	TRUE	yLVGVPQVLLDFIR	91%	n+304 (+304)	25.39	25.00
6675	seq=translation; coord=6:91630823..91633819:1; parent_transcript=GRMZM2G428518_T03; parent_gene=GRMZM2G428518	GRMZM2G428518_P03	TRUE	TRUE	lLPYMYTLMYEAHTTGAPIAR	95%	n+304 (+304)	83.17	25.54
6676	seq=translation; coord=9:68025092..68028223:1; parent_transcript=GRMZM2G442129_T01; parent_gene=GRMZM2G442129	GRMZM2G442129_P01,GRMZM5G839924_P01	TRUE	TRUE	vVSEPIEMTQEFR	86%	n+304 (+304)	25.12	25.00
6677	seq=translation; coord=10:95762835..95768339:-1; parent_transcript=GRMZM2G154509_T01; parent_gene=GRMZM2G154509	GRMZM2G154509_P01,GRMZM2G154509_P02, GRMZM2G154509_P04,GRMZM2G160184_P01, GRMZM2G160184_P03,GRMZM2G160184_P04	TRUE	TRUE	fMDWLWTR	92%	n+304 (+304)	29.00	25.00
6678	GRMZM2G178261_P04-R	GRMZM2G178261_P04-R	TRUE	TRUE	vVVLHMLFk	91%	n+304 (+304), K+304 (+304)	25.20	25.00
6679	seq=translation; coord=5:216495038..216502228:-1; parent_transcript=GRMZM2G013890_T01; parent_gene=GRMZM2G013890	GRMZM2G013890_P01	TRUE	TRUE	eFLNTVVTDILHLHGR	95%	n+304 (+304)	56.55	25.54
6680	seq=translation; coord=4:186487329..186488873:1; parent_transcript=GRMZM2G022799_T01; parent_gene=GRMZM2G022799	GRMZM2G022799_P01	TRUE	TRUE	acGAFSAALQVVLAAHPAPLTNR	95%	n+304 (+304), Carbamidomethyl (+57)	45.45	25.39
6681	seq=translation; coord=6:166051912..166055277:1; parent_transcript=GRMZM2G026013_T02; parent_gene=GRMZM2G026013	GRMZM2G026013_P02,GRMZM2G026013_P04	TRUE	TRUE	eDLVQSAVSFLk	95%	n+304 (+304), K+304 (+304)	40.29	25.67
6682	seq=translation; coord=6:166051912..166055277:1; parent_transcript=GRMZM2G026013_T02; parent_gene=GRMZM2G026013	GRMZM2G026013_P02,GRMZM2G026013_P04	TRUE	TRUE	vEPEAGPVAPVVPR	95%	n+304 (+304)	34.88	25.05

6683	seq=translation; coord=10:14974709..14978322:1; parent_transcript=GRMZM2G081541_T01; parent_gene=GRMZM2G081541	GRMZM2G081541_P01	TRUE	TRUE	aAELLFGLGFNk	95%	n+304 (+304), K+304 (+304)	45.93	25.00
6684	seq=translation; coord=6:89823772..89828523:1; parent_transcript=GRMZM2G082322_T01; parent_gene=GRMZM2G082322	GRMZM2G082322_P01,GRMZM2G082322_P02	TRUE	TRUE	lPFAPALAEALk	95%	n+304 (+304), K+304 (+304)	34.35	25.01
6685	seq=translation; coord=3:27391353..27395051:1; parent_transcript=GRMZM2G089365_T01; parent_gene=GRMZM2G089365	GRMZM2G089365_P01	TRUE	TRUE	aAAGSYSDELISTAk	95%	n+304 (+304), K+304 (+304)	33.65	25.24
6686	seq=translation; coord=3:27391353..27395051:1; parent_transcript=GRMZM2G089365_T01; parent_gene=GRMZM2G089365	GRMZM2G089365_P01	TRUE	TRUE	lSSIGLDNTEVNR	95%	n+304 (+304)	36.64	25.20
6687	seq=translation; coord=10:141350790..141353378:1; parent_transcript=GRMZM2G094051_T01; parent_gene=GRMZM2G094051	GRMZM2G094051_P01,GRMZM2G094051_P03, GRMZM2G094051_P04	TRUE	TRUE	iPLSNNQVIEEGLGk	95%	n+304 (+304), K+304 (+304)	33.59	25.19
6688	seq=translation; coord=1:27626302..27631083:1; parent_transcript=GRMZM2G111324_T01; parent_gene=GRMZM2G111324	GRMZM2G111324_P01,GRMZM2G111324_P02, GRMZM5G824920_P01	TRUE	TRUE	mLQAALDWAcGPGk	95%	n+304 (+304), Carbamidomethyl (+57), K+304 (+304)	36.29	25.39
6689	seq=translation; coord=6:81614390..81617700:-1; parent_transcript=GRMZM2G121354_T01; parent_gene=GRMZM2G121354	GRMZM2G121354_P01,GRMZM2G132060_P01, GRMZM2G132060_P02	TRUE	TRUE	mDIDYQVLHDAFFk	95%	n+304 (+304), K+304 (+304)	37.04	25.21
6690	seq=translation; coord=10:123373488..123378767:-1; parent_transcript=GRMZM2G135470_T01; parent_gene=GRMZM2G135470	GRMZM2G135470_P01	TRUE	TRUE	sPLIVFDDIGDIDk	95%	n+304 (+304), K+304 (+304)	42.00	25.80
6691	seq=translation; coord=9:8943775..8956824:1; parent_transcript=GRMZM2G142072_T01; parent_gene=GRMZM2G142072	GRMZM2G142072_P01,GRMZM2G171488_P01, GRMZM2G171488_P02	TRUE	TRUE	iLDMVILAk	95%	n+304 (+304), K+304 (+304)	33.07	25.00
6692	seq=translation; coord=6:93327267..93330462:-1; parent_transcript=GRMZM2G143767_T01; parent_gene=GRMZM2G143767	GRMZM2G143767_P01	TRUE	TRUE	aAAATADAGGPTIFDk	95%	n+304 (+304), K+304 (+304)	37.73	26.18
6693	seq=translation; coord=8:120773908..120778906:-1; parent_transcript=GRMZM2G083016_T01; parent_gene=GRMZM2G083016	GRMZM2G083016_P01	TRUE	TRUE	iGVIESLLEk	95%	n+304 (+304), K+304 (+304)	28.77	25.00
6694	seq=translation; coord=7:149250816..149253354:1; parent_transcript=GRMZM2G084757_T01; parent_gene=GRMZM2G084757	GRMZM2G084757_P01,GRMZM2G084757_P02, GRMZM2G180724_P01	TRUE	TRUE	yFEIILVDVAHTAIR	95%	n+304 (+304), iTRAQ8plex (+304)	28.90	25.00
6695	seq=translation; coord=5:180632305..180634311:1; parent_transcript=GRMZM2G106061_T01; parent_gene=GRMZM2G106061	GRMZM2G106061_P01,GRMZM2G407996_P01, GRMZM2G407996_P02	TRUE	TRUE	iVVFLNk	94%	n+304 (+304), K+304 (+304)	26.98	25.00
6696	seq=translation; coord=5:180632305..180634311:1; parent_transcript=GRMZM2G106061_T01; parent_gene=GRMZM2G106061	GRMZM2G106061_P01,GRMZM2G407996_P01, GRMZM2G407996_P02	TRUE	TRUE	tTLTAALTMVLASVGGSAPk	95%	n+304 (+304), K+304 (+304)	40.31	25.00
6697	seq=translation; coord=8:76128223..76129850:1; parent_transcript=AC231180.2_FGT006; parent_gene=AC231180.2_FG006	AC231180.2_FGP006	TRUE	TRUE	gAWMSVASFGAR	95%	n+304 (+304)	38.43	25.00
6698	seq=translation; coord=8:76128223..76129850:1; parent_transcript=AC231180.2_FGT006; parent_gene=AC231180.2_FG006	AC231180.2_FGP006	TRUE	TRUE	gDGQTLNTGAFAR	95%	n+304 (+304)	33.22	25.00
6699	seq=translation; coord=7:25812344..25817942:1; parent_transcript=GRMZM2G003789_T01; parent_gene=GRMZM2G003789	GRMZM2G003789_P01	TRUE	TRUE	aDMDALPVQELVDWEHk	95%	n+304 (+304), K+304 (+304)	87.16	25.44
6700	seq=translation; coord=7:25812344..25817942:1; parent_transcript=GRMZM2G003789_T01; parent_gene=GRMZM2G003789	GRMZM2G003789_P01	TRUE	TRUE	eIDPLQGAVVSVTFLk	95%	n+304 (+304), K+304 (+304)	39.13	25.00

6701	seq=translation; coord=1:171489898..171496325:1; parent_transcript=GRMZM2G019926_T02; parent_gene=GRMZM2G019926	GRMZM2G019926_P02,GRMZM2G019926_P03, GRMZM2G019926_P04	TRUE	TRUE	gLVDTHFIER	95%	n+304 (+304)	32.25	26.11
6702	seq=translation; coord=1:171489898..171496325:1; parent_transcript=GRMZM2G019926_T02; parent_gene=GRMZM2G019926	GRMZM2G019926_P02,GRMZM2G019926_P03, GRMZM2G019926_P04	TRUE	TRUE	gSVLAPMAGLVVk	95%	n+304 (+304), K+304 (+304)	35.75	25.00
6703	seq=translation; coord=2:8914517..8917818:-1; parent_transcript=GRMZM2G076239_T02; parent_gene=GRMZM2G076239	GRMZM2G076239_P02,GRMZM2G076239_P03, GRMZM2G076239_P04,GRMZM2G076239_P05	TRUE	TRUE	aIAVTVDAPR	95%	n+304 (+304)	40.15	27.02
6704	seq=translation; coord=2:8914517..8917818:-1; parent_transcript=GRMZM2G076239_T02; parent_gene=GRMZM2G076239	GRMZM2G076239_P02,GRMZM2G076239_P03, GRMZM2G076239_P04,GRMZM2G076239_P05	TRUE	TRUE	rVPVFLDGGIR	95%	n+304 (+304)	48.54	25.00
6705	seq=translation; coord=3:205146239..205150759:-1; parent_transcript=GRMZM2G098569_T01; parent_gene=GRMZM2G098569	GRMZM2G098569_P01	TRUE	TRUE	lEEVFHEYER	95%	n+304 (+304)	31.96	25.00
6706	seq=translation; coord=3:205146239..205150759:-1; parent_transcript=GRMZM2G098569_T01; parent_gene=GRMZM2G098569	GRMZM2G098569_P01	TRUE	TRUE	vPQVGLIAVNDGIILR	95%	n+304 (+304)	31.76	25.00
6707	seq=translation; coord=6:105434141..105436791:1; parent_transcript=GRMZM2G403076_T01; parent_gene=GRMZM2G403076	GRMZM2G403076_P01	TRUE	TRUE	sQRPEAFAVVDLSSLR	95%	n+304 (+304)	51.47	25.49
6708	seq=translation; coord=6:105434141..105436791:1; parent_transcript=GRMZM2G403076_T01; parent_gene=GRMZM2G403076	GRMZM2G403076_P01	TRUE	TRUE	wQEVAPALPEDLFIR	95%	n+304 (+304)	39.23	25.56
6709	seq=translation; coord=4:18676199..18682894:1; parent_transcript=GRMZM2G574782_T01; parent_gene=GRMZM2G574782	GRMZM2G574782_P01	TRUE	TRUE	lIVMSPSGVQk	95%	n+304 (+304), K+304 (+304)	38.01	25.16
6710	seq=translation; coord=4:18676199..18682894:1; parent_transcript=GRMZM2G574782_T01; parent_gene=GRMZM2G574782	GRMZM2G574782_P01	TRUE	TRUE	qLQGHIWR	95%	Pyro-cmC (-17), n+304 (+304)	46.66	25.00
6711	seq=translation; coord=8:152750732..152754708:1; parent_transcript=GRMZM5G823017_T01; parent_gene=GRMZM5G823017	GRMZM5G823017_P01,GRMZM5G823017_P02, GRMZM5G823017_P03	TRUE	TRUE	aEGTEAESGGDk	95%	n+304 (+304), K+304 (+304)	33.11	25.00
6712	seq=translation; coord=8:152750732..152754708:1; parent_transcript=GRMZM5G823017_T01; parent_gene=GRMZM5G823017	GRMZM5G823017_P01,GRMZM5G823017_P02, GRMZM5G823017_P03	TRUE	TRUE	aVFTADSALYEYLDGGWk	95%	n+304 (+304), K+304 (+304)	65.30	25.45
6713	seq=translation; coord=8:118191461..118195143:-1; parent_transcript=AC197705.4_FGT007; parent_gene=AC197705.4_FG007	AC197705.4_FGP007	TRUE	TRUE	lHDLDIFDAVk	94%	n+304 (+304), K+304 (+304)	27.71	25.05
6714	seq=translation; coord=8:118191461..118195143:-1; parent_transcript=AC197705.4_FGT007; parent_gene=AC197705.4_FG007	AC197705.4_FGP007	TRUE	TRUE	tQDELIEAEVAELLR	95%	n+304 (+304)	76.58	25.68
6715	seq=translation; coord=8:29145636..29147578:-1; parent_transcript=GRMZM2G007151_T01; parent_gene=GRMZM2G007151	GRMZM2G007151_P01,GRMZM2G007151_P02	TRUE	TRUE	aAAAGFFk	94%	n+304 (+304), K+304 (+304)	27.95	25.00
6716	seq=translation; coord=8:29145636..29147578:-1; parent_transcript=GRMZM2G007151_T01; parent_gene=GRMZM2G007151	GRMZM2G007151_P01,GRMZM2G007151_P02	TRUE	TRUE	sGASPLSPAITFILDk	95%	n+304 (+304), K+304 (+304)	29.60	25.00
6717	seq=translation; coord=3:2080450..2084636:1; parent_transcript=GRMZM2G153181_T01; parent_gene=GRMZM2G153181	GRMZM2G153181_P01	TRUE	TRUE	fEEMIGELEk	95%	n+304 (+304), K+304 (+304)	40.96	25.40
6718	seq=translation; coord=3:2080450..2084636:1; parent_transcript=GRMZM2G153181_T01; parent_gene=GRMZM2G153181	GRMZM2G153181_P01	TRUE	TRUE	iLLYILk	93%	n+304 (+304), K+304 (+304)	27.56	25.00

6719	seq=translation; coord=5:58525626..58534933:-1; parent_transcript=GRMZM2G397247_T04; parent_gene=GRMZM2G397247	GRMZM2G397247_P04	TRUE	TRUE	aGADVVSPSDMMDGR	95%	n+304 (+304)	57.62	25.00
6720	seq=translation; coord=5:58525626..58534933:-1; parent_transcript=GRMZM2G397247_T04; parent_gene=GRMZM2G397247	GRMZM2G397247_P04	TRUE	TRUE	dVGVNSFVLFPk	93%	n+304 (+304), K+304 (+304)	28.15	25.91
6721	seq=translation; coord=1:210463083..210467521:1; parent_transcript=GRMZM2G073814_T02; parent_gene=GRMZM2G073814	GRMZM2G073814_P02	TRUE	TRUE	aLGVELVSFDEAIAR	92%	n+304 (+304)	27.26	25.72
6722	seq=translation; coord=1:210463083..210467521:1; parent_transcript=GRMZM2G073814_T02; parent_gene=GRMZM2G073814	GRMZM2G073814_P02	TRUE	TRUE	lSEAGLAVLR	95%	n+304 (+304)	43.26	25.00
6723	seq=translation; coord=10:16517475..16520678:1; parent_transcript=GRMZM2G091819_T01; parent_gene=GRMZM2G091819	GRMZM2G091819_P01	TRUE	TRUE	ySTSVEScEYDEASGR	95%	n+304 (+304), Carbamidomethyl (+57)	74.46	25.00
6724	seq=translation; coord=10:16517475..16520678:1; parent_transcript=GRMZM2G091819_T01; parent_gene=GRMZM2G091819	GRMZM2G091819_P01	TRUE	TRUE	yVDDYVER	91%	n+304 (+304)	26.76	25.00
6725	seq=translation; coord=4:151648685..151655399:1; parent_transcript=GRMZM2G145226_T01; parent_gene=GRMZM2G145226	GRMZM2G145226_P01	TRUE	TRUE	iAENPNSMNFNVMALSk	95%	n+304 (+304), K+304 (+304)	67.52	25.55
6726	seq=translation; coord=4:151648685..151655399:1; parent_transcript=GRMZM2G145226_T01; parent_gene=GRMZM2G145226	GRMZM2G145226_P01	TRUE	TRUE	lVEGSYFEk	91%	n+304 (+304), K+304 (+304)	25.10	25.07
6727	seq=translation; coord=1:287289676..287296815:-1; parent_transcript=GRMZM2G178415_T01; parent_gene=GRMZM2G178415	GRMZM2G178415_P01,GRMZM2G178415_P02, GRMZM2G178415_P05,GRMZM2G178415_P06	TRUE	TRUE	gVLFATEALLAEHGk	95%	n+304 (+304), K+304 (+304)	67.07	25.61
6728	seq=translation; coord=1:287289676..287296815:-1; parent_transcript=GRMZM2G178415_T01; parent_gene=GRMZM2G178415	GRMZM2G178415_P01,GRMZM2G178415_P02, GRMZM2G178415_P05,GRMZM2G178415_P06	TRUE	TRUE	sLLIPFR	90%	n+304 (+304)	27.63	25.00
6729	seq=translation; coord=8:64567512..64568715:-1; parent_transcript=GRMZM2G075796_T01; parent_gene=GRMZM2G075796	GRMZM2G075796_P01	TRUE	TRUE	lHDAADEIESLR	90%	n+304 (+304)	27.37	25.00
6730	seq=translation; coord=8:64567512..64568715:-1; parent_transcript=GRMZM2G075796_T01; parent_gene=GRMZM2G075796	GRMZM2G075796_P01	TRUE	TRUE	lQDLLAAEk	95%	n+304 (+304), K+304 (+304)	33.11	26.70
6731	seq=translation; coord=9:143391688..143394080:-1; parent_transcript=GRMZM2G140799_T01; parent_gene=GRMZM2G140799	GRMZM2G140799_P01	TRUE	TRUE	aYPLADAQLTMGILDIIQQAANYk	95%	n+304 (+304), K+304 (+304)	61.94	25.00
6732	seq=translation; coord=1:35239930..35250741:-1; parent_transcript=GRMZM2G030422_T01; parent_gene=GRMZM2G030422	GRMZM2G030422_P01,GRMZM2G030422_P02, GRMZM2G030422_P03	TRUE	TRUE	dGESHLVEWNESEGAVk	95%	n+304 (+304), K+304 (+304)	36.35	25.00
6733	seq=translation; coord=5:74642485..74645616:-1; parent_transcript=GRMZM2G017229_T01; parent_gene=GRMZM2G017229	GRMZM2G017229_P01	TRUE	TRUE	eTSEGTEEDDAAk	95%	n+304 (+304), K+304 (+304)	50.56	25.00
6734	seq=translation; coord=1:7456720..7458916:-1; parent_transcript=GRMZM2G092719_T01; parent_gene=GRMZM2G092719	GRMZM2G092719_P01	TRUE	TRUE	nSIGcLFWLLSR	95%	n+304 (+304), Carbamidomethyl (+57)	35.74	26.04
6735	seq=translation; coord=5:6157585..6167807:-1; parent_transcript=GRMZM2G171588_T01; parent_gene=GRMZM2G171588	GRMZM2G171588_P01,GRMZM2G171588_P02, GRMZM5G886709_P01	TRUE	TRUE	eQVDLFLAEVAAk	94%	n+304 (+304), K+304 (+304)	28.50	25.37
6736	seq=translation; coord=5:6157585..6167807:-1; parent_transcript=GRMZM2G171588_T01; parent_gene=GRMZM2G171588	GRMZM2G171588_P01,GRMZM2G171588_P02, GRMZM5G886709_P01	TRUE	TRUE	lcSLSPALQEFVGASELAR	89%	n+304 (+304), Carbamidomethyl (+57)	25.17	25.00

6737	seq=translation; coord=4:162265417..162267770:-1; parent_transcript=GRMZM2G064960_T01; parent_gene=GRMZM2G064960	GRMZM2G064960_P01,GRMZM2G064960_P02	TRUE	TRUE	dPDQEEPPAPASAAEAEk	95%	n+304 (+304), K+304 (+304)	40.33	25.00
6738	seq=translation; coord=6:163979437..163984362:1; parent_transcript=GRMZM2G150257_T01; parent_gene=GRMZM2G150257	GRMZM2G150257_P01	TRUE	TRUE	eQVDLLVSQLQALGAR	95%	n+304 (+304)	39.81	25.15
6739	seq=translation; coord=5:3067129..3072217:1; parent_transcript=GRMZM2G057329_T01; parent_gene=GRMZM2G057329	GRMZM2G057329_P01	TRUE	TRUE	vLGDGLLVVR	95%	n+304 (+304)	30.55	25.00
6740	seq=translation; coord=5:144281597..144283761:-1; parent_transcript=GRMZM2G049390_T01; parent_gene=GRMZM2G049390	GRMZM2G049390_P01,GRMZM2G049390_P03	TRUE	TRUE	eLELLSDGANVYk	95%	n+304 (+304), K+304 (+304)	65.86	25.28
6741	seq=translation; coord=4:38821429..38825409:1; parent_transcript=GRMZM2G159250_T01; parent_gene=GRMZM2G159250	GRMZM2G159250_P01,GRMZM2G159250_P02	TRUE	TRUE	aIETVQANFNTVR	95%	n+304 (+304)	35.35	25.00
6742	seq=translation; coord=1:1665098..1666489:1; parent_transcript=GRMZM2G474194_T01; parent_gene=GRMZM2G474194	GRMZM2G474194_P01	TRUE	TRUE	vPcQYPGLTVTFHIER	92%	n+304 (+304), Carbamidomethyl (+57)	27.25	25.75
6743	seq=translation; coord=1:205574134..205574917:-1; parent_transcript=GRMZM2G015588_T01; parent_gene=GRMZM2G015588	GRMZM2G015588_P01,GRMZM2G095579_P01, GRMZM2G095579_P02	TRUE	TRUE	gVLDYLGVEITR	95%	n+304 (+304)	33.36	25.07
6744	seq=translation; coord=1:189474106..189481738:1; parent_transcript=GRMZM2G004582_T01; parent_gene=GRMZM2G004582	GRMZM2G004582_P01,GRMZM2G004582_P02, GRMZM2G004582_P03,GRMZM2G004582_P04, GRMZM2G004582_P05,GRMZM2G004582_P07, GRMZM2G004582_P08,GRMZM2G039359_P04	TRUE	TRUE	lVLAAAAILHR	95%	n+304 (+304)	69.79	25.00
6745	seq=translation; coord=6:162306497..162311290:-1; parent_transcript=GRMZM2G170400_T01; parent_gene=GRMZM2G170400	GRMZM2G170400_P01	TRUE	TRUE	eAGHVLALDFIESVIk	95%	n+304 (+304), K+304 (+304)	31.41	25.00
6746	seq=translation; coord=8:8281951..8288329:1; parent_transcript=GRMZM5G860137_T01; parent_gene=GRMZM5G860137	GRMZM5G860137_P01,GRMZM5G860137_P02	TRUE	TRUE	tPIGALLGSLSSLPATk	95%	n+304 (+304), K+304 (+304)	47.30	25.00
6747	seq=translation; coord=10:141964000..141969154:-1; parent_transcript=GRMZM2G028929_T01; parent_gene=GRMZM2G028929	GRMZM2G028929_P01,GRMZM2G028929_P02, GRMZM2G028929_P04	TRUE	TRUE	eAYLIFQDFAEk	95%	n+304 (+304), K+304 (+304)	37.97	26.12
6748	seq=translation; coord=4:145240980..145242532:1; parent_transcript=GRMZM2G111756_T01; parent_gene=GRMZM2G111756	GRMZM2G111756_P01	TRUE	TRUE	dLFMDPDEAR	95%	n+304 (+304)	42.50	25.00
6749	seq=translation; coord=7:138559787..138562909:1; parent_transcript=GRMZM5G876773_T01; parent_gene=GRMZM5G876773	GRMZM5G876773_P01	TRUE	TRUE	iYLQQPAPESQLR	95%	n+304 (+304)	34.52	25.00
6750	seq=translation; coord=5:77757345..77777262:-1; parent_transcript=GRMZM2G011456_T02; parent_gene=GRMZM2G011456	GRMZM2G011456_P02,GRMZM2G011456_P03, GRMZM2G169927_P01	TRUE	TRUE	lEGWESLVTVISSDMR	94%	n+304 (+304)	27.12	25.00
6751	seq=translation; coord=8:175400786..175402729:-1; parent_transcript=GRMZM2G002786_T01; parent_gene=GRMZM2G002786	GRMZM2G002786_P01	TRUE	TRUE	sPPNIPEVk	95%	n+304 (+304), K+304 (+304)	37.33	25.00
6752	seq=translation; coord=6:141144515..141145787:-1; parent_transcript=GRMZM2G030116_T01; parent_gene=GRMZM2G030116	GRMZM2G030116_P01,GRMZM2G034810_P01, GRMZM2G135332_P01,GRMZM2G359892_P01, GRMZM2G413687_P01	TRUE	TRUE	qAWGSLDALIGR	95%	n+304 (+304)	43.90	26.54
6753	seq=translation; coord=2:45518503..45524129:1; parent_transcript=GRMZM2G131321_T01; parent_gene=GRMZM2G131321	GRMZM2G131321_P01,GRMZM2G131321_P02, GRMZM2G131321_P03	TRUE	TRUE	eGVLVEGLFWk	95%	n+304 (+304), K+304 (+304)	40.14	25.60

6754	seq=translation; coord=9:14820841..14824999:-1; parent_transcript=GRMZM2G167156_T01; parent_gene=GRMZM2G167156	GRMZM2G167156_P01,GRMZM2G167156_P02	TRUE	TRUE	dVLQSLMAk	92%	n+304 (+304), K+304 (+304)	26.13	25.49
6755	seq=translation; coord=5:19048283..19055775:1; parent_transcript=GRMZM2G375984_T01; parent_gene=GRMZM2G375984	GRMZM2G375984_P01,GRMZM2G375984_P02	TRUE	TRUE	fVDDGTSSk	89%	n+304 (+304), K+304 (+304)	28.25	26.28
6756	seq=translation; coord=5:202224551..202225926:1; parent_transcript=GRMZM2G475349_T01; parent_gene=GRMZM2G475349	GRMZM2G475349_P01	TRUE	TRUE	lAGEDIAHLDDLIAVLSk	95%	n+304 (+304), K+304 (+304)	39.48	25.00
6757	seq=translation; coord=1:255254285..255262311:1; parent_transcript=GRMZM2G016875_T01; parent_gene=GRMZM2G016875	GRMZM2G016875_P01	TRUE	TRUE	tILQDVLEQk	91%	n+304 (+304), K+304 (+304)	26.22	25.68
6758	seq=translation; coord=1:193317120..193323452:1; parent_transcript=GRMZM2G424241_T01; parent_gene=GRMZM2G424241	GRMZM2G424241_P01,GRMZM2G424241_P03, GRMZM2G424241_P04	TRUE	TRUE	gRPWFGLLALLR	92%	n+304 (+304)	26.40	25.07
6759	seq=translation; coord=7:141644109..141654531:-1; parent_transcript=GRMZM2G073504_T01; parent_gene=GRMZM2G073504	GRMZM2G073504_P01	TRUE	TRUE	tDkLQFLFDFLDIGR	92%	n+304 (+304), K+304 (+304)	26.48	25.00
6760	seq=translation; coord=3:23430825..23436929:1; parent_transcript=GRMZM2G121683_T01; parent_gene=GRMZM2G121683	GRMZM2G121683_P01,GRMZM2G155709_P01, GRMZM2G155709_P02,GRMZM2G155709_P03	TRUE	TRUE	eAVDVFIWcLTQNAESYk	95%	n+304 (+304), Carbamidomethyl (+57), K+304 (+304)	30.24	25.00
6761	seq=translation; coord=3:23430825..23436929:1; parent_transcript=GRMZM2G121683_T01; parent_gene=GRMZM2G121683	GRMZM2G121683_P01,GRMZM2G155709_P01, GRMZM2G155709_P02,GRMZM2G155709_P03	TRUE	TRUE	lVPPVSFDLFMR	89%	n+304 (+304)	27.04	25.00
6762	seq=translation; coord=4:220605264..220609013:1; parent_transcript=GRMZM2G125239_T01; parent_gene=GRMZM2G125239	GRMZM2G125239_P01,GRMZM2G125239_P02, GRMZM2G125239_P03	TRUE	TRUE	eSAPSTANQSGDSQk	93%	n+304 (+304), K+304 (+304)	26.31	25.00
6763	seq=translation; coord=4:220605264..220609013:1; parent_transcript=GRMZM2G125239_T01; parent_gene=GRMZM2G125239	GRMZM2G125239_P01,GRMZM2G125239_P02, GRMZM2G125239_P03	TRUE	TRUE	vFEFIADEk	91%	n+304 (+304), K+304 (+304)	26.55	25.50
6764	seq=translation; coord=9:139614346..139617289:1; parent_transcript=GRMZM2G103490_T01; parent_gene=GRMZM2G103490	GRMZM2G103490_P01,GRMZM2G103490_P02, GRMZM2G160906_P01,GRMZM2G160906_P02, GRMZM2G160906_P03,GRMZM2G160906_P04	TRUE	TRUE	lLETLEEFMR	93%	n+304 (+304)	30.38	25.19
6765	seq=translation; coord=1:223161919..223165331:1; parent_transcript=GRMZM2G450498_T01; parent_gene=GRMZM2G450498	GRMZM2G450498_P01	TRUE	TRUE	aTFVDADDEAAVR	95%	n+304 (+304)	44.20	25.00
6766	seq=translation; coord=3:201782882..201788032:1; parent_transcript=GRMZM2G171426_T01; parent_gene=GRMZM2G171426	GRMZM2G171426_P01	TRUE	TRUE	sTGFGLIYDNLEAAk	95%	n+304 (+304), K+304 (+304)	41.07	25.73
6767	seq=translation; coord=7:153816870..153819108:1; parent_transcript=GRMZM2G009412_T02; parent_gene=GRMZM2G009412	GRMZM2G009412_P02,GRMZM2G009412_P04, GRMZM2G103059_P01,GRMZM2G125527_P01, GRMZM2G125527_P02,GRMZM2G160351_P01, GRMZM2G160351_P02	TRUE	TRUE	lQcQSck	95%	n+304 (+304), Carbamidomethyl (+57), Carbamidomethyl (+57), K+304 (+304)	33.20	25.29
6768	seq=translation; coord=1:241508117..241529441:-1; parent_transcript=GRMZM2G520535_T01; parent_gene=GRMZM2G520535	GRMZM2G520535_P01	TRUE	TRUE	gFLSDAEcDHLIALAk	95%	n+304 (+304), Carbamidomethyl (+57), K+304 (+304)	26.54	25.29
6769	seq=translation; coord=1:227250144..227255278:-1; parent_transcript=GRMZM2G449083_T01; parent_gene=GRMZM2G449083	GRMZM2G449083_P01,GRMZM2G449083_P02, GRMZM2G449083_P03,GRMZM2G449083_P04, GRMZM2G449083_P06	TRUE	TRUE	lPFQEMYAILEAMk	90%	n+304 (+304), K+304 (+304)	25.88	25.60
6770	seq=translation; coord=1:233330196..233331681:-1; parent_transcript=AC198361.3_FGT004; parent_gene=AC198361.3_FG004	AC198361.3_FGP004	TRUE	TRUE	gFGFVVFADPAVVDR	95%	n+304 (+304)	31.76	25.42

6771	seq=translation; coord=9:142806737..142811172:-1; parent_transcript=AC205419.3_FGT001; parent_gene=AC205419.3_FG001	AC205419.3_FGP001,GRMZM2G165944_P01	TRUE	TRUE	lLEFEQELIR	95%	n+304 (+304)	32.85	25.01
6772	seq=translation; coord=5:210593958..210594236:-1; parent_transcript=AC216353.2_FGT005; parent_gene=AC216353.2_FG005	AC216353.2_FGP005	TRUE	TRUE	vIQETNESWEk	95%	n+304 (+304), K+304 (+304)	34.80	25.00
6773	seq=translation; coord=10:149747387..149749334:-1; parent_transcript=AC234201.1_FGT003; parent_gene=AC234201.1_FG003	AC234201.1_FGP003	TRUE	TRUE	lDDGLLDQIFR	95%	n+304 (+304)	31.47	25.04
6774	seq=translation; coord=2:12128999..12130348:1; parent_transcript=GRMZM2G000397_T01; parent_gene=GRMZM2G000397	GRMZM2G000397_P01	TRUE	TRUE	lLITLVAAPALAMATk	95%	n+304 (+304), K+304 (+304)	36.81	25.00
6775	seq=translation; coord=1:267153118..267154071:-1; parent_transcript=GRMZM2G004106_T01; parent_gene=GRMZM2G004106	GRMZM2G004106_P01,GRMZM2G004160_P01	TRUE	TRUE	gGWSFNTANWPk	95%	n+304 (+304), K+304 (+304)	44.59	25.00
6776	seq=translation; coord=9:131576429..131579600:-1; parent_transcript=GRMZM2G006080_T01; parent_gene=GRMZM2G006080	GRMZM2G006080_P01,GRMZM2G100288_P01	TRUE	TRUE	eEVSLAEWALHcQk	94%	n+304 (+304), Carbamidomethyl (+57), K+304 (+304)	26.38	25.59
6777	seq=translation; coord=8:149039624..149041045:1; parent_transcript=GRMZM2G007630_T01; parent_gene=GRMZM2G007630	GRMZM2G007630_P01	TRUE	TRUE	lALGPTYLVAR	95%	n+304 (+304)	32.09	25.00
6778	seq=translation; coord=7:148179239..148183212:-1; parent_transcript=GRMZM2G018950_T01; parent_gene=GRMZM2G018950	GRMZM2G018950_P01	TRUE	TRUE	hNEATISNDLYDFLQAFFAEHPk	95%	n+304 (+304), K+304 (+304)	43.39	25.00
6779	seq=translation; coord=1:85054120..85057214:1; parent_transcript=GRMZM2G026767_T02; parent_gene=GRMZM2G026767	GRMZM2G026767_P02,GRMZM2G026767_P03	TRUE	TRUE	aSGEVPALAEFSLAELR	95%	n+304 (+304)	49.59	25.76
6780	seq=translation; coord=1:81602813..81603918:-1; parent_transcript=GRMZM2G028821_T01; parent_gene=GRMZM2G028821	GRMZM2G028821_P01	TRUE	TRUE	lFPAWYGMVk	95%	n+304 (+304), K+304 (+304)	31.25	25.65
6781	seq=translation; coord=5:208090222..208094428:-1; parent_transcript=GRMZM2G035503_T01; parent_gene=GRMZM2G035503	GRMZM2G035503_P01	TRUE	TRUE	aATSFPQVILTAASFNPHLWYR	95%	n+304 (+304)	51.18	25.65
6782	seq=translation; coord=4:236110495..236114268:-1; parent_transcript=GRMZM2G051276_T01; parent_gene=GRMZM2G051276	GRMZM2G051276_P01	TRUE	TRUE	vAAPTTGPIPAAELLGVIEAAAk	95%	n+304 (+304), K+304 (+304)	35.50	25.00
6783	seq=translation; coord=1:189081768..189085843:-1; parent_transcript=GRMZM2G055992_T01; parent_gene=GRMZM2G055992	GRMZM2G055992_P01	TRUE	TRUE	iLALPEAIGMLSSLAk	94%	n+304 (+304), K+304 (+304)	28.09	25.00
6784	seq=translation; coord=6:22305548..22306995:-1; parent_transcript=GRMZM2G060373_T02; parent_gene=GRMZM2G060373	GRMZM2G060373_P02,GRMZM2G060373_P03, GRMZM2G165208_P01,GRMZM2G165619_P02, GRMZM2G165619_P03,GRMZM2G305154_P01	TRUE	TRUE	vcANPHGLIR	95%	n+304 (+304), Carbamidomethyl (+57)	34.48	25.00
6785	seq=translation; coord=6:142484063..142487768:-1; parent_transcript=GRMZM2G062481_T01; parent_gene=GRMZM2G062481	GRMZM2G062481_P01	TRUE	TRUE	tAVDFIASHPQAk	95%	n+304 (+304), K+304 (+304)	32.21	25.48
6786	seq=translation; coord=9:134132145..134139171:-1; parent_transcript=GRMZM2G064725_T01; parent_gene=GRMZM2G064725	GRMZM2G064725_P01	TRUE	TRUE	eIPALIVWTLGGk	95%	n+304 (+304), K+304 (+304)	30.78	25.00
6787	seq=translation; coord=2:230866079..230869236:-1; parent_transcript=GRMZM2G066902_T01; parent_gene=GRMZM2G066902	GRMZM2G066902_P01	TRUE	TRUE	eIAEAYLGTTVk	95%	n+304 (+304), K+304 (+304)	38.03	26.04

6788	seq=translation; coord=4:45266261..45269925:-1; parent_transcript=GRMZM2G070693_T01; parent_gene=GRMZM2G070693	GRMZM2G070693_P01,GRMZM2G070693_P02, GRMZM2G070693_P03,GRMZM2G070693_P04, GRMZM2G070693_P05,GRMZM2G083551_P01, GRMZM2G083551_P02,GRMZM2G083551_P03	TRUE	TRUE	lDEALYNIQFEQHWLEAQTDR	95%	n+304 (+304)	80.48	25.00
6789	seq=translation; coord=5:13280268..13284484:-1; parent_transcript=GRMZM2G076676_T01; parent_gene=GRMZM2G076676	GRMZM2G076676_P01,GRMZM2G388911_P01	TRUE	TRUE	skPVVAALIDELER	95%	n+304 (+304), K+304 (+304)	37.53	25.00
6790	seq=translation; coord=6:62544640..62546740:1; parent_transcript=GRMZM2G077837_T01; parent_gene=GRMZM2G077837	GRMZM2G077837_P01,GRMZM2G077837_P02, GRMZM2G077837_P03,GRMZM2G077837_P04, GRMZM2G077837_P05,GRMZM2G077837_P06	TRUE	TRUE	gTPLPLILLGk	95%	n+304 (+304), K+304 (+304)	32.18	25.00
6791	seq=translation; coord=7:98556648..98559891:-1; parent_transcript=GRMZM2G082019_T01; parent_gene=GRMZM2G082019	GRMZM2G082019_P01	TRUE	TRUE	akEEIEAIVEAIHPk	95%	n+304 (+304), K+304 (+304), K+304 (+304)	27.83	25.00
6792	seq=translation; coord=1:59061226..59087208:1; parent_transcript=GRMZM2G083689_T01; parent_gene=GRMZM2G083689	GRMZM2G083689_P01,GRMZM2G083689_P02	TRUE	TRUE	dAIFEFLLQcTEQLPHk	95%	n+304 (+304), Carbamidomethyl (+57), K+304 (+304)	36.11	25.34
6793	seq=translation; coord=5:23896374..23903276:-1; parent_transcript=GRMZM2G085747_T01; parent_gene=GRMZM2G085747	GRMZM2G085747_P01,GRMZM2G085747_P02, GRMZM2G085747_P03,GRMZM2G085747_P04, GRMZM2G085747_P05	TRUE	TRUE	iLGLGDLGVQGIGIPIGk	95%	n+304 (+304), K+304 (+304)	52.22	25.00
6794	seq=translation; coord=10:138858423..138859497:-1; parent_transcript=GRMZM2G091534_T01; parent_gene=GRMZM2G091534	GRMZM2G091534_P01,GRMZM2G391272_P01, GRMZM2G391286_P01,GRMZM2G406313_P01, GRMZM2G410338_P01	TRUE	TRUE	lGVcANVLGLlk	95%	n+304 (+304), Carbamidomethyl (+57), K+304 (+304)	34.23	25.00
6795	seq=translation; coord=8:13336541..13339982:-1; parent_transcript=GRMZM2G094273_T01; parent_gene=GRMZM2G094273	GRMZM2G094273_P01	TRUE	TRUE	aGVEMALIDAVANSIR	95%	n+304 (+304)	37.30	25.85
6796	seq=translation; coord=5:6525725..6529505:1; parent_transcript=GRMZM2G095185_T03; parent_gene=GRMZM2G095185	GRMZM2G095185_P02,GRMZM2G095185_P03	TRUE	TRUE	vMPTGWFLLLR	95%	n+304 (+304)	33.68	25.00
6797	seq=translation; coord=8:135085183..135090834:-1; parent_transcript=GRMZM2G098212_T01; parent_gene=GRMZM2G098212	GRMZM2G098212_P01	TRUE	TRUE	tDVcEFLIGELGLPVDPk	95%	n+304 (+304), Carbamidomethyl (+57), K+304 (+304)	31.31	25.31
6798	seq=translation; coord=6:159759627..159763263:1; parent_transcript=GRMZM2G101457_T01; parent_gene=GRMZM2G101457	GRMZM2G101457_P01	TRUE	TRUE	vFVLTLTGDGEHR	95%	n+304 (+304)	49.75	25.59
6799	seq=translation; coord=8:171709672..171712582:-1; parent_transcript=GRMZM2G106344_T01; parent_gene=GRMZM2G106344	GRMZM2G106344_P01,GRMZM2G106344_P02	TRUE	TRUE	gAGFEVVLVScDEDRPSFER	92%	n+304 (+304), Carbamidomethyl (+57)	26.74	25.00
6800	seq=translation; coord=10:148815875..148817265:1; parent_transcript=GRMZM2G106792_T01; parent_gene=GRMZM2G106792	GRMZM2G106792_P01	TRUE	TRUE	gTQLLQGDVTAAEFR	95%	n+304 (+304)	56.52	25.00
6801	seq=translation; coord=1:182898704..182903336:-1; parent_transcript=GRMZM2G107163_T01; parent_gene=GRMZM2G107163	GRMZM2G107163_P01,GRMZM2G131957_P01, GRMZM2G131957_P02	TRUE	TRUE	gYcQVDFYDPGAAAAck	95%	n+304 (+304), Carbamidomethyl (+57), Carbamidomethyl (+57), K+304 (+304)	46.93	25.00
6802	seq=translation; coord=1:264209651..264226078:1; parent_transcript=GRMZM2G109056_T01; parent_gene=GRMZM2G109056	GRMZM2G109056_P01	TRUE	TRUE	aIIEAVLPTLGTFVDDTPk	95%	n+304 (+304), K+304 (+304)	52.56	25.00
6803	seq=translation; coord=1:200075221..200076952:-1; parent_transcript=GRMZM2G117378_T01; parent_gene=GRMZM2G117378	GRMZM2G117378_P01,GRMZM2G349749_P01	TRUE	TRUE	gIIPGTILAFLEEk	95%	n+304 (+304), K+304 (+304)	55.63	25.00

6804	seq=translation; coord=7:85262878..85264551:1; parent_transcript=GRMZM2G117878_T01; parent_gene=GRMZM2G117878	GRMZM2G117878_P01	TRUE	TRUE	iGVLLEGVLER	95%	n+304 (+304)	40.68	25.00
6805	seq=translation; coord=1:59670870..59675001:-1; parent_transcript=GRMZM2G124576_T02; parent_gene=GRMZM2G124576	GRMZM2G124576_P02	TRUE	TRUE	eSEEAPAAEGDGEk	95%	n+304 (+304), K+304 (+304)	50.25	25.00
6806	seq=translation; coord=6:82963690..82967765:1; parent_transcript=GRMZM2G126361_T01; parent_gene=GRMZM2G126361	GRMZM2G126361_P01,GRMZM2G126361_P02, GRMZM2G126361_P03	TRUE	TRUE	lELQEVVDFLk	95%	n+304 (+304), K+304 (+304)	48.90	25.45
6807	seq=translation; coord=1:86168160..86176332:-1; parent_transcript=GRMZM2G127168_T01; parent_gene=GRMZM2G127168	GRMZM2G127168_P01,GRMZM2G127168_P02	TRUE	TRUE	gVGAPFHDVGAPFR	95%	n+304 (+304)	31.10	25.00
6808	seq=translation; coord=6:153309231..153314326:1; parent_transcript=GRMZM2G127297_T01; parent_gene=GRMZM2G127297	GRMZM2G127297_P01,GRMZM2G410782_P01	TRUE	TRUE	aLLLLDSWQEAFGGPGGk	95%	n+304 (+304), K+304 (+304)	53.69	25.01
6809	seq=translation; coord=2:144192738..144194495:1; parent_transcript=GRMZM2G127418_T01; parent_gene=GRMZM2G127418	GRMZM2G127418_P01	TRUE	TRUE	iRPVAGLMSVIEVYP	95%	n+304 (+304)	34.52	25.74
6810	seq=translation; coord=2:181110668..181120123:1; parent_transcript=GRMZM2G127717_T01; parent_gene=GRMZM2G127717	GRMZM2G127717_P01,GRMZM2G127717_P02	TRUE	TRUE	gFcVVcVAEGAGQDLLQk	95%	n+304 (+304), Carbamidomethyl (+57), Carbamidomethyl (+57), K+304 (+304)	34.92	25.21
6811	seq=translation; coord=5:203712290..203717409:1; parent_transcript=GRMZM2G128922_T01; parent_gene=GRMZM2G128922	GRMZM2G128922_P01,GRMZM2G128922_P03, GRMZM2G129146_P01,GRMZM2G129146_P03, GRMZM2G129146_P04,GRMZM2G129146_P05	TRUE	TRUE	vPEVTMAFAGGAALk	95%	n+304 (+304), K+304 (+304)	46.18	26.19
6812	seq=translation; coord=7:156715826..156717458:-1; parent_transcript=GRMZM2G129973_T01; parent_gene=GRMZM2G129973	GRMZM2G129973_P01	TRUE	TRUE	aVTSFAALVAALAk	95%	n+304 (+304), K+304 (+304)	33.91	25.00
6813	seq=translation; coord=10:17930259..17933520:1; parent_transcript=GRMZM2G136563_T01; parent_gene=GRMZM2G136563	GRMZM2G136563_P01	TRUE	TRUE	eLDIPGEIYER	95%	n+304 (+304)	35.74	25.00
6814	seq=translation; coord=8:139450959..139452582:-1; parent_transcript=GRMZM2G142705_T01; parent_gene=GRMZM2G142705	GRMZM2G142705_P01	TRUE	TRUE	sLADALHLR	95%	n+304 (+304)	67.20	27.19
6815	seq=translation; coord=8:16755280..16757481:-1; parent_transcript=GRMZM2G143258_T01; parent_gene=GRMZM2G143258	GRMZM2G143258_P01,GRMZM2G143258_P02	TRUE	TRUE	lVVPTLLFFGASGTR	95%	n+304 (+304)	32.28	25.33
6816	seq=translation; coord=1:219379298..219385327:-1; parent_transcript=GRMZM2G146286_T01; parent_gene=GRMZM2G146286	GRMZM2G146286_P01,GRMZM2G146286_P02, GRMZM2G146286_P03,GRMZM2G146286_P04, GRMZM2G147712_P01	TRUE	TRUE	eYIEEVYAAYEQHk	95%	n+304 (+304), K+304 (+304)	33.77	25.00
6817	seq=translation; coord=8:20971995..20976240:-1; parent_transcript=GRMZM2G147882_T01; parent_gene=GRMZM2G147882	GRMZM2G147882_P01,GRMZM2G147882_P02, GRMZM2G147882_P03,GRMZM2G147882_P04	TRUE	TRUE	gGFIVNLGDMLER	95%	n+304 (+304)	42.30	25.40
6818	seq=translation; coord=1:205531096..205533366:-1; parent_transcript=GRMZM2G149414_T01; parent_gene=GRMZM2G149414	GRMZM2G149414_P01	TRUE	TRUE	eEAEGAPAVAAHH	95%	n+304 (+304)	57.96	25.00
6819	seq=translation; coord=7:164564301..164565776:1; parent_transcript=GRMZM2G152179_T01; parent_gene=GRMZM2G152179	GRMZM2G152179_P01	TRUE	TRUE	dMVVSLWHFLR	95%	n+304 (+304)	43.42	25.09
6820	seq=translation; coord=7:156569292..156577217:1; parent_transcript=GRMZM2G153162_T01; parent_gene=GRMZM2G153162	GRMZM2G153162_P01	TRUE	TRUE	dGIAADILGAVLDSIk	95%	n+304 (+304), K+304 (+304)	33.54	25.00

6821	seq=translation; coord=1:278907896..278910140:1; parent_transcript=GRMZM2G154828_T01; parent_gene=GRMZM2G154828	GRMZM2G154828_P01	TRUE	TRUE	sMLAVLLALQk	95%	n+304 (+304), K+304 (+304)	30.44	25.00
6822	seq=translation; coord=5:73229280..73231295:1; parent_transcript=GRMZM2G157026_T01; parent_gene=GRMZM2G157026	GRMZM2G157026_P01,GRMZM5G838961_P01	TRUE	TRUE	dILHEFTQEFR	95%	n+304 (+304)	32.13	25.00
6823	seq=translation; coord=1:14804662..14809417:1; parent_transcript=GRMZM2G162725_T01; parent_gene=GRMZM2G162725	GRMZM2G162725_P01	TRUE	TRUE	aLAADDLAAAATAVHEFLAIDAR	95%	n+304 (+304)	69.31	26.19
6824	seq=translation; coord=7:147155879..147158705:-1; parent_transcript=GRMZM2G168588_T01; parent_gene=GRMZM2G168588	GRMZM2G168588_P01	TRUE	TRUE	sLLAGQVLDVPLR	95%	n+304 (+304)	44.98	25.00
6825	seq=translation; coord=6:2447171..2452276:-1; parent_transcript=GRMZM2G178192_T01; parent_gene=GRMZM2G178192	GRMZM2G178192_P01,GRMZM2G178192_P02, GRMZM2G178192_P03	TRUE	TRUE	iDELLGSILEk	95%	n+304 (+304), K+304 (+304)	35.20	25.01
6826	seq=translation; coord=3:175775929..175780870:-1; parent_transcript=GRMZM2G178244_T01; parent_gene=GRMZM2G178244	GRMZM2G178244_P01	TRUE	TRUE	sATADGTQQWAFLTDALAR	95%	n+304 (+304)	55.02	25.00
6827	seq=translation; coord=2:211946552..211950130:1; parent_transcript=GRMZM2G314955_T03; parent_gene=GRMZM2G314955	GRMZM2G314955_P03	TRUE	TRUE	sVTPTASQQNPAER	95%	n+304 (+304)	29.73	25.00
6828	seq=translation; coord=1:205241339..205247763:-1; parent_transcript=GRMZM2G319357_T01; parent_gene=GRMZM2G319357	GRMZM2G319357_P01	TRUE	TRUE	dFDLILAMDR	95%	n+304 (+304)	31.60	25.00
6829	seq=translation; coord=4:239129384..239130094:-1; parent_transcript=GRMZM2G324162_T01; parent_gene=GRMZM2G324162	GRMZM2G324162_P01	TRUE	TRUE	lIWAHIk	92%	n+304 (+304), K+304 (+304)	26.35	25.00
6830	seq=translation; coord=2:229081805..229082275:1; parent_transcript=GRMZM2G371620_T01; parent_gene=GRMZM2G371620	GRMZM2G371620_P01,GRMZM5G816772_P01	TRUE	TRUE	iTLISDFGEISGSR	95%	n+304 (+304)	42.92	25.71
6831	seq=translation; coord=10:28388505..28394470:-1; parent_transcript=GRMZM2G383088_T01; parent_gene=GRMZM2G383088	GRMZM2G383088_P01	TRUE	TRUE	aGGIFLPLVk	95%	n+304 (+304), K+304 (+304)	31.80	25.00
6832	seq=translation; coord=8:133440146..133441157:1; parent_transcript=GRMZM2G386228_T01; parent_gene=GRMZM2G386228	GRMZM2G386228_P01	TRUE	TRUE	gTAAAAAGMR	95%	n+304 (+304)	33.77	25.17
6833	seq=translation; coord=7:143820122..143821948:-1; parent_transcript=GRMZM2G401664_T01; parent_gene=GRMZM2G401664	GRMZM2G401664_P01	TRUE	TRUE	lFIAEAFAk	95%	n+304 (+304), K+304 (+304)	34.97	25.00
6834	seq=translation; coord=4:7260920..7265403:1; parent_transcript=GRMZM2G403636_T01; parent_gene=GRMZM2G403636	GRMZM2G403636_P01	TRUE	TRUE	tAAFcFPIISGIMk	91%	n+304 (+304), Carbamidomethyl (+57), K+304 (+304)	26.36	25.48
6835	seq=translation; coord=4:233519831..233542576:1; parent_transcript=GRMZM2G417658_T01; parent_gene=GRMZM2G417658	GRMZM2G417658_P01,GRMZM2G417658_P02	TRUE	TRUE	lIFPEGTLAQTLAQLk	95%	n+304 (+304), K+304 (+304)	48.90	25.00
6836	seq=translation; coord=3:181276458..181277675:1; parent_transcript=GRMZM2G429982_T01; parent_gene=GRMZM2G429982	GRMZM2G429982_P01	TRUE	TRUE	tGcAGAGAQLHcATGDcGGR	95%	n+304 (+304), Carbamidomethyl (+57), Carbamidomethyl (+57), Carbamidomethyl (+57)	70.48	25.00
6837	seq=translation; coord=4:191750819..191753856:1; parent_transcript=GRMZM2G441541_T01; parent_gene=GRMZM2G441541	GRMZM2G441541_P01,GRMZM2G441541_P02, GRMZM2G441541_P03,GRMZM2G441541_P04	TRUE	TRUE	ePEFAGELFDALSR	95%	n+304 (+304)	35.36	25.00

6838	seq=translation; coord=7:104615801..104620303:1; parent_transcript=GRMZM2G472376_T02; parent_gene=GRMZM2G472376	GRMZM2G472376_P02	TRUE	TRUE	vSSIEIER	95%	n+304 (+304)	32.10	25.00
6839	seq=translation; coord=7:138591141..138594806:1; parent_transcript=GRMZM5G800734_T02; parent_gene=GRMZM5G800734	GRMZM5G800734_P02	TRUE	TRUE	yGAAVTLQNLDGk	95%	n+304 (+304), K+304 (+304)	32.40	26.54
6840	seq=translation; coord=10:88191606..88197105:1; parent_transcript=GRMZM5G803275_T01; parent_gene=GRMZM5G803275	GRMZM5G803275_P01,GRMZM5G817255_P03	TRUE	TRUE	mSVDWAFSSGPAk	95%	n+304 (+304), K+304 (+304)	41.79	25.38
6841	seq=translation; coord=2:193648937..193649473:-1; parent_transcript=GRMZM5G808017_T01; parent_gene=GRMZM5G808017	GRMZM5G808017_P01	TRUE	TRUE	lLEDLDALAGTIAVk	95%	n+304 (+304), K+304 (+304)	33.60	25.00
6842	seq=translation; coord=2:144982955..144985696:1; parent_transcript=GRMZM2G145651_T01; parent_gene=GRMZM2G145651	GRMZM2G145651_P01	TRUE	TRUE	eVWcVAk	94%	n+304 (+304), Carbamidomethyl (+57), K+304 (+304)	32.27	25.91
6843	seq=translation; coord=5:2705885..2708336:1; parent_transcript=GRMZM2G107076_T02; parent_gene=GRMZM2G107076	GRMZM2G107076_P02	TRUE	TRUE	aGVLDPDDRAETDHLLALAAGAGR	94%	n+304 (+304)	26.02	25.00
6844	seq=translation; coord=8:118889246..118891848:-1; parent_transcript=GRMZM5G827266_T02; parent_gene=GRMZM5G827266	GRMZM5G827266_P02	TRUE	TRUE	dVQEAcVYDGYVLPk	94%	n+304 (+304), Carbamidomethyl (+57), K+304 (+304)	29.16	25.85
6845	seq=translation; coord=5:64335786..64339361:-1; parent_transcript=AC190909.3_FGT004; parent_gene=AC190909.3_FG004	AC190909.3_FGP004,GRMZM2G430680_P01,GRMZM2G430680_P02	TRUE	TRUE	fVELLELLQk	95%	n+304 (+304), K+304 (+304)	30.71	25.00
6846	seq=translation; coord=8:139014642..139020725:-1; parent_transcript=AC196426.3_FGT007; parent_gene=AC196426.3_FG007	AC196426.3_FGP007,GRMZM5G873287_P01	TRUE	TRUE	fVPTVMNILk	95%	n+304 (+304), K+304 (+304)	35.33	25.00
6847	seq=translation; coord=7:170099729..170103818:1; parent_transcript=AC198481.3_FGT004; parent_gene=AC198481.3_FG004	AC198481.3_FGP004	TRUE	TRUE	lLAVVNALLHR	95%	n+304 (+304)	55.28	25.00
6848	seq=translation; coord=2:194391631..194395595:-1; parent_transcript=AC199782.5_FGT001; parent_gene=AC199782.5_FG001	AC199782.5_FGP001	TRUE	TRUE	sALQEAETELDR	95%	n+304 (+304)	39.00	25.00
6849	seq=translation; coord=7:140686655..140691401:1; parent_transcript=AC200057.4_FGT007; parent_gene=AC200057.4_FG007	AC200057.4_FGP007,GRMZM2G112483_P01,GRMZM2G112483_P02,GRMZM2G180847_P01	TRUE	TRUE	lAELALVDPk	92%	n+304 (+304), K+304 (+304)	25.98	25.00
6850	seq=translation; coord=10:83447249..83454536:1; parent_transcript=GRMZM2G003897_T01; parent_gene=GRMZM2G003897	GRMZM2G003897_P01	TRUE	TRUE	eAFAEFGEVLHAR	95%	n+304 (+304)	74.55	25.02
6851	seq=translation; coord=2:216610669..216612249:1; parent_transcript=GRMZM2G010991_T01; parent_gene=GRMZM2G010991	GRMZM2G010991_P01	TRUE	TRUE	iMEIASLEk	95%	n+304 (+304), K+304 (+304)	33.62	25.00
6852	seq=translation; coord=6:151770259..151778529:1; parent_transcript=GRMZM2G012052_T01; parent_gene=GRMZM2G012052	GRMZM2G012052_P01,GRMZM2G085600_P01, GRMZM2G085600_P02,GRMZM2G085600_P03	TRUE	TRUE	fTSEMWHPNVYPDGR	95%	n+304 (+304)	35.30	25.00
6853	seq=translation; coord=9:11216956..11220850:1; parent_transcript=GRMZM2G012863_T01; parent_gene=GRMZM2G012863	GRMZM2G012863_P01,GRMZM2G012863_P02	TRUE	TRUE	lLAGESGIGPIDR	95%	n+304 (+304)	45.91	26.18
6854	seq=translation; coord=6:156927300..156931413:-1; parent_transcript=GRMZM2G015100_T01; parent_gene=GRMZM2G015100	GRMZM2G015100_P01	TRUE	TRUE	mPLGIVDELFGPVk	95%	n+304 (+304), K+304 (+304)	50.90	25.13

6855	seq=translation; coord=6:40207604..40209489:1; parent_transcript=GRMZM2G017966_T01; parent_gene=GRMZM2G017966	GRMZM2G017966_P01,GRMZM2G017966_P02, GRMZM2G073535_P01,GRMZM2G073535_P02, GRMZM2G073535_P03,GRMZM2G073535_P04, GRMZM2G073535_P05,GRMZM2G113414_P01, GRMZM2G113414_P02,GRMZM5G835323_P01, GRMZM5G835323_P02	TRUE	TRUE	sLTTVQGLk	95%	n+304 (+304), K+304 (+304)	33.82	25.00
6856	seq=translation; coord=8:144958450..144963956:-1; parent_transcript=GRMZM2G024738_T04; parent_gene=GRMZM2G024738	GRMZM2G024738_P04,GRMZM2G025031_P01, GRMZM2G025031_P02,GRMZM2G377609_P03	TRUE	TRUE	tPEEAVAHFFDVTR	95%	n+304 (+304)	35.04	25.00
6857	seq=translation; coord=3:228543979..228551345:1; parent_transcript=GRMZM2G027867_T01; parent_gene=GRMZM2G027867	GRMZM2G027867_P01,GRMZM2G027867_P02	TRUE	TRUE	lFLLPDVLcLAk	95%	n+304 (+304), Carbamidomethyl (+57), K+304 (+304)	35.11	25.00
6858	seq=translation; coord=8:6394352..6397914:-1; parent_transcript=GRMZM2G029845_T01; parent_gene=GRMZM2G029845	GRMZM2G029845_P01,GRMZM2G029845_P02, GRMZM2G134149_P01,GRMZM2G134149_P02	TRUE	TRUE	sPELEVLGLTTTFGNVHTALATR	95%	n+304 (+304)	69.68	25.85
6859	seq=translation; coord=1:97673409..97679050:1; parent_transcript=GRMZM2G033526_T01; parent_gene=GRMZM2G033526	GRMZM2G033526_P01,GRMZM2G033526_P02, GRMZM2G144730_P01,GRMZM2G152686_P01	TRUE	TRUE	aEVSDIAIAVR	95%	n+304 (+304)	36.04	26.33
6860	seq=translation; coord=1:266950013..266955592:-1; parent_transcript=GRMZM2G041770_T01; parent_gene=GRMZM2G041770	GRMZM2G041770_P01	TRUE	TRUE	gFDLLQEILQHLR	95%	n+304 (+304)	30.35	25.50
6861	seq=translation; coord=7:140706875..140710245:1; parent_transcript=GRMZM2G043600_T01; parent_gene=GRMZM2G043600	GRMZM2G043600_P01	TRUE	TRUE	dASALEELFR	95%	n+304 (+304)	54.79	25.00
6862	seq=translation; coord=3:133067971..133077051:1; parent_transcript=GRMZM2G047362_T01; parent_gene=GRMZM2G047362	GRMZM2G047362_P01	TRUE	TRUE	nILDHLLLLGVESQWAPVALR	95%	n+304 (+304)	33.86	25.00
6863	seq=translation; coord=2:173282474..173286392:-1; parent_transcript=GRMZM2G047404_T01; parent_gene=GRMZM2G047404	GRMZM2G047404_P01,GRMZM2G103047_P01	TRUE	TRUE	vSPAVLQSAGLNLk	95%	n+304 (+304), K+304 (+304)	41.47	25.00
6864	seq=translation; coord=4:53440616..53446854:-1; parent_transcript=GRMZM2G049525_T01; parent_gene=GRMZM2G049525	GRMZM2G049525_P01,GRMZM2G049525_P02, GRMZM2G049525_P03	TRUE	TRUE	lSFVPGAIEWVHR	95%	n+304 (+304)	55.21	25.68
6865	seq=translation; coord=1:296002128..296008705:-1; parent_transcript=GRMZM2G053803_T01; parent_gene=GRMZM2G053803	GRMZM2G053803_P01,GRMZM2G053803_P02, GRMZM2G326195_P01	TRUE	TRUE	lEVDVAELR	92%	n+304 (+304)	30.02	27.04
6866	seq=translation; coord=5:76621430..76624952:-1; parent_transcript=GRMZM2G054706_T01; parent_gene=GRMZM2G054706	GRMZM2G054706_P01	TRUE	TRUE	fLHEVAFk	95%	n+304 (+304), K+304 (+304)	30.66	25.68
6867	seq=translation; coord=1:266913672..266916510:-1; parent_transcript=GRMZM2G057394_T01; parent_gene=GRMZM2G057394	GRMZM2G057394_P01,GRMZM2G057394_P02	TRUE	TRUE	tALDLLQSLPR	95%	n+304 (+304)	44.49	25.54
6868	seq=translation; coord=6:160625203..160629814:1; parent_transcript=GRMZM2G060817_T01; parent_gene=GRMZM2G060817	GRMZM2G060817_P01,GRMZM2G060817_P02	TRUE	TRUE	iLGELEGLDTMLHLAk	95%	n+304 (+304), K+304 (+304)	48.29	25.00
6869	seq=translation; coord=1:147082260..147087160:1; parent_transcript=GRMZM2G065205_T01; parent_gene=GRMZM2G065205	GRMZM2G065205_P01,GRMZM2G112074_P01	TRUE	TRUE	aLLLLLVGAPHR	95%	n+304 (+304)	45.80	25.00
6870	seq=translation; coord=3:169617043..169620923:-1; parent_transcript=GRMZM2G070562_T01; parent_gene=GRMZM2G070562	GRMZM2G070562_P01	TRUE	TRUE	iNIIAALVAAGR	95%	n+304 (+304)	38.26	25.00

6871	seq=translation; coord=9:147166752..147170885:-1; parent_transcript=GRMZM2G073498_T01; parent_gene=GRMZM2G073498	GRMZM2G073498_P01,GRMZM2G073498_P02, GRMZM2G073498_P03,GRMZM2G130987_P01, GRMZM2G130987_P02,GRMZM2G130987_P04, GRMZM2G307252_P01,GRMZM2G307252_P02	TRUE	TRUE	aTELGFFGF	95%	n+304 (+304)	45.55	25.00
6872	seq=translation; coord=10:148605305..148610112:-1; parent_transcript=GRMZM2G074787_T01; parent_gene=GRMZM2G074787	GRMZM2G074787_P01,GRMZM2G074787_P02	TRUE	TRUE	lLAVPLFELYDNIQR	95%	n+304 (+304)	30.95	26.30
6873	seq=translation; coord=3:226476662..226482081:1; parent_transcript=GRMZM2G078826_T01; parent_gene=GRMZM2G078826	GRMZM2G078826_P01	TRUE	TRUE	gRDDDEEGIIDEDGEIGEDDPR	95%	n+304 (+304)	74.83	25.00
6874	seq=translation; coord=6:137142553..137144384:-1; parent_transcript=GRMZM2G079440_T01; parent_gene=GRMZM2G079440	GRMZM2G079440_P01	TRUE	TRUE	tGGILHR	95%	n+304 (+304)	36.82	25.97
6875	seq=translation; coord=9:143329347..143333500:1; parent_transcript=GRMZM2G081175_T01; parent_gene=GRMZM2G081175	GRMZM2G081175_P01	TRUE	TRUE	lMPGEVILASLDGFGk	95%	n+304 (+304), K+304 (+304)	36.73	25.15
6876	seq=translation; coord=8:2110920..2115595:1; parent_transcript=GRMZM2G082214_T01; parent_gene=GRMZM2G082214	GRMZM2G082214_P01	TRUE	TRUE	iREEAGELIQTLLENEDQSR	95%	n+304 (+304)	33.64	25.00
6877	seq=translation; coord=3:135445422..135448935:-1; parent_transcript=GRMZM2G084647_T01; parent_gene=GRMZM2G084647	GRMZM2G084647_P01,GRMZM2G084647_P02, GRMZM2G084647_P03	TRUE	TRUE	rkPEAAEAVAAIMALAAVIR	95%	n+304 (+304), K+304 (+304)	37.45	25.00
6878	seq=translation; coord=1:51291271..51292598:1; parent_transcript=GRMZM2G084783_T02; parent_gene=GRMZM2G084783	GRMZM2G084783_P02	TRUE	TRUE	qVFDGDQPAAETTAEAVNR	95%	n+304 (+304)	36.98	25.00
6879	seq=translation; coord=9:149894129..149896657:-1; parent_transcript=GRMZM2G091362_T01; parent_gene=GRMZM2G091362	GRMZM2G091362_P01	TRUE	TRUE	rIDGSSDDFSFR	95%	n+304 (+304)	63.14	25.00
6880	seq=translation; coord=3:53980991..53987563:1; parent_transcript=GRMZM2G092661_T01; parent_gene=GRMZM2G092661	GRMZM2G092661_P01,GRMZM2G092661_P02, GRMZM2G092661_P03	TRUE	TRUE	wVPLSLEFGVPLFSPk	95%	n+304 (+304), K+304 (+304)	33.00	25.00
6881	seq=translation; coord=3:220129576..220134164:1; parent_transcript=GRMZM2G095534_T01; parent_gene=GRMZM2G095534	GRMZM2G095534_P01,GRMZM2G095534_P02, GRMZM2G095534_P03,GRMZM2G095534_P04, GRMZM2G095534_P05,GRMZM2G095534_P06	TRUE	TRUE	lSVETTVQSAVTEVAAMVGENVk	95%	n+304 (+304), K+304 (+304)	51.13	25.05
6882	seq=translation; coord=5:212233142..212237929:1; parent_transcript=GRMZM2G097977_T01; parent_gene=GRMZM2G097977	GRMZM2G097977_P01	TRUE	TRUE	aLLLDFAASLMPGLAAk	95%	n+304 (+304), K+304 (+304)	47.27	25.00
6883	seq=translation; coord=8:135062540..135065340:1; parent_transcript=GRMZM2G098153_T01; parent_gene=GRMZM2G098153	GRMZM2G098153_P01	TRUE	TRUE	vIGTLLGSLLPDGTVHVR	95%	n+304 (+304)	29.32	25.00
6884	seq=translation; coord=7:175302491..175305743:-1; parent_transcript=GRMZM2G102167_T01; parent_gene=GRMZM2G102167	GRMZM2G102167_P01,GRMZM2G102167_P04, GRMZM2G102167_P05	TRUE	TRUE	hVLLPVLDR	95%	n+304 (+304)	50.62	25.00
6885	seq=translation; coord=2:165262816..165266884:-1; parent_transcript=GRMZM2G103345_T01; parent_gene=GRMZM2G103345	GRMZM2G103345_P01,GRMZM2G103345_P02	TRUE	TRUE	iGQGAEITLER	95%	n+304 (+304)	47.74	26.46
6886	seq=translation; coord=5:28674682..28681161:1; parent_transcript=GRMZM2G104254_T01; parent_gene=GRMZM2G104254	GRMZM2G104254_P01,GRMZM2G104254_P02	TRUE	TRUE	iTMDMASQIFEILR	95%	n+304 (+304)	46.48	25.73
6887	seq=translation; coord=2:172843794..172863857:-1; parent_transcript=GRMZM2G104546_T02; parent_gene=GRMZM2G104546	GRMZM2G104546_P02,GRMZM2G136712_P01, GRMZM2G437977_P01	TRUE	TRUE	dDLSGTDVAR	95%	n+304 (+304)	33.18	25.00

6888	seq=translation; coord=1:73634614..73642065:-1; parent_transcript=GRMZM2G104983_T01; parent_gene=GRMZM2G104983	GRMZM2G104983_P01	TRUE	TRUE	lGVMSLLAVFk	95%	n+304 (+304), K+304 (+304)	33.18	25.00
6889	seq=translation; coord=6:56102468..56105100:-1; parent_transcript=GRMZM2G107073_T01; parent_gene=GRMZM2G107073	GRMZM2G107073_P01,GRMZM2G107073_P02	TRUE	TRUE	lQGIQGAPFR	89%	n+304 (+304)	26.17	25.00
6890	seq=translation; coord=7:7453972..7459323:1; parent_transcript=GRMZM2G107089_T01; parent_gene=GRMZM2G107089	GRMZM2G107089_P01,GRMZM2G107089_P02	TRUE	TRUE	iAPIQVIIVPIWk	95%	n+304 (+304), K+304 (+304)	30.25	25.00
6891	seq=translation; coord=5:12580030..12585678:-1; parent_transcript=GRMZM2G111475_T01; parent_gene=GRMZM2G111475	GRMZM2G111475_P01	TRUE	TRUE	mELSSLTQTNISLPFITATADGPk	95%	n+304 (+304), K+304 (+304)	27.97	25.00
6892	seq=translation; coord=6:121537212..121538751:1; parent_transcript=GRMZM2G117057_T01; parent_gene=GRMZM2G117057	GRMZM2G117057_P01,GRMZM2G117057_P02	TRUE	TRUE	hDLGLPVAAMFFNAQk	95%	n+304 (+304), K+304 (+304)	33.14	25.48
6893	seq=translation; coord=10:12600584..12608266:-1; parent_transcript=GRMZM2G118714_T01; parent_gene=GRMZM2G118714	GRMZM2G118714_P01	TRUE	TRUE	aNQLLGEIAEIQESLMHVk	95%	n+304 (+304), K+304 (+304)	54.31	25.00
6894	seq=translation; coord=5:213055055..213058997:1; parent_transcript=GRMZM2G122231_T01; parent_gene=GRMZM2G122231	GRMZM2G122231_P01,GRMZM2G312521_P01, GRMZM2G430607_P01	TRUE	TRUE	fLAMEQLLVEHR	95%	n+304 (+304)	77.09	25.88
6895	seq=translation; coord=6:45182651..45183616:1; parent_transcript=GRMZM2G124617_T01; parent_gene=GRMZM2G124617	GRMZM2G124617_P01	TRUE	TRUE	aQQAAAGGTGTSTDNk	95%	n+304 (+304), K+304 (+304)	38.94	25.00
6896	seq=translation; coord=1:82508572..82509915:1; parent_transcript=GRMZM2G125266_T01; parent_gene=GRMZM2G125266	GRMZM2G125266_P01	TRUE	TRUE	sVGTAVVDLLAAQLAYETHLAATk	95%	n+304 (+304), K+304 (+304)	42.64	25.00
6897	seq=translation; coord=4:30469845..30474256:1; parent_transcript=GRMZM2G126253_T01; parent_gene=GRMZM2G126253	GRMZM2G126253_P01	TRUE	TRUE	lFDTLLELIHR	95%	n+304 (+304)	40.14	25.00
6898	seq=translation; coord=6:131454814..131463174:-1; parent_transcript=GRMZM2G126435_T01; parent_gene=GRMZM2G126435	GRMZM2G126435_P01,GRMZM2G126435_P02	TRUE	TRUE	fEEAISAGDDER	95%	n+304 (+304)	56.47	25.00
6899	seq=translation; coord=7:5515951..5519578:-1; parent_transcript=GRMZM2G129357_T01; parent_gene=GRMZM2G129357	GRMZM2G129357_P01	TRUE	TRUE	vLEFVQFLQSk	95%	n+304 (+304), K+304 (+304)	47.22	25.00
6900	seq=translation; coord=3:4635480..4641804:1; parent_transcript=GRMZM2G133121_T01; parent_gene=GRMZM2G133121	GRMZM2G133121_P01	TRUE	TRUE	gIPVATVAIGIAENAGLLAAR	95%	n+304 (+304)	52.47	25.00
6901	seq=translation; coord=3:190321122..190324751:-1; parent_transcript=GRMZM2G138800_T01; parent_gene=GRMZM2G138800	GRMZM2G138800_P01	TRUE	TRUE	iWQVPEILSEEVLGk	95%	n+304 (+304), K+304 (+304)	48.56	25.00
6902	seq=translation; coord=1:247626491..247632248:-1; parent_transcript=GRMZM2G140602_T01; parent_gene=GRMZM2G140602	GRMZM2G140602_P01	TRUE	TRUE	vIEASDVILEVLDAR	95%	n+304 (+304)	37.57	25.56
6903	seq=translation; coord=10:59506710..59518288:1; parent_transcript=GRMZM2G141399_T01; parent_gene=GRMZM2G141399	GRMZM2G141399_P01,GRMZM2G141399_P02	TRUE	TRUE	qDLSIVGISNEFQTk	95%	n+304 (+304), K+304 (+304)	46.17	26.82
6904	seq=translation; coord=10:111287744..111290073:1; parent_transcript=GRMZM2G149543_T01; parent_gene=GRMZM2G149543	GRMZM2G149543_P01	TRUE	TRUE	tALADLHDLLGR	95%	n+304 (+304)	45.11	25.55
6905	GRMZM2G163711_P01-R	GRMZM2G163711_P01-R	TRUE	TRUE	lGLHSLR	95%	n+304 (+304)	30.42	26.49

6906	seq=translation; coord=3:3308380..3311497:1; parent_transcript=GRMZM2G166597_T01; parent_gene=GRMZM2G166597	GRMZM2G166597_P01	TRUE	TRUE	dTRPTGAYLLDAALQGINAIVGAR	95%	n+304 (+304)	50.56	25.00
6907	seq=translation; coord=1:299754155..299755202:-1; parent_transcript=GRMZM2G167031_T04; parent_gene=GRMZM2G167031	GRMZM2G167031_P04	TRUE	TRUE	fFALQILESVIk	95%	n+304 (+304), K+304 (+304)	33.00	25.00
6908	seq=translation; coord=3:19939526..19944315:1; parent_transcript=GRMZM2G169152_T02; parent_gene=GRMZM2G169152	GRMZM2G169152_P02,GRMZM2G169152_P03	TRUE	TRUE	lGVLGALEIIDAAR	95%	n+304 (+304)	39.90	25.00
6909	seq=translation; coord=4:80533096..80538602:-1; parent_transcript=GRMZM2G171921_T01; parent_gene=GRMZM2G171921	GRMZM2G171921_P01,GRMZM2G171921_P02, GRMZM2G171921_P03,GRMZM2G171921_P04, GRMZM2G171921_P05,GRMZM2G171921_P06	TRUE	TRUE	eLFSEVGHLk	95%	n+304 (+304), K+304 (+304)	42.32	26.50
6910	seq=translation; coord=6:145123773..145126701:-1; parent_transcript=GRMZM2G179346_T01; parent_gene=GRMZM2G179346	GRMZM2G179346_P01	TRUE	TRUE	mFFHIVLER	95%	n+304 (+304)	37.99	25.00
6911	seq=translation; coord=4:36122702..36125196:1; parent_transcript=GRMZM2G179976_T01; parent_gene=GRMZM2G179976	GRMZM2G179976_P01	TRUE	TRUE	fAVAAPVAAADSSAAAAPk	95%	n+304 (+304), K+304 (+304)	60.29	25.66
6912	seq=translation; coord=8:160763133..160770225:1; parent_transcript=GRMZM2G312365_T01; parent_gene=GRMZM2G312365	GRMZM2G312365_P01	TRUE	TRUE	dSDLSPLGLLWSELEGRPk	95%	n+304 (+304), K+304 (+304)	57.15	25.38
6913	seq=translation; coord=9:45354730..45357776:1; parent_transcript=GRMZM2G314094_T01; parent_gene=GRMZM2G314094	GRMZM2G314094_P01	TRUE	TRUE	iIGFLGGTDGLFAQk	95%	n+304 (+304), K+304 (+304)	32.79	25.12
6914	seq=translation; coord=8:165181600..165183220:-1; parent_transcript=GRMZM2G316362_T01; parent_gene=GRMZM2G316362	GRMZM2G316362_P01	TRUE	TRUE	dYADIVEFLVk	95%	n+304 (+304), K+304 (+304)	39.26	25.50
6915	seq=translation; coord=3:49619034..49624780:-1; parent_transcript=GRMZM2G322186_T01; parent_gene=GRMZM2G322186	GRMZM2G322186_P01,GRMZM2G322186_P02	TRUE	TRUE	lFLAQPDAFYFHVLQQGLLR	95%	n+304 (+304)	65.46	25.34
6916	seq=translation; coord=7:172727421..172731206:1; parent_transcript=GRMZM2G322413_T02; parent_gene=GRMZM2G322413	GRMZM2G322413_P02	TRUE	TRUE	aPLGQNTVLLR	89%	n+304 (+304)	26.18	25.00
6917	seq=translation; coord=6:113233723..113238779:1; parent_transcript=GRMZM2G348551_T01; parent_gene=GRMZM2G348551	GRMZM2G348551_P01	TRUE	TRUE	tVEGGWGLHDIIR	95%	n+304 (+304)	86.40	25.66
6918	seq=translation; coord=1:81918413..81920944:-1; parent_transcript=GRMZM2G353553_T01; parent_gene=GRMZM2G353553	GRMZM2G353553_P01	TRUE	TRUE	vQILQTEATTLSAQLTLLQR	95%	n+304 (+304)	80.51	25.00
6919	seq=translation; coord=5:207060907..207063925:-1; parent_transcript=GRMZM2G380184_T01; parent_gene=GRMZM2G380184	GRMZM2G380184_P01	TRUE	TRUE	lVNTLLDLIk	95%	n+304 (+304), K+304 (+304)	29.60	25.00
6920	seq=translation; coord=1:179988453..179990792:-1; parent_transcript=GRMZM2G392791_T01; parent_gene=GRMZM2G392791	GRMZM2G392791_P01	TRUE	TRUE	gAADAPAVLLIHGFPELWLSWR	95%	n+304 (+304)	35.77	25.84
6921	seq=translation; coord=3:174367886..174371254:-1; parent_transcript=GRMZM2G400604_T01; parent_gene=GRMZM2G400604	GRMZM2G400604_P01	TRUE	TRUE	tMAITDQAMALR	95%	n+304 (+304)	40.65	25.00
6922	seq=translation; coord=7:20171657..20172327:1; parent_transcript=GRMZM2G404688_T01; parent_gene=GRMZM2G404688	GRMZM2G404688_P01	TRUE	TRUE	fAPALVAAAEcGLLTAHGR	95%	n+304 (+304), Carbamidomethyl (+57)	74.15	26.21

6923	seq=translation; coord=10:70632831..70633760:-1; parent_transcript=GRMZM2G448627_T01; parent_gene=GRMZM2G448627	GRMZM2G448627_P01	TRUE	TRUE	eAAANVGASANAGMQk	95%	n+304 (+304), K+304 (+304)	58.72	25.35
6924	seq=translation; coord=9:33840187..33858081:-1; parent_transcript=GRMZM2G457415_T01; parent_gene=GRMZM2G457415	GRMZM2G457415_P01,GRMZM2G457415_P02, GRMZM2G457415_P03	TRUE	TRUE	vQPDGLVAGSLDLLTMVLk	95%	n+304 (+304), K+304 (+304)	56.82	25.00
6925	seq=translation; coord=5:96354714..96362409:1; parent_transcript=GRMZM2G462613_T01; parent_gene=GRMZM2G462613	GRMZM2G462613_P01	TRUE	TRUE	eHLWPFIQEFVDGALSHIVR	95%	n+304 (+304)	28.26	25.00
6926	seq=translation; coord=5:189804974..189811226:-1; parent_transcript=GRMZM2G463291_T01; parent_gene=GRMZM2G463291	GRMZM2G463291_P01	TRUE	TRUE	gGIkEENIVVFMYDDIANSALNPR	95%	n+304 (+304), K+304 (+304)	56.39	25.00
6927	seq=translation; coord=2:167977405..167982718:-1; parent_transcript=GRMZM2G504906_T01; parent_gene=GRMZM2G504906	GRMZM2G504906_P01	TRUE	TRUE	qSNQLLDITGDILQQIVR	95%	n+304 (+304)	70.38	25.00
6928	seq=translation; coord=7:81127590..81139739:-1; parent_transcript=GRMZM2G542753_T01; parent_gene=GRMZM2G542753	GRMZM2G542753_P01	TRUE	TRUE	nESTIEENEIR	95%	n+304 (+304)	56.45	25.00
6929	seq=translation; coord=6:86255384..86258220:1; parent_transcript=GRMZM5G832166_T01; parent_gene=GRMZM5G832166	GRMZM5G832166_P01,GRMZM5G832166_P02	TRUE	TRUE	gEPSGSTFDPSEYEENDMDELALVk	95%	n+304 (+304), K+304 (+304)	32.37	25.00
6930	seq=translation; coord=7:10775147..10779088:1; parent_transcript=GRMZM5G841619_T01; parent_gene=GRMZM5G841619	GRMZM5G841619_P01	TRUE	TRUE	eAGVHGLVVPDVPLEETDVLR	95%	n+304 (+304)	46.53	25.15
6931	seq=translation; coord=3:146437030..146439921:1; parent_transcript=GRMZM5G843368_T01; parent_gene=GRMZM5G843368	GRMZM5G843368_P01	TRUE	TRUE	iEALPTFIIFk	95%	n+304 (+304), K+304 (+304)	30.53	25.00
6932	seq=translation; coord=6:33247120..33251009:1; parent_transcript=GRMZM5G886561_T02; parent_gene=GRMZM5G886561	GRMZM5G886561_P02,GRMZM5G886561_P03	TRUE	TRUE	iPHPDAALEESISEVWDQSLQR	95%	n+304 (+304)	63.15	25.00
6933	seq=translation; coord=2:173604553..173607325:-1; parent_transcript=GRMZM5G887303_T01; parent_gene=GRMZM5G887303	GRMZM5G887303_P01	TRUE	TRUE	aGWPVADTPDPTLR	95%	n+304 (+304)	60.42	25.00
6934	seq=translation; coord=5:4253373..4257134:1; parent_transcript=GRMZM2G007063_T01; parent_gene=GRMZM2G007063	GRMZM2G007063_P01,GRMZM2G007063_P04, GRMZM2G016150_P01	TRUE	TRUE	aAHLNELEAQVAQLR	95%	n+304 (+304)	28.87	25.76
6935	seq=translation; coord=2:1717982..1719190:-1; parent_transcript=GRMZM2G436199_T01; parent_gene=GRMZM2G436199	GRMZM2G436199_P01	TRUE	TRUE	vYGLPTLILFk	92%	n+304 (+304), K+304 (+304)	26.64	25.00
6936	seq=translation; coord=10:131593783..131595368:-1; parent_transcript=GRMZM2G034651_T01; parent_gene=GRMZM2G034651	GRMZM2G034651_P01,GRMZM2G034709_P01, GRMZM2G041724_P01,GRMZM2G041724_P02, GRMZM2G046916_P01,GRMZM2G053622_P01, GRMZM2G073041_P01	TRUE	TRUE	rIDELDLPR	94%	n+304 (+304)	28.85	25.84
6937	seq=translation; coord=1:76339507..76340561:1; parent_transcript=GRMZM2G174708_T01; parent_gene=GRMZM2G174708	GRMZM2G174708_P01	TRUE	TRUE	aLPDLTFLR	94%	n+304 (+304)	30.27	25.00
6938	seq=translation; coord=5:36664128..36680454:1; parent_transcript=GRMZM2G051174_T01; parent_gene=GRMZM2G051174	GRMZM2G051174_P01	TRUE	TRUE	aVGEQLIAANPIELAVGNIVR	93%	n+304 (+304)	27.37	25.00
6939	seq=translation; coord=6:69621822..69626211:1; parent_transcript=GRMZM2G168299_T01; parent_gene=GRMZM2G168299	GRMZM2G168299_P01,GRMZM2G168299_P02	TRUE	TRUE	aVAWDMLk	94%	n+304 (+304), K+304 (+304)	28.77	26.45

6940	seq=translation; coord=1:264497788..264511128:1; parent_transcript=GRMZM2G033619_T01; parent_gene=GRMZM2G033619	GRMZM2G033619_P01,GRMZM2G033619_P02	TRUE	TRUE	nLEQVVGMVLNSFQDPHPR	93%	n+304 (+304)	27.29	25.00
6941	seq=translation; coord=8:155393198..155396041:-1; parent_transcript=GRMZM2G042034_T01; parent_gene=GRMZM2G042034	GRMZM2G042034_P01,GRMZM2G042034_P03, GRMZM2G069061_P01,GRMZM5G878308_P01, GRMZM5G878308_P03	TRUE	TRUE	vDDLMNWAR	92%	n+304 (+304)	28.64	25.00
6942	seq=translation; coord=8:167085302..167087579:1; parent_transcript=GRMZM2G162329_T01; parent_gene=GRMZM2G162329	GRMZM2G162329_P01	TRUE	TRUE	nVEEIIR	93%	n+304 (+304)	30.84	26.51
6943	seq=translation; coord=5:68395460..68398901:-1; parent_transcript=GRMZM2G074957_T01; parent_gene=GRMZM2G074957	GRMZM2G074957_P01	TRUE	TRUE	vEDAIEILEHVLk	93%	n+304 (+304), K+304 (+304)	27.03	25.00
6944	seq=translation; coord=5:209670201..209672210:1; parent_transcript=GRMZM2G072861_T01; parent_gene=GRMZM2G072861	GRMZM2G072861_P01	TRUE	TRUE	gVYSQPDNIMGR	92%	n+304 (+304)	29.22	25.00
6945	seq=translation; coord=4:48543090..48545401:-1; parent_transcript=GRMZM2G078283_T01; parent_gene=GRMZM2G078283	GRMZM2G078283_P01,GRMZM2G078283_P02	TRUE	TRUE	gLEVLAEFEk	90%	n+304 (+304), K+304 (+304)	25.74	25.42
6946	seq=translation; coord=6:140907550..140916545:1; parent_transcript=GRMZM2G141760_T01; parent_gene=GRMZM2G141760	GRMZM2G141760_P01,GRMZM2G141760_P02	TRUE	TRUE	lAEHPAIGPSDELR	92%	n+304 (+304)	27.08	25.74
6947	seq=translation; coord=2:156327934..156329194:-1; parent_transcript=GRMZM2G164440_T01; parent_gene=GRMZM2G164440	GRMZM2G164440_P01,GRMZM2G164440_P02	TRUE	TRUE	tTDPPAAPSQAccDALAGADLk	91%	n+304 (+304), Carbamidomethyl (+57), Carbamidomethyl (+57), K+304 (+304)	26.30	25.00
6948	seq=translation; coord=3:4344526..4345251:-1; parent_transcript=GRMZM2G346457_T01; parent_gene=GRMZM2G346457	GRMZM2G346457_P01	TRUE	TRUE	aLLADLLALR	91%	n+304 (+304)	29.57	26.35
6949	seq=translation; coord=8:110686795..110688334:1; parent_transcript=GRMZM2G150134_T01; parent_gene=GRMZM2G150134	GRMZM2G150134_P01	TRUE	TRUE	sLAPIFAVDITSPAALLR	91%	n+304 (+304)	26.05	25.00
6950	seq=translation; coord=7:138459627..138464600:1; parent_transcript=GRMZM2G166897_T01; parent_gene=GRMZM2G166897	GRMZM2G166897_P01,GRMZM2G166897_P02	TRUE	TRUE	lSADELLER	91%	n+304 (+304)	28.94	26.22
6951	seq=translation; coord=2:206365031..206369304:1; parent_transcript=GRMZM2G030713_T01; parent_gene=GRMZM2G030713	GRMZM2G030713_P01	TRUE	TRUE	eAELHALYLLR	91%	n+304 (+304)	26.63	25.74
6952	seq=translation; coord=4:1487016..1490811:1; parent_transcript=GRMZM2G107239_T01; parent_gene=GRMZM2G107239	GRMZM2G107239_P01,GRMZM2G107239_P02	TRUE	TRUE	iVSLAFDVISR	92%	n+304 (+304)	29.01	26.28
6953	seq=translation; coord=10:99471562..99472802:1; parent_transcript=GRMZM2G074585_T02; parent_gene=GRMZM2G074585	GRMZM2G074585_P02	TRUE	TRUE	iScDYQADPR	91%	n+304 (+304), Carbamidomethyl (+57)	27.78	25.00
6954	seq=translation; coord=1:157064978..157068691:1; parent_transcript=GRMZM2G315848_T01; parent_gene=GRMZM2G315848	GRMZM2G315848_P01,GRMZM2G315848_P02, GRMZM2G315848_P03	TRUE	TRUE	aSPLVLGLk	90%	n+304 (+304), K+304 (+304)	25.69	25.00
6955	seq=translation; coord=4:86282879..86284714:-1; parent_transcript=GRMZM2G089698_T01; parent_gene=GRMZM2G089698	GRMZM2G089698_P01	TRUE	TRUE	aALGAALVPFYPLAGR	90%	n+304 (+304)	25.67	25.00
6956	seq=translation; coord=8:89528703..89529666:1; parent_transcript=GRMZM2G000777_T01; parent_gene=GRMZM2G000777	GRMZM2G000777_P01	TRUE	TRUE	dAANEALAAFDALLAR	90%	n+304 (+304)	25.99	25.55
6957	GRMZM2G159013_P01-R	GRMZM2G159013_P01-R	TRUE	TRUE	lEILGLk	91%	n+304 (+304), K+304 (+304)	25.42	25.00

6958	seq=translation; coord=8:147431766..147436951:1; parent_transcript=GRMZM2G000177_T01; parent_gene=GRMZM2G000177	GRMZM2G000177_P01	TRUE	TRUE	vIFLDLVTMR	89%	n+304 (+304)	28.79	27.16
6959	seq=translation; coord=1:204683833..204695252:-1; parent_transcript=GRMZM2G058402_T01; parent_gene=GRMZM2G058402	GRMZM2G058402_P01	TRUE	TRUE	vLSEEER	88%	n+304 (+304)	26.84	25.56
6960	GRMZM2G180082_P01-R	GRMZM2G180082_P01-R	TRUE	TRUE	lQIDHAk	87%	n+304 (+304), K+304 (+304)	25.79	25.00
6961	seq=translation; coord=5:203313494..203315969:-1; parent_transcript=GRMZM2G158568_T01; parent_gene=GRMZM2G158568	GRMZM2G158568_P01	TRUE	TRUE	aMGTTDVR	86%	n+304 (+304)	25.26	25.00
6962	seq=translation; coord=8:69589821..69590301:1; parent_transcript=GRMZM2G025240_T01; parent_gene=GRMZM2G025240	GRMZM2G025240_P01	TRUE	TRUE	gGQYTTFMR	86%	n+304 (+304)	25.12	25.00
6963	seq=translation; coord=4:204339354..204343743:1; parent_transcript=GRMZM2G117064_T01; parent_gene=GRMZM2G117064	GRMZM2G117064_P01	TRUE	TRUE	lLWDMLVFR	87%	n+304 (+304)	27.09	26.86
6964	seq=translation; coord=5:6671638..6674577:-1; parent_transcript=GRMZM5G808610_T01; parent_gene=GRMZM5G808610	GRMZM5G808610_P01	TRUE	TRUE	gAALLLIR	87%	n+304 (+304)	25.10	25.00
6965	seq=translation; coord=9:152556214..152556891:-1; parent_transcript=AC149475.2_FGT007; parent_gene=AC149475.2_FG007	AC149475.2_FGP007	TRUE	TRUE	rLDLSASPLADPEAAAFASPR	95%	n+304 (+304)	44.18	25.00
6966	seq=translation; coord=5:4582792..4584392:1; parent_transcript=AC159612.1_FGT007; parent_gene=AC159612.1_FG007	AC159612.1_FGP007	TRUE	TRUE	vTFPALQNIQNALNEAGLGDTVk	95%	n+304 (+304), K+304 (+304)	32.21	25.00
6967	seq=translation; coord=5:128454834..128463337:1; parent_transcript=AC198206.5_FGT001; parent_gene=AC198206.5_FG001	AC198206.5_FGP001	TRUE	TRUE	gLFDSPWSDEVVGGVELAk	95%	n+304 (+304), K+304 (+304)	34.08	25.17
6968	seq=translation; coord=3:126509251..126510797:-1; parent_transcript=AC210168.4_FGT003; parent_gene=AC210168.4_FG003	AC210168.4_FGP003	TRUE	TRUE	tTPTDVEPVPQR	95%	n+304 (+304)	35.38	25.00
6969	seq=translation; coord=1:224037328..224040990:1; parent_transcript=AC210173.4_FGT005; parent_gene=AC210173.4_FG005	AC210173.4_FGP005	TRUE	TRUE	lQQELADVVGLDR	95%	n+304 (+304)	41.81	26.38
6970	seq=translation; coord=9:149274596..149278053:1; parent_transcript=GRMZM2G004172_T01; parent_gene=GRMZM2G004172	GRMZM2G004172_P01,GRMZM2G070716_P01, GRMZM2G070716_P02	TRUE	TRUE	sAILPMLQAEEDER	95%	n+304 (+304)	37.74	25.00
6971	seq=translation; coord=8:1523477..1527610:-1; parent_transcript=GRMZM2G005980_T01; parent_gene=GRMZM2G005980	GRMZM2G005980_P01,GRMZM2G130366_P01	TRUE	TRUE	gELYYVLLNLR	95%	n+304 (+304)	33.22	26.04
6972	seq=translation; coord=8:25119900..25122631:1; parent_transcript=GRMZM2G007021_T01; parent_gene=GRMZM2G007021	GRMZM2G007021_P01,GRMZM2G007021_P02, GRMZM2G102795_P01,GRMZM2G127850_P01, GRMZM5G871980_P01	TRUE	TRUE	dVAWAPNLGLPk	95%	n+304 (+304), K+304 (+304)	43.85	25.88
6973	seq=translation; coord=8:80681448..80685002:-1; parent_transcript=GRMZM2G008464_T02; parent_gene=GRMZM2G008464	GRMZM2G008464_P02,GRMZM5G886688_P01	TRUE	TRUE	ecSGVQPQLWAR	95%	n+304 (+304), Carbamidomethyl (+57)	32.50	25.00
6974	seq=translation; coord=5:14814824..14815810:1; parent_transcript=GRMZM2G010048_T01; parent_gene=GRMZM2G010048	GRMZM2G010048_P01	TRUE	TRUE	vGFEPITLAEYSLGGTGADR	95%	n+304 (+304)	43.10	25.00
6975	seq=translation; coord=7:15074371..15076305:-1; parent_transcript=GRMZM2G013065_T01; parent_gene=GRMZM2G013065	GRMZM2G013065_P01,GRMZM2G014400_P01, GRMZM2G022313_P01,GRMZM2G022313_P02, GRMZM2G144890_P01,GRMZM2G162954_P01	TRUE	TRUE	gFGFITYDSEDAVDk	95%	n+304 (+304), K+304 (+304)	70.28	25.00

6976	seq=translation; coord=9:16717314..16722369:1; parent_transcript=GRMZM2G014076_T01; parent_gene=GRMZM2G014076	GRMZM2G014076_P01,GRMZM2G014076_P02	TRUE	TRUE	lQIPDVETVAAPDNVkDEL	95%	n+304 (+304), K+304 (+304)	37.62	26.49
6977	seq=translation; coord=5:35276306..35278954:-1; parent_transcript=GRMZM2G018082_T01; parent_gene=GRMZM2G018082	GRMZM2G018082_P01	TRUE	TRUE	sILWAVDYELPETVIAHR	95%	n+304 (+304)	36.62	25.51
6978	seq=translation; coord=2:43117505..43119018:-1; parent_transcript=GRMZM2G018275_T01; parent_gene=GRMZM2G018275	GRMZM2G018275_P01	TRUE	TRUE	dLAGWGGLMGAGHDYyER	95%	n+304 (+304), iTRAQ8plex (+304)	28.35	25.00
6979	seq=translation; coord=1:235360116..235365801:1; parent_transcript=GRMZM2G020295_T01; parent_gene=GRMZM2G020295	GRMZM2G020295_P01,GRMZM2G020295_P02, GRMZM2G020295_P03	TRUE	TRUE	aILGSSVPFQSLk	95%	n+304 (+304), K+304 (+304)	45.40	25.00
6980	seq=translation; coord=1:267867135..267870655:-1; parent_transcript=GRMZM2G023110_T01; parent_gene=GRMZM2G023110	GRMZM2G023110_P01	TRUE	TRUE	iSEIGELQHEFIVPLR	95%	n+304 (+304)	45.66	25.82
6981	seq=translation; coord=5:84248220..84252180:1; parent_transcript=GRMZM2G026117_T01; parent_gene=GRMZM2G026117	GRMZM2G026117_P01,GRMZM2G026117_P02, GRMZM2G026117_P03,GRMZM2G041159_P01, GRMZM2G041159_P02	TRUE	TRUE	eGDVSATLLALAGLR	95%	n+304 (+304)	57.97	25.84
6982	seq=translation; coord=9:1443455..1448097:1; parent_transcript=GRMZM2G027627_T01; parent_gene=GRMZM2G027627	GRMZM2G027627_P01	TRUE	TRUE	gGDPVAAFAELMGR	95%	n+304 (+304)	38.32	25.00
6983	seq=translation; coord=5:4684019..4687923:-1; parent_transcript=GRMZM2G028218_T01; parent_gene=GRMZM2G028218	GRMZM2G028218_P01,GRMZM2G028218_P02, GRMZM2G134917_P01,GRMZM2G337845_P01	TRUE	TRUE	yYEILGVSk	95%	n+304 (+304), K+304 (+304)	36.27	25.00
6984	seq=translation; coord=3:225301514..225308308:1; parent_transcript=GRMZM2G040401_T01; parent_gene=GRMZM2G040401	GRMZM2G040401_P01,GRMZM2G040401_P02	TRUE	TRUE	hAFIGLGFR	95%	n+304 (+304)	39.60	25.00
6985	seq=translation; coord=1:63645310..63646802:1; parent_transcript=GRMZM2G040638_T01; parent_gene=GRMZM2G040638	GRMZM2G040638_P01	TRUE	TRUE	gMSLEDLVVLSGAHTVGR	95%	n+304 (+304)	64.72	25.62
6986	seq=translation; coord=1:195400126..195416505:1; parent_transcript=GRMZM2G043198_T01; parent_gene=GRMZM2G043198	GRMZM2G043198_P01,GRMZM2G043198_P03	TRUE	TRUE	mVGYALQAADILAk	95%	n+304 (+304), K+304 (+304)	31.92	25.00
6987	seq=translation; coord=7:163371037..163375463:1; parent_transcript=GRMZM2G044023_T01; parent_gene=GRMZM2G044023	GRMZM2G044023_P01,GRMZM2G044023_P03, GRMZM5G873123_P01,GRMZM5G873123_P02	TRUE	TRUE	aALAIIDEMVDAGFTPSk	95%	n+304 (+304), K+304 (+304)	51.34	26.28
6988	seq=translation; coord=9:43114595..43121822:1; parent_transcript=GRMZM2G047178_T01; parent_gene=GRMZM2G047178	GRMZM2G047178_P01,GRMZM2G105598_P02, GRMZM2G105598_P03,GRMZM2G105598_P04	TRUE	TRUE	fAVGALAFDDVSVAVDHLk	95%	n+304 (+304), K+304 (+304)	45.14	25.42
6989	seq=translation; coord=4:219992405..219994767:-1; parent_transcript=GRMZM2G051185_T01; parent_gene=GRMZM2G051185	GRMZM2G051185_P01	TRUE	TRUE	aAGTLNALADQDQIQLLLYHVLPR	95%	n+304 (+304)	71.32	25.00
6990	seq=translation; coord=4:187289565..187295583:1; parent_transcript=GRMZM2G055507_T01; parent_gene=GRMZM2G055507	GRMZM2G055507_P01	TRUE	TRUE	aVDQIIQIPTVLR	95%	n+304 (+304)	43.02	25.00
6991	seq=translation; coord=5:65203097..65206027:1; parent_transcript=GRMZM2G055682_T01; parent_gene=GRMZM2G055682	GRMZM2G055682_P01	TRUE	TRUE	aLAEIALEQQR	95%	n+304 (+304)	38.59	26.30
6992	seq=translation; coord=1:276989217..276990796:1; parent_transcript=GRMZM2G058032_T01; parent_gene=GRMZM2G058032	GRMZM2G058032_P01,GRMZM2G058032_P02	TRUE	TRUE	gEFcIDDIDFDEk	95%	n+304 (+304), Carbamidomethyl (+57), K+304 (+304)	37.00	25.00
6993	seq=translation; coord=8:16690779..16693942:-1; parent_transcript=GRMZM2G061187_T01; parent_gene=GRMZM2G061187	GRMZM2G061187_P01,GRMZM2G459861_P01	TRUE	TRUE	lLAVDIPMASGPDQR	95%	n+304 (+304)	44.13	25.93

6994	seq=translation; coord=1:290103711..290108581:1; parent_transcript=GRMZM2G062394_T01; parent_gene=GRMZM2G062394	GRMZM2G062394_P01,GRMZM2G108355_P01, GRMZM2G108355_P02,GRMZM2G108355_P03	TRUE	TRUE	sPVTIcGDIHGQFHDLAELFR	95%	n+304 (+304), Carbamidomethyl (+57)	35.07	25.07
6995	GRMZM2G066274_P01-R	GRMZM2G066274_P01-R	TRUE	TRUE	yNQILR	95%	n+304 (+304)	31.26	25.11
6996	seq=translation; coord=9:8459603..8468658:-1; parent_transcript=GRMZM2G066373_T01; parent_gene=GRMZM2G066373	GRMZM2G066373_P01	TRUE	TRUE	lWSEADEIALLTGAAAFk	95%	n+304 (+304), K+304 (+304)	37.57	25.00
6997	seq=translation; coord=2:203713965..203723595:1; parent_transcript=GRMZM2G067063_T01; parent_gene=GRMZM2G067063	GRMZM2G067063_P01,GRMZM2G067063_P02, GRMZM2G067063_P03	TRUE	TRUE	vDcTEEVELcR	95%	n+304 (+304), Carbamidomethyl (+57), Carbamidomethyl (+57)	39.44	25.00
6998	seq=translation; coord=7:163915244..163920524:-1; parent_transcript=GRMZM2G069405_T01; parent_gene=GRMZM2G069405	GRMZM2G069405_P01,GRMZM2G069405_P02	TRUE	TRUE	nLPFEATVEMVEQEFSk	95%	n+304 (+304), K+304 (+304)	51.46	25.00
6999	seq=translation; coord=7:127252507..127255099:-1; parent_transcript=GRMZM2G072240_T01; parent_gene=GRMZM2G072240	GRMZM2G072240_P01,GRMZM2G072240_P02	TRUE	TRUE	gIFLGNPLLDLYLYEk	95%	n+304 (+304), K+304 (+304)	38.00	25.00
7000	seq=translation; coord=3:222996465..222998811:-1; parent_transcript=GRMZM2G074599_T01; parent_gene=GRMZM2G074599	GRMZM2G074599_P01	TRUE	TRUE	sDTSDVSGEQNEGDEHk	95%	n+304 (+304), K+304 (+304)	42.25	25.00
7001	seq=translation; coord=9:1848054..1851533:1; parent_transcript=GRMZM2G075470_T01; parent_gene=GRMZM2G075470	GRMZM2G075470_P01,GRMZM2G075470_P02	TRUE	TRUE	aIEVLLGDFHDR	95%	n+304 (+304)	38.69	25.00
7002	seq=translation; coord=4:170814386..170816284:1; parent_transcript=GRMZM2G075513_T01; parent_gene=GRMZM2G075513	GRMZM2G075513_P01	TRUE	TRUE	gVVADPEGLAGR	95%	n+304 (+304)	32.72	25.26
7003	seq=translation; coord=1:300464398..300469096:1; parent_transcript=GRMZM2G077222_T01; parent_gene=GRMZM2G077222	GRMZM2G077222_P01	TRUE	TRUE	aAVESEMEVLLk	95%	n+304 (+304), K+304 (+304)	45.25	25.16
7004	seq=translation; coord=1:251088454..251096714:-1; parent_transcript=GRMZM2G080828_T01; parent_gene=GRMZM2G080828	GRMZM2G080828_P01,GRMZM2G080828_P02	TRUE	TRUE	lAPPLTISPEELAEASk	95%	n+304 (+304), K+304 (+304)	44.72	25.24
7005	seq=translation; coord=4:116701848..116708846:-1; parent_transcript=GRMZM2G082642_T01; parent_gene=GRMZM2G082642	GRMZM2G082642_P01,GRMZM2G082642_P02, GRMZM2G082642_P03,GRMZM2G082642_P05, GRMZM2G082642_P06,GRMZM2G082642_P07	TRUE	TRUE	lNLETDISHWIVR	95%	n+304 (+304)	53.81	26.67
7006	seq=translation; coord=1:202765503..202766778:1; parent_transcript=GRMZM2G090177_T01; parent_gene=GRMZM2G090177	GRMZM2G090177_P01	TRUE	TRUE	lPLRDVLDDAGGVGGAR	95%	n+304 (+304)	31.76	25.09
7007	seq=translation; coord=5:24436192..24442540:-1; parent_transcript=GRMZM2G090736_T01; parent_gene=GRMZM2G090736	GRMZM2G090736_P01	TRUE	TRUE	nEEAGQPESATEk	95%	n+304 (+304), K+304 (+304)	41.44	25.00
7008	seq=translation; coord=7:988166..993667:1; parent_transcript=GRMZM2G090824_T01; parent_gene=GRMZM2G090824	GRMZM2G090824_P01,GRMZM2G090824_P02	TRUE	TRUE	qSFITNLQALNcASFLGAk	95%	n+304 (+304), Carbamidomethyl (+57), K+304 (+304)	53.32	25.00
7009	seq=translation; coord=7:34171226..34185881:1; parent_transcript=GRMZM2G092129_T01; parent_gene=GRMZM2G092129	GRMZM2G092129_P01,GRMZM2G092129_P02	TRUE	TRUE	lAVNTLESEITQDATLk	95%	n+304 (+304), K+304 (+304)	38.75	26.06
7010	seq=translation; coord=9:154438057..154439339:1; parent_transcript=GRMZM2G092718_T01; parent_gene=GRMZM2G092718	GRMZM2G092718_P01	TRUE	TRUE	aMEAVADIYGIDSIAADVk	95%	n+304 (+304), K+304 (+304)	56.24	25.44
7011	seq=translation; coord=9:154404210..154409319:-1; parent_transcript=GRMZM2G092741_T01; parent_gene=GRMZM2G092741	GRMZM2G092741_P01,GRMZM2G092741_P02, GRMZM2G137236_P01	TRUE	TRUE	lVLFLGNk	95%	n+304 (+304), K+304 (+304)	29.67	25.00

7012	seq=translation; coord=2:64929995..64930706:1; parent_transcript=GRMZM2G097747_T01; parent_gene=GRMZM2G097747	GRMZM2G097747_P01	TRUE	TRUE	aARPDVDEEAAAVAEAAR	95%	n+304 (+304)	48.36	25.00
7013	seq=translation; coord=8:23887719..23890042:1; parent_transcript=GRMZM2G099483_T01; parent_gene=GRMZM2G099483	GRMZM2G099483_P01	TRUE	TRUE	sADGVDGEYAATASR	95%	n+304 (+304)	52.95	25.00
7014	seq=translation; coord=2:37049378..37052466:1; parent_transcript=GRMZM2G100315_T01; parent_gene=GRMZM2G100315	GRMZM2G100315_P01	TRUE	TRUE	aVGIEHVPEIVASSVENVQR	95%	n+304 (+304)	31.43	25.25
7015	seq=translation; coord=5:200934680..200937975:1; parent_transcript=GRMZM2G101287_T01; parent_gene=GRMZM2G101287	GRMZM2G101287_P01	TRUE	TRUE	hAFDWALGHIAR	95%	n+304 (+304)	44.69	25.00
7016	seq=translation; coord=8:1091537..1092833:-1; parent_transcript=GRMZM2G103512_T01; parent_gene=GRMZM2G103512	GRMZM2G103512_P01	TRUE	TRUE	tVATGDVFVFPR	95%	n+304 (+304)	65.84	26.03
7017	seq=translation; coord=8:153794205..153796938:-1; parent_transcript=GRMZM2G112535_T01; parent_gene=GRMZM2G112535	GRMZM2G112535_P01,GRMZM2G112535_P02	TRUE	TRUE	nVEATVLEILR	95%	n+304 (+304)	37.15	25.17
7018	seq=translation; coord=2:196406920..196412546:-1; parent_transcript=GRMZM2G114107_T01; parent_gene=GRMZM2G114107	GRMZM2G114107_P01,GRMZM2G114107_P02	TRUE	TRUE	hMNDQGWVPINLILGFNR	95%	n+304 (+304)	32.21	25.35
7019	seq=translation; coord=5:60777721..60779927:-1; parent_transcript=GRMZM2G115621_T01; parent_gene=GRMZM2G115621	GRMZM2G115621_P01,GRMZM2G115621_P02, GRMZM2G115621_P03	TRUE	TRUE	hLFYQDASDLR	95%	n+304 (+304)	49.69	25.00
7020	seq=translation; coord=1:70247200..70252204:1; parent_transcript=GRMZM2G117222_T02; parent_gene=GRMZM2G117222	GRMZM2G117222_P02,GRMZM2G117222_P03, GRMZM2G117222_P04	TRUE	TRUE	mGWSESAMVAGAR	95%	n+304 (+304)	34.53	25.00
[truncated: 149,876 more chars]
